# Multi-omics profiling of papillary thyroid microcarcinoma reveals different somatic mutations and a unique transcriptomic signature

**DOI:** 10.1186/s12967-023-04045-2

**Published:** 2023-03-20

**Authors:** Qiang Li, Tienan Feng, Tengteng Zhu, Weituo Zhang, Ying Qian, Huan Zhang, Xiangqian Zheng, Dapeng Li, Xinwei Yun, Jingzhu Zhao, Yangyang Li, Herbert Yu, Ming Gao, Biyun Qian

**Affiliations:** 1 Hongqiao International Institute of Medicine, Shanghai Tongren Hospital and School of Public Health, Shanghai Jiao Tong University School of Medicine, 277 South Chongqing Road, Huangpu District, Shanghai, 200025 China; 2 Clinical Research Institute, Shanghai Jiao Tong University School of Medicine, Shanghai, 200025 China; 3 Cancer Prevention Center, National Clinical Research Center for Cancer, Key Laboratory of Cancer Prevention and Therapy, Tianjin, Tianjin’s Clinical Research Center for Cancer, Tianjin Medical University Cancer Institute and Hospital, Tianjin, 300060 China; 4 Cancer Epidemiology Program, University of Hawaii Cancer Center, 701 Ilalo Street, Honolulu, HI 96813 USA; 5 Department of Thyroid and Breast Surgery, Tianjin Union Medical Center, Tianjin, Tianjin Key Laboratory of General Surgery in Construction, Tianjin Union Medical Center, Tianjin, 300121 China; 6 Department of Head and Neck Tumor, Tianjin Medical University Cancer Institute and Hospital, Tianjin, 300060 China; 7 Shanghai Tenth People’s Hospital Affiliated to Tongji University, Shanghai, 200072 China

**Keywords:** Whole exome sequencing, RNA-sequencing, Papillary thyroid microcarcinoma, Molecular classification, Immune microenvironment

## Abstract

**Background:**

Papillary thyroid microcarcinoma (PTMC) incidence has significantly increased, and some cases still exhibit invasive traits. The entire molecular landscape of PTMC, which can offer hints for the etiology of cancer, is currently absent.

**Methods:**

We compared our findings with those for PTMC in the TCGA by analyzing the largest study at the current stage of whole exome sequencing and RNA-sequencing data from 64 patients with PTMC. Then, we systematically demonstrated the differences between the two PTMC subtypes based on multi-omics analyses. Additionally, we created a molecular prediction model for the PTMC subtypes and validated them among TCGA patients for individualized integrative assessment.

**Results:**

In addition to the presence of *BRAF* mutations and *RET* fusions in the TCGA cohort, we also discovered a new molecular signature named PTMC-inflammatory that implies a potential response to immune intervention, which is enriched with *AFP* mutations, *IGH@-ext* fusions, elevated immune-related genes, positive peroxidase antibody, and positive thyroglobulin antibody. Additionally, a molecular prediction model for the PTMC-inflammatory patients was created and validated among TCGA patients, while the prognosis for these patients is poor.

**Conclusions:**

Our findings comprehensively define the clinical and molecular features of PTMC and may inspire new therapeutic hypotheses.

**Supplementary Information:**

The online version contains supplementary material available at 10.1186/s12967-023-04045-2.

## Background

Thyroid cancer (TC) is the most common endocrine malignancy, and its incidence has risen dramatically over the past several years around the world [1]. Differentiated thyroid cancer (DTC) represents the majority (90%) of all types of thyroid cancer, and papillary thyroid carcinoma (PTC) is the most prevalent kind of DTC [2]. The increase in TC is mostly attributable to an increase in the detection of papillary thyroid microcarcinoma (PTMC, diameter ≤ 10 mm) [3]. The two histological subtypes of PTMC that occur most frequently are classic and follicular-variant [4]. The majority of patients with PTMC are asymptomatic, with inert behavior and a favorable prognosis. In 2015, the American Thyroid Association (ATA) recommended active surveillance (AS) for low-risk PTMC [5] to save some patients from needless surgery; however, lymph node involvement or distant metastasis may still occur in some patients [6, 7], leading to anxiety among clinicians and patients, which hinders the implementation of AS [8]. Therefore, stratifying PTMC patients remains to be a challenge for the appropriate management of PTMC.

In the era of rapid development of next-generation sequencing technology, molecular profiling has emerged as a critical characterization for PTMC to enhance patient surveillance and treatment [9, 10]. However, most earlier investigations of genomic features focused on PTC samples, with only a few containing PTMC, and the patients under study were mostly from the European ancestry [11]. The molecular characteristics of PTC patients of different races are different. For instance, *BRAF* mutations are found in 72.4% of Chinese PTCs [12], which is much greater than that in Europeans, implying a racial difference between Chinese and Europeans. Furthermore, at present, the treatment of tumors, including PTMC, is mainly determined by their clinical characteristics, but a large-scale molecular profiling study has revealed that there is significant heterogeneity in cancer driver genes and pathways among tumor types and even histological subtypes. Many common tumors have been thoroughly defined by multi-omics analysis and characterization of genetic determinants of tumor behavior and outcome, leading to the development of personalized therapies [13, 14]. Despite the recent progress, information on genetic features, molecular subtypes, and therapeutic targets is still limited for PTMC. Therefore, a complete investigation of the molecular profile of PTMC is urgently needed, which will have significant implications for diagnosis and intervention.

In this study we profiled, to our knowledge, the first and largest Papillary Thyroid Microcarcinoma Exome and Transcriptome Atlas (PTMETA), to elucidate their transcriptomic and genomic features that may alter the therapeutic options available to cancer patients. Surprisingly, integrated analyses of multi-omic data revealed genomic and transcriptomic features of PTMC and identified a unique subgroup with distinct biology and clinical behavior, which in turn may provide a way for individualized intervention.

## Methods

### Biospecimen collection, pathological assessment, and public data processing

This retrospective study was approved by local ethical committees (Tongren Hospital-Shanghai Jiao Tong University School of Medicine), and written informed consents were obtained from all patients. A total of 64 PTMC patients were recruited among the 128 samples stored into the frozen tissue biobank of the Tongren Hospital-Shanghai Jiao Tong University School of Medicine from 2018 to 2019. The tissue samples were removed from the body in the operating room and then cut into 5 mm-long pieces on a sterile curved disc. The necrotic and calcified regions were carefully removed [15]. Immediately after thyroidectomy, the specimens were flash-frozen in liquid nitrogen and stored in the biobank. TNM stage of the disease was defined by pathologists according to the 8th AJCC/UICC staging system. The normal ranges for serum levels of thyroid peroxidase antibody (TPOAb) and thyroglobulin antibody (TgAb) were 0–4.1 IU/mL and 0–9 IU/mL. If the serum level of the thyroid antibody was over the upper limit, the status was termed positive. PTMETA patients’ demographic and clinical information are shown in Additional file 2: Table S1.

Somatic mutation data, copy number variation, fusion gene data, RNA-sequencing (RNA-seq) counts, and clinicopathological data for 28 PTMC-TCGA (tumor size ≤ 10 mm) samples were acquired from Genomic Data Commons (https://portal.gdc.cancer.gov), Tumor Fusion Gene Data Portal (https://www.tumorfusions.org/), R package “TCGAbiolinks (v.2.20.1)” [16], and Memorial Sloan Kettering Cancer Center cBioPortal (http://www.cbioportal.org/public-portal/study.do?cancer_study_id=thca_tcga). In addition, RNA-seq counts and survival data of 331 patients with early stage PTC (stage I and stage II) were downloaded for subsequent prediction model validation. TNM stage was redefined according to the 8th AJCC/UICC staging system. 28 PTMC-TCGA patients' demographic and clinical information are shown in Additional file 2: Table S2.

### DNA and RNA isolation, quantification, and qualification

DNA from the tumor and matched adjacent normal tissue samples was extracted with the QIAGEN DNA Tissue Extraction Kit according to the manufacturer’s protocol, which was then quantified using the Qubit HS DNA Assay kit and Qubit 2.0 fluorometer (Life Technologies Inc). RNA from the tumor and matched adjacent normal tissue samples was extracted using the QIAGEN miRNeasy Mini Kit with a QIAcube, according to the manufacturer’s protocol. Total RNA was quantitated using Nanodrop 2000 Spectrophotometer, and the LabChip GX Touch HT nucleic acid analyzer was used to confirm RNA integrity. For DNA, only samples with a concentration of at least 100.0 ng/µL were used for sequencing. For RNA, only samples with a concentration of at least 100.0 ng/µL and an RNA integrity number (RIN) number greater than 7 were included for sequencing.

### DNA-sequencing libraries

The SureSelect^XT^ Human All Exon V6 (Agilent Technologies Inc., USA) was used to create DNA libraries from 50 ng of genomic DNA, according to the manufacturer’s instructions. This procedure targets 58 Mb of the genomic region, which includes 99% of coding regions in addition to 5′ and 3′-untranslated region sequences. The Illumina HiSeq3000 platform (2 × 150 bp paired-end reads) was used to sequence WES libraries (64 tumor/matched adjacent normal tissue sample pairs).

### RNA-sequencing libraries

The TruSeq Stranded Total RNA Sample Prep Kit (Illumina Inc., USA) was used to construct RNA libraries from 550 ng of total RNA, according to the manufacturer’s instructions. The LabChip GX Touch HT nucleic acid analyzer was used to examine the RIN quality of isolated total RNA. The Illumina HiSeq3000 platform (2 × 150 bp paired-end reads) was used to sequence RNA-seq libraries (64 tumor/matched adjacent normal tissue sample pairs).

### Whole exome sequencing processing

Trimmomatic (v.0.39) [17] was used to filter the raw sequencing reads for low-quality reads and adapter regions. Then sequencing reads were aligned to the Human Genome Reference Consortium build 38 (GRCh38) using BWA (v.0.7.17, BWA-mem). We used the ‘Picard’ workflow (http://broadinstitute.github.io/picard) to combine data from multiple libraries and flow cell runs into a single BAM file per sample. In the following analyses, only reads that were uniquely aligned and de-duplicated were used. Realignment and base quality score recalibration were performed using the Genome Analysis Toolkit 4 (GATK4, V.4.1.4.1) [18]. All sites potentially containing small insertions or deletions in either tumor or matched adjacent normal tissue were realigned using GATK4. Sample identities were verified by determining the concordance of the genotypes using GATK4 HaplotypeCaller. The actual match will typically have upwards of 95% concordance between samples from the same individuals. A total of 64 tumor-normal pairs of samples were included in the downstream analysis.

### RNA-seq processing

To ensure data consistency and reproducibility, the raw reads were preprocessed using Trimmomatic (v.0.39) [17] to remove low-quality sequences and disjunction contamination, resulting in high-quality sequences (clean reads), and all subsequent analyses were based on clean data. Using a two-pass approach STAR (v.2.4.0) [19], clean RNA-seq reads were aligned to the GRCh38.d1.vd1 reference genome with GENCODE v22 annotation. Gene-level aligned fragment counts were generated using RSEM (v.1.2.28) [20].

### Mutation calling

To create the panel of normals (PoNs), we first used the 64 matched adjacent normal tissue samples from this study and deleted any mutation with a corresponding alternate allele present in > 1 PoN samples. For SNVs, we used positions that are called by GATK4 Mutect2 (v.4.1.4.1) [18]. For mutation calling, a minimum of five variant-containing reads and VAF ≥ 0.04 in the tumor were required. Any indel found in more than 1 PoN sample was eliminated. Variant call format (VCF) files were annotated with ANNOVAR [21]. The mutation data of the TCGA cohort was in Annotated Somatic Mutation format, and the workflow type is “MuTect2 Annotation”. The R package “maftools” (v.2.6.05) was used to visually analyze the mutation annotation format (MAF) file [22].

### Tumor mutation burden and mutational signatures

Tumor mutation burden (TMB) was defined as the total number of SNVs and Indels within exonic regions. To calculate the TMB per megabase (Mb), the total number of mutations counted is divided by the size of the coding region in a targeted territory (58 Mb for PTMETA and 38 Mb for TCGA [23]). The mutational signatures that were present in the samples were identified using the R package “musicatk” (v.1.2.0) [24]. COSMIC v2 SBS signatures were predicted using the NMF-based prediction approach. When the reconstruction error was minimized, 10 rounds with a random seed of 12345 were used to find the optimal number of output signatures (*k*, candidate range from 2 to 5). Finally, the reconstruction errors were minimal when we used *k* = 4 for PTMETA and *k* = 5 for TCGA.

### Somatic CNV detection

We analyzed sequencing coverage and copy number in aligned sequencing reads from targeted amplicon sequencing of the PTMETA tumor and matched adjacent normal tissue samples using the software Sequenza (v.2.1.2) [25]. Each cohort’s significantly amplified and deleted regions were identified using the GISTIC2 (v.2.0.23) [26] algorithm. Output segmentation from Sequenza (PTMETA) and masked copy number segment from TCGA were used as the input for GISTIC2. Specifically, the Seg.CN of PTMETA required by GISTIC2 was calculated with the depth.ratio estimation from Sequenza as Seg.CN = log_2_ (2 × depth.ratio) – 1. To find recurrently amplified or deleted genomic regions, the GISTIC2 was run with the following modified parameters: -ta 0.2 -td 0.2 -js 100 -broad 1 -brlen 0.7 -conf 0.95 -genegistic 1 -savegene 1. Chromosomal arms were deemed changed in chromosome arm level analysis if at least 60% of the arm was lost or gained with a relative log2-transfomred copy number change > 0.10. Significant peaks were defined as regions with q < 0.25 and were annotated with CGC (v.85).

### Fusion detection and reverse transcription PCR

We utilized STAR-Fusion (v.1.6.0) (github.com/STAR-Fusion) [27] to find gene fusions from RNA-seq data, which identifies fusion transcripts and publishes all supporting data discovered during alignment. FusionInspector (v.2.8.0) [28] results that assist in fusion transcript detection by doing a supervised analysis of fusion predictions, aiming to recover and re-score evidence for such predictions were used to reduce false-positive fusions. Any fusion couple including the mitochondrial or HLA gene partner, involving two immunoglobulin gene segments, and annotated as having been found in normal RNA-seq data sets was filtered. All fusion genes discovered by STAR-Fusion in normal samples were merged into a PoN to remove fusion genes found in those samples. The corresponding fusion was deleted if a fusion breakpoint from a tumor sample was discovered in the PoN. Finally, fusion candidates with less than 0.1 fusion fragments per million total (FFPM) were considered unsupported and discarded. PCR with reverse transcription was also used to confirm the fusion genes that resulted. cDNA was synthesized from 300 ng of RNA using SuperScript II reverse transcriptase (Life Technologies) according to the manufacturer’s instructions.

### Detection of significantly mutated genes

We adopted previously known methods to find driver mutations and genes [29]. As mentioned in ‘Mutation calling’, we utilized the classic GATK toolbox to call mutations and annotated mutations using ANNOVAR. The MAF was examined to discover significantly mutated genes (SMGs) after filtering for artifacts and establishing a final set of mutations. Based on 64 samples, the MutSigCV (v.1.41) [30] algorithms were used to achieve this. A cut-off value of q < 0.10 was utilized for MutSigCV.

### Differentially expressed genes analysis and gene set enrichment analysis

To evaluate gene expression levels and discover differential gene expression, the raw read counts were normalized with DESeq2 (v.1.30.1) [31]. Differentially expressed genes (DEGs, genes with at least ten samples displaying nonzero counts) by comparing tumors against matched normal samples were identified using an FDR < 0.05 and a fold-change threshold of at least 2. Gene Set Enrichment Analysis (GSEA) was performed using the R package “clusterProfiler” (v.4.1.4) [32]. Differential expression analysis outputs of DESeq2 (tumor vs. normal) were used to generate the ranked list file (ranked by (–log10[*p* value])/[sign of log2FoldChange]). Pathways and terms at FDR < 0.05 and|normalized enrichment score (NES)|> 2 were considered statistically significant.

### Expression-based unsupervised clustering

The normalized counts obtained from DESeq2 from two cohorts were utilized to identify PTMC clusters with the unsupervised clustering method single-cell consensus clustering (SC3) by using the R package “SC3” (v.1.18.0) [33]. To determine the optimal number of genes in SC3, we tested the clustering findings by selecting genes based on the standard deviations (SD) with the top 1000, 2000, 3000, 4000, and 5000 genes. An appropriate parameter *k* was tested from 2 to 5 iteratively. We determined the ideal *k* and SD top genes number using three SC3 results: silhouette score, consensus matrix, and cluster-specific genes. Finally, the average silhouette scores were the highest when we chose the SD top 5000 genes and *k*-means = 2 for PTMETA and SD top 2000 and *k*-means = 2 for TCGA (Additional file 2: Table S4). Marker genes in each cluster were identified by SC3 with the adjusted *p*-value < 0.05. The Kyoto Encyclopedia of Genes and Genomes (KEGG) analysis of marker genes in each cluster was carried out with default parameters of the R package “clusterprofiler” [34]. The NMF method from the R package “CancerSubtypes” (v.1.18.0) [35] was also utilized to get more reliable clustering subgroup results. The optimal number of genes and *k* were determined with the value of average silhouette width, and then genes and *k* were the same with SC3 (Additional file 2: Table S4).

### Subclass mapping of RNA subgroups

An unsupervised subclass mapping method (SubMap, v3.0) [36] was utilized to find correspondence or commonality of subgroups from the two cohorts to compare the RNA subgroups discovered from both cohorts. On the GenePattern online platform, the normalized counts using DESeq2 from two cohorts were utilized for SubMap analysis with default settings and a random seed of 12345. Bonferroni adjusted *p* < 0.05 was used to determine whether any correspondences were significant.

### Immune microenvironment characterization

To assess the characteristics of the immune microenvironment, the expression data normalized using DESeq2 and followed by log transformation was used. To increase the stability of the conclusion, the immunological landscape of several subgroups was examined using four immune-related algorithms. The presence of immune cell infiltration in tumor samples was determined using the R package “ESTIMATE” (v.1.0.13) [37], as well as with gene set variation analysis (GSVA) using the combined immune cell gene sets from Bindea et al. [38]. The relative abundance of immune cell populations was then calculated using the R package “immunedeconv” (v.2.0.4) [39], which allows the community to perform integrated deconvolution using several approaches such as EPIC [40] and CIBERSORT [41]. To further evaluate whether RNA subgroups would have different responses to immune therapy, we also scored the immune features predictive of checkpoint inhibitor immunotherapy with GSVA using IFN-γ related genes from Ayers et al. [42], as well as compared the expression levels of *PD1* (*PDCD1*), *PDL1* and *CTLA4*. The GSVA was processed using the R package “GSVA” (v.1.0.13) [43].

### Evaluation of the relationship of genomic, transcriptomic, and clinical features

Pairwise correlations of all features including SMGs, fusion genes, RNA subgroups, and clinical features were investigated using a variety of statistical tests. Wilcoxon rank-sum test was used to determine the significance for pairs of continuous variables. Fisher’s exact test was used to compare pairs of categorical variables. Only correlations with *p* < 0.10 were displayed in Fig. 5a. The driver mutation genes, fusion genes, and top 100 marker genes of each transcriptomic subgroup were interrogated with STRING (https://string-db.org/cgi/input.pl) [44] to build the network interaction. Cytoscape (v.3.7.2) [45] was used to construct and visualize the network. The number of nodes in the network symbolizes genes, while the edges reflect interactions between genes. The number of edges was utilized as a benchmark for the significance of the driver genes because a higher number of edges indicates a bigger number of genes involved with their function [44]. Finally, we compared and analyzed the expression levels of related genes in different variation states of hub driver genes to determine the relationship between them.

### Prediction model for molecular signature

The expression data from the PTMETA and TCGA cohorts were normalized with DESeq2 and followed by log transformation. The batch effect was adjusted by performing the ComBat function in the R package “sva” (v.3.46.0) [46]. The LASSO logistic regression [47] was used to construct a prediction model for molecular signatures in PTMETA. We performed a tenfold cross-validation to yield the optimal of regularization parameter (lambda) minimizing the sum of least square plus shrinkage penalty by using the R package “glmnet” (v.4.1–6) [48]. The model predicts inflammatory signature versus non-inflammatory signature using a risk score. Genes with nonzero coefficients were selected to calculate the risk score. The risk score was calculated using the following formula: risk score = expression level of gene 1 * c1 + expression level of gene 2 * c2 + … + expression level of gene n * cn, where c represents the coefficient. The LASSO model was trained in the PTMETA cohort, and its performance was evaluated using the area under the receiver-operating-characteristics (ROC) curve (AUC) [49]. The risk score cut-off point was determined based on Youden’s index provided in the output of the ROC analysis [50]. The prediction model was tested in the PTMC-TCGA (28 patients) and Early-Stage-PTC-TCGA (ESPTC-TCGA; Stage I and Stage II; 331 patients) cohorts. To check the model validity, we compared the characteristics of the tumor immune microenvironment between the two signatures using the method called “Immune microenvironment characterization”. For the two signatures, we also constructed their K-M survival curves and compared 5 year progression-free survival (PFS) rates [51]. K-M survival curves coupled with Log-rank test were performed using the R packages “survival” (v.3.4–0) and “survminer” (v.0.4.9).

### Quantification and statistical analysis

All analyses were performed using R software v4.0.3 (https://cran.r-project.org/). Wilcoxon rank-sum test was used to compare the distributions of continuous measurements between two groups. Fisher’s exact test was used to assess the enrichment of mutations in a given gene as compared to the background mutation rate. Chi-square test was used to assess the enrichment of mutation signatures in each cohort. Nominal *p*-values were reported throughout. Differential gene expression, GSEA enrichment analyses, and KEGG analyses (tumors & normals; C1 & C2) were subjected to multiple testing adjustments using the Benjamini–Hochberg False Discovery Rate method. With outliers not indicated, all box and whiskers plots in the main and supplemental figures were generated with boxes representing the 25th percentile, median, and 75th percentile, and whiskers displaying the maximum and minimum values within 1.5 times the inter-quartile range from the border of the box. Unless otherwise noted, a *p*-value < 0.05 was considered statistically significant.

## Results

### Study population and sequencing results

**Fig. 1:**
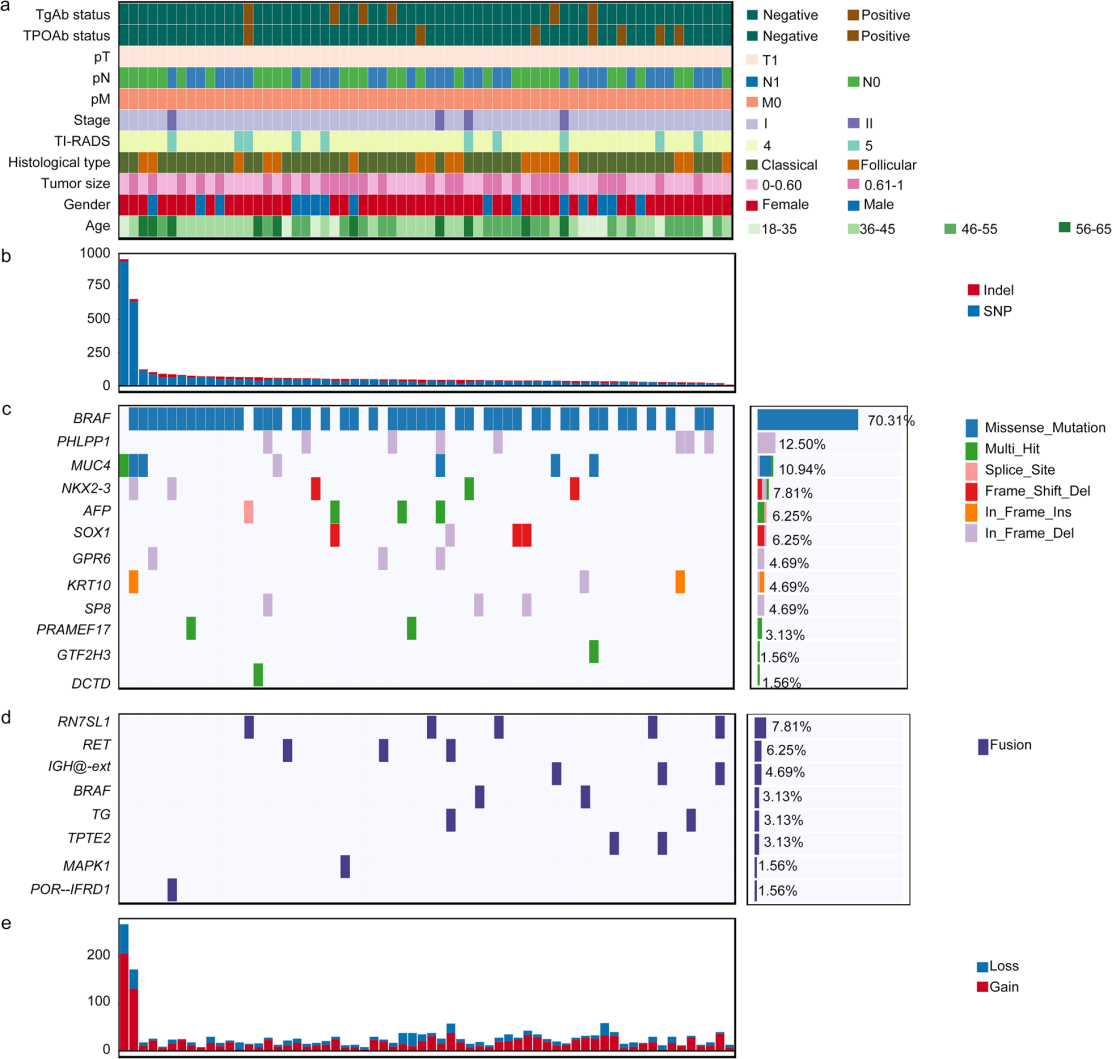
Molecular landscape of the PTMETA cohort. Each column represents an individual tumor (n = 64). **(a)** The top panel shows clinical features, including TgAb status, TPOAb status, TNM, stage, TI-RADS, histological type, tumor size, gender, and age, as per the color key. Each subsequent panel displays a specific molecular profile;** (b)** SNVs and indels per patient; **(c)** Mutated gene name, gene-sample matrix, and prevalence of somatic point mutations; **(d)** Fusion gene name, gene-sample matrix, and prevalence of gene fusion events; **(e)** SCNV segments per patient

WES data and RNA sequencing data on 64 pairs of tumor and matched adjacent normal tissues that were recruited from 2018 to 2019 were shown in Fig. 1a and Additional file 2: Table S1. To assess racial differences in molecular profiles, we also downloaded and processed the exome and RNA-seq data of 28 PTMC European patients from TCGA using the same analytical procedures (Additional file 2: Table S2). PTMC patients in the TCGA cohort had larger tumor size than those in the PTMETA cohort (Additional file 2: Table S3). In terms of age, clinical stage, and other clinical characteristics, the two cohorts were generally comparable.

**Fig. 2:**
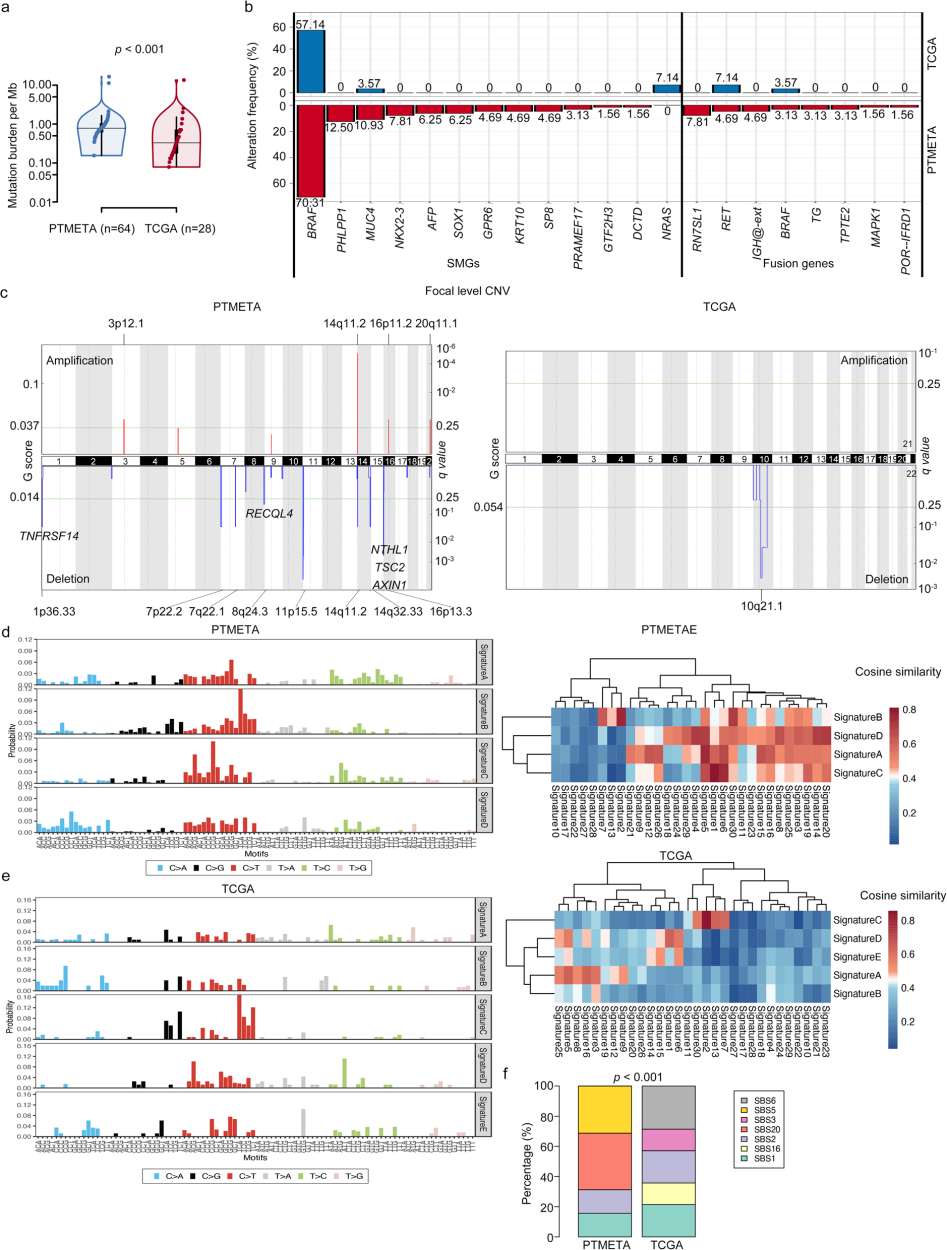
Comparison of genomic landscape between the PTMETA cohort and the TCGA cohort. **(a)** Distribution of mutation burdens in each cohort. The mutation burden calculated from a tumor-normal pair is represented by each dot. The Wilcoxon rank-sum test was used to calculate the *p*-value; **(b)** Gene-level alteration frequencies in the PTMETA and TCGA; **(c)** Significantly recurrent somatic CNAs in genomic regions;** (d)** Mutation contributions of the signatures from de novo decomposition by the NMF algorithm and cosine correlation of the signatures with the 30 COSMIC mutational signatures (v2) from the PTMETA cohort; **(e)** Mutation contributions of the signatures from de novo decomposition by the NMF algorithm and cosine correlation of the signatures with the 30 COSMIC mutational signatures (v2) from TCGA cohort; **(f)** The proportion of mutation signatures. The Chi-square test was used to calculate the *p*-value, which was based on the number of mutation signatures in each cohort

With Mutect, 3944 single-nucleotide variants (SNVs) and 714 insertions/deletions (indels) were discovered in the PTMETA cohort (Fig. 1b). Across the PTMETA cohort, the median TMB was 0.78 per megabase (Mb), much higher than that for the TCGA cohort (median 0.33 per Mb) (Fig. 2a).

### Driver genes

With a larger sample size, we aimed to identify driver genes, especially those rare drivers in the PTMETA cohort. Using the MutSigCV method, we identified 12 drivers at an FDR of 0.10 (Fig. 1c). From the RNA-seq data, we detected 8 gene-to-gene fusions and validated 9 out of 19 events using Sanger sequencing (Fig. 1c and Additional file 1: Fig S1).

The driver mutations in the PTMETA cohort were *BRAF* mutations (70.31% of tumors), followed by *PHLPP1* (12.50%), *MUC4* (10.93%), *NKX2-3* (7.81%), *AFP* (6.25%), *SOX1* (6.25%), *GPR6* (4.69%), *KRT10* (4.69%), *SP8* (4.69%), *PRAME17* (3.13%), *GTF2H3* (1.56%), and *DCTD* (1.56%) mutations (Fig. 1c). We also found fused genes including *RN7SL1* (7.81%), *RET* (6.25%), *IGH@-ext* (4.69%), *BRAF* (3.13%), *TG* (3.13%), *TPTE2* (3.13%), *MAPK1* (1.56%), and *POR-IFRD1* (1.56%) (Fig. 1d). Interestingly, when comparing driver genes across the cohorts, apart from *BRAF, MUC4,* and *RET,* the other 17 genes were present only in the PTMETA cohort, while *NRAS* mutation common in TCGA was absent in this cohort (Fig. 2b). All *BRAF* mutations in the PTMETA were V600E substitutions, whereas 14 out of 16 (87.50%) in TCGA were V600E substitutions (Additional file 1: Fig S2). The hallmark fusion gene *RET* showed little differences between PTMETA and TCGA cohorts (4.69% versus 7.14%, Fig. 2b).

### Somatic copy-number variations

Using Sequenza [25], we detected 1349 copy number gains and 591 losses across the PTMETA (Fig. 1e). At the chromosomal level, frequent chromosomal deletions of Chr. 1p, 7p, 7q, 8q, 11p, 14q, 16p, and amplifications of 3p, 14q, 16p, and 20q were the major features of the PTMETA cohort using GISTIC2.0 algorithm[26], and the PTMETA cohort had a higher degree of arm-level than the TCGA cohort (Fig. 2c and Additional file 1: Fig S3). We also found some deletions containing reported tumor suppressor genes (e.g., *TNFRSF14* on 1p36, *RECQL4* on 8q24, *NTHL1* on 16p13, *TSC2* on 16p13, and *AXIN1* on 16p13) and, interestingly, they were not found in TCGA (Fig. 2c).

### Mutational signatures

In the integrated analysis of mutational signatures of PTMETA, we identified four principal mutational signatures, Signature A—Signature D, which were similar to COSMIC signatures 5 (Unkown, clock-like and correlating with age), 2 (APOBEC), 1 (Age), and 20 (Defective DNA mismatch repair), respectively (Fig. 2d). We also discovered signatures 16 (Unkown), 3 (Defective homologous recombination-based DNA damage repair), 2 (APOBEC), 1 (Age), and 6 (Defective DNA mismatch repair) in the TCGA cohort (Fig. 2e). The proportions of signatures differed between the cohorts (Fig. 2F), indicating different exposure as well as tumorigenic mechanisms.

### Differential gene expression analysis between tumor and matched adjacent normal tissue

**Fig. 3:**
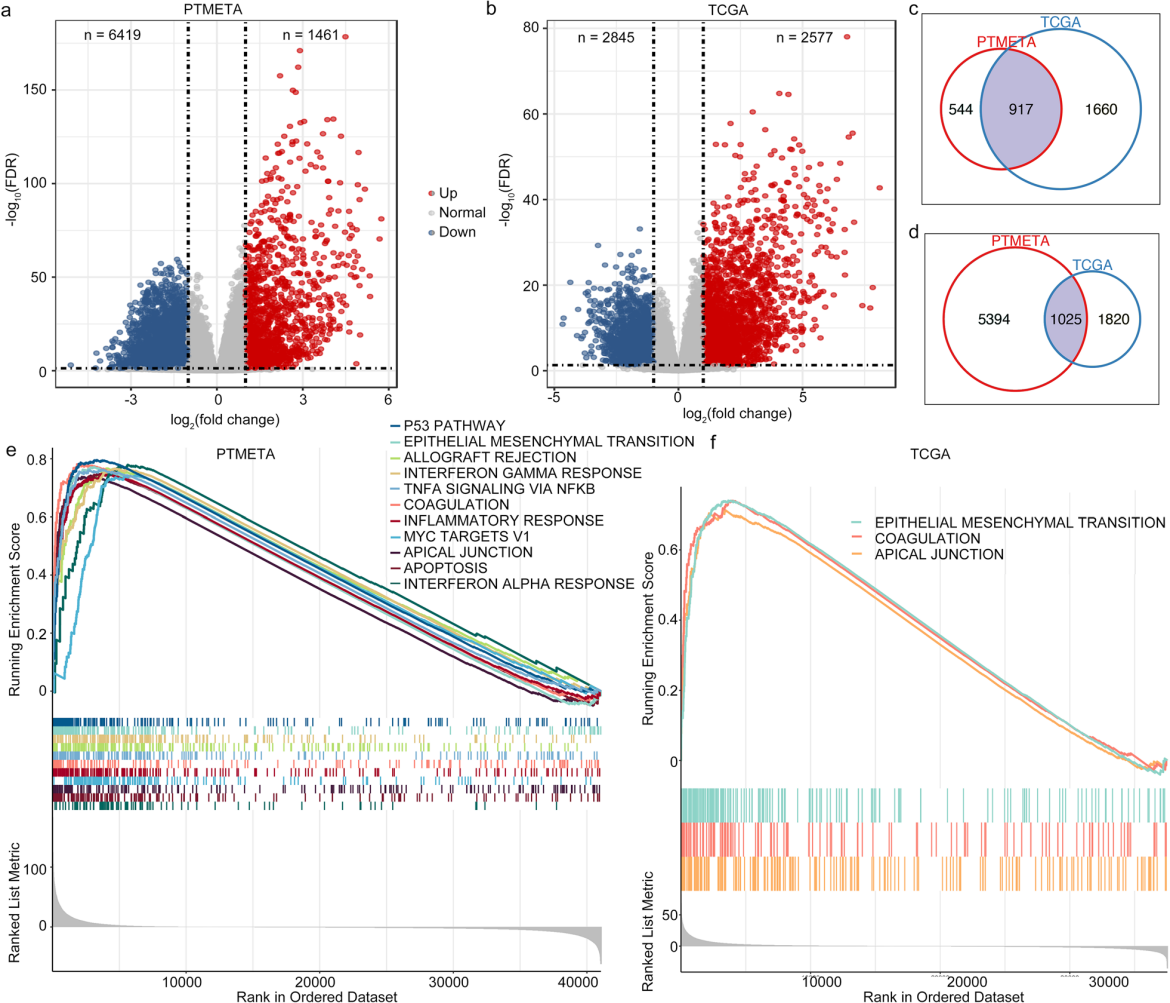
The transcriptome landscape of the PTMETA and TCGA cohort. Volcano plots of differentially expressed genes (DEGs) in the **(a)** PTMETA cohort and **(b)** TCGA cohort. Up- and down-regulated DEGs are plotted in red and blue, respectively; Venn diagrams representation of **(c)** upregulated DEGs in the two cohorts and **(d)** downregulated DEGs in the two cohorts; The Gene Set Enrichment Analysis (GSEA) terms in the **(e)** PTMETA cohort and** (f)** TCGA cohort

Transcriptomic characteristics of PTMC remain largely unknown. Here, we conducted whole-genome RNA sequencing on 64 pairs of tissue samples in PTMETA to identify DEGs between PTMC and matched normal tissues. We also compared our sequencing results to the transcriptomes of 28 pairs of PTMC and adjacent normal tissues in TCGA. PTMETA data showed 7880 DEGs, of which 6419 were upregulated and 1461 were downregulated (Fig. 3a and Additional file 2: Table S5). TCGA presented 5422 DEGs, including 2845 upregulated and 2577 downregulated genes (Fig. 3b and Additional file 2: Table S6). There were 917 upregulated genes and 1025 downregulated genes which were shared by both cohorts (Fig. 3c, d). By GSEA, we discovered that some pathways were activated in both cohorts, such as the epithelial-mesenchymal transition and coagulation, but several pathways were unique in each of the cohorts (Figs. 3e, f). It is interesting to note that, in comparison with TCGA, PTMETA had more immune-related pathways activated.

### A new inflammatory subgroup in the PTMETA

Previously, unsupervised clustering analysis of TCGA transcriptomes identified two major expression clusters in the PTC: *BRAF*-like and *RAS*-like [11]. In this study, we applied the unsupervised clustering method SC3 to analyze PTMETA and PTMC-TCGA transcriptomes. In both cohorts, the SC3 algorithm suggested two groups as the best clustering solutions (Additional file 2: Table S4).

**Fig. 4:**
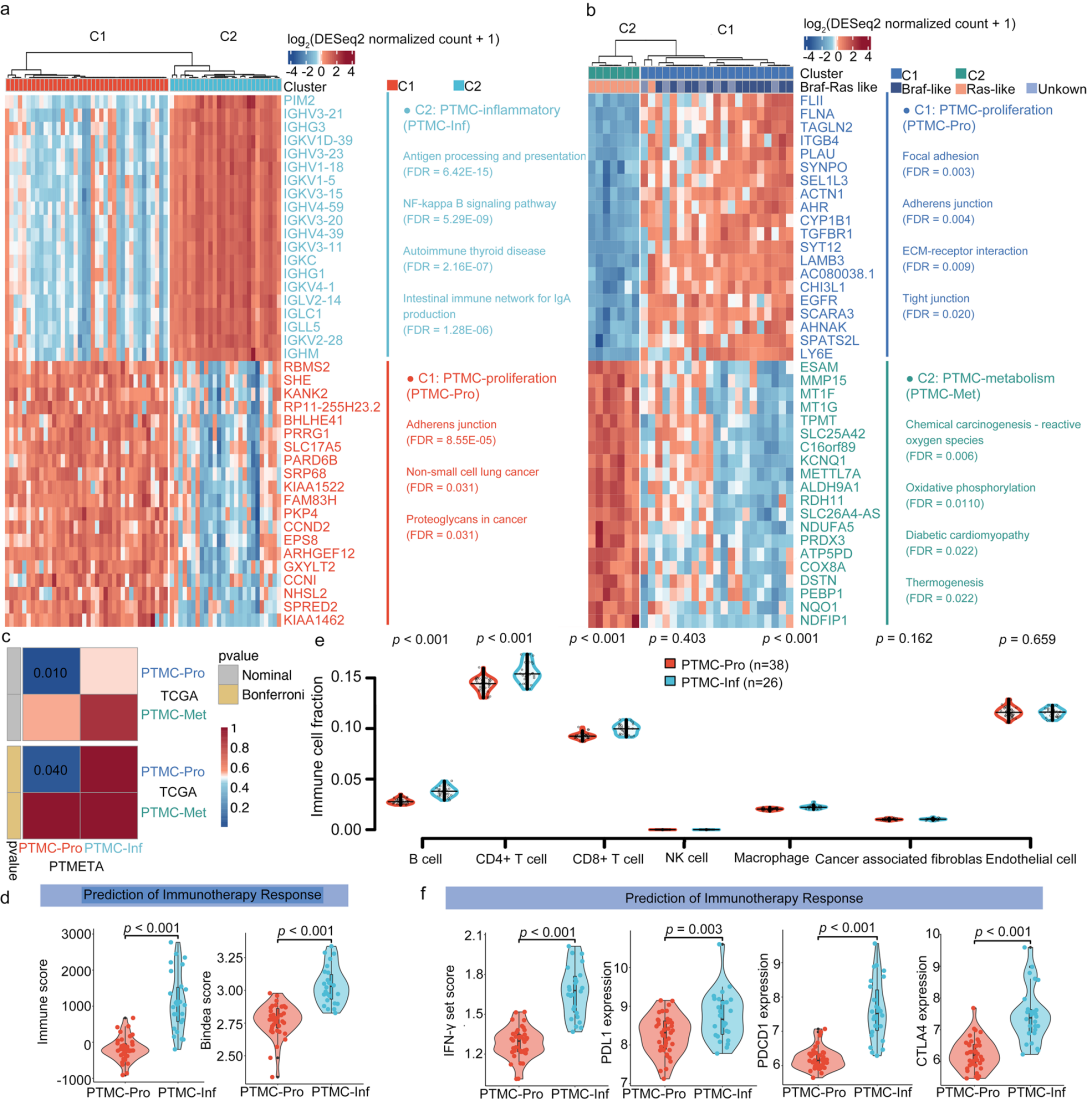
Transcriptomic clusters in the PTMETA and TCGA cohorts. Heatmaps depicting the expression of the top 20 AUROC-ranked marker genes in each subgroup of the **(a)** PTMETA cohort and **(b)** PTMC-TCGA cohort. The main enriched KEGG terms of all marker genes in each subgroup are listed on the right; **(c)** Subclass mapping of subgroups found in the PTMETA and PTMC-TCGA cohort. Significant correspondence between subgroups is highlighted in blue with Bonferroni adjusted p-values; **(d)** Comparison of the tumor immune microenvironment across different subgroups in the PTMETA cohort using two methods: ESTIMATE immune scores, GSVA using Bindea et al.’s combined immune gene set; **(e)** Comparison of the immune cell fraction distinguished by the different subgroups; **(f)** Comparison of IFN-γ scores, *PDL1*, *PDCD1*, and *CTLA4* expression. *p*-values from the Wilcoxon rank-sum tests. PTMC-Pro: PTMC-proliferation; PTMC-Inf: PTMC-inflammatory; PTMC-Met: PTMC-Metabolism

Based on the top 5000 most variable genes, 64 patients in PTMETA were classified into two subgroups: 38 (59.38%) into cluster 1 (C1) and 26 (40.62%) into cluster 2 (C2). Through the SC3 method with an adjusted *p*-value less than 0.05, 285 marker genes for C1 and 257 marker genes for C2 were identified (Additional file 2: Table S7). Each subgroup’s top 20 marker genes were visualized in a heatmap (Fig. 4a). According to enrichment analysis, C1 marker genes were enriched in the adherens junction and proteoglycans, while C2 marker genes were enriched in antigen processing and presentation, autoimmune thyroid disease, and other inflammation-related pathways. These two subgroups were named ‘PTMC-proliferation’ (PTMC-Pro) and ‘PTMC-inflammatory’ (PTMC-Inf), respectively, based on the roles of the marker genes in each subgroup. Twenty-eight PTMC patients in TCGA were classified into two subgroups based on the top 2000 most variable genes: 21 (75.00%) into cluster 1 (C1) and 7 (25.00%) into cluster 2 (C2). The top 20 marker genes for each subgroup were visualized in a heatmap, with C1 enriched in the adherens junction and C2 enriched in the oxygen metabolic related pathway (Fig. 4b). So ‘PTMC-proliferation’ (PTMC-Pro) and ‘PTMC-metabolism’ (PTMC-Met) were the names given to these two subgroups. We noticed that PTMC-Pro was mostly *BRAF*-like and PTMC-Met was *RAS*-like in TCGA, confirming the classification results from the previous analysis [11]. In comparison to the subgroups across cohorts, PTMC-Pro cluster of the two cohorts was strikingly similar, but the new subgroup PTMC-Inf was unique to the PTMETA cohort (Fig. 4c).

Given the differences in immune-related pathways between PTMC-Inf and PTMC-Pro in the PTMETA cohort, we analyzed the immune activity and immune cell types for the two clusters, using four different transcriptome-based computation algorithms: ESTIMATE [37], GSVA using gene sets for immune cells [38], abundances of 7 immune cell inferred by EPIC deconvolution analysis [40], and abundances of 22 immune cell subtypes inferred by CIBERSORT deconvolution analysis [41]. We first found that the PTMC-Inf patients had considerably higher immune score than the PTMC-Pro patients by the ESTIMATE algorithm (Fig. 4d). The GSVA algorithm checked the above results and found higher Bindea score which is related to immune (Fig. 4d). While there were more abundant T-cell subtypes in PTMC-Inf than PTMC-Pro by EPIC (CD4 + T cell and CD8 + T cell, *p* < 0.001; Fig. 4e) and CIBERSORT (T cells CD4 memory activated, *p* < 0.001; T cells CD8, *p* = 0.002; Additional file 1: Fig S4), we also found elevated expression of *PDL1*, *PD1*, *CTLA4*, and IFN-γ in PTMC-Inf, suggesting that patients in PTMC-Inf may be sensitive to immune checkpoint inhibitor (ICI) therapy (*p* = 0.003, < 0.001, < 0.001, < 0.001, respectively; Fig. 4f).

### Integrative analysis of multiple data layers in the PTMETA

**Fig. 5:**
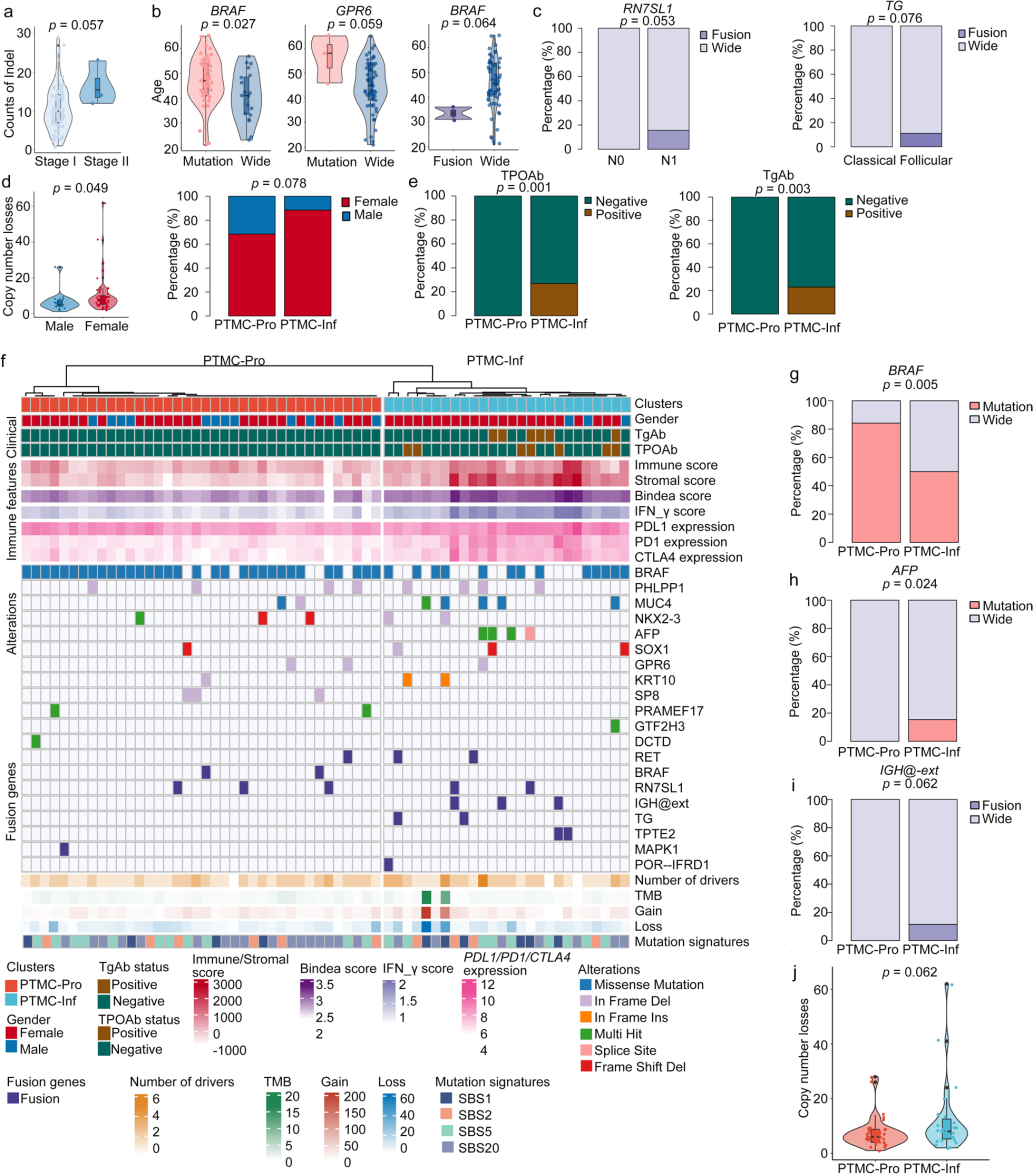
Correlation analysis of clinical, genomic, and transcriptomic features in the PTMETA cohort.** (a – e)** Correlation between molecular and clinical features in the PTMETA cohort. Only correlations with significant (*p* < 0.10) differences calculated by Wilcoxon rank-sum tests or Fisher’s exact tests are shown; **(f)** Phenotypes of transcriptomic subgroups. The top rows indicate the cluster assignment of patients. The following rows show the clinical, immune features, alterations, fusion genes, number of drivers, TMB, copy number gains, copy number losses, and mutation signatures, respectively; **(g –h)** Genomic features of transcriptomic subgroups. Only correlations with significant (*p* < 0.10) differences calculated by Wilcoxon rank-sum tests or Fisher’s exact tests are shown. PTMC-Pro: PTMC-proliferation; PTMC-Inf: PTMC-inflammatory

We decided to present the association between clinical features and molecular features with a higher threshold (*p* < 0.10) in order to find more potential clinically relevant biomarkers. Correlating molecular features with clinical variables in PTMETA, we found that Indel happened more frequently in stage II (*p* = 0.057; Fig. 5a). *BRAF* and *GPR6* mutations were more common in older patients, whereas *BRAF* fusions were more common in younger patients (*p* = 0.027, 0.059, 0.064, respectively; Fig. 5b). In addition, *RN7SL1* fusions were also linked to lymph node metastasis, and *TG* fusions were related to follicular-variant tumors (*p* = 0.053, 0.076, respectively; Fig. 5c). Females showed larger copy number losses, and PTMC-Inf was enriched in females (*p* = 0.049, 0.078, respectively; Fig. 5d). It is important to note that TPOAb-positive patients and TgAb-positive patients were only present in the PTMC-Inf (*p* = 0.001, 0.003, respectively; Fig. 5e).

We also evaluated the relationship between genomic features and transcriptome clusters in PTMETA (Fig. 5f). *AFP* mutations, *IGH@ext* fusions, *TG* fusions, and *TPTE2* fusions occurred only in PTMC-Inf, while *SP8* mutations and *BRAF* fusions were only in PTMC-Pro. *BRAF* mutations were enriched in PTMC-Pro (*p* = 0.005; Fig. 5g), while *AFP* mutations and *IGH@ext* fusions were enriched in PTMC-Inf (*p* = 0.024, 0.062, respectively; Fig. 5h, i). At the same time, network analysis found that *AFP* was associated with several immune-related genes in the PTMC-Inf (Additional file 1: Fig S5a, b), and several IGH@ext-related genes were also highly expressed in this subgroup (Additional file 1: Fig S5c), suggesting that variations of these two genes may lead to the activation of immune characteristics in the PTMC-Inf. Moreover, PTMC-Inf had more copy number losses (*p* = 0.062; Fig. 5j).

### Establishment and validation of a prediction model for molecular signatures

**Fig. 6:**
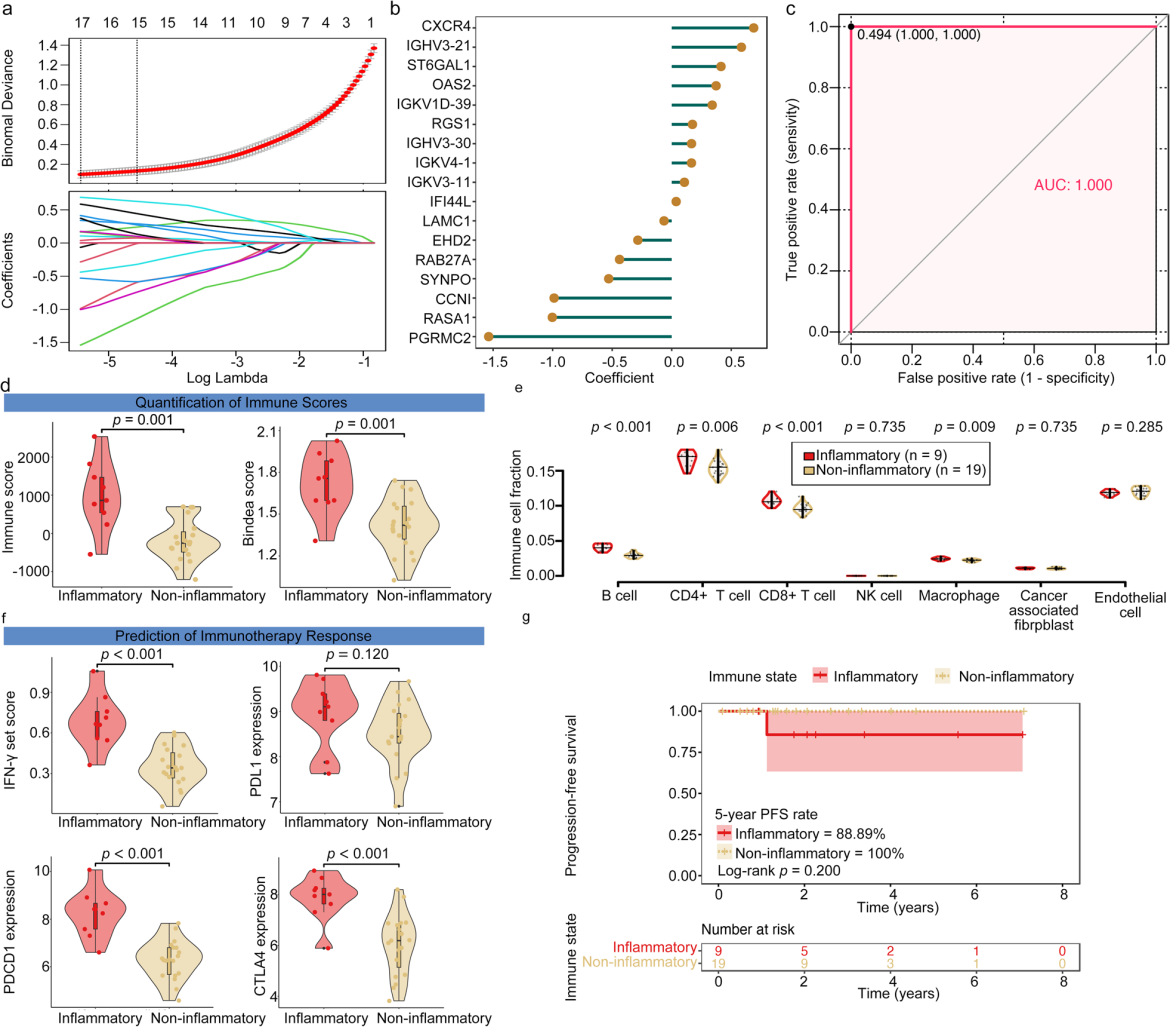
A diagnostic prediction model for subgroups was developed and validated via the LASSO regression. **(a)** In the PTMETA cohort (n = 64), the determination of the optimal λ was obtained when the binomial deviance reached the minimum value, and further generated LASSO coefficients of the most useful marker genes; **(b)** Coefficients of 17 marker genes finally obtained in LASSO regression; **(c)** Receiver operating characteristic (ROC) curves and the associated areas under curves (AUCs) of the diagnostic prediction model in the PTMETA cohort; **(d)** Comparison of the tumor immune microenvironment across different immune state according to the diagnostic prediction model in the PTMC-TCGA cohort using two methods: ESTIMATE immune scores, GSVA using Bindea et al.’s combined immune gene set; **(e)** Comparison of the immune cell fraction distinguished by the different immune state in the PTMC-TCGA cohort; **(f)** Comparison of IFN-γ scores, *PDL1*, *PDCD1*, and *CTLA4* expression by the different immune state in the PTMC-TCGA cohort. *p*-values from the Wilcoxon rank-sum tests; **(g)** Kaplan–Meier curves of PFS according to the diagnostic prediction model in the PTMC-TCGA cohort. *p*-value from the Log-rank test

Since there were notable variations in immunotherapy responses between subtypes, we wondered if subtype marker genes could serve as indicators of precise immune intervention. Based on the expression profiles of 542 marker genes for molecular signatures in PTMETA, we used the LASSO logistic regression analysis to build a prediction model for molecular signatures. In the LASSO regression, the optimal λ was obtained when the binomial deviance reached the minimum value (Fig. 6a). There were 17 gene markers in the final multinomial regression model. We used these markers and their coefficients to generate a risk score for each patient in PTMETA (Fig. 6b). An optimal cutoff was used for the risk score to classify patients into two groups of molecular signatures, inflammatory versus non-inflammatory subtypes. To assess its validity in PTMETA, we compared the model-generated classification with the initial unsupervised clusters to determine its validity in PTMETA and found a good concordance between the two methods on subgroup classification (AUC = 1) when using a cutoff value at 0.494 based on the Youden index [52] (Fig. 6c). We calculated the risk score for patients in the PTMC-TCGA cohort to further confirm the effectiveness of our model and demonstrate that PTMC-inflammatory patients also exist in other ethnic groups, such as the European population in TCGA. Of the 28 patients, 9 were classified as inflammatory and 19 were non-inflammatory using the same cut-off value. To evaluate the clinical significance of our classification, we compared the characteristics of tumor immune microenvironment between the two groups of PTMC-TCGA patients and discovered that patients with inflammatory signature had higher immune scores (Fig. 6d), more CD8 + T cells (Fig. 6e), and higher expression of immune checkpoint proteins (Fig. 6f), indicating that the prediction model may classify patients with different immunity. The PTMC-TCGA cohort was then subjected to a survival analysis, and although there was no statistically significant difference in PFS between the two groups, the 5 year PFS rate was lower in patients with inflammation than in patients without inflammation (88.89% & 100%; Log-rank *p* = 0.200; Fig. 6g). We also performed model validation and survival analysis in the Early-Stage-PTC-TCGA (ESPTC-TCGA; Stage I and Stage II; 331 patients) cohort due to the smaller sample size of the PTMC-TCGA cohort, the lack study of the PTMC cohort, and the improved PFS of the PTMC [53]. We found that patients in the ESPTC-TCGA cohort predicted by the model to be subtypes of inflammation also showed higher immune scores (Additional file 1: Fig S6a), more abundant T-cell subtypes (Additional file 1: Fig S6b), and higher expression of immune checkpoint proteins (Additional file 1: Fig S6c). Log-rank test results indicated that inflammatory patients had significantly worse prognosis for PFS (Log-rank *p* = 0.034; Additional file 1: Fig S6d).

## Discussion

**Fig. 7:**
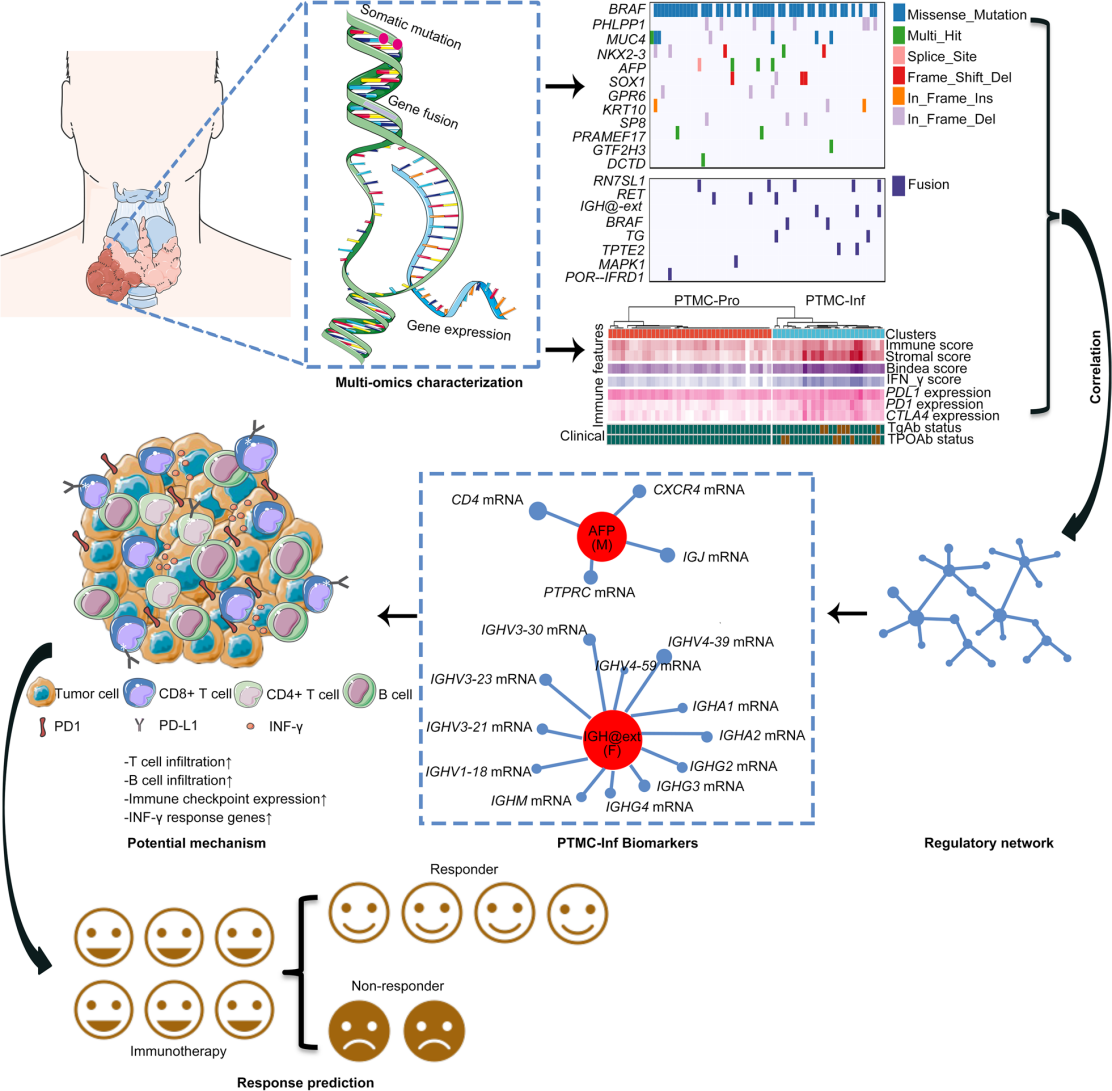
Schematic plot of multi-step PTMC-Inf discovery in the PTMETA cohort. PTMC-Inf: PTMC-inflammatory

To our knowledge, PTMETA is the largest multi-omics study on papillary thyroid microcarcinomas, and we found a new subgroup named PTMC-Inf with potential implications in immunotherapy. This finding deepens our understanding of the PTMC. When compared to the PTMC cohort in TCGA, we found that the PTMETA cohort had more unstable genomes manifested by multiple genomic alterations, as well as the activation of distinct immune-related pathways in the transcriptome. The PTMETA cohort had a unique inflammatory subgroup that showed possible responsiveness to immune intervention. *AFP* mutations and *IGH@ext* fusions were found only in the PTMC-Inf subgroup, suggesting that these mutations may be used as biomarkers to predict the effect of the immune intervention (Fig. 7). In addition, we created and validated a prediction model for PTMC subgroups and found that inflammatory patients had a lower rate of 5 year PFS.

In the present study, we found similar genomic and transcriptomic features between PTMETA and PTMC-TCGA which also did not show appreciable variations in clinical characteristics (Additional file 2: Table S3). Compared to other malignancies, PTMC in these cohorts displayed a low somatic mutation density [54] (Fig. 2a). Notably, the most common somatic changes in both groups were *BRAF* mutations, which are typically observed in PTC patients [55] and PTMC patients [56]. Additionally, *BRAF* fusions and *RET* fusions were found in both cohorts, and activation of epithelial to mesenchymal transition (EMT) was also shown in both transcriptomes, all of which are typical features of thyroid cancer [55, 57]. Finally, unsupervised cluster analysis revealed a subgroup characterized by proliferative pathway activation in both cohorts, and most of the patients in this subgroup had *BRAF* mutations, which was consistent with the results of a previous study [11]. These observations support the validity of our multi-omic analysis.

Interestingly, we also made several intriguing findings. First, the *BRAF* mutation rate was higher in PTMETA than in TCGA (70.3% & 57.1%, Fig. 2b), which was consistent with the finding of a previous study that also compared the mutation rates between China and TCGA (72.4% & 59.7%) [12]. Parvathareddy et al. showed *BRAF*^*V600E*^ mutations were detected in 45.7% (84/184) Middle Eastern PTMCs, which was less than the PTMETA cohort [58]. Iodine-rich diets or chronic thyroiditis were suspected to be possible reasons for high *BRAF*^*V600E*^ mutations in Chinese PTC [59]. Second, it is interesting to note that there are several driver genes detected in PTMETA by WES not targeted next-generation sequencing that have not been reported in either PTMC-TCGA (Fig. 2b) or TCGA-PTC [11] or other PTMC patients [60]. Among them, *PHLPP1*, a tumor suppressor gene for various cancer types [61], has the second highest mutation frequency in PTMETA (12.5%). *PHLPP1* directly dephosphorylates *AKT* to inhibit the Akt serine–threonine kinase and protein kinase C (PKC) signaling. All *PHLPP1* mutations in this study were in-frame deletions (Additional file 1: Fig S2), which could lead to functional defects. *NRAS* mutations were the second highest mutated gene (7.1%) in the PTMC-TCGA cohort, but this high frequency was not found in the PTMETA cohort. *NRAS* mutations were less common in Chinese PTC (0–3%) [12], which is consistent with our study. While 4.7% of 431 PTMCs were found to have *TERT* gene alterations, according to de Biase et al. [62]. Since the *TERT* gene mutation lies in the promoter and cannot be detected by WES, we did not analyze it in our study. Finally, the PTMETA cohort had a high frequency of CNVs. Radiation has been demonstrated in several studies to cause DNA replication and mismatch repair disorders, resulting in CNVs [63]. We also found that the PTMETA cohort had more mutation signature 20 (Defective DNA mismatch repair, Fig. 2f) than did the PTMC-TCGA cohort. Defective DNA mismatch repair leads to microsatellite instability and a high frequency of mutation [64]. We speculate that the difference in CNVs between the two cohorts is attributable to some environmental factors and racial differences since there were no significant differences in clinical features between the cohorts.

We also found a group of driver genes related to immunity in the PTMETA cohort, including *AFP* mutations, *IGH@-ext* fusions, *TPTE2* fusions, and *IFRD1* fusions (Fig. 2b). The PTMC-TCGA cohort didn’t show similar results (Fig. 2b). Furthermore, bioinformatic analysis of DEGs revealed that the PTMETA cohort had functional enrichment in the activation of immune-related pathways, supporting the finding of driver genes’ relation to immunity. These genes have been reported to be associated with immune responses and play distinct roles in different types of cancer [65–68]. With these unique genetic and molecular changes, our cluster analysis revealed a distinct subgroup of patients with activated immune cell signaling and interferon-γ response (Fig. 4a). By the study by Chen et al., we named this subgroup ‘PTMC-Inf’ [69]. By causing DNA damage, inflammation can start and foster tumorigenesis in inflammation-related tumorigenesis [70]. Inflammation can also hasten the development of cancer by accelerating the growth of cancer cells, reducing their immunogenicity, and avoiding immune destruction [71]. For instance, IFN-γ upregulates the expression of T cell depletion, stimulates STAT3 signaling, protects epithelial cells from CD8 + T cytotoxic cytokinesis, and promotes PD-L1 on the altered epithelial cells identified by T cells [72]. The network analysis showed that *AFP* and *IGH*@ext genes may be the hub genes leading to the emergence of PTMC-Inf (Additional file 1: Fig S5). It has been demonstrated that *AFP* is particularly useful for predicting how responsive hepatocellular carcinoma patients would be to ICI therapy [65]. A search of COSMIC [73] showed that *AFP* mutations are present in numerous cancers, including thyroid cancer, even though they have not been thoroughly investigated. A feature of chronic lymphocytic leukemia is *IGH@ext* fusions [74]. Additionally, a recent study revealed that individuals with colorectal cancer may have changes in their tumor immune microenvironment due to an Asia-specific variation of the IGHG1 gene [75]. The antigen-specific humoral immune responses induced by TPOAb and TgAb are considered to be associated with the development and prognosis of PTC [76]. Of note, TPOAb-positive patients and TgAb-positive patients were found only in the PTMC-Inf, which proves that these patients did have an immune disorder. Previous studies have shown that cancers defined as ‘hot tumors’ (many infiltrating T cells and high expression of *PD-L1*/*PDCD1*) [77] are more responsive to ICI treatment. Our analysis of immune cell subtypes and immune checkpoint gene expression revealed that the PTMC-Inf tumor shares the same characteristics (more infiltrating CD4 + and CD8 + T cells, and high expression of *PDL1*, *PDCD1*, and *CTLA4*, Fig. 4d–f). Anti-*CTLA-4* or anti-*PD-1* therapy is dependent on T-cell infiltration, hence these treatments may only be effective in hot tumors [78]. In clinics, the effects of *PD-L1* inhibitors, such as pembrolizumab, on thyroid cancer have been actively investigated in recent years, but the number of patients who responded to the treatment was small [79], suggesting that immunotherapy may have limited effects on the cancer [80]. This may also indicate that biomarkers are needed to predict which patients are responsive to ICI. Based on the expression of 17 marker genes, we could divide PTMETA patients into two molecular subgroups, inflammatory versus non-inflammatory. Further validation of the model classification in the PTMC-TCGA cohort revealed that the prediction model could identify patients with possible differences in immunity. Due to the small sample size, we only found that the 5 year PFS rate in the PTMC cohort was lower in patients with inflammation than in patients without inflammation. However, in a larger PTC cohort, we found that the survival of patients with inflammation was significantly worse than that of patients with non-inflammation, suggesting that patients with inflammation may improve their prognosis through immunotherapy. In addition, although PTMC patients had favorable PFS, which is consistent with previous studies [53], we still discovered that inflammatory patients have a worse prognosis for PFS, whether in the small sample size PTMC-TCGA cohort (Fig. 6) or in the large sample size ESPTC-TCGA cohort (Additional file 1: Fig S6), indicating that PTMC is not homogeneous in disease outcomes and that some inflammatory patients may be responsive to immunotherapy. In addition, we found *AFP* mutations, *IGH@ext* fusions, *TPTE2* fusions, and *IFRD1* fusion not only in Chinese PTMC but predominantly in the PTMC-Inf (Fig. 5e), suggesting that these characteristics may be used to select patients who are responsive to ICI to improve the treatment response. Aside from that, research has shown that patients who receive immunotherapy early have better disease control and favorable prognosis [81, 82], implying that treating early-stage cancers like PTMC has potential benefits. As a result, ICI treatment for PTMC-Inf patients is biologically relevant.

One of the limitations of our investigation is the lack of treatment and survival data due to the short follow-up time, making it difficult to assess the actual value of the biomarkers in clinical application. In addition, although TCGA had survival information, the number of PTMC patients was too small to have enough power to find significant differences in survival between inflammatory and non-inflammatory patients. Finally, bioinformatic analyses are not able to deeply elucidate the molecular mechanisms, experimental evidence is indispensable to further exploration.

## Conclusion

Taken together, we divided PTMC patients into two subtypes with clinicopathological features, genomic alterations, gene expressions, immune microenvironment patterns, and immunotherapeutic responses. In addition, a molecular prediction model was proposed for individualized integrative assessment. These provides new insights into the precise intervention of PTMC. Our results shed light on the understanding of molecular signatures in PTMC and offer fresh perspectives on the molecular mechanism for future research and relevant immunotherapy in PTMC.

## Supplementary Information

**Additional file 1: Fig S1.** Sanger sequencing validation of the PCR products of 9 fusions in the PTMETA cohort, respectively. **Fig S2.** Lollipop plot of the somatic mutations of the driver genes identified in the PTMETA cohort (a) and TCGA cohort (b). For each driver gene, a lollipop plot was generated depicting all amino acid changes found along the protein (grey bar) with their frequencies in the PTMETA cohort and TCGA cohort (the height). Protein motifs were shown with coloured boxes. **Fig S3.** Heat map showing somatic CNAs with estimated actual copy numbers between the PTMETA cohort and TCGA cohort. Red represents amplification and blue represents deletion. **Fig S4.** CIBERSORT analysis of PTMETA samples. Highlighted boxes indicate immune cell types that were significantly enriched in either PTMC-proliferation or PTMC-inflammatory relative to the other using a wilcoxon rank-sum test (p < 0.05). Outliers not shown. The boxes in box plots indicate 25th percentile, median, and 75th percentile, while whiskers show the maximum and minimum values within 1.5 times the inter-quartile range from the edge of the box. **Fig S5.** Identification of hub driver genes. a, A PPI network made up of the driver mutation genes, fusion genes, and top 100 marker genes for each subgroup. The PTMC-proliferation, PTMC-inflammatory, and driving genes are represented by the red, green, and yellow nodes, respectively. b, The expression level of PTMC-inflammatory marker genes (CD4, CXCR4, IGJ, and PTPRC) associated with AFP as a function of AFP mutation type. c, The expression levels of PTMC-inflammatory marker genes IGH@ext (IGHA1, IGHA2, IGHG2, IGHG3, IGHG4, IGHM, IGHV1-18, IGHV3-21, IGHV3-23, IGHV3-30, IGHV4-39, and IGHV4-59) as a function of IGH@ext fusion type. p values determined by the wilcoxon rank-sum test. **Fig S6.** The diagnostic prediction model for subgroups was validated using the ESPTC-TCGA cohort. a, Comparison of the tumor immune microenvironment across different immune state according to the diagnostic prediction model in the ESPTC-TCGA cohort using two methods: ESTIMATE immune scores, GSVA using Bindea et al.’s combined immune gene set. b, Comparison of the immune cell fraction distinguished by the different immune state in the ESPTC-TCGA cohort. c, Comparison of IFN-γ scores, PDL1, PDCD1, and CTLA4 expression by the different immune state in the PTMC-TCGA cohort. p-values from the Wilcoxon rank-sum tests. d, Kaplan–Meier curves of PFS according to the diagnostic prediction model in the ESPTC-TCGA cohort. p-value from the Log-rank test.

**Additional file 2: Table S1.** PTMETA cohort clinical data. **Table S2.** TCGA cohort clinical data. **Table S3.** Comparison of clinical data between PTMETA and TCGA. **Table S4.** Average silhouette width plots of the cancer subgroups for the PTMETA cohort and TCGA cohort. **Table S5.** Differential expression genes between PTMETA tumor samples and normal samples. **Table S6.** Differential expression genes between TCGA tumor samples and normal samples. **Table S7.** Maker genes in the two PTMETA subgroups.

## Data Availability

The datasets (raw data) generated in this study are available through the Genome Sequence Archive (GSA), BioProject ID: PRJCA013817, accession ID: HRA003678. Public data used in this work can be acquired from Genomic Data Commons (https://portal.gdc.cancer.gov), Tumor Fusion Gene Data Portal (https://www.tumorfusions.org/), R package “TCGAbiolinks (v.2.20.1)”, and Memorial Sloan Kettering Cancer Center cBioPortal (http://www.cbioportal.org/public-portal/study.do?cancer_study_id=thca_tcga).

## Abbreviations

PTMC: Papillary thyroid microcarcinoma
TC: Thyroid cancer
ATA: American Thyroid Association
AS: Active surveillance
PTC: Papillary thyroid cancer
PTMETA: Papillary Thyroid Microcarcinoma Exome and Transcriptome Atlas
TPOAb: Thyroid peroxidase antibody
TgAb: Thyroglobulin antibody
RNA-seq: RNA sequencing
RIN: RNA integrity number
WES: Whole exome sequencing
GRCh38: Genome reference consortium build 38
GATK4: Genome analysis toolkit 4
PoNs: Panel of normal
VCF: Variant call format
MAF: Mutation annotation format
TMB: Tumor mutation burden
Mb: Megabase
COSMIC: Catalogue of Somatic Mutations in Cancer
FFPM: Fusion fragments per million
SMGs: Significantly mutated genes
DEGs: Differentially expressed genes
GSEA: Gene set enrichment analysis
NES: Normalized enrichment score
SC3: Single-cell consensus clustering
SD: Standard deviations
KEGG: Kyoto Encyclopedia of Genes and Genomes
GSVA: Gene set variation analysis
ROC: Receiver-operating-characteristics
AUC: Area under the receiver-operating-characteristics curve
PFS: Progression-free survival
C1: Cluster 1
C2: Cluster 2
SNVs: Single-nucleotide variants
Indels: Insertions/deletions
PTMC-Pro: PTMC-proliferation
PTMC-Inf: PTMC-inflammatory
PTMC-Met: PTMC-metabolism
EMT: Epithelial to mesenchymal transition
PKC: Protein kinase C
ICI: Immune checkpoint inhibitor
