# Supplementary material for: Multi-omics profiling of papillary thyroid microcarcinoma reveals different somatic mutations and a unique transcriptomic signature
Source: J Transl Med. 2023 Mar 20;21:206. doi: 10.1186/s12967-023-04045-2 (PMC10026500; doi:10.1186/s12967-023-04045-2)
Supplement: Supplementary file 1 — Additional file 1: Fig S1. Sanger sequencing validation of the PCR products of 9 fusions in the PTMETA cohort, respectively. Fig S2. Lollipop plot of the somatic mutations of the driver genes identified in the PTMETA cohort (a) and TCGA cohort (b). For each driver gene, a lollipop plot was generated depicting all amino acid changes found along the protein (grey bar) with their frequencies in the PTMETA cohort and TCGA cohort (the height). Protein motifs were shown with coloured boxes. Fig S3. Heat map showing somatic CNAs with estimated actual copy numbers between the PTMETA cohort and TCGA cohort. Red represents amplification and blue represents deletion. Fig S4. CIBERSORT analysis of PTMETA samples. Highlighted boxes indicate immune cell types that were significantly enriched in either PTMC-proliferation or PTMC-inflammatory relative to the other using a wilcoxon rank-sum test (p < 0.05). Outliers not shown. The boxes in box plots indicate 25th percentile, median, and 75th percentile, while whiskers show the maximum and minimum values within 1.5 times the inter-quartile range from the edge of the box. Fig S5. Identification of hub driver genes. a, A PPI network made up of the driver mutation genes, fusion genes, and top 100 marker genes for each subgroup. The PTMC-proliferation, PTMC-inflammatory, and driving genes are represented by the red, green, and yellow nodes, respectively. b, The expression level of PTMC-inflammatory marker genes (CD4, CXCR4, IGJ, and PTPRC) associated with AFP as a function of AFP mutation type. c, The expression levels of PTMC-inflammatory marker genes IGH@ext (IGHA1, IGHA2, IGHG2, IGHG3, IGHG4, IGHM, IGHV1-18, IGHV3-21, IGHV3-23, IGHV3-30, IGHV4-39, and IGHV4-59) as a function of IGH@ext fusion type. p values determined by the wilcoxon rank-sum test. Fig S6. The diagnostic prediction model for subgroups was validated using the ESPTC-TCGA cohort. a, Comparison of the tumor immune microenvironment across different immune state according t [file 12967_2023_4045_MOESM1_ESM.docx]

**Multi-omics profiling of papillary thyroid microcarcinoma reveals different somatic mutations and a unique transcrptomic signature**

Qiang Li et al.

Additional file 1: Figures


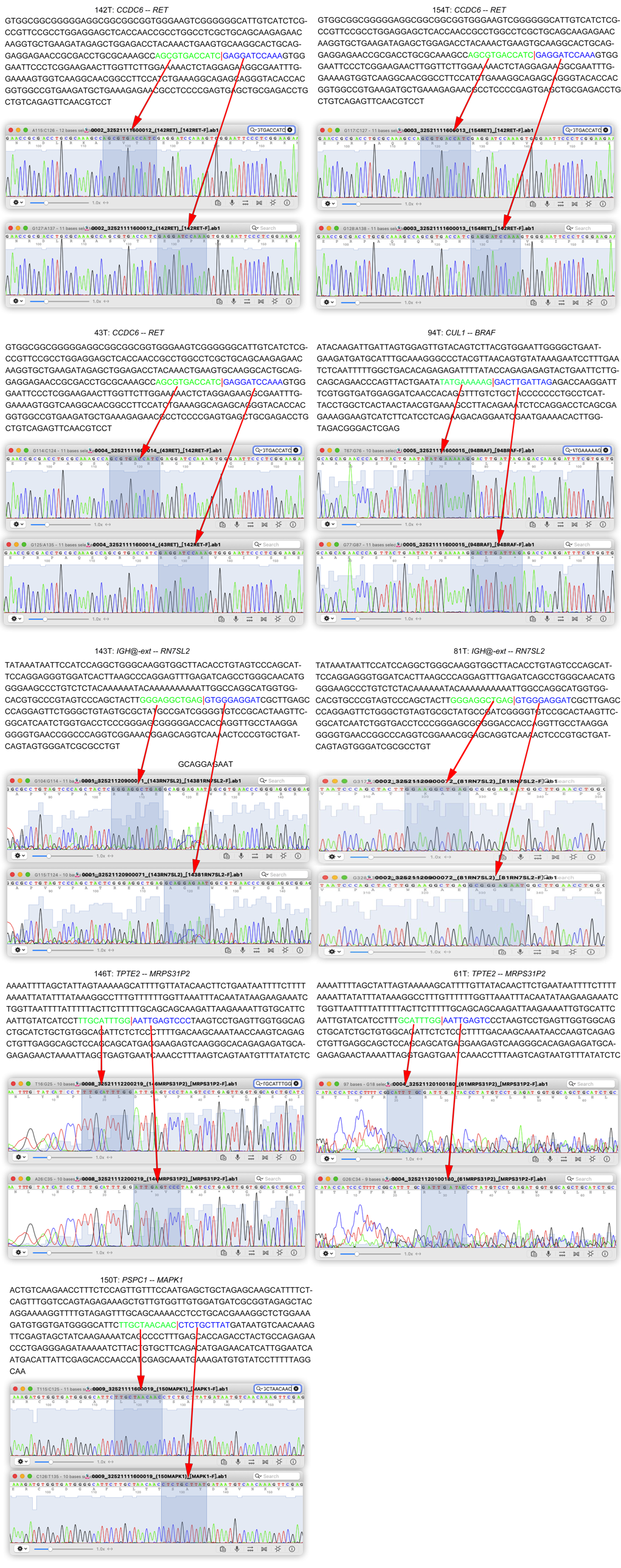


**Figure S1: Sanger sequencing validation of the PCR products of 9 fusions in the PTMETA cohort, respectively.**


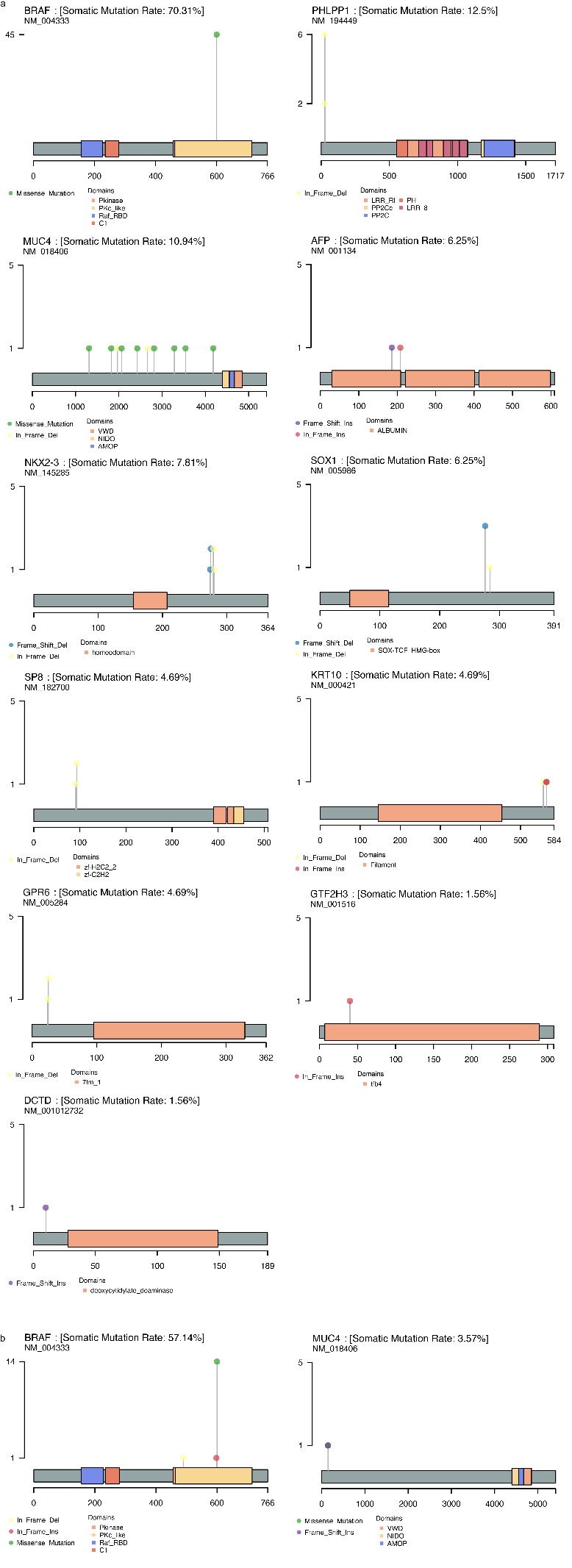


**Figure S2: Lollipop plot of the somatic mutations of the driver genes identified in the PTMETA cohort (a) and TCGA cohort (b).** For each driver gene, a lollipop plot was generated depicting all amino acid changes found along the protein (grey bar) with their frequencies in the PTMETA cohort and TCGA cohort (the height). Protein motifs were shown with coloured boxes.


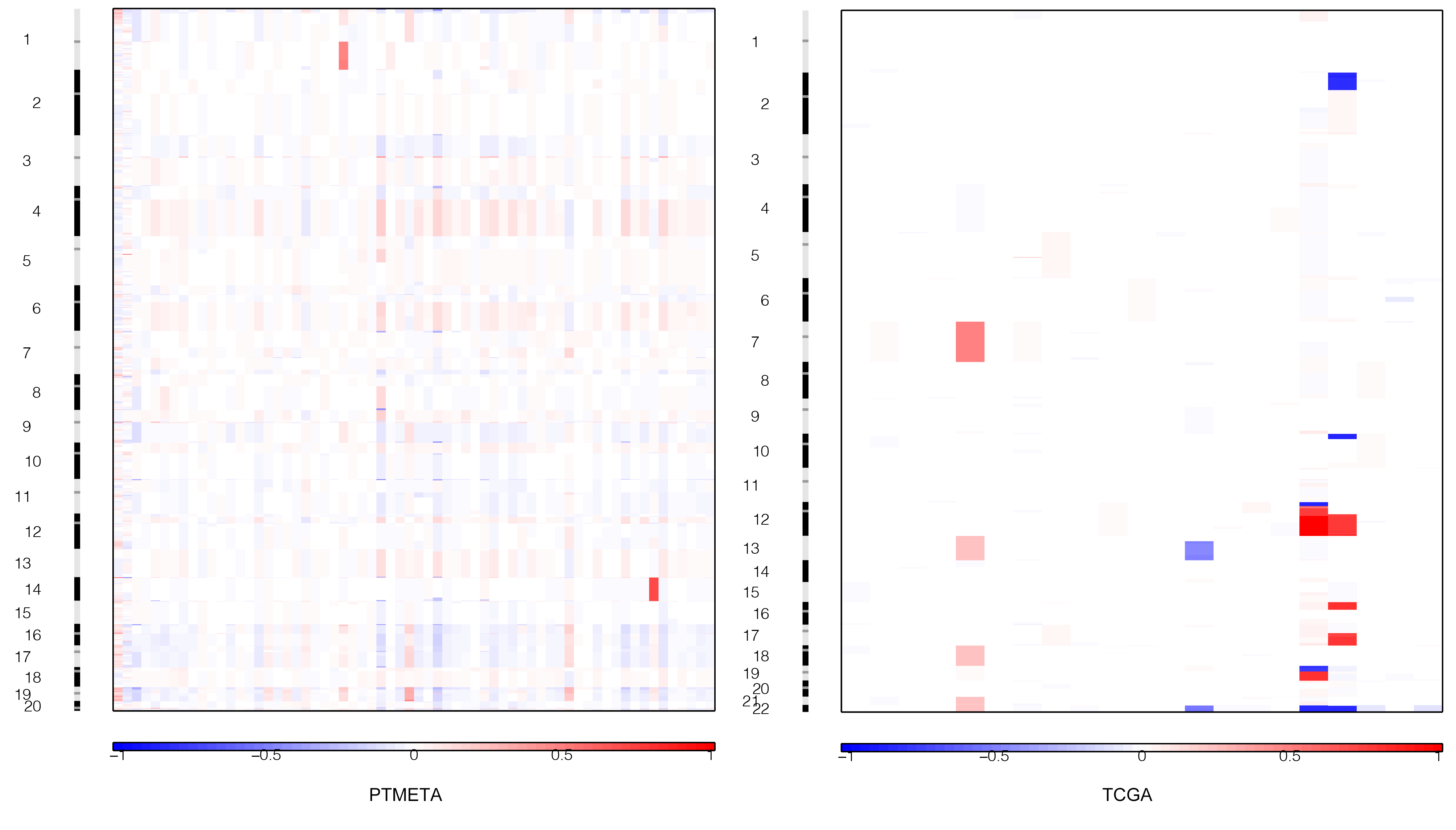


**Figure S3: Heat map showing somatic CNAs with estimated actual copy numbers between the PTMETA cohort and TCGA cohort.** Red represents amplification and blue represents deletion.

**
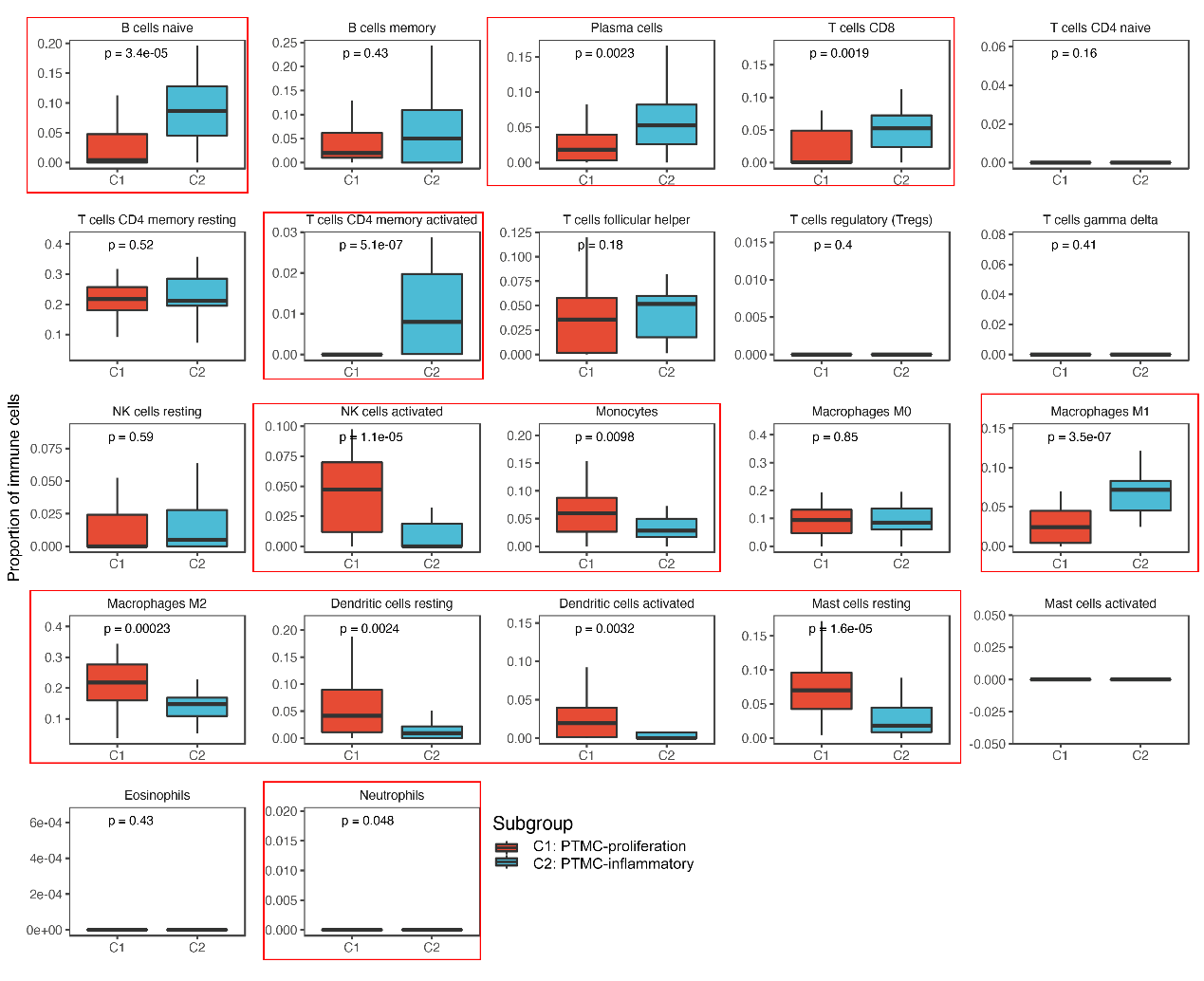
**

**Figure S4: CIBERSORT analysis of PTMETA samples.** Highlighted boxes indicate immune cell types that were significantly enriched in either PTMC-proliferation or PTMC-inflammatory relative to the other using a wilcoxon rank-sum test (*p* < 0.05). Outliers not shown. The boxes in box plots indicate 25th percentile, median, and 75th percentile, while whiskers show the maximum and minimum values within 1.5 times the inter-quartile range from the edge of the box.


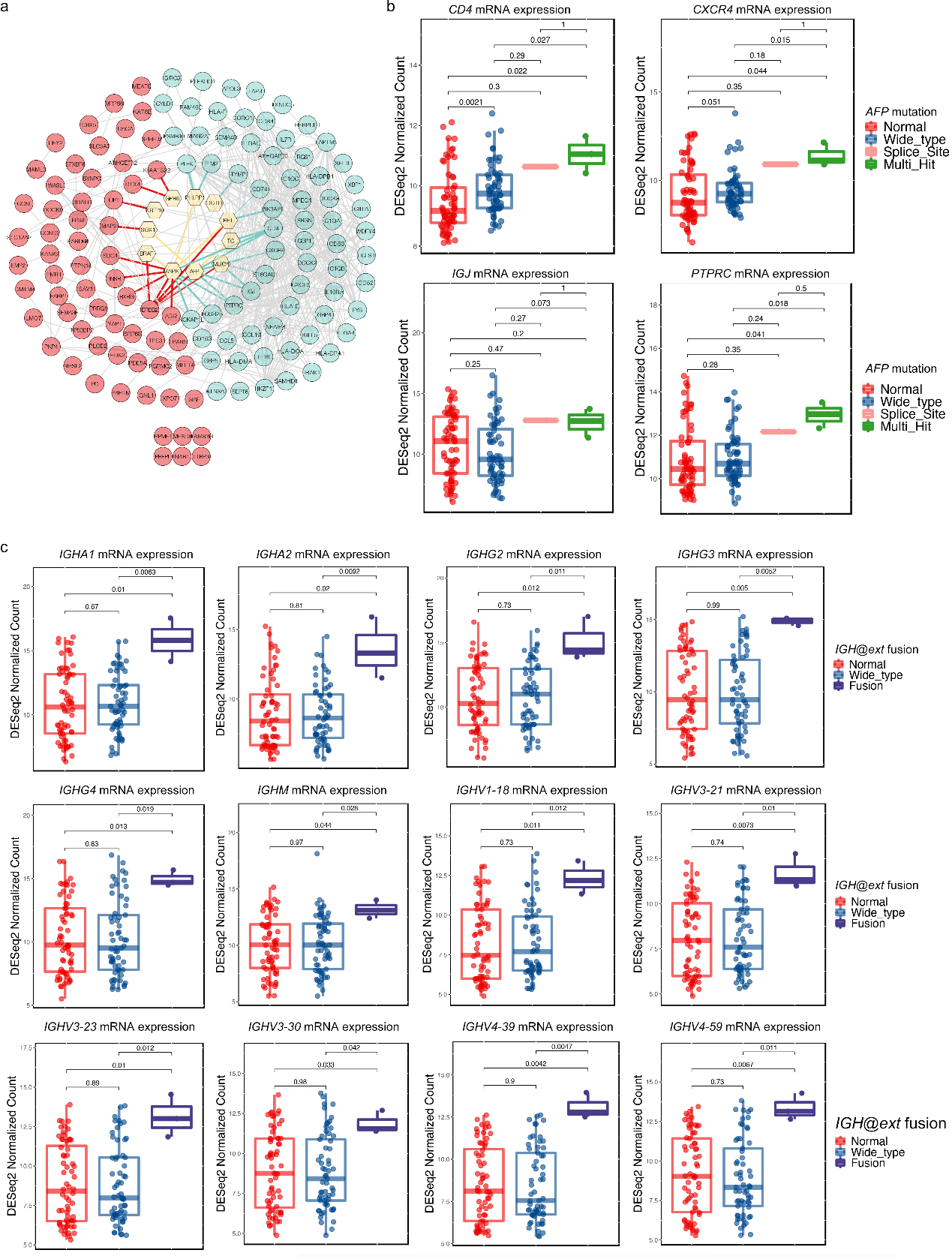


**Figure S5: Identification of hub driver genes.** a, A PPI network made up of the driver mutation genes, fusion genes, and top 100 marker genes for each subgroup. The PTMC-proliferation, PTMC-inflammatory, and driving genes are represented by the red, green, and yellow nodes, respectively. b, The expression level of PTMC-inflammatory marker genes (*CD4*, *CXCR4*, *IGJ*, and *PTPRC*) associated with *AFP* as a function of *AFP* mutation type. c, The expression levels of PTMC-inflammatory marker genes *IGH@ext* (*IGHA1*, *IGHA2*, *IGHG2*, *IGHG3*, *IGHG4*, *IGHM*, *IGHV1-18*, *IGHV3-21*, *IGHV3-23*, *IGHV3-30*, *IGHV4-39*, and *IGHV4-59*) as a function of *IGH@ext* fusion type. *p* values determined by the wilcoxon rank-sum test.


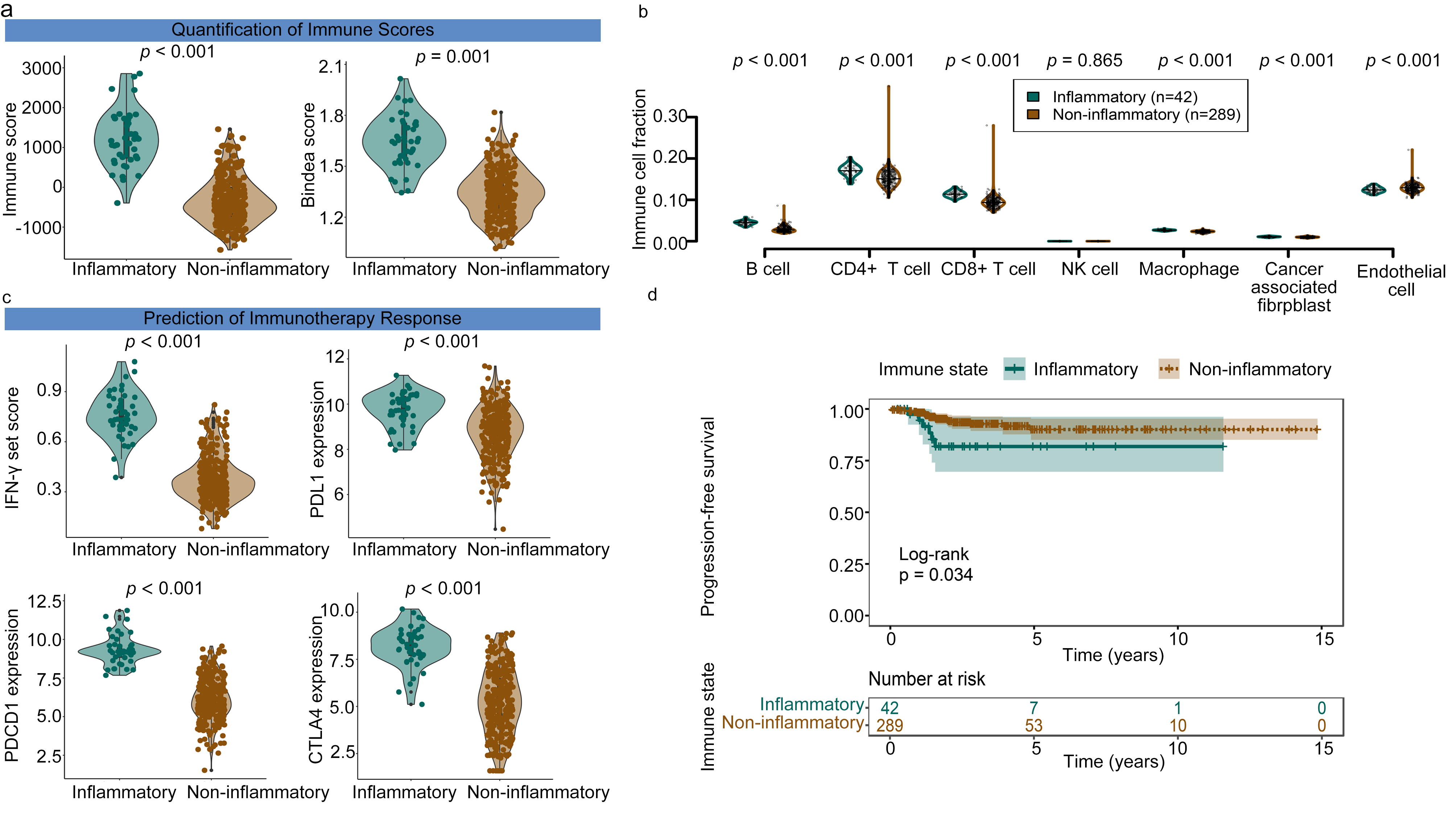


**Figure S6: The diagnostic prediction model for subgroups was validated using the ESPTC-TCGA cohort.** a, Comparison of the tumor immune microenvironment across different immune state according to the diagnostic prediction model in the ESPTC-TCGA cohort using two methods: ESTIMATE immune scores, GSVA using Bindea et al.’s combined immune gene set. b, Comparison of the immune cell fraction distinguished by the different immune state in the ESPTC-TCGA cohort. c, Comparison of IFN-γ scores, PDL1, PDCD1, and CTLA4 expression by the different immune state in the PTMC-TCGA cohort. *p*-values from the Wilcoxon rank-sum tests. d, Kaplan–Meier curves of PFS according to the diagnostic prediction model in the ESPTC-TCGA cohort. *p*-value from the Log-rank test.
