# Supplementary material for: Multi-omics profiling of papillary thyroid microcarcinoma reveals different somatic mutations and a unique transcriptomic signature
Source: J Transl Med. 2023 Mar 20;21:206. doi: 10.1186/s12967-023-04045-2 (PMC10026500; doi:10.1186/s12967-023-04045-2)
Supplement: Supplementary file 2 — Additional file 2: Table S1. PTMETA cohort clinical data. Table S2. TCGA cohort clinical data. Table S3. Comparison of clinical data between PTMETA and TCGA. Table S4. Average silhouette width plots of the cancer subgroups for the PTMETA cohort and TCGA cohort. Table S5. Differential expression genes between PTMETA tumor samples and normal samples. Table S6. Differential expression genes between TCGA tumor samples and normal samples. Table S7. Maker genes in the two PTMETA subgroups. [file 12967_2023_4045_MOESM2_ESM.doc]

**Table S1: PTMETA cohort clinical data.**

| Sample | Gender | Age | TI-RADS | pT | pN | pM | Stage | Histological type | Tumor size (greatest diameter, cm ) | TPOAb status | | TgAb status |
| --- | --- | --- | --- | --- | --- | --- | --- | --- | --- | --- | --- | --- |
| 101T | Female | 34.00 | 4 | T1 | N0 | M0 | I | Classical | 0.40 | | Negative | Negative |
| 108T | Female | 48.00 | 4 | T1 | N0 | M0 | I | Follicular-variant | 0.20 | | Positive | Negative |
| 109T | Female | 37.00 | 4 | T1 | N1 | M0 | I | Classical | 0.30 | | Negative | Negative |
| 110T | Female | 37.00 | 4 | T1 | N0 | M0 | I | Classical | 0.90 | | Negative | Negative |
| 113T | Female | 23.00 | 4 | T1 | N1 | M0 | I | Classical | 0.70 | | Negative | Positive |
| 121T | Female | 43.00 | 4 | T1 | N1 | M0 | I | Follicular-variant | 0.90 | | Negative | Negative |
| 126T | Female | 54.00 | 4 | T1 | N1 | M0 | I | Classical | 0.60 | | Negative | Negative |
| 129T | Female | 42.00 | 4 | T1 | N0 | M0 | I | Follicular-variant | 0.60 | | Positive | Negative |
| 130T | Female | 37.00 | 5 | T1 | N1 | M0 | I | Classical | 0.30 | | Positive | Positive |
| 131T | Female | 39.00 | 4 | T1 | N0 | M0 | I | Classical | 0.60 | | Negative | Negative |
| 133T | Male | 22.00 | 4 | T1 | N1 | M0 | I | Classical | 0.90 | | Negative | Negative |
| 138T | Female | 41.00 | 5 | T1 | N1 | M0 | I | Follicular-variant | 0.20 | | Negative | Negative |
| 139T | Male | 65.00 | 4 | T1 | N0 | M0 | I | Follicular-variant | 0.70 | | Negative | Negative |
| 142T | Female | 46.00 | 4 | T1 | N1 | M0 | I | Classical | 0.80 | | Negative | Negative |
| 143T | Female | 54.00 | 4 | T1 | N1 | M0 | I | Classical | 0.50 | | Negative | Negative |
| 144T | Female | 58.00 | 4 | T1 | N1 | M0 | II | Classical | 0.40 | | Negative | Negative |
| 146T | Male | 52.00 | 4 | T1 | N0 | M0 | I | Classical | 0.70 | | Negative | Negative |
| 150T | Female | 51.00 | 4 | T1 | N0 | M0 | I | Classical | 0.80 | | Negative | Negative |
| 152T | Female | 57.00 | 5 | T1 | N1 | M0 | II | Classical | 0.60 | | Negative | Negative |
| 154T | Female | 24.00 | 4 | T1 | N0 | M0 | I | Classical | 0.80 | | Negative | Negative |
| 157T | Male | 46.00 | 4 | T1 | N0 | M0 | I | Classical | 0.80 | | Negative | Negative |
| 158T | Female | 49.00 | 4 | T1 | N0 | M0 | I | Follicular-variant | 0.50 | | Negative | Negative |
| 160T | Female | 54.00 | 4 | T1 | N1 | M0 | I | Classical | 0.30 | | Negative | Negative |
| 180T | Female | 61.00 | 4 | T1 | N0 | M0 | I | Classical | 0.30 | | Negative | Negative |
| 181T | Female | 53.00 | 4 | T1 | N0 | M0 | I | Classical | 0.10 | | Negative | Negative |
| 188T | Female | 43.00 | 4 | T1 | N1 | M0 | I | Classical | 0.60 | | Negative | Negative |
| 193T | Female | 56.00 | 5 | T1 | N1 | M0 | II | Classical | 0.30 | | Negative | Negative |
| 198T | Female | 47.00 | 4 | T1 | N0 | M0 | I | Follicular-variant | 0.80 | | Negative | Negative |
| 20T | Female | 57.00 | 4 | T1 | N0 | M0 | I | Follicular-variant | 0.30 | | Negative | Negative |
| 21T | Female | 51.00 | 4 | T1 | N0 | M0 | I | Follicular-variant | 0.70 | | Positive | Negative |
| 23T | Female | 21.00 | 4 | T1 | N1 | M0 | I | Classical | 0.50 | | Negative | Negative |
| 28T | Female | 42.00 | 4 | T1 | N0 | M0 | I | Follicular-variant | 0.20 | | Negative | Negative |
| 29T | Female | 56.00 | 4 | T1 | N0 | M0 | I | Follicular-variant | 0.60 | | Negative | Negative |
| 2T | Male | 41.00 | 5 | T1 | N1 | M0 | I | Classical | 0.80 | | Negative | Negative |
| 30T | Male | 41.00 | 4 | T1 | N0 | M0 | I | Classical | 0.50 | | Negative | Negative |
| 31T | Female | 58.00 | 4 | T1 | N0 | M0 | I | Follicular-variant | 0.50 | | Negative | Negative |
| 32T | Male | 55.00 | 4 | T1 | N0 | M0 | I | Classical | 1.00 | | Negative | Negative |
| 33T | Female | 43.00 | 4 | T1 | N0 | M0 | I | Classical | 0.70 | | Negative | Negative |
| 35T | Female | 53.00 | 4 | T1 | N0 | M0 | I | Classical | 0.40 | | Negative | Positive |
| 3T | Male | 65.00 | 4 | T1 | N0 | M0 | I | Follicular-variant | 0.70 | | Negative | Negative |
| 40T | Female | 43.00 | 4 | T1 | N1 | M0 | I | Classical | 0.40 | | Negative | Negative |
| 42T | Male | 42.00 | 4 | T1 | N1 | M0 | I | Classical | 0.80 | | Negative | Negative |
| 43T | Female | 42.00 | 4 | T1 | N1 | M0 | I | Follicular-variant | 0.70 | | Negative | Negative |
| 46T | Female | 45.00 | 4 | T1 | N1 | M0 | I | Classical | 0.60 | | Negative | Negative |
| 47T | Male | 33.00 | 4 | T1 | N1 | M0 | I | Classical | 0.20 | | Negative | Negative |
| 4T | Male | 60.00 | 5 | T1 | N1 | M0 | II | Classical | 0.70 | | Negative | Negative |
| 54T | Female | 36.00 | 4 | T1 | N1 | M0 | I | Classical | 0.50 | | Negative | Negative |
| 60T | Female | 45.00 | 4 | T1 | N0 | M0 | I | Classical | 0.80 | | Positive | Negative |
| 61T | Female | 31.00 | 5 | T1 | N1 | M0 | I | Classical | 0.90 | | Positive | Negative |
| 68T | Male | 47.00 | 5 | T1 | N1 | M0 | I | Classical | 0.40 | | Negative | Negative |
| 6T | Female | 52.00 | 4 | T1 | N0 | M0 | I | Follicular-variant | 0.80 | | Negative | Negative |
| 76T | Female | 39.00 | 4 | T1 | N0 | M0 | I | Follicular-variant | 0.80 | | Negative | Negative |
| 7T | Male | 41.00 | 4 | T1 | N1 | M0 | I | Classical | 0.80 | | Negative | Negative |
| 80T | Male | 49.00 | 4 | T1 | N0 | M0 | I | Classical | 0.80 | | Negative | Negative |
| 81T | Female | 41.00 | 4 | T1 | N0 | M0 | I | Follicular-variant | 0.70 | | Negative | Positive |
| 82T | Female | 27.00 | 4 | T1 | N1 | M0 | I | Classical | 0.50 | | Positive | Positive |
| 85T | Female | 45.00 | 4 | T1 | N0 | M0 | I | Follicular-variant | 0.50 | | Negative | Negative |
| 87T | Female | 37.00 | 4 | T1 | N0 | M0 | I | Classical | 0.50 | | Negative | Negative |
| 88T | Female | 48.00 | 5 | T1 | N1 | M0 | I | Classical | 1.00 | | Negative | Negative |
| 90T | Female | 50.00 | 5 | T1 | N1 | M0 | I | Classical | 0.90 | | Negative | Negative |
| 92T | Female | 40.00 | 4 | T1 | N1 | M0 | I | Classical | 0.60 | | Negative | Negative |
| 93T | Female | 54.00 | 4 | T1 | N0 | M0 | I | Classical | 0.70 | | Negative | Positive |
| 94T | Male | 31.00 | 4 | T1 | N1 | M0 | I | Classical | 0.60 | | Negative | Negative |
| 98T | Female | 54.00 | 4 | T1 | N1 | M0 | I | Classical | 0.60 | | Negative | Negative |

**Table S2: TCGA cohort clinical data.**

| Tumor_Sample_Barcode | Gender | Age | TI-RADS | pT | pN | pM | Stage | Histological type | Tumor size (greatest diameter, cm ) | Race | BRAF_RAS_class |
| --- | --- | --- | --- | --- | --- | --- | --- | --- | --- | --- | --- |
| TCGA-BJ-A0ZC-01 | Male | 55 | Not reported | T1 | N0 | M0 | I | Classical | 0.4 | WHITE | Ras-like |
| TCGA-BJ-A28Z-01 | Female | 46 | Not reported | T1 | N1 | M0 | I | Classical | 1.0 | WHITE | Braf-like |
| TCGA-BJ-A45E-01 | Female | 46 | Not reported | T1 | N0 | M0 | I | Classical | 0.5 | WHITE | Unknown |
| TCGA-BJ-A45J-01 | Female | 39 | Not reported | T1 | N1 | M0 | I | Tall cell | 0.3 | WHITE | Braf-like |
| TCGA-DJ-A1QM-01 | Male | 42 | Not reported | T1 | N0 | M0 | I | Follicular-variant | 0.7 | WHITE | Braf-like |
| TCGA-DJ-A2PO-01 | Male | 54 | Not reported | T1 | N0 | M0 | I | Classical | 0.6 | WHITE | Braf-like |
| TCGA-DJ-A3UR-01 | Female | 51 | Not reported | T1 | N1 | M0 | I | Follicular-variant | 1.0 | WHITE | Braf-like |
| TCGA-DJ-A3UT-01 | Female | 43 | Not reported | T1 | N0 | M0 | I | Follicular-variant | 0.9 | WHITE | Ras-like |
| TCGA-DJ-A3UV-01 | Female | 18 | Not reported | T1 | N0 | M0 | I | Classical | 1.0 | WHITE | Unknown |
| TCGA-DJ-A3UX-01 | Female | 46 | Not reported | T1 | N0 | M0 | I | Classical | 0.5 | WHITE | Braf-like |
| TCGA-DJ-A3VD-01 | Female | 32 | Not reported | T1 | N1 | M0 | I | Classical | 0.8 | WHITE | Unknown |
| TCGA-DJ-A3VF-01 | Female | 60 | Not reported | T1 | N1 | M0 | II | Tall cell | 1.0 | WHITE | Braf-like |
| TCGA-DJ-A3VK-01 | Male | 63 | Not reported | T1 | N0 | M0 | I | Follicular-variant | 1.0 | WHITE | Ras-like |
| TCGA-DJ-A3VL-01 | Male | 38 | Not reported | T1 | N0 | M0 | I | Follicular-variant | 0.6 | WHITE | Ras-like |
| TCGA-DJ-A4UT-01 | Female | 32 | Not reported | T1 | N0 | M0 | I | Classical | 1.0 | WHITE | Braf-like |
| TCGA-DJ-A4V4-01 | Female | 48 | Not reported | T1 | N0 | M0 | I | Tall cell | 0.9 | WHITE | Braf-like |
| TCGA-E3-A3E5-01 | Male | 57 | Not reported | T4a | N1 | M0 | III | Classical | 1.0 | WHITE | Braf-like |
| TCGA-E8-A3X7-01 | Female | 57 | Not reported | T4a | N0 | M0 | III | Classical | 1.0 | WHITE | Unknown |
| TCGA-E8-A434-01 | Female | 56 | Not reported | T1 | N0 | M0 | I | Classical | 0.7 | WHITE | Unknown |
| TCGA-ET-A39S-01 | Female | 27 | Not reported | T1 | N0 | M0 | I | Classical | 1.0 | WHITE | Braf-like |
| TCGA-ET-A3DQ-01 | Female | 43 | Not reported | T1 | N0 | M0 | I | Follicular-variant | 1.0 | WHITE | Ras-like |
| TCGA-ET-A3DS-01 | Female | 33 | Not reported | T1 | N0 | M0 | I | Classical | 0.8 | WHITE | Ras-like |
| TCGA-FE-A3PB-01 | Female | 33 | Not reported | T1 | N1 | M0 | I | Classical | 1.0 | WHITE | Braf-like |
| TCGA-FK-A3SD-01 | Female | 61 | Not reported | T1 | N0 | M0 | I | Classical | 0.1 | WHITE | Ras-like |
| TCGA-FY-A3R9-01 | Female | 66 | Not reported | T1 | N0 | M0 | I | Classical | 0.7 | WHITE | Ras-like |
| TCGA-FY-A40K-01 | Female | 46 | Not reported | T1 | N0 | M0 | I | Classical | 1.0 | WHITE | Braf-like |
| TCGA-FY-A40N-01 | Female | 55 | Not reported | T1 | N0 | M0 | I | Follicular-variant | 0.9 | WHITE | Unknown |
| TCGA-J8-A3YF-01 | Male | 83 | Not reported | T1 | N1 | M0 | II | Classical | 0.5 | WHITE | Unknown |

**Table S3: Comparison of clinical data between PTMETA and TCGA.**

|  | PTMETA | TCGA | *p* |
| --- | --- | --- | --- |
| Number | 64 | 28 |  |
| Gender |  |  | 1.000 |
| Female | 49 (76.56%) | 21 (75.00%) |  |
| Male | 15 (23.44%) | 7 (25.00%) |  |
| Age, yrs | 45.12 (±10.25) | 47.50 (±13.66) | 0.359 |
| Stage |  |  | 0.153 |
| I | 60 (93.75%) | 24 (85.71%) |  |
| II | 4 (6.25%) | 2 (7.14%) |  |
| III | 0 (0) | 2 (7.14%) |  |
| pT |  |  | 0.090 |
| T1 | 64 (100%) | 26 (92.86%) |  |
| T4a | 0 (0) | 2 (7.14%) |  |
| pN |  |  | 0.069 |
| N0 | 32 (50%) | 20 (71.43%) |  |
| N1 | 32 (50%) | 8 (28.57%) |  |
| pM |  |  |  |
| M0 | 64 (100%) | 28 (100%) | NA |
| Histological type |  |  |  |
| Classical | 46 (71.88%) | 18 (64.29%) | 0.050 |
| Follicular-variant | 18 (28.12%) | 7 (25.00%) |  |
| Tall cell | 0 (0) | 3 (10.71%) |  |
| Tumor size, cm | 0.60 [0.48, 0.80] | 0.90 [0.60, 1.00] | 0.001 |

**Table S4: Average silhouette width plots of the cancer subgroups for the PTMETA cohort and TCGA cohort.**

| Gene number_cluster | 1000_2 | 1000_3 | 1000_4 | 1000_5 | 2000_2 | 2000_3 | 2000_4 | 2000_5 | 3000_2 | 3000_3 | 3000_4 | 3000_5 | 4000_2 | 4000_3 | 4000_4 | 4000_5 | 5000_2 | 5000_3 | 5000_4 | 5000_5 |
| --- | --- | --- | --- | --- | --- | --- | --- | --- | --- | --- | --- | --- | --- | --- | --- | --- | --- | --- | --- | --- |
| SC3_PTMETA | 0.61 | 0.67 | 0.56 | 0.51 | 0.68 | 0.56 | 0.59 | 0.53 | 0.77 | 0.59 | 0.59 | 0.48 | 0.83 | 0.5 | 0.6 | 0.42 | 0.87 | 0.52 | 0.52 | 0.44 |
| NMF_PTMETA | 0.73 | 0.54 | 0.25 | 0.39 | 0.66 | 0.49 | 0.44 | 0.39 | 0.66 | 0.33 | 0.41 | 0.21 | 0.6 | 0.4 | 0.36 | 0.13 | 0.75 | 0.36 | 0.29 | 0.19 |
| SC3_TCGA | 0.71 | 0.54 | 0.52 | 0.51 | 0.76 | 0.49 | 0.54 | 0.52 | 0.74 | 0.53 | 0.54 | 0.53 | 0.75 | 0.55 | 0.53 | 0.53 | 0.75 | 0.55 | 0.53 | 0.53 |
| NMF_TCGA | 0.59 | 0.41 | 0.33 | 0.14 | 0.6 | 0.33 | 0.26 | 0.14 | 0.48 | 0.27 | 0.24 | 0.21 | 0.56 | 0.38 | 0.14 | 0.27 | 0.49 | 0.46 | 0.3 | 0.24 |

**Table S5: Differential expression genes between PTMETA tumor samples and normal samples.**

| row | baseMean | log2FoldChange | lfcSE | stat | pvalue | padj | change |
| --- | --- | --- | --- | --- | --- | --- | --- |
| A1CF | 4.359074613 | -1.463478047 | 0.363808 | -4.02267 | 5.75E-05 | 0.000222 | Down |
| A2ML1 | 10.3806729 | -1.680778607 | 0.328937 | -5.10972 | 3.23E-07 | 1.78E-06 | Down |
| A3GALT2 | 1.903728724 | -2.116413329 | 0.487853 | -4.33822 | 1.44E-05 | 6.18E-05 | Down |
| AACSP1 | 1.263480549 | -2.375837433 | 0.735483 | -3.23031 | 0.001237 | 0.0036 | Down |
| AADAC | 2.322950206 | -2.404050971 | 0.356011 | -6.75274 | 1.45E-11 | 1.31E-10 | Down |
| AADACL2 | 6.874533872 | -2.403958734 | 0.295519 | -8.1347 | 4.13E-16 | 5.64E-15 | Down |
| AADACL4 | 2.070564091 | -3.020211304 | 0.650149 | -4.64541 | 3.39E-06 | 1.62E-05 | Down |
| AADACP1 | 43.23386216 | -2.848793647 | 0.217807 | -13.0794 | 4.32E-39 | 2.84E-37 | Down |
| AANAT | 4.522903183 | -1.124095053 | 0.241925 | -4.64647 | 3.38E-06 | 1.61E-05 | Down |
| AATK-AS1 | 1.634745864 | -1.186045729 | 0.500027 | -2.37196 | 0.017694 | 0.037207 | Down |
| ABC12-47964100C23.1 | 2.41345693 | -2.007537761 | 0.723815 | -2.77355 | 0.005545 | 0.013578 | Down |
| ABC7-42418200C9.1 | 1.163457963 | -2.737690056 | 1.174778 | -2.33039 | 0.019786 | 0.040965 | Down |
| ABCA10 | 276.6828063 | -1.899098018 | 0.164597 | -11.5379 | 8.50E-31 | 3.43E-29 | Down |
| ABCA12 | 15.38604367 | 1.290676503 | 0.265823 | 4.855394 | 1.20E-06 | 6.12E-06 | Up |
| ABCA6 | 739.171654 | -1.169716522 | 0.13872 | -8.43222 | 3.39E-17 | 5.07E-16 | Down |
| ABCA8 | 1669.287145 | -1.360198655 | 0.122371 | -11.1154 | 1.06E-28 | 3.71E-27 | Down |
| ABCA9 | 505.5667384 | -1.939176169 | 0.162726 | -11.9168 | 9.68E-33 | 4.40E-31 | Down |
| ABCA9-AS1 | 5.596052452 | -1.640099697 | 0.301551 | -5.43888 | 5.36E-08 | 3.27E-07 | Down |
| ABCB11 | 8.181530499 | -1.33129825 | 0.45714 | -2.91224 | 0.003589 | 0.009247 | Down |
| ABCC11 | 65.05538351 | 2.060197605 | 0.269295 | 7.650342 | 2.00E-14 | 2.39E-13 | Up |
| ABCC3 | 812.4733475 | 2.236284989 | 0.16174 | 13.82644 | 1.77E-43 | 1.53E-41 | Up |
| ABCC4 | 662.0914236 | -1.103268714 | 0.109616 | -10.0648 | 7.90E-24 | 1.95E-22 | Down |
| ABCC6P1 | 37.27925974 | 1.258063149 | 0.155497 | 8.090613 | 5.94E-16 | 7.99E-15 | Up |
| ABCC6P2 | 22.96811064 | 1.786158587 | 0.161196 | 11.08067 | 1.56E-28 | 5.42E-27 | Up |
| ABCC8 | 58.9790976 | -1.748989339 | 0.223831 | -7.81389 | 5.54E-15 | 6.97E-14 | Down |
| ABCD1P2 | 1.576741153 | -2.395934578 | 0.896523 | -2.67247 | 0.007529 | 0.017755 | Down |
| ABCG5 | 4.319244685 | -1.655858103 | 0.29782 | -5.55992 | 2.70E-08 | 1.71E-07 | Down |
| ABCG8 | 1.070353869 | -1.747341236 | 0.657324 | -2.65827 | 0.007854 | 0.018423 | Down |
| ABHD1 | 23.30238413 | -1.070835879 | 0.140658 | -7.61304 | 2.68E-14 | 3.15E-13 | Down |
| ABHD11-AS1 | 19.67185076 | 2.063508963 | 0.201464 | 10.24258 | 1.28E-24 | 3.32E-23 | Up |
| ABHD17AP4 | 1.519342477 | -2.386399315 | 0.622763 | -3.83196 | 0.000127 | 0.000459 | Down |
| ABHD17AP6 | 1.302130306 | -2.004475254 | 0.537855 | -3.72679 | 0.000194 | 0.000676 | Down |
| ABI3BP | 1910.059585 | -1.076445389 | 0.137092 | -7.85198 | 4.10E-15 | 5.20E-14 | Down |
| ABLIM3 | 362.3486433 | -1.061948159 | 0.106304 | -9.98975 | 1.69E-23 | 4.08E-22 | Down |
| ABTB2 | 284.0406922 | 2.25182819 | 0.119574 | 18.83206 | 4.12E-79 | 1.73E-76 | Up |
| AC000036.4 | 4.073890399 | -1.02474757 | 0.2894 | -3.54094 | 0.000399 | 0.001298 | Down |
| AC000099.1 | 1.989296096 | -1.924832926 | 0.593388 | -3.2438 | 0.001179 | 0.003448 | Down |
| AC000124.1 | 1.265734724 | -1.760356805 | 0.456648 | -3.85496 | 0.000116 | 0.000421 | Down |
| AC000374.1 | 1.953151241 | -1.558774961 | 0.444444 | -3.50725 | 0.000453 | 0.001455 | Down |
| AC002064.5 | 2.146279159 | -1.808862391 | 0.403363 | -4.48445 | 7.31E-06 | 3.30E-05 | Down |
| AC002064.7 | 0.984942883 | -1.309964225 | 0.496454 | -2.63864 | 0.008324 | 0.019401 | Down |
| AC002066.1 | 20.70786752 | -1.174117053 | 0.178368 | -6.58256 | 4.62E-11 | 3.97E-10 | Down |
| AC002117.1 | 3.039203293 | -1.052639129 | 0.432145 | -2.43585 | 0.014857 | 0.032061 | Down |
| AC002306.1 | 11.81345784 | -2.26701853 | 0.242475 | -9.3495 | 8.81E-21 | 1.74E-19 | Down |
| AC002331.1 | 1.450996419 | 1.81044945 | 0.357971 | 5.057531 | 4.25E-07 | 2.31E-06 | Up |
| AC002366.3 | 20.21519858 | -1.214673581 | 0.217435 | -5.58637 | 2.32E-08 | 1.48E-07 | Down |
| AC002451.3 | 16.3972453 | -1.295019356 | 0.182732 | -7.08698 | 1.37E-12 | 1.37E-11 | Down |
| AC002511.2 | 1.117120324 | -2.069013495 | 0.419 | -4.93798 | 7.89E-07 | 4.12E-06 | Down |
| AC002511.3 | 3.787433584 | -2.509381993 | 0.263811 | -9.51203 | 1.87E-21 | 3.89E-20 | Down |
| AC002519.6 | 1.72529581 | -1.643667408 | 0.561568 | -2.92692 | 0.003423 | 0.008869 | Down |
| AC003003.5 | 3.646643891 | -1.271925851 | 0.439224 | -2.89585 | 0.003781 | 0.009685 | Down |
| AC003005.2 | 1.132364583 | -1.233428816 | 0.321611 | -3.83516 | 0.000125 | 0.000453 | Down |
| AC003090.1 | 7.5409242 | -1.982121553 | 0.34656 | -5.71941 | 1.07E-08 | 7.14E-08 | Down |
| AC003956.1 | 0.480250782 | -1.397716273 | 0.612091 | -2.28351 | 0.0224 | 0.045577 | Down |
| AC003986.6 | 3.105864723 | -1.273163705 | 0.452031 | -2.81654 | 0.004854 | 0.012073 | Down |
| AC004019.10 | 5.45731228 | -2.759150861 | 0.39159 | -7.04602 | 1.84E-12 | 1.82E-11 | Down |
| AC004022.8 | 2.361829216 | -1.674948388 | 0.404757 | -4.13816 | 3.50E-05 | 0.000141 | Down |
| AC004051.2 | 3.705067981 | -1.562686174 | 0.385573 | -4.05289 | 5.06E-05 | 0.000197 | Down |
| AC004053.1 | 2.281197249 | -1.808085301 | 0.375713 | -4.81242 | 1.49E-06 | 7.49E-06 | Down |
| AC004054.1 | 0.589879419 | -1.228431727 | 0.541453 | -2.26877 | 0.023283 | 0.047092 | Down |
| AC004066.3 | 0.736500745 | 1.266734187 | 0.438064 | 2.891663 | 0.003832 | 0.009794 | Up |
| AC004221.2 | 2.103402689 | -1.79443477 | 0.519919 | -3.45137 | 0.000558 | 0.001754 | Down |
| AC004471.10 | 4.074357599 | -1.645055626 | 0.28984 | -5.67573 | 1.38E-08 | 9.09E-08 | Down |
| AC004692.4 | 1.117052076 | -1.54495701 | 0.517333 | -2.98639 | 0.002823 | 0.007467 | Down |
| AC004862.6 | 6.378697609 | -2.961494708 | 0.421315 | -7.02917 | 2.08E-12 | 2.04E-11 | Down |
| AC004870.4 | 0.867909255 | -1.467355107 | 0.480358 | -3.05471 | 0.002253 | 0.006121 | Down |
| AC004870.5 | 3.299642802 | -2.188482699 | 0.445935 | -4.90763 | 9.22E-07 | 4.77E-06 | Down |
| AC004899.3 | 1.309452087 | -1.924502222 | 0.767775 | -2.5066 | 0.01219 | 0.027058 | Down |
| AC004947.2 | 7.234632449 | -1.545659979 | 0.274912 | -5.62239 | 1.88E-08 | 1.22E-07 | Down |
| AC004951.5 | 2.239198992 | 1.090008889 | 0.306676 | 3.554265 | 0.000379 | 0.001241 | Up |
| AC005014.5 | 3.071342815 | -1.093543325 | 0.395024 | -2.7683 | 0.005635 | 0.013768 | Down |
| AC005037.4 | 2.842056248 | -1.058742211 | 0.310002 | -3.41527 | 0.000637 | 0.001978 | Down |
| AC005077.12 | 19.99120573 | -1.205511091 | 0.208157 | -5.79136 | 6.98E-09 | 4.75E-08 | Down |
| AC005077.14 | 21.54922235 | -1.500437494 | 0.226425 | -6.62663 | 3.43E-11 | 2.98E-10 | Down |
| AC005105.2 | 1.956616502 | 1.292952809 | 0.470343 | 2.748956 | 0.005979 | 0.014504 | Up |
| AC005237.4 | 11.79935437 | -1.122848168 | 0.180339 | -6.22631 | 4.78E-10 | 3.71E-09 | Down |
| AC005307.3 | 3.211568335 | -2.635757202 | 0.358792 | -7.34621 | 2.04E-13 | 2.20E-12 | Down |
| AC005324.6 | 0.798804305 | -1.797383492 | 0.664125 | -2.70639 | 0.006802 | 0.016264 | Down |
| AC005329.7 | 34.07748248 | -1.176758503 | 0.185726 | -6.33599 | 2.36E-10 | 1.89E-09 | Down |
| AC005351.1 | 2.061499761 | -1.650098772 | 0.455293 | -3.62425 | 0.00029 | 0.000973 | Down |
| AC005355.3 | 1.250332819 | -1.284224543 | 0.356459 | -3.60272 | 0.000315 | 0.00105 | Down |
| AC005477.1 | 9.942593588 | -2.155516432 | 0.281138 | -7.66711 | 1.76E-14 | 2.11E-13 | Down |
| AC005522.7 | 3.279011038 | -1.026228034 | 0.316897 | -3.23837 | 0.001202 | 0.00351 | Down |
| AC005532.5 | 2.026235632 | -1.789524943 | 0.411084 | -4.35318 | 1.34E-05 | 5.80E-05 | Down |
| AC005537.2 | 3.523845228 | -1.633538697 | 0.417332 | -3.91424 | 9.07E-05 | 0.000336 | Down |
| AC005550.3 | 15.5344826 | -1.520206285 | 0.221741 | -6.85576 | 7.09E-12 | 6.60E-11 | Down |
| AC005578.3 | 2.687955693 | -1.65897022 | 0.401169 | -4.13534 | 3.54E-05 | 0.000142 | Down |
| AC005588.2 | 0.50774999 | -1.288666309 | 0.504076 | -2.55649 | 0.010573 | 0.023882 | Down |
| AC005592.3 | 11.25634629 | -1.013845051 | 0.24499 | -4.13832 | 3.50E-05 | 0.000141 | Down |
| AC005593.2 | 1.519935176 | -1.735563543 | 0.343627 | -5.05072 | 4.40E-07 | 2.38E-06 | Down |
| AC005614.3 | 8.635473639 | -1.103804179 | 0.241212 | -4.57608 | 4.74E-06 | 2.20E-05 | Down |
| AC005616.1 | 1.526487147 | -2.782866755 | 0.886886 | -3.1378 | 0.001702 | 0.004786 | Down |
| AC005682.6 | 3.882680134 | -1.023973839 | 0.23227 | -4.40854 | 1.04E-05 | 4.58E-05 | Down |
| AC005703.3 | 1.263772102 | -2.025130132 | 0.65579 | -3.08808 | 0.002015 | 0.00555 | Down |
| AC005722.4 | 1.303741512 | -2.391853845 | 0.980472 | -2.43949 | 0.014708 | 0.031793 | Down |
| AC005775.2 | 1.626740093 | -1.215210703 | 0.47548 | -2.55576 | 0.010596 | 0.023926 | Down |
| AC005796.2 | 1.039925077 | -2.090923344 | 0.405069 | -5.1619 | 2.44E-07 | 1.37E-06 | Down |
| AC005863.1 | 1.587139891 | -2.597229479 | 1.130487 | -2.29744 | 0.021594 | 0.044161 | Down |
| AC005932.1 | 4.901607708 | -1.37644342 | 0.258406 | -5.32667 | 1.00E-07 | 5.90E-07 | Down |
| AC006004.1 | 1.237259653 | -3.111811546 | 1.356315 | -2.29431 | 0.021772 | 0.044454 | Down |
| AC006019.3 | 37.2852196 | -2.069506006 | 0.265594 | -7.79198 | 6.60E-15 | 8.24E-14 | Down |
| AC006019.4 | 41.37372898 | -2.247974979 | 0.294582 | -7.63106 | 2.33E-14 | 2.76E-13 | Down |
| AC006022.4 | 1.82818022 | 3.267887195 | 0.576758 | 5.665958 | 1.46E-08 | 9.59E-08 | Up |
| AC006033.22 | 3.589991054 | -1.466929305 | 0.220122 | -6.66416 | 2.66E-11 | 2.33E-10 | Down |
| AC006050.2 | 1.179943592 | -2.27670587 | 0.941761 | -2.4175 | 0.015628 | 0.03344 | Down |
| AC006077.4 | 0.499020175 | -1.512496586 | 0.559688 | -2.70239 | 0.006884 | 0.016428 | Down |
| AC006159.4 | 2.680739707 | 2.477978849 | 0.358839 | 6.905539 | 5.00E-12 | 4.73E-11 | Up |
| AC006159.5 | 4.040906315 | -1.286087677 | 0.286612 | -4.48721 | 7.22E-06 | 3.26E-05 | Down |
| AC006227.1 | 37.39210697 | -1.892172906 | 0.186395 | -10.1514 | 3.27E-24 | 8.25E-23 | Down |
| AC006262.4 | 0.859513395 | 1.947067015 | 0.512182 | 3.801516 | 0.000144 | 0.000514 | Up |
| AC006273.4 | 2.900286302 | -2.339990325 | 0.340533 | -6.87154 | 6.35E-12 | 5.94E-11 | Down |
| AC006273.5 | 17.12901765 | -1.945930031 | 0.180347 | -10.7899 | 3.84E-27 | 1.20E-25 | Down |
| AC006372.4 | 1.919747445 | -2.462434744 | 0.645787 | -3.81308 | 0.000137 | 0.000492 | Down |
| AC006427.2 | 2.669727281 | -1.926201934 | 0.351253 | -5.4838 | 4.16E-08 | 2.58E-07 | Down |
| AC006445.7 | 1.121000649 | -1.141589631 | 0.336098 | -3.3966 | 0.000682 | 0.002105 | Down |
| AC006486.10 | 13.01703408 | -1.055069078 | 0.142 | -7.43007 | 1.09E-13 | 1.20E-12 | Down |
| AC006499.1 | 1.189493799 | -1.43741321 | 0.471287 | -3.04997 | 0.002289 | 0.006205 | Down |
| AC006499.3 | 0.753394579 | -1.992701045 | 0.633785 | -3.14413 | 0.001666 | 0.004695 | Down |
| AC006499.5 | 1.20650483 | -2.118738472 | 0.577553 | -3.66848 | 0.000244 | 0.000833 | Down |
| AC006499.6 | 2.050736954 | -2.377343563 | 0.556791 | -4.26972 | 1.96E-05 | 8.23E-05 | Down |
| AC006538.8 | 2.557238064 | -1.043422027 | 0.459073 | -2.27289 | 0.023033 | 0.046667 | Down |
| AC006548.28 | 39.98711923 | -2.133416321 | 0.341524 | -6.24674 | 4.19E-10 | 3.28E-09 | Down |
| AC006552.1 | 1.477642628 | -2.897088099 | 1.185792 | -2.44317 | 0.014559 | 0.031514 | Down |
| AC006994.3 | 3.727418453 | -1.500016586 | 0.335772 | -4.46737 | 7.92E-06 | 3.56E-05 | Down |
| AC007003.1 | 1.298906672 | -2.657966864 | 0.49863 | -5.33054 | 9.79E-08 | 5.78E-07 | Down |
| AC007036.4 | 2.461268299 | -1.092874101 | 0.244157 | -4.47612 | 7.60E-06 | 3.42E-05 | Down |
| AC007041.2 | 2.280696368 | -2.124155225 | 0.487482 | -4.3574 | 1.32E-05 | 5.70E-05 | Down |
| AC007078.4 | 1.369502699 | -1.401925219 | 0.47233 | -2.9681 | 0.002996 | 0.007878 | Down |
| AC007091.1 | 0.839497161 | -2.311825118 | 0.457567 | -5.05243 | 4.36E-07 | 2.37E-06 | Down |
| AC007099.1 | 12.22364491 | 1.023367321 | 0.295111 | 3.467736 | 0.000525 | 0.001662 | Up |
| AC007163.3 | 2.594570211 | -2.56295307 | 0.574151 | -4.4639 | 8.05E-06 | 3.61E-05 | Down |
| AC007163.9 | 1.02900152 | -2.747282663 | 1.145321 | -2.3987 | 0.016453 | 0.034967 | Down |
| AC007204.2 | 40.84214598 | -1.520080702 | 0.217314 | -6.99487 | 2.66E-12 | 2.57E-11 | Down |
| AC007255.8 | 89.55804996 | 3.231513172 | 0.243906 | 13.24901 | 4.57E-40 | 3.22E-38 | Up |
| AC007278.2 | 1.90142599 | -1.327325173 | 0.482405 | -2.75147 | 0.005933 | 0.014408 | Down |
| AC007278.3 | 2.434941014 | -1.58267704 | 0.570728 | -2.77309 | 0.005553 | 0.013595 | Down |
| AC007319.1 | 11.93751014 | -1.270583971 | 0.213482 | -5.95171 | 2.65E-09 | 1.90E-08 | Down |
| AC007326.1 | 3.866869143 | -1.20535139 | 0.272253 | -4.42731 | 9.54E-06 | 4.23E-05 | Down |
| AC007349.4 | 1.15716053 | -1.884649764 | 0.54085 | -3.48461 | 0.000493 | 0.00157 | Down |
| AC007349.5 | 2.196633133 | -2.505554424 | 0.328014 | -7.63855 | 2.20E-14 | 2.61E-13 | Down |
| AC007349.7 | 1.111030364 | -2.408150914 | 0.442811 | -5.43833 | 5.38E-08 | 3.28E-07 | Down |
| AC007364.1 | 2.798634618 | -1.266249566 | 0.502271 | -2.52105 | 0.011701 | 0.026115 | Down |
| AC007365.1 | 2.133856137 | -2.027439367 | 0.782721 | -2.59024 | 0.009591 | 0.021954 | Down |
| AC007381.3 | 4.18084279 | -1.612021057 | 0.400897 | -4.02104 | 5.79E-05 | 0.000223 | Down |
| AC007386.2 | 7.288775581 | -1.423536503 | 0.533408 | -2.66876 | 0.007613 | 0.017934 | Down |
| AC007389.3 | 3.143271816 | -1.644368443 | 0.380413 | -4.32259 | 1.54E-05 | 6.60E-05 | Down |
| AC007392.3 | 1.011105549 | -1.834310377 | 0.648458 | -2.82873 | 0.004673 | 0.011685 | Down |
| AC007405.4 | 7.113433026 | -1.196935939 | 0.262309 | -4.56308 | 5.04E-06 | 2.34E-05 | Down |
| AC007461.2 | 4.592655309 | -1.927347338 | 0.350601 | -5.49727 | 3.86E-08 | 2.40E-07 | Down |
| AC007563.1 | 6.212592831 | -2.183221524 | 0.341957 | -6.38449 | 1.72E-10 | 1.40E-09 | Down |
| AC007563.3 | 2.329636415 | -1.801749483 | 0.387157 | -4.6538 | 3.26E-06 | 1.56E-05 | Down |
| AC007618.3 | 1.461315137 | -1.872758019 | 0.625724 | -2.99295 | 0.002763 | 0.007319 | Down |
| AC007743.1 | 189.3398708 | -1.431883787 | 0.108833 | -13.1567 | 1.56E-39 | 1.07E-37 | Down |
| AC007796.1 | 2.23750219 | -1.536300149 | 0.294339 | -5.2195 | 1.79E-07 | 1.03E-06 | Down |
| AC007906.1 | 3.453264971 | 1.096553111 | 0.310086 | 3.536287 | 0.000406 | 0.001319 | Up |
| AC007950.2 | 7.939519257 | -1.463823112 | 0.286191 | -5.11485 | 3.14E-07 | 1.73E-06 | Down |
| AC007952.5 | 47.45136447 | -1.331796729 | 0.219234 | -6.07477 | 1.24E-09 | 9.23E-09 | Down |
| AC007952.6 | 8.320158736 | -1.143822254 | 0.206131 | -5.549 | 2.87E-08 | 1.82E-07 | Down |
| AC008063.2 | 3.268120634 | 2.297520856 | 0.433201 | 5.303587 | 1.14E-07 | 6.65E-07 | Up |
| AC008074.3 | 2.061525267 | -1.175123773 | 0.349818 | -3.35924 | 0.000782 | 0.002376 | Down |
| AC008074.5 | 1.508620042 | -1.148803275 | 0.377354 | -3.04436 | 0.002332 | 0.006311 | Down |
| AC008079.10 | 82.75227467 | -1.339380382 | 0.213929 | -6.26086 | 3.83E-10 | 3.01E-09 | Down |
| AC008088.4 | 1.406354095 | -1.184972392 | 0.516009 | -2.29642 | 0.021652 | 0.044249 | Down |
| AC008103.3 | 2.970564183 | 4.28533954 | 0.404796 | 10.58641 | 3.45E-26 | 1.00E-24 | Up |
| AC008103.4 | 1.952146294 | 1.700842981 | 0.457745 | 3.715702 | 0.000203 | 0.000704 | Up |
| AC008132.14 | 35.01549454 | 1.719949674 | 0.197767 | 8.696855 | 3.41E-18 | 5.50E-17 | Up |
| AC008132.15 | 12.25886851 | 2.16908923 | 0.454739 | 4.769969 | 1.84E-06 | 9.14E-06 | Up |
| AC008265.2 | 1.470481114 | -1.725497869 | 0.760036 | -2.27028 | 0.02319 | 0.046933 | Down |
| AC008268.1 | 3.505936547 | -2.080863975 | 0.263545 | -7.89568 | 2.89E-15 | 3.71E-14 | Down |
| AC008271.1 | 2.574606171 | -2.381714312 | 0.674158 | -3.53287 | 0.000411 | 0.001334 | Down |
| AC008697.1 | 4.293714708 | -1.541728207 | 0.322197 | -4.78504 | 1.71E-06 | 8.51E-06 | Down |
| AC008703.1 | 3.166015452 | -2.043828973 | 0.438646 | -4.6594 | 3.17E-06 | 1.52E-05 | Down |
| AC008746.3 | 1.099676274 | -1.317824601 | 0.374566 | -3.51827 | 0.000434 | 0.001402 | Down |
| AC008753.6 | 2.153087733 | -1.327649137 | 0.279251 | -4.75433 | 1.99E-06 | 9.82E-06 | Down |
| AC008984.2 | 1.417039917 | -2.796571433 | 0.897237 | -3.11687 | 0.001828 | 0.005096 | Down |
| AC008985.1 | 12.49077372 | -1.073280984 | 0.254803 | -4.21219 | 2.53E-05 | 0.000104 | Down |
| AC009014.3 | 5.067148449 | -2.008576502 | 0.326884 | -6.14462 | 8.02E-10 | 6.09E-09 | Down |
| AC009120.10 | 1.22876699 | -1.011426069 | 0.413614 | -2.44534 | 0.014472 | 0.031338 | Down |
| AC009120.4 | 4.561519605 | -1.312423521 | 0.22917 | -5.72686 | 1.02E-08 | 6.85E-08 | Down |
| AC009120.8 | 1.160196774 | -1.870906967 | 0.517338 | -3.61641 | 0.000299 | 0.001 | Down |
| AC009120.9 | 0.773076721 | -1.513836753 | 0.561956 | -2.69387 | 0.007063 | 0.016797 | Down |
| AC009133.17 | 6.265988708 | -1.197601449 | 0.439767 | -2.72327 | 0.006464 | 0.015548 | Down |
| AC009229.5 | 2.726290646 | 1.843755389 | 0.27507 | 6.702865 | 2.04E-11 | 1.81E-10 | Up |
| AC009237.16 | 9.774625327 | -1.747957417 | 0.195971 | -8.91945 | 4.69E-19 | 8.12E-18 | Down |
| AC009237.17 | 89.96213705 | -1.8604392 | 0.212408 | -8.75879 | 1.97E-18 | 3.24E-17 | Down |
| AC009237.5 | 1.834914968 | -2.13911683 | 0.53608 | -3.9903 | 6.60E-05 | 0.000251 | Down |
| AC009238.7 | 101.601512 | -2.0432339 | 0.168847 | -12.1011 | 1.04E-33 | 4.97E-32 | Down |
| AC009238.8 | 3.342560423 | -1.964601495 | 0.234788 | -8.36756 | 5.88E-17 | 8.62E-16 | Down |
| AC009264.1 | 3.168667629 | -2.200570449 | 0.942605 | -2.33456 | 0.019566 | 0.040575 | Down |
| AC009336.19 | 8.078000949 | -1.001198045 | 0.434441 | -2.30457 | 0.021191 | 0.043461 | Down |
| AC009474.1 | 1.750530791 | -1.652353472 | 0.490789 | -3.36673 | 0.000761 | 0.002319 | Down |
| AC009502.1 | 1.499573796 | -2.754240403 | 0.925008 | -2.97753 | 0.002906 | 0.007666 | Down |
| AC009502.4 | 2.369202781 | -2.000539294 | 0.690105 | -2.89889 | 0.003745 | 0.009605 | Down |
| AC009541.1 | 1.422889214 | -2.265397952 | 1.000618 | -2.264 | 0.023574 | 0.047604 | Down |
| AC009960.2 | 1.599412805 | -2.166676006 | 0.657366 | -3.296 | 0.000981 | 0.002918 | Down |
| AC009960.3 | 1.143582871 | -2.463429921 | 0.874239 | -2.8178 | 0.004835 | 0.012033 | Down |
| AC009960.4 | 1.235325817 | -2.585940699 | 0.99332 | -2.60333 | 0.009232 | 0.021236 | Down |
| AC009960.5 | 1.232374152 | -2.639060446 | 0.838781 | -3.1463 | 0.001653 | 0.004664 | Down |
| AC009967.3 | 1.223530406 | -2.557220069 | 0.435842 | -5.86731 | 4.43E-09 | 3.08E-08 | Down |
| AC010096.1 | 1.937662101 | -2.455552397 | 0.666406 | -3.68477 | 0.000229 | 0.000787 | Down |
| AC010127.3 | 3.098780423 | -1.184771161 | 0.403331 | -2.93747 | 0.003309 | 0.008608 | Down |
| AC010148.1 | 6.542140238 | -1.124769525 | 0.314256 | -3.57915 | 0.000345 | 0.001139 | Down |
| AC010226.4 | 30.78382729 | -1.053183822 | 0.096969 | -10.8611 | 1.77E-27 | 5.69E-26 | Down |
| AC010524.4 | 1.421007681 | -1.351635507 | 0.469387 | -2.87958 | 0.003982 | 0.010133 | Down |
| AC010641.1 | 27.98151041 | -1.365534338 | 0.506415 | -2.69647 | 0.007008 | 0.016681 | Down |
| AC010729.2 | 2.068450633 | -3.253196163 | 1.190108 | -2.73353 | 0.006266 | 0.01512 | Down |
| AC010740.1 | 2.632163326 | -3.201131505 | 0.385518 | -8.30345 | 1.01E-16 | 1.45E-15 | Down |
| AC010883.5 | 4.194423964 | -1.404061938 | 0.189051 | -7.42691 | 1.11E-13 | 1.23E-12 | Down |
| AC010886.2 | 15.60393686 | -1.394948984 | 0.24289 | -5.74313 | 9.29E-09 | 6.25E-08 | Down |
| AC010890.1 | 1.988575681 | -1.213583518 | 0.257887 | -4.70587 | 2.53E-06 | 1.23E-05 | Down |
| AC010980.2 | 27.60510873 | -2.387801654 | 0.179822 | -13.2787 | 3.08E-40 | 2.18E-38 | Down |
| AC011196.3 | 0.706986648 | -1.370975212 | 0.510294 | -2.68664 | 0.007218 | 0.017111 | Down |
| AC011286.1 | 1.349184239 | -2.506622917 | 0.562034 | -4.45992 | 8.20E-06 | 3.68E-05 | Down |
| AC011298.2 | 18.85008345 | -2.59282551 | 0.265346 | -9.7715 | 1.49E-22 | 3.35E-21 | Down |
| AC011516.2 | 3.424237456 | -1.713841368 | 0.418419 | -4.09599 | 4.20E-05 | 0.000166 | Down |
| AC011530.4 | 0.711036499 | -1.309647617 | 0.399664 | -3.27687 | 0.00105 | 0.0031 | Down |
| AC011718.2 | 12.63215748 | 2.020773605 | 0.497714 | 4.060108 | 4.91E-05 | 0.000192 | Up |
| AC011738.4 | 3.914476581 | -1.990815517 | 0.367064 | -5.42362 | 5.84E-08 | 3.54E-07 | Down |
| AC011742.3 | 0.919076893 | -1.045398485 | 0.43071 | -2.42715 | 0.015218 | 0.032718 | Down |
| AC011999.1 | 2.555482868 | -1.065086774 | 0.289149 | -3.68352 | 0.00023 | 0.00079 | Down |
| AC012065.1 | 1.409773099 | -1.324103705 | 0.461887 | -2.86673 | 0.004147 | 0.010502 | Down |
| AC012074.2 | 16.87383247 | -1.32707823 | 0.18669 | -7.10847 | 1.17E-12 | 1.18E-11 | Down |
| AC012360.4 | 1.014506039 | -1.361516242 | 0.329436 | -4.13287 | 3.58E-05 | 0.000144 | Down |
| AC012360.6 | 22.02669553 | -1.527471513 | 0.239656 | -6.37361 | 1.85E-10 | 1.50E-09 | Down |
| AC012362.3 | 0.990076997 | -1.328595909 | 0.450477 | -2.94931 | 0.003185 | 0.008318 | Down |
| AC012363.7 | 2.771892967 | -1.259232186 | 0.390374 | -3.22571 | 0.001257 | 0.003649 | Down |
| AC012370.3 | 7.827555414 | -1.2448581 | 0.337645 | -3.68688 | 0.000227 | 0.00078 | Down |
| AC012454.4 | 1.804114952 | -2.470961872 | 0.851252 | -2.90274 | 0.003699 | 0.009497 | Down |
| AC012501.3 | 2.153939034 | -2.24757755 | 0.848834 | -2.64784 | 0.008101 | 0.018936 | Down |
| AC012507.4 | 0.62635756 | 2.407877073 | 0.611777 | 3.935872 | 8.29E-05 | 0.00031 | Up |
| AC012613.2 | 17.66861883 | -1.663304903 | 0.232431 | -7.15613 | 8.30E-13 | 8.46E-12 | Down |
| AC012668.2 | 3.661524698 | 2.577774512 | 0.391987 | 6.57618 | 4.83E-11 | 4.13E-10 | Up |
| AC013437.2 | 1.935697089 | -2.448991015 | 0.97991 | -2.4992 | 0.012447 | 0.027558 | Down |
| AC013444.1 | 6.980470493 | -1.286842379 | 0.457195 | -2.81465 | 0.004883 | 0.012131 | Down |
| AC013460.1 | 9.217258028 | -1.046481246 | 0.281972 | -3.7113 | 0.000206 | 0.000715 | Down |
| AC013470.6 | 1.274246172 | -3.107579042 | 0.822359 | -3.77886 | 0.000158 | 0.000559 | Down |
| AC013472.3 | 6.14047519 | -1.14883303 | 0.308767 | -3.72071 | 0.000199 | 0.000692 | Down |
| AC013474.4 | 4.457508399 | -1.382907038 | 0.284719 | -4.8571 | 1.19E-06 | 6.07E-06 | Down |
| AC016738.4 | 10.98881163 | -1.695393018 | 0.258314 | -6.5633 | 5.26E-11 | 4.49E-10 | Down |
| AC016772.4 | 1.42821734 | -1.813115402 | 0.5595 | -3.2406 | 0.001193 | 0.003484 | Down |
| AC016907.3 | 9.154783463 | -1.652191465 | 0.333753 | -4.95034 | 7.41E-07 | 3.88E-06 | Down |
| AC016909.1 | 4.572188791 | -1.36279655 | 0.31726 | -4.29552 | 1.74E-05 | 7.40E-05 | Down |
| AC016910.1 | 2.930277847 | -1.196291176 | 0.313134 | -3.82038 | 0.000133 | 0.000479 | Down |
| AC017002.4 | 0.896426236 | -1.97659101 | 0.804779 | -2.45607 | 0.014047 | 0.030554 | Down |
| AC017006.2 | 0.918692469 | -1.718154402 | 0.480978 | -3.57221 | 0.000354 | 0.001167 | Down |
| AC017078.1 | 1.677597518 | -1.837367308 | 0.307536 | -5.97448 | 2.31E-09 | 1.66E-08 | Down |
| AC017104.2 | 1.437641739 | -1.869262304 | 0.404851 | -4.61716 | 3.89E-06 | 1.84E-05 | Down |
| AC018647.3 | 8.960781072 | -1.155329021 | 0.210545 | -5.48732 | 4.08E-08 | 2.53E-07 | Down |
| AC018717.2 | 1.852039574 | -3.439678072 | 1.343923 | -2.55943 | 0.010484 | 0.023699 | Down |
| AC018735.1 | 1.233004944 | -2.207365379 | 0.637496 | -3.46255 | 0.000535 | 0.001691 | Down |
| AC018742.1 | 2.211493374 | -2.763547092 | 0.914067 | -3.02335 | 0.0025 | 0.006703 | Down |
| AC018755.17 | 2.155703368 | -1.130598363 | 0.392032 | -2.88394 | 0.003927 | 0.010009 | Down |
| AC018804.7 | 47.29586573 | -2.202368508 | 0.206524 | -10.664 | 1.50E-26 | 4.48E-25 | Down |
| AC018816.3 | 20.8095405 | 3.017599881 | 0.177184 | 17.03084 | 4.85E-65 | 1.16E-62 | Up |
| AC019100.7 | 2.643326824 | -1.383272363 | 0.594624 | -2.3263 | 0.020003 | 0.041344 | Down |
| AC019117.1 | 2.537979719 | 2.244634416 | 0.316537 | 7.091217 | 1.33E-12 | 1.33E-11 | Up |
| AC019118.3 | 2.164007173 | -1.51831807 | 0.320767 | -4.7334 | 2.21E-06 | 1.08E-05 | Down |
| AC019172.2 | 1.2021266 | -2.385309713 | 0.38795 | -6.14849 | 7.82E-10 | 5.95E-09 | Down |
| AC021016.7 | 0.777475196 | -1.605300752 | 0.424398 | -3.78254 | 0.000155 | 0.000552 | Down |
| AC022182.2 | 5.733585352 | -1.611079107 | 0.438443 | -3.67454 | 0.000238 | 0.000815 | Down |
| AC022819.3 | 15.65984903 | -1.28306292 | 0.282692 | -4.53873 | 5.66E-06 | 2.60E-05 | Down |
| AC023481.1 | 1.254716969 | -1.428369946 | 0.433138 | -3.29773 | 0.000975 | 0.002903 | Down |
| AC024995.1 | 2.179779377 | -1.246581936 | 0.35853 | -3.47692 | 0.000507 | 0.001611 | Down |
| AC027612.1 | 2.70533802 | -2.065533202 | 0.561776 | -3.67679 | 0.000236 | 0.000809 | Down |
| AC034110.1 | 1.445875391 | -2.804956261 | 1.221991 | -2.2954 | 0.02171 | 0.044346 | Down |
| AC034243.1 | 8.316555186 | -2.002483206 | 0.289076 | -6.9272 | 4.29E-12 | 4.08E-11 | Down |
| AC046143.3 | 15.72026889 | 1.198920313 | 0.114568 | 10.46467 | 1.26E-25 | 3.51E-24 | Up |
| AC051649.1 | 0.784799384 | -1.32643012 | 0.487415 | -2.72136 | 0.006501 | 0.015626 | Down |
| AC051649.12 | 0.641209325 | -1.618745711 | 0.507245 | -3.19125 | 0.001417 | 0.004058 | Down |
| AC053503.12 | 2.074304862 | -1.809995819 | 0.541251 | -3.3441 | 0.000826 | 0.002496 | Down |
| AC062028.1 | 1.436389141 | -1.141797801 | 0.450254 | -2.5359 | 0.011216 | 0.025143 | Down |
| AC067956.1 | 15.53262722 | -2.114896146 | 0.220957 | -9.57155 | 1.05E-21 | 2.23E-20 | Down |
| AC068039.4 | 14.09209183 | -1.083137725 | 0.218488 | -4.95743 | 7.14E-07 | 3.75E-06 | Down |
| AC068044.1 | 2.107806146 | -3.502000623 | 1.106345 | -3.16538 | 0.001549 | 0.004397 | Down |
| AC068138.1 | 103.3834751 | -1.984009174 | 0.193312 | -10.2632 | 1.03E-24 | 2.70E-23 | Down |
| AC068287.1 | 0.455154188 | -1.774769296 | 0.635661 | -2.79201 | 0.005238 | 0.012918 | Down |
| AC068831.10 | 5.531198696 | -1.59908285 | 0.310824 | -5.14465 | 2.68E-07 | 1.49E-06 | Down |
| AC068831.3 | 1.121496345 | -1.670173049 | 0.30839 | -5.41579 | 6.10E-08 | 3.69E-07 | Down |
| AC069154.4 | 0.564359562 | -1.517186192 | 0.573448 | -2.64573 | 0.008152 | 0.019039 | Down |
| AC069277.2 | 3.663439728 | -1.195374958 | 0.442463 | -2.70164 | 0.0069 | 0.016457 | Down |
| AC073043.1 | 6.479632273 | -1.636692604 | 0.387896 | -4.21942 | 2.45E-05 | 0.000101 | Down |
| AC073109.2 | 9.662324729 | -1.035729511 | 0.208892 | -4.9582 | 7.11E-07 | 3.74E-06 | Down |
| AC073130.1 | 22.67268421 | -1.636059792 | 0.209569 | -7.80678 | 5.87E-15 | 7.36E-14 | Down |
| AC073130.3 | 14.11948589 | -1.225619835 | 0.182028 | -6.73313 | 1.66E-11 | 1.48E-10 | Down |
| AC073133.1 | 9.118582046 | -1.427526928 | 0.193684 | -7.37038 | 1.70E-13 | 1.85E-12 | Down |
| AC073133.2 | 1.56461859 | -1.553571407 | 0.435526 | -3.56712 | 0.000361 | 0.001188 | Down |
| AC073150.6 | 1.38760869 | -1.141698793 | 0.425026 | -2.68618 | 0.007227 | 0.01713 | Down |
| AC073387.2 | 1.295175639 | -2.026568504 | 0.599397 | -3.38101 | 0.000722 | 0.002214 | Down |
| AC073464.11 | 1.844888904 | -2.480893103 | 0.340345 | -7.28935 | 3.11E-13 | 3.30E-12 | Down |
| AC073464.4 | 1.121319422 | -2.322622545 | 1.009943 | -2.29976 | 0.021462 | 0.043938 | Down |
| AC073464.7 | 1.747069233 | -2.591263668 | 0.347339 | -7.46033 | 8.63E-14 | 9.66E-13 | Down |
| AC073626.2 | 1.591552449 | -1.487576698 | 0.372548 | -3.99298 | 6.52E-05 | 0.000249 | Down |
| AC073850.6 | 1.535794183 | -1.973679537 | 0.301019 | -6.55666 | 5.50E-11 | 4.69E-10 | Down |
| AC073869.20 | 2.463071801 | -1.599299054 | 0.400413 | -3.99412 | 6.49E-05 | 0.000248 | Down |
| AC073934.6 | 0.938631115 | -1.774995798 | 0.411875 | -4.30955 | 1.64E-05 | 6.97E-05 | Down |
| AC078842.3 | 3.010374953 | -2.387587758 | 0.305938 | -7.80416 | 5.99E-15 | 7.50E-14 | Down |
| AC078842.4 | 8.300753045 | -2.060509134 | 0.320256 | -6.43395 | 1.24E-10 | 1.03E-09 | Down |
| AC078899.1 | 17.22535221 | -1.442463794 | 0.192581 | -7.49016 | 6.88E-14 | 7.78E-13 | Down |
| AC078899.3 | 16.67802506 | -2.359615128 | 0.295991 | -7.97192 | 1.56E-15 | 2.04E-14 | Down |
| AC079145.4 | 2.278085027 | -1.057705846 | 0.228083 | -4.63736 | 3.53E-06 | 1.68E-05 | Down |
| AC079466.1 | 6.055193571 | -2.219748055 | 0.40777 | -5.44363 | 5.22E-08 | 3.19E-07 | Down |
| AC079612.1 | 1.780247274 | -1.908724842 | 0.83523 | -2.28527 | 0.022297 | 0.045401 | Down |
| AC079630.2 | 69.84499529 | 5.072287342 | 0.318338 | 15.93368 | 3.70E-57 | 6.54E-55 | Up |
| AC079630.4 | 210.3548973 | 3.016248206 | 0.179645 | 16.79007 | 2.89E-63 | 6.44E-61 | Up |
| AC079753.5 | 1.312489933 | -1.754584729 | 0.723644 | -2.42465 | 0.015323 | 0.032899 | Down |
| AC079776.2 | 1.580201686 | -1.762128106 | 0.369763 | -4.76556 | 1.88E-06 | 9.32E-06 | Down |
| AC079779.5 | 1.725459804 | -2.281423497 | 0.945888 | -2.41194 | 0.015868 | 0.033883 | Down |
| AC079779.6 | 1.010130375 | -2.164449773 | 0.843811 | -2.56509 | 0.010315 | 0.023363 | Down |
| AC083843.2 | 3.587667655 | -2.044768314 | 0.282767 | -7.23127 | 4.78E-13 | 4.97E-12 | Down |
| AC084121.17 | 1.613445394 | -2.272739648 | 0.965285 | -2.35448 | 0.018549 | 0.038751 | Down |
| AC084125.4 | 2.543146901 | -1.694492678 | 0.242638 | -6.98363 | 2.88E-12 | 2.78E-11 | Down |
| AC084290.2 | 2.51079054 | 1.893644312 | 0.304848 | 6.211768 | 5.24E-10 | 4.06E-09 | Up |
| AC087430.1 | 2.36539681 | -1.875062963 | 0.628038 | -2.98559 | 0.00283 | 0.007484 | Down |
| AC087762.1 | 1.720747586 | -2.901816209 | 0.845807 | -3.43082 | 0.000602 | 0.00188 | Down |
| AC090286.4 | 1.344472556 | -2.553267042 | 1.003786 | -2.54364 | 0.01097 | 0.024651 | Down |
| AC090505.5 | 5.725709737 | 3.251160771 | 0.326506 | 9.957431 | 2.34E-23 | 5.57E-22 | Up |
| AC090617.1 | 1.181989745 | -1.534912686 | 0.610156 | -2.51561 | 0.011883 | 0.026471 | Down |
| AC090696.2 | 0.520383711 | -2.084266705 | 0.491314 | -4.24223 | 2.21E-05 | 9.22E-05 | Down |
| AC090952.5 | 1.515053229 | -1.511044032 | 0.438641 | -3.44483 | 0.000571 | 0.001792 | Down |
| AC090957.2 | 1.138797791 | -2.433100771 | 0.963307 | -2.52578 | 0.011544 | 0.025801 | Down |
| AC091492.2 | 1.827836702 | -1.529897385 | 0.325044 | -4.70674 | 2.52E-06 | 1.22E-05 | Down |
| AC091493.1 | 1.522991085 | -3.052530976 | 1.182926 | -2.58049 | 0.009866 | 0.022491 | Down |
| AC092155.4 | 1.301098575 | -1.849269323 | 0.485948 | -3.80549 | 0.000142 | 0.000506 | Down |
| AC092159.2 | 7.42312573 | -1.202467161 | 0.250405 | -4.8021 | 1.57E-06 | 7.86E-06 | Down |
| AC092162.1 | 8.652300254 | -1.96229971 | 0.322076 | -6.09266 | 1.11E-09 | 8.31E-09 | Down |
| AC092198.1 | 3.159377895 | -1.874637433 | 0.356266 | -5.2619 | 1.43E-07 | 8.26E-07 | Down |
| AC092535.3 | 19.33215697 | -1.091768769 | 0.21222 | -5.14451 | 2.68E-07 | 1.50E-06 | Down |
| AC092638.2 | 1.043056067 | -3.06189739 | 1.237967 | -2.47333 | 0.013386 | 0.029357 | Down |
| AC092667.2 | 11.21075388 | -1.477152098 | 0.405046 | -3.64687 | 0.000265 | 0.0009 | Down |
| AC092669.3 | 3.266997129 | -1.352173527 | 0.347791 | -3.88789 | 0.000101 | 0.000372 | Down |
| AC092684.1 | 1.118822508 | -3.314445926 | 1.073274 | -3.08816 | 0.002014 | 0.005549 | Down |
| AC092839.1 | 13.66522124 | -1.065097433 | 0.20115 | -5.29504 | 1.19E-07 | 6.95E-07 | Down |
| AC092839.4 | 1.389872568 | -1.170403687 | 0.381069 | -3.07137 | 0.002131 | 0.005832 | Down |
| AC093110.3 | 11.45078733 | -1.197390984 | 0.206195 | -5.80709 | 6.36E-09 | 4.34E-08 | Down |
| AC093166.3 | 1.250686873 | -1.733552156 | 0.598159 | -2.89815 | 0.003754 | 0.009625 | Down |
| AC093230.2 | 1.857468201 | -3.080919272 | 1.215903 | -2.53385 | 0.011282 | 0.025272 | Down |
| AC093390.1 | 4.013152065 | -1.358106362 | 0.444969 | -3.05214 | 0.002272 | 0.006165 | Down |
| AC093585.6 | 4.965068888 | -1.405821117 | 0.200136 | -7.02434 | 2.15E-12 | 2.11E-11 | Down |
| AC093609.1 | 24.89374782 | -1.650181247 | 0.155421 | -10.6175 | 2.47E-26 | 7.24E-25 | Down |
| AC093642.1 | 27.91635294 | -1.453020079 | 0.198205 | -7.3309 | 2.29E-13 | 2.45E-12 | Down |
| AC093642.3 | 32.23453282 | -1.105766297 | 0.192879 | -5.73295 | 9.87E-09 | 6.62E-08 | Down |
| AC093698.1 | 1.007448336 | -1.951735926 | 0.555995 | -3.51035 | 0.000448 | 0.001441 | Down |
| AC093843.1 | 1.423114249 | -1.867478497 | 0.367605 | -5.08012 | 3.77E-07 | 2.06E-06 | Down |
| AC093850.2 | 12.77200436 | 3.667820725 | 0.338246 | 10.84364 | 2.14E-27 | 6.84E-26 | Up |
| AC093901.1 | 0.990053443 | -1.019724393 | 0.41867 | -2.43563 | 0.014866 | 0.032076 | Down |
| AC096559.1 | 1.895226781 | -1.36564077 | 0.596772 | -2.28838 | 0.022115 | 0.045079 | Down |
| AC096574.4 | 11.59717763 | -1.552187345 | 0.268516 | -5.78061 | 7.44E-09 | 5.05E-08 | Down |
| AC096574.5 | 11.17603955 | -1.283342812 | 0.17558 | -7.30916 | 2.69E-13 | 2.87E-12 | Down |
| AC096669.1 | 5.214075028 | -2.339858588 | 0.267115 | -8.75974 | 1.96E-18 | 3.22E-17 | Down |
| AC096669.2 | 5.304689311 | -1.943931529 | 0.248038 | -7.83724 | 4.61E-15 | 5.83E-14 | Down |
| AC096669.3 | 130.6866893 | -1.436674676 | 0.255168 | -5.63031 | 1.80E-08 | 1.17E-07 | Down |
| AC097359.2 | 1.45346519 | -1.290103765 | 0.355299 | -3.63104 | 0.000282 | 0.000951 | Down |
| AC097374.2 | 11.62848202 | -1.362094387 | 0.362627 | -3.75619 | 0.000173 | 0.000608 | Down |
| AC097382.5 | 1.133416454 | -1.145069979 | 0.449473 | -2.54758 | 0.010847 | 0.024417 | Down |
| AC097461.4 | 1.405596987 | -1.004223308 | 0.396275 | -2.53416 | 0.011272 | 0.025252 | Down |
| AC097523.3 | 2.303989471 | -1.9897558 | 0.483424 | -4.11597 | 3.86E-05 | 0.000154 | Down |
| AC098592.7 | 0.491373471 | 1.56992551 | 0.683544 | 2.296745 | 0.021633 | 0.044215 | Up |
| AC098592.8 | 1.102216026 | 2.033838277 | 0.466534 | 4.359466 | 1.30E-05 | 5.65E-05 | Up |
| AC098820.3 | 4.821633349 | -1.135496712 | 0.176801 | -6.42246 | 1.34E-10 | 1.10E-09 | Down |
| AC098820.4 | 3.034334565 | -1.222664299 | 0.284758 | -4.2937 | 1.76E-05 | 7.45E-05 | Down |
| AC098824.6 | 3.540787572 | -1.02813907 | 0.327459 | -3.13975 | 0.001691 | 0.00476 | Down |
| AC099684.1 | 3.757893363 | -1.331112998 | 0.357796 | -3.72031 | 0.000199 | 0.000692 | Down |
| AC100830.3 | 6.175340218 | -1.129220747 | 0.26124 | -4.32254 | 1.54E-05 | 6.60E-05 | Down |
| AC104135.2 | 2.269113157 | -1.189276605 | 0.462093 | -2.57367 | 0.010063 | 0.022872 | Down |
| AC104297.1 | 1.319529171 | -1.536504328 | 0.521018 | -2.94904 | 0.003188 | 0.008324 | Down |
| AC104395.2 | 1.585805637 | -2.769830309 | 0.959356 | -2.88718 | 0.003887 | 0.009918 | Down |
| AC104623.1 | 9.029371467 | -2.300720111 | 0.294362 | -7.81596 | 5.45E-15 | 6.87E-14 | Down |
| AC104654.1 | 2.262951502 | -2.666210217 | 0.749778 | -3.556 | 0.000377 | 0.001234 | Down |
| AC104809.2 | 2.934329847 | -2.492590447 | 0.489069 | -5.09661 | 3.46E-07 | 1.90E-06 | Down |
| AC104809.3 | 6.538990974 | -1.109428749 | 0.434651 | -2.55246 | 0.010696 | 0.024119 | Down |
| AC104809.4 | 7.501977892 | -2.549897606 | 0.339658 | -7.50725 | 6.04E-14 | 6.88E-13 | Down |
| AC105053.3 | 11.74620463 | -1.179439808 | 0.200752 | -5.87511 | 4.23E-09 | 2.94E-08 | Down |
| AC105402.4 | 4.661300297 | -1.404383977 | 0.422495 | -3.32402 | 0.000887 | 0.002667 | Down |
| AC105921.5 | 5.077718037 | -1.726502391 | 0.368465 | -4.68567 | 2.79E-06 | 1.35E-05 | Down |
| AC106870.1 | 1.384962579 | 1.176745257 | 0.487314 | 2.414759 | 0.015746 | 0.033657 | Up |
| AC106873.4 | 2.614245771 | -1.072265514 | 0.309054 | -3.46951 | 0.000521 | 0.001651 | Down |
| AC107622.1 | 2.471940924 | -2.070388422 | 0.606908 | -3.41137 | 0.000646 | 0.002004 | Down |
| AC108462.1 | 0.893172091 | -1.733949697 | 0.417981 | -4.14839 | 3.35E-05 | 0.000135 | Down |
| AC108676.1 | 14.79505149 | -1.018588272 | 0.178063 | -5.72037 | 1.06E-08 | 7.10E-08 | Down |
| AC110620.2 | 1.454856404 | -2.779576446 | 0.896168 | -3.10162 | 0.001925 | 0.005331 | Down |
| AC111186.1 | 3.689496828 | -2.123601588 | 0.390981 | -5.43147 | 5.59E-08 | 3.40E-07 | Down |
| AC112198.1 | 4.867495007 | -1.034105489 | 0.339897 | -3.04241 | 0.002347 | 0.006348 | Down |
| AC112721.1 | 0.931721061 | 1.245880406 | 0.447544 | 2.783816 | 0.005372 | 0.013211 | Up |
| AC112721.2 | 1.737874656 | 2.243701494 | 0.42593 | 5.267767 | 1.38E-07 | 8.01E-07 | Up |
| AC113607.3 | 0.601482282 | -1.40571343 | 0.594207 | -2.3657 | 0.017996 | 0.037765 | Down |
| AC114730.2 | 1.619145088 | -1.119270323 | 0.24478 | -4.57256 | 4.82E-06 | 2.24E-05 | Down |
| AC114730.3 | 5.05851756 | -2.526523883 | 0.354952 | -7.11794 | 1.10E-12 | 1.11E-11 | Down |
| AC114760.1 | 2.373991469 | -1.276826704 | 0.372008 | -3.43226 | 0.000599 | 0.00187 | Down |
| AC114765.1 | 2.872989537 | -1.856338668 | 0.316847 | -5.85878 | 4.66E-09 | 3.24E-08 | Down |
| AC116035.1 | 3.005279553 | -1.549692876 | 0.247276 | -6.26705 | 3.68E-10 | 2.90E-09 | Down |
| AC116050.1 | 5.548268214 | -1.445292141 | 0.335261 | -4.31095 | 1.63E-05 | 6.93E-05 | Down |
| AC116614.1 | 1.409611616 | -1.184147718 | 0.353263 | -3.35203 | 0.000802 | 0.002432 | Down |
| AC117947.1 | 3.073934374 | -2.99109256 | 0.358999 | -8.33176 | 7.96E-17 | 1.15E-15 | Down |
| AC119403.1 | 0.970241883 | -1.461088955 | 0.628613 | -2.32431 | 0.020109 | 0.041541 | Down |
| AC124861.1 | 0.412759778 | -1.113312354 | 0.485483 | -2.29321 | 0.021836 | 0.044572 | Down |
| AC125634.1 | 2.003985418 | -1.372151274 | 0.60518 | -2.26735 | 0.023369 | 0.047246 | Down |
| AC127383.1 | 1.027061856 | -2.682153275 | 1.192932 | -2.24837 | 0.024553 | 0.04932 | Down |
| AC128709.4 | 0.656298688 | -1.307964228 | 0.477851 | -2.73718 | 0.006197 | 0.01497 | Down |
| AC129778.2 | 2.72058717 | -1.521662822 | 0.388908 | -3.91265 | 9.13E-05 | 0.000338 | Down |
| AC131097.4 | 20.58231149 | -1.976818881 | 0.180913 | -10.9269 | 8.57E-28 | 2.82E-26 | Down |
| AC133141.1 | 1.036267157 | -1.077099086 | 0.358264 | -3.00644 | 0.002643 | 0.00704 | Down |
| AC133680.1 | 1.045948196 | -1.149932359 | 0.352578 | -3.2615 | 0.001108 | 0.003257 | Down |
| AC133785.1 | 9.201700207 | -1.392491737 | 0.209247 | -6.65479 | 2.84E-11 | 2.48E-10 | Down |
| AC135050.5 | 1.153711062 | -1.037872363 | 0.458939 | -2.26146 | 0.023731 | 0.047883 | Down |
| AC136289.1 | 5.219722883 | -1.096663755 | 0.237108 | -4.62516 | 3.74E-06 | 1.77E-05 | Down |
| AC138517.4 | 1.392173477 | -1.429663598 | 0.61129 | -2.33876 | 0.019348 | 0.040166 | Down |
| AC139099.4 | 0.62817553 | -1.112955871 | 0.40863 | -2.72363 | 0.006457 | 0.015533 | Down |
| AC139099.7 | 3.308493359 | -2.481779654 | 0.384731 | -6.45068 | 1.11E-10 | 9.24E-10 | Down |
| AC139100.3 | 22.06157503 | -1.106826868 | 0.18008 | -6.14632 | 7.93E-10 | 6.03E-09 | Down |
| AC139100.4 | 11.05064879 | -1.273324741 | 0.181652 | -7.0097 | 2.39E-12 | 2.33E-11 | Down |
| AC139143.2 | 1.18391793 | -2.91717904 | 1.028432 | -2.83653 | 0.004561 | 0.011432 | Down |
| AC140542.2 | 1.159726919 | -2.678006363 | 1.118424 | -2.39445 | 0.016645 | 0.035321 | Down |
| AC140725.4 | 0.519471409 | -1.255066582 | 0.442555 | -2.83595 | 0.004569 | 0.011451 | Down |
| AC140912.1 | 2.536804298 | -2.556527474 | 0.742121 | -3.4449 | 0.000571 | 0.001792 | Down |
| AC144450.1 | 7.438250957 | -2.969909863 | 0.333975 | -8.89262 | 5.97E-19 | 1.02E-17 | Down |
| AC144450.2 | 1.654185273 | -1.42955492 | 0.584433 | -2.44605 | 0.014443 | 0.031292 | Down |
| AC159540.2 | 8.008746184 | -1.041495812 | 0.239844 | -4.34238 | 1.41E-05 | 6.07E-05 | Down |
| AC195454.1 | 1.602902783 | -1.799004164 | 0.444302 | -4.04905 | 5.14E-05 | 0.0002 | Down |
| ACACB | 1436.273017 | -1.251949295 | 0.102459 | -12.219 | 2.46E-34 | 1.21E-32 | Down |
| ACAN | 100.3686937 | 1.213815552 | 0.249479 | 4.865397 | 1.14E-06 | 5.84E-06 | Up |
| ACBD7 | 79.51436331 | 2.541340133 | 0.188402 | 13.48893 | 1.82E-41 | 1.40E-39 | Up |
| ACER1 | 1.813430658 | -1.03524404 | 0.414516 | -2.49748 | 0.012508 | 0.027682 | Down |
| ACER2 | 161.5715215 | 1.194844524 | 0.079622 | 15.00652 | 6.65E-51 | 8.43E-49 | Up |
| ACKR2 | 81.20784822 | -1.787391346 | 0.162561 | -10.9952 | 4.03E-28 | 1.36E-26 | Down |
| ACKR3 | 635.7182713 | -1.140787073 | 0.097603 | -11.6881 | 1.47E-31 | 6.28E-30 | Down |
| ACOT11 | 705.5060502 | -1.044320562 | 0.100195 | -10.4229 | 1.95E-25 | 5.37E-24 | Down |
| ACOT12 | 4.280203191 | 2.367973449 | 0.41556 | 5.698268 | 1.21E-08 | 8.03E-08 | Up |
| ACOT6 | 2.049771162 | -1.811847622 | 0.360465 | -5.02642 | 5.00E-07 | 2.69E-06 | Down |
| ACP5 | 513.5580174 | 1.309607667 | 0.192758 | 6.794054 | 1.09E-11 | 9.95E-11 | Up |
| ACPT | 1.23920377 | -1.378285932 | 0.337079 | -4.08891 | 4.33E-05 | 0.000171 | Down |
| ACR | 5.781930605 | -1.22303657 | 0.302361 | -4.04495 | 5.23E-05 | 0.000203 | Down |
| ACSBG1 | 23.14988986 | -1.247696676 | 0.31933 | -3.90724 | 9.34E-05 | 0.000345 | Down |
| ACSF2 | 1399.205308 | -1.060243826 | 0.073189 | -14.4863 | 1.48E-47 | 1.64E-45 | Down |
| ACSL6 | 77.28640308 | -1.204084961 | 0.217748 | -5.52972 | 3.21E-08 | 2.02E-07 | Down |
| ACSM2B | 7.316579073 | -1.762813263 | 0.398628 | -4.42221 | 9.77E-06 | 4.32E-05 | Down |
| ACSM4 | 1.336516626 | -2.142451005 | 0.644919 | -3.32205 | 0.000894 | 0.002684 | Down |
| ACSM5P1 | 1.52783758 | -2.111956366 | 0.405026 | -5.21437 | 1.84E-07 | 1.05E-06 | Down |
| ACTA1 | 39.3218728 | -3.568900059 | 0.583112 | -6.12044 | 9.33E-10 | 7.03E-09 | Down |
| ACTBL2 | 19.48019948 | 2.540797283 | 0.289854 | 8.76578 | 1.85E-18 | 3.06E-17 | Up |
| ACTBP14 | 1.554369274 | -1.189524565 | 0.45971 | -2.58756 | 0.009666 | 0.0221 | Down |
| ACTBP7 | 4.054235519 | 2.191330033 | 0.361081 | 6.068799 | 1.29E-09 | 9.57E-09 | Up |
| ACTBP9 | 1.299410917 | -2.350327281 | 0.838251 | -2.80385 | 0.00505 | 0.012497 | Down |
| ACTG1P12 | 1.342054896 | -2.560588845 | 0.807923 | -3.16935 | 0.001528 | 0.004343 | Down |
| ACTG1P9 | 1.955990661 | -1.143380402 | 0.497388 | -2.29877 | 0.021518 | 0.044035 | Down |
| ACTG2 | 99.84342752 | -1.002115077 | 0.237967 | -4.21116 | 2.54E-05 | 0.000105 | Down |
| ACTL7A | 2.957443091 | -1.075316464 | 0.318791 | -3.37311 | 0.000743 | 0.002272 | Down |
| ACTN2 | 11.61817739 | -1.90378974 | 0.291166 | -6.53851 | 6.21E-11 | 5.28E-10 | Down |
| ACTR3BP2 | 2.002615038 | -2.485245145 | 0.981701 | -2.53157 | 0.011355 | 0.025414 | Down |
| ACTR3BP4 | 3.121323574 | -1.084408148 | 0.356813 | -3.03915 | 0.002372 | 0.006411 | Down |
| ACTR3BP5 | 1.436450238 | -3.015533708 | 1.059219 | -2.84694 | 0.004414 | 0.011106 | Down |
| ADAD2 | 4.849121551 | -1.896037077 | 0.452331 | -4.1917 | 2.77E-05 | 0.000113 | Down |
| ADAM12 | 360.3155246 | 2.092754116 | 0.263297 | 7.948258 | 1.89E-15 | 2.46E-14 | Up |
| ADAM21P1 | 4.736866822 | -1.262432919 | 0.309754 | -4.0756 | 4.59E-05 | 0.000181 | Down |
| ADAM22 | 400.9967724 | -1.253981017 | 0.124542 | -10.0687 | 7.60E-24 | 1.88E-22 | Down |
| ADAM24P | 1.757899308 | -3.216106588 | 1.251934 | -2.56891 | 0.010202 | 0.023136 | Down |
| ADAM29 | 8.76281822 | -1.375377184 | 0.475453 | -2.89277 | 0.003819 | 0.009765 | Down |
| ADAM32 | 59.26233199 | -1.206681056 | 0.133423 | -9.04403 | 1.51E-19 | 2.72E-18 | Down |
| ADAM33 | 201.9164727 | -1.901984838 | 0.167716 | -11.3405 | 8.26E-30 | 3.11E-28 | Down |
| ADAMTS14 | 53.46162221 | 2.538439282 | 0.279031 | 9.097326 | 9.26E-20 | 1.70E-18 | Up |
| ADAMTS18 | 39.72181573 | 1.298334934 | 0.229796 | 5.649953 | 1.60E-08 | 1.05E-07 | Up |
| ADAMTS19 | 2.250791154 | -1.539896104 | 0.481888 | -3.19555 | 0.001396 | 0.004005 | Down |
| ADAMTS2 | 348.1891484 | 1.052793591 | 0.166156 | 6.336162 | 2.36E-10 | 1.89E-09 | Up |
| ADAMTS7P3 | 7.405002692 | -1.601648701 | 0.339025 | -4.72428 | 2.31E-06 | 1.13E-05 | Down |
| ADAMTS9 | 1663.132161 | 2.135908828 | 0.145969 | 14.63261 | 1.74E-48 | 2.03E-46 | Up |
| ADAMTS9-AS1 | 58.63479271 | 1.077795165 | 0.211848 | 5.087583 | 3.63E-07 | 1.98E-06 | Up |
| ADCY10 | 8.40028457 | -1.102615506 | 0.322701 | -3.41683 | 0.000634 | 0.001968 | Down |
| ADCY10P1 | 87.04593537 | -1.013820088 | 0.168601 | -6.01314 | 1.82E-09 | 1.33E-08 | Down |
| ADCY8 | 119.0616476 | 4.346564329 | 0.357272 | 12.16599 | 4.72E-34 | 2.29E-32 | Up |
| ADCYAP1R1 | 161.9312822 | -1.35869307 | 0.148499 | -9.14948 | 5.72E-20 | 1.06E-18 | Down |
| ADD3-AS1 | 29.66790056 | -1.616301647 | 0.154813 | -10.4404 | 1.62E-25 | 4.50E-24 | Down |
| ADH1B | 1368.782977 | -2.405812508 | 0.232491 | -10.348 | 4.27E-25 | 1.15E-23 | Down |
| ADH1C | 43.06612164 | -1.808593945 | 0.201236 | -8.98744 | 2.53E-19 | 4.48E-18 | Down |
| ADH4 | 5.285611276 | -1.296863418 | 0.305119 | -4.25035 | 2.13E-05 | 8.92E-05 | Down |
| ADH5P3 | 1.689373722 | -2.450852762 | 0.812069 | -3.01804 | 0.002544 | 0.006809 | Down |
| ADH5P4 | 2.443045827 | -1.396287509 | 0.345676 | -4.03929 | 5.36E-05 | 0.000208 | Down |
| ADH6 | 7.985735487 | -1.568665714 | 0.307703 | -5.09799 | 3.43E-07 | 1.89E-06 | Down |
| ADH7 | 1.124700272 | -2.325172504 | 0.842146 | -2.76101 | 0.005762 | 0.014039 | Down |
| ADIG | 9.510813643 | -2.036074173 | 0.196339 | -10.3702 | 3.39E-25 | 9.22E-24 | Down |
| ADIPOQ | 279.2878534 | -2.775610656 | 0.387416 | -7.16443 | 7.81E-13 | 7.98E-12 | Down |
| ADIPOQ-AS1 | 6.541857189 | -2.591030141 | 0.602049 | -4.30369 | 1.68E-05 | 7.14E-05 | Down |
| ADM | 317.1112022 | 1.312823274 | 0.1394 | 9.417659 | 4.61E-21 | 9.29E-20 | Up |
| ADM2 | 443.955409 | -1.934273085 | 0.131488 | -14.7106 | 5.51E-49 | 6.50E-47 | Down |
| ADORA1 | 309.9930394 | 2.41475414 | 0.124751 | 19.35657 | 1.79E-83 | 8.87E-81 | Up |
| ADRA1A | 14.31244145 | -1.371786813 | 0.233209 | -5.88222 | 4.05E-09 | 2.82E-08 | Down |
| ADRA1B | 47.22097223 | 1.949392423 | 0.154696 | 12.60147 | 2.07E-36 | 1.17E-34 | Up |
| ADRB3 | 5.803972907 | 1.877675275 | 0.285516 | 6.576431 | 4.82E-11 | 4.13E-10 | Up |
| ADTRP | 87.83563643 | 2.372513056 | 0.220332 | 10.7679 | 4.88E-27 | 1.51E-25 | Up |
| AE000660.4 | 2.470824138 | -2.350713979 | 0.70466 | -3.33596 | 0.00085 | 0.002564 | Down |
| AE000661.37 | 66.62700432 | 3.943546635 | 0.315774 | 12.4885 | 8.63E-36 | 4.70E-34 | Up |
| AE000661.50 | 1.782417904 | 3.569720536 | 0.426143 | 8.376812 | 5.44E-17 | 7.99E-16 | Up |
| AE000662.93 | 1.26067838 | 2.464889511 | 0.446418 | 5.52148 | 3.36E-08 | 2.11E-07 | Up |
| AF001548.4 | 5.345499631 | -1.349494966 | 0.329273 | -4.0984 | 4.16E-05 | 0.000165 | Down |
| AF001548.6 | 2.613763439 | -1.000813043 | 0.331275 | -3.02109 | 0.002519 | 0.006747 | Down |
| AF013593.1 | 1.283900502 | -3.020247086 | 1.037644 | -2.91068 | 0.003606 | 0.009286 | Down |
| AF064858.7 | 1.946032255 | -1.912315782 | 0.623197 | -3.06856 | 0.002151 | 0.005881 | Down |
| AF064860.5 | 1.364626528 | -1.612399831 | 0.391653 | -4.11691 | 3.84E-05 | 0.000153 | Down |
| AF064860.7 | 7.616377117 | -1.517560263 | 0.27752 | -5.46829 | 4.54E-08 | 2.80E-07 | Down |
| AF067845.1 | 6.286744393 | -1.634345783 | 0.289649 | -5.6425 | 1.68E-08 | 1.09E-07 | Down |
| AF121897.4 | 7.598049207 | -2.120885696 | 0.370621 | -5.72252 | 1.05E-08 | 7.02E-08 | Down |
| AF131215.8 | 30.74853902 | 1.47361143 | 0.17796 | 8.280583 | 1.23E-16 | 1.75E-15 | Up |
| AF131216.1 | 6.225372206 | -1.002772669 | 0.215536 | -4.65245 | 3.28E-06 | 1.57E-05 | Down |
| AF131216.5 | 66.14551272 | -2.506426091 | 0.18891 | -13.2678 | 3.56E-40 | 2.52E-38 | Down |
| AF131217.1 | 24.49497713 | -1.130011122 | 0.267124 | -4.23028 | 2.33E-05 | 9.68E-05 | Down |
| AF146191.4 | 7.162511347 | -1.037254372 | 0.177306 | -5.85009 | 4.91E-09 | 3.40E-08 | Down |
| AF213884.1 | 0.799704149 | -1.657559839 | 0.604845 | -2.74047 | 0.006135 | 0.014834 | Down |
| AF241726.2 | 1.442611794 | -2.515543936 | 0.458104 | -5.49121 | 3.99E-08 | 2.48E-07 | Down |
| AFF2 | 238.8663858 | 1.3171587 | 0.181445 | 7.259278 | 3.89E-13 | 4.08E-12 | Up |
| AGBL1 | 1.392541963 | -1.681491228 | 0.741992 | -2.26618 | 0.02344 | 0.047373 | Down |
| AGBL5-IT1 | 2.259871527 | -1.179602137 | 0.317255 | -3.71815 | 0.000201 | 0.000698 | Down |
| AGMO | 14.94360587 | -1.24306211 | 0.204347 | -6.08309 | 1.18E-09 | 8.79E-09 | Down |
| AGPAT4 | 361.6405485 | -1.319751198 | 0.103575 | -12.742 | 3.46E-37 | 2.04E-35 | Down |
| AGPAT4-IT1 | 18.27558394 | -1.365443854 | 0.18799 | -7.2634 | 3.77E-13 | 3.96E-12 | Down |
| AGR2 | 554.2365547 | 2.663530981 | 0.206681 | 12.88716 | 5.32E-38 | 3.28E-36 | Up |
| AGR3 | 33.02213703 | -2.760103236 | 0.253076 | -10.9062 | 1.08E-27 | 3.51E-26 | Down |
| AGT | 133.0800857 | 1.033034309 | 0.164971 | 6.261924 | 3.80E-10 | 2.99E-09 | Up |
| AGTR1 | 109.7823916 | -2.393212749 | 0.183527 | -13.0401 | 7.23E-39 | 4.70E-37 | Down |
| AGTR2 | 3.919228432 | -1.695981582 | 0.401602 | -4.22304 | 2.41E-05 | 9.97E-05 | Down |
| AHCYP5 | 0.950011108 | -1.373880838 | 0.583861 | -2.35309 | 0.018618 | 0.038869 | Down |
| AHNAK2 | 5277.332699 | 3.30568895 | 0.171619 | 19.26179 | 1.12E-82 | 5.30E-80 | Up |
| AHR | 6678.53378 | 1.232093026 | 0.076569 | 16.09125 | 2.94E-58 | 5.41E-56 | Up |
| AHSP | 1.622942643 | -1.458037919 | 0.318485 | -4.57805 | 4.69E-06 | 2.19E-05 | Down |
| AICDA | 22.82413586 | -1.569938108 | 0.520407 | -3.01675 | 0.002555 | 0.006834 | Down |
| AIF1L | 4426.619493 | -1.187060573 | 0.11004 | -10.7876 | 3.94E-27 | 1.23E-25 | Down |
| AIMP1P1 | 5.72297038 | -1.327572525 | 0.304327 | -4.36232 | 1.29E-05 | 5.58E-05 | Down |
| AIRE | 7.707785029 | -1.576977709 | 0.409037 | -3.85534 | 0.000116 | 0.00042 | Down |
| AJ011931.1 | 2.184316836 | -1.063668646 | 0.461204 | -2.30629 | 0.021095 | 0.043291 | Down |
| AJ011932.1 | 5.176189922 | -1.351789593 | 0.273219 | -4.94765 | 7.51E-07 | 3.94E-06 | Down |
| AJ239322.1 | 1.316469716 | -2.469230545 | 0.944679 | -2.61383 | 0.008953 | 0.020692 | Down |
| AJAP1 | 155.0356305 | 1.169420319 | 0.175071 | 6.679682 | 2.39E-11 | 2.11E-10 | Up |
| AK4P5 | 0.696642515 | -1.270797024 | 0.517259 | -2.45679 | 0.014018 | 0.030502 | Down |
| AKAP17BP | 2.824316599 | -1.52723215 | 0.407656 | -3.74638 | 0.000179 | 0.00063 | Down |
| AKAP3 | 67.08998528 | -1.085277965 | 0.139144 | -7.79967 | 6.21E-15 | 7.77E-14 | Down |
| AKIRIN1P1 | 2.642098094 | -2.387089278 | 0.321911 | -7.41536 | 1.21E-13 | 1.34E-12 | Down |
| AKIRIN1P2 | 1.215931813 | -1.459382783 | 0.490428 | -2.97573 | 0.002923 | 0.007704 | Down |
| AKNAD1 | 5.31234598 | -2.086850404 | 0.295373 | -7.06514 | 1.60E-12 | 1.59E-11 | Down |
| AKR1B10P1 | 1.501167982 | -1.7163232 | 0.521589 | -3.29057 | 0.001 | 0.002969 | Down |
| AKR1C1 | 294.4368494 | -1.9384891 | 0.170694 | -11.3565 | 6.88E-30 | 2.61E-28 | Down |
| AKR1C2 | 182.0724809 | -1.935262339 | 0.180668 | -10.7117 | 8.97E-27 | 2.72E-25 | Down |
| AKR1C3 | 208.7208341 | -1.015845644 | 0.116947 | -8.68636 | 3.74E-18 | 6.02E-17 | Down |
| AKR1C4 | 1.044701309 | -1.979304386 | 0.670033 | -2.95404 | 0.003136 | 0.008207 | Down |
| AKR1C8P | 1.464318049 | -1.388515028 | 0.574287 | -2.41781 | 0.015614 | 0.033415 | Down |
| AKR1D1P1 | 0.994271626 | -1.390311635 | 0.499947 | -2.78092 | 0.005421 | 0.013306 | Down |
| AKR1E2 | 81.73945419 | -1.741575312 | 0.145868 | -11.9394 | 7.37E-33 | 3.37E-31 | Down |
| AKR7A3 | 18.26998086 | -1.229659766 | 0.140476 | -8.75351 | 2.07E-18 | 3.39E-17 | Down |
| AKR7L | 8.977752044 | -1.218230574 | 0.260161 | -4.68259 | 2.83E-06 | 1.37E-05 | Down |
| AL022476.2 | 8.76080251 | -1.138348391 | 0.186444 | -6.10557 | 1.02E-09 | 7.68E-09 | Down |
| AL022578.1 | 0.929447919 | -1.290455323 | 0.556427 | -2.31918 | 0.020385 | 0.042027 | Down |
| AL035610.1 | 12.82684699 | -2.438760403 | 0.273271 | -8.92434 | 4.48E-19 | 7.79E-18 | Down |
| AL035610.2 | 8.065349196 | -2.408561965 | 0.26797 | -8.98818 | 2.51E-19 | 4.45E-18 | Down |
| AL050303.10 | 1.73288694 | -1.843559208 | 0.786198 | -2.34491 | 0.019032 | 0.039601 | Down |
| AL132709.8 | 3.561314735 | -1.167029939 | 0.350706 | -3.32766 | 0.000876 | 0.002635 | Down |
| AL157871.2 | 16.8036826 | -1.001774837 | 0.180025 | -5.56463 | 2.63E-08 | 1.67E-07 | Down |
| AL157902.3 | 1.044219078 | -1.810597554 | 0.436697 | -4.14612 | 3.38E-05 | 0.000136 | Down |
| AL161668.5 | 0.770811602 | -1.847928538 | 0.528852 | -3.49423 | 0.000475 | 0.001521 | Down |
| AL356585.3 | 3.567761758 | -1.190728736 | 0.357995 | -3.32611 | 0.000881 | 0.002649 | Down |
| AL358852.1 | 16.32936859 | -1.150990706 | 0.127462 | -9.0301 | 1.72E-19 | 3.08E-18 | Down |
| AL450992.2 | 17.79198533 | 1.615405196 | 0.221056 | 7.307682 | 2.72E-13 | 2.90E-12 | Up |
| AL592528.1 | 3.260106816 | -1.664281179 | 0.476755 | -3.49086 | 0.000481 | 0.001538 | Down |
| ALAS2 | 26.07614656 | -1.852823612 | 0.215397 | -8.60191 | 7.84E-18 | 1.23E-16 | Down |
| ALDH1A1 | 5484.524004 | -1.244328771 | 0.114595 | -10.8585 | 1.82E-27 | 5.84E-26 | Down |
| ALDH1A2 | 127.1647685 | -1.539475143 | 0.176782 | -8.70831 | 3.08E-18 | 4.99E-17 | Down |
| ALDH1A3 | 513.4451015 | 2.373645104 | 0.166147 | 14.2864 | 2.66E-46 | 2.74E-44 | Up |
| ALDH1L1 | 64.56223199 | -1.417589213 | 0.18264 | -7.76164 | 8.38E-15 | 1.04E-13 | Down |
| ALDH1L1-AS2 | 3.826633654 | -2.424998372 | 0.464833 | -5.21693 | 1.82E-07 | 1.04E-06 | Down |
| ALDH3B1 | 329.1496054 | 1.879298854 | 0.096064 | 19.56304 | 3.19E-85 | 1.70E-82 | Up |
| ALDH3B2 | 5.491260872 | 1.579913782 | 0.427358 | 3.696929 | 0.000218 | 0.000753 | Up |
| ALDH8A1 | 9.593817761 | -1.270936698 | 0.263281 | -4.82731 | 1.38E-06 | 6.98E-06 | Down |
| ALG3P1 | 3.44128814 | -1.246198692 | 0.337239 | -3.6953 | 0.00022 | 0.000757 | Down |
| ALLC | 1.805564417 | -1.982429591 | 0.542659 | -3.65318 | 0.000259 | 0.00088 | Down |
| ALOX15B | 336.2298126 | 2.615199699 | 0.18286 | 14.30162 | 2.14E-46 | 2.22E-44 | Up |
| ALOX5 | 1086.460804 | 2.328897785 | 0.217721 | 10.69673 | 1.05E-26 | 3.18E-25 | Up |
| ALOX5AP | 316.1887743 | 1.348017544 | 0.160476 | 8.400126 | 4.46E-17 | 6.61E-16 | Up |
| ALPP | 2.743243342 | -1.878271957 | 0.472863 | -3.97213 | 7.12E-05 | 0.00027 | Down |
| ALX1 | 2.594894913 | -1.489724763 | 0.329478 | -4.52148 | 6.14E-06 | 2.81E-05 | Down |
| ALX4 | 18.00766208 | -1.300174433 | 0.193178 | -6.73045 | 1.69E-11 | 1.51E-10 | Down |
| AMBP | 5.868085656 | -1.78403131 | 0.203945 | -8.7476 | 2.18E-18 | 3.56E-17 | Down |
| AMD1P1 | 1.388973182 | -1.190470069 | 0.420461 | -2.83134 | 0.004635 | 0.011597 | Down |
| AMELX | 1.608648979 | -1.821104487 | 0.391784 | -4.64823 | 3.35E-06 | 1.60E-05 | Down |
| AMELY | 0.636628356 | -2.007439176 | 0.742904 | -2.70215 | 0.006889 | 0.016435 | Down |
| AMIGO2 | 589.0948025 | 2.05768124 | 0.112824 | 18.23795 | 2.58E-74 | 9.05E-72 | Up |
| AMMECR1LP1 | 2.258086908 | -2.323126052 | 0.4273 | -5.43675 | 5.43E-08 | 3.31E-07 | Down |
| AMN | 30.51955558 | -1.537399578 | 0.190683 | -8.06261 | 7.47E-16 | 9.99E-15 | Down |
| AMOT | 1303.956049 | 1.171738663 | 0.085834 | 13.65124 | 1.98E-42 | 1.62E-40 | Up |
| AMTN | 1.682575326 | 1.624054915 | 0.44732 | 3.630632 | 0.000283 | 0.000953 | Up |
| AMY1B | 35.11691926 | 1.659050528 | 0.300974 | 5.512271 | 3.54E-08 | 2.21E-07 | Up |
| AMY1C | 211.7734439 | 1.761840931 | 0.176511 | 9.981474 | 1.84E-23 | 4.41E-22 | Up |
| AMZ2P2 | 9.426142346 | -1.624199847 | 0.238059 | -6.82266 | 8.94E-12 | 8.22E-11 | Down |
| ANGPT4 | 47.78461408 | -1.528838131 | 0.156737 | -9.75419 | 1.77E-22 | 3.95E-21 | Down |
| ANGPTL1 | 1305.680586 | -1.576525552 | 0.125996 | -12.5125 | 6.38E-36 | 3.51E-34 | Down |
| ANHX | 1.559776303 | -1.989707992 | 0.774324 | -2.5696 | 0.010181 | 0.023098 | Down |
| ANK2 | 2283.207406 | -1.688794043 | 0.133494 | -12.6508 | 1.11E-36 | 6.38E-35 | Down |
| ANKFN1 | 13.30998998 | -2.359034046 | 0.296531 | -7.95543 | 1.79E-15 | 2.32E-14 | Down |
| ANKH | 2440.509588 | 1.174183406 | 0.078229 | 15.0096 | 6.35E-51 | 8.07E-49 | Up |
| ANKK1 | 19.7161472 | -1.595144504 | 0.168723 | -9.45423 | 3.25E-21 | 6.66E-20 | Down |
| ANKRD18A | 199.7707633 | -1.954455532 | 0.18999 | -10.2872 | 8.05E-25 | 2.13E-23 | Down |
| ANKRD18B | 6.710872228 | -1.67348163 | 0.275006 | -6.08525 | 1.16E-09 | 8.68E-09 | Down |
| ANKRD18CP | 19.04023764 | -1.553130788 | 0.186957 | -8.30742 | 9.78E-17 | 1.40E-15 | Down |
| ANKRD18DP | 3.67334374 | -1.066958902 | 0.400519 | -2.66394 | 0.007723 | 0.018162 | Down |
| ANKRD20A1 | 31.18040752 | -1.667452153 | 0.223653 | -7.45553 | 8.95E-14 | 1.00E-12 | Down |
| ANKRD20A17P | 10.35187803 | -1.976380522 | 0.228147 | -8.66276 | 4.60E-18 | 7.36E-17 | Down |
| ANKRD20A19P | 4.618432618 | -1.10070701 | 0.478363 | -2.30099 | 0.021392 | 0.043819 | Down |
| ANKRD20A2 | 19.91516549 | -1.579533286 | 0.240172 | -6.57667 | 4.81E-11 | 4.12E-10 | Down |
| ANKRD20A3 | 28.08800577 | -2.051441181 | 0.243122 | -8.43791 | 3.23E-17 | 4.83E-16 | Down |
| ANKRD20A4 | 48.39126802 | -1.311287356 | 0.222395 | -5.89622 | 3.72E-09 | 2.61E-08 | Down |
| ANKRD20A7P | 18.13308365 | -1.063007791 | 0.269451 | -3.94509 | 7.98E-05 | 0.000299 | Down |
| ANKRD20A8P | 68.14754076 | -2.283857706 | 0.20288 | -11.2572 | 2.13E-29 | 7.87E-28 | Down |
| ANKRD22 | 34.37491646 | 1.64769172 | 0.286349 | 5.754142 | 8.71E-09 | 5.87E-08 | Up |
| ANKRD26P1 | 5.001447968 | -2.15438179 | 0.470892 | -4.57511 | 4.76E-06 | 2.21E-05 | Down |
| ANKRD26P3 | 2.087449477 | -1.683241463 | 0.723431 | -2.32675 | 0.019979 | 0.041303 | Down |
| ANKRD30A | 2.198663029 | -1.970772391 | 0.550634 | -3.5791 | 0.000345 | 0.001139 | Down |
| ANKRD30B | 10.11134949 | 1.277409036 | 0.321567 | 3.972445 | 7.11E-05 | 0.000269 | Up |
| ANKRD30BL | 6.751395056 | -1.532493766 | 0.285645 | -5.36503 | 8.09E-08 | 4.83E-07 | Down |
| ANKRD34C | 6.576189942 | -1.897240535 | 0.372935 | -5.08733 | 3.63E-07 | 1.99E-06 | Down |
| ANKRD37 | 141.6531297 | -1.326614752 | 0.116291 | -11.4077 | 3.83E-30 | 1.47E-28 | Down |
| ANKRD45 | 22.85326077 | -1.03659635 | 0.15228 | -6.80717 | 9.95E-12 | 9.12E-11 | Down |
| ANKRD53 | 34.47035944 | -1.030215473 | 0.125098 | -8.23527 | 1.79E-16 | 2.52E-15 | Down |
| ANKRD62 | 4.779787461 | -1.630622983 | 0.367738 | -4.43419 | 9.24E-06 | 4.11E-05 | Down |
| ANKRD7 | 16.7971125 | -1.028307341 | 0.144421 | -7.12022 | 1.08E-12 | 1.09E-11 | Down |
| ANKS1B | 573.2564835 | -1.872054989 | 0.120151 | -15.5808 | 9.83E-55 | 1.53E-52 | Down |
| ANO2 | 61.31599351 | -1.02897447 | 0.165373 | -6.22214 | 4.90E-10 | 3.81E-09 | Down |
| ANO4 | 10.88393396 | -1.533747253 | 0.214661 | -7.14497 | 9.00E-13 | 9.15E-12 | Down |
| ANO5 | 480.1357139 | -1.222111998 | 0.106648 | -11.4593 | 2.11E-30 | 8.28E-29 | Down |
| ANP32BP1 | 1.56183451 | -1.935394035 | 0.738555 | -2.62051 | 0.00878 | 0.020337 | Down |
| ANP32D | 2.972957167 | -1.44383441 | 0.428591 | -3.36879 | 0.000755 | 0.002304 | Down |
| ANTXRL | 4.894908324 | -2.235117946 | 0.508346 | -4.39684 | 1.10E-05 | 4.82E-05 | Down |
| ANXA1 | 15541.7472 | 2.1418196 | 0.125503 | 17.0659 | 2.66E-65 | 6.43E-63 | Up |
| ANXA13 | 1.688685458 | -2.118802257 | 0.796562 | -2.65994 | 0.007816 | 0.018346 | Down |
| ANXA8 | 14.22161202 | 1.033679076 | 0.420499 | 2.458219 | 0.013963 | 0.030411 | Up |
| AOAH-IT1 | 1.841124606 | -1.045254676 | 0.388131 | -2.69305 | 0.00708 | 0.016832 | Down |
| AOC4P | 10.42945245 | -1.784472493 | 0.301216 | -5.92422 | 3.14E-09 | 2.22E-08 | Down |
| AOX1 | 577.0943674 | -2.051568428 | 0.176363 | -11.6327 | 2.81E-31 | 1.17E-29 | Down |
| AOX2P | 1.079815457 | -2.004382379 | 0.778359 | -2.57514 | 0.01002 | 0.022794 | Down |
| AOX3P | 1.963561553 | -1.957243268 | 0.602157 | -3.25038 | 0.001152 | 0.003375 | Down |
| AP000221.1 | 0.663221397 | -1.507809526 | 0.66495 | -2.26755 | 0.023356 | 0.047223 | Down |
| AP000235.2 | 1.815392913 | -2.191633894 | 0.856019 | -2.56026 | 0.010459 | 0.023656 | Down |
| AP000253.1 | 4.97002751 | -1.332716255 | 0.295327 | -4.51268 | 6.40E-06 | 2.92E-05 | Down |
| AP000322.53 | 1.048716762 | -1.135811928 | 0.413758 | -2.74511 | 0.006049 | 0.014657 | Down |
| AP000344.3 | 1.891743922 | -2.084142367 | 0.322526 | -6.46193 | 1.03E-10 | 8.60E-10 | Down |
| AP000344.4 | 1.837190333 | -2.784603004 | 0.521138 | -5.34331 | 9.13E-08 | 5.41E-07 | Down |
| AP000432.2 | 1.086887925 | -2.741888962 | 1.110207 | -2.46971 | 0.013522 | 0.029601 | Down |
| AP000438.2 | 2.97229947 | -2.120195113 | 0.256701 | -8.25939 | 1.46E-16 | 2.08E-15 | Down |
| AP000439.1 | 2.63549705 | -1.63070802 | 0.434657 | -3.75171 | 0.000176 | 0.000618 | Down |
| AP000473.8 | 17.65320555 | -1.041436275 | 0.176748 | -5.89221 | 3.81E-09 | 2.67E-08 | Down |
| AP000593.5 | 0.983162076 | -1.997688304 | 0.766612 | -2.60587 | 0.009164 | 0.021097 | Down |
| AP000593.6 | 1.581540126 | -1.012074519 | 0.391116 | -2.58766 | 0.009663 | 0.022096 | Down |
| AP000641.1 | 1.515472227 | -2.802365507 | 1.081255 | -2.59177 | 0.009548 | 0.021876 | Down |
| AP000647.3 | 2.288740197 | -3.338967805 | 1.245871 | -2.68003 | 0.007362 | 0.01741 | Down |
| AP000662.4 | 19.02900181 | -1.072700161 | 0.176429 | -6.08008 | 1.20E-09 | 8.94E-09 | Down |
| AP000688.14 | 3.031612943 | -1.39671945 | 0.352783 | -3.95915 | 7.52E-05 | 0.000283 | Down |
| AP000688.29 | 7.123357338 | -1.009052947 | 0.285533 | -3.53393 | 0.000409 | 0.001329 | Down |
| AP000695.6 | 3.479754968 | 1.225474466 | 0.318061 | 3.852952 | 0.000117 | 0.000424 | Up |
| AP000711.1 | 3.150987371 | -2.192059007 | 0.436858 | -5.01778 | 5.23E-07 | 2.80E-06 | Down |
| AP000857.2 | 8.257439406 | -1.652242586 | 0.235832 | -7.00602 | 2.45E-12 | 2.39E-11 | Down |
| AP000857.3 | 2.29876646 | -1.500666237 | 0.345308 | -4.34588 | 1.39E-05 | 5.98E-05 | Down |
| AP000997.3 | 10.56482781 | 1.126761507 | 0.365591 | 3.082027 | 0.002056 | 0.00565 | Up |
| AP001046.6 | 1.432445853 | -1.981775721 | 0.744119 | -2.66325 | 0.007739 | 0.018193 | Down |
| AP001059.5 | 0.598336845 | -1.194246479 | 0.516155 | -2.31374 | 0.020682 | 0.042575 | Down |
| AP001065.7 | 1.863250393 | -2.638668687 | 0.604608 | -4.36426 | 1.28E-05 | 5.54E-05 | Down |
| AP001187.9 | 8.042598199 | -1.84172625 | 0.342174 | -5.38243 | 7.35E-08 | 4.41E-07 | Down |
| AP001258.4 | 303.9890532 | 1.517296418 | 0.094036 | 16.1352 | 1.44E-58 | 2.71E-56 | Up |
| AP001331.1 | 1.993596248 | -1.656213493 | 0.475173 | -3.4855 | 0.000491 | 0.001565 | Down |
| AP001434.2 | 1.599658187 | 1.1622724 | 0.328362 | 3.539609 | 0.000401 | 0.001304 | Up |
| AP001437.1 | 4.599252436 | -1.233567533 | 0.193561 | -6.37303 | 1.85E-10 | 1.50E-09 | Down |
| AP001471.1 | 0.90111152 | -1.030466302 | 0.348929 | -2.95322 | 0.003145 | 0.008226 | Down |
| AP001601.2 | 0.470150641 | -1.641505131 | 0.528031 | -3.10873 | 0.001879 | 0.005224 | Down |
| AP001604.3 | 2.777268793 | -1.580968007 | 0.451702 | -3.50003 | 0.000465 | 0.001491 | Down |
| AP002414.1 | 1.188066984 | -1.542067834 | 0.629396 | -2.45007 | 0.014283 | 0.030993 | Down |
| AP002856.7 | 1.081670199 | -2.033650348 | 0.85942 | -2.3663 | 0.017967 | 0.037709 | Down |
| AP003041.1 | 18.10330925 | 1.287534538 | 0.266337 | 4.834228 | 1.34E-06 | 6.76E-06 | Up |
| AP003774.6 | 2.775617499 | -1.316379631 | 0.422545 | -3.11536 | 0.001837 | 0.005118 | Down |
| AP003900.6 | 1.191563756 | -2.734378229 | 1.068547 | -2.55897 | 0.010498 | 0.023726 | Down |
| AP004782.1 | 10.7749455 | -1.363007501 | 0.280859 | -4.853 | 1.22E-06 | 6.19E-06 | Down |
| AP005530.2 | 0.909846871 | -2.467358376 | 0.73932 | -3.33733 | 0.000846 | 0.002554 | Down |
| APC2 | 55.23818731 | -1.092931313 | 0.202566 | -5.39544 | 6.84E-08 | 4.12E-07 | Down |
| API5P2 | 4.180673135 | -1.698179823 | 0.34013 | -4.99273 | 5.95E-07 | 3.17E-06 | Down |
| APLN | 36.06438113 | 1.481263903 | 0.224391 | 6.601261 | 4.08E-11 | 3.52E-10 | Up |
| APLP2 | 37727.00926 | 1.132971989 | 0.09746 | 11.62494 | 3.08E-31 | 1.28E-29 | Up |
| APOA1 | 35.94721128 | -2.167299989 | 0.182467 | -11.8777 | 1.54E-32 | 6.98E-31 | Down |
| APOA4 | 1.834348301 | -2.395822288 | 0.473445 | -5.0604 | 4.18E-07 | 2.28E-06 | Down |
| APOA5 | 3.134002585 | -2.197349896 | 0.351857 | -6.245 | 4.24E-10 | 3.31E-09 | Down |
| APOBEC3H | 14.31327557 | 1.195115659 | 0.187786 | 6.364257 | 1.96E-10 | 1.59E-09 | Up |
| APOBEC4 | 8.257629308 | -1.24545155 | 0.296216 | -4.20454 | 2.62E-05 | 0.000108 | Down |
| APOC1 | 954.5005581 | 2.146259985 | 0.238332 | 9.005341 | 2.15E-19 | 3.83E-18 | Up |
| APOC1P1 | 5.505641048 | 2.858355953 | 0.39568 | 7.223909 | 5.05E-13 | 5.24E-12 | Up |
| APOC4-APOC2 | 50.55147367 | 1.296090769 | 0.290843 | 4.456321 | 8.34E-06 | 3.73E-05 | Up |
| APOD | 1595.574653 | -1.714467669 | 0.182828 | -9.37751 | 6.75E-21 | 1.35E-19 | Down |
| APOE | 6462.381428 | 2.381272347 | 0.199372 | 11.94385 | 6.99E-33 | 3.20E-31 | Up |
| APOF | 3.776852764 | -1.918112369 | 0.331427 | -5.78744 | 7.15E-09 | 4.86E-08 | Down |
| APOL1 | 937.8152365 | 1.057586275 | 0.115832 | 9.130346 | 6.83E-20 | 1.26E-18 | Up |
| APOL4 | 207.2278143 | 1.512249139 | 0.154315 | 9.799758 | 1.13E-22 | 2.56E-21 | Up |
| AQP10 | 2.111773345 | -2.009044029 | 0.575221 | -3.49264 | 0.000478 | 0.001529 | Down |
| AQP12A | 0.840650338 | -1.890828623 | 0.452888 | -4.17505 | 2.98E-05 | 0.000121 | Down |
| AQP12B | 2.349937236 | -1.281956725 | 0.4263 | -3.00717 | 0.002637 | 0.007025 | Down |
| AQP4 | 860.8614073 | -1.231454144 | 0.149851 | -8.21786 | 2.07E-16 | 2.90E-15 | Down |
| AQP5 | 154.550449 | 1.354626891 | 0.161079 | 8.409721 | 4.11E-17 | 6.11E-16 | Up |
| AQP7 | 54.8448272 | -1.816128355 | 0.192221 | -9.44815 | 3.45E-21 | 7.05E-20 | Down |
| AQPEP | 22.33647484 | -1.913347458 | 0.243209 | -7.86709 | 3.63E-15 | 4.63E-14 | Down |
| AR | 695.0225719 | -1.389426666 | 0.108631 | -12.7903 | 1.86E-37 | 1.11E-35 | Down |
| ARG1 | 5.820551985 | -1.072189619 | 0.257266 | -4.16763 | 3.08E-05 | 0.000125 | Down |
| ARGFXP1 | 1.630552438 | -2.258187378 | 0.731256 | -3.0881 | 0.002014 | 0.00555 | Down |
| ARHGAP19 | 707.2692062 | 1.285132394 | 0.116988 | 10.98513 | 4.51E-28 | 1.51E-26 | Up |
| ARHGAP24 | 879.2214578 | -1.471903494 | 0.102789 | -14.3197 | 1.65E-46 | 1.72E-44 | Down |
| ARHGAP36 | 2023.935643 | 5.34904644 | 0.392485 | 13.62866 | 2.71E-42 | 2.18E-40 | Up |
| ARHGAP42P1 | 2.219062239 | -3.373164527 | 1.125107 | -2.99808 | 0.002717 | 0.00721 | Down |
| ARHGEF19-AS1 | 1.620753429 | 1.218438918 | 0.528396 | 2.30592 | 0.021115 | 0.043323 | Up |
| ARHGEF28 | 1280.424268 | -1.21527089 | 0.101557 | -11.9664 | 5.33E-33 | 2.45E-31 | Down |
| ARHGEF37 | 234.587346 | -1.018422211 | 0.114777 | -8.87306 | 7.12E-19 | 1.22E-17 | Down |
| ARHGEF38 | 372.3536776 | 1.470627518 | 0.202701 | 7.255156 | 4.01E-13 | 4.20E-12 | Up |
| ARL14EPL | 3.581767372 | 2.159558594 | 0.441833 | 4.887722 | 1.02E-06 | 5.24E-06 | Up |
| ARL5C | 2.780118657 | -1.285884807 | 0.354104 | -3.63138 | 0.000282 | 0.00095 | Down |
| ARMC3 | 11.32738554 | -2.350435116 | 0.335912 | -6.99718 | 2.61E-12 | 2.53E-11 | Down |
| ARMC4P1 | 15.90575075 | -1.576127914 | 0.171293 | -9.20136 | 3.53E-20 | 6.68E-19 | Down |
| ARMC8P1 | 3.977785885 | -2.774005055 | 0.314385 | -8.8236 | 1.11E-18 | 1.87E-17 | Down |
| ARMCX3 | 6113.410275 | 2.217214722 | 0.124328 | 17.83365 | 3.87E-71 | 1.18E-68 | Up |
| ARMCX6 | 1011.925041 | 1.295452593 | 0.082734 | 15.65801 | 2.93E-55 | 4.77E-53 | Up |
| ARMCX7P | 20.08384289 | 1.31603993 | 0.49745 | 2.645574 | 0.008155 | 0.019046 | Up |
| ARMS2 | 2.564937143 | -1.270752923 | 0.423649 | -2.99954 | 0.002704 | 0.007179 | Down |
| ARNTL | 1317.504503 | 1.771915363 | 0.137862 | 12.85279 | 8.30E-38 | 5.06E-36 | Up |
| ARNTL2 | 199.2236473 | 1.336409392 | 0.178669 | 7.479825 | 7.44E-14 | 8.38E-13 | Up |
| ARPP21 | 5.436926254 | -1.321072067 | 0.561951 | -2.35087 | 0.01873 | 0.039049 | Down |
| ARSE | 14.84758364 | 1.420150905 | 0.23899 | 5.942301 | 2.81E-09 | 2.00E-08 | Up |
| ARSF | 1.970935087 | -1.444135416 | 0.454529 | -3.17721 | 0.001487 | 0.004239 | Down |
| ARSI | 24.06925044 | 1.176038063 | 0.221078 | 5.319562 | 1.04E-07 | 6.12E-07 | Up |
| ARSJ | 137.4274002 | 1.299074594 | 0.143222 | 9.070367 | 1.19E-19 | 2.15E-18 | Up |
| ART1 | 6.81060107 | -1.085246002 | 0.311932 | -3.47911 | 0.000503 | 0.001599 | Down |
| ART2P | 3.051265191 | -2.619335807 | 0.344248 | -7.60886 | 2.77E-14 | 3.25E-13 | Down |
| ART4 | 196.0420661 | -1.423562358 | 0.187592 | -7.58862 | 3.23E-14 | 3.78E-13 | Down |
| ART5 | 25.65500476 | -1.43493267 | 0.169842 | -8.44864 | 2.95E-17 | 4.43E-16 | Down |
| ASB10 | 2.086486729 | -1.789432537 | 0.653801 | -2.73697 | 0.006201 | 0.014976 | Down |
| ASB15 | 3.583565685 | -1.827824276 | 0.635017 | -2.87839 | 0.003997 | 0.010166 | Down |
| ASB5 | 2.667971766 | -2.587714923 | 0.569471 | -4.54407 | 5.52E-06 | 2.54E-05 | Down |
| ASGR1 | 65.87935192 | -1.24208454 | 0.140925 | -8.81378 | 1.21E-18 | 2.03E-17 | Down |
| ASIC3 | 18.37622529 | -1.25382902 | 0.212293 | -5.90611 | 3.50E-09 | 2.46E-08 | Down |
| ASNSP3 | 1.577184677 | -2.019909487 | 0.631641 | -3.19787 | 0.001384 | 0.003976 | Down |
| ASNSP6 | 2.515763907 | -1.790276145 | 0.419346 | -4.26921 | 1.96E-05 | 8.25E-05 | Down |
| ASPA | 76.60241544 | -1.387413444 | 0.137361 | -10.1005 | 5.50E-24 | 1.37E-22 | Down |
| ASPHD1 | 12.87945626 | 1.116734557 | 0.274999 | 4.060867 | 4.89E-05 | 0.000191 | Up |
| ASPN | 746.9279156 | 1.394801359 | 0.166272 | 8.388653 | 4.92E-17 | 7.25E-16 | Up |
| ASS1P5 | 1.695187217 | -1.354914936 | 0.52202 | -2.59552 | 0.009445 | 0.021675 | Down |
| ASS1P7 | 1.22257453 | -1.083200787 | 0.453632 | -2.38784 | 0.016948 | 0.035873 | Down |
| ASS1P9 | 1.507634432 | -2.403264551 | 0.797983 | -3.01168 | 0.002598 | 0.006935 | Down |
| ASTN2-AS1 | 1.381250207 | -1.185221368 | 0.512989 | -2.31042 | 0.020865 | 0.042908 | Down |
| ASXL3 | 380.3122472 | -2.426990853 | 0.197921 | -12.2624 | 1.44E-34 | 7.18E-33 | Down |
| ASZ1 | 0.929614839 | -2.33559336 | 0.945717 | -2.46965 | 0.013524 | 0.029604 | Down |
| ATCAY | 3.86066185 | -1.121219408 | 0.466168 | -2.40518 | 0.016164 | 0.034421 | Down |
| ATF4P1 | 1.854338869 | -1.425537687 | 0.51442 | -2.77115 | 0.005586 | 0.013664 | Down |
| ATF4P2 | 1.854338869 | -1.425537687 | 0.51442 | -2.77115 | 0.005586 | 0.013664 | Down |
| ATF4P3 | 2.349475046 | -1.173248288 | 0.378707 | -3.09803 | 0.001948 | 0.005389 | Down |
| ATF4P4 | 3.075363353 | -1.178132161 | 0.407025 | -2.8945 | 0.003798 | 0.00972 | Down |
| ATG9B | 21.81099899 | -1.014609144 | 0.25261 | -4.0165 | 5.91E-05 | 0.000227 | Down |
| ATOH1 | 1.180189737 | -1.766174504 | 0.656263 | -2.69126 | 0.007118 | 0.016909 | Down |
| ATP10A | 297.5582729 | 1.046404712 | 0.134301 | 7.791466 | 6.62E-15 | 8.27E-14 | Up |
| ATP11A | 3370.15065 | 1.394102195 | 0.105089 | 13.26587 | 3.65E-40 | 2.58E-38 | Up |
| ATP11AUN | 1.466025052 | -1.573835576 | 0.528571 | -2.97753 | 0.002906 | 0.007666 | Down |
| ATP13A4 | 6348.408212 | 1.159776486 | 0.106529 | 10.88695 | 1.33E-27 | 4.31E-26 | Up |
| ATP1A3 | 18.56997788 | -1.541056328 | 0.328007 | -4.69824 | 2.62E-06 | 1.27E-05 | Down |
| ATP1B4 | 3.965067932 | -1.888771231 | 0.750092 | -2.51805 | 0.011801 | 0.026311 | Down |
| ATP2C2 | 271.1964186 | -2.148887681 | 0.135339 | -15.8778 | 9.03E-57 | 1.57E-54 | Down |
| ATP4A | 5.902996182 | -1.394913965 | 0.246932 | -5.64899 | 1.61E-08 | 1.05E-07 | Down |
| ATP4B | 6.837083767 | -1.037864777 | 0.19227 | -5.39795 | 6.74E-08 | 4.06E-07 | Down |
| ATP5A1P3 | 2.947102795 | -2.139420485 | 0.345286 | -6.19609 | 5.79E-10 | 4.46E-09 | Down |
| ATP5BP1 | 1.214097522 | -2.969166011 | 1.154573 | -2.57166 | 0.010121 | 0.022987 | Down |
| ATP6V0E2-AS1 | 96.39630367 | -1.47610759 | 0.115509 | -12.7792 | 2.14E-37 | 1.28E-35 | Down |
| ATP8B5P | 7.991200354 | -1.79548706 | 0.315285 | -5.69481 | 1.24E-08 | 8.18E-08 | Down |
| ATRNL1 | 105.7031799 | -1.363282793 | 0.117755 | -11.5773 | 5.37E-31 | 2.20E-29 | Down |
| AVPR1A | 639.8718087 | -1.851138358 | 0.13826 | -13.3889 | 7.02E-41 | 5.21E-39 | Down |
| AVPR2 | 38.02539715 | -1.106107522 | 0.129428 | -8.54611 | 1.27E-17 | 1.97E-16 | Down |
| AXDND1 | 25.72698411 | -1.290491411 | 0.186303 | -6.92683 | 4.30E-12 | 4.09E-11 | Down |
| AZGP1 | 11.82799353 | -1.679403274 | 0.334095 | -5.02672 | 4.99E-07 | 2.68E-06 | Down |
| AZU1 | 3.554966751 | 2.179537108 | 0.480243 | 4.538407 | 5.67E-06 | 2.60E-05 | Up |
| B3GALNT1P1 | 8.732034795 | -1.057012674 | 0.243914 | -4.33354 | 1.47E-05 | 6.30E-05 | Down |
| B3GALT1 | 21.16346232 | -1.548273191 | 0.259972 | -5.95554 | 2.59E-09 | 1.86E-08 | Down |
| B3GALT2 | 113.4631081 | -1.491050344 | 0.1306 | -11.417 | 3.44E-30 | 1.33E-28 | Down |
| B3GALT5 | 227.4345173 | -1.495790766 | 0.180392 | -8.29188 | 1.11E-16 | 1.59E-15 | Down |
| B3GALT5-AS1 | 30.34613972 | -1.020245691 | 0.181816 | -5.61141 | 2.01E-08 | 1.30E-07 | Down |
| B3GAT1 | 589.2055605 | 1.841158502 | 0.159679 | 11.5304 | 9.27E-31 | 3.73E-29 | Up |
| B3GNT3 | 360.7753864 | 4.636054614 | 0.254934 | 18.18529 | 6.75E-74 | 2.33E-71 | Up |
| B3GNT6 | 2.585493574 | 1.298801302 | 0.408338 | 3.180698 | 0.001469 | 0.004193 | Up |
| B3GNT7 | 359.4319734 | 1.686063508 | 0.104138 | 16.19059 | 5.88E-59 | 1.14E-56 | Up |
| B3GNT8 | 61.29492565 | 1.099200996 | 0.103845 | 10.585 | 3.50E-26 | 1.02E-24 | Up |
| B4GALNT2 | 279.6873972 | -1.938490528 | 0.229956 | -8.42983 | 3.46E-17 | 5.17E-16 | Down |
| B4GALNT3 | 506.6233797 | 1.79256103 | 0.113984 | 15.72639 | 9.97E-56 | 1.66E-53 | Up |
| B4GALNT4 | 11.70163862 | -1.135576934 | 0.261288 | -4.34607 | 1.39E-05 | 5.98E-05 | Down |
| B4GALT5 | 1085.265861 | 1.106074389 | 0.065387 | 16.9159 | 3.44E-64 | 7.92E-62 | Up |
| B4GALT6 | 175.4077518 | 1.386422169 | 0.134142 | 10.33551 | 4.87E-25 | 1.31E-23 | Up |
| BAAT | 2.279779258 | -1.731761972 | 0.385331 | -4.49422 | 6.98E-06 | 3.17E-05 | Down |
| BAIAP3 | 206.0619696 | 2.109314158 | 0.171575 | 12.29383 | 9.78E-35 | 4.93E-33 | Up |
| BANCR | 1.758699211 | -1.068749877 | 0.268199 | -3.98491 | 6.75E-05 | 0.000257 | Down |
| BANF1P5 | 0.514423311 | -1.311774807 | 0.545026 | -2.40681 | 0.016092 | 0.034289 | Down |
| BANF2 | 4.458930258 | -2.931273857 | 0.271274 | -10.8056 | 3.24E-27 | 1.02E-25 | Down |
| BARHL1 | 4.471625355 | -1.431815822 | 0.346996 | -4.12632 | 3.69E-05 | 0.000148 | Down |
| BARX1 | 8.777593072 | -1.026633449 | 0.306288 | -3.35186 | 0.000803 | 0.002433 | Down |
| BARX2 | 13.08272873 | -2.078597558 | 0.274293 | -7.57801 | 3.51E-14 | 4.08E-13 | Down |
| BASP1 | 604.8277327 | 1.060025551 | 0.109032 | 9.722187 | 2.43E-22 | 5.36E-21 | Up |
| BBC3 | 157.611307 | 1.446796614 | 0.085519 | 16.91778 | 3.33E-64 | 7.72E-62 | Up |
| BBOX1-AS1 | 1.55669246 | 1.28328792 | 0.514332 | 2.495057 | 0.012594 | 0.027852 | Up |
| BCAR1P1 | 1.643450017 | -2.698196524 | 1.093735 | -2.46696 | 0.013627 | 0.029784 | Down |
| BCAR4 | 0.770771683 | -2.395904729 | 0.874139 | -2.74087 | 0.006128 | 0.014818 | Down |
| BCAS1 | 12.27581144 | -1.207015015 | 0.299396 | -4.0315 | 5.54E-05 | 0.000214 | Down |
| BCAT1 | 963.9105169 | 1.706503978 | 0.160294 | 10.64611 | 1.82E-26 | 5.37E-25 | Up |
| BCL2 | 2938.955358 | -1.198217198 | 0.075243 | -15.9247 | 4.27E-57 | 7.49E-55 | Down |
| BCL9P1 | 2.194893594 | -1.235582191 | 0.509155 | -2.42673 | 0.015235 | 0.032745 | Down |
| BCO2 | 92.59104406 | -1.404375089 | 0.129779 | -10.8213 | 2.73E-27 | 8.65E-26 | Down |
| BDKRB1 | 5.870972451 | -1.127553313 | 0.255609 | -4.41124 | 1.03E-05 | 4.53E-05 | Down |
| BDNF | 107.157443 | 1.016632569 | 0.1764 | 5.763223 | 8.25E-09 | 5.58E-08 | Up |
| BDP1P | 1.608820289 | -2.713414391 | 0.636407 | -4.26365 | 2.01E-05 | 8.44E-05 | Down |
| BEAN1 | 124.7427717 | 1.092443879 | 0.150065 | 7.279815 | 3.34E-13 | 3.53E-12 | Up |
| BEGAIN | 30.55961325 | -1.581919441 | 0.215461 | -7.34204 | 2.10E-13 | 2.26E-12 | Down |
| BEND2 | 1.494179891 | -1.319875743 | 0.469259 | -2.81268 | 0.004913 | 0.012194 | Down |
| BEND4 | 24.29670655 | -1.259885313 | 0.401808 | -3.13554 | 0.001715 | 0.004818 | Down |
| BEST2 | 3.70628546 | -1.436834112 | 0.357024 | -4.02448 | 5.71E-05 | 0.00022 | Down |
| BEST3 | 4.808476255 | -2.264938245 | 0.685819 | -3.30253 | 0.000958 | 0.002858 | Down |
| BET1P1 | 1.810704416 | -1.034313219 | 0.335036 | -3.08717 | 0.002021 | 0.005565 | Down |
| BEX1 | 237.0417461 | -1.773565742 | 0.189223 | -9.37288 | 7.06E-21 | 1.41E-19 | Down |
| BFSP2 | 5.487393981 | -1.129613895 | 0.297174 | -3.80119 | 0.000144 | 0.000514 | Down |
| BGN | 2313.147987 | 1.074815409 | 0.117969 | 9.111022 | 8.16E-20 | 1.50E-18 | Up |
| BHLHE40 | 2259.105819 | 1.966782862 | 0.095076 | 20.68644 | 4.59E-95 | 3.49E-92 | Up |
| BHLHE41 | 4317.205429 | 1.451510351 | 0.110719 | 13.10991 | 2.89E-39 | 1.93E-37 | Up |
| BHMG1 | 2.360447416 | -2.197657621 | 0.482557 | -4.55419 | 5.26E-06 | 2.43E-05 | Down |
| BID | 620.0548473 | 2.018085016 | 0.114957 | 17.55506 | 5.44E-69 | 1.51E-66 | Up |
| BIRC7 | 62.36801111 | 4.294846146 | 0.322974 | 13.29779 | 2.38E-40 | 1.70E-38 | Up |
| BIRC8 | 2.137625019 | -2.292651837 | 0.775823 | -2.95512 | 0.003125 | 0.008181 | Down |
| BLACAT1 | 119.9416664 | 1.271672959 | 0.127971 | 9.937195 | 2.87E-23 | 6.78E-22 | Up |
| BLK | 99.39685243 | -1.100200528 | 0.366036 | -3.00572 | 0.00265 | 0.007053 | Down |
| BMP1 | 1003.204417 | 1.274699422 | 0.073248 | 17.4024 | 7.91E-68 | 2.10E-65 | Up |
| BMP15 | 1.319792063 | -2.75160313 | 0.948451 | -2.90115 | 0.003718 | 0.009541 | Down |
| BMP3 | 15.35778307 | -1.348459724 | 0.262804 | -5.13104 | 2.88E-07 | 1.60E-06 | Down |
| BMP5 | 39.65533617 | -1.540218443 | 0.18054 | -8.53117 | 1.45E-17 | 2.23E-16 | Down |
| BMP7-AS1 | 2.221517715 | -1.423515362 | 0.329842 | -4.31575 | 1.59E-05 | 6.79E-05 | Down |
| BMP8A | 739.9457207 | -2.599288679 | 0.206824 | -12.5676 | 3.18E-36 | 1.79E-34 | Down |
| BMP8B | 382.6317814 | -1.081093481 | 0.120791 | -8.9501 | 3.55E-19 | 6.21E-18 | Down |
| BMS1P11 | 5.948108296 | -1.421126329 | 0.338596 | -4.19711 | 2.70E-05 | 0.000111 | Down |
| BMS1P14 | 3.165058325 | -1.085552041 | 0.308612 | -3.51753 | 0.000436 | 0.001406 | Down |
| BMS1P16 | 1.758704755 | -1.432308997 | 0.478754 | -2.99174 | 0.002774 | 0.007346 | Down |
| BMS1P8 | 3.480443337 | -1.766702639 | 0.626278 | -2.82096 | 0.004788 | 0.011934 | Down |
| BNC1 | 108.192583 | 3.005024004 | 0.165491 | 18.15822 | 1.11E-73 | 3.75E-71 | Up |
| BNIP3P1 | 2.214810252 | -1.170331652 | 0.385297 | -3.03748 | 0.002386 | 0.006442 | Down |
| BNIP3P15 | 2.208131041 | 1.113242577 | 0.307404 | 3.621436 | 0.000293 | 0.000983 | Up |
| BNIP3P18 | 7.701021103 | -1.441167692 | 0.22512 | -6.40177 | 1.54E-10 | 1.26E-09 | Down |
| BNIP3P41 | 5.444185878 | -2.468191965 | 0.76613 | -3.22164 | 0.001275 | 0.003694 | Down |
| BNIPL | 242.8316483 | 1.91903496 | 0.146015 | 13.1427 | 1.87E-39 | 1.27E-37 | Up |
| bP-21201H5.1 | 3.924540816 | -2.053844955 | 0.430854 | -4.76691 | 1.87E-06 | 9.27E-06 | Down |
| bP-21201H5.2 | 3.122294326 | -1.524442531 | 0.464793 | -3.27983 | 0.001039 | 0.003075 | Down |
| bP-21264C1.1 | 28.26830003 | -1.623159239 | 0.190441 | -8.52318 | 1.55E-17 | 2.39E-16 | Down |
| bP-21264C1.3 | 25.62283725 | -1.630774526 | 0.184455 | -8.84102 | 9.48E-19 | 1.61E-17 | Down |
| bP-2171C21.2 | 2.177714734 | -1.366262164 | 0.324257 | -4.21352 | 2.51E-05 | 0.000104 | Down |
| bP-2189O9.1 | 4.666252325 | -2.127733349 | 0.41293 | -5.15277 | 2.57E-07 | 1.44E-06 | Down |
| bP-2189O9.2 | 59.71484027 | -2.146682793 | 0.176535 | -12.1601 | 5.07E-34 | 2.45E-32 | Down |
| bP-2189O9.4 | 2.32589359 | -1.742308189 | 0.474145 | -3.67463 | 0.000238 | 0.000815 | Down |
| BRD9P2 | 0.622947621 | -1.408036011 | 0.476888 | -2.95255 | 0.003152 | 0.00824 | Down |
| BRDT | 2.057838977 | -3.101938977 | 0.929753 | -3.3363 | 0.000849 | 0.002561 | Down |
| BRINP1 | 99.74665079 | 2.142946459 | 0.173381 | 12.35974 | 4.32E-35 | 2.26E-33 | Up |
| BRINP2 | 39.37411754 | 2.808778115 | 0.312704 | 8.98224 | 2.65E-19 | 4.69E-18 | Up |
| BRINP3 | 58.20779796 | -1.376255688 | 0.196381 | -7.0081 | 2.42E-12 | 2.35E-11 | Down |
| BRS3 | 4.662613642 | -2.212370138 | 0.354837 | -6.23488 | 4.52E-10 | 3.52E-09 | Down |
| BRSK2 | 54.89873477 | -1.697527888 | 0.149355 | -11.3657 | 6.20E-30 | 2.36E-28 | Down |
| BSN-AS2 | 3.47556562 | -1.767487583 | 0.394144 | -4.48437 | 7.31E-06 | 3.30E-05 | Down |
| BTBD11 | 786.0018378 | -1.197581701 | 0.102943 | -11.6335 | 2.79E-31 | 1.16E-29 | Down |
| BTBD16 | 3.641620422 | -1.603547692 | 0.297123 | -5.39692 | 6.78E-08 | 4.08E-07 | Down |
| BTBD17 | 10.65714084 | -2.570987734 | 0.23713 | -10.8421 | 2.17E-27 | 6.95E-26 | Down |
| BTBD7P1 | 4.366612163 | -1.036123599 | 0.325566 | -3.18253 | 0.00146 | 0.00417 | Down |
| BTF3P7 | 1.09810786 | 1.234076326 | 0.476335 | 2.590776 | 0.009576 | 0.021928 | Up |
| BTG4 | 0.733037486 | -1.825155769 | 0.567422 | -3.21658 | 0.001297 | 0.003753 | Down |
| BTN1A1 | 11.63664032 | -1.249230975 | 0.289837 | -4.31011 | 1.63E-05 | 6.95E-05 | Down |
| BTNL3 | 5.219696901 | -2.31186168 | 0.411169 | -5.62266 | 1.88E-08 | 1.22E-07 | Down |
| BTNL8 | 15.62305964 | -1.871082157 | 0.211463 | -8.84826 | 8.89E-19 | 1.51E-17 | Down |
| BTNL9 | 262.5530846 | -1.10608873 | 0.1586 | -6.97409 | 3.08E-12 | 2.96E-11 | Down |
| BVES | 109.0140267 | -1.035246923 | 0.114161 | -9.06827 | 1.21E-19 | 2.19E-18 | Down |
| BVES-AS1 | 5.299787161 | -2.234137105 | 0.288702 | -7.73855 | 1.01E-14 | 1.24E-13 | Down |
| BX255923.3 | 3.867635098 | -1.281091966 | 0.535977 | -2.3902 | 0.016839 | 0.035669 | Down |
| BX842568.2 | 8.985069862 | -1.15293976 | 0.151106 | -7.62998 | 2.35E-14 | 2.78E-13 | Down |
| C10orf105 | 17.91242657 | -1.035144293 | 0.249714 | -4.14533 | 3.39E-05 | 0.000137 | Down |
| C10orf107 | 39.0477184 | -1.643443324 | 0.252955 | -6.49698 | 8.20E-11 | 6.88E-10 | Down |
| C10orf113 | 10.49238538 | -1.299803674 | 0.240841 | -5.39693 | 6.78E-08 | 4.08E-07 | Down |
| C10orf120 | 1.415914987 | -2.543166423 | 1.114625 | -2.28163 | 0.022511 | 0.045761 | Down |
| C10orf55 | 37.04904681 | 2.125506612 | 0.193774 | 10.96902 | 5.39E-28 | 1.80E-26 | Up |
| C10orf62 | 2.288351167 | -1.801283522 | 0.419823 | -4.29058 | 1.78E-05 | 7.55E-05 | Down |
| C10orf67 | 13.12589096 | 1.216212776 | 0.216196 | 5.625505 | 1.85E-08 | 1.20E-07 | Up |
| C10orf71 | 11.13975855 | -1.704158968 | 0.53566 | -3.18142 | 0.001466 | 0.004184 | Down |
| C10orf82 | 43.08885912 | -1.437313111 | 0.152667 | -9.41472 | 4.74E-21 | 9.54E-20 | Down |
| C11orf53 | 1.663002389 | -2.128622575 | 0.592157 | -3.5947 | 0.000325 | 0.001079 | Down |
| C11orf74 | 473.3355515 | -1.641467214 | 0.111515 | -14.7197 | 4.82E-49 | 5.71E-47 | Down |
| C11orf80 | 231.5398865 | 1.181552831 | 0.088754 | 13.31272 | 1.95E-40 | 1.39E-38 | Up |
| C11orf85 | 4.368288568 | -1.059769249 | 0.25221 | -4.20193 | 2.65E-05 | 0.000109 | Down |
| C11orf86 | 0.915581676 | 1.424605772 | 0.587766 | 2.423765 | 0.015361 | 0.03297 | Up |
| C11orf87 | 4.246969562 | -1.923869929 | 0.355284 | -5.41501 | 6.13E-08 | 3.71E-07 | Down |
| C11orf88 | 7.829465816 | -2.36055835 | 0.278711 | -8.46954 | 2.46E-17 | 3.72E-16 | Down |
| C12orf49 | 6088.313793 | 1.156935034 | 0.090705 | 12.7549 | 2.93E-37 | 1.73E-35 | Up |
| C12orf56 | 21.03208563 | 1.74781186 | 0.274543 | 6.366249 | 1.94E-10 | 1.57E-09 | Up |
| C12orf74 | 7.644579394 | -1.083016854 | 0.453857 | -2.38625 | 0.017021 | 0.036001 | Down |
| C12orf77 | 2.880934312 | -2.034372186 | 0.580346 | -3.50545 | 0.000456 | 0.001464 | Down |
| C12orf80 | 2.806676445 | -3.231140675 | 0.795049 | -4.06408 | 4.82E-05 | 0.000189 | Down |
| C14orf105 | 4.353508249 | -1.34367717 | 0.397014 | -3.38446 | 0.000713 | 0.00219 | Down |
| C14orf178 | 1.446913252 | -1.003099791 | 0.37082 | -2.70509 | 0.006829 | 0.016315 | Down |
| C14orf180 | 12.73815133 | -2.726809291 | 0.273913 | -9.95504 | 2.40E-23 | 5.70E-22 | Down |
| C14orf39 | 5.68286628 | -1.3558894 | 0.270009 | -5.02165 | 5.12E-07 | 2.75E-06 | Down |
| C15orf27 | 20.33762024 | -1.024901328 | 0.206505 | -4.96308 | 6.94E-07 | 3.65E-06 | Down |
| C15orf43 | 1.275538308 | -2.891104313 | 0.740248 | -3.90559 | 9.40E-05 | 0.000348 | Down |
| C15orf48 | 136.9090983 | 2.169798551 | 0.199065 | 10.89993 | 1.15E-27 | 3.75E-26 | Up |
| C15orf59 | 53.18804978 | -1.448995021 | 0.159284 | -9.09692 | 9.29E-20 | 1.70E-18 | Down |
| C16orf45 | 621.425204 | 1.46274282 | 0.097696 | 14.97246 | 1.11E-50 | 1.40E-48 | Up |
| C16orf71 | 42.37862736 | -1.299981389 | 0.110949 | -11.717 | 1.04E-31 | 4.51E-30 | Down |
| C16orf74 | 48.70995186 | -1.056342133 | 0.114376 | -9.23571 | 2.57E-20 | 4.89E-19 | Down |
| C16orf82 | 1.661625155 | -2.636984522 | 0.689058 | -3.82694 | 0.00013 | 0.000467 | Down |
| C16orf89 | 3998.949613 | -1.181910115 | 0.134164 | -8.80942 | 1.26E-18 | 2.11E-17 | Down |
| C16orf90 | 1.497222719 | -1.312616102 | 0.471033 | -2.78667 | 0.005325 | 0.013109 | Down |
| C16orf96 | 18.38524166 | -1.324065729 | 0.151464 | -8.74181 | 2.29E-18 | 3.75E-17 | Down |
| C17orf104 | 20.40464842 | -1.521735655 | 0.245427 | -6.20036 | 5.63E-10 | 4.35E-09 | Down |
| C17orf107 | 154.6711423 | 1.525310074 | 0.115172 | 13.24382 | 4.90E-40 | 3.44E-38 | Up |
| C17orf47 | 3.92355147 | -1.342918065 | 0.366121 | -3.66796 | 0.000244 | 0.000834 | Down |
| C17orf50 | 4.384302974 | -1.090399878 | 0.231081 | -4.71869 | 2.37E-06 | 1.16E-05 | Down |
| C17orf61-PLSCR3 | 2.802721738 | -1.119894391 | 0.307063 | -3.64712 | 0.000265 | 0.000899 | Down |
| C17orf74 | 2.134484863 | -1.695037224 | 0.656625 | -2.58144 | 0.009839 | 0.022442 | Down |
| C17orf82 | 10.76962711 | -1.195465208 | 0.216575 | -5.51988 | 3.39E-08 | 2.13E-07 | Down |
| C17orf96 | 58.94095277 | 1.017434411 | 0.194553 | 5.229608 | 1.70E-07 | 9.74E-07 | Up |
| C17orf99 | 4.322607178 | -1.321517145 | 0.563298 | -2.34603 | 0.018974 | 0.039497 | Down |
| C19orf26 | 28.61886169 | 1.1162354 | 0.176909 | 6.309666 | 2.80E-10 | 2.23E-09 | Up |
| C19orf33 | 238.5670264 | 1.957972469 | 0.15931 | 12.29035 | 1.02E-34 | 5.14E-33 | Up |
| C19orf45 | 2.487410011 | -1.996056687 | 0.605933 | -3.29419 | 0.000987 | 0.002936 | Down |
| C1orf106 | 119.9079163 | 1.599015015 | 0.19919 | 8.0276 | 9.94E-16 | 1.32E-14 | Up |
| C1orf110 | 7.36843838 | -1.77859064 | 0.294333 | -6.04278 | 1.51E-09 | 1.12E-08 | Down |
| C1orf115 | 2399.179238 | 1.242302202 | 0.127729 | 9.726092 | 2.33E-22 | 5.17E-21 | Up |
| C1orf116 | 1056.001152 | 1.496264095 | 0.130993 | 11.42247 | 3.23E-30 | 1.25E-28 | Up |
| C1orf143 | 0.86599561 | -1.412803386 | 0.587887 | -2.40319 | 0.016253 | 0.034582 | Down |
| C1orf158 | 10.69749669 | -2.879212499 | 0.374109 | -7.69618 | 1.40E-14 | 1.70E-13 | Down |
| C1orf168 | 39.99562154 | -1.328722881 | 0.188882 | -7.03468 | 2.00E-12 | 1.96E-11 | Down |
| C1orf177 | 2.245751457 | -1.319283363 | 0.340089 | -3.87922 | 0.000105 | 0.000384 | Down |
| C1orf194 | 5.796046374 | -2.228418613 | 0.217474 | -10.2468 | 1.22E-24 | 3.18E-23 | Down |
| C1orf226 | 106.8533314 | 1.559569002 | 0.128581 | 12.12906 | 7.41E-34 | 3.56E-32 | Up |
| C1orf64 | 30.16008232 | -2.809429314 | 0.261269 | -10.753 | 5.74E-27 | 1.77E-25 | Down |
| C1orf87 | 1.685528495 | -2.502941541 | 0.737034 | -3.39597 | 0.000684 | 0.00211 | Down |
| C1orf94 | 1.595543353 | -2.217075706 | 0.73967 | -2.99738 | 0.002723 | 0.007225 | Down |
| C1orf95 | 50.39664145 | -1.103405712 | 0.168564 | -6.54592 | 5.91E-11 | 5.03E-10 | Down |
| C1QL1 | 39.10639848 | 2.749520079 | 0.318821 | 8.624012 | 6.46E-18 | 1.02E-16 | Up |
| C1QL2 | 16.92239511 | 1.060920812 | 0.380942 | 2.78499 | 0.005353 | 0.01317 | Up |
| C1QL4 | 27.0409446 | 1.006592927 | 0.217855 | 4.620465 | 3.83E-06 | 1.81E-05 | Up |
| C1QTNF4 | 20.18681329 | -1.354938416 | 0.201103 | -6.73754 | 1.61E-11 | 1.44E-10 | Down |
| C1QTNF7 | 224.56638 | -1.109835497 | 0.146515 | -7.57489 | 3.59E-14 | 4.18E-13 | Down |
| C1QTNF9 | 5.437638122 | -1.35470468 | 0.24608 | -5.50513 | 3.69E-08 | 2.30E-07 | Down |
| C20orf166-AS1 | 6.721554052 | -1.310998112 | 0.413701 | -3.16895 | 0.00153 | 0.004348 | Down |
| C20orf173 | 1.454130229 | -1.72569225 | 0.578452 | -2.98329 | 0.002852 | 0.007534 | Down |
| C22orf15 | 15.41764598 | -1.277119438 | 0.208263 | -6.13223 | 8.67E-10 | 6.55E-09 | Down |
| C2CD4A | 120.9555008 | 3.632491612 | 0.296717 | 12.24227 | 1.85E-34 | 9.14E-33 | Up |
| C2CD4B | 58.89596903 | 2.134172579 | 0.20294 | 10.5163 | 7.27E-26 | 2.07E-24 | Up |
| C2orf27AP3 | 1.783512723 | -1.862526373 | 0.469908 | -3.9636 | 7.38E-05 | 0.000279 | Down |
| C2orf50 | 8.301615035 | -1.269337045 | 0.211084 | -6.01341 | 1.82E-09 | 1.33E-08 | Down |
| C2orf54 | 7.824564272 | 1.079620494 | 0.338099 | 3.193208 | 0.001407 | 0.004033 | Up |
| C2orf57 | 1.664229495 | -1.877879611 | 0.587126 | -3.19843 | 0.001382 | 0.003969 | Down |
| C2orf70 | 26.26508921 | -1.594604871 | 0.148702 | -10.7235 | 7.90E-27 | 2.40E-25 | Down |
| C2orf72 | 7.817473043 | -1.506324576 | 0.337189 | -4.4673 | 7.92E-06 | 3.56E-05 | Down |
| C2orf73 | 15.17378506 | -1.674491359 | 0.168724 | -9.92442 | 3.26E-23 | 7.67E-22 | Down |
| C2orf78 | 2.940844358 | -1.56163924 | 0.664007 | -2.35184 | 0.018681 | 0.038971 | Down |
| C2orf83 | 2.270185109 | -2.777227545 | 1.013066 | -2.74141 | 0.006118 | 0.014796 | Down |
| C3AR1 | 196.4533166 | 1.007616793 | 0.1649 | 6.110467 | 9.93E-10 | 7.46E-09 | Up |
| C3orf30 | 2.48970325 | -2.182692578 | 0.74653 | -2.92378 | 0.003458 | 0.008946 | Down |
| C3orf56 | 1.66655221 | -2.493678923 | 0.778057 | -3.20501 | 0.001351 | 0.003887 | Down |
| C3orf80 | 25.41816424 | 1.357307669 | 0.213453 | 6.358816 | 2.03E-10 | 1.64E-09 | Up |
| C4A | 11762.98314 | 1.319776675 | 0.102002 | 12.93879 | 2.72E-38 | 1.70E-36 | Up |
| C4B | 12114.86721 | 1.341977354 | 0.112043 | 11.97733 | 4.67E-33 | 2.16E-31 | Up |
| C4BPA | 3.278382758 | -2.086080742 | 0.37607 | -5.54705 | 2.91E-08 | 1.84E-07 | Down |
| C4BPAP1 | 0.88401624 | -2.072868211 | 0.597658 | -3.46832 | 0.000524 | 0.001658 | Down |
| C4BPB | 4.022460126 | -1.294841445 | 0.37793 | -3.42615 | 0.000612 | 0.001909 | Down |
| C4orf19 | 41.91843088 | -1.066298995 | 0.119705 | -8.9077 | 5.21E-19 | 8.99E-18 | Down |
| C4orf22 | 8.681738268 | -2.16664183 | 0.200378 | -10.8128 | 2.99E-27 | 9.47E-26 | Down |
| C4orf47 | 33.1500177 | -1.137171802 | 0.11262 | -10.0975 | 5.67E-24 | 1.41E-22 | Down |
| C4orf48 | 90.65168085 | 1.761825989 | 0.125335 | 14.05693 | 6.99E-45 | 6.68E-43 | Up |
| C5AR2 | 86.75533289 | 1.238286111 | 0.153543 | 8.06475 | 7.34E-16 | 9.82E-15 | Up |
| C5orf46 | 7.064921368 | 2.067867061 | 0.381786 | 5.416303 | 6.08E-08 | 3.68E-07 | Up |
| C5orf47 | 2.209321862 | -1.042810797 | 0.391659 | -2.66255 | 0.007755 | 0.018227 | Down |
| C5orf49 | 22.87882406 | -1.430872913 | 0.156442 | -9.14635 | 5.89E-20 | 1.09E-18 | Down |
| C5orf60 | 3.107630423 | -1.349013133 | 0.400491 | -3.3684 | 0.000756 | 0.002306 | Down |
| C5orf64 | 10.28718474 | -2.74739386 | 0.273739 | -10.0365 | 1.05E-23 | 2.58E-22 | Down |
| C5orf66-AS2 | 1.466511206 | -2.117437871 | 0.726264 | -2.91552 | 0.003551 | 0.00916 | Down |
| C6 | 72.69371832 | -1.571035133 | 0.211181 | -7.43928 | 1.01E-13 | 1.13E-12 | Down |
| C6orf118 | 5.309770018 | -2.953296119 | 0.32358 | -9.12693 | 7.05E-20 | 1.30E-18 | Down |
| C6orf141 | 36.85071518 | -2.225381837 | 0.190582 | -11.6768 | 1.68E-31 | 7.14E-30 | Down |
| C6orf15 | 0.695296245 | 1.128104676 | 0.490245 | 2.301103 | 0.021386 | 0.04381 | Up |
| C6orf165 | 63.42785502 | -1.122994298 | 0.086135 | -13.0377 | 7.47E-39 | 4.84E-37 | Down |
| C6orf183 | 10.09815873 | -1.848787573 | 0.196443 | -9.41129 | 4.90E-21 | 9.85E-20 | Down |
| C6orf195 | 9.152460102 | -1.395180921 | 0.243593 | -5.72752 | 1.02E-08 | 6.82E-08 | Down |
| C6orf222 | 3.078835731 | -1.402796083 | 0.596436 | -2.35196 | 0.018675 | 0.038964 | Down |
| C6orf229 | 0.829576096 | -1.748145924 | 0.681003 | -2.56702 | 0.010258 | 0.023251 | Down |
| C6orf52 | 5.661047729 | -1.19707044 | 0.177343 | -6.75003 | 1.48E-11 | 1.33E-10 | Down |
| C7orf34 | 1.494595615 | -1.698669207 | 0.480248 | -3.53707 | 0.000405 | 0.001315 | Down |
| C7orf62 | 73.19461768 | -2.704052503 | 0.29147 | -9.2773 | 1.74E-20 | 3.35E-19 | Down |
| C7orf71 | 5.93008944 | -1.273171489 | 0.326108 | -3.90414 | 9.46E-05 | 0.000349 | Down |
| C7orf72 | 1.55090067 | -2.176321582 | 0.645809 | -3.36992 | 0.000752 | 0.002296 | Down |
| C8B | 2.170571862 | 1.996667205 | 0.537034 | 3.717953 | 0.000201 | 0.000698 | Up |
| C8orf37-AS1 | 2.720864783 | -1.247244914 | 0.312066 | -3.99674 | 6.42E-05 | 0.000245 | Down |
| C8orf4 | 786.2623687 | 1.971596351 | 0.138486 | 14.2368 | 5.42E-46 | 5.50E-44 | Up |
| C8orf86 | 1.95303938 | -1.763319742 | 0.661577 | -2.66533 | 0.007691 | 0.0181 | Down |
| C8orf88 | 132.4458444 | -1.88044854 | 0.13264 | -14.1771 | 1.27E-45 | 1.27E-43 | Down |
| C9orf117 | 30.67417199 | -1.061375528 | 0.135905 | -7.80967 | 5.73E-15 | 7.20E-14 | Down |
| C9orf152 | 9.048367161 | -1.935482888 | 0.326567 | -5.92675 | 3.09E-09 | 2.19E-08 | Down |
| C9orf171 | 4.345173319 | -1.809498615 | 0.281565 | -6.42657 | 1.31E-10 | 1.08E-09 | Down |
| C9orf24 | 7.285588349 | -2.168868307 | 0.242975 | -8.9263 | 4.40E-19 | 7.66E-18 | Down |
| C9orf43 | 14.65705549 | -1.111071956 | 0.112754 | -9.85395 | 6.59E-23 | 1.52E-21 | Down |
| C9orf47 | 30.87610367 | -1.028260988 | 0.221527 | -4.6417 | 3.46E-06 | 1.64E-05 | Down |
| C9orf57 | 1.862336819 | -2.277012674 | 0.742635 | -3.06613 | 0.002169 | 0.005923 | Down |
| C9orf66 | 45.2552076 | -1.191055202 | 0.107614 | -11.0679 | 1.80E-28 | 6.20E-27 | Down |
| C9orf92 | 2.154989094 | -2.795404054 | 0.310408 | -9.00557 | 2.15E-19 | 3.82E-18 | Down |
| CA10 | 5.903161739 | -1.311261377 | 0.382899 | -3.42457 | 0.000616 | 0.001919 | Down |
| CA11 | 462.3524548 | 1.036634571 | 0.09214 | 11.25068 | 2.30E-29 | 8.45E-28 | Up |
| CA12 | 557.3164233 | 1.858813971 | 0.215886 | 8.610179 | 7.29E-18 | 1.15E-16 | Up |
| CA2 | 113.8010283 | 1.025309152 | 0.120346 | 8.519673 | 1.60E-17 | 2.46E-16 | Up |
| CA4 | 224.2254209 | -2.526878488 | 0.223997 | -11.2809 | 1.63E-29 | 6.06E-28 | Down |
| CA5A | 2.100302141 | -1.367429671 | 0.472417 | -2.89454 | 0.003797 | 0.009719 | Down |
| CA7 | 11.34709528 | 2.048386151 | 0.241541 | 8.480486 | 2.24E-17 | 3.40E-16 | Up |
| CA9 | 1.418619198 | -1.300657638 | 0.38613 | -3.36845 | 0.000756 | 0.002306 | Down |
| CABP5 | 1.789241083 | -2.81690274 | 1.052076 | -2.67747 | 0.007418 | 0.017526 | Down |
| CABS1 | 1.525536443 | -2.996938851 | 1.10201 | -2.71952 | 0.006538 | 0.015702 | Down |
| CACNA1B | 22.0913515 | -1.198776796 | 0.274982 | -4.35947 | 1.30E-05 | 5.65E-05 | Down |
| CACNA1S | 3.13642005 | -2.501608154 | 0.468026 | -5.34502 | 9.04E-08 | 5.36E-07 | Down |
| CACNA2D3 | 55.82761899 | -1.028503451 | 0.125067 | -8.22364 | 1.97E-16 | 2.76E-15 | Down |
| CACNA2D3-AS1 | 2.51939298 | -2.146050487 | 0.381338 | -5.62769 | 1.83E-08 | 1.18E-07 | Down |
| CACNB4 | 142.3834187 | -1.37711016 | 0.120924 | -11.3882 | 4.79E-30 | 1.83E-28 | Down |
| CACNG4 | 54.72175728 | 3.202317615 | 0.236485 | 13.54131 | 8.92E-42 | 6.97E-40 | Up |
| CACNG5 | 1.451745356 | -1.230038044 | 0.512345 | -2.4008 | 0.016359 | 0.034791 | Down |
| CACYBPP3 | 1.783444077 | -1.069595955 | 0.426143 | -2.50994 | 0.012075 | 0.026847 | Down |
| CADM2 | 61.94035535 | -1.860449241 | 0.264964 | -7.02152 | 2.19E-12 | 2.15E-11 | Down |
| CADM2-AS1 | 2.397078007 | -2.350698483 | 0.581776 | -4.04056 | 5.33E-05 | 0.000207 | Down |
| CAGE1 | 3.818618776 | -1.308376915 | 0.353412 | -3.70213 | 0.000214 | 0.000739 | Down |
| CALB1 | 25.19603842 | -1.055061009 | 0.233389 | -4.5206 | 6.17E-06 | 2.82E-05 | Down |
| CALB2 | 13.17161672 | 1.925058345 | 0.303742 | 6.337799 | 2.33E-10 | 1.87E-09 | Up |
| CALCA | 26.98801608 | 1.405496242 | 0.474172 | 2.964106 | 0.003036 | 0.007971 | Up |
| CALHM3 | 1.317384732 | -1.534014534 | 0.64801 | -2.36727 | 0.01792 | 0.03763 | Down |
| CALML3-AS1 | 5.040388627 | -1.14011362 | 0.364827 | -3.12508 | 0.001778 | 0.004973 | Down |
| CALML6 | 4.984280849 | -1.117258022 | 0.282409 | -3.95618 | 7.62E-05 | 0.000286 | Down |
| CALN1 | 7.665970803 | -1.364120717 | 0.33731 | -4.04411 | 5.25E-05 | 0.000204 | Down |
| CAMK2N1 | 731.72122 | 3.506884485 | 0.149988 | 23.38113 | 6.65E-121 | 1.30E-117 | Up |
| CAP1P2 | 3.233363721 | -1.156126636 | 0.341321 | -3.38721 | 0.000706 | 0.002171 | Down |
| CAPN14 | 6.602889415 | -1.285084806 | 0.288998 | -4.4467 | 8.72E-06 | 3.89E-05 | Down |
| CAPN6 | 138.5125875 | -1.517683682 | 0.189778 | -7.99714 | 1.27E-15 | 1.68E-14 | Down |
| CAPSL | 42.87563762 | -2.034543147 | 0.192462 | -10.5712 | 4.05E-26 | 1.17E-24 | Down |
| CARNS1 | 464.0387956 | 1.129402354 | 0.140084 | 8.062336 | 7.48E-16 | 1.00E-14 | Up |
| CARTPT | 41.21934854 | -2.301229684 | 0.342442 | -6.72006 | 1.82E-11 | 1.62E-10 | Down |
| CASC18 | 2.196413097 | -1.318257847 | 0.461411 | -2.85701 | 0.004276 | 0.010794 | Down |
| CASC2 | 191.7943445 | -1.002468679 | 0.097452 | -10.2868 | 8.08E-25 | 2.13E-23 | Down |
| CASP12 | 12.80934676 | -1.026780167 | 0.15417 | -6.66006 | 2.74E-11 | 2.40E-10 | Down |
| CASP16 | 17.39955216 | -1.027277515 | 0.200281 | -5.12919 | 2.91E-07 | 1.61E-06 | Down |
| CASQ1 | 7.736846408 | -1.161892792 | 0.274932 | -4.22611 | 2.38E-05 | 9.85E-05 | Down |
| CASR | 6.823845582 | -1.140333063 | 0.427368 | -2.66827 | 0.007624 | 0.017958 | Down |
| CASZ1 | 311.0916834 | -1.041461967 | 0.109492 | -9.51174 | 1.88E-21 | 3.90E-20 | Down |
| CATIP-AS1 | 9.214052858 | -1.259832156 | 0.140767 | -8.94976 | 3.56E-19 | 6.23E-18 | Down |
| CATIP-AS2 | 2.024061663 | -1.340356529 | 0.281377 | -4.76356 | 1.90E-06 | 9.41E-06 | Down |
| CATSPER1 | 20.54909946 | 2.777991874 | 0.271903 | 10.21685 | 1.67E-24 | 4.29E-23 | Up |
| CATSPERB | 23.8814619 | -1.915905466 | 0.199365 | -9.61006 | 7.25E-22 | 1.55E-20 | Down |
| CBLN1 | 202.4125466 | 2.959862542 | 0.231089 | 12.80834 | 1.47E-37 | 8.87E-36 | Up |
| CBLN2 | 4.428695054 | -1.321447491 | 0.416338 | -3.17398 | 0.001504 | 0.00428 | Down |
| CBLN4 | 41.60612591 | 1.245094063 | 0.206924 | 6.017156 | 1.78E-09 | 1.30E-08 | Up |
| CBX3P1 | 1.567865317 | -2.555917403 | 1.074475 | -2.37876 | 0.017371 | 0.036622 | Down |
| CBX3P9 | 2.619879679 | -1.118977044 | 0.41407 | -2.70239 | 0.006884 | 0.016428 | Down |
| CC2D2B | 14.35452102 | 1.468602662 | 0.175725 | 8.357406 | 6.41E-17 | 9.35E-16 | Up |
| CCBE1 | 37.83906946 | -2.395116434 | 0.221584 | -10.8091 | 3.12E-27 | 9.84E-26 | Down |
| CCDC105 | 1.659385936 | -3.434721932 | 1.057309 | -3.24855 | 0.00116 | 0.003395 | Down |
| CCDC108 | 30.31285162 | -1.103948741 | 0.185054 | -5.96554 | 2.44E-09 | 1.75E-08 | Down |
| CCDC110 | 99.6295076 | -1.230549998 | 0.107344 | -11.4636 | 2.01E-30 | 7.90E-29 | Down |
| CCDC116 | 6.884588234 | -1.143505816 | 0.239882 | -4.76694 | 1.87E-06 | 9.27E-06 | Down |
| CCDC13-AS1 | 28.87478897 | -1.466627108 | 0.142079 | -10.3226 | 5.57E-25 | 1.49E-23 | Down |
| CCDC136 | 47.97691464 | -1.016066689 | 0.133997 | -7.58278 | 3.38E-14 | 3.94E-13 | Down |
| CCDC141 | 208.5717878 | -1.089583616 | 0.179916 | -6.05608 | 1.39E-09 | 1.03E-08 | Down |
| CCDC146 | 469.939678 | -1.60271371 | 0.100306 | -15.9783 | 1.81E-57 | 3.23E-55 | Down |
| CCDC148 | 343.9232239 | 1.026142663 | 0.135037 | 7.598998 | 2.98E-14 | 3.49E-13 | Up |
| CCDC160 | 5.766971223 | -1.190805674 | 0.263817 | -4.51375 | 6.37E-06 | 2.90E-05 | Down |
| CCDC162P | 10.61238215 | -1.049631366 | 0.236788 | -4.43279 | 9.30E-06 | 4.13E-05 | Down |
| CCDC166 | 1.721704969 | -2.871950244 | 0.855771 | -3.35598 | 0.000791 | 0.002401 | Down |
| CCDC169 | 26.5679629 | -1.086542303 | 0.194445 | -5.58793 | 2.30E-08 | 1.47E-07 | Down |
| CCDC173 | 26.57091943 | -1.009202249 | 0.133571 | -7.55558 | 4.17E-14 | 4.81E-13 | Down |
| CCDC175 | 3.734911585 | -1.909446895 | 0.314965 | -6.06241 | 1.34E-09 | 9.94E-09 | Down |
| CCDC178 | 28.24581165 | -1.029564456 | 0.174649 | -5.89506 | 3.75E-09 | 2.63E-08 | Down |
| CCDC36 | 50.40079246 | -1.310433734 | 0.126816 | -10.3333 | 4.98E-25 | 1.34E-23 | Down |
| CCDC38 | 6.790048812 | -1.593344758 | 0.300013 | -5.31092 | 1.09E-07 | 6.40E-07 | Down |
| CCDC39 | 58.23668909 | -1.284757993 | 0.143742 | -8.93793 | 3.97E-19 | 6.92E-18 | Down |
| CCDC42B | 8.874215631 | -1.04184092 | 0.188693 | -5.52135 | 3.36E-08 | 2.11E-07 | Down |
| CCDC54 | 4.116641413 | -1.127381729 | 0.306055 | -3.68359 | 0.00023 | 0.00079 | Down |
| CCDC63 | 1.11072744 | -2.52927738 | 1.101424 | -2.29637 | 0.021655 | 0.044253 | Down |
| CCDC64B | 215.418157 | 1.116595821 | 0.110434 | 10.111 | 4.94E-24 | 1.24E-22 | Up |
| CCDC67 | 20.68488229 | -1.076892376 | 0.14502 | -7.42583 | 1.12E-13 | 1.24E-12 | Down |
| CCDC70 | 1.565275562 | -2.417416034 | 0.864106 | -2.79759 | 0.005149 | 0.012721 | Down |
| CCDC73 | 9.429751383 | -1.284582351 | 0.322112 | -3.988 | 6.66E-05 | 0.000253 | Down |
| CCDC81 | 28.64629539 | -1.010120046 | 0.138302 | -7.30375 | 2.80E-13 | 2.98E-12 | Down |
| CCDC83 | 1.020860916 | -1.585877369 | 0.559559 | -2.83416 | 0.004595 | 0.011506 | Down |
| CCDC85A | 534.8198251 | -1.255906299 | 0.095681 | -13.126 | 2.34E-39 | 1.58E-37 | Down |
| CCER1 | 4.364059989 | -2.416477994 | 0.717084 | -3.36987 | 0.000752 | 0.002296 | Down |
| CCK | 2.436281715 | -1.931478524 | 0.44605 | -4.33018 | 1.49E-05 | 6.39E-05 | Down |
| CCL1 | 0.932677625 | 1.613296654 | 0.616666 | 2.616159 | 0.008893 | 0.020569 | Up |
| CCL11 | 2.89379401 | 2.558516965 | 0.488262 | 5.240049 | 1.61E-07 | 9.23E-07 | Up |
| CCL13 | 92.15284736 | 3.170109399 | 0.203362 | 15.58853 | 8.71E-55 | 1.36E-52 | Up |
| CCL14 | 398.04611 | -1.393043959 | 0.178394 | -7.80881 | 5.77E-15 | 7.25E-14 | Down |
| CCL15 | 2.210065327 | -1.628869066 | 0.636389 | -2.55955 | 0.010481 | 0.023694 | Down |
| CCL16 | 3.583593612 | -1.740063086 | 0.334531 | -5.2015 | 1.98E-07 | 1.12E-06 | Down |
| CCL17 | 66.67740357 | 3.343032066 | 0.290731 | 11.49872 | 1.34E-30 | 5.34E-29 | Up |
| CCL18 | 315.9664428 | 1.04876146 | 0.305681 | 3.430906 | 0.000602 | 0.001879 | Up |
| CCL20 | 15.04042592 | 2.9315394 | 0.263019 | 11.14571 | 7.51E-29 | 2.67E-27 | Up |
| CCL21 | 3209.242396 | -1.946653076 | 0.258093 | -7.54245 | 4.61E-14 | 5.30E-13 | Down |
| CCL22 | 106.1540474 | 1.341642727 | 0.267456 | 5.016317 | 5.27E-07 | 2.82E-06 | Up |
| CCL26 | 6.32726873 | 1.816470719 | 0.236106 | 7.693456 | 1.43E-14 | 1.73E-13 | Up |
| CCL7 | 3.892899671 | 2.881894152 | 0.455872 | 6.321718 | 2.59E-10 | 2.07E-09 | Up |
| CCNA1 | 20.18773489 | 2.958544325 | 0.21169 | 13.97585 | 2.19E-44 | 2.01E-42 | Up |
| CCND1 | 7426.341929 | 1.642228628 | 0.091392 | 17.96902 | 3.41E-72 | 1.08E-69 | Up |
| CCND2 | 12675.5623 | 1.437064226 | 0.08229 | 17.46345 | 2.72E-68 | 7.35E-66 | Up |
| CCNYL2 | 1.966480596 | -1.871135949 | 0.544606 | -3.43576 | 0.000591 | 0.001849 | Down |
| CCR12P | 1.978531095 | -1.999230308 | 0.709413 | -2.81815 | 0.00483 | 0.012023 | Down |
| CCR8 | 11.22824326 | 1.714228612 | 0.270336 | 6.341095 | 2.28E-10 | 1.83E-09 | Up |
| CCSER1 | 295.2265444 | -1.268917926 | 0.101217 | -12.5366 | 4.71E-36 | 2.61E-34 | Down |
| CCT4P1 | 1.448492697 | -2.641445937 | 0.731001 | -3.61346 | 0.000302 | 0.001011 | Down |
| CCT5P1 | 3.662284729 | -1.154002299 | 0.355769 | -3.24368 | 0.00118 | 0.003449 | Down |
| CCT7P2 | 1.88527043 | -2.821375178 | 0.386696 | -7.29611 | 2.96E-13 | 3.14E-12 | Down |
| CCT8L2 | 2.353445796 | -1.66083193 | 0.53158 | -3.12433 | 0.001782 | 0.004984 | Down |
| CD109 | 1031.913258 | 1.74645795 | 0.137996 | 12.6559 | 1.04E-36 | 5.98E-35 | Up |
| CD164L2 | 34.80019955 | 3.430305904 | 0.232065 | 14.78166 | 1.92E-49 | 2.30E-47 | Up |
| CD177 | 50.98954477 | 3.235659048 | 0.351626 | 9.201988 | 3.51E-20 | 6.64E-19 | Up |
| CD19 | 136.0767311 | -1.175483033 | 0.427387 | -2.75039 | 0.005952 | 0.014452 | Down |
| CD1A | 133.6813383 | 3.959664198 | 0.295079 | 13.41901 | 4.68E-41 | 3.54E-39 | Up |
| CD1B | 19.53468851 | 1.593700773 | 0.311296 | 5.119559 | 3.06E-07 | 1.69E-06 | Up |
| CD1E | 106.1656295 | 1.214504151 | 0.24859 | 4.885579 | 1.03E-06 | 5.30E-06 | Up |
| CD207 | 142.7342897 | 2.973804715 | 0.270566 | 10.99103 | 4.22E-28 | 1.42E-26 | Up |
| CD24P4 | 1.299479453 | -1.029924142 | 0.266124 | -3.8701 | 0.000109 | 0.000397 | Down |
| CD276 | 1099.646694 | 1.103654916 | 0.066818 | 16.51725 | 2.76E-61 | 5.77E-59 | Up |
| CD300LF | 64.53535819 | 1.157066975 | 0.239341 | 4.834397 | 1.34E-06 | 6.76E-06 | Up |
| CD300LG | 43.94155981 | -2.312732838 | 0.236194 | -9.79165 | 1.22E-22 | 2.77E-21 | Down |
| CD36 | 1479.878281 | -1.827279819 | 0.156926 | -11.6442 | 2.46E-31 | 1.03E-29 | Down |
| CD44 | 8782.249687 | 1.192597822 | 0.082882 | 14.38908 | 6.06E-47 | 6.55E-45 | Up |
| CD47 | 5160.240015 | 1.019457103 | 0.067612 | 15.07809 | 2.26E-51 | 2.94E-49 | Up |
| CD55 | 3013.719062 | 2.231839066 | 0.139673 | 15.97908 | 1.79E-57 | 3.20E-55 | Up |
| CD5L | 4.748345817 | -1.061549089 | 0.465185 | -2.28199 | 0.02249 | 0.045732 | Down |
| CD70 | 14.86832463 | 1.340389846 | 0.295518 | 4.535725 | 5.74E-06 | 2.64E-05 | Up |
| CDA | 16.32450682 | 1.102518728 | 0.164943 | 6.684257 | 2.32E-11 | 2.04E-10 | Up |
| CDC14C | 3.454834746 | -1.507904519 | 0.39106 | -3.85594 | 0.000115 | 0.000419 | Down |
| CDC20P1 | 5.855055351 | -1.49307865 | 0.22899 | -6.52029 | 7.02E-11 | 5.92E-10 | Down |
| CDC27P1 | 1.250780187 | -1.969204279 | 0.606404 | -3.24735 | 0.001165 | 0.003408 | Down |
| CDC42BPG | 674.6257113 | 1.295976144 | 0.087437 | 14.82188 | 1.06E-49 | 1.28E-47 | Up |
| CDC42EP1 | 789.682014 | 1.192804857 | 0.076499 | 15.59237 | 8.20E-55 | 1.30E-52 | Up |
| CDC42EP3 | 1421.048571 | 1.721162882 | 0.12176 | 14.13573 | 2.29E-45 | 2.26E-43 | Up |
| CDC42EP5 | 135.7010942 | 1.058693006 | 0.092429 | 11.45412 | 2.24E-30 | 8.76E-29 | Up |
| CDCP1 | 1370.692432 | 1.080670555 | 0.061507 | 17.56995 | 4.19E-69 | 1.18E-66 | Up |
| CDCP2 | 3.62956329 | -2.061267883 | 0.400071 | -5.15226 | 2.57E-07 | 1.44E-06 | Down |
| CDH10 | 2.316337301 | -1.615065267 | 0.462127 | -3.49486 | 0.000474 | 0.001518 | Down |
| CDH11 | 1966.935785 | 2.014752792 | 0.140247 | 14.3657 | 8.49E-47 | 9.08E-45 | Up |
| CDH12 | 4.451114603 | -1.563485234 | 0.68282 | -2.28975 | 0.022036 | 0.044936 | Down |
| CDH16 | 1185.483013 | -2.576367475 | 0.236028 | -10.9155 | 9.72E-28 | 3.18E-26 | Down |
| CDH17 | 11.17462064 | 1.597334146 | 0.333515 | 4.789392 | 1.67E-06 | 8.34E-06 | Up |
| CDH18 | 1.697819721 | -1.805199137 | 0.630967 | -2.86101 | 0.004223 | 0.010677 | Down |
| CDH2 | 635.9063243 | 2.167659504 | 0.257804 | 8.408165 | 4.16E-17 | 6.18E-16 | Up |
| CDH22 | 58.46781837 | 1.597193272 | 0.216858 | 7.365141 | 1.77E-13 | 1.92E-12 | Up |
| CDH3 | 1423.643947 | 3.70553674 | 0.15223 | 24.34176 | 7.09E-131 | 2.42E-127 | Up |
| CDH4 | 167.7756164 | 3.553501158 | 0.226463 | 15.69129 | 1.73E-55 | 2.86E-53 | Up |
| CDH6 | 1438.08365 | 2.558433967 | 0.140825 | 18.16748 | 9.34E-74 | 3.19E-71 | Up |
| CDHR2 | 7.865432691 | -1.412788604 | 0.32522 | -4.3441 | 1.40E-05 | 6.03E-05 | Down |
| CDHR3 | 83.90933728 | -1.8007611 | 0.148632 | -12.1156 | 8.73E-34 | 4.19E-32 | Down |
| CDHR4 | 2.264138972 | -1.449786766 | 0.407543 | -3.55738 | 0.000375 | 0.001228 | Down |
| CDK2AP2P2 | 3.245087379 | -1.347225435 | 0.195723 | -6.88333 | 5.85E-12 | 5.49E-11 | Down |
| CDK5RAP2 | 2682.960121 | 1.455058943 | 0.109078 | 13.33957 | 1.36E-40 | 9.86E-39 | Up |
| CDKL4 | 13.89459813 | -1.0564679 | 0.186695 | -5.65878 | 1.52E-08 | 9.98E-08 | Down |
| CDKN1A | 2105.314453 | 1.995546696 | 0.159344 | 12.52351 | 5.55E-36 | 3.07E-34 | Up |
| CDKN2A | 47.2900064 | 1.47374776 | 0.196897 | 7.484863 | 7.16E-14 | 8.08E-13 | Up |
| CDKN2B | 223.4632649 | 2.629366245 | 0.158977 | 16.53925 | 1.91E-61 | 4.07E-59 | Up |
| CDKN2B-AS1 | 12.44751653 | 1.492136016 | 0.187626 | 7.952735 | 1.82E-15 | 2.37E-14 | Up |
| CDON | 2315.729669 | -1.661825805 | 0.11843 | -14.0322 | 9.91E-45 | 9.35E-43 | Down |
| CDRT1 | 15.17810221 | -1.247948663 | 0.209367 | -5.96057 | 2.51E-09 | 1.80E-08 | Down |
| CDSN | 21.00204974 | 4.622104255 | 0.470434 | 9.825199 | 8.77E-23 | 2.00E-21 | Up |
| CDT1 | 102.4797049 | 1.195678471 | 0.136532 | 8.757468 | 2.00E-18 | 3.28E-17 | Up |
| CEACAM3 | 8.514337374 | -1.258313961 | 0.280283 | -4.48944 | 7.14E-06 | 3.23E-05 | Down |
| CEACAM6 | 105.2538152 | 3.18848141 | 0.350783 | 9.089605 | 9.94E-20 | 1.82E-18 | Up |
| CEACAM7 | 1.393530137 | -1.377421679 | 0.586144 | -2.34997 | 0.018775 | 0.039132 | Down |
| CEACAMP1 | 1.091000156 | -1.882718881 | 0.822293 | -2.2896 | 0.022045 | 0.044949 | Down |
| CEACAMP3 | 1.674654138 | -2.103870432 | 0.683047 | -3.08012 | 0.002069 | 0.00568 | Down |
| CEBPA-AS1 | 97.92276937 | -1.424277066 | 0.116119 | -12.2656 | 1.39E-34 | 6.92E-33 | Down |
| CECR2 | 155.0026418 | -1.720763343 | 0.167623 | -10.2657 | 1.01E-24 | 2.64E-23 | Down |
| CECR3 | 2.91530562 | -1.605880623 | 0.542125 | -2.9622 | 0.003055 | 0.008015 | Down |
| CELF2-AS2 | 3.419305159 | -1.14999435 | 0.306854 | -3.74769 | 0.000178 | 0.000627 | Down |
| CELF4 | 51.29787287 | 2.062628867 | 0.245017 | 8.418314 | 3.82E-17 | 5.69E-16 | Up |
| CELP | 3.245951567 | -1.239004765 | 0.402042 | -3.08178 | 0.002058 | 0.005654 | Down |
| CENPJ | 640.1920059 | -1.482280217 | 0.120295 | -12.3221 | 6.89E-35 | 3.53E-33 | Down |
| CENPUP2 | 10.43187283 | -1.092734465 | 0.308192 | -3.54563 | 0.000392 | 0.001279 | Down |
| CEP164P1 | 16.22100206 | -1.789407753 | 0.289543 | -6.18012 | 6.41E-10 | 4.91E-09 | Down |
| CEP170B | 1704.427023 | 1.113055501 | 0.080212 | 13.87648 | 8.80E-44 | 7.83E-42 | Up |
| CERS3 | 8.988572851 | -1.309340686 | 0.330026 | -3.96738 | 7.27E-05 | 0.000274 | Down |
| CES5A | 1.832525903 | -1.611986884 | 0.691696 | -2.33048 | 0.019781 | 0.040957 | Down |
| CETN1 | 1.765656709 | -1.901294288 | 0.831382 | -2.28691 | 0.022201 | 0.045225 | Down |
| CETP | 87.42777578 | -1.382052431 | 0.189156 | -7.30643 | 2.74E-13 | 2.92E-12 | Down |
| CFAP126 | 14.90874688 | -1.928196579 | 0.243275 | -7.926 | 2.26E-15 | 2.93E-14 | Down |
| CFAP221 | 79.66249527 | -1.414792045 | 0.113965 | -12.4143 | 2.19E-35 | 1.16E-33 | Down |
| CFAP43 | 208.0458518 | -1.508536601 | 0.110282 | -13.679 | 1.36E-42 | 1.12E-40 | Down |
| CFAP45 | 12.93728442 | -1.462277396 | 0.182133 | -8.02861 | 9.86E-16 | 1.31E-14 | Down |
| CFAP46 | 126.7420057 | -1.127420194 | 0.100333 | -11.2368 | 2.69E-29 | 9.84E-28 | Down |
| CFAP52 | 8.48746579 | -1.110605129 | 0.219789 | -5.05304 | 4.35E-07 | 2.36E-06 | Down |
| CFAP54 | 222.4444493 | -1.466289537 | 0.133982 | -10.9439 | 7.11E-28 | 2.35E-26 | Down |
| CFAP57 | 68.6466688 | -1.021946246 | 0.129689 | -7.87997 | 3.27E-15 | 4.19E-14 | Down |
| CFAP61 | 19.09689328 | -1.089941284 | 0.144225 | -7.55721 | 4.12E-14 | 4.75E-13 | Down |
| CFAP99 | 5.605833695 | -1.245309192 | 0.213937 | -5.82093 | 5.85E-09 | 4.02E-08 | Down |
| CFB | 1575.969237 | 1.618901743 | 0.178227 | 9.083354 | 1.05E-19 | 1.92E-18 | Up |
| CFD | 1602.752095 | -2.242123616 | 0.178419 | -12.5666 | 3.22E-36 | 1.81E-34 | Down |
| CFI | 1472.476613 | 1.959328795 | 0.088003 | 22.26443 | 8.18E-110 | 9.87E-107 | Up |
| CFL1P7 | 0.835985514 | -1.642841086 | 0.440938 | -3.72579 | 0.000195 | 0.000679 | Down |
| CH17-118O6.4 | 2.127497765 | -1.693920641 | 0.459375 | -3.68745 | 0.000227 | 0.000779 | Down |
| CH17-12M21.1 | 1.665385937 | -2.677967656 | 0.947004 | -2.82783 | 0.004686 | 0.01171 | Down |
| CH17-140K24.3 | 1.542370603 | -1.620877663 | 0.575644 | -2.81576 | 0.004866 | 0.012097 | Down |
| CH17-302M23.1 | 14.49952526 | -1.011320344 | 0.181283 | -5.57867 | 2.42E-08 | 1.55E-07 | Down |
| CH17-351M24.1 | 1.543690741 | -2.563286664 | 1.070238 | -2.39506 | 0.016618 | 0.035271 | Down |
| CH17-353B19.1 | 19.09384166 | -1.212279387 | 0.249177 | -4.86514 | 1.14E-06 | 5.85E-06 | Down |
| CH17-360D5.1 | 4.420636042 | -1.546139481 | 0.482993 | -3.20116 | 0.001369 | 0.003936 | Down |
| CH17-373J23.1 | 108.1188598 | -1.693282108 | 0.178795 | -9.47054 | 2.78E-21 | 5.73E-20 | Down |
| CH17-451C3.1 | 1.138221672 | -2.966309358 | 1.146617 | -2.58701 | 0.009681 | 0.022128 | Down |
| CH17-80A12.1 | 8.846744967 | -1.064951164 | 0.28523 | -3.73365 | 0.000189 | 0.00066 | Down |
| CH25H | 98.10431179 | 2.076112457 | 0.233712 | 8.88321 | 6.50E-19 | 1.11E-17 | Up |
| CH507-152C13.5 | 0.661840347 | -1.63804547 | 0.447742 | -3.65846 | 0.000254 | 0.000863 | Down |
| CH507-216K13.2 | 3.041104551 | -1.6975136 | 0.517993 | -3.2771 | 0.001049 | 0.003099 | Down |
| CH507-236L23.1 | 2.022300566 | -1.537570544 | 0.63614 | -2.41703 | 0.015648 | 0.033472 | Down |
| CH507-236L23.3 | 2.744765083 | -1.046986276 | 0.348296 | -3.00602 | 0.002647 | 0.007047 | Down |
| CH507-338C24.3 | 2.744765083 | -1.046986276 | 0.348296 | -3.00602 | 0.002647 | 0.007047 | Down |
| CH507-396I9.3 | 1.048716762 | -1.135811928 | 0.413758 | -2.74511 | 0.006049 | 0.014657 | Down |
| CH507-39O4.2 | 1.294133968 | -1.26431546 | 0.321728 | -3.92976 | 8.50E-05 | 0.000317 | Down |
| CH507-39O4.3 | 2.744765083 | -1.046986276 | 0.348296 | -3.00602 | 0.002647 | 0.007047 | Down |
| CHEK2P2 | 1.084883087 | -1.220478469 | 0.52923 | -2.30614 | 0.021103 | 0.043306 | Down |
| CHGB | 205.2200284 | 2.712278363 | 0.261011 | 10.39145 | 2.71E-25 | 7.43E-24 | Up |
| CHI3L1 | 3797.794218 | 4.536029106 | 0.279135 | 16.25031 | 2.22E-59 | 4.36E-57 | Up |
| CHI3L2 | 552.3962714 | 1.107633023 | 0.128512 | 8.618904 | 6.76E-18 | 1.07E-16 | Up |
| CHIAP2 | 2.090734487 | 1.340546202 | 0.440451 | 3.043574 | 0.002338 | 0.006326 | Up |
| CHIT1 | 200.9870424 | 1.731491836 | 0.287832 | 6.015642 | 1.79E-09 | 1.31E-08 | Up |
| CHL1-AS1 | 0.911081366 | -1.424147419 | 0.607491 | -2.34431 | 0.019062 | 0.039652 | Down |
| CHL1-AS2 | 0.989931794 | -1.349819131 | 0.448746 | -3.00798 | 0.00263 | 0.00701 | Down |
| CHMP1B2P | 161.0321632 | -1.518296818 | 0.210664 | -7.20719 | 5.71E-13 | 5.89E-12 | Down |
| CHODL | 40.06275064 | -1.414574476 | 0.154092 | -9.18009 | 4.31E-20 | 8.07E-19 | Down |
| CHP2 | 2.154526353 | -2.070459678 | 0.565772 | -3.65953 | 0.000253 | 0.00086 | Down |
| CHPT1 | 1673.200653 | 1.296110358 | 0.090201 | 14.36913 | 8.08E-47 | 8.66E-45 | Up |
| CHRDL1 | 1777.934749 | -2.065981613 | 0.20505 | -10.0755 | 7.09E-24 | 1.75E-22 | Down |
| CHRM1 | 5.838386372 | -2.119002063 | 0.392394 | -5.40018 | 6.66E-08 | 4.02E-07 | Down |
| CHRM3-AS2 | 24.61386077 | -1.10360472 | 0.299467 | -3.68523 | 0.000228 | 0.000785 | Down |
| CHRM4 | 4.529501742 | -1.251119522 | 0.307663 | -4.06653 | 4.77E-05 | 0.000187 | Down |
| CHRNA2 | 13.81487737 | -1.492901195 | 0.323295 | -4.61777 | 3.88E-06 | 1.83E-05 | Down |
| CHRNA4 | 76.61912393 | -1.55407742 | 0.239553 | -6.48741 | 8.73E-11 | 7.31E-10 | Down |
| CHRNA7 | 114.1956424 | -1.032677256 | 0.136447 | -7.56836 | 3.78E-14 | 4.38E-13 | Down |
| CHRNB3 | 1.316540422 | -1.796788639 | 0.796915 | -2.25468 | 0.024153 | 0.048652 | Down |
| CHST2 | 1359.683981 | 1.781509399 | 0.100894 | 17.65725 | 8.95E-70 | 2.64E-67 | Up |
| CHST6 | 48.44892755 | 1.474181087 | 0.234675 | 6.281796 | 3.35E-10 | 2.65E-09 | Up |
| CHST8 | 11.43315452 | -1.5325586 | 0.215793 | -7.10199 | 1.23E-12 | 1.24E-11 | Down |
| CHST9 | 8.157473185 | -1.044461851 | 0.241467 | -4.32548 | 1.52E-05 | 6.52E-05 | Down |
| CIB4 | 9.609698809 | -2.457210436 | 0.220828 | -11.1273 | 9.24E-29 | 3.27E-27 | Down |
| CICP13 | 2.113622942 | -1.284160036 | 0.503084 | -2.55258 | 0.010693 | 0.024112 | Down |
| CICP18 | 1.22390558 | -2.43961197 | 1.011777 | -2.41121 | 0.0159 | 0.033942 | Down |
| CICP20 | 3.194762514 | -1.632533971 | 0.439798 | -3.71201 | 0.000206 | 0.000713 | Down |
| CICP22 | 3.227181405 | -1.245339881 | 0.306991 | -4.05661 | 4.98E-05 | 0.000194 | Down |
| CICP24 | 2.615959027 | -2.001221272 | 0.652606 | -3.06651 | 0.002166 | 0.005917 | Down |
| CICP26 | 2.002156711 | -1.684050602 | 0.68336 | -2.46437 | 0.013725 | 0.029968 | Down |
| CICP5 | 2.824986476 | -1.967768984 | 0.342044 | -5.75297 | 8.77E-09 | 5.91E-08 | Down |
| CICP6 | 3.405293612 | -1.738786202 | 0.549403 | -3.16487 | 0.001552 | 0.004405 | Down |
| CICP7 | 1.198340189 | 2.34221176 | 0.82406 | 2.842282 | 0.004479 | 0.011249 | Up |
| CICP9 | 3.433538053 | -1.345937575 | 0.318677 | -4.22351 | 2.41E-05 | 9.95E-05 | Down |
| CIDEA | 13.74756001 | -2.580693225 | 0.364535 | -7.07942 | 1.45E-12 | 1.44E-11 | Down |
| CIDEC | 25.1144734 | -2.541353046 | 0.361593 | -7.02821 | 2.09E-12 | 2.05E-11 | Down |
| CIR1P2 | 3.33746536 | -2.91610695 | 0.471158 | -6.18923 | 6.05E-10 | 4.65E-09 | Down |
| CISTR | 1.476971596 | -2.143311867 | 0.3864 | -5.54688 | 2.91E-08 | 1.84E-07 | Down |
| CITED1 | 1578.909796 | 4.920982583 | 0.228298 | 21.55509 | 4.74E-103 | 4.33E-100 | Up |
| CITED2 | 3641.140152 | -1.581356953 | 0.115961 | -13.637 | 2.41E-42 | 1.95E-40 | Down |
| CITF22-49E9.3 | 11.0628027 | -1.06894599 | 0.183263 | -5.83285 | 5.45E-09 | 3.75E-08 | Down |
| CITF22-62D4.1 | 1.071746288 | -2.07429358 | 0.662947 | -3.1289 | 0.001755 | 0.004915 | Down |
| CITF22-92A6.2 | 37.33926114 | -1.433334677 | 0.17655 | -8.11857 | 4.72E-16 | 6.42E-15 | Down |
| CKAP2P1 | 1.647964506 | -3.178353312 | 1.026981 | -3.09485 | 0.001969 | 0.005442 | Down |
| CKBP1 | 1.181734424 | -2.998390015 | 1.205051 | -2.48819 | 0.01284 | 0.028328 | Down |
| CKLF | 368.2503883 | 1.088686099 | 0.081095 | 13.42477 | 4.33E-41 | 3.28E-39 | Up |
| CKM | 20.33783979 | -2.754090073 | 0.462059 | -5.96047 | 2.52E-09 | 1.80E-08 | Down |
| CLC | 3.512344684 | -1.351708982 | 0.337646 | -4.00333 | 6.25E-05 | 0.000239 | Down |
| CLCF1 | 163.2473036 | 1.006675576 | 0.095613 | 10.52863 | 6.38E-26 | 1.82E-24 | Up |
| CLCN3P1 | 2.439617775 | -1.606574175 | 0.27556 | -5.83022 | 5.54E-09 | 3.81E-08 | Down |
| CLCNKA | 86.62209226 | -2.150765827 | 0.207831 | -10.3486 | 4.25E-25 | 1.14E-23 | Down |
| CLCNKB | 416.3059816 | -2.474577431 | 0.184421 | -13.4181 | 4.74E-41 | 3.57E-39 | Down |
| CLCP1 | 0.797024018 | -2.097748137 | 0.474835 | -4.41784 | 9.97E-06 | 4.40E-05 | Down |
| CLDN1 | 5181.732241 | 3.882745633 | 0.178493 | 21.75296 | 6.48E-105 | 6.18E-102 | Up |
| CLDN10 | 222.298309 | 5.16860252 | 0.242654 | 21.30034 | 1.13E-100 | 9.64E-98 | Up |
| CLDN16 | 1538.688103 | 4.882633664 | 0.260045 | 18.77614 | 1.18E-78 | 4.81E-76 | Up |
| CLDN2 | 153.7499905 | 3.81239291 | 0.212103 | 17.97423 | 3.10E-72 | 9.87E-70 | Up |
| CLDN4 | 6066.319744 | 1.014188004 | 0.104842 | 9.673513 | 3.91E-22 | 8.50E-21 | Up |
| CLDN5 | 571.3461776 | -1.325621515 | 0.122047 | -10.8616 | 1.76E-27 | 5.66E-26 | Down |
| CLDN7 | 2486.780263 | 1.076423788 | 0.106661 | 10.09205 | 5.99E-24 | 1.49E-22 | Up |
| CLDN9 | 96.60670735 | 3.480409308 | 0.238866 | 14.57057 | 4.32E-48 | 4.92E-46 | Up |
| CLEC18C | 9.338672222 | -1.41693655 | 0.37603 | -3.76815 | 0.000164 | 0.000582 | Down |
| CLEC2L | 3.353789027 | -2.05264104 | 0.36802 | -5.57753 | 2.44E-08 | 1.56E-07 | Down |
| CLEC4E | 25.86915806 | -1.001989829 | 0.209918 | -4.77324 | 1.81E-06 | 9.00E-06 | Down |
| CLEC4M | 4.014678078 | -2.196661392 | 0.436141 | -5.03658 | 4.74E-07 | 2.56E-06 | Down |
| CLEC5A | 125.2221767 | 1.789349991 | 0.167692 | 10.67049 | 1.40E-26 | 4.19E-25 | Up |
| CLEC7A | 240.7143289 | 1.298303588 | 0.1916 | 6.776103 | 1.23E-11 | 1.12E-10 | Up |
| CLK3P2 | 2.741623951 | -2.097394601 | 0.414657 | -5.05815 | 4.23E-07 | 2.30E-06 | Down |
| CLMN | 745.7341386 | -1.207166519 | 0.095335 | -12.6623 | 9.57E-37 | 5.53E-35 | Down |
| CLPSL2 | 0.906190551 | 1.665916551 | 0.429883 | 3.875282 | 0.000107 | 0.00039 | Up |
| CLRN1 | 2.377194502 | -2.274521451 | 0.732317 | -3.10592 | 0.001897 | 0.005266 | Down |
| CLRN1-AS1 | 1.838081283 | -1.817840502 | 0.672049 | -2.70492 | 0.006832 | 0.016321 | Down |
| CLRN3 | 2.865711701 | 2.304015654 | 0.330915 | 6.962568 | 3.34E-12 | 3.21E-11 | Up |
| CLSTN2 | 507.8064049 | -1.291345875 | 0.169576 | -7.61513 | 2.63E-14 | 3.10E-13 | Down |
| CLSTN2-AS1 | 2.380297928 | -1.609355765 | 0.451384 | -3.56538 | 0.000363 | 0.001195 | Down |
| CLU | 72344.07393 | 1.684513085 | 0.105065 | 16.03301 | 7.52E-58 | 1.36E-55 | Up |
| CLUL1 | 137.8082891 | -1.777726713 | 0.145785 | -12.1941 | 3.34E-34 | 1.64E-32 | Down |
| CLVS1 | 24.08743553 | -1.44810643 | 0.234014 | -6.18812 | 6.09E-10 | 4.68E-09 | Down |
| CLYBL-AS2 | 2.00754461 | -1.37870499 | 0.433474 | -3.1806 | 0.00147 | 0.004194 | Down |
| CMB9-22P13.2 | 1.002197176 | -1.412175536 | 0.406489 | -3.47408 | 0.000513 | 0.001626 | Down |
| CMB9-52H4.1 | 0.566904531 | -1.199063632 | 0.485125 | -2.47166 | 0.013449 | 0.029478 | Down |
| CMTM2 | 7.828660197 | -1.364587845 | 0.18888 | -7.22465 | 5.02E-13 | 5.21E-12 | Down |
| CMTM5 | 5.280193274 | -1.456074037 | 0.283999 | -5.12704 | 2.94E-07 | 1.63E-06 | Down |
| CMYA5 | 1461.512388 | 1.045622125 | 0.116829 | 8.950041 | 3.55E-19 | 6.21E-18 | Up |
| CNFN | 15.13787638 | 1.777178383 | 0.160989 | 11.03914 | 2.47E-28 | 8.48E-27 | Up |
| CNGA4 | 3.963382715 | -1.074205612 | 0.271934 | -3.95025 | 7.81E-05 | 0.000293 | Down |
| CNGB3 | 4.494403403 | -2.265449723 | 0.376201 | -6.02192 | 1.72E-09 | 1.26E-08 | Down |
| CNN2P6 | 1.102587724 | -1.784089088 | 0.712057 | -2.50554 | 0.012226 | 0.027133 | Down |
| CNN3P1 | 1.275908762 | -2.25203248 | 0.907885 | -2.48053 | 0.013119 | 0.028847 | Down |
| CNNM1 | 14.11927969 | -1.79665146 | 0.276377 | -6.50073 | 7.99E-11 | 6.72E-10 | Down |
| CNOT7P2 | 2.438139319 | 2.435063647 | 0.50829 | 4.790696 | 1.66E-06 | 8.29E-06 | Up |
| CNR2 | 85.11797558 | -1.141179097 | 0.323247 | -3.53037 | 0.000415 | 0.001346 | Down |
| CNTFR | 76.39700376 | -2.442855101 | 0.222126 | -10.9976 | 3.92E-28 | 1.33E-26 | Down |
| CNTFR-AS1 | 0.790843813 | -1.317404571 | 0.586778 | -2.24515 | 0.024759 | 0.049678 | Down |
| CNTN2 | 34.32253735 | -1.480564902 | 0.251308 | -5.89144 | 3.83E-09 | 2.68E-08 | Down |
| CNTN3 | 94.42691637 | -1.782189683 | 0.187961 | -9.48171 | 2.50E-21 | 5.16E-20 | Down |
| CNTN4-AS1 | 4.462092269 | -1.477361731 | 0.280993 | -5.25765 | 1.46E-07 | 8.44E-07 | Down |
| CNTN4-AS2 | 1.33208243 | -1.07611403 | 0.350446 | -3.0707 | 0.002136 | 0.005844 | Down |
| CNTN5 | 175.3193915 | -2.44129821 | 0.213993 | -11.4083 | 3.80E-30 | 1.47E-28 | Down |
| CNTNAP2 | 51.30778886 | -1.565622569 | 0.24015 | -6.51935 | 7.06E-11 | 5.96E-10 | Down |
| CNTNAP3 | 135.4619097 | -1.893332178 | 0.152428 | -12.4211 | 2.01E-35 | 1.07E-33 | Down |
| CNTNAP3B | 150.5886908 | -1.771276147 | 0.176051 | -10.0612 | 8.20E-24 | 2.02E-22 | Down |
| CNTNAP4 | 3.168644243 | -1.115888541 | 0.491233 | -2.27161 | 0.02311 | 0.04679 | Down |
| COL10A1 | 982.1841622 | 3.731297482 | 0.221582 | 16.83933 | 1.26E-63 | 2.83E-61 | Up |
| COL11A1 | 521.4391772 | 4.413276107 | 0.322904 | 13.66744 | 1.59E-42 | 1.30E-40 | Up |
| COL12A1 | 2550.23417 | 1.284595538 | 0.178025 | 7.21582 | 5.36E-13 | 5.55E-12 | Up |
| COL13A1 | 119.2543557 | 2.130406766 | 0.187356 | 11.37091 | 5.84E-30 | 2.22E-28 | Up |
| COL1A1 | 15420.5904 | 2.901546239 | 0.185811 | 15.61557 | 5.70E-55 | 9.18E-53 | Up |
| COL1A2 | 19283.2945 | 1.561871475 | 0.158926 | 9.827679 | 8.56E-23 | 1.95E-21 | Up |
| COL20A1 | 5.555023658 | -1.340547676 | 0.291737 | -4.59505 | 4.33E-06 | 2.03E-05 | Down |
| COL23A1 | 1294.928443 | -1.808035065 | 0.155173 | -11.6518 | 2.25E-31 | 9.50E-30 | Down |
| COL25A1 | 128.5799884 | -1.514732869 | 0.142944 | -10.5967 | 3.09E-26 | 9.00E-25 | Down |
| COL26A1 | 11.88030665 | 1.41006422 | 0.217083 | 6.495505 | 8.28E-11 | 6.94E-10 | Up |
| COL27A1 | 450.1233757 | -1.236868866 | 0.135121 | -9.15377 | 5.50E-20 | 1.02E-18 | Down |
| COL28A1 | 56.78474574 | -1.467481412 | 0.169074 | -8.67954 | 3.97E-18 | 6.38E-17 | Down |
| COL3A1 | 24393.18617 | 2.111368543 | 0.175085 | 12.05912 | 1.74E-33 | 8.19E-32 | Up |
| COL4A2-AS1 | 1.763226876 | -1.045667784 | 0.29244 | -3.57567 | 0.000349 | 0.001153 | Down |
| COL4A6 | 68.02884398 | -1.807619265 | 0.170467 | -10.6039 | 2.86E-26 | 8.36E-25 | Down |
| COL5A1 | 2093.008037 | 1.675868075 | 0.155153 | 10.8014 | 3.39E-27 | 1.06E-25 | Up |
| COL5A2 | 2982.707645 | 1.953817071 | 0.167499 | 11.66467 | 1.93E-31 | 8.21E-30 | Up |
| COL6A3 | 8999.136582 | 1.168879563 | 0.156152 | 7.485516 | 7.13E-14 | 8.04E-13 | Up |
| COL6A5 | 11.84976011 | 1.032819218 | 0.4155 | 2.485729 | 0.012929 | 0.028495 | Up |
| COL7A1 | 181.0150342 | 1.432808065 | 0.217379 | 6.591285 | 4.36E-11 | 3.75E-10 | Up |
| COL8A1 | 7266.542575 | 2.183664489 | 0.093645 | 23.31852 | 2.88E-120 | 5.13E-117 | Up |
| COL8A2 | 2105.028144 | 2.154360613 | 0.098111 | 21.95851 | 7.18E-107 | 7.97E-104 | Up |
| COL9A3 | 683.9103004 | -2.502685959 | 0.23662 | -10.5768 | 3.82E-26 | 1.11E-24 | Down |
| COLEC10 | 23.97628564 | -1.816661562 | 0.200151 | -9.07644 | 1.12E-19 | 2.04E-18 | Down |
| COLEC11 | 98.7090721 | -1.129730845 | 0.180702 | -6.25189 | 4.06E-10 | 3.17E-09 | Down |
| COMP | 542.888423 | 4.344449905 | 0.254328 | 17.0821 | 2.02E-65 | 4.90E-63 | Up |
| CORO2A | 411.846358 | 2.141438772 | 0.091886 | 23.30533 | 3.91E-120 | 6.69E-117 | Up |
| CORO2B | 146.3797584 | -1.494608732 | 0.115645 | -12.9242 | 3.29E-38 | 2.04E-36 | Down |
| COX6A2 | 1.523061633 | -2.636971071 | 0.655567 | -4.02243 | 5.76E-05 | 0.000222 | Down |
| CPA1 | 2.48866028 | -1.996927352 | 0.483642 | -4.12894 | 3.64E-05 | 0.000146 | Down |
| CPA2 | 1.545225361 | -2.052562278 | 0.504676 | -4.06709 | 4.76E-05 | 0.000187 | Down |
| CPA3 | 53.15856785 | 1.118246946 | 0.216357 | 5.16852 | 2.36E-07 | 1.33E-06 | Up |
| CPA5 | 6.60097502 | -1.32511412 | 0.448795 | -2.9526 | 0.003151 | 0.008239 | Down |
| CPB2-AS1 | 9.787447497 | -1.545444714 | 0.236511 | -6.53435 | 6.39E-11 | 5.42E-10 | Down |
| CPEB2-AS1 | 6.632879583 | -1.016121654 | 0.320844 | -3.16703 | 0.00154 | 0.004375 | Down |
| CPHL1P | 1.038745923 | -1.469389359 | 0.541077 | -2.71567 | 0.006614 | 0.015864 | Down |
| CPLX3 | 1.471315697 | -1.266951867 | 0.526884 | -2.40461 | 0.01619 | 0.034466 | Down |
| CPNE4 | 79.69073815 | 3.127638482 | 0.311159 | 10.05157 | 9.04E-24 | 2.22E-22 | Up |
| CPNE6 | 18.3311793 | -1.273947211 | 0.237727 | -5.35887 | 8.37E-08 | 4.99E-07 | Down |
| CPS1-IT1 | 2.503241093 | -1.770855266 | 0.51442 | -3.44243 | 0.000577 | 0.001807 | Down |
| CPXM1 | 690.1360492 | -1.571653393 | 0.190575 | -8.2469 | 1.63E-16 | 2.29E-15 | Down |
| CR2 | 900.7053726 | -1.209447864 | 0.47937 | -2.523 | 0.011636 | 0.025988 | Down |
| CR848007.2 | 3.542328315 | -1.149397239 | 0.350455 | -3.27973 | 0.001039 | 0.003075 | Down |
| CRABP1 | 1192.079188 | -2.650456884 | 0.223065 | -11.882 | 1.47E-32 | 6.64E-31 | Down |
| CRABP2 | 634.6124798 | 1.738394281 | 0.132671 | 13.10307 | 3.16E-39 | 2.11E-37 | Up |
| CRB1 | 30.73100541 | -1.076594107 | 0.179277 | -6.0052 | 1.91E-09 | 1.39E-08 | Down |
| CRHBP | 57.96398745 | -1.079523335 | 0.170656 | -6.32574 | 2.52E-10 | 2.02E-09 | Down |
| CRIP3 | 13.11026433 | -1.105252442 | 0.222545 | -4.96642 | 6.82E-07 | 3.60E-06 | Down |
| CRISP3 | 2.506279964 | -3.253256751 | 0.487839 | -6.66871 | 2.58E-11 | 2.26E-10 | Down |
| CRLF1 | 508.0219465 | 2.069581701 | 0.203984 | 10.14578 | 3.46E-24 | 8.73E-23 | Up |
| CRLF2 | 44.91483654 | 3.737111799 | 0.295152 | 12.66165 | 9.65E-37 | 5.57E-35 | Up |
| CRNN | 2.218004526 | -1.4293057 | 0.441827 | -3.23499 | 0.001216 | 0.003548 | Down |
| CRP | 1.937846072 | -1.270182333 | 0.491081 | -2.5865 | 0.009696 | 0.022156 | Down |
| CRYAA | 1.908353251 | -1.284209899 | 0.508666 | -2.52466 | 0.011581 | 0.025877 | Down |
| CRYAB | 2139.596216 | -1.152169467 | 0.14301 | -8.05656 | 7.85E-16 | 1.05E-14 | Down |
| CRYBA2 | 1.333529102 | -2.641338227 | 1.154834 | -2.2872 | 0.022184 | 0.045197 | Down |
| CRYBB3 | 9.193217104 | -1.332202448 | 0.17042 | -7.81717 | 5.40E-15 | 6.81E-14 | Down |
| CRYM | 8.505473092 | -1.291679191 | 0.264743 | -4.87899 | 1.07E-06 | 5.47E-06 | Down |
| CRYM-AS1 | 16.52674074 | -1.010942599 | 0.126258 | -8.00695 | 1.18E-15 | 1.55E-14 | Down |
| CRYZP2 | 3.310622527 | -1.785582838 | 0.340223 | -5.24827 | 1.54E-07 | 8.86E-07 | Down |
| CSAG1 | 4.374653528 | -1.212797458 | 0.345676 | -3.50848 | 0.000451 | 0.001449 | Down |
| CSF2 | 15.26544538 | 4.983751516 | 0.240999 | 20.67956 | 5.29E-95 | 3.88E-92 | Up |
| CSF3 | 5.589376556 | -1.900020073 | 0.41161 | -4.61607 | 3.91E-06 | 1.85E-05 | Down |
| CSGALNACT1 | 2110.912099 | -1.629382422 | 0.116007 | -14.0456 | 8.20E-45 | 7.77E-43 | Down |
| CSH1 | 1.410368304 | -1.826831506 | 0.644317 | -2.8353 | 0.004578 | 0.011469 | Down |
| CSH2 | 2.360429054 | -1.912433079 | 0.595292 | -3.21259 | 0.001315 | 0.0038 | Down |
| CSMD1 | 72.83335783 | 1.039691556 | 0.282532 | 3.679912 | 0.000233 | 0.0008 | Up |
| CSN3 | 1.42106421 | -2.856020747 | 0.963263 | -2.96494 | 0.003027 | 0.007952 | Down |
| CSNK1A1L | 3.790024891 | -2.144114071 | 0.323729 | -6.62317 | 3.52E-11 | 3.05E-10 | Down |
| CSNK1A1P1 | 7.354976961 | -2.226757946 | 0.33322 | -6.68255 | 2.35E-11 | 2.07E-10 | Down |
| CSNK2B-LY6G5B-562 | 10.35520991 | -1.263127985 | 0.406752 | -3.1054 | 0.0019 | 0.005273 | Down |
| CSRP3 | 3.013347061 | -2.8381955 | 0.668882 | -4.24319 | 2.20E-05 | 9.18E-05 | Down |
| CST1 | 34.60011017 | 4.632181138 | 0.423891 | 10.92777 | 8.49E-28 | 2.79E-26 | Up |
| CST2 | 13.24535929 | 3.973732712 | 0.351449 | 11.30671 | 1.22E-29 | 4.54E-28 | Up |
| CST5 | 4.650651881 | 3.733776218 | 0.363267 | 10.27832 | 8.82E-25 | 2.33E-23 | Up |
| CST6 | 1137.516393 | 3.461229598 | 0.18879 | 18.33377 | 4.45E-75 | 1.62E-72 | Up |
| CT47B1 | 1.276870677 | -2.363711923 | 0.826511 | -2.85987 | 0.004238 | 0.010709 | Down |
| CT55 | 3.121351722 | -1.26809212 | 0.342041 | -3.70743 | 0.000209 | 0.000725 | Down |
| CTA-113A6.1 | 4.619446509 | -1.700261055 | 0.529815 | -3.20916 | 0.001331 | 0.003838 | Down |
| CTA-126B4.7 | 0.94809579 | 1.261116664 | 0.378735 | 3.329814 | 0.000869 | 0.002617 | Up |
| CTA-150C2.13 | 1.217711293 | 1.045484 | 0.379256 | 2.756672 | 0.005839 | 0.014205 | Up |
| CTA-212A2.1 | 27.3140352 | -1.047060836 | 0.137221 | -7.63049 | 2.34E-14 | 2.77E-13 | Down |
| CTA-212A2.4 | 2.109560436 | -2.5839678 | 0.878903 | -2.93999 | 0.003282 | 0.008545 | Down |
| CTA-212D2.2 | 2.487261183 | -1.08261199 | 0.254276 | -4.25763 | 2.07E-05 | 8.66E-05 | Down |
| CTA-215D11.4 | 0.858781533 | -1.134498674 | 0.381854 | -2.97102 | 0.002968 | 0.007811 | Down |
| CTA-221G9.7 | 1.586789566 | -1.695227363 | 0.350727 | -4.83347 | 1.34E-06 | 6.79E-06 | Down |
| CTA-228A9.4 | 49.41500453 | -1.26452188 | 0.224847 | -5.62391 | 1.87E-08 | 1.21E-07 | Down |
| CTA-243E7.4 | 30.50436039 | -1.234855314 | 0.238344 | -5.18097 | 2.21E-07 | 1.25E-06 | Down |
| CTA-268H5.14 | 1.969080921 | -1.371707772 | 0.440528 | -3.11378 | 0.001847 | 0.005143 | Down |
| CTA-342B11.3 | 8.748763416 | -1.583506078 | 0.633158 | -2.50097 | 0.012386 | 0.027445 | Down |
| CTA-363E6.1 | 1.787917941 | -2.369172114 | 0.651208 | -3.63812 | 0.000275 | 0.000928 | Down |
| CTA-363E6.3 | 2.564336447 | -1.443613793 | 0.430118 | -3.35632 | 0.00079 | 0.002398 | Down |
| CTA-363E6.6 | 1.706003662 | -1.739341663 | 0.365055 | -4.76461 | 1.89E-06 | 9.36E-06 | Down |
| CTA-363E6.8 | 2.178495654 | -1.29059652 | 0.561838 | -2.2971 | 0.021613 | 0.044194 | Down |
| CTA-390C10.9 | 1.66493721 | -2.124267349 | 0.660235 | -3.21744 | 0.001293 | 0.003742 | Down |
| CTA-398F10.2 | 4.164483841 | -1.945052237 | 0.240423 | -8.09011 | 5.96E-16 | 8.02E-15 | Down |
| CTA-481E9.2 | 1.625699969 | -2.942835294 | 1.300376 | -2.26307 | 0.023632 | 0.047709 | Down |
| CTA-722E9.1 | 7.009824935 | -1.062375345 | 0.39409 | -2.69577 | 0.007023 | 0.016709 | Down |
| CTA-796E4.5 | 13.60673373 | -1.841630905 | 0.555856 | -3.31315 | 0.000923 | 0.002763 | Down |
| CTA-797E19.2 | 0.508364244 | -1.123301169 | 0.464887 | -2.41629 | 0.01568 | 0.033535 | Down |
| CTA-929C8.9 | 7.675555072 | -3.056914666 | 0.619488 | -4.93458 | 8.03E-07 | 4.19E-06 | Down |
| CTA-941F9.9 | 3.264113782 | -1.216939496 | 0.251448 | -4.83972 | 1.30E-06 | 6.59E-06 | Down |
| CTA-992D9.6 | 2.087684878 | -2.226807056 | 0.865017 | -2.57429 | 0.010044 | 0.022841 | Down |
| CTA-992D9.9 | 2.124004425 | -3.193998018 | 1.187913 | -2.68875 | 0.007172 | 0.01702 | Down |
| CTAGE10P | 2.192973096 | -1.566050918 | 0.559898 | -2.79703 | 0.005158 | 0.012742 | Down |
| CTAGE11P | 3.280910492 | -1.620323202 | 0.44214 | -3.66473 | 0.000248 | 0.000844 | Down |
| CTAGE13P | 2.331586618 | -1.429109681 | 0.496783 | -2.87673 | 0.004018 | 0.010216 | Down |
| CTAGE14P | 4.694691328 | -1.90084763 | 0.443024 | -4.29062 | 1.78E-05 | 7.55E-05 | Down |
| CTAGE16P | 2.252343326 | -2.000091746 | 0.716721 | -2.79062 | 0.005261 | 0.012968 | Down |
| CTAGE3P | 6.937989828 | -1.652119894 | 0.293959 | -5.62024 | 1.91E-08 | 1.23E-07 | Down |
| CTAGE9 | 6.947330724 | -1.431677193 | 0.395064 | -3.62391 | 0.00029 | 0.000975 | Down |
| CTB-102L5.7 | 12.48012859 | -1.122223328 | 0.250318 | -4.4832 | 7.35E-06 | 3.32E-05 | Down |
| CTB-105N12.2 | 1.711622112 | -1.850349142 | 0.498235 | -3.7138 | 0.000204 | 0.000709 | Down |
| CTB-107G13.1 | 6.604217578 | -1.32571946 | 0.239614 | -5.53272 | 3.15E-08 | 1.99E-07 | Down |
| CTB-108O6.2 | 0.725576735 | 1.140150773 | 0.436302 | 2.613213 | 0.00897 | 0.020721 | Up |
| CTB-111H14.1 | 1.267118832 | -2.295623219 | 0.727849 | -3.15398 | 0.001611 | 0.004554 | Down |
| CTB-113P19.4 | 2.508968257 | -2.059897284 | 0.593111 | -3.47304 | 0.000515 | 0.001632 | Down |
| CTB-129P6.7 | 8.648125331 | 1.102657766 | 0.314943 | 3.50113 | 0.000463 | 0.001486 | Up |
| CTB-12O2.1 | 21.21354121 | 2.461342541 | 0.373866 | 6.58349 | 4.60E-11 | 3.94E-10 | Up |
| CTB-131B5.2 | 20.69018715 | -1.028761212 | 0.248807 | -4.13478 | 3.55E-05 | 0.000143 | Down |
| CTB-134H23.2 | 1.491861494 | -1.749578995 | 0.721286 | -2.42564 | 0.015281 | 0.032825 | Down |
| CTB-140J7.2 | 0.679676238 | 1.176051697 | 0.498932 | 2.357138 | 0.018416 | 0.038519 | Up |
| CTB-147C13.1 | 0.930455844 | -1.367485797 | 0.531828 | -2.57129 | 0.010132 | 0.023005 | Down |
| CTB-147N14.6 | 8.089167159 | 1.114761512 | 0.272939 | 4.084286 | 4.42E-05 | 0.000175 | Up |
| CTB-14A14.2 | 1.266399687 | -2.347928201 | 0.494661 | -4.74654 | 2.07E-06 | 1.02E-05 | Down |
| CTB-151G24.2 | 2.149840612 | -1.406212881 | 0.393987 | -3.56919 | 0.000358 | 0.001179 | Down |
| CTB-160O22.1 | 2.108209231 | -2.314404917 | 0.67601 | -3.42363 | 0.000618 | 0.001924 | Down |
| CTB-161M19.2 | 1.080958351 | -1.331406297 | 0.426758 | -3.11981 | 0.00181 | 0.00505 | Down |
| CTB-164N12.1 | 0.953661816 | -1.485470211 | 0.640041 | -2.3209 | 0.020292 | 0.041867 | Down |
| CTB-171A8.1 | 17.04826029 | -2.057023269 | 0.25744 | -7.9903 | 1.35E-15 | 1.77E-14 | Down |
| CTB-178M22.1 | 3.177131869 | -3.268745206 | 0.641363 | -5.09656 | 3.46E-07 | 1.90E-06 | Down |
| CTB-178M22.2 | 1.740324938 | -1.350299801 | 0.280639 | -4.81152 | 1.50E-06 | 7.52E-06 | Down |
| CTB-186G2.1 | 1.276213869 | 1.134384749 | 0.39625 | 2.862802 | 0.004199 | 0.010625 | Up |
| CTB-186H2.3 | 3.485754031 | -3.298567781 | 0.615948 | -5.35527 | 8.54E-08 | 5.08E-07 | Down |
| CTB-189B5.3 | 1.232737844 | -2.47192369 | 0.924167 | -2.67476 | 0.007478 | 0.017647 | Down |
| CTB-191K22.5 | 3.406237318 | 1.335751609 | 0.387521 | 3.44691 | 0.000567 | 0.001781 | Up |
| CTB-26E19.1 | 10.21897171 | -1.82907076 | 0.272245 | -6.71848 | 1.84E-11 | 1.63E-10 | Down |
| CTB-32H22.1 | 0.875837232 | -1.259187762 | 0.49718 | -2.53266 | 0.01132 | 0.025349 | Down |
| CTB-32O4.2 | 3.54348012 | -1.168987264 | 0.208128 | -5.61667 | 1.95E-08 | 1.26E-07 | Down |
| CTB-32O4.3 | 1.181956295 | -2.893613689 | 0.607145 | -4.76593 | 1.88E-06 | 9.31E-06 | Down |
| CTB-33O18.3 | 11.98399631 | -1.11174741 | 0.227351 | -4.89 | 1.01E-06 | 5.18E-06 | Down |
| CTB-36O1.7 | 6.746821955 | -1.108428166 | 0.326655 | -3.39327 | 0.000691 | 0.002128 | Down |
| CTB-47B8.5 | 1.551127879 | -3.114238424 | 0.874952 | -3.55932 | 0.000372 | 0.00122 | Down |
| CTB-49A3.5 | 2.760646053 | -1.603628753 | 0.714378 | -2.24479 | 0.024782 | 0.049722 | Down |
| CTB-50L17.16 | 1.037819467 | -1.18080608 | 0.347769 | -3.39537 | 0.000685 | 0.002113 | Down |
| CTB-51J22.1 | 29.3752957 | -1.188200409 | 0.10815 | -10.9866 | 4.43E-28 | 1.48E-26 | Down |
| CTB-55B8.1 | 1.527334129 | -1.366061195 | 0.386582 | -3.53369 | 0.00041 | 0.00133 | Down |
| CTB-55O6.4 | 1.112932704 | -1.361385047 | 0.604343 | -2.25267 | 0.02428 | 0.048849 | Down |
| CTB-60B18.12 | 1.768145114 | -1.470158338 | 0.512817 | -2.86683 | 0.004146 | 0.0105 | Down |
| CTB-73N10.1 | 2.290266933 | -1.206699625 | 0.315003 | -3.83076 | 0.000128 | 0.000461 | Down |
| CTB-78F1.1 | 2.255237794 | -2.30517453 | 0.410806 | -5.61134 | 2.01E-08 | 1.30E-07 | Down |
| CTB-78F1.2 | 1.094698978 | -2.022618324 | 0.365996 | -5.52633 | 3.27E-08 | 2.05E-07 | Down |
| CTB-88F18.2 | 1.437709399 | -3.287672361 | 0.800243 | -4.10834 | 3.99E-05 | 0.000159 | Down |
| CTBP2P4 | 1.594895768 | -1.373791809 | 0.538333 | -2.55194 | 0.010713 | 0.024145 | Down |
| CTC-203F4.2 | 24.80404563 | -1.173398942 | 0.154097 | -7.61468 | 2.64E-14 | 3.11E-13 | Down |
| CTC-205M6.1 | 17.27317921 | -1.195336866 | 0.120601 | -9.91151 | 3.71E-23 | 8.69E-22 | Down |
| CTC-232P5.3 | 2.351457513 | -1.68351154 | 0.329526 | -5.10889 | 3.24E-07 | 1.79E-06 | Down |
| CTC-232P5.4 | 0.432731919 | -1.205766977 | 0.525413 | -2.29489 | 0.021739 | 0.044397 | Down |
| CTC-239J10.1 | 1.219664121 | -1.263856308 | 0.414569 | -3.04861 | 0.002299 | 0.006232 | Down |
| CTC-244M17.1 | 2.689643766 | -1.072105482 | 0.261412 | -4.10121 | 4.11E-05 | 0.000163 | Down |
| CTC-250I14.1 | 1.158475771 | -1.450197706 | 0.379145 | -3.82491 | 0.000131 | 0.000471 | Down |
| CTC-250I14.6 | 10.08211741 | -1.088567102 | 0.243862 | -4.46386 | 8.05E-06 | 3.62E-05 | Down |
| CTC-255N20.1 | 17.16776818 | 2.575477441 | 0.206354 | 12.48088 | 9.49E-36 | 5.15E-34 | Up |
| CTC-260E6.3 | 37.45504745 | -1.17456548 | 0.193202 | -6.07948 | 1.21E-09 | 8.97E-09 | Down |
| CTC-265F19.1 | 12.11462486 | -1.138267742 | 0.264428 | -4.30464 | 1.67E-05 | 7.11E-05 | Down |
| CTC-265F19.2 | 5.234633072 | -1.172677884 | 0.290088 | -4.04249 | 5.29E-05 | 0.000205 | Down |
| CTC-265F19.3 | 1.282768391 | -1.194475054 | 0.373473 | -3.19829 | 0.001382 | 0.003971 | Down |
| CTC-276P9.4 | 1.664403295 | -2.55709222 | 0.884181 | -2.89205 | 0.003827 | 0.009783 | Down |
| CTC-277H1.7 | 9.230689558 | 1.041224374 | 0.261304 | 3.984728 | 6.76E-05 | 0.000257 | Up |
| CTC-278L1.1 | 1.840091955 | -2.759470085 | 0.854367 | -3.22984 | 0.001239 | 0.003605 | Down |
| CTC-297N7.9 | 5.104488429 | -1.177939658 | 0.198809 | -5.92497 | 3.12E-09 | 2.21E-08 | Down |
| CTC-301O7.4 | 11.30473171 | -1.12467961 | 0.150479 | -7.47401 | 7.78E-14 | 8.75E-13 | Down |
| CTC-312O10.3 | 2.616253814 | -2.370038696 | 0.754935 | -3.1394 | 0.001693 | 0.004764 | Down |
| CTC-321K16.1 | 2.439969349 | 2.37679883 | 0.324189 | 7.331529 | 2.28E-13 | 2.44E-12 | Up |
| CTC-329D1.3 | 0.79300465 | -1.389646908 | 0.557484 | -2.49271 | 0.012677 | 0.028013 | Down |
| CTC-338M12.5 | 15.07507591 | -1.126768871 | 0.132141 | -8.52702 | 1.50E-17 | 2.31E-16 | Down |
| CTC-340A15.2 | 2.747065784 | -1.683167428 | 0.697572 | -2.41289 | 0.015826 | 0.033808 | Down |
| CTC-347C20.1 | 1.716803213 | -1.357537295 | 0.416375 | -3.26037 | 0.001113 | 0.003269 | Down |
| CTC-347C20.2 | 1.507345241 | -1.725950609 | 0.302021 | -5.71467 | 1.10E-08 | 7.33E-08 | Down |
| CTC-360G5.9 | 7.613511449 | -1.218969963 | 0.259214 | -4.70257 | 2.57E-06 | 1.25E-05 | Down |
| CTC-360P9.2 | 0.699816368 | -1.835894083 | 0.511794 | -3.58717 | 0.000334 | 0.001108 | Down |
| CTC-378H22.2 | 10.72507703 | -1.420019695 | 0.354155 | -4.0096 | 6.08E-05 | 0.000233 | Down |
| CTC-400I9.1 | 1.670573281 | -2.742497644 | 0.415623 | -6.59851 | 4.15E-11 | 3.58E-10 | Down |
| CTC-428G20.1 | 2.283729132 | -1.233132308 | 0.289468 | -4.26 | 2.04E-05 | 8.57E-05 | Down |
| CTC-435M10.10 | 2.291359115 | -1.916904969 | 0.476078 | -4.02645 | 5.66E-05 | 0.000218 | Down |
| CTC-439O9.1 | 22.42161917 | -1.342950876 | 0.208858 | -6.42997 | 1.28E-10 | 1.05E-09 | Down |
| CTC-457E21.1 | 8.419655832 | -1.156896383 | 0.343715 | -3.36586 | 0.000763 | 0.002325 | Down |
| CTC-457L16.1 | 81.86042167 | -1.107309872 | 0.237765 | -4.65716 | 3.21E-06 | 1.53E-05 | Down |
| CTC-458G6.2 | 0.661771839 | -1.407347128 | 0.509829 | -2.76043 | 0.005773 | 0.014061 | Down |
| CTC-492K19.4 | 4.362275755 | -1.10049308 | 0.270984 | -4.06109 | 4.88E-05 | 0.000191 | Down |
| CTC-492K19.7 | 2.155094975 | -1.029514709 | 0.325928 | -3.15872 | 0.001585 | 0.004488 | Down |
| CTC-493L21.1 | 1.384755947 | -2.017522198 | 0.665896 | -3.02979 | 0.002447 | 0.006582 | Down |
| CTC-498M16.2 | 3.365506581 | -1.508548348 | 0.560284 | -2.69247 | 0.007092 | 0.016853 | Down |
| CTC-499B15.7 | 8.701389931 | -1.141662504 | 0.289307 | -3.94619 | 7.94E-05 | 0.000298 | Down |
| CTC-499J9.1 | 2.919883577 | -1.026498342 | 0.364671 | -2.81486 | 0.00488 | 0.012124 | Down |
| CTC-510F12.4 | 1.472063998 | -1.965464056 | 0.437181 | -4.49577 | 6.93E-06 | 3.14E-05 | Down |
| CTC-513N18.6 | 1.080821916 | -1.042680381 | 0.395748 | -2.63471 | 0.008421 | 0.019603 | Down |
| CTC-523E23.10 | 0.726255827 | -1.682800492 | 0.659472 | -2.55174 | 0.010719 | 0.024157 | Down |
| CTC-526N19.1 | 53.74182608 | -1.069666309 | 0.118497 | -9.02698 | 1.76E-19 | 3.16E-18 | Down |
| CTC-539A10.7 | 1.523960434 | -3.268884111 | 1.187491 | -2.75276 | 0.005909 | 0.01436 | Down |
| CTC-543D15.1 | 2.183441527 | -1.826002813 | 0.514756 | -3.54731 | 0.000389 | 0.001271 | Down |
| CTC-546K23.1 | 1.35493324 | -1.883167766 | 0.762067 | -2.47113 | 0.013469 | 0.029512 | Down |
| CTC-546K23.2 | 1.393983367 | -2.749712357 | 1.167019 | -2.35618 | 0.018464 | 0.038603 | Down |
| CTC-558O2.1 | 19.07154983 | -1.019080988 | 0.233094 | -4.37197 | 1.23E-05 | 5.36E-05 | Down |
| CTC-563A5.4 | 1.113381992 | -1.51025341 | 0.631321 | -2.39221 | 0.016747 | 0.035502 | Down |
| CTC-575I10.1 | 0.603476293 | -1.23822726 | 0.444566 | -2.78525 | 0.005349 | 0.013161 | Down |
| CTCFL | 4.072653796 | -1.123216166 | 0.364953 | -3.0777 | 0.002086 | 0.005721 | Down |
| CTD-2001C12.1 | 5.114167473 | -1.605544512 | 0.244216 | -6.57429 | 4.89E-11 | 4.18E-10 | Down |
| CTD-2001J20.1 | 17.19696931 | -2.498540008 | 0.218955 | -11.4112 | 3.68E-30 | 1.42E-28 | Down |
| CTD-2003D5.1 | 0.672338506 | 1.193810674 | 0.464411 | 2.570591 | 0.010153 | 0.023043 | Up |
| CTD-2006K23.1 | 5.793443332 | -1.096963025 | 0.361804 | -3.03193 | 0.00243 | 0.006541 | Down |
| CTD-2008L17.1 | 1.674113797 | -2.208904561 | 0.522206 | -4.22995 | 2.34E-05 | 9.70E-05 | Down |
| CTD-2008L17.2 | 28.63447787 | -2.513731848 | 0.211026 | -11.9119 | 1.03E-32 | 4.65E-31 | Down |
| CTD-2008P7.8 | 1.398384471 | -1.442093044 | 0.538242 | -2.67926 | 0.007378 | 0.017444 | Down |
| CTD-2012K14.2 | 1.031494597 | -1.513808974 | 0.371497 | -4.07489 | 4.60E-05 | 0.000181 | Down |
| CTD-2012K14.5 | 25.57203544 | -1.566286206 | 0.223301 | -7.01422 | 2.31E-12 | 2.26E-11 | Down |
| CTD-2012K14.6 | 11.05887246 | -1.17805074 | 0.219966 | -5.35561 | 8.53E-08 | 5.08E-07 | Down |
| CTD-2012K14.7 | 8.506735255 | -1.166690183 | 0.230018 | -5.07217 | 3.93E-07 | 2.14E-06 | Down |
| CTD-2013N17.7 | 2.089617296 | -1.981691603 | 0.421905 | -4.69701 | 2.64E-06 | 1.28E-05 | Down |
| CTD-2014N11.3 | 1.422882851 | -2.498367201 | 0.554904 | -4.50234 | 6.72E-06 | 3.06E-05 | Down |
| CTD-2016O11.1 | 1.123364897 | -1.130725742 | 0.388327 | -2.91179 | 0.003594 | 0.009259 | Down |
| CTD-2017F17.1 | 1.481081669 | -1.231047394 | 0.386242 | -3.18725 | 0.001436 | 0.004108 | Down |
| CTD-2021A8.3 | 1.508551279 | -1.714414192 | 0.549766 | -3.11844 | 0.001818 | 0.005072 | Down |
| CTD-2022H16.2 | 1.548465733 | -2.058189135 | 0.425692 | -4.83492 | 1.33E-06 | 6.74E-06 | Down |
| CTD-2022H16.3 | 2.440393456 | -1.088877283 | 0.28891 | -3.76892 | 0.000164 | 0.00058 | Down |
| CTD-2024F21.1 | 11.34226325 | -1.082701217 | 0.252268 | -4.29186 | 1.77E-05 | 7.51E-05 | Down |
| CTD-2033D15.2 | 15.90065127 | 1.443513731 | 0.244301 | 5.908752 | 3.45E-09 | 2.43E-08 | Up |
| CTD-2034I21.2 | 2.071875201 | -1.660287594 | 0.520451 | -3.19009 | 0.001422 | 0.004072 | Down |
| CTD-2036J7.1 | 2.997590572 | -1.557734766 | 0.312875 | -4.97878 | 6.40E-07 | 3.39E-06 | Down |
| CTD-2044J15.1 | 8.179424808 | -1.248647054 | 0.296233 | -4.21508 | 2.50E-05 | 0.000103 | Down |
| CTD-2050N2.1 | 1.517694105 | -2.121894811 | 0.668405 | -3.17456 | 0.001501 | 0.004272 | Down |
| CTD-2063L20.1 | 2.609611132 | -1.764501836 | 0.353108 | -4.99705 | 5.82E-07 | 3.10E-06 | Down |
| CTD-2078B5.2 | 2.086544069 | -1.638169966 | 0.35127 | -4.66356 | 3.11E-06 | 1.49E-05 | Down |
| CTD-2086O20.1 | 5.920293465 | -1.03871976 | 0.278578 | -3.72865 | 0.000193 | 0.000672 | Down |
| CTD-2089N3.1 | 1.103207523 | -2.687962288 | 0.878556 | -3.05952 | 0.002217 | 0.006039 | Down |
| CTD-2089N3.2 | 0.842523108 | -2.024555842 | 0.721488 | -2.80609 | 0.005015 | 0.012419 | Down |
| CTD-2090I13.1 | 1.107856743 | -2.554523529 | 1.076006 | -2.37408 | 0.017593 | 0.037032 | Down |
| CTD-2119L1.1 | 2.568706188 | -2.96858828 | 0.392343 | -7.5663 | 3.84E-14 | 4.44E-13 | Down |
| CTD-2126E3.4 | 2.266903977 | -1.041782907 | 0.350296 | -2.97401 | 0.002939 | 0.007742 | Down |
| CTD-2126E3.5 | 2.829190475 | -2.101197666 | 0.578849 | -3.62996 | 0.000283 | 0.000955 | Down |
| CTD-2135D7.5 | 5.099184501 | -1.079035951 | 0.209375 | -5.1536 | 2.56E-07 | 1.43E-06 | Down |
| CTD-2135J3.3 | 3.73393553 | -1.109613904 | 0.268633 | -4.1306 | 3.62E-05 | 0.000145 | Down |
| CTD-2140B24.6 | 1.142558838 | -1.342602272 | 0.489644 | -2.74199 | 0.006107 | 0.014776 | Down |
| CTD-2141G3.1 | 1.690861157 | -3.223782944 | 0.989726 | -3.25725 | 0.001125 | 0.003302 | Down |
| CTD-2147F2.2 | 1.224627367 | -1.93571267 | 0.584118 | -3.31391 | 0.00092 | 0.002757 | Down |
| CTD-2154I11.2 | 7.943803035 | 1.308980068 | 0.152772 | 8.568188 | 1.05E-17 | 1.64E-16 | Up |
| CTD-2156G10.1 | 0.854650432 | -1.643597026 | 0.467544 | -3.51538 | 0.000439 | 0.001416 | Down |
| CTD-2158P22.1 | 1.242085319 | -1.048231837 | 0.402038 | -2.6073 | 0.009126 | 0.021032 | Down |
| CTD-2160D9.1 | 1.556481859 | -3.380067702 | 1.376473 | -2.4556 | 0.014065 | 0.030582 | Down |
| CTD-2162K18.3 | 4.22541782 | -1.429247042 | 0.38115 | -3.74983 | 0.000177 | 0.000622 | Down |
| CTD-2170G1.2 | 2.856118589 | -3.137619332 | 0.672681 | -4.66435 | 3.10E-06 | 1.49E-05 | Down |
| CTD-2173L22.4 | 188.306303 | -1.152283238 | 0.088087 | -13.0812 | 4.22E-39 | 2.78E-37 | Down |
| CTD-2175A23.1 | 0.463163457 | 1.774778398 | 0.525358 | 3.378227 | 0.00073 | 0.002234 | Up |
| CTD-2183H9.3 | 1.655456038 | -1.864511047 | 0.456712 | -4.08246 | 4.46E-05 | 0.000176 | Down |
| CTD-2184C24.2 | 2.5471077 | -1.479328791 | 0.40502 | -3.65249 | 0.00026 | 0.000882 | Down |
| CTD-2192J16.11 | 1.122918033 | -1.135647318 | 0.356252 | -3.18776 | 0.001434 | 0.004101 | Down |
| CTD-2192J16.21 | 3.469345908 | -1.262295365 | 0.290337 | -4.34769 | 1.38E-05 | 5.94E-05 | Down |
| CTD-2193P3.1 | 1.330893139 | -1.050543372 | 0.361324 | -2.90748 | 0.003644 | 0.00937 | Down |
| CTD-2193P3.2 | 5.56731501 | -1.231706418 | 0.341803 | -3.60356 | 0.000314 | 0.001047 | Down |
| CTD-2194A8.2 | 6.313954292 | -2.583999671 | 0.288692 | -8.95071 | 3.53E-19 | 6.18E-18 | Down |
| CTD-2196P11.2 | 0.488601974 | -1.668954128 | 0.533577 | -3.12786 | 0.001761 | 0.004931 | Down |
| CTD-2197I11.1 | 0.880985526 | -1.177146668 | 0.450271 | -2.61431 | 0.008941 | 0.020664 | Down |
| CTD-2199O4.1 | 1.271851861 | -1.558633409 | 0.550998 | -2.82875 | 0.004673 | 0.011685 | Down |
| CTD-2215E18.1 | 12.12909329 | -1.422089115 | 0.306727 | -4.63634 | 3.55E-06 | 1.68E-05 | Down |
| CTD-2218G20.2 | 1.618441374 | -3.024681363 | 0.986489 | -3.06611 | 0.002169 | 0.005923 | Down |
| CTD-2231H16.1 | 27.35096593 | -1.813186213 | 0.15395 | -11.7778 | 5.08E-32 | 2.23E-30 | Down |
| CTD-2236F14.1 | 4.705219271 | -1.004580993 | 0.267875 | -3.75018 | 0.000177 | 0.000622 | Down |
| CTD-2240J17.4 | 1.743748847 | -1.181059455 | 0.332942 | -3.54734 | 0.000389 | 0.001271 | Down |
| CTD-2249K22.1 | 4.210904568 | -1.263726254 | 0.192036 | -6.58066 | 4.68E-11 | 4.02E-10 | Down |
| CTD-2256P15.1 | 1.808382961 | -1.195239768 | 0.364774 | -3.27666 | 0.00105 | 0.003102 | Down |
| CTD-2258A20.3 | 0.628141961 | 1.145850493 | 0.49419 | 2.318643 | 0.020414 | 0.042083 | Up |
| CTD-2265D6.2 | 10.36212246 | -1.752468158 | 0.234198 | -7.48285 | 7.27E-14 | 8.20E-13 | Down |
| CTD-2265M8.2 | 0.770092464 | -1.139851591 | 0.446276 | -2.55414 | 0.010645 | 0.024019 | Down |
| CTD-2266L18.1 | 0.461094917 | -1.572939732 | 0.629199 | -2.49991 | 0.012423 | 0.027508 | Down |
| CTD-2270L9.2 | 3.485947 | -1.010743571 | 0.288035 | -3.5091 | 0.00045 | 0.001446 | Down |
| CTD-2270P14.5 | 67.73359497 | -1.255471343 | 0.128616 | -9.7614 | 1.65E-22 | 3.69E-21 | Down |
| CTD-2272G21.2 | 4.614390748 | -1.007617522 | 0.433484 | -2.32446 | 0.020101 | 0.041528 | Down |
| CTD-2278I10.1 | 3.36700792 | -1.58530771 | 0.556461 | -2.84891 | 0.004387 | 0.011045 | Down |
| CTD-2281E23.1 | 8.815123226 | -1.181479101 | 0.322155 | -3.66742 | 0.000245 | 0.000836 | Down |
| CTD-2281M20.1 | 3.839396169 | -1.197168809 | 0.402429 | -2.97486 | 0.002931 | 0.007724 | Down |
| CTD-2284J15.1 | 9.869518655 | -1.000986949 | 0.159666 | -6.26926 | 3.63E-10 | 2.86E-09 | Down |
| CTD-2287O16.4 | 1.81684537 | -1.307680196 | 0.414984 | -3.15116 | 0.001626 | 0.004594 | Down |
| CTD-2290P7.1 | 1.329595571 | -1.277860961 | 0.568101 | -2.24935 | 0.02449 | 0.049216 | Down |
| CTD-2291D10.4 | 12.56373864 | 1.182714414 | 0.157967 | 7.487075 | 7.04E-14 | 7.95E-13 | Up |
| CTD-2292M16.7 | 1.230769738 | -3.368562601 | 1.026379 | -3.28199 | 0.001031 | 0.003054 | Down |
| CTD-2292P10.4 | 9.629261056 | -1.167481793 | 0.264741 | -4.40991 | 1.03E-05 | 4.56E-05 | Down |
| CTD-2293H3.3 | 1.62685911 | -1.105678657 | 0.349652 | -3.16223 | 0.001566 | 0.004441 | Down |
| CTD-2299I21.1 | 1.347262302 | -1.619271179 | 0.530029 | -3.05506 | 0.00225 | 0.006116 | Down |
| CTD-2299N12.1 | 2.545988084 | -2.448002715 | 0.627066 | -3.9039 | 9.47E-05 | 0.00035 | Down |
| CTD-2302E22.4 | 4.525091653 | -1.015029161 | 0.198222 | -5.12067 | 3.04E-07 | 1.69E-06 | Down |
| CTD-2308G16.1 | 1.440612198 | -1.362955701 | 0.415564 | -3.27977 | 0.001039 | 0.003075 | Down |
| CTD-2311B13.5 | 2.049816601 | -2.919448754 | 1.057626 | -2.76038 | 0.005773 | 0.014062 | Down |
| CTD-2313F11.1 | 3.354011847 | -1.536063815 | 0.457524 | -3.35734 | 0.000787 | 0.002391 | Down |
| CTD-2314G24.2 | 6.956724689 | -1.63629897 | 0.375932 | -4.35264 | 1.35E-05 | 5.81E-05 | Down |
| CTD-2318B16.2 | 1.509175379 | -2.329465609 | 0.945239 | -2.46442 | 0.013724 | 0.029965 | Down |
| CTD-2320B12.3 | 1.36348124 | -2.936280178 | 1.248728 | -2.35142 | 0.018702 | 0.039005 | Down |
| CTD-2325K12.1 | 2.877187926 | -2.98953609 | 0.535002 | -5.5879 | 2.30E-08 | 1.47E-07 | Down |
| CTD-2330K9.3 | 3.457391073 | -1.162218453 | 0.293635 | -3.95803 | 7.56E-05 | 0.000284 | Down |
| CTD-2340D6.1 | 1.01951176 | -1.363219263 | 0.524142 | -2.60086 | 0.009299 | 0.021368 | Down |
| CTD-2349P21.7 | 1.292549048 | -2.325906132 | 0.718536 | -3.23701 | 0.001208 | 0.003525 | Down |
| CTD-2354A18.1 | 2.447411684 | -1.514731794 | 0.502583 | -3.01389 | 0.002579 | 0.00689 | Down |
| CTD-2369P2.8 | 25.22005003 | 1.479282178 | 0.18986 | 7.791457 | 6.62E-15 | 8.27E-14 | Up |
| CTD-2372A4.1 | 1.947771683 | -1.161064432 | 0.359956 | -3.22558 | 0.001257 | 0.00365 | Down |
| CTD-2376I20.1 | 1.62646821 | -1.042021556 | 0.407822 | -2.55509 | 0.010616 | 0.02396 | Down |
| CTD-2377O17.1 | 21.0274102 | -1.318766851 | 0.137881 | -9.56455 | 1.13E-21 | 2.37E-20 | Down |
| CTD-2378E12.1 | 3.741227755 | -1.148521862 | 0.327474 | -3.50722 | 0.000453 | 0.001455 | Down |
| CTD-2501M5.1 | 0.731370423 | 2.042150253 | 0.542537 | 3.764077 | 0.000167 | 0.000591 | Up |
| CTD-2509G16.2 | 2.230226856 | -2.230635884 | 0.624765 | -3.57036 | 0.000356 | 0.001174 | Down |
| CTD-2509G16.5 | 1.957616343 | -1.475071962 | 0.558658 | -2.64038 | 0.008281 | 0.019313 | Down |
| CTD-2515H24.1 | 2.329243861 | -1.374790815 | 0.25462 | -5.39938 | 6.69E-08 | 4.03E-07 | Down |
| CTD-2515H24.2 | 18.38058346 | -1.297369898 | 0.15538 | -8.34965 | 6.85E-17 | 9.97E-16 | Down |
| CTD-2515H24.4 | 4.970215829 | -1.138067016 | 0.237297 | -4.79596 | 1.62E-06 | 8.08E-06 | Down |
| CTD-2515O10.5 | 2.810718192 | -1.314258769 | 0.366597 | -3.58503 | 0.000337 | 0.001116 | Down |
| CTD-2516K3.3 | 2.855211788 | -2.426069872 | 0.763535 | -3.17742 | 0.001486 | 0.004236 | Down |
| CTD-2521M24.11 | 1.784385252 | -2.093947702 | 0.498836 | -4.19767 | 2.70E-05 | 0.000111 | Down |
| CTD-2521M24.5 | 2.922000604 | -1.150411946 | 0.471639 | -2.43918 | 0.014721 | 0.031812 | Down |
| CTD-2521M24.6 | 6.161561425 | -1.052837877 | 0.208271 | -5.05514 | 4.30E-07 | 2.34E-06 | Down |
| CTD-2525I3.2 | 1.161880063 | -1.475486759 | 0.451376 | -3.26886 | 0.00108 | 0.003181 | Down |
| CTD-2525P14.5 | 9.852415089 | -1.007174472 | 0.226764 | -4.4415 | 8.93E-06 | 3.98E-05 | Down |
| CTD-2526A2.2 | 6.496377485 | -1.383013821 | 0.244617 | -5.65378 | 1.57E-08 | 1.03E-07 | Down |
| CTD-2526A2.4 | 2.016755035 | -1.483430037 | 0.609452 | -2.43404 | 0.014931 | 0.032195 | Down |
| CTD-2527I21.15 | 1.892455933 | -2.898953971 | 0.883655 | -3.28064 | 0.001036 | 0.003067 | Down |
| CTD-2527I21.5 | 3.416316839 | -1.687272858 | 0.3679 | -4.58622 | 4.51E-06 | 2.11E-05 | Down |
| CTD-2529P6.4 | 0.996511237 | -1.842041339 | 0.69299 | -2.65811 | 0.007858 | 0.018429 | Down |
| CTD-2532N20.1 | 5.378619973 | -1.072202464 | 0.195643 | -5.48041 | 4.24E-08 | 2.62E-07 | Down |
| CTD-2534I21.8 | 1.071805312 | -1.625658153 | 0.436218 | -3.72671 | 0.000194 | 0.000677 | Down |
| CTD-2536I1.2 | 34.5386364 | -1.043836677 | 0.140943 | -7.4061 | 1.30E-13 | 1.43E-12 | Down |
| CTD-2536I1.3 | 2.021174274 | -1.170808012 | 0.301963 | -3.87732 | 0.000106 | 0.000387 | Down |
| CTD-2538A21.1 | 0.754453239 | -1.152350883 | 0.478159 | -2.40997 | 0.015954 | 0.034036 | Down |
| CTD-2540B15.9 | 2.574351479 | -1.236348119 | 0.239701 | -5.15788 | 2.50E-07 | 1.40E-06 | Down |
| CTD-2541J13.1 | 9.069492157 | -1.107502808 | 0.213135 | -5.19626 | 2.03E-07 | 1.15E-06 | Down |
| CTD-2542C24.14 | 4.46915201 | -1.894367017 | 0.326936 | -5.79431 | 6.86E-09 | 4.67E-08 | Down |
| CTD-2542C24.2 | 3.45737181 | -2.843709854 | 0.484016 | -5.87524 | 4.22E-09 | 2.94E-08 | Down |
| CTD-2542C24.5 | 13.28698433 | -1.035014993 | 0.241917 | -4.27839 | 1.88E-05 | 7.94E-05 | Down |
| CTD-2544H17.1 | 1.867947313 | -1.663964419 | 0.418138 | -3.97947 | 6.91E-05 | 0.000262 | Down |
| CTD-2544L4.1 | 1.3272688 | -2.421622704 | 0.682999 | -3.54557 | 0.000392 | 0.001279 | Down |
| CTD-2545H1.2 | 0.840923559 | -1.372165842 | 0.587059 | -2.33736 | 0.019421 | 0.040305 | Down |
| CTD-2545M3.8 | 10.12798507 | -1.855357437 | 0.317456 | -5.84446 | 5.08E-09 | 3.51E-08 | Down |
| CTD-2547E10.3 | 1.688191786 | -1.322754325 | 0.249195 | -5.30811 | 1.11E-07 | 6.49E-07 | Down |
| CTD-2547L16.1 | 19.77205543 | -1.026451727 | 0.135449 | -7.57814 | 3.51E-14 | 4.08E-13 | Down |
| CTD-2547L16.3 | 10.5495795 | -1.050757427 | 0.191953 | -5.47403 | 4.40E-08 | 2.72E-07 | Down |
| CTD-2555A7.2 | 1.704028461 | -2.448101826 | 0.691137 | -3.54214 | 0.000397 | 0.001294 | Down |
| CTD-2561B21.5 | 18.76174236 | -1.00989232 | 0.199199 | -5.06976 | 3.98E-07 | 2.17E-06 | Down |
| CTD-2562G15.2 | 2.057780472 | -1.534058721 | 0.444392 | -3.45204 | 0.000556 | 0.00175 | Down |
| CTD-2562J15.6 | 0.737889108 | -1.13220145 | 0.429398 | -2.63672 | 0.008371 | 0.019497 | Down |
| CTD-2571E19.3 | 1.39199458 | -2.787246311 | 0.98955 | -2.81668 | 0.004852 | 0.012069 | Down |
| CTD-2576D5.1 | 6.602468204 | -1.468429444 | 0.461839 | -3.17952 | 0.001475 | 0.004208 | Down |
| CTD-2576F9.1 | 2.240589512 | -1.086900826 | 0.466955 | -2.32764 | 0.019931 | 0.041221 | Down |
| CTD-2576N18.1 | 6.191958486 | -1.476579145 | 0.259396 | -5.69237 | 1.25E-08 | 8.29E-08 | Down |
| CTD-2588J6.1 | 1.508513489 | -1.588960894 | 0.697718 | -2.27737 | 0.022764 | 0.046212 | Down |
| CTD-2589H19.6 | 8.569930511 | -1.290639977 | 0.170979 | -7.54854 | 4.40E-14 | 5.06E-13 | Down |
| CTD-2589M5.5 | 0.643208932 | 1.029945264 | 0.433712 | 2.374719 | 0.017562 | 0.036976 | Up |
| CTD-2595P9.4 | 4.539182503 | -1.197785504 | 0.270492 | -4.42817 | 9.50E-06 | 4.21E-05 | Down |
| CTD-2619J13.1 | 1.159706285 | -1.152548561 | 0.438954 | -2.62567 | 0.008648 | 0.020071 | Down |
| CTD-2619J13.13 | 12.76955338 | 3.250007141 | 0.21751 | 14.94184 | 1.76E-50 | 2.19E-48 | Up |
| CTD-2619J13.19 | 25.58315489 | -1.260347718 | 0.175399 | -7.18559 | 6.69E-13 | 6.86E-12 | Down |
| CTD-2621I17.6 | 1.899092304 | -1.607511881 | 0.601732 | -2.67148 | 0.007552 | 0.017802 | Down |
| CTD-2647E9.1 | 2.942911954 | -1.503604559 | 0.486506 | -3.09062 | 0.001997 | 0.00551 | Down |
| CTD-2649C14.3 | 1.602287494 | -1.147882137 | 0.272161 | -4.21766 | 2.47E-05 | 0.000102 | Down |
| CTD-2650P22.2 | 3.759485196 | -1.575940637 | 0.374164 | -4.2119 | 2.53E-05 | 0.000104 | Down |
| CTD-2651B20.4 | 2.031189584 | -2.72881407 | 0.760372 | -3.58879 | 0.000332 | 0.001101 | Down |
| CTD-2651B20.6 | 62.77565585 | -1.001840446 | 0.219314 | -4.56807 | 4.92E-06 | 2.28E-05 | Down |
| CTD-2655K5.1 | 0.861439283 | -1.737329048 | 0.427681 | -4.06221 | 4.86E-05 | 0.00019 | Down |
| CTD-3010D24.3 | 1.652963275 | -1.114164333 | 0.317499 | -3.50919 | 0.000449 | 0.001446 | Down |
| CTD-3014M21.1 | 377.5302906 | -2.068138276 | 0.184538 | -11.2071 | 3.76E-29 | 1.36E-27 | Down |
| CTD-3022L24.1 | 11.61726624 | -1.19126591 | 0.262473 | -4.53862 | 5.66E-06 | 2.60E-05 | Down |
| CTD-3023L14.2 | 1.192152218 | -2.077163066 | 0.373679 | -5.55869 | 2.72E-08 | 1.72E-07 | Down |
| CTD-3028N15.1 | 1.352641255 | -2.133710968 | 0.590494 | -3.61343 | 0.000302 | 0.001011 | Down |
| CTD-3035K23.7 | 20.6423845 | 1.480437521 | 0.174994 | 8.459927 | 2.68E-17 | 4.03E-16 | Up |
| CTD-3064C13.1 | 1.044823484 | -1.726342897 | 0.484644 | -3.56209 | 0.000368 | 0.001208 | Down |
| CTD-3064M3.4 | 2.740455483 | -1.547906494 | 0.429811 | -3.60137 | 0.000317 | 0.001055 | Down |
| CTD-3075F15.1 | 1.193379762 | -1.307921273 | 0.340312 | -3.8433 | 0.000121 | 0.00044 | Down |
| CTD-3076M17.1 | 1.804204132 | -2.198735095 | 0.943171 | -2.33122 | 0.019742 | 0.040892 | Down |
| CTD-3088G3.6 | 2.405957711 | -1.971133385 | 0.558601 | -3.5287 | 0.000418 | 0.001353 | Down |
| CTD-3094K11.1 | 13.4915508 | -1.048223696 | 0.117017 | -8.95784 | 3.31E-19 | 5.81E-18 | Down |
| CTD-3105H18.11 | 1.932202599 | -1.149197196 | 0.396703 | -2.89687 | 0.003769 | 0.00966 | Down |
| CTD-3105H18.8 | 2.478650065 | -1.468346167 | 0.399169 | -3.67851 | 0.000235 | 0.000804 | Down |
| CTD-3116E22.6 | 7.574348007 | -1.467673674 | 0.181644 | -8.07995 | 6.48E-16 | 8.71E-15 | Down |
| CTD-3118D7.1 | 9.684448917 | -2.070681499 | 0.319919 | -6.47251 | 9.64E-11 | 8.04E-10 | Down |
| CTD-3126B10.5 | 10.56688007 | -1.147486132 | 0.336446 | -3.41061 | 0.000648 | 0.002009 | Down |
| CTD-3148I10.15 | 2.658810678 | -1.122001283 | 0.312552 | -3.58981 | 0.000331 | 0.001098 | Down |
| CTD-3154N5.1 | 2.33301943 | -1.418632356 | 0.304732 | -4.65534 | 3.23E-06 | 1.55E-05 | Down |
| CTD-3162L10.1 | 55.06161743 | -1.256764475 | 0.181749 | -6.91485 | 4.68E-12 | 4.44E-11 | Down |
| CTD-3162L10.3 | 1.037354752 | -1.198622712 | 0.480553 | -2.49426 | 0.012622 | 0.027904 | Down |
| CTD-3179P9.1 | 4.430824149 | -1.71811755 | 0.241202 | -7.12316 | 1.05E-12 | 1.07E-11 | Down |
| CTD-3179P9.2 | 2.801008286 | -2.07323082 | 0.346806 | -5.97807 | 2.26E-09 | 1.63E-08 | Down |
| CTD-3193K9.11 | 2.418090728 | -1.270079168 | 0.258947 | -4.90478 | 9.35E-07 | 4.84E-06 | Down |
| CTD-3193K9.4 | 1.029055323 | 1.272238811 | 0.564038 | 2.25559 | 0.024096 | 0.048544 | Up |
| CTD-3193O13.1 | 2.761057841 | 1.732352788 | 0.309515 | 5.596991 | 2.18E-08 | 1.40E-07 | Up |
| CTD-3214H19.16 | 5.127728542 | 4.870113194 | 1.196005 | 4.071983 | 4.66E-05 | 0.000183 | Up |
| CTD-3229J4.1 | 1.764932562 | -3.683079161 | 1.221712 | -3.01469 | 0.002572 | 0.006875 | Down |
| CTD-3247F14.2 | 17.80937573 | -1.938278916 | 0.355045 | -5.45925 | 4.78E-08 | 2.94E-07 | Down |
| CTD-3253I12.1 | 2.256872718 | -1.079666929 | 0.45745 | -2.36019 | 0.018266 | 0.038249 | Down |
| CTH | 100.9624927 | -1.152849563 | 0.093609 | -12.3156 | 7.47E-35 | 3.81E-33 | Down |
| CTHRC1 | 318.308371 | 2.268081492 | 0.193329 | 11.73171 | 8.77E-32 | 3.80E-30 | Up |
| CTNNA2 | 6.374943568 | -1.84488168 | 0.327396 | -5.63502 | 1.75E-08 | 1.14E-07 | Down |
| CTNND2 | 9.485839985 | -1.198147895 | 0.268434 | -4.46348 | 8.06E-06 | 3.62E-05 | Down |
| CTRB2 | 3.988131926 | -1.001848758 | 0.284765 | -3.51816 | 0.000435 | 0.001403 | Down |
| CTSB | 97639.1992 | 1.441498908 | 0.090909 | 15.85657 | 1.27E-56 | 2.19E-54 | Up |
| CTSC | 5131.406516 | 2.025945635 | 0.133894 | 15.13094 | 1.01E-51 | 1.34E-49 | Up |
| CTSH | 4642.045464 | 2.24027006 | 0.118073 | 18.97361 | 2.82E-80 | 1.22E-77 | Up |
| CTSLP1 | 3.016588551 | -1.150429498 | 0.316986 | -3.62928 | 0.000284 | 0.000957 | Down |
| CTSLP4 | 1.059152237 | -1.294407315 | 0.432967 | -2.98962 | 0.002793 | 0.007397 | Down |
| CTSLP7 | 6.497360681 | -1.163417505 | 0.197416 | -5.89321 | 3.79E-09 | 2.65E-08 | Down |
| CTSS | 3251.739214 | 1.164404391 | 0.174128 | 6.687066 | 2.28E-11 | 2.01E-10 | Up |
| CTXN1 | 58.21158437 | 2.520919659 | 0.169575 | 14.86613 | 5.47E-50 | 6.72E-48 | Up |
| CUX2 | 112.1766577 | -2.598975098 | 0.268525 | -9.67872 | 3.71E-22 | 8.09E-21 | Down |
| CWH43 | 327.3580129 | -2.097722925 | 0.204307 | -10.2675 | 9.87E-25 | 2.59E-23 | Down |
| CXADRP2 | 1.476478926 | -1.211718061 | 0.512864 | -2.36265 | 0.018145 | 0.038027 | Down |
| CXCL1 | 12.57654519 | 1.726325406 | 0.246786 | 6.995227 | 2.65E-12 | 2.56E-11 | Up |
| CXCL10 | 304.6858216 | 1.015945425 | 0.309163 | 3.286119 | 0.001016 | 0.003013 | Up |
| CXCL14 | 2420.299845 | 2.608156837 | 0.18711 | 13.93914 | 3.66E-44 | 3.31E-42 | Up |
| CXCL16 | 556.6042867 | 1.330457497 | 0.092383 | 14.4015 | 5.06E-47 | 5.53E-45 | Up |
| CXCL17 | 71.56008763 | 3.588685633 | 0.231412 | 15.50777 | 3.07E-54 | 4.66E-52 | Up |
| CXCL2 | 126.2084228 | 1.816465949 | 0.194956 | 9.317301 | 1.19E-20 | 2.33E-19 | Up |
| CXCL5 | 14.29889089 | 1.088407121 | 0.416198 | 2.615117 | 0.00892 | 0.020625 | Up |
| CXCL8 | 79.51680217 | 2.213586922 | 0.229413 | 9.648927 | 4.97E-22 | 1.07E-20 | Up |
| CXCR1 | 18.07811707 | -1.694466571 | 0.208501 | -8.1269 | 4.40E-16 | 6.00E-15 | Down |
| CXCR2P1 | 20.72270854 | -1.116245844 | 0.329921 | -3.38337 | 0.000716 | 0.002197 | Down |
| CXorf22 | 83.04216939 | -2.576603856 | 0.19349 | -13.3165 | 1.86E-40 | 1.33E-38 | Down |
| CXorf65 | 5.804249332 | -1.016684095 | 0.390115 | -2.60612 | 0.009158 | 0.021089 | Down |
| CXorf66 | 1.214159343 | -2.880037888 | 1.127201 | -2.55503 | 0.010618 | 0.023962 | Down |
| CXorf67 | 4.627253924 | -1.068455487 | 0.282657 | -3.78005 | 0.000157 | 0.000557 | Down |
| CXXC4 | 262.5267418 | -1.202680684 | 0.121089 | -9.93221 | 3.01E-23 | 7.12E-22 | Down |
| CYCSP25 | 0.766271408 | -1.127081957 | 0.472011 | -2.38783 | 0.016948 | 0.035873 | Down |
| CYLC1 | 1.906290263 | -3.284066072 | 0.979064 | -3.35429 | 0.000796 | 0.002414 | Down |
| CYP17A1 | 64.29755776 | -1.821755362 | 0.1571 | -11.5961 | 4.31E-31 | 1.78E-29 | Down |
| CYP17A1-AS1 | 13.17247649 | -1.679610505 | 0.176662 | -9.50748 | 1.95E-21 | 4.06E-20 | Down |
| CYP1A1 | 4.804989738 | -1.510798441 | 0.290737 | -5.19645 | 2.03E-07 | 1.15E-06 | Down |
| CYP1A2 | 35.15375378 | -1.215431545 | 0.151995 | -7.99654 | 1.28E-15 | 1.68E-14 | Down |
| CYP1B1 | 1784.441782 | 2.416579852 | 0.14521 | 16.64197 | 3.46E-62 | 7.44E-60 | Up |
| CYP21A1P | 15.13070856 | 1.060054197 | 0.192764 | 5.499242 | 3.81E-08 | 2.38E-07 | Up |
| CYP26A1 | 11.16832877 | 2.304509601 | 0.366178 | 6.29341 | 3.11E-10 | 2.47E-09 | Up |
| CYP26B1 | 147.9421468 | -1.493379944 | 0.129985 | -11.4888 | 1.50E-30 | 5.97E-29 | Down |
| CYP2A7 | 6.076629243 | -1.28929863 | 0.40037 | -3.22027 | 0.001281 | 0.00371 | Down |
| CYP2C18 | 1.386918665 | -2.146644039 | 0.659517 | -3.25487 | 0.001134 | 0.003326 | Down |
| CYP2S1 | 106.2237559 | 2.459716096 | 0.151179 | 16.27018 | 1.61E-59 | 3.22E-57 | Up |
| CYP3A4 | 3.908821627 | -1.32090648 | 0.34547 | -3.82351 | 0.000132 | 0.000473 | Down |
| CYP3A43 | 1.562419707 | -1.406759398 | 0.542794 | -2.5917 | 0.00955 | 0.021879 | Down |
| CYP46A1 | 45.8764951 | -1.408491886 | 0.17011 | -8.27987 | 1.23E-16 | 1.76E-15 | Down |
| CYP4A22-AS1 | 1.274796795 | 1.130088057 | 0.38448 | 2.939263 | 0.00329 | 0.008564 | Up |
| CYP4B1 | 522.9024072 | 1.550873286 | 0.179057 | 8.661324 | 4.66E-18 | 7.45E-17 | Up |
| CYP4F12 | 29.47645651 | -1.750312402 | 0.170768 | -10.2496 | 1.19E-24 | 3.09E-23 | Down |
| CYP4F2 | 1.602561157 | -2.377866975 | 0.714095 | -3.3299 | 0.000869 | 0.002616 | Down |
| CYP4F3 | 9.414100753 | -1.493995263 | 0.297116 | -5.02832 | 4.95E-07 | 2.66E-06 | Down |
| CYP4F32P | 2.043731072 | -1.783466409 | 0.300021 | -5.94447 | 2.77E-09 | 1.98E-08 | Down |
| CYP51A1-AS1 | 10.94267696 | -1.106780822 | 0.18622 | -5.9434 | 2.79E-09 | 1.99E-08 | Down |
| CYP51A1P3 | 12.14672246 | -1.110461367 | 0.216731 | -5.12367 | 3.00E-07 | 1.66E-06 | Down |
| CYP7A1 | 3.569030718 | -1.830876758 | 0.414839 | -4.41346 | 1.02E-05 | 4.49E-05 | Down |
| CYP8B1 | 18.00614057 | -1.930190985 | 0.225782 | -8.54891 | 1.24E-17 | 1.92E-16 | Down |
| CYR61 | 1775.716776 | -1.181525623 | 0.189177 | -6.24559 | 4.22E-10 | 3.30E-09 | Down |
| CYSLTR2 | 869.7876769 | 3.177889997 | 0.2058 | 15.44167 | 8.58E-54 | 1.27E-51 | Up |
| CYSRT1 | 45.02024416 | 1.442412715 | 0.133191 | 10.82964 | 2.49E-27 | 7.93E-26 | Up |
| DAB1 | 19.16546718 | -1.498719642 | 0.228354 | -6.56313 | 5.27E-11 | 4.50E-10 | Down |
| DAB1-AS1 | 4.709968708 | -1.039408888 | 0.390429 | -2.66222 | 0.007763 | 0.018239 | Down |
| DACH2 | 14.6277991 | 1.066654483 | 0.28481 | 3.745144 | 0.00018 | 0.000633 | Up |
| DACT2 | 28.70999364 | -1.569909872 | 0.247557 | -6.3416 | 2.27E-10 | 1.83E-09 | Down |
| DAND5 | 4.777781753 | -1.675242015 | 0.268411 | -6.24134 | 4.34E-10 | 3.38E-09 | Down |
| DAPK2 | 5173.263179 | 1.373211193 | 0.137202 | 10.00865 | 1.40E-23 | 3.39E-22 | Up |
| DAW1 | 7.232474876 | 1.53356313 | 0.240379 | 6.379759 | 1.77E-10 | 1.44E-09 | Up |
| DBF4P1 | 2.432789406 | -1.099645166 | 0.376988 | -2.91692 | 0.003535 | 0.009123 | Down |
| DBX2 | 10.09354742 | -1.947361931 | 0.230414 | -8.45156 | 2.87E-17 | 4.32E-16 | Down |
| DCAF12L1 | 27.73383685 | -1.138399212 | 0.214619 | -5.30429 | 1.13E-07 | 6.62E-07 | Down |
| DCAF13P1 | 1.369073266 | -2.188890041 | 0.528843 | -4.13902 | 3.49E-05 | 0.00014 | Down |
| DCAF4L1 | 13.62228496 | -1.166334452 | 0.223871 | -5.20984 | 1.89E-07 | 1.08E-06 | Down |
| DCBLD2 | 2789.370793 | 1.097707797 | 0.071576 | 15.33625 | 4.38E-53 | 6.22E-51 | Up |
| DCDC1 | 112.0262121 | -1.379175391 | 0.130429 | -10.5741 | 3.93E-26 | 1.14E-24 | Down |
| DCDC2C | 4.427712923 | -3.181094583 | 0.289438 | -10.9906 | 4.24E-28 | 1.42E-26 | Down |
| DCHS2 | 186.7786413 | 2.131717077 | 0.219555 | 9.709271 | 2.75E-22 | 6.06E-21 | Up |
| DCSTAMP | 1447.735944 | 4.866795439 | 0.317434 | 15.33166 | 4.70E-53 | 6.63E-51 | Up |
| DCT | 25.79828642 | -1.045321544 | 0.165306 | -6.32355 | 2.56E-10 | 2.04E-09 | Down |
| DCTN1-AS1 | 6.046848157 | -1.525623141 | 0.273892 | -5.57016 | 2.55E-08 | 1.62E-07 | Down |
| DCUN1D3 | 673.4747298 | 1.00229179 | 0.064224 | 15.60613 | 6.61E-55 | 1.06E-52 | Up |
| DDB2 | 456.4854365 | 1.161038663 | 0.073691 | 15.75557 | 6.29E-56 | 1.07E-53 | Up |
| DDC-AS1 | 1.179075086 | -2.761852228 | 1.082912 | -2.55039 | 0.01076 | 0.024238 | Down |
| DDN | 3.991675557 | -1.363669832 | 0.332312 | -4.10358 | 4.07E-05 | 0.000162 | Down |
| DDX11L1 | 9.03768306 | -1.055416242 | 0.33353 | -3.16438 | 0.001554 | 0.004411 | Down |
| DDX11L10 | 3.151953886 | -1.298149397 | 0.31535 | -4.11653 | 3.85E-05 | 0.000154 | Down |
| DDX11L16 | 4.407138716 | -1.229477418 | 0.268602 | -4.57732 | 4.71E-06 | 2.19E-05 | Down |
| DDX11L9 | 15.90934313 | -1.562877532 | 0.284269 | -5.49788 | 3.84E-08 | 2.39E-07 | Down |
| DDX25 | 84.55043365 | -1.806632264 | 0.157623 | -11.4618 | 2.05E-30 | 8.06E-29 | Down |
| DDX39B-AS1 | 1.975084378 | -1.342750111 | 0.326918 | -4.1073 | 4.00E-05 | 0.000159 | Down |
| DDX3P3 | 4.603649285 | -1.247331428 | 0.462404 | -2.69749 | 0.006986 | 0.016638 | Down |
| DDX43P1 | 2.394077692 | -1.692281678 | 0.568381 | -2.97737 | 0.002907 | 0.007669 | Down |
| DDX50P2 | 2.028453881 | -2.295974966 | 0.481426 | -4.76912 | 1.85E-06 | 9.17E-06 | Down |
| DDX53 | 10.37905553 | -2.444358324 | 0.400098 | -6.1094 | 1.00E-09 | 7.51E-09 | Down |
| DDX6P1 | 1.133943777 | -3.008264985 | 0.973818 | -3.08915 | 0.002007 | 0.005536 | Down |
| DEFA1 | 1.889845305 | -1.53679096 | 0.434753 | -3.53486 | 0.000408 | 0.001325 | Down |
| DEFB124 | 1.205750993 | -1.569059148 | 0.490228 | -3.20067 | 0.001371 | 0.003941 | Down |
| DEFB131 | 1.718246344 | -1.323671417 | 0.318229 | -4.15949 | 3.19E-05 | 0.000129 | Down |
| DEFB132 | 1.39452815 | -2.061298018 | 0.814711 | -2.5301 | 0.011403 | 0.025513 | Down |
| DENND2A | 144.5690463 | -1.167208884 | 0.094711 | -12.3238 | 6.74E-35 | 3.47E-33 | Down |
| DEPDC1B | 123.0449332 | 1.89245566 | 0.243043 | 7.78652 | 6.89E-15 | 8.59E-14 | Up |
| DEPTOR | 2337.011731 | -1.66536104 | 0.124785 | -13.3459 | 1.25E-40 | 9.08E-39 | Down |
| DES | 528.8266091 | -2.081406657 | 0.274599 | -7.57979 | 3.46E-14 | 4.03E-13 | Down |
| DGKI | 2630.917386 | -2.101347043 | 0.152218 | -13.8048 | 2.38E-43 | 2.04E-41 | Down |
| DHDH | 7.676084284 | 2.744168754 | 0.246667 | 11.125 | 9.48E-29 | 3.35E-27 | Up |
| DHRS3 | 4618.604693 | 1.809460868 | 0.102869 | 17.58995 | 2.94E-69 | 8.44E-67 | Up |
| DHRS4L1 | 4.131858254 | -1.088301184 | 0.191901 | -5.67115 | 1.42E-08 | 9.32E-08 | Down |
| DHX9P1 | 2.945450301 | -1.550854802 | 0.446405 | -3.4741 | 0.000513 | 0.001626 | Down |
| DICER1-AS1 | 81.087606 | -1.063537839 | 0.109071 | -9.7509 | 1.83E-22 | 4.08E-21 | Down |
| DIO1 | 1345.986497 | -2.946286202 | 0.269138 | -10.9471 | 6.86E-28 | 2.27E-26 | Down |
| DIO2 | 11833.60439 | -1.210436322 | 0.126299 | -9.58393 | 9.34E-22 | 1.98E-20 | Down |
| DIO3 | 24.10395927 | -1.152883616 | 0.215518 | -5.34936 | 8.83E-08 | 5.24E-07 | Down |
| DIO3OS | 59.0856837 | -1.505797015 | 0.178384 | -8.44131 | 3.14E-17 | 4.70E-16 | Down |
| DIRAS2 | 147.3914599 | -2.037703196 | 0.155041 | -13.143 | 1.87E-39 | 1.27E-37 | Down |
| DIRAS3 | 210.3462098 | 2.236938895 | 0.150057 | 14.90724 | 2.96E-50 | 3.67E-48 | Up |
| DISC1-IT1 | 2.113999597 | -1.293233988 | 0.446409 | -2.89697 | 0.003768 | 0.009658 | Down |
| DISC1FP1 | 1.288087177 | -1.820991833 | 0.588276 | -3.09547 | 0.001965 | 0.005431 | Down |
| DKFZp313A047 | 1.235272929 | -3.344728704 | 1.300181 | -2.57251 | 0.010096 | 0.022936 | Down |
| DKFZp434J0226 | 7.633936619 | 1.134454381 | 0.416136 | 2.72616 | 0.006408 | 0.015428 | Up |
| DKFZP434K028 | 1.712543304 | -1.503663349 | 0.646183 | -2.32699 | 0.019966 | 0.041283 | Down |
| DKFZP434L187 | 26.64512547 | -1.320646438 | 0.258126 | -5.11629 | 3.12E-07 | 1.72E-06 | Down |
| DLG1-AS1 | 9.469438637 | -1.134020464 | 0.139303 | -8.14069 | 3.93E-16 | 5.38E-15 | Down |
| DLG2 | 623.4657419 | -1.956079707 | 0.119374 | -16.3862 | 2.40E-60 | 4.90E-58 | Down |
| DLG2-AS1_1 | 0.878261251 | -1.681111231 | 0.415109 | -4.0498 | 5.13E-05 | 0.0002 | Down |
| DLG2-AS1_2 | 1.370299864 | -1.47039966 | 0.360902 | -4.07424 | 4.62E-05 | 0.000182 | Down |
| DLG4 | 401.1639501 | 1.070405052 | 0.08299 | 12.89794 | 4.62E-38 | 2.86E-36 | Up |
| DLGAP1 | 46.92309084 | -1.001655966 | 0.207489 | -4.82751 | 1.38E-06 | 6.98E-06 | Down |
| DLGAP1-AS5 | 3.239952165 | -1.629834924 | 0.630906 | -2.58332 | 0.009785 | 0.022338 | Down |
| DLGAP2-AS1 | 1.617420004 | -1.790302084 | 0.782648 | -2.28749 | 0.022167 | 0.045168 | Down |
| DLL3 | 7.110872854 | -1.836731933 | 0.276945 | -6.63211 | 3.31E-11 | 2.87E-10 | Down |
| DLX2 | 9.036968355 | -1.572974208 | 0.262563 | -5.99084 | 2.09E-09 | 1.51E-08 | Down |
| DMBX1 | 13.33479823 | 1.940718279 | 0.522422 | 3.714847 | 0.000203 | 0.000706 | Up |
| DMD | 1777.019055 | 1.660999104 | 0.155624 | 10.67319 | 1.36E-26 | 4.08E-25 | Up |
| DMP1 | 16.45297618 | 2.011917248 | 0.432116 | 4.655963 | 3.22E-06 | 1.54E-05 | Up |
| DMRTB1 | 6.672068227 | -2.809543152 | 0.437383 | -6.42353 | 1.33E-10 | 1.10E-09 | Down |
| DMRTC1B | 30.62908567 | -1.088603215 | 0.142107 | -7.66042 | 1.85E-14 | 2.22E-13 | Down |
| DNAAF1 | 90.33965662 | -1.697289621 | 0.1528 | -11.1079 | 1.15E-28 | 4.03E-27 | Down |
| DNAH12 | 25.30930829 | -1.735203923 | 0.267582 | -6.48475 | 8.89E-11 | 7.44E-10 | Down |
| DNAH3 | 8.051820533 | -1.15188768 | 0.280119 | -4.11214 | 3.92E-05 | 0.000156 | Down |
| DNAH8 | 21.67813767 | -1.030804937 | 0.322398 | -3.1973 | 0.001387 | 0.003982 | Down |
| DNAH9 | 78.88212494 | -1.259279009 | 0.189541 | -6.64383 | 3.06E-11 | 2.66E-10 | Down |
| DNAI2 | 3.965149843 | -1.969773827 | 0.331743 | -5.93766 | 2.89E-09 | 2.06E-08 | Down |
| DNAJA1P5 | 1.620720259 | -3.224243158 | 1.09892 | -2.93401 | 0.003346 | 0.008691 | Down |
| DNAJB13 | 3.172507742 | -1.2018041 | 0.320555 | -3.74914 | 0.000177 | 0.000624 | Down |
| DNAJB1P1 | 17.82245507 | -1.212165362 | 0.226395 | -5.3542 | 8.59E-08 | 5.11E-07 | Down |
| DNAJB3 | 1.177590801 | -1.799601812 | 0.695026 | -2.58926 | 0.009618 | 0.022013 | Down |
| DNAJB8-AS1 | 1.378306287 | -2.657856397 | 1.029844 | -2.58083 | 0.009856 | 0.022474 | Down |
| DNASE1L3 | 229.9296498 | -1.590136974 | 0.176707 | -8.99871 | 2.28E-19 | 4.06E-18 | Down |
| DNASE2B | 7.042804891 | 1.6261469 | 0.262672 | 6.190795 | 5.99E-10 | 4.61E-09 | Up |
| DNER | 10.13982416 | -1.554836987 | 0.345722 | -4.49736 | 6.88E-06 | 3.12E-05 | Down |
| DNM1P35 | 15.62244593 | -1.094825976 | 0.181696 | -6.02558 | 1.69E-09 | 1.24E-08 | Down |
| DNM1P51 | 60.31142233 | -1.324904768 | 0.234022 | -5.66145 | 1.50E-08 | 9.83E-08 | Down |
| DNTT | 3.400030558 | -2.618801596 | 0.262745 | -9.96707 | 2.12E-23 | 5.06E-22 | Down |
| DOC2A | 10.49667903 | -1.130672802 | 0.244895 | -4.61697 | 3.89E-06 | 1.84E-05 | Down |
| DOC2GP | 12.37947831 | -1.311443463 | 0.152641 | -8.59167 | 8.57E-18 | 1.34E-16 | Down |
| DOCK11P1 | 2.649551372 | -1.127203623 | 0.333343 | -3.38152 | 0.000721 | 0.00221 | Down |
| DOCK9 | 7746.593345 | 1.599213616 | 0.088917 | 17.98546 | 2.53E-72 | 8.12E-70 | Up |
| DOCK9-AS2 | 106.8696726 | 2.038129151 | 0.126983 | 16.0504 | 5.68E-58 | 1.03E-55 | Up |
| DOK5 | 101.8429084 | -1.0020059 | 0.123582 | -8.10801 | 5.15E-16 | 6.97E-15 | Down |
| DOK7 | 104.6331382 | 1.16727547 | 0.157142 | 7.428173 | 1.10E-13 | 1.22E-12 | Up |
| DPEP3 | 6.165214026 | -1.658129392 | 0.374893 | -4.42294 | 9.74E-06 | 4.31E-05 | Down |
| DPF1 | 5.029915593 | -1.273842355 | 0.355198 | -3.58629 | 0.000335 | 0.001111 | Down |
| DPH6-AS1 | 13.80689766 | -1.149071933 | 0.171366 | -6.70537 | 2.01E-11 | 1.78E-10 | Down |
| DPP10-AS1 | 2.084488097 | 1.50156951 | 0.481272 | 3.120002 | 0.001808 | 0.005048 | Up |
| DPP3P2 | 1.227046832 | -2.793034441 | 0.948975 | -2.94321 | 0.003248 | 0.008463 | Down |
| DPP4 | 2128.063374 | 3.708700973 | 0.164759 | 22.50979 | 3.33E-112 | 4.27E-109 | Up |
| DPP6 | 770.6254134 | -2.120145969 | 0.209793 | -10.1059 | 5.20E-24 | 1.30E-22 | Down |
| DPPA2P2 | 1.342421263 | -2.679260896 | 0.892611 | -3.0016 | 0.002686 | 0.007138 | Down |
| DPPA2P4 | 2.81955644 | -1.54578527 | 0.367945 | -4.20113 | 2.66E-05 | 0.000109 | Down |
| DPPA3P2 | 2.777130154 | -1.433517903 | 0.328605 | -4.36243 | 1.29E-05 | 5.58E-05 | Down |
| DPPA5P4 | 0.887271546 | -2.291197609 | 0.505742 | -4.53037 | 5.89E-06 | 2.70E-05 | Down |
| DPRXP7 | 1.613753748 | 3.136434156 | 0.482874 | 6.495353 | 8.28E-11 | 6.95E-10 | Up |
| DPT | 1435.668715 | -2.138969049 | 0.204033 | -10.4834 | 1.03E-25 | 2.91E-24 | Down |
| DPY19L2 | 344.9822119 | -1.80162845 | 0.145355 | -12.3947 | 2.79E-35 | 1.47E-33 | Down |
| DPY19L2P2 | 118.1364795 | -1.02125827 | 0.088133 | -11.5877 | 4.76E-31 | 1.96E-29 | Down |
| DPY19L2P4 | 8.478787929 | -2.333133894 | 0.224562 | -10.3897 | 2.76E-25 | 7.56E-24 | Down |
| DPYSL3 | 5069.394933 | 1.058804368 | 0.091778 | 11.53656 | 8.63E-31 | 3.48E-29 | Up |
| DRAM1 | 534.033874 | 1.204893982 | 0.097231 | 12.39204 | 2.89E-35 | 1.52E-33 | Up |
| DRAXIN | 66.49593917 | 2.529843605 | 0.245005 | 10.32567 | 5.39E-25 | 1.44E-23 | Up |
| DRC1 | 10.71275661 | -1.668098992 | 0.225109 | -7.41017 | 1.26E-13 | 1.39E-12 | Down |
| DRC7 | 8.564582933 | -1.793646011 | 0.254743 | -7.04101 | 1.91E-12 | 1.88E-11 | Down |
| DRD1 | 9.013040626 | -1.544097 | 0.271583 | -5.68555 | 1.30E-08 | 8.61E-08 | Down |
| DRD4 | 9.851602566 | -1.259929443 | 0.161442 | -7.80423 | 5.99E-15 | 7.50E-14 | Down |
| DRD5P1 | 1.962912712 | -1.788452648 | 0.685593 | -2.60862 | 0.009091 | 0.020963 | Down |
| DSC1 | 5.627911451 | -1.578269855 | 0.472209 | -3.34231 | 0.000831 | 0.002511 | Down |
| DSC2 | 1063.478701 | 1.420095025 | 0.088095 | 16.12007 | 1.84E-58 | 3.44E-56 | Up |
| DSC3 | 205.0667035 | 3.57588073 | 0.278009 | 12.86246 | 7.32E-38 | 4.49E-36 | Up |
| DSCAML1 | 47.97556794 | -1.389920348 | 0.163941 | -8.47816 | 2.29E-17 | 3.47E-16 | Down |
| DSCR9 | 7.887536392 | -1.101593917 | 0.191051 | -5.76598 | 8.12E-09 | 5.49E-08 | Down |
| DSG1 | 2.952330452 | -1.37599482 | 0.427772 | -3.21665 | 0.001297 | 0.003752 | Down |
| DTX4 | 6686.89747 | 2.805823329 | 0.1308 | 21.4512 | 4.45E-102 | 3.97E-99 | Up |
| DUSP1 | 6412.714046 | -1.066419822 | 0.199274 | -5.35152 | 8.72E-08 | 5.18E-07 | Down |
| DUSP13 | 6.211182848 | 2.238625649 | 0.359605 | 6.22523 | 4.81E-10 | 3.74E-09 | Up |
| DUSP15 | 71.23003289 | -1.265733436 | 0.112907 | -11.2104 | 3.63E-29 | 1.32E-27 | Down |
| DUSP26 | 17.27957555 | -1.103668255 | 0.228216 | -4.83608 | 1.32E-06 | 6.71E-06 | Down |
| DUSP4 | 718.4598669 | 2.551449328 | 0.129414 | 19.71548 | 1.59E-86 | 8.93E-84 | Up |
| DUSP5 | 1145.606178 | 2.739604989 | 0.151846 | 18.04199 | 9.12E-73 | 2.97E-70 | Up |
| DUSP5P1 | 1.879368483 | -1.05895672 | 0.366766 | -2.88728 | 0.003886 | 0.009916 | Down |
| DUSP5P2 | 1.647853495 | -2.526567904 | 0.55748 | -4.53212 | 5.84E-06 | 2.68E-05 | Down |
| DUSP6 | 6205.64516 | 2.556397155 | 0.129298 | 19.77143 | 5.25E-87 | 3.03E-84 | Up |
| DUSP8P3 | 12.00969504 | -1.247271498 | 0.234799 | -5.31209 | 1.08E-07 | 6.36E-07 | Down |
| DUSP8P4 | 6.254585878 | -1.822111807 | 0.326143 | -5.58686 | 2.31E-08 | 1.48E-07 | Down |
| DYDC2 | 4.269212505 | -1.065465285 | 0.268952 | -3.96155 | 7.45E-05 | 0.000281 | Down |
| DYNC1I1 | 91.63376469 | -1.228064395 | 0.118904 | -10.3282 | 5.25E-25 | 1.41E-23 | Down |
| DYNLL1P1 | 0.508004222 | -1.189246459 | 0.445317 | -2.67056 | 0.007572 | 0.017845 | Down |
| DYNLRB2 | 22.48696924 | -1.257920363 | 0.107514 | -11.7001 | 1.27E-31 | 5.47E-30 | Down |
| DYNLT3P1 | 0.894534085 | 1.675788499 | 0.609619 | 2.748913 | 0.005979 | 0.014505 | Up |
| E2F1 | 103.9078751 | 1.630676607 | 0.158661 | 10.27777 | 8.88E-25 | 2.34E-23 | Up |
| E2F3P2 | 11.74732678 | -1.568914682 | 0.228163 | -6.8763 | 6.14E-12 | 5.75E-11 | Down |
| E2F4P1 | 1.35442691 | -2.755007995 | 0.666792 | -4.13173 | 3.60E-05 | 0.000144 | Down |
| EBF3 | 243.511611 | -1.247134623 | 0.111509 | -11.1842 | 4.87E-29 | 1.75E-27 | Down |
| EBF4 | 436.9549939 | -1.34072902 | 0.113171 | -11.8469 | 2.23E-32 | 1.00E-30 | Down |
| EBI3 | 60.47309769 | 1.128991815 | 0.213819 | 5.280135 | 1.29E-07 | 7.51E-07 | Up |
| EBLN1 | 1.031523613 | -2.035297151 | 0.824743 | -2.4678 | 0.013595 | 0.029724 | Down |
| ECE1 | 6310.817811 | 1.383172617 | 0.075397 | 18.3451 | 3.61E-75 | 1.32E-72 | Up |
| ECEL1P1 | 0.88666633 | -1.511859134 | 0.500234 | -3.02231 | 0.002509 | 0.006723 | Down |
| ECM1 | 594.4449751 | 2.505733474 | 0.204908 | 12.22857 | 2.19E-34 | 1.08E-32 | Up |
| ECM1P1 | 2.105885055 | -2.456997388 | 0.660297 | -3.72105 | 0.000198 | 0.000691 | Down |
| ECM1P2 | 1.443582828 | -2.413864309 | 0.988773 | -2.44127 | 0.014636 | 0.03165 | Down |
| EDA2R | 277.7331478 | 1.547836794 | 0.102828 | 15.05269 | 3.31E-51 | 4.28E-49 | Up |
| EDIL3 | 556.6931879 | 1.558250175 | 0.149779 | 10.40363 | 2.39E-25 | 6.55E-24 | Up |
| EDN3 | 357.838969 | -1.521341922 | 0.294802 | -5.16056 | 2.46E-07 | 1.38E-06 | Down |
| EEF1A1P14 | 2.98211178 | -1.022391835 | 0.417828 | -2.44692 | 0.014408 | 0.031231 | Down |
| EEF1A1P26 | 1.48053841 | -3.029124979 | 0.942443 | -3.21412 | 0.001308 | 0.003782 | Down |
| EEF1A1P31 | 1.317152196 | -1.529719533 | 0.407252 | -3.7562 | 0.000173 | 0.000608 | Down |
| EEF1A1P33 | 1.556691993 | -1.790548413 | 0.440702 | -4.06295 | 4.85E-05 | 0.00019 | Down |
| EEF1A1P34 | 1.118550794 | -1.814980433 | 0.555467 | -3.26749 | 0.001085 | 0.003195 | Down |
| EEF1DP3 | 32.692048 | -1.067553575 | 0.15245 | -7.00265 | 2.51E-12 | 2.44E-11 | Down |
| EEF1E1P1 | 0.811303354 | -1.458167847 | 0.615092 | -2.37065 | 0.017757 | 0.037313 | Down |
| EEF1GP7 | 1.294957597 | -1.128973228 | 0.398393 | -2.83382 | 0.0046 | 0.011516 | Down |
| EFCAB1 | 3.904030574 | -1.044818066 | 0.325189 | -3.21295 | 0.001314 | 0.003795 | Down |
| EFCAB12 | 41.56021573 | -1.228906422 | 0.159234 | -7.71763 | 1.19E-14 | 1.45E-13 | Down |
| EFCAB6-AS1 | 6.02790669 | -1.159894616 | 0.284191 | -4.08139 | 4.48E-05 | 0.000176 | Down |
| EFCC1 | 78.65129056 | -1.08052282 | 0.109503 | -9.86752 | 5.76E-23 | 1.33E-21 | Down |
| EFEMP1 | 5290.75251 | -1.462262149 | 0.14658 | -9.97586 | 1.94E-23 | 4.64E-22 | Down |
| EFHB | 26.36459936 | -1.241708257 | 0.185347 | -6.69938 | 2.09E-11 | 1.85E-10 | Down |
| EFHC2 | 73.96135715 | -1.342185616 | 0.100697 | -13.3289 | 1.57E-40 | 1.13E-38 | Down |
| EFHD2 | 1749.471967 | 1.096142648 | 0.081921 | 13.38054 | 7.86E-41 | 5.78E-39 | Up |
| EFNA5 | 138.454959 | -1.404071075 | 0.144615 | -9.70903 | 2.76E-22 | 6.07E-21 | Down |
| EGFEM1P | 60.69393092 | 2.491302979 | 0.237883 | 10.4728 | 1.15E-25 | 3.24E-24 | Up |
| EGFL6 | 15.62623875 | 2.030473805 | 0.277927 | 7.305789 | 2.76E-13 | 2.94E-12 | Up |
| EHF | 426.7867486 | 1.593419687 | 0.13154 | 12.11359 | 8.95E-34 | 4.29E-32 | Up |
| EI24P2 | 1.664840654 | -2.605139712 | 0.818545 | -3.18265 | 0.001459 | 0.004168 | Down |
| EI24P4 | 1.390265936 | -2.626479026 | 1.149581 | -2.28473 | 0.022329 | 0.045445 | Down |
| EIF2B5-IT1 | 0.876130334 | -1.673360143 | 0.510918 | -3.2752 | 0.001056 | 0.003117 | Down |
| EIF2S2P2 | 2.989479946 | -1.167027963 | 0.274062 | -4.25827 | 2.06E-05 | 8.63E-05 | Down |
| EIF3LP1 | 1.417586043 | -1.834879991 | 0.591722 | -3.10092 | 0.001929 | 0.005342 | Down |
| EIF4A1P1 | 0.769055256 | -1.645338435 | 0.486788 | -3.37999 | 0.000725 | 0.002221 | Down |
| EIF4A1P5 | 1.078358147 | -1.790318214 | 0.52585 | -3.40462 | 0.000663 | 0.00205 | Down |
| EIF4A2P4 | 1.888830657 | -1.264049441 | 0.411561 | -3.07135 | 0.002131 | 0.005832 | Down |
| EIF4BP2 | 14.54693315 | -1.161353257 | 0.228138 | -5.09058 | 3.57E-07 | 1.96E-06 | Down |
| EIF4BP9 | 1.779907219 | -2.636629443 | 0.757529 | -3.48057 | 0.0005 | 0.001591 | Down |
| EIF4E2P2 | 1.494335061 | -2.681611941 | 0.699494 | -3.83365 | 0.000126 | 0.000456 | Down |
| ELDR | 1.903933262 | -1.381706641 | 0.592396 | -2.3324 | 0.019679 | 0.040776 | Down |
| ELF3 | 547.736143 | 2.148856001 | 0.11171 | 19.236 | 1.85E-82 | 8.63E-80 | Up |
| ELFN1-AS1 | 2.629640989 | -1.863294384 | 0.604033 | -3.08476 | 0.002037 | 0.005605 | Down |
| ELFN2 | 60.50631894 | 1.141364662 | 0.363122 | 3.143196 | 0.001671 | 0.004709 | Up |
| ELK2BP | 1.668692787 | -2.245040661 | 0.934264 | -2.40301 | 0.016261 | 0.034596 | Down |
| ELL2P1 | 5.204236613 | -1.01070882 | 0.268572 | -3.76326 | 0.000168 | 0.000592 | Down |
| ELMO1 | 1890.534222 | -1.837386506 | 0.131295 | -13.9944 | 1.69E-44 | 1.56E-42 | Down |
| ELMO1-AS1 | 1.967655645 | -1.893887016 | 0.385516 | -4.9126 | 8.99E-07 | 4.66E-06 | Down |
| ELMOD1 | 6.936110011 | -1.030815 | 0.319671 | -3.22461 | 0.001261 | 0.003661 | Down |
| EMILIN2 | 803.3448993 | 1.23894222 | 0.071436 | 17.34327 | 2.22E-67 | 5.65E-65 | Up |
| EMILIN3 | 63.63514768 | 1.336180871 | 0.1857 | 7.195369 | 6.23E-13 | 6.40E-12 | Up |
| ENAM | 12.42022107 | -1.449644518 | 0.363473 | -3.98831 | 6.65E-05 | 0.000253 | Down |
| ENC1 | 558.0053118 | 1.377273005 | 0.091253 | 15.0929 | 1.80E-51 | 2.36E-49 | Up |
| ENDOD1 | 2681.041401 | 1.577415208 | 0.072475 | 21.76485 | 5.00E-105 | 5.00E-102 | Up |
| ENKUR | 19.25834409 | -1.734983875 | 0.166405 | -10.4263 | 1.88E-25 | 5.19E-24 | Down |
| ENO1-AS1 | 6.327192432 | -1.336398845 | 0.148225 | -9.01604 | 1.95E-19 | 3.48E-18 | Down |
| ENO4 | 27.33905446 | -1.049299024 | 0.135851 | -7.72392 | 1.13E-14 | 1.38E-13 | Down |
| ENPP1 | 4657.186049 | -1.097258448 | 0.113534 | -9.66456 | 4.26E-22 | 9.26E-21 | Down |
| ENPP6 | 20.08739457 | -1.256680436 | 0.25084 | -5.0099 | 5.45E-07 | 2.91E-06 | Down |
| ENPP7 | 4.058408913 | -1.719683794 | 0.545274 | -3.1538 | 0.001612 | 0.004556 | Down |
| ENPP7P14 | 2.049733503 | -1.004235489 | 0.412451 | -2.4348 | 0.0149 | 0.032142 | Down |
| ENPP7P5 | 3.305120007 | -1.106738712 | 0.231215 | -4.78661 | 1.70E-06 | 8.45E-06 | Down |
| ENTHD1 | 7.288463762 | -1.477813554 | 0.440141 | -3.35759 | 0.000786 | 0.002389 | Down |
| ENTPD1 | 4593.066027 | 2.34559435 | 0.124322 | 18.86711 | 2.13E-79 | 9.00E-77 | Up |
| ENTPD2 | 19.8775543 | 1.479473767 | 0.205277 | 7.207194 | 5.71E-13 | 5.89E-12 | Up |
| ENTPD8 | 34.26448691 | 1.211374205 | 0.161397 | 7.505559 | 6.12E-14 | 6.96E-13 | Up |
| EPB42 | 9.878824523 | -1.424199974 | 0.276552 | -5.14984 | 2.61E-07 | 1.46E-06 | Down |
| EPHA10 | 134.1428168 | 2.591940431 | 0.25627 | 10.11411 | 4.78E-24 | 1.20E-22 | Up |
| EPHA2 | 1064.193837 | 1.037230955 | 0.102237 | 10.14537 | 3.47E-24 | 8.76E-23 | Up |
| EPHA3 | 1465.809669 | -1.027465793 | 0.117944 | -8.71147 | 3.00E-18 | 4.85E-17 | Down |
| EPHA4 | 3609.944177 | 1.412546423 | 0.10122 | 13.95515 | 2.93E-44 | 2.67E-42 | Up |
| EPHA5 | 125.7744922 | -1.899656738 | 0.182287 | -10.4213 | 1.98E-25 | 5.46E-24 | Down |
| EPHA5-AS1 | 7.860656005 | -1.593163264 | 0.391893 | -4.0653 | 4.80E-05 | 0.000188 | Down |
| EPHA6 | 53.21916513 | -1.644243689 | 0.144503 | -11.3786 | 5.34E-30 | 2.04E-28 | Down |
| EPHB1 | 330.0865953 | -1.623276928 | 0.113231 | -14.336 | 1.30E-46 | 1.38E-44 | Down |
| EPHB3 | 231.8163232 | 2.115848493 | 0.108954 | 19.41972 | 5.26E-84 | 2.70E-81 | Up |
| EPHX3 | 13.1533721 | 1.807178012 | 0.206949 | 8.732487 | 2.49E-18 | 4.05E-17 | Up |
| EPHX4 | 50.75746994 | 1.348813406 | 0.153063 | 8.812138 | 1.23E-18 | 2.06E-17 | Up |
| EPO | 0.69675359 | -1.258327277 | 0.555849 | -2.26379 | 0.023587 | 0.047625 | Down |
| EPPIN | 10.50362185 | -2.335062291 | 0.329257 | -7.09192 | 1.32E-12 | 1.32E-11 | Down |
| EPPK1 | 1251.086865 | 2.187642695 | 0.145594 | 15.02566 | 4.99E-51 | 6.42E-49 | Up |
| EPS8 | 2754.229625 | 1.860747063 | 0.092721 | 20.06818 | 1.40E-89 | 9.27E-87 | Up |
| EPX | 2.546183064 | -1.050462529 | 0.300096 | -3.50042 | 0.000465 | 0.001489 | Down |
| EPYC | 19.26565648 | 3.592390545 | 0.550582 | 6.524718 | 6.81E-11 | 5.76E-10 | Up |
| EQTN | 2.733458146 | -1.308100842 | 0.248346 | -5.26724 | 1.38E-07 | 8.04E-07 | Down |
| ERBB3 | 1386.541014 | 1.622047069 | 0.11546 | 14.04856 | 7.86E-45 | 7.49E-43 | Up |
| ERC2-IT1 | 2.764618431 | -1.282678842 | 0.407179 | -3.15016 | 0.001632 | 0.004608 | Down |
| ERICH1-AS1 | 5.622252152 | -2.190198352 | 0.347676 | -6.29954 | 2.99E-10 | 2.37E-09 | Down |
| ERICH4 | 2.574440721 | -2.043575136 | 0.433606 | -4.71297 | 2.44E-06 | 1.19E-05 | Down |
| ERICH6 | 5.735781216 | -1.005565727 | 0.215133 | -4.67415 | 2.95E-06 | 1.42E-05 | Down |
| ERN2 | 4.110222814 | -1.109423779 | 0.467394 | -2.37364 | 0.017614 | 0.037067 | Down |
| ERP27 | 421.8550841 | 1.794194717 | 0.129305 | 13.87564 | 8.90E-44 | 7.91E-42 | Up |
| ERVMER34-1 | 18.42207305 | -1.181427598 | 0.174335 | -6.77679 | 1.23E-11 | 1.11E-10 | Down |
| ESM1 | 95.98895906 | 2.662646674 | 0.215559 | 12.35228 | 4.74E-35 | 2.47E-33 | Up |
| ESPN | 29.89077765 | 2.728326536 | 0.262387 | 10.3981 | 2.53E-25 | 6.93E-24 | Up |
| ESRRB | 10.31369106 | -1.181296182 | 0.295559 | -3.99681 | 6.42E-05 | 0.000245 | Down |
| ESRRG | 370.3217461 | -1.417193029 | 0.132576 | -10.6897 | 1.14E-26 | 3.43E-25 | Down |
| ESX1 | 1.401536324 | -3.161909932 | 0.8581 | -3.68478 | 0.000229 | 0.000787 | Down |
| ETHE1 | 435.069519 | 1.498263555 | 0.091393 | 16.3937 | 2.12E-60 | 4.38E-58 | Up |
| ETNK2 | 648.493405 | 1.356883765 | 0.098293 | 13.80451 | 2.39E-43 | 2.05E-41 | Up |
| ETNPPL | 18.82444878 | -2.123425241 | 0.257485 | -8.2468 | 1.63E-16 | 2.29E-15 | Down |
| ETV4 | 253.1497243 | 2.863837734 | 0.145706 | 19.65492 | 5.25E-86 | 2.91E-83 | Up |
| ETV5 | 1449.319826 | 1.786344446 | 0.085527 | 20.88642 | 7.12E-97 | 5.73E-94 | Up |
| ETV5-AS1 | 3.684770455 | 1.162619793 | 0.261915 | 4.438916 | 9.04E-06 | 4.02E-05 | Up |
| ETV7 | 78.10448746 | 1.336526986 | 0.18612 | 7.181012 | 6.92E-13 | 7.09E-12 | Up |
| EVA1A | 229.2281681 | 2.461978919 | 0.11978 | 20.55426 | 7.05E-94 | 5.08E-91 | Up |
| EVPL | 1166.99002 | 1.166963913 | 0.095827 | 12.17781 | 4.08E-34 | 1.99E-32 | Up |
| EXD1 | 1.58353463 | -1.811734085 | 0.590288 | -3.06924 | 0.002146 | 0.005871 | Down |
| EXOC7P1 | 1.792842922 | -3.39111249 | 0.99893 | -3.39474 | 0.000687 | 0.002118 | Down |
| EXTL3-AS1 | 11.76045101 | -1.269311136 | 0.268316 | -4.73067 | 2.24E-06 | 1.10E-05 | Down |
| EYA1 | 44.05378051 | -1.566992927 | 0.241834 | -6.47963 | 9.19E-11 | 7.68E-10 | Down |
| EYA4 | 114.3978845 | -1.051244579 | 0.176984 | -5.93978 | 2.85E-09 | 2.03E-08 | Down |
| F10 | 112.3250177 | -1.851720136 | 0.168195 | -11.0093 | 3.44E-28 | 1.17E-26 | Down |
| F11 | 1.429427899 | -1.612903893 | 0.65619 | -2.45798 | 0.013972 | 0.030425 | Down |
| F11-AS1 | 2.683666048 | -2.460028915 | 0.647742 | -3.79785 | 0.000146 | 0.000521 | Down |
| F2R | 919.3451592 | 1.1694883 | 0.100886 | 11.59212 | 4.52E-31 | 1.87E-29 | Up |
| F2RL2 | 58.07591535 | 1.4410041 | 0.19385 | 7.433597 | 1.06E-13 | 1.17E-12 | Up |
| F9 | 1.293624252 | -2.333535618 | 1.016026 | -2.29673 | 0.021634 | 0.044215 | Down |
| FAAH | 770.4154196 | -1.04955011 | 0.0844 | -12.4354 | 1.68E-35 | 8.99E-34 | Down |
| FAAHP1 | 12.8900943 | -1.031208043 | 0.17322 | -5.95315 | 2.63E-09 | 1.88E-08 | Down |
| FABP1 | 2.172344759 | -2.558459418 | 0.647013 | -3.95426 | 7.68E-05 | 0.000289 | Down |
| FABP2 | 2.415239355 | -1.551624114 | 0.456199 | -3.4012 | 0.000671 | 0.002073 | Down |
| FABP3 | 77.92044647 | 1.155978795 | 0.166806 | 6.930097 | 4.21E-12 | 4.00E-11 | Up |
| FABP4 | 740.2748833 | -2.509642318 | 0.240008 | -10.4565 | 1.37E-25 | 3.82E-24 | Down |
| FABP5P2 | 0.815697585 | -1.053685068 | 0.426021 | -2.47332 | 0.013386 | 0.029357 | Down |
| FABP5P7 | 0.794216799 | -1.090431314 | 0.464237 | -2.34887 | 0.01883 | 0.039233 | Down |
| FABP7 | 3.213169055 | -2.477765091 | 0.550078 | -4.50438 | 6.66E-06 | 3.03E-05 | Down |
| FABP7P2 | 1.307692799 | -1.775157762 | 0.460243 | -3.857 | 0.000115 | 0.000418 | Down |
| FAIM2 | 41.24816839 | -1.125460414 | 0.202862 | -5.54792 | 2.89E-08 | 1.83E-07 | Down |
| FAM101A | 21.3667796 | 1.285176751 | 0.211361 | 6.080472 | 1.20E-09 | 8.92E-09 | Up |
| FAM110C | 429.6153641 | 1.016107855 | 0.096582 | 10.52062 | 6.94E-26 | 1.98E-24 | Up |
| FAM111B | 172.5134005 | 1.882645878 | 0.223624 | 8.418782 | 3.80E-17 | 5.67E-16 | Up |
| FAM124A | 122.298037 | -1.49589404 | 0.133285 | -11.2233 | 3.13E-29 | 1.14E-27 | Down |
| FAM129B | 5890.741629 | 1.00556198 | 0.079735 | 12.61133 | 1.83E-36 | 1.04E-34 | Up |
| FAM129C | 107.4530755 | -1.221032955 | 0.403096 | -3.02913 | 0.002453 | 0.006594 | Down |
| FAM131C | 13.49557812 | -1.228349179 | 0.174996 | -7.01928 | 2.23E-12 | 2.18E-11 | Down |
| FAM132B | 488.7344474 | 1.58378929 | 0.158724 | 9.978259 | 1.90E-23 | 4.54E-22 | Up |
| FAM138B | 2.335071116 | -1.453003779 | 0.583812 | -2.48882 | 0.012817 | 0.028288 | Down |
| FAM138C | 1.421641845 | -1.649833843 | 0.690125 | -2.39063 | 0.01682 | 0.035637 | Down |
| FAM138D | 1.749232929 | -1.997281376 | 0.777048 | -2.57035 | 0.01016 | 0.023056 | Down |
| FAM149B1P1 | 1.653044743 | -2.128575959 | 0.688517 | -3.09154 | 0.001991 | 0.005496 | Down |
| FAM151A | 7.485737734 | -1.282545968 | 0.314808 | -4.07406 | 4.62E-05 | 0.000182 | Down |
| FAM153C | 8.553767278 | -1.18353228 | 0.262456 | -4.50944 | 6.50E-06 | 2.96E-05 | Down |
| FAM154A | 4.538939906 | -1.736397962 | 0.280994 | -6.17949 | 6.43E-10 | 4.93E-09 | Down |
| FAM163A | 18.25516249 | -2.143350233 | 0.225676 | -9.49747 | 2.15E-21 | 4.46E-20 | Down |
| FAM167A | 2623.393064 | -2.140693525 | 0.159957 | -13.3829 | 7.61E-41 | 5.64E-39 | Down |
| FAM167A-AS1 | 10.87703318 | -2.381828123 | 0.206054 | -11.5592 | 6.63E-31 | 2.71E-29 | Down |
| FAM170B | 1.902565828 | -1.294198619 | 0.546509 | -2.36812 | 0.017879 | 0.03755 | Down |
| FAM171A2 | 144.0242964 | 1.357146338 | 0.128013 | 10.60166 | 2.93E-26 | 8.55E-25 | Up |
| FAM178B | 11.06734435 | 2.662040428 | 0.266005 | 10.00748 | 1.41E-23 | 3.43E-22 | Up |
| FAM180B | 66.40704618 | -2.260852846 | 0.204172 | -11.0733 | 1.69E-28 | 5.87E-27 | Down |
| FAM181A | 33.30430276 | -1.265995399 | 0.142151 | -8.90599 | 5.29E-19 | 9.12E-18 | Down |
| FAM181A-AS1 | 12.03564941 | -2.104602746 | 0.208693 | -10.0847 | 6.46E-24 | 1.60E-22 | Down |
| FAM181B | 61.58718659 | -1.910862658 | 0.144526 | -13.2216 | 6.58E-40 | 4.60E-38 | Down |
| FAM182A | 23.81537722 | -1.356338937 | 0.238196 | -5.69421 | 1.24E-08 | 8.21E-08 | Down |
| FAM182B | 23.03720913 | -1.140554859 | 0.208004 | -5.48334 | 4.17E-08 | 2.58E-07 | Down |
| FAM183B | 1.871175133 | -2.080608898 | 0.564916 | -3.68304 | 0.00023 | 0.000791 | Down |
| FAM186A | 26.44468084 | -1.00726843 | 0.225984 | -4.45726 | 8.30E-06 | 3.72E-05 | Down |
| FAM189A1 | 125.2320967 | -1.719367966 | 0.184904 | -9.29869 | 1.42E-20 | 2.76E-19 | Down |
| FAM19A5 | 38.29361593 | 1.034318678 | 0.202864 | 5.098584 | 3.42E-07 | 1.88E-06 | Up |
| FAM201A | 71.18892307 | -1.29720568 | 0.111876 | -11.5951 | 4.37E-31 | 1.80E-29 | Down |
| FAM201B | 1.918708688 | -1.369379632 | 0.347002 | -3.94632 | 7.94E-05 | 0.000298 | Down |
| FAM205A | 13.00313825 | -1.23365463 | 0.249805 | -4.93848 | 7.87E-07 | 4.11E-06 | Down |
| FAM205BP | 8.465001997 | -1.671884906 | 0.310021 | -5.39281 | 6.94E-08 | 4.17E-07 | Down |
| FAM205CP | 12.37911595 | -2.68910629 | 0.321933 | -8.35301 | 6.65E-17 | 9.70E-16 | Down |
| FAM20A | 985.8172316 | 2.330419982 | 0.143647 | 16.22319 | 3.46E-59 | 6.73E-57 | Up |
| FAM20C | 667.6613989 | 1.103688166 | 0.077238 | 14.2895 | 2.54E-46 | 2.63E-44 | Up |
| FAM210CP | 0.580432895 | -1.254168309 | 0.536229 | -2.33887 | 0.019342 | 0.040157 | Down |
| FAM212B-AS1 | 11.16721358 | -1.023293174 | 0.142044 | -7.20406 | 5.84E-13 | 6.02E-12 | Down |
| FAM217A | 5.451635452 | -1.574138219 | 0.423232 | -3.71933 | 0.0002 | 0.000695 | Down |
| FAM218A | 82.16814988 | -1.110567433 | 0.090511 | -12.27 | 1.31E-34 | 6.56E-33 | Down |
| FAM220CP | 2.986244185 | -1.395259491 | 0.296644 | -4.70348 | 2.56E-06 | 1.24E-05 | Down |
| FAM230A | 5.91366491 | 1.410799047 | 0.474289 | 2.974554 | 0.002934 | 0.00773 | Up |
| FAM230B | 37.71366851 | 1.611545352 | 0.363762 | 4.430224 | 9.41E-06 | 4.18E-05 | Up |
| FAM231D | 9.661200926 | 1.194706067 | 0.531797 | 2.246545 | 0.024669 | 0.049532 | Up |
| FAM26D | 1.246415368 | -1.964790086 | 0.686593 | -2.86165 | 0.004214 | 0.01066 | Down |
| FAM27E3 | 40.80685528 | -1.191181692 | 0.188735 | -6.31139 | 2.77E-10 | 2.20E-09 | Down |
| FAM3B | 114.0785052 | -1.125789147 | 0.164315 | -6.8514 | 7.31E-12 | 6.80E-11 | Down |
| FAM41C | 7.004467751 | -1.16709551 | 0.32665 | -3.57292 | 0.000353 | 0.001164 | Down |
| FAM43A | 443.4412976 | 1.495157054 | 0.110668 | 13.51028 | 1.36E-41 | 1.05E-39 | Up |
| FAM46B | 137.4721678 | -1.205523933 | 0.160609 | -7.50594 | 6.10E-14 | 6.94E-13 | Down |
| FAM46D | 6.41327044 | -1.389087694 | 0.283565 | -4.89866 | 9.65E-07 | 4.98E-06 | Down |
| FAM47A | 5.90804301 | -2.648013619 | 0.356894 | -7.41961 | 1.17E-13 | 1.30E-12 | Down |
| FAM47C | 4.071731021 | -2.423372405 | 0.464895 | -5.21273 | 1.86E-07 | 1.06E-06 | Down |
| FAM57B | 3.096180995 | -1.209518959 | 0.45568 | -2.65432 | 0.007947 | 0.018623 | Down |
| FAM65C | 107.178957 | -1.362346665 | 0.150209 | -9.06966 | 1.19E-19 | 2.17E-18 | Down |
| FAM71A | 3.013124134 | -1.82258155 | 0.478693 | -3.80741 | 0.00014 | 0.000503 | Down |
| FAM71C | 10.42310254 | -2.073833312 | 0.280976 | -7.38081 | 1.57E-13 | 1.72E-12 | Down |
| FAM71F1 | 1.727697195 | -2.323872118 | 0.528652 | -4.39585 | 1.10E-05 | 4.84E-05 | Down |
| FAM71F2 | 40.98772706 | -1.045219559 | 0.187722 | -5.56791 | 2.58E-08 | 1.64E-07 | Down |
| FAM74A4 | 2.893582427 | -1.645198201 | 0.567863 | -2.89718 | 0.003765 | 0.009652 | Down |
| FAM83C | 3.514005505 | -1.669513123 | 0.520657 | -3.20655 | 0.001343 | 0.003869 | Down |
| FAM83E | 5.978425344 | -1.253031294 | 0.286625 | -4.37168 | 1.23E-05 | 5.37E-05 | Down |
| FAM84A | 422.0978829 | 1.836314575 | 0.117739 | 15.59646 | 7.69E-55 | 1.22E-52 | Up |
| FAM8A6P | 1.251376572 | -2.504812145 | 0.96642 | -2.59185 | 0.009546 | 0.021873 | Down |
| FAM90A10P | 1.275556544 | -3.15042415 | 0.986924 | -3.19216 | 0.001412 | 0.004046 | Down |
| FAM90A24P | 5.777167522 | -1.56858329 | 0.385719 | -4.06664 | 4.77E-05 | 0.000187 | Down |
| FAM90A28P | 1.508849831 | -2.071336616 | 0.669471 | -3.09399 | 0.001975 | 0.005456 | Down |
| FAM92A1P2 | 1.679307319 | -1.648391713 | 0.586553 | -2.81031 | 0.004949 | 0.012275 | Down |
| FAM95B1 | 78.93843126 | -1.496714951 | 0.203592 | -7.35154 | 1.96E-13 | 2.12E-12 | Down |
| FAM95C | 70.15366372 | -2.082543309 | 0.208522 | -9.98718 | 1.73E-23 | 4.18E-22 | Down |
| FAM9C | 3.357565723 | -2.44931502 | 0.43016 | -5.69397 | 1.24E-08 | 8.22E-08 | Down |
| FANCD2OS | 1.38378477 | -1.296816858 | 0.453724 | -2.85816 | 0.004261 | 0.010761 | Down |
| FAP | 420.7172476 | 1.840570378 | 0.167996 | 10.95604 | 6.22E-28 | 2.06E-26 | Up |
| FAR1-IT1 | 3.024062785 | -1.094178398 | 0.328321 | -3.33265 | 0.00086 | 0.002593 | Down |
| FAR2P2 | 179.0881944 | -1.433553437 | 0.120169 | -11.9295 | 8.31E-33 | 3.79E-31 | Down |
| FAR2P3 | 5.725818729 | -1.510367242 | 0.189561 | -7.96769 | 1.62E-15 | 2.11E-14 | Down |
| FARSBP1 | 1.513880106 | -2.89744491 | 1.19215 | -2.43044 | 0.015081 | 0.032464 | Down |
| FAS | 698.0388658 | 1.393498747 | 0.097609 | 14.27635 | 3.07E-46 | 3.15E-44 | Up |
| FAT2 | 195.1078223 | 1.435414324 | 0.177227 | 8.099303 | 5.53E-16 | 7.46E-15 | Up |
| FAXC | 237.9644408 | 2.668634685 | 0.146147 | 18.25998 | 1.72E-74 | 6.10E-72 | Up |
| FAXDC2 | 1430.531553 | -1.216640206 | 0.088225 | -13.7902 | 2.92E-43 | 2.49E-41 | Down |
| FBLL1 | 16.82621677 | 1.044842381 | 0.238615 | 4.378785 | 1.19E-05 | 5.21E-05 | Up |
| FBLN1 | 3444.238598 | -1.197495931 | 0.175764 | -6.81309 | 9.55E-12 | 8.77E-11 | Down |
| FBLN5 | 917.1934181 | -1.195066449 | 0.120256 | -9.9377 | 2.85E-23 | 6.75E-22 | Down |
| FBLN7 | 352.4033245 | -1.392122877 | 0.083371 | -16.698 | 1.36E-62 | 2.96E-60 | Down |
| FBXO15 | 47.09089728 | -1.439690489 | 0.121907 | -11.8098 | 3.48E-32 | 1.54E-30 | Down |
| FBXO2 | 269.433243 | 1.455554759 | 0.08717 | 16.69792 | 1.36E-62 | 2.96E-60 | Up |
| FBXO40 | 11.32323944 | -2.452140739 | 0.334869 | -7.32269 | 2.43E-13 | 2.60E-12 | Down |
| FBXO43 | 10.02933813 | -1.114304939 | 0.239258 | -4.65734 | 3.20E-06 | 1.53E-05 | Down |
| FBXW10 | 19.28053555 | -1.891598491 | 0.213578 | -8.85672 | 8.24E-19 | 1.40E-17 | Down |
| FBXW12 | 6.711543699 | -1.296993838 | 0.265544 | -4.88429 | 1.04E-06 | 5.33E-06 | Down |
| FCAMR | 67.27813677 | -1.583409574 | 0.568815 | -2.7837 | 0.005374 | 0.013214 | Down |
| FCER1A | 169.4557 | 1.32366997 | 0.178961 | 7.396407 | 1.40E-13 | 1.53E-12 | Up |
| FCER1G | 323.8986847 | 1.002086778 | 0.187772 | 5.336726 | 9.46E-08 | 5.60E-07 | Up |
| FCER2 | 102.1204135 | -1.23348377 | 0.468796 | -2.63117 | 0.008509 | 0.019784 | Down |
| FCF1P3 | 1.403865836 | -1.850693537 | 0.52316 | -3.53753 | 0.000404 | 0.001313 | Down |
| FCGBP | 28626.87063 | -1.391625549 | 0.136089 | -10.2259 | 1.52E-24 | 3.91E-23 | Down |
| FCGR1A | 147.4782582 | 1.6661834 | 0.220147 | 7.568504 | 3.78E-14 | 4.37E-13 | Up |
| FCGR1C | 9.825629751 | 1.365014838 | 0.22801 | 5.986639 | 2.14E-09 | 1.55E-08 | Up |
| FCGR2A | 900.3499651 | 1.289603786 | 0.132552 | 9.729025 | 2.27E-22 | 5.03E-21 | Up |
| FCGR3A | 810.6067401 | 1.905017558 | 0.198859 | 9.579747 | 9.73E-22 | 2.06E-20 | Up |
| FCGR3B | 79.19403045 | -1.240997794 | 0.178654 | -6.94638 | 3.75E-12 | 3.58E-11 | Down |
| FCN2 | 1.601318235 | -1.385958414 | 0.446083 | -3.10695 | 0.00189 | 0.00525 | Down |
| FCRL1 | 123.4735447 | -1.174683103 | 0.456603 | -2.57266 | 0.010092 | 0.02293 | Down |
| FCRL3 | 170.2762816 | -1.124557264 | 0.390601 | -2.87905 | 0.003989 | 0.010148 | Down |
| FCRLA | 128.9599349 | -1.04760474 | 0.434771 | -2.40955 | 0.015972 | 0.03407 | Down |
| FDPSP4 | 2.132683263 | -1.45500252 | 0.393635 | -3.69633 | 0.000219 | 0.000754 | Down |
| FEM1AP3 | 1.355045705 | -3.194853441 | 0.995083 | -3.21064 | 0.001324 | 0.003822 | Down |
| FEM1AP4 | 2.622881565 | -2.651864852 | 0.71061 | -3.73182 | 0.00019 | 0.000664 | Down |
| FEN1P1 | 3.174917953 | -2.075412556 | 0.35747 | -5.80584 | 6.40E-09 | 4.37E-08 | Down |
| FENDRR | 27.1872494 | -1.66546238 | 0.197223 | -8.44455 | 3.05E-17 | 4.58E-16 | Down |
| FER1L5 | 20.2127309 | -1.608536292 | 0.218241 | -7.37045 | 1.70E-13 | 1.85E-12 | Down |
| FER1L6 | 189.2368499 | -3.025453902 | 0.236211 | -12.8083 | 1.47E-37 | 8.87E-36 | Down |
| FER1L6-AS1 | 4.187526519 | -3.026815658 | 0.432641 | -6.99614 | 2.63E-12 | 2.55E-11 | Down |
| FER1L6-AS2 | 2.223200366 | -2.153808067 | 0.397134 | -5.42338 | 5.85E-08 | 3.55E-07 | Down |
| FERMT1 | 164.8451511 | -1.264858474 | 0.146527 | -8.63227 | 6.01E-18 | 9.54E-17 | Down |
| FEZF1 | 2.066067335 | -1.44380827 | 0.629135 | -2.29491 | 0.021738 | 0.044397 | Down |
| FEZF1-AS1 | 1.132919531 | -1.840910095 | 0.77566 | -2.37335 | 0.017628 | 0.03709 | Down |
| FEZF2 | 1.926651232 | -2.152312448 | 0.749444 | -2.87188 | 0.00408 | 0.010352 | Down |
| FFAR3 | 9.348305544 | -1.28243491 | 0.221129 | -5.7995 | 6.65E-09 | 4.53E-08 | Down |
| FGA | 2.284060881 | -1.457335593 | 0.610469 | -2.38724 | 0.016975 | 0.035917 | Down |
| FGB | 1.688173809 | -2.318538988 | 0.91316 | -2.53903 | 0.011116 | 0.024944 | Down |
| FGF1 | 379.6638681 | 1.450480098 | 0.094558 | 15.33953 | 4.16E-53 | 5.93E-51 | Up |
| FGF10 | 81.38293561 | -1.811320399 | 0.163638 | -11.0691 | 1.77E-28 | 6.13E-27 | Down |
| FGF10-AS1 | 4.625383924 | -2.00456617 | 0.296189 | -6.76786 | 1.31E-11 | 1.18E-10 | Down |
| FGF11 | 212.9820491 | -1.204864318 | 0.09514 | -12.6641 | 9.35E-37 | 5.41E-35 | Down |
| FGF12-AS2 | 1.905664704 | -1.110278385 | 0.462802 | -2.39903 | 0.016438 | 0.034939 | Down |
| FGF14-IT1 | 5.63756444 | -1.107964582 | 0.266071 | -4.16416 | 3.12E-05 | 0.000127 | Down |
| FGF16 | 5.034075828 | -1.561799566 | 0.341907 | -4.56791 | 4.93E-06 | 2.29E-05 | Down |
| FGF17 | 33.79595186 | -1.15468916 | 0.184874 | -6.24581 | 4.22E-10 | 3.30E-09 | Down |
| FGF19 | 5.899183284 | -1.131647517 | 0.451995 | -2.50367 | 0.012291 | 0.02727 | Down |
| FGF20 | 12.43080904 | 1.385121865 | 0.238338 | 5.811597 | 6.19E-09 | 4.23E-08 | Up |
| FGF23 | 8.607535236 | -1.327817425 | 0.427207 | -3.10813 | 0.001883 | 0.005233 | Down |
| FGFBP2 | 15.49842387 | -1.30527587 | 0.183894 | -7.09799 | 1.27E-12 | 1.27E-11 | Down |
| FGG | 2.376328221 | -1.955449295 | 0.707034 | -2.76571 | 0.00568 | 0.013864 | Down |
| FGL1 | 3.169817792 | -1.126497392 | 0.455758 | -2.4717 | 0.013447 | 0.029476 | Down |
| FHL1 | 6545.8861 | -1.834772019 | 0.143555 | -12.781 | 2.09E-37 | 1.25E-35 | Down |
| FIBCD1 | 46.28162062 | 3.448188575 | 0.330666 | 10.42801 | 1.85E-25 | 5.10E-24 | Up |
| FIGF | 8.98514645 | -1.646651902 | 0.234149 | -7.0325 | 2.03E-12 | 1.99E-11 | Down |
| FIRRE | 9.47871789 | -1.003014964 | 0.184673 | -5.43129 | 5.59E-08 | 3.40E-07 | Down |
| FKBP1C | 4.633541222 | -1.030003962 | 0.267171 | -3.85523 | 0.000116 | 0.00042 | Down |
| FKBP4P1 | 3.614265111 | -1.684139717 | 0.304277 | -5.53489 | 3.11E-08 | 1.96E-07 | Down |
| FKBP4P7 | 2.816628319 | -2.78367643 | 1.149422 | -2.42181 | 0.015444 | 0.033122 | Down |
| FKBP6 | 1.441639565 | -2.285888871 | 0.678634 | -3.36837 | 0.000756 | 0.002306 | Down |
| FKSG59 | 5.502217653 | -1.284455084 | 0.340432 | -3.77302 | 0.000161 | 0.000572 | Down |
| FKSG61 | 6.199903404 | -1.358146614 | 0.277507 | -4.89409 | 9.88E-07 | 5.08E-06 | Down |
| FKSG66 | 3.046482494 | -1.502162105 | 0.327863 | -4.58168 | 4.61E-06 | 2.15E-05 | Down |
| FKSG70 | 1.792946617 | -1.10957985 | 0.405295 | -2.73771 | 0.006187 | 0.014949 | Down |
| FLJ16779 | 13.81586869 | 1.320755478 | 0.283677 | 4.655841 | 3.23E-06 | 1.54E-05 | Up |
| FLJ20019 | 2.182389416 | -2.111683628 | 0.574851 | -3.67344 | 0.000239 | 0.000818 | Down |
| FLJ30679 | 2.176115506 | -1.778871733 | 0.446532 | -3.98375 | 6.78E-05 | 0.000258 | Down |
| FLJ31356 | 4.998468087 | -1.14091499 | 0.355925 | -3.2055 | 0.001348 | 0.003882 | Down |
| FLJ37505 | 1.494734149 | -2.906686955 | 1.03388 | -2.81144 | 0.004932 | 0.012239 | Down |
| FLJ38122 | 9.392823788 | -2.233502235 | 0.363 | -6.1529 | 7.61E-10 | 5.80E-09 | Down |
| FLJ39080 | 2.163528326 | -2.243920641 | 0.604328 | -3.71308 | 0.000205 | 0.00071 | Down |
| FLJ41941 | 3.664681648 | -1.822741585 | 0.581694 | -3.1335 | 0.001727 | 0.004848 | Down |
| FLJ42393 | 22.88175067 | -1.042772819 | 0.208295 | -5.00622 | 5.55E-07 | 2.96E-06 | Down |
| FLJ42969 | 4.476233977 | -1.532875674 | 0.293737 | -5.21853 | 1.80E-07 | 1.03E-06 | Down |
| FLJ46066 | 1.781457129 | -3.075042747 | 1.208412 | -2.5447 | 0.010937 | 0.024593 | Down |
| FLNC | 906.7464386 | -1.328569918 | 0.187678 | -7.079 | 1.45E-12 | 1.45E-11 | Down |
| FLRT1 | 179.4335562 | -2.157910125 | 0.153426 | -14.0648 | 6.25E-45 | 6.00E-43 | Down |
| FLRT2 | 1637.376706 | -1.291918266 | 0.119002 | -10.8563 | 1.86E-27 | 5.98E-26 | Down |
| FLRT3 | 810.8697082 | 1.879125523 | 0.148775 | 12.63065 | 1.43E-36 | 8.18E-35 | Up |
| FLT4 | 1301.744036 | -1.300376986 | 0.097986 | -13.2711 | 3.41E-40 | 2.41E-38 | Down |
| FLYWCH1P1 | 1.997459915 | -2.030861529 | 0.517024 | -3.92798 | 8.57E-05 | 0.000319 | Down |
| FMO2 | 597.7323212 | -1.340400885 | 0.157499 | -8.51054 | 1.73E-17 | 2.65E-16 | Down |
| FMO6P | 2.828955794 | -1.636983959 | 0.43927 | -3.7266 | 0.000194 | 0.000677 | Down |
| FMOD | 2320.656324 | -1.230094086 | 0.112665 | -10.9182 | 9.44E-28 | 3.09E-26 | Down |
| FMR1-AS1 | 7.371637156 | -1.526112502 | 0.298114 | -5.11922 | 3.07E-07 | 1.70E-06 | Down |
| FN1 | 254681.6616 | 4.681701552 | 0.301931 | 15.50584 | 3.17E-54 | 4.78E-52 | Up |
| FNDC4 | 286.5001448 | 2.274130532 | 0.14929 | 15.23293 | 2.14E-52 | 2.91E-50 | Up |
| FNDC7 | 1.574592642 | -1.589623651 | 0.534461 | -2.97425 | 0.002937 | 0.007736 | Down |
| FOLH1 | 72.08145835 | 1.517953976 | 0.175118 | 8.668183 | 4.39E-18 | 7.03E-17 | Up |
| FOLR1 | 465.1900155 | -1.256974517 | 0.143954 | -8.73177 | 2.51E-18 | 4.08E-17 | Down |
| FOLR3 | 5.42907845 | -1.318397067 | 0.286073 | -4.60861 | 4.05E-06 | 1.91E-05 | Down |
| FOS | 10595.84512 | -1.160710393 | 0.224796 | -5.1634 | 2.43E-07 | 1.36E-06 | Down |
| FOSB | 3331.549914 | -2.25452213 | 0.27643 | -8.15585 | 3.47E-16 | 4.76E-15 | Down |
| FOXA2 | 107.0863154 | -2.118155065 | 0.213857 | -9.90455 | 3.98E-23 | 9.29E-22 | Down |
| FOXD3 | 5.927289618 | -2.100431363 | 0.362907 | -5.78779 | 7.13E-09 | 4.85E-08 | Down |
| FOXD3-AS1 | 1.51511816 | -1.683152352 | 0.456901 | -3.68385 | 0.00023 | 0.000789 | Down |
| FOXD4L3 | 5.705942327 | -1.302432688 | 0.453259 | -2.87349 | 0.00406 | 0.010306 | Down |
| FOXD4L4 | 12.32413048 | -1.074486136 | 0.300017 | -3.58142 | 0.000342 | 0.00113 | Down |
| FOXD4L5 | 10.94357894 | -1.276329726 | 0.315544 | -4.04485 | 5.24E-05 | 0.000204 | Down |
| FOXF2 | 42.99908233 | -1.122796912 | 0.164993 | -6.80513 | 1.01E-11 | 9.25E-11 | Down |
| FOXI2 | 36.01453135 | 1.601904964 | 0.316573 | 5.06014 | 4.19E-07 | 2.28E-06 | Up |
| FOXJ1 | 117.4244784 | -2.386648401 | 0.210932 | -11.3148 | 1.11E-29 | 4.16E-28 | Down |
| FOXN1 | 12.85153368 | -1.482328503 | 0.256219 | -5.78541 | 7.23E-09 | 4.91E-08 | Down |
| FOXN3P1 | 1.490875828 | -3.205327101 | 1.037871 | -3.08837 | 0.002013 | 0.005546 | Down |
| FOXN4 | 2.43129519 | -1.926530385 | 0.473721 | -4.0668 | 4.77E-05 | 0.000187 | Down |
| FOXO1B | 2.139156754 | -3.288061752 | 0.670927 | -4.90077 | 9.55E-07 | 4.93E-06 | Down |
| FOXP1-AS1 | 0.928561354 | -1.630876446 | 0.48465 | -3.36506 | 0.000765 | 0.002331 | Down |
| FOXP2 | 472.2365076 | -1.579237806 | 0.116766 | -13.5248 | 1.12E-41 | 8.70E-40 | Down |
| FOXQ1 | 214.0259524 | 2.473579257 | 0.170162 | 14.53657 | 7.11E-48 | 8.01E-46 | Up |
| FOXR1 | 1.221370797 | -2.162009854 | 0.596232 | -3.62612 | 0.000288 | 0.000967 | Down |
| FP236383.5 | 6.28244193 | -1.205799911 | 0.356952 | -3.37805 | 0.00073 | 0.002235 | Down |
| FP325317.1 | 4.018665189 | -1.930640421 | 0.378743 | -5.09749 | 3.44E-07 | 1.89E-06 | Down |
| FPR2 | 17.75801246 | -1.004599642 | 0.205195 | -4.89583 | 9.79E-07 | 5.04E-06 | Down |
| FPR3 | 504.3810518 | 1.071782885 | 0.187462 | 5.71732 | 1.08E-08 | 7.22E-08 | Up |
| FRAS1 | 3290.58457 | -1.157504751 | 0.134339 | -8.61627 | 6.92E-18 | 1.09E-16 | Down |
| FREM2-AS1 | 0.995260653 | -1.129321353 | 0.392722 | -2.87562 | 0.004032 | 0.010246 | Down |
| FRMD1 | 5.252463182 | -2.510350409 | 0.424676 | -5.91122 | 3.40E-09 | 2.39E-08 | Down |
| FRMD3 | 3574.548197 | 2.493896127 | 0.136933 | 18.21255 | 4.10E-74 | 1.43E-71 | Up |
| FRMD5 | 198.6313778 | 2.904958823 | 0.220562 | 13.17073 | 1.29E-39 | 8.91E-38 | Up |
| FRMD7 | 2.638033716 | -1.518684081 | 0.463954 | -3.27335 | 0.001063 | 0.003135 | Down |
| FRMD8P1 | 2.065250924 | -2.866650689 | 0.710681 | -4.03367 | 5.49E-05 | 0.000213 | Down |
| FRMPD1 | 21.17769544 | -1.467313185 | 0.225704 | -6.50105 | 7.98E-11 | 6.70E-10 | Down |
| FRY-AS1 | 2.778870595 | -2.156629007 | 0.31322 | -6.88534 | 5.76E-12 | 5.42E-11 | Down |
| FSCB | 8.430612737 | -1.138577783 | 0.35299 | -3.22552 | 0.001257 | 0.003651 | Down |
| FSCN3 | 4.982208797 | -1.335258657 | 0.299193 | -4.46286 | 8.09E-06 | 3.63E-05 | Down |
| FSHR | 1.588248442 | -1.742925412 | 0.718717 | -2.42505 | 0.015306 | 0.03287 | Down |
| FSTL3 | 675.7934662 | 1.301540193 | 0.089569 | 14.53122 | 7.68E-48 | 8.64E-46 | Up |
| FSTL4 | 28.36020538 | 1.717483044 | 0.245845 | 6.986039 | 2.83E-12 | 2.73E-11 | Up |
| FTCD | 8.481824565 | -1.44540013 | 0.273866 | -5.27777 | 1.31E-07 | 7.60E-07 | Down |
| FTCDNL1 | 130.423388 | -1.440527884 | 0.101328 | -14.2165 | 7.24E-46 | 7.30E-44 | Down |
| FTLP10 | 0.91351783 | -4.234281268 | 1.774605 | -2.38604 | 0.017031 | 0.036016 | Down |
| FTLP16 | 0.486427898 | -1.73541279 | 0.528227 | -3.28535 | 0.001019 | 0.00302 | Down |
| FTMT | 1.130917125 | -2.175027882 | 0.887018 | -2.45207 | 0.014204 | 0.030848 | Down |
| FUCA1P1 | 1.217914414 | -1.483780388 | 0.534402 | -2.77653 | 0.005494 | 0.01347 | Down |
| FUNDC2P2 | 0.912788924 | -2.093390734 | 0.825739 | -2.53517 | 0.011239 | 0.025189 | Down |
| FUT3 | 13.91927895 | 1.104180797 | 0.404433 | 2.730193 | 0.00633 | 0.015263 | Up |
| FUT6 | 3.451893084 | -1.409124511 | 0.425374 | -3.31268 | 0.000924 | 0.002768 | Down |
| FXYD1 | 93.54380623 | -1.535656002 | 0.19741 | -7.77903 | 7.31E-15 | 9.09E-14 | Down |
| FXYD3 | 28.43415726 | 2.316657911 | 0.306593 | 7.556127 | 4.15E-14 | 4.79E-13 | Up |
| FXYD4 | 0.874462546 | -2.00440856 | 0.666124 | -3.00906 | 0.002621 | 0.006988 | Down |
| FXYD5 | 1005.41606 | 1.225682411 | 0.134604 | 9.105862 | 8.56E-20 | 1.57E-18 | Up |
| FXYD6 | 1061.199395 | -1.325850152 | 0.098878 | -13.409 | 5.36E-41 | 4.00E-39 | Down |
| FXYD7 | 5.57339629 | -1.003996724 | 0.330147 | -3.04106 | 0.002357 | 0.006374 | Down |
| FZD10-AS1 | 55.3397715 | -1.209039641 | 0.149689 | -8.077 | 6.64E-16 | 8.91E-15 | Down |
| FZD8 | 527.8022306 | -1.011093897 | 0.103899 | -9.73155 | 2.21E-22 | 4.91E-21 | Down |
| FZD9 | 15.2595817 | -2.449635259 | 0.216936 | -11.292 | 1.44E-29 | 5.35E-28 | Down |
| G0S2 | 309.546566 | 1.801575064 | 0.175306 | 10.27674 | 8.97E-25 | 2.36E-23 | Up |
| G6PC | 1.661223285 | -1.392549 | 0.440465 | -3.16154 | 0.001569 | 0.004449 | Down |
| GABARAPL3 | 6.288320123 | -1.409077292 | 0.39531 | -3.56449 | 0.000365 | 0.001198 | Down |
| GABBR2 | 886.4119603 | 2.652609785 | 0.163061 | 16.26756 | 1.68E-59 | 3.34E-57 | Up |
| GABRA1 | 1.648334968 | 1.975924963 | 0.508999 | 3.881982 | 0.000104 | 0.00038 | Up |
| GABRA2 | 8.833004706 | -1.374591157 | 0.310272 | -4.43028 | 9.41E-06 | 4.18E-05 | Down |
| GABRA4 | 3.219899194 | -1.579373149 | 0.42123 | -3.74943 | 0.000177 | 0.000623 | Down |
| GABRA5 | 6.091871581 | -1.038550363 | 0.340981 | -3.04577 | 0.002321 | 0.006286 | Down |
| GABRB1 | 3.650736298 | -1.630802014 | 0.316867 | -5.14665 | 2.65E-07 | 1.48E-06 | Down |
| GABRB2 | 2274.132931 | 4.773436185 | 0.297075 | 16.06814 | 4.27E-58 | 7.78E-56 | Up |
| GABRB3 | 327.7397805 | -1.325452472 | 0.172352 | -7.69039 | 1.47E-14 | 1.77E-13 | Down |
| GABRD | 55.89904551 | 2.967809296 | 0.200177 | 14.82593 | 9.96E-50 | 1.21E-47 | Up |
| GABRG1 | 16.34084602 | -1.754056852 | 0.256055 | -6.85031 | 7.37E-12 | 6.85E-11 | Down |
| GABRG3 | 53.14636146 | 1.573308707 | 0.2512 | 6.263163 | 3.77E-10 | 2.97E-09 | Up |
| GABRQ | 9.109760549 | 1.145323858 | 0.231121 | 4.955509 | 7.21E-07 | 3.79E-06 | Up |
| GABRR3 | 2.314198732 | -1.360842252 | 0.407145 | -3.3424 | 0.000831 | 0.00251 | Down |
| GAD1 | 11.90782975 | -1.237690663 | 0.269261 | -4.59661 | 4.29E-06 | 2.01E-05 | Down |
| GAL | 2.261940991 | -1.154849383 | 0.501577 | -2.30244 | 0.02131 | 0.043675 | Down |
| GAL3ST2 | 2.173882062 | -1.499157716 | 0.463013 | -3.23783 | 0.001204 | 0.003516 | Down |
| GALE | 561.6993228 | 2.84025933 | 0.103111 | 27.54554 | 5.00E-167 | 6.85E-163 | Up |
| GALNT5 | 129.5808031 | 2.719273248 | 0.216013 | 12.58848 | 2.44E-36 | 1.37E-34 | Up |
| GALNT7 | 1723.750032 | 2.205831526 | 0.081249 | 27.14891 | 2.61E-162 | 2.68E-158 | Up |
| GALNT8 | 28.46834453 | -1.154199298 | 0.205871 | -5.60643 | 2.07E-08 | 1.33E-07 | Down |
| GALNT9 | 200.7039034 | -1.417051357 | 0.14185 | -9.98979 | 1.69E-23 | 4.08E-22 | Down |
| GALNTL5 | 1.156015478 | -2.318793864 | 0.990175 | -2.3418 | 0.019191 | 0.039887 | Down |
| GALNTL6 | 17.70984168 | 1.506840795 | 0.218042 | 6.910786 | 4.82E-12 | 4.56E-11 | Up |
| GALR3 | 2.120995636 | -1.437032519 | 0.394156 | -3.64585 | 0.000267 | 0.000903 | Down |
| GAP43 | 66.47612985 | 3.85864877 | 0.288839 | 13.35919 | 1.05E-40 | 7.65E-39 | Up |
| GAPDHP15 | 2.022068269 | -1.152662624 | 0.352556 | -3.26945 | 0.001078 | 0.003176 | Down |
| GAPDHP34 | 1.059617013 | -1.773299433 | 0.559964 | -3.16681 | 0.001541 | 0.004378 | Down |
| GAPDHP36 | 0.910386029 | -1.66542326 | 0.582406 | -2.85956 | 0.004242 | 0.010717 | Down |
| GAPDHP54 | 4.777343027 | -1.072914987 | 0.26779 | -4.00656 | 6.16E-05 | 0.000236 | Down |
| GAPDHP55 | 4.471502646 | 2.25719217 | 0.387042 | 5.831908 | 5.48E-09 | 3.77E-08 | Up |
| GAPDHP60 | 12.90930316 | -1.006869889 | 0.211112 | -4.76937 | 1.85E-06 | 9.16E-06 | Down |
| GARNL3 | 275.3099885 | -1.043890787 | 0.08924 | -11.6976 | 1.31E-31 | 5.63E-30 | Down |
| GAS1 | 440.2730245 | -1.203505566 | 0.154859 | -7.7716 | 7.75E-15 | 9.61E-14 | Down |
| GAS2 | 38.28618213 | -1.101580054 | 0.131526 | -8.37536 | 5.51E-17 | 8.08E-16 | Down |
| GAS2L1P1 | 3.746526765 | -3.041564821 | 0.425874 | -7.14193 | 9.20E-13 | 9.35E-12 | Down |
| GAS2L1P2 | 1.931808339 | -3.176424442 | 0.539993 | -5.88235 | 4.04E-09 | 2.82E-08 | Down |
| GATA5 | 8.558227481 | -2.023006506 | 0.342683 | -5.90343 | 3.56E-09 | 2.50E-08 | Down |
| GATM-AS1 | 4.308527669 | -1.920366642 | 0.350179 | -5.48396 | 4.16E-08 | 2.58E-07 | Down |
| GATSL2 | 112.5016053 | 1.274218056 | 0.09928 | 12.83462 | 1.05E-37 | 6.36E-36 | Up |
| GBA3 | 22.7316794 | -1.234675531 | 0.240535 | -5.13304 | 2.85E-07 | 1.58E-06 | Down |
| GBP2 | 2031.268823 | 1.474936642 | 0.146647 | 10.05772 | 8.49E-24 | 2.09E-22 | Up |
| GBP3 | 1823.126117 | 1.270777629 | 0.109198 | 11.63737 | 2.66E-31 | 1.11E-29 | Up |
| GBX1 | 2.630908142 | -3.440849178 | 0.641364 | -5.36489 | 8.10E-08 | 4.83E-07 | Down |
| GCK | 19.58974374 | -1.789609063 | 0.200842 | -8.91052 | 5.08E-19 | 8.78E-18 | Down |
| GCM1 | 2.098011058 | -1.61633798 | 0.539522 | -2.99587 | 0.002737 | 0.007256 | Down |
| GCNT1P3 | 2.615661666 | 1.1075031 | 0.325206 | 3.40554 | 0.00066 | 0.002044 | Up |
| GCNT3 | 49.5167926 | -1.893533839 | 0.196383 | -9.64203 | 5.31E-22 | 1.14E-20 | Down |
| GCNT4 | 174.0966051 | 1.182343226 | 0.150495 | 7.856352 | 3.95E-15 | 5.03E-14 | Up |
| GCNT6 | 2.209285021 | -1.514467577 | 0.325008 | -4.65979 | 3.17E-06 | 1.52E-05 | Down |
| GCOM1 | 63.35682095 | -1.008402286 | 0.158596 | -6.35831 | 2.04E-10 | 1.65E-09 | Down |
| GCSAM | 160.152084 | -1.182054241 | 0.226349 | -5.22226 | 1.77E-07 | 1.01E-06 | Down |
| GCSAML-AS1 | 2.330651171 | -1.552127984 | 0.555483 | -2.79419 | 0.005203 | 0.012843 | Down |
| GCSH | 1160.107099 | -1.163691117 | 0.09828 | -11.8406 | 2.41E-32 | 1.08E-30 | Down |
| GDF10 | 154.2075167 | -2.241913458 | 0.197671 | -11.3416 | 8.16E-30 | 3.08E-28 | Down |
| GDF15 | 376.6259153 | 4.12941205 | 0.217255 | 19.0072 | 1.49E-80 | 6.49E-78 | Up |
| GDF2 | 1.488346204 | -2.308647556 | 0.842016 | -2.74181 | 0.00611 | 0.014781 | Down |
| GDF6 | 30.87772232 | -1.21080607 | 0.177213 | -6.8325 | 8.34E-12 | 7.71E-11 | Down |
| GDF9 | 16.96386184 | -1.048711645 | 0.139681 | -7.50792 | 6.01E-14 | 6.85E-13 | Down |
| GDNF | 3.204617867 | 1.012315151 | 0.381481 | 2.653642 | 0.007963 | 0.018652 | Up |
| GDPD4 | 4.762705635 | -1.238248744 | 0.378839 | -3.26853 | 0.001081 | 0.003184 | Down |
| GFI1B | 2.278408553 | -1.62656386 | 0.338197 | -4.80952 | 1.51E-06 | 7.58E-06 | Down |
| GFRA1 | 544.3967556 | -1.69866733 | 0.188953 | -8.98988 | 2.48E-19 | 4.38E-18 | Down |
| GFRA3 | 8.006269547 | -1.653001596 | 0.27819 | -5.94199 | 2.82E-09 | 2.00E-08 | Down |
| GGCT | 1158.928298 | 2.552575407 | 0.103277 | 24.71582 | 7.23E-135 | 2.70E-131 | Up |
| GGT2 | 73.89130529 | 1.711235583 | 0.166859 | 10.2556 | 1.12E-24 | 2.91E-23 | Up |
| GGT3P | 147.1386729 | 2.033265534 | 0.165139 | 12.31243 | 7.76E-35 | 3.95E-33 | Up |
| GGT6 | 54.9969286 | 2.429356924 | 0.251045 | 9.676966 | 3.78E-22 | 8.23E-21 | Up |
| GGTLC2 | 17.87523993 | -1.116108898 | 0.239906 | -4.65227 | 3.28E-06 | 1.57E-05 | Down |
| GGTLC3 | 27.85068258 | 1.918528567 | 0.262669 | 7.303966 | 2.79E-13 | 2.97E-12 | Up |
| GGTLC5P | 10.45546978 | 1.981120724 | 0.417014 | 4.750733 | 2.03E-06 | 9.99E-06 | Up |
| GHR | 269.0401657 | -1.492119128 | 0.118745 | -12.5657 | 3.26E-36 | 1.82E-34 | Down |
| GHRHR | 1.307123663 | -1.988332563 | 0.464981 | -4.27616 | 1.90E-05 | 8.02E-05 | Down |
| GJA10 | 1.354028612 | -1.917596884 | 0.772666 | -2.48179 | 0.013072 | 0.028762 | Down |
| GJA8 | 1.901212419 | -1.588548431 | 0.679539 | -2.33769 | 0.019404 | 0.040272 | Down |
| GJB1 | 9.814283451 | -1.668685134 | 0.296231 | -5.63305 | 1.77E-08 | 1.15E-07 | Down |
| GJB2 | 101.9167565 | 1.051127573 | 0.159247 | 6.600613 | 4.09E-11 | 3.53E-10 | Up |
| GJB3 | 133.5869597 | 3.921015122 | 0.188482 | 20.80314 | 4.05E-96 | 3.20E-93 | Up |
| GJB4 | 21.3780705 | 1.065099103 | 0.344472 | 3.091975 | 0.001988 | 0.005489 | Up |
| GJB6 | 121.2605755 | -1.695279094 | 0.184339 | -9.19653 | 3.70E-20 | 6.97E-19 | Down |
| GJB7 | 7.532125104 | -1.546970709 | 0.278702 | -5.55063 | 2.85E-08 | 1.80E-07 | Down |
| GK2 | 1.812730796 | -2.081551355 | 0.692438 | -3.00612 | 0.002646 | 0.007046 | Down |
| GKN2 | 1.212115895 | -2.090311472 | 0.824105 | -2.53646 | 0.011198 | 0.025109 | Down |
| GLDC | 176.1069954 | -2.820576932 | 0.229174 | -12.3076 | 8.25E-35 | 4.18E-33 | Down |
| GLDCP1 | 1.829235795 | -1.780225015 | 0.578202 | -3.0789 | 0.002078 | 0.005701 | Down |
| GLDN | 367.5713122 | 3.074359895 | 0.270579 | 11.36215 | 6.45E-30 | 2.45E-28 | Up |
| GLI1 | 90.04492614 | -1.065340003 | 0.149931 | -7.10553 | 1.20E-12 | 1.20E-11 | Down |
| GLIS2-AS1 | 6.974972886 | -1.301253503 | 0.170723 | -7.62203 | 2.50E-14 | 2.95E-13 | Down |
| GLP1R | 47.4058578 | 2.061360345 | 0.283763 | 7.264368 | 3.75E-13 | 3.93E-12 | Up |
| GLP2R | 11.98404026 | -1.700757779 | 0.277986 | -6.11815 | 9.47E-10 | 7.13E-09 | Down |
| GLRA3 | 4.654958218 | -1.238321524 | 0.442426 | -2.79893 | 0.005127 | 0.012672 | Down |
| GLRA4 | 1.491878599 | -1.608466868 | 0.616195 | -2.61032 | 0.009046 | 0.02087 | Down |
| GLRB | 100.4467669 | 1.723078103 | 0.127572 | 13.50672 | 1.43E-41 | 1.10E-39 | Up |
| GLS2 | 143.5884148 | 1.593470001 | 0.111892 | 14.24112 | 5.09E-46 | 5.18E-44 | Up |
| GLT1D1 | 132.5853167 | 2.45318189 | 0.133911 | 18.31943 | 5.79E-75 | 2.09E-72 | Up |
| GLT8D2 | 483.0415518 | -1.250133397 | 0.122076 | -10.2406 | 1.30E-24 | 3.38E-23 | Down |
| GLUD1P9 | 1.007520079 | -2.082797147 | 0.827224 | -2.51781 | 0.011809 | 0.026326 | Down |
| GLULP4 | 3.687543354 | -2.206194014 | 0.316694 | -6.96633 | 3.25E-12 | 3.12E-11 | Down |
| GLYAT | 5.557755105 | -2.895523574 | 0.483216 | -5.9922 | 2.07E-09 | 1.50E-08 | Down |
| GMCL1P1 | 1.354000576 | -1.580389771 | 0.651404 | -2.42613 | 0.015261 | 0.032791 | Down |
| GNA14 | 371.0709013 | -1.692629066 | 0.109224 | -15.4969 | 3.64E-54 | 5.47E-52 | Down |
| GNA14-AS1 | 1.652294303 | -1.326025079 | 0.374872 | -3.53728 | 0.000404 | 0.001314 | Down |
| GNA15 | 113.0727541 | 1.191081773 | 0.147403 | 8.08047 | 6.45E-16 | 8.67E-15 | Up |
| GNAT1 | 2.122288872 | -2.773780601 | 0.726834 | -3.81625 | 0.000135 | 0.000486 | Down |
| GNAT3 | 1.125200298 | -1.526658662 | 0.371665 | -4.10762 | 4.00E-05 | 0.000159 | Down |
| GNGT1 | 1.212606532 | -2.537185502 | 1.082338 | -2.34417 | 0.019069 | 0.039661 | Down |
| GNL2P1 | 4.084853254 | -2.00718676 | 0.388257 | -5.16974 | 2.34E-07 | 1.32E-06 | Down |
| GNLY | 147.9452273 | 1.708110656 | 0.219259 | 7.790381 | 6.68E-15 | 8.34E-14 | Up |
| GNMT | 16.26801172 | -1.031206417 | 0.133079 | -7.74885 | 9.27E-15 | 1.14E-13 | Down |
| GOLGA2P9 | 0.995060696 | -2.360407959 | 1.026434 | -2.29962 | 0.02147 | 0.043947 | Down |
| GOLGA6A | 9.782303922 | -2.061634246 | 0.39787 | -5.18168 | 2.20E-07 | 1.24E-06 | Down |
| GOLGA6B | 3.876008022 | -1.289068335 | 0.56421 | -2.28473 | 0.022329 | 0.045445 | Down |
| GOLGA6L17P | 1.484795861 | -1.858972546 | 0.572378 | -3.24781 | 0.001163 | 0.003403 | Down |
| GOLGA6L2 | 3.056353501 | -1.833952663 | 0.662561 | -2.76798 | 0.005641 | 0.013781 | Down |
| GOLGA6L3 | 3.409079101 | -1.204892212 | 0.507581 | -2.37379 | 0.017606 | 0.037056 | Down |
| GOLGA6L6 | 5.950566974 | -1.684052443 | 0.661597 | -2.54543 | 0.010914 | 0.024546 | Down |
| GOLGA6L7P | 2.678026604 | -1.225332583 | 0.423349 | -2.89438 | 0.003799 | 0.009722 | Down |
| GOLGA7B | 91.30372261 | 1.563123977 | 0.187471 | 8.337936 | 7.56E-17 | 1.10E-15 | Up |
| GOLGA8DP | 1.88376201 | -1.96625072 | 0.407646 | -4.82343 | 1.41E-06 | 7.11E-06 | Down |
| GOLGA8EP | 1.493907787 | -1.780627457 | 0.747897 | -2.38085 | 0.017273 | 0.036449 | Down |
| GOLGA8IP | 26.21894629 | -1.729066022 | 0.181259 | -9.53918 | 1.44E-21 | 3.02E-20 | Down |
| GOLGA8J | 7.994800768 | -1.452908259 | 0.30947 | -4.69482 | 2.67E-06 | 1.29E-05 | Down |
| GOLGA8K | 49.14947051 | -1.097501921 | 0.152124 | -7.21453 | 5.41E-13 | 5.60E-12 | Down |
| GOLGA8M | 126.5641788 | -1.833532653 | 0.166652 | -11.0022 | 3.73E-28 | 1.26E-26 | Down |
| GOLGA8N | 1700.792403 | -1.28500541 | 0.113892 | -11.2827 | 1.60E-29 | 5.94E-28 | Down |
| GOLGA8O | 148.1563514 | -1.251987871 | 0.204031 | -6.13626 | 8.45E-10 | 6.40E-09 | Down |
| GOLGA8Q | 349.4720324 | -1.827646554 | 0.154546 | -11.8259 | 2.87E-32 | 1.28E-30 | Down |
| GOLGA8R | 266.4726896 | -1.146600288 | 0.122081 | -9.39215 | 5.88E-21 | 1.17E-19 | Down |
| GOLGA8S | 48.56481414 | -1.355045338 | 0.151934 | -8.91863 | 4.72E-19 | 8.17E-18 | Down |
| GOLGA8T | 25.738209 | -1.109564448 | 0.170481 | -6.50845 | 7.59E-11 | 6.39E-10 | Down |
| GOLGA8VP | 1.596048025 | -1.359672115 | 0.324467 | -4.19048 | 2.78E-05 | 0.000114 | Down |
| GOLT1A | 75.63488767 | 3.457501571 | 0.15323 | 22.56415 | 9.75E-113 | 1.33E-109 | Up |
| GOT2P2 | 1.620767675 | -1.158179765 | 0.425011 | -2.72506 | 0.006429 | 0.015474 | Down |
| GOT2P3 | 1.966062206 | -1.14998729 | 0.43758 | -2.62806 | 0.008587 | 0.019943 | Down |
| GP2 | 1.916564516 | -2.24734417 | 0.763578 | -2.94317 | 0.003249 | 0.008464 | Down |
| GP5 | 21.90481611 | -1.450191343 | 0.179334 | -8.08653 | 6.14E-16 | 8.26E-15 | Down |
| GP6 | 12.1221508 | -1.14150901 | 0.257201 | -4.4382 | 9.07E-06 | 4.04E-05 | Down |
| GP9 | 2.002794655 | -1.251401816 | 0.343722 | -3.64073 | 0.000272 | 0.000919 | Down |
| GPAA1P1 | 6.53131852 | 1.5725395 | 0.307651 | 5.111444 | 3.20E-07 | 1.76E-06 | Up |
| GPC3 | 305.7892683 | -2.337690282 | 0.192128 | -12.1674 | 4.64E-34 | 2.25E-32 | Down |
| GPD1 | 131.7399285 | -1.057672228 | 0.225185 | -4.6969 | 2.64E-06 | 1.28E-05 | Down |
| GPIHBP1 | 59.1656187 | -1.13083332 | 0.137509 | -8.22371 | 1.97E-16 | 2.76E-15 | Down |
| GPM6A | 332.5592688 | -2.107435915 | 0.175676 | -11.9962 | 3.72E-33 | 1.73E-31 | Down |
| GPNMB | 3843.898146 | 1.029326822 | 0.174926 | 5.884344 | 4.00E-09 | 2.79E-08 | Up |
| GPR115 | 15.13758172 | 2.689324669 | 0.376948 | 7.134477 | 9.72E-13 | 9.85E-12 | Up |
| GPR12 | 10.67049872 | -2.144016913 | 0.346455 | -6.18845 | 6.08E-10 | 4.67E-09 | Down |
| GPR123 | 4.124410057 | -1.292813398 | 0.341091 | -3.79023 | 0.000151 | 0.000536 | Down |
| GPR125 | 1680.259621 | -1.3492679 | 0.088291 | -15.2821 | 1.01E-52 | 1.39E-50 | Down |
| GPR128 | 2.040659234 | -2.226458521 | 0.725983 | -3.06682 | 0.002164 | 0.005912 | Down |
| GPR139 | 2.109866138 | -2.395969132 | 0.845436 | -2.834 | 0.004597 | 0.011511 | Down |
| GPR141 | 23.57313344 | 1.047786822 | 0.20598 | 5.086833 | 3.64E-07 | 1.99E-06 | Up |
| GPR142 | 9.210601525 | -2.124325287 | 0.218518 | -9.72149 | 2.44E-22 | 5.39E-21 | Down |
| GPR149 | 1.720181309 | -2.010549184 | 0.699896 | -2.87264 | 0.004071 | 0.010329 | Down |
| GPR151 | 7.002788513 | -1.091138675 | 0.248006 | -4.39965 | 1.08E-05 | 4.76E-05 | Down |
| GPR152 | 3.024383782 | -1.144683829 | 0.398249 | -2.87429 | 0.004049 | 0.010284 | Down |
| GPR158 | 36.65286934 | -1.735140568 | 0.19562 | -8.86993 | 7.32E-19 | 1.25E-17 | Down |
| GPR158-AS1 | 1.528529809 | -2.4725919 | 0.9876 | -2.50364 | 0.012292 | 0.027271 | Down |
| GPR179 | 27.47803194 | -1.849497681 | 0.33699 | -5.48828 | 4.06E-08 | 2.52E-07 | Down |
| GPR182 | 42.0471452 | -1.580325449 | 0.151381 | -10.4394 | 1.64E-25 | 4.54E-24 | Down |
| GPR27 | 278.5567346 | 1.163187192 | 0.110584 | 10.51856 | 7.09E-26 | 2.02E-24 | Up |
| GPR32 | 1.462486149 | -1.864992633 | 0.596153 | -3.12838 | 0.001758 | 0.004924 | Down |
| GPR37 | 13.91412905 | 1.278120235 | 0.284761 | 4.488402 | 7.18E-06 | 3.25E-05 | Up |
| GPR37L1 | 46.06899985 | -1.853343504 | 0.142628 | -12.9942 | 1.32E-38 | 8.41E-37 | Down |
| GPR42 | 2.417927877 | -1.481455534 | 0.370609 | -3.99736 | 6.41E-05 | 0.000244 | Down |
| GPR45 | 4.782368535 | -1.107354716 | 0.246466 | -4.49292 | 7.03E-06 | 3.18E-05 | Down |
| GPR50-AS1 | 1.534332096 | -3.446636871 | 1.451476 | -2.37457 | 0.017569 | 0.036988 | Down |
| GPR6 | 1.16829335 | -2.600455201 | 0.734703 | -3.53946 | 0.000401 | 0.001305 | Down |
| GPR64 | 220.0827617 | 1.152892908 | 0.128212 | 8.992118 | 2.43E-19 | 4.30E-18 | Up |
| GPR83 | 226.4179844 | -1.656119034 | 0.160117 | -10.3432 | 4.49E-25 | 1.21E-23 | Down |
| GPR84 | 22.37620404 | 1.128345469 | 0.215486 | 5.236276 | 1.64E-07 | 9.41E-07 | Up |
| GPR87 | 9.656216544 | 1.979709464 | 0.256489 | 7.718505 | 1.18E-14 | 1.44E-13 | Up |
| GPR97 | 24.16706677 | -1.882728541 | 0.171655 | -10.9681 | 5.44E-28 | 1.81E-26 | Down |
| GPR98 | 1494.624515 | -2.137443666 | 0.160515 | -13.3162 | 1.86E-40 | 1.33E-38 | Down |
| GPRC5B | 1923.432066 | 1.20526301 | 0.078603 | 15.33355 | 4.56E-53 | 6.46E-51 | Up |
| GPRC6A | 1.830566901 | -2.180241479 | 0.695706 | -3.13385 | 0.001725 | 0.004843 | Down |
| GPS2P2 | 1.858730705 | -2.395335608 | 0.771302 | -3.10558 | 0.001899 | 0.00527 | Down |
| GPX1P2 | 1.222595828 | -2.007504188 | 0.653607 | -3.07142 | 0.00213 | 0.005831 | Down |
| GPX3 | 37617.00041 | -1.15425405 | 0.125227 | -9.21727 | 3.05E-20 | 5.79E-19 | Down |
| GPX6 | 1.378328456 | -2.949891659 | 1.047932 | -2.81497 | 0.004878 | 0.012121 | Down |
| GRAMD2 | 172.8561528 | -1.704476785 | 0.123303 | -13.8235 | 1.84E-43 | 1.59E-41 | Down |
| GRAPL | 40.62799855 | -1.107127495 | 0.234553 | -4.72016 | 2.36E-06 | 1.15E-05 | Down |
| GRB14 | 70.12747283 | -1.238569644 | 0.152495 | -8.12204 | 4.58E-16 | 6.24E-15 | Down |
| GRB7 | 439.7165921 | 2.461720425 | 0.114817 | 21.44039 | 5.61E-102 | 4.90E-99 | Up |
| GRHL1 | 186.8108215 | 1.167509018 | 0.098909 | 11.80381 | 3.73E-32 | 1.65E-30 | Up |
| GRHL3 | 46.96793992 | 2.913679182 | 0.228733 | 12.73834 | 3.62E-37 | 2.13E-35 | Up |
| GRIA1 | 24.8537267 | -2.330916812 | 0.297681 | -7.83025 | 4.87E-15 | 6.15E-14 | Down |
| GRIA4 | 11.86432652 | -1.586814722 | 0.301926 | -5.25564 | 1.48E-07 | 8.53E-07 | Down |
| GRIFIN | 0.540144914 | -1.513461047 | 0.521145 | -2.9041 | 0.003683 | 0.00946 | Down |
| GRIK2 | 141.069594 | -1.003341916 | 0.17865 | -5.61625 | 1.95E-08 | 1.26E-07 | Down |
| GRIK4 | 100.3465644 | -2.00607535 | 0.162566 | -12.3401 | 5.51E-35 | 2.86E-33 | Down |
| GRIN2B | 49.10867111 | -1.28968967 | 0.294512 | -4.37907 | 1.19E-05 | 5.20E-05 | Down |
| GRIN2C | 534.1580992 | -2.123549903 | 0.185631 | -11.4397 | 2.65E-30 | 1.03E-28 | Down |
| GRIN3A | 54.18658169 | -1.084849346 | 0.167921 | -6.46049 | 1.04E-10 | 8.68E-10 | Down |
| GRK5-IT1 | 2.286424767 | -1.358679494 | 0.319861 | -4.24772 | 2.16E-05 | 9.02E-05 | Down |
| GRM1 | 20.90172537 | -1.175094851 | 0.192981 | -6.08916 | 1.14E-09 | 8.48E-09 | Down |
| GRM3 | 7.771495746 | -1.200235763 | 0.290457 | -4.13223 | 3.59E-05 | 0.000144 | Down |
| GRM4 | 54.26340422 | 1.309486002 | 0.343873 | 3.808049 | 0.00014 | 0.000502 | Up |
| GRM5-AS1 | 5.108008791 | 2.532411577 | 0.46506 | 5.445344 | 5.17E-08 | 3.16E-07 | Up |
| GRP | 9.603117484 | 2.087853484 | 0.423878 | 4.925601 | 8.41E-07 | 4.37E-06 | Up |
| GRTP1-AS1 | 2.39602926 | -1.380228456 | 0.273947 | -5.0383 | 4.70E-07 | 2.54E-06 | Down |
| GS1-114I9.1 | 52.02169892 | 2.380608978 | 0.211737 | 11.24325 | 2.50E-29 | 9.16E-28 | Up |
| GS1-124K5.13 | 7.684083373 | -1.100527341 | 0.260533 | -4.22415 | 2.40E-05 | 9.93E-05 | Down |
| GS1-204I12.4 | 1.058405048 | -1.390093366 | 0.487694 | -2.85034 | 0.004367 | 0.010999 | Down |
| GS1-24F4.2 | 2.942596777 | -1.674228189 | 0.560765 | -2.98561 | 0.00283 | 0.007484 | Down |
| GS1-279B7.2 | 5.927183704 | -1.329136719 | 0.268339 | -4.95319 | 7.30E-07 | 3.83E-06 | Down |
| GS1-309P15.2 | 1.384515179 | -2.013756871 | 0.886339 | -2.27199 | 0.023087 | 0.046749 | Down |
| GS1-421I3.2 | 0.641450251 | -1.840898681 | 0.611068 | -3.01259 | 0.00259 | 0.006916 | Down |
| GSC | 23.96951925 | -1.450046576 | 0.148644 | -9.75519 | 1.75E-22 | 3.92E-21 | Down |
| GSDMA | 13.18955805 | 1.178208976 | 0.28372 | 4.152722 | 3.29E-05 | 0.000133 | Up |
| GSG1 | 8.377336657 | -1.41041479 | 0.26159 | -5.3917 | 6.98E-08 | 4.20E-07 | Down |
| GSTM3 | 1521.723351 | -1.459462742 | 0.117223 | -12.4504 | 1.39E-35 | 7.49E-34 | Down |
| GSTM5 | 97.72700027 | -1.794118248 | 0.175835 | -10.2034 | 1.91E-24 | 4.90E-23 | Down |
| GTF2F2P2 | 0.796329728 | -1.881400273 | 0.751637 | -2.50307 | 0.012312 | 0.027307 | Down |
| GUCA1A | 2.118214408 | -1.474620081 | 0.61714 | -2.38944 | 0.016874 | 0.035732 | Down |
| GUCY1B2 | 2.389221355 | -1.29183489 | 0.409552 | -3.15426 | 0.001609 | 0.00455 | Down |
| GUSBP3 | 89.48400552 | -1.022741642 | 0.171809 | -5.9528 | 2.64E-09 | 1.88E-08 | Down |
| GUSBP9 | 284.4157626 | -1.134900685 | 0.13895 | -8.16768 | 3.14E-16 | 4.33E-15 | Down |
| GXYLT1P3 | 11.84592346 | -1.89682682 | 0.242539 | -7.82072 | 5.25E-15 | 6.63E-14 | Down |
| GXYLT1P5 | 6.653649352 | -1.041124679 | 0.30808 | -3.3794 | 0.000726 | 0.002225 | Down |
| GXYLT1P6 | 1.883004225 | -2.189864164 | 0.544429 | -4.02232 | 5.76E-05 | 0.000222 | Down |
| GYPA | 2.676261997 | -2.071189427 | 0.465886 | -4.4457 | 8.76E-06 | 3.91E-05 | Down |
| GYPB | 1.325818434 | -1.606343418 | 0.508196 | -3.16087 | 0.001573 | 0.004458 | Down |
| GYS2 | 5.040862063 | -1.807903954 | 0.268699 | -6.72835 | 1.72E-11 | 1.53E-10 | Down |
| H1FNT | 3.239751867 | -1.626973159 | 0.434084 | -3.74806 | 0.000178 | 0.000626 | Down |
| H2AFB1 | 1.746004571 | -1.588653608 | 0.620734 | -2.55931 | 0.010488 | 0.023706 | Down |
| HABP2 | 3.153406697 | -2.349607436 | 0.562387 | -4.17792 | 2.94E-05 | 0.00012 | Down |
| HAGLR | 82.87454953 | 1.640021774 | 0.193832 | 8.46103 | 2.65E-17 | 3.99E-16 | Up |
| HAGLROS | 6.653775528 | 2.651422369 | 0.249576 | 10.62372 | 2.31E-26 | 6.79E-25 | Up |
| HAL | 11.44911887 | -1.580937022 | 0.246993 | -6.40074 | 1.55E-10 | 1.26E-09 | Down |
| HAND1 | 1.745839209 | -1.921730131 | 0.734198 | -2.61745 | 0.008859 | 0.020496 | Down |
| HAND2 | 35.85151706 | -1.227502715 | 0.193574 | -6.34127 | 2.28E-10 | 1.83E-09 | Down |
| HAND2-AS1 | 36.81596449 | -1.26063018 | 0.205243 | -6.14212 | 8.14E-10 | 6.18E-09 | Down |
| HAO2 | 2.84485927 | -1.216809356 | 0.456791 | -2.66382 | 0.007726 | 0.018166 | Down |
| HAP1 | 70.51712821 | -2.368507135 | 0.179523 | -13.1933 | 9.58E-40 | 6.63E-38 | Down |
| HAPLN1 | 41.13266946 | 2.311692731 | 0.342061 | 6.758132 | 1.40E-11 | 1.26E-10 | Up |
| HAPLN2 | 4.245205568 | -1.344490246 | 0.321811 | -4.17789 | 2.94E-05 | 0.00012 | Down |
| HAS2 | 120.5003411 | 1.119321627 | 0.172746 | 6.479596 | 9.20E-11 | 7.68E-10 | Up |
| HBA1 | 1536.512243 | -2.237985437 | 0.210206 | -10.6466 | 1.81E-26 | 5.35E-25 | Down |
| HBA2 | 2673.342542 | -2.203202077 | 0.210941 | -10.4447 | 1.55E-25 | 4.31E-24 | Down |
| HBB | 4706.269811 | -2.159183529 | 0.205466 | -10.5087 | 7.87E-26 | 2.23E-24 | Down |
| HBD | 1.32836977 | -1.183283488 | 0.373728 | -3.16617 | 0.001545 | 0.004387 | Down |
| HBG2 | 57.98731757 | -2.545661888 | 0.336065 | -7.57491 | 3.59E-14 | 4.18E-13 | Down |
| HBM | 1.100938446 | -2.141280922 | 0.436845 | -4.9017 | 9.50E-07 | 4.91E-06 | Down |
| HCCAT5 | 1.590927954 | -1.21387469 | 0.500135 | -2.42709 | 0.01522 | 0.032718 | Down |
| HCG14 | 1.455879725 | -1.391705511 | 0.289188 | -4.81245 | 1.49E-06 | 7.49E-06 | Down |
| HCG22 | 56.55927105 | 1.206210274 | 0.243545 | 4.952722 | 7.32E-07 | 3.84E-06 | Up |
| HCG9 | 1.337999375 | -1.201563364 | 0.310967 | -3.86395 | 0.000112 | 0.000407 | Down |
| HCN4 | 70.0710784 | 2.006907316 | 0.32242 | 6.224515 | 4.83E-10 | 3.75E-09 | Up |
| HDAC1P2 | 11.21690824 | -1.124913352 | 0.237738 | -4.73173 | 2.23E-06 | 1.09E-05 | Down |
| HEATR4 | 19.28710484 | -1.304939035 | 0.178838 | -7.29676 | 2.95E-13 | 3.13E-12 | Down |
| HECW1 | 77.09127262 | -1.373936176 | 0.180739 | -7.60178 | 2.92E-14 | 3.42E-13 | Down |
| HECW1-IT1 | 2.245696645 | -2.148788251 | 0.52993 | -4.05485 | 5.02E-05 | 0.000196 | Down |
| HEMGN | 13.94044704 | -1.916474449 | 0.2052 | -9.33955 | 9.67E-21 | 1.91E-19 | Down |
| HEPACAM | 10.70880679 | -1.758174033 | 0.318774 | -5.51542 | 3.48E-08 | 2.18E-07 | Down |
| HEPACAM2 | 2.175764728 | -1.166357929 | 0.384599 | -3.03266 | 0.002424 | 0.006529 | Down |
| HEPHL1 | 24.00651288 | -1.20917483 | 0.204181 | -5.92208 | 3.18E-09 | 2.25E-08 | Down |
| HEPN1 | 3.112799891 | -1.170291195 | 0.3665 | -3.19315 | 0.001407 | 0.004034 | Down |
| HERC2P5 | 10.13453164 | -1.28974394 | 0.338664 | -3.80832 | 0.00014 | 0.000501 | Down |
| HERC2P8 | 8.755853872 | -1.221847453 | 0.349817 | -3.49282 | 0.000478 | 0.001528 | Down |
| HES2 | 28.23124854 | 1.279155274 | 0.336076 | 3.806154 | 0.000141 | 0.000505 | Up |
| HES6 | 121.3197549 | 1.811629811 | 0.13336 | 13.58452 | 4.95E-42 | 3.91E-40 | Up |
| HEXDC-IT1 | 8.591555355 | -1.03633543 | 0.257903 | -4.01831 | 5.86E-05 | 0.000225 | Down |
| HEY2 | 331.0885714 | 1.804208673 | 0.13253 | 13.6136 | 3.32E-42 | 2.66E-40 | Up |
| HFM1 | 179.2102322 | -1.671030681 | 0.147083 | -11.3611 | 6.53E-30 | 2.48E-28 | Down |
| HGD | 567.4734609 | -2.223120855 | 0.154657 | -14.3745 | 7.48E-47 | 8.04E-45 | Down |
| HHATL-AS1 | 2.173720744 | -1.692844031 | 0.305544 | -5.54043 | 3.02E-08 | 1.90E-07 | Down |
| HHIP | 75.16367456 | -1.566704525 | 0.220239 | -7.11365 | 1.13E-12 | 1.14E-11 | Down |
| HHIP-AS1 | 8.499320864 | -1.220863936 | 0.202705 | -6.02287 | 1.71E-09 | 1.26E-08 | Down |
| HHIPL2 | 37.17642982 | -2.412197611 | 0.301721 | -7.99479 | 1.30E-15 | 1.71E-14 | Down |
| HID1-AS1 | 7.026378716 | -1.215268291 | 0.172343 | -7.05146 | 1.77E-12 | 1.75E-11 | Down |
| HIF3A | 104.8621379 | -2.156791789 | 0.179721 | -12.0008 | 3.52E-33 | 1.64E-31 | Down |
| HIGD1AP11 | 0.617401974 | -1.465479822 | 0.55747 | -2.62881 | 0.008569 | 0.019905 | Down |
| HIGD2B | 2.787446235 | -1.991307095 | 0.333622 | -5.96875 | 2.39E-09 | 1.72E-08 | Down |
| HIPK1-AS1 | 12.50587574 | -1.353654852 | 0.200741 | -6.7433 | 1.55E-11 | 1.39E-10 | Down |
| HIRAP1 | 1.080062952 | 2.428923463 | 0.86172 | 2.818691 | 0.004822 | 0.012007 | Up |
| HIST3H2A | 68.58309549 | 1.257516058 | 0.165924 | 7.578868 | 3.49E-14 | 4.06E-13 | Up |
| HK2 | 1325.539724 | 1.283106229 | 0.124829 | 10.27891 | 8.77E-25 | 2.31E-23 | Up |
| HK3 | 107.2759045 | 1.286579588 | 0.212025 | 6.068055 | 1.29E-09 | 9.61E-09 | Up |
| HKDC1 | 242.7192311 | -1.768422523 | 0.145002 | -12.1959 | 3.27E-34 | 1.60E-32 | Down |
| HLA-DQA1 | 3015.016827 | 1.02199343 | 0.242868 | 4.208016 | 2.58E-05 | 0.000106 | Up |
| HLA-DQA2 | 591.12049 | 1.179440269 | 0.340675 | 3.462071 | 0.000536 | 0.001694 | Up |
| HLA-DQB1 | 3124.571348 | 1.315864147 | 0.24427 | 5.386935 | 7.17E-08 | 4.30E-07 | Up |
| HLA-DQB2 | 300.5294836 | 2.239797802 | 0.254276 | 8.808517 | 1.27E-18 | 2.12E-17 | Up |
| HLA-DRB1 | 6225.463264 | 1.022444719 | 0.187549 | 5.451607 | 4.99E-08 | 3.06E-07 | Up |
| HLA-G | 108.6631065 | 3.207761083 | 0.278016 | 11.53803 | 8.48E-31 | 3.43E-29 | Up |
| HLF | 947.102309 | -1.396436927 | 0.139294 | -10.0251 | 1.18E-23 | 2.89E-22 | Down |
| HM13-AS1 | 2.250651156 | -1.724869147 | 0.284868 | -6.05498 | 1.40E-09 | 1.04E-08 | Down |
| HMCN2 | 400.9258994 | -1.519631108 | 0.161375 | -9.41676 | 4.65E-21 | 9.36E-20 | Down |
| HMGA1P1 | 1.387631484 | -3.14548033 | 1.132377 | -2.77777 | 0.005473 | 0.013423 | Down |
| HMGA2 | 756.7595884 | 3.896801464 | 0.25802 | 15.10271 | 1.55E-51 | 2.04E-49 | Up |
| HMGB1P1 | 3.656257548 | -1.062917409 | 0.274649 | -3.87009 | 0.000109 | 0.000397 | Down |
| HMGB1P16 | 0.905319627 | -2.194945404 | 0.491912 | -4.46207 | 8.12E-06 | 3.64E-05 | Down |
| HMGB1P29 | 0.640857108 | 1.697865444 | 0.491355 | 3.455479 | 0.000549 | 0.001732 | Up |
| HMGB1P37 | 1.128463308 | -1.4452459 | 0.589665 | -2.45096 | 0.014248 | 0.030924 | Down |
| HMGB1P39 | 1.532697788 | -1.318163734 | 0.469678 | -2.80652 | 0.005008 | 0.012404 | Down |
| HMGB2P1 | 1.423786327 | -1.221362163 | 0.485945 | -2.51338 | 0.011958 | 0.026619 | Down |
| HMGB3P10 | 1.159068793 | -1.064869255 | 0.387334 | -2.74922 | 0.005974 | 0.014494 | Down |
| HMGB3P18 | 0.894720064 | -1.807280556 | 0.674315 | -2.68017 | 0.007358 | 0.017404 | Down |
| HMGB3P2 | 1.018646157 | -1.518249241 | 0.622015 | -2.44086 | 0.014652 | 0.031683 | Down |
| HMGCLL1 | 26.84498364 | -1.112671111 | 0.162634 | -6.84158 | 7.83E-12 | 7.26E-11 | Down |
| HMGCS2 | 8.220062947 | -2.866249813 | 0.349498 | -8.20104 | 2.38E-16 | 3.32E-15 | Down |
| HMGN2P17 | 1.427508773 | -2.458004229 | 0.831881 | -2.95475 | 0.003129 | 0.00819 | Down |
| HMGN2P30 | 0.80174872 | -1.468588045 | 0.458431 | -3.20351 | 0.001358 | 0.003905 | Down |
| HMGN2P40 | 0.994148348 | -1.112728377 | 0.398286 | -2.79379 | 0.005209 | 0.012856 | Down |
| HMP19 | 6.550097452 | -1.551209333 | 0.431671 | -3.5935 | 0.000326 | 0.001083 | Down |
| HMX1 | 1.803726278 | -1.802267563 | 0.575357 | -3.13243 | 0.001734 | 0.004862 | Down |
| HNRNPA1P13 | 1.792181267 | -2.726468942 | 1.065952 | -2.55778 | 0.010534 | 0.0238 | Down |
| HNRNPA1P2 | 3.189180248 | -1.75523341 | 0.406788 | -4.31486 | 1.60E-05 | 6.82E-05 | Down |
| HNRNPA1P20 | 2.059879315 | -2.750208144 | 0.974795 | -2.82132 | 0.004783 | 0.011925 | Down |
| HNRNPA1P22 | 3.176618148 | -2.432237708 | 0.448412 | -5.42411 | 5.82E-08 | 3.53E-07 | Down |
| HNRNPA1P25 | 3.73595684 | -1.730718508 | 0.382541 | -4.52427 | 6.06E-06 | 2.77E-05 | Down |
| HNRNPA1P27 | 18.36723035 | 2.95658812 | 0.26586 | 11.12084 | 9.93E-29 | 3.50E-27 | Up |
| HNRNPA1P28 | 1.451725708 | -2.740780301 | 0.953578 | -2.87421 | 0.00405 | 0.010286 | Down |
| HNRNPA1P31 | 1.748313689 | -2.010879226 | 0.827806 | -2.42917 | 0.015134 | 0.032564 | Down |
| HNRNPA1P32 | 1.90099882 | -2.151340548 | 0.925256 | -2.32513 | 0.020065 | 0.04146 | Down |
| HNRNPA1P34 | 3.969833546 | -1.331939455 | 0.392292 | -3.39528 | 0.000686 | 0.002114 | Down |
| HNRNPA1P39 | 1.934886046 | -2.297241913 | 0.986443 | -2.32881 | 0.019869 | 0.041107 | Down |
| HNRNPA1P4 | 1.951843276 | -1.773443772 | 0.71007 | -2.49756 | 0.012505 | 0.027677 | Down |
| HNRNPA1P41 | 1.583146183 | -1.410580564 | 0.494641 | -2.85173 | 0.004348 | 0.010955 | Down |
| HNRNPA1P42 | 1.608012423 | -1.839149336 | 0.725692 | -2.53434 | 0.011266 | 0.025241 | Down |
| HNRNPA1P46 | 1.482853594 | -2.494342874 | 0.913465 | -2.73064 | 0.006321 | 0.015245 | Down |
| HNRNPA1P5 | 3.690588337 | -1.036898465 | 0.32954 | -3.14651 | 0.001652 | 0.004661 | Down |
| HNRNPA1P51 | 2.183071506 | -2.151409356 | 0.805216 | -2.67184 | 0.007544 | 0.017785 | Down |
| HNRNPA1P52 | 1.746496539 | -1.9286211 | 0.799117 | -2.41344 | 0.015803 | 0.033763 | Down |
| HNRNPA1P56 | 2.216220443 | -2.131689375 | 0.875629 | -2.43447 | 0.014914 | 0.032163 | Down |
| HNRNPA1P58 | 1.415519896 | -2.732924099 | 0.958086 | -2.85248 | 0.004338 | 0.010933 | Down |
| HNRNPA1P6 | 2.07452655 | -1.790086375 | 0.545924 | -3.279 | 0.001042 | 0.003082 | Down |
| HNRNPA1P61 | 2.1724364 | -1.802282115 | 0.463239 | -3.89061 | 1.00E-04 | 0.000368 | Down |
| HNRNPA1P64 | 2.844518091 | -2.79019577 | 1.027268 | -2.71613 | 0.006605 | 0.015844 | Down |
| HNRNPA1P65 | 1.191275062 | -2.598452602 | 0.945617 | -2.74789 | 0.005998 | 0.014545 | Down |
| HNRNPA1P66 | 1.891437717 | -1.5624581 | 0.441785 | -3.53669 | 0.000405 | 0.001317 | Down |
| HNRNPA1P67 | 1.830767852 | -2.266057562 | 0.737648 | -3.072 | 0.002126 | 0.005822 | Down |
| HNRNPA1P69 | 9.922517312 | -2.807399294 | 0.324366 | -8.65503 | 4.93E-18 | 7.85E-17 | Down |
| HNRNPA1P72 | 2.334988708 | -2.12113381 | 0.463852 | -4.57287 | 4.81E-06 | 2.24E-05 | Down |
| HNRNPA1P73 | 1.936982837 | -2.845602711 | 1.079472 | -2.63611 | 0.008386 | 0.019529 | Down |
| HNRNPA1P74 | 2.120709409 | -2.542081663 | 0.582568 | -4.36358 | 1.28E-05 | 5.55E-05 | Down |
| HNRNPA1P9 | 2.87066709 | -1.315233594 | 0.439794 | -2.99057 | 0.002785 | 0.007374 | Down |
| HNRNPA3P12 | 1.931563308 | -1.219522343 | 0.453554 | -2.68881 | 0.007171 | 0.017018 | Down |
| HNRNPA3P14 | 2.468082062 | -1.553421877 | 0.50815 | -3.05701 | 0.002236 | 0.006081 | Down |
| HNRNPA3P15 | 6.789452734 | -1.303830167 | 0.383108 | -3.4033 | 0.000666 | 0.002059 | Down |
| HNRNPA3P16 | 4.219795321 | -3.128141116 | 0.620704 | -5.03967 | 4.66E-07 | 2.52E-06 | Down |
| HNRNPA3P2 | 1.936957787 | -1.189229883 | 0.460627 | -2.58177 | 0.00983 | 0.022426 | Down |
| HNRNPA3P4 | 1.108288712 | -2.469106078 | 0.96775 | -2.55139 | 0.010729 | 0.024179 | Down |
| HNRNPA3P7 | 7.869212767 | -1.218746409 | 0.243855 | -4.99783 | 5.80E-07 | 3.09E-06 | Down |
| HNRNPA3P8 | 2.337980842 | -2.131558549 | 0.907863 | -2.34789 | 0.01888 | 0.039331 | Down |
| HNRNPCL1 | 2.087314508 | -1.131655696 | 0.483265 | -2.34169 | 0.019197 | 0.039893 | Down |
| HNRNPCL2 | 1.744581141 | -2.337547216 | 0.817121 | -2.86071 | 0.004227 | 0.010685 | Down |
| HNRNPCP10 | 1.252733864 | -3.349600206 | 1.366374 | -2.45145 | 0.014228 | 0.030889 | Down |
| HNRNPCP4 | 1.616506512 | -1.419498627 | 0.546937 | -2.59536 | 0.009449 | 0.021683 | Down |
| HNRNPCP6 | 10.89582212 | -1.549933017 | 0.224866 | -6.89269 | 5.47E-12 | 5.16E-11 | Down |
| HNRNPCP8 | 1.216964059 | -1.129787351 | 0.433491 | -2.60625 | 0.009154 | 0.021082 | Down |
| HNRNPFP1 | 1.066070987 | -2.980155852 | 1.059054 | -2.81398 | 0.004893 | 0.012153 | Down |
| HNRNPH1P3 | 1.451949358 | -2.062875157 | 0.881796 | -2.3394 | 0.019315 | 0.040106 | Down |
| HNRNPKP1 | 2.180250267 | -1.595955762 | 0.455226 | -3.50586 | 0.000455 | 0.001462 | Down |
| HNRNPKP3 | 1.282188549 | -2.355834864 | 0.937184 | -2.51374 | 0.011946 | 0.026597 | Down |
| HNRNPLP1 | 2.076855501 | -2.277046102 | 0.633223 | -3.59596 | 0.000323 | 0.001075 | Down |
| HOGA1 | 50.57902143 | -1.905238879 | 0.143976 | -13.233 | 5.66E-40 | 3.97E-38 | Down |
| HOMER2P1 | 2.016000071 | -2.519301028 | 0.597265 | -4.21806 | 2.46E-05 | 0.000102 | Down |
| HORMAD2 | 1.695240278 | 1.020567405 | 0.419666 | 2.431857 | 0.015022 | 0.032352 | Up |
| HOTAIR | 3.532795467 | -2.117459479 | 0.536332 | -3.94804 | 7.88E-05 | 0.000296 | Down |
| HOXA-AS3 | 5.112834241 | -1.172172876 | 0.304103 | -3.85453 | 0.000116 | 0.000422 | Down |
| HOXA2 | 19.70914691 | -1.170473807 | 0.192797 | -6.07101 | 1.27E-09 | 9.44E-09 | Down |
| HOXA6 | 2.686820708 | -1.050728679 | 0.264055 | -3.97921 | 6.91E-05 | 0.000262 | Down |
| HOXA7 | 12.16037071 | -1.129346377 | 0.213139 | -5.29865 | 1.17E-07 | 6.83E-07 | Down |
| HOXA9 | 8.562451974 | -1.719335141 | 0.301254 | -5.70725 | 1.15E-08 | 7.64E-08 | Down |
| HOXC-AS1 | 0.757927518 | -1.281046092 | 0.555823 | -2.30477 | 0.021179 | 0.043441 | Down |
| HOXC13 | 2.806512536 | -2.111907523 | 0.734231 | -2.87635 | 0.004023 | 0.010226 | Down |
| HOXC5 | 5.03412204 | -1.589132183 | 0.387146 | -4.10474 | 4.05E-05 | 0.000161 | Down |
| HOXC6 | 8.003349795 | -1.041379048 | 0.249157 | -4.17961 | 2.92E-05 | 0.000119 | Down |
| HOXD1 | 20.03349514 | 2.311492669 | 0.305387 | 7.569064 | 3.76E-14 | 4.36E-13 | Up |
| HOXD12 | 1.575163906 | -3.050050629 | 0.896081 | -3.40377 | 0.000665 | 0.002055 | Down |
| HPCAL4 | 398.5437644 | 4.473549332 | 0.240301 | 18.61646 | 2.36E-77 | 9.42E-75 | Up |
| HPD | 4.59456646 | -1.218490763 | 0.219018 | -5.56344 | 2.65E-08 | 1.68E-07 | Down |
| HPGDS | 56.60025952 | 1.264738044 | 0.148992 | 8.488631 | 2.09E-17 | 3.18E-16 | Up |
| HPN | 930.4042455 | 1.659395252 | 0.123302 | 13.45798 | 2.76E-41 | 2.10E-39 | Up |
| HPSE2 | 26.83996584 | 1.50422232 | 0.294486 | 5.107966 | 3.26E-07 | 1.80E-06 | Up |
| HPVC1 | 2.192717849 | -2.889403472 | 1.053251 | -2.74332 | 0.006082 | 0.014723 | Down |
| HRASLS5 | 16.5137878 | -1.135389332 | 0.19493 | -5.82459 | 5.73E-09 | 3.93E-08 | Down |
| HRH1 | 301.5963233 | 1.403626681 | 0.136509 | 10.28229 | 8.47E-25 | 2.24E-23 | Up |
| HRK | 186.8887916 | 2.017079007 | 0.228663 | 8.821195 | 1.13E-18 | 1.90E-17 | Up |
| HS3ST5 | 5.354432674 | -1.818579112 | 0.368912 | -4.92957 | 8.24E-07 | 4.29E-06 | Down |
| HS6ST1P1 | 1.92023659 | -2.134271512 | 0.54466 | -3.91854 | 8.91E-05 | 0.000331 | Down |
| HS6ST2 | 154.9570215 | 2.98244209 | 0.271063 | 11.00275 | 3.71E-28 | 1.26E-26 | Up |
| HS6ST3 | 132.448697 | -1.021960862 | 0.192123 | -5.3193 | 1.04E-07 | 6.13E-07 | Down |
| HSD11B2 | 123.2412078 | -1.654718524 | 0.134759 | -12.2791 | 1.17E-34 | 5.89E-33 | Down |
| HSD17B13 | 5.166123578 | -1.238440784 | 0.273107 | -4.53464 | 5.77E-06 | 2.65E-05 | Down |
| HSD17B3 | 16.73191729 | -1.632916201 | 0.181954 | -8.97435 | 2.85E-19 | 5.03E-18 | Down |
| HSD17B6 | 730.4820513 | -1.114588399 | 0.178881 | -6.23089 | 4.64E-10 | 3.61E-09 | Down |
| HSD17B7P1 | 1.47354797 | -2.313023312 | 0.377948 | -6.11996 | 9.36E-10 | 7.05E-09 | Down |
| HSD3B1 | 8.184639664 | -1.317184028 | 0.406112 | -3.2434 | 0.001181 | 0.003452 | Down |
| HSD3BP1 | 1.304539684 | -1.177777881 | 0.443037 | -2.65842 | 0.007851 | 0.018416 | Down |
| HSD52 | 2.356293044 | -2.011947067 | 0.414744 | -4.85106 | 1.23E-06 | 6.25E-06 | Down |
| HSF5 | 11.67993334 | -1.335977924 | 0.320616 | -4.16691 | 3.09E-05 | 0.000125 | Down |
| HSP90AA5P | 8.362876754 | -1.069939595 | 0.297238 | -3.5996 | 0.000319 | 0.001061 | Down |
| HSP90B3P | 2.08434352 | -1.878911994 | 0.493484 | -3.80744 | 0.00014 | 0.000503 | Down |
| HSPA8P14 | 2.275059882 | -1.408465696 | 0.39185 | -3.5944 | 0.000325 | 0.00108 | Down |
| HSPA8P16 | 16.31700424 | -2.678509421 | 0.271721 | -9.85757 | 6.36E-23 | 1.47E-21 | Down |
| HSPA8P6 | 1.565756284 | -2.589773546 | 0.753066 | -3.43897 | 0.000584 | 0.001829 | Down |
| HSPA9P1 | 2.519427199 | -1.060625898 | 0.465802 | -2.27699 | 0.022787 | 0.046241 | Down |
| HSPB3 | 1.707419482 | -2.425333933 | 0.462579 | -5.24307 | 1.58E-07 | 9.09E-07 | Down |
| HSPB6 | 66.71648398 | -1.381291451 | 0.195558 | -7.06333 | 1.63E-12 | 1.61E-11 | Down |
| HSPB7 | 180.624543 | -1.021265665 | 0.190207 | -5.36924 | 7.91E-08 | 4.72E-07 | Down |
| HSPD1P10 | 3.17063835 | -1.739690233 | 0.443279 | -3.9246 | 8.69E-05 | 0.000323 | Down |
| HSPD1P14 | 1.649447496 | -2.951618961 | 1.250958 | -2.35949 | 0.0183 | 0.038317 | Down |
| HSPD1P16 | 1.318439501 | -1.133952264 | 0.342978 | -3.3062 | 0.000946 | 0.002826 | Down |
| HSPD1P5 | 3.780623529 | -1.268587962 | 0.406567 | -3.12024 | 0.001807 | 0.005044 | Down |
| HSPD1P6 | 6.978790541 | -1.388272684 | 0.357983 | -3.87804 | 0.000105 | 0.000386 | Down |
| HSPD1P7 | 2.284335688 | -2.186491412 | 0.593892 | -3.68163 | 0.000232 | 0.000795 | Down |
| HSPE1P11 | 6.844588018 | -1.08046803 | 0.215611 | -5.01119 | 5.41E-07 | 2.89E-06 | Down |
| HSPE1P27 | 0.861417886 | -1.11107415 | 0.447057 | -2.48531 | 0.012944 | 0.028521 | Down |
| HTR1A | 2.617012151 | -1.743955679 | 0.384105 | -4.54031 | 5.62E-06 | 2.58E-05 | Down |
| HTR1E | 15.18728288 | -2.191634251 | 0.262963 | -8.33437 | 7.79E-17 | 1.13E-15 | Down |
| HTR2A | 15.47107314 | -1.576709492 | 0.256623 | -6.14406 | 8.04E-10 | 6.11E-09 | Down |
| HTR3A | 10.15950018 | -1.288747762 | 0.408914 | -3.15163 | 0.001624 | 0.004588 | Down |
| HTR3B | 1.137226061 | -2.064653225 | 0.904507 | -2.28263 | 0.022452 | 0.045665 | Down |
| HTR3C | 2.128529425 | -2.444937315 | 0.430298 | -5.68196 | 1.33E-08 | 8.78E-08 | Down |
| HTR3D | 1.111350094 | -2.316804954 | 0.860267 | -2.69312 | 0.007079 | 0.01683 | Down |
| HTR3E | 2.144331644 | -1.243147531 | 0.432933 | -2.87145 | 0.004086 | 0.010363 | Down |
| HTR5A | 1.639642814 | -2.029895475 | 0.703996 | -2.88339 | 0.003934 | 0.010023 | Down |
| HTR5BP | 4.237167096 | -1.101452845 | 0.315236 | -3.49405 | 0.000476 | 0.001522 | Down |
| HTR6 | 4.111115914 | -1.138842791 | 0.338247 | -3.3669 | 0.00076 | 0.002318 | Down |
| HUS1B | 5.368625809 | -1.035675844 | 0.277491 | -3.73228 | 0.00019 | 0.000663 | Down |
| HYAL4 | 2.433660548 | -2.138518628 | 0.552287 | -3.87212 | 0.000108 | 0.000394 | Down |
| HYI-AS1 | 2.269315629 | -1.0108336 | 0.32983 | -3.06471 | 0.002179 | 0.005947 | Down |
| HYMAI | 23.01684985 | -1.059518717 | 0.283234 | -3.74079 | 0.000183 | 0.000643 | Down |
| IAPP | 1.066401225 | -2.447016043 | 0.990307 | -2.47097 | 0.013475 | 0.029521 | Down |
| IBSP | 10.38415058 | 2.497059976 | 0.514824 | 4.850314 | 1.23E-06 | 6.27E-06 | Up |
| ICAM1 | 2538.914895 | 2.317798286 | 0.117976 | 19.64638 | 6.21E-86 | 3.40E-83 | Up |
| ICAM4 | 59.91223108 | 3.055282423 | 0.187035 | 16.33538 | 5.53E-60 | 1.11E-57 | Up |
| ICAM5 | 206.4970939 | 2.834425814 | 0.208134 | 13.61829 | 3.12E-42 | 2.50E-40 | Up |
| ID2-AS1 | 15.29282457 | -1.014683358 | 0.151158 | -6.71274 | 1.91E-11 | 1.70E-10 | Down |
| ID3 | 2616.962446 | -1.086907271 | 0.111092 | -9.78384 | 1.32E-22 | 2.98E-21 | Down |
| ID4 | 6633.080271 | -1.316234694 | 0.098413 | -13.3746 | 8.51E-41 | 6.25E-39 | Down |
| IDO2 | 13.36081569 | -1.286434154 | 0.216581 | -5.93974 | 2.85E-09 | 2.03E-08 | Down |
| IER5L | 408.2237881 | 2.044259744 | 0.099983 | 20.44602 | 6.52E-93 | 4.46E-90 | Up |
| IFIT1B | 5.070687169 | -1.587357756 | 0.329788 | -4.81327 | 1.48E-06 | 7.46E-06 | Down |
| IFITM5 | 3.531645949 | -1.409740314 | 0.313864 | -4.49156 | 7.07E-06 | 3.20E-05 | Down |
| IFNA10 | 1.399585709 | -2.152569502 | 0.65868 | -3.268 | 0.001083 | 0.00319 | Down |
| IFNA16 | 1.321636727 | -1.811130114 | 0.758547 | -2.38763 | 0.016957 | 0.03589 | Down |
| IFNA21 | 1.434377789 | -1.833924919 | 0.526152 | -3.48554 | 0.000491 | 0.001565 | Down |
| IFNA5 | 1.162077681 | -1.333895658 | 0.505988 | -2.63622 | 0.008384 | 0.019524 | Down |
| IFNE | 15.95491471 | 2.00783608 | 0.227918 | 8.809478 | 1.26E-18 | 2.11E-17 | Up |
| IFNL4 | 1.400504414 | -2.679004624 | 0.853712 | -3.13807 | 0.001701 | 0.004783 | Down |
| IFNW1 | 1.441321371 | -1.543732053 | 0.446764 | -3.45537 | 0.00055 | 0.001733 | Down |
| IFNWP19 | 22.45840291 | 2.83866774 | 0.181105 | 15.67415 | 2.27E-55 | 3.72E-53 | Up |
| IFNWP2 | 1.304715113 | 1.630977383 | 0.36928 | 4.416644 | 1.00E-05 | 4.43E-05 | Up |
| IGBP1-AS1 | 1.362583216 | -1.092971498 | 0.422369 | -2.58772 | 0.009661 | 0.022093 | Down |
| IGBP1P4 | 2.568350599 | -1.157691915 | 0.297 | -3.89795 | 9.70E-05 | 0.000358 | Down |
| IGF2-AS | 2.8511361 | -1.614327888 | 0.442119 | -3.65134 | 0.000261 | 0.000886 | Down |
| IGF2BP2 | 663.8246053 | 1.829958298 | 0.108212 | 16.9109 | 3.74E-64 | 8.57E-62 | Up |
| IGFBP5 | 39138.77046 | 1.272163211 | 0.14094 | 9.026297 | 1.78E-19 | 3.18E-18 | Up |
| IGFBP6 | 2417.105453 | 1.72514586 | 0.144046 | 11.97634 | 4.73E-33 | 2.18E-31 | Up |
| IGFBPL1 | 231.0698738 | -2.490375948 | 0.189847 | -13.1178 | 2.60E-39 | 1.75E-37 | Down |
| IGFL1 | 1.283023179 | 2.752430284 | 0.697049 | 3.948688 | 7.86E-05 | 0.000295 | Up |
| IGFL2 | 108.8845719 | 4.80902092 | 0.31473 | 15.27985 | 1.04E-52 | 1.43E-50 | Up |
| IGSF1 | 4703.971453 | 3.394802407 | 0.237223 | 14.31062 | 1.88E-46 | 1.96E-44 | Up |
| IGSF10 | 532.0332538 | -2.253053571 | 0.169026 | -13.3296 | 1.56E-40 | 1.13E-38 | Down |
| IGSF11-AS1 | 1.530802191 | -2.517482922 | 0.872244 | -2.88621 | 0.003899 | 0.009946 | Down |
| IGSF21 | 32.11779835 | 1.035733113 | 0.179077 | 5.783732 | 7.31E-09 | 4.96E-08 | Up |
| IGSF3 | 1386.551032 | 1.602826103 | 0.10207 | 15.70315 | 1.44E-55 | 2.39E-53 | Up |
| IGSF5 | 12.64378048 | -1.334032276 | 0.209298 | -6.37384 | 1.84E-10 | 1.50E-09 | Down |
| IGSF9B | 286.9099712 | -1.112366571 | 0.123806 | -8.98476 | 2.59E-19 | 4.58E-18 | Down |
| IHH | 8.439777 | -3.319210676 | 0.406088 | -8.17363 | 2.99E-16 | 4.13E-15 | Down |
| IKZF2 | 2488.83026 | 1.258587315 | 0.082998 | 15.1641 | 6.11E-52 | 8.12E-50 | Up |
| IL17C | 2.951909753 | -1.091094354 | 0.407376 | -2.67835 | 0.007399 | 0.017486 | Down |
| IL17RD | 444.0725577 | 1.235073353 | 0.108326 | 11.40145 | 4.11E-30 | 1.58E-28 | Up |
| IL1RAP | 828.7935395 | 2.255228327 | 0.103056 | 21.88349 | 3.73E-106 | 4.03E-103 | Up |
| IL1RAPL1 | 26.21795655 | -2.083271449 | 0.193957 | -10.7409 | 6.54E-27 | 2.00E-25 | Down |
| IL1RL1 | 188.4199552 | 2.304923617 | 0.298195 | 7.729578 | 1.08E-14 | 1.32E-13 | Up |
| IL1RN | 168.0026498 | 2.1579745 | 0.18544 | 11.63705 | 2.67E-31 | 1.12E-29 | Up |
| IL20 | 1.168267398 | -2.096733466 | 0.729885 | -2.87269 | 0.00407 | 0.010328 | Down |
| IL20RA | 65.50301108 | -1.08119408 | 0.148646 | -7.27364 | 3.50E-13 | 3.68E-12 | Down |
| IL21-AS1 | 6.553308772 | -1.615154252 | 0.526977 | -3.06494 | 0.002177 | 0.005943 | Down |
| IL22RA1 | 76.29414441 | -1.126440032 | 0.126882 | -8.87785 | 6.82E-19 | 1.17E-17 | Down |
| IL22RA2 | 16.06954947 | 2.009611543 | 0.406488 | 4.943841 | 7.66E-07 | 4.01E-06 | Up |
| IL25 | 1.474008315 | -2.724956383 | 0.63527 | -4.28945 | 1.79E-05 | 7.58E-05 | Down |
| IL31 | 1.091031301 | -1.64756832 | 0.474165 | -3.47468 | 0.000511 | 0.001623 | Down |
| IL36A | 0.911596156 | 1.745333027 | 0.685489 | 2.546112 | 0.010893 | 0.024506 | Up |
| IL36RN | 20.97539835 | 1.545651312 | 0.334629 | 4.618996 | 3.86E-06 | 1.82E-05 | Up |
| IL37 | 0.503760989 | 1.969307658 | 0.689514 | 2.85608 | 0.004289 | 0.010824 | Up |
| IL6RP1 | 1.066842781 | -2.412057248 | 1.021895 | -2.36038 | 0.018256 | 0.038233 | Down |
| IL6STP1 | 2.093713871 | -1.224965379 | 0.362154 | -3.38244 | 0.000718 | 0.002204 | Down |
| ILF2P2 | 0.869935502 | -1.278149225 | 0.543499 | -2.3517 | 0.018688 | 0.038982 | Down |
| IMMTP1 | 7.041977608 | -1.812313136 | 0.246261 | -7.35933 | 1.85E-13 | 2.00E-12 | Down |
| IMP3P2 | 0.663196936 | -1.414618344 | 0.571047 | -2.47724 | 0.01324 | 0.029077 | Down |
| IMPA2 | 373.3082889 | -1.088466039 | 0.096497 | -11.2797 | 1.65E-29 | 6.13E-28 | Down |
| INA | 5.514398743 | 1.281831742 | 0.278878 | 4.596384 | 4.30E-06 | 2.01E-05 | Up |
| INGX | 3.087713979 | -1.208496247 | 0.389513 | -3.10258 | 0.001918 | 0.005318 | Down |
| INHA | 9.794780492 | -1.3898685 | 0.212776 | -6.53206 | 6.49E-11 | 5.49E-10 | Down |
| INHBA | 460.4367577 | 1.795366238 | 0.156531 | 11.46969 | 1.87E-30 | 7.38E-29 | Up |
| INHBB | 1746.131857 | 1.90229016 | 0.10948 | 17.37577 | 1.26E-67 | 3.27E-65 | Up |
| INHBC | 6.782176091 | -2.227575207 | 0.271216 | -8.21329 | 2.15E-16 | 3.00E-15 | Down |
| INHBE | 4.841378669 | -1.171442161 | 0.295314 | -3.96677 | 7.29E-05 | 0.000275 | Down |
| INMT | 2186.823294 | 1.37922075 | 0.195796 | 7.044176 | 1.87E-12 | 1.84E-11 | Up |
| INSL3 | 5.655590439 | 1.523770477 | 0.272747 | 5.586757 | 2.31E-08 | 1.48E-07 | Up |
| INSL5 | 0.863308438 | -1.629416179 | 0.468094 | -3.48096 | 0.0005 | 0.001589 | Down |
| INSM2 | 5.189119283 | -1.17492888 | 0.314194 | -3.7395 | 0.000184 | 0.000646 | Down |
| IP6K3 | 259.8340582 | -1.782549749 | 0.139117 | -12.8134 | 1.38E-37 | 8.33E-36 | Down |
| IPCEF1 | 2293.387864 | -2.431248517 | 0.172752 | -14.0736 | 5.52E-45 | 5.32E-43 | Down |
| IQCB2P | 2.54463043 | -1.120228451 | 0.470196 | -2.38247 | 0.017197 | 0.036307 | Down |
| IQGAP2 | 1719.397575 | -1.335875593 | 0.121801 | -10.9677 | 5.47E-28 | 1.82E-26 | Down |
| IRAK2 | 293.4855316 | 1.448371575 | 0.074951 | 19.32426 | 3.36E-83 | 1.64E-80 | Up |
| IRGC | 1.664690752 | -1.543788665 | 0.624716 | -2.47118 | 0.013467 | 0.029509 | Down |
| IRGM | 2.883769046 | -1.341778061 | 0.375866 | -3.56983 | 0.000357 | 0.001176 | Down |
| IRS1 | 3496.89956 | -1.067680543 | 0.091618 | -11.6536 | 2.20E-31 | 9.31E-30 | Down |
| IRS4 | 91.11568334 | -2.0750594 | 0.189204 | -10.9673 | 5.49E-28 | 1.82E-26 | Down |
| IRX1P1 | 1.120845093 | -3.059938176 | 1.190148 | -2.57106 | 0.010139 | 0.023017 | Down |
| IRX2 | 106.2841184 | 1.539128301 | 0.210915 | 7.297388 | 2.93E-13 | 3.12E-12 | Up |
| IRX3 | 212.5966469 | 1.052321191 | 0.127423 | 8.258519 | 1.47E-16 | 2.09E-15 | Up |
| IRX4 | 1.773377447 | -2.088088405 | 0.655731 | -3.18437 | 0.001451 | 0.004145 | Down |
| IRX6 | 17.51595145 | -1.817895 | 0.256761 | -7.0801 | 1.44E-12 | 1.44E-11 | Down |
| ISCA2P1 | 1.508815122 | -1.628330595 | 0.366655 | -4.44104 | 8.95E-06 | 3.99E-05 | Down |
| ISL1 | 4.728096575 | -1.416988334 | 0.311314 | -4.55163 | 5.32E-06 | 2.46E-05 | Down |
| ISL2 | 5.064249634 | -1.672024663 | 0.455437 | -3.67125 | 0.000241 | 0.000825 | Down |
| ISM2 | 14.85685158 | -1.035994071 | 0.215117 | -4.81595 | 1.46E-06 | 7.37E-06 | Down |
| ISX | 3.134744504 | -1.735061348 | 0.468799 | -3.70108 | 0.000215 | 0.000742 | Down |
| ITFG1-AS1 | 1.251196398 | -1.685413443 | 0.357445 | -4.71516 | 2.42E-06 | 1.18E-05 | Down |
| ITGA2 | 2313.776604 | 3.153882251 | 0.132462 | 23.80964 | 2.65E-125 | 6.41E-122 | Up |
| ITGA3 | 10538.5246 | 1.646319977 | 0.094862 | 17.35489 | 1.81E-67 | 4.65E-65 | Up |
| ITGAD | 20.13124624 | -1.164677428 | 0.384885 | -3.02604 | 0.002478 | 0.006656 | Down |
| ITGBL1 | 713.3902594 | 1.130896248 | 0.163388 | 6.921554 | 4.47E-12 | 4.24E-11 | Up |
| ITIH1 | 3.940060882 | -1.088582103 | 0.391248 | -2.78233 | 0.005397 | 0.013262 | Down |
| ITIH2 | 5.763883836 | 1.144182017 | 0.27714 | 4.128536 | 3.65E-05 | 0.000146 | Up |
| ITIH3 | 47.03705908 | -1.057774853 | 0.196426 | -5.38511 | 7.24E-08 | 4.35E-07 | Down |
| ITLN1 | 18.33411709 | -2.293036684 | 0.38361 | -5.97751 | 2.27E-09 | 1.63E-08 | Down |
| ITLN2 | 1.391289195 | -1.633853004 | 0.578735 | -2.82315 | 0.004755 | 0.011864 | Down |
| ITM2BP1 | 5.231052984 | -1.304155871 | 0.230203 | -5.66525 | 1.47E-08 | 9.62E-08 | Down |
| ITPR1 | 5019.210397 | -1.205305867 | 0.100115 | -12.0392 | 2.21E-33 | 1.04E-31 | Down |
| ITPR1-AS1 | 10.40400583 | -1.539547109 | 0.172684 | -8.91541 | 4.86E-19 | 8.40E-18 | Down |
| ITPR3 | 6777.655498 | 1.134278911 | 0.096442 | 11.76127 | 6.18E-32 | 2.70E-30 | Up |
| IVL | 20.4774763 | 4.065599452 | 0.368278 | 11.0395 | 2.46E-28 | 8.45E-27 | Up |
| IYD | 32751.95345 | -1.049556791 | 0.139596 | -7.51853 | 5.54E-14 | 6.32E-13 | Down |
| IZUMO1 | 7.934560426 | -1.312424736 | 0.295124 | -4.44703 | 8.71E-06 | 3.89E-05 | Down |
| JAKMIP3 | 25.88798015 | -1.499075476 | 0.160037 | -9.36708 | 7.46E-21 | 1.48E-19 | Down |
| JAZF1-AS1 | 6.103806407 | -1.323453037 | 0.320852 | -4.12481 | 3.71E-05 | 0.000149 | Down |
| JUN | 6300.090446 | -1.401720361 | 0.171541 | -8.17133 | 3.05E-16 | 4.20E-15 | Down |
| KARSP1 | 6.553214198 | -1.023174852 | 0.249934 | -4.09378 | 4.24E-05 | 0.000168 | Down |
| KARSP2 | 2.752485807 | -2.105181113 | 0.325046 | -6.47657 | 9.38E-11 | 7.83E-10 | Down |
| KATNAL2 | 265.9936318 | -1.582338858 | 0.102127 | -15.4938 | 3.82E-54 | 5.72E-52 | Down |
| KB-1183D5.13 | 30.00673432 | 3.308770702 | 0.342825 | 9.651496 | 4.84E-22 | 1.05E-20 | Up |
| KB-1440D3.13 | 2.830023586 | -1.152754778 | 0.261911 | -4.40132 | 1.08E-05 | 4.73E-05 | Down |
| KB-1440D3.14 | 0.848580212 | -1.690020785 | 0.5262 | -3.21175 | 0.001319 | 0.003808 | Down |
| KB-1448A5.1 | 4.849597626 | -2.017186024 | 0.252685 | -7.983 | 1.43E-15 | 1.87E-14 | Down |
| KB-1471A8.1 | 29.82456792 | -1.664120161 | 0.160206 | -10.3874 | 2.83E-25 | 7.73E-24 | Down |
| KB-1562D12.2 | 1.586792035 | -1.037746166 | 0.401743 | -2.58311 | 0.009791 | 0.022348 | Down |
| KB-1592A4.14 | 2.528559679 | 1.818644847 | 0.48897 | 3.719337 | 0.0002 | 0.000695 | Up |
| KB-1732A1.1 | 21.10526 | -1.298763539 | 0.149555 | -8.68417 | 3.82E-18 | 6.13E-17 | Down |
| KB-176G8.1 | 1.977666285 | 1.244400401 | 0.486565 | 2.557523 | 0.010542 | 0.023815 | Up |
| KB-1958F4.1 | 14.08930524 | -1.417218561 | 0.203575 | -6.96165 | 3.36E-12 | 3.22E-11 | Down |
| KB-1958F4.2 | 3.734720968 | -1.581594095 | 0.312956 | -5.05372 | 4.33E-07 | 2.35E-06 | Down |
| KB-1980E6.2 | 1.657837339 | -1.683412944 | 0.504464 | -3.33703 | 0.000847 | 0.002556 | Down |
| KB-318B8.7 | 6.348023073 | -1.111583066 | 0.213399 | -5.20894 | 1.90E-07 | 1.08E-06 | Down |
| KB-68A7.1 | 1.965291135 | -1.029450646 | 0.23391 | -4.40106 | 1.08E-05 | 4.73E-05 | Down |
| KB-68A7.2 | 0.905594438 | -2.021305976 | 0.582777 | -3.46841 | 0.000524 | 0.001658 | Down |
| KBTBD11 | 392.8042028 | -1.544942056 | 0.108179 | -14.2814 | 2.86E-46 | 2.93E-44 | Down |
| KBTBD12 | 14.36812788 | -1.185729513 | 0.276887 | -4.28236 | 1.85E-05 | 7.81E-05 | Down |
| KBTBD13 | 2.861495854 | -1.821667877 | 0.448218 | -4.06425 | 4.82E-05 | 0.000189 | Down |
| KC6 | 9.080822653 | -1.506101027 | 0.403882 | -3.72906 | 0.000192 | 0.000671 | Down |
| KCNA1 | 87.0735104 | -1.988241986 | 0.234211 | -8.4891 | 2.08E-17 | 3.17E-16 | Down |
| KCNA2 | 57.57604256 | -1.220132854 | 0.224331 | -5.43899 | 5.36E-08 | 3.27E-07 | Down |
| KCNA4 | 5.599556581 | -1.624470301 | 0.392959 | -4.13394 | 3.57E-05 | 0.000143 | Down |
| KCNAB1 | 934.2285429 | -1.300847447 | 0.156431 | -8.31577 | 9.12E-17 | 1.31E-15 | Down |
| KCNB1 | 48.44337388 | -1.311891792 | 0.19749 | -6.64282 | 3.08E-11 | 2.68E-10 | Down |
| KCNB2 | 6.707651623 | -1.659964428 | 0.370111 | -4.48505 | 7.29E-06 | 3.29E-05 | Down |
| KCNC4-AS1 | 2.390394347 | -1.31824742 | 0.290393 | -4.53953 | 5.64E-06 | 2.59E-05 | Down |
| KCNG1 | 18.31953376 | -1.525081864 | 0.377566 | -4.03924 | 5.36E-05 | 0.000208 | Down |
| KCNG2 | 12.83474169 | -1.446718951 | 0.210537 | -6.87156 | 6.35E-12 | 5.94E-11 | Down |
| KCNG4 | 2.136691213 | -1.343787434 | 0.546429 | -2.45922 | 0.013924 | 0.030347 | Down |
| KCNH6 | 2.547628745 | -1.342288412 | 0.403929 | -3.32308 | 0.00089 | 0.002676 | Down |
| KCNH7 | 6.378582975 | -1.03561125 | 0.320827 | -3.22794 | 0.001247 | 0.003625 | Down |
| KCNH8 | 26.90263742 | -1.057577353 | 0.201282 | -5.25419 | 1.49E-07 | 8.59E-07 | Down |
| KCNIP1 | 17.72711669 | -1.205663931 | 0.176848 | -6.81752 | 9.26E-12 | 8.51E-11 | Down |
| KCNIP2 | 50.42946577 | -1.457436555 | 0.19737 | -7.38427 | 1.53E-13 | 1.68E-12 | Down |
| KCNIP3 | 538.4983759 | -1.16428286 | 0.112612 | -10.3389 | 4.70E-25 | 1.26E-23 | Down |
| KCNIP4 | 157.1899522 | -1.773742411 | 0.111356 | -15.9286 | 4.01E-57 | 7.07E-55 | Down |
| KCNJ1 | 34.48401999 | -1.426734556 | 0.226773 | -6.29147 | 3.14E-10 | 2.49E-09 | Down |
| KCNJ10 | 38.84082436 | -1.210186579 | 0.24527 | -4.9341 | 8.05E-07 | 4.20E-06 | Down |
| KCNJ13 | 194.802999 | -1.460872573 | 0.137403 | -10.632 | 2.11E-26 | 6.22E-25 | Down |
| KCNJ2 | 1238.536721 | 3.857538859 | 0.153953 | 25.05668 | 1.48E-138 | 7.57E-135 | Up |
| KCNJ2-AS1 | 57.97527534 | 1.982406729 | 0.143169 | 13.84658 | 1.33E-43 | 1.16E-41 | Up |
| KCNJ4 | 2.135009275 | -1.620119138 | 0.56565 | -2.86417 | 0.004181 | 0.010582 | Down |
| KCNJ6 | 22.34980671 | 1.60457551 | 0.356523 | 4.500618 | 6.78E-06 | 3.08E-05 | Up |
| KCNK1 | 254.3217924 | 1.227858371 | 0.080508 | 15.2514 | 1.61E-52 | 2.20E-50 | Up |
| KCNK15 | 154.4341187 | 1.22255992 | 0.116518 | 10.49248 | 9.35E-26 | 2.65E-24 | Up |
| KCNK16 | 6.160929172 | -1.319215728 | 0.249262 | -5.29248 | 1.21E-07 | 7.05E-07 | Down |
| KCNK18 | 1.204926207 | -2.214823193 | 0.833686 | -2.65666 | 0.007892 | 0.018503 | Down |
| KCNK2 | 353.1474843 | -1.680197702 | 0.143519 | -11.7072 | 1.17E-31 | 5.06E-30 | Down |
| KCNK5 | 851.954445 | 1.237050772 | 0.087707 | 14.10442 | 3.57E-45 | 3.48E-43 | Up |
| KCNK9 | 2.598374795 | -1.788428884 | 0.421314 | -4.24488 | 2.19E-05 | 9.12E-05 | Down |
| KCNMB2 | 20.73567899 | -1.456963367 | 0.206029 | -7.07164 | 1.53E-12 | 1.52E-11 | Down |
| KCNMB3P1 | 2.971698803 | -1.99289947 | 0.45905 | -4.34136 | 1.42E-05 | 6.09E-05 | Down |
| KCNN4 | 423.9452289 | 2.73219319 | 0.208168 | 13.12497 | 2.37E-39 | 1.60E-37 | Up |
| KCNQ1-AS1 | 2.149435679 | -1.523439113 | 0.378504 | -4.0249 | 5.70E-05 | 0.00022 | Down |
| KCNQ1DN | 1.114330378 | -2.654086047 | 0.737481 | -3.59886 | 0.00032 | 0.001064 | Down |
| KCNQ3 | 710.2158836 | 3.657048467 | 0.198179 | 18.4533 | 4.91E-76 | 1.86E-73 | Up |
| KCNQ4 | 15.73336529 | -1.205961227 | 0.224801 | -5.36457 | 8.11E-08 | 4.84E-07 | Down |
| KCNQ5-AS1 | 4.016568215 | -1.287499156 | 0.288612 | -4.461 | 8.16E-06 | 3.66E-05 | Down |
| KCNQ5-IT1 | 2.666561896 | -1.297649692 | 0.316511 | -4.09985 | 4.13E-05 | 0.000164 | Down |
| KCNS1 | 14.81539201 | -1.109518158 | 0.24255 | -4.57439 | 4.78E-06 | 2.22E-05 | Down |
| KCNS3 | 167.3401472 | 1.127327715 | 0.100397 | 11.22868 | 2.95E-29 | 1.08E-27 | Up |
| KCNV1 | 26.16613906 | 1.121734206 | 0.213338 | 5.258006 | 1.46E-07 | 8.43E-07 | Up |
| KCP | 95.03840309 | 1.79171174 | 0.181563 | 9.868246 | 5.72E-23 | 1.32E-21 | Up |
| KCTD10P1 | 2.080598241 | -2.556967967 | 0.346837 | -7.37225 | 1.68E-13 | 1.83E-12 | Down |
| KCTD16 | 265.1503321 | -2.151786874 | 0.184689 | -11.6508 | 2.27E-31 | 9.59E-30 | Down |
| KCTD19 | 10.24308399 | -1.484265014 | 0.330689 | -4.4884 | 7.18E-06 | 3.25E-05 | Down |
| KCTD8 | 71.14019426 | -1.463532303 | 0.14265 | -10.2596 | 1.07E-24 | 2.80E-23 | Down |
| KCTD9P4 | 7.489742737 | -1.403027745 | 0.23614 | -5.94151 | 2.82E-09 | 2.01E-08 | Down |
| KDELR3 | 354.5518593 | 1.062479447 | 0.075922 | 13.99438 | 1.69E-44 | 1.56E-42 | Up |
| KEL | 20.94065156 | -1.616612716 | 0.303987 | -5.31804 | 1.05E-07 | 6.17E-07 | Down |
| KHDRBS2 | 293.6274172 | -2.087573082 | 0.145637 | -14.3341 | 1.34E-46 | 1.42E-44 | Down |
| KIAA0087 | 4.141425761 | -1.587990594 | 0.484305 | -3.27891 | 0.001042 | 0.003083 | Down |
| KIAA0408 | 307.022851 | 2.374060621 | 0.187996 | 12.62826 | 1.48E-36 | 8.42E-35 | Up |
| KIAA1045 | 41.62079545 | -1.284366778 | 0.171181 | -7.50299 | 6.24E-14 | 7.08E-13 | Down |
| KIAA1324 | 908.5666814 | -2.298248428 | 0.188801 | -12.1729 | 4.34E-34 | 2.11E-32 | Down |
| KIAA1456 | 2810.168228 | -1.410798207 | 0.110408 | -12.7781 | 2.17E-37 | 1.29E-35 | Down |
| KIAA1467 | 528.0829457 | -1.283570451 | 0.079517 | -16.1421 | 1.29E-58 | 2.43E-56 | Down |
| KIAA1549L | 87.66764722 | 1.513102626 | 0.249211 | 6.071569 | 1.27E-09 | 9.42E-09 | Up |
| KIAA2022 | 113.1134736 | -1.114887843 | 0.117072 | -9.5231 | 1.68E-21 | 3.51E-20 | Down |
| KIF12 | 402.1284342 | -1.281591165 | 0.119292 | -10.7433 | 6.37E-27 | 1.95E-25 | Down |
| KIF19 | 106.7152798 | -2.031220083 | 0.2087 | -9.73274 | 2.19E-22 | 4.86E-21 | Down |
| KIF1A | 91.58808836 | -1.725166484 | 0.231458 | -7.45347 | 9.09E-14 | 1.02E-12 | Down |
| KIF25 | 2.084761676 | -1.521233303 | 0.391917 | -3.88152 | 0.000104 | 0.000381 | Down |
| KIF25-AS1 | 1.632865698 | -1.807985417 | 0.657099 | -2.75146 | 0.005933 | 0.014408 | Down |
| KIF6 | 58.55759729 | -1.775125968 | 0.143888 | -12.3369 | 5.73E-35 | 2.97E-33 | Down |
| KIR2DL1 | 2.123415109 | -1.894022797 | 0.537664 | -3.52269 | 0.000427 | 0.001381 | Down |
| KIR3DL1 | 3.305486339 | -1.581433485 | 0.436767 | -3.62077 | 0.000294 | 0.000985 | Down |
| KIR3DL2 | 3.110672718 | -1.206650718 | 0.484501 | -2.4905 | 0.012756 | 0.028165 | Down |
| KIR3DL3 | 1.609467125 | -1.960599152 | 0.731931 | -2.67867 | 0.007392 | 0.017471 | Down |
| KIR3DX1 | 4.919724999 | -1.884416417 | 0.494272 | -3.81251 | 0.000138 | 0.000493 | Down |
| KIRREL3 | 12.50617247 | -1.097641405 | 0.280252 | -3.91663 | 8.98E-05 | 0.000333 | Down |
| KISS1 | 1.040498766 | 1.411418136 | 0.394091 | 3.581456 | 0.000342 | 0.00113 | Up |
| KISS1R | 8.789688877 | 1.561536383 | 0.357268 | 4.370769 | 1.24E-05 | 5.39E-05 | Up |
| KIT | 1840.789215 | -2.023930118 | 0.147446 | -13.7266 | 7.03E-43 | 5.90E-41 | Down |
| KIZ-AS1 | 30.81094079 | -1.247879759 | 0.203685 | -6.12652 | 8.98E-10 | 6.78E-09 | Down |
| KL | 249.8061413 | 1.455058585 | 0.155837 | 9.337053 | 9.91E-21 | 1.95E-19 | Up |
| KLB | 34.13581371 | -1.25193173 | 0.183363 | -6.82762 | 8.63E-12 | 7.96E-11 | Down |
| KLC3 | 38.89158875 | 1.494065415 | 0.200859 | 7.438378 | 1.02E-13 | 1.13E-12 | Up |
| KLF1 | 3.068256939 | -1.272527552 | 0.324546 | -3.92095 | 8.82E-05 | 0.000328 | Down |
| KLF15 | 127.129099 | -1.260838892 | 0.122056 | -10.33 | 5.16E-25 | 1.38E-23 | Down |
| KLF2P1 | 1.744089618 | -3.060893604 | 0.706245 | -4.33404 | 1.46E-05 | 6.29E-05 | Down |
| KLF2P2 | 1.122879593 | -1.641679564 | 0.596126 | -2.75391 | 0.005889 | 0.014315 | Down |
| KLF2P3 | 1.379100919 | -3.120173048 | 1.043736 | -2.98943 | 0.002795 | 0.0074 | Down |
| KLF7P1 | 1.198815029 | -2.223322125 | 0.786455 | -2.82702 | 0.004698 | 0.011737 | Down |
| KLHDC7B | 63.38416961 | 1.039869155 | 0.275389 | 3.776001 | 0.000159 | 0.000565 | Up |
| KLHDC8A | 384.2506035 | 4.090874504 | 0.252975 | 16.17107 | 8.07E-59 | 1.54E-56 | Up |
| KLHL2 | 892.0368573 | 1.028940144 | 0.062839 | 16.37414 | 2.93E-60 | 5.92E-58 | Up |
| KLHL33 | 12.26034538 | -1.328991565 | 0.201397 | -6.59888 | 4.14E-11 | 3.57E-10 | Down |
| KLHL4 | 132.5500248 | -2.116260598 | 0.162407 | -13.0306 | 8.20E-39 | 5.28E-37 | Down |
| KLHL40 | 2.84360998 | -1.96828697 | 0.488252 | -4.03129 | 5.55E-05 | 0.000214 | Down |
| KLHL7-AS1 | 10.206388 | -1.135409016 | 0.251432 | -4.51577 | 6.31E-06 | 2.88E-05 | Down |
| KLK1 | 33.1192733 | -1.99629431 | 0.268164 | -7.44431 | 9.75E-14 | 1.08E-12 | Down |
| KLK10 | 475.0318826 | 4.245656514 | 0.25209 | 16.84183 | 1.20E-63 | 2.73E-61 | Up |
| KLK11 | 200.7580941 | 3.966497932 | 0.392458 | 10.10681 | 5.15E-24 | 1.29E-22 | Up |
| KLK12 | 23.40862121 | 3.854337576 | 0.441123 | 8.737549 | 2.38E-18 | 3.89E-17 | Up |
| KLK13 | 12.76531476 | 1.524450591 | 0.218951 | 6.962504 | 3.34E-12 | 3.21E-11 | Up |
| KLK14 | 1.274723853 | -1.076802313 | 0.311821 | -3.45327 | 0.000554 | 0.001743 | Down |
| KLK15 | 7.036688493 | -1.418363648 | 0.288512 | -4.91614 | 8.83E-07 | 4.58E-06 | Down |
| KLK2 | 70.42182048 | -2.236993911 | 0.223783 | -9.99624 | 1.58E-23 | 3.82E-22 | Down |
| KLK3 | 7.726818005 | -1.394817694 | 0.270104 | -5.16401 | 2.42E-07 | 1.36E-06 | Down |
| KLK4 | 38.04698845 | -1.644236674 | 0.221597 | -7.41993 | 1.17E-13 | 1.29E-12 | Down |
| KLK5 | 17.53878765 | 1.285114259 | 0.291569 | 4.407577 | 1.05E-05 | 4.60E-05 | Up |
| KLK6 | 31.66064232 | 2.957863421 | 0.430037 | 6.878153 | 6.06E-12 | 5.69E-11 | Up |
| KLK7 | 330.1379683 | 4.404602411 | 0.338185 | 13.02423 | 8.91E-39 | 5.70E-37 | Up |
| KLK8 | 3.614861346 | 2.380324582 | 0.379381 | 6.274228 | 3.51E-10 | 2.78E-09 | Up |
| KLKP1 | 3.714900739 | -2.067068449 | 0.491918 | -4.20206 | 2.65E-05 | 0.000109 | Down |
| KNOP1P1 | 1.630494866 | -1.876213622 | 0.803517 | -2.335 | 0.019543 | 0.040535 | Down |
| KNOP1P4 | 7.681983332 | -1.999955567 | 0.324266 | -6.16763 | 6.93E-10 | 5.29E-09 | Down |
| KRBOX1-AS1 | 4.237379084 | -1.942360899 | 0.500365 | -3.88189 | 0.000104 | 0.00038 | Down |
| KRT1 | 3.793715963 | -1.207171374 | 0.420432 | -2.87127 | 0.004088 | 0.010368 | Down |
| KRT14 | 21.94453532 | 1.008028916 | 0.271446 | 3.713547 | 0.000204 | 0.000709 | Up |
| KRT15 | 71.54173887 | 2.198066818 | 0.212678 | 10.33517 | 4.89E-25 | 1.31E-23 | Up |
| KRT17 | 157.4331167 | 3.336768121 | 0.297303 | 11.22345 | 3.13E-29 | 1.14E-27 | Up |
| KRT17P5 | 1.415864558 | -2.185945161 | 0.722477 | -3.02563 | 0.002481 | 0.006664 | Down |
| KRT18P11 | 2.072556201 | -1.523478378 | 0.370414 | -4.11291 | 3.91E-05 | 0.000156 | Down |
| KRT18P14 | 0.891956836 | -1.376469778 | 0.609397 | -2.25874 | 0.0239 | 0.048188 | Down |
| KRT18P19 | 2.715261664 | -1.668215837 | 0.285955 | -5.83384 | 5.42E-09 | 3.73E-08 | Down |
| KRT18P21 | 1.069319017 | -2.653936609 | 1.155015 | -2.29775 | 0.021576 | 0.04413 | Down |
| KRT18P26 | 2.025974783 | -1.021318961 | 0.299672 | -3.40812 | 0.000654 | 0.002026 | Down |
| KRT18P38 | 6.638823915 | -1.489779942 | 0.310814 | -4.79315 | 1.64E-06 | 8.19E-06 | Down |
| KRT18P40 | 1.214258366 | -2.463094222 | 0.792761 | -3.10698 | 0.00189 | 0.00525 | Down |
| KRT18P55 | 1.406208622 | -1.705877924 | 0.658552 | -2.59034 | 0.009588 | 0.02195 | Down |
| KRT18P59 | 5.136532646 | -1.437186775 | 0.280404 | -5.12542 | 2.97E-07 | 1.65E-06 | Down |
| KRT18P60 | 4.284752934 | -1.014498753 | 0.24805 | -4.08989 | 4.32E-05 | 0.000171 | Down |
| KRT18P62 | 4.230806972 | -1.182035354 | 0.279186 | -4.23386 | 2.30E-05 | 9.54E-05 | Down |
| KRT19 | 2716.175408 | 2.555087084 | 0.13163 | 19.41114 | 6.21E-84 | 3.15E-81 | Up |
| KRT19P2 | 10.20147148 | -1.358179307 | 0.24503 | -5.54291 | 2.97E-08 | 1.88E-07 | Down |
| KRT2 | 4.90430956 | -1.869316141 | 0.49954 | -3.74207 | 0.000183 | 0.00064 | Down |
| KRT20 | 1.128623446 | -1.959466496 | 0.806192 | -2.43052 | 0.015077 | 0.032458 | Down |
| KRT223P | 1.147031862 | -2.262820046 | 0.72962 | -3.10137 | 0.001926 | 0.005335 | Down |
| KRT24 | 1.652046363 | -1.502504964 | 0.558011 | -2.69261 | 0.00709 | 0.016848 | Down |
| KRT3 | 3.696330848 | -1.3224233 | 0.437697 | -3.02132 | 0.002517 | 0.006742 | Down |
| KRT38 | 1.166410833 | -2.58402549 | 0.893407 | -2.89233 | 0.003824 | 0.009775 | Down |
| KRT4 | 8.114550329 | -2.139017446 | 0.426376 | -5.01673 | 5.26E-07 | 2.82E-06 | Down |
| KRT6A | 20.20877119 | 1.123282496 | 0.387288 | 2.900384 | 0.003727 | 0.009563 | Up |
| KRT6C | 3.632645421 | -1.945492851 | 0.514644 | -3.78027 | 0.000157 | 0.000557 | Down |
| KRT71 | 1.618343746 | -1.587436593 | 0.579368 | -2.73995 | 0.006145 | 0.014857 | Down |
| KRT72 | 3.381990764 | -2.215222432 | 0.633231 | -3.49829 | 0.000468 | 0.0015 | Down |
| KRT73 | 2.535655734 | -1.7806246 | 0.535301 | -3.3264 | 0.00088 | 0.002646 | Down |
| KRT74 | 2.379375862 | -1.645010557 | 0.611977 | -2.68803 | 0.007188 | 0.017052 | Down |
| KRT78 | 5.58478322 | -1.362580775 | 0.427922 | -3.18418 | 0.001452 | 0.004147 | Down |
| KRT80 | 159.3768873 | 2.572988945 | 0.198322 | 12.97377 | 1.72E-38 | 1.09E-36 | Up |
| KRT85 | 4.661686738 | -2.463543917 | 0.293301 | -8.39937 | 4.49E-17 | 6.65E-16 | Down |
| KRT87P | 4.41741255 | -1.462151362 | 0.277177 | -5.27516 | 1.33E-07 | 7.71E-07 | Down |
| KRT89P | 4.234850225 | -1.349297987 | 0.24085 | -5.60224 | 2.12E-08 | 1.36E-07 | Down |
| KRT8P10 | 1.787097943 | -1.193588609 | 0.41989 | -2.84262 | 0.004474 | 0.011239 | Down |
| KRT8P11 | 2.394760755 | -1.060157133 | 0.427403 | -2.48046 | 0.013121 | 0.028849 | Down |
| KRT8P13 | 1.725054152 | -1.433389401 | 0.549473 | -2.60866 | 0.00909 | 0.020962 | Down |
| KRT8P21 | 1.325398844 | -2.697809506 | 0.748419 | -3.60468 | 0.000313 | 0.001042 | Down |
| KRT8P24 | 1.09238469 | -1.412102989 | 0.583099 | -2.42172 | 0.015447 | 0.033124 | Down |
| KRT8P32 | 1.562825875 | -1.268812249 | 0.515998 | -2.45895 | 0.013934 | 0.030364 | Down |
| KRT8P37 | 1.266759666 | -2.159725295 | 0.680156 | -3.17534 | 0.001497 | 0.004262 | Down |
| KRT8P42 | 2.988707462 | -1.51487732 | 0.321774 | -4.70789 | 2.50E-06 | 1.22E-05 | Down |
| KRTAP10-1 | 2.228231978 | -2.404937253 | 0.641393 | -3.74956 | 0.000177 | 0.000623 | Down |
| KRTAP10-10 | 2.080900722 | -2.16614793 | 0.452439 | -4.78772 | 1.69E-06 | 8.40E-06 | Down |
| KRTAP10-11 | 2.229915573 | -1.378806652 | 0.34923 | -3.94814 | 7.88E-05 | 0.000296 | Down |
| KRTAP10-12 | 2.169195667 | -2.08406549 | 0.459669 | -4.53384 | 5.79E-06 | 2.66E-05 | Down |
| KRTAP10-13P | 1.306662288 | -2.21798912 | 0.423697 | -5.23485 | 1.65E-07 | 9.48E-07 | Down |
| KRTAP10-2 | 1.890587215 | -1.666209218 | 0.443529 | -3.75671 | 0.000172 | 0.000607 | Down |
| KRTAP10-4 | 2.40079163 | -1.96443917 | 0.460702 | -4.26402 | 2.01E-05 | 8.43E-05 | Down |
| KRTAP10-5 | 2.024239415 | -2.213317489 | 0.749338 | -2.9537 | 0.00314 | 0.008216 | Down |
| KRTAP10-7 | 2.256057494 | -1.711306388 | 0.434425 | -3.93924 | 8.17E-05 | 0.000306 | Down |
| KRTAP10-8 | 1.420548014 | -1.857427165 | 0.541552 | -3.42982 | 0.000604 | 0.001885 | Down |
| KRTAP10-9 | 1.719509806 | -1.184802216 | 0.345639 | -3.42786 | 0.000608 | 0.001898 | Down |
| KRTAP12-1 | 1.020344882 | -2.112125091 | 0.55002 | -3.84008 | 0.000123 | 0.000445 | Down |
| KRTAP12-2 | 1.317612629 | -1.605358472 | 0.404639 | -3.96739 | 7.27E-05 | 0.000274 | Down |
| KRTAP13-4 | 1.086521872 | -2.497616389 | 0.829542 | -3.01084 | 0.002605 | 0.006951 | Down |
| KRTAP16-1 | 2.622996647 | -2.180248452 | 0.621753 | -3.50662 | 0.000454 | 0.001458 | Down |
| KRTAP2-1 | 1.330673141 | -2.825887957 | 0.866439 | -3.2615 | 0.001108 | 0.003257 | Down |
| KRTAP29-1 | 1.29890465 | -1.91197716 | 0.644153 | -2.9682 | 0.002995 | 0.007877 | Down |
| KRTAP3-3 | 1.487526174 | -2.344509947 | 0.957159 | -2.44945 | 0.014308 | 0.031039 | Down |
| KRTAP4-11 | 1.471077672 | -2.029180994 | 0.691807 | -2.93316 | 0.003355 | 0.008711 | Down |
| KRTAP4-12 | 1.173138233 | -1.657042582 | 0.673628 | -2.45988 | 0.013898 | 0.030295 | Down |
| KRTAP4-6 | 1.419962142 | -2.042921398 | 0.74843 | -2.72961 | 0.006341 | 0.015286 | Down |
| KRTAP4-7 | 0.838313889 | -1.362698171 | 0.605095 | -2.25204 | 0.02432 | 0.048918 | Down |
| KRTAP4-8 | 1.128913851 | -2.352397959 | 0.876557 | -2.68368 | 0.007282 | 0.017242 | Down |
| KRTAP4-9 | 1.471487592 | -1.742016457 | 0.653657 | -2.66503 | 0.007698 | 0.018114 | Down |
| KRTAP5-2 | 3.188274425 | 1.116784211 | 0.350468 | 3.186547 | 0.00144 | 0.004117 | Up |
| KRTAP5-4 | 1.948271895 | -2.324167557 | 0.664719 | -3.49646 | 0.000471 | 0.001509 | Down |
| KRTAP5-8 | 2.294997483 | -1.001859121 | 0.4235 | -2.36566 | 0.017998 | 0.037767 | Down |
| KRTAP9-8 | 1.584915967 | -2.448988501 | 0.788526 | -3.10578 | 0.001898 | 0.005268 | Down |
| KY | 188.5283703 | -1.784835597 | 0.122193 | -14.6066 | 2.55E-48 | 2.95E-46 | Down |
| L1TD1 | 31.98226888 | -1.0914419 | 0.245265 | -4.45006 | 8.58E-06 | 3.83E-05 | Down |
| L29074.3 | 0.551484195 | -1.112110053 | 0.447295 | -2.4863 | 0.012908 | 0.028457 | Down |
| L3MBTL4 | 393.1410555 | -1.549212132 | 0.105257 | -14.7184 | 4.91E-49 | 5.81E-47 | Down |
| LA16c-306A4.1 | 4.447279361 | -1.466035105 | 0.287234 | -5.10397 | 3.33E-07 | 1.83E-06 | Down |
| LA16c-325D7.2 | 2.873004454 | -1.277296165 | 0.334687 | -3.81639 | 0.000135 | 0.000486 | Down |
| LA16c-329F2.1 | 36.95177201 | -2.436356657 | 0.174642 | -13.9506 | 3.12E-44 | 2.83E-42 | Down |
| LA16c-352F7.1 | 10.26197804 | -1.0773294 | 0.221238 | -4.86955 | 1.12E-06 | 5.73E-06 | Down |
| LA16c-380A1.2 | 8.730110849 | -1.318378315 | 0.19759 | -6.67228 | 2.52E-11 | 2.21E-10 | Down |
| LA16c-380F5.1 | 22.8492902 | -1.214495244 | 0.182685 | -6.64803 | 2.97E-11 | 2.59E-10 | Down |
| LA16c-380H5.2 | 1.337657021 | 1.937476805 | 0.387284 | 5.00273 | 5.65E-07 | 3.01E-06 | Up |
| LA16c-380H5.4 | 0.899310696 | 1.697189241 | 0.496648 | 3.41729 | 0.000632 | 0.001965 | Up |
| LA16c-380H5.5 | 28.00896577 | 1.613790567 | 0.187833 | 8.591607 | 8.58E-18 | 1.35E-16 | Up |
| LA16c-380H5.6 | 2.393531272 | 1.569681718 | 0.295915 | 5.304505 | 1.13E-07 | 6.62E-07 | Up |
| LA16c-425C2.1 | 1.70718917 | -1.311496752 | 0.280075 | -4.68267 | 2.83E-06 | 1.37E-05 | Down |
| LA16c-60G3.7 | 1.704865217 | -2.915725314 | 1.131187 | -2.57758 | 0.009949 | 0.022655 | Down |
| LACTBL1 | 1.841092827 | -3.059998572 | 0.831053 | -3.68207 | 0.000231 | 0.000794 | Down |
| LAD1 | 438.4442076 | 2.641557668 | 0.110439 | 23.91867 | 1.96E-126 | 5.02E-123 | Up |
| LAMA5-AS1 | 5.831733473 | -1.427250527 | 0.184439 | -7.73832 | 1.01E-14 | 1.24E-13 | Down |
| LAMB3 | 2268.294545 | 4.333302042 | 0.178947 | 24.21557 | 1.53E-129 | 4.82E-126 | Up |
| LAMB4 | 7.894413255 | -1.375623342 | 0.231375 | -5.94542 | 2.76E-09 | 1.97E-08 | Down |
| LAMC2 | 4041.616747 | 1.693129152 | 0.104186 | 16.25105 | 2.20E-59 | 4.33E-57 | Up |
| LAMC3 | 41.97355103 | -1.310932861 | 0.192512 | -6.80963 | 9.78E-12 | 8.97E-11 | Down |
| LAMP3 | 299.6877867 | 1.00179567 | 0.234641 | 4.269478 | 1.96E-05 | 8.24E-05 | Up |
| LAMP5 | 258.4493067 | 3.593238313 | 0.219438 | 16.37472 | 2.90E-60 | 5.89E-58 | Up |
| LATS2-AS1 | 4.505309471 | -1.147330296 | 0.267532 | -4.28857 | 1.80E-05 | 7.61E-05 | Down |
| LAYN | 481.5736915 | -1.54569377 | 0.115274 | -13.4089 | 5.37E-41 | 4.00E-39 | Down |
| LBX1-AS1 | 2.10169667 | -2.122181071 | 0.83014 | -2.55641 | 0.010576 | 0.023886 | Down |
| LCA5 | 967.6340043 | 1.122072074 | 0.079596 | 14.09701 | 3.96E-45 | 3.84E-43 | Up |
| LCE1B | 1.410419673 | -1.806097825 | 0.700718 | -2.57749 | 0.009952 | 0.022659 | Down |
| LCN10 | 382.49503 | 1.156188712 | 0.189058 | 6.115527 | 9.62E-10 | 7.24E-09 | Up |
| LCN15 | 1.3781745 | -1.78821316 | 0.72798 | -2.4564 | 0.014034 | 0.030528 | Down |
| LCN2 | 157.0722487 | 4.071846055 | 0.237117 | 17.17232 | 4.28E-66 | 1.06E-63 | Up |
| LCN6 | 163.9526459 | 3.372319713 | 0.253293 | 13.31388 | 1.92E-40 | 1.37E-38 | Up |
| LCNL1 | 15.4771171 | -1.80850762 | 0.349207 | -5.1789 | 2.23E-07 | 1.26E-06 | Down |
| LCT | 6.322829388 | -1.046492134 | 0.284474 | -3.67869 | 0.000234 | 0.000804 | Down |
| LDB3 | 76.88372185 | -1.092052663 | 0.163636 | -6.67368 | 2.49E-11 | 2.19E-10 | Down |
| LDHAL6A | 7.64540811 | -1.024869113 | 0.23121 | -4.43263 | 9.31E-06 | 4.13E-05 | Down |
| LDHAL6DP | 1.256536454 | -1.176152692 | 0.456049 | -2.57901 | 0.009909 | 0.022577 | Down |
| LDHAL6EP | 3.710364181 | -2.462482924 | 0.319595 | -7.70502 | 1.31E-14 | 1.59E-13 | Down |
| LDHAP3 | 2.195514483 | -2.433462393 | 0.718818 | -3.38537 | 0.000711 | 0.002183 | Down |
| LDHAP5 | 1.305616792 | -1.469919804 | 0.625133 | -2.35137 | 0.018704 | 0.039008 | Down |
| LDHAP7 | 2.426839742 | -1.387147011 | 0.421663 | -3.28971 | 0.001003 | 0.002977 | Down |
| LDHBP1 | 1.698882809 | -2.09144643 | 0.523037 | -3.99866 | 6.37E-05 | 0.000243 | Down |
| LDHD | 236.2690495 | -1.418766354 | 0.101452 | -13.9846 | 1.94E-44 | 1.79E-42 | Down |
| LDLR | 577.3452108 | 1.694519748 | 0.13498 | 12.5539 | 3.78E-36 | 2.11E-34 | Up |
| LDLRAD1 | 3.34281827 | -2.790954103 | 0.48315 | -5.77658 | 7.62E-09 | 5.17E-08 | Down |
| LECT1 | 38.48762195 | -2.29922039 | 0.225319 | -10.2043 | 1.90E-24 | 4.86E-23 | Down |
| LEFTY1 | 3.447962562 | -1.27245568 | 0.359086 | -3.5436 | 0.000395 | 0.001287 | Down |
| LEFTY2 | 6.054500099 | -1.562767827 | 0.302512 | -5.16597 | 2.39E-07 | 1.34E-06 | Down |
| LEMD1 | 53.30889116 | 2.315787325 | 0.131189 | 17.65234 | 9.76E-70 | 2.86E-67 | Up |
| LEP | 16.2533579 | -3.151857646 | 0.383821 | -8.2118 | 2.18E-16 | 3.04E-15 | Down |
| LGALS1 | 2430.459845 | 1.234894135 | 0.098596 | 12.52478 | 5.46E-36 | 3.03E-34 | Up |
| LGALS12 | 8.845354139 | -1.188381151 | 0.255742 | -4.64679 | 3.37E-06 | 1.61E-05 | Down |
| LGALS3 | 2529.469677 | 2.268926306 | 0.096949 | 23.40321 | 3.96E-121 | 8.14E-118 | Up |
| LGALS9B | 2.988575328 | -1.349506466 | 0.538 | -2.50838 | 0.012129 | 0.026948 | Down |
| LGALS9C | 5.380349214 | -1.204144831 | 0.351415 | -3.42656 | 0.000611 | 0.001906 | Down |
| LGI1 | 12.08641728 | -2.014968771 | 0.289074 | -6.97043 | 3.16E-12 | 3.04E-11 | Down |
| LGI3 | 240.3491081 | -2.051246903 | 0.146883 | -13.9652 | 2.54E-44 | 2.32E-42 | Down |
| LGI4 | 129.7188315 | -1.182967514 | 0.128822 | -9.18297 | 4.19E-20 | 7.87E-19 | Down |
| LHCGR | 17.78200287 | -1.908906177 | 0.258395 | -7.38756 | 1.50E-13 | 1.64E-12 | Down |
| LHFPL3 | 2.264469892 | -1.697905555 | 0.544974 | -3.11557 | 0.001836 | 0.005115 | Down |
| LHFPL3-AS1 | 1.423083181 | -2.447323403 | 0.78022 | -3.13671 | 0.001709 | 0.004801 | Down |
| LHFPL3-AS2 | 3.717574854 | -1.678216897 | 0.388093 | -4.32426 | 1.53E-05 | 6.56E-05 | Down |
| LHX3 | 1.520476325 | -2.894723159 | 0.905474 | -3.19691 | 0.001389 | 0.003987 | Down |
| LIF | 197.0250929 | 1.106895289 | 0.119047 | 9.297978 | 1.43E-20 | 2.78E-19 | Up |
| LIFR | 4522.885322 | -1.958182279 | 0.125448 | -15.6095 | 6.27E-55 | 1.01E-52 | Down |
| LIFR-AS1 | 110.9008466 | -1.477015066 | 0.097602 | -15.1331 | 9.80E-52 | 1.30E-49 | Down |
| LIMK1 | 946.2420031 | 1.021665659 | 0.073374 | 13.92416 | 4.52E-44 | 4.07E-42 | Up |
| LIMS3L | 28.68737559 | 1.399517357 | 0.232223 | 6.026618 | 1.67E-09 | 1.23E-08 | Up |
| LINC-ROR | 1.918041893 | -1.621062912 | 0.621077 | -2.61008 | 0.009052 | 0.020882 | Down |
| LINC00029 | 1.048079684 | -2.199334635 | 0.906006 | -2.42751 | 0.015203 | 0.032695 | Down |
| LINC00032 | 4.045942039 | -1.757801 | 0.257444 | -6.82791 | 8.62E-12 | 7.95E-11 | Down |
| LINC00086 | 979.0179352 | 1.761953633 | 0.144617 | 12.18358 | 3.80E-34 | 1.86E-32 | Up |
| LINC00092 | 89.40443682 | -1.537864556 | 0.137065 | -11.2199 | 3.25E-29 | 1.18E-27 | Down |
| LINC00102 | 5.761644826 | -1.40316781 | 0.299363 | -4.68719 | 2.77E-06 | 1.34E-05 | Down |
| LINC00113 | 1.066035339 | 1.607967435 | 0.493172 | 3.260462 | 0.001112 | 0.003268 | Up |
| LINC00152 | 255.9098101 | 1.187570602 | 0.098217 | 12.09135 | 1.17E-33 | 5.59E-32 | Up |
| LINC00158 | 4.544972622 | -1.369499328 | 0.347077 | -3.94581 | 7.95E-05 | 0.000298 | Down |
| LINC00163 | 2.164662265 | -1.240273659 | 0.475199 | -2.61001 | 0.009054 | 0.020886 | Down |
| LINC00167 | 6.362781783 | -1.316321853 | 0.17819 | -7.38718 | 1.50E-13 | 1.64E-12 | Down |
| LINC00173 | 8.322462484 | -1.550835474 | 0.284114 | -5.4585 | 4.80E-08 | 2.95E-07 | Down |
| LINC00184 | 2.551793916 | -1.827776354 | 0.51629 | -3.54021 | 0.0004 | 0.001302 | Down |
| LINC00200 | 2.392427413 | -1.795142348 | 0.777724 | -2.3082 | 0.020988 | 0.043127 | Down |
| LINC00202-1 | 14.92566101 | -1.189729701 | 0.260222 | -4.57198 | 4.83E-06 | 2.25E-05 | Down |
| LINC00211 | 3.531651042 | -1.219842018 | 0.426532 | -2.85991 | 0.004238 | 0.010708 | Down |
| LINC00222 | 6.934875616 | -1.078107931 | 0.2832 | -3.80688 | 0.000141 | 0.000504 | Down |
| LINC00237 | 2.148014211 | -2.671451983 | 0.420206 | -6.35748 | 2.05E-10 | 1.66E-09 | Down |
| LINC00261 | 20.26061171 | -2.739247512 | 0.372026 | -7.36305 | 1.80E-13 | 1.95E-12 | Down |
| LINC00269 | 1.339620834 | -1.901621767 | 0.73412 | -2.59034 | 0.009588 | 0.02195 | Down |
| LINC00276 | 2.61877382 | -1.786614755 | 0.647358 | -2.75985 | 0.005783 | 0.014082 | Down |
| LINC00282 | 14.43725857 | -1.365586944 | 0.318057 | -4.29353 | 1.76E-05 | 7.46E-05 | Down |
| LINC00284 | 32.29736528 | 1.827445252 | 0.276977 | 6.597825 | 4.17E-11 | 3.59E-10 | Up |
| LINC00298 | 1.083379466 | -1.540845382 | 0.592474 | -2.6007 | 0.009303 | 0.021377 | Down |
| LINC00303 | 1.232395387 | -1.682287406 | 0.603124 | -2.78929 | 0.005282 | 0.013017 | Down |
| LINC00304 | 8.939119545 | -1.694099739 | 0.299455 | -5.65728 | 1.54E-08 | 1.01E-07 | Down |
| LINC00309 | 2.556984833 | -2.575877388 | 0.59532 | -4.32688 | 1.51E-05 | 6.49E-05 | Down |
| LINC00311 | 2.795186962 | -1.248609762 | 0.36199 | -3.4493 | 0.000562 | 0.001767 | Down |
| LINC00314 | 0.733536251 | -2.144715512 | 0.49101 | -4.36797 | 1.25E-05 | 5.45E-05 | Down |
| LINC00315 | 8.550508541 | -1.307719006 | 0.208825 | -6.26227 | 3.79E-10 | 2.98E-09 | Down |
| LINC00319 | 5.943214297 | -1.636938003 | 0.478215 | -3.42302 | 0.000619 | 0.001928 | Down |
| LINC00323 | 5.429355511 | -1.592206741 | 0.301633 | -5.27862 | 1.30E-07 | 7.57E-07 | Down |
| LINC00330 | 2.427734068 | -3.656434595 | 0.898872 | -4.06781 | 4.75E-05 | 0.000186 | Down |
| LINC00332 | 1.305720865 | -2.549418911 | 0.767666 | -3.321 | 0.000897 | 0.002694 | Down |
| LINC00337 | 2.382941498 | -1.540484279 | 0.552472 | -2.78835 | 0.005298 | 0.013051 | Down |
| LINC00355 | 1.549087354 | -3.014799135 | 1.154535 | -2.61127 | 0.009021 | 0.020823 | Down |
| LINC00359 | 1.441161001 | -1.155411122 | 0.430066 | -2.68659 | 0.007219 | 0.017112 | Down |
| LINC00371 | 1.826249772 | -2.479218502 | 0.758394 | -3.26904 | 0.001079 | 0.00318 | Down |
| LINC00377 | 2.124129154 | -1.939269865 | 0.335511 | -5.78005 | 7.47E-09 | 5.07E-08 | Down |
| LINC00402 | 24.06755569 | -1.15080486 | 0.476361 | -2.41583 | 0.0157 | 0.033571 | Down |
| LINC00415 | 1.224334277 | -1.79018228 | 0.411598 | -4.34935 | 1.37E-05 | 5.90E-05 | Down |
| LINC00423 | 1.32184561 | 1.564466622 | 0.578551 | 2.704114 | 0.006849 | 0.016357 | Up |
| LINC00437 | 0.509371484 | -1.899581323 | 0.631619 | -3.00748 | 0.002634 | 0.00702 | Down |
| LINC00441 | 7.601306059 | -1.0239503 | 0.246807 | -4.1488 | 3.34E-05 | 0.000135 | Down |
| LINC00449 | 2.97372013 | -1.787876054 | 0.369633 | -4.8369 | 1.32E-06 | 6.68E-06 | Down |
| LINC00452 | 2.631771886 | -2.022635391 | 0.563834 | -3.58729 | 0.000334 | 0.001107 | Down |
| LINC00470 | 6.02393481 | -1.60201996 | 0.470869 | -3.40227 | 0.000668 | 0.002066 | Down |
| LINC00473 | 46.63577011 | -2.704274746 | 0.318073 | -8.50206 | 1.86E-17 | 2.85E-16 | Down |
| LINC00475 | 9.320310706 | 1.162105307 | 0.263153 | 4.416075 | 1.01E-05 | 4.44E-05 | Up |
| LINC00477 | 1.862819746 | -1.653933014 | 0.688615 | -2.40183 | 0.016313 | 0.034701 | Down |
| LINC00479 | 1.893312697 | -1.367342892 | 0.561845 | -2.43366 | 0.014947 | 0.032221 | Down |
| LINC00485 | 3.80584359 | -1.464094746 | 0.617132 | -2.37242 | 0.017672 | 0.037169 | Down |
| LINC00487 | 5.508867917 | -1.004520559 | 0.349292 | -2.87588 | 0.004029 | 0.01024 | Down |
| LINC00488 | 1.606091232 | -2.651589172 | 0.889866 | -2.97976 | 0.002885 | 0.007612 | Down |
| LINC00491 | 1.877875518 | -2.114232047 | 0.620577 | -3.40688 | 0.000657 | 0.002035 | Down |
| LINC00494 | 15.29573896 | -1.251238484 | 0.447867 | -2.79378 | 0.00521 | 0.012856 | Down |
| LINC00506 | 22.58212882 | -1.274958088 | 0.185341 | -6.879 | 6.03E-12 | 5.65E-11 | Down |
| LINC00511 | 43.70212162 | 1.571967436 | 0.258945 | 6.070668 | 1.27E-09 | 9.46E-09 | Up |
| LINC00517 | 9.653097094 | -1.951197121 | 0.298606 | -6.53436 | 6.39E-11 | 5.42E-10 | Down |
| LINC00521 | 1.68496401 | -1.914923595 | 0.81744 | -2.34259 | 0.019151 | 0.03981 | Down |
| LINC00536 | 2.343112701 | -1.747391784 | 0.389901 | -4.48163 | 7.41E-06 | 3.34E-05 | Down |
| LINC00538 | 4.021046176 | -2.410277706 | 0.358693 | -6.71961 | 1.82E-11 | 1.62E-10 | Down |
| LINC00543 | 2.935471763 | -2.404893243 | 0.41501 | -5.79479 | 6.84E-09 | 4.66E-08 | Down |
| LINC00548 | 3.535643716 | -1.557666185 | 0.397913 | -3.91459 | 9.06E-05 | 0.000336 | Down |
| LINC00551 | 1.344791569 | -2.10724305 | 0.911164 | -2.31269 | 0.020739 | 0.042684 | Down |
| LINC00552 | 1.956814482 | -1.400919901 | 0.501645 | -2.79265 | 0.005228 | 0.012895 | Down |
| LINC00562 | 8.749405494 | -1.183704736 | 0.224662 | -5.26883 | 1.37E-07 | 7.97E-07 | Down |
| LINC00563 | 3.63279774 | -1.258930386 | 0.426661 | -2.95066 | 0.003171 | 0.008285 | Down |
| LINC00570 | 2.147056404 | -2.526294278 | 0.612032 | -4.12772 | 3.66E-05 | 0.000147 | Down |
| LINC00596 | 2.13788907 | -1.479062304 | 0.52887 | -2.79665 | 0.005164 | 0.012756 | Down |
| LINC00598 | 8.229235201 | -1.251301452 | 0.198711 | -6.2971 | 3.03E-10 | 2.41E-09 | Down |
| LINC00602 | 4.502296906 | -2.797470844 | 0.380198 | -7.35793 | 1.87E-13 | 2.02E-12 | Down |
| LINC00605 | 5.85288941 | -1.085483794 | 0.234814 | -4.62273 | 3.79E-06 | 1.79E-05 | Down |
| LINC00607 | 22.06362999 | 1.088968247 | 0.20758 | 5.246026 | 1.55E-07 | 8.95E-07 | Up |
| LINC00612 | 22.65005087 | -1.270537726 | 0.195719 | -6.49165 | 8.49E-11 | 7.12E-10 | Down |
| LINC00618 | 1.075388405 | -1.215829292 | 0.378162 | -3.2151 | 0.001304 | 0.00377 | Down |
| LINC00626 | 1.847950642 | -1.169138524 | 0.37971 | -3.07903 | 0.002077 | 0.005699 | Down |
| LINC00629 | 3.790639637 | -1.18902802 | 0.206396 | -5.76092 | 8.37E-09 | 5.65E-08 | Down |
| LINC00635 | 1.539640328 | -1.922324537 | 0.745544 | -2.57842 | 0.009925 | 0.022613 | Down |
| LINC00636 | 5.368913121 | -1.616856071 | 0.216568 | -7.46582 | 8.28E-14 | 9.28E-13 | Down |
| LINC00637 | 6.269480466 | -1.26460342 | 0.299049 | -4.22875 | 2.35E-05 | 9.74E-05 | Down |
| LINC00640 | 2.108050654 | -1.211155181 | 0.386453 | -3.13403 | 0.001724 | 0.004841 | Down |
| LINC00642 | 1.630335624 | -2.599070129 | 1.047099 | -2.48216 | 0.013059 | 0.028737 | Down |
| LINC00643 | 5.130248586 | -2.073282808 | 0.568917 | -3.64426 | 0.000268 | 0.000908 | Down |
| LINC00648 | 4.915694216 | -1.452544506 | 0.459056 | -3.1642 | 0.001555 | 0.004413 | Down |
| LINC00652 | 12.33593604 | -2.085410262 | 0.214428 | -9.72547 | 2.35E-22 | 5.20E-21 | Down |
| LINC00661 | 1.422956145 | -2.222422089 | 0.907599 | -2.44868 | 0.014338 | 0.031093 | Down |
| LINC00671 | 5.237111047 | -1.862649523 | 0.303154 | -6.14425 | 8.03E-10 | 6.10E-09 | Down |
| LINC00675 | 3.42958928 | -1.184341362 | 0.308118 | -3.84379 | 0.000121 | 0.000439 | Down |
| LINC00685 | 2.163849879 | -1.1953035 | 0.364302 | -3.28107 | 0.001034 | 0.003062 | Down |
| LINC00689 | 21.2779972 | -1.767703263 | 0.279288 | -6.32932 | 2.46E-10 | 1.97E-09 | Down |
| LINC00690 | 1.299901903 | -2.708209092 | 1.177489 | -2.29999 | 0.021449 | 0.043915 | Down |
| LINC00691 | 7.03137242 | -1.601109508 | 0.353902 | -4.52416 | 6.06E-06 | 2.77E-05 | Down |
| LINC00693 | 19.78989215 | -1.505548295 | 0.183207 | -8.21776 | 2.07E-16 | 2.90E-15 | Down |
| LINC00694 | 2.400511442 | -1.653677085 | 0.400997 | -4.12391 | 3.72E-05 | 0.000149 | Down |
| LINC00698 | 1.42534942 | -2.530932579 | 0.863171 | -2.93213 | 0.003366 | 0.008736 | Down |
| LINC00703 | 1.566649884 | -2.562933371 | 0.885606 | -2.89399 | 0.003804 | 0.009732 | Down |
| LINC00710 | 8.237304527 | -1.904896402 | 0.273318 | -6.96953 | 3.18E-12 | 3.06E-11 | Down |
| LINC00838 | 1.641340027 | -2.857022289 | 1.261159 | -2.26539 | 0.023488 | 0.047457 | Down |
| LINC00839 | 12.4202725 | -1.03061742 | 0.163465 | -6.30481 | 2.89E-10 | 2.30E-09 | Down |
| LINC00844 | 1.681979327 | -1.569044624 | 0.386174 | -4.06305 | 4.84E-05 | 0.000189 | Down |
| LINC00846 | 2.525715455 | -1.600172534 | 0.408672 | -3.91555 | 9.02E-05 | 0.000335 | Down |
| LINC00862 | 2.380374302 | -1.145478538 | 0.413761 | -2.76846 | 0.005632 | 0.013763 | Down |
| LINC00864 | 1.400820983 | -1.946584552 | 0.859204 | -2.26557 | 0.023478 | 0.04744 | Down |
| LINC00867 | 0.924647005 | -1.457687583 | 0.516646 | -2.82144 | 0.004781 | 0.011922 | Down |
| LINC00880 | 6.793856941 | -1.390138779 | 0.287443 | -4.83623 | 1.32E-06 | 6.70E-06 | Down |
| LINC00881 | 10.32212107 | -1.314198608 | 0.230448 | -5.70279 | 1.18E-08 | 7.83E-08 | Down |
| LINC00885 | 2.914437384 | -2.20982841 | 0.372914 | -5.92583 | 3.11E-09 | 2.20E-08 | Down |
| LINC00886 | 141.9981026 | -1.086437959 | 0.119261 | -9.10977 | 8.26E-20 | 1.52E-18 | Down |
| LINC00887 | 16.84575793 | 1.070567208 | 0.316115 | 3.386634 | 0.000708 | 0.002175 | Up |
| LINC00891 | 312.761792 | 2.040551722 | 0.163834 | 12.45497 | 1.31E-35 | 7.08E-34 | Up |
| LINC00895 | 1.145585616 | -2.31055701 | 0.900579 | -2.56564 | 0.010299 | 0.023331 | Down |
| LINC00896 | 35.65424476 | -1.091402873 | 0.150804 | -7.23724 | 4.58E-13 | 4.77E-12 | Down |
| LINC00901 | 2.297387182 | -1.517541107 | 0.529535 | -2.8658 | 0.00416 | 0.01053 | Down |
| LINC00923 | 29.52716131 | -1.07477115 | 0.231759 | -4.63744 | 3.53E-06 | 1.68E-05 | Down |
| LINC00924 | 31.19064404 | -1.173663066 | 0.168644 | -6.9594 | 3.42E-12 | 3.27E-11 | Down |
| LINC00926 | 105.8680872 | -1.167474626 | 0.329205 | -3.54635 | 0.000391 | 0.001275 | Down |
| LINC00930 | 7.816761829 | -1.222446821 | 0.24117 | -5.06882 | 4.00E-07 | 2.18E-06 | Down |
| LINC00934 | 1.443611914 | -1.55256799 | 0.641214 | -2.42129 | 0.015465 | 0.033156 | Down |
| LINC00939 | 4.070399127 | -1.12966276 | 0.422122 | -2.67616 | 0.007447 | 0.017585 | Down |
| LINC00940 | 3.057948022 | -2.015366342 | 0.41431 | -4.86439 | 1.15E-06 | 5.87E-06 | Down |
| LINC00943 | 10.8860729 | -1.498971126 | 0.354777 | -4.22511 | 2.39E-05 | 9.89E-05 | Down |
| LINC00948 | 2.892060601 | -1.887543622 | 0.682448 | -2.76584 | 0.005678 | 0.013861 | Down |
| LINC00958 | 89.25178618 | 1.169797052 | 0.128519 | 9.102167 | 8.85E-20 | 1.62E-18 | Up |
| LINC00964 | 2.675752978 | -1.239245346 | 0.526898 | -2.35197 | 0.018674 | 0.038964 | Down |
| LINC00967 | 2.509047446 | -3.344383063 | 1.151239 | -2.90503 | 0.003672 | 0.009436 | Down |
| LINC00968 | 6.851337536 | -1.399544231 | 0.28388 | -4.93006 | 8.22E-07 | 4.28E-06 | Down |
| LINC00973 | 4.051859376 | 1.797307239 | 0.508275 | 3.536094 | 0.000406 | 0.00132 | Up |
| LINC00976 | 1.541135108 | -2.770297933 | 0.787789 | -3.51655 | 0.000437 | 0.00141 | Down |
| LINC00977 | 1.293399248 | -2.307827103 | 0.735013 | -3.13985 | 0.00169 | 0.004759 | Down |
| LINC00982 | 206.2791624 | -1.525687756 | 0.136216 | -11.2005 | 4.06E-29 | 1.47E-27 | Down |
| LINC00994 | 3.226602873 | -1.610852919 | 0.490904 | -3.2814 | 0.001033 | 0.00306 | Down |
| LINC01010 | 17.20334756 | 1.386656106 | 0.307222 | 4.513529 | 6.38E-06 | 2.91E-05 | Up |
| LINC01013 | 10.09371878 | -1.325028393 | 0.455065 | -2.91174 | 0.003594 | 0.00926 | Down |
| LINC01014 | 2.718506508 | -2.500833265 | 0.258899 | -9.6595 | 4.48E-22 | 9.69E-21 | Down |
| LINC01028 | 1.435034615 | -1.592955463 | 0.529688 | -3.00735 | 0.002635 | 0.007022 | Down |
| LINC01031 | 0.668909747 | -1.352805893 | 0.561068 | -2.41113 | 0.015903 | 0.033948 | Down |
| LINC01059 | 7.029242536 | -1.091815398 | 0.321001 | -3.40128 | 0.000671 | 0.002073 | Down |
| LINC01065 | 0.660711391 | -1.624367615 | 0.461955 | -3.51629 | 0.000438 | 0.001411 | Down |
| LINC01085 | 1.075417666 | -1.928167302 | 0.796197 | -2.42172 | 0.015447 | 0.033124 | Down |
| LINC01094 | 47.41396293 | 1.311470866 | 0.167445 | 7.832239 | 4.79E-15 | 6.06E-14 | Up |
| LINC01102 | 4.155832511 | -2.369123208 | 0.547062 | -4.33063 | 1.49E-05 | 6.38E-05 | Down |
| LINC01124 | 5.698030676 | -1.045997786 | 0.245838 | -4.25482 | 2.09E-05 | 8.75E-05 | Down |
| LINC01126 | 38.37041038 | -1.492910981 | 0.16519 | -9.03754 | 1.60E-19 | 2.88E-18 | Down |
| LINC01133 | 7.175602929 | -1.770579937 | 0.262175 | -6.75342 | 1.44E-11 | 1.30E-10 | Down |
| LINC01135 | 23.8205348 | -1.087610117 | 0.111573 | -9.748 | 1.88E-22 | 4.19E-21 | Down |
| LINC01136 | 8.344955609 | -1.474070261 | 0.285052 | -5.17122 | 2.33E-07 | 1.31E-06 | Down |
| LINC01142 | 2.696209872 | -2.008383513 | 0.420165 | -4.77999 | 1.75E-06 | 8.71E-06 | Down |
| LINC01144 | 45.18249295 | -1.060397849 | 0.099 | -10.7111 | 9.03E-27 | 2.74E-25 | Down |
| LINC01152 | 32.27889464 | -1.197693738 | 0.175756 | -6.81452 | 9.46E-12 | 8.69E-11 | Down |
| LINC01153 | 2.38650856 | -1.530391315 | 0.513367 | -2.98109 | 0.002872 | 0.007582 | Down |
| LINC01165 | 1.437589764 | -1.70588381 | 0.42438 | -4.01971 | 5.83E-05 | 0.000224 | Down |
| LINC01166 | 4.902091189 | -2.560803151 | 0.381098 | -6.71954 | 1.82E-11 | 1.62E-10 | Down |
| LINC01170 | 2.332892859 | 2.30891962 | 0.362766 | 6.364757 | 1.96E-10 | 1.58E-09 | Up |
| LINC01180 | 1.364941113 | -3.062944254 | 1.274376 | -2.40348 | 0.01624 | 0.03456 | Down |
| LINC01185 | 1.539799517 | -2.670841989 | 0.576137 | -4.63578 | 3.56E-06 | 1.69E-05 | Down |
| LINC01191 | 1.035847611 | -1.344547478 | 0.511373 | -2.62929 | 0.008556 | 0.019881 | Down |
| LINC01198 | 1.662254859 | -3.022918912 | 1.112926 | -2.71619 | 0.006604 | 0.015842 | Down |
| LINC01208 | 0.854475134 | 1.351638287 | 0.435762 | 3.101784 | 0.001924 | 0.005329 | Up |
| LINC01210 | 1.6447602 | -3.240516852 | 1.187984 | -2.72775 | 0.006377 | 0.01536 | Down |
| LINC01213 | 0.850890668 | -1.249293788 | 0.374976 | -3.33166 | 0.000863 | 0.002601 | Down |
| LINC01220 | 7.51984003 | -1.064461426 | 0.325651 | -3.26872 | 0.00108 | 0.003183 | Down |
| LINC01225 | 4.083764466 | -1.647432708 | 0.533261 | -3.08935 | 0.002006 | 0.005532 | Down |
| LINC01226 | 3.609470493 | -1.830514999 | 0.590517 | -3.09985 | 0.001936 | 0.005358 | Down |
| LINC01227 | 1.333621595 | -2.683500703 | 1.035026 | -2.59269 | 0.009523 | 0.021829 | Down |
| LINC01229 | 4.805476291 | -1.5699694 | 0.245529 | -6.39424 | 1.61E-10 | 1.32E-09 | Down |
| LINC01249 | 1.600165225 | -2.487892086 | 1.104967 | -2.25155 | 0.024351 | 0.048965 | Down |
| LINC01257 | 7.879594017 | -2.647146798 | 0.310589 | -8.52299 | 1.55E-17 | 2.39E-16 | Down |
| LINC01258 | 2.024799221 | -2.572252704 | 0.513349 | -5.01073 | 5.42E-07 | 2.90E-06 | Down |
| LINC01277 | 6.69683074 | -1.260726915 | 0.240081 | -5.25125 | 1.51E-07 | 8.72E-07 | Down |
| LINC01301 | 14.87828515 | -1.461467267 | 0.205503 | -7.11166 | 1.15E-12 | 1.15E-11 | Down |
| LINC01307 | 0.765110213 | -1.688789171 | 0.711761 | -2.37269 | 0.017659 | 0.037148 | Down |
| LINC01310 | 1.839527292 | -2.649361448 | 0.995623 | -2.66101 | 0.007791 | 0.018299 | Down |
| LINC01314 | 362.252479 | 2.910554082 | 0.247071 | 11.78024 | 4.93E-32 | 2.17E-30 | Up |
| LINC01342 | 1.568665473 | -2.144940935 | 0.700477 | -3.06211 | 0.002198 | 0.005992 | Down |
| LINC01344 | 1.457636395 | -2.498750122 | 0.618282 | -4.04144 | 5.31E-05 | 0.000206 | Down |
| LINC01348 | 10.9221141 | -1.442891452 | 0.243491 | -5.92585 | 3.11E-09 | 2.20E-08 | Down |
| LINC01353 | 2.055016262 | -1.088266559 | 0.331905 | -3.27885 | 0.001042 | 0.003083 | Down |
| LINC01354 | 24.47886453 | -1.581760024 | 0.157987 | -10.012 | 1.35E-23 | 3.28E-22 | Down |
| LINC01360 | 16.48921849 | -1.672513281 | 0.30911 | -5.41075 | 6.28E-08 | 3.80E-07 | Down |
| LINC01361 | 3.245972102 | -2.045050888 | 0.335307 | -6.09905 | 1.07E-09 | 7.99E-09 | Down |
| LINC01375 | 3.578935243 | -1.224739602 | 0.421269 | -2.90726 | 0.003646 | 0.009375 | Down |
| LINC01378 | 2.597530283 | -1.897124973 | 0.8213 | -2.3099 | 0.020893 | 0.042958 | Down |
| LINC01384 | 11.9989791 | -2.792039555 | 0.30059 | -9.28854 | 1.56E-20 | 3.03E-19 | Down |
| LINC01398 | 1.466533389 | -1.317119125 | 0.404788 | -3.25385 | 0.001139 | 0.003337 | Down |
| LINC01412 | 0.987901515 | -1.052272023 | 0.321024 | -3.27786 | 0.001046 | 0.003093 | Down |
| LINC01419 | 1.203140709 | -2.962742212 | 1.269238 | -2.33427 | 0.019582 | 0.040601 | Down |
| LINC01431 | 5.385449782 | -1.202995175 | 0.184799 | -6.50976 | 7.53E-11 | 6.34E-10 | Down |
| LINC01441 | 2.348539641 | -3.401274643 | 0.839576 | -4.05118 | 5.10E-05 | 0.000199 | Down |
| LINC01446 | 2.125955077 | -1.845008167 | 0.735269 | -2.5093 | 0.012097 | 0.026893 | Down |
| LINC01447 | 2.432484114 | -1.288697713 | 0.487809 | -2.64181 | 0.008247 | 0.019243 | Down |
| LINC01452 | 2.941136474 | -1.866310074 | 0.598357 | -3.11906 | 0.001814 | 0.005062 | Down |
| LINC01470 | 2.520549679 | -1.380058004 | 0.544386 | -2.53507 | 0.011242 | 0.025193 | Down |
| LINC01474 | 1.318004779 | -2.060337996 | 0.320858 | -6.42134 | 1.35E-10 | 1.11E-09 | Down |
| LINC01478 | 1.921206831 | -2.123244068 | 0.733091 | -2.89629 | 0.003776 | 0.009674 | Down |
| LINC01483 | 10.68574967 | 1.687240431 | 0.299522 | 5.633109 | 1.77E-08 | 1.15E-07 | Up |
| LINC01485 | 2.143935803 | -1.84237351 | 0.638884 | -2.88374 | 0.00393 | 0.010014 | Down |
| LINC01497 | 1.255422332 | -1.888454268 | 0.445854 | -4.23559 | 2.28E-05 | 9.47E-05 | Down |
| LINC01502 | 3.624673686 | -2.121250327 | 0.498402 | -4.2561 | 2.08E-05 | 8.71E-05 | Down |
| LINC01503 | 22.17926402 | 1.012357656 | 0.109428 | 9.251387 | 2.22E-20 | 4.24E-19 | Up |
| LINC01505 | 1.115257329 | -2.024754009 | 0.869268 | -2.32926 | 0.019845 | 0.041067 | Down |
| LINC01508 | 48.36230716 | -1.478157623 | 0.140562 | -10.5161 | 7.29E-26 | 2.07E-24 | Down |
| LINC01510 | 3.037034812 | 3.911153978 | 0.363481 | 10.76026 | 5.30E-27 | 1.63E-25 | Up |
| LINC01511 | 1.518195748 | -2.515185867 | 0.521145 | -4.82627 | 1.39E-06 | 7.02E-06 | Down |
| LINC01516 | 3.005465532 | -1.744496363 | 0.382066 | -4.56595 | 4.97E-06 | 2.31E-05 | Down |
| LINC01519 | 3.386072956 | -2.017898332 | 0.301277 | -6.69783 | 2.12E-11 | 1.87E-10 | Down |
| LINC01529 | 1.992072021 | -1.727696695 | 0.536451 | -3.22061 | 0.001279 | 0.003706 | Down |
| LINC01532 | 2.612726428 | -2.32598066 | 0.404694 | -5.7475 | 9.06E-09 | 6.10E-08 | Down |
| LINC01537 | 13.62010013 | -1.862992375 | 0.198274 | -9.39603 | 5.67E-21 | 1.13E-19 | Down |
| LINC01538 | 1.723539632 | -2.832315896 | 1.218575 | -2.32429 | 0.02011 | 0.041541 | Down |
| LINC01539 | 26.14726545 | -2.09447919 | 0.210105 | -9.96873 | 2.09E-23 | 4.98E-22 | Down |
| LINC01540 | 5.524555226 | 1.413317825 | 0.266096 | 5.311306 | 1.09E-07 | 6.39E-07 | Up |
| LINC01541 | 1.710716858 | -2.921964168 | 0.872726 | -3.34809 | 0.000814 | 0.002463 | Down |
| LINC01550 | 74.51000193 | -1.089816913 | 0.126984 | -8.58233 | 9.30E-18 | 1.45E-16 | Down |
| LINC01552 | 1.597388805 | 2.26342153 | 0.35252 | 6.420689 | 1.36E-10 | 1.12E-09 | Up |
| LINC01562 | 4.478045588 | -1.040240409 | 0.295228 | -3.52351 | 0.000426 | 0.001377 | Down |
| LINC01564 | 1.249370593 | -1.345794551 | 0.348721 | -3.85923 | 0.000114 | 0.000414 | Down |
| LINC01570 | 1.924897637 | -1.945293717 | 0.652391 | -2.98179 | 0.002866 | 0.007567 | Down |
| LINC01571 | 52.53283821 | -1.371610072 | 0.133936 | -10.2408 | 1.30E-24 | 3.38E-23 | Down |
| LINC01586 | 30.32857905 | -2.112456955 | 0.215893 | -9.78474 | 1.31E-22 | 2.95E-21 | Down |
| LINGO2 | 84.14405852 | -2.74778748 | 0.227868 | -12.0587 | 1.75E-33 | 8.23E-32 | Down |
| LIPF | 4.155121131 | -3.217158328 | 0.433533 | -7.42079 | 1.16E-13 | 1.29E-12 | Down |
| LIPH | 762.3306915 | 4.616606967 | 0.231225 | 19.96585 | 1.09E-88 | 6.69E-86 | Up |
| LIPI | 22.90841376 | -2.213769059 | 0.27075 | -8.17642 | 2.92E-16 | 4.04E-15 | Down |
| LIPJ | 14.92274469 | -1.975603926 | 0.20652 | -9.56618 | 1.11E-21 | 2.34E-20 | Down |
| LIPM | 1.232468167 | 1.740900726 | 0.397893 | 4.375301 | 1.21E-05 | 5.29E-05 | Up |
| LL09NC01-254D11.1 | 1.471149125 | -3.44737097 | 1.53464 | -2.24637 | 0.02468 | 0.049552 | Down |
| LL0XNC01-116E7.4 | 1.763902524 | -1.098110613 | 0.392794 | -2.79564 | 0.00518 | 0.012793 | Down |
| LL0XNC01-131B10.2 | 28.75282962 | 3.135260632 | 0.245769 | 12.75694 | 2.85E-37 | 1.69E-35 | Up |
| LL0XNC01-16G2.1 | 1.489094832 | -3.132870618 | 1.322007 | -2.36978 | 0.017799 | 0.037393 | Down |
| LL0XNC01-221F2.2 | 1.674392327 | -1.758551046 | 0.61295 | -2.86899 | 0.004118 | 0.010435 | Down |
| LL0XNC01-39B3.1 | 1.669941116 | -1.735426789 | 0.476814 | -3.63963 | 0.000273 | 0.000923 | Down |
| LL21NC02-1C16.1 | 2.430264196 | -1.073333049 | 0.254786 | -4.21269 | 2.52E-05 | 0.000104 | Down |
| LL21NC02-21A1.1 | 4.004615518 | -1.139735733 | 0.235125 | -4.84736 | 1.25E-06 | 6.36E-06 | Down |
| LL22NC03-104C7.1 | 1.016147864 | -1.770078246 | 0.469695 | -3.76857 | 0.000164 | 0.000581 | Down |
| LL22NC03-22D1.1 | 7.140480465 | -3.011906313 | 0.864225 | -3.48509 | 0.000492 | 0.001567 | Down |
| LL22NC03-23C6.13 | 1.075101178 | -2.348242904 | 0.920986 | -2.54971 | 0.010781 | 0.024278 | Down |
| LL22NC03-2H8.4 | 1.167732859 | -1.459465496 | 0.448809 | -3.25186 | 0.001147 | 0.003358 | Down |
| LL22NC03-2H8.5 | 36.47830091 | -1.408144907 | 0.258649 | -5.44424 | 5.20E-08 | 3.18E-07 | Down |
| LL22NC03-75H12.2 | 17.58755113 | 1.127556823 | 0.15889 | 7.096463 | 1.28E-12 | 1.28E-11 | Up |
| LL22NC03-80A10.11 | 1.14078931 | -1.990734169 | 0.86881 | -2.29134 | 0.021944 | 0.044776 | Down |
| LLNLR-276E7.1 | 1.551339203 | -1.375663747 | 0.347156 | -3.96267 | 7.41E-05 | 0.00028 | Down |
| LMAN1L | 3.08612638 | -1.27556388 | 0.554595 | -2.29999 | 0.021449 | 0.043915 | Down |
| LMCD1-AS1 | 14.0635394 | -1.229211831 | 0.208334 | -5.9002 | 3.63E-09 | 2.55E-08 | Down |
| LMNTD1 | 3.94176353 | -2.264620502 | 0.239785 | -9.44438 | 3.58E-21 | 7.28E-20 | Down |
| LMO1 | 4.476554156 | -1.020588907 | 0.297766 | -3.42749 | 0.000609 | 0.0019 | Down |
| LMO3 | 5407.700824 | 1.210620654 | 0.109369 | 11.06913 | 1.77E-28 | 6.13E-27 | Up |
| LMO7DN | 2.32771195 | -1.047566623 | 0.336542 | -3.11274 | 0.001854 | 0.005158 | Down |
| LMOD1 | 1348.322439 | -1.643703561 | 0.141232 | -11.6383 | 2.63E-31 | 1.10E-29 | Down |
| LMOD2 | 7.833338474 | -3.127207118 | 0.413283 | -7.56675 | 3.83E-14 | 4.43E-13 | Down |
| LMOD3 | 13.44901013 | -1.182451667 | 0.261864 | -4.51553 | 6.32E-06 | 2.88E-05 | Down |
| LMX1A | 8.532538497 | -1.928303842 | 0.253529 | -7.60585 | 2.83E-14 | 3.32E-13 | Down |
| LMX1B | 35.57133915 | -1.570534823 | 0.180374 | -8.7071 | 3.12E-18 | 5.04E-17 | Down |
| LNX1-AS2 | 2.587343849 | -1.086890866 | 0.296393 | -3.66706 | 0.000245 | 0.000837 | Down |
| LOC100421166 | 19.39356562 | -1.359050579 | 0.212066 | -6.40862 | 1.47E-10 | 1.20E-09 | Down |
| LOH12CR2 | 41.1902705 | -1.232113342 | 0.102225 | -12.053 | 1.87E-33 | 8.79E-32 | Down |
| LONRF2 | 3882.521565 | 2.394439458 | 0.160167 | 14.94964 | 1.57E-50 | 1.96E-48 | Up |
| LONRF2P1 | 2.998333948 | -1.336235756 | 0.537255 | -2.48715 | 0.012877 | 0.028396 | Down |
| LONRF3 | 641.2125463 | -1.056244344 | 0.133729 | -7.89841 | 2.82E-15 | 3.63E-14 | Down |
| LOR | 1.312802543 | -1.588402251 | 0.622042 | -2.55353 | 0.010664 | 0.024054 | Down |
| LOXHD1 | 14.90638419 | -1.085932065 | 0.249906 | -4.34536 | 1.39E-05 | 6.00E-05 | Down |
| LOXL1 | 295.1225682 | 1.064054921 | 0.115957 | 9.176263 | 4.46E-20 | 8.35E-19 | Up |
| LPA | 6.357775718 | -1.030399621 | 0.318628 | -3.23386 | 0.001221 | 0.00356 | Down |
| LPAR3 | 22.03803522 | 1.399453668 | 0.22772 | 6.14551 | 7.97E-10 | 6.06E-09 | Up |
| LPAR5 | 416.9313722 | 2.35884919 | 0.161569 | 14.59966 | 2.82E-48 | 3.24E-46 | Up |
| LPHN3 | 170.382109 | -1.176508588 | 0.214966 | -5.47301 | 4.42E-08 | 2.73E-07 | Down |
| LPP-AS2 | 44.58598664 | -1.110350697 | 0.106067 | -10.4684 | 1.21E-25 | 3.38E-24 | Down |
| LRFN1 | 23.7545819 | -1.183542328 | 0.170637 | -6.93603 | 4.03E-12 | 3.85E-11 | Down |
| LRG1 | 120.1398002 | 2.773652329 | 0.165306 | 16.77886 | 3.48E-63 | 7.73E-61 | Up |
| LRGUK | 38.3298706 | -1.408946808 | 0.120595 | -11.6833 | 1.55E-31 | 6.63E-30 | Down |
| LRIT2 | 2.286710456 | -1.182792611 | 0.494732 | -2.39077 | 0.016813 | 0.03563 | Down |
| LRIT3 | 10.01075754 | -1.405417814 | 0.254819 | -5.51535 | 3.48E-08 | 2.18E-07 | Down |
| LRP1B | 2336.093513 | -2.650856429 | 0.215175 | -12.3195 | 7.11E-35 | 3.64E-33 | Down |
| LRP2 | 15034.44252 | -1.611157394 | 0.132463 | -12.1631 | 4.89E-34 | 2.37E-32 | Down |
| LRP4 | 3674.49756 | 3.88585764 | 0.178556 | 21.76268 | 5.24E-105 | 5.12E-102 | Up |
| LRP4-AS1 | 16.85595901 | 2.377962049 | 0.304119 | 7.819178 | 5.32E-15 | 6.70E-14 | Up |
| LRRC10 | 2.625574904 | -1.664400969 | 0.562378 | -2.95958 | 0.003081 | 0.008077 | Down |
| LRRC14B | 3.579394358 | -1.14642765 | 0.444279 | -2.58043 | 0.009868 | 0.022494 | Down |
| LRRC15 | 502.4708298 | 1.068709882 | 0.226584 | 4.716609 | 2.40E-06 | 1.17E-05 | Up |
| LRRC2 | 994.6405888 | -1.058719312 | 0.092497 | -11.446 | 2.46E-30 | 9.58E-29 | Down |
| LRRC2-AS1 | 15.02158304 | -1.325333398 | 0.199191 | -6.65356 | 2.86E-11 | 2.50E-10 | Down |
| LRRC26 | 2.236984833 | -2.481155353 | 0.778475 | -3.1872 | 0.001437 | 0.004109 | Down |
| LRRC31 | 2.473285136 | -1.856567386 | 0.4912 | -3.77966 | 0.000157 | 0.000558 | Down |
| LRRC37A11P | 12.49618476 | -1.79723709 | 0.225447 | -7.97188 | 1.56E-15 | 2.04E-14 | Down |
| LRRC37A12P | 1.303751502 | -1.304468806 | 0.563821 | -2.31362 | 0.020688 | 0.042586 | Down |
| LRRC37A5P | 2.789046701 | -1.281026672 | 0.392642 | -3.26258 | 0.001104 | 0.003246 | Down |
| LRRC38 | 3.456100335 | -1.753263186 | 0.640491 | -2.73737 | 0.006193 | 0.014963 | Down |
| LRRC3B | 4.977822005 | -1.612312319 | 0.350195 | -4.60404 | 4.14E-06 | 1.95E-05 | Down |
| LRRC3C | 1.223462469 | -1.28911161 | 0.438107 | -2.94246 | 0.003256 | 0.008482 | Down |
| LRRC4C | 110.2998281 | -1.178404056 | 0.140379 | -8.39446 | 4.68E-17 | 6.91E-16 | Down |
| LRRC52 | 21.39747581 | 2.772285903 | 0.389007 | 7.126564 | 1.03E-12 | 1.04E-11 | Up |
| LRRC53 | 2.5396558 | -1.125288048 | 0.367447 | -3.06245 | 0.002195 | 0.005986 | Down |
| LRRC7 | 131.0250006 | -2.209452561 | 0.15179 | -14.556 | 5.35E-48 | 6.05E-46 | Down |
| LRRC71 | 4.975813802 | -1.480895238 | 0.290872 | -5.09122 | 3.56E-07 | 1.95E-06 | Down |
| LRRC75A | 300.9069559 | 1.497971442 | 0.085587 | 17.50242 | 1.37E-68 | 3.73E-66 | Up |
| LRRC9 | 5.647527094 | -1.754082482 | 0.271521 | -6.46021 | 1.05E-10 | 8.70E-10 | Down |
| LRRIQ1 | 113.5723901 | -1.328007875 | 0.11542 | -11.5059 | 1.23E-30 | 4.92E-29 | Down |
| LRRIQ3 | 49.0617132 | -1.241282857 | 0.143782 | -8.63307 | 5.97E-18 | 9.48E-17 | Down |
| LRRIQ4 | 4.113302603 | -1.272754435 | 0.278523 | -4.56966 | 4.89E-06 | 2.27E-05 | Down |
| LRRK2 | 5767.124874 | 3.093245926 | 0.138518 | 22.33104 | 1.85E-110 | 2.30E-107 | Up |
| LRRN4CL | 111.7853308 | -1.631595807 | 0.175323 | -9.30621 | 1.32E-20 | 2.58E-19 | Down |
| LRRTM1 | 53.52088115 | -1.956438975 | 0.312072 | -6.2692 | 3.63E-10 | 2.86E-09 | Down |
| LRRTM3 | 6.242407307 | -2.309879726 | 0.41997 | -5.50011 | 3.80E-08 | 2.36E-07 | Down |
| LRRTM4 | 4.84828074 | -1.521336592 | 0.380662 | -3.99655 | 6.43E-05 | 0.000245 | Down |
| LRTM1 | 1.516093427 | -1.261598561 | 0.378157 | -3.33618 | 0.000849 | 0.002562 | Down |
| LRTM2 | 4.078660601 | -1.762712108 | 0.394907 | -4.46362 | 8.06E-06 | 3.62E-05 | Down |
| LSMEM2 | 7.576971114 | -1.077565202 | 0.259409 | -4.15393 | 3.27E-05 | 0.000132 | Down |
| LTV1P1 | 4.339996828 | -1.048182972 | 0.30095 | -3.48291 | 0.000496 | 0.001579 | Down |
| LUCAT1 | 41.03225945 | 1.630755529 | 0.168186 | 9.696164 | 3.13E-22 | 6.85E-21 | Up |
| LUM | 4148.007656 | 1.042062317 | 0.158322 | 6.581903 | 4.64E-11 | 3.98E-10 | Up |
| LURAP1L | 113.3630958 | 1.284696524 | 0.119587 | 10.74275 | 6.41E-27 | 1.97E-25 | Up |
| LY6D | 5.631009864 | 3.001411706 | 0.485214 | 6.185742 | 6.18E-10 | 4.75E-09 | Up |
| LY6E | 2162.444018 | 1.58647332 | 0.095201 | 16.66446 | 2.38E-62 | 5.13E-60 | Up |
| LY6G6D | 1.109737734 | -1.44780544 | 0.354171 | -4.08788 | 4.35E-05 | 0.000172 | Down |
| LY6G6E | 3.71575567 | -1.187454766 | 0.384292 | -3.08998 | 0.002002 | 0.005521 | Down |
| LY6K | 13.35763699 | -1.606983254 | 0.225099 | -7.13902 | 9.40E-13 | 9.54E-12 | Down |
| LY86-AS1 | 5.941814056 | -1.434515725 | 0.496855 | -2.88719 | 0.003887 | 0.009918 | Down |
| LYNX1 | 644.200924 | 1.127942605 | 0.102626 | 10.99076 | 4.23E-28 | 1.42E-26 | Up |
| LYPD1 | 48.34556918 | 1.633893408 | 0.204698 | 7.981974 | 1.44E-15 | 1.89E-14 | Up |
| LYPD2 | 0.898326865 | -1.13842591 | 0.49246 | -2.31171 | 0.020794 | 0.042785 | Down |
| LYPD6 | 18.96557288 | -2.105753953 | 0.230347 | -9.14164 | 6.15E-20 | 1.14E-18 | Down |
| LYPD6B | 5.283518703 | -1.613105183 | 0.476549 | -3.38497 | 0.000712 | 0.002186 | Down |
| LYPLAL1-AS1 | 5.742753231 | -1.169717805 | 0.159297 | -7.34299 | 2.09E-13 | 2.25E-12 | Down |
| LYVE1 | 1020.269281 | -2.669918704 | 0.182378 | -14.6395 | 1.57E-48 | 1.84E-46 | Down |
| LYZ | 5475.077023 | 1.269957506 | 0.286126 | 4.438456 | 9.06E-06 | 4.03E-05 | Up |
| LZTS1 | 204.5319702 | 1.494560955 | 0.113994 | 13.11089 | 2.85E-39 | 1.91E-37 | Up |
| LZTS1-AS1 | 1.757174036 | 1.162164358 | 0.421824 | 2.755092 | 0.005868 | 0.014266 | Up |
| MAB21L3 | 4.8251292 | -1.1263631 | 0.28007 | -4.02171 | 5.78E-05 | 0.000222 | Down |
| MACC1 | 986.0588573 | 1.773546045 | 0.114483 | 15.49177 | 3.94E-54 | 5.89E-52 | Up |
| MACROD2-IT1 | 0.805941416 | -1.404339716 | 0.576574 | -2.43566 | 0.014865 | 0.032074 | Down |
| MADCAM1 | 26.96519175 | -1.082185728 | 0.313532 | -3.45159 | 0.000557 | 0.001753 | Down |
| MAFA | 8.259210475 | -1.224411647 | 0.31322 | -3.90911 | 9.26E-05 | 0.000343 | Down |
| MAFF | 430.9684105 | 1.346163233 | 0.107274 | 12.54883 | 4.03E-36 | 2.24E-34 | Up |
| MAG | 80.52968467 | 2.114098041 | 0.241052 | 8.770312 | 1.78E-18 | 2.95E-17 | Up |
| MAGEA1 | 1.753598374 | -2.61884633 | 0.766602 | -3.41617 | 0.000635 | 0.001972 | Down |
| MAGEA10 | 1.522671456 | -1.629912371 | 0.633123 | -2.5744 | 0.010041 | 0.022835 | Down |
| MAGEA11 | 1.424573758 | -2.03198062 | 0.74971 | -2.71035 | 0.006721 | 0.016088 | Down |
| MAGEA2 | 3.001490621 | -1.465098198 | 0.541891 | -2.70368 | 0.006858 | 0.016374 | Down |
| MAGEA3 | 1.287543046 | -1.421684208 | 0.559001 | -2.54326 | 0.010982 | 0.024673 | Down |
| MAGEA8 | 2.724128177 | -1.578031929 | 0.402303 | -3.9225 | 8.76E-05 | 0.000326 | Down |
| MAGEB1 | 1.505242667 | -2.766230872 | 0.878794 | -3.14776 | 0.001645 | 0.004643 | Down |
| MAGEB18 | 1.329665399 | -2.689434574 | 0.919078 | -2.92623 | 0.003431 | 0.008886 | Down |
| MAGEB3 | 1.232430062 | -2.643319217 | 0.896252 | -2.9493 | 0.003185 | 0.008318 | Down |
| MAGEB4 | 1.693373991 | -2.393398637 | 0.896647 | -2.66928 | 0.007601 | 0.01791 | Down |
| MAGEC2 | 1.466118897 | -2.968206119 | 1.034338 | -2.86967 | 0.004109 | 0.010415 | Down |
| MAGEL2 | 45.67374872 | -1.532828423 | 0.163428 | -9.37924 | 6.65E-21 | 1.33E-19 | Down |
| MAGI1-AS1 | 8.142506323 | -1.020259423 | 0.290096 | -3.51697 | 0.000436 | 0.001408 | Down |
| MAMDC2 | 513.8722188 | -1.54540353 | 0.146844 | -10.5241 | 6.69E-26 | 1.91E-24 | Down |
| MAMLD1 | 187.0455118 | 2.10813887 | 0.142265 | 14.81839 | 1.11E-49 | 1.35E-47 | Up |
| MAN1A2P1 | 13.90968109 | -2.349960615 | 0.225794 | -10.4075 | 2.29E-25 | 6.29E-24 | Down |
| MANEAL | 207.5946935 | 1.457475507 | 0.094553 | 15.41436 | 1.31E-53 | 1.92E-51 | Up |
| MAOB | 139.4670011 | -1.422854598 | 0.13558 | -10.4946 | 9.15E-26 | 2.59E-24 | Down |
| MAP1LC3C | 13.59808233 | -1.08806563 | 0.155763 | -6.9854 | 2.84E-12 | 2.74E-11 | Down |
| MAP2 | 1804.910152 | 1.374807571 | 0.095829 | 14.34642 | 1.12E-46 | 1.19E-44 | Up |
| MAP3K1 | 5935.208754 | 1.019260548 | 0.060671 | 16.79993 | 2.44E-63 | 5.48E-61 | Up |
| MAPK13 | 1429.051848 | 1.415511375 | 0.076656 | 18.46569 | 3.90E-76 | 1.50E-73 | Up |
| MAPK4 | 378.1183532 | -2.451727169 | 0.195796 | -12.5218 | 5.67E-36 | 3.13E-34 | Down |
| MAPT-AS1 | 1.677143471 | -2.10915338 | 0.835367 | -2.52482 | 0.011576 | 0.025868 | Down |
| MART1 | 1276.207204 | -1.058503603 | 0.107514 | -9.84524 | 7.19E-23 | 1.65E-21 | Down |
| MART2 | 505.4149487 | -1.032637757 | 0.093877 | -10.9999 | 3.82E-28 | 1.29E-26 | Down |
| MART11 | 3.193715691 | 1.867343892 | 0.555584 | 3.361045 | 0.000776 | 0.002362 | Up |
| MART4 | 7.825360295 | 2.309704043 | 0.332282 | 6.951043 | 3.63E-12 | 3.47E-11 | Up |
| MARCO | 161.7774354 | 2.024398652 | 0.288065 | 7.027575 | 2.10E-12 | 2.06E-11 | Up |
| MARK2P10 | 1.304046778 | -3.481048864 | 1.509558 | -2.30601 | 0.02111 | 0.043317 | Down |
| MARK2P13 | 1.5883178 | -3.423929102 | 1.250839 | -2.73731 | 0.006194 | 0.014965 | Down |
| MARK2P15 | 1.884351025 | -2.994601237 | 0.632022 | -4.73813 | 2.16E-06 | 1.06E-05 | Down |
| MARK3P1 | 5.541598337 | -1.316828763 | 0.26815 | -4.91079 | 9.07E-07 | 4.70E-06 | Down |
| MAS1L | 1.733357656 | -1.128953847 | 0.482869 | -2.33801 | 0.019387 | 0.040239 | Down |
| MASP1 | 94.46937597 | -2.442540981 | 0.187407 | -13.0334 | 7.90E-39 | 5.10E-37 | Down |
| MATN2 | 7901.805807 | -2.132249979 | 0.136685 | -15.5997 | 7.31E-55 | 1.16E-52 | Down |
| MATN3 | 53.31998138 | 2.122378203 | 0.229639 | 9.242245 | 2.41E-20 | 4.60E-19 | Up |
| MATN4 | 8.832704729 | -1.732434521 | 0.209964 | -8.2511 | 1.57E-16 | 2.22E-15 | Down |
| MB | 30.49571444 | -1.902680895 | 0.221922 | -8.57363 | 1.00E-17 | 1.56E-16 | Down |
| MBOAT2 | 1480.81216 | 1.062030244 | 0.081007 | 13.1103 | 2.87E-39 | 1.92E-37 | Up |
| MC3R | 1.319170284 | -1.642514918 | 0.67673 | -2.42713 | 0.015219 | 0.032718 | Down |
| MC5R | 1.468892065 | -1.38539852 | 0.600595 | -2.30671 | 0.021071 | 0.043252 | Down |
| MCCD1 | 3.220881584 | -1.382533361 | 0.299276 | -4.61959 | 3.84E-06 | 1.82E-05 | Down |
| MCEMP1 | 16.55198658 | 2.620432088 | 0.371133 | 7.060624 | 1.66E-12 | 1.64E-11 | Up |
| MCF2 | 23.85287808 | -1.29880491 | 0.174466 | -7.44448 | 9.73E-14 | 1.08E-12 | Down |
| MCTP2 | 1847.701833 | 1.357059021 | 0.067976 | 19.96389 | 1.14E-88 | 6.85E-86 | Up |
| MDFI | 146.4418311 | 1.29090081 | 0.140763 | 9.170763 | 4.70E-20 | 8.76E-19 | Up |
| MDGA2 | 5.317227228 | -1.731704065 | 0.384186 | -4.50746 | 6.56E-06 | 2.99E-05 | Down |
| MDH1B | 129.7280716 | -1.15078582 | 0.098055 | -11.7362 | 8.32E-32 | 3.61E-30 | Down |
| MDK | 1310.695735 | 2.353402421 | 0.097216 | 24.20809 | 1.83E-129 | 5.36E-126 | Up |
| MDS2 | 8.595162934 | -1.027485821 | 0.190952 | -5.38085 | 7.41E-08 | 4.44E-07 | Down |
| MED13 | 8158.988097 | 1.243925493 | 0.082567 | 15.06573 | 2.72E-51 | 3.52E-49 | Up |
| MED15P9 | 1.59467413 | -2.152480936 | 0.785746 | -2.73941 | 0.006155 | 0.014878 | Down |
| MED28P7 | 2.775020374 | -1.237221599 | 0.236859 | -5.22346 | 1.76E-07 | 1.01E-06 | Down |
| MEF2B | 50.39103522 | -1.188309516 | 0.336565 | -3.53069 | 0.000414 | 0.001344 | Down |
| MEF2C-AS1 | 33.60625196 | -1.229776392 | 0.122848 | -10.0105 | 1.37E-23 | 3.33E-22 | Down |
| MEGF10 | 62.01160651 | 1.034303153 | 0.244384 | 4.232284 | 2.31E-05 | 9.60E-05 | Up |
| MEGF9 | 4319.909165 | 1.602060573 | 0.092793 | 17.26485 | 8.65E-67 | 2.17E-64 | Up |
| MEI4 | 117.0006348 | -1.008262051 | 0.167038 | -6.03612 | 1.58E-09 | 1.16E-08 | Down |
| MEIKIN | 2.193138059 | 1.415648666 | 0.312466 | 4.530567 | 5.88E-06 | 2.70E-05 | Up |
| MEIOB | 2.436849164 | -1.051691327 | 0.337155 | -3.11931 | 0.001813 | 0.005058 | Down |
| MEIS1-AS2 | 3.792606936 | -1.35131763 | 0.304857 | -4.43263 | 9.31E-06 | 4.13E-05 | Down |
| MEOX2-AS1 | 2.656661991 | -1.080955674 | 0.271621 | -3.97965 | 6.90E-05 | 0.000262 | Down |
| MEP1B | 2.064228775 | -1.130415949 | 0.323038 | -3.49933 | 0.000466 | 0.001495 | Down |
| MESTP4 | 1.474718737 | -2.20604862 | 0.667079 | -3.30703 | 0.000943 | 0.002818 | Down |
| MET | 13450.06358 | 2.921217212 | 0.117331 | 24.89723 | 7.97E-137 | 3.27E-133 | Up |
| METTL15P3 | 1.038678809 | -1.627243886 | 0.616805 | -2.63818 | 0.008335 | 0.019424 | Down |
| METTL24 | 21.38228336 | -1.425892447 | 0.146741 | -9.71705 | 2.55E-22 | 5.62E-21 | Down |
| METTL7B | 389.0153617 | 4.06734364 | 0.162085 | 25.09392 | 5.79E-139 | 3.40E-135 | Up |
| MFAP2 | 290.7004139 | 1.862004808 | 0.105507 | 17.64819 | 1.05E-69 | 3.06E-67 | Up |
| MFAP4 | 1376.766261 | -1.564929643 | 0.165673 | -9.4459 | 3.52E-21 | 7.18E-20 | Down |
| MFGE8 | 2711.513583 | 1.947204181 | 0.100388 | 19.39677 | 8.22E-84 | 4.11E-81 | Up |
| MFSD2B | 5.874242446 | -1.450857102 | 0.274218 | -5.29089 | 1.22E-07 | 7.11E-07 | Down |
| MGAT3 | 564.4949976 | 1.990906993 | 0.148453 | 13.41102 | 5.21E-41 | 3.91E-39 | Up |
| MGAT4B | 1987.215264 | 1.212540716 | 0.062786 | 19.31223 | 4.24E-83 | 2.02E-80 | Up |
| MGAT4D | 1.251486848 | -2.15514846 | 0.764922 | -2.81747 | 0.00484 | 0.012043 | Down |
| MGC15885 | 5.670913747 | -1.018747128 | 0.338436 | -3.01016 | 0.002611 | 0.006964 | Down |
| MGC27382 | 1.991082695 | -3.256500162 | 0.736715 | -4.4203 | 9.86E-06 | 4.36E-05 | Down |
| MGC32805 | 2.759710045 | -1.878855301 | 0.373461 | -5.03093 | 4.88E-07 | 2.63E-06 | Down |
| MGC45922 | 2.303392476 | -1.856269124 | 0.404322 | -4.59107 | 4.41E-06 | 2.06E-05 | Down |
| MIA2 | 2.217349858 | 1.307748703 | 0.481186 | 2.717761 | 0.006573 | 0.015779 | Up |
| MICAL2 | 1866.583877 | 1.105025101 | 0.085655 | 12.90093 | 4.45E-38 | 2.76E-36 | Up |
| MICALCL | 110.842303 | 1.533989431 | 0.129819 | 11.81639 | 3.21E-32 | 1.43E-30 | Up |
| MIMT1 | 2.727019807 | -1.247581621 | 0.434628 | -2.87046 | 0.004099 | 0.010392 | Down |
| MINA | 1914.153577 | -1.080757688 | 0.097241 | -11.1142 | 1.07E-28 | 3.76E-27 | Down |
| MINOS1P4 | 0.878555026 | -1.36232245 | 0.605168 | -2.25115 | 0.024376 | 0.049009 | Down |
| MIOX | 156.0822703 | -2.165059053 | 0.153974 | -14.0612 | 6.57E-45 | 6.30E-43 | Down |
| MIPEPP1 | 2.771058711 | -1.17736279 | 0.372023 | -3.16476 | 0.001552 | 0.004406 | Down |
| MIR124-2HG | 2.024539736 | -3.375820206 | 1.324543 | -2.54867 | 0.010814 | 0.024348 | Down |
| MIR210HG | 20.76739112 | -1.067283631 | 0.138318 | -7.71613 | 1.20E-14 | 1.46E-13 | Down |
| MIR219A2 | 6.391473445 | -1.409333043 | 0.310032 | -4.54576 | 5.47E-06 | 2.52E-05 | Down |
| MIR222HG | 236.6200867 | 1.751498538 | 0.185904 | 9.421529 | 4.45E-21 | 8.99E-20 | Up |
| MIR3180-1 | 1.340107843 | -1.152037864 | 0.511228 | -2.25347 | 0.024229 | 0.048771 | Down |
| MIR3180-4 | 3.725555097 | -1.467729697 | 0.642959 | -2.28277 | 0.022444 | 0.045652 | Down |
| MIR31HG | 49.73584717 | 2.397979228 | 0.165731 | 14.46908 | 1.90E-47 | 2.10E-45 | Up |
| MIR4500HG | 2.639847799 | -2.694743996 | 0.430959 | -6.2529 | 4.03E-10 | 3.16E-09 | Down |
| MIR4697HG | 219.4795122 | -1.14641195 | 0.12829 | -8.93608 | 4.03E-19 | 7.03E-18 | Down |
| MIR646HG | 32.52416521 | 1.347292384 | 0.219699 | 6.132438 | 8.65E-10 | 6.55E-09 | Up |
| MIR663AHG | 7.900733957 | -1.014501595 | 0.260779 | -3.89027 | 0.0001 | 0.000368 | Down |
| MISP | 111.5222081 | 2.454242218 | 0.168533 | 14.56242 | 4.87E-48 | 5.52E-46 | Up |
| MKRN3 | 12.7505637 | -1.135060987 | 0.3617 | -3.13813 | 0.0017 | 0.004782 | Down |
| MKRN3-AS1 | 0.941484975 | -1.767173742 | 0.624385 | -2.83026 | 0.004651 | 0.011634 | Down |
| MKRN9P | 15.48944089 | 1.734691025 | 0.326474 | 5.313411 | 1.08E-07 | 6.32E-07 | Up |
| MKX | 163.2310601 | 2.778177614 | 0.230214 | 12.06781 | 1.56E-33 | 7.40E-32 | Up |
| MKX-AS1 | 1.200444518 | 2.030905373 | 0.637018 | 3.188145 | 0.001432 | 0.004097 | Up |
| MLIP | 45.63634345 | -1.087905123 | 0.162057 | -6.71309 | 1.91E-11 | 1.69E-10 | Down |
| MLIP-AS1 | 3.91326142 | -2.080984581 | 0.397897 | -5.22996 | 1.70E-07 | 9.72E-07 | Down |
| MLNR | 6.390937728 | -1.977477243 | 0.23226 | -8.51405 | 1.68E-17 | 2.57E-16 | Down |
| MMD2 | 1.420306661 | -3.046119032 | 1.019313 | -2.9884 | 0.002804 | 0.007423 | Down |
| MMEL1 | 11.5891597 | -1.16189128 | 0.208996 | -5.55939 | 2.71E-08 | 1.72E-07 | Down |
| MMP1 | 30.80503263 | 3.123922531 | 0.352752 | 8.855856 | 8.30E-19 | 1.41E-17 | Up |
| MMP10 | 3.067931825 | 3.013337087 | 0.377217 | 7.988345 | 1.37E-15 | 1.79E-14 | Up |
| MMP11 | 431.4315746 | 3.548849241 | 0.246199 | 14.41456 | 4.19E-47 | 4.61E-45 | Up |
| MMP12 | 33.97660762 | 1.378315099 | 0.341319 | 4.0382 | 5.39E-05 | 0.000209 | Up |
| MMP13 | 118.2555065 | 5.000215475 | 0.529972 | 9.434867 | 3.91E-21 | 7.95E-20 | Up |
| MMP14 | 3026.73577 | 1.326955657 | 0.090806 | 14.61305 | 2.32E-48 | 2.69E-46 | Up |
| MMP16 | 530.5689478 | 2.151864608 | 0.167337 | 12.8595 | 7.61E-38 | 4.65E-36 | Up |
| MMP21 | 4.589281672 | -1.304573195 | 0.231122 | -5.64453 | 1.66E-08 | 1.08E-07 | Down |
| MMP27 | 1.268182078 | -1.910727241 | 0.437876 | -4.36363 | 1.28E-05 | 5.55E-05 | Down |
| MMP7 | 198.1286799 | 4.197815787 | 0.30329 | 13.84094 | 1.44E-43 | 1.25E-41 | Up |
| MMRN1 | 2272.23774 | -2.685728921 | 0.207115 | -12.9674 | 1.87E-38 | 1.18E-36 | Down |
| MNX1 | 2.62490057 | -2.35468766 | 0.486438 | -4.84068 | 1.29E-06 | 6.57E-06 | Down |
| MOB3B | 660.7264817 | -1.188000658 | 0.084624 | -14.0386 | 9.05E-45 | 8.56E-43 | Down |
| MOBP | 4.97043129 | -1.016516374 | 0.291486 | -3.48736 | 0.000488 | 0.001556 | Down |
| MOGAT3 | 1.190547708 | -2.197196425 | 0.889128 | -2.47118 | 0.013467 | 0.029509 | Down |
| MORC2-AS1 | 15.32500029 | -1.020498975 | 0.189799 | -5.37674 | 7.58E-08 | 4.54E-07 | Down |
| MORF4L1P4 | 3.627194141 | -1.388526266 | 0.391736 | -3.54455 | 0.000393 | 0.001283 | Down |
| MORF4L1P5 | 2.321805185 | -1.276024525 | 0.363741 | -3.50805 | 0.000451 | 0.001451 | Down |
| MORF4L1P7 | 1.064532678 | -2.039049987 | 0.626863 | -3.25279 | 0.001143 | 0.003349 | Down |
| MORN5 | 0.71560759 | -1.19602129 | 0.424797 | -2.81551 | 0.00487 | 0.012105 | Down |
| MPL | 9.620513452 | -1.201986586 | 0.162475 | -7.39798 | 1.38E-13 | 1.52E-12 | Down |
| MPO | 8.052309995 | -1.046976485 | 0.323648 | -3.23492 | 0.001217 | 0.003549 | Down |
| MPP4 | 3.423408728 | -1.422830961 | 0.376406 | -3.78005 | 0.000157 | 0.000557 | Down |
| MPPED2 | 627.8720702 | -2.42128636 | 0.172019 | -14.0757 | 5.36E-45 | 5.17E-43 | Down |
| MPZL2 | 3538.353905 | 2.558212027 | 0.112062 | 22.82855 | 2.39E-115 | 3.50E-112 | Up |
| MPZL3 | 672.8073922 | 1.412835933 | 0.087429 | 16.15981 | 9.69E-59 | 1.84E-56 | Up |
| MRAP | 1.589507391 | -1.950537539 | 0.500628 | -3.89618 | 9.77E-05 | 0.00036 | Down |
| MRAP2 | 20.64321726 | -1.084898091 | 0.188396 | -5.7586 | 8.48E-09 | 5.72E-08 | Down |
| MRC2 | 2808.508058 | 1.994743783 | 0.099591 | 20.02937 | 3.05E-89 | 1.96E-86 | Up |
| MREG | 276.0256678 | 1.235547493 | 0.11026 | 11.2058 | 3.82E-29 | 1.38E-27 | Up |
| MRGPRE | 16.94560543 | 1.318986128 | 0.247025 | 5.339492 | 9.32E-08 | 5.52E-07 | Up |
| MRGPRF-AS1 | 3.036185896 | -2.331896669 | 0.451043 | -5.17001 | 2.34E-07 | 1.32E-06 | Down |
| MRGPRG-AS1 | 1.553483227 | -2.36343611 | 0.695262 | -3.39935 | 0.000675 | 0.002086 | Down |
| MRGPRX1 | 1.686984755 | -2.378294137 | 0.688443 | -3.4546 | 0.000551 | 0.001737 | Down |
| MRGPRX3 | 2.014230939 | -1.815471166 | 0.59466 | -3.05296 | 0.002266 | 0.006151 | Down |
| MRO | 212.9307247 | -2.438509071 | 0.17661 | -13.8073 | 2.30E-43 | 1.98E-41 | Down |
| MROH2A | 26.93490235 | -1.790905804 | 0.432657 | -4.13932 | 3.48E-05 | 0.00014 | Down |
| MROH2B | 1.373593466 | -1.342354183 | 0.497131 | -2.7002 | 0.00693 | 0.016517 | Down |
| MROH6 | 88.79110383 | 1.577412953 | 0.142728 | 11.0519 | 2.15E-28 | 7.40E-27 | Up |
| MROH7 | 239.4657303 | -1.373762127 | 0.134538 | -10.211 | 1.77E-24 | 4.55E-23 | Down |
| MROH7-TTC4 | 52.90939963 | -1.124184141 | 0.241751 | -4.65018 | 3.32E-06 | 1.58E-05 | Down |
| MRPL2P1 | 0.864381146 | -1.779596143 | 0.653822 | -2.72183 | 0.006492 | 0.015607 | Down |
| MRPL35P1 | 0.750188946 | -1.775424649 | 0.605473 | -2.93229 | 0.003365 | 0.008732 | Down |
| MRPL40P1 | 0.926012271 | 1.267162402 | 0.397622 | 3.186853 | 0.001438 | 0.004113 | Up |
| MRPL42P6 | 0.756505707 | -1.401365199 | 0.506351 | -2.76757 | 0.005648 | 0.013795 | Down |
| MRPL50P2 | 1.255267184 | -1.279271277 | 0.392497 | -3.25931 | 0.001117 | 0.003279 | Down |
| MRPS15P1 | 0.935860035 | -1.437809921 | 0.601435 | -2.39063 | 0.016819 | 0.035637 | Down |
| MS4A1 | 814.284895 | -1.342889138 | 0.419108 | -3.20416 | 0.001355 | 0.003898 | Down |
| MS4A18 | 1.49461321 | -1.931182287 | 0.812694 | -2.37627 | 0.017488 | 0.036839 | Down |
| MS4A3 | 1.864987782 | -2.182935287 | 0.554685 | -3.93545 | 8.30E-05 | 0.00031 | Down |
| MSLNL | 1.531930959 | -2.695858726 | 0.782773 | -3.44399 | 0.000573 | 0.001798 | Down |
| MSMP | 4.982386716 | 1.546874926 | 0.270685 | 5.714673 | 1.10E-08 | 7.33E-08 | Up |
| MSN | 15438.0294 | 1.172985886 | 0.061212 | 19.1626 | 7.60E-82 | 3.47E-79 | Up |
| MSR1 | 848.1173746 | 1.625867648 | 0.172816 | 9.408083 | 5.05E-21 | 1.01E-19 | Up |
| MST1R | 154.7221428 | 1.042101438 | 0.130421 | 7.990268 | 1.35E-15 | 1.77E-14 | Up |
| MSTN | 11.19290902 | -1.191650089 | 0.217912 | -5.46849 | 4.54E-08 | 2.80E-07 | Down |
| MSX2P1 | 2.281171162 | -1.466728585 | 0.397019 | -3.69435 | 0.00022 | 0.00076 | Down |
| MT1A | 7.262456246 | -1.3597652 | 0.248211 | -5.47826 | 4.30E-08 | 2.65E-07 | Down |
| MT1DP | 0.419770143 | -1.648081322 | 0.426075 | -3.86805 | 0.00011 | 0.0004 | Down |
| MT1E | 1541.042813 | -1.278477287 | 0.153592 | -8.32387 | 8.51E-17 | 1.23E-15 | Down |
| MT1F | 1003.463688 | -2.490982447 | 0.18216 | -13.6747 | 1.44E-42 | 1.18E-40 | Down |
| MT1G | 1416.271609 | -3.042094865 | 0.263357 | -11.5512 | 7.28E-31 | 2.95E-29 | Down |
| MT1H | 228.8314518 | -3.059181759 | 0.306573 | -9.97864 | 1.89E-23 | 4.53E-22 | Down |
| MT1JP | 1.26815769 | -1.852107948 | 0.457696 | -4.04659 | 5.20E-05 | 0.000202 | Down |
| MT1L | 67.93117613 | -1.188517212 | 0.139017 | -8.54945 | 1.24E-17 | 1.92E-16 | Down |
| MT1M | 84.14396454 | -1.964273159 | 0.181183 | -10.8414 | 2.19E-27 | 7.00E-26 | Down |
| MTAPP1 | 1.067735287 | -2.214772314 | 0.564235 | -3.92526 | 8.66E-05 | 0.000322 | Down |
| MTCYBP3 | 10.73351153 | -1.177993032 | 0.249493 | -4.72155 | 2.34E-06 | 1.14E-05 | Down |
| MTHFD1L | 352.5507998 | 1.198076273 | 0.07779 | 15.40149 | 1.60E-53 | 2.31E-51 | Up |
| MTHFD2P1 | 2.563563783 | -2.344082595 | 0.769827 | -3.04495 | 0.002327 | 0.006301 | Down |
| MTHFD2P6 | 1.521217456 | -1.766881306 | 0.582587 | -3.03282 | 0.002423 | 0.006526 | Down |
| MTMR11 | 229.9429191 | 1.635876426 | 0.107662 | 15.19458 | 3.84E-52 | 5.17E-50 | Up |
| MTND1P16 | 2.966522989 | -1.855671178 | 0.519155 | -3.5744 | 0.000351 | 0.001158 | Down |
| MTND1P21 | 2.782715101 | -1.118872876 | 0.309084 | -3.61996 | 0.000295 | 0.000988 | Down |
| MTND1P27 | 1.594177116 | -2.433580756 | 0.864154 | -2.81614 | 0.00486 | 0.012086 | Down |
| MTND1P3 | 3.512514303 | -1.122030676 | 0.346933 | -3.23414 | 0.00122 | 0.003557 | Down |
| MTND1P36 | 4.359131163 | -1.130906982 | 0.324673 | -3.48322 | 0.000495 | 0.001577 | Down |
| MTND1P6 | 1.995167801 | -1.183182803 | 0.487458 | -2.42725 | 0.015214 | 0.032712 | Down |
| MTND1P9 | 7.103437035 | -1.001091985 | 0.302311 | -3.31146 | 0.000928 | 0.002779 | Down |
| MTND2P11 | 2.601531363 | -1.265736442 | 0.302655 | -4.18211 | 2.89E-05 | 0.000118 | Down |
| MTND2P15 | 1.452988792 | -3.166911761 | 1.372727 | -2.30702 | 0.021054 | 0.043225 | Down |
| MTND2P16 | 2.766197021 | -2.225955561 | 0.59765 | -3.72451 | 0.000196 | 0.000682 | Down |
| MTND2P23 | 2.711176419 | -1.895485178 | 0.482391 | -3.92935 | 8.52E-05 | 0.000318 | Down |
| MTND2P29 | 1.712637778 | -1.387373956 | 0.441399 | -3.14313 | 0.001672 | 0.00471 | Down |
| MTND2P31 | 1.809126438 | -3.187943788 | 1.3207 | -2.41383 | 0.015786 | 0.033736 | Down |
| MTND2P4 | 1.448390179 | -2.199885558 | 0.86188 | -2.55243 | 0.010698 | 0.02412 | Down |
| MTND2P9 | 2.105518055 | -2.086607186 | 0.879603 | -2.37221 | 0.017682 | 0.037186 | Down |
| MTND3P9 | 1.130089201 | -3.02326366 | 1.292052 | -2.33989 | 0.019289 | 0.040059 | Down |
| MTND4P14 | 10.82065336 | -1.141651108 | 0.284066 | -4.01896 | 5.85E-05 | 0.000225 | Down |
| MTND4P16 | 3.235970249 | -1.367578296 | 0.40696 | -3.36048 | 0.000778 | 0.002367 | Down |
| MTND4P22 | 1.522382372 | -2.39494208 | 0.937101 | -2.55569 | 0.010598 | 0.023928 | Down |
| MTND5P1 | 2.387731964 | -1.140176357 | 0.429 | -2.65775 | 0.007866 | 0.018447 | Down |
| MTND5P11 | 13.00599775 | -1.089761076 | 0.308761 | -3.52946 | 0.000416 | 0.00135 | Down |
| MTND5P14 | 9.396757169 | -1.439757671 | 0.29773 | -4.83579 | 1.33E-06 | 6.72E-06 | Down |
| MTND5P16 | 5.869748719 | -1.547215093 | 0.357036 | -4.3335 | 1.47E-05 | 6.30E-05 | Down |
| MTND5P2 | 8.591388664 | -1.16155297 | 0.280426 | -4.14211 | 3.44E-05 | 0.000138 | Down |
| MTND5P26 | 11.74141097 | -1.129689268 | 0.260792 | -4.33176 | 1.48E-05 | 6.35E-05 | Down |
| MTND5P28 | 7.709915923 | -1.226858342 | 0.315512 | -3.88846 | 0.000101 | 0.000371 | Down |
| MTND5P31 | 2.177325099 | -2.120972864 | 0.67495 | -3.14242 | 0.001676 | 0.004721 | Down |
| MTND5P5 | 4.185559865 | -1.398336719 | 0.350488 | -3.98968 | 6.62E-05 | 0.000252 | Down |
| MTND6P10 | 1.482082435 | -3.217513702 | 1.390859 | -2.31333 | 0.020705 | 0.042617 | Down |
| MTND6P18 | 4.177347465 | -1.66302582 | 0.352794 | -4.71387 | 2.43E-06 | 1.18E-05 | Down |
| MTND6P4 | 4.020812017 | -1.653903009 | 0.621786 | -2.65992 | 0.007816 | 0.018346 | Down |
| MTND6P5 | 4.270932439 | -1.756662198 | 0.390411 | -4.49952 | 6.81E-06 | 3.09E-05 | Down |
| MTNR1B | 1.609300925 | -1.364342046 | 0.606752 | -2.2486 | 0.024538 | 0.049296 | Down |
| MTRNR2L1 | 190.313063 | 1.280537146 | 0.533203 | 2.401594 | 0.016324 | 0.034719 | Up |
| MTRNR2L9 | 58.8714 | 1.307596726 | 0.296922 | 4.403832 | 1.06E-05 | 4.68E-05 | Up |
| MTTP | 9.229387445 | -1.49108662 | 0.234304 | -6.36389 | 1.97E-10 | 1.59E-09 | Down |
| MTUS1 | 2684.897666 | 1.007234277 | 0.073991 | 13.61291 | 3.36E-42 | 2.68E-40 | Up |
| MTUS2 | 37.73311055 | -1.059975091 | 0.196681 | -5.38932 | 7.07E-08 | 4.25E-07 | Down |
| MTUS2-AS1 | 2.216094269 | -1.810755867 | 0.535926 | -3.37874 | 0.000728 | 0.00223 | Down |
| MUC1 | 424.1147684 | 1.92984587 | 0.150138 | 12.85381 | 8.19E-38 | 5.00E-36 | Up |
| MUC13 | 2.723956303 | -1.758006149 | 0.509313 | -3.45172 | 0.000557 | 0.001752 | Down |
| MUC17 | 26.23739978 | -1.443054876 | 0.607156 | -2.37675 | 0.017466 | 0.036794 | Down |
| MUC2 | 14.74493915 | -1.379202943 | 0.504517 | -2.73371 | 0.006262 | 0.015114 | Down |
| MUC21 | 87.24857465 | 4.01055193 | 0.315178 | 12.72471 | 4.31E-37 | 2.54E-35 | Up |
| MUC4 | 48.41145913 | -1.148083223 | 0.257545 | -4.4578 | 8.28E-06 | 3.71E-05 | Down |
| MUC5AC | 12.36867156 | -1.386967088 | 0.544748 | -2.54607 | 0.010894 | 0.024507 | Down |
| MUM1L1 | 421.9180583 | -1.627866151 | 0.164646 | -9.88705 | 4.74E-23 | 1.10E-21 | Down |
| MUSK | 17.97433343 | -1.315142776 | 0.317287 | -4.14496 | 3.40E-05 | 0.000137 | Down |
| MUSTN1 | 77.81326044 | -1.618017434 | 0.278123 | -5.81763 | 5.97E-09 | 4.09E-08 | Down |
| MVP | 3615.006025 | 1.360092411 | 0.072389 | 18.78867 | 9.35E-79 | 3.84E-76 | Up |
| MXRA5 | 5192.8737 | 1.108017022 | 0.109018 | 10.1636 | 2.88E-24 | 7.30E-23 | Up |
| MXRA5Y | 56.7941724 | 1.191531538 | 0.221141 | 5.388118 | 7.12E-08 | 4.28E-07 | Up |
| MXRA8 | 3680.78941 | 1.650950207 | 0.092037 | 17.93795 | 5.96E-72 | 1.85E-69 | Up |
| MYADML2 | 3.107685949 | -2.6788605 | 0.557411 | -4.8059 | 1.54E-06 | 7.71E-06 | Down |
| MYBPC1 | 18.28995658 | -2.737308153 | 0.406718 | -6.73023 | 1.69E-11 | 1.51E-10 | Down |
| MYBPH | 15.33328055 | 3.673175878 | 0.347479 | 10.57092 | 4.06E-26 | 1.17E-24 | Up |
| MYBPHL | 7.913838637 | 1.902415871 | 0.327573 | 5.807613 | 6.34E-09 | 4.33E-08 | Up |
| MYCL | 272.8337354 | -1.123563266 | 0.105801 | -10.6196 | 2.42E-26 | 7.09E-25 | Down |
| MYCN | 105.4748216 | -1.475074665 | 0.119674 | -12.3257 | 6.58E-35 | 3.40E-33 | Down |
| MYCNOS | 3.794556009 | -2.648034097 | 0.270126 | -9.80294 | 1.09E-22 | 2.48E-21 | Down |
| MYEOV | 14.61397691 | 1.55984015 | 0.250443 | 6.228316 | 4.71E-10 | 3.67E-09 | Up |
| MYF6 | 1.123517158 | -1.413014443 | 0.554155 | -2.54985 | 0.010777 | 0.024271 | Down |
| MYH1 | 6.698706925 | -1.343507717 | 0.589989 | -2.27717 | 0.022776 | 0.046229 | Down |
| MYH10 | 4498.099019 | 1.107824478 | 0.095642 | 11.58301 | 5.03E-31 | 2.06E-29 | Up |
| MYH11 | 3691.641414 | -1.152183511 | 0.229931 | -5.01101 | 5.41E-07 | 2.90E-06 | Down |
| MYH15 | 30.91962616 | -2.162035903 | 0.196931 | -10.9786 | 4.84E-28 | 1.62E-26 | Down |
| MYH2 | 47.09461066 | -3.768460735 | 0.610275 | -6.17502 | 6.62E-10 | 5.06E-09 | Down |
| MYH6 | 5.614399084 | -1.155953892 | 0.397047 | -2.91138 | 0.003598 | 0.009269 | Down |
| MYH7 | 32.26292191 | -3.645126938 | 0.481819 | -7.56535 | 3.87E-14 | 4.47E-13 | Down |
| MYH7B | 73.60288901 | -1.100282713 | 0.19287 | -5.70479 | 1.16E-08 | 7.74E-08 | Down |
| MYH8 | 1.962766061 | -1.711586376 | 0.642024 | -2.66592 | 0.007678 | 0.018075 | Down |
| MYL1 | 7.21505773 | -4.078261859 | 0.838741 | -4.86236 | 1.16E-06 | 5.92E-06 | Down |
| MYL2 | 3.964909081 | -2.954899461 | 0.549323 | -5.37916 | 7.48E-08 | 4.48E-07 | Down |
| MYL7 | 1.090172914 | -2.38166938 | 0.68914 | -3.456 | 0.000548 | 0.001729 | Down |
| MYLK3 | 22.07884128 | -1.930109624 | 0.327692 | -5.89001 | 3.86E-09 | 2.70E-08 | Down |
| MYLKP1 | 1.681132774 | -1.930805533 | 0.645984 | -2.98894 | 0.0028 | 0.007411 | Down |
| MYO16 | 16.67462445 | -1.118658777 | 0.279424 | -4.00345 | 6.24E-05 | 0.000239 | Down |
| MYO1G | 632.1875831 | 1.487566808 | 0.209529 | 7.099575 | 1.25E-12 | 1.26E-11 | Up |
| MYO1H | 15.97037789 | -1.137300185 | 0.195594 | -5.8146 | 6.08E-09 | 4.16E-08 | Down |
| MYO3B | 235.9702873 | 1.150980638 | 0.115062 | 10.0031 | 1.48E-23 | 3.58E-22 | Up |
| MYO5BP2 | 10.7581182 | -1.550296443 | 0.40009 | -3.87487 | 0.000107 | 0.00039 | Down |
| MYO5BP3 | 1.605511405 | -1.700470927 | 0.656472 | -2.59032 | 0.009589 | 0.02195 | Down |
| MYOC | 554.1755868 | -2.822936588 | 0.279729 | -10.0917 | 6.01E-24 | 1.50E-22 | Down |
| MYOG | 1.964175048 | -2.216183848 | 0.688223 | -3.22015 | 0.001281 | 0.00371 | Down |
| MYOT | 20.13147919 | -1.774344956 | 0.204363 | -8.68231 | 3.88E-18 | 6.23E-17 | Down |
| MYOZ2 | 5.634462713 | -1.534949934 | 0.274139 | -5.59917 | 2.15E-08 | 1.38E-07 | Down |
| MYPN | 6.723161815 | -2.82696016 | 0.566787 | -4.98769 | 6.11E-07 | 3.25E-06 | Down |
| MYRIP | 155.6784431 | -1.035499947 | 0.118931 | -8.70675 | 3.13E-18 | 5.05E-17 | Down |
| MYT1 | 36.07893284 | -1.255418158 | 0.167787 | -7.48221 | 7.31E-14 | 8.24E-13 | Down |
| MYT1L | 4.016092071 | -1.350416835 | 0.346588 | -3.89631 | 9.77E-05 | 0.00036 | Down |
| MYZAP | 102.0558372 | -1.488435319 | 0.131422 | -11.3256 | 9.80E-30 | 3.69E-28 | Down |
| NAA11 | 1.602573171 | -1.544194426 | 0.583494 | -2.64646 | 0.008134 | 0.019005 | Down |
| NAALAD2 | 68.81839379 | -1.050632932 | 0.119652 | -8.78076 | 1.62E-18 | 2.69E-17 | Down |
| NAB2 | 1539.612542 | 2.275905819 | 0.107465 | 21.1782 | 1.52E-99 | 1.27E-96 | Up |
| NALCN-AS1 | 3.799640175 | -1.673089838 | 0.4171 | -4.01124 | 6.04E-05 | 0.000232 | Down |
| NAMA | 11.68031026 | -2.309751232 | 0.337583 | -6.84202 | 7.81E-12 | 7.24E-11 | Down |
| NANOGNBP2 | 2.323859279 | -1.648139827 | 0.418282 | -3.94026 | 8.14E-05 | 0.000305 | Down |
| NANOGP1 | 1.553120425 | -2.313068531 | 0.86785 | -2.66528 | 0.007692 | 0.018102 | Down |
| NANOGP11 | 0.861649295 | -1.441167683 | 0.551938 | -2.61111 | 0.009025 | 0.020831 | Down |
| NANOGP2 | 3.870760445 | -1.014969522 | 0.277241 | -3.66097 | 0.000251 | 0.000855 | Down |
| NANOGP6 | 1.238048149 | -2.572728456 | 0.745346 | -3.45172 | 0.000557 | 0.001752 | Down |
| NANOGP7 | 1.816077067 | -1.773004046 | 0.504261 | -3.51604 | 0.000438 | 0.001413 | Down |
| NANOS2 | 1.360633666 | -2.724391144 | 1.00839 | -2.70172 | 0.006898 | 0.016454 | Down |
| NAP1L1P1 | 4.173153937 | -1.698042498 | 0.218051 | -7.78738 | 6.84E-15 | 8.53E-14 | Down |
| NAP1L4P3 | 1.303338835 | -1.525460255 | 0.52234 | -2.92044 | 0.003495 | 0.009031 | Down |
| NAPSA | 143.170419 | 2.284544945 | 0.21187 | 10.78275 | 4.15E-27 | 1.29E-25 | Up |
| NAT16 | 10.30357769 | -2.371752393 | 0.344075 | -6.89313 | 5.46E-12 | 5.15E-11 | Down |
| NAT8L | 400.0023117 | 2.364665776 | 0.178817 | 13.22394 | 6.38E-40 | 4.47E-38 | Up |
| NAV2 | 4128.510691 | 1.352796813 | 0.112341 | 12.04192 | 2.14E-33 | 1.00E-31 | Up |
| NAV2-AS1 | 20.64631931 | 1.478722656 | 0.218129 | 6.779131 | 1.21E-11 | 1.10E-10 | Up |
| NBEAP1 | 8.95991438 | -1.297714517 | 0.287698 | -4.51068 | 6.46E-06 | 2.94E-05 | Down |
| NCAM1 | 1412.45565 | -1.879984904 | 0.169805 | -11.0715 | 1.73E-28 | 5.98E-27 | Down |
| NCAM1-AS1 | 3.12680373 | -2.406164914 | 0.327932 | -7.33738 | 2.18E-13 | 2.34E-12 | Down |
| NCAN | 9.559946595 | -1.525092717 | 0.307496 | -4.95971 | 7.06E-07 | 3.71E-06 | Down |
| NCAPD2P1 | 1.600146266 | -1.978376689 | 0.79826 | -2.47836 | 0.013199 | 0.029 | Down |
| NCF2 | 445.6152297 | 1.093434945 | 0.169062 | 6.467651 | 9.95E-11 | 8.29E-10 | Up |
| NCLP2 | 1.702876824 | -3.244525633 | 1.123605 | -2.8876 | 0.003882 | 0.009908 | Down |
| NCMAP | 333.7495639 | 1.024756978 | 0.114643 | 8.938644 | 3.94E-19 | 6.88E-18 | Up |
| NCRNA00250 | 0.420737859 | -1.205173221 | 0.523923 | -2.30029 | 0.021432 | 0.043887 | Down |
| NDE1P2 | 2.448688811 | -2.298296133 | 0.692337 | -3.31962 | 0.000901 | 0.002705 | Down |
| NDNF | 112.242892 | -1.157252595 | 0.166479 | -6.95134 | 3.62E-12 | 3.46E-11 | Down |
| NDST4 | 2.073512462 | -1.646058259 | 0.537758 | -3.06096 | 0.002206 | 0.006013 | Down |
| NDUFB8P1 | 1.111801143 | -1.539916105 | 0.445892 | -3.45356 | 0.000553 | 0.001742 | Down |
| NEB | 411.8429582 | -1.825185649 | 0.201751 | -9.04672 | 1.47E-19 | 2.66E-18 | Down |
| NECAB1 | 168.9946957 | -1.760592567 | 0.117829 | -14.9419 | 1.76E-50 | 2.19E-48 | Down |
| NECAP1P1 | 1.196234378 | -2.743542831 | 0.691036 | -3.97019 | 7.18E-05 | 0.000272 | Down |
| NEGR1 | 282.1861194 | -1.437554236 | 0.131749 | -10.9113 | 1.02E-27 | 3.33E-26 | Down |
| NEK10 | 39.64480247 | -1.163171749 | 0.125746 | -9.25017 | 2.24E-20 | 4.29E-19 | Down |
| NELL1 | 5.017512075 | -2.81799374 | 0.370589 | -7.60409 | 2.87E-14 | 3.36E-13 | Down |
| NELL2 | 3807.357001 | 2.640068834 | 0.150802 | 17.50689 | 1.27E-68 | 3.47E-66 | Up |
| NEU4 | 7.958277296 | -1.7341389 | 0.350289 | -4.9506 | 7.40E-07 | 3.88E-06 | Down |
| NEUROG3 | 1.334923174 | -2.158632343 | 0.613227 | -3.52012 | 0.000431 | 0.001393 | Down |
| NEXN-AS1 | 15.91649569 | -1.551644182 | 0.174144 | -8.91011 | 5.10E-19 | 8.81E-18 | Down |
| NFE2L3 | 710.0995265 | 1.3242916 | 0.135075 | 9.804085 | 1.08E-22 | 2.45E-21 | Up |
| NFIA-AS1 | 5.941930427 | -1.018171347 | 0.223403 | -4.55755 | 5.18E-06 | 2.40E-05 | Down |
| NGEF | 398.0808174 | 3.638921092 | 0.190612 | 19.09072 | 3.02E-81 | 1.35E-78 | Up |
| NGFR | 503.0911 | 1.475536786 | 0.156815 | 9.409435 | 4.99E-21 | 1.00E-19 | Up |
| NHSL2 | 502.3715218 | 1.563152442 | 0.08719 | 17.92804 | 7.12E-72 | 2.20E-69 | Up |
| NIFKP1 | 1.264355995 | -3.196362101 | 1.158543 | -2.75895 | 0.005799 | 0.014117 | Down |
| NIFKP3 | 1.129883447 | -2.464302395 | 1.023776 | -2.40707 | 0.016081 | 0.03427 | Down |
| NIM1K | 16.66757156 | -1.361729844 | 0.179226 | -7.59784 | 3.01E-14 | 3.52E-13 | Down |
| NIPA2P2 | 1.482992392 | -2.312466016 | 0.715599 | -3.23151 | 0.001231 | 0.003587 | Down |
| NIPA2P4 | 1.426328821 | -2.390317698 | 0.794664 | -3.00796 | 0.00263 | 0.00701 | Down |
| NKAIN1 | 4.323533285 | -2.063716958 | 0.481936 | -4.28214 | 1.85E-05 | 7.82E-05 | Down |
| NKAIN1P1 | 1.382519732 | -2.349602516 | 0.622361 | -3.7753 | 0.00016 | 0.000567 | Down |
| NKAIN3 | 2.441370086 | -1.633278894 | 0.46139 | -3.53991 | 0.0004 | 0.001303 | Down |
| NKAIN4 | 42.46122879 | 1.264254012 | 0.17167 | 7.364463 | 1.78E-13 | 1.93E-12 | Up |
| NKD2 | 48.06104091 | 1.919683187 | 0.216149 | 8.881308 | 6.61E-19 | 1.13E-17 | Up |
| NKX2-2 | 1.637892495 | -1.842163362 | 0.530174 | -3.47464 | 0.000512 | 0.001623 | Down |
| NKX2-4 | 3.122174784 | -1.740112875 | 0.515463 | -3.37583 | 0.000736 | 0.002251 | Down |
| NKX2-5 | 1.816248 | -2.094917429 | 0.563185 | -3.71977 | 0.000199 | 0.000694 | Down |
| NKX6-2 | 6.674117753 | -1.526570513 | 0.310214 | -4.92102 | 8.61E-07 | 4.47E-06 | Down |
| NLGN1 | 58.30298131 | -1.762031525 | 0.169989 | -10.3656 | 3.56E-25 | 9.65E-24 | Down |
| NLGN1-AS1 | 0.474389408 | -1.527782641 | 0.673742 | -2.26761 | 0.023353 | 0.047218 | Down |
| NLRP11 | 4.27444677 | -1.415620674 | 0.418776 | -3.38037 | 0.000724 | 0.002219 | Down |
| NLRP13 | 2.246590749 | -3.09591566 | 0.897679 | -3.4488 | 0.000563 | 0.001769 | Down |
| NLRP4 | 10.79305166 | -1.310461417 | 0.451707 | -2.90113 | 0.003718 | 0.009541 | Down |
| NLRP5 | 3.159368529 | -1.616045703 | 0.638629 | -2.53049 | 0.01139 | 0.025487 | Down |
| NLRP6 | 15.15167848 | -1.449235139 | 0.315314 | -4.59617 | 4.30E-06 | 2.02E-05 | Down |
| NLRP9 | 1.979045608 | -2.696400272 | 0.762859 | -3.5346 | 0.000408 | 0.001326 | Down |
| NMB | 687.7641633 | 1.810687061 | 0.201393 | 8.990801 | 2.45E-19 | 4.35E-18 | Up |
| NMD3P1 | 12.46967253 | -1.123145943 | 0.144521 | -7.77153 | 7.75E-15 | 9.62E-14 | Down |
| NMNAT1P1 | 136.8197908 | -1.005046103 | 0.208133 | -4.82886 | 1.37E-06 | 6.93E-06 | Down |
| NMRK2 | 1.289492635 | -1.58525683 | 0.397239 | -3.99069 | 6.59E-05 | 0.000251 | Down |
| NMU | 58.63148158 | 4.514218495 | 0.353114 | 12.78402 | 2.01E-37 | 1.20E-35 | Up |
| NMUR1 | 21.79949537 | -1.361709999 | 0.154294 | -8.82544 | 1.09E-18 | 1.84E-17 | Down |
| NOD1 | 1700.368236 | 2.560059625 | 0.130552 | 19.60948 | 1.28E-85 | 6.93E-83 | Up |
| NOL4 | 72.77494458 | -2.037258606 | 0.204875 | -9.94393 | 2.68E-23 | 6.34E-22 | Down |
| NOS1 | 70.19182441 | -1.850612825 | 0.299374 | -6.18161 | 6.35E-10 | 4.86E-09 | Down |
| NOX4 | 201.924422 | 1.297642105 | 0.147009 | 8.826981 | 1.08E-18 | 1.81E-17 | Up |
| NPAP1P2 | 1.494372429 | -2.86533317 | 1.108598 | -2.58464 | 0.009748 | 0.022261 | Down |
| NPAP1P4 | 13.0371513 | -1.956926596 | 0.583345 | -3.35466 | 0.000795 | 0.002411 | Down |
| NPAS4 | 6.397112918 | -2.154829929 | 0.51557 | -4.17951 | 2.92E-05 | 0.000119 | Down |
| NPBWR1 | 3.230106264 | 1.109952585 | 0.342566 | 3.240114 | 0.001195 | 0.00349 | Up |
| NPC2 | 15812.90125 | 2.653658093 | 0.10022 | 26.47833 | 1.72E-154 | 1.41E-150 | Up |
| NPFFR2 | 16.84946367 | 1.230351171 | 0.264405 | 4.653285 | 3.27E-06 | 1.56E-05 | Up |
| NPHS1 | 15.22570326 | -1.712045218 | 0.576799 | -2.96818 | 0.002996 | 0.007877 | Down |
| NPHS2 | 1.312174254 | -1.360777322 | 0.516506 | -2.63458 | 0.008424 | 0.019607 | Down |
| NPIPB7 | 2.852892378 | -1.257074241 | 0.30275 | -4.15218 | 3.29E-05 | 0.000133 | Down |
| NPM1P11 | 1.760884544 | -2.24867427 | 0.578695 | -3.88577 | 0.000102 | 0.000375 | Down |
| NPM1P18 | 3.118906413 | -2.185839208 | 0.317122 | -6.89273 | 5.47E-12 | 5.16E-11 | Down |
| NPM1P22 | 1.387663604 | -2.61422276 | 0.908072 | -2.87887 | 0.003991 | 0.010152 | Down |
| NPM1P30 | 9.435243674 | -1.033551263 | 0.260594 | -3.96613 | 7.30E-05 | 0.000276 | Down |
| NPM1P33 | 2.155087354 | -1.860586145 | 0.598087 | -3.11089 | 0.001865 | 0.005188 | Down |
| NPM1P35 | 1.166459477 | -2.291669855 | 0.863927 | -2.65262 | 0.007987 | 0.018703 | Down |
| NPM1P38 | 1.522186042 | -3.271880406 | 1.220167 | -2.6815 | 0.007329 | 0.017341 | Down |
| NPM1P43 | 2.565655808 | -1.44736455 | 0.379935 | -3.80951 | 0.000139 | 0.000499 | Down |
| NPM1P49 | 0.814789735 | -1.213020142 | 0.538678 | -2.25185 | 0.024332 | 0.048935 | Down |
| NPM1P5 | 1.378307442 | -2.822107973 | 0.844848 | -3.34037 | 0.000837 | 0.002528 | Down |
| NPM1P51 | 1.487869557 | -2.751847699 | 0.925128 | -2.97456 | 0.002934 | 0.00773 | Down |
| NPM1P52 | 1.92924694 | -1.675955536 | 0.623829 | -2.68656 | 0.007219 | 0.017113 | Down |
| NPM1P8 | 1.814770924 | -2.164855795 | 0.472872 | -4.5781 | 4.69E-06 | 2.19E-05 | Down |
| NPPC | 8.362172553 | -1.183370515 | 0.252521 | -4.68623 | 2.78E-06 | 1.34E-05 | Down |
| NPR1 | 386.8448657 | -1.09489011 | 0.103308 | -10.5983 | 3.03E-26 | 8.85E-25 | Down |
| NPR3 | 1203.774641 | -1.772534726 | 0.159584 | -11.1073 | 1.16E-28 | 4.05E-27 | Down |
| NPTX1 | 21.50398952 | -1.704612525 | 0.339211 | -5.02523 | 5.03E-07 | 2.70E-06 | Down |
| NPTX2 | 23.46804364 | 1.786739526 | 0.235919 | 7.573533 | 3.63E-14 | 4.22E-13 | Up |
| NPTXR | 447.4473164 | 1.483993097 | 0.084139 | 17.63732 | 1.27E-69 | 3.68E-67 | Up |
| NPW | 14.6850297 | 2.722605153 | 0.343972 | 7.915204 | 2.47E-15 | 3.19E-14 | Up |
| NPY | 2.261481453 | -1.142305172 | 0.342879 | -3.33151 | 0.000864 | 0.002602 | Down |
| NPY1R | 335.7411117 | -1.007541457 | 0.133474 | -7.54859 | 4.40E-14 | 5.06E-13 | Down |
| NPY4R | 5.422993755 | -1.343022497 | 0.379162 | -3.54208 | 0.000397 | 0.001294 | Down |
| NPY5R | 19.77553785 | -2.081192469 | 0.186997 | -11.1296 | 9.01E-29 | 3.19E-27 | Down |
| NR1D1 | 261.7085811 | 1.517518152 | 0.183282 | 8.27971 | 1.23E-16 | 1.76E-15 | Up |
| NR1H4 | 3.95262749 | -1.809811021 | 0.70688 | -2.56028 | 0.010459 | 0.023656 | Down |
| NR1I3 | 11.14219345 | -1.024718754 | 0.223298 | -4.58901 | 4.45E-06 | 2.08E-05 | Down |
| NR2E3 | 9.64756588 | -1.676454883 | 0.250008 | -6.70561 | 2.01E-11 | 1.78E-10 | Down |
| NR2F1-AS1 | 298.771889 | 2.395202665 | 0.127289 | 18.81707 | 5.47E-79 | 2.27E-76 | Up |
| NR2F2-AS1 | 50.34920194 | -1.022791499 | 0.115723 | -8.83826 | 9.72E-19 | 1.64E-17 | Down |
| NR4A1 | 1661.635753 | -1.330094122 | 0.22415 | -5.93394 | 2.96E-09 | 2.10E-08 | Down |
| NRADDP | 0.975521702 | -1.205390543 | 0.343019 | -3.51407 | 0.000441 | 0.001422 | Down |
| NRAP | 15.68698898 | -4.204901576 | 0.639134 | -6.57906 | 4.73E-11 | 4.06E-10 | Down |
| NRCAM | 1217.283353 | 2.658789306 | 0.126062 | 21.0911 | 9.60E-99 | 7.88E-96 | Up |
| NRIP1 | 3473.575018 | 1.573841827 | 0.077405 | 20.33265 | 6.61E-92 | 4.45E-89 | Up |
| NRK | 124.7023893 | 1.086631295 | 0.18603 | 5.841173 | 5.18E-09 | 3.58E-08 | Up |
| NRP2 | 4467.392748 | 1.714628995 | 0.0998 | 17.18059 | 3.71E-66 | 9.23E-64 | Up |
| NRXN1 | 71.41998998 | -1.689282079 | 0.225524 | -7.49048 | 6.86E-14 | 7.76E-13 | Down |
| NSRP1P1 | 16.03442392 | -1.042388867 | 0.247672 | -4.20874 | 2.57E-05 | 0.000106 | Down |
| NT5C1A | 19.29945814 | 2.237817764 | 0.299473 | 7.472516 | 7.87E-14 | 8.84E-13 | Up |
| NT5DC4 | 4.490376507 | -1.177254956 | 0.500063 | -2.35421 | 0.018562 | 0.038774 | Down |
| NT5E | 2241.630431 | 1.491904588 | 0.103707 | 14.38575 | 6.36E-47 | 6.85E-45 | Up |
| NTAN1P3 | 8.289173718 | 3.69410606 | 0.375586 | 9.835576 | 7.91E-23 | 1.81E-21 | Up |
| NTM | 140.2106812 | 1.506173971 | 0.187546 | 8.030959 | 9.67E-16 | 1.28E-14 | Up |
| NTN1 | 200.9308353 | -1.718543061 | 0.125495 | -13.6941 | 1.10E-42 | 9.14E-41 | Down |
| NTNG1 | 15.5801174 | -1.180221849 | 0.254751 | -4.63284 | 3.61E-06 | 1.71E-05 | Down |
| NTS | 24.40500565 | -2.074481364 | 0.253524 | -8.1826 | 2.78E-16 | 3.84E-15 | Down |
| NTSR2 | 1.674778634 | -2.68360952 | 0.745435 | -3.60006 | 0.000318 | 0.001059 | Down |
| NUDT11 | 110.0934477 | 2.325061084 | 0.186777 | 12.4483 | 1.43E-35 | 7.66E-34 | Up |
| NUGGC | 214.5324939 | -1.407390987 | 0.224472 | -6.26979 | 3.62E-10 | 2.85E-09 | Down |
| NUP62CL | 81.29485466 | -1.101926483 | 0.088518 | -12.4486 | 1.42E-35 | 7.65E-34 | Down |
| NUTF2P3 | 1.194466136 | -1.65041343 | 0.360186 | -4.58212 | 4.60E-06 | 2.15E-05 | Down |
| NUTM2F | 5.31341939 | -1.274421844 | 0.434111 | -2.9357 | 0.003328 | 0.00865 | Down |
| NWD1 | 253.7424508 | -2.398322007 | 0.200304 | -11.9734 | 4.90E-33 | 2.26E-31 | Down |
| NWD2 | 30.76600675 | -2.832226692 | 0.283071 | -10.0053 | 1.44E-23 | 3.50E-22 | Down |
| NXF2B | 2.091949468 | -2.502816812 | 0.61906 | -4.04293 | 5.28E-05 | 0.000205 | Down |
| NXNL1 | 1.325502039 | -2.368738727 | 0.55886 | -4.23852 | 2.25E-05 | 9.36E-05 | Down |
| NXPE2 | 3.754984968 | -1.033544828 | 0.293074 | -3.52657 | 0.000421 | 0.001363 | Down |
| NXPH2 | 1.653777114 | -1.716728204 | 0.4645 | -3.69586 | 0.000219 | 0.000755 | Down |
| NYAP1 | 19.10703194 | -1.391733682 | 0.173095 | -8.04031 | 8.96E-16 | 1.19E-14 | Down |
| OACYLP | 4.521603247 | 1.296815007 | 0.357805 | 3.624366 | 0.00029 | 0.000973 | Up |
| OCA2 | 181.5266312 | -2.463766822 | 0.180556 | -13.6454 | 2.15E-42 | 1.74E-40 | Down |
| OCIAD2 | 691.7525735 | 1.078326848 | 0.075831 | 14.22005 | 6.88E-46 | 6.96E-44 | Up |
| OCM | 2.426926565 | -1.076044231 | 0.237417 | -4.5323 | 5.83E-06 | 2.68E-05 | Down |
| ODAM | 33.59541641 | -1.708087197 | 0.248342 | -6.87797 | 6.07E-12 | 5.69E-11 | Down |
| ODF1 | 1.017379176 | -2.746805653 | 0.746163 | -3.68124 | 0.000232 | 0.000796 | Down |
| ODF3L1 | 10.09334986 | -1.783051105 | 0.21621 | -8.24683 | 1.63E-16 | 2.29E-15 | Down |
| ODF3L2 | 3.496269587 | -1.587986032 | 0.279627 | -5.67895 | 1.36E-08 | 8.93E-08 | Down |
| ODF4 | 1.423617493 | -2.590405247 | 1.046875 | -2.47442 | 0.013345 | 0.029281 | Down |
| OGDHL | 915.6339317 | -2.028203535 | 0.155142 | -13.0732 | 4.68E-39 | 3.08E-37 | Down |
| OGFOD1P1 | 6.42488422 | -1.16100623 | 0.235561 | -4.92869 | 8.28E-07 | 4.31E-06 | Down |
| OGFR-AS1 | 0.667220196 | -1.385658479 | 0.394144 | -3.51562 | 0.000439 | 0.001415 | Down |
| OGN | 680.8001542 | -1.250835102 | 0.190025 | -6.58248 | 4.63E-11 | 3.97E-10 | Down |
| OIT3 | 22.63333682 | 2.260869178 | 0.195874 | 11.54249 | 8.06E-31 | 3.26E-29 | Up |
| OLA1P2 | 1.717511646 | -3.19832019 | 0.906727 | -3.52732 | 0.00042 | 0.001359 | Down |
| OLA1P3 | 2.044894447 | -1.753068245 | 0.552648 | -3.17212 | 0.001513 | 0.004307 | Down |
| OLAH | 15.91255988 | 2.051375497 | 0.282381 | 7.264578 | 3.74E-13 | 3.93E-12 | Up |
| OLFM2 | 142.5154229 | -1.156259898 | 0.119841 | -9.64826 | 5.00E-22 | 1.08E-20 | Down |
| OLFM3 | 12.33784976 | -2.790897702 | 0.261428 | -10.6756 | 1.32E-26 | 3.98E-25 | Down |
| OLFM4 | 17.42282726 | -1.808528211 | 0.282945 | -6.39181 | 1.64E-10 | 1.34E-09 | Down |
| OLR1 | 171.9866629 | 2.100281244 | 0.222925 | 9.42148 | 4.45E-21 | 8.99E-20 | Up |
| OPCML | 62.65805715 | 1.609633219 | 0.315026 | 5.109525 | 3.23E-07 | 1.78E-06 | Up |
| OPCML-IT1 | 1.770452473 | 2.570230145 | 0.621376 | 4.136354 | 3.53E-05 | 0.000142 | Up |
| OPN5 | 2.394925883 | 1.80125611 | 0.465398 | 3.87036 | 0.000109 | 0.000397 | Up |
| OPRK1 | 12.66902851 | -1.845672485 | 0.315717 | -5.84597 | 5.04E-09 | 3.48E-08 | Down |
| OR10A7 | 1.462432717 | -3.183719596 | 0.996321 | -3.19548 | 0.001396 | 0.004005 | Down |
| OR10AB1P | 1.999930907 | -1.635622971 | 0.344134 | -4.75287 | 2.01E-06 | 9.89E-06 | Down |
| OR10AH1P | 1.775363169 | -1.826114363 | 0.77424 | -2.35859 | 0.018345 | 0.03839 | Down |
| OR10D1P | 1.449087079 | -1.516970202 | 0.547571 | -2.77036 | 0.005599 | 0.013692 | Down |
| OR10D3 | 1.557963685 | -2.268820064 | 0.846689 | -2.67964 | 0.00737 | 0.017428 | Down |
| OR10G2 | 2.067986141 | -1.673407318 | 0.533288 | -3.1379 | 0.001702 | 0.004784 | Down |
| OR10G6 | 2.007978312 | -2.879831485 | 0.653087 | -4.40957 | 1.04E-05 | 4.56E-05 | Down |
| OR10H2 | 2.691822543 | -2.30394444 | 0.690188 | -3.33814 | 0.000843 | 0.002547 | Down |
| OR10H4 | 1.593832208 | -2.544210124 | 0.799794 | -3.18108 | 0.001467 | 0.004188 | Down |
| OR10H5 | 2.913960487 | -2.708462563 | 0.704163 | -3.84636 | 0.00012 | 0.000435 | Down |
| OR10J1 | 1.474364473 | -1.855477232 | 0.756176 | -2.45376 | 0.014137 | 0.030727 | Down |
| OR10J3 | 1.36574036 | -2.704827607 | 0.875978 | -3.08778 | 0.002017 | 0.005555 | Down |
| OR10J7P | 1.371817847 | -3.135669705 | 1.370793 | -2.28749 | 0.022167 | 0.045168 | Down |
| OR10K1 | 2.948269308 | -2.63784321 | 0.841806 | -3.13355 | 0.001727 | 0.004847 | Down |
| OR10K2 | 1.277985652 | -2.133955714 | 0.905044 | -2.35785 | 0.018381 | 0.038459 | Down |
| OR10P1 | 1.757647565 | -1.786181917 | 0.711958 | -2.50883 | 0.012113 | 0.026924 | Down |
| OR10Q2P | 1.275360421 | -2.640097169 | 1.099775 | -2.40058 | 0.016369 | 0.034808 | Down |
| OR10S1 | 1.507228934 | -1.351891412 | 0.599942 | -2.25337 | 0.024236 | 0.048782 | Down |
| OR10V1 | 0.995936145 | -2.317043102 | 0.757766 | -3.05773 | 0.00223 | 0.00607 | Down |
| OR10V3P | 1.093847598 | -1.493423277 | 0.660605 | -2.26069 | 0.023778 | 0.047965 | Down |
| OR11G1P | 1.531162 | -3.074474101 | 0.890192 | -3.45372 | 0.000553 | 0.001742 | Down |
| OR11H1 | 1.882905239 | -1.804173253 | 0.783462 | -2.30282 | 0.021289 | 0.043635 | Down |
| OR11H12 | 1.325610092 | -1.899678007 | 0.81898 | -2.31957 | 0.020364 | 0.041993 | Down |
| OR11H13P | 1.768282794 | -1.911361511 | 0.6873 | -2.78097 | 0.00542 | 0.013305 | Down |
| OR11H4 | 1.868853766 | -1.768406844 | 0.536164 | -3.29826 | 0.000973 | 0.002899 | Down |
| OR11K2P | 1.384840797 | -2.847117825 | 1.142421 | -2.49218 | 0.012696 | 0.02805 | Down |
| OR11L1 | 2.133345836 | -2.377643839 | 0.430765 | -5.51958 | 3.40E-08 | 2.13E-07 | Down |
| OR13C1P | 3.070467028 | -1.003367981 | 0.38945 | -2.57637 | 0.009984 | 0.022726 | Down |
| OR13C3 | 1.896310645 | -1.925195188 | 0.490994 | -3.92102 | 8.82E-05 | 0.000328 | Down |
| OR13C4 | 1.266068813 | -1.387899028 | 0.555024 | -2.50061 | 0.012398 | 0.027463 | Down |
| OR13C5 | 1.792702479 | -1.919369263 | 0.47438 | -4.04606 | 5.21E-05 | 0.000203 | Down |
| OR13C8 | 1.348910119 | -1.403392241 | 0.587893 | -2.38715 | 0.016979 | 0.035922 | Down |
| OR13C9 | 2.574303948 | -1.061450213 | 0.377349 | -2.81291 | 0.004909 | 0.012187 | Down |
| OR13D1 | 6.200979462 | -1.089726615 | 0.307186 | -3.54745 | 0.000389 | 0.001271 | Down |
| OR13D2P | 1.220007875 | -2.366811429 | 0.636398 | -3.71908 | 0.0002 | 0.000695 | Down |
| OR13F1 | 1.238475808 | -1.282281959 | 0.54783 | -2.34066 | 0.01925 | 0.039988 | Down |
| OR13G1 | 1.568075963 | -1.814982402 | 0.393732 | -4.6097 | 4.03E-06 | 1.90E-05 | Down |
| OR13H1 | 1.76581207 | -2.783120465 | 0.947897 | -2.9361 | 0.003324 | 0.008642 | Down |
| OR13I1P | 2.503043169 | -1.405037498 | 0.418602 | -3.3565 | 0.000789 | 0.002397 | Down |
| OR14A16 | 1.358708835 | -2.303233733 | 0.549117 | -4.19443 | 2.74E-05 | 0.000112 | Down |
| OR14A2 | 1.82341084 | -1.702883623 | 0.465978 | -3.65443 | 0.000258 | 0.000876 | Down |
| OR14J1 | 1.260142662 | -2.204695296 | 0.886686 | -2.48644 | 0.012903 | 0.028448 | Down |
| OR14K1 | 1.266657555 | -1.207386736 | 0.504061 | -2.39532 | 0.016606 | 0.035254 | Down |
| OR1A1 | 1.466829506 | -2.276179794 | 0.812512 | -2.80141 | 0.005088 | 0.012584 | Down |
| OR1A2 | 1.317465243 | -2.490247718 | 0.861784 | -2.88964 | 0.003857 | 0.009851 | Down |
| OR1AA1P | 1.286544519 | -2.794585117 | 1.185361 | -2.35758 | 0.018394 | 0.038483 | Down |
| OR1B1 | 1.968161889 | -1.674149579 | 0.482178 | -3.47206 | 0.000516 | 0.001638 | Down |
| OR1C1 | 2.262940396 | -2.647913162 | 0.560983 | -4.72013 | 2.36E-06 | 1.15E-05 | Down |
| OR1D2 | 1.569649265 | -2.112323357 | 0.812499 | -2.59979 | 0.009328 | 0.021433 | Down |
| OR1D3P | 1.473883128 | -2.245720315 | 0.7827 | -2.8692 | 0.004115 | 0.010429 | Down |
| OR1D4 | 1.860914835 | -1.930467172 | 0.751329 | -2.5694 | 0.010187 | 0.023109 | Down |
| OR1E1 | 1.950932076 | -1.716865233 | 0.625694 | -2.74394 | 0.006071 | 0.0147 | Down |
| OR1F1 | 1.638137948 | -2.292618229 | 0.820454 | -2.79433 | 0.005201 | 0.012839 | Down |
| OR1F12 | 5.588397741 | -1.374283977 | 0.290046 | -4.73816 | 2.16E-06 | 1.06E-05 | Down |
| OR1F2P | 2.121721014 | -2.776395878 | 0.674194 | -4.1181 | 3.82E-05 | 0.000153 | Down |
| OR1H1P | 1.992381377 | -1.245193311 | 0.438922 | -2.83694 | 0.004555 | 0.011421 | Down |
| OR1J2 | 1.385846393 | -1.536363742 | 0.574045 | -2.67638 | 0.007442 | 0.017577 | Down |
| OR1J4 | 1.935977208 | -2.309484084 | 0.659879 | -3.49986 | 0.000466 | 0.001492 | Down |
| OR1L1 | 1.667093887 | -1.991148748 | 0.552139 | -3.60625 | 0.000311 | 0.001037 | Down |
| OR1L3 | 1.512890441 | -2.499155082 | 0.590271 | -4.23391 | 2.30E-05 | 9.54E-05 | Down |
| OR1L4 | 3.505283069 | -1.400736097 | 0.382054 | -3.66633 | 0.000246 | 0.000839 | Down |
| OR1L6 | 2.247816808 | -1.479694645 | 0.447698 | -3.30512 | 0.000949 | 0.002835 | Down |
| OR1L8 | 3.102454778 | -1.417621529 | 0.366733 | -3.86554 | 0.000111 | 0.000404 | Down |
| OR1M1 | 1.554513226 | -3.05575612 | 0.81607 | -3.74448 | 0.000181 | 0.000634 | Down |
| OR1N2 | 1.620416533 | -1.518280896 | 0.567269 | -2.67648 | 0.00744 | 0.017573 | Down |
| OR1P1 | 1.10432821 | -2.377856508 | 1.019401 | -2.3326 | 0.019669 | 0.040759 | Down |
| OR1Q1 | 2.644824209 | -1.349211486 | 0.449825 | -2.99942 | 0.002705 | 0.007182 | Down |
| OR1S1 | 1.737406422 | -2.675953129 | 0.795637 | -3.36328 | 0.00077 | 0.002345 | Down |
| OR1X5P | 1.590706624 | -1.794648686 | 0.763185 | -2.35153 | 0.018697 | 0.038996 | Down |
| OR2A1 | 61.1397174 | -1.054585657 | 0.196308 | -5.3721 | 7.78E-08 | 4.65E-07 | Down |
| OR2A42 | 20.7011478 | -1.186202544 | 0.273902 | -4.33076 | 1.49E-05 | 6.38E-05 | Down |
| OR2AI1P | 1.020949542 | -1.907177182 | 0.85008 | -2.24353 | 0.024863 | 0.049868 | Down |
| OR2AJ1 | 1.600710264 | -2.033391564 | 0.490391 | -4.14647 | 3.38E-05 | 0.000136 | Down |
| OR2AK2 | 2.459948742 | -1.767031308 | 0.408005 | -4.3309 | 1.48E-05 | 6.38E-05 | Down |
| OR2B11 | 2.681494741 | -1.390983398 | 0.370439 | -3.75496 | 0.000173 | 0.000611 | Down |
| OR2B4P | 1.295207257 | -2.796117562 | 1.099307 | -2.54353 | 0.010974 | 0.024658 | Down |
| OR2B7P | 1.861129769 | -1.915320578 | 0.455251 | -4.20717 | 2.59E-05 | 0.000106 | Down |
| OR2B8P | 2.174757533 | -1.780772357 | 0.426692 | -4.17343 | 3.00E-05 | 0.000122 | Down |
| OR2F1 | 2.771341069 | -1.136583147 | 0.359548 | -3.16115 | 0.001571 | 0.004454 | Down |
| OR2F2 | 2.469460823 | -1.16290475 | 0.401532 | -2.89617 | 0.003777 | 0.009676 | Down |
| OR2G2 | 1.631583762 | -1.754337773 | 0.717939 | -2.44358 | 0.014543 | 0.031482 | Down |
| OR2G6 | 1.495387286 | -1.678094985 | 0.625366 | -2.68338 | 0.007288 | 0.017256 | Down |
| OR2H2 | 2.549117504 | -1.703972873 | 0.461457 | -3.69259 | 0.000222 | 0.000765 | Down |
| OR2J1 | 1.689547217 | -1.489361218 | 0.618249 | -2.409 | 0.015996 | 0.03412 | Down |
| OR2J2 | 1.979306976 | -1.713271793 | 0.618843 | -2.76851 | 0.005631 | 0.013762 | Down |
| OR2K2 | 0.995418027 | -2.23788229 | 0.953506 | -2.347 | 0.018925 | 0.03941 | Down |
| OR2L13 | 4.182997688 | -2.089578646 | 0.365337 | -5.71959 | 1.07E-08 | 7.13E-08 | Down |
| OR2L1P | 2.898739452 | -1.9217969 | 0.399296 | -4.81296 | 1.49E-06 | 7.47E-06 | Down |
| OR2L2 | 1.686456183 | -1.417354304 | 0.518499 | -2.73357 | 0.006265 | 0.015119 | Down |
| OR2L3 | 2.117669781 | -1.980605153 | 0.487249 | -4.06488 | 4.81E-05 | 0.000188 | Down |
| OR2L5 | 1.955116697 | -1.583079002 | 0.426662 | -3.71038 | 0.000207 | 0.000717 | Down |
| OR2L6P | 2.730385524 | -2.017327155 | 0.42711 | -4.72321 | 2.32E-06 | 1.13E-05 | Down |
| OR2L8 | 2.79713904 | -1.695362651 | 0.393801 | -4.30512 | 1.67E-05 | 7.10E-05 | Down |
| OR2L9P | 1.774703549 | -2.059933098 | 0.497552 | -4.14013 | 3.47E-05 | 0.00014 | Down |
| OR2M2 | 1.785846355 | -2.153222863 | 0.818643 | -2.63023 | 0.008533 | 0.019834 | Down |
| OR2M3 | 2.5638333 | -2.405399111 | 0.659917 | -3.645 | 0.000267 | 0.000906 | Down |
| OR2M5 | 1.261748557 | -2.163745781 | 0.689168 | -3.13965 | 0.001692 | 0.004761 | Down |
| OR2N1P | 1.362863828 | -3.010732274 | 1.055203 | -2.85323 | 0.004328 | 0.010909 | Down |
| OR2S1P | 1.763988088 | -1.262260786 | 0.516352 | -2.44457 | 0.014502 | 0.031397 | Down |
| OR2S2 | 1.597304885 | -1.102453713 | 0.486693 | -2.26519 | 0.023501 | 0.047477 | Down |
| OR2T10 | 1.187415274 | -1.59208476 | 0.634711 | -2.50836 | 0.012129 | 0.026948 | Down |
| OR2T11 | 1.579996808 | -1.824622027 | 0.606744 | -3.00724 | 0.002636 | 0.007024 | Down |
| OR2T12 | 1.331080577 | -1.578247443 | 0.669973 | -2.35569 | 0.018488 | 0.038649 | Down |
| OR2T2 | 2.858493932 | -1.568189667 | 0.662791 | -2.36604 | 0.017979 | 0.037732 | Down |
| OR2T3 | 1.701871634 | -2.185780121 | 0.70095 | -3.11831 | 0.001819 | 0.005074 | Down |
| OR2T32P | 3.922969466 | -2.026025145 | 0.329647 | -6.14604 | 7.94E-10 | 6.04E-09 | Down |
| OR2T33 | 4.415662621 | -1.875742995 | 0.319165 | -5.87703 | 4.18E-09 | 2.91E-08 | Down |
| OR2T5 | 2.019874282 | -1.902234662 | 0.657655 | -2.89245 | 0.003823 | 0.009773 | Down |
| OR2T6 | 1.425156749 | -2.149690614 | 0.872697 | -2.46327 | 0.013768 | 0.030047 | Down |
| OR2T7 | 1.870302848 | -1.818503084 | 0.704682 | -2.5806 | 0.009863 | 0.022485 | Down |
| OR2T8 | 5.556469371 | -1.714223468 | 0.20589 | -8.32591 | 8.37E-17 | 1.21E-15 | Down |
| OR2V1 | 2.540699054 | -2.517746498 | 0.521239 | -4.83031 | 1.36E-06 | 6.89E-06 | Down |
| OR2V2 | 1.438164898 | -2.855355139 | 0.791153 | -3.60911 | 0.000307 | 0.001026 | Down |
| OR2W2P | 1.69781296 | -3.062489743 | 0.795651 | -3.84903 | 0.000119 | 0.00043 | Down |
| OR2W3 | 42.15813286 | -1.723068426 | 0.144096 | -11.9578 | 5.91E-33 | 2.72E-31 | Down |
| OR2W5 | 0.989372835 | -2.172241951 | 0.729624 | -2.97721 | 0.002909 | 0.007672 | Down |
| OR2W6P | 1.456327448 | -2.032618042 | 0.677819 | -2.99876 | 0.002711 | 0.007196 | Down |
| OR2X1P | 0.898021598 | -1.946396573 | 0.472836 | -4.11643 | 3.85E-05 | 0.000154 | Down |
| OR2Y1 | 1.312494719 | -2.704174579 | 0.956765 | -2.82637 | 0.004708 | 0.011758 | Down |
| OR3A3 | 1.594763089 | -1.573697487 | 0.538278 | -2.92358 | 0.00346 | 0.008951 | Down |
| OR3D1P | 1.513271058 | -2.225518017 | 0.509127 | -4.37124 | 1.24E-05 | 5.38E-05 | Down |
| OR4B1 | 1.341459858 | -2.67674254 | 0.725087 | -3.69162 | 0.000223 | 0.000767 | Down |
| OR4C4P | 2.04771957 | -3.140336197 | 1.077737 | -2.91382 | 0.00357 | 0.009205 | Down |
| OR4C50P | 1.674296326 | -3.023999489 | 0.996993 | -3.03312 | 0.00242 | 0.006521 | Down |
| OR4D1 | 1.996187597 | -2.135674721 | 0.76272 | -2.80008 | 0.005109 | 0.012631 | Down |
| OR4D10 | 7.068368992 | 2.132642904 | 0.415065 | 5.138089 | 2.78E-07 | 1.54E-06 | Up |
| OR4D11 | 1.177753082 | -1.759530276 | 0.73388 | -2.39757 | 0.016504 | 0.035063 | Down |
| OR4D5 | 1.252634555 | -1.719580944 | 0.530621 | -3.24069 | 0.001192 | 0.003483 | Down |
| OR4D6 | 5.402638475 | 1.824492328 | 0.327507 | 5.570845 | 2.54E-08 | 1.61E-07 | Up |
| OR4F14P | 1.689495492 | -2.360244436 | 0.98109 | -2.40574 | 0.01614 | 0.034378 | Down |
| OR4F28P | 2.436950182 | -2.132165281 | 0.849546 | -2.50977 | 0.012081 | 0.026859 | Down |
| OR4F3 | 3.590441232 | -1.669775902 | 0.723031 | -2.30941 | 0.020921 | 0.043003 | Down |
| OR4F6 | 2.080739872 | -3.181214212 | 0.991612 | -3.20812 | 0.001336 | 0.003851 | Down |
| OR4F7P | 2.493669352 | -1.443490746 | 0.615057 | -2.34692 | 0.018929 | 0.039417 | Down |
| OR4G11P | 1.78304664 | -2.820382516 | 1.13458 | -2.48584 | 0.012925 | 0.028487 | Down |
| OR4G1P | 1.380383694 | -2.440131771 | 1.074991 | -2.26991 | 0.023213 | 0.046974 | Down |
| OR4G2P | 1.905384062 | -2.416924365 | 0.978403 | -2.47028 | 0.013501 | 0.029562 | Down |
| OR4K11P | 1.272766703 | -2.483383591 | 0.993174 | -2.50045 | 0.012403 | 0.027473 | Down |
| OR4K14 | 1.605232316 | -2.688593459 | 1.072406 | -2.50707 | 0.012174 | 0.027027 | Down |
| OR4N1P | 1.394565971 | -1.963336935 | 0.732945 | -2.6787 | 0.007391 | 0.017471 | Down |
| OR4N2 | 2.383944039 | -2.027520212 | 0.700571 | -2.8941 | 0.003803 | 0.009729 | Down |
| OR4N5 | 1.150122993 | -2.029400883 | 0.821566 | -2.47016 | 0.013505 | 0.02957 | Down |
| OR4S1 | 1.762067898 | -2.610823923 | 1.003193 | -2.60251 | 0.009254 | 0.021277 | Down |
| OR51A5P | 1.282622041 | -3.263555457 | 1.369264 | -2.38344 | 0.017152 | 0.036229 | Down |
| OR51AB1P | 1.533368715 | -2.559436749 | 0.873157 | -2.93125 | 0.003376 | 0.008757 | Down |
| OR51C1P | 1.289342648 | -2.296361754 | 0.820359 | -2.79921 | 0.005123 | 0.012662 | Down |
| OR51G1 | 1.514783092 | -2.219741864 | 0.83421 | -2.66089 | 0.007793 | 0.018303 | Down |
| OR51H1 | 1.189725124 | -2.432329027 | 1.058572 | -2.29774 | 0.021576 | 0.04413 | Down |
| OR51L1 | 1.864981223 | -3.073075817 | 1.032508 | -2.97632 | 0.002917 | 0.007692 | Down |
| OR51R1P | 1.563438639 | -1.369816626 | 0.540562 | -2.53406 | 0.011275 | 0.025258 | Down |
| OR52A4 | 2.407902311 | -3.087549511 | 1.051658 | -2.93589 | 0.003326 | 0.008646 | Down |
| OR52B1P | 2.034097257 | -2.263341782 | 0.768702 | -2.94437 | 0.003236 | 0.008435 | Down |
| OR52B2 | 1.565342266 | -1.833187786 | 0.459154 | -3.99253 | 6.54E-05 | 0.000249 | Down |
| OR52B4 | 1.6212852 | -2.059939733 | 0.620547 | -3.31956 | 0.000902 | 0.002706 | Down |
| OR52J3 | 1.526640496 | -2.415824966 | 0.905678 | -2.66742 | 0.007644 | 0.018 | Down |
| OR52K1 | 1.544786623 | -1.397721602 | 0.54285 | -2.57478 | 0.01003 | 0.022815 | Down |
| OR52K2 | 2.044430198 | -1.573333466 | 0.544671 | -2.8886 | 0.00387 | 0.00988 | Down |
| OR52L1 | 1.309274642 | -1.911032593 | 0.628988 | -3.03826 | 0.002379 | 0.006428 | Down |
| OR52N3P | 1.693840195 | -1.803559431 | 0.734482 | -2.45555 | 0.014067 | 0.030584 | Down |
| OR52R1 | 1.775488847 | -2.369831226 | 0.931965 | -2.54283 | 0.010996 | 0.024696 | Down |
| OR52X1P | 1.095215349 | -2.601487309 | 1.100066 | -2.36485 | 0.018037 | 0.037831 | Down |
| OR55B1P | 2.222895269 | -1.023358019 | 0.336978 | -3.03687 | 0.00239 | 0.006452 | Down |
| OR56A1 | 1.312766493 | -2.309054184 | 0.75413 | -3.06188 | 0.0022 | 0.005996 | Down |
| OR56A3 | 1.599949567 | -2.187837632 | 0.722868 | -3.02661 | 0.002473 | 0.006646 | Down |
| OR56A5 | 1.252089263 | -2.298705411 | 0.884547 | -2.59874 | 0.009357 | 0.021493 | Down |
| OR56B3P | 0.882975273 | -1.540741858 | 0.6582 | -2.34084 | 0.01924 | 0.039971 | Down |
| OR56B4 | 2.809007363 | -1.200344573 | 0.357491 | -3.3577 | 0.000786 | 0.002388 | Down |
| OR5A1 | 4.625795907 | 2.539544588 | 0.432687 | 5.869239 | 4.38E-09 | 3.04E-08 | Up |
| OR5A2 | 5.102406096 | 3.339241231 | 0.474347 | 7.039653 | 1.93E-12 | 1.90E-11 | Up |
| OR5AK2 | 1.457369901 | -2.251124287 | 0.656403 | -3.42948 | 0.000605 | 0.001887 | Down |
| OR5AK3P | 1.347200778 | -2.707227645 | 0.887267 | -3.0512 | 0.002279 | 0.006183 | Down |
| OR5AN1 | 12.49973765 | 3.33590288 | 0.392993 | 8.488461 | 2.09E-17 | 3.18E-16 | Up |
| OR5AN2P | 16.78872815 | 4.181222536 | 0.410721 | 10.18019 | 2.43E-24 | 6.18E-23 | Up |
| OR5AP2 | 1.454028849 | -2.838330182 | 1.002449 | -2.8314 | 0.004635 | 0.011596 | Down |
| OR5AQ1P | 1.435684805 | -3.001560274 | 1.193068 | -2.51583 | 0.011875 | 0.026455 | Down |
| OR5AZ1P | 1.191263858 | -2.57965497 | 1.066811 | -2.4181 | 0.015602 | 0.033393 | Down |
| OR5B12 | 1.572992939 | -2.81147963 | 0.734865 | -3.82584 | 0.00013 | 0.000469 | Down |
| OR5B2 | 1.176929242 | -2.455006021 | 0.721095 | -3.40455 | 0.000663 | 0.00205 | Down |
| OR5B21 | 2.546115024 | -2.112970588 | 0.536202 | -3.94063 | 8.13E-05 | 0.000304 | Down |
| OR5BB1P | 14.57353429 | 3.409789269 | 0.433192 | 7.871317 | 3.51E-15 | 4.48E-14 | Up |
| OR5BR1P | 23.95629645 | 3.986472606 | 0.389492 | 10.23506 | 1.38E-24 | 3.57E-23 | Up |
| OR5D13 | 0.922032392 | -2.06922811 | 0.914745 | -2.26208 | 0.023692 | 0.04781 | Down |
| OR5D14 | 1.858278636 | -2.344498069 | 0.899474 | -2.60652 | 0.009147 | 0.021071 | Down |
| OR5D18 | 1.513580423 | -2.642078453 | 0.935789 | -2.82337 | 0.004752 | 0.011857 | Down |
| OR5G5P | 1.219160921 | -2.991377275 | 1.021255 | -2.92912 | 0.003399 | 0.008811 | Down |
| OR5H2 | 1.620491996 | -3.001289895 | 0.745923 | -4.02359 | 5.73E-05 | 0.000221 | Down |
| OR5H6 | 0.825498801 | -1.533946494 | 0.555693 | -2.76042 | 0.005773 | 0.014061 | Down |
| OR5H8 | 1.700651873 | -1.897649616 | 0.429533 | -4.41794 | 9.96E-06 | 4.40E-05 | Down |
| OR5J1P | 1.507858196 | -3.126019372 | 1.32807 | -2.35381 | 0.018582 | 0.038811 | Down |
| OR5K2 | 4.171613036 | -1.223483527 | 0.260625 | -4.69443 | 2.67E-06 | 1.30E-05 | Down |
| OR5L1 | 1.545143802 | -2.013708476 | 0.760761 | -2.64696 | 0.008122 | 0.018977 | Down |
| OR5L2 | 1.125616356 | -2.135453302 | 0.823811 | -2.59216 | 0.009537 | 0.021855 | Down |
| OR5P2 | 1.089323566 | -1.980408071 | 0.873541 | -2.2671 | 0.023384 | 0.047273 | Down |
| OR5R1 | 1.254807198 | -2.549936991 | 0.971777 | -2.62399 | 0.008691 | 0.020154 | Down |
| OR6B2 | 1.755452503 | -2.686920025 | 0.819939 | -3.27698 | 0.001049 | 0.003099 | Down |
| OR6C3 | 2.308370855 | -2.98873963 | 0.94356 | -3.16752 | 0.001537 | 0.004368 | Down |
| OR6C4 | 1.053538372 | -2.343200204 | 0.850393 | -2.75543 | 0.005861 | 0.014252 | Down |
| OR6C76 | 1.171451931 | -2.153749731 | 0.88641 | -2.42974 | 0.015109 | 0.032516 | Down |
| OR6D1P | 1.997749885 | -3.427870377 | 1.3697 | -2.50264 | 0.012327 | 0.027334 | Down |
| OR6E1P | 7.178037278 | -1.242729581 | 0.210858 | -5.89367 | 3.78E-09 | 2.65E-08 | Down |
| OR6F1 | 1.734700336 | -2.15088981 | 0.4231 | -5.08364 | 3.70E-07 | 2.02E-06 | Down |
| OR6J1 | 1.462627185 | -2.125979019 | 0.597646 | -3.55726 | 0.000375 | 0.001229 | Down |
| OR6K2 | 1.66727834 | -3.002202509 | 1.081507 | -2.77594 | 0.005504 | 0.01349 | Down |
| OR6K3 | 1.516565411 | -2.132915937 | 0.946773 | -2.25283 | 0.02427 | 0.048832 | Down |
| OR6K6 | 1.483125185 | -1.950282732 | 0.804136 | -2.42532 | 0.015295 | 0.032848 | Down |
| OR6L1P | 1.974105782 | -1.367674054 | 0.603737 | -2.26535 | 0.023491 | 0.04746 | Down |
| OR6M1 | 2.107310416 | -2.26321379 | 0.644481 | -3.51169 | 0.000445 | 0.001434 | Down |
| OR6M2P | 2.372951793 | -2.024241531 | 0.525772 | -3.85003 | 0.000118 | 0.000429 | Down |
| OR6M3P | 1.166654084 | -2.331970255 | 0.683961 | -3.40951 | 0.000651 | 0.002017 | Down |
| OR6R1P | 3.786085341 | -3.018480489 | 0.313589 | -9.62559 | 6.24E-22 | 1.34E-20 | Down |
| OR6T1 | 1.758339315 | -1.778491497 | 0.400938 | -4.43583 | 9.17E-06 | 4.08E-05 | Down |
| OR6V1 | 2.272975071 | -1.778889824 | 0.464975 | -3.82578 | 0.00013 | 0.000469 | Down |
| OR6W1P | 1.602583189 | -1.786173666 | 0.502252 | -3.55633 | 0.000376 | 0.001233 | Down |
| OR6X1 | 1.665122201 | -1.779706705 | 0.580693 | -3.0648 | 0.002178 | 0.005946 | Down |
| OR7A19P | 5.683437766 | -1.29604154 | 0.276334 | -4.69012 | 2.73E-06 | 1.32E-05 | Down |
| OR7C2 | 1.546782261 | -2.376275265 | 0.979019 | -2.4272 | 0.015216 | 0.032715 | Down |
| OR7D2 | 8.255314584 | -1.162445569 | 0.42597 | -2.72894 | 0.006354 | 0.015309 | Down |
| OR7D4 | 1.597766995 | -2.651348978 | 0.748832 | -3.54064 | 0.000399 | 0.0013 | Down |
| OR7E105P | 1.785437265 | -1.843667344 | 0.654551 | -2.81669 | 0.004852 | 0.012069 | Down |
| OR7E108P | 1.920800573 | -2.113441227 | 0.453735 | -4.65788 | 3.19E-06 | 1.53E-05 | Down |
| OR7E109P | 1.483368497 | -2.673626867 | 0.954448 | -2.80123 | 0.005091 | 0.012589 | Down |
| OR7E116P | 2.171109112 | -2.861479982 | 0.426089 | -6.71569 | 1.87E-11 | 1.66E-10 | Down |
| OR7E11P | 1.293386994 | -2.570056955 | 0.811751 | -3.16607 | 0.001545 | 0.004388 | Down |
| OR7E121P | 1.327382761 | -3.250046517 | 1.168115 | -2.7823 | 0.005398 | 0.013263 | Down |
| OR7E136P | 1.307414885 | -2.642066448 | 0.749707 | -3.52413 | 0.000425 | 0.001374 | Down |
| OR7E155P | 1.210925706 | -2.292727581 | 0.723435 | -3.16922 | 0.001528 | 0.004345 | Down |
| OR7E158P | 1.951672999 | -2.056029528 | 0.490376 | -4.19276 | 2.76E-05 | 0.000113 | Down |
| OR7E15P | 2.046981804 | -3.540480107 | 1.176124 | -3.0103 | 0.00261 | 0.006962 | Down |
| OR7E162P | 1.313289827 | -3.097409435 | 1.284764 | -2.41088 | 0.015914 | 0.033966 | Down |
| OR7E18P | 1.07669841 | -3.078086514 | 1.356733 | -2.26875 | 0.023284 | 0.047092 | Down |
| OR7E1P | 1.426287137 | -1.843161793 | 0.771821 | -2.38807 | 0.016937 | 0.035854 | Down |
| OR7E23P | 1.30343074 | -3.300029569 | 0.925185 | -3.56689 | 0.000361 | 0.001189 | Down |
| OR7E29P | 2.08976705 | -2.458107119 | 0.510821 | -4.81208 | 1.49E-06 | 7.50E-06 | Down |
| OR7E31P | 1.849750106 | -2.293597992 | 0.569436 | -4.02784 | 5.63E-05 | 0.000217 | Down |
| OR7E33P | 1.707334081 | -2.978897823 | 1.118168 | -2.66409 | 0.00772 | 0.018157 | Down |
| OR7E36P | 2.607936877 | -2.33044346 | 0.606188 | -3.84442 | 0.000121 | 0.000438 | Down |
| OR7E37P | 1.06183662 | -2.218969729 | 0.753223 | -2.94597 | 0.003219 | 0.008399 | Down |
| OR7E41P | 1.330780613 | -1.410332356 | 0.626387 | -2.25154 | 0.024352 | 0.048965 | Down |
| OR7E43P | 1.273184347 | -3.130584767 | 1.308907 | -2.39176 | 0.016768 | 0.035542 | Down |
| OR7E46P | 1.37909364 | -2.22500416 | 0.904021 | -2.46123 | 0.013846 | 0.030199 | Down |
| OR7E53P | 1.688767294 | -2.939910192 | 0.960446 | -3.06098 | 0.002206 | 0.006013 | Down |
| OR7E59P | 1.730870343 | -2.838491098 | 0.968954 | -2.92944 | 0.003396 | 0.008804 | Down |
| OR7E66P | 1.233940135 | -1.710827312 | 0.747445 | -2.2889 | 0.022085 | 0.045025 | Down |
| OR7E83P | 4.101269326 | -2.556438399 | 0.358143 | -7.13804 | 9.47E-13 | 9.60E-12 | Down |
| OR7E85P | 6.199077665 | -1.69997457 | 0.235347 | -7.22325 | 5.08E-13 | 5.26E-12 | Down |
| OR7E86P | 1.424369844 | -1.300168983 | 0.473782 | -2.74424 | 0.006065 | 0.01469 | Down |
| OR7E87P | 1.687927947 | -1.242202536 | 0.4649 | -2.67198 | 0.007541 | 0.017779 | Down |
| OR7E91P | 5.669502208 | -1.086095744 | 0.21751 | -4.99332 | 5.94E-07 | 3.16E-06 | Down |
| OR7G1 | 1.465976998 | -3.042793343 | 1.020446 | -2.98183 | 0.002865 | 0.007567 | Down |
| OR7G2 | 1.224475915 | -2.209421427 | 0.972891 | -2.27098 | 0.023148 | 0.046852 | Down |
| OR7K1P | 1.177525755 | -3.087837014 | 0.831919 | -3.7117 | 0.000206 | 0.000714 | Down |
| OR8A1 | 1.994887934 | -2.316056842 | 0.784782 | -2.95121 | 0.003165 | 0.008271 | Down |
| OR8B10P | 1.287430252 | -2.745673274 | 1.173188 | -2.34035 | 0.019266 | 0.040015 | Down |
| OR8B12 | 1.69729872 | -2.606638818 | 0.827008 | -3.15189 | 0.001622 | 0.004584 | Down |
| OR8B2 | 1.378795226 | -2.582890525 | 0.826757 | -3.12412 | 0.001783 | 0.004987 | Down |
| OR8D4 | 2.109445309 | -1.990078145 | 0.455844 | -4.3657 | 1.27E-05 | 5.51E-05 | Down |
| OR8G1 | 1.440509684 | -1.834938681 | 0.752757 | -2.43763 | 0.014784 | 0.031928 | Down |
| OR8G2P | 1.388794457 | -2.550789455 | 0.896071 | -2.84664 | 0.004418 | 0.011115 | Down |
| OR8G5 | 1.830378458 | -1.677260137 | 0.691054 | -2.4271 | 0.01522 | 0.032718 | Down |
| OR8H1 | 0.995889923 | -2.31946113 | 0.932072 | -2.4885 | 0.012828 | 0.02831 | Down |
| OR8U1 | 1.145603151 | -2.166990375 | 0.87621 | -2.47314 | 0.013393 | 0.02937 | Down |
| OR9A1P | 2.472423337 | -1.259437924 | 0.490102 | -2.56975 | 0.010177 | 0.023092 | Down |
| OR9A2 | 2.193581879 | -1.668224626 | 0.509681 | -3.27308 | 0.001064 | 0.003138 | Down |
| OR9G2P | 1.993001243 | -3.426342843 | 1.490362 | -2.299 | 0.021505 | 0.044012 | Down |
| OR9H1P | 0.711829011 | -1.465012169 | 0.572367 | -2.55957 | 0.01048 | 0.023694 | Down |
| OR9M1P | 1.249693702 | -2.907968426 | 1.111043 | -2.61733 | 0.008862 | 0.020502 | Down |
| OR9Q1 | 1.782496503 | -1.788442895 | 0.70176 | -2.54851 | 0.010818 | 0.024358 | Down |
| OR9Q2 | 1.62525303 | -2.261318178 | 0.767596 | -2.94598 | 0.003219 | 0.008399 | Down |
| OSBPL9P4 | 4.970794461 | -1.505991275 | 0.309141 | -4.87154 | 1.11E-06 | 5.67E-06 | Down |
| OSCAR | 48.60210375 | 1.734564886 | 0.220613 | 7.862472 | 3.77E-15 | 4.80E-14 | Up |
| OSM | 24.7504268 | 1.217634531 | 0.250459 | 4.861607 | 1.16E-06 | 5.94E-06 | Up |
| OSR1 | 155.3768976 | -2.050882352 | 0.18234 | -11.2476 | 2.38E-29 | 8.74E-28 | Down |
| OSTCP1 | 1.760525403 | -1.313700648 | 0.463555 | -2.83397 | 0.004597 | 0.011512 | Down |
| OSTN | 5.324386607 | -2.47735268 | 0.389435 | -6.36141 | 2.00E-10 | 1.62E-09 | Down |
| OTC | 8.111617329 | -2.112448407 | 0.28042 | -7.53315 | 4.95E-14 | 5.68E-13 | Down |
| OTOL1 | 1.506441786 | -2.591912904 | 0.765011 | -3.38807 | 0.000704 | 0.002165 | Down |
| OTOP1 | 1.281428246 | -2.076268161 | 0.739394 | -2.80807 | 0.004984 | 0.012351 | Down |
| OTOP2 | 2.611910529 | -2.855017109 | 0.725532 | -3.93507 | 8.32E-05 | 0.000311 | Down |
| OTOP3 | 1.026218895 | -1.458859386 | 0.531439 | -2.74511 | 0.006049 | 0.014657 | Down |
| OTOS | 448.9915918 | -2.159945092 | 0.176322 | -12.25 | 1.68E-34 | 8.32E-33 | Down |
| OTX2 | 1.473161001 | -1.81190091 | 0.728185 | -2.48824 | 0.012838 | 0.028327 | Down |
| OVCH1 | 5.591106004 | 1.048185678 | 0.338326 | 3.09815 | 0.001947 | 0.005387 | Up |
| OXCT2 | 29.76497303 | -1.029584426 | 0.159303 | -6.46307 | 1.03E-10 | 8.54E-10 | Down |
| OXCT2P1 | 27.51711172 | -1.642344552 | 0.177875 | -9.23312 | 2.63E-20 | 5.00E-19 | Down |
| OXGR1 | 13.28253669 | -1.189792329 | 0.199553 | -5.96227 | 2.49E-09 | 1.78E-08 | Down |
| P2RX2 | 3.617907998 | -1.392201407 | 0.332485 | -4.18727 | 2.82E-05 | 0.000115 | Down |
| P2RX3 | 2.53259647 | -1.683880152 | 0.576899 | -2.91885 | 0.003513 | 0.009073 | Down |
| P2RX5 | 102.6880967 | -1.099424283 | 0.391841 | -2.80579 | 0.005019 | 0.012428 | Down |
| P2RY6 | 108.2467787 | 1.648476465 | 0.159723 | 10.32086 | 5.67E-25 | 1.51E-23 | Up |
| P3H2-AS1 | 1.081473053 | -2.064887974 | 0.812891 | -2.54018 | 0.01108 | 0.024868 | Down |
| P4HA2 | 956.4036361 | 1.758857297 | 0.07923 | 22.19948 | 3.47E-109 | 4.07E-106 | Up |
| PA2G4P2 | 2.379873084 | -1.819216275 | 0.458384 | -3.96876 | 7.22E-05 | 0.000273 | Down |
| PA2G4P3 | 1.824048488 | -3.172377655 | 1.354104 | -2.34279 | 0.01914 | 0.039799 | Down |
| PABPC1L2B | 2.067307586 | -1.485663067 | 0.582075 | -2.55236 | 0.0107 | 0.024124 | Down |
| PABPC1P11 | 3.276635022 | -2.776014996 | 0.497659 | -5.57815 | 2.43E-08 | 1.55E-07 | Down |
| PABPC1P2 | 1.407515788 | -2.83446209 | 0.779482 | -3.63634 | 0.000277 | 0.000934 | Down |
| PABPC3 | 9.509268961 | -1.296218869 | 0.200223 | -6.47388 | 9.55E-11 | 7.97E-10 | Down |
| PABPC5-AS1 | 6.012322214 | -1.197764255 | 0.148054 | -8.09008 | 5.96E-16 | 8.02E-15 | Down |
| PABPN1L | 1.811544445 | -1.298439023 | 0.38047 | -3.41273 | 0.000643 | 0.001995 | Down |
| PACRG-AS1 | 1.647717011 | -1.665559324 | 0.555681 | -2.99733 | 0.002724 | 0.007226 | Down |
| PADI3 | 1.830580943 | -2.862351749 | 0.971483 | -2.94637 | 0.003215 | 0.00839 | Down |
| PADI4 | 6.421320969 | -1.422031232 | 0.302988 | -4.69336 | 2.69E-06 | 1.30E-05 | Down |
| PAH | 18.06377703 | -1.256594822 | 0.220654 | -5.69487 | 1.23E-08 | 8.18E-08 | Down |
| PAICSP2 | 1.576421684 | -2.851099312 | 1.028843 | -2.77117 | 0.005586 | 0.013664 | Down |
| PAICSP4 | 1.499919856 | -1.504472082 | 0.55579 | -2.70691 | 0.006791 | 0.016241 | Down |
| PAICSP5 | 2.727912693 | -1.016273287 | 0.426941 | -2.38036 | 0.017296 | 0.036492 | Down |
| PAK3 | 633.6272578 | -1.694472602 | 0.143581 | -11.8015 | 3.84E-32 | 1.69E-30 | Down |
| PAK7 | 7.596288414 | -1.94669538 | 0.302412 | -6.43723 | 1.22E-10 | 1.01E-09 | Down |
| PALM3 | 214.6354228 | 1.127615117 | 0.172738 | 6.527899 | 6.67E-11 | 5.64E-10 | Up |
| PAM | 7532.100518 | 1.06002615 | 0.066448 | 15.95272 | 2.73E-57 | 4.85E-55 | Up |
| PAMR1 | 178.2203798 | -1.176400555 | 0.127236 | -9.24584 | 2.33E-20 | 4.46E-19 | Down |
| PANX3 | 1.487882253 | -1.699931954 | 0.663756 | -2.56108 | 0.010435 | 0.023609 | Down |
| PAPLN | 2133.024341 | 1.071269162 | 0.079836 | 13.41833 | 4.72E-41 | 3.57E-39 | Up |
| PAPOLB | 10.97024586 | -2.027397928 | 0.268855 | -7.54086 | 4.67E-14 | 5.36E-13 | Down |
| PAPPA2 | 94.19770578 | -1.259245638 | 0.170936 | -7.36676 | 1.75E-13 | 1.90E-12 | Down |
| PAPSS2 | 810.1966868 | -1.343766751 | 0.142267 | -9.44541 | 3.54E-21 | 7.21E-20 | Down |
| PAQR4 | 153.0222743 | 1.054923986 | 0.116842 | 9.02866 | 1.74E-19 | 3.12E-18 | Up |
| PARP14 | 7020.034901 | 1.0448308 | 0.083639 | 12.49221 | 8.23E-36 | 4.50E-34 | Up |
| PARP4 | 3818.883775 | 1.015134854 | 0.057268 | 17.72618 | 2.63E-70 | 7.95E-68 | Up |
| PART1 | 28.59952719 | -2.097219049 | 0.272398 | -7.69911 | 1.37E-14 | 1.66E-13 | Down |
| PATE1 | 2.09896783 | -2.125949073 | 0.659509 | -3.22353 | 0.001266 | 0.003673 | Down |
| PATE2 | 2.08598839 | -1.581794581 | 0.530187 | -2.98347 | 0.00285 | 0.00753 | Down |
| PATE3 | 1.572051995 | -2.571612037 | 0.795883 | -3.23114 | 0.001233 | 0.003591 | Down |
| PATE4 | 5.2750821 | -1.232193399 | 0.381557 | -3.22938 | 0.001241 | 0.00361 | Down |
| PAWRP1 | 2.389244097 | -1.528699763 | 0.383521 | -3.98596 | 6.72E-05 | 0.000255 | Down |
| PAX1 | 25.35966634 | -2.293854183 | 0.300511 | -7.63319 | 2.29E-14 | 2.72E-13 | Down |
| PAX4 | 1.476022707 | -2.027869863 | 0.750984 | -2.70029 | 0.006928 | 0.016513 | Down |
| PAX5 | 216.6849277 | -1.250121421 | 0.410634 | -3.04437 | 0.002332 | 0.006311 | Down |
| PAX8-AS1 | 3432.226185 | -1.06530025 | 0.179588 | -5.93191 | 2.99E-09 | 2.13E-08 | Down |
| PBX4 | 178.764783 | -2.14007596 | 0.149952 | -14.2717 | 3.28E-46 | 3.35E-44 | Down |
| PC | 774.1949837 | 1.349517459 | 0.085634 | 15.75913 | 5.94E-56 | 1.02E-53 | Up |
| PCAT14 | 24.37106786 | -2.353325079 | 0.235625 | -9.98757 | 1.73E-23 | 4.16E-22 | Down |
| PCAT29 | 3.845987652 | -1.212853936 | 0.359165 | -3.37687 | 0.000733 | 0.002244 | Down |
| PCDH11X | 104.289593 | -2.09927121 | 0.271284 | -7.73827 | 1.01E-14 | 1.24E-13 | Down |
| PCDH20 | 37.50735787 | 1.000659307 | 0.212792 | 4.702532 | 2.57E-06 | 1.25E-05 | Up |
| PCDH8P1 | 6.64350167 | -2.896398662 | 0.358394 | -8.0816 | 6.39E-16 | 8.59E-15 | Down |
| PCDHAC1 | 152.9286517 | 1.101341147 | 0.114261 | 9.638784 | 5.48E-22 | 1.18E-20 | Up |
| PCDHAC2 | 267.1457756 | 1.731497189 | 0.132327 | 13.08501 | 4.01E-39 | 2.66E-37 | Up |
| PCDHB17P | 9.84599157 | -1.296577589 | 0.298508 | -4.34353 | 1.40E-05 | 6.04E-05 | Down |
| PCGF7P | 1.816836754 | -1.647461658 | 0.582764 | -2.82698 | 0.004699 | 0.011738 | Down |
| PCK1 | 18.29258264 | -2.264371969 | 0.31013 | -7.30136 | 2.85E-13 | 3.03E-12 | Down |
| PCNAP1 | 1.397830666 | -1.908731097 | 0.464926 | -4.10545 | 4.04E-05 | 0.00016 | Down |
| PCNPP3 | 0.951577334 | 1.615914326 | 0.520231 | 3.106146 | 0.001895 | 0.005263 | Up |
| PCOLCE-AS1 | 7.53201767 | -1.198130405 | 0.303364 | -3.94948 | 7.83E-05 | 0.000294 | Down |
| PCOLCE2 | 146.7699663 | -1.650764476 | 0.191627 | -8.61446 | 7.03E-18 | 1.11E-16 | Down |
| PCSK1N | 193.5963857 | 3.563764763 | 0.263998 | 13.49919 | 1.58E-41 | 1.22E-39 | Up |
| PCSK2 | 3170.781016 | 3.692147346 | 0.246279 | 14.99172 | 8.32E-51 | 1.05E-48 | Up |
| PCSK6 | 166.121992 | -1.575435375 | 0.135074 | -11.6635 | 1.96E-31 | 8.31E-30 | Down |
| PDCL2 | 0.510994287 | 1.690789195 | 0.613444 | 2.756223 | 0.005847 | 0.014221 | Up |
| PDCL3P1 | 1.818874545 | -3.100070873 | 1.013348 | -3.05924 | 0.002219 | 0.006042 | Down |
| PDCL3P6 | 3.950036109 | -1.383736541 | 0.347533 | -3.9816 | 6.85E-05 | 0.00026 | Down |
| PDE10A | 654.7581353 | -1.48152299 | 0.110912 | -13.3577 | 1.07E-40 | 7.79E-39 | Down |
| PDE2A | 502.3852233 | -1.287604893 | 0.105046 | -12.2575 | 1.53E-34 | 7.61E-33 | Down |
| PDE4C | 169.0433922 | 2.489429622 | 0.21076 | 11.81166 | 3.40E-32 | 1.51E-30 | Up |
| PDE5A | 7920.441508 | 2.2250438 | 0.107146 | 20.76647 | 8.70E-96 | 6.74E-93 | Up |
| PDE9A | 315.952063 | 1.069585627 | 0.091717 | 11.66185 | 2.00E-31 | 8.47E-30 | Up |
| PDGFC | 1534.796493 | 1.220806922 | 0.078102 | 15.63096 | 4.48E-55 | 7.27E-53 | Up |
| PDILT | 0.990748136 | -2.325236822 | 0.791979 | -2.93598 | 0.003325 | 0.008645 | Down |
| PDLIM1P1 | 1.815018751 | -1.418726688 | 0.507119 | -2.79762 | 0.005148 | 0.012721 | Down |
| PDLIM4 | 1274.275885 | 2.901292296 | 0.102559 | 28.28899 | 4.72E-176 | 9.69E-172 | Up |
| PDSS1P2 | 1.177754372 | -2.081380019 | 0.619906 | -3.35757 | 0.000786 | 0.002389 | Down |
| PDZK1IP1 | 251.1118352 | 3.841180917 | 0.2125 | 18.07614 | 4.91E-73 | 1.63E-70 | Up |
| PDZRN4 | 298.3281144 | 2.391838213 | 0.224637 | 10.64755 | 1.79E-26 | 5.30E-25 | Up |
| PEAR1 | 384.7149305 | -1.154100655 | 0.106839 | -10.8022 | 3.36E-27 | 1.06E-25 | Down |
| PELI1 | 593.0854598 | 1.025914719 | 0.091197 | 11.24948 | 2.33E-29 | 8.56E-28 | Up |
| PERM1 | 17.52705693 | 1.986077268 | 0.238245 | 8.336283 | 7.67E-17 | 1.11E-15 | Up |
| PERP | 2835.293217 | 1.946447582 | 0.107854 | 18.04712 | 8.31E-73 | 2.73E-70 | Up |
| PEX5L | 45.86758257 | -1.973106116 | 0.212231 | -9.29696 | 1.45E-20 | 2.80E-19 | Down |
| PF4 | 5.636750106 | -1.656022341 | 0.258158 | -6.41475 | 1.41E-10 | 1.16E-09 | Down |
| PFKFB1 | 11.33024785 | -1.912133709 | 0.186797 | -10.2364 | 1.36E-24 | 3.53E-23 | Down |
| PGA3 | 66.25765156 | -1.908439773 | 0.180693 | -10.5618 | 4.48E-26 | 1.29E-24 | Down |
| PGA4 | 52.33884458 | -1.760422281 | 0.190283 | -9.2516 | 2.21E-20 | 4.24E-19 | Down |
| PGA5 | 28.66650209 | -1.730283198 | 0.180058 | -9.60958 | 7.28E-22 | 1.55E-20 | Down |
| PGAM1P10 | 3.929510714 | -1.444642093 | 0.262469 | -5.50404 | 3.71E-08 | 2.31E-07 | Down |
| PGBD4P1 | 2.42878943 | 1.704106355 | 0.490241 | 3.476055 | 0.000509 | 0.001616 | Up |
| PGC | 1.296918543 | -1.252694796 | 0.51147 | -2.4492 | 0.014317 | 0.031057 | Down |
| PGDP1 | 1.5382744 | -1.868501783 | 0.725923 | -2.57397 | 0.010054 | 0.022856 | Down |
| PGF | 1121.489391 | -1.302534674 | 0.113475 | -11.4786 | 1.69E-30 | 6.69E-29 | Down |
| PGGT1BP1 | 1.472893261 | -1.925145707 | 0.48612 | -3.96023 | 7.49E-05 | 0.000282 | Down |
| PGK1P2 | 2.543852796 | -1.342711404 | 0.331492 | -4.05051 | 5.11E-05 | 0.000199 | Down |
| PGK2 | 2.118747552 | -3.491060267 | 0.661992 | -5.27357 | 1.34E-07 | 7.77E-07 | Down |
| PGLYRP2 | 3.944570005 | -1.303821229 | 0.311726 | -4.18258 | 2.88E-05 | 0.000118 | Down |
| PGM5 | 817.364847 | -1.549671824 | 0.13048 | -11.8767 | 1.56E-32 | 7.06E-31 | Down |
| PGM5-AS1 | 9.826630867 | -2.317611043 | 0.230392 | -10.0594 | 8.35E-24 | 2.06E-22 | Down |
| PGM5P2 | 26.22291026 | -1.033273777 | 0.152595 | -6.77135 | 1.28E-11 | 1.16E-10 | Down |
| PGM5P3-AS1 | 10.38392375 | -2.041829757 | 0.220618 | -9.25503 | 2.14E-20 | 4.10E-19 | Down |
| PGM5P4-AS1 | 5.090743171 | -1.373172753 | 0.247232 | -5.55419 | 2.79E-08 | 1.77E-07 | Down |
| PGPEP1L | 2.322765266 | -1.906082624 | 0.369516 | -5.15832 | 2.49E-07 | 1.40E-06 | Down |
| PHACTR3 | 21.00328497 | 1.517837037 | 0.254207 | 5.970874 | 2.36E-09 | 1.70E-08 | Up |
| PHBP7 | 3.675339731 | -1.21946973 | 0.259801 | -4.69386 | 2.68E-06 | 1.30E-05 | Down |
| PHEX | 350.1091299 | 1.674096263 | 0.249446 | 6.71125 | 1.93E-11 | 1.71E-10 | Up |
| PHF21B | 35.54946488 | -1.013215872 | 0.206932 | -4.89636 | 9.76E-07 | 5.03E-06 | Down |
| PHF5CP | 3.260453704 | -2.015365176 | 0.295703 | -6.81551 | 9.39E-12 | 8.63E-11 | Down |
| PHLDA2 | 74.52955018 | 2.137713967 | 0.125862 | 16.98456 | 1.07E-64 | 2.52E-62 | Up |
| PHLDA3 | 634.3857841 | 1.445998054 | 0.082239 | 17.58295 | 3.33E-69 | 9.49E-67 | Up |
| PHOX2A | 3.843246148 | -1.499835275 | 0.274377 | -5.46634 | 4.59E-08 | 2.83E-07 | Down |
| PHYHIP | 212.2628388 | 1.892986894 | 0.132192 | 14.31994 | 1.64E-46 | 1.72E-44 | Up |
| PHYHIPL | 8.30915925 | -1.541385864 | 0.252274 | -6.10997 | 9.97E-10 | 7.49E-09 | Down |
| PI16 | 464.1886969 | -1.41210008 | 0.221706 | -6.36924 | 1.90E-10 | 1.54E-09 | Down |
| PI4K2A | 856.2090548 | 1.092551692 | 0.075816 | 14.4106 | 4.44E-47 | 4.87E-45 | Up |
| PID1 | 262.9643127 | -1.657741124 | 0.155467 | -10.663 | 1.52E-26 | 4.53E-25 | Down |
| PIGQP1 | 0.955854198 | -1.651971598 | 0.409223 | -4.03685 | 5.42E-05 | 0.00021 | Down |
| PIH1D3 | 2.137690011 | -2.771971801 | 0.353614 | -7.83898 | 4.54E-15 | 5.75E-14 | Down |
| PIK3C2G | 13.15380398 | -2.147341597 | 0.376383 | -5.7052 | 1.16E-08 | 7.72E-08 | Down |
| PIN1P1 | 1.65520263 | -2.490994974 | 0.477668 | -5.2149 | 1.84E-07 | 1.05E-06 | Down |
| PIP | 1.267046594 | -1.644422492 | 0.34384 | -4.78252 | 1.73E-06 | 8.61E-06 | Down |
| PIRT | 8.15328719 | -1.508658038 | 0.305163 | -4.94378 | 7.66E-07 | 4.01E-06 | Down |
| PITX1 | 4.35025805 | -1.420449817 | 0.362332 | -3.9203 | 8.84E-05 | 0.000329 | Down |
| PITX3 | 3.564893782 | 1.193292628 | 0.412731 | 2.89121 | 0.003838 | 0.009805 | Up |
| PIWIL1 | 50.78770209 | 1.877352257 | 0.269921 | 6.955181 | 3.52E-12 | 3.37E-11 | Up |
| PIWIL2 | 32.80395409 | -1.297669318 | 0.125655 | -10.3273 | 5.30E-25 | 1.42E-23 | Down |
| PKD2L2 | 8.621821176 | -1.091950342 | 0.266035 | -4.10453 | 4.05E-05 | 0.000161 | Down |
| PKDCC | 184.8282521 | -1.054915056 | 0.130272 | -8.0978 | 5.60E-16 | 7.55E-15 | Down |
| PKDREJ | 33.96349638 | -1.074590838 | 0.145122 | -7.40472 | 1.31E-13 | 1.45E-12 | Down |
| PKHD1L1 | 11035.97219 | -2.68953678 | 0.259359 | -10.3699 | 3.40E-25 | 9.24E-24 | Down |
| PKIB | 75.15625562 | 1.504081286 | 0.151945 | 9.898825 | 4.21E-23 | 9.82E-22 | Up |
| PKLR | 2.05932042 | -1.451556938 | 0.513278 | -2.82801 | 0.004684 | 0.011706 | Down |
| PKMP1 | 2.298088523 | -1.574163842 | 0.475897 | -3.30778 | 0.00094 | 0.002812 | Down |
| PKMP5 | 4.231718667 | -1.802191619 | 0.392261 | -4.59437 | 4.34E-06 | 2.03E-05 | Down |
| PKNOX2 | 331.399061 | -1.554577584 | 0.154094 | -10.0885 | 6.21E-24 | 1.54E-22 | Down |
| PKP2 | 166.3407785 | 1.108923819 | 0.222696 | 4.979546 | 6.37E-07 | 3.38E-06 | Up |
| PLA2G10 | 3.550510825 | 1.161680664 | 0.365734 | 3.176298 | 0.001492 | 0.004251 | Up |
| PLA2G12B | 19.97953802 | 3.594372304 | 0.315088 | 11.40752 | 3.84E-30 | 1.48E-28 | Up |
| PLA2G2A | 79.41483033 | 1.599746262 | 0.31387 | 5.096849 | 3.45E-07 | 1.90E-06 | Up |
| PLA2G2E | 1.435878825 | 2.952441133 | 0.419591 | 7.036467 | 1.97E-12 | 1.94E-11 | Up |
| PLA2G4D | 2.884514397 | -1.646847541 | 0.491359 | -3.35162 | 0.000803 | 0.002435 | Down |
| PLA2G4E | 3.921933679 | -1.552464767 | 0.383638 | -4.04669 | 5.19E-05 | 0.000202 | Down |
| PLA2G4E-AS1 | 2.100175925 | -1.74648402 | 0.469425 | -3.72047 | 0.000199 | 0.000692 | Down |
| PLA2G4F | 121.6591387 | -1.578288936 | 0.175485 | -8.99389 | 2.39E-19 | 4.24E-18 | Down |
| PLA2G7 | 441.7080076 | -1.021226793 | 0.130345 | -7.83481 | 4.70E-15 | 5.94E-14 | Down |
| PLA2R1 | 4603.668875 | -2.127972472 | 0.159917 | -13.3067 | 2.12E-40 | 1.51E-38 | Down |
| PLAC1 | 1.402755056 | -1.994446296 | 0.543858 | -3.66722 | 0.000245 | 0.000836 | Down |
| PLAC8L1 | 1.211083931 | -1.144665668 | 0.331921 | -3.44861 | 0.000563 | 0.00177 | Down |
| PLAC9 | 265.6023312 | -1.124302684 | 0.123035 | -9.13809 | 6.36E-20 | 1.18E-18 | Down |
| PLAG1 | 709.1976524 | 1.980299965 | 0.122887 | 16.11481 | 2.01E-58 | 3.73E-56 | Up |
| PLAU | 2286.386253 | 3.276499308 | 0.144366 | 22.69585 | 4.92E-114 | 6.97E-111 | Up |
| PLAUR | 304.5297595 | 1.872896935 | 0.166917 | 11.22055 | 3.23E-29 | 1.18E-27 | Up |
| PLCD3 | 1227.68358 | 2.483331313 | 0.127089 | 19.54016 | 5.00E-85 | 2.63E-82 | Up |
| PLCD4 | 85.17669669 | -1.017075152 | 0.11181 | -9.09648 | 9.33E-20 | 1.71E-18 | Down |
| PLCH1 | 539.6752901 | -1.870230116 | 0.146903 | -12.7311 | 3.97E-37 | 2.34E-35 | Down |
| PLCH1-AS1 | 5.178648119 | -2.041809418 | 0.261034 | -7.82202 | 5.20E-15 | 6.56E-14 | Down |
| PLD5 | 20.58056504 | -2.219774524 | 0.269921 | -8.22378 | 1.97E-16 | 2.76E-15 | Down |
| PLEKHA4 | 839.3522828 | 1.374463018 | 0.085741 | 16.03047 | 7.83E-58 | 1.41E-55 | Up |
| PLEKHD1 | 118.067596 | -2.474746316 | 0.191354 | -12.9328 | 2.94E-38 | 1.83E-36 | Down |
| PLEKHG4 | 345.7825911 | 1.085247464 | 0.112927 | 9.610203 | 7.24E-22 | 1.54E-20 | Up |
| PLEKHG4B | 348.150064 | -1.781828181 | 0.151669 | -11.7481 | 7.22E-32 | 3.14E-30 | Down |
| PLEKHN1 | 46.11359819 | 2.989110947 | 0.171836 | 17.39517 | 8.98E-68 | 2.36E-65 | Up |
| PLG | 7.145530789 | -1.125820335 | 0.332521 | -3.38571 | 0.00071 | 0.002181 | Down |
| PLIN1 | 181.628218 | -2.33556072 | 0.285318 | -8.18582 | 2.70E-16 | 3.74E-15 | Down |
| PLIN4 | 469.0611103 | -2.04932107 | 0.251292 | -8.15514 | 3.49E-16 | 4.78E-15 | Down |
| PLK5 | 1.747425436 | -1.164137339 | 0.500386 | -2.32648 | 0.019993 | 0.041326 | Down |
| PLP2 | 751.3537938 | 1.171004199 | 0.07401 | 15.82231 | 2.18E-56 | 3.75E-54 | Up |
| PLSCR4 | 1238.548363 | -1.021377896 | 0.083012 | -12.304 | 8.62E-35 | 4.36E-33 | Down |
| PLXNA4 | 461.7323679 | 1.128816739 | 0.153732 | 7.342765 | 2.09E-13 | 2.25E-12 | Up |
| PLXNC1 | 3180.372235 | 2.28879609 | 0.135663 | 16.87115 | 7.33E-64 | 1.67E-61 | Up |
| PMAIP1 | 79.62277173 | 1.600502629 | 0.202776 | 7.892974 | 2.95E-15 | 3.79E-14 | Up |
| PMCHL2 | 3.430481333 | -3.430343549 | 0.593506 | -5.7798 | 7.48E-09 | 5.07E-08 | Down |
| PMEPA1 | 1053.306442 | 1.245557711 | 0.089849 | 13.86279 | 1.06E-43 | 9.38E-42 | Up |
| PMP2 | 16.30635099 | -1.820558338 | 0.314745 | -5.78424 | 7.28E-09 | 4.95E-08 | Down |
| PNLDC1 | 2.376091636 | -1.55717207 | 0.394477 | -3.94744 | 7.90E-05 | 0.000296 | Down |
| PNLIPRP3 | 1.628053835 | -3.108671857 | 0.707816 | -4.39192 | 1.12E-05 | 4.92E-05 | Down |
| PNMT | 14.15248958 | 1.167815811 | 0.210281 | 5.553608 | 2.80E-08 | 1.77E-07 | Up |
| PNP | 1223.082413 | 2.256898427 | 0.166261 | 13.57444 | 5.68E-42 | 4.46E-40 | Up |
| PNPLA1 | 3.15890886 | -1.771671871 | 0.392728 | -4.51119 | 6.45E-06 | 2.94E-05 | Down |
| PNPLA5 | 10.29528295 | 2.781090024 | 0.467441 | 5.949605 | 2.69E-09 | 1.92E-08 | Up |
| PODN | 874.4475157 | -1.477347869 | 0.173385 | -8.5206 | 1.59E-17 | 2.44E-16 | Down |
| PODNL1 | 35.18409754 | 1.004189137 | 0.193656 | 5.185424 | 2.16E-07 | 1.22E-06 | Up |
| POM121B | 11.03909358 | -1.061510507 | 0.267449 | -3.96902 | 7.22E-05 | 0.000273 | Down |
| POM121L12 | 1.878998236 | -2.639906865 | 0.894818 | -2.95022 | 0.003176 | 0.008296 | Down |
| POM121L1P | 5.148287364 | -1.808402795 | 0.389671 | -4.64084 | 3.47E-06 | 1.65E-05 | Down |
| POM121L3P | 1.500733978 | -2.319481298 | 0.565857 | -4.09906 | 4.15E-05 | 0.000164 | Down |
| PON2 | 1083.030651 | 1.101748844 | 0.062724 | 17.56497 | 4.57E-69 | 1.28E-66 | Up |
| POPDC3 | 6.86028515 | -1.206722974 | 0.311351 | -3.87576 | 0.000106 | 0.000389 | Down |
| POSTN | 2817.012368 | 3.109363898 | 0.22781 | 13.64896 | 2.05E-42 | 1.67E-40 | Up |
| POTEF | 2.914765477 | -1.925979313 | 0.497266 | -3.87314 | 0.000107 | 0.000393 | Down |
| POTEJ | 2.745497328 | -1.443357558 | 0.53739 | -2.68587 | 0.007234 | 0.017142 | Down |
| POU1F1 | 1.999921119 | -1.112636461 | 0.327498 | -3.39738 | 0.00068 | 0.0021 | Down |
| POU2F3 | 133.1423611 | 1.73626978 | 0.119562 | 14.52191 | 8.80E-48 | 9.82E-46 | Up |
| POU4F3 | 1.977488289 | -1.19506778 | 0.374306 | -3.19276 | 0.001409 | 0.004039 | Down |
| POU5F1P6 | 1.716315619 | -1.36526753 | 0.438557 | -3.11309 | 0.001851 | 0.005153 | Down |
| PPAP2C | 450.1888678 | 1.223713079 | 0.168172 | 7.276537 | 3.42E-13 | 3.61E-12 | Up |
| PPAPDC1A | 12.44927328 | 2.784277116 | 0.280586 | 9.92307 | 3.30E-23 | 7.77E-22 | Up |
| PPARG | 303.8873229 | -1.468242052 | 0.11913 | -12.3247 | 6.67E-35 | 3.43E-33 | Down |
| PPARGC1A | 1126.67695 | -1.901125006 | 0.152875 | -12.4358 | 1.67E-35 | 8.95E-34 | Down |
| PPATP1 | 1.148623927 | -1.828193873 | 0.760881 | -2.40273 | 0.016273 | 0.034618 | Down |
| PPEF1 | 21.45688282 | 1.095908101 | 0.197682 | 5.543781 | 2.96E-08 | 1.87E-07 | Up |
| PPEF2 | 3.216163003 | -1.839244283 | 0.497871 | -3.69422 | 0.000221 | 0.00076 | Down |
| PPIAL4B | 1.017464518 | -1.742261363 | 0.770018 | -2.26263 | 0.023659 | 0.047749 | Down |
| PPIAL4C | 5.921233329 | -1.109042487 | 0.361693 | -3.06625 | 0.002168 | 0.005921 | Down |
| PPIAL4E | 1.694487286 | -2.355753756 | 0.801733 | -2.93833 | 0.0033 | 0.008586 | Down |
| PPIAL4G | 11.40016945 | -1.384923511 | 0.33794 | -4.09814 | 4.16E-05 | 0.000165 | Down |
| PPIAP14 | 0.808043543 | -1.57026895 | 0.693995 | -2.26265 | 0.023657 | 0.047749 | Down |
| PPIAP26 | 14.77782221 | -1.40602491 | 0.238383 | -5.89818 | 3.68E-09 | 2.58E-08 | Down |
| PPL | 5891.625137 | 1.304652314 | 0.109153 | 11.95246 | 6.30E-33 | 2.89E-31 | Up |
| PPM1AP1 | 4.58978353 | 1.802699622 | 0.359888 | 5.009056 | 5.47E-07 | 2.92E-06 | Up |
| PPM1L | 1340.727928 | -1.216842596 | 0.085133 | -14.2934 | 2.41E-46 | 2.50E-44 | Down |
| PPP1R10P1 | 1.952394897 | -2.692611224 | 0.897475 | -3.00021 | 0.002698 | 0.007165 | Down |
| PPP1R1A | 45.25816289 | -1.275344731 | 0.239019 | -5.33575 | 9.51E-08 | 5.63E-07 | Down |
| PPP1R1B | 90.00856405 | 2.770091672 | 0.198357 | 13.96519 | 2.54E-44 | 2.32E-42 | Up |
| PPP1R1C | 36.7952386 | 1.661149221 | 0.232903 | 7.132367 | 9.87E-13 | 1.00E-11 | Up |
| PPP1R27 | 4.832808608 | -1.411119728 | 0.300841 | -4.69058 | 2.72E-06 | 1.32E-05 | Down |
| PPP1R2P10 | 0.87901859 | -1.808858341 | 0.758627 | -2.38439 | 0.017108 | 0.036154 | Down |
| PPP1R2P9 | 1.008004924 | -1.612415037 | 0.639976 | -2.51949 | 0.011752 | 0.026219 | Down |
| PPP1R3A | 5.012984466 | -2.56311327 | 0.615684 | -4.16303 | 3.14E-05 | 0.000127 | Down |
| PPP1R42 | 7.2229393 | -1.627515735 | 0.183886 | -8.85069 | 8.70E-19 | 1.48E-17 | Down |
| PPP3R2 | 3.462012314 | -1.784250095 | 0.511488 | -3.48835 | 0.000486 | 0.001551 | Down |
| PPY | 4.479815516 | -2.786683401 | 0.471465 | -5.91069 | 3.41E-09 | 2.40E-08 | Down |
| PQLC2L | 62.37176225 | -1.984439662 | 0.145429 | -13.6455 | 2.15E-42 | 1.74E-40 | Down |
| PRAME | 4.543717153 | -1.00916099 | 0.432019 | -2.33592 | 0.019496 | 0.04044 | Down |
| PRAMEF11 | 1.95294167 | -1.426343848 | 0.57374 | -2.48604 | 0.012917 | 0.028474 | Down |
| PRAMEF14 | 2.404709895 | -2.392397074 | 0.820652 | -2.91524 | 0.003554 | 0.009166 | Down |
| PRAMEF4 | 2.464535161 | -2.17378856 | 0.677929 | -3.20651 | 0.001344 | 0.003869 | Down |
| PRAMEF8 | 2.511911782 | -2.098797524 | 0.754531 | -2.78159 | 0.005409 | 0.013286 | Down |
| PRAMENP | 4.406443884 | -1.432117677 | 0.518655 | -2.76121 | 0.005759 | 0.014032 | Down |
| PRB4 | 2.164416774 | -1.143567347 | 0.469461 | -2.43592 | 0.014854 | 0.032058 | Down |
| PRDM1 | 1491.851123 | 1.484859907 | 0.134579 | 11.03338 | 2.64E-28 | 8.99E-27 | Up |
| PRDM14 | 1.176065147 | -2.667087971 | 0.909685 | -2.93188 | 0.003369 | 0.008742 | Down |
| PRDM7 | 3.881547585 | -1.499115423 | 0.539052 | -2.78102 | 0.005419 | 0.013304 | Down |
| PREX2 | 1240.054477 | -1.069442297 | 0.092257 | -11.592 | 4.53E-31 | 1.87E-29 | Down |
| PRG4 | 40.58669946 | -1.611678368 | 0.287951 | -5.59706 | 2.18E-08 | 1.40E-07 | Down |
| PRICKLE1 | 1544.963427 | 1.51405052 | 0.078807 | 19.21209 | 2.93E-82 | 1.35E-79 | Up |
| PRICKLE2-AS1 | 35.79985915 | -1.297922233 | 0.221336 | -5.86404 | 4.52E-09 | 3.14E-08 | Down |
| PRICKLE2-AS2 | 0.620929048 | -1.003388064 | 0.446207 | -2.2487 | 0.024531 | 0.049292 | Down |
| PRICKLE2-AS3 | 11.11034765 | -1.450381806 | 0.242797 | -5.97364 | 2.32E-09 | 1.67E-08 | Down |
| PRIMA1 | 36.99492966 | -1.837330032 | 0.174118 | -10.5522 | 4.96E-26 | 1.43E-24 | Down |
| PRKACG | 2.4389963 | -1.375793283 | 0.468165 | -2.93869 | 0.003296 | 0.008578 | Down |
| PRKAG3 | 2.989659608 | -1.431944697 | 0.395797 | -3.61788 | 0.000297 | 0.000995 | Down |
| PRKCA-AS1 | 10.20820927 | -1.132313844 | 0.273245 | -4.14395 | 3.41E-05 | 0.000137 | Down |
| PRKCG | 2.19893768 | -1.274520762 | 0.411455 | -3.09759 | 0.001951 | 0.005396 | Down |
| PRKG1-AS1 | 5.167420812 | -1.514443893 | 0.281458 | -5.3807 | 7.42E-08 | 4.45E-07 | Down |
| PRKG2 | 40.70245527 | -1.662066734 | 0.242808 | -6.84518 | 7.64E-12 | 7.08E-11 | Down |
| PRKRIRP1 | 2.536539771 | -1.363830763 | 0.400271 | -3.40727 | 0.000656 | 0.002032 | Down |
| PRKRIRP2 | 3.183323698 | -2.44386885 | 0.469518 | -5.20506 | 1.94E-07 | 1.10E-06 | Down |
| PRKRIRP5 | 3.476603478 | -2.85069259 | 0.510108 | -5.58841 | 2.29E-08 | 1.47E-07 | Down |
| PRKRIRP6 | 1.394502478 | -3.011646118 | 1.042531 | -2.88878 | 0.003867 | 0.009875 | Down |
| PRKRIRP8 | 1.427030572 | -1.371120917 | 0.596328 | -2.29927 | 0.02149 | 0.043985 | Down |
| PRKX-AS1 | 1.992576038 | -1.500330772 | 0.347359 | -4.31926 | 1.57E-05 | 6.70E-05 | Down |
| PRL | 1.049666486 | -1.973420323 | 0.726487 | -2.71639 | 0.0066 | 0.015835 | Down |
| PRMT8 | 25.67669876 | 1.976481097 | 0.415843 | 4.752954 | 2.00E-06 | 9.88E-06 | Up |
| PRODH2 | 2.028473314 | -2.287547295 | 0.794183 | -2.88038 | 0.003972 | 0.010111 | Down |
| PROK1 | 2.100422291 | -1.195749614 | 0.430368 | -2.77843 | 0.005462 | 0.013399 | Down |
| PROK2 | 4.812380779 | -1.771081696 | 0.288268 | -6.14387 | 8.05E-10 | 6.11E-09 | Down |
| PROKR2 | 1.792753835 | -1.154872579 | 0.432526 | -2.67006 | 0.007584 | 0.01787 | Down |
| PROM1 | 273.0828215 | -2.02800387 | 0.229505 | -8.83643 | 9.88E-19 | 1.67E-17 | Down |
| PROP1 | 1.680861753 | -2.173165673 | 0.745328 | -2.91572 | 0.003549 | 0.009156 | Down |
| PROS1 | 4907.125178 | 3.046916149 | 0.122026 | 24.96947 | 1.31E-137 | 5.98E-134 | Up |
| PROSER2-AS1 | 38.01764857 | -1.253417272 | 0.154708 | -8.10182 | 5.41E-16 | 7.32E-15 | Down |
| PROX1 | 1147.415319 | -1.996875706 | 0.141302 | -14.1319 | 2.41E-45 | 2.38E-43 | Down |
| PROX1-AS1 | 68.15666681 | -2.109989342 | 0.174801 | -12.0708 | 1.51E-33 | 7.15E-32 | Down |
| PROZ | 4.016950383 | -1.060782849 | 0.282636 | -3.75317 | 0.000175 | 0.000615 | Down |
| PRPF38AP1 | 1.614167666 | -2.932046423 | 0.981701 | -2.9867 | 0.00282 | 0.00746 | Down |
| PRPS1L1 | 1.518055171 | -1.896885012 | 0.648813 | -2.92362 | 0.00346 | 0.00895 | Down |
| PRPS1P1 | 0.907804918 | -2.321260572 | 0.645272 | -3.59734 | 0.000321 | 0.00107 | Down |
| PRR13P3 | 0.916417766 | -1.358827385 | 0.533488 | -2.54706 | 0.010863 | 0.024448 | Down |
| PRR15 | 997.695147 | 4.730542802 | 0.250249 | 18.90331 | 1.07E-79 | 4.58E-77 | Up |
| PRR15L | 1054.112598 | -1.285755245 | 0.117156 | -10.9747 | 5.06E-28 | 1.69E-26 | Down |
| PRR18 | 6.446177702 | -1.444580177 | 0.286013 | -5.05074 | 4.40E-07 | 2.38E-06 | Down |
| PRR23B | 1.935136452 | -2.027161188 | 0.683908 | -2.96408 | 0.003036 | 0.007971 | Down |
| PRR23D1 | 1.444646825 | -1.464044841 | 0.545421 | -2.68425 | 0.007269 | 0.017216 | Down |
| PRR23D2 | 1.444646825 | -1.464044841 | 0.545421 | -2.68425 | 0.007269 | 0.017216 | Down |
| PRR25 | 1.828869797 | -1.414976519 | 0.430905 | -3.28373 | 0.001024 | 0.003036 | Down |
| PRR26 | 81.91196643 | -1.166888716 | 0.199859 | -5.83857 | 5.27E-09 | 3.64E-08 | Down |
| PRR36 | 58.57115664 | 1.112704852 | 0.224024 | 4.966902 | 6.80E-07 | 3.59E-06 | Up |
| PRR9 | 2.659224256 | -1.783841543 | 0.422794 | -4.21917 | 2.45E-05 | 0.000101 | Down |
| PRRG3 | 82.70917142 | -1.475132178 | 0.191464 | -7.70447 | 1.31E-14 | 1.59E-13 | Down |
| PRRT1 | 30.58662507 | -1.06257379 | 0.126808 | -8.37938 | 5.32E-17 | 7.83E-16 | Down |
| PRRT4 | 7.164579112 | -1.640767047 | 0.289398 | -5.66959 | 1.43E-08 | 9.39E-08 | Down |
| PRSS1 | 59.88671838 | 3.754533021 | 0.272339 | 13.78627 | 3.08E-43 | 2.62E-41 | Up |
| PRSS22 | 93.87833544 | 1.396761379 | 0.112543 | 12.41094 | 2.28E-35 | 1.21E-33 | Up |
| PRSS23 | 4894.08078 | 1.946117173 | 0.124575 | 15.62211 | 5.15E-55 | 8.32E-53 | Up |
| PRSS33 | 1.560134158 | -2.825512115 | 0.587164 | -4.81213 | 1.49E-06 | 7.50E-06 | Down |
| PRSS37 | 6.919292207 | -1.01972846 | 0.29947 | -3.40511 | 0.000661 | 0.002047 | Down |
| PRSS3P1 | 0.867719712 | 1.401705904 | 0.592927 | 2.364045 | 0.018077 | 0.037903 | Up |
| PRSS3P2 | 196.0997914 | 5.337986726 | 0.346525 | 15.40432 | 1.53E-53 | 2.22E-51 | Up |
| PRSS42 | 4.089361743 | -1.089095721 | 0.30407 | -3.58173 | 0.000341 | 0.001129 | Down |
| PRSS57 | 1.33225538 | 1.606679252 | 0.389185 | 4.128318 | 3.65E-05 | 0.000146 | Up |
| PRTG | 1694.974983 | -1.511946546 | 0.116367 | -12.9929 | 1.34E-38 | 8.51E-37 | Down |
| PRTN3 | 4.311080792 | 1.296161143 | 0.349896 | 3.704421 | 0.000212 | 0.000733 | Up |
| PSAT1P2 | 0.978169907 | -1.745289741 | 0.464707 | -3.75568 | 0.000173 | 0.000609 | Down |
| PSAT1P3 | 1.428959303 | -1.345625822 | 0.524439 | -2.56584 | 0.010293 | 0.023319 | Down |
| PSCA | 3.169279238 | -1.015706702 | 0.370546 | -2.74111 | 0.006123 | 0.014808 | Down |
| PSD3 | 3734.071252 | 2.125437679 | 0.089333 | 23.79229 | 4.01E-125 | 9.15E-122 | Up |
| PSG1 | 19.82232876 | 1.444039831 | 0.371852 | 3.883368 | 0.000103 | 0.000378 | Up |
| PSG2 | 2.288681395 | -1.650474439 | 0.590773 | -2.79375 | 0.00521 | 0.012856 | Down |
| PSG8 | 39.15237739 | 2.395336812 | 0.41376 | 5.789187 | 7.07E-09 | 4.81E-08 | Up |
| PSKH2 | 1.049052144 | -1.67227256 | 0.706585 | -2.3667 | 0.017948 | 0.037679 | Down |
| PSMA2P1 | 3.981476522 | -1.272795981 | 0.252665 | -5.03749 | 4.72E-07 | 2.55E-06 | Down |
| PSMA8 | 3.303980778 | -1.845156043 | 0.411583 | -4.48308 | 7.36E-06 | 3.32E-05 | Down |
| PSMC1P2 | 1.636703969 | -2.628389085 | 0.763715 | -3.44158 | 0.000578 | 0.001813 | Down |
| PSMC1P3 | 1.894753054 | -2.998151356 | 1.109552 | -2.70213 | 0.00689 | 0.016435 | Down |
| PSMC1P7 | 1.422054689 | -1.881169305 | 0.740088 | -2.54182 | 0.011028 | 0.024758 | Down |
| PSMC1P8 | 1.224157589 | -3.004592603 | 1.25312 | -2.39769 | 0.016499 | 0.035057 | Down |
| PSMC1P9 | 2.188763331 | -2.717240932 | 0.675696 | -4.0214 | 5.79E-05 | 0.000223 | Down |
| PSMD10P3 | 0.888481272 | -1.533797187 | 0.5676 | -2.70225 | 0.006887 | 0.016432 | Down |
| PSMD12P | 1.468003972 | -2.718503938 | 0.936504 | -2.90282 | 0.003698 | 0.009495 | Down |
| PSMD4P1 | 0.70120806 | 1.279393387 | 0.500472 | 2.556373 | 0.010577 | 0.023886 | Up |
| PSORS1C3 | 1.140717626 | -1.075820223 | 0.422498 | -2.54633 | 0.010886 | 0.024493 | Down |
| PTCHD1 | 64.29383103 | -1.477067194 | 0.245667 | -6.01247 | 1.83E-09 | 1.33E-08 | Down |
| PTCHD3 | 13.63040622 | -1.125652784 | 0.290232 | -3.87846 | 0.000105 | 0.000385 | Down |
| PTCHD3P3 | 0.728786228 | -1.324448141 | 0.525731 | -2.51925 | 0.01176 | 0.026233 | Down |
| PTCHD4 | 403.0035313 | 2.896991153 | 0.185799 | 15.59207 | 8.24E-55 | 1.30E-52 | Up |
| PTF1A | 1.599610762 | -2.810848688 | 0.727154 | -3.86555 | 0.000111 | 0.000404 | Down |
| PTGDR2 | 10.72420773 | -1.342216619 | 0.246835 | -5.43771 | 5.40E-08 | 3.29E-07 | Down |
| PTGER1 | 6.62016671 | 1.119605913 | 0.284623 | 3.93364 | 8.37E-05 | 0.000313 | Up |
| PTGER4P2 | 0.792501307 | -1.157442709 | 0.3957 | -2.92505 | 0.003444 | 0.008918 | Down |
| PTGFR | 62.5248421 | -1.049410066 | 0.209291 | -5.01411 | 5.33E-07 | 2.85E-06 | Down |
| PTGS2 | 287.8544819 | 1.388739679 | 0.189413 | 7.331791 | 2.27E-13 | 2.44E-12 | Up |
| PTMAP3 | 1.408505106 | -1.319740031 | 0.499136 | -2.64405 | 0.008192 | 0.019128 | Down |
| PTMAP8 | 1.525021663 | -1.501565511 | 0.566843 | -2.649 | 0.008073 | 0.018885 | Down |
| PTP4A1P2 | 0.584110798 | -1.362683072 | 0.522788 | -2.60657 | 0.009145 | 0.02107 | Down |
| PTP4A3 | 577.33244 | 1.661285051 | 0.136662 | 12.15614 | 5.32E-34 | 2.57E-32 | Up |
| PTPLA | 135.2730414 | 1.242484306 | 0.139679 | 8.89531 | 5.83E-19 | 1.00E-17 | Up |
| PTPN20A | 62.34841378 | -1.615489836 | 0.159411 | -10.1341 | 3.90E-24 | 9.82E-23 | Down |
| PTPN20CP | 3.473951121 | -1.328671947 | 0.276314 | -4.80855 | 1.52E-06 | 7.62E-06 | Down |
| PTPRE | 1253.837959 | 1.187606238 | 0.082528 | 14.39035 | 5.95E-47 | 6.46E-45 | Up |
| PTPRF | 6691.487742 | 1.073150444 | 0.076584 | 14.01272 | 1.30E-44 | 1.22E-42 | Up |
| PTPRO | 115.6051178 | 1.053600105 | 0.198918 | 5.296668 | 1.18E-07 | 6.90E-07 | Up |
| PTPRQ | 204.9386785 | -2.116036474 | 0.181728 | -11.644 | 2.46E-31 | 1.04E-29 | Down |
| PTPRVP | 10.6376229 | -1.050741016 | 0.249832 | -4.20579 | 2.60E-05 | 0.000107 | Down |
| PTX3 | 50.6539855 | -1.637780128 | 0.151605 | -10.803 | 3.33E-27 | 1.05E-25 | Down |
| PTX4 | 2.059442348 | -1.366500529 | 0.389408 | -3.50918 | 0.000449 | 0.001446 | Down |
| PURG | 16.39907609 | -1.39164972 | 0.177951 | -7.82042 | 5.26E-15 | 6.64E-14 | Down |
| PVALB | 6.239889896 | -1.204993305 | 0.352636 | -3.4171 | 0.000633 | 0.001966 | Down |
| PVRL4 | 333.6572565 | 3.981234133 | 0.198496 | 20.05705 | 1.75E-89 | 1.14E-86 | Up |
| PWWP2AP1 | 5.742266489 | -1.380139219 | 0.31322 | -4.40629 | 1.05E-05 | 4.63E-05 | Down |
| PYDC1 | 1.863890127 | 1.72832707 | 0.393403 | 4.393275 | 1.12E-05 | 4.89E-05 | Up |
| PYGM | 122.4391325 | -1.532647203 | 0.123559 | -12.4041 | 2.48E-35 | 1.31E-33 | Down |
| PYY | 2.701269095 | -2.830350447 | 0.451507 | -6.26867 | 3.64E-10 | 2.87E-09 | Down |
| QPCT | 944.164582 | 3.615771888 | 0.156539 | 23.09826 | 4.82E-118 | 7.91E-115 | Up |
| QRSL1P1 | 1.423494633 | -1.678191192 | 0.696254 | -2.41031 | 0.015939 | 0.034011 | Down |
| QSOX1 | 1868.570615 | 1.265581563 | 0.075852 | 16.68487 | 1.69E-62 | 3.67E-60 | Up |
| RAB27A | 1427.744586 | 1.726735418 | 0.08438 | 20.4638 | 4.53E-93 | 3.15E-90 | Up |
| RAB27B | 142.9271826 | 2.336315631 | 0.214332 | 10.90048 | 1.15E-27 | 3.73E-26 | Up |
| RAB34 | 2000.818389 | 1.070067261 | 0.066167 | 16.1722 | 7.92E-59 | 1.52E-56 | Up |
| RAB38 | 179.7197349 | 1.021736607 | 0.089767 | 11.38207 | 5.14E-30 | 1.96E-28 | Up |
| RAB3B | 18.21029269 | 1.022596309 | 0.260494 | 3.925609 | 8.65E-05 | 0.000322 | Up |
| RAB6C | 7.592666096 | -1.512754275 | 0.302273 | -5.0046 | 5.60E-07 | 2.99E-06 | Down |
| RAB6C-AS1 | 9.787734899 | -2.034701751 | 0.309639 | -6.57121 | 4.99E-11 | 4.27E-10 | Down |
| RABGAP1L-IT1 | 3.962867402 | -1.065226876 | 0.302196 | -3.52495 | 0.000424 | 0.001371 | Down |
| RAD17P1 | 5.337555043 | -2.936673289 | 0.381138 | -7.70502 | 1.31E-14 | 1.59E-13 | Down |
| RAD23B | 4575.080494 | 1.35727083 | 0.073957 | 18.35217 | 3.17E-75 | 1.17E-72 | Up |
| RAD51AP2 | 4.29439377 | -1.432854577 | 0.303767 | -4.71695 | 2.39E-06 | 1.17E-05 | Down |
| RADIL | 93.28974233 | -2.040871247 | 0.151868 | -13.4384 | 3.60E-41 | 2.74E-39 | Down |
| RAET1G | 22.77669193 | 1.525537472 | 0.16015 | 9.525657 | 1.64E-21 | 3.42E-20 | Up |
| RAET1L | 2.878799813 | 2.037492889 | 0.326922 | 6.232346 | 4.60E-10 | 3.58E-09 | Up |
| RAF1P1 | 4.34487217 | -1.807134388 | 0.339224 | -5.32726 | 9.97E-08 | 5.88E-07 | Down |
| RAG1 | 153.3038691 | -1.437559209 | 0.115247 | -12.4737 | 1.04E-35 | 5.63E-34 | Down |
| RAG2 | 64.00111348 | -2.168969215 | 0.220066 | -9.85601 | 6.46E-23 | 1.49E-21 | Down |
| RAMP1 | 124.1189952 | 2.060176948 | 0.173809 | 11.85309 | 2.07E-32 | 9.30E-31 | Up |
| RAMP2-AS1 | 54.06338195 | -1.237318255 | 0.111462 | -11.1008 | 1.24E-28 | 4.35E-27 | Down |
| RANBP3L | 131.899112 | -1.418430657 | 0.144682 | -9.80381 | 1.08E-22 | 2.46E-21 | Down |
| RAP1AP | 10.4473938 | 2.25866529 | 0.230866 | 9.783442 | 1.33E-22 | 2.99E-21 | Up |
| RAP1GAP | 2688.018394 | -1.726813294 | 0.12316 | -14.0209 | 1.16E-44 | 1.09E-42 | Down |
| RAP2CP1 | 1.07472241 | -2.800907536 | 0.809624 | -3.45952 | 0.000541 | 0.001709 | Down |
| RAPGEF4-AS1 | 4.467452496 | -1.183870755 | 0.430453 | -2.75029 | 0.005954 | 0.014456 | Down |
| RAPGEF5 | 1299.422496 | 1.04460905 | 0.096092 | 10.87096 | 1.59E-27 | 5.12E-26 | Up |
| RASA1 | 2187.606994 | 1.137548932 | 0.061147 | 18.6035 | 3.01E-77 | 1.19E-74 | Up |
| RASD2 | 179.5632308 | 2.617035685 | 0.149979 | 17.44939 | 3.48E-68 | 9.27E-66 | Up |
| RASGRF1 | 134.5040889 | 2.807771092 | 0.243955 | 11.50938 | 1.18E-30 | 4.74E-29 | Up |
| RASGRF2-AS1 | 3.042682553 | -1.191703197 | 0.235619 | -5.05775 | 4.24E-07 | 2.31E-06 | Down |
| RASSF10 | 20.39245385 | -1.693190118 | 0.201586 | -8.39935 | 4.49E-17 | 6.65E-16 | Down |
| RASSF6 | 251.631796 | -1.872608923 | 0.180752 | -10.3601 | 3.77E-25 | 1.02E-23 | Down |
| RASSF9 | 442.8548115 | -1.595084508 | 0.12128 | -13.1521 | 1.66E-39 | 1.13E-37 | Down |
| RAX2 | 3.049628896 | -1.456994484 | 0.334798 | -4.35186 | 1.35E-05 | 5.83E-05 | Down |
| RBBP4P3 | 1.492050934 | -2.473763485 | 0.669197 | -3.69662 | 0.000218 | 0.000753 | Down |
| RBBP8 | 1034.625049 | 1.094177518 | 0.062256 | 17.57555 | 3.79E-69 | 1.07E-66 | Up |
| RBBP8NL | 99.47664721 | -1.057166067 | 0.107462 | -9.83762 | 7.75E-23 | 1.77E-21 | Down |
| RBM17P1 | 2.222092367 | -2.431272416 | 0.401242 | -6.05936 | 1.37E-09 | 1.01E-08 | Down |
| RBM17P3 | 1.696233027 | -1.272182387 | 0.461169 | -2.7586 | 0.005805 | 0.014129 | Down |
| RBM20 | 82.47303642 | -1.462322887 | 0.162675 | -8.98922 | 2.49E-19 | 4.41E-18 | Down |
| RBM22P5 | 2.015863418 | -1.467242939 | 0.517427 | -2.83565 | 0.004573 | 0.011459 | Down |
| RBM24 | 24.78002867 | -1.25704234 | 0.191264 | -6.57227 | 4.96E-11 | 4.24E-10 | Down |
| RBM48P1 | 1.496068233 | -3.214343058 | 1.367059 | -2.35128 | 0.018709 | 0.039015 | Down |
| RBMX2P3 | 4.080110296 | -1.316799481 | 0.262341 | -5.01943 | 5.18E-07 | 2.78E-06 | Down |
| RBMX2P5 | 1.422938943 | -2.528120554 | 0.904721 | -2.79437 | 0.0052 | 0.012839 | Down |
| RBMXL2 | 4.673633425 | -1.694044506 | 0.370393 | -4.57364 | 4.79E-06 | 2.23E-05 | Down |
| RBMXL3 | 5.393466153 | -1.384764224 | 0.321658 | -4.30509 | 1.67E-05 | 7.10E-05 | Down |
| RBP2 | 1.982312491 | -1.037557295 | 0.347457 | -2.98615 | 0.002825 | 0.007472 | Down |
| RBP3 | 2.696510614 | -1.464980323 | 0.511134 | -2.86614 | 0.004155 | 0.010519 | Down |
| RBP4 | 33.0202612 | -1.03857297 | 0.240154 | -4.32461 | 1.53E-05 | 6.55E-05 | Down |
| RBPJP6 | 1.654885472 | -2.061411977 | 0.593428 | -3.47374 | 0.000513 | 0.001628 | Down |
| RBPMS2P1 | 2.794484604 | -1.440020538 | 0.33098 | -4.35077 | 1.36E-05 | 5.86E-05 | Down |
| RCC2P7 | 0.92841274 | -1.447087773 | 0.63439 | -2.28107 | 0.022544 | 0.04582 | Down |
| RCC2P8 | 1.001581017 | -1.877061647 | 0.833618 | -2.2517 | 0.024341 | 0.04895 | Down |
| RCVRN | 2.908332251 | -1.706169887 | 0.410401 | -4.15732 | 3.22E-05 | 0.00013 | Down |
| RDH12 | 20.76398577 | -1.155173905 | 0.220591 | -5.23672 | 1.63E-07 | 9.39E-07 | Down |
| RDH5 | 149.8919113 | 1.794492383 | 0.129545 | 13.85229 | 1.23E-43 | 1.08E-41 | Up |
| REEP1 | 133.3645379 | -1.165625766 | 0.123399 | -9.44596 | 3.52E-21 | 7.18E-20 | Down |
| REEP2 | 53.12347617 | 1.116003342 | 0.121294 | 9.200818 | 3.55E-20 | 6.71E-19 | Up |
| REG4 | 2.055499415 | -1.598287378 | 0.667643 | -2.39393 | 0.016669 | 0.03536 | Down |
| RELN | 790.0419586 | -2.279856378 | 0.196786 | -11.5855 | 4.88E-31 | 2.01E-29 | Down |
| REN | 11.94293119 | 4.232572809 | 0.260844 | 16.22644 | 3.28E-59 | 6.41E-57 | Up |
| RERGL | 83.56909282 | -1.583567408 | 0.24856 | -6.37096 | 1.88E-10 | 1.52E-09 | Down |
| RETN | 8.832828856 | 2.725944904 | 0.443407 | 6.147726 | 7.86E-10 | 5.98E-09 | Up |
| REV3L-IT1 | 0.870489819 | -1.166953223 | 0.501479 | -2.32702 | 0.019964 | 0.041283 | Down |
| REXO1L1P | 42.44321939 | -1.708670724 | 0.463961 | -3.68279 | 0.000231 | 0.000792 | Down |
| RFPL2 | 3.437793182 | -1.100539527 | 0.336258 | -3.2729 | 0.001064 | 0.00314 | Down |
| RFPL3 | 1.568596559 | -2.406161341 | 0.833829 | -2.88568 | 0.003906 | 0.009962 | Down |
| RFPL4A | 2.967447954 | -1.136941336 | 0.420597 | -2.70316 | 0.006868 | 0.016396 | Down |
| RFPL4AL1 | 1.2659121 | -1.215228526 | 0.530904 | -2.28898 | 0.022081 | 0.045018 | Down |
| RFPL4AP1 | 0.748647064 | -1.663353296 | 0.741393 | -2.24355 | 0.024861 | 0.049867 | Down |
| RFWD2P1 | 6.922520516 | -1.529887094 | 0.232581 | -6.57788 | 4.77E-11 | 4.09E-10 | Down |
| RFX4 | 11.12471141 | -1.808305394 | 0.298779 | -6.05232 | 1.43E-09 | 1.06E-08 | Down |
| RFX6 | 1.589195623 | -2.088138853 | 0.723409 | -2.88653 | 0.003895 | 0.009938 | Down |
| RGS13 | 57.26969373 | -1.722559277 | 0.456316 | -3.77493 | 0.00016 | 0.000568 | Down |
| RGS16 | 1539.950925 | -1.150759735 | 0.12487 | -9.21569 | 3.09E-20 | 5.87E-19 | Down |
| RGS17 | 138.8238089 | -1.355654758 | 0.135338 | -10.0168 | 1.29E-23 | 3.13E-22 | Down |
| RGS21 | 1.692036423 | -2.786714262 | 1.008936 | -2.76203 | 0.005744 | 0.014002 | Down |
| RGS4 | 202.1022823 | 1.930504678 | 0.199022 | 9.699975 | 3.02E-22 | 6.61E-21 | Up |
| RGS7 | 1.98872817 | -1.123148257 | 0.456369 | -2.46105 | 0.013853 | 0.030209 | Down |
| RGS8 | 469.7913938 | -2.991674447 | 0.247952 | -12.0655 | 1.61E-33 | 7.60E-32 | Down |
| RGS9BP | 18.67565241 | -1.526151927 | 0.182743 | -8.35137 | 6.75E-17 | 9.83E-16 | Down |
| RHAG | 1.05583655 | -1.785071408 | 0.36891 | -4.83878 | 1.31E-06 | 6.62E-06 | Down |
| RHBG | 2.254140963 | -2.220351412 | 0.601697 | -3.69015 | 0.000224 | 0.000771 | Down |
| RHOV | 33.19377352 | 1.67734475 | 0.202985 | 8.263379 | 1.42E-16 | 2.01E-15 | Up |
| RHOXF2B | 2.855345045 | -1.259233082 | 0.525875 | -2.39455 | 0.016641 | 0.035313 | Down |
| RILPL1 | 655.8709987 | -1.037490093 | 0.089066 | -11.6486 | 2.33E-31 | 9.84E-30 | Down |
| RIMKLA | 92.23252822 | -1.291636617 | 0.128038 | -10.0879 | 6.25E-24 | 1.55E-22 | Down |
| RIMS1 | 27.31450794 | -1.12345034 | 0.18017 | -6.23551 | 4.50E-10 | 3.51E-09 | Down |
| RIMS2 | 155.8753974 | 2.235638395 | 0.227347 | 9.833612 | 8.07E-23 | 1.84E-21 | Up |
| RIMS4 | 341.9104643 | -1.561424325 | 0.141479 | -11.0365 | 2.55E-28 | 8.70E-27 | Down |
| RIN1 | 100.7177028 | 1.399488627 | 0.104584 | 13.38147 | 7.76E-41 | 5.72E-39 | Up |
| RIPPLY1 | 2.576300912 | 1.11312739 | 0.369995 | 3.00849 | 0.002625 | 0.006999 | Up |
| RLBP1 | 1.633833336 | -1.462960365 | 0.487263 | -3.0024 | 0.002679 | 0.007122 | Down |
| RMI2 | 111.591676 | 1.207079284 | 0.120349 | 10.02978 | 1.13E-23 | 2.76E-22 | Up |
| RN7SKP150 | 0.978174102 | -1.103638227 | 0.434094 | -2.5424 | 0.01101 | 0.024723 | Down |
| RN7SKP195 | 1.035682451 | -2.240105042 | 0.389195 | -5.75574 | 8.63E-09 | 5.82E-08 | Down |
| RN7SKP198 | 1.447338674 | -1.206244931 | 0.368227 | -3.27582 | 0.001054 | 0.00311 | Down |
| RN7SKP245 | 4.961975977 | -1.491624211 | 0.283866 | -5.25468 | 1.48E-07 | 8.57E-07 | Down |
| RN7SKP264 | 0.596884867 | -1.017968996 | 0.446573 | -2.27951 | 0.022637 | 0.045989 | Down |
| RN7SKP268 | 0.757394413 | -1.126450824 | 0.444925 | -2.53178 | 0.011349 | 0.025402 | Down |
| RN7SKP285 | 1.668858583 | -1.119258626 | 0.338746 | -3.30412 | 0.000953 | 0.002843 | Down |
| RN7SKP296 | 1.227115439 | 1.626972804 | 0.381721 | 4.262204 | 2.02E-05 | 8.49E-05 | Up |
| RN7SKP53 | 0.537345865 | -1.993342244 | 0.457734 | -4.35481 | 1.33E-05 | 5.77E-05 | Down |
| RN7SKP99 | 0.423659449 | -1.559794167 | 0.606551 | -2.57158 | 0.010124 | 0.022991 | Down |
| RN7SL164P | 1.022884263 | -1.361107724 | 0.343034 | -3.96785 | 7.25E-05 | 0.000274 | Down |
| RN7SL173P | 0.936401717 | -1.082601618 | 0.407867 | -2.6543 | 0.007947 | 0.018623 | Down |
| RN7SL209P | 1.13160117 | -1.003121871 | 0.345556 | -2.90292 | 0.003697 | 0.009493 | Down |
| RN7SL251P | 0.541921399 | -1.490025749 | 0.616206 | -2.41806 | 0.015603 | 0.033395 | Down |
| RN7SL308P | 1.535247346 | -1.011097878 | 0.308464 | -3.27785 | 0.001046 | 0.003093 | Down |
| RN7SL42P | 0.545346343 | 1.560755183 | 0.454385 | 3.434873 | 0.000593 | 0.001854 | Up |
| RN7SL435P | 2.022399762 | 3.634624248 | 0.495953 | 7.328559 | 2.33E-13 | 2.49E-12 | Up |
| RN7SL455P | 0.949061005 | -1.039137936 | 0.448694 | -2.31591 | 0.020563 | 0.042363 | Down |
| RN7SL472P | 0.804971461 | -1.225967195 | 0.489475 | -2.50466 | 0.012257 | 0.027198 | Down |
| RN7SL531P | 1.407181245 | -1.143611656 | 0.389901 | -2.93308 | 0.003356 | 0.008713 | Down |
| RN7SL538P | 0.384990482 | -1.199355253 | 0.519942 | -2.30671 | 0.021071 | 0.043252 | Down |
| RN7SL604P | 1.058770084 | -1.59788729 | 0.428152 | -3.73205 | 0.00019 | 0.000664 | Down |
| RN7SL653P | 1.104367663 | -1.143508298 | 0.366381 | -3.12109 | 0.001802 | 0.005031 | Down |
| RN7SL724P | 0.924067636 | -1.035596956 | 0.343341 | -3.01623 | 0.002559 | 0.006844 | Down |
| RN7SL806P | 1.543232215 | -1.254388967 | 0.369217 | -3.39743 | 0.00068 | 0.0021 | Down |
| RN7SL832P | 10.42153115 | -1.284609902 | 0.179205 | -7.16839 | 7.59E-13 | 7.76E-12 | Down |
| RN7SL92P | 0.787162304 | -1.030266625 | 0.41217 | -2.49961 | 0.012433 | 0.027528 | Down |
| RNASE2 | 6.925007561 | 1.855783645 | 0.289842 | 6.402734 | 1.53E-10 | 1.25E-09 | Up |
| RNF103-CHMP3 | 3.946843517 | 1.037728442 | 0.369962 | 2.804957 | 0.005032 | 0.012458 | Up |
| RNF112 | 29.25921448 | -1.152498798 | 0.152434 | -7.56062 | 4.01E-14 | 4.63E-13 | Down |
| RNF150 | 839.1537493 | -1.658930105 | 0.103212 | -16.0731 | 3.94E-58 | 7.22E-56 | Down |
| RNF157 | 105.7385177 | -1.003932379 | 0.145432 | -6.9031 | 5.09E-12 | 4.81E-11 | Down |
| RNF165 | 157.6435739 | -1.027349291 | 0.12181 | -8.43406 | 3.34E-17 | 4.99E-16 | Down |
| RNF17 | 3.946357569 | -1.416398123 | 0.407478 | -3.47601 | 0.000509 | 0.001616 | Down |
| RNF183 | 24.1437792 | 2.121393881 | 0.291164 | 7.285907 | 3.20E-13 | 3.38E-12 | Up |
| RNF212B | 1.805123405 | -1.781921169 | 0.456568 | -3.90286 | 9.51E-05 | 0.000351 | Down |
| RNF222 | 12.1063636 | -1.156479661 | 0.239469 | -4.82935 | 1.37E-06 | 6.92E-06 | Down |
| RNF225 | 8.997914609 | 2.13576655 | 0.275147 | 7.762269 | 8.34E-15 | 1.03E-13 | Up |
| RNF24 | 1030.533438 | 1.243289769 | 0.06302 | 19.72849 | 1.23E-86 | 7.00E-84 | Up |
| RNF2P1 | 2.592119616 | -1.946832034 | 0.340721 | -5.71386 | 1.10E-08 | 7.36E-08 | Down |
| RNU1-120P | 2.517373536 | -1.156112319 | 0.23794 | -4.85885 | 1.18E-06 | 6.02E-06 | Down |
| RNU1-13P | 5.956947427 | -1.462490012 | 0.200185 | -7.3057 | 2.76E-13 | 2.94E-12 | Down |
| RNU2-5P | 2.166412394 | -1.233975034 | 0.328012 | -3.76198 | 0.000169 | 0.000595 | Down |
| RNU6-36P | 3.395318456 | -1.547497087 | 0.55325 | -2.79711 | 0.005156 | 0.012739 | Down |
| RNU6ATAC35P | 3.06272226 | -1.30269918 | 0.221279 | -5.88713 | 3.93E-09 | 2.75E-08 | Down |
| RNVU1-14 | 72.14716548 | -1.111787947 | 0.165597 | -6.71382 | 1.90E-11 | 1.68E-10 | Down |
| RNVU1-15 | 45.22580253 | -1.45710293 | 0.174694 | -8.34086 | 7.37E-17 | 1.07E-15 | Down |
| RNVU1-19 | 13.08365366 | -1.716829711 | 0.196512 | -8.7365 | 2.40E-18 | 3.92E-17 | Down |
| RNVU1-20 | 3.984076369 | -1.31638354 | 0.205258 | -6.41332 | 1.42E-10 | 1.17E-09 | Down |
| RNVU1-3 | 2.526817701 | -1.212395518 | 0.275664 | -4.39809 | 1.09E-05 | 4.79E-05 | Down |
| RNVU1-6 | 24.28458951 | -1.866427326 | 0.196883 | -9.47987 | 2.55E-21 | 5.25E-20 | Down |
| ROBO3 | 151.1324575 | -1.08377175 | 0.109151 | -9.9291 | 3.11E-23 | 7.32E-22 | Down |
| ROPN1B | 25.3349719 | -1.208103069 | 0.156929 | -7.69842 | 1.38E-14 | 1.67E-13 | Down |
| ROPN1L | 8.602163475 | -1.203831834 | 0.165349 | -7.28057 | 3.32E-13 | 3.51E-12 | Down |
| ROR1 | 149.2701696 | 1.132803315 | 0.120482 | 9.402299 | 5.34E-21 | 1.07E-19 | Up |
| ROR2 | 485.4741037 | -1.020626349 | 0.121779 | -8.38096 | 5.25E-17 | 7.73E-16 | Down |
| RORA-AS2 | 1.144459842 | -1.537226059 | 0.459289 | -3.34697 | 0.000817 | 0.002472 | Down |
| RORB-AS1 | 2.116357067 | -1.504094288 | 0.424072 | -3.54679 | 0.00039 | 0.001274 | Down |
| RP1-101G11.2 | 1.837781481 | -2.609738546 | 0.942234 | -2.76973 | 0.00561 | 0.013716 | Down |
| RP1-102G20.2 | 0.633756452 | -1.269519037 | 0.472807 | -2.68507 | 0.007252 | 0.01718 | Down |
| RP1-102K2.6 | 1.902041876 | -2.066583058 | 0.762083 | -2.71176 | 0.006693 | 0.016031 | Down |
| RP1-106C24.1 | 1.168014716 | -1.959712692 | 0.606617 | -3.23056 | 0.001235 | 0.003597 | Down |
| RP1-118J21.5 | 1.261601809 | -1.58821036 | 0.423776 | -3.74776 | 0.000178 | 0.000627 | Down |
| RP1-122K4.3 | 0.756428143 | -1.68660147 | 0.659249 | -2.55837 | 0.010516 | 0.023764 | Down |
| RP1-127B20.4 | 1.857077597 | -1.164429416 | 0.367298 | -3.17026 | 0.001523 | 0.004331 | Down |
| RP1-127D3.4 | 0.558034556 | -1.117209131 | 0.462646 | -2.41483 | 0.015743 | 0.033652 | Down |
| RP1-12G14.9 | 0.976193217 | -1.193370851 | 0.389887 | -3.06081 | 0.002207 | 0.006015 | Down |
| RP1-131F15.2 | 85.0919256 | -1.419030123 | 0.182447 | -7.77778 | 7.38E-15 | 9.17E-14 | Down |
| RP1-134E15.3 | 7.479702031 | 1.009706431 | 0.237356 | 4.253975 | 2.10E-05 | 8.78E-05 | Up |
| RP1-13D10.2 | 1.642949852 | -1.418054643 | 0.429672 | -3.30032 | 0.000966 | 0.002879 | Down |
| RP1-140C12.2 | 12.99502434 | -2.392160486 | 0.257878 | -9.27632 | 1.75E-20 | 3.38E-19 | Down |
| RP1-142F18.2 | 1.354581635 | -3.162670171 | 1.292319 | -2.44728 | 0.014394 | 0.031205 | Down |
| RP1-142L7.9 | 9.588884204 | -1.037755831 | 0.25979 | -3.9946 | 6.48E-05 | 0.000247 | Down |
| RP1-145M24.1 | 9.063547182 | 1.339223627 | 0.278813 | 4.803297 | 1.56E-06 | 7.81E-06 | Up |
| RP1-148H17.1 | 1.103998359 | -1.408572606 | 0.56338 | -2.50022 | 0.012412 | 0.027488 | Down |
| RP1-149A16.3 | 3.928237966 | -1.389636445 | 0.307234 | -4.52306 | 6.10E-06 | 2.79E-05 | Down |
| RP1-150O5.3 | 0.944257651 | -1.046754343 | 0.380762 | -2.74911 | 0.005976 | 0.014499 | Down |
| RP1-161N10.1 | 55.99797535 | 3.39349514 | 0.225911 | 15.02139 | 5.32E-51 | 6.80E-49 | Up |
| RP1-164L12.1 | 1.571784109 | -1.747126724 | 0.523646 | -3.33647 | 0.000849 | 0.00256 | Down |
| RP1-167F1.2 | 63.62246631 | -1.513874777 | 0.144095 | -10.5061 | 8.10E-26 | 2.30E-24 | Down |
| RP1-167G20.1 | 1.599212033 | -1.825085015 | 0.539635 | -3.38207 | 0.000719 | 0.002206 | Down |
| RP1-168L15.5 | 9.035735642 | -1.577491967 | 0.208989 | -7.54819 | 4.41E-14 | 5.07E-13 | Down |
| RP1-168L15.6 | 7.52256606 | -1.783741365 | 0.260138 | -6.85691 | 7.04E-12 | 6.55E-11 | Down |
| RP1-187B23.1 | 23.32387997 | -1.041798751 | 0.208006 | -5.00851 | 5.49E-07 | 2.93E-06 | Down |
| RP1-18D14.7 | 27.44600793 | -1.466498896 | 0.192783 | -7.60701 | 2.81E-14 | 3.29E-13 | Down |
| RP1-193H18.2 | 51.63622368 | -1.037532345 | 0.112912 | -9.18883 | 3.97E-20 | 7.47E-19 | Down |
| RP1-193H18.3 | 2.910237266 | -1.919987521 | 0.327183 | -5.86823 | 4.40E-09 | 3.06E-08 | Down |
| RP1-215K18.4 | 1.135653398 | -2.451296775 | 0.654959 | -3.74267 | 0.000182 | 0.000639 | Down |
| RP1-228H13.5 | 49.39872081 | 1.410674492 | 0.116129 | 12.1475 | 5.91E-34 | 2.86E-32 | Up |
| RP1-228P16.4 | 1.465869743 | -2.572591405 | 0.965146 | -2.66549 | 0.007688 | 0.018095 | Down |
| RP1-22N22.1 | 0.616131081 | -1.675708137 | 0.614948 | -2.72496 | 0.006431 | 0.015478 | Down |
| RP1-240B8.3 | 34.06824371 | -2.209678943 | 0.192604 | -11.4727 | 1.81E-30 | 7.14E-29 | Down |
| RP1-244F24.1 | 19.09927102 | 1.981566297 | 0.241529 | 8.204243 | 2.32E-16 | 3.23E-15 | Up |
| RP1-249I4.2 | 1.46210967 | -1.096661629 | 0.35898 | -3.05494 | 0.002251 | 0.006118 | Down |
| RP1-251M9.2 | 0.54633477 | -1.361183666 | 0.541767 | -2.51249 | 0.011988 | 0.026677 | Down |
| RP1-253P7.4 | 80.79229617 | -1.095232624 | 0.176809 | -6.19444 | 5.85E-10 | 4.50E-09 | Down |
| RP1-257C22.2 | 13.05987969 | -1.164525473 | 0.238171 | -4.88946 | 1.01E-06 | 5.20E-06 | Down |
| RP1-278O22.2 | 0.823552358 | -1.229626626 | 0.428849 | -2.86727 | 0.00414 | 0.010487 | Down |
| RP1-27O5.3 | 4.901888795 | 1.038437706 | 0.313441 | 3.313022 | 0.000923 | 0.002764 | Up |
| RP1-281H8.3 | 2.065689127 | -1.935333166 | 0.531591 | -3.64064 | 0.000272 | 0.000919 | Down |
| RP1-283K11.3 | 0.838723514 | -2.078407081 | 0.581665 | -3.5732 | 0.000353 | 0.001163 | Down |
| RP1-288H2.2 | 1.705984758 | 1.295976717 | 0.352733 | 3.674105 | 0.000239 | 0.000816 | Up |
| RP1-28C20.1 | 1.833738717 | -2.657971744 | 0.556436 | -4.77678 | 1.78E-06 | 8.85E-06 | Down |
| RP1-292L20.3 | 8.090040343 | -1.103853515 | 0.153381 | -7.1968 | 6.16E-13 | 6.34E-12 | Down |
| RP1-29C18.10 | 27.49831744 | -1.145871102 | 0.266937 | -4.29266 | 1.77E-05 | 7.48E-05 | Down |
| RP1-29C18.8 | 28.35952588 | -1.120566879 | 0.254156 | -4.40897 | 1.04E-05 | 4.58E-05 | Down |
| RP1-29C18.9 | 13.32832147 | -1.358932257 | 0.290779 | -4.67342 | 2.96E-06 | 1.43E-05 | Down |
| RP1-301L19.1 | 7.557459717 | -1.268951278 | 0.369369 | -3.43546 | 0.000592 | 0.001851 | Down |
| RP1-302G2.5 | 3.998412577 | -1.075686817 | 0.249437 | -4.31245 | 1.61E-05 | 6.89E-05 | Down |
| RP1-303A1.1 | 0.86552987 | -1.749438124 | 0.657116 | -2.6623 | 0.007761 | 0.018237 | Down |
| RP1-309I22.2 | 671.1528471 | -1.142545435 | 0.173246 | -6.59494 | 4.25E-11 | 3.66E-10 | Down |
| RP1-310O13.7 | 8.83433637 | -1.282752378 | 0.271065 | -4.73228 | 2.22E-06 | 1.09E-05 | Down |
| RP1-315G1.3 | 8.965852198 | -1.158657711 | 0.154754 | -7.48711 | 7.04E-14 | 7.95E-13 | Down |
| RP1-34L19.1 | 2.117588117 | -1.85175587 | 0.655566 | -2.82467 | 0.004733 | 0.011819 | Down |
| RP1-34M23.5 | 1.323076393 | 2.180585793 | 0.483286 | 4.511998 | 6.42E-06 | 2.93E-05 | Up |
| RP1-34P24.3 | 1.684376695 | -2.912048866 | 1.164543 | -2.50059 | 0.012399 | 0.027463 | Down |
| RP1-40E16.9 | 2.560276403 | -2.458319978 | 0.442854 | -5.55109 | 2.84E-08 | 1.80E-07 | Down |
| RP1-45I4.3 | 1.727707816 | -1.156454211 | 0.331138 | -3.49236 | 0.000479 | 0.00153 | Down |
| RP1-45N11.1 | 1.263089323 | -1.716592209 | 0.685857 | -2.50284 | 0.01232 | 0.027322 | Down |
| RP1-47A17.1 | 12.04886506 | -1.697260513 | 0.195755 | -8.67033 | 4.31E-18 | 6.91E-17 | Down |
| RP1-47M23.3 | 17.98230427 | -1.203007462 | 0.31228 | -3.85233 | 0.000117 | 0.000425 | Down |
| RP1-53O8.2 | 1.618218776 | 1.024381797 | 0.362237 | 2.827934 | 0.004685 | 0.011707 | Up |
| RP1-55C23.7 | 1.06156945 | -1.581206679 | 0.613688 | -2.57657 | 0.009979 | 0.022715 | Down |
| RP1-58B11.1 | 1.653207326 | -3.400468432 | 1.449489 | -2.34598 | 0.018977 | 0.039501 | Down |
| RP1-59B16.1 | 1.546876993 | -2.755788677 | 1.060341 | -2.59896 | 0.009351 | 0.021481 | Down |
| RP1-59D14.10 | 0.711548659 | -1.121697824 | 0.451504 | -2.48436 | 0.012978 | 0.028578 | Down |
| RP1-60O19.1 | 14.97367637 | -2.083909511 | 0.254341 | -8.19338 | 2.54E-16 | 3.53E-15 | Down |
| RP1-63G5.7 | 1.498962665 | -2.667963949 | 1.086157 | -2.45633 | 0.014036 | 0.030533 | Down |
| RP1-65J11.1 | 1.215292381 | 1.036278222 | 0.397601 | 2.606325 | 0.009152 | 0.021079 | Up |
| RP1-68D18.3 | 1.04555711 | -2.013913773 | 0.734113 | -2.74333 | 0.006082 | 0.014723 | Down |
| RP1-72A23.4 | 6.654775958 | -1.924377591 | 0.354592 | -5.42702 | 5.73E-08 | 3.48E-07 | Down |
| RP1-79C4.1 | 1.390337029 | -2.898428745 | 1.07036 | -2.7079 | 0.006771 | 0.016195 | Down |
| RP1-7G5.6 | 1.256668014 | -2.319301801 | 0.651804 | -3.55828 | 0.000373 | 0.001225 | Down |
| RP1-8B1.4 | 4.363831657 | -1.389875196 | 0.199358 | -6.97175 | 3.13E-12 | 3.01E-11 | Down |
| RP11-1006G14.1 | 9.443841707 | -1.406824811 | 0.301418 | -4.66735 | 3.05E-06 | 1.47E-05 | Down |
| RP11-1007J8.1 | 6.141930248 | -1.236827872 | 0.255503 | -4.84076 | 1.29E-06 | 6.56E-06 | Down |
| RP11-1007O24.2 | 7.169423992 | -1.074808094 | 0.222745 | -4.82528 | 1.40E-06 | 7.05E-06 | Down |
| RP11-1007O24.3 | 56.06423741 | -1.010069791 | 0.124294 | -8.12646 | 4.42E-16 | 6.02E-15 | Down |
| RP11-1007O24.4 | 1.147577111 | -1.211084858 | 0.46708 | -2.59288 | 0.009517 | 0.021819 | Down |
| RP11-1008C21.1 | 11.59550381 | 2.328172509 | 0.310258 | 7.503989 | 6.19E-14 | 7.03E-13 | Up |
| RP11-100G15.5 | 0.903865269 | -1.087251628 | 0.465323 | -2.33655 | 0.019463 | 0.040382 | Down |
| RP11-100L22.1 | 5.412223618 | -1.024967019 | 0.273358 | -3.74953 | 0.000177 | 0.000623 | Down |
| RP11-101E7.2 | 1.967399346 | -1.276765425 | 0.304104 | -4.19845 | 2.69E-05 | 0.00011 | Down |
| RP11-101P17.15 | 0.555599575 | -1.909171722 | 0.44084 | -4.33076 | 1.49E-05 | 6.38E-05 | Down |
| RP11-1022B3.1 | 9.329205715 | -2.952883689 | 0.236407 | -12.4907 | 8.39E-36 | 4.58E-34 | Down |
| RP11-1026M7.2 | 1.541192953 | -1.686467007 | 0.525517 | -3.20916 | 0.001331 | 0.003838 | Down |
| RP11-102M11.2 | 7.219387964 | -1.632023615 | 0.305131 | -5.34861 | 8.86E-08 | 5.26E-07 | Down |
| RP11-1036E20.7 | 48.41859234 | 3.509778915 | 0.33239 | 10.55921 | 4.61E-26 | 1.33E-24 | Up |
| RP11-1036E20.9 | 72.2737767 | 2.889887873 | 0.357083 | 8.09304 | 5.82E-16 | 7.85E-15 | Up |
| RP11-1042B17.3 | 2.811629024 | -1.978082875 | 0.30261 | -6.53675 | 6.29E-11 | 5.34E-10 | Down |
| RP11-104F15.9 | 5.854632807 | -1.067887695 | 0.279101 | -3.82617 | 0.00013 | 0.000469 | Down |
| RP11-104H15.10 | 22.90262942 | -1.714157866 | 0.26835 | -6.38778 | 1.68E-10 | 1.37E-09 | Down |
| RP11-1055B8.1 | 8.582000751 | -1.367316542 | 0.244627 | -5.5894 | 2.28E-08 | 1.46E-07 | Down |
| RP11-1055B8.2 | 1.464690264 | 2.114950095 | 0.344673 | 6.1361 | 8.46E-10 | 6.41E-09 | Up |
| RP11-1055B8.3 | 1.218178022 | -1.025601684 | 0.361464 | -2.83736 | 0.004549 | 0.011408 | Down |
| RP11-1057B8.2 | 1.555766096 | -2.822984523 | 0.746048 | -3.78392 | 0.000154 | 0.000549 | Down |
| RP11-1060G2.2 | 5.990982721 | -1.420462671 | 0.346055 | -4.10474 | 4.05E-05 | 0.000161 | Down |
| RP11-1069G10.1 | 35.28589443 | -1.139459862 | 0.190508 | -5.98116 | 2.22E-09 | 1.60E-08 | Down |
| RP11-106A1.2 | 0.660768408 | -1.655328697 | 0.53393 | -3.10027 | 0.001933 | 0.005353 | Down |
| RP11-106M3.1 | 0.655422675 | -1.026249008 | 0.435331 | -2.3574 | 0.018403 | 0.038498 | Down |
| RP11-1070N10.3 | 12.09147965 | -1.197667156 | 0.221286 | -5.4123 | 6.22E-08 | 3.76E-07 | Down |
| RP11-1070N10.5 | 2.681815756 | -1.507379808 | 0.444011 | -3.39491 | 0.000686 | 0.002117 | Down |
| RP11-1072C15.6 | 12.4242144 | -1.801381226 | 0.241431 | -7.46127 | 8.57E-14 | 9.59E-13 | Down |
| RP11-1074O12.1 | 13.40008124 | -1.063622836 | 0.192298 | -5.5311 | 3.18E-08 | 2.00E-07 | Down |
| RP11-1078H9.6 | 1.851503036 | -2.278812612 | 0.91315 | -2.49555 | 0.012576 | 0.027819 | Down |
| RP11-107I14.4 | 2.626172616 | -3.218796069 | 1.357626 | -2.3709 | 0.017745 | 0.037295 | Down |
| RP11-107K17.2 | 1.729285721 | -1.094250481 | 0.369165 | -2.96413 | 0.003035 | 0.007971 | Down |
| RP11-1081L13.3 | 1.281615693 | -2.797828043 | 1.083241 | -2.58283 | 0.009799 | 0.022361 | Down |
| RP11-1084I9.1 | 2.598437319 | -2.305928949 | 0.943856 | -2.44309 | 0.014562 | 0.031519 | Down |
| RP11-1084J3.1 | 1.240592765 | -2.037116602 | 0.617705 | -3.29788 | 0.000974 | 0.002902 | Down |
| RP11-1085N6.5 | 1.154725768 | -2.811222883 | 1.189797 | -2.36277 | 0.018139 | 0.038021 | Down |
| RP11-108A14.1 | 4.895507195 | 1.998196421 | 0.322927 | 6.187766 | 6.10E-10 | 4.69E-09 | Up |
| RP11-108M9.3 | 23.85014172 | -1.281118778 | 0.213297 | -6.00626 | 1.90E-09 | 1.38E-08 | Down |
| RP11-108M9.6 | 3.463020501 | -1.254297193 | 0.288337 | -4.35011 | 1.36E-05 | 5.88E-05 | Down |
| RP11-1099M24.9 | 12.40531687 | -1.002673377 | 0.241555 | -4.15092 | 3.31E-05 | 0.000134 | Down |
| RP11-109D9.4 | 1.255765451 | -2.096741543 | 0.389474 | -5.38352 | 7.30E-08 | 4.38E-07 | Down |
| RP11-109E24.2 | 2.566252169 | -1.272124899 | 0.224322 | -5.67098 | 1.42E-08 | 9.32E-08 | Down |
| RP11-109G23.3 | 7.219825736 | -1.94722356 | 0.268424 | -7.25429 | 4.04E-13 | 4.22E-12 | Down |
| RP11-109I13.2 | 3.707650741 | -1.204123149 | 0.42698 | -2.8201 | 0.004801 | 0.011962 | Down |
| RP11-109M19.4 | 1.701689542 | -1.825929201 | 0.765497 | -2.38529 | 0.017066 | 0.036071 | Down |
| RP11-109N23.1 | 1.066807189 | -1.492155313 | 0.399298 | -3.73694 | 0.000186 | 0.000652 | Down |
| RP11-10F11.2 | 1.357166931 | -2.393662805 | 0.859575 | -2.78471 | 0.005358 | 0.013179 | Down |
| RP11-10G15.4 | 2.811747263 | -2.323605368 | 0.36168 | -6.42448 | 1.32E-10 | 1.09E-09 | Down |
| RP11-10I6.2 | 0.815901299 | -1.412362245 | 0.58346 | -2.42067 | 0.015492 | 0.033203 | Down |
| RP11-10J21.2 | 17.52920823 | -1.02015075 | 0.235139 | -4.3385 | 1.43E-05 | 6.17E-05 | Down |
| RP11-10K16.1 | 44.78014434 | 1.090811576 | 0.109646 | 9.948472 | 2.56E-23 | 6.07E-22 | Up |
| RP11-1100L3.8 | 24.18758238 | -1.741118849 | 0.299457 | -5.81425 | 6.09E-09 | 4.17E-08 | Down |
| RP11-1101K5.1 | 2.181870834 | -3.046226957 | 0.354078 | -8.60326 | 7.75E-18 | 1.22E-16 | Down |
| RP11-1105O14.1 | 2.313112018 | -2.476793552 | 0.299273 | -8.27604 | 1.27E-16 | 1.81E-15 | Down |
| RP11-110H1.8 | 2.117994402 | -3.061590175 | 1.346719 | -2.27337 | 0.023004 | 0.046618 | Down |
| RP11-1112C15.1 | 1.816168552 | -3.280943203 | 1.086798 | -3.01891 | 0.002537 | 0.006793 | Down |
| RP11-111M22.5 | 1.844249523 | -1.062430402 | 0.323098 | -3.28826 | 0.001008 | 0.002991 | Down |
| RP11-1124B17.1 | 1.862949879 | -1.917015773 | 0.833036 | -2.30124 | 0.021378 | 0.043801 | Down |
| RP11-1127D7.1 | 1.433338567 | -2.672447851 | 0.727717 | -3.67237 | 0.00024 | 0.000821 | Down |
| RP11-112L6.3 | 2.648709549 | -1.019306599 | 0.316495 | -3.22061 | 0.001279 | 0.003706 | Down |
| RP11-1134I14.4 | 1.558739167 | 1.722655872 | 0.267332 | 6.443878 | 1.16E-10 | 9.64E-10 | Up |
| RP11-1134I14.8 | 32.31056231 | 1.184725462 | 0.14039 | 8.438791 | 3.21E-17 | 4.80E-16 | Up |
| RP11-113C12.4 | 4.469937344 | -1.022231642 | 0.197332 | -5.18027 | 2.22E-07 | 1.25E-06 | Down |
| RP11-1143G9.5 | 9.523336869 | -1.035002211 | 0.332197 | -3.11563 | 0.001836 | 0.005115 | Down |
| RP11-1148O4.1 | 2.251052754 | -1.866130632 | 0.443847 | -4.20445 | 2.62E-05 | 0.000108 | Down |
| RP11-1149O23.2 | 17.61161569 | 1.15320177 | 0.133966 | 8.608195 | 7.42E-18 | 1.17E-16 | Up |
| RP11-114F10.2 | 0.955712907 | -1.047753055 | 0.352129 | -2.97548 | 0.002925 | 0.007709 | Down |
| RP11-114G11.3 | 1.4881243 | -2.879916748 | 1.223161 | -2.35449 | 0.018548 | 0.038751 | Down |
| RP11-114H23.1 | 2.095017649 | -1.50177749 | 0.275897 | -5.44326 | 5.23E-08 | 3.20E-07 | Down |
| RP11-114H23.2 | 1.64503914 | -1.199103946 | 0.299814 | -3.99949 | 6.35E-05 | 0.000242 | Down |
| RP11-114H24.2 | 5.898982439 | -1.465625209 | 0.361947 | -4.04928 | 5.14E-05 | 0.0002 | Down |
| RP11-1152H15.1 | 1.458948165 | -3.123425154 | 1.180468 | -2.64592 | 0.008147 | 0.019031 | Down |
| RP11-1157N2_B.2 | 1.162616515 | 2.886038141 | 0.459001 | 6.287644 | 3.22E-10 | 2.55E-09 | Up |
| RP11-115D19.2 | 1.954492023 | -1.313126071 | 0.330212 | -3.97662 | 6.99E-05 | 0.000265 | Down |
| RP11-115H18.1 | 7.057967479 | -1.072073556 | 0.206866 | -5.18244 | 2.19E-07 | 1.24E-06 | Down |
| RP11-115J23.1 | 1.736092671 | -2.30693856 | 0.496187 | -4.64933 | 3.33E-06 | 1.59E-05 | Down |
| RP11-115L11.1 | 3.166649973 | -1.240369898 | 0.284193 | -4.36453 | 1.27E-05 | 5.53E-05 | Down |
| RP11-115N12.1 | 1.564414347 | -1.139269411 | 0.294143 | -3.87319 | 0.000107 | 0.000393 | Down |
| RP11-1166P10.1 | 5.785623547 | 1.053004556 | 0.201757 | 5.219176 | 1.80E-07 | 1.03E-06 | Up |
| RP11-116B13.1 | 0.676926042 | -1.560011029 | 0.478664 | -3.25909 | 0.001118 | 0.003281 | Down |
| RP11-116D17.3 | 4.54582948 | -2.316356476 | 0.310999 | -7.44811 | 9.47E-14 | 1.06E-12 | Down |
| RP11-116D17.4 | 1.985922685 | -2.403768011 | 0.502457 | -4.78403 | 1.72E-06 | 8.55E-06 | Down |
| RP11-116D2.1 | 1.547656355 | -3.051039902 | 1.314555 | -2.32097 | 0.020289 | 0.041861 | Down |
| RP11-116N8.4 | 2.361590142 | -1.503935827 | 0.385337 | -3.90291 | 9.50E-05 | 0.000351 | Down |
| RP11-116O18.1 | 9.182239423 | -2.041516642 | 0.312506 | -6.53273 | 6.46E-11 | 5.47E-10 | Down |
| RP11-1180F24.1 | 1.987526524 | -1.012387032 | 0.291399 | -3.47423 | 0.000512 | 0.001626 | Down |
| RP11-1182P23.5 | 1.484644962 | -1.598790194 | 0.326844 | -4.89159 | 1.00E-06 | 5.15E-06 | Down |
| RP11-118A3.1 | 0.713130284 | 2.073906537 | 0.66797 | 3.104788 | 0.001904 | 0.005283 | Up |
| RP11-118D22.3 | 2.434191604 | -1.393358073 | 0.375445 | -3.71122 | 0.000206 | 0.000715 | Down |
| RP11-118F19.1 | 4.960317666 | -1.215441754 | 0.199546 | -6.09104 | 1.12E-09 | 8.38E-09 | Down |
| RP11-118M9.3 | 1.425145675 | -1.25991831 | 0.303422 | -4.15237 | 3.29E-05 | 0.000133 | Down |
| RP11-1191J2.2 | 7.261085953 | -1.742222496 | 0.373244 | -4.66779 | 3.04E-06 | 1.46E-05 | Down |
| RP11-1197K16.2 | 0.822580346 | -1.674872498 | 0.633757 | -2.64277 | 0.008223 | 0.019192 | Down |
| RP11-119H12.3 | 1.079536648 | -2.459122895 | 0.905673 | -2.71524 | 0.006623 | 0.015881 | Down |
| RP11-119N19.1 | 1.199656895 | -1.843612746 | 0.564776 | -3.26432 | 0.001097 | 0.003228 | Down |
| RP11-11N7.4 | 1.77943853 | -1.381226586 | 0.303538 | -4.55042 | 5.35E-06 | 2.47E-05 | Down |
| RP11-120D5.1 | 9.283726765 | -1.290730011 | 0.162301 | -7.95269 | 1.83E-15 | 2.37E-14 | Down |
| RP11-120K18.2 | 2.374000601 | -1.462571653 | 0.495318 | -2.95279 | 0.003149 | 0.008235 | Down |
| RP11-1217F2.10 | 1.82120778 | -3.710148384 | 1.400125 | -2.64987 | 0.008052 | 0.01884 | Down |
| RP11-121C6.5 | 1.21464199 | -1.319375195 | 0.571787 | -2.30746 | 0.021029 | 0.043192 | Down |
| RP11-121G22.3 | 1.750957364 | -1.182462154 | 0.4586 | -2.57842 | 0.009925 | 0.022613 | Down |
| RP11-121L10.2 | 12.2740496 | -1.390131058 | 0.259076 | -5.36572 | 8.06E-08 | 4.81E-07 | Down |
| RP11-121M22.1 | 12.2416587 | -1.15046711 | 0.17462 | -6.58839 | 4.45E-11 | 3.82E-10 | Down |
| RP11-1220K2.2 | 12.89589236 | -1.768379265 | 0.313332 | -5.64379 | 1.66E-08 | 1.08E-07 | Down |
| RP11-122C5.3 | 1.377468392 | -1.603109695 | 0.344845 | -4.64879 | 3.34E-06 | 1.59E-05 | Down |
| RP11-122M14.1 | 1.639991677 | -2.113014663 | 0.65178 | -3.24192 | 0.001187 | 0.003469 | Down |
| RP11-123B3.2 | 0.8055501 | -1.12898089 | 0.379384 | -2.97583 | 0.002922 | 0.007702 | Down |
| RP11-123K19.1 | 2.945577654 | -2.583705532 | 0.367509 | -7.03033 | 2.06E-12 | 2.02E-11 | Down |
| RP11-123K3.4 | 10.7687248 | 1.369718371 | 0.373266 | 3.669553 | 0.000243 | 0.00083 | Up |
| RP11-124O11.1 | 1.394583717 | -2.521926445 | 0.797025 | -3.16417 | 0.001555 | 0.004413 | Down |
| RP11-1252I4.2 | 0.554851601 | -1.516717385 | 0.438211 | -3.46116 | 0.000538 | 0.001699 | Down |
| RP11-1259L22.1 | 1.741593435 | -1.522358135 | 0.329302 | -4.62298 | 3.78E-06 | 1.79E-05 | Down |
| RP11-1259L22.2 | 1.476937389 | -2.073792071 | 0.372417 | -5.56847 | 2.57E-08 | 1.64E-07 | Down |
| RP11-125H8.1 | 2.589001099 | -1.447275196 | 0.4038 | -3.58414 | 0.000338 | 0.001119 | Down |
| RP11-125O18.1 | 2.18462022 | -1.284850921 | 0.352577 | -3.64417 | 0.000268 | 0.000909 | Down |
| RP11-1260E13.1 | 1.290492938 | 1.46233627 | 0.471025 | 3.104583 | 0.001905 | 0.005285 | Up |
| RP11-126O22.1 | 8.440709176 | -2.266329652 | 0.605473 | -3.74307 | 0.000182 | 0.000638 | Down |
| RP11-127I20.5 | 1.247429716 | -1.453981762 | 0.455019 | -3.19543 | 0.001396 | 0.004006 | Down |
| RP11-127I20.7 | 1.173600936 | -1.324568452 | 0.43897 | -3.01745 | 0.002549 | 0.00682 | Down |
| RP11-1280N14.3 | 317.1016381 | -1.002329715 | 0.146792 | -6.82824 | 8.60E-12 | 7.93E-11 | Down |
| RP11-1281K21.1 | 2.095913261 | -2.881849243 | 0.408759 | -7.05023 | 1.79E-12 | 1.77E-11 | Down |
| RP11-1281K21.6 | 4.31123811 | -2.595758002 | 0.573236 | -4.52825 | 5.95E-06 | 2.72E-05 | Down |
| RP11-1281K21.8 | 7.373194206 | -3.60999898 | 0.431538 | -8.36542 | 5.99E-17 | 8.77E-16 | Down |
| RP11-128A17.1 | 3.60295271 | -1.085460141 | 0.286887 | -3.78358 | 0.000155 | 0.00055 | Down |
| RP11-128P10.1 | 1.057524683 | -1.157736451 | 0.404302 | -2.86355 | 0.004189 | 0.010602 | Down |
| RP11-1299A16.3 | 38.6248371 | -1.484024068 | 0.197109 | -7.52893 | 5.12E-14 | 5.86E-13 | Down |
| RP11-129G17.2 | 0.510601217 | -1.423313605 | 0.527894 | -2.69621 | 0.007013 | 0.016693 | Down |
| RP11-129H15.4 | 1.594702714 | -2.710813382 | 1.037651 | -2.61245 | 0.00899 | 0.020762 | Down |
| RP11-12A2.1 | 3.611802565 | -1.252885425 | 0.426315 | -2.93887 | 0.003294 | 0.008574 | Down |
| RP11-12A2.3 | 4.474641159 | -2.458981478 | 0.303285 | -8.10784 | 5.15E-16 | 6.98E-15 | Down |
| RP11-12A20.12 | 0.962576805 | -1.185567985 | 0.479647 | -2.47175 | 0.013445 | 0.029474 | Down |
| RP11-12A20.4 | 10.36939871 | -1.214730636 | 0.217616 | -5.582 | 2.38E-08 | 1.52E-07 | Down |
| RP11-12A20.7 | 8.872886506 | -1.107142149 | 0.198332 | -5.58226 | 2.37E-08 | 1.52E-07 | Down |
| RP11-12J10.3 | 90.90384075 | -1.12720699 | 0.112736 | -9.9986 | 1.55E-23 | 3.74E-22 | Down |
| RP11-12M5.3 | 0.80858548 | -1.385676539 | 0.386261 | -3.58741 | 0.000334 | 0.001107 | Down |
| RP11-130C6.1 | 0.554835941 | -1.011304958 | 0.413294 | -2.44694 | 0.014408 | 0.031231 | Down |
| RP11-131J3.1 | 3.169689433 | -1.247652868 | 0.516193 | -2.41703 | 0.015648 | 0.033472 | Down |
| RP11-131K5.2 | 4.866804477 | -1.201777294 | 0.365843 | -3.28495 | 0.00102 | 0.003023 | Down |
| RP11-131P10.2 | 1.136447176 | -2.766910334 | 1.075316 | -2.57311 | 0.010079 | 0.022904 | Down |
| RP11-132J14.3 | 2.073115975 | -1.169304982 | 0.43436 | -2.69202 | 0.007102 | 0.016873 | Down |
| RP11-132M7.3 | 2.124684661 | -1.743030963 | 0.4407 | -3.95514 | 7.65E-05 | 0.000288 | Down |
| RP11-132N15.2 | 1.721927801 | -3.135556685 | 1.335292 | -2.34822 | 0.018864 | 0.039298 | Down |
| RP11-133K1.7 | 4.179188448 | -1.766089079 | 0.427975 | -4.12662 | 3.68E-05 | 0.000147 | Down |
| RP11-133K1.8 | 9.324936686 | -2.183328605 | 0.262617 | -8.31373 | 9.27E-17 | 1.33E-15 | Down |
| RP11-133M8.3 | 7.440832201 | -1.509224744 | 0.240683 | -6.2706 | 3.60E-10 | 2.84E-09 | Down |
| RP11-1348G14.6 | 3.254206875 | -1.036599073 | 0.31365 | -3.30496 | 0.00095 | 0.002836 | Down |
| RP11-134E15.1 | 7.270298579 | -1.152707734 | 0.254526 | -4.52884 | 5.93E-06 | 2.72E-05 | Down |
| RP11-134E15.2 | 1.451672723 | -1.010564355 | 0.342356 | -2.95179 | 0.003159 | 0.008258 | Down |
| RP11-134K13.2 | 2.35655932 | -1.536330316 | 0.627938 | -2.44663 | 0.01442 | 0.031251 | Down |
| RP11-134O21.1 | 1.938893378 | -1.268381393 | 0.347979 | -3.645 | 0.000267 | 0.000906 | Down |
| RP11-136K7.2 | 9.438178553 | -2.284034454 | 0.340847 | -6.70106 | 2.07E-11 | 1.83E-10 | Down |
| RP11-1379J22.7 | 1.569567022 | -1.899502185 | 0.709641 | -2.67671 | 0.007435 | 0.017561 | Down |
| RP11-138B4.1 | 6.228384072 | -1.488595256 | 0.433589 | -3.43319 | 0.000597 | 0.001864 | Down |
| RP11-138E2.1 | 1.937174287 | -1.071653221 | 0.256471 | -4.17846 | 2.93E-05 | 0.00012 | Down |
| RP11-138I1.2 | 5.403440527 | -1.234783868 | 0.283787 | -4.35109 | 1.35E-05 | 5.85E-05 | Down |
| RP11-138I17.1 | 2.745064491 | -2.990649293 | 0.466671 | -6.40847 | 1.47E-10 | 1.20E-09 | Down |
| RP11-138I18.1 | 2.715811092 | -1.044825874 | 0.420009 | -2.48763 | 0.01286 | 0.028363 | Down |
| RP11-138I18.2 | 4.623165117 | -1.376580714 | 0.547471 | -2.51444 | 0.011922 | 0.026547 | Down |
| RP11-138P22.1 | 5.117755001 | -1.278173338 | 0.329526 | -3.87883 | 0.000105 | 0.000385 | Down |
| RP11-139H14.5 | 7.061770855 | -1.418714639 | 0.292716 | -4.84673 | 1.26E-06 | 6.38E-06 | Down |
| RP11-13E1.5 | 0.861409895 | -1.065491326 | 0.440754 | -2.41743 | 0.015631 | 0.033444 | Down |
| RP11-13K12.6 | 1.410600713 | -3.022026919 | 0.994146 | -3.03982 | 0.002367 | 0.006399 | Down |
| RP11-140L24.3 | 0.837985622 | -1.27953882 | 0.417042 | -3.06813 | 0.002154 | 0.005889 | Down |
| RP11-141A19.1 | 0.729900425 | -1.485701941 | 0.544068 | -2.73073 | 0.006319 | 0.015242 | Down |
| RP11-141M1.1 | 12.65968993 | -1.951868426 | 0.253219 | -7.70822 | 1.28E-14 | 1.55E-13 | Down |
| RP11-141M1.3 | 2.102534665 | -1.532190632 | 0.298927 | -5.12564 | 2.97E-07 | 1.64E-06 | Down |
| RP11-141M1.4 | 14.80262131 | -2.048506718 | 0.199459 | -10.2703 | 9.59E-25 | 2.52E-23 | Down |
| RP11-141M3.6 | 13.12784616 | -1.302526901 | 0.216656 | -6.01197 | 1.83E-09 | 1.34E-08 | Down |
| RP11-142A22.3 | 2.090291093 | -3.006364013 | 0.637246 | -4.71774 | 2.38E-06 | 1.16E-05 | Down |
| RP11-142A23.1 | 1.776968777 | -1.136939427 | 0.455272 | -2.49727 | 0.012515 | 0.027697 | Down |
| RP11-142C4.4 | 2.375628313 | -1.763640228 | 0.494052 | -3.56975 | 0.000357 | 0.001176 | Down |
| RP11-142L1.3 | 1.322299144 | -1.401536155 | 0.532183 | -2.63356 | 0.008449 | 0.019659 | Down |
| RP11-142L4.3 | 1.358020492 | -1.716833995 | 0.532891 | -3.22174 | 0.001274 | 0.003693 | Down |
| RP11-143E21.2 | 1.449671668 | -2.943839736 | 0.753349 | -3.90767 | 9.32E-05 | 0.000345 | Down |
| RP11-143E21.3 | 1.317546788 | -2.622795425 | 1.003209 | -2.61441 | 0.008938 | 0.02066 | Down |
| RP11-143I21.1 | 4.564997676 | -1.701227114 | 0.311334 | -5.46431 | 4.65E-08 | 2.86E-07 | Down |
| RP11-143N13.2 | 0.94787056 | -2.3789725 | 0.430139 | -5.53071 | 3.19E-08 | 2.01E-07 | Down |
| RP11-144G7.2 | 1.381365385 | -1.826500218 | 0.530076 | -3.44574 | 0.00057 | 0.001788 | Down |
| RP11-144H23.2 | 3.711809339 | -1.079388165 | 0.354466 | -3.04511 | 0.002326 | 0.006298 | Down |
| RP11-145E5.5 | 2.086715999 | 1.681945003 | 0.492894 | 3.412389 | 0.000644 | 0.001997 | Up |
| RP11-145G20.1 | 1.850461182 | -2.402969097 | 0.772669 | -3.10996 | 0.001871 | 0.005203 | Down |
| RP11-145O15.3 | 2.280842865 | -2.855609336 | 1.125612 | -2.53694 | 0.011183 | 0.025077 | Down |
| RP11-146D12.2 | 36.10187336 | -1.687843277 | 0.160048 | -10.5458 | 5.31E-26 | 1.52E-24 | Down |
| RP11-146F11.5 | 1.251621777 | -1.196056243 | 0.386139 | -3.09747 | 0.001952 | 0.005398 | Down |
| RP11-148L24.1 | 1.579052914 | -2.029344483 | 0.605219 | -3.35307 | 0.000799 | 0.002423 | Down |
| RP11-148O21.4 | 2.90403011 | -1.31347966 | 0.553585 | -2.37268 | 0.01766 | 0.037148 | Down |
| RP11-148O21.6 | 3.485689784 | -1.550956284 | 0.526739 | -2.94445 | 0.003235 | 0.008433 | Down |
| RP11-149B9.2 | 1.198748689 | -2.672317907 | 0.785501 | -3.40206 | 0.000669 | 0.002067 | Down |
| RP11-14I17.1 | 2.328383527 | -1.273386597 | 0.413398 | -3.08029 | 0.002068 | 0.005678 | Down |
| RP11-14I2.2 | 1.649326013 | -2.571824021 | 0.700795 | -3.66986 | 0.000243 | 0.000829 | Down |
| RP11-14J7.6 | 1.842730834 | -2.623917765 | 0.920077 | -2.85185 | 0.004347 | 0.010951 | Down |
| RP11-14N7.2 | 12.99333287 | -1.207887076 | 0.193489 | -6.24268 | 4.30E-10 | 3.36E-09 | Down |
| RP11-150O12.3 | 5.20155684 | -1.613852753 | 0.364947 | -4.42216 | 9.77E-06 | 4.32E-05 | Down |
| RP11-151F5.2 | 1.140898531 | -1.216399177 | 0.426768 | -2.85026 | 0.004368 | 0.011001 | Down |
| RP11-152H18.4 | 2.603318568 | -1.01641817 | 0.253681 | -4.00667 | 6.16E-05 | 0.000236 | Down |
| RP11-153F1.1 | 0.687247342 | -1.752679338 | 0.487841 | -3.59272 | 0.000327 | 0.001087 | Down |
| RP11-153F5.7 | 7.108155901 | 1.034972661 | 0.256633 | 4.032891 | 5.51E-05 | 0.000213 | Up |
| RP11-154D17.1 | 2.685740747 | -2.268740318 | 0.450177 | -5.03967 | 4.66E-07 | 2.52E-06 | Down |
| RP11-154D6.2 | 2.695775851 | -1.127678739 | 0.40642 | -2.77467 | 0.005526 | 0.013538 | Down |
| RP11-155D18.13 | 14.04631865 | -1.185579964 | 0.181792 | -6.52161 | 6.96E-11 | 5.87E-10 | Down |
| RP11-156E8.1 | 32.81889847 | -1.414820846 | 0.129535 | -10.9223 | 9.02E-28 | 2.96E-26 | Down |
| RP11-156L14.1 | 2.729748052 | -1.243130675 | 0.534222 | -2.32699 | 0.019966 | 0.041283 | Down |
| RP11-157G21.2 | 7.04310625 | -1.510248246 | 0.329548 | -4.58278 | 4.59E-06 | 2.14E-05 | Down |
| RP11-157J24.1 | 4.899426651 | -1.441648925 | 0.315279 | -4.57262 | 4.82E-06 | 2.24E-05 | Down |
| RP11-157L3.5 | 1.4149125 | -2.433190371 | 0.635819 | -3.82686 | 0.00013 | 0.000467 | Down |
| RP11-158H5.8 | 15.82387452 | -1.203529245 | 0.250333 | -4.80771 | 1.53E-06 | 7.65E-06 | Down |
| RP11-158I9.5 | 8.284008515 | -1.583482403 | 0.400815 | -3.95065 | 7.79E-05 | 0.000293 | Down |
| RP11-159D12.10 | 4.175309574 | -1.524972913 | 0.534212 | -2.85462 | 0.004309 | 0.010867 | Down |
| RP11-159D23.2 | 2.032055774 | -1.709057558 | 0.468423 | -3.64853 | 0.000264 | 0.000895 | Down |
| RP11-159F24.3 | 10.35740544 | -1.08029785 | 0.154407 | -6.99642 | 2.63E-12 | 2.54E-11 | Down |
| RP11-159H10.1 | 2.413683465 | -2.785436953 | 0.724118 | -3.84666 | 0.00012 | 0.000434 | Down |
| RP11-159H10.3 | 2.99205855 | -1.331236134 | 0.466304 | -2.85487 | 0.004305 | 0.01086 | Down |
| RP11-159K7.2 | 0.964318788 | -1.409104822 | 0.543417 | -2.59304 | 0.009513 | 0.02181 | Down |
| RP11-15B17.1 | 2.298935775 | -1.25451201 | 0.35624 | -3.52154 | 0.000429 | 0.001386 | Down |
| RP11-15B24.5 | 2.456916701 | -1.835314883 | 0.517305 | -3.54784 | 0.000388 | 0.001269 | Down |
| RP11-15E1.1 | 5.235961915 | -1.849706071 | 0.345172 | -5.3588 | 8.38E-08 | 4.99E-07 | Down |
| RP11-15E1.5 | 10.72318486 | -1.799382154 | 0.235738 | -7.63298 | 2.29E-14 | 2.72E-13 | Down |
| RP11-15E1.6 | 2.257617544 | -1.774619923 | 0.515067 | -3.44542 | 0.00057 | 0.001789 | Down |
| RP11-15G8.1 | 1.554169058 | -3.310984077 | 1.283558 | -2.57954 | 0.009893 | 0.022548 | Down |
| RP11-15H7.2 | 1.435781387 | -1.767843276 | 0.728636 | -2.42624 | 0.015256 | 0.032785 | Down |
| RP11-15I20.1 | 1.385670717 | -1.003734327 | 0.390206 | -2.57232 | 0.010102 | 0.022945 | Down |
| RP11-15J22.8 | 1.031296955 | -1.409757867 | 0.516375 | -2.7301 | 0.006331 | 0.015264 | Down |
| RP11-15P13.1 | 0.821828967 | -1.003534561 | 0.37135 | -2.7024 | 0.006884 | 0.016428 | Down |
| RP11-160C18.2 | 1.632069617 | -2.939030012 | 0.979483 | -3.00059 | 0.002695 | 0.007158 | Down |
| RP11-160E2.11 | 27.2693553 | -1.386590569 | 0.237605 | -5.83569 | 5.36E-09 | 3.70E-08 | Down |
| RP11-160E2.19 | 11.62287606 | -1.212444376 | 0.249879 | -4.85212 | 1.22E-06 | 6.22E-06 | Down |
| RP11-160E2.6 | 315.8532589 | -1.260271588 | 0.291592 | -4.32204 | 1.55E-05 | 6.62E-05 | Down |
| RP11-160H12.2 | 4.455311402 | -2.114093215 | 0.272548 | -7.75679 | 8.71E-15 | 1.08E-13 | Down |
| RP11-160H22.5 | 1.217445438 | -1.032159974 | 0.33878 | -3.04669 | 0.002314 | 0.006268 | Down |
| RP11-160O5.1 | 33.54447617 | -1.372145795 | 0.122115 | -11.2365 | 2.70E-29 | 9.86E-28 | Down |
| RP11-161D15.3 | 1.908097494 | -3.353676348 | 1.259232 | -2.66327 | 0.007739 | 0.018193 | Down |
| RP11-161I2.1 | 20.35496711 | 1.425494238 | 0.241511 | 5.90239 | 3.58E-09 | 2.52E-08 | Up |
| RP11-161M6.2 | 64.10939763 | 1.270637948 | 0.19199 | 6.618266 | 3.63E-11 | 3.14E-10 | Up |
| RP11-162A23.5 | 1.506963151 | -1.081674466 | 0.388403 | -2.78493 | 0.005354 | 0.013171 | Down |
| RP11-162G10.5 | 16.28505021 | -1.165543145 | 0.150686 | -7.7349 | 1.03E-14 | 1.27E-13 | Down |
| RP11-162J8.2 | 8.656995172 | -1.262330642 | 0.244554 | -5.16176 | 2.45E-07 | 1.37E-06 | Down |
| RP11-162K11.4 | 2.705116131 | -1.668658592 | 0.475307 | -3.5107 | 0.000447 | 0.001439 | Down |
| RP11-163O19.16 | 0.976676247 | -2.113584913 | 0.911561 | -2.31864 | 0.020414 | 0.042083 | Down |
| RP11-163O19.8 | 1.380762602 | -3.146392508 | 1.05325 | -2.98732 | 0.002814 | 0.007447 | Down |
| RP11-164H13.1 | 9.271347201 | -2.045744703 | 0.671317 | -3.04736 | 0.002309 | 0.006255 | Down |
| RP11-165A20.3 | 0.615588352 | -1.438356409 | 0.413381 | -3.47949 | 0.000502 | 0.001597 | Down |
| RP11-166B2.7 | 1.577905442 | -1.380412737 | 0.414231 | -3.33247 | 0.000861 | 0.002594 | Down |
| RP11-167H9.3 | 1.816838133 | -1.843147968 | 0.571604 | -3.22452 | 0.001262 | 0.003662 | Down |
| RP11-167N4.5 | 2.228339541 | -1.081178084 | 0.384392 | -2.8127 | 0.004913 | 0.012194 | Down |
| RP11-168K11.3 | 3.138573815 | -1.731024834 | 0.384112 | -4.50657 | 6.59E-06 | 3.00E-05 | Down |
| RP11-16B13.1 | 0.64046174 | -1.678227986 | 0.62254 | -2.69578 | 0.007022 | 0.016709 | Down |
| RP11-16C1.1 | 0.846110423 | -1.079827929 | 0.392372 | -2.75205 | 0.005922 | 0.014386 | Down |
| RP11-16D22.2 | 0.492849449 | 1.867559236 | 0.551317 | 3.387452 | 0.000705 | 0.002169 | Up |
| RP11-16E18.1 | 2.711112176 | -2.199448845 | 0.852056 | -2.58134 | 0.009842 | 0.022445 | Down |
| RP11-16F15.2 | 0.72639709 | -1.06267376 | 0.469314 | -2.26431 | 0.023555 | 0.047572 | Down |
| RP11-16K12.1 | 15.43854271 | 1.425939668 | 0.256224 | 5.565206 | 2.62E-08 | 1.67E-07 | Up |
| RP11-16L9.1 | 1.182830055 | -2.326154655 | 0.797627 | -2.91634 | 0.003542 | 0.009138 | Down |
| RP11-16N11.2 | 47.57210985 | -1.088702518 | 0.088375 | -12.3192 | 7.14E-35 | 3.65E-33 | Down |
| RP11-170K4.2 | 6.869362884 | -1.10332582 | 0.268537 | -4.10865 | 3.98E-05 | 0.000158 | Down |
| RP11-171G2.1 | 3.034182897 | -1.133058066 | 0.272628 | -4.15606 | 3.24E-05 | 0.000131 | Down |
| RP11-171I2.1 | 13.66842033 | -1.100185845 | 0.2414 | -4.55752 | 5.18E-06 | 2.40E-05 | Down |
| RP11-171N4.1 | 1.063143353 | -2.508197219 | 1.069801 | -2.34454 | 0.01905 | 0.039631 | Down |
| RP11-172C16.4 | 2.349472028 | -1.225615268 | 0.404743 | -3.02814 | 0.002461 | 0.006614 | Down |
| RP11-173D14.3 | 4.725547378 | -1.525391946 | 0.373816 | -4.0806 | 4.49E-05 | 0.000177 | Down |
| RP11-173E2.1 | 2.568701056 | -1.13299036 | 0.385791 | -2.9368 | 0.003316 | 0.008623 | Down |
| RP11-173P15.7 | 0.923983068 | -1.132805837 | 0.377829 | -2.9982 | 0.002716 | 0.007208 | Down |
| RP11-174J11.1 | 0.663427285 | -1.308020924 | 0.516543 | -2.53226 | 0.011333 | 0.025376 | Down |
| RP11-174O3.1 | 1.663485 | -1.944901625 | 0.460088 | -4.22724 | 2.37E-05 | 9.80E-05 | Down |
| RP11-177B4.2 | 2.070174317 | -2.189093024 | 0.571935 | -3.82752 | 0.000129 | 0.000466 | Down |
| RP11-178D12.2 | 2.754315131 | -1.054537191 | 0.264199 | -3.99145 | 6.57E-05 | 0.00025 | Down |
| RP11-179B15.6 | 2.781618072 | -2.269119824 | 0.478697 | -4.7402 | 2.14E-06 | 1.05E-05 | Down |
| RP11-179B2.2 | 15.33579629 | -1.016702114 | 0.123231 | -8.25041 | 1.58E-16 | 2.23E-15 | Down |
| RP11-179K3.2 | 2.844924276 | -2.97984183 | 0.618607 | -4.81702 | 1.46E-06 | 7.33E-06 | Down |
| RP11-17E2.2 | 3.071541478 | -2.836806134 | 0.40253 | -7.04743 | 1.82E-12 | 1.80E-11 | Down |
| RP11-17E3.1 | 0.692153895 | -1.098074864 | 0.487041 | -2.25458 | 0.024159 | 0.048659 | Down |
| RP11-180D15.1 | 0.904882794 | -1.876274551 | 0.780352 | -2.40439 | 0.016199 | 0.034485 | Down |
| RP11-180I4.4 | 4.082367137 | -1.946320157 | 0.421728 | -4.61511 | 3.93E-06 | 1.85E-05 | Down |
| RP11-180N14.1 | 11.77836407 | -1.211768959 | 0.192319 | -6.30082 | 2.96E-10 | 2.36E-09 | Down |
| RP11-181B18.1 | 1.178466665 | -2.230869978 | 0.421186 | -5.29664 | 1.18E-07 | 6.90E-07 | Down |
| RP11-182J1.10 | 1.099493484 | -1.235478716 | 0.54821 | -2.25366 | 0.024218 | 0.04875 | Down |
| RP11-182J1.13 | 4.336207122 | -1.109445093 | 0.316329 | -3.50725 | 0.000453 | 0.001455 | Down |
| RP11-182M20.2 | 1.038526451 | -1.588485362 | 0.41006 | -3.87379 | 0.000107 | 0.000392 | Down |
| RP11-182N22.7 | 2.998286409 | -1.492684195 | 0.434652 | -3.43421 | 0.000594 | 0.001858 | Down |
| RP11-183E9.2 | 6.054057318 | -1.219157283 | 0.335636 | -3.63238 | 0.000281 | 0.000947 | Down |
| RP11-184B22.2 | 0.841441138 | -1.07345263 | 0.408281 | -2.6292 | 0.008559 | 0.019885 | Down |
| RP11-184E9.1 | 0.877296809 | -1.784166087 | 0.68091 | -2.62027 | 0.008786 | 0.020345 | Down |
| RP11-184E9.2 | 1.31956695 | -2.808097502 | 0.964911 | -2.91022 | 0.003612 | 0.009299 | Down |
| RP11-185N2.1 | 3.549006826 | -2.117895925 | 0.432597 | -4.89577 | 9.79E-07 | 5.05E-06 | Down |
| RP11-18F14.4 | 3.854708577 | -1.245990624 | 0.430284 | -2.89574 | 0.003783 | 0.009687 | Down |
| RP11-190A12.9 | 1.432365545 | -1.475900586 | 0.443222 | -3.32994 | 0.000869 | 0.002616 | Down |
| RP11-190C22.8 | 1.723561071 | -1.356012541 | 0.359343 | -3.77358 | 0.000161 | 0.00057 | Down |
| RP11-190C22.9 | 1.620466854 | -1.17324993 | 0.357079 | -3.28569 | 0.001017 | 0.003017 | Down |
| RP11-190D6.1 | 21.99072461 | -1.387012812 | 0.213462 | -6.49769 | 8.16E-11 | 6.85E-10 | Down |
| RP11-190D6.2 | 2.237886809 | -1.454432188 | 0.334484 | -4.34828 | 1.37E-05 | 5.92E-05 | Down |
| RP11-191L17.1 | 5.447579373 | -1.232582941 | 0.169176 | -7.2858 | 3.20E-13 | 3.38E-12 | Down |
| RP11-191L9.4 | 2.007947544 | -3.006005742 | 1.309321 | -2.29585 | 0.021684 | 0.044303 | Down |
| RP11-192H23.4 | 114.194098 | -1.429537697 | 0.127635 | -11.2002 | 4.07E-29 | 1.47E-27 | Down |
| RP11-192H23.8 | 8.950892365 | -1.499253822 | 0.156329 | -9.59036 | 8.78E-22 | 1.87E-20 | Down |
| RP11-193H22.2 | 11.34716785 | -1.416564098 | 0.322043 | -4.39868 | 1.09E-05 | 4.78E-05 | Down |
| RP11-193J6.1 | 20.46686588 | -1.110825854 | 0.201664 | -5.5083 | 3.62E-08 | 2.26E-07 | Down |
| RP11-193M21.1 | 3.828985308 | -1.1052904 | 0.238118 | -4.64177 | 3.45E-06 | 1.64E-05 | Down |
| RP11-195B21.3 | 2.7943975 | -1.580570771 | 0.3783 | -4.17809 | 2.94E-05 | 0.00012 | Down |
| RP11-195C7.1 | 4.299219441 | -1.12122942 | 0.158575 | -7.07067 | 1.54E-12 | 1.53E-11 | Down |
| RP11-195C7.3 | 0.836782307 | -1.208268234 | 0.384269 | -3.14433 | 0.001665 | 0.004693 | Down |
| RP11-195M16.3 | 2.55605771 | -3.076237909 | 0.730034 | -4.21383 | 2.51E-05 | 0.000104 | Down |
| RP11-196B3.1 | 15.53605545 | -1.357874386 | 0.270244 | -5.02461 | 5.04E-07 | 2.71E-06 | Down |
| RP11-196B3.2 | 17.91718829 | -1.331689231 | 0.250633 | -5.3133 | 1.08E-07 | 6.32E-07 | Down |
| RP11-196B3.3 | 2.585210622 | -1.205806051 | 0.336191 | -3.58667 | 0.000335 | 0.001109 | Down |
| RP11-196G11.3 | 12.22589709 | -1.00031956 | 0.203593 | -4.91333 | 8.95E-07 | 4.64E-06 | Down |
| RP11-196I18.3 | 1.474900999 | -2.865081448 | 0.772298 | -3.70981 | 0.000207 | 0.000719 | Down |
| RP11-197N18.7 | 8.76497115 | -1.119063466 | 0.25653 | -4.36231 | 1.29E-05 | 5.58E-05 | Down |
| RP11-197N18.8 | 30.37013915 | -1.083405498 | 0.225113 | -4.81272 | 1.49E-06 | 7.48E-06 | Down |
| RP11-19E18.2 | 3.264254925 | -1.262076675 | 0.344738 | -3.66097 | 0.000251 | 0.000855 | Down |
| RP11-19N8.4 | 3.523709038 | -1.258582056 | 0.407904 | -3.08549 | 0.002032 | 0.005593 | Down |
| RP11-19P22.5 | 1.646587895 | -2.607575012 | 1.022839 | -2.54935 | 0.010792 | 0.024302 | Down |
| RP11-1C1.4 | 1.119936728 | -2.703316882 | 0.993536 | -2.72091 | 0.00651 | 0.015644 | Down |
| RP11-1C8.4 | 2.847244705 | 1.89310065 | 0.375128 | 5.046552 | 4.50E-07 | 2.44E-06 | Up |
| RP11-1C8.7 | 1.060152288 | 2.663306597 | 0.400755 | 6.645727 | 3.02E-11 | 2.63E-10 | Up |
| RP11-1D12.1 | 2.984570455 | -1.149478493 | 0.305189 | -3.76645 | 0.000166 | 0.000585 | Down |
| RP11-1D12.2 | 1.278282285 | -1.604619505 | 0.338608 | -4.73887 | 2.15E-06 | 1.06E-05 | Down |
| RP11-1E1.2 | 2.329764218 | -2.267429895 | 0.587849 | -3.85716 | 0.000115 | 0.000417 | Down |
| RP11-1J11.1 | 6.143816824 | -2.205008752 | 0.257722 | -8.55576 | 1.17E-17 | 1.82E-16 | Down |
| RP11-202D1.2 | 1.052453203 | -2.510551586 | 0.847674 | -2.96169 | 0.00306 | 0.008028 | Down |
| RP11-203B7.2 | 17.68840729 | -1.692111957 | 0.578208 | -2.92648 | 0.003428 | 0.00888 | Down |
| RP11-203I16.7 | 1.885701721 | -1.455648654 | 0.391334 | -3.71971 | 0.000199 | 0.000694 | Down |
| RP11-203I2.1 | 21.65788547 | -1.458028483 | 0.166669 | -8.74807 | 2.17E-18 | 3.55E-17 | Down |
| RP11-203J24.8 | 17.60316376 | -1.433369192 | 0.205375 | -6.97928 | 2.97E-12 | 2.86E-11 | Down |
| RP11-203M5.8 | 1.17386415 | 2.053095563 | 0.466825 | 4.398003 | 1.09E-05 | 4.79E-05 | Up |
| RP11-204E4.4 | 1.56994348 | -2.854586628 | 1.077821 | -2.64848 | 0.008085 | 0.018909 | Down |
| RP11-204N11.1 | 7.387682978 | -1.154556648 | 0.391923 | -2.94588 | 0.00322 | 0.0084 | Down |
| RP11-205K6.3 | 1.766202892 | -1.994987931 | 0.560644 | -3.55839 | 0.000373 | 0.001224 | Down |
| RP11-206F17.2 | 5.194016723 | -1.397680321 | 0.260696 | -5.36134 | 8.26E-08 | 4.92E-07 | Down |
| RP11-207F8.1 | 2.073763169 | -1.32217385 | 0.457414 | -2.89054 | 0.003846 | 0.009824 | Down |
| RP11-208N14.4 | 2.183902808 | -2.165824241 | 0.77981 | -2.77737 | 0.00548 | 0.013438 | Down |
| RP11-208P4.1 | 1.325253006 | -2.984066838 | 0.93386 | -3.19541 | 0.001396 | 0.004006 | Down |
| RP11-209D14.2 | 3.407240804 | -2.24365606 | 0.441372 | -5.08337 | 3.71E-07 | 2.03E-06 | Down |
| RP11-209M4.1 | 6.262029452 | -1.568278813 | 0.286445 | -5.47498 | 4.38E-08 | 2.70E-07 | Down |
| RP11-20B24.2 | 0.841543774 | -1.179083724 | 0.477731 | -2.46809 | 0.013584 | 0.029706 | Down |
| RP11-20J15.3 | 39.07001222 | 3.369878739 | 0.229899 | 14.6581 | 1.20E-48 | 1.40E-46 | Up |
| RP11-20J15.5 | 9.756019468 | 2.277197186 | 0.25885 | 8.797377 | 1.40E-18 | 2.33E-17 | Up |
| RP11-20P5.2 | 3.658301809 | -2.370913454 | 0.320947 | -7.38724 | 1.50E-13 | 1.64E-12 | Down |
| RP11-210H10_A.1 | 2.061015433 | -1.467900556 | 0.358181 | -4.09821 | 4.16E-05 | 0.000165 | Down |
| RP11-211F2.1 | 1.141419225 | -2.182224079 | 0.552882 | -3.947 | 7.91E-05 | 0.000297 | Down |
| RP11-211N8.6 | 2.959927862 | -1.346490249 | 0.435211 | -3.09388 | 0.001976 | 0.005458 | Down |
| RP11-212D3.4 | 1.960024693 | -1.355885646 | 0.478801 | -2.83183 | 0.004628 | 0.011582 | Down |
| RP11-212I21.3 | 8.163107714 | -1.07541266 | 0.261608 | -4.11078 | 3.94E-05 | 0.000157 | Down |
| RP11-213G2.5 | 4.183440947 | -1.965950358 | 0.399912 | -4.91596 | 8.84E-07 | 4.58E-06 | Down |
| RP11-213H15.4 | 1.756766663 | 1.646907744 | 0.492192 | 3.346066 | 0.00082 | 0.00248 | Up |
| RP11-214L13.1 | 2.077102728 | -3.416420928 | 1.099485 | -3.10729 | 0.001888 | 0.005245 | Down |
| RP11-214O1.1 | 1.171873698 | -2.415165191 | 0.984318 | -2.45364 | 0.014142 | 0.030734 | Down |
| RP11-214O1.3 | 8.549316 | -1.434763846 | 0.387122 | -3.70623 | 0.00021 | 0.000728 | Down |
| RP11-215H22.1 | 1.650396736 | -3.247918002 | 1.114308 | -2.91474 | 0.00356 | 0.00918 | Down |
| RP11-215P8.4 | 0.975298425 | -2.193243798 | 0.884199 | -2.48049 | 0.01312 | 0.028849 | Down |
| RP11-216B9.8 | 1.77381114 | -1.190129184 | 0.399735 | -2.97729 | 0.002908 | 0.007671 | Down |
| RP11-216L13.16 | 75.47310418 | 2.140576304 | 0.247436 | 8.651021 | 5.10E-18 | 8.12E-17 | Up |
| RP11-217B1.2 | 15.28778718 | -1.734319862 | 0.231692 | -7.48546 | 7.13E-14 | 8.05E-13 | Down |
| RP11-217O12.1 | 1.90036113 | -2.235103091 | 0.614652 | -3.63637 | 0.000277 | 0.000933 | Down |
| RP11-218I21.2 | 1.35252978 | -2.793112454 | 0.956464 | -2.92025 | 0.003498 | 0.009035 | Down |
| RP11-218M11.1 | 2.684916458 | -2.397441113 | 0.523521 | -4.57946 | 4.66E-06 | 2.17E-05 | Down |
| RP11-218M11.7 | 1.729957117 | -2.315767004 | 0.507199 | -4.5658 | 4.98E-06 | 2.31E-05 | Down |
| RP11-219B17.3 | 5.501755845 | -1.119281149 | 0.161349 | -6.93702 | 4.00E-12 | 3.82E-11 | Down |
| RP11-219B4.7 | 6.188439372 | -1.403006573 | 0.226464 | -6.19528 | 5.82E-10 | 4.48E-09 | Down |
| RP11-21A7A.2 | 3.130334123 | -2.450604745 | 0.290969 | -8.42223 | 3.69E-17 | 5.51E-16 | Down |
| RP11-21A7A.3 | 0.874039049 | -1.859944247 | 0.698307 | -2.6635 | 0.007733 | 0.018182 | Down |
| RP11-21A7A.4 | 3.69167711 | -1.541534138 | 0.254432 | -6.05873 | 1.37E-09 | 1.02E-08 | Down |
| RP11-21B21.4 | 1.97926602 | -1.039704143 | 0.321346 | -3.23546 | 0.001214 | 0.003543 | Down |
| RP11-21G15.1 | 6.812644629 | -1.532043317 | 0.305311 | -5.01797 | 5.22E-07 | 2.80E-06 | Down |
| RP11-21L1.1 | 2.130814463 | -3.275110739 | 0.990722 | -3.30578 | 0.000947 | 0.002829 | Down |
| RP11-21M24.2 | 10.68943905 | -1.033583402 | 0.193666 | -5.33694 | 9.45E-08 | 5.59E-07 | Down |
| RP11-220I1.2 | 2.788649517 | -1.659350916 | 0.366794 | -4.52393 | 6.07E-06 | 2.78E-05 | Down |
| RP11-220I1.5 | 2.128364425 | -1.310868461 | 0.34179 | -3.8353 | 0.000125 | 0.000453 | Down |
| RP11-221N13.3 | 18.79651421 | 3.624526219 | 0.232993 | 15.55636 | 1.44E-54 | 2.21E-52 | Up |
| RP11-221N13.4 | 1.10638423 | 3.095936908 | 0.418137 | 7.404117 | 1.32E-13 | 1.45E-12 | Up |
| RP11-222A5.1 | 2.06573811 | -2.107717533 | 0.679513 | -3.10181 | 0.001923 | 0.005329 | Down |
| RP11-223J6.1 | 1.064492348 | -1.505440291 | 0.502259 | -2.99734 | 0.002723 | 0.007226 | Down |
| RP11-225N10.1 | 1.82147693 | -1.452613796 | 0.61481 | -2.3627 | 0.018142 | 0.038025 | Down |
| RP11-227D13.1 | 8.717105092 | -1.013561016 | 0.194312 | -5.21615 | 1.83E-07 | 1.04E-06 | Down |
| RP11-227H15.4 | 1.374933129 | -2.306615207 | 0.643063 | -3.58692 | 0.000335 | 0.001108 | Down |
| RP11-227H15.5 | 0.839247277 | -1.810910101 | 0.499465 | -3.6257 | 0.000288 | 0.000969 | Down |
| RP11-229O3.1 | 10.75364301 | 1.028469524 | 0.263575 | 3.901995 | 9.54E-05 | 0.000352 | Up |
| RP11-22H5.2 | 4.055267487 | -1.063420146 | 0.439665 | -2.4187 | 0.015576 | 0.033353 | Down |
| RP11-22L13.1 | 41.20863018 | -1.123095496 | 0.184025 | -6.10296 | 1.04E-09 | 7.81E-09 | Down |
| RP11-22N19.2 | 5.851469855 | -1.213266905 | 0.277977 | -4.36463 | 1.27E-05 | 5.53E-05 | Down |
| RP11-22P4.2 | 3.047156135 | -1.306139149 | 0.325792 | -4.00912 | 6.09E-05 | 0.000233 | Down |
| RP11-230G5.2 | 20.65508631 | 3.065329008 | 0.333576 | 9.1893 | 3.95E-20 | 7.44E-19 | Up |
| RP11-231E19.1 | 10.23729608 | -1.053348823 | 0.229831 | -4.58315 | 4.58E-06 | 2.14E-05 | Down |
| RP11-231E4.2 | 1.434012726 | -2.727357239 | 1.208474 | -2.25686 | 0.024017 | 0.048388 | Down |
| RP11-231N9.1 | 6.598271867 | -1.132865156 | 0.282726 | -4.00693 | 6.15E-05 | 0.000235 | Down |
| RP11-232L2.1 | 0.812786156 | -2.102979765 | 0.580165 | -3.6248 | 0.000289 | 0.000971 | Down |
| RP11-232L2.2 | 0.607682576 | -1.987851236 | 0.519323 | -3.82778 | 0.000129 | 0.000466 | Down |
| RP11-234B24.2 | 3.124023893 | -2.021440495 | 0.418335 | -4.83211 | 1.35E-06 | 6.83E-06 | Down |
| RP11-234B24.5 | 2.792183098 | -2.287493786 | 0.57977 | -3.94552 | 7.96E-05 | 0.000298 | Down |
| RP11-234G16.4 | 1.866850904 | -1.1811694 | 0.417381 | -2.82996 | 0.004655 | 0.011644 | Down |
| RP11-234G16.5 | 1.624059771 | -1.347412169 | 0.309484 | -4.35373 | 1.34E-05 | 5.79E-05 | Down |
| RP11-235C23.6 | 1.620453928 | -2.436280504 | 0.687275 | -3.54484 | 0.000393 | 0.001282 | Down |
| RP11-235P11.1 | 2.61170382 | 1.731843683 | 0.381497 | 4.539596 | 5.64E-06 | 2.59E-05 | Up |
| RP11-236L14.2 | 9.103290787 | -1.577915758 | 0.219858 | -7.17699 | 7.13E-13 | 7.30E-12 | Down |
| RP11-236P24.3 | 1.872913567 | -1.057597272 | 0.389676 | -2.71404 | 0.006647 | 0.015927 | Down |
| RP11-238K6.1 | 15.78497999 | -2.489141059 | 0.266318 | -9.3465 | 9.06E-21 | 1.79E-19 | Down |
| RP11-23I7.1 | 1.220097898 | -2.401666415 | 0.688321 | -3.48917 | 0.000485 | 0.001547 | Down |
| RP11-240D10.4 | 1.600584045 | -1.336490564 | 0.441801 | -3.0251 | 0.002486 | 0.006674 | Down |
| RP11-240L7.4 | 3.865276513 | -1.340964543 | 0.248613 | -5.39378 | 6.90E-08 | 4.15E-07 | Down |
| RP11-241F15.10 | 1.673018588 | -1.414231401 | 0.329359 | -4.29389 | 1.76E-05 | 7.45E-05 | Down |
| RP11-241F15.3 | 1.125370472 | -1.481547849 | 0.49129 | -3.01563 | 0.002564 | 0.006855 | Down |
| RP11-241F15.7 | 1.158536512 | -2.78353735 | 0.526787 | -5.28399 | 1.26E-07 | 7.37E-07 | Down |
| RP11-241G9.3 | 2.12106605 | -1.771208646 | 0.261617 | -6.77023 | 1.29E-11 | 1.16E-10 | Down |
| RP11-242F24.1 | 3.033336149 | -3.041959619 | 0.431644 | -7.04738 | 1.82E-12 | 1.80E-11 | Down |
| RP11-243M5.2 | 5.741653208 | -1.312314535 | 0.280769 | -4.674 | 2.95E-06 | 1.42E-05 | Down |
| RP11-243M5.5 | 8.795737538 | -1.435200655 | 0.298031 | -4.81561 | 1.47E-06 | 7.38E-06 | Down |
| RP11-244F12.1 | 1.370079748 | -2.061519285 | 0.749147 | -2.75182 | 0.005926 | 0.014394 | Down |
| RP11-244M2.1 | 7.291161881 | 1.684459465 | 0.29977 | 5.619172 | 1.92E-08 | 1.24E-07 | Up |
| RP11-244N9.6 | 4.447678681 | -1.557891029 | 0.326233 | -4.7754 | 1.79E-06 | 8.91E-06 | Down |
| RP11-245J9.6 | 2.250145319 | -1.290987305 | 0.342871 | -3.76523 | 0.000166 | 0.000588 | Down |
| RP11-247L20.3 | 0.863691093 | -1.328076195 | 0.337702 | -3.93269 | 8.40E-05 | 0.000314 | Down |
| RP11-248B24.1 | 3.917440785 | -2.22794976 | 0.435039 | -5.12126 | 3.04E-07 | 1.68E-06 | Down |
| RP11-248J23.6 | 37.79416702 | 1.264657082 | 0.19144 | 6.606018 | 3.95E-11 | 3.41E-10 | Up |
| RP11-248J23.7 | 4.862455527 | 1.575519713 | 0.298474 | 5.27859 | 1.30E-07 | 7.57E-07 | Up |
| RP11-248M19.1 | 2.868557079 | -1.016504468 | 0.220086 | -4.61867 | 3.86E-06 | 1.82E-05 | Down |
| RP11-248N22.1 | 0.923920997 | -1.823956882 | 0.758251 | -2.40548 | 0.016151 | 0.034395 | Down |
| RP11-249C24.10 | 2.037970216 | -1.503597657 | 0.419806 | -3.58165 | 0.000341 | 0.001129 | Down |
| RP11-24B13.2 | 5.206748726 | -2.104345935 | 0.367156 | -5.73148 | 9.96E-09 | 6.68E-08 | Down |
| RP11-24C3.2 | 1.293666319 | -1.284881168 | 0.427051 | -3.00873 | 0.002623 | 0.006994 | Down |
| RP11-24F11.2 | 1.811457577 | 1.406954966 | 0.37374 | 3.764527 | 0.000167 | 0.00059 | Up |
| RP11-24H2.3 | 1.138086823 | -2.803894051 | 0.414716 | -6.76099 | 1.37E-11 | 1.24E-10 | Down |
| RP11-24I21.1 | 3.544130236 | -1.297444211 | 0.281732 | -4.60524 | 4.12E-06 | 1.94E-05 | Down |
| RP11-24M17.4 | 3.067141964 | -1.440475511 | 0.420898 | -3.42239 | 0.000621 | 0.001932 | Down |
| RP11-24M17.5 | 0.998571735 | -2.674048254 | 1.082916 | -2.4693 | 0.013538 | 0.029629 | Down |
| RP11-24M17.6 | 3.379821051 | -1.875564196 | 0.492061 | -3.81165 | 0.000138 | 0.000495 | Down |
| RP11-250B2.5 | 15.90377928 | -1.137712364 | 0.208322 | -5.46133 | 4.73E-08 | 2.91E-07 | Down |
| RP11-250H24.2 | 1.025471533 | -1.282039832 | 0.486962 | -2.63273 | 0.00847 | 0.019701 | Down |
| RP11-251P6.1 | 1.264160723 | -3.063316079 | 0.820023 | -3.73565 | 0.000187 | 0.000655 | Down |
| RP11-252C15.1 | 0.894228891 | -1.68831349 | 0.602819 | -2.8007 | 0.005099 | 0.012608 | Down |
| RP11-252I13.1 | 1.322730927 | -1.714702435 | 0.734562 | -2.33432 | 0.019579 | 0.040597 | Down |
| RP11-252I14.1 | 1.776343817 | -1.034107402 | 0.32071 | -3.22444 | 0.001262 | 0.003663 | Down |
| RP11-252P19.1 | 2.350640472 | -2.822229782 | 0.494628 | -5.70576 | 1.16E-08 | 7.70E-08 | Down |
| RP11-253D19.1 | 0.470749186 | -1.15098904 | 0.507561 | -2.26769 | 0.023348 | 0.047211 | Down |
| RP11-253E3.1 | 0.917863156 | -1.785316826 | 0.756962 | -2.35853 | 0.018347 | 0.038395 | Down |
| RP11-254A17.1 | 0.529025238 | -1.241858416 | 0.55011 | -2.25747 | 0.023978 | 0.048325 | Down |
| RP11-254A24.2 | 6.969199922 | -1.351584969 | 0.191771 | -7.04791 | 1.82E-12 | 1.79E-11 | Down |
| RP11-254F19.4 | 6.054824629 | -2.053328115 | 0.34675 | -5.92164 | 3.19E-09 | 2.25E-08 | Down |
| RP11-254I22.3 | 0.960840554 | -1.065206918 | 0.430526 | -2.4742 | 0.013354 | 0.029298 | Down |
| RP11-255B23.1 | 6.602332542 | -1.202021524 | 0.31116 | -3.86304 | 0.000112 | 0.000408 | Down |
| RP11-255M2.3 | 0.85619286 | -1.065992698 | 0.434484 | -2.45347 | 0.014149 | 0.030746 | Down |
| RP11-256I23.1 | 1.344139487 | -1.166676068 | 0.434985 | -2.68211 | 0.007316 | 0.017313 | Down |
| RP11-256I9.2 | 2.258445659 | -1.833525399 | 0.612439 | -2.99381 | 0.002755 | 0.007301 | Down |
| RP11-257I14.1 | 1.543391202 | -2.966666785 | 1.204837 | -2.4623 | 0.013805 | 0.030116 | Down |
| RP11-258B16.1 | 2.670150368 | -2.893767567 | 0.584839 | -4.94797 | 7.50E-07 | 3.93E-06 | Down |
| RP11-259G18.2 | 1.480076238 | -2.073236936 | 0.84365 | -2.45746 | 0.013992 | 0.030461 | Down |
| RP11-259K15.2 | 15.08865955 | 1.871462448 | 0.160318 | 11.67347 | 1.74E-31 | 7.42E-30 | Up |
| RP11-259K5.1 | 1.889980915 | -1.064844074 | 0.403821 | -2.63692 | 0.008366 | 0.019487 | Down |
| RP11-259O2.1 | 1.5825815 | -1.176486443 | 0.342006 | -3.43996 | 0.000582 | 0.001823 | Down |
| RP11-259P15.4 | 2.587214228 | -1.243208596 | 0.326428 | -3.80852 | 0.00014 | 0.000501 | Down |
| RP11-25D3.1 | 1.292496986 | -1.334233753 | 0.485626 | -2.74745 | 0.006006 | 0.014561 | Down |
| RP11-25G10.2 | 2.484554597 | -1.019917625 | 0.281538 | -3.62267 | 0.000292 | 0.000979 | Down |
| RP11-25J3.2 | 2.567770161 | -2.797332291 | 0.484433 | -5.77445 | 7.72E-09 | 5.23E-08 | Down |
| RP11-25L3.1 | 1.294651048 | -2.412387787 | 0.95473 | -2.52677 | 0.011512 | 0.025731 | Down |
| RP11-25O10.2 | 0.998124835 | -1.065494664 | 0.381939 | -2.7897 | 0.005276 | 0.013002 | Down |
| RP11-260E18.1 | 4.057014772 | -1.034779441 | 0.31127 | -3.32438 | 0.000886 | 0.002664 | Down |
| RP11-261B23.1 | 5.317666221 | -1.320295464 | 0.350416 | -3.76779 | 0.000165 | 0.000583 | Down |
| RP11-261C10.7 | 1.947555608 | -1.623719384 | 0.495319 | -3.27813 | 0.001045 | 0.00309 | Down |
| RP11-261N11.8 | 4.060053488 | -2.449535003 | 0.394675 | -6.20646 | 5.42E-10 | 4.19E-09 | Down |
| RP11-261P13.5 | 2.568888651 | -1.32993301 | 0.409935 | -3.24426 | 0.001178 | 0.003443 | Down |
| RP11-262H14.3 | 79.51502825 | -1.021692324 | 0.330833 | -3.08824 | 0.002013 | 0.005548 | Down |
| RP11-262H14.7 | 22.67307676 | -1.314075052 | 0.3348 | -3.92496 | 8.67E-05 | 0.000323 | Down |
| RP11-263K4.3 | 2.579972651 | -1.8600302 | 0.447025 | -4.16091 | 3.17E-05 | 0.000128 | Down |
| RP11-263K4.5 | 1.049156559 | -1.501043809 | 0.390442 | -3.84447 | 0.000121 | 0.000438 | Down |
| RP11-264E20.1 | 1.814963245 | -1.918646816 | 0.588941 | -3.25779 | 0.001123 | 0.003295 | Down |
| RP11-264J4.10 | 1.890630896 | -2.600816331 | 1.077529 | -2.41369 | 0.015792 | 0.033747 | Down |
| RP11-264L1.1 | 17.73705337 | -1.311951917 | 0.231532 | -5.66639 | 1.46E-08 | 9.57E-08 | Down |
| RP11-264L1.2 | 48.61978363 | -1.388689331 | 0.20056 | -6.92406 | 4.39E-12 | 4.16E-11 | Down |
| RP11-264L1.3 | 9.067965212 | -1.526606613 | 0.238912 | -6.38982 | 1.66E-10 | 1.35E-09 | Down |
| RP11-264L1.4 | 10.86425338 | -1.401016936 | 0.237089 | -5.90925 | 3.44E-09 | 2.42E-08 | Down |
| RP11-264M12.4 | 3.095328004 | -1.370045116 | 0.484081 | -2.8302 | 0.004652 | 0.011635 | Down |
| RP11-265N7.1 | 2.289361967 | -1.086883318 | 0.458666 | -2.36966 | 0.017804 | 0.037403 | Down |
| RP11-265O12.1 | 1.668061303 | -1.742814647 | 0.52175 | -3.34033 | 0.000837 | 0.002528 | Down |
| RP11-266A24.1 | 3.816276587 | 1.037815258 | 0.209031 | 4.96488 | 6.87E-07 | 3.62E-06 | Up |
| RP11-266I3.1 | 1.610530504 | -2.683354512 | 1.081949 | -2.48011 | 0.013134 | 0.028873 | Down |
| RP11-266L9.1 | 5.362164911 | -1.822967166 | 0.312579 | -5.83202 | 5.48E-09 | 3.77E-08 | Down |
| RP11-266L9.3 | 6.665994534 | -1.017189476 | 0.239861 | -4.24075 | 2.23E-05 | 9.28E-05 | Down |
| RP11-267J23.1 | 1.717066682 | -1.002544879 | 0.315781 | -3.17481 | 0.001499 | 0.004269 | Down |
| RP11-267J23.4 | 1.955003207 | -1.474578502 | 0.396187 | -3.72192 | 0.000198 | 0.000689 | Down |
| RP11-267N12.2 | 1.979485079 | -1.606142561 | 0.528846 | -3.03707 | 0.002389 | 0.006449 | Down |
| RP11-268G12.1 | 4.08770439 | -1.610747357 | 0.401564 | -4.01118 | 6.04E-05 | 0.000232 | Down |
| RP11-268G13.1 | 2.454574419 | -2.586684392 | 0.536377 | -4.82251 | 1.42E-06 | 7.14E-06 | Down |
| RP11-268P4.4 | 0.949686621 | -1.55942994 | 0.546147 | -2.85533 | 0.004299 | 0.010847 | Down |
| RP11-26C10.1 | 1.316832258 | -3.108497271 | 1.371923 | -2.2658 | 0.023464 | 0.047416 | Down |
| RP11-26J3.1 | 3.650745734 | -2.21448913 | 0.444436 | -4.98269 | 6.27E-07 | 3.33E-06 | Down |
| RP11-26L20.4 | 0.918886412 | -2.372597671 | 0.807811 | -2.93707 | 0.003313 | 0.008618 | Down |
| RP11-271K11.1 | 0.378524036 | -1.187413382 | 0.439891 | -2.69934 | 0.006948 | 0.016558 | Down |
| RP11-271K21.12 | 4.828367352 | -1.708505051 | 0.339289 | -5.03555 | 4.76E-07 | 2.57E-06 | Down |
| RP11-271M24.2 | 5.148462755 | -1.281411322 | 0.276407 | -4.63597 | 3.55E-06 | 1.69E-05 | Down |
| RP11-272K23.3 | 1.337569523 | -2.281458623 | 0.432861 | -5.27065 | 1.36E-07 | 7.90E-07 | Down |
| RP11-274B18.3 | 2.259350255 | -1.901126914 | 0.660077 | -2.88016 | 0.003975 | 0.010117 | Down |
| RP11-274B18.4 | 0.922650408 | -1.626858138 | 0.46647 | -3.48759 | 0.000487 | 0.001555 | Down |
| RP11-274B21.12 | 35.3688267 | -1.309761154 | 0.193604 | -6.76516 | 1.33E-11 | 1.20E-10 | Down |
| RP11-274B21.9 | 9.29106826 | -1.13645613 | 0.235299 | -4.82983 | 1.37E-06 | 6.90E-06 | Down |
| RP11-274H24.1 | 1.492355959 | -2.319793178 | 0.904241 | -2.56546 | 0.010304 | 0.023339 | Down |
| RP11-274J2.1 | 1.097105319 | -1.864756492 | 0.639787 | -2.91465 | 0.003561 | 0.009182 | Down |
| RP11-274M17.1 | 1.703693684 | -3.082346055 | 1.110439 | -2.77579 | 0.005507 | 0.013495 | Down |
| RP11-276H1.2 | 0.900689646 | -1.506673331 | 0.446803 | -3.37212 | 0.000746 | 0.002279 | Down |
| RP11-276H19.1 | 16.15229447 | -1.668251927 | 0.185741 | -8.98162 | 2.67E-19 | 4.71E-18 | Down |
| RP11-278H7.1 | 1.092496253 | -1.430341401 | 0.393237 | -3.63735 | 0.000275 | 0.00093 | Down |
| RP11-278I4.2 | 2.002922434 | -2.600070915 | 0.481741 | -5.39723 | 6.77E-08 | 4.08E-07 | Down |
| RP11-278J20.2 | 1.659888339 | -1.861670827 | 0.405613 | -4.58977 | 4.44E-06 | 2.08E-05 | Down |
| RP11-278J6.4 | 2.602672406 | -1.409881503 | 0.312847 | -4.50662 | 6.59E-06 | 3.00E-05 | Down |
| RP11-279O17.1 | 1.283826652 | -2.237052113 | 0.859187 | -2.60368 | 0.009223 | 0.021217 | Down |
| RP11-279O9.4 | 3.047542445 | -1.489385633 | 0.21182 | -7.03136 | 2.05E-12 | 2.01E-11 | Down |
| RP11-27N21.3 | 7.651385384 | -1.182101676 | 0.144121 | -8.20213 | 2.36E-16 | 3.29E-15 | Down |
| RP11-280O1.2 | 119.3837942 | 4.058984737 | 0.357892 | 11.34136 | 8.19E-30 | 3.09E-28 | Up |
| RP11-281P23.1 | 3.95971952 | -1.130766049 | 0.443476 | -2.54978 | 0.010779 | 0.024274 | Down |
| RP11-283G6.3 | 17.63930795 | 1.013341909 | 0.138857 | 7.297712 | 2.93E-13 | 3.11E-12 | Up |
| RP11-283G6.4 | 7.339105727 | 1.502037002 | 0.234132 | 6.415335 | 1.41E-10 | 1.15E-09 | Up |
| RP11-283G6.5 | 8.816141994 | 2.399199067 | 0.352996 | 6.796669 | 1.07E-11 | 9.78E-11 | Up |
| RP11-283I3.2 | 4.652730758 | -1.209957298 | 0.211438 | -5.72252 | 1.05E-08 | 7.02E-08 | Down |
| RP11-284F21.11 | 0.64134449 | -1.427876342 | 0.621148 | -2.29877 | 0.021518 | 0.044035 | Down |
| RP11-284F21.7 | 1.847846456 | -1.480915254 | 0.584883 | -2.53199 | 0.011342 | 0.025393 | Down |
| RP11-284F21.8 | 3.353198169 | -1.87564986 | 0.396103 | -4.73526 | 2.19E-06 | 1.07E-05 | Down |
| RP11-285E23.2 | 7.65174546 | 1.230444248 | 0.20882 | 5.892363 | 3.81E-09 | 2.67E-08 | Up |
| RP11-286E11.2 | 8.268087847 | -1.396136462 | 0.250306 | -5.57771 | 2.44E-08 | 1.56E-07 | Down |
| RP11-286H15.1 | 9.841702332 | -1.331074707 | 0.19312 | -6.89247 | 5.48E-12 | 5.17E-11 | Down |
| RP11-286N22.16 | 4.36016305 | -2.014531556 | 0.353957 | -5.69146 | 1.26E-08 | 8.33E-08 | Down |
| RP11-288A5.2 | 7.228074542 | 1.089687351 | 0.37459 | 2.909015 | 0.003626 | 0.009331 | Up |
| RP11-288D15.1 | 1.497828237 | -1.843455075 | 0.525212 | -3.50992 | 0.000448 | 0.001443 | Down |
| RP11-288K12.1 | 23.71383811 | -1.182880311 | 0.213867 | -5.53092 | 3.19E-08 | 2.00E-07 | Down |
| RP11-28F1.2 | 23.98535802 | -1.119474966 | 0.182186 | -6.14467 | 8.01E-10 | 6.09E-09 | Down |
| RP11-290F5.2 | 4.250126251 | -1.523822857 | 0.391284 | -3.89442 | 9.84E-05 | 0.000363 | Down |
| RP11-290H9.5 | 9.662560168 | -1.049972836 | 0.202848 | -5.17615 | 2.27E-07 | 1.28E-06 | Down |
| RP11-290L1.2 | 2.152032414 | -1.155566506 | 0.289008 | -3.99839 | 6.38E-05 | 0.000243 | Down |
| RP11-290L7.3 | 2.621845154 | -1.943151686 | 0.452609 | -4.29322 | 1.76E-05 | 7.47E-05 | Down |
| RP11-290L7.5 | 2.580341639 | -2.864827414 | 0.398532 | -7.18844 | 6.55E-13 | 6.72E-12 | Down |
| RP11-290M5.4 | 0.708217797 | -1.173743164 | 0.417154 | -2.81369 | 0.004898 | 0.012162 | Down |
| RP11-291L22.7 | 0.698012173 | -1.159265127 | 0.449221 | -2.58061 | 0.009863 | 0.022485 | Down |
| RP11-291L22.8 | 2.754186579 | -1.808497722 | 0.401954 | -4.49927 | 6.82E-06 | 3.10E-05 | Down |
| RP11-292B8.1 | 6.749915083 | -2.562769867 | 0.365988 | -7.00233 | 2.52E-12 | 2.45E-11 | Down |
| RP11-292B8.2 | 0.896933041 | -1.353890373 | 0.428262 | -3.16136 | 0.00157 | 0.004451 | Down |
| RP11-292E2.2 | 1.031967011 | -1.645109225 | 0.638394 | -2.57695 | 0.009968 | 0.022691 | Down |
| RP11-293A21.1 | 7.870318149 | -1.373993423 | 0.167671 | -8.19456 | 2.52E-16 | 3.49E-15 | Down |
| RP11-293B7.1 | 1.328467805 | -1.850619658 | 0.784515 | -2.35893 | 0.018327 | 0.038359 | Down |
| RP11-293D9.2 | 1.138255783 | -3.405798321 | 1.357622 | -2.50865 | 0.012119 | 0.026935 | Down |
| RP11-294N21.3 | 1.367586836 | -1.47297028 | 0.337431 | -4.36525 | 1.27E-05 | 5.52E-05 | Down |
| RP11-295G24.5 | 3.07094166 | -1.104300865 | 0.24773 | -4.45768 | 8.29E-06 | 3.71E-05 | Down |
| RP11-295K3.1 | 12.99391076 | 1.2724092 | 0.209189 | 6.082596 | 1.18E-09 | 8.81E-09 | Up |
| RP11-295M18.6 | 4.158179651 | -1.089614921 | 0.225826 | -4.82502 | 1.40E-06 | 7.06E-06 | Down |
| RP11-296E3.2 | 1.302500726 | -1.179445743 | 0.435089 | -2.71082 | 0.006712 | 0.016068 | Down |
| RP11-296E7.1 | 20.65342148 | -1.664947829 | 0.21855 | -7.61816 | 2.57E-14 | 3.03E-13 | Down |
| RP11-297A16.2 | 1.668896006 | -2.264197818 | 0.584914 | -3.87099 | 0.000108 | 0.000396 | Down |
| RP11-297B17.3 | 2.542808103 | -1.766948857 | 0.657814 | -2.68609 | 0.007229 | 0.017134 | Down |
| RP11-297C4.3 | 2.440881898 | -1.303425101 | 0.427093 | -3.05185 | 0.002274 | 0.00617 | Down |
| RP11-297D21.2 | 1.426239902 | -2.202047392 | 0.392629 | -5.60846 | 2.04E-08 | 1.32E-07 | Down |
| RP11-298C2.1 | 2.911177797 | -1.011778602 | 0.31802 | -3.18149 | 0.001465 | 0.004183 | Down |
| RP11-298D21.1 | 1.600189794 | -1.928888709 | 0.592983 | -3.25286 | 0.001143 | 0.003348 | Down |
| RP11-298E2.2 | 2.035828607 | -1.485719414 | 0.302809 | -4.90646 | 9.27E-07 | 4.80E-06 | Down |
| RP11-298H24.1 | 1.560731958 | -3.275310051 | 1.284132 | -2.5506 | 0.010754 | 0.024227 | Down |
| RP11-299G20.2 | 16.96638964 | -1.262444625 | 0.175388 | -7.198 | 6.11E-13 | 6.29E-12 | Down |
| RP11-299G20.5 | 9.943541793 | -1.240181742 | 0.179092 | -6.92482 | 4.37E-12 | 4.14E-11 | Down |
| RP11-299H21.1 | 38.28075256 | -1.443593581 | 0.098772 | -14.6154 | 2.24E-48 | 2.60E-46 | Down |
| RP11-299H22.3 | 3.243428709 | -2.693558106 | 0.653627 | -4.12094 | 3.77E-05 | 0.000151 | Down |
| RP11-299L17.1 | 1.748040526 | -1.643465596 | 0.311227 | -5.28059 | 1.29E-07 | 7.50E-07 | Down |
| RP11-299L17.3 | 3.854189059 | -2.328304981 | 0.26342 | -8.83875 | 9.68E-19 | 1.64E-17 | Down |
| RP11-299P2.1 | 0.923137765 | -1.217265913 | 0.413735 | -2.94214 | 0.00326 | 0.008489 | Down |
| RP11-29B11.5 | 2.371170585 | -1.651565397 | 0.459254 | -3.59619 | 0.000323 | 0.001074 | Down |
| RP11-29P20.1 | 2.240987445 | -2.312741689 | 0.739093 | -3.12916 | 0.001753 | 0.004912 | Down |
| RP11-2B6.3 | 1.381784134 | 1.038577392 | 0.320109 | 3.244444 | 0.001177 | 0.003441 | Up |
| RP11-2C24.5 | 1.689479974 | -1.770324796 | 0.467793 | -3.78442 | 0.000154 | 0.000548 | Down |
| RP11-2E11.5 | 5.126677313 | -1.81279709 | 0.351633 | -5.15537 | 2.53E-07 | 1.42E-06 | Down |
| RP11-2E17.2 | 0.816121549 | -1.128794739 | 0.381221 | -2.961 | 0.003066 | 0.008044 | Down |
| RP11-2I17.1 | 3.106401874 | -1.23078785 | 0.307239 | -4.00596 | 6.18E-05 | 0.000236 | Down |
| RP11-2I17.4 | 0.579768884 | -1.715835243 | 0.486104 | -3.52977 | 0.000416 | 0.001348 | Down |
| RP11-2N1.2 | 2.011940418 | -3.434599932 | 0.57237 | -6.00066 | 1.97E-09 | 1.43E-08 | Down |
| RP11-2O17.2 | 1.275847264 | -1.293495054 | 0.502061 | -2.57637 | 0.009984 | 0.022726 | Down |
| RP11-300E4.2 | 0.855077142 | -1.148020622 | 0.472313 | -2.43063 | 0.015072 | 0.032449 | Down |
| RP11-300G22.2 | 1.251714572 | -1.265100865 | 0.414201 | -3.05432 | 0.002256 | 0.006126 | Down |
| RP11-301G19.2 | 1.409005311 | -3.14312621 | 1.380165 | -2.27736 | 0.022765 | 0.046212 | Down |
| RP11-301G7.1 | 6.452225394 | -2.5342808 | 0.275378 | -9.20293 | 3.48E-20 | 6.59E-19 | Down |
| RP11-301L8.2 | 56.87589465 | -1.966347932 | 0.199774 | -9.84286 | 7.36E-23 | 1.69E-21 | Down |
| RP11-302F12.1 | 1.348917418 | 3.470486705 | 0.386512 | 8.978988 | 2.73E-19 | 4.82E-18 | Up |
| RP11-302F12.5 | 1.966347044 | -1.622265546 | 0.592726 | -2.73696 | 0.006201 | 0.014976 | Down |
| RP11-302M6.5 | 2.662745924 | -1.27142646 | 0.283148 | -4.49033 | 7.11E-06 | 3.22E-05 | Down |
| RP11-303E16.7 | 8.557404117 | -1.580530428 | 0.163822 | -9.64786 | 5.02E-22 | 1.08E-20 | Down |
| RP11-303E16.8 | 36.76925929 | -1.203024561 | 0.103368 | -11.6383 | 2.63E-31 | 1.10E-29 | Down |
| RP11-304C12.3 | 2.928624011 | -1.613186893 | 0.423797 | -3.80651 | 0.000141 | 0.000504 | Down |
| RP11-304F15.4 | 2.815193641 | -1.338388989 | 0.492027 | -2.72016 | 0.006525 | 0.015677 | Down |
| RP11-304L19.1 | 4.583036598 | -1.164442744 | 0.196908 | -5.91365 | 3.35E-09 | 2.36E-08 | Down |
| RP11-304L19.3 | 6.468055633 | -1.025853574 | 0.183493 | -5.59068 | 2.26E-08 | 1.45E-07 | Down |
| RP11-305E17.4 | 0.720870151 | -1.346922537 | 0.500012 | -2.69378 | 0.007065 | 0.016801 | Down |
| RP11-305F18.1 | 0.769443245 | -2.25858882 | 0.431667 | -5.23224 | 1.67E-07 | 9.61E-07 | Down |
| RP11-305O6.3 | 20.92333681 | -1.451306216 | 0.227776 | -6.37164 | 1.87E-10 | 1.52E-09 | Down |
| RP11-305P14.1 | 0.416865698 | -1.35701648 | 0.509884 | -2.66142 | 0.007781 | 0.018277 | Down |
| RP11-306O13.1 | 6.024034253 | -2.056678807 | 0.289905 | -7.09433 | 1.30E-12 | 1.30E-11 | Down |
| RP11-307B6.3 | 3.740010029 | -1.212186552 | 0.252766 | -4.79568 | 1.62E-06 | 8.10E-06 | Down |
| RP11-307C12.13 | 5.607316637 | -1.582192853 | 0.161982 | -9.76772 | 1.55E-22 | 3.48E-21 | Down |
| RP11-307O13.1 | 1.492552976 | -1.325468025 | 0.580324 | -2.28401 | 0.022371 | 0.045523 | Down |
| RP11-307P5.1 | 1.063502825 | -1.974480932 | 0.608896 | -3.24272 | 0.001184 | 0.00346 | Down |
| RP11-309L24.4 | 49.4843312 | 1.212719406 | 0.178773 | 6.78356 | 1.17E-11 | 1.07E-10 | Up |
| RP11-30B1.1 | 6.280582148 | 1.265527523 | 0.308437 | 4.103035 | 4.08E-05 | 0.000162 | Up |
| RP11-30P6.1 | 0.909861623 | -1.699094734 | 0.721537 | -2.35483 | 0.018531 | 0.038722 | Down |
| RP11-310H4.2 | 1.516872465 | -2.241580757 | 0.811731 | -2.76148 | 0.005754 | 0.014024 | Down |
| RP11-310N16.1 | 1.333605681 | -2.427730961 | 0.673255 | -3.60596 | 0.000311 | 0.001038 | Down |
| RP11-310P5.1 | 1.091972203 | -1.741813851 | 0.689973 | -2.52447 | 0.011587 | 0.025889 | Down |
| RP11-312J18.6 | 1.485987308 | -1.337441211 | 0.373957 | -3.57646 | 0.000348 | 0.00115 | Down |
| RP11-313A24.1 | 3.147014376 | -1.066080991 | 0.359485 | -2.96558 | 0.003021 | 0.007937 | Down |
| RP11-313C15.1 | 1.674164053 | -2.913660405 | 0.942709 | -3.09073 | 0.001997 | 0.005509 | Down |
| RP11-313F23.4 | 2.420522756 | -1.309261535 | 0.30299 | -4.32113 | 1.55E-05 | 6.64E-05 | Down |
| RP11-313I2.7 | 1.367301602 | -3.249554309 | 1.250842 | -2.59789 | 0.00938 | 0.021536 | Down |
| RP11-314C9.2 | 0.61410815 | 1.003864868 | 0.424045 | 2.367356 | 0.017916 | 0.037623 | Up |
| RP11-314N2.2 | 1.443686432 | -2.965479719 | 0.75141 | -3.94656 | 7.93E-05 | 0.000297 | Down |
| RP11-314P15.1 | 2.940026923 | -2.513280822 | 0.532151 | -4.72287 | 2.33E-06 | 1.14E-05 | Down |
| RP11-315I20.1 | 6.590442677 | -1.122326107 | 0.217613 | -5.15743 | 2.50E-07 | 1.40E-06 | Down |
| RP11-315O6.1 | 9.455045491 | -1.673583181 | 0.163096 | -10.2613 | 1.05E-24 | 2.75E-23 | Down |
| RP11-316M20.1 | 1.818154642 | -2.3535065 | 0.685303 | -3.43426 | 0.000594 | 0.001858 | Down |
| RP11-316N24.2 | 1.307108597 | -2.328531038 | 0.663149 | -3.51133 | 0.000446 | 0.001436 | Down |
| RP11-316O14.1 | 4.33672481 | -1.217759573 | 0.209467 | -5.81362 | 6.11E-09 | 4.18E-08 | Down |
| RP11-317B3.2 | 12.93606231 | -2.161809486 | 0.289403 | -7.4699 | 8.03E-14 | 9.02E-13 | Down |
| RP11-317F20.2 | 3.624522261 | -1.274543751 | 0.298613 | -4.26822 | 1.97E-05 | 8.28E-05 | Down |
| RP11-317F20.3 | 8.091077283 | -1.989987243 | 0.292056 | -6.81372 | 9.51E-12 | 8.73E-11 | Down |
| RP11-317L10.1 | 0.543649034 | -1.805893792 | 0.4965 | -3.63725 | 0.000276 | 0.000931 | Down |
| RP11-317M11.1 | 1.767036075 | -2.289521827 | 0.587138 | -3.89946 | 9.64E-05 | 0.000356 | Down |
| RP11-317N12.1 | 1.304252884 | -3.174038204 | 1.316724 | -2.41056 | 0.015928 | 0.03399 | Down |
| RP11-317N8.5 | 14.46344845 | -1.177276261 | 0.185932 | -6.33176 | 2.42E-10 | 1.94E-09 | Down |
| RP11-317P15.4 | 8.62958771 | -1.017249644 | 0.19969 | -5.09415 | 3.50E-07 | 1.92E-06 | Down |
| RP11-319F12.2 | 10.48023766 | -1.335317457 | 0.485574 | -2.74998 | 0.00596 | 0.014468 | Down |
| RP11-319G9.1 | 26.54684593 | -1.33662423 | 0.213684 | -6.25514 | 3.97E-10 | 3.11E-09 | Down |
| RP11-319G9.3 | 47.14688386 | -1.500579452 | 0.210769 | -7.11955 | 1.08E-12 | 1.09E-11 | Down |
| RP11-319G9.5 | 5.326196879 | -1.683726285 | 0.26108 | -6.44909 | 1.13E-10 | 9.33E-10 | Down |
| RP11-320G24.1 | 1.582343558 | -1.850652402 | 0.590522 | -3.13392 | 0.001725 | 0.004842 | Down |
| RP11-320N7.2 | 3.934721662 | 2.890928832 | 0.28385 | 10.18471 | 2.32E-24 | 5.90E-23 | Up |
| RP11-320P11.1 | 7.36704708 | -1.059162199 | 0.248867 | -4.25594 | 2.08E-05 | 8.71E-05 | Down |
| RP11-322E11.2 | 6.24931257 | -1.848352969 | 0.292584 | -6.31735 | 2.66E-10 | 2.12E-09 | Down |
| RP11-322E11.5 | 20.53940957 | -2.117835208 | 0.191275 | -11.0722 | 1.71E-28 | 5.93E-27 | Down |
| RP11-324L17.1 | 1.714198811 | -1.555824428 | 0.421751 | -3.68897 | 0.000225 | 0.000775 | Down |
| RP11-325I22.3 | 44.03405601 | -1.600732197 | 0.135267 | -11.8339 | 2.61E-32 | 1.16E-30 | Down |
| RP11-325L12.7 | 1.519138388 | -1.586320733 | 0.342855 | -4.62679 | 3.71E-06 | 1.76E-05 | Down |
| RP11-325N19.3 | 4.50718433 | -1.584595778 | 0.310883 | -5.09707 | 3.45E-07 | 1.90E-06 | Down |
| RP11-325P15.2 | 1.95709699 | -2.448568943 | 0.6825 | -3.58765 | 0.000334 | 0.001106 | Down |
| RP11-326A19.3 | 0.867248353 | -1.543317987 | 0.41999 | -3.67465 | 0.000238 | 0.000815 | Down |
| RP11-326C3.11 | 21.44080857 | -1.132864164 | 0.172711 | -6.55929 | 5.41E-11 | 4.61E-10 | Down |
| RP11-326C3.12 | 1.860062815 | -1.283629818 | 0.372492 | -3.44606 | 0.000569 | 0.001786 | Down |
| RP11-326C3.13 | 1.290625526 | -1.121063061 | 0.318188 | -3.52328 | 0.000426 | 0.001378 | Down |
| RP11-326I11.4 | 9.225962115 | -1.078585323 | 0.196468 | -5.48987 | 4.02E-08 | 2.50E-07 | Down |
| RP11-326L17.1 | 1.148678025 | -1.769728643 | 0.711783 | -2.48633 | 0.012907 | 0.028456 | Down |
| RP11-327I22.5 | 1.765373489 | -1.734306869 | 0.767727 | -2.25902 | 0.023882 | 0.048158 | Down |
| RP11-327J17.1 | 0.675710137 | -1.041635091 | 0.43457 | -2.39693 | 0.016533 | 0.035113 | Down |
| RP11-327J17.2 | 2.724894031 | -1.027646219 | 0.275183 | -3.7344 | 0.000188 | 0.000658 | Down |
| RP11-327J17.7 | 7.450769241 | -1.846220297 | 0.297524 | -6.20528 | 5.46E-10 | 4.22E-09 | Down |
| RP11-327J17.9 | 7.419490579 | -1.120956485 | 0.206188 | -5.43657 | 5.43E-08 | 3.31E-07 | Down |
| RP11-327O17.2 | 2.626308799 | -1.443960888 | 0.343688 | -4.20137 | 2.65E-05 | 0.000109 | Down |
| RP11-327P2.5 | 88.94857399 | -1.083290533 | 0.080108 | -13.5229 | 1.15E-41 | 8.91E-40 | Down |
| RP11-328C8.5 | 1.336336554 | 1.630185919 | 0.452801 | 3.600223 | 0.000318 | 0.001059 | Up |
| RP11-328D5.1 | 0.487962534 | -1.290390585 | 0.475995 | -2.71093 | 0.006709 | 0.016064 | Down |
| RP11-328J2.1 | 1.742273018 | -3.049084944 | 1.212203 | -2.51532 | 0.011892 | 0.026489 | Down |
| RP11-328N19.1 | 5.511339154 | -1.664730826 | 0.343165 | -4.85111 | 1.23E-06 | 6.25E-06 | Down |
| RP11-329B9.3 | 4.273481631 | 1.039971126 | 0.228551 | 4.550286 | 5.36E-06 | 2.47E-05 | Up |
| RP11-329B9.4 | 10.64961496 | 1.419592351 | 0.131534 | 10.79257 | 3.73E-27 | 1.17E-25 | Up |
| RP11-32B5.1 | 21.9828745 | -1.280500623 | 0.207892 | -6.15945 | 7.30E-10 | 5.57E-09 | Down |
| RP11-32B5.7 | 27.29840393 | -1.460475504 | 0.247875 | -5.89199 | 3.82E-09 | 2.67E-08 | Down |
| RP11-330H6.6 | 9.07605363 | -1.294593856 | 0.28092 | -4.60841 | 4.06E-06 | 1.91E-05 | Down |
| RP11-330M19.1 | 4.377857433 | -1.261493844 | 0.353172 | -3.5719 | 0.000354 | 0.001168 | Down |
| RP11-331D5.2 | 2.48517781 | -1.908744443 | 0.635559 | -3.00325 | 0.002671 | 0.007104 | Down |
| RP11-332H18.3 | 4.850310965 | -1.100257694 | 0.233104 | -4.72002 | 2.36E-06 | 1.15E-05 | Down |
| RP11-332H18.5 | 1.424746433 | -1.383075175 | 0.392794 | -3.52112 | 0.00043 | 0.001388 | Down |
| RP11-333A23.4 | 2.346203552 | -1.865538227 | 0.517972 | -3.60162 | 0.000316 | 0.001054 | Down |
| RP11-333E13.4 | 6.849608243 | -1.255051358 | 0.173697 | -7.22554 | 4.99E-13 | 5.18E-12 | Down |
| RP11-334A14.8 | 2.016524179 | -2.10761511 | 0.490589 | -4.29609 | 1.74E-05 | 7.38E-05 | Down |
| RP11-334E6.2 | 1.959087114 | -1.847310389 | 0.411756 | -4.48641 | 7.24E-06 | 3.27E-05 | Down |
| RP11-334G8.1 | 1.043682895 | -2.158317587 | 0.550635 | -3.91969 | 8.87E-05 | 0.000329 | Down |
| RP11-334L9.1 | 2.086797087 | -1.346564948 | 0.437878 | -3.07521 | 0.002104 | 0.005765 | Down |
| RP11-335E14.1 | 3.278594612 | -1.236515828 | 0.288071 | -4.2924 | 1.77E-05 | 7.49E-05 | Down |
| RP11-335F8.2 | 1.043393492 | -2.318272688 | 0.977888 | -2.37069 | 0.017755 | 0.03731 | Down |
| RP11-335K5.2 | 1.709317891 | -2.255440325 | 0.786453 | -2.86787 | 0.004133 | 0.010469 | Down |
| RP11-335L23.2 | 1.95887821 | -3.413385545 | 1.040002 | -3.28209 | 0.00103 | 0.003053 | Down |
| RP11-335L23.4 | 1.266150272 | -2.224141178 | 0.978917 | -2.27204 | 0.023084 | 0.046748 | Down |
| RP11-335L23.5 | 4.831247127 | -1.606014009 | 0.249238 | -6.4437 | 1.17E-10 | 9.65E-10 | Down |
| RP11-336N8.2 | 1.926186059 | -1.434780736 | 0.474305 | -3.02501 | 0.002486 | 0.006675 | Down |
| RP11-337N6.2 | 0.719267046 | -1.017780694 | 0.442851 | -2.29825 | 0.021548 | 0.044085 | Down |
| RP11-338C15.5 | 2.140731842 | -1.321937718 | 0.500053 | -2.64359 | 0.008203 | 0.019151 | Down |
| RP11-338K13.1 | 1.925724544 | -1.727351936 | 0.460598 | -3.75024 | 0.000177 | 0.000622 | Down |
| RP11-339B21.8 | 3.302012241 | -1.806291463 | 0.395195 | -4.57063 | 4.86E-06 | 2.26E-05 | Down |
| RP11-339F13.2 | 1.164061845 | -2.223320052 | 0.90133 | -2.46671 | 0.013636 | 0.029798 | Down |
| RP11-33A14.1 | 3.638421864 | -1.747054732 | 0.330713 | -5.2827 | 1.27E-07 | 7.42E-07 | Down |
| RP11-33A14.3 | 0.98869751 | -2.302705618 | 0.515437 | -4.46748 | 7.91E-06 | 3.56E-05 | Down |
| RP11-340E6.1 | 0.990181812 | -1.580257868 | 0.354742 | -4.45467 | 8.40E-06 | 3.76E-05 | Down |
| RP11-340I6.7 | 1.490523986 | -2.316090994 | 0.842222 | -2.74998 | 0.00596 | 0.014468 | Down |
| RP11-340I6.8 | 1.993827871 | -1.163523994 | 0.234362 | -4.96464 | 6.88E-07 | 3.63E-06 | Down |
| RP11-341D18.2 | 1.426234679 | -1.840870989 | 0.790003 | -2.33021 | 0.019795 | 0.040981 | Down |
| RP11-341G23.2 | 0.562777495 | -1.81068441 | 0.557031 | -3.2506 | 0.001152 | 0.003372 | Down |
| RP11-341G5.2 | 1.264148127 | -2.730955889 | 1.17069 | -2.33277 | 0.01966 | 0.040747 | Down |
| RP11-342D14.1 | 0.957849807 | -1.375970928 | 0.505235 | -2.72343 | 0.006461 | 0.015542 | Down |
| RP11-342F17.1 | 8.765134849 | -1.740550099 | 0.310788 | -5.60044 | 2.14E-08 | 1.38E-07 | Down |
| RP11-342K6.4 | 5.558768779 | -1.178898449 | 0.211025 | -5.58654 | 2.32E-08 | 1.48E-07 | Down |
| RP11-342M1.7 | 1.652244031 | -2.826182282 | 0.780368 | -3.6216 | 0.000293 | 0.000982 | Down |
| RP11-343D24.1 | 1.114683713 | -2.001424925 | 0.754411 | -2.65297 | 0.007979 | 0.018685 | Down |
| RP11-343H19.1 | 5.422338648 | -1.201591429 | 0.222104 | -5.41005 | 6.30E-08 | 3.81E-07 | Down |
| RP11-343H5.6 | 0.487281097 | -1.533899468 | 0.389965 | -3.93342 | 8.37E-05 | 0.000313 | Down |
| RP11-344A16.2 | 1.855707664 | -1.038480235 | 0.41965 | -2.47464 | 0.013337 | 0.029265 | Down |
| RP11-344B23.2 | 1.545334778 | -2.094415203 | 0.720947 | -2.90509 | 0.003672 | 0.009434 | Down |
| RP11-344E13.4 | 1.592733047 | -2.429496685 | 0.92616 | -2.62319 | 0.008711 | 0.020196 | Down |
| RP11-345K9.3 | 1.478309013 | -2.799834437 | 1.241532 | -2.25514 | 0.024124 | 0.048598 | Down |
| RP11-346I3.2 | 1.511348628 | -2.41511644 | 0.606978 | -3.97892 | 6.92E-05 | 0.000263 | Down |
| RP11-348J12.2 | 0.722111689 | -1.137821438 | 0.377254 | -3.01606 | 0.002561 | 0.006847 | Down |
| RP11-348J24.2 | 1.771554307 | -2.877203197 | 0.653054 | -4.40577 | 1.05E-05 | 4.64E-05 | Down |
| RP11-34F13.2 | 2.43437583 | -1.774027702 | 0.478952 | -3.70398 | 0.000212 | 0.000734 | Down |
| RP11-34P1.1 | 1.285707695 | -1.413810721 | 0.364932 | -3.87417 | 0.000107 | 0.000391 | Down |
| RP11-350A1.1 | 9.616863617 | -1.773228481 | 0.438549 | -4.04339 | 5.27E-05 | 0.000205 | Down |
| RP11-350F16.1 | 0.690539768 | -1.104698651 | 0.435132 | -2.53877 | 0.011124 | 0.024959 | Down |
| RP11-350G24.2 | 1.860319779 | -1.319872639 | 0.424731 | -3.10755 | 0.001886 | 0.005242 | Down |
| RP11-350J20.12 | 4.550445143 | 2.495442586 | 0.246968 | 10.10432 | 5.29E-24 | 1.32E-22 | Up |
| RP11-350N15.4 | 3.292250767 | -1.391001814 | 0.323403 | -4.30114 | 1.70E-05 | 7.22E-05 | Down |
| RP11-351J23.1 | 3.745320166 | -1.075563422 | 0.346357 | -3.10536 | 0.0019 | 0.005274 | Down |
| RP11-351J23.2 | 9.69032854 | -2.783302243 | 0.298636 | -9.32004 | 1.16E-20 | 2.27E-19 | Down |
| RP11-353N14.2 | 6.406129881 | 2.243930257 | 0.27373 | 8.197595 | 2.45E-16 | 3.41E-15 | Up |
| RP11-354K4.1 | 3.839671522 | -1.257521818 | 0.473665 | -2.65487 | 0.007934 | 0.018593 | Down |
| RP11-355N15.1 | 4.389372875 | -2.137320124 | 0.40585 | -5.26629 | 1.39E-07 | 8.08E-07 | Down |
| RP11-355N15.3 | 1.841350951 | -1.402084387 | 0.41805 | -3.35387 | 0.000797 | 0.002417 | Down |
| RP11-356M20.1 | 1.505974543 | -1.05360243 | 0.457325 | -2.30384 | 0.021232 | 0.043523 | Down |
| RP11-356M20.2 | 1.276228408 | -1.601328117 | 0.655665 | -2.4423 | 0.014594 | 0.031575 | Down |
| RP11-356O9.1 | 1.326635985 | -1.192151473 | 0.418434 | -2.84908 | 0.004385 | 0.011041 | Down |
| RP11-357H14.17 | 2.226683345 | -2.120671506 | 0.698999 | -3.03387 | 0.002414 | 0.006507 | Down |
| RP11-358M14.2 | 2.62783801 | -1.547620794 | 0.258714 | -5.98199 | 2.20E-09 | 1.59E-08 | Down |
| RP11-358N2.2 | 4.996273247 | -1.037722015 | 0.195548 | -5.30674 | 1.12E-07 | 6.54E-07 | Down |
| RP11-359E3.4 | 95.31688227 | -1.266268507 | 0.10569 | -11.9809 | 4.47E-33 | 2.07E-31 | Down |
| RP11-359H18.2 | 1.571661454 | -1.936695361 | 0.650595 | -2.97681 | 0.002913 | 0.007681 | Down |
| RP11-359K18.3 | 2.028365287 | -1.802184979 | 0.335781 | -5.36714 | 8.00E-08 | 4.78E-07 | Down |
| RP11-359N11.1 | 2.368718337 | -2.067715567 | 0.296182 | -6.98124 | 2.93E-12 | 2.82E-11 | Down |
| RP11-35J10.4 | 1.029401044 | -1.938231949 | 0.514091 | -3.77021 | 0.000163 | 0.000577 | Down |
| RP11-35P15.1 | 0.919921885 | -1.773368735 | 0.501436 | -3.53658 | 0.000405 | 0.001317 | Down |
| RP11-360A10.1 | 2.975784917 | -1.483471859 | 0.403504 | -3.67648 | 0.000236 | 0.00081 | Down |
| RP11-360K13.1 | 1.545897751 | -2.746972897 | 1.077153 | -2.55022 | 0.010766 | 0.02425 | Down |
| RP11-360N9.2 | 8.679660915 | -1.014969297 | 0.304629 | -3.33182 | 0.000863 | 0.0026 | Down |
| RP11-360O19.4 | 2.444319364 | -1.077735325 | 0.30747 | -3.50517 | 0.000456 | 0.001465 | Down |
| RP11-361D14.2 | 2.312926674 | -2.580506962 | 1.056402 | -2.44273 | 0.014577 | 0.031541 | Down |
| RP11-361M10.3 | 1.574070563 | -1.194135639 | 0.496115 | -2.40697 | 0.016085 | 0.034276 | Down |
| RP11-362F19.1 | 51.22377086 | -1.234145128 | 0.168498 | -7.32439 | 2.40E-13 | 2.57E-12 | Down |
| RP11-362F19.2 | 20.72956536 | -1.384858475 | 0.24106 | -5.74486 | 9.20E-09 | 6.19E-08 | Down |
| RP11-362K2.2 | 2.239675235 | -2.967658399 | 0.739523 | -4.01293 | 6.00E-05 | 0.00023 | Down |
| RP11-363D14.1 | 0.67212047 | -1.227808606 | 0.383887 | -3.19836 | 0.001382 | 0.00397 | Down |
| RP11-363E6.4 | 4.289848173 | -1.93737647 | 0.344368 | -5.62589 | 1.85E-08 | 1.20E-07 | Down |
| RP11-363E7.4 | 130.9061067 | 1.165044607 | 0.085188 | 13.67615 | 1.41E-42 | 1.16E-40 | Up |
| RP11-363G2.4 | 1.802488829 | -2.284743982 | 0.691552 | -3.30379 | 0.000954 | 0.002847 | Down |
| RP11-363J20.1 | 5.010259587 | -1.752147302 | 0.289125 | -6.06017 | 1.36E-09 | 1.01E-08 | Down |
| RP11-363L24.3 | 1.155271341 | -1.160796019 | 0.416138 | -2.78945 | 0.00528 | 0.013011 | Down |
| RP11-364B14.3 | 1.108449387 | -1.45668228 | 0.400002 | -3.64169 | 0.000271 | 0.000916 | Down |
| RP11-364P2.2 | 1.261204696 | -1.926101715 | 0.645098 | -2.98575 | 0.002829 | 0.007481 | Down |
| RP11-364P22.1 | 1.453794486 | -1.353617906 | 0.471146 | -2.87303 | 0.004066 | 0.010318 | Down |
| RP11-365F18.6 | 3.361606964 | -1.013090099 | 0.272943 | -3.71173 | 0.000206 | 0.000714 | Down |
| RP11-365H23.1 | 1.166501174 | -1.782479416 | 0.61545 | -2.89622 | 0.003777 | 0.009675 | Down |
| RP11-366L20.2 | 297.1526054 | 2.604682233 | 0.203015 | 12.83001 | 1.11E-37 | 6.74E-36 | Up |
| RP11-366L20.3 | 3.420804678 | 3.932105728 | 0.411245 | 9.561475 | 1.16E-21 | 2.44E-20 | Up |
| RP11-366L5.1 | 18.44199396 | -1.051575228 | 0.170278 | -6.17565 | 6.59E-10 | 5.04E-09 | Down |
| RP11-367F23.1 | 0.964136653 | -1.643353176 | 0.340236 | -4.83003 | 1.37E-06 | 6.90E-06 | Down |
| RP11-367J11.2 | 17.51643964 | -1.062522273 | 0.222423 | -4.77703 | 1.78E-06 | 8.84E-06 | Down |
| RP11-367O10.1 | 0.623798948 | -1.064168389 | 0.416448 | -2.55534 | 0.010608 | 0.023949 | Down |
| RP11-368I23.2 | 3.318871505 | 3.226383482 | 0.349304 | 9.236606 | 2.54E-20 | 4.85E-19 | Up |
| RP11-368I23.3 | 2.001295649 | 2.579307534 | 0.511319 | 5.044422 | 4.55E-07 | 2.46E-06 | Up |
| RP11-368I23.4 | 0.888324402 | 2.105439495 | 0.554226 | 3.79888 | 0.000145 | 0.000519 | Up |
| RP11-368J22.2 | 3.969418042 | -1.415358887 | 0.357799 | -3.95573 | 7.63E-05 | 0.000287 | Down |
| RP11-368M16.10 | 2.410250535 | -2.400621969 | 0.942329 | -2.54754 | 0.010849 | 0.024419 | Down |
| RP11-369K16.1 | 25.4860615 | -1.424928017 | 0.185787 | -7.66967 | 1.72E-14 | 2.07E-13 | Down |
| RP11-36B6.1 | 1.732442395 | -1.607897378 | 0.617667 | -2.60318 | 0.009236 | 0.021243 | Down |
| RP11-374M1.10 | 1.012229097 | -1.724733522 | 0.684205 | -2.52079 | 0.011709 | 0.026133 | Down |
| RP11-374M1.11 | 0.588505056 | -1.303401989 | 0.527857 | -2.46923 | 0.01354 | 0.02963 | Down |
| RP11-374M1.2 | 7.387014166 | -1.367120322 | 0.284399 | -4.80705 | 1.53E-06 | 7.67E-06 | Down |
| RP11-375I20.6 | 10.35194049 | -1.403808621 | 0.212926 | -6.59295 | 4.31E-11 | 3.71E-10 | Down |
| RP11-377D9.3 | 5.514582791 | -1.921463998 | 0.296892 | -6.47194 | 9.68E-11 | 8.07E-10 | Down |
| RP11-378A12.1 | 2.050995544 | -2.734887021 | 0.580336 | -4.71259 | 2.45E-06 | 1.19E-05 | Down |
| RP11-378E13.4 | 0.576524873 | -1.303923255 | 0.475556 | -2.74189 | 0.006109 | 0.01478 | Down |
| RP11-378I6.2 | 1.353330496 | -3.006151559 | 1.15955 | -2.59252 | 0.009528 | 0.021837 | Down |
| RP11-379K17.12 | 3.659396994 | -5.102456612 | 1.402228 | -3.63882 | 0.000274 | 0.000925 | Down |
| RP11-379K17.2 | 1.242605199 | -1.910450079 | 0.76007 | -2.51352 | 0.011953 | 0.026611 | Down |
| RP11-379K17.4 | 13.81318597 | -1.54007811 | 0.267335 | -5.76086 | 8.37E-09 | 5.65E-08 | Down |
| RP11-379K22.2 | 9.558604578 | -1.177604751 | 0.292576 | -4.02495 | 5.70E-05 | 0.00022 | Down |
| RP11-379L18.1 | 2.969188511 | -2.262505719 | 0.299088 | -7.56469 | 3.89E-14 | 4.49E-13 | Down |
| RP11-379L18.2 | 0.986604078 | -2.331719239 | 0.420857 | -5.5404 | 3.02E-08 | 1.90E-07 | Down |
| RP11-37J13.1 | 4.700734442 | -1.89859248 | 0.306376 | -6.19693 | 5.76E-10 | 4.44E-09 | Down |
| RP11-37O16.3 | 7.147860266 | -1.186875986 | 0.29901 | -3.96936 | 7.21E-05 | 0.000272 | Down |
| RP11-37O16.4 | 3.223002585 | -1.168800505 | 0.322698 | -3.62196 | 0.000292 | 0.000981 | Down |
| RP11-37O16.6 | 4.648027082 | -1.190320983 | 0.33515 | -3.5516 | 0.000383 | 0.001253 | Down |
| RP11-380P13.1 | 0.421968872 | -1.533290873 | 0.617469 | -2.48319 | 0.013021 | 0.028665 | Down |
| RP11-381O7.4 | 6.11592421 | -1.45694916 | 0.385626 | -3.77814 | 0.000158 | 0.000561 | Down |
| RP11-381O7.6 | 4.102834064 | -1.400283981 | 0.390767 | -3.58343 | 0.000339 | 0.001122 | Down |
| RP11-382D12.2 | 3.917641518 | -1.011425019 | 0.31523 | -3.20853 | 0.001334 | 0.003846 | Down |
| RP11-382E9.1 | 1.955914414 | -2.637637302 | 1.030659 | -2.55918 | 0.010492 | 0.023713 | Down |
| RP11-382H24.2 | 1.685323666 | -3.175839239 | 1.287503 | -2.46667 | 0.013638 | 0.0298 | Down |
| RP11-382M14.1 | 1.851518087 | -1.860716954 | 0.523014 | -3.55768 | 0.000374 | 0.001227 | Down |
| RP11-382N13.3 | 1.926394898 | -1.324317622 | 0.536981 | -2.46623 | 0.013654 | 0.029832 | Down |
| RP11-383D22.1 | 1.489497017 | -1.117620101 | 0.33315 | -3.35471 | 0.000794 | 0.002411 | Down |
| RP11-384F7.2 | 4.579000982 | -2.148239425 | 0.237668 | -9.03883 | 1.58E-19 | 2.85E-18 | Down |
| RP11-384G23.1 | 2.796507796 | -2.709632708 | 0.328138 | -8.25759 | 1.49E-16 | 2.11E-15 | Down |
| RP11-384G23.2 | 1.578171354 | -3.124973649 | 0.597937 | -5.22626 | 1.73E-07 | 9.91E-07 | Down |
| RP11-384K6.4 | 0.468914161 | -1.049700007 | 0.440804 | -2.38133 | 0.01725 | 0.036407 | Down |
| RP11-384K6.8 | 5.736501725 | -1.097834098 | 0.259569 | -4.22946 | 2.34E-05 | 9.71E-05 | Down |
| RP11-384O8.1 | 19.41317495 | -2.528783389 | 0.193297 | -13.0824 | 4.15E-39 | 2.74E-37 | Down |
| RP11-384P7.7 | 10.39762708 | -1.119866742 | 0.239518 | -4.67551 | 2.93E-06 | 1.41E-05 | Down |
| RP11-385D13.1 | 13.00896368 | -1.500741704 | 0.292363 | -5.13314 | 2.85E-07 | 1.58E-06 | Down |
| RP11-385H1.1 | 10.82473639 | -2.236380602 | 0.331567 | -6.74489 | 1.53E-11 | 1.37E-10 | Down |
| RP11-385M4.3 | 1.240862377 | -1.724824937 | 0.625275 | -2.75851 | 0.005807 | 0.014131 | Down |
| RP11-386I14.3 | 1.451918645 | -1.851449914 | 0.490951 | -3.77115 | 0.000162 | 0.000575 | Down |
| RP11-386I23.1 | 21.90557335 | -1.031325285 | 0.241681 | -4.2673 | 1.98E-05 | 8.31E-05 | Down |
| RP11-386I8.4 | 1.356376799 | -1.747646603 | 0.378325 | -4.61943 | 3.85E-06 | 1.82E-05 | Down |
| RP11-386I8.6 | 1.842076605 | -1.435414217 | 0.342513 | -4.19084 | 2.78E-05 | 0.000114 | Down |
| RP11-386M24.3 | 18.37278526 | -1.567086711 | 0.23069 | -6.79305 | 1.10E-11 | 1.00E-10 | Down |
| RP11-386M24.6 | 32.45634618 | -1.535153304 | 0.217046 | -7.07293 | 1.52E-12 | 1.51E-11 | Down |
| RP11-386M24.9 | 0.848733824 | -1.625696834 | 0.388387 | -4.18576 | 2.84E-05 | 0.000116 | Down |
| RP11-386O9.2 | 0.977036807 | -1.130298266 | 0.380208 | -2.97284 | 0.002951 | 0.00777 | Down |
| RP11-387A1.5 | 3.093434456 | -1.377994858 | 0.380017 | -3.62614 | 0.000288 | 0.000967 | Down |
| RP11-388C12.1 | 3.029773891 | -1.318538694 | 0.221457 | -5.95393 | 2.62E-09 | 1.87E-08 | Down |
| RP11-388C12.5 | 1.948166345 | -1.275692487 | 0.373667 | -3.41398 | 0.00064 | 0.001986 | Down |
| RP11-388G3.1 | 0.861392076 | -1.403614322 | 0.576792 | -2.43348 | 0.014954 | 0.032231 | Down |
| RP11-389C8.1 | 1.580370375 | -1.775416457 | 0.396452 | -4.47826 | 7.53E-06 | 3.39E-05 | Down |
| RP11-389G6.3 | 5.491834298 | -2.102007444 | 0.291781 | -7.20407 | 5.84E-13 | 6.02E-12 | Down |
| RP11-38L15.2 | 6.372148487 | -1.483303146 | 0.329784 | -4.4978 | 6.87E-06 | 3.12E-05 | Down |
| RP11-38O14.5 | 1.245940774 | -2.402905216 | 0.961592 | -2.49888 | 0.012459 | 0.027582 | Down |
| RP11-390F4.3 | 6.966312571 | -1.694340211 | 0.335068 | -5.05671 | 4.27E-07 | 2.32E-06 | Down |
| RP11-390F4.6 | 6.540639243 | -2.925141584 | 0.275195 | -10.6293 | 2.18E-26 | 6.40E-25 | Down |
| RP11-390M11.1 | 8.599457151 | -1.575009832 | 0.300531 | -5.24076 | 1.60E-07 | 9.19E-07 | Down |
| RP11-391J13.1 | 2.111095242 | -3.347999148 | 0.987576 | -3.39012 | 0.000699 | 0.002151 | Down |
| RP11-391M7.3 | 2.272897137 | -2.063313765 | 0.539983 | -3.82107 | 0.000133 | 0.000477 | Down |
| RP11-392A14.8 | 7.858166193 | -1.166095889 | 0.319767 | -3.6467 | 0.000266 | 0.0009 | Down |
| RP11-392E22.10 | 1.369241687 | -2.275678032 | 0.482743 | -4.71406 | 2.43E-06 | 1.18E-05 | Down |
| RP11-392E22.11 | 7.436734169 | -1.851144134 | 0.236651 | -7.82225 | 5.19E-15 | 6.55E-14 | Down |
| RP11-392E22.9 | 11.71357554 | -1.924051649 | 0.230769 | -8.33756 | 7.58E-17 | 1.10E-15 | Down |
| RP11-394I13.1 | 3.678747997 | -1.359195022 | 0.404685 | -3.35865 | 0.000783 | 0.00238 | Down |
| RP11-394I13.3 | 0.892814907 | -1.516534934 | 0.37594 | -4.03398 | 5.48E-05 | 0.000212 | Down |
| RP11-395C17.1 | 5.410704536 | -3.059014832 | 0.401874 | -7.61187 | 2.70E-14 | 3.18E-13 | Down |
| RP11-395L14.13 | 0.96833802 | -2.152351449 | 0.738743 | -2.91353 | 0.003574 | 0.009212 | Down |
| RP11-395N3.1 | 8.299956945 | -1.324556755 | 0.208518 | -6.35223 | 2.12E-10 | 1.71E-09 | Down |
| RP11-395N3.2 | 16.13439574 | -1.448110994 | 0.111963 | -12.9338 | 2.90E-38 | 1.81E-36 | Down |
| RP11-396O20.2 | 1.105697413 | -1.989124468 | 0.390425 | -5.09477 | 3.49E-07 | 1.92E-06 | Down |
| RP11-397A16.3 | 2.100742646 | -2.867575306 | 0.844482 | -3.39566 | 0.000685 | 0.002112 | Down |
| RP11-398A8.1 | 1.640616797 | -1.273678114 | 0.507334 | -2.51053 | 0.012055 | 0.026808 | Down |
| RP11-398E10.1 | 17.6864868 | -1.163216012 | 0.313868 | -3.70607 | 0.000211 | 0.000728 | Down |
| RP11-398H6.1 | 2.396333095 | -1.53307649 | 0.489256 | -3.13348 | 0.001727 | 0.004848 | Down |
| RP11-398J10.2 | 1.221936859 | -1.219842719 | 0.40598 | -3.00469 | 0.002659 | 0.007075 | Down |
| RP11-398M15.1 | 2.28282681 | -1.089669558 | 0.353699 | -3.08078 | 0.002065 | 0.00567 | Down |
| RP11-399B17.1 | 101.2157604 | -1.599159891 | 0.101887 | -15.6955 | 1.62E-55 | 2.69E-53 | Down |
| RP11-399E6.4 | 4.449256693 | -1.298553472 | 0.214969 | -6.04064 | 1.53E-09 | 1.13E-08 | Down |
| RP11-399H11.2 | 1.213996682 | 1.376181764 | 0.425623 | 3.233335 | 0.001224 | 0.003566 | Up |
| RP11-399J13.2 | 4.472517615 | -1.251640371 | 0.313442 | -3.99321 | 6.52E-05 | 0.000248 | Down |
| RP11-399K21.14 | 15.62408866 | -1.171694645 | 0.161545 | -7.25306 | 4.07E-13 | 4.26E-12 | Down |
| RP11-39C10.1 | 2.349781759 | -1.601236751 | 0.484369 | -3.30582 | 0.000947 | 0.002829 | Down |
| RP11-3B7.1 | 10.90978435 | -1.259159435 | 0.153799 | -8.18707 | 2.68E-16 | 3.71E-15 | Down |
| RP11-3N2.7 | 1.64885209 | -2.341515442 | 0.931945 | -2.5125 | 0.011988 | 0.026677 | Down |
| RP11-3P17.4 | 1.653826021 | -1.689742165 | 0.583197 | -2.89738 | 0.003763 | 0.009647 | Down |
| RP11-400K9.2 | 1.931172427 | -1.894165006 | 0.622749 | -3.04162 | 0.002353 | 0.006362 | Down |
| RP11-400N13.3 | 2.286287226 | 3.464105007 | 0.537627 | 6.44332 | 1.17E-10 | 9.68E-10 | Up |
| RP11-401E9.3 | 1.619338524 | -1.364062133 | 0.363573 | -3.75183 | 0.000176 | 0.000618 | Down |
| RP11-401F2.3 | 3.649979296 | -1.122755814 | 0.223138 | -5.03166 | 4.86E-07 | 2.62E-06 | Down |
| RP11-401L13.7 | 2.214039331 | -1.175000448 | 0.396016 | -2.96705 | 0.003007 | 0.007903 | Down |
| RP11-401P9.1 | 3.393445751 | -1.550586202 | 0.414956 | -3.73675 | 0.000186 | 0.000652 | Down |
| RP11-401P9.7 | 9.637497233 | -1.424556647 | 0.335354 | -4.24792 | 2.16E-05 | 9.01E-05 | Down |
| RP11-402D21.2 | 2.232161591 | -1.19225327 | 0.235239 | -5.06827 | 4.01E-07 | 2.19E-06 | Down |
| RP11-403A21.1 | 2.005237507 | -1.197917267 | 0.425171 | -2.81749 | 0.00484 | 0.012043 | Down |
| RP11-403A21.3 | 1.925522272 | -1.096297814 | 0.444162 | -2.46824 | 0.013578 | 0.029698 | Down |
| RP11-403A3.1 | 1.920270847 | -2.487161228 | 0.855145 | -2.90847 | 0.003632 | 0.009344 | Down |
| RP11-403A3.3 | 1.874594472 | -1.159601092 | 0.388643 | -2.98372 | 0.002848 | 0.007525 | Down |
| RP11-403B2.7 | 23.21187617 | -1.309133027 | 0.186204 | -7.03064 | 2.06E-12 | 2.02E-11 | Down |
| RP11-403D15.1 | 0.834360357 | -1.174994225 | 0.380378 | -3.08901 | 0.002008 | 0.005537 | Down |
| RP11-403I13.9 | 1.212513824 | -1.181896438 | 0.446277 | -2.64835 | 0.008089 | 0.018913 | Down |
| RP11-403P17.2 | 1.240628108 | -1.165353341 | 0.356859 | -3.26559 | 0.001092 | 0.003214 | Down |
| RP11-404G16.2 | 1.8214697 | -1.926912355 | 0.718525 | -2.68176 | 0.007324 | 0.017328 | Down |
| RP11-404O13.4 | 3.974962282 | 1.027851464 | 0.236026 | 4.354822 | 1.33E-05 | 5.77E-05 | Up |
| RP11-404P21.5 | 1.790033053 | -1.723672776 | 0.413911 | -4.16435 | 3.12E-05 | 0.000127 | Down |
| RP11-404P21.6 | 2.318243869 | -2.694224374 | 0.550663 | -4.8927 | 9.95E-07 | 5.12E-06 | Down |
| RP11-405A12.1 | 1.999154347 | -2.41446563 | 0.684349 | -3.52812 | 0.000419 | 0.001356 | Down |
| RP11-406A9.2 | 4.106282492 | -2.373906906 | 0.390275 | -6.08266 | 1.18E-09 | 8.81E-09 | Down |
| RP11-406H4.1 | 1.549785934 | -2.026588924 | 0.532306 | -3.80719 | 0.000141 | 0.000503 | Down |
| RP11-406O23.2 | 0.463871501 | -1.579905346 | 0.546713 | -2.88982 | 0.003855 | 0.009846 | Down |
| RP11-407B7.1 | 33.60649531 | -2.000946203 | 0.175606 | -11.3945 | 4.45E-30 | 1.71E-28 | Down |
| RP11-407N17.3 | 2.780452064 | 2.662196368 | 0.920687 | 2.891532 | 0.003834 | 0.009797 | Up |
| RP11-407P18.1 | 2.944573721 | -2.347439769 | 0.447823 | -5.24189 | 1.59E-07 | 9.14E-07 | Down |
| RP11-408B11.2 | 0.572020005 | -1.495474972 | 0.591696 | -2.52744 | 0.01149 | 0.025688 | Down |
| RP11-408H1.3 | 4.291951636 | -1.455368894 | 0.42194 | -3.44924 | 0.000562 | 0.001767 | Down |
| RP11-408H20.3 | 1.611149149 | -1.882815461 | 0.518445 | -3.63166 | 0.000282 | 0.000949 | Down |
| RP11-408J6.1 | 0.733610878 | -1.638182886 | 0.720594 | -2.27338 | 0.023003 | 0.046618 | Down |
| RP11-409C19.2 | 9.893877868 | -1.484445952 | 0.220251 | -6.7398 | 1.59E-11 | 1.42E-10 | Down |
| RP11-409I10.2 | 1.802146381 | -1.729472538 | 0.314122 | -5.50573 | 3.68E-08 | 2.29E-07 | Down |
| RP11-40C11.2 | 2.227901443 | -1.455393029 | 0.309291 | -4.70559 | 2.53E-06 | 1.23E-05 | Down |
| RP11-40E6.2 | 0.918336322 | -1.650780461 | 0.704684 | -2.34258 | 0.019151 | 0.03981 | Down |
| RP11-40G16.1 | 1.662791622 | -1.772910782 | 0.529757 | -3.34665 | 0.000818 | 0.002475 | Down |
| RP11-410D17.2 | 1.730111535 | -1.542516048 | 0.448523 | -3.4391 | 0.000584 | 0.001828 | Down |
| RP11-411B10.2 | 3.851523432 | -1.103930708 | 0.254054 | -4.34526 | 1.39E-05 | 6.00E-05 | Down |
| RP11-411B10.7 | 6.053451093 | -1.112409141 | 0.292537 | -3.80263 | 0.000143 | 0.000512 | Down |
| RP11-411B10.8 | 1.796615081 | -1.487491869 | 0.358237 | -4.15226 | 3.29E-05 | 0.000133 | Down |
| RP11-411K7.1 | 73.47308305 | -1.006246538 | 0.161135 | -6.24474 | 4.25E-10 | 3.32E-09 | Down |
| RP11-412P11.1 | 1.473725939 | -2.781921669 | 0.950201 | -2.92772 | 0.003415 | 0.008848 | Down |
| RP11-413B19.2 | 2.200760542 | 1.703915783 | 0.45558 | 3.740104 | 0.000184 | 0.000644 | Up |
| RP11-413G15.1 | 3.249376202 | -1.847031449 | 0.352764 | -5.23589 | 1.64E-07 | 9.43E-07 | Down |
| RP11-414H23.3 | 1.101680667 | 1.016184064 | 0.452415 | 2.246132 | 0.024696 | 0.049571 | Up |
| RP11-415C15.2 | 1.534865851 | -3.03294652 | 1.192346 | -2.54368 | 0.010969 | 0.02465 | Down |
| RP11-416H1.2 | 2.280183436 | -1.247387591 | 0.481874 | -2.58862 | 0.009636 | 0.022042 | Down |
| RP11-416I2.1 | 10.71441236 | 1.272360732 | 0.406556 | 3.129608 | 0.00175 | 0.004905 | Up |
| RP11-417E7.1 | 3.674112289 | 1.707032072 | 0.285295 | 5.983402 | 2.19E-09 | 1.58E-08 | Up |
| RP11-417E7.2 | 2.558926514 | 2.548659193 | 0.372179 | 6.847934 | 7.49E-12 | 6.96E-11 | Up |
| RP11-417L14.1 | 1.906382662 | -1.414395562 | 0.566554 | -2.49649 | 0.012543 | 0.027749 | Down |
| RP11-417O11.5 | 1.451901347 | -1.499714178 | 0.445435 | -3.36685 | 0.00076 | 0.002318 | Down |
| RP11-417O18.1 | 1.573089139 | -2.373294094 | 0.61265 | -3.87382 | 0.000107 | 0.000392 | Down |
| RP11-418J17.3 | 3.69754602 | -1.752418988 | 0.232859 | -7.52567 | 5.24E-14 | 6.00E-13 | Down |
| RP11-419L20.2 | 9.709812317 | -2.742049535 | 0.296609 | -9.24466 | 2.36E-20 | 4.51E-19 | Down |
| RP11-41L14.1 | 1.993856622 | -1.034137635 | 0.407778 | -2.53603 | 0.011212 | 0.025136 | Down |
| RP11-41O4.2 | 1.882859174 | -1.503084418 | 0.665684 | -2.25795 | 0.023949 | 0.048279 | Down |
| RP11-420A6.2 | 3.109114253 | -1.022682554 | 0.310727 | -3.29126 | 0.000997 | 0.002963 | Down |
| RP11-420B22.1 | 2.006354332 | 2.122327086 | 0.370379 | 5.730151 | 1.00E-08 | 6.73E-08 | Up |
| RP11-420B22.2 | 1.845164165 | 1.754198887 | 0.365576 | 4.798445 | 1.60E-06 | 7.99E-06 | Up |
| RP11-420J11.1 | 3.436717483 | -2.108836736 | 0.434575 | -4.85264 | 1.22E-06 | 6.20E-06 | Down |
| RP11-420K14.1 | 2.949457162 | -1.187161512 | 0.271557 | -4.37169 | 1.23E-05 | 5.37E-05 | Down |
| RP11-420O16.2 | 2.307262167 | -2.281436535 | 0.660612 | -3.45352 | 0.000553 | 0.001742 | Down |
| RP11-422N16.3 | 11.08431049 | -1.896760958 | 0.28972 | -6.54689 | 5.87E-11 | 5.00E-10 | Down |
| RP11-423C15.3 | 4.93685503 | -1.354053222 | 0.355844 | -3.80519 | 0.000142 | 0.000507 | Down |
| RP11-423H2.3 | 15.78489704 | -1.577256289 | 0.190176 | -8.29367 | 1.10E-16 | 1.57E-15 | Down |
| RP11-423H2.4 | 1.561333472 | -1.697616771 | 0.399915 | -4.24494 | 2.19E-05 | 9.12E-05 | Down |
| RP11-424M24.5 | 4.153517285 | -1.262344012 | 0.199376 | -6.33146 | 2.43E-10 | 1.94E-09 | Down |
| RP11-425A23.1 | 0.984427074 | -1.209407874 | 0.382917 | -3.15841 | 0.001586 | 0.004493 | Down |
| RP11-426J5.1 | 2.395962388 | -1.004033151 | 0.277479 | -3.61841 | 0.000296 | 0.000994 | Down |
| RP11-427J23.1 | 6.717788264 | -1.514279029 | 0.308207 | -4.91318 | 8.96E-07 | 4.65E-06 | Down |
| RP11-428C6.2 | 1.383254654 | -2.535766955 | 0.728958 | -3.47862 | 0.000504 | 0.001602 | Down |
| RP11-428G5.7 | 2.197658451 | -1.285958648 | 0.380764 | -3.37731 | 0.000732 | 0.002241 | Down |
| RP11-428J1.4 | 14.12787175 | -1.719874439 | 0.193538 | -8.88648 | 6.31E-19 | 1.08E-17 | Down |
| RP11-428L9.2 | 1.761150792 | -1.543315093 | 0.664832 | -2.32136 | 0.020267 | 0.041822 | Down |
| RP11-428P16.2 | 9.416380724 | -1.38104101 | 0.263184 | -5.24743 | 1.54E-07 | 8.89E-07 | Down |
| RP11-428P16.3 | 1.279278995 | -1.632509987 | 0.498054 | -3.27778 | 0.001046 | 0.003093 | Down |
| RP11-429A20.3 | 1.428682448 | -1.965413686 | 0.525356 | -3.74111 | 0.000183 | 0.000642 | Down |
| RP11-429A20.4 | 1.859778047 | -1.75450886 | 0.414007 | -4.23787 | 2.26E-05 | 9.38E-05 | Down |
| RP11-429K17.1 | 2.049296753 | -3.280271336 | 1.231347 | -2.66397 | 0.007722 | 0.018161 | Down |
| RP11-42H13.1 | 1.351785012 | -2.418501281 | 0.698556 | -3.46215 | 0.000536 | 0.001693 | Down |
| RP11-42O15.2 | 1.574818695 | -2.945642641 | 1.187822 | -2.47987 | 0.013143 | 0.028888 | Down |
| RP11-42O15.3 | 2.306902064 | -1.338825198 | 0.278381 | -4.80932 | 1.51E-06 | 7.59E-06 | Down |
| RP11-430C7.4 | 2.785404685 | -1.268862745 | 0.292257 | -4.34159 | 1.41E-05 | 6.09E-05 | Down |
| RP11-430C7.5 | 15.93896693 | -1.215388893 | 0.254 | -4.785 | 1.71E-06 | 8.52E-06 | Down |
| RP11-430G17.1 | 1.109610102 | -1.803906883 | 0.57122 | -3.15799 | 0.001589 | 0.004498 | Down |
| RP11-431D12.1 | 0.657857096 | -1.948462403 | 0.474429 | -4.10697 | 4.01E-05 | 0.000159 | Down |
| RP11-431K24.1 | 22.87434341 | -1.451971287 | 0.196653 | -7.38343 | 1.54E-13 | 1.69E-12 | Down |
| RP11-431K24.2 | 0.927508528 | -1.232094301 | 0.541453 | -2.27553 | 0.022874 | 0.046386 | Down |
| RP11-431K24.3 | 0.371507696 | -1.092004694 | 0.475692 | -2.29561 | 0.021698 | 0.044327 | Down |
| RP11-431M7.3 | 8.204099231 | -1.278775151 | 0.149024 | -8.58101 | 9.40E-18 | 1.47E-16 | Down |
| RP11-432I5.4 | 2.43586634 | -1.471254979 | 0.315387 | -4.66492 | 3.09E-06 | 1.48E-05 | Down |
| RP11-432I5.8 | 3.166497274 | -1.201137041 | 0.279224 | -4.3017 | 1.69E-05 | 7.21E-05 | Down |
| RP11-432N13.2 | 1.455097546 | -1.523759343 | 0.500871 | -3.04222 | 0.002348 | 0.006351 | Down |
| RP11-433J8.1 | 2.903531983 | -1.765898294 | 0.497074 | -3.55259 | 0.000381 | 0.001248 | Down |
| RP11-433P17.2 | 10.68995322 | -1.2037012 | 0.241578 | -4.98265 | 6.27E-07 | 3.33E-06 | Down |
| RP11-434D9.1 | 2.249942449 | -1.788093496 | 0.47586 | -3.7576 | 0.000172 | 0.000605 | Down |
| RP11-434H6.2 | 1.836941206 | -2.641246547 | 0.615197 | -4.29334 | 1.76E-05 | 7.46E-05 | Down |
| RP11-435I10.3 | 2.191364262 | -1.463509886 | 0.403436 | -3.62761 | 0.000286 | 0.000963 | Down |
| RP11-435I10.5 | 2.144919731 | -1.264801749 | 0.437909 | -2.88828 | 0.003874 | 0.009889 | Down |
| RP11-436D10.3 | 1.135294248 | -1.397975256 | 0.354242 | -3.94639 | 7.93E-05 | 0.000298 | Down |
| RP11-436I9.3 | 3.573906894 | -1.043812627 | 0.396191 | -2.63462 | 0.008423 | 0.019607 | Down |
| RP11-436K8.1 | 9.912805031 | -2.541618811 | 0.262315 | -9.68917 | 3.35E-22 | 7.32E-21 | Down |
| RP11-437F6.1 | 1.109296667 | -1.309942454 | 0.392322 | -3.33895 | 0.000841 | 0.00254 | Down |
| RP11-438B23.2 | 41.05644418 | -2.896979885 | 0.25927 | -11.1736 | 5.49E-29 | 1.97E-27 | Down |
| RP11-438F14.3 | 1.568947515 | -2.750852078 | 0.823228 | -3.34154 | 0.000833 | 0.002518 | Down |
| RP11-438L7.3 | 3.059201318 | -1.469080366 | 0.393989 | -3.72873 | 0.000192 | 0.000671 | Down |
| RP11-439C15.4 | 2.294608974 | -1.974244475 | 0.338452 | -5.83315 | 5.44E-09 | 3.75E-08 | Down |
| RP11-439C15.5 | 0.965777327 | -1.51448123 | 0.427607 | -3.54176 | 0.000397 | 0.001295 | Down |
| RP11-439E19.9 | 8.846165739 | -1.25946586 | 0.247794 | -5.08271 | 3.72E-07 | 2.03E-06 | Down |
| RP11-439M15.1 | 6.134018204 | -1.196705345 | 0.231595 | -5.16722 | 2.38E-07 | 1.34E-06 | Down |
| RP11-43A14.1 | 0.506022407 | -1.06519038 | 0.419048 | -2.54193 | 0.011024 | 0.024752 | Down |
| RP11-43F13.3 | 11.27374091 | -1.086615196 | 0.288786 | -3.7627 | 0.000168 | 0.000594 | Down |
| RP11-440D17.6 | 3.131274126 | -2.703864185 | 0.363326 | -7.44197 | 9.92E-14 | 1.10E-12 | Down |
| RP11-440G9.1 | 0.759550254 | 1.720856667 | 0.633803 | 2.71513 | 0.006625 | 0.015883 | Up |
| RP11-440I14.2 | 1.982688368 | -1.724613704 | 0.668402 | -2.58021 | 0.009874 | 0.022507 | Down |
| RP11-440I14.3 | 1.260660245 | -3.374304667 | 1.017876 | -3.31505 | 0.000916 | 0.002747 | Down |
| RP11-440L14.3 | 1.117327837 | -1.610348885 | 0.624168 | -2.57999 | 0.00988 | 0.02252 | Down |
| RP11-440L14.4 | 18.69688621 | -1.190689222 | 0.198672 | -5.99324 | 2.06E-09 | 1.49E-08 | Down |
| RP11-442O1.3 | 6.031268317 | -1.832435372 | 0.298813 | -6.13237 | 8.66E-10 | 6.55E-09 | Down |
| RP11-442O1.4 | 2.839235686 | -1.866713992 | 0.407065 | -4.58579 | 4.52E-06 | 2.11E-05 | Down |
| RP11-443O13.3 | 0.762179131 | -1.155036162 | 0.436087 | -2.64864 | 0.008082 | 0.018903 | Down |
| RP11-443P15.2 | 6.968330717 | -1.066682722 | 0.261761 | -4.07503 | 4.60E-05 | 0.000181 | Down |
| RP11-444D3.1 | 26.75571315 | -1.113012678 | 0.137484 | -8.09558 | 5.70E-16 | 7.69E-15 | Down |
| RP11-444P10.1 | 1.526377964 | -3.054775891 | 1.171566 | -2.60743 | 0.009122 | 0.021027 | Down |
| RP11-445F6.2 | 4.495741409 | -1.148041452 | 0.398639 | -2.8799 | 0.003978 | 0.010124 | Down |
| RP11-445O16.3 | 1.526643214 | -1.02403419 | 0.418047 | -2.44957 | 0.014303 | 0.031032 | Down |
| RP11-445P17.3 | 1.468878259 | -1.850544882 | 0.693903 | -2.66686 | 0.007656 | 0.018027 | Down |
| RP11-446E24.3 | 6.854458353 | -2.618782678 | 0.391886 | -6.68252 | 2.35E-11 | 2.07E-10 | Down |
| RP11-446E24.4 | 4.844749871 | -1.107861498 | 0.275705 | -4.01829 | 5.86E-05 | 0.000225 | Down |
| RP11-446H18.6 | 0.92979886 | -1.179784603 | 0.501387 | -2.35304 | 0.018621 | 0.038873 | Down |
| RP11-446J8.1 | 0.558383619 | -1.549246653 | 0.509974 | -3.03789 | 0.002382 | 0.006436 | Down |
| RP11-447H19.1 | 1.996593422 | -2.335688114 | 0.558386 | -4.18293 | 2.88E-05 | 0.000117 | Down |
| RP11-447H19.3 | 2.472376167 | -1.806952856 | 0.453605 | -3.98354 | 6.79E-05 | 0.000258 | Down |
| RP11-448G15.1 | 3.207941911 | -1.101196475 | 0.234387 | -4.69819 | 2.62E-06 | 1.27E-05 | Down |
| RP11-449J21.5 | 27.39749797 | -1.490286225 | 0.153063 | -9.73644 | 2.11E-22 | 4.69E-21 | Down |
| RP11-449L23.2 | 3.134079295 | -2.095047452 | 0.456606 | -4.58831 | 4.47E-06 | 2.09E-05 | Down |
| RP11-449L23.3 | 1.829142483 | -1.849576956 | 0.476148 | -3.88446 | 0.000103 | 0.000377 | Down |
| RP11-44F14.1 | 3.561394617 | -1.37456168 | 0.376997 | -3.64608 | 0.000266 | 0.000902 | Down |
| RP11-44F14.8 | 5.81293035 | -1.061399081 | 0.375783 | -2.8245 | 0.004735 | 0.011823 | Down |
| RP11-44F14.9 | 1.62650872 | -1.739917105 | 0.57865 | -3.00685 | 0.00264 | 0.007031 | Down |
| RP11-44N11.2 | 16.38549719 | 3.126786541 | 0.242669 | 12.88501 | 5.47E-38 | 3.36E-36 | Up |
| RP11-44N11.3 | 3.043143661 | 3.240750967 | 0.347712 | 9.32022 | 1.16E-20 | 2.27E-19 | Up |
| RP11-44N21.3 | 3.571812215 | -1.418751244 | 0.357361 | -3.97008 | 7.18E-05 | 0.000272 | Down |
| RP11-450I1.4 | 1.734506633 | -1.834263129 | 0.703848 | -2.60605 | 0.009159 | 0.02109 | Down |
| RP11-451F20.1 | 1.220314419 | -2.558571537 | 1.06421 | -2.4042 | 0.016208 | 0.034498 | Down |
| RP11-451G4.2 | 0.629091054 | -1.758992994 | 0.737772 | -2.3842 | 0.017116 | 0.036167 | Down |
| RP11-451O13.1 | 1.313444519 | -2.516461542 | 1.117003 | -2.25287 | 0.024267 | 0.048829 | Down |
| RP11-452D2.2 | 1.327267511 | -1.805113396 | 0.673844 | -2.67883 | 0.007388 | 0.017465 | Down |
| RP11-454C18.1 | 0.738981263 | -1.919105262 | 0.434564 | -4.41616 | 1.00E-05 | 4.44E-05 | Down |
| RP11-454H19.2 | 19.31225185 | -1.529625903 | 0.199313 | -7.67451 | 1.66E-14 | 1.99E-13 | Down |
| RP11-454K7.3 | 2.11384392 | -1.457301035 | 0.359078 | -4.05845 | 4.94E-05 | 0.000193 | Down |
| RP11-455B3.1 | 1.517015192 | -1.965212492 | 0.639182 | -3.07457 | 0.002108 | 0.005776 | Down |
| RP11-455F5.4 | 1.773387814 | -1.740532359 | 0.400824 | -4.34239 | 1.41E-05 | 6.07E-05 | Down |
| RP11-455F5.6 | 6.241451334 | -1.013218013 | 0.269689 | -3.75699 | 0.000172 | 0.000607 | Down |
| RP11-455I9.1 | 10.41849611 | -1.249901989 | 0.277719 | -4.5006 | 6.78E-06 | 3.08E-05 | Down |
| RP11-455O6.5 | 33.57207302 | -1.074072872 | 0.156105 | -6.88047 | 5.97E-12 | 5.60E-11 | Down |
| RP11-456J20.1 | 8.827352665 | -1.156442872 | 0.244327 | -4.73318 | 2.21E-06 | 1.08E-05 | Down |
| RP11-456O19.2 | 1.254929472 | -2.248596019 | 0.420305 | -5.34991 | 8.80E-08 | 5.23E-07 | Down |
| RP11-456O19.3 | 2.322395461 | -2.00089895 | 0.321404 | -6.2255 | 4.80E-10 | 3.73E-09 | Down |
| RP11-456O19.5 | 1.470069407 | -3.18916261 | 0.416566 | -7.65584 | 1.92E-14 | 2.29E-13 | Down |
| RP11-457M11.6 | 2.706751384 | -1.300784564 | 0.39705 | -3.27612 | 0.001052 | 0.003108 | Down |
| RP11-457M11.7 | 12.13209067 | -1.16505218 | 0.279649 | -4.16612 | 3.10E-05 | 0.000126 | Down |
| RP11-459C13.1 | 3.311798481 | -2.213635031 | 0.256524 | -8.62936 | 6.17E-18 | 9.78E-17 | Down |
| RP11-459E5.1 | 4.190909329 | -1.307041835 | 0.296621 | -4.40644 | 1.05E-05 | 4.62E-05 | Down |
| RP11-459O16.8 | 8.82174416 | -1.360064829 | 0.27302 | -4.98156 | 6.31E-07 | 3.34E-06 | Down |
| RP11-45L9.1 | 1.089775061 | 1.190308046 | 0.315586 | 3.771742 | 0.000162 | 0.000574 | Up |
| RP11-460B17.1 | 3.097849646 | -1.63928596 | 0.362935 | -4.51675 | 6.28E-06 | 2.87E-05 | Down |
| RP11-460I13.2 | 1.131453554 | -1.039715911 | 0.357275 | -2.91013 | 0.003613 | 0.009301 | Down |
| RP11-461C20.1 | 0.773427345 | -1.93838847 | 0.768534 | -2.52219 | 0.011663 | 0.02604 | Down |
| RP11-461F11.3 | 2.221462244 | -1.801707964 | 0.42183 | -4.27117 | 1.94E-05 | 8.18E-05 | Down |
| RP11-461L13.5 | 1.196750588 | -1.151003966 | 0.471835 | -2.43942 | 0.014711 | 0.031794 | Down |
| RP11-461L18.1 | 1.891728216 | -2.217300175 | 0.688366 | -3.2211 | 0.001277 | 0.003701 | Down |
| RP11-462D18.2 | 8.464141004 | -1.144878849 | 0.211202 | -5.42077 | 5.93E-08 | 3.60E-07 | Down |
| RP11-462D18.4 | 0.681518227 | -1.373717139 | 0.415176 | -3.30876 | 0.000937 | 0.002803 | Down |
| RP11-462G12.1 | 36.05202788 | -1.327222912 | 0.122569 | -10.8284 | 2.53E-27 | 8.03E-26 | Down |
| RP11-462G12.2 | 4.277137423 | -1.312683456 | 0.223729 | -5.8673 | 4.43E-09 | 3.08E-08 | Down |
| RP11-463J17.2 | 6.458925218 | -1.458422683 | 0.250368 | -5.82513 | 5.71E-09 | 3.92E-08 | Down |
| RP11-463O12.5 | 537.5052737 | -1.012779935 | 0.186419 | -5.43282 | 5.55E-08 | 3.38E-07 | Down |
| RP11-463O9.1 | 3.801521852 | -1.051377952 | 0.244548 | -4.29927 | 1.71E-05 | 7.28E-05 | Down |
| RP11-463O9.2 | 5.095625882 | -1.412079078 | 0.2814 | -5.01806 | 5.22E-07 | 2.80E-06 | Down |
| RP11-463O9.9 | 10.05283998 | -1.850218819 | 0.25351 | -7.2984 | 2.91E-13 | 3.10E-12 | Down |
| RP11-464C19.3 | 0.547177989 | -1.852082555 | 0.725724 | -2.55205 | 0.010709 | 0.024141 | Down |
| RP11-464F9.21 | 0.870664181 | -1.460225558 | 0.516351 | -2.82797 | 0.004684 | 0.011707 | Down |
| RP11-465B22.3 | 11.02376674 | 1.062368405 | 0.14933 | 7.114224 | 1.13E-12 | 1.14E-11 | Up |
| RP11-466F5.10 | 0.75013862 | -1.218502662 | 0.438652 | -2.77784 | 0.005472 | 0.013421 | Down |
| RP11-466F5.3 | 2.555597366 | -1.345478954 | 0.31714 | -4.24254 | 2.21E-05 | 9.21E-05 | Down |
| RP11-466F5.6 | 2.397543993 | -1.7108241 | 0.351711 | -4.86429 | 1.15E-06 | 5.87E-06 | Down |
| RP11-466G12.2 | 1.819514617 | -1.128980512 | 0.304634 | -3.70602 | 0.000211 | 0.000728 | Down |
| RP11-466G12.3 | 2.750783726 | -1.524955511 | 0.375711 | -4.05885 | 4.93E-05 | 0.000193 | Down |
| RP11-466L17.1 | 10.53892741 | -1.866593972 | 0.293248 | -6.36524 | 1.95E-10 | 1.58E-09 | Down |
| RP11-467J12.2 | 1.55504297 | 1.177407085 | 0.433585 | 2.715513 | 0.006617 | 0.01587 | Up |
| RP11-467J12.4 | 0.597164393 | 1.309884237 | 0.472433 | 2.772636 | 0.00556 | 0.01361 | Up |
| RP11-467K18.2 | 2.392990052 | -1.584116733 | 0.376311 | -4.20959 | 2.56E-05 | 0.000105 | Down |
| RP11-467L19.14 | 5.164541944 | -1.264806696 | 0.417111 | -3.0323 | 0.002427 | 0.006535 | Down |
| RP11-467L19.16 | 2.783318482 | -1.880785896 | 0.423978 | -4.43605 | 9.16E-06 | 4.07E-05 | Down |
| RP11-467P9.1 | 8.823716024 | -1.099242767 | 0.157226 | -6.99149 | 2.72E-12 | 2.63E-11 | Down |
| RP11-468O2.1 | 7.930701149 | -1.798827582 | 0.315284 | -5.70542 | 1.16E-08 | 7.72E-08 | Down |
| RP11-469H8.6 | 11.98996654 | 1.074668527 | 0.199509 | 5.386579 | 7.18E-08 | 4.31E-07 | Up |
| RP11-469N6.1 | 18.84088298 | -1.166726926 | 0.305202 | -3.82281 | 0.000132 | 0.000474 | Down |
| RP11-46C24.3 | 1.353656423 | -2.518613852 | 0.861504 | -2.92351 | 0.003461 | 0.008952 | Down |
| RP11-46H11.12 | 2.930333862 | -1.149602549 | 0.210465 | -5.46219 | 4.70E-08 | 2.89E-07 | Down |
| RP11-470E16.2 | 1.87824338 | -2.732691611 | 0.400153 | -6.82911 | 8.54E-12 | 7.89E-11 | Down |
| RP11-472F14.4 | 1.256013569 | -2.956406978 | 0.561429 | -5.26586 | 1.40E-07 | 8.09E-07 | Down |
| RP11-473C19.1 | 0.665874337 | -1.641352466 | 0.419193 | -3.91551 | 9.02E-05 | 0.000335 | Down |
| RP11-473M10.3 | 1.767042806 | -3.03295957 | 0.827619 | -3.66468 | 0.000248 | 0.000844 | Down |
| RP11-473M20.16 | 11.35557977 | -1.182392308 | 0.164848 | -7.17263 | 7.36E-13 | 7.53E-12 | Down |
| RP11-474C8.7 | 2.654021511 | -1.901644194 | 0.608728 | -3.12397 | 0.001784 | 0.004989 | Down |
| RP11-474C8.8 | 1.379450799 | -1.010058395 | 0.336204 | -3.0043 | 0.002662 | 0.007083 | Down |
| RP11-474D1.2 | 1.697911984 | -3.118527705 | 1.016125 | -3.06904 | 0.002147 | 0.005873 | Down |
| RP11-474I16.8 | 2.228127232 | -1.016688445 | 0.241659 | -4.20711 | 2.59E-05 | 0.000106 | Down |
| RP11-474O21.5 | 97.76043826 | 3.391282579 | 0.202401 | 16.75527 | 5.18E-63 | 1.14E-60 | Up |
| RP11-474P2.2 | 1.324276429 | -1.544880159 | 0.326811 | -4.72714 | 2.28E-06 | 1.11E-05 | Down |
| RP11-474P2.7 | 39.9459597 | -1.048987614 | 0.186786 | -5.61597 | 1.95E-08 | 1.26E-07 | Down |
| RP11-475B2.1 | 0.986638021 | -1.766778805 | 0.596378 | -2.96251 | 0.003051 | 0.008009 | Down |
| RP11-475D10.4 | 2.219251074 | -1.061420608 | 0.350223 | -3.0307 | 0.00244 | 0.006564 | Down |
| RP11-475O6.1 | 59.97718217 | -1.316176648 | 0.158025 | -8.32892 | 8.16E-17 | 1.18E-15 | Down |
| RP11-476C8.2 | 8.455965818 | -1.243380021 | 0.21693 | -5.73171 | 9.94E-09 | 6.67E-08 | Down |
| RP11-476D10.1 | 46.17681644 | 3.951102775 | 0.370737 | 10.65742 | 1.61E-26 | 4.79E-25 | Up |
| RP11-476H24.1 | 1.763648585 | -2.332474932 | 0.450818 | -5.17387 | 2.29E-07 | 1.29E-06 | Down |
| RP11-478B9.2 | 1.866964748 | -2.700275042 | 0.988382 | -2.73202 | 0.006295 | 0.015186 | Down |
| RP11-478B9.3 | 1.40064332 | -1.717416831 | 0.762246 | -2.2531 | 0.024253 | 0.048812 | Down |
| RP11-478J18.2 | 23.05437169 | -1.929656821 | 0.194306 | -9.93103 | 3.05E-23 | 7.19E-22 | Down |
| RP11-479O17.10 | 13.14812052 | -1.498975312 | 0.299374 | -5.00703 | 5.53E-07 | 2.95E-06 | Down |
| RP11-479O17.9 | 4.278832839 | -1.568037739 | 0.373899 | -4.19375 | 2.74E-05 | 0.000112 | Down |
| RP11-47A17.2 | 1.488565912 | -1.220418879 | 0.49627 | -2.45918 | 0.013925 | 0.030348 | Down |
| RP11-47G4.1 | 1.582804882 | -3.124339717 | 0.816829 | -3.82496 | 0.000131 | 0.000471 | Down |
| RP11-480C22.1 | 1.140494365 | 2.660644664 | 0.39958 | 6.658598 | 2.76E-11 | 2.42E-10 | Up |
| RP11-480D4.1 | 1.511960691 | -2.349078539 | 0.83215 | -2.8229 | 0.004759 | 0.011872 | Down |
| RP11-480G7.2 | 4.094175997 | -1.952953333 | 0.367023 | -5.32107 | 1.03E-07 | 6.07E-07 | Down |
| RP11-480I12.10 | 1.860057391 | -2.059383097 | 0.353733 | -5.82186 | 5.82E-09 | 4.00E-08 | Down |
| RP11-480I12.7 | 3.639835647 | -1.610194278 | 0.339468 | -4.74328 | 2.10E-06 | 1.03E-05 | Down |
| RP11-481A20.10 | 4.091213918 | 2.193642312 | 0.472762 | 4.640057 | 3.48E-06 | 1.66E-05 | Up |
| RP11-481F24.3 | 2.453368698 | -3.157768058 | 0.846419 | -3.73074 | 0.000191 | 0.000667 | Down |
| RP11-481G8.2 | 4.949972753 | -1.646074078 | 0.3098 | -5.31334 | 1.08E-07 | 6.32E-07 | Down |
| RP11-481H12.1 | 11.33548467 | -1.540425153 | 0.1845 | -8.3492 | 6.87E-17 | 1.00E-15 | Down |
| RP11-481J13.1 | 3.396190056 | -1.744685146 | 0.282369 | -6.17874 | 6.46E-10 | 4.95E-09 | Down |
| RP11-481J2.2 | 1.274268887 | -2.184973353 | 0.620153 | -3.52328 | 0.000426 | 0.001378 | Down |
| RP11-481J8.3 | 1.795921779 | -2.815834049 | 0.788027 | -3.57327 | 0.000353 | 0.001163 | Down |
| RP11-482D24.2 | 2.267127614 | -2.027019299 | 0.355927 | -5.69504 | 1.23E-08 | 8.17E-08 | Down |
| RP11-482H16.1 | 68.3064525 | -1.483612855 | 0.174392 | -8.50735 | 1.78E-17 | 2.72E-16 | Down |
| RP11-482L11.1 | 12.75915391 | -1.659329514 | 0.255126 | -6.50395 | 7.82E-11 | 6.58E-10 | Down |
| RP11-483E17.1 | 3.790700774 | -1.276062327 | 0.364853 | -3.49747 | 0.00047 | 0.001504 | Down |
| RP11-483H20.4 | 1.235381902 | -1.037429358 | 0.385238 | -2.69296 | 0.007082 | 0.016835 | Down |
| RP11-483I13.2 | 3.169689433 | -1.247652868 | 0.516193 | -2.41703 | 0.015648 | 0.033472 | Down |
| RP11-484O2.1 | 4.14440144 | -1.240526897 | 0.24576 | -5.04771 | 4.47E-07 | 2.42E-06 | Down |
| RP11-485O10.3 | 1.396616983 | -1.622487707 | 0.564913 | -2.8721 | 0.004078 | 0.010346 | Down |
| RP11-486A14.1 | 1.812459701 | -1.510715121 | 0.481641 | -3.1366 | 0.001709 | 0.004802 | Down |
| RP11-486G15.2 | 13.97613387 | -1.248006579 | 0.147999 | -8.43253 | 3.38E-17 | 5.05E-16 | Down |
| RP11-486L19.2 | 1.373505393 | -1.678194363 | 0.74659 | -2.24781 | 0.024588 | 0.049384 | Down |
| RP11-488L18.1 | 1.736730949 | -1.227008699 | 0.54421 | -2.25466 | 0.024155 | 0.048652 | Down |
| RP11-490O6.2 | 34.4519268 | -1.268993721 | 0.1269 | -9.99992 | 1.53E-23 | 3.69E-22 | Down |
| RP11-491F9.1 | 3.467048779 | 1.136500942 | 0.271678 | 4.183268 | 2.87E-05 | 0.000117 | Up |
| RP11-494O16.4 | 2.672636234 | -1.496851616 | 0.42042 | -3.56038 | 0.00037 | 0.001216 | Down |
| RP11-495K9.2 | 6.803006285 | -3.389806789 | 0.346377 | -9.78645 | 1.29E-22 | 2.91E-21 | Down |
| RP11-495K9.3 | 6.399243913 | -2.460703698 | 0.268785 | -9.15492 | 5.44E-20 | 1.01E-18 | Down |
| RP11-495K9.6 | 4.314618756 | -2.621448217 | 0.349944 | -7.49105 | 6.83E-14 | 7.73E-13 | Down |
| RP11-496B10.3 | 0.738701315 | -1.870556505 | 0.356241 | -5.25082 | 1.51E-07 | 8.74E-07 | Down |
| RP11-496N12.6 | 1.830087224 | -2.219693903 | 0.408638 | -5.43193 | 5.57E-08 | 3.39E-07 | Down |
| RP11-497E19.1 | 10.87120886 | -1.442448628 | 0.168507 | -8.56016 | 1.13E-17 | 1.75E-16 | Down |
| RP11-497E19.2 | 1.400911425 | -1.316905716 | 0.347514 | -3.78951 | 0.000151 | 0.000538 | Down |
| RP11-497G19.1 | 1.091460184 | -1.705914739 | 0.531104 | -3.21202 | 0.001318 | 0.003806 | Down |
| RP11-497G19.3 | 1.399276668 | -1.756338963 | 0.328244 | -5.35071 | 8.76E-08 | 5.20E-07 | Down |
| RP11-497H16.5 | 86.42530283 | -1.148621807 | 0.289964 | -3.96125 | 7.46E-05 | 0.000281 | Down |
| RP11-498B4.5 | 2.584592453 | -1.46656686 | 0.444654 | -3.29822 | 0.000973 | 0.002899 | Down |
| RP11-498C9.12 | 1.534907106 | -1.132540156 | 0.328225 | -3.4505 | 0.00056 | 0.001759 | Down |
| RP11-498M5.2 | 1.111300165 | -1.207190745 | 0.448897 | -2.68924 | 0.007162 | 0.017001 | Down |
| RP11-499O7.7 | 0.622091966 | -1.43918022 | 0.403545 | -3.56635 | 0.000362 | 0.001191 | Down |
| RP11-49G2.3 | 1.87254832 | -1.040182043 | 0.422248 | -2.46344 | 0.013761 | 0.030035 | Down |
| RP11-49I11.4 | 1.302250248 | 1.648244924 | 0.297612 | 5.538234 | 3.06E-08 | 1.93E-07 | Up |
| RP11-49K24.3 | 3.040774806 | -1.48549135 | 0.293926 | -5.05397 | 4.33E-07 | 2.35E-06 | Down |
| RP11-49K24.8 | 53.08999701 | -1.005877076 | 0.155232 | -6.47983 | 9.18E-11 | 7.68E-10 | Down |
| RP11-49K24.9 | 27.16481373 | -1.85252304 | 0.643482 | -2.8789 | 0.003991 | 0.010151 | Down |
| RP11-4C20.3 | 7.403245089 | 1.133863716 | 0.23759 | 4.772348 | 1.82E-06 | 9.03E-06 | Up |
| RP11-4C20.4 | 1.292519143 | 1.028538327 | 0.324801 | 3.166675 | 0.001542 | 0.00438 | Up |
| RP11-4L24.1 | 1.689158498 | -2.22449576 | 0.760738 | -2.92413 | 0.003454 | 0.008939 | Down |
| RP11-4L24.3 | 9.80371404 | -1.194501428 | 0.224333 | -5.32467 | 1.01E-07 | 5.96E-07 | Down |
| RP11-4M23.2 | 11.31731102 | -1.234240226 | 0.381453 | -3.23563 | 0.001214 | 0.003541 | Down |
| RP11-500G22.4 | 1.582782428 | -1.368165939 | 0.329133 | -4.15688 | 3.23E-05 | 0.000131 | Down |
| RP11-501E14.1 | 1.342316162 | -1.58811454 | 0.493891 | -3.21551 | 0.001302 | 0.003766 | Down |
| RP11-503C24.4 | 1.992626102 | -1.490688449 | 0.596579 | -2.49873 | 0.012464 | 0.027592 | Down |
| RP11-503C24.6 | 1.443916495 | -3.143897987 | 0.959246 | -3.27747 | 0.001047 | 0.003096 | Down |
| RP11-503N18.1 | 9.428437423 | -1.266729079 | 0.22451 | -5.64219 | 1.68E-08 | 1.09E-07 | Down |
| RP11-505K9.3 | 3.286226063 | -1.315995908 | 0.343429 | -3.83193 | 0.000127 | 0.000459 | Down |
| RP11-506B6.6 | 8.458060616 | -1.136886354 | 0.313686 | -3.62428 | 0.00029 | 0.000973 | Down |
| RP11-506H21.5 | 4.625604221 | -1.271433138 | 0.228985 | -5.55247 | 2.82E-08 | 1.78E-07 | Down |
| RP11-506K6.4 | 28.29260999 | -1.332894193 | 0.14971 | -8.90315 | 5.43E-19 | 9.34E-18 | Down |
| RP11-507B12.1 | 1.57062835 | 3.175839765 | 0.577942 | 5.49508 | 3.91E-08 | 2.43E-07 | Up |
| RP11-507B12.2 | 15.102123 | 2.885358129 | 0.566731 | 5.091226 | 3.56E-07 | 1.95E-06 | Up |
| RP11-507K13.4 | 1.607598096 | -3.025291261 | 1.084413 | -2.7898 | 0.005274 | 0.012999 | Down |
| RP11-508O18.1 | 1.495291394 | -3.519079625 | 1.176107 | -2.99214 | 0.00277 | 0.007338 | Down |
| RP11-509E10.3 | 1.835956099 | -1.807746205 | 0.642437 | -2.81389 | 0.004895 | 0.012156 | Down |
| RP11-50C13.2 | 2.380652608 | -2.004443853 | 0.500078 | -4.00826 | 6.12E-05 | 0.000234 | Down |
| RP11-510N19.5 | 3.299359514 | 1.364831255 | 0.417557 | 3.268614 | 0.001081 | 0.003184 | Up |
| RP11-511B23.1 | 1.778719499 | -1.57169076 | 0.639697 | -2.45693 | 0.014013 | 0.030497 | Down |
| RP11-511B23.3 | 2.16213612 | -1.409286386 | 0.583918 | -2.4135 | 0.0158 | 0.033761 | Down |
| RP11-511I11.2 | 1.201228114 | -2.778644327 | 1.122064 | -2.47637 | 0.013273 | 0.029139 | Down |
| RP11-511P7.4 | 2.141531883 | -2.249519327 | 0.512203 | -4.39185 | 1.12E-05 | 4.92E-05 | Down |
| RP11-512C24.3 | 3.145611273 | -1.819773793 | 0.601923 | -3.02327 | 0.002501 | 0.006705 | Down |
| RP11-513G11.2 | 2.574866742 | -1.047524863 | 0.438272 | -2.39012 | 0.016843 | 0.035675 | Down |
| RP11-513O17.2 | 2.759293542 | -2.036448416 | 0.881479 | -2.31026 | 0.020874 | 0.042924 | Down |
| RP11-515O17.2 | 16.63800205 | -1.511945491 | 0.231833 | -6.52169 | 6.95E-11 | 5.87E-10 | Down |
| RP11-515O17.3 | 3.567984404 | -2.556837198 | 0.404485 | -6.32121 | 2.60E-10 | 2.07E-09 | Down |
| RP11-516A11.1 | 5.231766376 | -1.038703018 | 0.242386 | -4.28532 | 1.82E-05 | 7.72E-05 | Down |
| RP11-517C16.2 | 26.59360453 | -1.394082507 | 0.187437 | -7.43761 | 1.03E-13 | 1.14E-12 | Down |
| RP11-518L10.5 | 1.422105693 | -2.430380059 | 0.606688 | -4.00598 | 6.18E-05 | 0.000236 | Down |
| RP11-519G16.3 | 14.83827988 | 1.043937621 | 0.298443 | 3.497944 | 0.000469 | 0.001502 | Up |
| RP11-519G16.5 | 8.854466661 | 2.130775415 | 0.274939 | 7.749993 | 9.19E-15 | 1.13E-13 | Up |
| RP11-51C14.1 | 1.393930126 | -1.591005439 | 0.68537 | -2.32138 | 0.020266 | 0.041822 | Down |
| RP11-520B13.8 | 6.150726815 | -1.640360316 | 0.243195 | -6.74505 | 1.53E-11 | 1.37E-10 | Down |
| RP11-520D19.2 | 0.901464423 | -1.431799082 | 0.49248 | -2.90733 | 0.003645 | 0.009374 | Down |
| RP11-521D12.5 | 2.390191782 | -1.890809043 | 0.456247 | -4.14427 | 3.41E-05 | 0.000137 | Down |
| RP11-522B15.3 | 18.52870445 | -1.306292831 | 0.220116 | -5.93457 | 2.95E-09 | 2.09E-08 | Down |
| RP11-522B15.6 | 2.397850299 | -1.355398054 | 0.365287 | -3.71051 | 0.000207 | 0.000717 | Down |
| RP11-522L3.6 | 1.178071824 | -1.490707179 | 0.447339 | -3.33239 | 0.000861 | 0.002595 | Down |
| RP11-523L20.2 | 1.128282235 | -1.958113356 | 0.838011 | -2.33662 | 0.019459 | 0.040377 | Down |
| RP11-524H19.2 | 3.092471242 | -1.448804001 | 0.205865 | -7.03766 | 1.95E-12 | 1.92E-11 | Down |
| RP11-525A16.4 | 1.564100088 | 2.84551837 | 0.380809 | 7.472303 | 7.88E-14 | 8.85E-13 | Up |
| RP11-525G12.1 | 1.402031646 | -1.074989111 | 0.371596 | -2.89289 | 0.003817 | 0.009762 | Down |
| RP11-525K10.3 | 2.559618736 | -1.975967716 | 0.434902 | -4.54348 | 5.53E-06 | 2.55E-05 | Down |
| RP11-526I2.1 | 3.635029541 | -1.065114124 | 0.267512 | -3.98155 | 6.85E-05 | 0.00026 | Down |
| RP11-526K17.2 | 1.301770851 | -1.416903695 | 0.344817 | -4.10915 | 3.97E-05 | 0.000158 | Down |
| RP11-527H14.1 | 1.758514686 | -1.864240919 | 0.370902 | -5.02623 | 5.00E-07 | 2.69E-06 | Down |
| RP11-527L4.2 | 0.98782692 | -2.074053463 | 0.456503 | -4.54335 | 5.54E-06 | 2.55E-05 | Down |
| RP11-527L4.6 | 0.670616172 | -1.52035862 | 0.534711 | -2.84333 | 0.004465 | 0.011217 | Down |
| RP11-528A10.1 | 8.718806975 | -2.137488279 | 0.243916 | -8.76322 | 1.90E-18 | 3.12E-17 | Down |
| RP11-528A10.2 | 17.46364509 | -2.215357102 | 0.258617 | -8.56617 | 1.07E-17 | 1.66E-16 | Down |
| RP11-52L11.6 | 1.967363771 | -1.064557122 | 0.446672 | -2.38331 | 0.017158 | 0.036239 | Down |
| RP11-52L11.7 | 1.802482467 | -1.086174685 | 0.469979 | -2.31112 | 0.020826 | 0.042838 | Down |
| RP11-531A21.2 | 2.15548793 | -1.07152737 | 0.381497 | -2.80874 | 0.004974 | 0.012327 | Down |
| RP11-531A21.4 | 0.902779867 | -1.102148391 | 0.470869 | -2.34067 | 0.019249 | 0.039988 | Down |
| RP11-531A24.5 | 30.69217822 | -1.031540281 | 0.169862 | -6.07281 | 1.26E-09 | 9.35E-09 | Down |
| RP11-531F16.3 | 1.034501898 | -1.175337928 | 0.384008 | -3.06072 | 0.002208 | 0.006016 | Down |
| RP11-532N4.2 | 2.943879021 | -1.909813763 | 0.633176 | -3.01625 | 0.002559 | 0.006844 | Down |
| RP11-534L6.3 | 1.367332748 | -1.248820863 | 0.537718 | -2.32245 | 0.020209 | 0.041726 | Down |
| RP11-534P6.1 | 16.35192948 | -1.461626342 | 0.28315 | -5.16202 | 2.44E-07 | 1.37E-06 | Down |
| RP11-536C10.12 | 2.174542462 | -1.18050011 | 0.484883 | -2.43461 | 0.014908 | 0.032154 | Down |
| RP11-536G4.2 | 1.143595915 | -1.706722109 | 0.475127 | -3.59214 | 0.000328 | 0.001089 | Down |
| RP11-538D16.2 | 7.143043712 | -1.905359002 | 0.319303 | -5.96725 | 2.41E-09 | 1.73E-08 | Down |
| RP11-538D16.3 | 1.57883402 | -1.306445827 | 0.464777 | -2.81091 | 0.00494 | 0.012256 | Down |
| RP11-538P18.2 | 3.115861901 | -2.264705824 | 0.441404 | -5.13068 | 2.89E-07 | 1.60E-06 | Down |
| RP11-539E19.2 | 2.633020297 | -1.270115535 | 0.299649 | -4.23868 | 2.25E-05 | 9.36E-05 | Down |
| RP11-539G18.3 | 8.744729057 | -1.7648694 | 0.181364 | -9.7311 | 2.22E-22 | 4.93E-21 | Down |
| RP11-53I6.5 | 1.058683319 | -1.758468021 | 0.54218 | -3.24333 | 0.001181 | 0.003453 | Down |
| RP11-53M11.3 | 3.903124431 | 1.933244804 | 0.34367 | 5.625293 | 1.85E-08 | 1.20E-07 | Up |
| RP11-540A21.2 | 12.38793141 | -1.261366628 | 0.201055 | -6.27373 | 3.52E-10 | 2.78E-09 | Down |
| RP11-541G9.2 | 1.737591621 | -1.52269877 | 0.320172 | -4.75588 | 1.98E-06 | 9.75E-06 | Down |
| RP11-541H12.1 | 17.00351799 | 3.776111682 | 0.428499 | 8.812414 | 1.22E-18 | 2.05E-17 | Up |
| RP11-542B15.1 | 38.58690992 | 3.091329071 | 0.30292 | 10.20508 | 1.88E-24 | 4.82E-23 | Up |
| RP11-542M13.3 | 2.367382193 | -1.229467016 | 0.515685 | -2.38414 | 0.017119 | 0.03617 | Down |
| RP11-543H23.1 | 4.332363506 | -2.438049657 | 0.416725 | -5.8505 | 4.90E-09 | 3.39E-08 | Down |
| RP11-544I20.2 | 8.675834525 | -1.034907935 | 0.243485 | -4.25039 | 2.13E-05 | 8.92E-05 | Down |
| RP11-544L8_B.4 | 4.078928473 | -2.220943886 | 0.284259 | -7.81309 | 5.58E-15 | 7.01E-14 | Down |
| RP11-544M22.1 | 2.472292555 | -2.90888226 | 0.632078 | -4.6021 | 4.18E-06 | 1.96E-05 | Down |
| RP11-544M22.8 | 2.186741644 | 1.624976933 | 0.520678 | 3.120884 | 0.001803 | 0.005034 | Up |
| RP11-544P1.3 | 1.473392548 | -2.377718175 | 0.92893 | -2.55963 | 0.010478 | 0.023691 | Down |
| RP11-545A16.1 | 2.973238623 | -1.356278826 | 0.378683 | -3.58157 | 0.000342 | 0.001129 | Down |
| RP11-545A16.3 | 11.11005578 | -1.815195571 | 0.195657 | -9.27742 | 1.74E-20 | 3.35E-19 | Down |
| RP11-545A16.4 | 1.635442226 | -2.149345675 | 0.43393 | -4.95321 | 7.30E-07 | 3.83E-06 | Down |
| RP11-545D22.1 | 1.003630135 | -1.372564767 | 0.276928 | -4.9564 | 7.18E-07 | 3.77E-06 | Down |
| RP11-545M17.1 | 0.674005527 | -1.177171284 | 0.437669 | -2.68964 | 0.007153 | 0.016983 | Down |
| RP11-546B8.6 | 2.261146127 | -1.69425428 | 0.64962 | -2.60807 | 0.009105 | 0.020994 | Down |
| RP11-546K22.3 | 3.341311688 | -1.091628405 | 0.222574 | -4.90456 | 9.36E-07 | 4.84E-06 | Down |
| RP11-547C13.1 | 0.744612291 | -1.35141292 | 0.410958 | -3.28844 | 0.001007 | 0.00299 | Down |
| RP11-547C5.1 | 0.825313489 | 2.30394365 | 0.43951 | 5.242078 | 1.59E-07 | 9.13E-07 | Up |
| RP11-547D24.1 | 6.200090749 | 1.420958264 | 0.324973 | 4.37254 | 1.23E-05 | 5.35E-05 | Up |
| RP11-547D24.3 | 0.763402017 | -1.797952481 | 0.73596 | -2.443 | 0.014566 | 0.03152 | Down |
| RP11-548N1.1 | 1.158226165 | -1.70410007 | 0.533714 | -3.19291 | 0.001408 | 0.004037 | Down |
| RP11-549B18.1 | 14.69214761 | 1.175870706 | 0.163026 | 7.212766 | 5.48E-13 | 5.67E-12 | Up |
| RP11-54A9.1 | 1.436439591 | 2.713755449 | 0.39525 | 6.865927 | 6.61E-12 | 6.17E-11 | Up |
| RP11-54C4.2 | 1.547833999 | -1.352306358 | 0.548752 | -2.46433 | 0.013727 | 0.029969 | Down |
| RP11-54D18.4 | 2.001927102 | -1.676709783 | 0.459934 | -3.64555 | 0.000267 | 0.000904 | Down |
| RP11-550F7.1 | 4.520364568 | -1.175310097 | 0.303277 | -3.87537 | 0.000106 | 0.00039 | Down |
| RP11-551L14.1 | 11.83406376 | -1.268453612 | 0.27351 | -4.63769 | 3.52E-06 | 1.67E-05 | Down |
| RP11-552E4.3 | 4.277113635 | -1.17363339 | 0.245098 | -4.78843 | 1.68E-06 | 8.38E-06 | Down |
| RP11-553A21.3 | 13.15959314 | 1.17049725 | 0.247716 | 4.725153 | 2.30E-06 | 1.12E-05 | Up |
| RP11-553L6.3 | 7.145091534 | -1.050383009 | 0.407497 | -2.57764 | 0.009948 | 0.022652 | Down |
| RP11-553N16.1 | 1.962189444 | -1.110700251 | 0.382028 | -2.90738 | 0.003645 | 0.009373 | Down |
| RP11-554A11.4 | 6.14636162 | -1.260657725 | 0.228971 | -5.50575 | 3.68E-08 | 2.29E-07 | Down |
| RP11-554A11.8 | 1.557534409 | -1.870774227 | 0.557332 | -3.35666 | 0.000789 | 0.002396 | Down |
| RP11-554D14.2 | 2.142399111 | 1.186834952 | 0.370142 | 3.206432 | 0.001344 | 0.00387 | Up |
| RP11-554D15.1 | 0.52345731 | -1.51507681 | 0.453779 | -3.3388 | 0.000841 | 0.002541 | Down |
| RP11-554D15.3 | 1.636418356 | -2.175805444 | 0.684395 | -3.17917 | 0.001477 | 0.004212 | Down |
| RP11-554D20.2 | 26.11699165 | -1.161925644 | 0.210601 | -5.5172 | 3.44E-08 | 2.16E-07 | Down |
| RP11-554F20.1 | 4.697035836 | -1.681566987 | 0.254297 | -6.61261 | 3.78E-11 | 3.26E-10 | Down |
| RP11-554I8.1 | 1.699509064 | -1.309688088 | 0.418662 | -3.12827 | 0.001758 | 0.004925 | Down |
| RP11-554L12.1 | 0.465112718 | -1.531641433 | 0.586215 | -2.61276 | 0.008981 | 0.020745 | Down |
| RP11-555H23.1 | 1.337169117 | -2.897659018 | 0.877334 | -3.3028 | 0.000957 | 0.002856 | Down |
| RP11-555K12.1 | 1.614981875 | -1.189770904 | 0.419194 | -2.83824 | 0.004536 | 0.01138 | Down |
| RP11-555K2.4 | 1.253315301 | -2.331942806 | 1.02741 | -2.26973 | 0.023224 | 0.04699 | Down |
| RP11-556E13.1 | 0.577574282 | 1.47222545 | 0.514622 | 2.860789 | 0.004226 | 0.010684 | Up |
| RP11-556H2.1 | 8.02618592 | -1.619331556 | 0.253373 | -6.39111 | 1.65E-10 | 1.34E-09 | Down |
| RP11-556H2.3 | 1.049541812 | -1.164366814 | 0.430722 | -2.70329 | 0.006866 | 0.016392 | Down |
| RP11-556H2.4 | 0.840518012 | -1.540721406 | 0.36103 | -4.26757 | 1.98E-05 | 8.30E-05 | Down |
| RP11-556I13.2 | 3.832252623 | -1.537510416 | 0.371368 | -4.14012 | 3.47E-05 | 0.00014 | Down |
| RP11-556N21.1 | 2.042005444 | -1.363500763 | 0.422574 | -3.22666 | 0.001252 | 0.003638 | Down |
| RP11-556N4.1 | 1.602183935 | -1.272263238 | 0.54623 | -2.32917 | 0.01985 | 0.041074 | Down |
| RP11-557F20.2 | 1.884742061 | -1.038701782 | 0.458671 | -2.26459 | 0.023538 | 0.047542 | Down |
| RP11-557H15.3 | 1.488862242 | -1.846034235 | 0.634653 | -2.90873 | 0.003629 | 0.009339 | Down |
| RP11-557H15.4 | 3.358710728 | 1.114598005 | 0.218079 | 5.110991 | 3.20E-07 | 1.77E-06 | Up |
| RP11-558O12.1 | 0.905180198 | -1.574512179 | 0.531118 | -2.96452 | 0.003032 | 0.007961 | Down |
| RP11-559M23.1 | 3.099160894 | -1.915284508 | 0.413704 | -4.6296 | 3.66E-06 | 1.74E-05 | Down |
| RP11-559N14.5 | 5.708524753 | -1.886150717 | 0.380309 | -4.95952 | 7.07E-07 | 3.72E-06 | Down |
| RP11-560A15.6 | 5.278797661 | -2.220509212 | 0.280369 | -7.91995 | 2.38E-15 | 3.07E-14 | Down |
| RP11-560F18.1 | 2.71276703 | -2.116792284 | 0.640212 | -3.30639 | 0.000945 | 0.002824 | Down |
| RP11-560G2.1 | 1.450109106 | -2.082878984 | 0.582015 | -3.57874 | 0.000345 | 0.00114 | Down |
| RP11-560G2.2 | 2.387847809 | -1.589382681 | 0.472747 | -3.36202 | 0.000774 | 0.002354 | Down |
| RP11-560O20.1 | 2.359304166 | -3.061799223 | 0.668428 | -4.5806 | 4.64E-06 | 2.16E-05 | Down |
| RP11-563N4.1 | 5.03976941 | -1.170330046 | 0.186448 | -6.27696 | 3.45E-10 | 2.73E-09 | Down |
| RP11-564A8.4 | 3.872819944 | -1.11271687 | 0.422161 | -2.63576 | 0.008395 | 0.019548 | Down |
| RP11-564A8.8 | 3.084351509 | -1.786438022 | 0.525356 | -3.40043 | 0.000673 | 0.002078 | Down |
| RP11-564C24.1 | 1.29067713 | -2.315011402 | 0.720376 | -3.21362 | 0.001311 | 0.003788 | Down |
| RP11-565P22.6 | 4.65172827 | 1.67273335 | 0.251457 | 6.652163 | 2.89E-11 | 2.52E-10 | Up |
| RP11-566K19.11 | 0.548731465 | -1.405718939 | 0.550808 | -2.55211 | 0.010707 | 0.024138 | Down |
| RP11-566K19.5 | 1.11654666 | -1.597837128 | 0.471875 | -3.38615 | 0.000709 | 0.002178 | Down |
| RP11-566K19.6 | 9.534066988 | -2.134401212 | 0.217018 | -9.83512 | 7.95E-23 | 1.82E-21 | Down |
| RP11-567E21.3 | 2.343255589 | -1.005317254 | 0.382389 | -2.62904 | 0.008563 | 0.019893 | Down |
| RP11-567G24.1 | 3.209595385 | -1.704424198 | 0.388794 | -4.38388 | 1.17E-05 | 5.09E-05 | Down |
| RP11-567M16.2 | 1.20400858 | 1.413796698 | 0.437426 | 3.23208 | 0.001229 | 0.00358 | Up |
| RP11-567M16.3 | 0.999575544 | -1.675548146 | 0.535042 | -3.13162 | 0.001738 | 0.004874 | Down |
| RP11-567P17.1 | 0.775414503 | -1.629043054 | 0.659335 | -2.47074 | 0.013484 | 0.029535 | Down |
| RP11-568J23.8 | 1.480823741 | -1.628941436 | 0.302546 | -5.3841 | 7.28E-08 | 4.37E-07 | Down |
| RP11-569G13.3 | 2.549163401 | -3.395778706 | 0.83075 | -4.08761 | 4.36E-05 | 0.000172 | Down |
| RP11-56A10.1 | 0.668577479 | 1.42966835 | 0.559872 | 2.553563 | 0.010663 | 0.024053 | Up |
| RP11-56F10.3 | 0.686143946 | -1.828401908 | 0.477367 | -3.83018 | 0.000128 | 0.000462 | Down |
| RP11-56H16.1 | 1.964297146 | -3.085492655 | 1.070765 | -2.88158 | 0.003957 | 0.010075 | Down |
| RP11-570L14.1 | 4.190263943 | -1.233504432 | 0.386474 | -3.19169 | 0.001414 | 0.004052 | Down |
| RP11-571E6.4 | 1.330807391 | -2.658354834 | 0.725552 | -3.66391 | 0.000248 | 0.000846 | Down |
| RP11-572N21.1 | 1.682050273 | -3.167585543 | 1.060125 | -2.98794 | 0.002809 | 0.007433 | Down |
| RP11-573D15.2 | 2.970638923 | -1.107445551 | 0.394598 | -2.80652 | 0.005008 | 0.012404 | Down |
| RP11-573D15.8 | 3.714986071 | -1.219377427 | 0.477225 | -2.55514 | 0.010614 | 0.023959 | Down |
| RP11-573M3.3 | 6.412982063 | -1.006029189 | 0.196408 | -5.12214 | 3.02E-07 | 1.67E-06 | Down |
| RP11-573M3.6 | 0.689970801 | -1.215950702 | 0.46372 | -2.62217 | 0.008737 | 0.020248 | Down |
| RP11-574E24.3 | 12.03304935 | -1.689503673 | 0.390202 | -4.32982 | 1.49E-05 | 6.40E-05 | Down |
| RP11-574F11.3 | 2.799029737 | -1.049281918 | 0.389655 | -2.69285 | 0.007084 | 0.016837 | Down |
| RP11-574O16.1 | 1.610233988 | -2.587149003 | 0.637112 | -4.06074 | 4.89E-05 | 0.000191 | Down |
| RP11-575F12.2 | 1.013098836 | -1.496079476 | 0.428242 | -3.49354 | 0.000477 | 0.001525 | Down |
| RP11-575I8.1 | 1.683829618 | -1.878923129 | 0.638742 | -2.9416 | 0.003265 | 0.008503 | Down |
| RP11-575M22.1 | 7.497704779 | -1.503922845 | 0.292538 | -5.14094 | 2.73E-07 | 1.52E-06 | Down |
| RP11-576D8.4 | 3.964846457 | -1.785213293 | 0.34213 | -5.21794 | 1.81E-07 | 1.03E-06 | Down |
| RP11-577N1.2 | 1.448674475 | -2.967037562 | 1.28547 | -2.30813 | 0.020992 | 0.043132 | Down |
| RP11-578F21.10 | 1.815885052 | -1.7784837 | 0.47096 | -3.7763 | 0.000159 | 0.000565 | Down |
| RP11-578F21.6 | 6.331221867 | -1.909982697 | 0.254702 | -7.4989 | 6.44E-14 | 7.30E-13 | Down |
| RP11-57A19.2 | 7.156820311 | -1.163575205 | 0.215038 | -5.41102 | 6.27E-08 | 3.79E-07 | Down |
| RP11-57A19.5 | 4.7876251 | -1.473477127 | 0.347779 | -4.23682 | 2.27E-05 | 9.42E-05 | Down |
| RP11-57C13.6 | 1.320116104 | -2.816634511 | 0.392873 | -7.16932 | 7.54E-13 | 7.71E-12 | Down |
| RP11-57G10.8 | 5.609692004 | -1.506256581 | 0.204569 | -7.36308 | 1.80E-13 | 1.95E-12 | Down |
| RP11-580I1.2 | 3.138917299 | -1.879006868 | 0.307006 | -6.12043 | 9.33E-10 | 7.03E-09 | Down |
| RP11-583F24.4 | 1.377897732 | -2.493669267 | 0.902448 | -2.76323 | 0.005723 | 0.013955 | Down |
| RP11-583F24.5 | 1.503844343 | -3.111277983 | 0.959582 | -3.24233 | 0.001186 | 0.003464 | Down |
| RP11-583F24.7 | 1.411076868 | -2.503474224 | 0.789902 | -3.16935 | 0.001528 | 0.004343 | Down |
| RP11-584P21.2 | 3.291194203 | -1.508460918 | 0.34718 | -4.3449 | 1.39E-05 | 6.01E-05 | Down |
| RP11-586D19.1 | 2.926199518 | -1.35446378 | 0.517298 | -2.61834 | 0.008836 | 0.020451 | Down |
| RP11-586K12.2 | 2.386393571 | -1.207825714 | 0.472847 | -2.55437 | 0.010638 | 0.024004 | Down |
| RP11-588H23.5 | 1.887574819 | -3.20194344 | 0.843828 | -3.79454 | 0.000148 | 0.000528 | Down |
| RP11-589C21.5 | 2.30933764 | -1.23682898 | 0.32986 | -3.74955 | 0.000177 | 0.000623 | Down |
| RP11-589F5.4 | 2.039805426 | -1.939900645 | 0.861001 | -2.25308 | 0.024254 | 0.048812 | Down |
| RP11-58A18.2 | 0.726266608 | -1.382418678 | 0.613598 | -2.25297 | 0.024261 | 0.048821 | Down |
| RP11-58E21.1 | 0.969926176 | -1.937001814 | 0.696476 | -2.78115 | 0.005417 | 0.0133 | Down |
| RP11-58O9.2 | 18.39559233 | -1.249410686 | 0.209049 | -5.97665 | 2.28E-09 | 1.64E-08 | Down |
| RP11-593F23.1 | 1.606646605 | -2.017261298 | 0.497282 | -4.05658 | 4.98E-05 | 0.000194 | Down |
| RP11-594N15.3 | 33.67169245 | -1.083319357 | 0.159849 | -6.77713 | 1.23E-11 | 1.11E-10 | Down |
| RP11-595O22.1 | 2.387617458 | -2.047920656 | 0.551506 | -3.71332 | 0.000205 | 0.00071 | Down |
| RP11-596C23.2 | 12.33532482 | -1.160550727 | 0.182529 | -6.35817 | 2.04E-10 | 1.65E-09 | Down |
| RP11-598F7.3 | 3.083424543 | 1.661576711 | 0.393576 | 4.221743 | 2.42E-05 | 0.0001 | Up |
| RP11-598O12.1 | 2.413485064 | -2.440645975 | 0.339242 | -7.19442 | 6.27E-13 | 6.45E-12 | Down |
| RP11-599B13.7 | 0.523629637 | -1.057306763 | 0.416368 | -2.53936 | 0.011106 | 0.024922 | Down |
| RP11-59D5_B.2 | 2.162082504 | -2.223871186 | 0.618219 | -3.59722 | 0.000322 | 0.00107 | Down |
| RP11-5A19.5 | 23.78381712 | -1.182694092 | 0.218761 | -5.40632 | 6.43E-08 | 3.89E-07 | Down |
| RP11-5O23.2 | 26.53184654 | -2.678456278 | 0.244463 | -10.9565 | 6.19E-28 | 2.05E-26 | Down |
| RP11-5P18.5 | 2.343935733 | -2.759622508 | 0.44727 | -6.16992 | 6.83E-10 | 5.22E-09 | Down |
| RP11-5P22.1 | 1.200454857 | -1.186754895 | 0.39356 | -3.01544 | 0.002566 | 0.006859 | Down |
| RP11-600K15.1 | 2.018557881 | -1.832750356 | 0.605587 | -3.0264 | 0.002475 | 0.006649 | Down |
| RP11-602M11.3 | 2.917119377 | -1.153694506 | 0.371895 | -3.1022 | 0.001921 | 0.005324 | Down |
| RP11-604N13.1 | 1.088103332 | -1.412535555 | 0.614535 | -2.29854 | 0.021531 | 0.044057 | Down |
| RP11-605F14.2 | 1.428391195 | -2.983920831 | 1.213743 | -2.45845 | 0.013954 | 0.030397 | Down |
| RP11-609L3.2 | 1.802285632 | -1.469787534 | 0.541437 | -2.71461 | 0.006635 | 0.015907 | Down |
| RP11-60C6.5 | 3.732102519 | -1.008939526 | 0.355683 | -2.83663 | 0.004559 | 0.01143 | Down |
| RP11-60L3.1 | 22.37924304 | 3.442747096 | 0.349666 | 9.845827 | 7.14E-23 | 1.64E-21 | Up |
| RP11-614O9.1 | 0.546504622 | -1.834754304 | 0.698429 | -2.62697 | 0.008615 | 0.020002 | Down |
| RP11-615I2.2 | 11.42343386 | 1.752406071 | 0.200632 | 8.734412 | 2.45E-18 | 3.99E-17 | Up |
| RP11-615I2.3 | 3.013365282 | 1.177012684 | 0.361685 | 3.254246 | 0.001137 | 0.003333 | Up |
| RP11-615I2.6 | 6.265619442 | -1.455977815 | 0.318 | -4.57855 | 4.68E-06 | 2.18E-05 | Down |
| RP11-615J4.4 | 2.633894143 | -1.009042249 | 0.361993 | -2.78746 | 0.005312 | 0.013085 | Down |
| RP11-616K6.1 | 1.415456948 | -1.939741161 | 0.453879 | -4.2737 | 1.92E-05 | 8.10E-05 | Down |
| RP11-616M22.1 | 4.938682201 | -2.161255669 | 0.244192 | -8.85064 | 8.70E-19 | 1.48E-17 | Down |
| RP11-616M22.12 | 0.936632412 | -1.189342037 | 0.42729 | -2.78345 | 0.005378 | 0.013223 | Down |
| RP11-616M22.2 | 2.868248123 | -1.957439517 | 0.295559 | -6.62283 | 3.52E-11 | 3.05E-10 | Down |
| RP11-618N24.1 | 3.354783297 | -1.144470697 | 0.279148 | -4.09986 | 4.13E-05 | 0.000164 | Down |
| RP11-618P13.1 | 1.300124162 | -1.992687979 | 0.795372 | -2.50535 | 0.012233 | 0.027146 | Down |
| RP11-619A14.2 | 1.71155526 | -2.668310465 | 0.548693 | -4.86303 | 1.16E-06 | 5.90E-06 | Down |
| RP11-61J19.5 | 50.04351826 | -1.110252362 | 0.103432 | -10.7341 | 7.04E-27 | 2.15E-25 | Down |
| RP11-61K12.2 | 3.607735973 | -1.721326029 | 0.52039 | -3.30776 | 0.00094 | 0.002812 | Down |
| RP11-61L19.2 | 3.286429954 | -1.06438224 | 0.272459 | -3.90658 | 9.36E-05 | 0.000346 | Down |
| RP11-622C24.1 | 4.022839109 | -1.405947261 | 0.322047 | -4.36566 | 1.27E-05 | 5.51E-05 | Down |
| RP11-622C24.2 | 76.33542929 | -1.034634721 | 0.175503 | -5.89526 | 3.74E-09 | 2.62E-08 | Down |
| RP11-624A21.1 | 29.46806105 | -1.561414425 | 0.268733 | -5.81028 | 6.24E-09 | 4.26E-08 | Down |
| RP11-624L4.2 | 2.059602088 | -1.36720838 | 0.465296 | -2.93836 | 0.0033 | 0.008586 | Down |
| RP11-624M8.1 | 19.15932013 | 1.048630173 | 0.19094 | 5.491944 | 3.98E-08 | 2.47E-07 | Up |
| RP11-626E13.1 | 20.31046856 | -1.863949631 | 0.206665 | -9.01918 | 1.90E-19 | 3.39E-18 | Down |
| RP11-626G11.1 | 1.672174092 | -1.611016334 | 0.463372 | -3.47672 | 0.000508 | 0.001612 | Down |
| RP11-626G3.1 | 1.591436472 | -1.837988161 | 0.463139 | -3.96854 | 7.23E-05 | 0.000273 | Down |
| RP11-626H12.1 | 1.431833809 | 1.904618495 | 0.345407 | 5.51413 | 3.51E-08 | 2.19E-07 | Up |
| RP11-626H12.2 | 2.640237065 | 1.12954288 | 0.309537 | 3.649137 | 0.000263 | 0.000893 | Up |
| RP11-626H12.3 | 4.709358826 | -1.157634254 | 0.418974 | -2.76302 | 0.005727 | 0.013963 | Down |
| RP11-626I20.1 | 1.165834674 | -3.091827921 | 1.183088 | -2.61335 | 0.008966 | 0.020716 | Down |
| RP11-629F19.1 | 2.43451574 | -1.487246556 | 0.571458 | -2.60255 | 0.009253 | 0.021277 | Down |
| RP11-629G13.1 | 11.23359202 | -2.419419743 | 0.234796 | -10.3043 | 6.73E-25 | 1.79E-23 | Down |
| RP11-629N8.4 | 2.49339593 | -1.123115177 | 0.336631 | -3.33634 | 0.000849 | 0.002561 | Down |
| RP11-631M21.1 | 0.545336834 | -1.953903101 | 0.605827 | -3.22518 | 0.001259 | 0.003655 | Down |
| RP11-631M6.3 | 30.39263207 | -1.026538839 | 0.21382 | -4.80096 | 1.58E-06 | 7.90E-06 | Down |
| RP11-632K20.8 | 3.307685176 | -1.565413285 | 0.245759 | -6.36972 | 1.89E-10 | 1.54E-09 | Down |
| RP11-632K5.2 | 3.248133289 | -1.425261587 | 0.300438 | -4.74394 | 2.10E-06 | 1.03E-05 | Down |
| RP11-632K5.3 | 1.551750771 | -3.000045274 | 1.043557 | -2.87483 | 0.004042 | 0.010268 | Down |
| RP11-632P5.1 | 2.315834788 | -1.824619563 | 0.394011 | -4.63088 | 3.64E-06 | 1.73E-05 | Down |
| RP11-634B7.4 | 3.816784869 | -1.867531936 | 0.354861 | -5.26272 | 1.42E-07 | 8.22E-07 | Down |
| RP11-637N19.1 | 29.6436848 | -1.454949024 | 0.196762 | -7.39445 | 1.42E-13 | 1.56E-12 | Down |
| RP11-638F5.2 | 9.488582783 | -2.465217252 | 0.24928 | -9.88934 | 4.63E-23 | 1.08E-21 | Down |
| RP11-638I2.8 | 1.146917933 | 1.286190495 | 0.462227 | 2.782597 | 0.005393 | 0.013255 | Up |
| RP11-638L3.4 | 1.912870332 | -2.895175853 | 0.850454 | -3.40427 | 0.000663 | 0.002052 | Down |
| RP11-639F1.3 | 0.891765798 | -1.393338333 | 0.357829 | -3.89386 | 9.87E-05 | 0.000363 | Down |
| RP11-63B19.1 | 1.340075086 | -3.36886568 | 1.423335 | -2.36688 | 0.017939 | 0.037666 | Down |
| RP11-63E16.1 | 1.614942357 | -3.071194036 | 1.147209 | -2.6771 | 0.007426 | 0.017543 | Down |
| RP11-63E9.1 | 6.314928723 | -1.121563457 | 0.409362 | -2.73979 | 0.006148 | 0.014863 | Down |
| RP11-63I8.1 | 4.394259974 | -1.381397621 | 0.28974 | -4.76771 | 1.86E-06 | 9.23E-06 | Down |
| RP11-63N8.3 | 1.831596323 | -2.252748072 | 0.324975 | -6.93207 | 4.15E-12 | 3.95E-11 | Down |
| RP11-640N11.2 | 8.136289188 | -1.505302281 | 0.36812 | -4.08917 | 4.33E-05 | 0.000171 | Down |
| RP11-641C17.2 | 3.304312677 | -1.147146471 | 0.299656 | -3.82821 | 0.000129 | 0.000465 | Down |
| RP11-641C17.4 | 6.101969806 | -1.259900887 | 0.257106 | -4.90031 | 9.57E-07 | 4.94E-06 | Down |
| RP11-641J8.1 | 2.702340065 | -1.772963882 | 0.5224 | -3.39388 | 0.000689 | 0.002124 | Down |
| RP11-642D21.1 | 8.86096961 | -1.228832466 | 0.221117 | -5.5574 | 2.74E-08 | 1.74E-07 | Down |
| RP11-642D21.2 | 5.370276817 | -1.644087991 | 0.252809 | -6.50327 | 7.86E-11 | 6.61E-10 | Down |
| RP11-643A5.2 | 1.1740261 | -1.529321034 | 0.487347 | -3.13805 | 0.001701 | 0.004783 | Down |
| RP11-643C9.2 | 0.501685658 | 1.367754016 | 0.515126 | 2.655186 | 0.007926 | 0.018578 | Up |
| RP11-643G16.3 | 1.197450899 | -1.39073226 | 0.357123 | -3.89426 | 9.85E-05 | 0.000363 | Down |
| RP11-643G16.4 | 4.145688149 | -1.892664175 | 0.360695 | -5.24727 | 1.54E-07 | 8.90E-07 | Down |
| RP11-645N11.3 | 1.385954315 | -1.412722058 | 0.608459 | -2.3218 | 0.020244 | 0.041787 | Down |
| RP11-648O15.1 | 11.54597278 | -1.093814887 | 0.222749 | -4.91053 | 9.08E-07 | 4.70E-06 | Down |
| RP11-649A18.4 | 3.135911621 | -1.173330241 | 0.316327 | -3.70923 | 0.000208 | 0.00072 | Down |
| RP11-64B16.4 | 2.935354583 | -1.275226237 | 0.289571 | -4.40384 | 1.06E-05 | 4.68E-05 | Down |
| RP11-64C12.1 | 9.587072031 | -2.201821365 | 0.284417 | -7.74154 | 9.82E-15 | 1.21E-13 | Down |
| RP11-64C12.3 | 1.476057027 | -2.145489119 | 0.879164 | -2.44037 | 0.014672 | 0.031722 | Down |
| RP11-64D22.1 | 3.354599408 | -2.459398452 | 0.347241 | -7.08268 | 1.41E-12 | 1.41E-11 | Down |
| RP11-64D22.5 | 0.518137682 | -1.880808687 | 0.518062 | -3.63047 | 0.000283 | 0.000953 | Down |
| RP11-64D24.2 | 2.326660752 | -1.731275764 | 0.365848 | -4.73223 | 2.22E-06 | 1.09E-05 | Down |
| RP11-650L12.4 | 3.632026075 | -1.01693252 | 0.28981 | -3.50896 | 0.00045 | 0.001447 | Down |
| RP11-651L5.3 | 21.8620806 | -1.119076262 | 0.144557 | -7.74143 | 9.83E-15 | 1.21E-13 | Down |
| RP11-654A16.3 | 1.851518346 | -1.21416386 | 0.310793 | -3.90666 | 9.36E-05 | 0.000346 | Down |
| RP11-654E17.2 | 0.947111402 | -1.421380286 | 0.491217 | -2.89359 | 0.003809 | 0.009742 | Down |
| RP11-655B23.1 | 2.752798762 | -1.475377259 | 0.351157 | -4.20148 | 2.65E-05 | 0.000109 | Down |
| RP11-65D24.2 | 1.68001565 | -3.114680457 | 1.029488 | -3.02546 | 0.002483 | 0.006667 | Down |
| RP11-65E22.2 | 2.425414655 | -1.385132153 | 0.421459 | -3.28652 | 0.001014 | 0.003009 | Down |
| RP11-65I12.1 | 1497.438672 | -1.179666388 | 0.190561 | -6.19048 | 6.00E-10 | 4.61E-09 | Down |
| RP11-65J21.3 | 4.612051388 | -1.026838411 | 0.271428 | -3.78309 | 0.000155 | 0.000551 | Down |
| RP11-65J3.14 | 10.68360714 | -1.853237371 | 0.2365 | -7.83609 | 4.65E-15 | 5.88E-14 | Down |
| RP11-65J3.2 | 2.31906957 | -1.344868763 | 0.358831 | -3.74791 | 0.000178 | 0.000626 | Down |
| RP11-65J3.3 | 5.964183153 | -1.58135798 | 0.267112 | -5.9202 | 3.22E-09 | 2.27E-08 | Down |
| RP11-65M17.1 | 1.460712814 | -2.051265921 | 0.91206 | -2.24905 | 0.02451 | 0.049251 | Down |
| RP11-65M17.3 | 1.58994239 | -1.663110737 | 0.519809 | -3.19947 | 0.001377 | 0.003956 | Down |
| RP11-65N13.8 | 2.682171085 | -2.310060898 | 0.298592 | -7.73652 | 1.02E-14 | 1.25E-13 | Down |
| RP11-661A12.5 | 2.611123363 | -1.059072306 | 0.27233 | -3.88893 | 0.000101 | 0.00037 | Down |
| RP11-663N22.1 | 2.025457667 | -1.920213596 | 0.373957 | -5.13486 | 2.82E-07 | 1.57E-06 | Down |
| RP11-664I21.5 | 1.062882467 | -1.803304153 | 0.664626 | -2.71326 | 0.006662 | 0.015963 | Down |
| RP11-666A8.9 | 10.83106657 | -1.489172023 | 0.306928 | -4.85186 | 1.22E-06 | 6.22E-06 | Down |
| RP11-666O2.2 | 4.621746982 | -1.303667086 | 0.160328 | -8.13126 | 4.25E-16 | 5.80E-15 | Down |
| RP11-669C19.1 | 5.15796117 | -1.242880771 | 0.361776 | -3.4355 | 0.000591 | 0.00185 | Down |
| RP11-669I1.1 | 1.443897624 | -3.14994765 | 1.267958 | -2.48427 | 0.012982 | 0.028584 | Down |
| RP11-669N7.2 | 3.279717032 | -2.255699504 | 0.803881 | -2.80601 | 0.005016 | 0.012421 | Down |
| RP11-66B24.9 | 1.825018853 | 1.055512022 | 0.352304 | 2.99603 | 0.002735 | 0.007253 | Up |
| RP11-66D17.3 | 4.129553769 | -1.68026865 | 0.483554 | -3.47483 | 0.000511 | 0.001623 | Down |
| RP11-66D17.5 | 1.573211933 | -1.251307602 | 0.361328 | -3.46308 | 0.000534 | 0.001688 | Down |
| RP11-66N24.7 | 7.78616094 | -1.675311393 | 0.315173 | -5.31554 | 1.06E-07 | 6.25E-07 | Down |
| RP11-671J11.4 | 3.036242447 | -1.790556866 | 0.377574 | -4.74227 | 2.11E-06 | 1.04E-05 | Down |
| RP11-672A2.6 | 1.330893247 | -2.17200392 | 0.578541 | -3.75428 | 0.000174 | 0.000612 | Down |
| RP11-673E1.4 | 1.051748675 | -1.252814165 | 0.544413 | -2.30122 | 0.021379 | 0.043801 | Down |
| RP11-673E11.2 | 1.976146133 | -2.026448966 | 0.627566 | -3.22906 | 0.001242 | 0.003613 | Down |
| RP11-674E16.4 | 6.238922463 | -2.365945344 | 0.334175 | -7.07995 | 1.44E-12 | 1.44E-11 | Down |
| RP11-674N23.4 | 0.837472252 | -1.24324216 | 0.510156 | -2.43699 | 0.01481 | 0.031979 | Down |
| RP11-675F6.3 | 0.478512631 | 2.115986167 | 0.525604 | 4.025815 | 5.68E-05 | 0.000219 | Up |
| RP11-676B18.1 | 5.27721937 | -1.719076452 | 0.371494 | -4.62747 | 3.70E-06 | 1.75E-05 | Down |
| RP11-677M14.3 | 65.67474172 | -1.10606652 | 0.08813 | -12.5505 | 3.95E-36 | 2.20E-34 | Down |
| RP11-677M24.1 | 1.065735066 | 3.101237656 | 0.489754 | 6.332233 | 2.42E-10 | 1.94E-09 | Up |
| RP11-678G14.4 | 4.617226735 | -2.091585192 | 0.426012 | -4.90969 | 9.12E-07 | 4.72E-06 | Down |
| RP11-678L1.1 | 2.647142422 | -2.862699632 | 0.371679 | -7.70209 | 1.34E-14 | 1.62E-13 | Down |
| RP11-679C8.2 | 8.677657503 | -1.052737794 | 0.208321 | -5.05344 | 4.34E-07 | 2.35E-06 | Down |
| RP11-67H24.3 | 7.7143339 | -1.567386808 | 0.421195 | -3.72129 | 0.000198 | 0.00069 | Down |
| RP11-680B3.2 | 1.352931748 | -2.966125841 | 1.262927 | -2.34861 | 0.018844 | 0.039259 | Down |
| RP11-680C21.1 | 13.90121916 | 1.035605964 | 0.338792 | 3.056762 | 0.002237 | 0.006086 | Up |
| RP11-680F20.10 | 3.243942496 | -1.071797723 | 0.321543 | -3.3333 | 0.000858 | 0.002587 | Down |
| RP11-680F20.12 | 25.99801361 | -1.34572706 | 0.36339 | -3.70326 | 0.000213 | 0.000736 | Down |
| RP11-680F20.4 | 1.006348834 | -2.264082564 | 0.71988 | -3.14508 | 0.00166 | 0.004682 | Down |
| RP11-680F20.6 | 2.222568015 | -1.373905415 | 0.283807 | -4.84098 | 1.29E-06 | 6.56E-06 | Down |
| RP11-680F8.1 | 20.3463411 | -1.12946807 | 0.182567 | -6.18659 | 6.15E-10 | 4.72E-09 | Down |
| RP11-680G10.1 | 0.784702274 | -1.09415134 | 0.383586 | -2.85243 | 0.004339 | 0.010934 | Down |
| RP11-680H20.2 | 2.229565352 | -1.933007258 | 0.366877 | -5.26881 | 1.37E-07 | 7.97E-07 | Down |
| RP11-681L4.2 | 5.818184876 | -1.522118176 | 0.399225 | -3.81268 | 0.000137 | 0.000493 | Down |
| RP11-681L8.1 | 1.378690244 | -1.583802211 | 0.414579 | -3.82027 | 0.000133 | 0.000479 | Down |
| RP11-684B21.1 | 0.682748623 | -1.599998244 | 0.460538 | -3.4742 | 0.000512 | 0.001626 | Down |
| RP11-685B14.1 | 1.678811015 | -1.46487773 | 0.482626 | -3.03522 | 0.002404 | 0.00648 | Down |
| RP11-685B14.3 | 1.171114401 | -1.218966919 | 0.44797 | -2.72109 | 0.006507 | 0.015636 | Down |
| RP11-686D22.5 | 1.745530083 | -1.143914178 | 0.305307 | -3.74676 | 0.000179 | 0.000629 | Down |
| RP11-687F6.5 | 14.41697448 | -1.111221258 | 0.208746 | -5.32332 | 1.02E-07 | 6.00E-07 | Down |
| RP11-687M24.7 | 0.643053893 | -1.205173183 | 0.505829 | -2.38257 | 0.017192 | 0.036301 | Down |
| RP11-687M24.8 | 4.08509556 | -1.660527957 | 0.262313 | -6.33032 | 2.45E-10 | 1.96E-09 | Down |
| RP11-689K5.3 | 3.551013836 | 1.901540107 | 0.2498 | 7.612248 | 2.69E-14 | 3.17E-13 | Up |
| RP11-68E19.1 | 3.346538061 | -1.804431265 | 0.381982 | -4.72386 | 2.31E-06 | 1.13E-05 | Down |
| RP11-68L18.1 | 20.98487357 | -1.146619766 | 0.224859 | -5.09929 | 3.41E-07 | 1.88E-06 | Down |
| RP11-690P14.4 | 29.6677088 | 1.603015725 | 0.1502 | 10.67252 | 1.37E-26 | 4.10E-25 | Up |
| RP11-693J15.5 | 71.14747954 | -1.394247744 | 0.408192 | -3.41566 | 0.000636 | 0.001975 | Down |
| RP11-693M3.1 | 30.54089135 | -1.057587416 | 0.189652 | -5.57646 | 2.45E-08 | 1.57E-07 | Down |
| RP11-694D5.1 | 1.711747544 | 2.787617246 | 0.359887 | 7.745821 | 9.50E-15 | 1.17E-13 | Up |
| RP11-694I15.7 | 4.28369398 | -1.063291478 | 0.162639 | -6.53775 | 6.25E-11 | 5.30E-10 | Down |
| RP11-696D21.2 | 1.611910628 | -1.259460956 | 0.362981 | -3.46977 | 0.000521 | 0.00165 | Down |
| RP11-696N14.1 | 36.39895542 | -1.33561393 | 0.175525 | -7.60925 | 2.76E-14 | 3.24E-13 | Down |
| RP11-696P8.2 | 0.443854921 | 1.551646457 | 0.653727 | 2.373539 | 0.017619 | 0.037075 | Up |
| RP11-697M17.2 | 0.947730452 | -1.362010539 | 0.535701 | -2.54248 | 0.011007 | 0.02472 | Down |
| RP11-698E18.1 | 1.536374529 | -2.998137319 | 1.278095 | -2.34579 | 0.018987 | 0.039517 | Down |
| RP11-699L21.1 | 5.834447255 | -1.240520944 | 0.199642 | -6.21373 | 5.17E-10 | 4.01E-09 | Down |
| RP11-69I8.3 | 7.608732146 | -1.23261327 | 0.24171 | -5.09955 | 3.40E-07 | 1.87E-06 | Down |
| RP11-69J7.1 | 4.39472659 | -1.220268673 | 0.301984 | -4.04084 | 5.33E-05 | 0.000207 | Down |
| RP11-69M1.6 | 21.80181077 | -1.043731809 | 0.113605 | -9.18739 | 4.02E-20 | 7.56E-19 | Down |
| RP11-6B19.1 | 7.919343293 | 1.306817523 | 0.256059 | 5.103581 | 3.33E-07 | 1.84E-06 | Up |
| RP11-6F2.3 | 0.698804214 | -1.285079826 | 0.506692 | -2.53621 | 0.011206 | 0.025124 | Down |
| RP11-6F2.5 | 2.269534827 | -1.268934422 | 0.504164 | -2.51691 | 0.011839 | 0.026385 | Down |
| RP11-6L6.2 | 0.738797505 | 1.046892626 | 0.42932 | 2.438491 | 0.014749 | 0.031864 | Up |
| RP11-6N17.2 | 3.244901469 | -1.56610474 | 0.396567 | -3.94915 | 7.84E-05 | 0.000294 | Down |
| RP11-6O2.2 | 6.112079126 | -1.015408462 | 0.273209 | -3.7166 | 0.000202 | 0.000702 | Down |
| RP11-6O2.4 | 10.96337718 | -1.477476711 | 0.258984 | -5.70489 | 1.16E-08 | 7.74E-08 | Down |
| RP11-700H6.1 | 5.801709147 | -1.29847731 | 0.269542 | -4.81734 | 1.45E-06 | 7.32E-06 | Down |
| RP11-700H6.2 | 2.108518427 | -1.463993309 | 0.359528 | -4.07198 | 4.66E-05 | 0.000183 | Down |
| RP11-700H6.4 | 1.010824199 | -2.520384672 | 0.455346 | -5.53509 | 3.11E-08 | 1.96E-07 | Down |
| RP11-700P18.1 | 4.267855239 | -1.861443384 | 0.395908 | -4.70171 | 2.58E-06 | 1.25E-05 | Down |
| RP11-701H16.4 | 0.829044614 | 1.128409143 | 0.415568 | 2.71534 | 0.006621 | 0.015877 | Up |
| RP11-701P16.5 | 1.520408669 | 1.418871028 | 0.432506 | 3.280583 | 0.001036 | 0.003067 | Up |
| RP11-702H23.4 | 0.496050137 | -1.102455926 | 0.4693 | -2.34915 | 0.018816 | 0.039208 | Down |
| RP11-703G6.1 | 2.039220132 | -1.894705104 | 0.696583 | -2.72 | 0.006528 | 0.015682 | Down |
| RP11-703I16.1 | 5.278764786 | -1.229178406 | 0.170918 | -7.19163 | 6.40E-13 | 6.58E-12 | Down |
| RP11-704M14.1 | 3.591011555 | -2.659233255 | 0.427038 | -6.22716 | 4.75E-10 | 3.69E-09 | Down |
| RP11-705O1.8 | 13.99895053 | -1.094758935 | 0.182416 | -6.00144 | 1.96E-09 | 1.42E-08 | Down |
| RP11-707A18.1 | 21.57883628 | -2.961941777 | 0.307668 | -9.62706 | 6.15E-22 | 1.32E-20 | Down |
| RP11-707G18.1 | 7.474365194 | -1.045809007 | 0.192485 | -5.43319 | 5.54E-08 | 3.37E-07 | Down |
| RP11-707M1.1 | 1.36404342 | -1.546103754 | 0.649656 | -2.37988 | 0.017318 | 0.036528 | Down |
| RP11-707O23.1 | 6.866158333 | 1.255792166 | 0.158275 | 7.934227 | 2.12E-15 | 2.75E-14 | Up |
| RP11-708H21.1 | 0.663270218 | -2.018435745 | 0.550179 | -3.66869 | 0.000244 | 0.000832 | Down |
| RP11-709D24.5 | 1.624244686 | -2.126606196 | 0.71989 | -2.95407 | 0.003136 | 0.008207 | Down |
| RP11-70C1.1 | 5.141519087 | -1.943777951 | 0.2415 | -8.04879 | 8.36E-16 | 1.11E-14 | Down |
| RP11-70D24.1 | 5.841353725 | -2.015322117 | 0.351817 | -5.72832 | 1.01E-08 | 6.79E-08 | Down |
| RP11-70F11.11 | 1.672889123 | -2.078468973 | 0.901715 | -2.30502 | 0.021166 | 0.043417 | Down |
| RP11-70O5.2 | 1.803066431 | -1.315411192 | 0.40088 | -3.28131 | 0.001033 | 0.00306 | Down |
| RP11-710F7.3 | 6.914058205 | -1.549988235 | 0.31484 | -4.9231 | 8.52E-07 | 4.43E-06 | Down |
| RP11-713C5.1 | 7.691010742 | 1.444418835 | 0.302206 | 4.779584 | 1.76E-06 | 8.73E-06 | Up |
| RP11-713N11.4 | 0.498134321 | -1.247676614 | 0.443195 | -2.81519 | 0.004875 | 0.012115 | Down |
| RP11-713N11.6 | 12.75017897 | -1.071638733 | 0.220344 | -4.86348 | 1.15E-06 | 5.89E-06 | Down |
| RP11-713P17.3 | 16.82158626 | -1.313672166 | 0.213463 | -6.15409 | 7.55E-10 | 5.75E-09 | Down |
| RP11-713P17.4 | 4.610744053 | -1.088400933 | 0.242694 | -4.48466 | 7.30E-06 | 3.30E-05 | Down |
| RP11-714L20.1 | 2.69027173 | -1.461441836 | 0.511897 | -2.85495 | 0.004304 | 0.010857 | Down |
| RP11-715G18.3 | 1.642361867 | -1.741528104 | 0.437087 | -3.9844 | 6.77E-05 | 0.000257 | Down |
| RP11-715H19.2 | 1.560102089 | -2.662794549 | 0.328732 | -8.10019 | 5.49E-16 | 7.41E-15 | Down |
| RP11-715J22.2 | 9.229873442 | -1.762945461 | 0.277036 | -6.36359 | 1.97E-10 | 1.59E-09 | Down |
| RP11-716H6.1 | 1.479080396 | -2.461491775 | 0.494927 | -4.97344 | 6.58E-07 | 3.48E-06 | Down |
| RP11-716H6.2 | 1.017718385 | -1.435313201 | 0.46175 | -3.10842 | 0.001881 | 0.005229 | Down |
| RP11-718B12.5 | 2.855534045 | -1.083007752 | 0.334876 | -3.23405 | 0.00122 | 0.003558 | Down |
| RP11-719L21.1 | 1.322557262 | -2.703308088 | 1.010365 | -2.67557 | 0.00746 | 0.017611 | Down |
| RP11-71B7.1 | 9.618974544 | -2.599946278 | 0.265575 | -9.78987 | 1.24E-22 | 2.81E-21 | Down |
| RP11-71G12.1 | 1.287327808 | -1.5967801 | 0.556235 | -2.87069 | 0.004096 | 0.010385 | Down |
| RP11-71L14.3 | 8.096074259 | -1.536864906 | 0.313445 | -4.90314 | 9.43E-07 | 4.87E-06 | Down |
| RP11-71N10.1 | 2.091317645 | -2.666668523 | 0.928455 | -2.87216 | 0.004077 | 0.010344 | Down |
| RP11-720L2.2 | 1.141977008 | -1.238006428 | 0.34673 | -3.57052 | 0.000356 | 0.001173 | Down |
| RP11-720N19.2 | 1.900320593 | -1.094837548 | 0.321435 | -3.40609 | 0.000659 | 0.00204 | Down |
| RP11-723J4.3 | 8.834053515 | -1.063785598 | 0.259812 | -4.09444 | 4.23E-05 | 0.000167 | Down |
| RP11-723O4.2 | 5.952860081 | -1.036109239 | 0.195153 | -5.30922 | 1.10E-07 | 6.46E-07 | Down |
| RP11-723O4.9 | 4.253458967 | -1.279635031 | 0.216955 | -5.89817 | 3.68E-09 | 2.58E-08 | Down |
| RP11-724N1.1 | 1.756025316 | -1.401401376 | 0.440626 | -3.18047 | 0.00147 | 0.004195 | Down |
| RP11-725G5.2 | 2.117725696 | -3.417488739 | 0.746561 | -4.57764 | 4.70E-06 | 2.19E-05 | Down |
| RP11-725P16.2 | 4.883243845 | -1.857177561 | 0.319805 | -5.80722 | 6.35E-09 | 4.34E-08 | Down |
| RP11-727A23.10 | 10.17700777 | -1.736968504 | 0.293128 | -5.92564 | 3.11E-09 | 2.20E-08 | Down |
| RP11-728F11.4 | 20.4466744 | -2.247155992 | 0.175319 | -12.8176 | 1.31E-37 | 7.90E-36 | Down |
| RP11-728G15.1 | 1.174347293 | -2.809389649 | 1.066851 | -2.63335 | 0.008455 | 0.01967 | Down |
| RP11-728K20.2 | 30.07109883 | -1.15923002 | 0.24249 | -4.78052 | 1.75E-06 | 8.69E-06 | Down |
| RP11-72K17.2 | 1.678648681 | -1.636987846 | 0.631511 | -2.59218 | 0.009537 | 0.021855 | Down |
| RP11-730K11.1 | 9.258355979 | -1.439815492 | 0.319035 | -4.51303 | 6.39E-06 | 2.91E-05 | Down |
| RP11-730N24.1 | 1.617090955 | -3.453857897 | 1.386377 | -2.49128 | 0.012728 | 0.028115 | Down |
| RP11-731F5.1 | 5.764543367 | -1.226297483 | 0.405917 | -3.02105 | 0.002519 | 0.006747 | Down |
| RP11-731J8.1 | 1.859436429 | -1.049000957 | 0.350988 | -2.98871 | 0.002802 | 0.007416 | Down |
| RP11-732A19.9 | 0.484643202 | -1.33976956 | 0.50897 | -2.63232 | 0.00848 | 0.019722 | Down |
| RP11-732A21.2 | 3.854663127 | -1.191607044 | 0.353863 | -3.36743 | 0.000759 | 0.002314 | Down |
| RP11-733C7.1 | 0.947804973 | -1.718760139 | 0.455084 | -3.77679 | 0.000159 | 0.000564 | Down |
| RP11-734I18.1 | 1.307933975 | -2.058169702 | 0.773931 | -2.65937 | 0.007829 | 0.018371 | Down |
| RP11-736K20.4 | 173.2378404 | -1.152147258 | 0.103948 | -11.0839 | 1.50E-28 | 5.24E-27 | Down |
| RP11-736K20.5 | 82.4383024 | -1.070369247 | 0.09831 | -10.8877 | 1.32E-27 | 4.28E-26 | Down |
| RP11-736N17.10 | 2.677239379 | -1.534951702 | 0.352781 | -4.351 | 1.36E-05 | 5.85E-05 | Down |
| RP11-736N17.4 | 3.231817113 | -1.656475534 | 0.50165 | -3.30205 | 0.00096 | 0.002863 | Down |
| RP11-739P1.2 | 1.771862177 | -1.415474247 | 0.295564 | -4.78907 | 1.68E-06 | 8.35E-06 | Down |
| RP11-73B8.2 | 2.350399994 | -2.547253356 | 0.724876 | -3.51406 | 0.000441 | 0.001422 | Down |
| RP11-73K9.3 | 2.5346885 | -1.504110643 | 0.315866 | -4.76186 | 1.92E-06 | 9.48E-06 | Down |
| RP11-73M18.11 | 5.179815228 | -1.161557816 | 0.2454 | -4.73332 | 2.21E-06 | 1.08E-05 | Down |
| RP11-73M18.2 | 90.20108665 | 1.129072093 | 0.39344 | 2.869741 | 0.004108 | 0.010414 | Up |
| RP11-742B18.1 | 2.136205865 | 2.000368289 | 0.496377 | 4.029934 | 5.58E-05 | 0.000216 | Up |
| RP11-742D12.2 | 6.312913716 | -1.061484434 | 0.327431 | -3.24186 | 0.001188 | 0.003469 | Down |
| RP11-744J10.3 | 1.284609517 | -2.487407228 | 0.579185 | -4.29467 | 1.75E-05 | 7.42E-05 | Down |
| RP11-744N12.3 | 1.983535547 | -2.459618323 | 0.790067 | -3.11318 | 0.001851 | 0.005152 | Down |
| RP11-746P2.3 | 1.112986308 | -2.821809494 | 1.245764 | -2.26512 | 0.023505 | 0.047483 | Down |
| RP11-747H7.1 | 1.574059045 | -1.863483214 | 0.400138 | -4.6571 | 3.21E-06 | 1.54E-05 | Down |
| RP11-747H7.3 | 95.30677431 | -1.563055204 | 0.146958 | -10.636 | 2.03E-26 | 5.97E-25 | Down |
| RP11-748H22.1 | 9.207251458 | -1.197056224 | 0.178635 | -6.70114 | 2.07E-11 | 1.83E-10 | Down |
| RP11-748H22.2 | 1.618370451 | -3.159719475 | 1.323437 | -2.38751 | 0.016963 | 0.035895 | Down |
| RP11-748L13.6 | 2.667411825 | -1.64625323 | 0.428799 | -3.83922 | 0.000123 | 0.000446 | Down |
| RP11-749H17.1 | 3.113342214 | -2.449323086 | 0.533689 | -4.58942 | 4.44E-06 | 2.08E-05 | Down |
| RP11-749I16.3 | 7.485111387 | -1.059288802 | 0.286533 | -3.69692 | 0.000218 | 0.000753 | Down |
| RP11-74C1.4 | 3.995531638 | -1.159835598 | 0.233585 | -4.96536 | 6.86E-07 | 3.62E-06 | Down |
| RP11-74C13.4 | 2.597823512 | 1.284452435 | 0.36299 | 3.538537 | 0.000402 | 0.001308 | Up |
| RP11-74E22.3 | 6.467688987 | -1.04199907 | 0.141344 | -7.37206 | 1.68E-13 | 1.83E-12 | Down |
| RP11-74K11.2 | 0.961425081 | 1.594465159 | 0.379889 | 4.197188 | 2.70E-05 | 0.000111 | Up |
| RP11-74M13.4 | 2.68974248 | -1.229368307 | 0.43579 | -2.82101 | 0.004787 | 0.011934 | Down |
| RP11-752G15.4 | 1.601740869 | -2.764276169 | 0.697742 | -3.96174 | 7.44E-05 | 0.00028 | Down |
| RP11-752L20.5 | 1.295823377 | -2.513217559 | 0.952768 | -2.63781 | 0.008344 | 0.019441 | Down |
| RP11-755B10.3 | 4.562742338 | -2.523760768 | 0.372843 | -6.76897 | 1.30E-11 | 1.17E-10 | Down |
| RP11-755J8.1 | 7.656129741 | -1.658760903 | 0.291598 | -5.68851 | 1.28E-08 | 8.46E-08 | Down |
| RP11-756A22.7 | 1.226345241 | -1.806222385 | 0.563753 | -3.20393 | 0.001356 | 0.0039 | Down |
| RP11-756D7.2 | 1.852640291 | -1.944102306 | 0.686587 | -2.83155 | 0.004632 | 0.011591 | Down |
| RP11-756P10.2 | 1.888092191 | -1.248215181 | 0.480621 | -2.59709 | 0.009402 | 0.021584 | Down |
| RP11-756P10.7 | 1.561445761 | -3.189631598 | 1.405402 | -2.26955 | 0.023235 | 0.047009 | Down |
| RP11-757F18.3 | 4.601898599 | -1.350800906 | 0.319466 | -4.22831 | 2.35E-05 | 9.76E-05 | Down |
| RP11-757F18.5 | 106.2118314 | -1.310661879 | 0.184257 | -7.11323 | 1.13E-12 | 1.14E-11 | Down |
| RP11-757G1.6 | 25.01879476 | -1.012274452 | 0.185383 | -5.46046 | 4.75E-08 | 2.92E-07 | Down |
| RP11-757G14.3 | 1.396234299 | -1.106870292 | 0.421263 | -2.6275 | 0.008601 | 0.019973 | Down |
| RP11-758M2.1 | 4.116673164 | -1.918930563 | 0.483219 | -3.97114 | 7.15E-05 | 0.000271 | Down |
| RP11-758N13.1 | 3.877316445 | -1.03193823 | 0.361798 | -2.85225 | 0.004341 | 0.010939 | Down |
| RP11-759A24.1 | 19.5659763 | -1.22849653 | 0.223448 | -5.49791 | 3.84E-08 | 2.39E-07 | Down |
| RP11-759A24.2 | 9.306505858 | -1.182951672 | 0.24374 | -4.85334 | 1.21E-06 | 6.18E-06 | Down |
| RP11-75A9.1 | 1.62552217 | -3.237374035 | 1.226476 | -2.63957 | 0.008301 | 0.019353 | Down |
| RP11-760D2.5 | 3.589345208 | -1.66617899 | 0.606017 | -2.74939 | 0.005971 | 0.014489 | Down |
| RP11-760H22.2 | 86.12871906 | -1.582211541 | 0.129423 | -12.2251 | 2.28E-34 | 1.13E-32 | Down |
| RP11-761N21.1 | 1.258617927 | 2.21850861 | 0.423705 | 5.235977 | 1.64E-07 | 9.42E-07 | Up |
| RP11-766F14.2 | 6.498517262 | -1.166065087 | 0.334725 | -3.48365 | 0.000495 | 0.001575 | Down |
| RP11-767I20.1 | 1.708991801 | -2.742877326 | 0.963282 | -2.84743 | 0.004407 | 0.011091 | Down |
| RP11-768G7.1 | 1.432089706 | -1.505379599 | 0.446703 | -3.36998 | 0.000752 | 0.002295 | Down |
| RP11-76C10.3 | 9.153958942 | -1.231234709 | 0.21338 | -5.77016 | 7.92E-09 | 5.36E-08 | Down |
| RP11-76C10.4 | 6.222067473 | -1.648211545 | 0.269233 | -6.12189 | 9.25E-10 | 6.97E-09 | Down |
| RP11-76C10.5 | 17.11424616 | -1.275248811 | 0.188458 | -6.76676 | 1.32E-11 | 1.19E-10 | Down |
| RP11-76C10.6 | 25.58981286 | -1.198758512 | 0.168612 | -7.10958 | 1.16E-12 | 1.17E-11 | Down |
| RP11-76E16.2 | 2.462865076 | -1.793903859 | 0.448068 | -4.00365 | 6.24E-05 | 0.000238 | Down |
| RP11-76I14.1 | 8.496416742 | -1.119297122 | 0.337018 | -3.32118 | 0.000896 | 0.002692 | Down |
| RP11-770E5.1 | 2.47523065 | -1.979755885 | 0.628228 | -3.15133 | 0.001625 | 0.004592 | Down |
| RP11-770J1.4 | 2.525490832 | -1.676607007 | 0.435018 | -3.85411 | 0.000116 | 0.000422 | Down |
| RP11-770J1.7 | 1.264369035 | -1.025406568 | 0.434158 | -2.36183 | 0.018185 | 0.038099 | Down |
| RP11-771F20.1 | 1.681377429 | 1.113217026 | 0.473072 | 2.353167 | 0.018614 | 0.038864 | Up |
| RP11-771K4.1 | 1.478332972 | -1.854802336 | 0.617467 | -3.00389 | 0.002666 | 0.007091 | Down |
| RP11-774O3.3 | 194.9781591 | -1.012663024 | 0.094247 | -10.7448 | 6.27E-27 | 1.93E-25 | Down |
| RP11-775C24.3 | 4.964509029 | -1.691681532 | 0.331639 | -5.10097 | 3.38E-07 | 1.86E-06 | Down |
| RP11-775C24.4 | 6.206074607 | -1.389198332 | 0.321197 | -4.32507 | 1.52E-05 | 6.54E-05 | Down |
| RP11-775L16.1 | 1.810583321 | -2.558382703 | 0.94226 | -2.71515 | 0.006624 | 0.015883 | Down |
| RP11-776B3.1 | 1.784256612 | -1.37555133 | 0.318616 | -4.31727 | 1.58E-05 | 6.75E-05 | Down |
| RP11-776H12.1 | 3.624907417 | -2.896327288 | 0.296669 | -9.76282 | 1.63E-22 | 3.64E-21 | Down |
| RP11-777B9.1 | 2.06190093 | -1.368545633 | 0.350284 | -3.90696 | 9.35E-05 | 0.000346 | Down |
| RP11-778J16.2 | 2.02939288 | -2.242012988 | 0.611733 | -3.66502 | 0.000247 | 0.000843 | Down |
| RP11-779O18.1 | 0.619545913 | -1.598192575 | 0.513031 | -3.1152 | 0.001838 | 0.00512 | Down |
| RP11-779O18.2 | 1.44622469 | -1.62832817 | 0.364514 | -4.46712 | 7.93E-06 | 3.56E-05 | Down |
| RP11-77A13.1 | 2.623321479 | 2.171451848 | 0.714917 | 3.037349 | 0.002387 | 0.006444 | Up |
| RP11-77I22.2 | 3.313447343 | -1.30459787 | 0.254919 | -5.1177 | 3.09E-07 | 1.71E-06 | Down |
| RP11-77O7.1 | 3.618167266 | -1.164030825 | 0.27585 | -4.2198 | 2.45E-05 | 0.000101 | Down |
| RP11-781E19.1 | 1.516123927 | -2.821348423 | 0.850074 | -3.31894 | 0.000904 | 0.002711 | Down |
| RP11-783K16.10 | 6.371091482 | -2.256754573 | 0.257868 | -8.7516 | 2.10E-18 | 3.44E-17 | Down |
| RP11-787D11.1 | 1.714465873 | -2.051385312 | 0.306895 | -6.68433 | 2.32E-11 | 2.04E-10 | Down |
| RP11-78A19.4 | 1.704199717 | -1.10562626 | 0.372005 | -2.97207 | 0.002958 | 0.007787 | Down |
| RP11-78F17.1 | 2.448044649 | -1.131179021 | 0.352714 | -3.20708 | 0.001341 | 0.003863 | Down |
| RP11-791G16.2 | 4.943712801 | -1.297412592 | 0.308515 | -4.20535 | 2.61E-05 | 0.000107 | Down |
| RP11-791N19.1 | 2.029136862 | -1.953803935 | 0.593768 | -3.29052 | 0.001 | 0.00297 | Down |
| RP11-793H13.11 | 19.06607436 | -1.082421536 | 0.100933 | -10.7241 | 7.84E-27 | 2.39E-25 | Down |
| RP11-793H13.3 | 45.92880013 | -1.570712376 | 0.174092 | -9.0223 | 1.84E-19 | 3.29E-18 | Down |
| RP11-794C22.2 | 0.663355 | -1.567437615 | 0.438437 | -3.57506 | 0.00035 | 0.001155 | Down |
| RP11-794G24.1 | 5.296272467 | -1.640227035 | 0.340693 | -4.81439 | 1.48E-06 | 7.42E-06 | Down |
| RP11-794P6.1 | 3.381157113 | -1.958348572 | 0.39586 | -4.94708 | 7.53E-07 | 3.95E-06 | Down |
| RP11-794P6.6 | 1.174473966 | -2.345185562 | 0.801769 | -2.92501 | 0.003444 | 0.008918 | Down |
| RP11-795A2.2 | 1.587245358 | -3.191813705 | 1.285808 | -2.48234 | 0.013052 | 0.028725 | Down |
| RP11-797E24.1 | 1.101254899 | 1.985520508 | 0.50682 | 3.917605 | 8.94E-05 | 0.000332 | Up |
| RP11-798K23.3 | 2.784312601 | -2.163272058 | 0.638347 | -3.38887 | 0.000702 | 0.002159 | Down |
| RP11-798K3.2 | 0.938969457 | -2.282287134 | 0.898613 | -2.53979 | 0.011092 | 0.024893 | Down |
| RP11-798L4.1 | 2.197294007 | -1.414685052 | 0.480609 | -2.94352 | 0.003245 | 0.008455 | Down |
| RP11-798M19.6 | 26.65732515 | -1.151918325 | 0.111308 | -10.3489 | 4.23E-25 | 1.14E-23 | Down |
| RP11-799B12.2 | 3.908929772 | -1.024503743 | 0.364023 | -2.8144 | 0.004887 | 0.01214 | Down |
| RP11-799B12.4 | 1.882361761 | -1.37678362 | 0.389788 | -3.53214 | 0.000412 | 0.001337 | Down |
| RP11-79C6.2 | 0.417504707 | -1.472338519 | 0.595974 | -2.47047 | 0.013493 | 0.029552 | Down |
| RP11-79D8.2 | 6.317234684 | -1.001061573 | 0.213815 | -4.68191 | 2.84E-06 | 1.37E-05 | Down |
| RP11-79P5.3 | 0.973339316 | -2.438127698 | 1.022737 | -2.38393 | 0.017129 | 0.036187 | Down |
| RP11-7F17.3 | 8.500731339 | -1.221952671 | 0.238466 | -5.12422 | 2.99E-07 | 1.66E-06 | Down |
| RP11-7M10.2 | 2.390467857 | -1.075931483 | 0.357185 | -3.01225 | 0.002593 | 0.006923 | Down |
| RP11-802D6.1 | 1.292018028 | -1.671106001 | 0.382325 | -4.3709 | 1.24E-05 | 5.39E-05 | Down |
| RP11-806K15.1 | 0.904305562 | -2.082672923 | 0.493045 | -4.22411 | 2.40E-05 | 9.93E-05 | Down |
| RP11-806L2.2 | 10.61429199 | -1.669216864 | 0.208955 | -7.9884 | 1.37E-15 | 1.79E-14 | Down |
| RP11-807H7.2 | 3.141233267 | -2.537136107 | 0.321228 | -7.89825 | 2.83E-15 | 3.64E-14 | Down |
| RP11-809H16.4 | 1.94973865 | -2.973256706 | 0.952259 | -3.12232 | 0.001794 | 0.005014 | Down |
| RP11-809N15.3 | 1.443615779 | -2.990184092 | 1.15924 | -2.57944 | 0.009896 | 0.022552 | Down |
| RP11-80F22.10 | 1.180567929 | -2.479346899 | 0.874952 | -2.83369 | 0.004601 | 0.011519 | Down |
| RP11-80F22.11 | 1.348917341 | -3.133856571 | 1.299975 | -2.41071 | 0.015922 | 0.033978 | Down |
| RP11-80H5.2 | 0.636515974 | -1.365382882 | 0.471723 | -2.89446 | 0.003798 | 0.00972 | Down |
| RP11-80H5.9 | 3.681919679 | -1.448148146 | 0.287087 | -5.04428 | 4.55E-07 | 2.46E-06 | Down |
| RP11-80I3.1 | 1.635515675 | -1.545479793 | 0.568813 | -2.71703 | 0.006587 | 0.015808 | Down |
| RP11-811I15.1 | 0.925934431 | -1.779916464 | 0.480982 | -3.70059 | 0.000215 | 0.000743 | Down |
| RP11-812E19.3 | 1.687751115 | -2.709242135 | 1.112808 | -2.4346 | 0.014908 | 0.032154 | Down |
| RP11-815J4.7 | 2.949195599 | -1.426303271 | 0.458169 | -3.11305 | 0.001852 | 0.005153 | Down |
| RP11-815M8.1 | 0.99756024 | 2.816902714 | 0.482908 | 5.83321 | 5.44E-09 | 3.75E-08 | Up |
| RP11-817G13.3 | 1.123227236 | -1.45756901 | 0.410173 | -3.55355 | 0.00038 | 0.001244 | Down |
| RP11-817J15.2 | 4.712773576 | 2.637557864 | 0.348388 | 7.570757 | 3.71E-14 | 4.30E-13 | Up |
| RP11-817J15.3 | 2.981342103 | 2.922892566 | 0.43853 | 6.665213 | 2.64E-11 | 2.32E-10 | Up |
| RP11-818C3.1 | 0.460169037 | -1.442294097 | 0.572633 | -2.51871 | 0.011779 | 0.026268 | Down |
| RP11-81A1.7 | 1.893238857 | -2.813374861 | 0.708533 | -3.9707 | 7.17E-05 | 0.000271 | Down |
| RP11-81H3.2 | 1.299224198 | -3.049008394 | 1.045666 | -2.91585 | 0.003547 | 0.009152 | Down |
| RP11-820I16.1 | 0.430177709 | -1.269173378 | 0.519862 | -2.44137 | 0.014632 | 0.031645 | Down |
| RP11-820I16.3 | 0.913377364 | -1.599723983 | 0.470655 | -3.39893 | 0.000676 | 0.002089 | Down |
| RP11-822E23.5 | 1.329927622 | -3.049802033 | 1.259835 | -2.42079 | 0.015487 | 0.033197 | Down |
| RP11-823E8.3 | 147.6599122 | 1.398575888 | 0.146651 | 9.536739 | 1.47E-21 | 3.08E-20 | Up |
| RP11-826F13.1 | 12.03985475 | -2.139797609 | 0.251377 | -8.5123 | 1.71E-17 | 2.61E-16 | Down |
| RP11-82L18.2 | 8.177144706 | 1.504109767 | 0.214801 | 7.002334 | 2.52E-12 | 2.45E-11 | Up |
| RP11-830F9.5 | 12.50184677 | -1.020174537 | 0.28741 | -3.54954 | 0.000386 | 0.001261 | Down |
| RP11-832A4.7 | 18.35745267 | 1.090450766 | 0.194692 | 5.600913 | 2.13E-08 | 1.37E-07 | Up |
| RP11-834C11.11 | 7.399749385 | -1.075351126 | 0.320321 | -3.35711 | 0.000788 | 0.002393 | Down |
| RP11-834C11.8 | 0.582890176 | -1.170980355 | 0.494035 | -2.37024 | 0.017777 | 0.037353 | Down |
| RP11-837J7.3 | 5.004275341 | -1.397548887 | 0.24415 | -5.72414 | 1.04E-08 | 6.95E-08 | Down |
| RP11-839D17.3 | 3.297825733 | -1.64948042 | 0.227495 | -7.25062 | 4.15E-13 | 4.34E-12 | Down |
| RP11-83A16.1 | 3.33701469 | -1.307035912 | 0.308555 | -4.23599 | 2.28E-05 | 9.45E-05 | Down |
| RP11-83B20.3 | 11.42312894 | -1.065487459 | 0.256455 | -4.15468 | 3.26E-05 | 0.000132 | Down |
| RP11-83J16.3 | 7.134000514 | -1.01368485 | 0.215413 | -4.70577 | 2.53E-06 | 1.23E-05 | Down |
| RP11-83N9.6 | 6.095936145 | -1.516416373 | 0.24412 | -6.21177 | 5.24E-10 | 4.06E-09 | Down |
| RP11-844P9.2 | 10.89145582 | -1.92004487 | 0.280732 | -6.83943 | 7.95E-12 | 7.36E-11 | Down |
| RP11-84A19.2 | 1.73549548 | -1.78431885 | 0.692178 | -2.57783 | 0.009942 | 0.022642 | Down |
| RP11-84D1.2 | 2.12024457 | -3.096399152 | 0.888317 | -3.48569 | 0.000491 | 0.001565 | Down |
| RP11-84G21.1 | 43.80248475 | -1.059827331 | 0.111119 | -9.53774 | 1.46E-21 | 3.05E-20 | Down |
| RP11-850A17.1 | 9.281713003 | -1.268649167 | 0.186922 | -6.78706 | 1.14E-11 | 1.04E-10 | Down |
| RP11-851M3.1 | 1.757661427 | -1.490862887 | 0.359324 | -4.14908 | 3.34E-05 | 0.000135 | Down |
| RP11-855A2.1 | 15.44960858 | -1.143369163 | 0.236214 | -4.84039 | 1.30E-06 | 6.57E-06 | Down |
| RP11-855A2.5 | 3.003788097 | -2.138539865 | 0.305409 | -7.00221 | 2.52E-12 | 2.45E-11 | Down |
| RP11-856M7.6 | 1.178158146 | -2.491345102 | 0.983582 | -2.53293 | 0.011311 | 0.025333 | Down |
| RP11-857B24.1 | 0.594807512 | -1.312198833 | 0.505013 | -2.59835 | 0.009367 | 0.021513 | Down |
| RP11-857B24.5 | 0.69131411 | -1.288864615 | 0.412353 | -3.12564 | 0.001774 | 0.004966 | Down |
| RP11-85A1.3 | 5.825639138 | -1.704038311 | 0.207349 | -8.21823 | 2.07E-16 | 2.89E-15 | Down |
| RP11-85B7.2 | 13.16835922 | -2.553598648 | 0.308134 | -8.28729 | 1.16E-16 | 1.65E-15 | Down |
| RP11-85D18.1 | 0.894317816 | -1.700800259 | 0.400772 | -4.24381 | 2.20E-05 | 9.16E-05 | Down |
| RP11-85E16.1 | 2.123345601 | -3.116272079 | 0.6752 | -4.61533 | 3.92E-06 | 1.85E-05 | Down |
| RP11-85I17.2 | 6.82000357 | -1.709832607 | 0.361247 | -4.73314 | 2.21E-06 | 1.08E-05 | Down |
| RP11-85I21.1 | 3.873642665 | -1.434840818 | 0.326116 | -4.39978 | 1.08E-05 | 4.76E-05 | Down |
| RP11-862L9.3 | 3.589794162 | -1.423522353 | 0.256455 | -5.55077 | 2.84E-08 | 1.80E-07 | Down |
| RP11-863P13.4 | 15.01541271 | -1.147848694 | 0.215739 | -5.32054 | 1.03E-07 | 6.09E-07 | Down |
| RP11-863P13.5 | 1.158527468 | -1.09751744 | 0.359327 | -3.05437 | 0.002255 | 0.006126 | Down |
| RP11-864I4.4 | 0.733500423 | -1.549432566 | 0.625051 | -2.47889 | 0.013179 | 0.028962 | Down |
| RP11-864N7.4 | 2.938613798 | -1.375635317 | 0.50461 | -2.72613 | 0.006408 | 0.015428 | Down |
| RP11-867G23.1 | 3.339027519 | -1.434660864 | 0.235683 | -6.08724 | 1.15E-09 | 8.58E-09 | Down |
| RP11-86H7.6 | 3.291703008 | -2.644722453 | 0.487586 | -5.42412 | 5.82E-08 | 3.53E-07 | Down |
| RP11-86K22.1 | 1.400025122 | -1.930571716 | 0.450944 | -4.28118 | 1.86E-05 | 7.85E-05 | Down |
| RP11-873E20.1 | 9.15283765 | -1.05632486 | 0.202419 | -5.21852 | 1.80E-07 | 1.03E-06 | Down |
| RP11-874J12.4 | 1.279306848 | -1.611062282 | 0.479015 | -3.36328 | 0.00077 | 0.002345 | Down |
| RP11-875O11.2 | 3.372465288 | -1.352928727 | 0.45059 | -3.00257 | 0.002677 | 0.007119 | Down |
| RP11-875O11.3 | 1.92566653 | 1.670286848 | 0.411989 | 4.054202 | 5.03E-05 | 0.000196 | Up |
| RP11-876N24.1 | 5.148873956 | -1.560614327 | 0.447071 | -3.49076 | 0.000482 | 0.001539 | Down |
| RP11-879F14.2 | 7.1278952 | 1.229425609 | 0.215301 | 5.710277 | 1.13E-08 | 7.51E-08 | Up |
| RP11-87E22.1 | 3.690404332 | -1.363352993 | 0.333736 | -4.08512 | 4.41E-05 | 0.000174 | Down |
| RP11-87E22.2 | 18.26677466 | -1.352008342 | 0.246416 | -5.48668 | 4.10E-08 | 2.54E-07 | Down |
| RP11-87M18.2 | 1.338874464 | -3.684367398 | 1.502644 | -2.45192 | 0.01421 | 0.030855 | Down |
| RP11-881M11.4 | 1.505538869 | -1.267569603 | 0.410995 | -3.08415 | 0.002041 | 0.005615 | Down |
| RP11-882I15.1 | 1.287138231 | -1.221383488 | 0.370587 | -3.29581 | 0.000981 | 0.00292 | Down |
| RP11-883G14.1 | 1.264588037 | -2.517774569 | 0.734472 | -3.42801 | 0.000608 | 0.001897 | Down |
| RP11-885L14.1 | 5.479679192 | 1.459618682 | 0.411902 | 3.543611 | 0.000395 | 0.001287 | Up |
| RP11-885N19.6 | 0.751353235 | -1.712050098 | 0.448896 | -3.81391 | 0.000137 | 0.000491 | Down |
| RP11-889L3.4 | 1.251913852 | -2.050920782 | 0.879616 | -2.33161 | 0.019721 | 0.040853 | Down |
| RP11-88G17.6 | 11.97483857 | -1.005426171 | 0.180315 | -5.57593 | 2.46E-08 | 1.57E-07 | Down |
| RP11-88I18.2 | 84.84672549 | -1.016409582 | 0.121308 | -8.37872 | 5.35E-17 | 7.87E-16 | Down |
| RP11-88I18.3 | 3.806388185 | -1.988593293 | 0.264908 | -7.50672 | 6.06E-14 | 6.90E-13 | Down |
| RP11-893F2.14 | 2.259848626 | -1.73027457 | 0.523481 | -3.30533 | 0.000949 | 0.002833 | Down |
| RP11-893F2.6 | 0.370147479 | 1.578427283 | 0.599229 | 2.634098 | 0.008436 | 0.019631 | Up |
| RP11-89C3.3 | 1.596959404 | -1.332137184 | 0.367183 | -3.62799 | 0.000286 | 0.000961 | Down |
| RP11-8L2.1 | 2.895275478 | -3.478166028 | 0.555761 | -6.25839 | 3.89E-10 | 3.05E-09 | Down |
| RP11-90C4.2 | 3.199389797 | -1.219220804 | 0.331541 | -3.67744 | 0.000236 | 0.000807 | Down |
| RP11-90D11.1 | 1.804115547 | -1.745610669 | 0.500851 | -3.48529 | 0.000492 | 0.001566 | Down |
| RP11-90D4.3 | 2.56990188 | -1.19050389 | 0.319164 | -3.73007 | 0.000191 | 0.000668 | Down |
| RP11-90E9.1 | 1.222009386 | -1.663001059 | 0.467002 | -3.56101 | 0.000369 | 0.001213 | Down |
| RP11-90K6.1 | 2.88009512 | -1.774785003 | 0.399773 | -4.43948 | 9.02E-06 | 4.01E-05 | Down |
| RP11-91H12.1 | 2.174831381 | -1.011470936 | 0.301966 | -3.34962 | 0.000809 | 0.002451 | Down |
| RP11-91I20.3 | 2.230072258 | -2.388004483 | 0.842201 | -2.83543 | 0.004576 | 0.011465 | Down |
| RP11-91I8.2 | 4.473902363 | -2.382183099 | 0.429119 | -5.55133 | 2.84E-08 | 1.79E-07 | Down |
| RP11-91I8.3 | 5.779908634 | -1.350956602 | 0.22936 | -5.89011 | 3.86E-09 | 2.70E-08 | Down |
| RP11-91J3.1 | 1.110642094 | -1.455830307 | 0.529818 | -2.74779 | 0.006 | 0.014547 | Down |
| RP11-91P24.5 | 2.020993183 | -1.476096657 | 0.402811 | -3.66449 | 0.000248 | 0.000844 | Down |
| RP11-923I11.3 | 1.232855935 | -1.50393194 | 0.582608 | -2.58138 | 0.009841 | 0.022444 | Down |
| RP11-923I11.5 | 1.276792346 | 1.570438567 | 0.380744 | 4.12466 | 3.71E-05 | 0.000149 | Up |
| RP11-930O11.3 | 2.337861851 | -1.449212805 | 0.24611 | -5.88848 | 3.90E-09 | 2.72E-08 | Down |
| RP11-932O9.9 | 83.83402956 | -1.54613387 | 0.142613 | -10.8414 | 2.19E-27 | 7.00E-26 | Down |
| RP11-933H2.4 | 68.35706626 | -1.39690571 | 0.171004 | -8.16886 | 3.11E-16 | 4.29E-15 | Down |
| RP11-93B14.6 | 4.390893475 | -1.369057386 | 0.352187 | -3.8873 | 0.000101 | 0.000373 | Down |
| RP11-93G23.2 | 8.672505911 | -1.418963595 | 0.307846 | -4.60933 | 4.04E-06 | 1.90E-05 | Down |
| RP11-93I21.2 | 1.496993279 | -1.858247794 | 0.641328 | -2.8975 | 0.003762 | 0.009644 | Down |
| RP11-93K7.1 | 5.679560923 | -1.184123496 | 0.283451 | -4.17752 | 2.95E-05 | 0.00012 | Down |
| RP11-93O17.2 | 3.5241128 | -1.442066056 | 0.475826 | -3.03066 | 0.00244 | 0.006564 | Down |
| RP11-946P6.4 | 1.11873685 | 1.939205076 | 0.475424 | 4.078894 | 4.53E-05 | 0.000178 | Up |
| RP11-94C24.13 | 15.59475179 | -2.535235464 | 0.231548 | -10.9491 | 6.71E-28 | 2.22E-26 | Down |
| RP11-950C14.10 | 17.21903541 | -1.012000786 | 0.184909 | -5.47295 | 4.43E-08 | 2.73E-07 | Down |
| RP11-956J14.1 | 1.672989327 | -2.263401102 | 0.307502 | -7.3606 | 1.83E-13 | 1.98E-12 | Down |
| RP11-959F10.5 | 1.766556404 | -2.422889376 | 0.832351 | -2.9109 | 0.003604 | 0.009281 | Down |
| RP11-959I15.1 | 1.413332672 | -1.854345347 | 0.580002 | -3.19714 | 0.001388 | 0.003984 | Down |
| RP11-95G17.2 | 2.435611738 | -1.566072718 | 0.498572 | -3.14112 | 0.001683 | 0.004741 | Down |
| RP11-95P9.1 | 2.776305288 | -1.728869361 | 0.312912 | -5.52511 | 3.29E-08 | 2.07E-07 | Down |
| RP11-963H1.1 | 2.368634611 | -2.585520359 | 0.658737 | -3.92497 | 8.67E-05 | 0.000323 | Down |
| RP11-963H4.5 | 0.891329401 | -1.84973135 | 0.599761 | -3.08411 | 0.002042 | 0.005616 | Down |
| RP11-96B5.3 | 3.72915114 | -2.048203087 | 0.241624 | -8.47681 | 2.31E-17 | 3.50E-16 | Down |
| RP11-96B5.4 | 1.807283301 | -2.904058363 | 1.07 | -2.71407 | 0.006646 | 0.015927 | Down |
| RP11-96C23.13 | 23.72226892 | -1.148530709 | 0.234676 | -4.89411 | 9.87E-07 | 5.08E-06 | Down |
| RP11-96D1.3 | 1.88556649 | -1.048104432 | 0.430767 | -2.43311 | 0.01497 | 0.032254 | Down |
| RP11-96D1.5 | 2.35602977 | -1.084702643 | 0.320801 | -3.38123 | 0.000722 | 0.002213 | Down |
| RP11-96H17.1 | 5.899962899 | -1.435881783 | 0.353045 | -4.06713 | 4.76E-05 | 0.000187 | Down |
| RP11-96J19.1 | 0.696896592 | -1.762070724 | 0.526047 | -3.34964 | 0.000809 | 0.002451 | Down |
| RP11-96J19.4 | 1.572895118 | -3.158688521 | 1.268709 | -2.48969 | 0.012786 | 0.028226 | Down |
| RP11-972L6.2 | 0.857168026 | -1.121953075 | 0.434695 | -2.58101 | 0.009851 | 0.022463 | Down |
| RP11-972P1.10 | 1.344143594 | -1.556221488 | 0.335365 | -4.64038 | 3.48E-06 | 1.65E-05 | Down |
| RP11-972P1.11 | 7.309606021 | -1.181259664 | 0.215276 | -5.48718 | 4.08E-08 | 2.53E-07 | Down |
| RP11-972P1.7 | 56.25417402 | -1.056087211 | 0.185532 | -5.6922 | 1.25E-08 | 8.29E-08 | Down |
| RP11-973H7.1 | 3.005875553 | -1.434770257 | 0.486632 | -2.94837 | 0.003195 | 0.008341 | Down |
| RP11-973N13.2 | 2.104214139 | -2.346902789 | 0.626765 | -3.74447 | 0.000181 | 0.000634 | Down |
| RP11-974F13.5 | 65.74704883 | -1.519230396 | 0.195935 | -7.75375 | 8.92E-15 | 1.10E-13 | Down |
| RP11-976B16.1 | 8.171670388 | -1.074681536 | 0.237717 | -4.52084 | 6.16E-06 | 2.81E-05 | Down |
| RP11-978I15.10 | 0.960864859 | -1.49966615 | 0.493926 | -3.03622 | 0.002396 | 0.006462 | Down |
| RP11-97C18.1 | 1.557679299 | -3.104271215 | 1.241357 | -2.50071 | 0.012395 | 0.027462 | Down |
| RP11-989E6.10 | 5.937273584 | -1.935815114 | 0.751993 | -2.57425 | 0.010046 | 0.022843 | Down |
| RP11-989E6.13 | 7.567674025 | 1.100213844 | 0.193513 | 5.685466 | 1.30E-08 | 8.61E-08 | Up |
| RP11-989F5.1 | 1.419828864 | -1.333514871 | 0.369032 | -3.61355 | 0.000302 | 0.001011 | Down |
| RP11-98D18.9 | 20.96156519 | 1.211612862 | 0.142663 | 8.492841 | 2.02E-17 | 3.07E-16 | Up |
| RP11-993B23.3 | 0.961932037 | -1.007554225 | 0.442785 | -2.27549 | 0.022876 | 0.046387 | Down |
| RP11-99A1.2 | 1.670687689 | -1.917012377 | 0.785006 | -2.44204 | 0.014605 | 0.031595 | Down |
| RP11-9D8.1 | 0.784231137 | -1.296221108 | 0.47807 | -2.71136 | 0.006701 | 0.016048 | Down |
| RP11-9G1.3 | 2.890917889 | -1.125057032 | 0.369413 | -3.04552 | 0.002323 | 0.00629 | Down |
| RP11-9N12.3 | 0.526405631 | 1.560969744 | 0.557235 | 2.801278 | 0.00509 | 0.012588 | Up |
| RP11-9N20.3 | 0.85377854 | -1.129493914 | 0.495716 | -2.27851 | 0.022696 | 0.04609 | Down |
| RP13-1032I1.11 | 3.459096785 | -1.144512554 | 0.363409 | -3.14938 | 0.001636 | 0.00462 | Down |
| RP13-1039J1.4 | 11.89711387 | 1.179510587 | 0.328994 | 3.585203 | 0.000337 | 0.001115 | Up |
| RP13-13A3.1 | 1.102391846 | -1.619470708 | 0.415949 | -3.89344 | 9.88E-05 | 0.000364 | Down |
| RP13-143G15.4 | 16.55135095 | -1.061503283 | 0.244617 | -4.33945 | 1.43E-05 | 6.14E-05 | Down |
| RP13-15M17.1 | 2.880977224 | -1.711876495 | 0.277479 | -6.16938 | 6.86E-10 | 5.24E-09 | Down |
| RP13-16H11.8 | 5.530004386 | -1.561777884 | 0.292438 | -5.34054 | 9.27E-08 | 5.49E-07 | Down |
| RP13-192B19.2 | 10.18602962 | -1.713391786 | 0.306723 | -5.58612 | 2.32E-08 | 1.49E-07 | Down |
| RP13-20L14.1 | 1.061217026 | -1.751564977 | 0.401996 | -4.35717 | 1.32E-05 | 5.71E-05 | Down |
| RP13-212L9.1 | 0.701105677 | -1.648454862 | 0.673234 | -2.44856 | 0.014343 | 0.031102 | Down |
| RP13-225O21.5 | 1.61977128 | -2.108738993 | 0.530952 | -3.97162 | 7.14E-05 | 0.00027 | Down |
| RP13-238N7.2 | 1.239431251 | -3.152587634 | 1.254066 | -2.51389 | 0.011941 | 0.026587 | Down |
| RP13-262C2.3 | 1.099493484 | -1.235478716 | 0.54821 | -2.25366 | 0.024218 | 0.04875 | Down |
| RP13-266I11.1 | 2.949511458 | 1.744448432 | 0.551731 | 3.161774 | 0.001568 | 0.004447 | Up |
| RP13-270P17.1 | 11.24543706 | -1.105972729 | 0.160718 | -6.88146 | 5.92E-12 | 5.56E-11 | Down |
| RP13-383K5.4 | 1.579537371 | -1.39505436 | 0.410621 | -3.39742 | 0.00068 | 0.0021 | Down |
| RP13-395E19.2 | 8.504094315 | -1.178222887 | 0.289252 | -4.07335 | 4.63E-05 | 0.000182 | Down |
| RP13-395E19.4 | 3.285361778 | -1.668378879 | 0.356105 | -4.68508 | 2.80E-06 | 1.35E-05 | Down |
| RP13-415G19.2 | 3.628511642 | -1.27087993 | 0.378381 | -3.35873 | 0.000783 | 0.00238 | Down |
| RP13-465B17.5 | 1.725249313 | -1.079621234 | 0.310493 | -3.47712 | 0.000507 | 0.00161 | Down |
| RP13-467H17.1 | 9.848944687 | -1.768223759 | 0.208924 | -8.46347 | 2.60E-17 | 3.92E-16 | Down |
| RP13-487C10.1 | 4.222617829 | -1.604488015 | 0.337495 | -4.75411 | 1.99E-06 | 9.83E-06 | Down |
| RP13-497K6.1 | 1.249220589 | -2.512932071 | 0.455741 | -5.51395 | 3.51E-08 | 2.19E-07 | Down |
| RP13-49I15.6 | 0.668655207 | -1.421885832 | 0.495556 | -2.86927 | 0.004114 | 0.010428 | Down |
| RP13-507P19.2 | 1.89506619 | -1.913624211 | 0.596069 | -3.2104 | 0.001325 | 0.003825 | Down |
| RP13-514E23.1 | 22.91239006 | -1.139909815 | 0.116897 | -9.75142 | 1.82E-22 | 4.06E-21 | Down |
| RP13-514E23.2 | 2.528924491 | -2.028437645 | 0.412144 | -4.92168 | 8.58E-07 | 4.46E-06 | Down |
| RP13-516M14.2 | 6.564844934 | -1.15119682 | 0.220246 | -5.22686 | 1.72E-07 | 9.89E-07 | Down |
| RP13-516M14.4 | 5.105856286 | -1.736445638 | 0.296823 | -5.85011 | 4.91E-09 | 3.40E-08 | Down |
| RP13-554M15.5 | 2.248288682 | -1.093107141 | 0.332268 | -3.28983 | 0.001002 | 0.002976 | Down |
| RP13-580B18.2 | 2.174844076 | -2.027173587 | 0.712656 | -2.84453 | 0.004448 | 0.011178 | Down |
| RP13-580B18.4 | 13.75300146 | -1.889665626 | 0.394286 | -4.79262 | 1.65E-06 | 8.21E-06 | Down |
| RP13-616I3.1 | 5.510823293 | -1.501395439 | 0.222643 | -6.74353 | 1.55E-11 | 1.39E-10 | Down |
| RP13-638C3.5 | 6.338374599 | -1.173297833 | 0.288737 | -4.06356 | 4.83E-05 | 0.000189 | Down |
| RP13-650J16.1 | 2.347645526 | -1.785242003 | 0.325576 | -5.48333 | 4.17E-08 | 2.58E-07 | Down |
| RP13-735L24.1 | 8.032574755 | -1.266773361 | 0.298859 | -4.2387 | 2.25E-05 | 9.36E-05 | Down |
| RP13-766D20.4 | 1.706691 | -1.662051773 | 0.482398 | -3.4454 | 0.00057 | 0.001789 | Down |
| RP13-786C16.1 | 1.198831443 | -1.303695616 | 0.579898 | -2.24815 | 0.024567 | 0.049346 | Down |
| RP13-870H17.3 | 9.101162362 | -1.447515578 | 0.239813 | -6.03602 | 1.58E-09 | 1.16E-08 | Down |
| RP13-895J2.2 | 6.211789227 | -2.366392558 | 0.271372 | -8.7201 | 2.78E-18 | 4.51E-17 | Down |
| RP13-895J2.3 | 52.42136099 | -2.500136534 | 0.201143 | -12.4297 | 1.80E-35 | 9.63E-34 | Down |
| RP13-895J2.4 | 14.29337909 | -2.057632837 | 0.266425 | -7.72311 | 1.14E-14 | 1.39E-13 | Down |
| RP13-895J2.6 | 7.059129463 | -2.128422982 | 0.270944 | -7.85559 | 3.98E-15 | 5.06E-14 | Down |
| RP13-977J11.3 | 2.533218392 | -1.374670411 | 0.308502 | -4.45595 | 8.35E-06 | 3.74E-05 | Down |
| RP13-977J11.8 | 0.992843936 | -1.114270551 | 0.372113 | -2.99444 | 0.00275 | 0.007289 | Down |
| RP3-323B6.1 | 1.310205493 | -1.290879318 | 0.494634 | -2.60977 | 0.00906 | 0.020898 | Down |
| RP3-323K23.3 | 1.184593366 | -1.969889968 | 0.382097 | -5.15548 | 2.53E-07 | 1.42E-06 | Down |
| RP3-331H24.4 | 2.061874013 | -1.657023371 | 0.427461 | -3.87644 | 0.000106 | 0.000388 | Down |
| RP3-332B22.1 | 17.68320205 | -1.876192121 | 0.183122 | -10.2456 | 1.24E-24 | 3.22E-23 | Down |
| RP3-333H23.8 | 2.245177749 | -2.187577645 | 0.635885 | -3.44021 | 0.000581 | 0.001821 | Down |
| RP3-340B19.2 | 2.559892071 | -1.07924454 | 0.265122 | -4.07075 | 4.69E-05 | 0.000184 | Down |
| RP3-340B19.3 | 22.35739223 | -1.016970271 | 0.138543 | -7.34045 | 2.13E-13 | 2.29E-12 | Down |
| RP3-347M6.2 | 7.060981687 | -1.304352778 | 0.422851 | -3.08467 | 0.002038 | 0.005607 | Down |
| RP3-359N14.1 | 4.040237038 | -1.381455812 | 0.408722 | -3.37994 | 0.000725 | 0.002221 | Down |
| RP3-382I10.3 | 2.628256813 | -1.255069458 | 0.280998 | -4.46647 | 7.95E-06 | 3.57E-05 | Down |
| RP3-400B16.1 | 5.217819174 | -1.169568299 | 0.227727 | -5.13584 | 2.81E-07 | 1.56E-06 | Down |
| RP3-400B16.2 | 1.546123823 | -1.856737108 | 0.421559 | -4.40445 | 1.06E-05 | 4.66E-05 | Down |
| RP3-406P24.3 | 0.594372444 | -1.220497228 | 0.478474 | -2.55081 | 0.010747 | 0.024215 | Down |
| RP3-407E4.3 | 1.451098569 | -3.003889602 | 1.102468 | -2.7247 | 0.006436 | 0.015489 | Down |
| RP3-412A9.17 | 6.086165644 | -1.422233604 | 0.587177 | -2.42216 | 0.015429 | 0.033095 | Down |
| RP3-414A15.10 | 8.808151346 | -1.655397571 | 0.175911 | -9.4104 | 4.94E-21 | 9.93E-20 | Down |
| RP3-414A15.2 | 1.442636235 | -1.073890556 | 0.347201 | -3.09299 | 0.001982 | 0.005472 | Down |
| RP3-416J7.4 | 1.418182481 | -2.842339267 | 0.982947 | -2.89165 | 0.003832 | 0.009794 | Down |
| RP3-425C14.5 | 24.50054289 | -1.026150911 | 0.222588 | -4.61009 | 4.02E-06 | 1.90E-05 | Down |
| RP3-436N22.3 | 1.206872756 | -1.929124025 | 0.50969 | -3.7849 | 0.000154 | 0.000547 | Down |
| RP3-437I16.1 | 3.277625937 | -1.40097011 | 0.46709 | -2.99936 | 0.002706 | 0.007183 | Down |
| RP3-441A12.1 | 1.439701355 | -1.471046656 | 0.331588 | -4.43637 | 9.15E-06 | 4.07E-05 | Down |
| RP3-442L6.4 | 4.846778307 | -3.64744992 | 0.4244 | -8.59436 | 8.37E-18 | 1.31E-16 | Down |
| RP3-449M8.6 | 20.94027726 | 2.168213901 | 0.172608 | 12.56152 | 3.44E-36 | 1.92E-34 | Up |
| RP3-449M8.9 | 8.875789759 | 1.748556465 | 0.21321 | 8.201083 | 2.38E-16 | 3.32E-15 | Up |
| RP3-453C12.15 | 6.160239062 | -1.046640789 | 0.258002 | -4.05671 | 4.98E-05 | 0.000194 | Down |
| RP3-453C12.8 | 1.383962305 | -1.014671213 | 0.329895 | -3.07574 | 0.0021 | 0.005756 | Down |
| RP3-455J7.4 | 7.530627017 | -1.310074956 | 0.33745 | -3.88228 | 0.000103 | 0.00038 | Down |
| RP3-460G2.2 | 1.26934218 | 1.029067306 | 0.344853 | 2.984073 | 0.002844 | 0.007517 | Up |
| RP3-461P17.10 | 9.980235279 | -1.220256441 | 0.269247 | -4.53211 | 5.84E-06 | 2.68E-05 | Down |
| RP3-466P17.1 | 16.15303559 | -1.375235981 | 0.142392 | -9.65813 | 4.54E-22 | 9.81E-21 | Down |
| RP3-467K16.2 | 28.1244116 | -1.091291841 | 0.207488 | -5.25954 | 1.44E-07 | 8.36E-07 | Down |
| RP3-467N11.2 | 1.615765815 | -1.583086212 | 0.489814 | -3.23202 | 0.001229 | 0.00358 | Down |
| RP3-468B3.2 | 7.433396062 | -1.871299663 | 0.563314 | -3.32195 | 0.000894 | 0.002685 | Down |
| RP3-471C18.1 | 2.980808036 | -1.252031325 | 0.274286 | -4.56469 | 5.00E-06 | 2.32E-05 | Down |
| RP3-471C18.2 | 0.890491799 | -1.184245884 | 0.391354 | -3.02602 | 0.002478 | 0.006656 | Down |
| RP3-483K16.4 | 5.131431664 | -2.039019956 | 0.224443 | -9.0848 | 1.04E-19 | 1.90E-18 | Down |
| RP3-495K2.2 | 1.934605655 | -1.403063167 | 0.573729 | -2.44552 | 0.014465 | 0.031328 | Down |
| RP3-496C20.1 | 13.40049528 | -1.207210479 | 0.169242 | -7.13304 | 9.82E-13 | 9.95E-12 | Down |
| RP3-497J21.1 | 4.386597612 | -1.34218857 | 0.26174 | -5.12794 | 2.93E-07 | 1.63E-06 | Down |
| RP3-500L14.2 | 6.513109375 | -1.362184791 | 0.231411 | -5.88642 | 3.95E-09 | 2.76E-08 | Down |
| RP3-508D13.1 | 1.599215681 | -1.796037935 | 0.766674 | -2.34264 | 0.019148 | 0.03981 | Down |
| RP3-508I15.14 | 5.495404817 | -1.013194841 | 0.18487 | -5.48058 | 4.24E-08 | 2.62E-07 | Down |
| RP3-509I19.6 | 1.710078167 | -1.44403604 | 0.402712 | -3.58578 | 0.000336 | 0.001113 | Down |
| RP3-510D11.1 | 4.558419578 | 1.119284915 | 0.199293 | 5.616276 | 1.95E-08 | 1.26E-07 | Up |
| RP3-510D11.2 | 15.69052422 | 1.314098634 | 0.143934 | 9.129879 | 6.86E-20 | 1.27E-18 | Up |
| RP3-510H16.3 | 1.546543386 | -1.071047498 | 0.289279 | -3.70247 | 0.000214 | 0.000738 | Down |
| RP3-510O8.4 | 0.774153444 | -1.333323667 | 0.47712 | -2.79453 | 0.005198 | 0.012834 | Down |
| RP3-521E19.3 | 2.09586978 | -1.721136373 | 0.461775 | -3.72722 | 0.000194 | 0.000675 | Down |
| RP3-522D1.1 | 2.378392855 | 1.124104132 | 0.267597 | 4.200733 | 2.66E-05 | 0.000109 | Up |
| RP3-522J7.6 | 1.704101825 | -1.184588008 | 0.441591 | -2.68255 | 0.007306 | 0.017295 | Down |
| RP3-522P13.2 | 2.387999091 | -1.554400074 | 0.600397 | -2.58895 | 0.009627 | 0.022023 | Down |
| RP3-523C21.2 | 1.674016798 | -2.19350318 | 0.406505 | -5.39601 | 6.81E-08 | 4.10E-07 | Down |
| RP3-523C21.3 | 3.110139157 | -1.895510806 | 0.442866 | -4.2801 | 1.87E-05 | 7.89E-05 | Down |
| RP3-523E19.2 | 2.162474385 | -1.016319603 | 0.289655 | -3.50873 | 0.00045 | 0.001448 | Down |
| RP4-533D7.5 | 0.61497825 | -1.621576298 | 0.449562 | -3.60701 | 0.00031 | 0.001034 | Down |
| RP4-539M6.14 | 2.665020719 | -2.386361138 | 0.696526 | -3.42609 | 0.000612 | 0.001909 | Down |
| RP4-539M6.22 | 1.369342069 | -1.424672196 | 0.396653 | -3.59174 | 0.000328 | 0.00109 | Down |
| RP4-545C24.1 | 121.4920537 | -1.270499232 | 0.115485 | -11.0014 | 3.76E-28 | 1.27E-26 | Down |
| RP4-545K15.5 | 42.10381534 | 1.778864006 | 0.166415 | 10.6893 | 1.14E-26 | 3.44E-25 | Up |
| RP4-549L20.3 | 2.001772736 | -1.734043208 | 0.474408 | -3.65517 | 0.000257 | 0.000873 | Down |
| RP4-550H1.7 | 2.04271608 | -1.182072973 | 0.225507 | -5.24184 | 1.59E-07 | 9.14E-07 | Down |
| RP4-551E13.2 | 1.596471894 | -3.398902708 | 1.456721 | -2.33326 | 0.019635 | 0.040698 | Down |
| RP4-555D20.1 | 169.4938044 | -1.344312877 | 0.173874 | -7.73153 | 1.06E-14 | 1.30E-13 | Down |
| RP4-555D20.2 | 738.8643942 | -1.435229133 | 0.177885 | -8.0683 | 7.13E-16 | 9.55E-15 | Down |
| RP4-555D20.3 | 2.07081708 | -1.010626155 | 0.282447 | -3.57811 | 0.000346 | 0.001143 | Down |
| RP4-555D20.4 | 12.95257528 | -1.330844073 | 0.135398 | -9.82912 | 8.44E-23 | 1.92E-21 | Down |
| RP4-562J12.1 | 0.910735269 | -1.597565657 | 0.39824 | -4.01156 | 6.03E-05 | 0.000231 | Down |
| RP4-564M11.2 | 1.093675646 | 3.184362323 | 0.483171 | 6.590551 | 4.38E-11 | 3.77E-10 | Up |
| RP4-568B10.1 | 2.75523487 | -2.707929981 | 0.39795 | -6.8047 | 1.01E-11 | 9.27E-11 | Down |
| RP4-568F9.6 | 6.278774558 | -1.074311446 | 0.28148 | -3.81666 | 0.000135 | 0.000486 | Down |
| RP4-584D14.6 | 1.522102064 | -1.929105439 | 0.488316 | -3.95053 | 7.80E-05 | 0.000293 | Down |
| RP4-584D14.7 | 2.415464212 | -1.096404298 | 0.423641 | -2.58805 | 0.009652 | 0.022075 | Down |
| RP4-587D13.2 | 1.262015936 | -1.882544998 | 0.56567 | -3.32799 | 0.000875 | 0.002633 | Down |
| RP4-597J3.1 | 2.853910264 | 1.31680792 | 0.324704 | 4.055413 | 5.00E-05 | 0.000195 | Up |
| RP4-601P9.2 | 7.763358314 | -1.064081384 | 0.188772 | -5.63685 | 1.73E-08 | 1.13E-07 | Down |
| RP4-610C12.3 | 5.311741286 | 1.17523819 | 0.384847 | 3.053783 | 0.00226 | 0.006136 | Up |
| RP4-613A2.1 | 0.762891223 | -1.390585511 | 0.596136 | -2.33267 | 0.019666 | 0.040754 | Down |
| RP4-614C15.2 | 0.695157969 | -1.744992273 | 0.627432 | -2.78117 | 0.005416 | 0.0133 | Down |
| RP4-614C15.3 | 0.601661332 | -1.11277116 | 0.428303 | -2.5981 | 0.009374 | 0.021524 | Down |
| RP4-616B8.4 | 1.051407812 | -1.441986498 | 0.341904 | -4.21752 | 2.47E-05 | 0.000102 | Down |
| RP4-616B8.5 | 1.530102478 | -1.944518687 | 0.657538 | -2.95727 | 0.003104 | 0.008133 | Down |
| RP4-621B10.8 | 4.015547248 | -1.38943742 | 0.280261 | -4.95766 | 7.13E-07 | 3.75E-06 | Down |
| RP4-621N11.2 | 3.882375947 | -1.196376033 | 0.273722 | -4.37078 | 1.24E-05 | 5.39E-05 | Down |
| RP4-631H13.2 | 0.826645617 | -1.305462121 | 0.476765 | -2.73817 | 0.006178 | 0.01493 | Down |
| RP4-641G12.4 | 1.703373566 | -1.794229239 | 0.763181 | -2.35099 | 0.018724 | 0.039038 | Down |
| RP4-647J21.1 | 33.19004902 | 1.107261695 | 0.25632 | 4.319847 | 1.56E-05 | 6.68E-05 | Up |
| RP4-659I19.1 | 1.793125113 | -2.866344578 | 0.899592 | -3.18627 | 0.001441 | 0.00412 | Down |
| RP4-663N10.1 | 3.911846488 | -1.035795957 | 0.297164 | -3.4856 | 0.000491 | 0.001565 | Down |
| RP4-663N10.2 | 0.499895607 | 1.462746275 | 0.523922 | 2.791913 | 0.00524 | 0.012919 | Up |
| RP4-665J23.1 | 45.10209204 | -1.229764115 | 0.115235 | -10.6718 | 1.38E-26 | 4.13E-25 | Down |
| RP4-665N4.4 | 4.604023162 | -2.035257295 | 0.231787 | -8.78071 | 1.62E-18 | 2.69E-17 | Down |
| RP4-666F24.3 | 0.576771185 | -1.342382231 | 0.467471 | -2.87159 | 0.004084 | 0.01036 | Down |
| RP4-667H12.4 | 1.158496837 | -2.28425813 | 0.8626 | -2.64811 | 0.008094 | 0.018925 | Down |
| RP4-668J24.2 | 2.707101257 | -2.035423837 | 0.470178 | -4.32905 | 1.50E-05 | 6.42E-05 | Down |
| RP4-676L2.1 | 1.141841001 | 2.345235766 | 0.375239 | 6.249978 | 4.11E-10 | 3.21E-09 | Up |
| RP4-680D5.2 | 10.98411386 | -1.695523281 | 0.256667 | -6.60593 | 3.95E-11 | 3.41E-10 | Down |
| RP4-682C21.5 | 1.206737157 | -3.104809753 | 1.111464 | -2.79344 | 0.005215 | 0.012867 | Down |
| RP4-686C3.7 | 1.501222531 | -1.163755049 | 0.323399 | -3.59851 | 0.00032 | 0.001065 | Down |
| RP4-697P8.3 | 1.678983524 | -1.621930816 | 0.54869 | -2.95601 | 0.003116 | 0.00816 | Down |
| RP4-704D23.1 | 2.065940583 | 1.286855484 | 0.430856 | 2.986743 | 0.00282 | 0.007459 | Up |
| RP4-705D16.3 | 3.186302415 | -1.044536303 | 0.334477 | -3.1229 | 0.001791 | 0.005006 | Down |
| RP4-710M3.1 | 3.198630501 | -2.085283596 | 0.232514 | -8.96841 | 3.01E-19 | 5.29E-18 | Down |
| RP4-710M3.2 | 6.371321265 | -2.41264567 | 0.259926 | -9.28204 | 1.66E-20 | 3.21E-19 | Down |
| RP4-713A8.1 | 1.832301021 | -1.674015046 | 0.396615 | -4.22075 | 2.43E-05 | 0.000101 | Down |
| RP4-718D20.3 | 3.102808206 | -2.674765586 | 0.517014 | -5.17349 | 2.30E-07 | 1.30E-06 | Down |
| RP4-725G10.4 | 11.77298103 | -2.007567271 | 0.18897 | -10.6237 | 2.31E-26 | 6.79E-25 | Down |
| RP4-734G22.3 | 39.35875994 | -1.575096518 | 0.188148 | -8.3716 | 5.68E-17 | 8.34E-16 | Down |
| RP4-738P11.3 | 0.987477286 | -1.366565213 | 0.559717 | -2.44153 | 0.014625 | 0.031633 | Down |
| RP4-738P15.1 | 9.219902624 | -2.41685801 | 0.313658 | -7.7054 | 1.30E-14 | 1.58E-13 | Down |
| RP4-751H13.7 | 30.90814835 | -1.415439524 | 0.164325 | -8.61367 | 7.08E-18 | 1.12E-16 | Down |
| RP4-753D4.2 | 1.498088804 | -2.594482047 | 0.887104 | -2.92467 | 0.003448 | 0.008925 | Down |
| RP4-753M9.1 | 8.892972841 | -1.115962389 | 0.257014 | -4.34202 | 1.41E-05 | 6.08E-05 | Down |
| RP4-758J18.10 | 7.511446611 | 1.536447066 | 0.208177 | 7.380478 | 1.58E-13 | 1.72E-12 | Up |
| RP4-765H13.1 | 1.285598235 | 1.778940234 | 0.389656 | 4.56541 | 4.99E-06 | 2.31E-05 | Up |
| RP4-775C13.1 | 2.646118234 | -1.047311645 | 0.314553 | -3.32952 | 0.00087 | 0.00262 | Down |
| RP4-777L9.2 | 1.195247225 | -1.981636932 | 0.470231 | -4.21418 | 2.51E-05 | 0.000103 | Down |
| RP4-792G4.3 | 1.214251421 | -3.023878205 | 1.072412 | -2.8197 | 0.004807 | 0.011973 | Down |
| RP4-800M22.2 | 5.537660167 | -1.123176692 | 0.284717 | -3.94488 | 7.98E-05 | 0.000299 | Down |
| RP4-800O15.2 | 1.262274027 | -1.915453546 | 0.649873 | -2.94743 | 0.003204 | 0.008365 | Down |
| RP4-803J11.2 | 2.943211906 | -1.065682372 | 0.262365 | -4.06183 | 4.87E-05 | 0.00019 | Down |
| RP4-806M20.5 | 2.339528488 | -3.578849318 | 1.191117 | -3.00462 | 0.002659 | 0.007076 | Down |
| RP4-809F18.2 | 2.927901417 | 2.826795351 | 0.411933 | 6.862266 | 6.78E-12 | 6.32E-11 | Up |
| RP4-811H24.9 | 2.761507813 | -1.189359469 | 0.363019 | -3.2763 | 0.001052 | 0.003106 | Down |
| RP4-813D12.3 | 12.19235884 | -1.027811053 | 0.180711 | -5.6876 | 1.29E-08 | 8.51E-08 | Down |
| RP4-816N1.1 | 1.075160445 | -1.327797173 | 0.461178 | -2.87914 | 0.003988 | 0.010145 | Down |
| RP5-1018K9.1 | 1.129031511 | -2.546771418 | 0.547768 | -4.64936 | 3.33E-06 | 1.59E-05 | Down |
| RP5-1024C24.1 | 14.02073898 | -2.380211103 | 0.224915 | -10.5827 | 3.58E-26 | 1.04E-24 | Down |
| RP5-1024G6.7 | 8.248464156 | -1.816931193 | 0.26216 | -6.93061 | 4.19E-12 | 3.99E-11 | Down |
| RP5-1025A1.3 | 3.359360357 | -1.659602303 | 0.264884 | -6.2654 | 3.72E-10 | 2.93E-09 | Down |
| RP5-1027G4.3 | 3.984111815 | -2.009531172 | 0.376192 | -5.34176 | 9.20E-08 | 5.45E-07 | Down |
| RP5-1028K7.1 | 1.927920881 | -1.393510018 | 0.456173 | -3.05478 | 0.002252 | 0.00612 | Down |
| RP5-1037N22.2 | 2.779015111 | -1.100170618 | 0.349925 | -3.14402 | 0.001666 | 0.004697 | Down |
| RP5-1039K5.16 | 2.258680878 | -1.193610549 | 0.378051 | -3.15728 | 0.001593 | 0.004507 | Down |
| RP5-1039K5.18 | 1.669342196 | -2.962992046 | 0.960778 | -3.08395 | 0.002043 | 0.005618 | Down |
| RP5-1039K5.19 | 307.0608299 | -1.069247069 | 0.183766 | -5.81852 | 5.94E-09 | 4.07E-08 | Down |
| RP5-1042K10.10 | 1.52784231 | -1.139943201 | 0.421292 | -2.70583 | 0.006813 | 0.016287 | Down |
| RP5-1048B16.1 | 4.49748929 | -2.014288189 | 0.369453 | -5.45208 | 4.98E-08 | 3.05E-07 | Down |
| RP5-1049N15.2 | 3.495044819 | -2.527159505 | 0.386828 | -6.53303 | 6.45E-11 | 5.46E-10 | Down |
| RP5-1055C14.6 | 0.837385172 | -1.639175309 | 0.45185 | -3.6277 | 0.000286 | 0.000962 | Down |
| RP5-1056H1.2 | 8.991313315 | -2.486647163 | 0.273199 | -9.10196 | 8.87E-20 | 1.63E-18 | Down |
| RP5-1057B20.3 | 2.242457319 | -2.043436156 | 0.549581 | -3.71817 | 0.000201 | 0.000698 | Down |
| RP5-1057I20.2 | 1.109125972 | 1.624666183 | 0.482857 | 3.364696 | 0.000766 | 0.002333 | Up |
| RP5-1061H20.3 | 2.909474476 | -1.390861 | 0.455219 | -3.05537 | 0.002248 | 0.006112 | Down |
| RP5-1063M23.2 | 5.806268835 | -1.088569597 | 0.380973 | -2.85734 | 0.004272 | 0.010787 | Down |
| RP5-1065J22.2 | 0.758732984 | -1.531878047 | 0.618986 | -2.47482 | 0.01333 | 0.029251 | Down |
| RP5-1065J22.4 | 7.01261902 | -1.177051928 | 0.210555 | -5.59024 | 2.27E-08 | 1.45E-07 | Down |
| RP5-1065J22.8 | 18.47734514 | -1.084098189 | 0.126865 | -8.54532 | 1.28E-17 | 1.98E-16 | Down |
| RP5-1069C8.2 | 2.099212185 | -2.350958823 | 0.658265 | -3.57145 | 0.000355 | 0.00117 | Down |
| RP5-1073O3.2 | 0.582319217 | -1.151511642 | 0.488399 | -2.35773 | 0.018387 | 0.03847 | Down |
| RP5-1074L1.1 | 4.856397781 | -1.04107175 | 0.307395 | -3.38676 | 0.000707 | 0.002174 | Down |
| RP5-1092L12.2 | 3.190193354 | -1.880910834 | 0.49073 | -3.83289 | 0.000127 | 0.000457 | Down |
| RP5-1098D14.1 | 1.361514822 | -1.273620227 | 0.371853 | -3.42507 | 0.000615 | 0.001916 | Down |
| RP5-1102E8.3 | 1.051775474 | -1.092661862 | 0.374145 | -2.92042 | 0.003496 | 0.009031 | Down |
| RP5-1107A17.3 | 0.675503533 | -1.432844142 | 0.487539 | -2.93893 | 0.003293 | 0.008573 | Down |
| RP5-1107A17.4 | 11.95539174 | -1.447384984 | 0.252597 | -5.73002 | 1.00E-08 | 6.73E-08 | Down |
| RP5-1116H23.5 | 1.769812511 | -1.754104586 | 0.49889 | -3.51601 | 0.000438 | 0.001413 | Down |
| RP5-1119A7.14 | 0.558435639 | -1.182995387 | 0.403769 | -2.92988 | 0.003391 | 0.008793 | Down |
| RP5-1121E10.2 | 0.642111707 | -1.266664885 | 0.481691 | -2.62962 | 0.008548 | 0.019863 | Down |
| RP5-1158E12.3 | 0.856911078 | 1.492940401 | 0.512331 | 2.914015 | 0.003568 | 0.0092 | Up |
| RP5-1172N10.2 | 2.356759632 | -1.701154036 | 0.377877 | -4.50187 | 6.74E-06 | 3.06E-05 | Down |
| RP5-1181K21.4 | 1.258365068 | -2.517684481 | 1.101949 | -2.28476 | 0.022327 | 0.045445 | Down |
| RP5-1185H19.2 | 1.664790776 | -2.128243616 | 0.473992 | -4.49004 | 7.12E-06 | 3.22E-05 | Down |
| RP5-1185I7.1 | 3.381120283 | -3.408751217 | 0.82959 | -4.10896 | 3.97E-05 | 0.000158 | Down |
| RP5-1186N24.3 | 7.354371349 | -2.176128317 | 0.298867 | -7.28125 | 3.31E-13 | 3.49E-12 | Down |
| RP5-826L7.1 | 0.999674056 | -1.597708935 | 0.549307 | -2.90859 | 0.003631 | 0.009342 | Down |
| RP5-827C21.2 | 1.051177105 | -1.104151346 | 0.449482 | -2.4565 | 0.01403 | 0.030522 | Down |
| RP5-834N19.1 | 0.947303479 | -1.729165772 | 0.473026 | -3.65554 | 0.000257 | 0.000872 | Down |
| RP5-836J3.1 | 1.862718685 | -1.36482014 | 0.333372 | -4.09399 | 4.24E-05 | 0.000168 | Down |
| RP5-837I24.1 | 4.382909856 | -1.708184588 | 0.301289 | -5.66958 | 1.43E-08 | 9.39E-08 | Down |
| RP5-837I24.4 | 0.963944687 | -2.126104299 | 0.503071 | -4.22625 | 2.38E-05 | 9.84E-05 | Down |
| RP5-837I24.5 | 0.876919681 | -1.650073632 | 0.58185 | -2.83591 | 0.00457 | 0.011452 | Down |
| RP5-837M10.1 | 1.146771542 | -2.902701209 | 1.00705 | -2.88238 | 0.003947 | 0.010051 | Down |
| RP5-837O21.3 | 1.574108857 | -1.664567905 | 0.554822 | -3.00018 | 0.002698 | 0.007165 | Down |
| RP5-837O21.4 | 2.071998626 | -1.378917113 | 0.597833 | -2.30652 | 0.021081 | 0.043268 | Down |
| RP5-839B4.8 | 5.302561457 | 4.283095029 | 0.420116 | 10.19503 | 2.09E-24 | 5.32E-23 | Up |
| RP5-842K16.1 | 5.364906048 | -1.64248916 | 0.30419 | -5.39955 | 6.68E-08 | 4.03E-07 | Down |
| RP5-842K16.2 | 0.642268956 | -1.162922019 | 0.486876 | -2.38854 | 0.016916 | 0.035813 | Down |
| RP5-844F9.1 | 4.920802868 | -1.578262701 | 0.347109 | -4.54688 | 5.44E-06 | 2.51E-05 | Down |
| RP5-854E16.2 | 1.689728381 | -1.651101539 | 0.51374 | -3.21389 | 0.00131 | 0.003785 | Down |
| RP5-856G1.2 | 4.849901876 | -1.026029863 | 0.31195 | -3.28908 | 0.001005 | 0.002983 | Down |
| RP5-857K21.4 | 9.135919433 | -1.057440928 | 0.261058 | -4.0506 | 5.11E-05 | 0.000199 | Down |
| RP5-857K21.7 | 4.044756196 | -1.184303331 | 0.496257 | -2.38647 | 0.017011 | 0.035981 | Down |
| RP5-875H18.9 | 5.557896798 | 2.631713016 | 0.276404 | 9.521252 | 1.71E-21 | 3.57E-20 | Up |
| RP5-879K22.1 | 1.310538654 | -1.171763904 | 0.400002 | -2.9294 | 0.003396 | 0.008804 | Down |
| RP5-884C9.2 | 0.687142324 | 1.611563145 | 0.439742 | 3.664791 | 0.000248 | 0.000844 | Up |
| RP5-886K2.1 | 4.203672651 | -1.005099744 | 0.346642 | -2.89954 | 0.003737 | 0.009586 | Down |
| RP5-890O15.3 | 4.527020091 | -1.421195176 | 0.279251 | -5.08932 | 3.59E-07 | 1.97E-06 | Down |
| RP5-899B16.1 | 5.179789731 | -1.876026441 | 0.355046 | -5.28389 | 1.26E-07 | 7.37E-07 | Down |
| RP5-907D15.4 | 3.917562443 | 1.875287185 | 0.307647 | 6.095577 | 1.09E-09 | 8.16E-09 | Up |
| RP5-908M14.5 | 2.386361773 | -1.147452219 | 0.289671 | -3.96122 | 7.46E-05 | 0.000281 | Down |
| RP5-912I13.1 | 1.629273596 | -2.846728989 | 1.219111 | -2.33509 | 0.019539 | 0.040528 | Down |
| RP5-915N17.11 | 3.442975324 | -1.127169741 | 0.264821 | -4.25635 | 2.08E-05 | 8.70E-05 | Down |
| RP5-928E24.2 | 1.051785283 | -2.530950326 | 0.97752 | -2.58915 | 0.009621 | 0.022016 | Down |
| RP5-933K21.2 | 1.502861876 | -2.026692686 | 0.478097 | -4.23908 | 2.24E-05 | 9.34E-05 | Down |
| RP5-934G17.6 | 2.412395991 | -2.275127045 | 0.52614 | -4.32418 | 1.53E-05 | 6.56E-05 | Down |
| RP5-935K16.2 | 2.76702534 | -1.190913604 | 0.419991 | -2.83557 | 0.004574 | 0.011461 | Down |
| RP5-943J3.2 | 11.07610912 | -1.31075723 | 0.131802 | -9.94489 | 2.65E-23 | 6.28E-22 | Down |
| RP5-952N6.1 | 1.672186517 | 1.964774833 | 0.488118 | 4.025203 | 5.69E-05 | 0.00022 | Up |
| RP5-965F6.2 | 1.204103484 | -1.656025749 | 0.326911 | -5.06567 | 4.07E-07 | 2.22E-06 | Down |
| RP5-965G21.4 | 7.227122781 | -1.113308335 | 0.280373 | -3.97082 | 7.16E-05 | 0.000271 | Down |
| RP5-971N18.3 | 1.277622723 | -1.193011505 | 0.486559 | -2.45193 | 0.014209 | 0.030855 | Down |
| RP5-978I12.1 | 1.275868991 | -2.181179033 | 0.633184 | -3.44478 | 0.000572 | 0.001792 | Down |
| RP5-981L23.2 | 6.833466972 | -1.113423876 | 0.237952 | -4.6792 | 2.88E-06 | 1.39E-05 | Down |
| RP5-984P4.6 | 1.828847091 | -2.238397731 | 0.708105 | -3.16111 | 0.001572 | 0.004454 | Down |
| RP5-997D24.3 | 4.534974996 | -1.145816996 | 0.293641 | -3.90211 | 9.54E-05 | 0.000352 | Down |
| RP6-186E3.1 | 1.218918041 | -2.521947519 | 0.834693 | -3.02141 | 0.002516 | 0.006741 | Down |
| RP6-191P20.3 | 0.537461427 | 1.379760291 | 0.588583 | 2.344206 | 0.019068 | 0.039661 | Up |
| RP6-201G10.2 | 5.115226068 | -1.50747458 | 0.226947 | -6.64242 | 3.09E-11 | 2.69E-10 | Down |
| RP6-218J18.2 | 1.749409433 | -2.27738091 | 0.600599 | -3.79185 | 0.00015 | 0.000533 | Down |
| RP6-24A23.3 | 21.77389306 | -1.633922496 | 0.186786 | -8.74758 | 2.18E-18 | 3.56E-17 | Down |
| RP6-24A23.6 | 2.781916153 | -2.022863596 | 0.332655 | -6.08097 | 1.19E-09 | 8.90E-09 | Down |
| RP6-24A23.7 | 67.28647373 | -2.20938482 | 0.198379 | -11.1372 | 8.27E-29 | 2.94E-27 | Down |
| RP6-24A23.8 | 6.197255401 | -2.331027839 | 0.263453 | -8.84799 | 8.91E-19 | 1.51E-17 | Down |
| RP6-60B16.1 | 0.842720596 | -1.947513914 | 0.663018 | -2.93735 | 0.00331 | 0.008611 | Down |
| RP6-65G23.5 | 1.494238865 | 1.333759975 | 0.270478 | 4.931128 | 8.18E-07 | 4.26E-06 | Up |
| RP6-99M1.3 | 17.38670607 | 1.662335865 | 0.233686 | 7.113542 | 1.13E-12 | 1.14E-11 | Up |
| RPA2P3 | 0.535606171 | -1.513510647 | 0.614051 | -2.4648 | 0.013709 | 0.029938 | Down |
| RPL10P15 | 0.41576862 | -1.24780199 | 0.495134 | -2.52013 | 0.011731 | 0.026176 | Down |
| RPL12P24 | 5.419749294 | -1.357908939 | 0.242446 | -5.60087 | 2.13E-08 | 1.37E-07 | Down |
| RPL12P38 | 1.743776369 | -1.312293024 | 0.530516 | -2.47362 | 0.013375 | 0.02934 | Down |
| RPL13AP17 | 2.807362584 | -1.341353567 | 0.353904 | -3.79017 | 0.000151 | 0.000536 | Down |
| RPL13AP20 | 6.92893151 | 1.236144279 | 0.1974 | 6.262117 | 3.80E-10 | 2.98E-09 | Up |
| RPL15P4 | 0.659835441 | -1.220158027 | 0.465403 | -2.62172 | 0.008749 | 0.020271 | Down |
| RPL17P3 | 34.89668952 | -1.016793776 | 0.24784 | -4.10262 | 4.08E-05 | 0.000162 | Down |
| RPL21P43 | 2.196980379 | 2.871729693 | 0.402264 | 7.138913 | 9.41E-13 | 9.55E-12 | Up |
| RPL23AP10 | 0.91279444 | -1.470997996 | 0.436866 | -3.36716 | 0.000759 | 0.002316 | Down |
| RPL23AP81 | 6.064330689 | -1.124152951 | 0.236867 | -4.74592 | 2.08E-06 | 1.02E-05 | Down |
| RPL30P11 | 0.984769563 | -1.412102623 | 0.456788 | -3.09137 | 0.001992 | 0.005498 | Down |
| RPL31P52 | 2.619942077 | -1.047502241 | 0.296264 | -3.53571 | 0.000407 | 0.001321 | Down |
| RPL34-AS1 | 9.767258219 | -1.445573923 | 0.184619 | -7.83003 | 4.88E-15 | 6.16E-14 | Down |
| RPL35P6 | 2.63473681 | 1.703148906 | 0.332848 | 5.116892 | 3.11E-07 | 1.72E-06 | Up |
| RPL36AP33 | 3.088307129 | -1.167428334 | 0.320568 | -3.64175 | 0.000271 | 0.000916 | Down |
| RPL39P5 | 2.481742606 | -2.062973391 | 0.519422 | -3.97167 | 7.14E-05 | 0.00027 | Down |
| RPL3L | 4.219773585 | -3.734684615 | 0.672932 | -5.54987 | 2.86E-08 | 1.81E-07 | Down |
| RPL3P13 | 0.650717516 | -1.118495014 | 0.471109 | -2.37417 | 0.017588 | 0.037025 | Down |
| RPL5P14 | 2.202897082 | -1.358799355 | 0.522156 | -2.60228 | 0.00926 | 0.021289 | Down |
| RPL5P20 | 5.10168893 | -1.439578869 | 0.332733 | -4.32653 | 1.51E-05 | 6.50E-05 | Down |
| RPL5P25 | 1.22569291 | -2.797056544 | 1.112186 | -2.51492 | 0.011906 | 0.026516 | Down |
| RPL5P27 | 0.991952587 | -2.262514166 | 0.825277 | -2.74152 | 0.006116 | 0.014792 | Down |
| RPL5P29 | 1.391140989 | -1.747852007 | 0.684367 | -2.55397 | 0.01065 | 0.024028 | Down |
| RPL5P3 | 1.347596982 | -1.755570918 | 0.46772 | -3.75347 | 0.000174 | 0.000614 | Down |
| RPL5P32 | 2.484084069 | -2.200676321 | 0.369189 | -5.96083 | 2.51E-09 | 1.80E-08 | Down |
| RPL6P12 | 6.087986923 | -1.37838879 | 0.280607 | -4.91218 | 9.01E-07 | 4.67E-06 | Down |
| RPL6P21 | 1.090457211 | -3.738362576 | 1.500756 | -2.49099 | 0.012739 | 0.028132 | Down |
| RPL6P7 | 5.243499364 | -2.622961853 | 0.34677 | -7.56399 | 3.91E-14 | 4.51E-13 | Down |
| RPL6P8 | 2.842013663 | -1.02591536 | 0.338977 | -3.02651 | 0.002474 | 0.006647 | Down |
| RPL7L1P4 | 1.331176721 | -1.787232828 | 0.474001 | -3.77053 | 0.000163 | 0.000577 | Down |
| RPL7L1P9 | 6.03349574 | -1.14128002 | 0.22904 | -4.98288 | 6.26E-07 | 3.32E-06 | Down |
| RPL7P19 | 1.302998731 | -1.722106482 | 0.505536 | -3.40649 | 0.000658 | 0.002037 | Down |
| RPL7P22 | 1.544529135 | -1.003359574 | 0.353178 | -2.84095 | 0.004498 | 0.011293 | Down |
| RPL7P28 | 1.069712646 | -1.413288124 | 0.569703 | -2.48075 | 0.013111 | 0.028831 | Down |
| RPL7P29 | 1.717777266 | -1.532097523 | 0.39841 | -3.84553 | 0.00012 | 0.000436 | Down |
| RPL7P39 | 1.285134725 | -2.035744203 | 0.691739 | -2.94294 | 0.003251 | 0.00847 | Down |
| RPL7P4 | 1.269962499 | -1.402923098 | 0.387961 | -3.61614 | 0.000299 | 0.001001 | Down |
| RPL7P41 | 0.907580168 | -2.453607601 | 0.673546 | -3.64282 | 0.00027 | 0.000913 | Down |
| RPL7P42 | 0.8118344 | -1.171880535 | 0.466569 | -2.5117 | 0.012015 | 0.026732 | Down |
| RPL7P60 | 0.684470986 | -1.26280307 | 0.460028 | -2.74506 | 0.00605 | 0.014658 | Down |
| RPL7P8 | 1.505977287 | -1.852774017 | 0.433057 | -4.27836 | 1.88E-05 | 7.94E-05 | Down |
| RPL9P16 | 6.612537813 | -1.082108289 | 0.268805 | -4.02562 | 5.68E-05 | 0.000219 | Down |
| RPL9P33 | 1.300832518 | -1.295490913 | 0.454129 | -2.85269 | 0.004335 | 0.010926 | Down |
| RPLP0P2 | 10.71752877 | 2.093065175 | 0.32522 | 6.435835 | 1.23E-10 | 1.01E-09 | Up |
| RPRML | 1.422857879 | -1.937207652 | 0.768184 | -2.5218 | 0.011676 | 0.026065 | Down |
| RPS10-NUDT3 | 130.9788109 | 1.25440694 | 0.350055 | 3.583455 | 0.000339 | 0.001122 | Up |
| RPS10P14 | 9.483629249 | -1.971258199 | 0.242693 | -8.12244 | 4.57E-16 | 6.22E-15 | Down |
| RPS16P5 | 11.17535984 | -1.373075386 | 0.243632 | -5.63587 | 1.74E-08 | 1.13E-07 | Down |
| RPS17P13 | 2.778327804 | -1.674069153 | 0.288426 | -5.80416 | 6.47E-09 | 4.41E-08 | Down |
| RPS18P1 | 1.124837688 | -1.193899072 | 0.518698 | -2.30172 | 0.021351 | 0.043751 | Down |
| RPS23P1 | 1.904017151 | -1.896587004 | 0.26861 | -7.06073 | 1.66E-12 | 1.64E-11 | Down |
| RPS24P14 | 1.570601977 | -1.20518465 | 0.362811 | -3.3218 | 0.000894 | 0.002686 | Down |
| RPS26P11 | 2.564275725 | 1.283885931 | 0.401243 | 3.199769 | 0.001375 | 0.003953 | Up |
| RPS2P35 | 2.472631527 | -1.281287165 | 0.367196 | -3.48939 | 0.000484 | 0.001546 | Down |
| RPS3AP2 | 0.982792449 | -1.650658059 | 0.377482 | -4.37281 | 1.23E-05 | 5.34E-05 | Down |
| RPS3AP44 | 5.059725049 | -1.376579563 | 0.250947 | -5.48554 | 4.12E-08 | 2.55E-07 | Down |
| RPS3AP54 | 0.890058493 | -2.090279072 | 0.528973 | -3.95158 | 7.76E-05 | 0.000292 | Down |
| RPS3P7 | 10.22576844 | -1.280793243 | 0.240337 | -5.32915 | 9.87E-08 | 5.82E-07 | Down |
| RPS4XP5 | 3.839079312 | -1.759430598 | 0.340499 | -5.16722 | 2.38E-07 | 1.34E-06 | Down |
| RPS6KA5 | 468.8455418 | -1.060511 | 0.075088 | -14.1235 | 2.72E-45 | 2.66E-43 | Down |
| RPS6KA6 | 643.0066775 | -1.299343101 | 0.093158 | -13.9477 | 3.25E-44 | 2.94E-42 | Down |
| RPS6KL1 | 109.4410504 | -1.006123306 | 0.102095 | -9.85474 | 6.54E-23 | 1.51E-21 | Down |
| RPS6P20 | 1.063076472 | -2.851102775 | 1.06871 | -2.6678 | 0.007635 | 0.017981 | Down |
| RPS6P22 | 1.437159217 | -1.061670177 | 0.411132 | -2.58231 | 0.009814 | 0.022393 | Down |
| RPSAP31 | 0.89897365 | -1.085809773 | 0.395232 | -2.74727 | 0.006009 | 0.014567 | Down |
| RPSAP52 | 23.06806661 | 3.089477276 | 0.294971 | 10.47382 | 1.14E-25 | 3.21E-24 | Up |
| RPSAP56 | 0.454985341 | -1.366771208 | 0.55327 | -2.47035 | 0.013498 | 0.02956 | Down |
| RRM2P3 | 4.528597015 | -1.068850042 | 0.213266 | -5.01182 | 5.39E-07 | 2.88E-06 | Down |
| RRS1-AS1 | 16.22712897 | -1.224429471 | 0.241754 | -5.06477 | 4.09E-07 | 2.23E-06 | Down |
| RS1 | 14.92422746 | -1.408030178 | 0.184932 | -7.61378 | 2.66E-14 | 3.13E-13 | Down |
| RSPH10B | 8.889658111 | -1.224816917 | 0.240018 | -5.10302 | 3.34E-07 | 1.84E-06 | Down |
| RSPH10B2 | 7.422287056 | -1.635577921 | 0.302436 | -5.40801 | 6.37E-08 | 3.85E-07 | Down |
| RSPH4A | 41.37192907 | -1.15910988 | 0.124401 | -9.31751 | 1.19E-20 | 2.33E-19 | Down |
| RSPH6A | 61.41230929 | -1.141182285 | 0.140694 | -8.11107 | 5.02E-16 | 6.80E-15 | Down |
| RSPO2 | 5.66327872 | -1.069342342 | 0.426378 | -2.50797 | 0.012143 | 0.02697 | Down |
| RSU1P3 | 13.73932071 | -1.637603107 | 0.251668 | -6.50699 | 7.67E-11 | 6.45E-10 | Down |
| RTBDN | 1.706122711 | 1.263137808 | 0.442014 | 2.857689 | 0.004267 | 0.010775 | Up |
| RTN4R | 150.3687541 | 1.081417217 | 0.097119 | 11.13499 | 8.48E-29 | 3.00E-27 | Up |
| RTP1 | 1.761260708 | -1.674948898 | 0.723168 | -2.31613 | 0.020551 | 0.042346 | Down |
| RTP2 | 1.168778243 | -2.622797293 | 0.856416 | -3.06253 | 0.002195 | 0.005985 | Down |
| RTP3 | 5.765777474 | -2.053038106 | 0.250012 | -8.21177 | 2.18E-16 | 3.04E-15 | Down |
| RTP5 | 6.187032715 | -1.992060552 | 0.466685 | -4.26853 | 1.97E-05 | 8.27E-05 | Down |
| RUNDC3A-AS1 | 61.41960015 | 1.340478258 | 0.163723 | 8.187471 | 2.67E-16 | 3.70E-15 | Up |
| RUNX1 | 853.4110492 | 1.534113695 | 0.150599 | 10.18678 | 2.27E-24 | 5.79E-23 | Up |
| RUNX1T1 | 361.1996398 | -1.252769514 | 0.109337 | -11.4579 | 2.15E-30 | 8.41E-29 | Down |
| RUNX2 | 407.6141791 | 1.767105456 | 0.124632 | 14.17861 | 1.24E-45 | 1.24E-43 | Up |
| RXFP1 | 56.80558636 | -1.872504884 | 0.176108 | -10.6327 | 2.10E-26 | 6.18E-25 | Down |
| RXFP2 | 2.717470746 | -1.380723414 | 0.430098 | -3.21025 | 0.001326 | 0.003826 | Down |
| RXRG | 810.6432231 | 4.302375707 | 0.198553 | 21.66865 | 4.05E-104 | 3.78E-101 | Up |
| RYR1 | 564.2321635 | 2.697229297 | 0.172992 | 15.59168 | 8.29E-55 | 1.30E-52 | Up |
| RYR2 | 1918.839193 | -2.419491204 | 0.17453 | -13.8629 | 1.06E-43 | 9.38E-42 | Down |
| S100A1 | 2217.273699 | 1.833195646 | 0.155633 | 11.77897 | 5.01E-32 | 2.20E-30 | Up |
| S100A11 | 2999.348458 | 1.544759753 | 0.077681 | 19.88586 | 5.39E-88 | 3.21E-85 | Up |
| S100A12 | 3.757540268 | -1.274241674 | 0.296672 | -4.29512 | 1.75E-05 | 7.41E-05 | Down |
| S100A13 | 3510.48835 | 1.056556266 | 0.089769 | 11.76973 | 5.59E-32 | 2.45E-30 | Up |
| S100A2 | 266.7157413 | 2.418719758 | 0.190246 | 12.71367 | 4.97E-37 | 2.89E-35 | Up |
| S100A3 | 10.1042661 | 1.002672428 | 0.157994 | 6.346255 | 2.21E-10 | 1.78E-09 | Up |
| S100A4 | 1705.043189 | 1.074499575 | 0.119449 | 8.995498 | 2.35E-19 | 4.18E-18 | Up |
| S100A5 | 53.91780384 | 1.867332206 | 0.146379 | 12.75683 | 2.86E-37 | 1.69E-35 | Up |
| S100A6 | 18794.5515 | 1.832353164 | 0.101249 | 18.09742 | 3.34E-73 | 1.11E-70 | Up |
| S100B | 156.023449 | 2.210988765 | 0.194234 | 11.3831 | 5.08E-30 | 1.94E-28 | Up |
| SAA2 | 7.606696893 | 1.549919949 | 0.388644 | 3.98802 | 6.66E-05 | 0.000253 | Up |
| SALL4 | 85.8581119 | -1.255339529 | 0.141859 | -8.84923 | 8.81E-19 | 1.50E-17 | Down |
| SALL4P1 | 1.728283895 | -2.602961924 | 0.689139 | -3.77712 | 0.000159 | 0.000563 | Down |
| SALL4P6 | 1.630552433 | -2.814634408 | 1.042031 | -2.70111 | 0.006911 | 0.016478 | Down |
| SALRNA1 | 4.20040576 | -1.215410845 | 0.327216 | -3.71439 | 0.000204 | 0.000707 | Down |
| SAMD4A | 1554.5527 | 1.356932412 | 0.089356 | 15.18568 | 4.40E-52 | 5.90E-50 | Up |
| SAMD5 | 150.0704866 | -1.600032392 | 0.151375 | -10.57 | 4.10E-26 | 1.18E-24 | Down |
| SAMD7 | 1.707186711 | -2.308338446 | 0.733177 | -3.14841 | 0.001642 | 0.004634 | Down |
| SAR1P3 | 1.233326767 | -1.107893221 | 0.34211 | -3.23841 | 0.001202 | 0.003509 | Down |
| SATL1 | 2.153385424 | -1.638550233 | 0.499987 | -3.27719 | 0.001048 | 0.003098 | Down |
| SBK3 | 5.360954832 | -1.629393731 | 0.351188 | -4.63967 | 3.49E-06 | 1.66E-05 | Down |
| SBSN | 4.511117055 | -1.136966922 | 0.36166 | -3.14375 | 0.001668 | 0.004701 | Down |
| SBSPON | 170.3339072 | -1.906148093 | 0.149875 | -12.7182 | 4.68E-37 | 2.74E-35 | Down |
| SCARA5 | 971.2977253 | -1.92823783 | 0.224537 | -8.58761 | 8.88E-18 | 1.39E-16 | Down |
| SCD | 705.3713411 | 1.217718482 | 0.180395 | 6.75029 | 1.48E-11 | 1.33E-10 | Up |
| SCD5 | 2909.93795 | 1.077680031 | 0.10041 | 10.73278 | 7.14E-27 | 2.18E-25 | Up |
| SCEL | 1463.835562 | 3.914896865 | 0.19779 | 19.79316 | 3.41E-87 | 2.00E-84 | Up |
| SCG3 | 26.96280589 | -1.905026894 | 0.175436 | -10.8588 | 1.81E-27 | 5.82E-26 | Down |
| SCG5 | 345.3248238 | 2.321778239 | 0.139842 | 16.60284 | 6.65E-62 | 1.42E-59 | Up |
| SCGB1D2 | 1.870222022 | -2.18154893 | 0.378484 | -5.76391 | 8.22E-09 | 5.56E-08 | Down |
| SCGB3A1 | 38.245255 | 1.396772275 | 0.235829 | 5.922817 | 3.16E-09 | 2.24E-08 | Up |
| SCML2 | 73.74451792 | -1.316056143 | 0.090219 | -14.5874 | 3.38E-48 | 3.85E-46 | Down |
| SCN11A | 21.71792399 | -1.265586439 | 0.159254 | -7.94697 | 1.91E-15 | 2.48E-14 | Down |
| SCN1A | 8.554169936 | -1.824234051 | 0.426687 | -4.27535 | 1.91E-05 | 8.04E-05 | Down |
| SCN1B | 254.03279 | 1.393385232 | 0.124005 | 11.23652 | 2.70E-29 | 9.86E-28 | Up |
| SCN3A | 204.1575142 | -1.448133783 | 0.146159 | -9.90793 | 3.85E-23 | 9.00E-22 | Down |
| SCN3B | 194.7984847 | -1.804637318 | 0.125985 | -14.3242 | 1.55E-46 | 1.62E-44 | Down |
| SCN4B | 642.2375601 | -1.195864399 | 0.114738 | -10.4226 | 1.96E-25 | 5.38E-24 | Down |
| SCNN1B | 191.938444 | -1.197114731 | 0.124415 | -9.62197 | 6.46E-22 | 1.38E-20 | Down |
| SCUBE3 | 2112.97963 | -1.019127978 | 0.127468 | -7.99519 | 1.29E-15 | 1.70E-14 | Down |
| SDAD1P4 | 1.376889072 | -2.832480294 | 0.931026 | -3.04232 | 0.002348 | 0.00635 | Down |
| SDC4 | 9212.161749 | 2.886371749 | 0.125283 | 23.0389 | 1.90E-117 | 2.89E-114 | Up |
| SDK1 | 704.858313 | 2.044195088 | 0.156407 | 13.0697 | 4.91E-39 | 3.21E-37 | Up |
| SDK2 | 337.3166133 | -1.173950061 | 0.127774 | -9.1877 | 4.01E-20 | 7.54E-19 | Down |
| SDPR | 2913.509759 | -1.191715826 | 0.120794 | -9.8657 | 5.86E-23 | 1.36E-21 | Down |
| SDR16C5 | 19.94769369 | 1.935648305 | 0.339387 | 5.703366 | 1.17E-08 | 7.80E-08 | Up |
| SDR42E2 | 4.930138772 | -1.296661718 | 0.27549 | -4.70675 | 2.52E-06 | 1.22E-05 | Down |
| SDR9C7 | 1.74235845 | -1.737892981 | 0.643136 | -2.70222 | 0.006888 | 0.016433 | Down |
| SEC14L2 | 546.4334578 | 1.292179575 | 0.085512 | 15.11101 | 1.37E-51 | 1.80E-49 | Up |
| SEC14L3 | 43.24843398 | -3.102482932 | 0.296354 | -10.4688 | 1.20E-25 | 3.37E-24 | Down |
| SEC14L6 | 15.69356186 | 1.116659438 | 0.275274 | 4.056543 | 4.98E-05 | 0.000194 | Up |
| SEL1L2 | 2.141770884 | -1.450443223 | 0.481406 | -3.01293 | 0.002587 | 0.006909 | Down |
| SEL1L3 | 3154.503918 | 1.037168917 | 0.148858 | 6.967497 | 3.23E-12 | 3.10E-11 | Up |
| SELENBP1 | 5677.293693 | -1.317671002 | 0.109085 | -12.0793 | 1.36E-33 | 6.46E-32 | Down |
| SELV | 43.5564012 | -2.421010432 | 0.22458 | -10.7801 | 4.27E-27 | 1.33E-25 | Down |
| SEMA3B-AS1 | 7.164410594 | -1.289898491 | 0.148976 | -8.65842 | 4.78E-18 | 7.63E-17 | Down |
| SEMA3D | 2933.975382 | -2.686052333 | 0.221207 | -12.1427 | 6.27E-34 | 3.02E-32 | Down |
| SEMA3E | 128.7894079 | 1.142248601 | 0.21738 | 5.254618 | 1.48E-07 | 8.57E-07 | Up |
| SEMA3F | 993.4130626 | 1.219529918 | 0.082915 | 14.70823 | 5.71E-49 | 6.71E-47 | Up |
| SEMA6A | 532.1381785 | -1.063166916 | 0.094864 | -11.2073 | 3.75E-29 | 1.36E-27 | Down |
| SEMA6D | 648.7841726 | -1.03397852 | 0.109624 | -9.43201 | 4.02E-21 | 8.16E-20 | Down |
| SEMG2 | 2.071788612 | -2.380820147 | 0.865091 | -2.7521 | 0.005921 | 0.014385 | Down |
| SEPHS1P4 | 0.922616026 | -1.069520418 | 0.45369 | -2.35738 | 0.018404 | 0.038498 | Down |
| SEPHS1P6 | 9.328611566 | -1.790832531 | 0.195814 | -9.14557 | 5.93E-20 | 1.10E-18 | Down |
| SEPT10P1 | 3.564063642 | -1.473228768 | 0.417668 | -3.52727 | 0.00042 | 0.001359 | Down |
| SEPT3 | 66.4691546 | 1.366110477 | 0.175511 | 7.783626 | 7.05E-15 | 8.77E-14 | Up |
| SEPT4-AS1 | 7.892785699 | -1.134465487 | 0.157054 | -7.22339 | 5.07E-13 | 5.26E-12 | Down |
| SERBP1P3 | 2.197308678 | -1.651669035 | 0.498918 | -3.3105 | 0.000931 | 0.002787 | Down |
| SERBP1P5 | 15.2782632 | -1.230759053 | 0.199435 | -6.17123 | 6.78E-10 | 5.18E-09 | Down |
| SERBP1P6 | 4.595796983 | -1.289372401 | 0.230309 | -5.59845 | 2.16E-08 | 1.39E-07 | Down |
| SERHL2 | 77.14446147 | -1.475149766 | 0.124893 | -11.8113 | 3.41E-32 | 1.51E-30 | Down |
| SERINC2 | 763.9981193 | 1.645366832 | 0.108229 | 15.20261 | 3.40E-52 | 4.60E-50 | Up |
| SERPINA1 | 18653.10457 | 4.489138278 | 0.155287 | 28.9086 | 9.31E-184 | 3.82E-179 | Up |
| SERPINA11 | 1.364876262 | -1.264178161 | 0.548426 | -2.3051 | 0.021161 | 0.04341 | Down |
| SERPINA5 | 55.09114003 | -1.794951947 | 0.215774 | -8.31867 | 8.90E-17 | 1.28E-15 | Down |
| SERPINA9 | 30.24788605 | -1.817105174 | 0.582502 | -3.11948 | 0.001812 | 0.005055 | Down |
| SERPINB2 | 6.349986556 | 2.166161819 | 0.377473 | 5.738589 | 9.55E-09 | 6.42E-08 | Up |
| SERPINB7 | 2.731240551 | 1.474020251 | 0.560331 | 2.630624 | 0.008523 | 0.019812 | Up |
| SERPINB8 | 259.5969876 | 1.069331129 | 0.090137 | 11.86334 | 1.84E-32 | 8.26E-31 | Up |
| SERPIND1 | 36.25763103 | -1.378469597 | 0.153719 | -8.96745 | 3.03E-19 | 5.34E-18 | Down |
| SERPINE1 | 416.583397 | 1.859049873 | 0.216653 | 8.580789 | 9.42E-18 | 1.47E-16 | Up |
| SERPINH1P1 | 3.026876146 | -1.034013606 | 0.346113 | -2.98751 | 0.002813 | 0.007443 | Down |
| SERPINI2 | 3.490232921 | -1.232633038 | 0.335582 | -3.67312 | 0.00024 | 0.000819 | Down |
| SERTM1 | 143.1135445 | -2.498225139 | 0.230763 | -10.8259 | 2.59E-27 | 8.24E-26 | Down |
| SETP16 | 0.935608619 | -1.658471838 | 0.528218 | -3.13975 | 0.001691 | 0.00476 | Down |
| SETP2 | 1.537902218 | -2.078821618 | 0.674818 | -3.08057 | 0.002066 | 0.005673 | Down |
| SETP21 | 2.39042551 | -1.308469963 | 0.33389 | -3.91886 | 8.90E-05 | 0.00033 | Down |
| SETSIP | 1.425308786 | -1.964190236 | 0.669624 | -2.93327 | 0.003354 | 0.008709 | Down |
| SEZ6L2 | 167.2052406 | 3.009421778 | 0.1834 | 16.40908 | 1.65E-60 | 3.41E-58 | Up |
| SF3A3P2 | 5.915620796 | -1.141216204 | 0.279621 | -4.08129 | 4.48E-05 | 0.000177 | Down |
| SFN | 112.2020945 | 3.776487297 | 0.211702 | 17.83867 | 3.54E-71 | 1.08E-68 | Up |
| SFRP1 | 404.8511056 | -2.236216326 | 0.174644 | -12.8044 | 1.55E-37 | 9.30E-36 | Down |
| SFRP2 | 2400.080819 | 1.280896845 | 0.208523 | 6.142716 | 8.11E-10 | 6.15E-09 | Up |
| SFRP5 | 10.0753923 | -1.240013125 | 0.253629 | -4.88909 | 1.01E-06 | 5.21E-06 | Down |
| SFTA2 | 14.51781281 | 1.893000996 | 0.231816 | 8.165969 | 3.19E-16 | 4.38E-15 | Up |
| SFTA3 | 2682.095977 | 1.136479779 | 0.100902 | 11.26316 | 1.99E-29 | 7.37E-28 | Up |
| SFTPA1 | 107.7436097 | 3.669453455 | 0.387788 | 9.46252 | 3.01E-21 | 6.17E-20 | Up |
| SFTPA2 | 44.23888537 | 2.577619517 | 0.381326 | 6.759619 | 1.38E-11 | 1.25E-10 | Up |
| SFTPB | 5732.730811 | 4.416758971 | 0.269454 | 16.39153 | 2.20E-60 | 4.51E-58 | Up |
| SFTPC | 15.06210997 | -1.441259059 | 0.249252 | -5.78233 | 7.37E-09 | 5.00E-08 | Down |
| SGCD | 869.9911277 | -1.197251795 | 0.162861 | -7.35135 | 1.96E-13 | 2.12E-12 | Down |
| SGCG | 4.141783712 | -1.292853358 | 0.326377 | -3.96123 | 7.46E-05 | 0.000281 | Down |
| SGCZ | 0.953572634 | -2.257823257 | 0.888361 | -2.54156 | 0.011036 | 0.024774 | Down |
| SGK3 | 1258.457234 | 1.072632319 | 0.097776 | 10.97035 | 5.31E-28 | 1.77E-26 | Up |
| SGMS2 | 2920.314477 | 1.200899547 | 0.0912 | 13.16778 | 1.34E-39 | 9.25E-38 | Up |
| SGOL1P1 | 4.235106613 | -2.202795371 | 0.329384 | -6.68763 | 2.27E-11 | 2.00E-10 | Down |
| SH2D4A | 1000.71863 | 1.097737571 | 0.086852 | 12.63912 | 1.28E-36 | 7.37E-35 | Up |
| SH2D4B | 9.188666726 | -1.612080289 | 0.256129 | -6.29401 | 3.09E-10 | 2.46E-09 | Down |
| SH2D6 | 25.8401961 | -2.569859879 | 0.183846 | -13.9783 | 2.11E-44 | 1.95E-42 | Down |
| SH3GL2 | 46.29230446 | -1.88841987 | 0.244867 | -7.71203 | 1.24E-14 | 1.51E-13 | Down |
| SHANK2 | 1051.686302 | -1.657850303 | 0.122765 | -13.5042 | 1.48E-41 | 1.14E-39 | Down |
| SHANK2-AS1 | 16.27756492 | -2.113895249 | 0.256462 | -8.24253 | 1.69E-16 | 2.37E-15 | Down |
| SHANK2-AS2 | 0.914288777 | -1.93993876 | 0.433967 | -4.47025 | 7.81E-06 | 3.52E-05 | Down |
| SHANK2-AS3 | 7.538992571 | -2.176219642 | 0.293483 | -7.41516 | 1.21E-13 | 1.34E-12 | Down |
| SHBG | 16.81491762 | -1.090398051 | 0.307915 | -3.54123 | 0.000398 | 0.001297 | Down |
| SHC1P2 | 2.393269662 | -1.66469087 | 0.492477 | -3.38024 | 0.000724 | 0.002219 | Down |
| SHC3 | 304.7103806 | 1.405937566 | 0.162518 | 8.650982 | 5.11E-18 | 8.12E-17 | Up |
| SHD | 4.451079031 | -1.450166603 | 0.543363 | -2.66887 | 0.007611 | 0.017929 | Down |
| SHISA6 | 141.8973422 | 1.791486077 | 0.229495 | 7.806211 | 5.89E-15 | 7.39E-14 | Up |
| SHISA8 | 6.671035063 | -1.334035498 | 0.51219 | -2.60457 | 0.009199 | 0.021165 | Down |
| SHROOM4 | 2742.445086 | 2.634010773 | 0.110781 | 23.7767 | 5.82E-125 | 1.26E-121 | Up |
| SIGLEC11 | 33.36904464 | -1.436987252 | 0.143509 | -10.0132 | 1.33E-23 | 3.24E-22 | Down |
| SIGLEC12 | 25.31909656 | 1.014481751 | 0.30223 | 3.356652 | 0.000789 | 0.002396 | Up |
| SIGLEC15 | 49.53276091 | 1.741412035 | 0.20364 | 8.551441 | 1.22E-17 | 1.88E-16 | Up |
| SIGLEC29P | 1.355498884 | -2.981657627 | 1.092425 | -2.72939 | 0.006345 | 0.015293 | Down |
| SIGLEC6 | 77.74470033 | 2.101774707 | 0.317376 | 6.62235 | 3.54E-11 | 3.06E-10 | Up |
| SIGLEC9 | 64.18683978 | 1.028161718 | 0.16052 | 6.405201 | 1.50E-10 | 1.23E-09 | Up |
| SIM1 | 2.843125438 | -1.303964385 | 0.45374 | -2.87381 | 0.004055 | 0.010297 | Down |
| SIPA1L2 | 1584.351357 | 1.451019564 | 0.078427 | 18.50142 | 2.01E-76 | 7.79E-74 | Up |
| SIX2 | 11.13325101 | -1.269349054 | 0.272263 | -4.66222 | 3.13E-06 | 1.50E-05 | Down |
| SKOR1 | 13.00266662 | -1.086231837 | 0.210802 | -5.15287 | 2.57E-07 | 1.43E-06 | Down |
| SLA | 3360.06851 | -1.510631341 | 0.192419 | -7.85073 | 4.14E-15 | 5.25E-14 | Down |
| SLAMF9 | 2.010331947 | 1.741797772 | 0.42064 | 4.140827 | 3.46E-05 | 0.000139 | Up |
| SLC10A5P1 | 1.339812341 | -1.708217212 | 0.515994 | -3.31053 | 0.000931 | 0.002787 | Down |
| SLC12A2 | 1774.884299 | 1.032508626 | 0.066851 | 15.44486 | 8.17E-54 | 1.21E-51 | Up |
| SLC12A3 | 9.264299143 | -1.200138912 | 0.300854 | -3.9891 | 6.63E-05 | 0.000252 | Down |
| SLC13A2 | 1.537748392 | -2.13599602 | 0.651367 | -3.27925 | 0.001041 | 0.00308 | Down |
| SLC13A5 | 10.08927675 | -1.315017873 | 0.309318 | -4.25134 | 2.12E-05 | 8.88E-05 | Down |
| SLC14A1 | 219.9523373 | -1.184510712 | 0.156351 | -7.57598 | 3.56E-14 | 4.14E-13 | Down |
| SLC14A2 | 19.67717817 | -1.665959674 | 0.268275 | -6.20989 | 5.30E-10 | 4.10E-09 | Down |
| SLC15A5 | 1.862600214 | -1.475759699 | 0.467288 | -3.15814 | 0.001588 | 0.004496 | Down |
| SLC16A2 | 822.9447999 | -1.071007917 | 0.086917 | -12.3221 | 6.88E-35 | 3.53E-33 | Down |
| SLC16A3 | 562.4869244 | 1.227595889 | 0.13127 | 9.351698 | 8.63E-21 | 1.71E-19 | Up |
| SLC17A3 | 2.586402488 | -1.706055555 | 0.57915 | -2.94579 | 0.003221 | 0.008401 | Down |
| SLC17A5 | 2808.753997 | 1.451062029 | 0.094859 | 15.29699 | 8.01E-53 | 1.12E-50 | Up |
| SLC17A7 | 35.55322447 | -1.681083205 | 0.203254 | -8.27086 | 1.33E-16 | 1.89E-15 | Down |
| SLC18A1 | 4.08819216 | -1.202328229 | 0.535992 | -2.24318 | 0.024885 | 0.049907 | Down |
| SLC18A2 | 26.82344943 | 2.002474463 | 0.239817 | 8.350007 | 6.83E-17 | 9.94E-16 | Up |
| SLC18A3 | 8.934116756 | 1.576034587 | 0.500584 | 3.148392 | 0.001642 | 0.004634 | Up |
| SLC19A1 | 211.7278588 | -1.201350887 | 0.109161 | -11.0053 | 3.60E-28 | 1.22E-26 | Down |
| SLC19A3 | 25.19295527 | -1.236952913 | 0.286265 | -4.32101 | 1.55E-05 | 6.65E-05 | Down |
| SLC1A5 | 471.8062123 | 1.333971442 | 0.143276 | 9.310482 | 1.27E-20 | 2.48E-19 | Up |
| SLC1A7 | 39.64928828 | -1.727559345 | 0.192821 | -8.95941 | 3.26E-19 | 5.73E-18 | Down |
| SLC22A10 | 1.2226591 | -1.988514867 | 0.664369 | -2.99309 | 0.002762 | 0.007317 | Down |
| SLC22A14 | 3.352305743 | -1.354652965 | 0.385111 | -3.51756 | 0.000436 | 0.001406 | Down |
| SLC22A2 | 2.325984991 | -1.033404416 | 0.454991 | -2.27126 | 0.023131 | 0.046822 | Down |
| SLC22A25 | 1.567406004 | -1.726802351 | 0.569711 | -3.03102 | 0.002437 | 0.006558 | Down |
| SLC22A31 | 217.363298 | 4.703364277 | 0.255799 | 18.38692 | 1.67E-75 | 6.30E-73 | Up |
| SLC22A7 | 3.278278209 | -1.728197181 | 0.438514 | -3.94103 | 8.11E-05 | 0.000304 | Down |
| SLC22A8 | 2.683411977 | -1.169267248 | 0.471964 | -2.47745 | 0.013233 | 0.029065 | Down |
| SLC22A9 | 1.134410384 | -2.049788925 | 0.739978 | -2.77007 | 0.005604 | 0.013702 | Down |
| SLC23A1 | 16.32322604 | -1.160892911 | 0.328379 | -3.53522 | 0.000407 | 0.001324 | Down |
| SLC24A2 | 25.95139877 | 1.238829397 | 0.376074 | 3.294113 | 0.000987 | 0.002936 | Up |
| SLC24A3 | 127.8100048 | -1.345029205 | 0.112658 | -11.939 | 7.41E-33 | 3.38E-31 | Down |
| SLC25A15 | 354.3991029 | -1.702787535 | 0.145503 | -11.7028 | 1.23E-31 | 5.31E-30 | Down |
| SLC25A15P3 | 0.616568661 | -2.210345128 | 0.441662 | -5.00461 | 5.60E-07 | 2.99E-06 | Down |
| SLC25A1P1 | 0.839961755 | -1.130117947 | 0.456381 | -2.47626 | 0.013277 | 0.029145 | Down |
| SLC25A1P3 | 1.149877373 | -1.427305805 | 0.534522 | -2.67025 | 0.00758 | 0.017861 | Down |
| SLC25A30-AS1 | 2.035888073 | -1.18594561 | 0.399073 | -2.97175 | 0.002961 | 0.007795 | Down |
| SLC25A31 | 1.059926546 | -2.107224612 | 0.767684 | -2.74491 | 0.006053 | 0.014664 | Down |
| SLC25A33 | 185.467109 | -1.144477723 | 0.087729 | -13.0456 | 6.73E-39 | 4.38E-37 | Down |
| SLC25A47 | 4.142936463 | -1.767568138 | 0.265987 | -6.64532 | 3.03E-11 | 2.64E-10 | Down |
| SLC25A47P1 | 16.71559831 | 5.741931374 | 0.294655 | 19.48699 | 1.42E-84 | 7.36E-82 | Up |
| SLC25A48 | 11.76869913 | 1.261233662 | 0.319069 | 3.952857 | 7.72E-05 | 0.00029 | Up |
| SLC25A5-AS1 | 65.92355516 | -1.051104659 | 0.094088 | -11.1715 | 5.62E-29 | 2.02E-27 | Down |
| SLC25A5P5 | 1.232029053 | -1.961276803 | 0.615148 | -3.1883 | 0.001431 | 0.004096 | Down |
| SLC25A6P5 | 1.97070428 | -1.788241728 | 0.607739 | -2.94245 | 0.003256 | 0.008482 | Down |
| SLC26A3 | 9.009132127 | -2.141081786 | 0.327684 | -6.53399 | 6.40E-11 | 5.43E-10 | Down |
| SLC26A4 | 11400.28304 | -2.212009452 | 0.153628 | -14.3984 | 5.29E-47 | 5.76E-45 | Down |
| SLC26A4-AS1 | 6749.012099 | -2.18879217 | 0.168043 | -13.0252 | 8.80E-39 | 5.64E-37 | Down |
| SLC26A7 | 19231.64484 | -2.39073067 | 0.167974 | -14.2327 | 5.74E-46 | 5.82E-44 | Down |
| SLC26A9 | 37.26306499 | -1.002991001 | 0.198585 | -5.0507 | 4.40E-07 | 2.38E-06 | Down |
| SLC27A2 | 206.8418208 | 1.314314273 | 0.115351 | 11.39404 | 4.48E-30 | 1.72E-28 | Up |
| SLC27A6 | 1410.524103 | 2.428047034 | 0.186902 | 12.99104 | 1.38E-38 | 8.71E-37 | Up |
| SLC28A1 | 2.796515645 | -1.362437902 | 0.404489 | -3.36829 | 0.000756 | 0.002307 | Down |
| SLC28A2 | 6.005294365 | -1.779522401 | 0.330099 | -5.39087 | 7.01E-08 | 4.21E-07 | Down |
| SLC28A3 | 70.97412837 | 2.135422504 | 0.241078 | 8.857794 | 8.16E-19 | 1.39E-17 | Up |
| SLC29A4 | 196.9070334 | -2.128186778 | 0.150211 | -14.168 | 1.45E-45 | 1.44E-43 | Down |
| SLC2A2 | 2.806479572 | -1.496167359 | 0.553706 | -2.7021 | 0.00689 | 0.016436 | Down |
| SLC30A10 | 2.931252624 | -1.293777442 | 0.428468 | -3.01954 | 0.002532 | 0.006779 | Down |
| SLC30A2 | 723.8733102 | 1.896768581 | 0.201029 | 9.435318 | 3.90E-21 | 7.92E-20 | Up |
| SLC30A3 | 27.10391475 | 1.46902139 | 0.321577 | 4.568174 | 4.92E-06 | 2.28E-05 | Up |
| SLC32A1 | 1.564403106 | -1.557422321 | 0.581536 | -2.67812 | 0.007404 | 0.017496 | Down |
| SLC34A2 | 21869.65632 | 4.946714526 | 0.211816 | 23.35379 | 1.26E-120 | 2.35E-117 | Up |
| SLC35F2 | 653.2081197 | 1.665755178 | 0.0834 | 19.97299 | 9.46E-89 | 5.88E-86 | Up |
| SLC35F3 | 49.10807406 | 1.026024202 | 0.198784 | 5.161495 | 2.45E-07 | 1.38E-06 | Up |
| SLC35F4 | 18.99368405 | -2.293396223 | 0.250096 | -9.17007 | 4.73E-20 | 8.82E-19 | Down |
| SLC35G3 | 3.126744094 | -1.647464393 | 0.548976 | -3.00098 | 0.002691 | 0.00715 | Down |
| SLC36A2 | 3.186690047 | -1.945611877 | 0.736634 | -2.64122 | 0.008261 | 0.019269 | Down |
| SLC39A12-AS1 | 1.467128216 | -2.095040998 | 0.886182 | -2.36412 | 0.018073 | 0.037897 | Down |
| SLC47A1 | 450.9669458 | 1.150874296 | 0.105697 | 10.88847 | 1.31E-27 | 4.24E-26 | Up |
| SLC4A1 | 14.51699136 | -1.761030966 | 0.262589 | -6.70643 | 1.99E-11 | 1.77E-10 | Down |
| SLC4A10 | 7.775137075 | -1.005234246 | 0.27005 | -3.7224 | 0.000197 | 0.000687 | Down |
| SLC4A4 | 2324.460044 | -2.273153615 | 0.166188 | -13.6782 | 1.37E-42 | 1.13E-40 | Down |
| SLC5A1 | 4.525543928 | -1.531988143 | 0.376687 | -4.06701 | 4.76E-05 | 0.000187 | Down |
| SLC5A5 | 755.066425 | -2.693624951 | 0.29278 | -9.20018 | 3.57E-20 | 6.75E-19 | Down |
| SLC5A7 | 32.10357616 | -2.39102717 | 0.259105 | -9.22803 | 2.76E-20 | 5.24E-19 | Down |
| SLC5A8 | 830.692523 | -2.614818029 | 0.228943 | -11.4213 | 3.27E-30 | 1.27E-28 | Down |
| SLC6A14 | 54.53561354 | 3.067990362 | 0.321092 | 9.554865 | 1.24E-21 | 2.60E-20 | Up |
| SLC6A15 | 23.45683572 | -2.405109538 | 0.28633 | -8.39977 | 4.47E-17 | 6.63E-16 | Down |
| SLC6A16 | 28.37613065 | -1.325770064 | 0.165719 | -8.00011 | 1.24E-15 | 1.64E-14 | Down |
| SLC6A17 | 20.95067969 | -1.268742566 | 0.225182 | -5.6343 | 1.76E-08 | 1.14E-07 | Down |
| SLC6A2 | 2.538986331 | -1.02704813 | 0.457651 | -2.24417 | 0.024821 | 0.049792 | Down |
| SLC6A20 | 109.3040864 | 3.358598707 | 0.328128 | 10.23563 | 1.37E-24 | 3.55E-23 | Up |
| SLC6A3 | 33.29380019 | -2.339958761 | 0.20645 | -11.3342 | 8.88E-30 | 3.34E-28 | Down |
| SLC6A4 | 37.38559955 | -2.535543851 | 0.243027 | -10.4332 | 1.75E-25 | 4.84E-24 | Down |
| SLC7A10 | 10.7723847 | -1.284230593 | 0.253874 | -5.05853 | 4.23E-07 | 2.30E-06 | Down |
| SLC7A2 | 3999.962832 | 1.035771991 | 0.125774 | 8.235194 | 1.79E-16 | 2.52E-15 | Up |
| SLC7A4 | 9.686915274 | 1.409842545 | 0.235802 | 5.978914 | 2.25E-09 | 1.62E-08 | Up |
| SLC9A3 | 5.476774929 | -1.050835183 | 0.208976 | -5.0285 | 4.94E-07 | 2.66E-06 | Down |
| SLC9A3P2 | 1.73056456 | -2.652667861 | 1.132962 | -2.34136 | 0.019214 | 0.039923 | Down |
| SLC9A3P3 | 1.630338406 | -1.407255369 | 0.541966 | -2.59657 | 0.009416 | 0.021615 | Down |
| SLC9A7P1 | 118.2732131 | 1.5152617 | 0.120983 | 12.52462 | 5.48E-36 | 3.03E-34 | Up |
| SLC9C2 | 4.72587958 | -1.331014743 | 0.331329 | -4.0172 | 5.89E-05 | 0.000226 | Down |
| SLCO1A2 | 4.597458298 | -2.10050898 | 0.410712 | -5.11431 | 3.15E-07 | 1.74E-06 | Down |
| SLCO1B7 | 2.111743271 | 3.515743981 | 0.507219 | 6.931409 | 4.17E-12 | 3.97E-11 | Up |
| SLCO4C1 | 97.5452977 | 2.300365477 | 0.21292 | 10.80389 | 3.30E-27 | 1.04E-25 | Up |
| SLCO6A1 | 1.566154421 | -2.806734158 | 0.797859 | -3.51783 | 0.000435 | 0.001405 | Down |
| SLIT1 | 557.3582488 | 4.790617492 | 0.277326 | 17.27429 | 7.35E-67 | 1.85E-64 | Up |
| SLITRK2 | 44.26623769 | -2.59426432 | 0.255598 | -10.1498 | 3.32E-24 | 8.39E-23 | Down |
| SLITRK3 | 10.15557012 | -1.571708213 | 0.287704 | -5.46293 | 4.68E-08 | 2.88E-07 | Down |
| SLITRK4 | 124.0084245 | 1.471006334 | 0.172339 | 8.53555 | 1.39E-17 | 2.15E-16 | Up |
| SLITRK5 | 228.0491174 | -2.055382283 | 0.229131 | -8.97034 | 2.96E-19 | 5.21E-18 | Down |
| SLMO1 | 59.47720015 | 1.639005121 | 0.122248 | 13.40717 | 5.49E-41 | 4.09E-39 | Up |
| SLN | 4.639118424 | -3.051126197 | 0.467169 | -6.5311 | 6.53E-11 | 5.52E-10 | Down |
| SLPI | 662.5237715 | 2.380441268 | 0.181389 | 13.12343 | 2.42E-39 | 1.63E-37 | Up |
| SMAD9 | 2006.69104 | -1.389167738 | 0.105136 | -13.213 | 7.38E-40 | 5.14E-38 | Down |
| SMAD9-IT1 | 1.643719049 | -1.616258295 | 0.381497 | -4.23662 | 2.27E-05 | 9.43E-05 | Down |
| SMARCE1P2 | 1.873044785 | -2.884413262 | 0.811388 | -3.55491 | 0.000378 | 0.001239 | Down |
| SMARCE1P3 | 1.978655467 | -2.759845483 | 0.925892 | -2.98074 | 0.002875 | 0.00759 | Down |
| SMARCE1P6 | 3.045418587 | -1.815984847 | 0.41266 | -4.40068 | 1.08E-05 | 4.74E-05 | Down |
| SMCO1 | 2.166327884 | -1.792291811 | 0.64132 | -2.79469 | 0.005195 | 0.012828 | Down |
| SMCO3 | 32.71837391 | -1.577053788 | 0.157958 | -9.98403 | 1.79E-23 | 4.31E-22 | Down |
| SMIM1 | 130.1983814 | 1.05632628 | 0.104792 | 10.08023 | 6.76E-24 | 1.68E-22 | Up |
| SMIM22 | 10.22058643 | 1.646562992 | 0.304724 | 5.403465 | 6.54E-08 | 3.94E-07 | Up |
| SMIM24 | 14.54491773 | 2.287191685 | 0.402045 | 5.688893 | 1.28E-08 | 8.45E-08 | Up |
| SMIM3 | 445.2520134 | 1.016822863 | 0.071893 | 14.14355 | 2.05E-45 | 2.02E-43 | Up |
| SMLR1 | 6.770413482 | -1.530551064 | 0.250579 | -6.10807 | 1.01E-09 | 7.57E-09 | Down |
| SMOC2 | 2564.953857 | -1.988744783 | 0.179556 | -11.0759 | 1.64E-28 | 5.71E-27 | Down |
| SMPD4P1 | 1.764626699 | -2.815520146 | 0.804987 | -3.4976 | 0.000469 | 0.001503 | Down |
| SMR3A | 4.073737086 | -2.337924073 | 0.415982 | -5.62026 | 1.91E-08 | 1.23E-07 | Down |
| SMR3B | 4.193376513 | -3.027645099 | 0.506949 | -5.97229 | 2.34E-09 | 1.68E-08 | Down |
| SMUG1P1 | 1.727379259 | -1.891385715 | 0.354186 | -5.34009 | 9.29E-08 | 5.50E-07 | Down |
| SMYD1 | 4.40979388 | -2.893826018 | 0.784629 | -3.68814 | 0.000226 | 0.000777 | Down |
| SMYD3-IT1 | 1.915421154 | -1.627954176 | 0.337957 | -4.81704 | 1.46E-06 | 7.33E-06 | Down |
| SNAI1 | 135.8859638 | -1.187507682 | 0.113727 | -10.4417 | 1.60E-25 | 4.44E-24 | Down |
| SNAP23P | 0.527835057 | -1.178348122 | 0.508802 | -2.31593 | 0.020562 | 0.042363 | Down |
| SNAP25 | 49.66618495 | 1.697694598 | 0.169935 | 9.990273 | 1.68E-23 | 4.06E-22 | Up |
| SNAP91 | 28.0116273 | -1.548210565 | 0.239039 | -6.47681 | 9.37E-11 | 7.82E-10 | Down |
| SNCA | 125.7646529 | -1.028562198 | 0.113896 | -9.03071 | 1.71E-19 | 3.06E-18 | Down |
| SNCA-AS1 | 1.753096429 | -2.65052685 | 0.540787 | -4.90124 | 9.52E-07 | 4.92E-06 | Down |
| SNCB | 2.057615236 | -1.503990661 | 0.493625 | -3.04683 | 0.002313 | 0.006266 | Down |
| SNORA11D | 5.345036948 | -1.368461952 | 0.248061 | -5.51663 | 3.46E-08 | 2.16E-07 | Down |
| SNORA11E | 5.345036948 | -1.368461952 | 0.248061 | -5.51663 | 3.46E-08 | 2.16E-07 | Down |
| SNORA36C | 1.330237309 | -1.200616157 | 0.426737 | -2.81348 | 0.004901 | 0.012168 | Down |
| SNORD115-17 | 1.603782111 | 1.382636934 | 0.574031 | 2.408645 | 0.016012 | 0.034142 | Up |
| SNORD115-18 | 1.603782111 | 1.382636934 | 0.574031 | 2.408645 | 0.016012 | 0.034142 | Up |
| SNORD115-19 | 1.603782111 | 1.382636934 | 0.574031 | 2.408645 | 0.016012 | 0.034142 | Up |
| snoU2-30 | 1.288020241 | -1.27352559 | 0.42426 | -3.00176 | 0.002684 | 0.007134 | Down |
| SNRK-AS1 | 6.827172887 | -1.26263249 | 0.268973 | -4.69428 | 2.68E-06 | 1.30E-05 | Down |
| SNRPEP8 | 0.518495506 | 1.946179889 | 0.827396 | 2.352174 | 0.018664 | 0.03895 | Up |
| SNTA1 | 1891.042672 | -1.060619094 | 0.101012 | -10.4999 | 8.64E-26 | 2.45E-24 | Down |
| SNTG2 | 25.6873745 | -1.439367668 | 0.145118 | -9.91858 | 3.46E-23 | 8.11E-22 | Down |
| SNX18P14 | 8.553008834 | -2.158474136 | 0.254782 | -8.47186 | 2.42E-17 | 3.65E-16 | Down |
| SNX18P23 | 2.021761835 | -3.650607406 | 0.476586 | -7.65991 | 1.86E-14 | 2.22E-13 | Down |
| SNX18P24 | 2.01301571 | -3.408034251 | 0.4075 | -8.36328 | 6.10E-17 | 8.93E-16 | Down |
| SNX18P25 | 4.018793823 | -3.546368911 | 0.397511 | -8.92143 | 4.60E-19 | 7.98E-18 | Down |
| SNX18P3 | 88.68684682 | -2.002276812 | 0.184069 | -10.8779 | 1.47E-27 | 4.76E-26 | Down |
| SNX18P4 | 6.338397605 | -1.965362331 | 0.371159 | -5.29521 | 1.19E-07 | 6.95E-07 | Down |
| SNX18P5 | 2.668740426 | -1.262127798 | 0.400416 | -3.15204 | 0.001621 | 0.004582 | Down |
| SNX18P9 | 12.80372427 | -1.395947935 | 0.223409 | -6.24839 | 4.15E-10 | 3.25E-09 | Down |
| SNX19P1 | 2.004053805 | -3.24293051 | 0.833405 | -3.89118 | 9.98E-05 | 0.000367 | Down |
| SNX19P2 | 1.749946457 | -2.4043151 | 0.743865 | -3.23219 | 0.001228 | 0.003579 | Down |
| SNX19P3 | 2.143170884 | -1.320093427 | 0.41952 | -3.14667 | 0.001651 | 0.004659 | Down |
| SNX22 | 2627.319797 | 1.948601611 | 0.161663 | 12.05347 | 1.86E-33 | 8.75E-32 | Up |
| SNX25 | 1507.941281 | 1.405544732 | 0.090634 | 15.50799 | 3.06E-54 | 4.66E-52 | Up |
| SNX29P1 | 27.51062704 | -1.144007623 | 0.184657 | -6.19532 | 5.82E-10 | 4.48E-09 | Down |
| SNX29P2 | 171.013516 | -1.27491325 | 0.292849 | -4.35349 | 1.34E-05 | 5.79E-05 | Down |
| SNX32 | 39.24007711 | -1.038897298 | 0.158865 | -6.5395 | 6.17E-11 | 5.24E-10 | Down |
| SOCS5P5 | 1.65095795 | -2.411469503 | 1.061257 | -2.27228 | 0.02307 | 0.046728 | Down |
| SOD3 | 1202.944127 | -1.954905824 | 0.142508 | -13.7179 | 7.93E-43 | 6.62E-41 | Down |
| SOGA3 | 263.9625031 | 2.679550462 | 0.138676 | 19.32242 | 3.48E-83 | 1.68E-80 | Up |
| SOHLH2 | 13.98870461 | -1.210470495 | 0.312413 | -3.87458 | 0.000107 | 0.000391 | Down |
| SORBS2 | 5590.439906 | -1.802374979 | 0.119547 | -15.0767 | 2.31E-51 | 2.99E-49 | Down |
| SORCS3 | 5.079397744 | -1.748269965 | 0.458132 | -3.81609 | 0.000136 | 0.000487 | Down |
| SOST | 4.709422772 | -1.252035668 | 0.463623 | -2.70055 | 0.006923 | 0.016502 | Down |
| SOSTDC1 | 36.37200482 | -2.060834843 | 0.212498 | -9.69815 | 3.07E-22 | 6.72E-21 | Down |
| SOWAHA | 154.053337 | 1.015118108 | 0.146542 | 6.927149 | 4.29E-12 | 4.08E-11 | Up |
| SOWAHB | 290.7823603 | 1.297448097 | 0.093678 | 13.85013 | 1.27E-43 | 1.11E-41 | Up |
| SOX10 | 11.95456044 | -1.027048129 | 0.280101 | -3.6667 | 0.000246 | 0.000838 | Down |
| SOX11 | 32.76049956 | 1.515100368 | 0.377585 | 4.012612 | 6.01E-05 | 0.00023 | Up |
| SOX2 | 12.17418707 | -1.134130303 | 0.292824 | -3.87308 | 0.000107 | 0.000393 | Down |
| SOX21 | 5.688082361 | -2.141746031 | 0.312263 | -6.85878 | 6.95E-12 | 6.47E-11 | Down |
| SOX21-AS1 | 7.217184614 | -2.472127803 | 0.317741 | -7.78033 | 7.23E-15 | 9.00E-14 | Down |
| SOX3 | 3.543449102 | -2.269700047 | 0.353998 | -6.41163 | 1.44E-10 | 1.18E-09 | Down |
| SOX4 | 1986.070836 | 1.493147484 | 0.084528 | 17.6645 | 7.87E-70 | 2.34E-67 | Up |
| SOX5P | 1.073881762 | -2.289139519 | 1.003213 | -2.28181 | 0.022501 | 0.045745 | Down |
| SOX9-AS1 | 39.10708903 | -1.75483188 | 0.190429 | -9.21516 | 3.11E-20 | 5.90E-19 | Down |
| SP3P | 2.723285171 | -1.389654218 | 0.508204 | -2.73444 | 0.006249 | 0.015085 | Down |
| SP5 | 6.591170621 | -1.635351127 | 0.34105 | -4.79505 | 1.63E-06 | 8.12E-06 | Down |
| SPACA5 | 2.142195644 | -1.192135525 | 0.257897 | -4.62252 | 3.79E-06 | 1.79E-05 | Down |
| SPAG5 | 310.5186994 | -1.20120074 | 0.111414 | -10.7815 | 4.21E-27 | 1.31E-25 | Down |
| SPAM1 | 1.858419968 | -2.176570186 | 0.836538 | -2.60188 | 0.009271 | 0.021311 | Down |
| SPANXA2-OT1 | 4.11614868 | 1.090770429 | 0.33029 | 3.302465 | 0.000958 | 0.002859 | Up |
| SPANXN1 | 1.921575567 | -2.850656146 | 0.988292 | -2.88443 | 0.003921 | 0.009997 | Down |
| SPANXN2 | 1.145503113 | -2.790160944 | 1.032151 | -2.70325 | 0.006867 | 0.016393 | Down |
| SPANXN5 | 1.510384085 | -3.178227959 | 1.379456 | -2.30397 | 0.021224 | 0.043513 | Down |
| SPARC | 19808.25612 | 1.294318264 | 0.105375 | 12.28301 | 1.12E-34 | 5.61E-33 | Up |
| SPATA16 | 1.266496914 | -1.965848799 | 0.81307 | -2.41781 | 0.015614 | 0.033415 | Down |
| SPATA18 | 140.0721421 | 1.364649619 | 0.173351 | 7.872155 | 3.49E-15 | 4.45E-14 | Up |
| SPATA22 | 3.424393704 | -1.257745536 | 0.360262 | -3.4912 | 0.000481 | 0.001537 | Down |
| SPATA31A3 | 9.744991366 | -1.819004924 | 0.643428 | -2.82705 | 0.004698 | 0.011737 | Down |
| SPATA31A7 | 8.255406098 | -1.589561209 | 0.638968 | -2.4877 | 0.012857 | 0.028358 | Down |
| SPATA4 | 18.60169438 | -1.440507686 | 0.115504 | -12.4715 | 1.07E-35 | 5.79E-34 | Down |
| SPATA9 | 11.8387133 | 1.232021084 | 0.131889 | 9.341377 | 9.51E-21 | 1.88E-19 | Up |
| SPCS2P1 | 1.310246503 | -2.787091539 | 0.912278 | -3.05509 | 0.00225 | 0.006115 | Down |
| SPCS2P3 | 1.739022241 | -2.847744721 | 0.654023 | -4.35419 | 1.34E-05 | 5.78E-05 | Down |
| SPDYE7P | 1.23659016 | -1.785583766 | 0.484217 | -3.68757 | 0.000226 | 0.000779 | Down |
| SPECC1 | 1247.172291 | 1.127761299 | 0.079374 | 14.20813 | 8.16E-46 | 8.19E-44 | Up |
| SPHKAP | 24.9163875 | -2.512699175 | 0.362405 | -6.9334 | 4.11E-12 | 3.92E-11 | Down |
| SPIC | 7.8959237 | -1.300728379 | 0.516558 | -2.51807 | 0.0118 | 0.026311 | Down |
| SPINK2 | 19.29272582 | 1.380743562 | 0.215571 | 6.405061 | 1.50E-10 | 1.23E-09 | Up |
| SPINK5 | 60.85523398 | -1.700559304 | 0.293195 | -5.80009 | 6.63E-09 | 4.52E-08 | Down |
| SPINK6 | 0.801829545 | 1.605158905 | 0.396613 | 4.047165 | 5.18E-05 | 0.000202 | Up |
| SPINT1 | 2433.317409 | 1.708916192 | 0.083172 | 20.54675 | 8.23E-94 | 5.82E-91 | Up |
| SPOCD1 | 30.05055124 | 1.698498811 | 0.248935 | 6.823051 | 8.91E-12 | 8.20E-11 | Up |
| SPOCK1 | 610.3385326 | 1.69766896 | 0.146898 | 11.55677 | 6.82E-31 | 2.78E-29 | Up |
| SPOCK2 | 3774.713248 | 1.84611279 | 0.156834 | 11.77114 | 5.50E-32 | 2.41E-30 | Up |
| SPON1 | 406.5476383 | 1.136910979 | 0.169907 | 6.69138 | 2.21E-11 | 1.95E-10 | Up |
| SPP1 | 965.5649927 | 1.538356513 | 0.235776 | 6.524647 | 6.82E-11 | 5.76E-10 | Up |
| SPRR1B | 2.111319249 | 2.482605477 | 0.595025 | 4.172271 | 3.02E-05 | 0.000123 | Up |
| SPRR2D | 1.029807976 | 2.138202669 | 0.761005 | 2.809709 | 0.004959 | 0.012293 | Up |
| SPRY1 | 1310.038066 | 1.144715617 | 0.093768 | 12.20797 | 2.82E-34 | 1.39E-32 | Up |
| SPSB4 | 16.64285454 | -1.440681018 | 0.276426 | -5.21181 | 1.87E-07 | 1.07E-06 | Down |
| SPTBN2 | 393.1705672 | 2.379630986 | 0.160443 | 14.8316 | 9.15E-50 | 1.11E-47 | Up |
| SPX | 339.4079362 | -2.387017222 | 0.160446 | -14.8774 | 4.62E-50 | 5.70E-48 | Down |
| SRCIN1 | 144.3065584 | 1.744503703 | 0.208672 | 8.360014 | 6.27E-17 | 9.16E-16 | Up |
| SRD5A1P1 | 1.090333736 | -2.863004857 | 1.187496 | -2.41096 | 0.015911 | 0.03396 | Down |
| SRD5A3P1 | 1.223425713 | -2.145606926 | 0.794552 | -2.7004 | 0.006926 | 0.016509 | Down |
| SRGAP3-AS4 | 1.795702836 | -1.050505491 | 0.301468 | -3.48464 | 0.000493 | 0.00157 | Down |
| SRL | 362.9523815 | 1.327688142 | 0.127118 | 10.44455 | 1.55E-25 | 4.32E-24 | Up |
| SRP72P1 | 2.060147942 | -3.088782703 | 0.956556 | -3.22907 | 0.001242 | 0.003613 | Down |
| SRPK2P | 1.964573244 | -1.915607223 | 0.647835 | -2.95694 | 0.003107 | 0.00814 | Down |
| SRPX2 | 122.6068267 | 1.460504134 | 0.151167 | 9.661521 | 4.39E-22 | 9.51E-21 | Up |
| SRRM1P3 | 19.55946194 | -1.334656516 | 0.264973 | -5.03696 | 4.73E-07 | 2.55E-06 | Down |
| SRRM4 | 8.114624302 | -1.5021102 | 0.271671 | -5.52914 | 3.22E-08 | 2.02E-07 | Down |
| SRSF10P1 | 1.72508839 | -1.807178433 | 0.5237 | -3.45079 | 0.000559 | 0.001758 | Down |
| SSC4D | 80.93800733 | -1.083228969 | 0.1387 | -7.80989 | 5.72E-15 | 7.19E-14 | Down |
| SSPO | 96.72163911 | -1.298144898 | 0.194894 | -6.66078 | 2.72E-11 | 2.38E-10 | Down |
| SSR1P1 | 62.6782202 | -1.604799903 | 0.251367 | -6.38429 | 1.72E-10 | 1.40E-09 | Down |
| SSTR1 | 14.72774602 | -1.242941965 | 0.226533 | -5.48681 | 4.09E-08 | 2.54E-07 | Down |
| SSTR3 | 46.41090084 | 1.030943356 | 0.331155 | 3.113178 | 0.001851 | 0.005152 | Up |
| SSTR4 | 2.508136362 | -1.882309654 | 0.461833 | -4.07574 | 4.59E-05 | 0.000181 | Down |
| SSTR5-AS1 | 2.212089967 | -1.785008315 | 0.711435 | -2.50902 | 0.012107 | 0.026912 | Down |
| SSX1 | 23.63970738 | 1.54136284 | 0.243072 | 6.341172 | 2.28E-10 | 1.83E-09 | Up |
| SSX2B | 15.30443642 | -1.035407118 | 0.264865 | -3.90919 | 9.26E-05 | 0.000343 | Down |
| SSX3 | 1.62445885 | -1.184524289 | 0.448468 | -2.64127 | 0.00826 | 0.019269 | Down |
| SSX4 | 1.737616941 | -2.696734407 | 0.58698 | -4.59425 | 4.34E-06 | 2.03E-05 | Down |
| SSX4B | 1.756855822 | -2.645240218 | 0.579806 | -4.56229 | 5.06E-06 | 2.34E-05 | Down |
| SSX7 | 1.443692217 | -2.685012439 | 0.990407 | -2.71102 | 0.006708 | 0.016061 | Down |
| SSXP3 | 1.419735345 | -2.610915757 | 0.833803 | -3.13133 | 0.00174 | 0.004878 | Down |
| ST13P11 | 2.685569626 | -1.574139938 | 0.433035 | -3.63513 | 0.000278 | 0.000938 | Down |
| ST13P12 | 1.412754881 | -2.935460477 | 1.090115 | -2.6928 | 0.007086 | 0.016839 | Down |
| ST13P13 | 1.360846524 | -2.521706154 | 0.765736 | -3.29318 | 0.000991 | 0.002945 | Down |
| ST13P16 | 1.379113961 | -1.376351823 | 0.384347 | -3.58101 | 0.000342 | 0.001131 | Down |
| ST13P19 | 3.333415529 | -1.419618138 | 0.400098 | -3.54818 | 0.000388 | 0.001268 | Down |
| ST13P20 | 3.2800151 | -1.645604655 | 0.30896 | -5.32628 | 1.00E-07 | 5.91E-07 | Down |
| ST13P3 | 4.51314298 | -1.008337824 | 0.357508 | -2.82046 | 0.004795 | 0.011949 | Down |
| ST14 | 2695.374933 | 1.006560159 | 0.07541 | 13.34777 | 1.22E-40 | 8.88E-39 | Up |
| ST3GAL5 | 2269.873393 | 1.190720106 | 0.073734 | 16.14896 | 1.15E-58 | 2.18E-56 | Up |
| ST6GALNAC5 | 166.2340585 | 3.575373925 | 0.278469 | 12.8394 | 9.86E-38 | 6.00E-36 | Up |
| ST7-AS1 | 55.80450418 | -1.319654365 | 0.094253 | -14.0012 | 1.53E-44 | 1.43E-42 | Down |
| ST8SIA5 | 15.47139604 | -1.558993129 | 0.314362 | -4.95922 | 7.08E-07 | 3.72E-06 | Down |
| ST8SIA6-AS1 | 3.012592245 | -1.756690954 | 0.381672 | -4.60262 | 4.17E-06 | 1.96E-05 | Down |
| STAB2 | 211.4284965 | -2.320687595 | 0.211908 | -10.9514 | 6.54E-28 | 2.17E-26 | Down |
| STAC | 112.5636445 | 1.979659759 | 0.202654 | 9.768673 | 1.53E-22 | 3.45E-21 | Up |
| STARD13 | 1858.020793 | -1.364767281 | 0.099055 | -13.7779 | 3.46E-43 | 2.94E-41 | Down |
| STARD13-AS | 123.3444496 | -1.607485898 | 0.183453 | -8.76237 | 1.91E-18 | 3.15E-17 | Down |
| STAT1 | 6399.306431 | 1.089119194 | 0.144018 | 7.562401 | 3.96E-14 | 4.57E-13 | Up |
| STATH | 1.340189265 | -2.641594462 | 1.028481 | -2.56844 | 0.010216 | 0.023165 | Down |
| STAU2-AS1 | 9.07838385 | -1.098349531 | 0.276713 | -3.96927 | 7.21E-05 | 0.000273 | Down |
| STEAP2-AS1 | 1.266286942 | -1.883039617 | 0.470735 | -4.00021 | 6.33E-05 | 0.000242 | Down |
| STEAP3 | 329.9892894 | 1.003659525 | 0.109396 | 9.174541 | 4.54E-20 | 8.48E-19 | Up |
| STIP1P3 | 1.560976931 | -2.06606774 | 0.739459 | -2.79403 | 0.005206 | 0.012849 | Down |
| STK25P1 | 1.892131157 | -1.346174895 | 0.338352 | -3.97862 | 6.93E-05 | 0.000263 | Down |
| STK31 | 17.00092578 | -1.288243564 | 0.200831 | -6.41457 | 1.41E-10 | 1.16E-09 | Down |
| STK32A | 1213.033335 | 2.295106234 | 0.151185 | 15.18075 | 4.74E-52 | 6.34E-50 | Up |
| STK32B | 63.26167525 | -1.262045293 | 0.117579 | -10.7336 | 7.08E-27 | 2.16E-25 | Down |
| STMN2 | 45.78715225 | 3.316179317 | 0.307719 | 10.77666 | 4.44E-27 | 1.38E-25 | Up |
| STMND1 | 2.004825686 | -2.042108739 | 0.831076 | -2.45719 | 0.014003 | 0.030478 | Down |
| STOX2 | 922.5738631 | -1.158852597 | 0.085957 | -13.4818 | 2.00E-41 | 1.53E-39 | Down |
| STPG2 | 21.85179543 | -1.004477115 | 0.132585 | -7.5761 | 3.56E-14 | 4.14E-13 | Down |
| STRA6 | 190.9164318 | 3.217266033 | 0.23329 | 13.79084 | 2.89E-43 | 2.47E-41 | Up |
| STRADB | 694.3719118 | 1.01001824 | 0.07864 | 12.84353 | 9.35E-38 | 5.69E-36 | Up |
| STRADBP1 | 1.361096703 | -2.74719025 | 0.819809 | -3.35101 | 0.000805 | 0.00244 | Down |
| STRC | 25.66656518 | -1.47124647 | 0.243437 | -6.04365 | 1.51E-09 | 1.11E-08 | Down |
| STRCP1 | 4.354544289 | -1.035880307 | 0.363665 | -2.84845 | 0.004393 | 0.011059 | Down |
| STX3 | 760.1875009 | 1.087611726 | 0.070759 | 15.3706 | 2.58E-53 | 3.71E-51 | Up |
| STXBP5L | 561.3240105 | -2.511020714 | 0.187791 | -13.3714 | 8.89E-41 | 6.52E-39 | Down |
| STXBP6 | 1290.079343 | 1.035954842 | 0.124737 | 8.305114 | 9.97E-17 | 1.43E-15 | Up |
| SUCLA2P1 | 2.084152101 | -2.406374861 | 0.609332 | -3.9492 | 7.84E-05 | 0.000294 | Down |
| SUCNR1 | 620.7898311 | -1.181126752 | 0.139007 | -8.49687 | 1.95E-17 | 2.97E-16 | Down |
| SULT1A2 | 77.21788331 | -1.256555064 | 0.113738 | -11.0478 | 2.25E-28 | 7.72E-27 | Down |
| SULT1C4 | 57.88644495 | -1.237451438 | 0.119164 | -10.3844 | 2.92E-25 | 7.97E-24 | Down |
| SULT2B1 | 41.41873686 | 1.267878211 | 0.163037 | 7.776613 | 7.45E-15 | 9.25E-14 | Up |
| SULT4A1 | 2.487222455 | 1.703001873 | 0.403322 | 4.22244 | 2.42E-05 | 1.00E-04 | Up |
| SUMO2P14 | 1.770224476 | -1.036468236 | 0.396209 | -2.61596 | 0.008898 | 0.020578 | Down |
| SUPT20HL1 | 2.057910325 | -1.380798951 | 0.561326 | -2.45989 | 0.013898 | 0.030295 | Down |
| SUPT20HL2 | 1.988741816 | -1.829010554 | 0.611115 | -2.9929 | 0.002763 | 0.00732 | Down |
| SUSD4 | 111.1055642 | 1.599356074 | 0.128663 | 12.43058 | 1.78E-35 | 9.53E-34 | Up |
| SV2B | 106.1648308 | -1.017676801 | 0.187445 | -5.42922 | 5.66E-08 | 3.44E-07 | Down |
| SVEP1 | 1457.93401 | -1.229884255 | 0.150392 | -8.17786 | 2.89E-16 | 3.99E-15 | Down |
| SYCE1 | 8.836019884 | -1.989172352 | 0.278567 | -7.14074 | 9.28E-13 | 9.42E-12 | Down |
| SYN2 | 9.661180338 | -1.321378944 | 0.250412 | -5.27682 | 1.31E-07 | 7.64E-07 | Down |
| SYN3 | 13.7737188 | -1.128325835 | 0.198844 | -5.67442 | 1.39E-08 | 9.15E-08 | Down |
| SYNDIG1 | 148.6149123 | -1.464709409 | 0.134092 | -10.9231 | 8.93E-28 | 2.93E-26 | Down |
| SYNDIG1L | 22.38600387 | 2.581450864 | 0.209475 | 12.32342 | 6.78E-35 | 3.48E-33 | Up |
| SYNE1 | 12412.21907 | -1.332643504 | 0.106903 | -12.4659 | 1.15E-35 | 6.19E-34 | Down |
| SYNE1-AS1 | 6.837752418 | -1.179148406 | 0.17459 | -6.7538 | 1.44E-11 | 1.30E-10 | Down |
| SYNGR1 | 299.324428 | -1.065716311 | 0.088778 | -12.0043 | 3.37E-33 | 1.57E-31 | Down |
| SYNGR4 | 1.50264056 | -1.456638725 | 0.494809 | -2.94384 | 0.003242 | 0.008448 | Down |
| SYNM | 1076.467167 | -1.2663124 | 0.132764 | -9.53807 | 1.46E-21 | 3.05E-20 | Down |
| SYNPO2L | 12.58104163 | -1.531526525 | 0.284285 | -5.38729 | 7.15E-08 | 4.29E-07 | Down |
| SYP | 73.03689195 | -1.304283639 | 0.13404 | -9.73058 | 2.23E-22 | 4.95E-21 | Down |
| SYT1 | 120.5421762 | 2.054713491 | 0.178957 | 11.48157 | 1.63E-30 | 6.47E-29 | Up |
| SYT12 | 686.4811942 | 4.60517928 | 0.271367 | 16.9703 | 1.36E-64 | 3.20E-62 | Up |
| SYT15 | 244.2964391 | -1.025645811 | 0.139553 | -7.34949 | 1.99E-13 | 2.15E-12 | Down |
| SYT16 | 34.19417374 | -1.114782391 | 0.197527 | -5.64369 | 1.66E-08 | 1.09E-07 | Down |
| SYT3 | 13.70352829 | -1.36484222 | 0.243209 | -5.61181 | 2.00E-08 | 1.29E-07 | Down |
| SYT4 | 4.892633765 | -1.36809097 | 0.361358 | -3.78597 | 0.000153 | 0.000545 | Down |
| SYT9 | 81.94676064 | -1.679343916 | 0.134115 | -12.5217 | 5.68E-36 | 3.13E-34 | Down |
| SYTL1 | 690.6346097 | 1.517217057 | 0.107065 | 14.17093 | 1.39E-45 | 1.38E-43 | Up |
| SYTL3 | 698.117215 | -1.123989025 | 0.088391 | -12.7161 | 4.81E-37 | 2.81E-35 | Down |
| SYTL5 | 505.1865069 | 4.655964088 | 0.243495 | 19.12139 | 1.68E-81 | 7.56E-79 | Up |
| T | 2.013820297 | -1.272713401 | 0.417588 | -3.04777 | 0.002305 | 0.006248 | Down |
| TAAR3 | 1.551235672 | -2.679509639 | 0.969395 | -2.7641 | 0.005708 | 0.013923 | Down |
| TAAR4P | 1.581968903 | -2.880529049 | 1.227224 | -2.34719 | 0.018916 | 0.039393 | Down |
| TAAR5 | 1.796937473 | -3.282833549 | 0.833259 | -3.93975 | 8.16E-05 | 0.000305 | Down |
| TAAR6 | 1.505851423 | -2.226229812 | 0.854467 | -2.6054 | 0.009177 | 0.021122 | Down |
| TACR3 | 3.096413711 | -1.406664547 | 0.304293 | -4.62274 | 3.79E-06 | 1.79E-05 | Down |
| TACSTD2 | 3988.254979 | 4.24375895 | 0.212298 | 19.98959 | 6.79E-89 | 4.28E-86 | Up |
| TAGLN3 | 11.72875804 | 2.374672807 | 0.319224 | 7.438887 | 1.02E-13 | 1.13E-12 | Up |
| TAL1 | 192.1432276 | -1.135653837 | 0.116341 | -9.76143 | 1.65E-22 | 3.69E-21 | Down |
| TAPT1-AS1 | 109.7585519 | -1.073131704 | 0.092333 | -11.6224 | 3.17E-31 | 1.32E-29 | Down |
| TARDBPP1 | 2.300773343 | -2.123136902 | 0.617036 | -3.44086 | 0.00058 | 0.001817 | Down |
| TARDBPP2 | 5.11085878 | -1.474752311 | 0.374667 | -3.93616 | 8.28E-05 | 0.000309 | Down |
| TARID | 12.13459331 | -1.473506424 | 0.285657 | -5.15831 | 2.49E-07 | 1.40E-06 | Down |
| TAS1R1 | 4.945709781 | -1.437088197 | 0.231645 | -6.20385 | 5.51E-10 | 4.25E-09 | Down |
| TAS2R1 | 1.375128658 | -2.022990029 | 0.689789 | -2.93277 | 0.00336 | 0.008721 | Down |
| TAS2R16 | 1.004236379 | -2.063342305 | 0.898703 | -2.29591 | 0.021681 | 0.044298 | Down |
| TAS2R40 | 2.606114337 | -1.628188909 | 0.38487 | -4.23049 | 2.33E-05 | 9.68E-05 | Down |
| TAS2R62P | 2.191977341 | -1.672830238 | 0.520525 | -3.21374 | 0.00131 | 0.003786 | Down |
| TAS2R7 | 1.664288528 | -1.902936835 | 0.726016 | -2.62107 | 0.008766 | 0.020308 | Down |
| TAS2R8 | 1.208428905 | -1.274835725 | 0.449411 | -2.83668 | 0.004559 | 0.011428 | Down |
| TAS2R9 | 1.626250201 | -1.339134214 | 0.485116 | -2.76044 | 0.005772 | 0.014061 | Down |
| TAT-AS1 | 1.049811559 | -1.78200756 | 0.506634 | -3.51735 | 0.000436 | 0.001407 | Down |
| TATDN2P1 | 4.275529616 | -2.414576158 | 0.373066 | -6.47225 | 9.66E-11 | 8.05E-10 | Down |
| TBC1D2 | 832.3666905 | 2.060748808 | 0.091512 | 22.51881 | 2.72E-112 | 3.60E-109 | Up |
| TBC1D26 | 3.322208148 | -1.693810362 | 0.44316 | -3.82212 | 0.000132 | 0.000476 | Down |
| TBC1D28 | 2.496531874 | -2.458202945 | 0.777061 | -3.16346 | 0.001559 | 0.004423 | Down |
| TBC1D29 | 7.188640426 | -1.752097029 | 0.280636 | -6.2433 | 4.28E-10 | 3.35E-09 | Down |
| TBC1D4 | 2523.527071 | -1.386762465 | 0.096272 | -14.4046 | 4.84E-47 | 5.30E-45 | Down |
| TBCAP2 | 1.660688971 | -1.770245965 | 0.377921 | -4.68417 | 2.81E-06 | 1.36E-05 | Down |
| TBPL2 | 2.531586425 | -3.127698468 | 0.698976 | -4.47469 | 7.65E-06 | 3.45E-05 | Down |
| TBR1 | 4.010875231 | -1.176485324 | 0.427929 | -2.74925 | 0.005973 | 0.014494 | Down |
| TBX1 | 71.80143282 | -1.877829476 | 0.165283 | -11.3613 | 6.52E-30 | 2.47E-28 | Down |
| TBX2-AS1 | 30.26848397 | -1.253695873 | 0.201231 | -6.23013 | 4.66E-10 | 3.63E-09 | Down |
| TBX22 | 98.42997028 | -2.632647942 | 0.258236 | -10.1947 | 2.09E-24 | 5.34E-23 | Down |
| TBX4 | 3.728828378 | -1.36531094 | 0.384884 | -3.54733 | 0.000389 | 0.001271 | Down |
| TBX5 | 34.38010151 | -1.72828684 | 0.27476 | -6.29017 | 3.17E-10 | 2.51E-09 | Down |
| TBX5-AS1 | 11.39481655 | -1.320922911 | 0.355624 | -3.71438 | 0.000204 | 0.000707 | Down |
| TCAP | 42.18906328 | -1.390377376 | 0.222705 | -6.24314 | 4.29E-10 | 3.35E-09 | Down |
| TCEA1P3 | 3.027176182 | -2.987810945 | 0.360145 | -8.29612 | 1.08E-16 | 1.54E-15 | Down |
| TCEAL2 | 338.172651 | -1.667384559 | 0.181762 | -9.17344 | 4.58E-20 | 8.56E-19 | Down |
| TCEAL4P1 | 1.414249884 | -2.557002027 | 0.599222 | -4.2672 | 1.98E-05 | 8.31E-05 | Down |
| TCEAL5 | 19.29639805 | -2.391782417 | 0.211304 | -11.3192 | 1.05E-29 | 3.96E-28 | Down |
| TCEAL6 | 5.524813021 | -2.00083361 | 0.37825 | -5.28972 | 1.23E-07 | 7.15E-07 | Down |
| TCEB1P20 | 0.669791396 | 2.571605916 | 0.465874 | 5.519957 | 3.39E-08 | 2.13E-07 | Up |
| TCEB1P33 | 2.212720341 | -1.084461417 | 0.3331 | -3.25566 | 0.001131 | 0.003318 | Down |
| TCEB3B | 19.72600088 | -1.733155734 | 0.224815 | -7.70924 | 1.27E-14 | 1.54E-13 | Down |
| TCEB3C | 102.8188455 | -2.172692464 | 0.271295 | -8.00859 | 1.16E-15 | 1.53E-14 | Down |
| TCEB3CL | 26.80029647 | -1.723195961 | 0.331381 | -5.20004 | 1.99E-07 | 1.13E-06 | Down |
| TCEB3CL2 | 41.07738458 | -1.544626689 | 0.388878 | -3.97201 | 7.13E-05 | 0.00027 | Down |
| TCERG1L | 1003.556682 | 1.264219917 | 0.163019 | 7.755039 | 8.83E-15 | 1.09E-13 | Up |
| TCERG1L-AS1 | 1.471007663 | 1.01788305 | 0.348945 | 2.917029 | 0.003534 | 0.009121 | Up |
| TCF24 | 1.686026672 | -2.079506065 | 0.472581 | -4.40032 | 1.08E-05 | 4.75E-05 | Down |
| TCF7L1 | 146.7745508 | -1.846810004 | 0.142507 | -12.9595 | 2.08E-38 | 1.31E-36 | Down |
| TCF7L1-IT1 | 3.956973545 | -2.041830909 | 0.282416 | -7.22987 | 4.83E-13 | 5.02E-12 | Down |
| TCHHL1 | 2.854558678 | -1.337830287 | 0.539018 | -2.48198 | 0.013066 | 0.028749 | Down |
| TCL1A | 107.131213 | -1.480870481 | 0.520102 | -2.84727 | 0.00441 | 0.011096 | Down |
| TCL1B | 2.830618885 | -2.202672585 | 0.722329 | -3.0494 | 0.002293 | 0.006216 | Down |
| TCL6 | 41.56287448 | -1.869795156 | 0.36981 | -5.0561 | 4.28E-07 | 2.32E-06 | Down |
| TCN1 | 7.083254035 | 2.306793089 | 0.383149 | 6.020613 | 1.74E-09 | 1.27E-08 | Up |
| TCP10L2 | 2.188633656 | -1.056334871 | 0.424826 | -2.48651 | 0.0129 | 0.028444 | Down |
| TCP11X2 | 2.445739291 | -2.066759425 | 0.774676 | -2.6679 | 0.007633 | 0.017977 | Down |
| TCP1P1 | 1.432435366 | -1.795557065 | 0.662975 | -2.70833 | 0.006762 | 0.016177 | Down |
| TCTE1 | 4.977145034 | -1.881446989 | 0.318882 | -5.90013 | 3.63E-09 | 2.55E-08 | Down |
| TDGF1 | 20.99741472 | -1.809374531 | 0.214232 | -8.44588 | 3.02E-17 | 4.53E-16 | Down |
| TDGF1P3 | 1.123386625 | -1.666577705 | 0.711794 | -2.34138 | 0.019213 | 0.039922 | Down |
| TDGF1P7 | 0.931567785 | -1.155857059 | 0.336164 | -3.43837 | 0.000585 | 0.001833 | Down |
| TDGP1 | 1.534061864 | -2.316071223 | 0.649824 | -3.56415 | 0.000365 | 0.0012 | Down |
| TDH | 17.67238194 | -2.226840147 | 0.184423 | -12.0746 | 1.44E-33 | 6.83E-32 | Down |
| TDO2 | 27.86519324 | 1.164796677 | 0.332959 | 3.498322 | 0.000468 | 0.0015 | Up |
| TDRD1 | 11.01046022 | -1.079139934 | 0.208592 | -5.17345 | 2.30E-07 | 1.30E-06 | Down |
| TDRD12 | 24.02511775 | -2.256136951 | 0.269101 | -8.38398 | 5.12E-17 | 7.54E-16 | Down |
| TDRD15 | 12.47029323 | -2.308604567 | 0.448248 | -5.15029 | 2.60E-07 | 1.45E-06 | Down |
| TDRD5 | 36.28513275 | -1.04242037 | 0.141104 | -7.38758 | 1.50E-13 | 1.64E-12 | Down |
| TDRD6 | 267.718133 | -1.344009933 | 0.115884 | -11.5978 | 4.23E-31 | 1.75E-29 | Down |
| TDRD9 | 246.1168546 | -1.850133395 | 0.187182 | -9.88416 | 4.88E-23 | 1.13E-21 | Down |
| TDRG1 | 1.364123652 | -3.124507458 | 1.20966 | -2.58296 | 0.009796 | 0.022355 | Down |
| TECRL | 7.546300898 | -1.748477573 | 0.416501 | -4.19802 | 2.69E-05 | 0.00011 | Down |
| TECTB | 5.230895053 | -1.823111859 | 0.308519 | -5.90923 | 3.44E-09 | 2.42E-08 | Down |
| TEDDM1 | 10.12641926 | -1.31188687 | 0.260058 | -5.0446 | 4.54E-07 | 2.46E-06 | Down |
| TEKT1 | 5.407724134 | -2.010809199 | 0.321016 | -6.26389 | 3.75E-10 | 2.95E-09 | Down |
| TEKT3 | 10.16847464 | -1.092830293 | 0.165926 | -6.58626 | 4.51E-11 | 3.87E-10 | Down |
| TEKT4 | 14.27518507 | -2.273017134 | 0.227746 | -9.98051 | 1.86E-23 | 4.45E-22 | Down |
| TENM1 | 1706.824574 | 3.439884118 | 0.18799 | 18.29826 | 8.54E-75 | 3.05E-72 | Up |
| TENM2 | 131.5052164 | -1.622505775 | 0.214456 | -7.56567 | 3.86E-14 | 4.46E-13 | Down |
| TERF1P2 | 1.625331709 | -1.689739363 | 0.690725 | -2.44633 | 0.014432 | 0.031271 | Down |
| TERT | 19.0973512 | -1.02712795 | 0.410273 | -2.50352 | 0.012296 | 0.027278 | Down |
| TESC | 722.3959266 | 2.778473147 | 0.161886 | 17.1632 | 5.01E-66 | 1.23E-63 | Up |
| TET1P1 | 3.828004548 | -1.325010277 | 0.475451 | -2.78685 | 0.005322 | 0.013105 | Down |
| TEX11 | 1.208366378 | 1.646122594 | 0.483598 | 3.40391 | 0.000664 | 0.002055 | Up |
| TEX13A | 1.269375293 | -1.942242828 | 0.858203 | -2.26315 | 0.023626 | 0.0477 | Down |
| TEX15 | 8.106252268 | -1.467322013 | 0.311605 | -4.70891 | 2.49E-06 | 1.21E-05 | Down |
| TEX19 | 2.15916929 | -1.683072764 | 0.527542 | -3.1904 | 0.001421 | 0.004069 | Down |
| TEX29 | 1.622814601 | -1.142267277 | 0.292869 | -3.90026 | 9.61E-05 | 0.000354 | Down |
| TEX35 | 2.777051215 | -2.606278911 | 0.709733 | -3.6722 | 0.00024 | 0.000822 | Down |
| TEX40 | 1.36727428 | -2.133619621 | 0.508089 | -4.1993 | 2.68E-05 | 0.00011 | Down |
| TF | 30.55427714 | -1.27553284 | 0.208403 | -6.12052 | 9.33E-10 | 7.03E-09 | Down |
| TFAP2D | 1.789774128 | -2.728322095 | 0.830445 | -3.28537 | 0.001018 | 0.00302 | Down |
| TFCP2L1 | 2204.625807 | -2.404562667 | 0.181324 | -13.2611 | 3.89E-40 | 2.74E-38 | Down |
| TFDP3 | 1.279613801 | -2.048233434 | 0.832497 | -2.46035 | 0.01388 | 0.030265 | Down |
| TFF1 | 1.228979467 | -2.786074249 | 0.425432 | -6.54881 | 5.80E-11 | 4.94E-10 | Down |
| TFF2 | 8.978789521 | -2.707294092 | 0.279637 | -9.68147 | 3.61E-22 | 7.88E-21 | Down |
| TFF3 | 7875.642046 | -2.827313431 | 0.280177 | -10.0912 | 6.04E-24 | 1.50E-22 | Down |
| TFPI | 1192.76467 | -1.58294456 | 0.128517 | -12.317 | 7.34E-35 | 3.74E-33 | Down |
| TG | 1085898.215 | -1.20442219 | 0.138215 | -8.71413 | 2.93E-18 | 4.75E-17 | Down |
| TGFA | 1186.485021 | 2.768534665 | 0.115543 | 23.96109 | 7.08E-127 | 1.94E-123 | Up |
| TGFB2 | 497.805707 | 1.483190887 | 0.128178 | 11.57136 | 5.76E-31 | 2.35E-29 | Up |
| TGFB2-OT1 | 18.88864806 | 1.190271624 | 0.499811 | 2.381442 | 0.017245 | 0.036399 | Up |
| TGFBI | 2563.880658 | 1.501985443 | 0.121614 | 12.35044 | 4.84E-35 | 2.52E-33 | Up |
| TGFBR1 | 3611.755264 | 1.720825902 | 0.078893 | 21.81227 | 1.77E-105 | 1.82E-102 | Up |
| TGFBR3 | 1116.427712 | -1.111697336 | 0.092104 | -12.07 | 1.52E-33 | 7.21E-32 | Down |
| TGFBR3L | 10.27241279 | 3.072977648 | 0.284869 | 10.78732 | 3.95E-27 | 1.23E-25 | Up |
| TGM1 | 91.1858099 | 1.387411779 | 0.151095 | 9.182404 | 4.22E-20 | 7.91E-19 | Up |
| TGM2 | 3172.36203 | 1.408769752 | 0.14017 | 10.05047 | 9.14E-24 | 2.25E-22 | Up |
| TGM4 | 10.89883377 | -1.486731699 | 0.288371 | -5.15563 | 2.53E-07 | 1.42E-06 | Down |
| THBS1 | 4203.13835 | 2.269000519 | 0.170014 | 13.34596 | 1.25E-40 | 9.08E-39 | Up |
| THBS2 | 2374.818938 | 2.002955406 | 0.144648 | 13.84705 | 1.33E-43 | 1.16E-41 | Up |
| THEG | 10.74849426 | -1.325738235 | 0.428862 | -3.09129 | 0.001993 | 0.005499 | Down |
| THEM5 | 3.971705626 | 1.352009024 | 0.283739 | 4.764982 | 1.89E-06 | 9.35E-06 | Up |
| THRB-AS1 | 14.38460086 | -1.677656281 | 0.213145 | -7.87096 | 3.52E-15 | 4.49E-14 | Down |
| THRB-IT1 | 13.78308168 | -1.201289095 | 0.212122 | -5.6632 | 1.49E-08 | 9.74E-08 | Down |
| THRSP | 119.1563937 | 1.474602571 | 0.172195 | 8.563572 | 1.09E-17 | 1.70E-16 | Up |
| TIAM1 | 1277.932038 | 2.478962692 | 0.135048 | 18.35616 | 2.95E-75 | 1.10E-72 | Up |
| TIMD4 | 77.27396411 | -1.597212527 | 0.374306 | -4.26713 | 1.98E-05 | 8.32E-05 | Down |
| TIMM8AP1 | 1.90904518 | 2.95780708 | 0.347218 | 8.518589 | 1.62E-17 | 2.48E-16 | Up |
| TIMP1 | 5344.968896 | 2.751379479 | 0.104318 | 26.37498 | 2.65E-153 | 1.82E-149 | Up |
| TIMP4 | 55.31110321 | -2.119909007 | 0.201323 | -10.5299 | 6.29E-26 | 1.80E-24 | Down |
| TINCR | 21.96459117 | 2.071643352 | 0.337903 | 6.130874 | 8.74E-10 | 6.61E-09 | Up |
| TIPARP | 2302.292461 | 1.734321247 | 0.126354 | 13.72592 | 7.10E-43 | 5.95E-41 | Up |
| TKTL1 | 4.842958225 | -2.052506291 | 0.388883 | -5.27795 | 1.31E-07 | 7.60E-07 | Down |
| TLE1 | 580.3007871 | -1.088980659 | 0.073338 | -14.8488 | 7.08E-50 | 8.67E-48 | Down |
| TLR12P | 1.908798057 | -1.923654276 | 0.718347 | -2.67789 | 0.007409 | 0.017507 | Down |
| TLR2 | 338.7899162 | 1.132771864 | 0.129263 | 8.763337 | 1.90E-18 | 3.12E-17 | Up |
| TLX3 | 1.502269272 | -3.040376448 | 0.923737 | -3.29139 | 0.000997 | 0.002962 | Down |
| TM4SF1 | 2689.533537 | 1.587520919 | 0.12959 | 12.25038 | 1.67E-34 | 8.30E-33 | Up |
| TM4SF1-AS1 | 9.696942081 | 2.373219236 | 0.225606 | 10.51932 | 7.04E-26 | 2.00E-24 | Up |
| TM4SF20 | 6.563865245 | -1.095062781 | 0.312105 | -3.50864 | 0.00045 | 0.001448 | Down |
| TM4SF4 | 78.5683845 | 3.921928525 | 0.25682 | 15.27114 | 1.19E-52 | 1.63E-50 | Up |
| TMC3-AS1 | 17.55938923 | -1.135576031 | 0.174398 | -6.51142 | 7.44E-11 | 6.27E-10 | Down |
| TMC6 | 1498.873664 | 1.258153673 | 0.170895 | 7.362166 | 1.81E-13 | 1.96E-12 | Up |
| TMCC3 | 1691.537101 | 1.258003796 | 0.093469 | 13.45907 | 2.72E-41 | 2.08E-39 | Up |
| TMCO5B | 2.443439531 | -2.256653146 | 0.637274 | -3.5411 | 0.000398 | 0.001298 | Down |
| TMED11P | 1.241851964 | -1.104818736 | 0.480785 | -2.29795 | 0.021565 | 0.044117 | Down |
| TMEFF2 | 35.61382745 | -1.435888396 | 0.23352 | -6.14888 | 7.80E-10 | 5.94E-09 | Down |
| TMEM100 | 228.6522288 | 2.12513861 | 0.142578 | 14.9051 | 3.05E-50 | 3.78E-48 | Up |
| TMEM108 | 176.0681735 | 1.536419786 | 0.139128 | 11.04318 | 2.37E-28 | 8.12E-27 | Up |
| TMEM108-AS1 | 16.30942427 | 1.105511903 | 0.257187 | 4.298483 | 1.72E-05 | 7.30E-05 | Up |
| TMEM130 | 77.68200397 | 2.375840832 | 0.225232 | 10.54843 | 5.17E-26 | 1.48E-24 | Up |
| TMEM132C | 38.35158919 | -2.029779744 | 0.210976 | -9.6209 | 6.53E-22 | 1.40E-20 | Down |
| TMEM132D | 96.36060191 | 2.274102137 | 0.337798 | 6.732145 | 1.67E-11 | 1.49E-10 | Up |
| TMEM139 | 308.0845533 | -1.808678692 | 0.198122 | -9.1291 | 6.91E-20 | 1.28E-18 | Down |
| TMEM163 | 186.3386216 | 2.11988225 | 0.249008 | 8.513322 | 1.69E-17 | 2.59E-16 | Up |
| TMEM171 | 328.7662373 | -2.252914283 | 0.152193 | -14.803 | 1.40E-49 | 1.68E-47 | Down |
| TMEM173 | 1011.494975 | 1.1170676 | 0.082051 | 13.61429 | 3.29E-42 | 2.64E-40 | Up |
| TMEM174 | 4.709059898 | -2.768828211 | 0.355415 | -7.79041 | 6.68E-15 | 8.34E-14 | Down |
| TMEM178A | 81.19960072 | -1.41086059 | 0.104114 | -13.5511 | 7.80E-42 | 6.11E-40 | Down |
| TMEM184A | 172.6346924 | 1.915218968 | 0.160763 | 11.91333 | 1.01E-32 | 4.58E-31 | Up |
| TMEM196 | 6.852089689 | -2.29501359 | 0.383009 | -5.99206 | 2.07E-09 | 1.50E-08 | Down |
| TMEM198B | 333.3733707 | -1.146253602 | 0.083718 | -13.6919 | 1.14E-42 | 9.42E-41 | Down |
| TMEM200A | 162.0099011 | 1.061723281 | 0.146628 | 7.240948 | 4.46E-13 | 4.65E-12 | Up |
| TMEM207 | 1.666893702 | -2.538651107 | 0.884125 | -2.87137 | 0.004087 | 0.010365 | Down |
| TMEM212 | 1.53416428 | -1.748366342 | 0.62857 | -2.7815 | 0.005411 | 0.013288 | Down |
| TMEM215 | 210.2688855 | 3.912893391 | 0.332328 | 11.7742 | 5.30E-32 | 2.33E-30 | Up |
| TMEM225 | 1.133265995 | -1.663206727 | 0.654049 | -2.54294 | 0.010992 | 0.024691 | Down |
| TMEM232 | 158.0178892 | -1.133802378 | 0.097394 | -11.6414 | 2.54E-31 | 1.07E-29 | Down |
| TMEM235 | 1.515919943 | -2.19160778 | 0.650331 | -3.36999 | 0.000752 | 0.002295 | Down |
| TMEM246 | 305.0127387 | -1.01128345 | 0.159382 | -6.34502 | 2.22E-10 | 1.79E-09 | Down |
| TMEM252 | 15.24796188 | -1.042138067 | 0.236211 | -4.4119 | 1.02E-05 | 4.52E-05 | Down |
| TMEM253 | 12.74460096 | -1.481328822 | 0.201521 | -7.35074 | 1.97E-13 | 2.13E-12 | Down |
| TMEM256P2 | 2.30021718 | 1.027586889 | 0.359789 | 2.856084 | 0.004289 | 0.010824 | Up |
| TMEM257 | 2.446370211 | -2.723356866 | 0.487264 | -5.58908 | 2.28E-08 | 1.46E-07 | Down |
| TMEM31 | 1.678960334 | -1.051218845 | 0.425866 | -2.46843 | 0.013571 | 0.029684 | Down |
| TMEM40 | 4.071743244 | 1.321380414 | 0.409602 | 3.226009 | 0.001255 | 0.003646 | Up |
| TMEM59L | 22.54766049 | 1.093539956 | 0.219138 | 4.990195 | 6.03E-07 | 3.21E-06 | Up |
| TMEM74 | 6.474801154 | -1.416344796 | 0.244056 | -5.80335 | 6.50E-09 | 4.43E-08 | Down |
| TMEM75 | 17.29633829 | -1.070169958 | 0.235849 | -4.53753 | 5.69E-06 | 2.61E-05 | Down |
| TMEM79 | 216.4905292 | 1.623600362 | 0.095255 | 17.04484 | 3.82E-65 | 9.16E-63 | Up |
| TMEM82 | 1.635007192 | -1.752028974 | 0.600974 | -2.91532 | 0.003553 | 0.009165 | Down |
| TMEM88B | 0.886247235 | 2.284745942 | 0.559559 | 4.08312 | 4.44E-05 | 0.000175 | Up |
| TMEM92 | 48.10037028 | 4.11775507 | 0.269068 | 15.3038 | 7.21E-53 | 1.01E-50 | Up |
| TMEM92-AS1 | 1.088592435 | 1.352892103 | 0.454232 | 2.978417 | 0.002897 | 0.007645 | Up |
| TMEM95 | 2.777083862 | -2.805837268 | 0.656401 | -4.27458 | 1.91E-05 | 8.07E-05 | Down |
| TMEM98 | 1586.232192 | 1.229043412 | 0.090457 | 13.58699 | 4.78E-42 | 3.80E-40 | Up |
| TMOD1 | 441.5636928 | -1.41544403 | 0.136336 | -10.3821 | 2.99E-25 | 8.16E-24 | Down |
| TMPOP2 | 2.93615218 | -1.496605361 | 0.32315 | -4.63131 | 3.63E-06 | 1.72E-05 | Down |
| TMPRSS11E | 30.2852546 | 3.630704127 | 0.362685 | 10.01062 | 1.37E-23 | 3.33E-22 | Up |
| TMPRSS11F | 2.446224911 | 1.291973466 | 0.461003 | 2.802529 | 0.00507 | 0.012543 | Up |
| TMPRSS15 | 1.040985975 | -2.131188746 | 0.680831 | -3.13028 | 0.001746 | 0.004894 | Down |
| TMPRSS3 | 119.0053454 | -1.605617057 | 0.163432 | -9.8244 | 8.84E-23 | 2.01E-21 | Down |
| TMPRSS4 | 408.674327 | 3.933601768 | 0.303682 | 12.95302 | 2.26E-38 | 1.42E-36 | Up |
| TMPRSS6 | 496.6817914 | 4.835672773 | 0.292667 | 16.5228 | 2.51E-61 | 5.32E-59 | Up |
| TMPRSS9 | 11.82875886 | -1.217695051 | 0.23051 | -5.28262 | 1.27E-07 | 7.42E-07 | Down |
| TMSB10 | 12330.11657 | 1.103019311 | 0.084799 | 13.00743 | 1.11E-38 | 7.09E-37 | Up |
| TMSB15A | 15.37597988 | -1.656086679 | 0.184284 | -8.98661 | 2.55E-19 | 4.51E-18 | Down |
| TNC | 3164.596317 | 2.088967936 | 0.208267 | 10.03022 | 1.12E-23 | 2.75E-22 | Up |
| TNFAIP6 | 43.62178659 | 2.299077623 | 0.232176 | 9.902321 | 4.07E-23 | 9.49E-22 | Up |
| TNFAIP8L3 | 85.40943119 | -1.045898545 | 0.117211 | -8.92322 | 4.53E-19 | 7.86E-18 | Down |
| TNFRSF10A | 366.3966706 | 1.041768393 | 0.077655 | 13.4154 | 4.91E-41 | 3.70E-39 | Up |
| TNFRSF10B | 1163.919661 | 1.150283182 | 0.065062 | 17.6797 | 6.01E-70 | 1.80E-67 | Up |
| TNFRSF10C | 260.4972245 | 2.035874942 | 0.12634 | 16.11422 | 2.03E-58 | 3.75E-56 | Up |
| TNFRSF11B | 1346.24667 | -1.544347452 | 0.148149 | -10.4243 | 1.92E-25 | 5.30E-24 | Down |
| TNFRSF12A | 1748.665968 | 3.451483073 | 0.184596 | 18.69751 | 5.19E-78 | 2.09E-75 | Up |
| TNFRSF13C | 164.1815297 | -1.060800343 | 0.426944 | -2.48464 | 0.012968 | 0.028562 | Down |
| TNFRSF18 | 36.09429758 | 1.118640878 | 0.241292 | 4.63605 | 3.55E-06 | 1.69E-05 | Up |
| TNFSF15 | 319.6701576 | 1.539163001 | 0.126305 | 12.18609 | 3.69E-34 | 1.80E-32 | Up |
| TNFSF18 | 37.83867525 | 2.997279896 | 0.277172 | 10.81378 | 2.96E-27 | 9.37E-26 | Up |
| TNFSF4 | 77.20181958 | 1.068607955 | 0.176669 | 6.048643 | 1.46E-09 | 1.08E-08 | Up |
| TNFSF9 | 20.42206264 | 1.382751031 | 0.270439 | 5.112987 | 3.17E-07 | 1.75E-06 | Up |
| TNIK | 866.0721926 | 1.3101646 | 0.101877 | 12.86028 | 7.53E-38 | 4.61E-36 | Up |
| TNMD | 4.252060238 | -1.363015504 | 0.308727 | -4.41495 | 1.01E-05 | 4.46E-05 | Down |
| TNN | 97.66883098 | -1.403462538 | 0.194561 | -7.21349 | 5.45E-13 | 5.64E-12 | Down |
| TNNT1 | 6.545936248 | -1.491861539 | 0.48303 | -3.08855 | 0.002011 | 0.005544 | Down |
| TNNT3 | 38.59220012 | -1.979884339 | 0.175974 | -11.251 | 2.29E-29 | 8.43E-28 | Down |
| TNP1 | 0.445914741 | -1.329084176 | 0.480747 | -2.76462 | 0.005699 | 0.013907 | Down |
| TNPO1P3 | 2.067504467 | -2.082944115 | 0.59079 | -3.52569 | 0.000422 | 0.001367 | Down |
| TNRC6C-AS1 | 371.2217291 | 1.807776215 | 0.164038 | 11.02046 | 3.04E-28 | 1.04E-26 | Up |
| TNXB | 5083.234078 | -1.569945042 | 0.137588 | -11.4105 | 3.71E-30 | 1.43E-28 | Down |
| TOP1P1 | 0.635820486 | -1.531523879 | 0.493713 | -3.10205 | 0.001922 | 0.005326 | Down |
| TOPORSLP | 3.221454497 | -1.692831579 | 0.446161 | -3.79421 | 0.000148 | 0.000528 | Down |
| TP53AIP1 | 3.96278498 | -1.556997554 | 0.367967 | -4.23135 | 2.32E-05 | 9.64E-05 | Down |
| TP53TG3D | 6.770814765 | -1.419404681 | 0.385682 | -3.68025 | 0.000233 | 0.000799 | Down |
| TP63 | 118.4610175 | 1.246533416 | 0.203864 | 6.114546 | 9.68E-10 | 7.28E-09 | Up |
| TPD52L1 | 1434.002946 | 1.84224168 | 0.111504 | 16.52169 | 2.56E-61 | 5.39E-59 | Up |
| TPH2 | 3.508481613 | -2.297853746 | 0.594251 | -3.86681 | 0.00011 | 0.000402 | Down |
| TPI1P4 | 1.233649106 | -2.198705909 | 0.544213 | -4.04016 | 5.34E-05 | 0.000207 | Down |
| TPO | 26169.62585 | -2.569402337 | 0.252517 | -10.1752 | 2.56E-24 | 6.50E-23 | Down |
| TPPP | 816.7305246 | -1.995002612 | 0.143859 | -13.8678 | 9.93E-44 | 8.80E-42 | Down |
| TPPP2 | 2.789079939 | -1.876254042 | 0.466371 | -4.0231 | 5.74E-05 | 0.000221 | Down |
| TPRXL | 2.698894121 | -1.383766418 | 0.388824 | -3.55885 | 0.000372 | 0.001222 | Down |
| TPSG1 | 2.310683628 | -1.505524954 | 0.301007 | -5.00163 | 5.68E-07 | 3.03E-06 | Down |
| TPST2P1 | 3.297810037 | -1.106540178 | 0.316246 | -3.49899 | 0.000467 | 0.001496 | Down |
| TPT1P12 | 3.147541791 | -1.229772404 | 0.407564 | -3.01737 | 0.00255 | 0.006821 | Down |
| TPTE2P1 | 46.36492148 | -2.305851492 | 0.189339 | -12.1784 | 4.05E-34 | 1.98E-32 | Down |
| TPTE2P6 | 3.006331533 | -1.520477706 | 0.39898 | -3.81091 | 0.000138 | 0.000496 | Down |
| TRAF6P1 | 1.365166252 | -1.654627907 | 0.646369 | -2.55988 | 0.010471 | 0.023676 | Down |
| TRAPPC13P1 | 1.609584056 | -2.496968601 | 0.817448 | -3.05459 | 0.002254 | 0.006123 | Down |
| TRAV8-5 | 1.074988687 | -1.779538507 | 0.672023 | -2.64803 | 0.008096 | 0.018928 | Down |
| TRBV11-1 | 1.055768698 | 1.062394706 | 0.461117 | 2.30396 | 0.021225 | 0.043513 | Up |
| TRBV12-1 | 0.631115047 | 1.998158584 | 0.756632 | 2.640859 | 0.00827 | 0.019288 | Up |
| TRBV12-2 | 0.516168551 | 2.331156453 | 0.893104 | 2.610172 | 0.00905 | 0.020878 | Up |
| TRBV12-5 | 2.946164823 | 1.241380093 | 0.35589 | 3.488101 | 0.000486 | 0.001552 | Up |
| TRBV17 | 0.610410604 | 1.982465267 | 0.694373 | 2.855043 | 0.004303 | 0.010856 | Up |
| TRBV22-1 | 0.637896027 | 1.955996159 | 0.751912 | 2.601363 | 0.009285 | 0.021339 | Up |
| TRBV26 | 0.745326516 | 1.980439958 | 0.655935 | 3.019264 | 0.002534 | 0.006785 | Up |
| TRBV5-4 | 13.87091372 | 1.061329447 | 0.270344 | 3.925847 | 8.64E-05 | 0.000322 | Up |
| TRBV5-7 | 0.896816346 | 1.977625509 | 0.626006 | 3.159117 | 0.001582 | 0.004483 | Up |
| TRBV6-7 | 0.87245941 | 2.088869785 | 0.507132 | 4.118985 | 3.81E-05 | 0.000152 | Up |
| TRBV6-8 | 0.591106584 | 1.731608058 | 0.621963 | 2.784103 | 0.005368 | 0.0132 | Up |
| TRBV7-4 | 2.147003939 | 2.543953468 | 0.438931 | 5.795796 | 6.80E-09 | 4.63E-08 | Up |
| TRBV7-5 | 0.720653633 | 1.772360988 | 0.619425 | 2.861302 | 0.004219 | 0.01067 | Up |
| TRBV7-7 | 1.298065156 | 1.053522917 | 0.463459 | 2.273175 | 0.023016 | 0.046639 | Up |
| TRBVA | 0.626699019 | 1.631405748 | 0.596991 | 2.732713 | 0.006282 | 0.015155 | Up |
| TRDC | 316.365505 | 3.926874975 | 0.289054 | 13.58526 | 4.90E-42 | 3.88E-40 | Up |
| TRDN | 6.487181391 | -3.032205264 | 0.419959 | -7.22025 | 5.19E-13 | 5.37E-12 | Down |
| TREM1 | 86.48080739 | 2.247444021 | 0.21687 | 10.36312 | 3.65E-25 | 9.89E-24 | Up |
| TREM2 | 145.7790666 | 2.100062794 | 0.205844 | 10.20222 | 1.94E-24 | 4.95E-23 | Up |
| TRGV9 | 6.074530561 | -1.636749083 | 0.39798 | -4.11264 | 3.91E-05 | 0.000156 | Down |
| TRHDE-AS1 | 22.5727255 | -2.683109168 | 0.354238 | -7.57431 | 3.61E-14 | 4.19E-13 | Down |
| TRHR | 17.98835096 | 1.508351816 | 0.328844 | 4.586828 | 4.50E-06 | 2.10E-05 | Up |
| TRIM29 | 87.12257243 | 1.225604075 | 0.221213 | 5.540375 | 3.02E-08 | 1.90E-07 | Up |
| TRIM40 | 1.451194851 | -2.037084776 | 0.905075 | -2.25073 | 0.024402 | 0.049057 | Down |
| TRIM42 | 2.047932666 | -1.569044018 | 0.649307 | -2.41649 | 0.015671 | 0.03352 | Down |
| TRIM47 | 528.2597168 | 1.006111045 | 0.073246 | 13.73596 | 6.18E-43 | 5.21E-41 | Up |
| TRIM50 | 9.365165841 | -2.125632968 | 0.22862 | -9.29769 | 1.44E-20 | 2.79E-19 | Down |
| TRIM51BP | 1.738018685 | -1.561466521 | 0.587057 | -2.65982 | 0.007818 | 0.018351 | Down |
| TRIM51CP | 1.596075004 | -2.195738669 | 0.825251 | -2.66069 | 0.007798 | 0.018312 | Down |
| TRIM51FP | 1.602242642 | -2.189847149 | 0.770532 | -2.84199 | 0.004483 | 0.011258 | Down |
| TRIM51HP | 1.745771045 | -1.501200025 | 0.633172 | -2.37092 | 0.017744 | 0.037295 | Down |
| TRIM54 | 21.25191497 | 1.621338007 | 0.240487 | 6.741897 | 1.56E-11 | 1.40E-10 | Up |
| TRIM58 | 308.9052156 | -1.725185633 | 0.131193 | -13.15 | 1.70E-39 | 1.16E-37 | Down |
| TRIM63 | 15.73587177 | 2.116520134 | 0.349938 | 6.048264 | 1.46E-09 | 1.08E-08 | Up |
| TRIM64FP | 1.182367207 | -1.785750262 | 0.562024 | -3.17736 | 0.001486 | 0.004237 | Down |
| TRIM72 | 9.272853549 | -1.689786546 | 0.308676 | -5.47431 | 4.39E-08 | 2.71E-07 | Down |
| TRIM75P | 1.924693243 | -1.584942377 | 0.391048 | -4.05306 | 5.06E-05 | 0.000197 | Down |
| TRPC4 | 29.87423184 | 1.084301514 | 0.157652 | 6.877826 | 6.08E-12 | 5.70E-11 | Up |
| TRPC5 | 1078.836455 | 4.917475308 | 0.290269 | 16.94112 | 2.24E-64 | 5.22E-62 | Up |
| TRPC5OS | 10.19731281 | 1.723868792 | 0.413222 | 4.171776 | 3.02E-05 | 0.000123 | Up |
| TRPC6P | 1.483833157 | -2.296620728 | 0.722978 | -3.17661 | 0.00149 | 0.004247 | Down |
| TRPM3 | 157.9811109 | -2.339678018 | 0.200965 | -11.6422 | 2.51E-31 | 1.06E-29 | Down |
| TRPM5 | 14.56387854 | -1.578487262 | 0.195502 | -8.07403 | 6.80E-16 | 9.12E-15 | Down |
| TRPV5 | 3.146151553 | -1.958291835 | 0.419826 | -4.66453 | 3.09E-06 | 1.49E-05 | Down |
| TRPV6 | 60.55738864 | -1.501796066 | 0.184718 | -8.13023 | 4.28E-16 | 5.84E-15 | Down |
| TSC22D1 | 10532.6251 | 2.116009945 | 0.134947 | 15.6803 | 2.06E-55 | 3.39E-53 | Up |
| TSGA10IP | 15.50945753 | -2.234159826 | 0.213784 | -10.4506 | 1.46E-25 | 4.06E-24 | Down |
| TSGA13 | 1.361974146 | -2.024217794 | 0.533918 | -3.79125 | 0.00015 | 0.000534 | Down |
| TSIX | 162.9355839 | -1.708208559 | 0.341005 | -5.00934 | 5.46E-07 | 2.92E-06 | Down |
| TSKS | 9.779875383 | -1.10973439 | 0.19446 | -5.70675 | 1.15E-08 | 7.66E-08 | Down |
| TSNAXIP1 | 22.48698479 | -1.511716521 | 0.133859 | -11.2934 | 1.41E-29 | 5.27E-28 | Down |
| TSPAN19 | 7.301935209 | -2.62708699 | 0.259892 | -10.1084 | 5.07E-24 | 1.27E-22 | Down |
| TSPAN5 | 648.9235936 | -1.0357512 | 0.085621 | -12.0969 | 1.10E-33 | 5.23E-32 | Down |
| TSPAN8 | 39.27133236 | -1.460158117 | 0.198102 | -7.37075 | 1.70E-13 | 1.84E-12 | Down |
| TSPEAR | 41.60975518 | -1.462121407 | 0.1681 | -8.69793 | 3.38E-18 | 5.45E-17 | Down |
| TSPEAR-AS1 | 9.65892789 | -1.541437227 | 0.204162 | -7.55008 | 4.35E-14 | 5.01E-13 | Down |
| TSPEAR-AS2 | 18.28375846 | -1.804917685 | 0.211821 | -8.52094 | 1.58E-17 | 2.43E-16 | Down |
| TSSC1-IT1 | 13.07761634 | -1.190373561 | 0.144406 | -8.24324 | 1.68E-16 | 2.36E-15 | Down |
| TSSK1A | 2.491631699 | -1.32064606 | 0.24984 | -5.28597 | 1.25E-07 | 7.29E-07 | Down |
| TTC16 | 20.92867219 | -1.189783708 | 0.190468 | -6.24664 | 4.19E-10 | 3.28E-09 | Down |
| TTC21B-AS1 | 10.1105467 | -1.107122732 | 0.246637 | -4.48887 | 7.16E-06 | 3.24E-05 | Down |
| TTC24 | 13.79566658 | -1.11187194 | 0.404064 | -2.75172 | 0.005928 | 0.014398 | Down |
| TTC29 | 5.785713746 | -1.729262547 | 0.255836 | -6.75926 | 1.39E-11 | 1.25E-10 | Down |
| TTC30A | 538.8929346 | -1.077259833 | 0.068493 | -15.728 | 9.72E-56 | 1.63E-53 | Down |
| TTC34 | 60.16849226 | -1.050769294 | 0.154091 | -6.81914 | 9.16E-12 | 8.42E-11 | Down |
| TTC39A | 338.9958401 | 1.107870183 | 0.112943 | 9.809128 | 1.03E-22 | 2.34E-21 | Up |
| TTC39B | 1290.656156 | 1.497114401 | 0.092045 | 16.26504 | 1.75E-59 | 3.47E-57 | Up |
| TTC39DP | 1.322528783 | -2.391193958 | 0.66259 | -3.60886 | 0.000308 | 0.001027 | Down |
| TTLL13P | 8.032959873 | -1.019412593 | 0.229059 | -4.45045 | 8.57E-06 | 3.83E-05 | Down |
| TTLL6 | 5.4116004 | -2.346921768 | 0.28824 | -8.14226 | 3.88E-16 | 5.31E-15 | Down |
| TTLL7-IT1 | 7.501601234 | -1.681842268 | 0.275279 | -6.10959 | 9.99E-10 | 7.50E-09 | Down |
| TTLL9 | 25.64808452 | -1.014497926 | 0.164982 | -6.14914 | 7.79E-10 | 5.93E-09 | Down |
| TTN | 4032.731022 | -1.261258085 | 0.184434 | -6.83854 | 8.00E-12 | 7.40E-11 | Down |
| TUBA3C | 1.735747616 | -1.977081896 | 0.707642 | -2.7939 | 0.005208 | 0.012853 | Down |
| TUBA3E | 3.399445098 | -1.364848891 | 0.353282 | -3.86334 | 0.000112 | 0.000408 | Down |
| TUBA3FP | 11.99899117 | -1.01150211 | 0.208954 | -4.84078 | 1.29E-06 | 6.56E-06 | Down |
| TUBA3GP | 0.975472634 | -1.997655446 | 0.761079 | -2.62477 | 0.008671 | 0.020114 | Down |
| TUBA4B | 5.785992113 | -1.567672731 | 0.28298 | -5.53987 | 3.03E-08 | 1.91E-07 | Down |
| TUBA8 | 74.48521387 | -1.31788527 | 0.135468 | -9.72839 | 2.28E-22 | 5.05E-21 | Down |
| TUBB1 | 26.49702034 | -1.040442965 | 0.144327 | -7.20893 | 5.64E-13 | 5.82E-12 | Down |
| TUBB2BP1 | 1.030518901 | -2.228214693 | 0.567701 | -3.92498 | 8.67E-05 | 0.000323 | Down |
| TUBB3 | 293.8062609 | 2.91359458 | 0.206302 | 14.12299 | 2.74E-45 | 2.68E-43 | Up |
| TUBB3P1 | 1.453865126 | -1.311828767 | 0.481233 | -2.72598 | 0.006411 | 0.015434 | Down |
| TUBB4A | 8.61212216 | -1.086561316 | 0.304614 | -3.56701 | 0.000361 | 0.001188 | Down |
| TUBB4BP7 | 1.225614575 | -2.873210917 | 0.963374 | -2.98245 | 0.00286 | 0.007552 | Down |
| TUBB8 | 11.75275465 | -1.542686523 | 0.260888 | -5.9132 | 3.36E-09 | 2.37E-08 | Down |
| TUBB8P10 | 1.43042844 | -2.333393154 | 0.967423 | -2.41197 | 0.015867 | 0.033882 | Down |
| TUBB8P11 | 1.691256712 | -1.213747701 | 0.529948 | -2.29032 | 0.022003 | 0.044882 | Down |
| TUBBP6 | 1.716868506 | -1.770684107 | 0.636462 | -2.78207 | 0.005401 | 0.013271 | Down |
| TUBBP8 | 1.407556504 | -2.184443216 | 0.902135 | -2.42141 | 0.01546 | 0.033147 | Down |
| TUBBP9 | 1.461691528 | -2.705123446 | 0.844212 | -3.20432 | 0.001354 | 0.003896 | Down |
| TUFMP1 | 1.668329806 | -1.918400904 | 0.816199 | -2.35041 | 0.018753 | 0.039089 | Down |
| TULP2 | 2.120914001 | -2.192375205 | 0.420286 | -5.21638 | 1.82E-07 | 1.04E-06 | Down |
| TUSC3 | 985.6389237 | 2.357727996 | 0.126899 | 18.57954 | 4.71E-77 | 1.84E-74 | Up |
| TUSC5 | 49.19452181 | -2.537427106 | 0.359909 | -7.0502 | 1.79E-12 | 1.77E-11 | Down |
| TUSC8 | 1.182952676 | -1.829143594 | 0.449612 | -4.06828 | 4.74E-05 | 0.000186 | Down |
| TYMSOS | 5.000817745 | 1.204987848 | 0.216015 | 5.57826 | 2.43E-08 | 1.55E-07 | Up |
| TYRO3 | 275.8234072 | 1.33229905 | 0.119444 | 11.15421 | 6.83E-29 | 2.44E-27 | Up |
| U73166.2 | 45.13895011 | -1.127336009 | 0.163769 | -6.88368 | 5.83E-12 | 5.48E-11 | Down |
| UBE2Q2L | 2.086887785 | -1.112665641 | 0.254689 | -4.36872 | 1.25E-05 | 5.43E-05 | Down |
| UBE2QL1 | 230.3910393 | 2.384502913 | 0.159334 | 14.96546 | 1.23E-50 | 1.55E-48 | Up |
| UBE2U | 1.188049813 | -2.37678886 | 0.658272 | -3.61065 | 0.000305 | 0.001021 | Down |
| UBL4B | 1.399227932 | -1.18793431 | 0.469605 | -2.52964 | 0.011418 | 0.025542 | Down |
| UBQLN4P1 | 2.304738041 | -1.177795871 | 0.441872 | -2.66547 | 0.007688 | 0.018095 | Down |
| UBTFL11 | 1.574448078 | -3.240953937 | 1.224042 | -2.64775 | 0.008103 | 0.01894 | Down |
| UBTFL2 | 1.475140558 | -2.500973563 | 0.749956 | -3.33483 | 0.000854 | 0.002573 | Down |
| UBTFL5 | 6.118565719 | -1.904913302 | 0.354583 | -5.37226 | 7.78E-08 | 4.65E-07 | Down |
| UBTFL7 | 1.310957581 | -2.445567727 | 0.833792 | -2.93307 | 0.003356 | 0.008713 | Down |
| UBXN10-AS1 | 11.58586161 | 1.293598611 | 0.144149 | 8.974013 | 2.86E-19 | 5.04E-18 | Up |
| UCN | 12.0923344 | 1.392400451 | 0.170498 | 8.166663 | 3.17E-16 | 4.36E-15 | Up |
| UCN3 | 1.635104512 | -2.207584071 | 0.857072 | -2.57573 | 0.010003 | 0.022761 | Down |
| UG0898H09 | 9.170072321 | -1.454921832 | 0.32324 | -4.50106 | 6.76E-06 | 3.07E-05 | Down |
| UGT1A10 | 2.143316717 | -1.286082883 | 0.497061 | -2.58738 | 0.009671 | 0.022109 | Down |
| UGT1A12P | 1.056167051 | -1.900166601 | 0.760074 | -2.49998 | 0.01242 | 0.027504 | Down |
| UGT1A2P | 1.383350189 | -2.060587396 | 0.706075 | -2.91837 | 0.003519 | 0.009086 | Down |
| UGT1A9 | 2.38293441 | -1.182487 | 0.493295 | -2.39712 | 0.016524 | 0.035098 | Down |
| UGT2A3 | 1.043556215 | -2.596239235 | 1.105904 | -2.34762 | 0.018894 | 0.039352 | Down |
| UGT2B11 | 11.59511507 | -2.141458781 | 0.240623 | -8.89963 | 5.60E-19 | 9.64E-18 | Down |
| UGT8 | 217.2334353 | -1.390259624 | 0.121144 | -11.4761 | 1.74E-30 | 6.87E-29 | Down |
| ULBP1 | 35.2474023 | 2.188604246 | 0.262175 | 8.347861 | 6.95E-17 | 1.01E-15 | Up |
| ULBP2 | 61.48664908 | 2.862787142 | 0.163971 | 17.45912 | 2.93E-68 | 7.87E-66 | Up |
| ULK4P3 | 13.5110888 | -1.042491552 | 0.242675 | -4.29584 | 1.74E-05 | 7.39E-05 | Down |
| UMOD | 1.011776674 | -2.263659921 | 0.957028 | -2.3653 | 0.018015 | 0.037794 | Down |
| UMODL1 | 2.789546472 | -1.210811454 | 0.364708 | -3.31995 | 0.0009 | 0.002703 | Down |
| UMODL1-AS1 | 1.840706021 | -1.488151449 | 0.496357 | -2.99814 | 0.002716 | 0.007209 | Down |
| UNC13C | 879.1340637 | -1.285823039 | 0.153111 | -8.39799 | 4.54E-17 | 6.72E-16 | Down |
| UNC45B | 9.923717081 | -1.407097397 | 0.252382 | -5.57526 | 2.47E-08 | 1.58E-07 | Down |
| UNC5A | 16.20184929 | -1.339276257 | 0.207747 | -6.44667 | 1.14E-10 | 9.48E-10 | Down |
| UNC5B | 1148.940111 | 1.876084276 | 0.085324 | 21.98782 | 3.77E-107 | 4.29E-104 | Up |
| UNC5B-AS1 | 10.38789093 | 2.984647598 | 0.230725 | 12.93593 | 2.82E-38 | 1.76E-36 | Up |
| UNC5CL | 103.5202484 | 2.317349199 | 0.158684 | 14.60357 | 2.67E-48 | 3.07E-46 | Up |
| UNC5D | 36.83536139 | -1.34481124 | 0.248389 | -5.41414 | 6.16E-08 | 3.73E-07 | Down |
| UNC80 | 86.63649748 | -1.701134408 | 0.185307 | -9.18008 | 4.31E-20 | 8.07E-19 | Down |
| UNC93A | 1.55869095 | -2.40456652 | 0.836866 | -2.8733 | 0.004062 | 0.01031 | Down |
| UNC93B3 | 1.156129263 | -1.222974973 | 0.449798 | -2.71894 | 0.006549 | 0.015728 | Down |
| UNC93B7 | 3.606681913 | -1.74914743 | 0.257112 | -6.80305 | 1.02E-11 | 9.38E-11 | Down |
| UNGP3 | 6.94294265 | -1.526186478 | 0.180423 | -8.45892 | 2.70E-17 | 4.06E-16 | Down |
| UOX | 0.76433196 | -1.248311735 | 0.370943 | -3.36524 | 0.000765 | 0.00233 | Down |
| UPF3AP2 | 2.389437203 | -1.395606593 | 0.423376 | -3.29637 | 0.000979 | 0.002915 | Down |
| UPK1A | 1.645695481 | -2.231385102 | 0.784413 | -2.84466 | 0.004446 | 0.011175 | Down |
| UPK1B | 2.723375381 | -1.103382307 | 0.322686 | -3.41937 | 0.000628 | 0.001951 | Down |
| UPK3A | 43.02159639 | -1.264840282 | 0.252833 | -5.00266 | 5.65E-07 | 3.02E-06 | Down |
| UPK3BP1 | 1.719502381 | -1.103679658 | 0.397642 | -2.77556 | 0.005511 | 0.013504 | Down |
| UPP1 | 427.7044734 | 2.105692099 | 0.101813 | 20.68203 | 5.03E-95 | 3.75E-92 | Up |
| UPP2-IT1 | 1.038417936 | 1.103163458 | 0.45443 | 2.427574 | 0.0152 | 0.03269 | Up |
| URAD | 1.32958406 | -2.785586463 | 1.055086 | -2.64015 | 0.008287 | 0.019323 | Down |
| UROC1 | 1.702960041 | -1.366971663 | 0.584562 | -2.33846 | 0.019364 | 0.040195 | Down |
| USH1C | 18.31821483 | -1.90832086 | 0.313659 | -6.08406 | 1.17E-09 | 8.74E-09 | Down |
| USP17L11 | 1.977293888 | -2.964147477 | 1.145096 | -2.58856 | 0.009638 | 0.022044 | Down |
| USP17L14P | 1.749786518 | -1.897281875 | 0.553865 | -3.42553 | 0.000614 | 0.001913 | Down |
| USP17L15 | 9.975530871 | -1.464104385 | 0.384609 | -3.80674 | 0.000141 | 0.000504 | Down |
| USP17L22 | 1.55512974 | -1.848312008 | 0.689682 | -2.67995 | 0.007363 | 0.017414 | Down |
| USP2 | 76.23135025 | -1.074975382 | 0.147927 | -7.26692 | 3.68E-13 | 3.86E-12 | Down |
| USP2-AS1 | 10.67162935 | -1.192660527 | 0.236203 | -5.0493 | 4.43E-07 | 2.40E-06 | Down |
| USP29 | 2.715486815 | -1.79468551 | 0.777768 | -2.30748 | 0.021028 | 0.043192 | Down |
| USP49 | 381.8987922 | -1.251215619 | 0.095967 | -13.0379 | 7.44E-39 | 4.83E-37 | Down |
| USP50 | 5.74886115 | -1.134030262 | 0.197546 | -5.7406 | 9.43E-09 | 6.34E-08 | Down |
| UTS2B | 6.912569378 | -1.285948792 | 0.245095 | -5.24673 | 1.55E-07 | 8.92E-07 | Down |
| UTS2R | 12.06023168 | -1.120497056 | 0.274383 | -4.08369 | 4.43E-05 | 0.000175 | Down |
| VASN | 1287.333819 | 1.635001329 | 0.090736 | 18.01923 | 1.38E-72 | 4.45E-70 | Up |
| VCAN | 4302.977395 | 2.221361753 | 0.175714 | 12.64189 | 1.24E-36 | 7.12E-35 | Up |
| VCAN-AS1 | 1.37313956 | 2.123156083 | 0.425183 | 4.993513 | 5.93E-07 | 3.16E-06 | Up |
| VCX | 1.391690764 | -2.427111231 | 0.702599 | -3.45448 | 0.000551 | 0.001737 | Down |
| VCX3A | 2.271444183 | -1.741747309 | 0.522801 | -3.33157 | 0.000864 | 0.002602 | Down |
| VCX3B | 1.830585049 | -2.038474869 | 0.489264 | -4.16641 | 3.09E-05 | 0.000126 | Down |
| VDAC1P3 | 1.145305252 | -2.40433033 | 0.474655 | -5.06543 | 4.07E-07 | 2.22E-06 | Down |
| VDAC1P5 | 0.94181261 | -1.976336665 | 0.84428 | -2.34086 | 0.01924 | 0.039971 | Down |
| VDAC2P1 | 1.290873783 | -1.425073368 | 0.60155 | -2.369 | 0.017836 | 0.037464 | Down |
| VDAC3P1 | 1.60331307 | -3.21617854 | 0.877345 | -3.66581 | 0.000247 | 0.000841 | Down |
| VDR | 329.1363624 | 1.331281187 | 0.129301 | 10.29602 | 7.34E-25 | 1.94E-23 | Up |
| VENTXP7 | 0.837733863 | -2.358416512 | 0.713653 | -3.30471 | 0.000951 | 0.002838 | Down |
| VEPH1 | 57.75294116 | -1.395705256 | 0.136681 | -10.2114 | 1.76E-24 | 4.54E-23 | Down |
| VEZF1P1 | 2.528389336 | -1.799172238 | 0.546169 | -3.29417 | 0.000987 | 0.002936 | Down |
| VGLL1 | 9.201928014 | 2.848795855 | 0.39412 | 7.228243 | 4.89E-13 | 5.08E-12 | Up |
| VGLL2 | 2.986647982 | -2.942565911 | 0.875518 | -3.36094 | 0.000777 | 0.002363 | Down |
| VHLL | 1.138037961 | -1.417844916 | 0.441433 | -3.21192 | 0.001319 | 0.003807 | Down |
| VIPR1 | 186.5634229 | -1.904687963 | 0.131356 | -14.5002 | 1.21E-47 | 1.34E-45 | Down |
| VIPR1-AS1 | 13.77325929 | -2.122108159 | 0.291855 | -7.27109 | 3.57E-13 | 3.75E-12 | Down |
| VIT | 137.2386415 | -2.403014559 | 0.222479 | -10.8011 | 3.40E-27 | 1.07E-25 | Down |
| VLDLR | 965.6609892 | -1.521436874 | 0.109991 | -13.8324 | 1.63E-43 | 1.41E-41 | Down |
| VLDLR-AS1 | 49.15450672 | -2.239956671 | 0.16171 | -13.8517 | 1.24E-43 | 1.09E-41 | Down |
| VN1R2 | 1.672832156 | -1.736130954 | 0.578868 | -2.99918 | 0.002707 | 0.007186 | Down |
| VN1R20P | 1.656086388 | -1.506798087 | 0.450649 | -3.34362 | 0.000827 | 0.0025 | Down |
| VN1R3 | 1.323370914 | -2.785443614 | 0.970287 | -2.87074 | 0.004095 | 0.010384 | Down |
| VN1R34P | 1.503371786 | -1.86165505 | 0.532408 | -3.49667 | 0.000471 | 0.001508 | Down |
| VN1R48P | 3.750991046 | -3.020263047 | 0.298677 | -10.1121 | 4.88E-24 | 1.23E-22 | Down |
| VN1R5 | 1.79879076 | -1.111069602 | 0.46064 | -2.41201 | 0.015865 | 0.03388 | Down |
| VN1R51P | 1.614669985 | -1.648644739 | 0.42094 | -3.91658 | 8.98E-05 | 0.000333 | Down |
| VN1R81P | 15.58926263 | 1.074935715 | 0.156697 | 6.859947 | 6.89E-12 | 6.42E-11 | Up |
| VN2R17P | 2.877817412 | -1.122534719 | 0.279296 | -4.01916 | 5.84E-05 | 0.000225 | Down |
| VN2R1P | 1.346268971 | -2.845077337 | 1.054133 | -2.69897 | 0.006955 | 0.016574 | Down |
| VNN3 | 6.29081622 | -1.641396302 | 0.277583 | -5.91316 | 3.36E-09 | 2.37E-08 | Down |
| VPREB3 | 57.08830887 | -1.203648474 | 0.429658 | -2.80141 | 0.005088 | 0.012584 | Down |
| VRTN | 5.616228226 | -2.200331581 | 0.500254 | -4.39843 | 1.09E-05 | 4.79E-05 | Down |
| VSIG8 | 6.680567561 | -1.087139285 | 0.219021 | -4.96362 | 6.92E-07 | 3.65E-06 | Down |
| VSTM2A | 8.531199642 | -2.50814078 | 0.378474 | -6.62699 | 3.43E-11 | 2.97E-10 | Down |
| VSTM2A-OT1 | 1.358285045 | -2.932937016 | 0.849242 | -3.45359 | 0.000553 | 0.001742 | Down |
| VSTM2L | 77.43545356 | 1.799566552 | 0.160713 | 11.19738 | 4.20E-29 | 1.51E-27 | Up |
| VTCN1 | 31.40671576 | 3.339647094 | 0.427391 | 7.814041 | 5.54E-15 | 6.97E-14 | Up |
| VTN | 18.81424264 | -1.306685738 | 0.167583 | -7.79726 | 6.33E-15 | 7.92E-14 | Down |
| VTRNA1-1 | 32.6951758 | 1.101881609 | 0.268323 | 4.106543 | 4.02E-05 | 0.00016 | Up |
| VTRNA1-2 | 1.941571409 | 3.012035947 | 0.471352 | 6.390211 | 1.66E-10 | 1.35E-09 | Up |
| VWA3A | 10.22137248 | -2.458752547 | 0.280728 | -8.7585 | 1.98E-18 | 3.25E-17 | Down |
| VWA8-AS1 | 4.400764154 | -1.476032127 | 0.247935 | -5.95331 | 2.63E-09 | 1.88E-08 | Down |
| WARS | 4909.780844 | 1.133592952 | 0.166097 | 6.824905 | 8.80E-12 | 8.11E-11 | Up |
| WASF3 | 779.6637346 | -1.331619227 | 0.080806 | -16.4793 | 5.17E-61 | 1.08E-58 | Down |
| WASF3-AS1 | 5.97895248 | -1.664330968 | 0.321996 | -5.1688 | 2.36E-07 | 1.33E-06 | Down |
| WASF4P | 3.671679267 | -1.499916835 | 0.367917 | -4.07678 | 4.57E-05 | 0.00018 | Down |
| WBSCR17 | 319.2991763 | -1.24611807 | 0.153483 | -8.11893 | 4.70E-16 | 6.40E-15 | Down |
| WDR11-AS1 | 11.17741388 | -1.333417003 | 0.211839 | -6.29448 | 3.08E-10 | 2.45E-09 | Down |
| WDR45BP1 | 1.864125199 | -1.230668241 | 0.532062 | -2.31302 | 0.020722 | 0.04265 | Down |
| WDR49 | 24.45611299 | -1.059590022 | 0.168359 | -6.29364 | 3.10E-10 | 2.46E-09 | Down |
| WDR7-OT1 | 0.700777606 | -1.595752585 | 0.608325 | -2.62319 | 0.008711 | 0.020196 | Down |
| WDR72 | 2839.790651 | -1.555521269 | 0.114095 | -13.6336 | 2.53E-42 | 2.04E-40 | Down |
| WDR86-AS1 | 171.642445 | 1.2496891 | 0.119684 | 10.44161 | 1.60E-25 | 4.45E-24 | Up |
| WDR93 | 45.24486312 | -1.175322107 | 0.112437 | -10.4532 | 1.42E-25 | 3.95E-24 | Down |
| WEE2 | 4.387707773 | -1.360516209 | 0.260408 | -5.22455 | 1.75E-07 | 1.00E-06 | Down |
| WFDC21P | 31.36904911 | 1.264041294 | 0.197397 | 6.403561 | 1.52E-10 | 1.24E-09 | Up |
| WFDC5 | 1.240740349 | -1.993092897 | 0.564826 | -3.52868 | 0.000418 | 0.001353 | Down |
| WFDC6 | 1.436196586 | -2.677880225 | 0.629519 | -4.25385 | 2.10E-05 | 8.79E-05 | Down |
| WFDC8 | 1.915289408 | -2.091904978 | 0.431686 | -4.84589 | 1.26E-06 | 6.40E-06 | Down |
| WFIKKN1 | 35.16507559 | -1.322703617 | 0.179793 | -7.35681 | 1.88E-13 | 2.04E-12 | Down |
| WFS1 | 1474.584862 | -1.236323462 | 0.078582 | -15.7329 | 9.00E-56 | 1.52E-53 | Down |
| WI2-2994D6.1 | 1.944923184 | -2.262763267 | 0.778441 | -2.90679 | 0.003652 | 0.009386 | Down |
| WI2-8325B5.1 | 0.934875746 | 1.923785169 | 0.498072 | 3.862467 | 0.000112 | 0.000409 | Up |
| WI2-85898F10.2 | 6.355267235 | -1.423485543 | 0.275447 | -5.16792 | 2.37E-07 | 1.33E-06 | Down |
| WIF1 | 6.609366703 | 1.492687069 | 0.481911 | 3.097435 | 0.001952 | 0.005398 | Up |
| WISP1 | 109.0953366 | 1.444336157 | 0.173658 | 8.317146 | 9.01E-17 | 1.30E-15 | Up |
| WNK2 | 93.2362818 | -1.521591636 | 0.175373 | -8.67632 | 4.09E-18 | 6.56E-17 | Down |
| WNK4 | 11.69128009 | -1.829919366 | 0.315258 | -5.80451 | 6.46E-09 | 4.40E-08 | Down |
| WNT1 | 2.506547074 | -1.112409479 | 0.411431 | -2.70375 | 0.006856 | 0.016372 | Down |
| WNT10A | 122.8730446 | 2.069599943 | 0.224833 | 9.205054 | 3.41E-20 | 6.47E-19 | Up |
| WNT11 | 104.1893347 | -1.881938789 | 0.17663 | -10.6547 | 1.66E-26 | 4.93E-25 | Down |
| WNT3 | 117.0774168 | 1.124040587 | 0.129217 | 8.698844 | 3.35E-18 | 5.41E-17 | Up |
| WNT3A | 2.821154938 | -1.582974999 | 0.470724 | -3.36285 | 0.000771 | 0.002348 | Down |
| WNT4 | 69.49194636 | -1.59208017 | 0.155081 | -10.2661 | 1.00E-24 | 2.63E-23 | Down |
| WNT8B | 5.450670375 | -1.099157849 | 0.302225 | -3.63689 | 0.000276 | 0.000932 | Down |
| WSCD2 | 1218.588646 | -2.55857519 | 0.22169 | -11.5412 | 8.17E-31 | 3.31E-29 | Down |
| WWC2-AS2 | 35.48503972 | -1.318720163 | 0.109627 | -12.0292 | 2.50E-33 | 1.17E-31 | Down |
| WWOX | 538.9553794 | -1.042722181 | 0.067121 | -15.5349 | 2.01E-54 | 3.09E-52 | Down |
| WWTR1-AS1 | 26.70603325 | -1.28602664 | 0.116018 | -11.0847 | 1.49E-28 | 5.20E-27 | Down |
| XAGE1A | 1.248021755 | -2.077577142 | 0.664218 | -3.12785 | 0.001761 | 0.004931 | Down |
| XAGE1B | 1.473853944 | -1.513927444 | 0.532264 | -2.84432 | 0.004451 | 0.011184 | Down |
| XDH | 34.99972483 | 1.256731696 | 0.286136 | 4.392072 | 1.12E-05 | 4.92E-05 | Up |
| XIRP2 | 42.02283798 | -3.864605883 | 0.519757 | -7.43541 | 1.04E-13 | 1.16E-12 | Down |
| XKR3 | 2.066426533 | -1.660630435 | 0.712797 | -2.32974 | 0.01982 | 0.041024 | Down |
| XKR5 | 7.125804581 | 1.932077158 | 0.227373 | 8.497375 | 1.94E-17 | 2.96E-16 | Up |
| XKR9 | 23.61462464 | -1.443675139 | 0.185734 | -7.77281 | 7.68E-15 | 9.53E-14 | Down |
| XKRX | 259.470267 | 3.454834939 | 0.201719 | 17.127 | 9.33E-66 | 2.28E-63 | Up |
| XPOTP1 | 3.756861921 | -1.304677962 | 0.347628 | -3.75308 | 0.000175 | 0.000615 | Down |
| XPR1 | 2756.852721 | 1.70422227 | 0.07393 | 23.05178 | 1.41E-117 | 2.23E-114 | Up |
| XRCC6P1 | 1.277637772 | -2.213612009 | 0.767953 | -2.88248 | 0.003946 | 0.010049 | Down |
| XRCC6P3 | 1.439423093 | -3.152659311 | 1.133039 | -2.78248 | 0.005394 | 0.013258 | Down |
| XRCC6P4 | 1.021869401 | -2.418039335 | 0.94913 | -2.54764 | 0.010845 | 0.024415 | Down |
| XRCC6P5 | 1.240071677 | -2.906651187 | 0.908963 | -3.19776 | 0.001385 | 0.003977 | Down |
| XX-FW83563B9.5 | 54.13023969 | -1.031553179 | 0.089285 | -11.5535 | 7.09E-31 | 2.88E-29 | Down |
| XXbac-B33L19.12 | 8.839321474 | 4.981205227 | 0.341278 | 14.59573 | 2.99E-48 | 3.42E-46 | Up |
| XXbac-B444P24.8 | 3.408073005 | -1.36680054 | 0.336874 | -4.05731 | 4.96E-05 | 0.000194 | Down |
| XXbac-B476C20.13 | 3.965938622 | -1.225754617 | 0.338735 | -3.61862 | 0.000296 | 0.000993 | Down |
| XXbac-BPG116M5.17 | 24.79816664 | 1.334770138 | 0.576493 | 2.315328 | 0.020595 | 0.042417 | Up |
| XXbac-BPG154L12.4 | 13.28544233 | -1.418018552 | 0.275387 | -5.14919 | 2.62E-07 | 1.46E-06 | Down |
| XXbac-BPG154L12.5 | 1.635056898 | -1.214003117 | 0.424903 | -2.85713 | 0.004275 | 0.010792 | Down |
| XXbac-BPG32J3.19 | 1.765716357 | -1.522330055 | 0.43867 | -3.47033 | 0.00052 | 0.001647 | Down |
| XXbac-BPGBPG55C20.1 | 5.051689951 | -1.097817551 | 0.255409 | -4.29828 | 1.72E-05 | 7.31E-05 | Down |
| XXyac-YM21GA2.1 | 1.207847684 | -2.500694158 | 0.932748 | -2.681 | 0.00734 | 0.017364 | Down |
| YBX1P4 | 5.221189079 | -1.10071857 | 0.274392 | -4.01148 | 6.03E-05 | 0.000231 | Down |
| YBX1P5 | 1.526408322 | -3.394024024 | 0.910817 | -3.72635 | 0.000194 | 0.000677 | Down |
| YBX1P6 | 2.473030247 | -2.074735521 | 0.574261 | -3.61288 | 0.000303 | 0.001013 | Down |
| yR211F11.2 | 0.877432963 | -1.012069697 | 0.444014 | -2.27937 | 0.022645 | 0.046002 | Down |
| YTHDF1P1 | 2.83886473 | -1.496611016 | 0.433143 | -3.45524 | 0.00055 | 0.001733 | Down |
| YWHABP1 | 0.47735914 | -1.297277168 | 0.576309 | -2.25101 | 0.024385 | 0.049024 | Down |
| YWHAEP1 | 1.768844092 | -1.488255965 | 0.490699 | -3.03293 | 0.002422 | 0.006525 | Down |
| YWHAEP5 | 2.530079387 | -1.272778737 | 0.375037 | -3.39374 | 0.000689 | 0.002125 | Down |
| YWHAZP6 | 1.547490806 | -1.690112642 | 0.396375 | -4.26392 | 2.01E-05 | 8.43E-05 | Down |
| Z82214.2 | 2.991685194 | 2.244320527 | 0.2914 | 7.701863 | 1.34E-14 | 1.63E-13 | Up |
| Z95114.3 | 1.414741199 | -2.031400115 | 0.861894 | -2.3569 | 0.018428 | 0.03854 | Down |
| Z95704.3 | 1.213367989 | -2.273474619 | 0.818019 | -2.77924 | 0.005449 | 0.01337 | Down |
| ZAN | 5.146170993 | -1.35988212 | 0.382278 | -3.55731 | 0.000375 | 0.001229 | Down |
| ZBED1P1 | 1.268763923 | -2.723087517 | 1.049471 | -2.59472 | 0.009467 | 0.021717 | Down |
| ZBTB16 | 971.9593327 | -1.100828643 | 0.168508 | -6.53281 | 6.45E-11 | 5.47E-10 | Down |
| ZBTB20-AS4 | 7.870891598 | -1.002516194 | 0.170687 | -5.8734 | 4.27E-09 | 2.97E-08 | Down |
| ZC2HC1B | 0.735948552 | -1.054102882 | 0.430965 | -2.44592 | 0.014448 | 0.031301 | Down |
| ZC3H11B | 5.16878414 | -1.200474705 | 0.320754 | -3.74266 | 0.000182 | 0.000639 | Down |
| ZCCHC12 | 3523.365611 | 5.680689778 | 0.312892 | 18.15544 | 1.16E-73 | 3.91E-71 | Up |
| ZCCHC16 | 116.1856313 | 3.991771977 | 0.307216 | 12.99337 | 1.33E-38 | 8.48E-37 | Up |
| ZDHHC11 | 86.65629407 | -1.715813135 | 0.170689 | -10.0523 | 8.98E-24 | 2.21E-22 | Down |
| ZDHHC11B | 97.62931295 | -2.017730654 | 0.154187 | -13.0863 | 3.94E-39 | 2.62E-37 | Down |
| ZDHHC19 | 1.053338701 | -1.271383131 | 0.468358 | -2.71456 | 0.006636 | 0.015907 | Down |
| ZDHHC8P1 | 13.62306657 | -1.153196001 | 0.185783 | -6.20721 | 5.39E-10 | 4.17E-09 | Down |
| ZEB2P1 | 4.225576987 | -2.531181447 | 0.376426 | -6.72425 | 1.77E-11 | 1.57E-10 | Down |
| ZFAND6P1 | 0.668078896 | -1.100833886 | 0.444214 | -2.47816 | 0.013206 | 0.029014 | Down |
| ZFP36L1 | 5507.870348 | 1.29470342 | 0.097132 | 13.32931 | 1.56E-40 | 1.13E-38 | Up |
| ZFP36L2 | 5203.147278 | -1.136913881 | 0.091587 | -12.4135 | 2.21E-35 | 1.17E-33 | Down |
| ZFP69B | 81.40279903 | 1.317648417 | 0.075882 | 17.36454 | 1.53E-67 | 3.95E-65 | Up |
| ZFPM2 | 566.3559803 | -1.406927145 | 0.110444 | -12.7389 | 3.60E-37 | 2.12E-35 | Down |
| ZFPM2-AS1 | 6.041916408 | -1.051184188 | 0.237454 | -4.4269 | 9.56E-06 | 4.24E-05 | Down |
| ZFYVE9P1 | 1.203166005 | -2.498242931 | 0.914758 | -2.73104 | 0.006313 | 0.015229 | Down |
| ZIC2 | 4.300052082 | -2.793696781 | 0.364295 | -7.66879 | 1.74E-14 | 2.08E-13 | Down |
| ZIK1P1 | 1.287793372 | -3.280975173 | 1.444109 | -2.27197 | 0.023088 | 0.046749 | Down |
| ZIM2 | 20.57582678 | -1.411344868 | 0.149295 | -9.45343 | 3.28E-21 | 6.71E-20 | Down |
| ZIM3 | 2.181460515 | -3.051951538 | 0.984407 | -3.1003 | 0.001933 | 0.005353 | Down |
| ZMAT3 | 2264.177531 | 1.871337448 | 0.085541 | 21.87653 | 4.35E-106 | 4.58E-103 | Up |
| ZMAT4 | 365.6205792 | -2.498597803 | 0.242005 | -10.3246 | 5.46E-25 | 1.46E-23 | Down |
| ZNF157 | 32.30635011 | -1.214092349 | 0.116118 | -10.4556 | 1.38E-25 | 3.85E-24 | Down |
| ZNF192P2 | 1.668943816 | -3.153659817 | 0.77004 | -4.09545 | 4.21E-05 | 0.000167 | Down |
| ZNF217 | 1884.117016 | 1.05424374 | 0.070216 | 15.01434 | 5.91E-51 | 7.54E-49 | Up |
| ZNF259P1 | 11.51980591 | -1.021083405 | 0.253241 | -4.03206 | 5.53E-05 | 0.000214 | Down |
| ZNF280A | 2.938660924 | -1.877413254 | 0.426918 | -4.3976 | 1.09E-05 | 4.80E-05 | Down |
| ZNF30-AS1 | 2.996334141 | -1.327566654 | 0.254555 | -5.21524 | 1.84E-07 | 1.05E-06 | Down |
| ZNF33AP1 | 1.874167718 | -2.018292859 | 0.601271 | -3.35671 | 0.000789 | 0.002396 | Down |
| ZNF479 | 1.754536049 | -1.909898357 | 0.668267 | -2.85799 | 0.004263 | 0.010767 | Down |
| ZNF488 | 10.45144437 | -1.848322084 | 0.229092 | -8.06805 | 7.14E-16 | 9.57E-15 | Down |
| ZNF536 | 120.0867187 | -2.462507589 | 0.18889 | -13.0367 | 7.56E-39 | 4.89E-37 | Down |
| ZNF541 | 20.59281333 | -1.368384669 | 0.146797 | -9.32159 | 1.15E-20 | 2.24E-19 | Down |
| ZNF560 | 12.75352461 | 2.321645538 | 0.407599 | 5.695912 | 1.23E-08 | 8.13E-08 | Up |
| ZNF645 | 3.031727329 | -1.933626206 | 0.544538 | -3.55095 | 0.000384 | 0.001255 | Down |
| ZNF705A | 3.047085592 | -2.106668983 | 0.405522 | -5.19496 | 2.05E-07 | 1.16E-06 | Down |
| ZNF705G | 2.357909106 | -1.820252165 | 0.672941 | -2.70492 | 0.006832 | 0.016321 | Down |
| ZNF714 | 979.4532453 | 1.025776026 | 0.098865 | 10.37557 | 3.20E-25 | 8.72E-24 | Up |
| ZNF720P1 | 1.713458829 | -2.16720364 | 0.798751 | -2.71324 | 0.006663 | 0.015963 | Down |
| ZNF729 | 4.292500316 | -1.56660876 | 0.430204 | -3.64154 | 0.000271 | 0.000917 | Down |
| ZNF730 | 38.98931938 | 1.067229531 | 0.174139 | 6.128604 | 8.87E-10 | 6.70E-09 | Up |
| ZNF734P | 1.153432827 | -3.383057657 | 1.502114 | -2.2522 | 0.02431 | 0.048904 | Down |
| ZNF735 | 1.305486193 | -2.148460675 | 0.814459 | -2.6379 | 0.008342 | 0.019437 | Down |
| ZNF781 | 107.9180086 | -1.078397347 | 0.103002 | -10.4696 | 1.19E-25 | 3.34E-24 | Down |
| ZNF804B | 603.0166551 | -2.426189264 | 0.253979 | -9.55273 | 1.26E-21 | 2.65E-20 | Down |
| ZNF806 | 2.623358456 | -2.099007531 | 0.459276 | -4.57026 | 4.87E-06 | 2.26E-05 | Down |
| ZNF840P | 5.821597624 | -1.239701891 | 0.256836 | -4.82681 | 1.39E-06 | 7.00E-06 | Down |
| ZNF90P2 | 1.111429856 | -2.786838898 | 1.195557 | -2.331 | 0.019754 | 0.040912 | Down |
| ZNF969P | 2.341379874 | -2.366876245 | 0.649875 | -3.64205 | 0.00027 | 0.000915 | Down |
| ZNRF2 | 1029.003138 | 1.195942508 | 0.080602 | 14.83769 | 8.36E-50 | 1.02E-47 | Up |
| ZNRF2P3 | 2.230758821 | -1.25231465 | 0.302865 | -4.1349 | 3.55E-05 | 0.000143 | Down |
| ZNRF3-IT1 | 2.714765173 | -1.716768472 | 0.57763 | -2.97209 | 0.002958 | 0.007787 | Down |
| ZNRF4 | 1.157977797 | -1.786878159 | 0.770164 | -2.32013 | 0.020334 | 0.041946 | Down |
| ZP1 | 3.981635797 | -1.520175533 | 0.327523 | -4.64143 | 3.46E-06 | 1.65E-05 | Down |
| ZP2 | 4.463638956 | -2.311835968 | 0.420834 | -5.49346 | 3.94E-08 | 2.45E-07 | Down |
| ZPBP | 6.764282118 | -1.839567031 | 0.218582 | -8.41592 | 3.90E-17 | 5.80E-16 | Down |
| ZPBP2 | 2.161133733 | -1.358232847 | 0.589954 | -2.30227 | 0.02132 | 0.043693 | Down |
| ZSCAN1 | 23.54178993 | -1.203586172 | 0.1574 | -7.64667 | 2.06E-14 | 2.45E-13 | Down |
| ZSCAN5B | 2.148947638 | -1.534837312 | 0.378404 | -4.05608 | 4.99E-05 | 0.000195 | Down |
| ZSCAN5C | 1.647931789 | -1.790547049 | 0.642543 | -2.78666 | 0.005325 | 0.013109 | Down |
| ZSWIM2 | 2.136916142 | -2.384171606 | 0.750432 | -3.17707 | 0.001488 | 0.00424 | Down |
| ZSWIM5P2 | 1.708961955 | -3.190834645 | 1.064347 | -2.99793 | 0.002718 | 0.007213 | Down |
| ZYG11A | 154.1907673 | -1.154001362 | 0.11267 | -10.2423 | 1.28E-24 | 3.33E-23 | Down |

Table S6: Differential expression genes between TCGA tumor samples and normal samples.

| row | baseMean | log2FoldChange | lfcSE | stat | pvalue | padj | change |
| --- | --- | --- | --- | --- | --- | --- | --- |
| TNMD | 5.552144212 | -1.047714206 | 0.301412 | -3.47602 | 0.000509 | 0.002144 | Down |
| SEMA3F | 3526.689617 | 1.502434506 | 0.1183 | 12.70018 | 5.90E-37 | 1.83E-34 | Up |
| AOC1 | 97.57110637 | 2.383431262 | 0.382239 | 6.235441 | 4.51E-10 | 6.67E-09 | Up |
| TFPI | 1478.80566 | -1.15768889 | 0.147755 | -7.83521 | 4.68E-15 | 1.49E-13 | Down |
| PLXND1 | 6819.011374 | 1.562202605 | 0.123274 | 12.67264 | 8.38E-37 | 2.56E-34 | Up |
| CD38 | 501.9111542 | -1.020894381 | 0.322517 | -3.1654 | 0.001549 | 0.005671 | Down |
| HSPB6 | 753.4256055 | -1.087619514 | 0.202871 | -5.36115 | 8.27E-08 | 8.20E-07 | Down |
| SLC22A16 | 2.709228926 | 1.115517665 | 0.423134 | 2.636326 | 0.008381 | 0.024232 | Up |
| ARX | 51.59158466 | 1.671390523 | 0.486315 | 3.436847 | 0.000589 | 0.002439 | Up |
| PRSS22 | 467.0207149 | 1.403215425 | 0.171526 | 8.180786 | 2.82E-16 | 1.04E-14 | Up |
| HOXA11 | 2.549999537 | 3.912855416 | 0.555854 | 7.039353 | 1.93E-12 | 4.24E-11 | Up |
| ITGA3 | 27163.32887 | 1.535248207 | 0.128326 | 11.96366 | 5.51E-33 | 1.19E-30 | Up |
| CRLF1 | 1488.68245 | 3.252474331 | 0.30475 | 10.67261 | 1.37E-26 | 1.60E-24 | Up |
| TMEM98 | 5705.124264 | 1.197278584 | 0.134559 | 8.897812 | 5.70E-19 | 2.96E-17 | Up |
| TMEM132A | 478.2251525 | 1.311482174 | 0.219038 | 5.98746 | 2.13E-09 | 2.81E-08 | Up |
| TAC1 | 29.36657376 | -1.917944819 | 0.410439 | -4.67291 | 2.97E-06 | 2.15E-05 | Down |
| CX3CL1 | 4291.232916 | 1.279782041 | 0.148125 | 8.63987 | 5.63E-18 | 2.55E-16 | Up |
| TNFRSF12A | 5089.10211 | 1.276516658 | 0.344952 | 3.700559 | 0.000215 | 0.000998 | Up |
| ETV1 | 1811.429318 | 1.14267513 | 0.162 | 7.053567 | 1.74E-12 | 3.86E-11 | Up |
| ALDH3B1 | 1097.553303 | 2.348973745 | 0.200889 | 11.69289 | 1.39E-31 | 2.72E-29 | Up |
| CCL26 | 8.95338928 | 1.978438529 | 0.254849 | 7.763181 | 8.28E-15 | 2.55E-13 | Up |
| GGCT | 2249.163217 | 2.026816013 | 0.149281 | 13.57715 | 5.47E-42 | 2.93E-39 | Up |
| PROM1 | 747.6759985 | -2.496370631 | 0.362594 | -6.88476 | 5.79E-12 | 1.18E-10 | Down |
| SLC13A2 | 2.426788862 | -2.757873603 | 0.482416 | -5.71679 | 1.09E-08 | 1.26E-07 | Down |
| CEACAM7 | 1.025180024 | -1.453327228 | 0.550688 | -2.63911 | 0.008312 | 0.024075 | Down |
| CD79B | 1079.936122 | -1.401613456 | 0.505371 | -2.77343 | 0.005547 | 0.017047 | Down |
| SCN4A | 935.2352929 | 1.119344498 | 0.179402 | 6.239292 | 4.40E-10 | 6.52E-09 | Up |
| BAIAP3 | 624.6480934 | 3.495710746 | 0.274413 | 12.73886 | 3.60E-37 | 1.14E-34 | Up |
| SYN1 | 61.7759745 | 2.764184657 | 0.250594 | 11.03054 | 2.72E-28 | 3.87E-26 | Up |
| CAMK1G | 15.54220027 | 1.190310951 | 0.35534 | 3.349784 | 0.000809 | 0.003216 | Up |
| ADAM22 | 946.1334397 | -1.131869135 | 0.18104 | -6.25202 | 4.05E-10 | 6.05E-09 | Down |
| TENM1 | 2102.966673 | 4.661949699 | 0.28908 | 16.12683 | 1.65E-58 | 6.89E-55 | Up |
| MLXIPL | 386.537519 | 1.759368349 | 0.253173 | 6.949269 | 3.67E-12 | 7.77E-11 | Up |
| GIPR | 69.31839195 | 1.643672214 | 0.267231 | 6.150764 | 7.71E-10 | 1.09E-08 | Up |
| PRSS3 | 6.561652682 | 1.315150199 | 0.266293 | 4.938727 | 7.86E-07 | 6.42E-06 | Up |
| FYN | 6557.777242 | 1.22948103 | 0.110144 | 11.16254 | 6.22E-29 | 9.49E-27 | Up |
| AC080038.1 | 5212.609637 | 2.907081488 | 0.231798 | 12.54146 | 4.43E-36 | 1.22E-33 | Up |
| SYT7 | 1490.23435 | -1.213057416 | 0.243013 | -4.99173 | 5.98E-07 | 5.00E-06 | Down |
| PLAUR | 1091.655613 | 1.667196524 | 0.348198 | 4.788074 | 1.68E-06 | 1.28E-05 | Up |
| DCN | 21640.19979 | -1.087688418 | 0.241422 | -4.50534 | 6.63E-06 | 4.43E-05 | Down |
| LTF | 2788.516876 | -1.904826813 | 0.32848 | -5.79891 | 6.67E-09 | 8.01E-08 | Down |
| ALOX5 | 2013.5825 | 2.626518881 | 0.377149 | 6.964141 | 3.30E-12 | 7.05E-11 | Up |
| SLC7A14 | 3.490501444 | -1.334491641 | 0.49278 | -2.70809 | 0.006767 | 0.020201 | Down |
| CLDN11 | 172.0621999 | 1.226672115 | 0.204947 | 5.985319 | 2.16E-09 | 2.85E-08 | Up |
| MVP | 9878.53348 | 1.319883371 | 0.170517 | 7.740494 | 9.90E-15 | 3.03E-13 | Up |
| MAMLD1 | 487.1011742 | 2.575485222 | 0.188095 | 13.69243 | 1.13E-42 | 6.50E-40 | Up |
| ACPP | 258.9002597 | 1.034473051 | 0.201289 | 5.139244 | 2.76E-07 | 2.47E-06 | Up |
| MTMR11 | 377.1003738 | 2.017746987 | 0.167312 | 12.05981 | 1.72E-33 | 3.85E-31 | Up |
| BID | 1119.735152 | 1.755433617 | 0.17905 | 9.804161 | 1.08E-22 | 8.46E-21 | Up |
| STMN4 | 0.845784934 | 1.237083118 | 0.469555 | 2.634586 | 0.008424 | 0.024345 | Up |
| ISL1 | 16.30032445 | -2.230330656 | 0.425308 | -5.24403 | 1.57E-07 | 1.47E-06 | Down |
| IL20RA | 195.8159325 | -1.53518279 | 0.209465 | -7.32908 | 2.32E-13 | 5.85E-12 | Down |
| CNTN1 | 207.0447284 | -1.266440702 | 0.269336 | -4.70209 | 2.58E-06 | 1.88E-05 | Down |
| ZNF806 | 1.132001825 | -1.221629137 | 0.491438 | -2.48583 | 0.012925 | 0.034855 | Down |
| MARCO | 492.0937126 | 1.549414925 | 0.464827 | 3.333313 | 0.000858 | 0.003386 | Up |
| CYP24A1 | 381.8066313 | 1.468237777 | 0.421697 | 3.481736 | 0.000498 | 0.002103 | Up |
| SAMD4A | 2052.627646 | 1.076384083 | 0.173335 | 6.209849 | 5.30E-10 | 7.72E-09 | Up |
| C8B | 3.907931863 | 2.450496811 | 0.634074 | 3.864685 | 0.000111 | 0.000557 | Up |
| FHL1 | 34067.26063 | -1.167115395 | 0.216366 | -5.39417 | 6.88E-08 | 6.93E-07 | Down |
| GABRA1 | 1.551048399 | 4.144247149 | 0.867836 | 4.775378 | 1.79E-06 | 1.35E-05 | Up |
| CD44 | 21069.00457 | 1.225136845 | 0.123923 | 9.886296 | 4.77E-23 | 3.90E-21 | Up |
| AGPAT4 | 760.2287499 | -1.338806052 | 0.14932 | -8.966 | 3.07E-19 | 1.65E-17 | Down |
| ARNTL2 | 219.7015282 | 1.168165245 | 0.28754 | 4.062621 | 4.85E-05 | 0.000266 | Up |
| IBSP | 10.23909946 | 6.006254521 | 0.522516 | 11.49488 | 1.40E-30 | 2.49E-28 | Up |
| MUSK | 14.05828282 | -1.149192714 | 0.341257 | -3.36752 | 0.000758 | 0.003039 | Down |
| LRRC7 | 455.1936402 | -1.555835253 | 0.201724 | -7.7127 | 1.23E-14 | 3.71E-13 | Down |
| MYOC | 870.3763183 | -1.54092278 | 0.443596 | -3.47371 | 0.000513 | 0.00216 | Down |
| DEPDC1B | 243.5215232 | 1.276479422 | 0.263963 | 4.835836 | 1.33E-06 | 1.03E-05 | Up |
| DAPK2 | 13482.60824 | 1.420078851 | 0.215113 | 6.601559 | 4.07E-11 | 7.25E-10 | Up |
| STAP1 | 162.064877 | -2.021318657 | 0.603715 | -3.34813 | 0.000814 | 0.003233 | Down |
| OTC | 11.22225636 | -1.891010776 | 0.355376 | -5.32115 | 1.03E-07 | 1.00E-06 | Down |
| VCAN | 1871.357905 | 2.235563671 | 0.299131 | 7.473521 | 7.81E-14 | 2.09E-12 | Up |
| C6 | 93.59855697 | -1.494117872 | 0.281145 | -5.3144 | 1.07E-07 | 1.04E-06 | Down |
| RTN4R | 534.7421018 | 1.254029368 | 0.15071 | 8.320835 | 8.73E-17 | 3.50E-15 | Up |
| RAB27B | 140.4514133 | 2.851664497 | 0.32227 | 8.848688 | 8.86E-19 | 4.45E-17 | Up |
| MYO16 | 33.81517308 | -2.120381929 | 0.27701 | -7.65453 | 1.94E-14 | 5.67E-13 | Down |
| TNC | 4042.692702 | 1.884063331 | 0.359151 | 5.245885 | 1.56E-07 | 1.46E-06 | Up |
| ZPBP | 15.91356631 | -1.510852517 | 0.202841 | -7.44846 | 9.44E-14 | 2.50E-12 | Down |
| BARX2 | 45.82125569 | -1.494908995 | 0.280199 | -5.33517 | 9.55E-08 | 9.35E-07 | Down |
| ZIC2 | 2.801932076 | -2.531940842 | 0.452049 | -5.60103 | 2.13E-08 | 2.34E-07 | Down |
| EPHA3 | 2107.52791 | -1.402413389 | 0.183518 | -7.64183 | 2.14E-14 | 6.21E-13 | Down |
| CP | 657.3160077 | -1.058357516 | 0.281204 | -3.76366 | 0.000167 | 0.000802 | Down |
| ARHGAP6 | 4108.33632 | -1.063999904 | 0.139131 | -7.64748 | 2.05E-14 | 5.96E-13 | Down |
| ROS1 | 2.739136898 | 1.339248027 | 0.545568 | 2.454777 | 0.014097 | 0.037482 | Up |
| HDAC9 | 806.8715237 | 1.386815669 | 0.246223 | 5.632347 | 1.78E-08 | 1.98E-07 | Up |
| LMO3 | 11377.36884 | 1.34274591 | 0.120026 | 11.18714 | 4.71E-29 | 7.31E-27 | Up |
| ADAMTS6 | 52.82570242 | 1.054402249 | 0.215415 | 4.894757 | 9.84E-07 | 7.87E-06 | Up |
| UTS2 | 10.57783542 | 1.049001331 | 0.441284 | 2.377156 | 0.017447 | 0.044694 | Up |
| LTBP1 | 1317.055281 | 1.2524203 | 0.241097 | 5.194678 | 2.05E-07 | 1.89E-06 | Up |
| NEXMIF | 135.2388167 | -1.183200738 | 0.199587 | -5.92824 | 3.06E-09 | 3.93E-08 | Down |
| NFE2L3 | 1023.54125 | 2.274837491 | 0.285954 | 7.95526 | 1.79E-15 | 5.99E-14 | Up |
| COL23A1 | 10247.55148 | -1.560314987 | 0.177136 | -8.80858 | 1.27E-18 | 6.26E-17 | Down |
| FSTL4 | 87.78971062 | 3.38037151 | 0.296176 | 11.41339 | 3.58E-30 | 6.23E-28 | Up |
| METTL24 | 43.61851288 | -1.066658516 | 0.2308 | -4.62157 | 3.81E-06 | 2.68E-05 | Down |
| ENTPD2 | 160.0190807 | 2.84462017 | 0.277154 | 10.26369 | 1.03E-24 | 9.93E-23 | Up |
| CBLN4 | 78.5798891 | 2.189190018 | 0.298021 | 7.345758 | 2.05E-13 | 5.19E-12 | Up |
| CHRDL2 | 12.8836793 | 1.189306981 | 0.293619 | 4.05051 | 5.11E-05 | 0.000279 | Up |
| CCDC85A | 610.0782013 | -1.45598977 | 0.215894 | -6.744 | 1.54E-11 | 2.94E-10 | Down |
| PKP2 | 181.2459292 | 1.597640657 | 0.33476 | 4.77249 | 1.82E-06 | 1.37E-05 | Up |
| F7 | 13.83386979 | 1.015106164 | 0.261996 | 3.874512 | 0.000107 | 0.000537 | Up |
| PRDM1 | 4131.758055 | 1.822359223 | 0.205346 | 8.87459 | 7.02E-19 | 3.60E-17 | Up |
| LAMC2 | 8775.639095 | 1.100923239 | 0.148098 | 7.43377 | 1.06E-13 | 2.77E-12 | Up |
| RASGRF1 | 308.4655071 | 4.360796471 | 0.389583 | 11.19349 | 4.39E-29 | 6.86E-27 | Up |
| STYK1 | 22.49993744 | 1.39670499 | 0.304108 | 4.592794 | 4.37E-06 | 3.04E-05 | Up |
| GNA15 | 254.543617 | 1.061720831 | 0.323568 | 3.28129 | 0.001033 | 0.003983 | Up |
| PTPRU | 1772.478962 | 1.180693392 | 0.176542 | 6.687884 | 2.26E-11 | 4.20E-10 | Up |
| RIMBP2 | 195.0706731 | 1.502148195 | 0.234772 | 6.398324 | 1.57E-10 | 2.53E-09 | Up |
| COL11A1 | 479.4900983 | 5.762231771 | 0.489784 | 11.76484 | 5.92E-32 | 1.20E-29 | Up |
| BCAT1 | 781.1104435 | 1.386116988 | 0.268775 | 5.157167 | 2.51E-07 | 2.26E-06 | Up |
| LZTS1 | 405.4733968 | 2.17370716 | 0.204856 | 10.61092 | 2.65E-26 | 3.02E-24 | Up |
| WNT8A | 1.201328061 | 1.078517554 | 0.405526 | 2.659553 | 0.007824 | 0.02287 | Up |
| SPAG4 | 257.9968007 | 1.06401367 | 0.280591 | 3.792051 | 0.000149 | 0.000725 | Up |
| GUCY1B1 | 640.028911 | 1.267078372 | 0.129631 | 9.774529 | 1.45E-22 | 1.12E-20 | Up |
| CDH3 | 2396.359995 | 3.743496564 | 0.259828 | 14.40762 | 4.63E-47 | 4.58E-44 | Up |
| SLC6A16 | 123.6319936 | -1.24707959 | 0.141809 | -8.79409 | 1.44E-18 | 7.04E-17 | Down |
| CA11 | 2080.146677 | 1.270274182 | 0.140953 | 9.012045 | 2.02E-19 | 1.12E-17 | Up |
| ATP2C2 | 1326.561274 | -2.073720911 | 0.21359 | -9.70886 | 2.76E-22 | 2.09E-20 | Down |
| NGFR | 1306.939822 | 1.774604232 | 0.263799 | 6.727103 | 1.73E-11 | 3.27E-10 | Up |
| CDON | 4754.185712 | -1.147878638 | 0.198423 | -5.785 | 7.25E-09 | 8.66E-08 | Down |
| NTN1 | 283.3664939 | -1.460044128 | 0.2008 | -7.27112 | 3.57E-13 | 8.76E-12 | Down |
| GLP2R | 18.93252358 | -1.193273406 | 0.347662 | -3.43228 | 0.000599 | 0.002475 | Down |
| ERBB3 | 2567.536854 | 1.420286556 | 0.186452 | 7.617418 | 2.59E-14 | 7.39E-13 | Up |
| ROPN1 | 1.192830324 | -1.635651075 | 0.547898 | -2.98532 | 0.002833 | 0.009549 | Down |
| SNAP91 | 69.54279185 | -1.555172176 | 0.282053 | -5.51376 | 3.51E-08 | 3.71E-07 | Down |
| NGEF | 800.2938422 | 4.988431383 | 0.318292 | 15.6725 | 2.33E-55 | 5.15E-52 | Up |
| MPPED2 | 3208.909537 | -1.608134536 | 0.233527 | -6.88629 | 5.73E-12 | 1.17E-10 | Down |
| ACSM2B | 10.30580349 | -2.456575822 | 0.298117 | -8.24032 | 1.72E-16 | 6.57E-15 | Down |
| KLF6 | 34855.76239 | -1.183055676 | 0.292327 | -4.04703 | 5.19E-05 | 0.000283 | Down |
| CACNB1 | 733.8099141 | 1.557072208 | 0.139179 | 11.18753 | 4.69E-29 | 7.31E-27 | Up |
| SYT1 | 198.7478127 | 3.515480972 | 0.348746 | 10.08034 | 6.75E-24 | 6.15E-22 | Up |
| HDAC4 | 1835.764981 | -1.045551521 | 0.145518 | -7.18502 | 6.72E-13 | 1.60E-11 | Down |
| FGFR3 | 329.4110582 | 1.564315441 | 0.202962 | 7.707422 | 1.28E-14 | 3.85E-13 | Up |
| ATP11A | 7842.841653 | 1.366062354 | 0.155646 | 8.776733 | 1.68E-18 | 8.17E-17 | Up |
| STON1-GTF2A1L | 14.92546613 | -1.022402243 | 0.300971 | -3.39701 | 0.000681 | 0.002767 | Down |
| RASGRP2 | 713.9675239 | -1.1456966 | 0.365083 | -3.13818 | 0.0017 | 0.006132 | Down |
| ADGRF5 | 5976.137451 | 1.048741065 | 0.157658 | 6.651998 | 2.89E-11 | 5.27E-10 | Up |
| PLA2G10 | 7.005889316 | 1.301509042 | 0.270472 | 4.811985 | 1.49E-06 | 1.15E-05 | Up |
| HES2 | 43.63236265 | 2.795068867 | 0.418344 | 6.681266 | 2.37E-11 | 4.38E-10 | Up |
| RAB27A | 5106.522252 | 1.554593557 | 0.116753 | 13.31527 | 1.89E-40 | 8.64E-38 | Up |
| FGF10 | 73.7725069 | -2.227268234 | 0.297875 | -7.47719 | 7.59E-14 | 2.04E-12 | Down |
| FSTL3 | 1485.387062 | 1.294188054 | 0.199829 | 6.476474 | 9.39E-11 | 1.57E-09 | Up |
| FRMPD1 | 36.01438712 | -1.00531917 | 0.231203 | -4.34821 | 1.37E-05 | 8.54E-05 | Down |
| CNGB1 | 8.07082645 | 1.293362475 | 0.257811 | 5.016701 | 5.26E-07 | 4.44E-06 | Up |
| CHAT | 0.811760914 | 2.835850198 | 0.740933 | 3.827406 | 0.00013 | 0.000638 | Up |
| SLC12A3 | 18.21052367 | -1.055012052 | 0.425663 | -2.47852 | 0.013193 | 0.035455 | Down |
| TRPM5 | 85.75521172 | -1.370191027 | 0.229224 | -5.97751 | 2.27E-09 | 2.97E-08 | Down |
| AP001034.1 | 10.06545043 | 1.648349149 | 0.395967 | 4.16284 | 3.14E-05 | 0.00018 | Up |
| CDH19 | 32.52871827 | -1.950707137 | 0.259362 | -7.52118 | 5.43E-14 | 1.49E-12 | Down |
| SLC6A15 | 67.63681305 | -3.550332945 | 0.401434 | -8.84413 | 9.22E-19 | 4.62E-17 | Down |
| RPS6KA6 | 1861.026761 | -1.191274214 | 0.159271 | -7.47954 | 7.46E-14 | 2.00E-12 | Down |
| SREBF1 | 5232.342665 | 1.137113207 | 0.117806 | 9.652447 | 4.80E-22 | 3.53E-20 | Up |
| TRPC5 | 320.663906 | 4.939576055 | 0.565401 | 8.736416 | 2.41E-18 | 1.15E-16 | Up |
| TRHDE | 17.24826133 | -1.546755183 | 0.268078 | -5.7698 | 7.94E-09 | 9.41E-08 | Down |
| P4HA2 | 2810.885382 | 1.856074025 | 0.137234 | 13.52491 | 1.11E-41 | 5.65E-39 | Up |
| CYP2W1 | 18.29431416 | 2.662690494 | 0.4061 | 6.556733 | 5.50E-11 | 9.62E-10 | Up |
| TP63 | 142.2397884 | 2.35243598 | 0.312484 | 7.528179 | 5.15E-14 | 1.41E-12 | Up |
| GSDMB | 716.1459654 | 1.219275341 | 0.184246 | 6.617638 | 3.65E-11 | 6.55E-10 | Up |
| ABCB11 | 3.923650991 | -1.861824128 | 0.618269 | -3.01135 | 0.002601 | 0.008871 | Down |
| IGF2BP2 | 1693.818455 | 1.674718386 | 0.18574 | 9.016469 | 1.94E-19 | 1.08E-17 | Up |
| NOTCH3 | 5358.641914 | 1.310167489 | 0.137831 | 9.505629 | 1.99E-21 | 1.36E-19 | Up |
| CA12 | 2000.396361 | 2.116502587 | 0.335736 | 6.304071 | 2.90E-10 | 4.46E-09 | Up |
| IPCEF1 | 6897.792711 | -2.406879118 | 0.261565 | -9.20183 | 3.52E-20 | 2.11E-18 | Down |
| WSCD2 | 5110.81343 | -1.376524488 | 0.342864 | -4.01479 | 5.95E-05 | 0.000318 | Down |
| CACNG4 | 88.53221591 | 3.897050705 | 0.406127 | 9.595646 | 8.34E-22 | 5.95E-20 | Up |
| MOCOS | 136.8699002 | 1.055952925 | 0.190836 | 5.533301 | 3.14E-08 | 3.35E-07 | Up |
| RGS11 | 141.4422769 | 1.311637649 | 0.209761 | 6.253001 | 4.03E-10 | 6.02E-09 | Up |
| ACACB | 4051.610868 | -1.082333875 | 0.139303 | -7.76964 | 7.87E-15 | 2.44E-13 | Down |
| AP002956.1 | 4018.437525 | 1.239530374 | 0.137855 | 8.991573 | 2.44E-19 | 1.33E-17 | Up |
| RAP1GAP | 22787.52836 | -1.102830569 | 0.194143 | -5.6805 | 1.34E-08 | 1.53E-07 | Down |
| NMRK2 | 4.447734955 | -1.354327558 | 0.390424 | -3.46886 | 0.000523 | 0.002195 | Down |
| PAK3 | 1150.041133 | -1.807709294 | 0.243081 | -7.43667 | 1.03E-13 | 2.72E-12 | Down |
| CAPN6 | 419.8058053 | -1.805846175 | 0.289725 | -6.23297 | 4.58E-10 | 6.77E-09 | Down |
| DCX | 4.001279693 | -1.494287337 | 0.43516 | -3.43388 | 0.000595 | 0.002462 | Down |
| TYR | 0.34851811 | 1.936253251 | 0.699734 | 2.767126 | 0.005655 | 0.017326 | Up |
| ACTN2 | 23.7474184 | -1.380594865 | 0.27389 | -5.04069 | 4.64E-07 | 3.96E-06 | Down |
| NAALAD2 | 99.53471969 | -1.013142014 | 0.179333 | -5.64951 | 1.61E-08 | 1.81E-07 | Down |
| FBLN1 | 9603.760923 | -1.086510323 | 0.255779 | -4.24784 | 2.16E-05 | 0.000128 | Down |
| FAP | 336.979798 | 2.049799365 | 0.280925 | 7.296598 | 2.95E-13 | 7.33E-12 | Up |
| RBFOX1 | 43.22560759 | 3.711187647 | 0.426159 | 8.708455 | 3.08E-18 | 1.45E-16 | Up |
| FGF20 | 25.92340255 | 1.587238656 | 0.241718 | 6.566485 | 5.15E-11 | 9.06E-10 | Up |
| BRINP1 | 213.8322965 | 3.080891034 | 0.284787 | 10.81824 | 2.82E-27 | 3.61E-25 | Up |
| CLUL1 | 511.9231902 | -1.449104836 | 0.209844 | -6.90562 | 5.00E-12 | 1.03E-10 | Down |
| CDH17 | 14.04319888 | 1.445298823 | 0.268764 | 5.377582 | 7.55E-08 | 7.54E-07 | Up |
| DUSP13 | 15.86450913 | 3.244009295 | 0.481451 | 6.737985 | 1.61E-11 | 3.05E-10 | Up |
| DDX43 | 106.7726356 | -1.031044645 | 0.30233 | -3.41033 | 0.000649 | 0.002654 | Down |
| CRYBG3 | 5927.594354 | -1.049202003 | 0.264332 | -3.96926 | 7.21E-05 | 0.000378 | Down |
| EPHA6 | 95.19960545 | -1.942098158 | 0.202465 | -9.59227 | 8.62E-22 | 6.12E-20 | Down |
| SLC4A4 | 6186.2074 | -1.634985675 | 0.267879 | -6.10344 | 1.04E-09 | 1.43E-08 | Down |
| PIH1D3 | 8.12598933 | -1.126730346 | 0.252618 | -4.46021 | 8.19E-06 | 5.37E-05 | Down |
| COL5A3 | 887.4785798 | 1.520283971 | 0.182123 | 8.347584 | 6.97E-17 | 2.81E-15 | Up |
| KCNN2 | 29.62987981 | 1.040017586 | 0.167591 | 6.205704 | 5.45E-10 | 7.91E-09 | Up |
| IGSF9B | 380.3930087 | -1.398609373 | 0.193187 | -7.23967 | 4.50E-13 | 1.09E-11 | Down |
| CXCL2 | 968.9726723 | 1.26701787 | 0.385804 | 3.284098 | 0.001023 | 0.003948 | Up |
| LRP2 | 16856.9511 | -1.000696339 | 0.216739 | -4.61706 | 3.89E-06 | 2.74E-05 | Down |
| PCDHA6 | 15.24579159 | 1.500324702 | 0.280596 | 5.346919 | 8.95E-08 | 8.80E-07 | Up |
| STRADB | 1390.264938 | 1.198629346 | 0.120021 | 9.986809 | 1.74E-23 | 1.51E-21 | Up |
| COL19A1 | 59.90960846 | 2.274602391 | 0.517106 | 4.398717 | 1.09E-05 | 6.93E-05 | Up |
| KCNK2 | 1264.816416 | -1.642691667 | 0.265106 | -6.19636 | 5.78E-10 | 8.36E-09 | Down |
| OPRK1 | 15.5603957 | -2.834973986 | 0.329129 | -8.61356 | 7.08E-18 | 3.15E-16 | Down |
| SEMA5B | 247.9054702 | 1.137784128 | 0.222299 | 5.118257 | 3.08E-07 | 2.74E-06 | Up |
| TRPM3 | 197.706585 | -1.795724655 | 0.275306 | -6.52265 | 6.91E-11 | 1.19E-09 | Down |
| EPYC | 29.17975147 | 3.526105705 | 0.671372 | 5.25209 | 1.50E-07 | 1.42E-06 | Up |
| SLCO1A2 | 3.725181145 | -2.046063448 | 0.381041 | -5.36966 | 7.89E-08 | 7.85E-07 | Down |
| APOB | 5.15966789 | 1.009215937 | 0.406119 | 2.485024 | 0.012954 | 0.034917 | Up |
| GCKR | 12.36618045 | 2.264356574 | 0.29485 | 7.679696 | 1.59E-14 | 4.72E-13 | Up |
| FCN1 | 263.5309901 | 1.267403367 | 0.338518 | 3.743973 | 0.000181 | 0.000858 | Up |
| WNT11 | 355.675558 | -1.46363559 | 0.239358 | -6.11483 | 9.67E-10 | 1.34E-08 | Down |
| AQP6 | 17.66303436 | 2.06589807 | 0.302905 | 6.820273 | 9.09E-12 | 1.80E-10 | Up |
| FOLH1 | 85.1040319 | 1.978251759 | 0.252239 | 7.842762 | 4.41E-15 | 1.41E-13 | Up |
| NME8 | 34.86458999 | 2.576708938 | 0.373672 | 6.895648 | 5.36E-12 | 1.10E-10 | Up |
| CEACAM6 | 180.4073054 | 3.88694081 | 0.503795 | 7.715325 | 1.21E-14 | 3.64E-13 | Up |
| FAT2 | 138.3230519 | 1.176999614 | 0.237624 | 4.953211 | 7.30E-07 | 6.00E-06 | Up |
| PPEF1 | 22.04927747 | 1.599899556 | 0.222013 | 7.206348 | 5.75E-13 | 1.38E-11 | Up |
| NOX4 | 193.6799693 | 2.345530259 | 0.22222 | 10.555 | 4.82E-26 | 5.34E-24 | Up |
| PPP1R15A | 10763.33887 | -1.116260696 | 0.222532 | -5.01618 | 5.27E-07 | 4.45E-06 | Down |
| ADAMTS2 | 542.2796857 | 1.562430454 | 0.240308 | 6.501785 | 7.94E-11 | 1.35E-09 | Up |
| TMPRSS11E | 41.37320736 | 5.355071256 | 0.517639 | 10.34519 | 4.40E-25 | 4.45E-23 | Up |
| PHACTR3 | 37.41902485 | 2.532643708 | 0.281994 | 8.981203 | 2.68E-19 | 1.45E-17 | Up |
| SULT2B1 | 225.2195587 | 1.441791777 | 0.255745 | 5.637604 | 1.72E-08 | 1.93E-07 | Up |
| FER1L4 | 198.0795855 | 1.737258472 | 0.339253 | 5.120828 | 3.04E-07 | 2.71E-06 | Up |
| DOCK9 | 10294.34739 | 1.229277958 | 0.120252 | 10.22251 | 1.57E-24 | 1.50E-22 | Up |
| TMEM40 | 6.904172572 | 1.933341141 | 0.334534 | 5.779215 | 7.51E-09 | 8.93E-08 | Up |
| CPXM1 | 3517.755493 | -1.198534745 | 0.294052 | -4.07592 | 4.58E-05 | 0.000253 | Down |
| F11 | 2.285816378 | -2.280554898 | 0.579416 | -3.93595 | 8.29E-05 | 0.000427 | Down |
| TESC | 2620.655775 | 3.204941215 | 0.246218 | 13.01666 | 9.84E-39 | 3.69E-36 | Up |
| CFAP61 | 58.94855508 | -1.300416705 | 0.171591 | -7.57856 | 3.49E-14 | 9.77E-13 | Down |
| RPH3A | 11.11967847 | 1.729647033 | 0.378753 | 4.566685 | 4.95E-06 | 3.41E-05 | Up |
| CHGB | 408.586236 | 1.1756536 | 0.358594 | 3.278509 | 0.001044 | 0.004017 | Up |
| NOS1 | 92.78916832 | -1.72004468 | 0.342374 | -5.02387 | 5.06E-07 | 4.30E-06 | Down |
| FXYD5 | 4087.797314 | 1.578000755 | 0.277143 | 5.693816 | 1.24E-08 | 1.43E-07 | Up |
| FXYD3 | 71.45122107 | 2.987568072 | 0.400271 | 7.463871 | 8.40E-14 | 2.24E-12 | Up |
| ICAM1 | 3343.590697 | 1.87941764 | 0.261922 | 7.17549 | 7.20E-13 | 1.70E-11 | Up |
| FETUB | 1.250879924 | 3.534584459 | 0.768652 | 4.598417 | 4.26E-06 | 2.96E-05 | Up |
| NRCAM | 1872.365862 | 2.169546988 | 0.169739 | 12.78168 | 2.08E-37 | 6.71E-35 | Up |
| SLC26A4 | 42301.15729 | -1.190019784 | 0.23387 | -5.08837 | 3.61E-07 | 3.16E-06 | Down |
| SLC26A3 | 29.66560987 | -1.00753673 | 0.376177 | -2.67836 | 0.007398 | 0.021796 | Down |
| SEL1L3 | 5018.143849 | 1.153281752 | 0.232572 | 4.958817 | 7.09E-07 | 5.84E-06 | Up |
| APOH | 0.797910468 | 1.958280288 | 0.833281 | 2.350085 | 0.018769 | 0.047463 | Up |
| RGS17 | 333.8633634 | -1.123836923 | 0.231463 | -4.85536 | 1.20E-06 | 9.42E-06 | Down |
| MYH7 | 33.9132689 | -2.393877108 | 0.489115 | -4.89431 | 9.87E-07 | 7.88E-06 | Down |
| TGM1 | 187.6564678 | 1.59367494 | 0.21438 | 7.433895 | 1.05E-13 | 2.77E-12 | Up |
| TYRO3 | 498.8562409 | 1.990401412 | 0.142751 | 13.94314 | 3.46E-44 | 2.50E-41 | Up |
| CAPN3 | 94.37025739 | 1.619358675 | 0.227714 | 7.111378 | 1.15E-12 | 2.64E-11 | Up |
| GABRP | 13.59544814 | 1.553024614 | 0.269775 | 5.756745 | 8.58E-09 | 1.01E-07 | Up |
| KRT31 | 0.997916972 | 1.285872996 | 0.524656 | 2.450889 | 0.01425 | 0.037817 | Up |
| FMO2 | 624.4167011 | -1.274733846 | 0.245358 | -5.19541 | 2.04E-07 | 1.88E-06 | Down |
| TBC1D2 | 2738.224871 | 1.793816707 | 0.155115 | 11.56446 | 6.24E-31 | 1.16E-28 | Up |
| CYP26A1 | 15.89865891 | 3.497999874 | 0.420751 | 8.313715 | 9.28E-17 | 3.69E-15 | Up |
| CRTAC1 | 909.8432334 | -1.033382924 | 0.197129 | -5.24216 | 1.59E-07 | 1.49E-06 | Down |
| IL11 | 6.451547046 | 1.114521143 | 0.395634 | 2.817048 | 0.004847 | 0.015191 | Up |
| TPSD1 | 6.019487781 | 2.081247248 | 0.552626 | 3.766106 | 0.000166 | 0.000796 | Up |
| SMIM24 | 39.35159215 | 3.347229612 | 0.422017 | 7.931502 | 2.17E-15 | 7.19E-14 | Up |
| TREM2 | 466.5495655 | 1.361996708 | 0.366431 | 3.716927 | 0.000202 | 0.000944 | Up |
| FKBP5 | 10933.3994 | -1.441339781 | 0.301774 | -4.77622 | 1.79E-06 | 1.35E-05 | Down |
| MLN | 0.826877955 | 1.573783922 | 0.566216 | 2.779475 | 0.005445 | 0.016781 | Up |
| SYDE2 | 546.8353295 | -1.056647752 | 0.156425 | -6.75497 | 1.43E-11 | 2.74E-10 | Down |
| SCD | 1015.358413 | 1.134433195 | 0.302182 | 3.754145 | 0.000174 | 0.000828 | Up |
| STX1B | 72.17218314 | 1.33064367 | 0.225566 | 5.899126 | 3.65E-09 | 4.61E-08 | Up |
| CBARP | 68.96058947 | 1.70106851 | 0.221403 | 7.683141 | 1.55E-14 | 4.60E-13 | Up |
| MISP | 175.93327 | 2.962920546 | 0.361049 | 8.206424 | 2.28E-16 | 8.55E-15 | Up |
| HCN2 | 27.75295633 | 1.357282622 | 0.402639 | 3.370967 | 0.000749 | 0.003003 | Up |
| CDHR5 | 10.96456407 | -1.188677728 | 0.254272 | -4.67482 | 2.94E-06 | 2.13E-05 | Down |
| PALM | 1970.570291 | 1.508049633 | 0.204935 | 7.358662 | 1.86E-13 | 4.73E-12 | Up |
| MADCAM1 | 78.18043614 | -1.050871354 | 0.355872 | -2.95295 | 0.003148 | 0.010463 | Down |
| MMP11 | 536.9588758 | 3.978247557 | 0.31544 | 12.61174 | 1.82E-36 | 5.42E-34 | Up |
| CECR2 | 349.153252 | -2.011236409 | 0.221272 | -9.08943 | 9.96E-20 | 5.71E-18 | Down |
| DERL3 | 2215.83328 | 1.120063622 | 0.367585 | 3.047091 | 0.002311 | 0.007994 | Up |
| SLC7A4 | 21.11308622 | 2.839491557 | 0.25038 | 11.34073 | 8.25E-30 | 1.38E-27 | Up |
| SUSD2 | 2257.624679 | 1.019154924 | 0.191032 | 5.334996 | 9.55E-08 | 9.36E-07 | Up |
| SEC14L2 | 1053.608813 | 1.005658989 | 0.110379 | 9.110979 | 8.16E-20 | 4.74E-18 | Up |
| SEC14L3 | 79.43019941 | -2.958205891 | 0.406551 | -7.27635 | 3.43E-13 | 8.45E-12 | Down |
| PRODH | 393.5651829 | 1.005651482 | 0.239076 | 4.206415 | 2.59E-05 | 0.000151 | Up |
| LRP5L | 920.4984231 | 1.168718778 | 0.132092 | 8.847764 | 8.93E-19 | 4.48E-17 | Up |
| LGALS1 | 6635.657047 | 1.518522022 | 0.204251 | 7.43457 | 1.05E-13 | 2.76E-12 | Up |
| SEPT3 | 187.5740851 | 1.421412303 | 0.208922 | 6.803568 | 1.02E-11 | 2.00E-10 | Up |
| KDELR3 | 919.6241036 | 1.556579069 | 0.161301 | 9.650163 | 4.91E-22 | 3.60E-20 | Up |
| MIOX | 826.7166444 | -1.005371163 | 0.207341 | -4.84888 | 1.24E-06 | 9.70E-06 | Down |
| APOBEC3H | 53.38961967 | 1.421760227 | 0.30009 | 4.737779 | 2.16E-06 | 1.61E-05 | Up |
| RASD2 | 546.9325473 | 2.851580148 | 0.220764 | 12.91685 | 3.62E-38 | 1.29E-35 | Up |
| PDGFB | 1344.978837 | 1.0242554 | 0.119222 | 8.591192 | 8.61E-18 | 3.80E-16 | Up |
| PNPLA5 | 32.599347 | 5.479176827 | 0.724594 | 7.561724 | 3.98E-14 | 1.11E-12 | Up |
| UPK3A | 152.0477908 | -1.575125996 | 0.362606 | -4.3439 | 1.40E-05 | 8.70E-05 | Down |
| CTSG | 14.52277346 | 1.565835125 | 0.366584 | 4.271424 | 1.94E-05 | 0.000116 | Up |
| CDKL1 | 195.7244339 | -1.376807922 | 0.169628 | -8.11665 | 4.79E-16 | 1.72E-14 | Down |
| AL139020.1 | 334.7796666 | -2.993023195 | 0.69265 | -4.32112 | 1.55E-05 | 9.52E-05 | Down |
| RPS6KA5 | 902.2765254 | -1.55659599 | 0.123698 | -12.5838 | 2.59E-36 | 7.38E-34 | Down |
| CPNE6 | 17.07741338 | -1.167492432 | 0.264149 | -4.41982 | 9.88E-06 | 6.35E-05 | Down |
| SGK2 | 57.31445935 | 1.049924576 | 0.190505 | 5.511275 | 3.56E-08 | 3.76E-07 | Up |
| R3HDML | 2.120168316 | 1.495885051 | 0.620737 | 2.409853 | 0.015959 | 0.041506 | Up |
| HNF4A | 2.481116049 | -1.840564934 | 0.625928 | -2.94054 | 0.003276 | 0.010844 | Down |
| RIMS4 | 1198.388498 | -1.283376579 | 0.234907 | -5.46335 | 4.67E-08 | 4.86E-07 | Down |
| SALL4 | 227.7953155 | -1.082335417 | 0.212297 | -5.09822 | 3.43E-07 | 3.02E-06 | Down |
| SLCO4A1 | 246.3861445 | 1.499015042 | 0.307596 | 4.873323 | 1.10E-06 | 8.69E-06 | Up |
| SLC17A9 | 372.5137483 | 1.429063006 | 0.306035 | 4.669603 | 3.02E-06 | 2.18E-05 | Up |
| BIRC7 | 221.1984541 | 5.762016369 | 0.436527 | 13.19969 | 8.81E-40 | 3.80E-37 | Up |
| NKAIN4 | 202.5204723 | 2.486907552 | 0.25672 | 9.687221 | 3.42E-22 | 2.54E-20 | Up |
| AVP | 0.703276285 | 1.802448331 | 0.602141 | 2.993397 | 0.002759 | 0.009335 | Up |
| CHRNA4 | 256.1631963 | -1.72580175 | 0.310677 | -5.55497 | 2.78E-08 | 3.00E-07 | Down |
| EEF1A2 | 59.33285509 | 1.05642864 | 0.384358 | 2.748556 | 0.005986 | 0.018203 | Up |
| ISM1 | 5147.175798 | -1.136054612 | 0.234144 | -4.85196 | 1.22E-06 | 9.57E-06 | Down |
| RNF24 | 2208.965006 | 1.014593801 | 0.089565 | 11.32807 | 9.53E-30 | 1.58E-27 | Up |
| SLC52A3 | 68.52988548 | 1.624580083 | 0.206318 | 7.874147 | 3.43E-15 | 1.11E-13 | Up |
| RSPO4 | 492.0321451 | 2.161753782 | 0.291616 | 7.413024 | 1.23E-13 | 3.22E-12 | Up |
| FERMT1 | 539.4330178 | -1.356216104 | 0.218073 | -6.21909 | 5.00E-10 | 7.34E-09 | Down |
| PAK5 | 11.69638907 | -1.937459078 | 0.267184 | -7.2514 | 4.12E-13 | 1.01E-11 | Down |
| E2F1 | 315.2781806 | 1.796933139 | 0.256711 | 6.99983 | 2.56E-12 | 5.54E-11 | Up |
| EPPIN | 19.59235172 | -2.672253179 | 0.393458 | -6.79171 | 1.11E-11 | 2.16E-10 | Down |
| SYNDIG1 | 685.4330333 | -1.189039459 | 0.200862 | -5.9197 | 3.23E-09 | 4.13E-08 | Down |
| CELF4 | 120.5934414 | 2.947801752 | 0.23191 | 12.71098 | 5.14E-37 | 1.61E-34 | Up |
| ST8SIA5 | 31.57769257 | 1.224851442 | 0.299209 | 4.093626 | 4.25E-05 | 0.000236 | Up |
| NOL4 | 275.4888544 | -2.371474953 | 0.272581 | -8.70007 | 3.32E-18 | 1.55E-16 | Down |
| CHRDL1 | 2896.555246 | -2.101115476 | 0.285459 | -7.36049 | 1.83E-13 | 4.67E-12 | Down |
| PLP2 | 3053.57685 | 1.249367644 | 0.171429 | 7.287968 | 3.15E-13 | 7.78E-12 | Up |
| SCML2 | 226.4899204 | -1.232918837 | 0.164397 | -7.49964 | 6.40E-14 | 1.73E-12 | Down |
| PCSK1N | 291.0209213 | 3.980704713 | 0.369649 | 10.76887 | 4.83E-27 | 6.08E-25 | Up |
| PHEX | 1499.75229 | 2.667088918 | 0.419859 | 6.352341 | 2.12E-10 | 3.34E-09 | Up |
| PCYT1B | 6.903131654 | 1.141161931 | 0.36818 | 3.099468 | 0.001939 | 0.006876 | Up |
| BRS3 | 3.486422661 | -1.860009322 | 0.38455 | -4.83685 | 1.32E-06 | 1.02E-05 | Down |
| VGLL1 | 32.78896257 | 4.877916706 | 0.626598 | 7.784768 | 6.98E-15 | 2.18E-13 | Up |
| TIMP1 | 16647.72924 | 2.79206779 | 0.243699 | 11.45701 | 2.17E-30 | 3.82E-28 | Up |
| KLHL4 | 135.2274302 | -2.001041174 | 0.211462 | -9.46288 | 3.00E-21 | 2.01E-19 | Down |
| PCDH11X | 49.13198262 | -2.823628353 | 0.409987 | -6.88712 | 5.69E-12 | 1.16E-10 | Down |
| SRPX2 | 132.7662579 | 2.104726758 | 0.243921 | 8.628717 | 6.20E-18 | 2.78E-16 | Up |
| DRP2 | 8.603807984 | 1.617858774 | 0.396178 | 4.083667 | 4.43E-05 | 0.000245 | Up |
| ARMCX3 | 11660.37536 | 1.64890799 | 0.161489 | 10.21065 | 1.78E-24 | 1.68E-22 | Up |
| HTR2A | 13.5356198 | -1.287620674 | 0.297473 | -4.32853 | 1.50E-05 | 9.24E-05 | Down |
| MLNR | 24.62265145 | -2.119877198 | 0.257402 | -8.23568 | 1.79E-16 | 6.80E-15 | Down |
| TSC22D1 | 27743.88379 | 1.865709742 | 0.167272 | 11.15374 | 6.87E-29 | 1.03E-26 | Up |
| OLFM4 | 36.40480272 | -2.115880546 | 0.379912 | -5.5694 | 2.56E-08 | 2.77E-07 | Down |
| MSLN | 67.40170309 | 3.035087269 | 0.380403 | 7.9786 | 1.48E-15 | 5.01E-14 | Up |
| CBLN1 | 452.6165023 | 3.780993607 | 0.300358 | 12.58827 | 2.45E-36 | 7.07E-34 | Up |
| CCL22 | 216.0719727 | 1.08685815 | 0.415136 | 2.618079 | 0.008843 | 0.02538 | Up |
| CCL17 | 131.5534353 | 3.16983707 | 0.462439 | 6.854607 | 7.15E-12 | 1.44E-10 | Up |
| HAGHL | 184.397404 | 1.364566004 | 0.312763 | 4.362935 | 1.28E-05 | 8.05E-05 | Up |
| ZP2 | 3.191253677 | -1.533069538 | 0.389261 | -3.93841 | 8.20E-05 | 0.000423 | Down |
| AQP8 | 5.092403625 | 2.698800032 | 0.378276 | 7.134482 | 9.72E-13 | 2.25E-11 | Up |
| TMC5 | 243.3836279 | -1.017984276 | 0.241375 | -4.21743 | 2.47E-05 | 0.000144 | Down |
| AQP9 | 74.87291325 | 1.354319659 | 0.366989 | 3.690354 | 0.000224 | 0.001035 | Up |
| CTSH | 11535.04346 | 2.294825473 | 0.230054 | 9.975176 | 1.96E-23 | 1.68E-21 | Up |
| CD276 | 2862.48752 | 1.066650521 | 0.099422 | 10.7285 | 7.48E-27 | 9.08E-25 | Up |
| OCA2 | 787.3341647 | -2.299109918 | 0.281314 | -8.17276 | 3.01E-16 | 1.11E-14 | Down |
| FAM189A1 | 862.9824082 | -1.327843321 | 0.271118 | -4.89767 | 9.70E-07 | 7.76E-06 | Down |
| SCG3 | 73.8216871 | -1.036535441 | 0.318372 | -3.25574 | 0.001131 | 0.004301 | Down |
| RHOV | 129.3329237 | 2.050626188 | 0.262296 | 7.817987 | 5.37E-15 | 1.70E-13 | Up |
| MYEF2 | 765.3635656 | 1.325530453 | 0.139749 | 9.485082 | 2.42E-21 | 1.64E-19 | Up |
| RP1 | 3.856137689 | 2.172901661 | 0.526535 | 4.126795 | 3.68E-05 | 0.000207 | Up |
| CA2 | 466.8396293 | 1.21133208 | 0.189421 | 6.394908 | 1.61E-10 | 2.58E-09 | Up |
| EYA1 | 68.65911602 | -2.196325139 | 0.25303 | -8.6801 | 3.95E-18 | 1.83E-16 | Down |
| CCN4 | 91.67879205 | 1.837160408 | 0.299309 | 6.138009 | 8.36E-10 | 1.18E-08 | Up |
| STMN2 | 48.95813342 | 3.372301283 | 0.476068 | 7.083648 | 1.40E-12 | 3.17E-11 | Up |
| SH2D4A | 2325.115954 | 1.08049451 | 0.116135 | 9.303764 | 1.36E-20 | 8.55E-19 | Up |
| TUSC3 | 1710.288586 | 3.027406557 | 0.203399 | 14.88405 | 4.18E-50 | 5.82E-47 | Up |
| FGL1 | 3.641119694 | 2.7379528 | 0.428128 | 6.395174 | 1.60E-10 | 2.58E-09 | Up |
| KCNN4 | 1090.589964 | 3.123247241 | 0.405064 | 7.710504 | 1.25E-14 | 3.77E-13 | Up |
| DHDH | 31.95661869 | 2.450990583 | 0.31645 | 7.745265 | 9.54E-15 | 2.92E-13 | Up |
| LHB | 3.212556831 | 1.617605871 | 0.378399 | 4.274872 | 1.91E-05 | 0.000115 | Up |
| SLC17A7 | 125.3573124 | -1.230404964 | 0.172587 | -7.12919 | 1.01E-12 | 2.34E-11 | Down |
| KLC3 | 73.3761909 | 1.336146873 | 0.206097 | 6.483087 | 8.99E-11 | 1.51E-09 | Up |
| RETN | 15.5358544 | 4.718509745 | 0.505314 | 9.337781 | 9.84E-21 | 6.29E-19 | Up |
| FCER2 | 197.1816343 | -1.779658197 | 0.65053 | -2.7357 | 0.006225 | 0.018811 | Down |
| CLEC4M | 2.970188381 | -1.47453106 | 0.428776 | -3.43893 | 0.000584 | 0.002421 | Down |
| ASF1B | 285.7745957 | 1.191290816 | 0.292859 | 4.067795 | 4.75E-05 | 0.000261 | Up |
| EPHX3 | 46.06860207 | 2.466833704 | 0.230659 | 10.69472 | 1.08E-26 | 1.28E-24 | Up |
| LGALS13 | 1.758799372 | 2.837360249 | 0.9244 | 3.069407 | 0.002145 | 0.007503 | Up |
| CNTD2 | 241.9960322 | 1.248994297 | 0.247725 | 5.041856 | 4.61E-07 | 3.94E-06 | Up |
| PLD3 | 18667.97866 | 1.209913876 | 0.099825 | 12.12033 | 8.24E-34 | 1.89E-31 | Up |
| EBI3 | 204.746811 | 1.222725009 | 0.395617 | 3.090679 | 0.001997 | 0.007053 | Up |
| CLIP3 | 679.1360511 | 1.536851521 | 0.176282 | 8.718148 | 2.83E-18 | 1.34E-16 | Up |
| SLC1A5 | 1192.830122 | 1.389558195 | 0.253958 | 5.471596 | 4.46E-08 | 4.65E-07 | Up |
| BBC3 | 477.5026831 | 1.892950524 | 0.19197 | 9.860639 | 6.17E-23 | 4.95E-21 | Up |
| AC011462.1 | 2819.741261 | 1.428892638 | 0.212643 | 6.71968 | 1.82E-11 | 3.43E-10 | Up |
| ICAM5 | 491.5216967 | 3.898785637 | 0.32088 | 12.15027 | 5.72E-34 | 1.32E-31 | Up |
| SULT2A1 | 0.722783376 | 2.165717916 | 0.545621 | 3.969274 | 7.21E-05 | 0.000378 | Up |
| CNFN | 70.51022047 | 1.873843935 | 0.264514 | 7.084114 | 1.40E-12 | 3.16E-11 | Up |
| SIGLEC6 | 99.78523048 | 4.018020475 | 0.59788 | 6.720452 | 1.81E-11 | 3.41E-10 | Up |
| PLEKHA4 | 3934.329086 | 1.299759764 | 0.183777 | 7.07249 | 1.52E-12 | 3.41E-11 | Up |
| MAST1 | 23.28460699 | 1.083242448 | 0.175823 | 6.160965 | 7.23E-10 | 1.03E-08 | Up |
| SLC5A5 | 3536.612752 | -2.200324662 | 0.479822 | -4.58571 | 4.52E-06 | 3.14E-05 | Down |
| ARRDC2 | 3573.331277 | 1.035123201 | 0.138517 | 7.472875 | 7.85E-14 | 2.10E-12 | Up |
| PDE4C | 401.8235056 | 3.066410143 | 0.290062 | 10.57157 | 4.04E-26 | 4.50E-24 | Up |
| ISYNA1 | 2361.541255 | 1.561893097 | 0.129096 | 12.0987 | 1.07E-33 | 2.43E-31 | Up |
| COMP | 1269.983036 | 4.489149515 | 0.408769 | 10.98212 | 4.66E-28 | 6.43E-26 | Up |
| ATP4A | 63.90590869 | -1.239111848 | 0.243884 | -5.08074 | 3.76E-07 | 3.28E-06 | Down |
| MAG | 274.1513803 | 1.663386729 | 0.335576 | 4.956804 | 7.17E-07 | 5.90E-06 | Up |
| TMEM59L | 148.4031194 | 2.973471765 | 0.312369 | 9.519087 | 1.75E-21 | 1.20E-19 | Up |
| HPN | 3250.101616 | 1.716490002 | 0.191812 | 8.948836 | 3.59E-19 | 1.92E-17 | Up |
| SCN1B | 704.9554745 | 2.259718319 | 0.20971 | 10.77546 | 4.50E-27 | 5.70E-25 | Up |
| PBX4 | 1068.549864 | -1.880780765 | 0.165482 | -11.3655 | 6.21E-30 | 1.05E-27 | Down |
| ETHE1 | 1768.969641 | 1.319027189 | 0.156813 | 8.411473 | 4.05E-17 | 1.66E-15 | Up |
| DLX5 | 53.49223611 | 1.23795212 | 0.197837 | 6.257428 | 3.91E-10 | 5.87E-09 | Up |
| MET | 12216.57433 | 2.37652111 | 0.188447 | 12.61111 | 1.83E-36 | 5.42E-34 | Up |
| HOXA13 | 1.645130561 | 2.087980291 | 0.352639 | 5.92101 | 3.20E-09 | 4.10E-08 | Up |
| EVX1 | 3.801797492 | 1.224937771 | 0.336853 | 3.636417 | 0.000276 | 0.00125 | Up |
| NOD1 | 2059.958499 | 2.438395306 | 0.21468 | 11.35828 | 6.75E-30 | 1.13E-27 | Up |
| CRHR2 | 57.89614204 | 1.705701655 | 0.237664 | 7.176957 | 7.13E-13 | 1.69E-11 | Up |
| NPTX2 | 59.27357388 | 2.544368273 | 0.290623 | 8.75487 | 2.04E-18 | 9.82E-17 | Up |
| HYAL4 | 3.406574091 | -1.922223202 | 0.561112 | -3.42574 | 0.000613 | 0.002528 | Down |
| FSCN3 | 5.191088599 | -1.110185264 | 0.2882 | -3.85213 | 0.000117 | 0.000583 | Down |
| AGR2 | 1263.258277 | 3.141678507 | 0.325761 | 9.644108 | 5.21E-22 | 3.80E-20 | Up |
| LIMK1 | 3263.465846 | 1.117599618 | 0.106209 | 10.52267 | 6.79E-26 | 7.45E-24 | Up |
| CNTNAP3 | 90.45048545 | -1.267789275 | 0.250503 | -5.06098 | 4.17E-07 | 3.60E-06 | Down |
| MEGF9 | 6892.37246 | 1.054787792 | 0.134915 | 7.81815 | 5.36E-15 | 1.70E-13 | Up |
| CORO2A | 812.5177463 | 1.823707212 | 0.144667 | 12.60624 | 1.95E-36 | 5.72E-34 | Up |
| TGFBR1 | 3966.972636 | 1.615993768 | 0.140535 | 11.49885 | 1.34E-30 | 2.40E-28 | Up |
| OGN | 1154.193252 | -1.558509442 | 0.301609 | -5.16732 | 2.37E-07 | 2.16E-06 | Down |
| ASPN | 1232.126028 | 1.638267297 | 0.28727 | 5.702875 | 1.18E-08 | 1.36E-07 | Up |
| TLE4 | 1558.503556 | -1.37989152 | 0.135102 | -10.2137 | 1.72E-24 | 1.64E-22 | Down |
| SUSD1 | 656.6001651 | 1.01975351 | 0.131764 | 7.739225 | 1.00E-14 | 3.05E-13 | Up |
| AMBP | 32.48932095 | -1.101600361 | 0.284406 | -3.87334 | 0.000107 | 0.000539 | Down |
| ENG | 10566.45964 | 1.254412183 | 0.144527 | 8.679409 | 3.98E-18 | 1.84E-16 | Up |
| AK1 | 1595.601843 | 1.481033336 | 0.147008 | 10.07454 | 7.16E-24 | 6.47E-22 | Up |
| NPDC1 | 3040.96496 | 1.616979908 | 0.180877 | 8.939685 | 3.90E-19 | 2.08E-17 | Up |
| SH3GL2 | 201.1209193 | -2.402374457 | 0.319078 | -7.52911 | 5.11E-14 | 1.40E-12 | Down |
| UNC5B | 1720.080462 | 2.006930237 | 0.166979 | 12.01907 | 2.82E-33 | 6.16E-31 | Up |
| SPOCK2 | 9053.262426 | 2.281105624 | 0.30697 | 7.431026 | 1.08E-13 | 2.83E-12 | Up |
| FGF8 | 1.130777282 | -1.449374659 | 0.588404 | -2.46323 | 0.013769 | 0.03674 | Down |
| PITX3 | 6.225976651 | 3.369397974 | 0.419416 | 8.033551 | 9.47E-16 | 3.28E-14 | Up |
| NEURL1 | 29.44837263 | 1.475620558 | 0.247521 | 5.961608 | 2.50E-09 | 3.26E-08 | Up |
| DKK1 | 21.39999881 | 1.314485296 | 0.29556 | 4.447441 | 8.69E-06 | 5.67E-05 | Up |
| LGI1 | 9.938517184 | -2.211658591 | 0.290477 | -7.61389 | 2.66E-14 | 7.58E-13 | Down |
| CSF3 | 141.8848405 | -1.755604623 | 0.663795 | -2.6448 | 0.008174 | 0.023734 | Down |
| WNT3 | 278.8546998 | 1.208018055 | 0.178881 | 6.753184 | 1.45E-11 | 2.77E-10 | Up |
| HOXB6 | 34.4562637 | 1.281304012 | 0.228485 | 5.607816 | 2.05E-08 | 2.26E-07 | Up |
| ASIC2 | 3.081047689 | 1.378092151 | 0.367573 | 3.749168 | 0.000177 | 0.000843 | Up |
| CCL1 | 1.02809757 | 2.160424468 | 0.660917 | 3.268827 | 0.00108 | 0.004136 | Up |
| CNTNAP1 | 368.9061954 | 1.058830199 | 0.156077 | 6.784031 | 1.17E-11 | 2.27E-10 | Up |
| COL1A1 | 38670.02042 | 3.14718935 | 0.35067 | 8.974785 | 2.84E-19 | 1.53E-17 | Up |
| ABCC3 | 2477.505675 | 2.416676604 | 0.250945 | 9.630318 | 5.95E-22 | 4.32E-20 | Up |
| PPY | 40.25751621 | -1.454853024 | 0.476429 | -3.05366 | 0.002261 | 0.007848 | Down |
| HLF | 3080.276701 | -1.237177789 | 0.206461 | -5.9923 | 2.07E-09 | 2.74E-08 | Down |
| EFNB3 | 90.0987655 | 1.172666104 | 0.206204 | 5.686923 | 1.29E-08 | 1.48E-07 | Up |
| FAM20A | 2698.754631 | 2.4316778 | 0.270383 | 8.993462 | 2.40E-19 | 1.30E-17 | Up |
| FOXN1 | 52.67328934 | -1.643831551 | 0.243605 | -6.74794 | 1.50E-11 | 2.87E-10 | Down |
| CWH43 | 1290.710902 | -1.872019007 | 0.273824 | -6.83658 | 8.11E-12 | 1.62E-10 | Down |
| ODAM | 170.9515274 | -2.36263626 | 0.291329 | -8.10986 | 5.07E-16 | 1.81E-14 | Down |
| SMR3A | 23.16344126 | -1.129919057 | 0.365877 | -3.08825 | 0.002013 | 0.007099 | Down |
| NMU | 300.8277591 | 4.876438447 | 0.487271 | 10.00765 | 1.41E-23 | 1.24E-21 | Up |
| AREG | 203.6863773 | 1.00660279 | 0.415177 | 2.424518 | 0.015329 | 0.040182 | Up |
| IL2 | 4.256840355 | -1.137190427 | 0.375068 | -3.03196 | 0.00243 | 0.008367 | Down |
| GALNT7 | 2926.148588 | 2.097679125 | 0.125851 | 16.66795 | 2.24E-62 | 1.68E-58 | Up |
| NKX3-2 | 30.64472713 | 2.087425052 | 0.358279 | 5.826262 | 5.67E-09 | 6.91E-08 | Up |
| GLRB | 181.1568875 | 1.945095793 | 0.196936 | 9.876799 | 5.25E-23 | 4.27E-21 | Up |
| SNX25 | 3023.615422 | 1.45151369 | 0.124576 | 11.65161 | 2.25E-31 | 4.38E-29 | Up |
| PPARGC1A | 4419.089033 | -1.426819787 | 0.259719 | -5.4937 | 3.94E-08 | 4.13E-07 | Down |
| DDX25 | 344.2658207 | -1.674286081 | 0.175777 | -9.52506 | 1.65E-21 | 1.13E-19 | Down |
| CRYAB | 20269.56632 | -1.169308836 | 0.195182 | -5.99086 | 2.09E-09 | 2.76E-08 | Down |
| CTSC | 9567.550711 | 2.15024971 | 0.198861 | 10.81284 | 2.99E-27 | 3.82E-25 | Up |
| ZBTB16 | 2088.008856 | -2.01800673 | 0.28102 | -7.181 | 6.92E-13 | 1.64E-11 | Down |
| B3GAT1 | 1309.103247 | 1.717649726 | 0.182571 | 9.408105 | 5.05E-21 | 3.34E-19 | Up |
| DTX4 | 9575.329086 | 3.037403855 | 0.219838 | 13.81654 | 2.03E-43 | 1.31E-40 | Up |
| CCND1 | 15804.71031 | 1.550096845 | 0.128713 | 12.04306 | 2.11E-33 | 4.66E-31 | Up |
| FOLR3 | 50.2168644 | -1.286561605 | 0.295201 | -4.35825 | 1.31E-05 | 8.20E-05 | Down |
| UPK2 | 9.172659978 | 1.930478225 | 0.288798 | 6.684535 | 2.32E-11 | 4.30E-10 | Up |
| KIAA1549L | 47.00103885 | 1.866894479 | 0.275015 | 6.788341 | 1.13E-11 | 2.21E-10 | Up |
| SCGB2A2 | 0.670074771 | -1.639693146 | 0.659006 | -2.48813 | 0.012842 | 0.034657 | Down |
| MDK | 5350.376326 | 2.580830814 | 0.23403 | 11.02779 | 2.81E-28 | 3.97E-26 | Up |
| SLC35F2 | 1281.530881 | 1.543394607 | 0.122651 | 12.58365 | 2.60E-36 | 7.38E-34 | Up |
| P3H3 | 2267.175443 | 1.073056742 | 0.118899 | 9.024914 | 1.80E-19 | 1.00E-17 | Up |
| GLI1 | 111.0734888 | -1.679923551 | 0.226792 | -7.40733 | 1.29E-13 | 3.34E-12 | Down |
| PRMT8 | 111.2758102 | 4.300659068 | 0.396092 | 10.85772 | 1.83E-27 | 2.39E-25 | Up |
| MYL2 | 8.302920509 | -2.501180499 | 0.45317 | -5.5193 | 3.40E-08 | 3.61E-07 | Down |
| CUX2 | 798.2167805 | -1.423238553 | 0.416132 | -3.42016 | 0.000626 | 0.002573 | Down |
| KCNA1 | 214.5883976 | -3.374862685 | 0.38097 | -8.8586 | 8.10E-19 | 4.12E-17 | Down |
| ART4 | 254.8088662 | -1.52856706 | 0.209011 | -7.31334 | 2.61E-13 | 6.52E-12 | Down |
| RERGL | 172.1620076 | -1.350625315 | 0.279875 | -4.82581 | 1.39E-06 | 1.08E-05 | Down |
| CHPT1 | 5566.063946 | 1.16734554 | 0.110136 | 10.59911 | 3.01E-26 | 3.42E-24 | Up |
| GYS2 | 14.51709142 | -1.277435799 | 0.246883 | -5.17425 | 2.29E-07 | 2.09E-06 | Down |
| AICDA | 70.70606384 | -2.681710865 | 0.682605 | -3.92864 | 8.54E-05 | 0.000439 | Down |
| RFX4 | 26.64683386 | -1.239163743 | 0.244283 | -5.07265 | 3.92E-07 | 3.40E-06 | Down |
| RSPH4A | 118.0272856 | -1.224032674 | 0.200396 | -6.10808 | 1.01E-09 | 1.40E-08 | Down |
| MAK | 94.74140555 | -1.007232079 | 0.205697 | -4.89668 | 9.75E-07 | 7.80E-06 | Down |
| ADTRP | 277.5294354 | 2.9724907 | 0.319707 | 9.297543 | 1.44E-20 | 9.03E-19 | Up |
| TPD52L1 | 4732.146848 | 1.729505072 | 0.139291 | 12.41651 | 2.13E-35 | 5.39E-33 | Up |
| ULBP1 | 76.71135514 | 2.184540201 | 0.263774 | 8.281865 | 1.21E-16 | 4.76E-15 | Up |
| OPRM1 | 0.648544241 | -1.296949598 | 0.529241 | -2.45058 | 0.014262 | 0.037847 | Down |
| SLC26A8 | 84.54275371 | 1.592749176 | 0.28086 | 5.670962 | 1.42E-08 | 1.61E-07 | Up |
| RHAG | 1.607875001 | -2.387260513 | 0.495109 | -4.82169 | 1.42E-06 | 1.10E-05 | Down |
| GLP1R | 87.84064227 | 2.348804222 | 0.367916 | 6.384081 | 1.72E-10 | 2.75E-09 | Up |
| RBM24 | 107.0766854 | -1.791243909 | 0.216624 | -8.26892 | 1.35E-16 | 5.25E-15 | Down |
| KHDRBS2 | 795.2419488 | -1.123711346 | 0.256716 | -4.37726 | 1.20E-05 | 7.59E-05 | Down |
| COL9A1 | 40.79733264 | 1.026229332 | 0.236932 | 4.331329 | 1.48E-05 | 9.15E-05 | Up |
| VNN2 | 191.984138 | -1.052453816 | 0.441425 | -2.38422 | 0.017115 | 0.043998 | Down |
| PERP | 7976.016685 | 1.394045305 | 0.136652 | 10.20141 | 1.95E-24 | 1.85E-22 | Up |
| C6orf118 | 21.03470561 | -2.547045158 | 0.302224 | -8.42767 | 3.53E-17 | 1.45E-15 | Down |
| MDFI | 255.7381732 | 2.322460694 | 0.220886 | 10.51429 | 7.42E-26 | 8.12E-24 | Up |
| SMOC2 | 8576.831325 | -1.822810262 | 0.244205 | -7.46426 | 8.38E-14 | 2.24E-12 | Down |
| SRF | 8856.582067 | -1.355051103 | 0.226712 | -5.97696 | 2.27E-09 | 2.98E-08 | Down |
| PCDHB2 | 156.4806509 | 1.364220401 | 0.246713 | 5.529595 | 3.21E-08 | 3.42E-07 | Up |
| GHR | 589.2027077 | -1.467426612 | 0.168745 | -8.69612 | 3.43E-18 | 1.60E-16 | Down |
| SPARC | 51866.57085 | 1.132606638 | 0.115381 | 9.816194 | 9.59E-23 | 7.54E-21 | Up |
| THBS4 | 1079.646224 | 2.021503427 | 0.306303 | 6.59969 | 4.12E-11 | 7.33E-10 | Up |
| BTNL8 | 31.04515177 | -1.152110624 | 0.236105 | -4.87964 | 1.06E-06 | 8.44E-06 | Down |
| CDH6 | 1067.80207 | 2.936080049 | 0.207862 | 14.12513 | 2.66E-45 | 2.08E-42 | Up |
| NPR3 | 2223.237559 | -1.236841157 | 0.25571 | -4.83689 | 1.32E-06 | 1.02E-05 | Down |
| SLC27A6 | 1400.837882 | 3.104192777 | 0.418056 | 7.425302 | 1.13E-13 | 2.95E-12 | Up |
| ST8SIA4 | 897.787214 | 1.329000938 | 0.246006 | 5.402309 | 6.58E-08 | 6.65E-07 | Up |
| PCDH12 | 1306.875193 | 1.167336902 | 0.13147 | 8.879127 | 6.74E-19 | 3.46E-17 | Up |
| FGF1 | 557.4284527 | 1.425998247 | 0.161386 | 8.835943 | 9.93E-19 | 4.93E-17 | Up |
| LIFR | 7710.293749 | -1.41138393 | 0.208688 | -6.76312 | 1.35E-11 | 2.60E-10 | Down |
| DPYSL3 | 7668.92258 | 1.147087054 | 0.20519 | 5.590373 | 2.27E-08 | 2.48E-07 | Up |
| CNTN3 | 262.8689371 | -1.270785309 | 0.23947 | -5.30665 | 1.12E-07 | 1.08E-06 | Down |
| KNG1 | 2.525706611 | 1.770713974 | 0.383695 | 4.614899 | 3.93E-06 | 2.76E-05 | Up |
| HRG | 1.717123483 | 2.65001064 | 0.481414 | 5.504644 | 3.70E-08 | 3.90E-07 | Up |
| CLDN16 | 1632.334406 | 4.64359994 | 0.387485 | 11.98394 | 4.31E-33 | 9.36E-31 | Up |
| SERPINI2 | 2.66467806 | -1.513966727 | 0.3986 | -3.79821 | 0.000146 | 0.000709 | Down |
| COL7A1 | 301.172977 | 2.222498422 | 0.29939 | 7.423416 | 1.14E-13 | 2.99E-12 | Up |
| ROPN1B | 56.45859714 | -1.170079687 | 0.183451 | -6.37817 | 1.79E-10 | 2.85E-09 | Down |
| CSPG5 | 56.79694487 | 1.121870858 | 0.161329 | 6.953932 | 3.55E-12 | 7.54E-11 | Up |
| PLSCR4 | 3164.96087 | -1.072337044 | 0.13297 | -8.06452 | 7.35E-16 | 2.57E-14 | Down |
| PEX5L | 33.0269352 | -1.001561567 | 0.181213 | -5.52699 | 3.26E-08 | 3.47E-07 | Down |
| AADAC | 9.123904121 | -2.009561565 | 0.420074 | -4.78383 | 1.72E-06 | 1.30E-05 | Down |
| PLCH1 | 1322.307309 | -1.70495501 | 0.215729 | -7.90321 | 2.72E-15 | 8.89E-14 | Down |
| IL1A | 24.61855105 | -1.036283806 | 0.330083 | -3.13946 | 0.001693 | 0.006111 | Down |
| CCL20 | 53.31197196 | 2.630573948 | 0.451572 | 5.825373 | 5.70E-09 | 6.94E-08 | Up |
| STEAP3 | 1163.564691 | 1.001117379 | 0.188032 | 5.324185 | 1.01E-07 | 9.87E-07 | Up |
| TFCP2L1 | 11101.85957 | -2.01222393 | 0.306523 | -6.56467 | 5.21E-11 | 9.16E-10 | Down |
| SLC30A3 | 153.6204866 | 3.483173005 | 0.403934 | 8.623131 | 6.51E-18 | 2.91E-16 | Up |
| FNDC4 | 695.9487491 | 2.173211739 | 0.244503 | 8.888283 | 6.21E-19 | 3.21E-17 | Up |
| EVA1A | 486.1915692 | 2.549893374 | 0.226719 | 11.24694 | 2.40E-29 | 3.80E-27 | Up |
| EFEMP1 | 11922.40056 | -1.313942026 | 0.164857 | -7.97018 | 1.58E-15 | 5.34E-14 | Down |
| FN1 | 244049.2365 | 5.162608748 | 0.352663 | 14.63894 | 1.59E-48 | 1.92E-45 | Up |
| DNAH6 | 556.2925918 | -1.208498061 | 0.148131 | -8.15828 | 3.40E-16 | 1.24E-14 | Down |
| IGFBP2 | 1421.070029 | 1.608958355 | 0.176926 | 9.093941 | 9.55E-20 | 5.51E-18 | Up |
| KCNJ13 | 289.4486461 | -1.2422577 | 0.307344 | -4.04191 | 5.30E-05 | 0.000288 | Down |
| OTX1 | 4.520526188 | -1.180511537 | 0.411238 | -2.87063 | 0.004097 | 0.013153 | Down |
| GNLY | 673.1784501 | 1.971553048 | 0.370181 | 5.32592 | 1.00E-07 | 9.79E-07 | Up |
| ST3GAL5 | 8098.44627 | 1.300514035 | 0.115096 | 11.29937 | 1.32E-29 | 2.15E-27 | Up |
| SLC5A7 | 87.06818946 | -1.399726821 | 0.416007 | -3.36467 | 0.000766 | 0.003067 | Down |
| PASK | 1936.726636 | 1.457459143 | 0.235756 | 6.182079 | 6.33E-10 | 9.07E-09 | Up |
| PROC | 31.99971207 | 1.325401726 | 0.229146 | 5.784089 | 7.29E-09 | 8.70E-08 | Up |
| QPCT | 2159.9143 | 3.495096933 | 0.249878 | 13.98722 | 1.87E-44 | 1.40E-41 | Up |
| KISS1R | 15.00373438 | 3.54727351 | 0.3583 | 9.900285 | 4.15E-23 | 3.42E-21 | Up |
| CD207 | 251.6355776 | 3.33909806 | 0.506376 | 6.594107 | 4.28E-11 | 7.59E-10 | Up |
| VAX2 | 4.882743911 | 1.831665418 | 0.312069 | 5.869422 | 4.37E-09 | 5.44E-08 | Up |
| ATP6V1B1 | 6.486902946 | 1.091000287 | 0.300245 | 3.633701 | 0.000279 | 0.001262 | Up |
| EPHA4 | 4072.369706 | 1.093137522 | 0.204186 | 5.353648 | 8.62E-08 | 8.51E-07 | Up |
| PAPPA2 | 99.38823671 | -1.375816213 | 0.253027 | -5.43743 | 5.41E-08 | 5.55E-07 | Down |
| ANGPTL1 | 3334.906542 | -1.158096346 | 0.181685 | -6.37419 | 1.84E-10 | 2.92E-09 | Down |
| QSOX1 | 5468.142921 | 1.410051587 | 0.13148 | 10.72447 | 7.81E-27 | 9.43E-25 | Up |
| KIAA1324 | 2450.349364 | -1.778019887 | 0.280569 | -6.33719 | 2.34E-10 | 3.66E-09 | Down |
| FBXO2 | 1320.978547 | 1.437041131 | 0.189244 | 7.593587 | 3.11E-14 | 8.77E-13 | Up |
| MAD2L2 | 1197.122265 | 1.195053614 | 0.168376 | 7.097545 | 1.27E-12 | 2.90E-11 | Up |
| NCF2 | 638.9790133 | 1.004746396 | 0.293148 | 3.427438 | 0.000609 | 0.002516 | Up |
| CTH | 596.6225882 | -1.36045409 | 0.188275 | -7.22588 | 4.98E-13 | 1.20E-11 | Down |
| NID1 | 2211.43489 | 1.272095269 | 0.135739 | 9.371607 | 7.14E-21 | 4.66E-19 | Up |
| NT5C1A | 9.656812398 | 3.703755392 | 0.359083 | 10.31448 | 6.06E-25 | 6.03E-23 | Up |
| HPCAL4 | 333.5264633 | 4.657349522 | 0.309505 | 15.04775 | 3.57E-51 | 5.16E-48 | Up |
| SIPA1L2 | 2251.658618 | 1.276901179 | 0.130321 | 9.798158 | 1.15E-22 | 8.96E-21 | Up |
| ST6GALNAC5 | 192.4907364 | 4.426638567 | 0.396588 | 11.16182 | 6.27E-29 | 9.53E-27 | Up |
| MFAP2 | 527.2743601 | 2.279325783 | 0.235231 | 9.68973 | 3.33E-22 | 2.49E-20 | Up |
| RGS4 | 327.4739971 | 1.414126383 | 0.332446 | 4.253705 | 2.10E-05 | 0.000125 | Up |
| IGSF21 | 76.4157732 | 1.257536863 | 0.279566 | 4.498175 | 6.85E-06 | 4.57E-05 | Up |
| GBP3 | 2808.837204 | 1.136100575 | 0.230212 | 4.935017 | 8.01E-07 | 6.53E-06 | Up |
| GBP1 | 9776.193607 | -1.310882549 | 0.310862 | -4.21693 | 2.48E-05 | 0.000145 | Down |
| ECE1 | 19712.30255 | 1.4382494 | 0.108792 | 13.22018 | 6.71E-40 | 2.96E-37 | Up |
| GALE | 1774.679251 | 2.990637263 | 0.17542 | 17.04843 | 3.59E-65 | 3.37E-61 | Up |
| CR2 | 2096.113449 | -1.75393719 | 0.640866 | -2.73682 | 0.006204 | 0.018758 | Down |
| MPL | 37.58229218 | -1.049731887 | 0.155376 | -6.75609 | 1.42E-11 | 2.73E-10 | Down |
| TNFSF4 | 141.9097995 | 1.281788858 | 0.240312 | 5.333861 | 9.61E-08 | 9.41E-07 | Up |
| PLPPR5 | 1.729966145 | 3.249629893 | 0.73463 | 4.42349 | 9.71E-06 | 6.25E-05 | Up |
| PROX1 | 1809.823572 | -1.309736611 | 0.213408 | -6.13724 | 8.40E-10 | 1.18E-08 | Down |
| CHRNB4 | 28.1845366 | 1.491637615 | 0.23997 | 6.215942 | 5.10E-10 | 7.46E-09 | Up |
| A4GNT | 5.429597243 | -1.100271735 | 0.414838 | -2.65229 | 0.007995 | 0.023293 | Down |
| MMP8 | 1.815242908 | 2.021492959 | 0.559068 | 3.615827 | 0.000299 | 0.001343 | Up |
| SLC8A2 | 8.686520391 | 1.073183269 | 0.356943 | 3.006598 | 0.002642 | 0.008992 | Up |
| TNNT2 | 9.962428404 | 1.09195518 | 0.33005 | 3.308455 | 0.000938 | 0.003663 | Up |
| TNP1 | 9.853057962 | -1.888624948 | 0.366836 | -5.14842 | 2.63E-07 | 2.36E-06 | Down |
| NRP2 | 5672.367418 | 2.159243189 | 0.172675 | 12.50467 | 7.04E-36 | 1.90E-33 | Up |
| B4GALT6 | 346.07042 | 1.366641632 | 0.155077 | 8.812671 | 1.22E-18 | 6.04E-17 | Up |
| ATP10B | 92.85045264 | 2.989908808 | 0.350181 | 8.53817 | 1.36E-17 | 5.86E-16 | Up |
| CCN2 | 57944.31304 | -1.221548307 | 0.308853 | -3.95511 | 7.65E-05 | 0.000398 | Down |
| OLFM3 | 33.65828807 | -2.007883806 | 0.374015 | -5.36846 | 7.94E-08 | 7.89E-07 | Down |
| SPP1 | 1804.781413 | 1.04752717 | 0.367653 | 2.849225 | 0.004383 | 0.013944 | Up |
| PCDH17 | 735.527674 | 1.344645035 | 0.18144 | 7.410961 | 1.25E-13 | 3.26E-12 | Up |
| CCND2 | 13474.09716 | 1.234844018 | 0.125404 | 9.84693 | 7.07E-23 | 5.63E-21 | Up |
| FGF23 | 1.21337404 | -2.728372625 | 0.679834 | -4.01329 | 5.99E-05 | 0.00032 | Down |
| DNAH7 | 690.0849973 | -1.146179121 | 0.149664 | -7.65834 | 1.88E-14 | 5.52E-13 | Down |
| LCN1P1 | 8.640916643 | 1.015940567 | 0.27878 | 3.644244 | 0.000268 | 0.001217 | Up |
| NR4A3 | 3762.130692 | -1.784261725 | 0.435813 | -4.0941 | 4.24E-05 | 0.000235 | Down |
| NPC2 | 59679.46688 | 2.414464934 | 0.177488 | 13.60353 | 3.82E-42 | 2.11E-39 | Up |
| DPPA5P4 | 4.890251606 | -2.333789276 | 0.446676 | -5.2248 | 1.74E-07 | 1.62E-06 | Down |
| GPR68 | 248.0512659 | 1.022283874 | 0.290384 | 3.520459 | 0.000431 | 0.001852 | Up |
| BCL11A | 468.2266244 | -1.153838021 | 0.204644 | -5.63826 | 1.72E-08 | 1.92E-07 | Down |
| CNNM1 | 23.1387013 | -1.919874889 | 0.290001 | -6.62025 | 3.59E-11 | 6.44E-10 | Down |
| C10orf95 | 151.0313207 | 1.068601925 | 0.150031 | 7.122549 | 1.06E-12 | 2.45E-11 | Up |
| IFNA8 | 0.524555398 | 1.737606802 | 0.68231 | 2.546655 | 0.010876 | 0.030143 | Up |
| GRIA2 | 6.398883035 | 1.747330589 | 0.510656 | 3.421737 | 0.000622 | 0.002559 | Up |
| MTHFD1L | 1158.849542 | 1.115931881 | 0.123525 | 9.03409 | 1.65E-19 | 9.28E-18 | Up |
| TNFSF18 | 46.27344677 | 3.325333646 | 0.357102 | 9.312012 | 1.25E-20 | 7.98E-19 | Up |
| KCNJ5 | 565.8130015 | 1.060474304 | 0.284197 | 3.731473 | 0.00019 | 0.000896 | Up |
| TP53AIP1 | 5.597045676 | -1.318719539 | 0.285684 | -4.616 | 3.91E-06 | 2.75E-05 | Down |
| TEX11 | 1.660445996 | 1.079267778 | 0.401284 | 2.689539 | 0.007155 | 0.021206 | Up |
| IQSEC3 | 135.708632 | 1.512606665 | 0.245889 | 6.151576 | 7.67E-10 | 1.09E-08 | Up |
| SMAD9 | 5618.700603 | -1.307462999 | 0.186046 | -7.02763 | 2.10E-12 | 4.59E-11 | Down |
| TGFBI | 3475.68702 | 1.626231493 | 0.225717 | 7.204725 | 5.82E-13 | 1.39E-11 | Up |
| MYOT | 29.34518017 | -1.54879232 | 0.222949 | -6.94683 | 3.74E-12 | 7.88E-11 | Down |
| EGR1 | 116773.33 | -1.540384449 | 0.272554 | -5.65167 | 1.59E-08 | 1.79E-07 | Down |
| DUSP4 | 1735.569938 | 2.344035434 | 0.301604 | 7.7719 | 7.73E-15 | 2.40E-13 | Up |
| CLU | 248165.7626 | 1.418268585 | 0.182604 | 7.7669 | 8.04E-15 | 2.49E-13 | Up |
| PTK2B | 4869.813603 | 1.155587182 | 0.149252 | 7.742532 | 9.75E-15 | 2.98E-13 | Up |
| TBX2 | 1030.097643 | 1.047179529 | 0.240119 | 4.361081 | 1.29E-05 | 8.11E-05 | Up |
| ABCC11 | 94.96987604 | 4.091983338 | 0.313215 | 13.06447 | 5.25E-39 | 2.08E-36 | Up |
| HCRTR1 | 23.0162264 | -1.020364014 | 0.185869 | -5.4897 | 4.03E-08 | 4.22E-07 | Down |
| SLITRK3 | 20.05429999 | -1.548749005 | 0.354405 | -4.36999 | 1.24E-05 | 7.82E-05 | Down |
| CSMD2 | 55.63391485 | 1.209249702 | 0.184974 | 6.53741 | 6.26E-11 | 1.08E-09 | Up |
| TMIGD3 | 189.7466909 | 1.072215914 | 0.291416 | 3.679334 | 0.000234 | 0.001075 | Up |
| TBX22 | 168.5884624 | -1.582184891 | 0.400272 | -3.95278 | 7.72E-05 | 0.000402 | Down |
| OPN4 | 9.708259841 | 1.146468976 | 0.443889 | 2.582783 | 0.009801 | 0.027656 | Up |
| PTGFR | 48.09583689 | -1.497859272 | 0.335605 | -4.46316 | 8.08E-06 | 5.30E-05 | Down |
| NPY | 27.82039105 | 1.329981379 | 0.36498 | 3.643981 | 0.000268 | 0.001218 | Up |
| INHBA | 719.3926765 | 1.01865102 | 0.318936 | 3.193903 | 0.001404 | 0.005203 | Up |
| PHF24 | 53.09037414 | -1.279847017 | 0.215557 | -5.93741 | 2.90E-09 | 3.73E-08 | Down |
| CNTFR | 292.2527234 | -1.00239959 | 0.345733 | -2.89935 | 0.003739 | 0.012152 | Down |
| SFTPA1 | 136.7908716 | 5.022782545 | 0.53356 | 9.413712 | 4.79E-21 | 3.18E-19 | Up |
| PLAU | 4656.912203 | 3.269341016 | 0.29738 | 10.9938 | 4.09E-28 | 5.69E-26 | Up |
| EGR2 | 16249.51453 | -1.86627888 | 0.323552 | -5.76809 | 8.02E-09 | 9.50E-08 | Down |
| CDKN2C | 810.6980705 | 1.075755475 | 0.185918 | 5.786169 | 7.20E-09 | 8.61E-08 | Up |
| BHLHE41 | 11150.9747 | 1.052601078 | 0.14502 | 7.258328 | 3.92E-13 | 9.59E-12 | Up |
| NECAB1 | 493.4576367 | -1.099725921 | 0.209671 | -5.24502 | 1.56E-07 | 1.47E-06 | Down |
| CENPK | 94.18647447 | 1.050349912 | 0.254071 | 4.13408 | 3.56E-05 | 0.000201 | Up |
| NR4A1 | 29278.08059 | -1.079628282 | 0.321922 | -3.35369 | 0.000797 | 0.003179 | Down |
| COL10A1 | 488.3540052 | 4.267499902 | 0.343132 | 12.43691 | 1.65E-35 | 4.26E-33 | Up |
| TNFAIP6 | 76.50967514 | 2.003003125 | 0.362057 | 5.532285 | 3.16E-08 | 3.37E-07 | Up |
| G0S2 | 791.1962809 | 2.646809974 | 0.338252 | 7.824955 | 5.08E-15 | 1.61E-13 | Up |
| KCNJ2 | 911.7307892 | 3.872386551 | 0.267961 | 14.45133 | 2.46E-47 | 2.49E-44 | Up |
| PFKFB2 | 9516.289077 | -1.096713715 | 0.157597 | -6.95896 | 3.43E-12 | 7.29E-11 | Down |
| C4BPA | 11.73479483 | -1.016129353 | 0.339577 | -2.99234 | 0.002768 | 0.009362 | Down |
| GPR83 | 697.7724467 | -1.386556828 | 0.250192 | -5.54197 | 2.99E-08 | 3.21E-07 | Down |
| DAW1 | 11.0770312 | 1.548832431 | 0.321625 | 4.815641 | 1.47E-06 | 1.13E-05 | Up |
| SLPI | 1905.409874 | 2.891222296 | 0.321912 | 8.9814 | 2.67E-19 | 1.45E-17 | Up |
| SLC12A5 | 40.88995801 | 2.137179337 | 0.281829 | 7.583249 | 3.37E-14 | 9.45E-13 | Up |
| ARHGAP40 | 61.52527131 | 1.339297496 | 0.424649 | 3.153894 | 0.001611 | 0.005861 | Up |
| SDC4 | 25253.10335 | 2.130950007 | 0.195101 | 10.92231 | 9.02E-28 | 1.22E-25 | Up |
| MATN4 | 21.53791723 | -1.117526586 | 0.235284 | -4.74968 | 2.04E-06 | 1.52E-05 | Down |
| ZNF831 | 233.7553459 | -1.088436423 | 0.364216 | -2.98844 | 0.002804 | 0.009466 | Down |
| SNAI1 | 820.5297557 | -1.124140409 | 0.169483 | -6.63275 | 3.29E-11 | 5.96E-10 | Down |
| PMEPA1 | 2636.994748 | 1.277223838 | 0.128864 | 9.911401 | 3.71E-23 | 3.07E-21 | Up |
| KCNK15 | 361.5966894 | 1.247320511 | 0.192565 | 6.477391 | 9.33E-11 | 1.56E-09 | Up |
| CHST8 | 28.18224811 | -1.082036091 | 0.263369 | -4.10844 | 3.98E-05 | 0.000222 | Down |
| IL17C | 3.16778483 | 1.233133638 | 0.330225 | 3.734227 | 0.000188 | 0.000887 | Up |
| POF1B | 56.63268374 | -1.218115009 | 0.193772 | -6.28632 | 3.25E-10 | 4.94E-09 | Down |
| PSG8 | 63.66295948 | 4.980676763 | 0.748098 | 6.657786 | 2.78E-11 | 5.09E-10 | Up |
| CEACAM8 | 2.743903351 | 3.854806124 | 0.608746 | 6.332373 | 2.41E-10 | 3.77E-09 | Up |
| CRISP2 | 2.314547277 | -1.583649584 | 0.437124 | -3.62288 | 0.000291 | 0.001311 | Down |
| GRM4 | 175.4444686 | 5.187820122 | 0.373065 | 13.90595 | 5.83E-44 | 4.13E-41 | Up |
| SLC17A3 | 1.87704932 | -1.137670487 | 0.477847 | -2.38083 | 0.017274 | 0.044321 | Down |
| SLC17A1 | 1.67655692 | 1.610373837 | 0.555465 | 2.899144 | 0.003742 | 0.012157 | Up |
| HIST1H1D | 6.42969934 | 1.085539968 | 0.373912 | 2.9032 | 0.003694 | 0.012017 | Up |
| UNC5CL | 205.0090844 | 2.59949019 | 0.221037 | 11.76044 | 6.24E-32 | 1.26E-29 | Up |
| SPDEF | 8.361336169 | 2.791597117 | 0.315823 | 8.839107 | 9.65E-19 | 4.81E-17 | Up |
| TREM1 | 250.7487295 | 2.497887915 | 0.353233 | 7.071497 | 1.53E-12 | 3.43E-11 | Up |
| SOX4 | 4705.646849 | 1.361568903 | 0.158508 | 8.589894 | 8.70E-18 | 3.83E-16 | Up |
| RUNX2 | 605.2605822 | 2.147484502 | 0.236898 | 9.065011 | 1.25E-19 | 7.07E-18 | Up |
| OPN5 | 1.418794763 | 2.637549557 | 0.45524 | 5.793752 | 6.88E-09 | 8.25E-08 | Up |
| CXCL6 | 2.012099088 | 2.584065168 | 0.530758 | 4.868635 | 1.12E-06 | 8.87E-06 | Up |
| EREG | 36.10560033 | 2.960657699 | 0.549743 | 5.385526 | 7.22E-08 | 7.24E-07 | Up |
| MYRF | 275.4649548 | 2.588394052 | 0.294059 | 8.80229 | 1.34E-18 | 6.58E-17 | Up |
| SCGB1D2 | 9.998237821 | -1.616059662 | 0.28649 | -5.6409 | 1.69E-08 | 1.89E-07 | Down |
| PIWIL1 | 341.6549064 | 3.397339402 | 0.381375 | 8.908122 | 5.19E-19 | 2.71E-17 | Up |
| GPR18 | 118.4557798 | -1.527728132 | 0.474572 | -3.21917 | 0.001286 | 0.004817 | Down |
| ABCC4 | 1241.067164 | -1.002722632 | 0.181059 | -5.53809 | 3.06E-08 | 3.27E-07 | Down |
| SOX21 | 6.778407895 | -1.267621236 | 0.32085 | -3.95083 | 7.79E-05 | 0.000404 | Down |
| TMEM255A | 133.5550181 | 1.897548984 | 0.285974 | 6.635391 | 3.24E-11 | 5.86E-10 | Up |
| AMELX | 0.634896696 | -1.364769457 | 0.507502 | -2.68919 | 0.007163 | 0.021224 | Down |
| TEKT3 | 40.29056738 | -1.001533502 | 0.13669 | -7.32704 | 2.35E-13 | 5.93E-12 | Down |
| IL37 | 2.437686246 | 4.057356031 | 0.631542 | 6.42452 | 1.32E-10 | 2.16E-09 | Up |
| CD70 | 49.33492599 | 1.545052044 | 0.452219 | 3.416602 | 0.000634 | 0.002603 | Up |
| C3 | 18381.04421 | 1.224920899 | 0.307369 | 3.985179 | 6.74E-05 | 0.000356 | Up |
| FOSB | 73868.83261 | -2.126465958 | 0.3781 | -5.62409 | 1.86E-08 | 2.07E-07 | Down |
| RTN2 | 222.9806304 | 1.237615903 | 0.134821 | 9.179688 | 4.32E-20 | 2.58E-18 | Up |
| TGM3 | 5.81639055 | 1.505014266 | 0.298827 | 5.036409 | 4.74E-07 | 4.04E-06 | Up |
| FOXA2 | 760.3156423 | -1.783647492 | 0.348577 | -5.11695 | 3.11E-07 | 2.75E-06 | Down |
| FAM182A | 20.48698107 | -1.221626638 | 0.315633 | -3.8704 | 0.000109 | 0.000545 | Down |
| PAX1 | 141.43253 | -1.71530256 | 0.411317 | -4.17027 | 3.04E-05 | 0.000174 | Down |
| NKX2-2 | 1.525826714 | -1.676809028 | 0.614436 | -2.72902 | 0.006352 | 0.019144 | Down |
| BMP2 | 5387.947359 | -1.83822647 | 0.278671 | -6.5964 | 4.21E-11 | 7.48E-10 | Down |
| FLRT3 | 1509.093201 | 1.401879244 | 0.231236 | 6.062548 | 1.34E-09 | 1.83E-08 | Up |
| PCSK2 | 13497.91545 | 2.962392494 | 0.465042 | 6.370154 | 1.89E-10 | 2.99E-09 | Up |
| LAMP5 | 398.8498956 | 3.008389267 | 0.373799 | 8.048148 | 8.41E-16 | 2.93E-14 | Up |
| BANF2 | 20.55815025 | -1.734825897 | 0.335251 | -5.17471 | 2.28E-07 | 2.08E-06 | Down |
| TMEM74B | 84.45359522 | 1.548242522 | 0.188893 | 8.196401 | 2.48E-16 | 9.25E-15 | Up |
| CITED1 | 5337.712306 | 4.787079674 | 0.361584 | 13.2392 | 5.21E-40 | 2.36E-37 | Up |
| BPIFB9P | 18.11321501 | 2.780058195 | 0.284297 | 9.778697 | 1.39E-22 | 1.08E-20 | Up |
| F10 | 283.8601697 | -1.565457651 | 0.190005 | -8.23904 | 1.74E-16 | 6.63E-15 | Down |
| FFAR1 | 8.563355534 | 1.874279122 | 0.467354 | 4.010407 | 6.06E-05 | 0.000324 | Up |
| CCR7 | 662.7074456 | -1.320347728 | 0.452601 | -2.91724 | 0.003531 | 0.01156 | Down |
| NR1D1 | 1790.440036 | 1.406197003 | 0.278703 | 5.045497 | 4.52E-07 | 3.87E-06 | Up |
| RRAS | 3786.379116 | 1.023300345 | 0.147795 | 6.923759 | 4.40E-12 | 9.17E-11 | Up |
| FLRT1 | 709.6749596 | -1.258512582 | 0.311576 | -4.03919 | 5.36E-05 | 0.000291 | Down |
| DACH2 | 27.29392213 | 2.995023087 | 0.322245 | 9.294244 | 1.48E-20 | 9.29E-19 | Up |
| SSX1 | 109.8928217 | 2.466598154 | 0.357372 | 6.90204 | 5.13E-12 | 1.06E-10 | Up |
| AIF1L | 17750.40453 | -1.069675077 | 0.163506 | -6.54212 | 6.07E-11 | 1.05E-09 | Down |
| RGS13 | 163.1636972 | -2.255466609 | 0.710408 | -3.17489 | 0.001499 | 0.005509 | Down |
| MASP1 | 152.9267518 | -1.883240054 | 0.235647 | -7.9918 | 1.33E-15 | 4.52E-14 | Down |
| TSPAN8 | 128.7612623 | -1.698103405 | 0.275661 | -6.16011 | 7.27E-10 | 1.03E-08 | Down |
| TRPV5 | 2.214005114 | -1.2019143 | 0.357989 | -3.35741 | 0.000787 | 0.003141 | Down |
| PLA2G5 | 82.58831992 | 1.020707569 | 0.287648 | 3.548458 | 0.000387 | 0.001684 | Up |
| OR7C1 | 1.296976391 | 3.223218634 | 0.505816 | 6.372311 | 1.86E-10 | 2.95E-09 | Up |
| HIP1 | 4469.160342 | 1.136564484 | 0.114506 | 9.925832 | 3.21E-23 | 2.69E-21 | Up |
| STEAP4 | 2966.526385 | 1.157996085 | 0.127669 | 9.070301 | 1.19E-19 | 6.75E-18 | Up |
| SPINK2 | 58.7033703 | 1.619863885 | 0.324158 | 4.997138 | 5.82E-07 | 4.88E-06 | Up |
| VPREB3 | 160.4765558 | -1.719150976 | 0.481821 | -3.56803 | 0.00036 | 0.001578 | Down |
| MGAT3 | 940.6188649 | 3.050187636 | 0.286938 | 10.63014 | 2.16E-26 | 2.49E-24 | Up |
| CDC42EP1 | 2980.254955 | 1.356744472 | 0.128248 | 10.57909 | 3.73E-26 | 4.21E-24 | Up |
| IGLL1 | 7.360128863 | 1.203067078 | 0.494151 | 2.434614 | 0.014908 | 0.039251 | Up |
| KRT17 | 554.4140526 | 3.648426444 | 0.460602 | 7.920996 | 2.36E-15 | 7.80E-14 | Up |
| SPECC1 | 1097.838482 | 1.081966028 | 0.125441 | 8.625285 | 6.39E-18 | 2.86E-16 | Up |
| CDHR3 | 421.3946294 | -2.037446208 | 0.192641 | -10.5764 | 3.83E-26 | 4.32E-24 | Down |
| VGF | 73.37857067 | 2.862238121 | 0.49347 | 5.800233 | 6.62E-09 | 7.95E-08 | Up |
| FOXP2 | 1170.248642 | -1.432649747 | 0.2102 | -6.81564 | 9.38E-12 | 1.86E-10 | Down |
| FLNC | 665.0236124 | -1.363868084 | 0.231267 | -5.89738 | 3.69E-09 | 4.66E-08 | Down |
| LRRC4 | 94.09178029 | 1.362500731 | 0.212814 | 6.402323 | 1.53E-10 | 2.47E-09 | Up |
| IRF5 | 605.5293812 | 1.206486801 | 0.238501 | 5.058622 | 4.22E-07 | 3.64E-06 | Up |
| LRRC17 | 315.9777163 | 1.104570739 | 0.170898 | 6.463344 | 1.02E-10 | 1.70E-09 | Up |
| HOXD1 | 9.188933365 | 3.066551795 | 0.358999 | 8.541957 | 1.32E-17 | 5.67E-16 | Up |
| PDE11A | 370.1192622 | -1.302386319 | 0.23412 | -5.5629 | 2.65E-08 | 2.87E-07 | Down |
| HOXD10 | 5.757099784 | -1.36501151 | 0.369428 | -3.69493 | 0.00022 | 0.001018 | Down |
| ALDH1A2 | 179.9789943 | -1.114715836 | 0.236194 | -4.71949 | 2.36E-06 | 1.74E-05 | Down |
| LOXL1 | 713.2439468 | 1.105939701 | 0.174697 | 6.330614 | 2.44E-10 | 3.80E-09 | Up |
| PIMREG | 34.80475297 | 1.106858381 | 0.249846 | 4.430164 | 9.42E-06 | 6.09E-05 | Up |
| MTUS1 | 5085.721638 | 1.064755261 | 0.108328 | 9.829037 | 8.44E-23 | 6.68E-21 | Up |
| KLK10 | 1197.737881 | 5.483414139 | 0.403184 | 13.60029 | 3.99E-42 | 2.17E-39 | Up |
| KLK8 | 5.995504444 | 2.518366104 | 0.497143 | 5.065682 | 4.07E-07 | 3.52E-06 | Up |
| FOXA1 | 14.50737488 | 1.164193865 | 0.434065 | 2.68207 | 0.007317 | 0.0216 | Up |
| FOXJ1 | 784.1883948 | -2.243353532 | 0.317256 | -7.07112 | 1.54E-12 | 3.44E-11 | Down |
| FGF13 | 425.2953025 | -1.61046451 | 0.184087 | -8.74841 | 2.16E-18 | 1.04E-16 | Down |
| CDH15 | 17.11405213 | 3.041456465 | 0.353506 | 8.603695 | 7.72E-18 | 3.42E-16 | Up |
| PLPPR3 | 6.237098358 | 2.441259691 | 0.350145 | 6.972134 | 3.12E-12 | 6.69E-11 | Up |
| LBP | 32.3448992 | 2.039038572 | 0.526223 | 3.87486 | 0.000107 | 0.000537 | Up |
| TNNI3 | 4.436423933 | 1.57848396 | 0.346776 | 4.551888 | 5.32E-06 | 3.63E-05 | Up |
| PRRG3 | 137.670379 | -1.533433326 | 0.328594 | -4.66665 | 3.06E-06 | 2.21E-05 | Down |
| APOE | 20564.73024 | 2.133357616 | 0.384309 | 5.551148 | 2.84E-08 | 3.06E-07 | Up |
| APOC1 | 3133.034379 | 1.645416244 | 0.412626 | 3.987668 | 6.67E-05 | 0.000353 | Up |
| DPP6 | 4181.871886 | -2.089611288 | 0.331823 | -6.29737 | 3.03E-10 | 4.63E-09 | Down |
| MAS1 | 2.815753495 | -1.432336471 | 0.362747 | -3.94858 | 7.86E-05 | 0.000407 | Down |
| UNC13A | 49.20489852 | 1.252852789 | 0.275994 | 4.539414 | 5.64E-06 | 3.83E-05 | Up |
| KLHDC7B | 329.4502494 | 1.716056937 | 0.448468 | 3.826484 | 0.00013 | 0.00064 | Up |
| PXDN | 1336.429818 | 1.712920715 | 0.169849 | 10.08494 | 6.44E-24 | 5.89E-22 | Up |
| GDF15 | 1774.36814 | 3.547734138 | 0.323131 | 10.97926 | 4.81E-28 | 6.61E-26 | Up |
| JUND | 25618.7736 | -1.134746134 | 0.226684 | -5.00585 | 5.56E-07 | 4.68E-06 | Down |
| SULT4A1 | 5.450306048 | 2.759618842 | 0.424308 | 6.503807 | 7.83E-11 | 1.33E-09 | Up |
| TNNT3 | 119.7122672 | -2.250566264 | 0.215521 | -10.4425 | 1.59E-25 | 1.67E-23 | Down |
| COL5A1 | 3221.336257 | 2.319048692 | 0.279372 | 8.300926 | 1.03E-16 | 4.08E-15 | Up |
| FIBCD1 | 111.7867436 | 4.177217629 | 0.580629 | 7.194291 | 6.28E-13 | 1.50E-11 | Up |
| NPAS1 | 42.90706841 | 1.267114323 | 0.212417 | 5.965209 | 2.44E-09 | 3.19E-08 | Up |
| DUSP9 | 1.381788901 | 2.813998685 | 0.552842 | 5.090059 | 3.58E-07 | 3.14E-06 | Up |
| CASZ1 | 505.1856198 | -1.128797916 | 0.128743 | -8.76786 | 1.82E-18 | 8.78E-17 | Down |
| PKDREJ | 31.02169122 | -1.232991784 | 0.174803 | -7.0536 | 1.74E-12 | 3.86E-11 | Down |
| HSD17B3 | 106.3975901 | -1.279057322 | 0.219946 | -5.81531 | 6.05E-09 | 7.33E-08 | Down |
| FBP2 | 5.904855165 | -3.50887921 | 0.673039 | -5.21349 | 1.85E-07 | 1.72E-06 | Down |
| ULBP2 | 108.8172649 | 2.549967693 | 0.20782 | 12.27011 | 1.31E-34 | 3.13E-32 | Up |
| AKAP12 | 6235.019097 | -1.110850352 | 0.171011 | -6.49577 | 8.26E-11 | 1.40E-09 | Down |
| SYNE1 | 15598.51774 | -1.184723253 | 0.183007 | -6.47366 | 9.57E-11 | 1.60E-09 | Down |
| TTLL9 | 82.75998866 | -1.021128644 | 0.145616 | -7.01247 | 2.34E-12 | 5.09E-11 | Down |
| COX4I2 | 268.8991211 | 1.423140239 | 0.220816 | 6.444904 | 1.16E-10 | 1.91E-09 | Up |
| EDA2R | 554.7588645 | 1.140279697 | 0.131642 | 8.661959 | 4.64E-18 | 2.13E-16 | Up |
| C1QL1 | 82.55053454 | 3.733712391 | 0.322636 | 11.57253 | 5.68E-31 | 1.06E-28 | Up |
| HIGD1B | 113.7326716 | 2.284055769 | 0.244798 | 9.330366 | 1.06E-20 | 6.73E-19 | Up |
| SLC34A1 | 1.203024191 | 1.365953654 | 0.407342 | 3.353334 | 0.000798 | 0.003183 | Up |
| C3orf20 | 14.89379013 | -1.157151201 | 0.216879 | -5.33546 | 9.53E-08 | 9.34E-07 | Down |
| NAPSA | 427.2125516 | 3.29055198 | 0.293658 | 11.2054 | 3.84E-29 | 6.02E-27 | Up |
| PDLIM4 | 2437.855907 | 3.005360066 | 0.277371 | 10.83518 | 2.35E-27 | 3.03E-25 | Up |
| KREMEN2 | 15.22960319 | 1.040271328 | 0.382077 | 2.722677 | 0.006476 | 0.019452 | Up |
| PPP1R1B | 266.6991204 | 4.706943656 | 0.34741 | 13.54868 | 8.07E-42 | 4.26E-39 | Up |
| KRT17P1 | 1.111731188 | 1.34719833 | 0.529642 | 2.543602 | 0.010972 | 0.030368 | Up |
| LRRC9 | 7.772321946 | -1.788910969 | 0.287017 | -6.23277 | 4.58E-10 | 6.77E-09 | Down |
| LGALS3 | 10106.39019 | 2.525203904 | 0.21724 | 11.62401 | 3.11E-31 | 5.93E-29 | Up |
| PODNL1 | 103.8874981 | 1.583271545 | 0.265743 | 5.957898 | 2.56E-09 | 3.33E-08 | Up |
| RTBDN | 6.394575159 | 2.896169836 | 0.425914 | 6.799894 | 1.05E-11 | 2.05E-10 | Up |
| MATN3 | 93.23592336 | 2.492683465 | 0.223303 | 11.1628 | 6.20E-29 | 9.49E-27 | Up |
| LINC00470 | 4.459355223 | -2.396831066 | 0.486049 | -4.93126 | 8.17E-07 | 6.64E-06 | Down |
| EMILIN2 | 1736.045649 | 1.740689276 | 0.163896 | 10.62072 | 2.39E-26 | 2.74E-24 | Up |
| RAMP1 | 291.8598403 | 2.201323854 | 0.313123 | 7.030218 | 2.06E-12 | 4.51E-11 | Up |
| PTPRE | 2006.468672 | 2.215866951 | 0.211299 | 10.48689 | 9.92E-26 | 1.07E-23 | Up |
| POPDC3 | 15.34807417 | -1.249863909 | 0.256039 | -4.88154 | 1.05E-06 | 8.36E-06 | Down |
| DDC | 13.14248952 | -1.143674892 | 0.451216 | -2.53465 | 0.011256 | 0.031004 | Down |
| ITGB4 | 3246.157341 | 1.729266781 | 0.177456 | 9.744737 | 1.94E-22 | 1.49E-20 | Up |
| AC087289.1 | 1413.676651 | 1.390525625 | 0.175003 | 7.945709 | 1.93E-15 | 6.45E-14 | Up |
| DLG4 | 1328.472155 | 1.489675944 | 0.138799 | 10.73258 | 7.16E-27 | 8.78E-25 | Up |
| RGS22 | 29.04660874 | -1.286748606 | 0.202382 | -6.35802 | 2.04E-10 | 3.22E-09 | Down |
| MATN2 | 34955.38912 | -1.685990641 | 0.210901 | -7.99424 | 1.30E-15 | 4.45E-14 | Down |
| REEP2 | 161.8855889 | 1.3002816 | 0.15757 | 8.252115 | 1.56E-16 | 6.00E-15 | Up |
| SNAP25 | 141.3802031 | 1.760799381 | 0.296588 | 5.93686 | 2.91E-09 | 3.74E-08 | Up |
| ALDH3B2 | 7.385494497 | 3.444006389 | 0.427124 | 8.063252 | 7.43E-16 | 2.59E-14 | Up |
| VSTM2L | 377.7684346 | 2.457168119 | 0.26618 | 9.231226 | 2.68E-20 | 1.64E-18 | Up |
| KANK4 | 91.62479338 | 1.223186321 | 0.308042 | 3.970843 | 7.16E-05 | 0.000375 | Up |
| SYT4 | 20.12630002 | -1.883918351 | 0.367422 | -5.1274 | 2.94E-07 | 2.62E-06 | Down |
| SLC14A2 | 212.1015678 | 1.415486834 | 0.371093 | 3.814372 | 0.000137 | 0.000668 | Up |
| PDE6A | 5.931207135 | 1.309619708 | 0.280174 | 4.674305 | 2.95E-06 | 2.13E-05 | Up |
| ATP8A2 | 76.34344193 | 1.268649039 | 0.251036 | 5.053659 | 4.33E-07 | 3.72E-06 | Up |
| WASF3 | 3026.15385 | -1.007247066 | 0.169579 | -5.93971 | 2.86E-09 | 3.68E-08 | Down |
| RNF17 | 14.40899542 | -2.218842573 | 0.409918 | -5.41289 | 6.20E-08 | 6.31E-07 | Down |
| GPR12 | 6.329641968 | -1.953558594 | 0.317715 | -6.14879 | 7.81E-10 | 1.10E-08 | Down |
| CHRM3 | 237.8186232 | 1.227776774 | 0.192311 | 6.384317 | 1.72E-10 | 2.75E-09 | Up |
| MYH10 | 8101.200559 | 1.161752031 | 0.141353 | 8.218824 | 2.06E-16 | 7.77E-15 | Up |
| CHI3L1 | 8368.568804 | 4.59388812 | 0.420917 | 10.914 | 9.88E-28 | 1.33E-25 | Up |
| MYBPH | 48.24535244 | 3.870051685 | 0.387523 | 9.986647 | 1.74E-23 | 1.51E-21 | Up |
| CCNA1 | 54.54569306 | 2.213851455 | 0.295822 | 7.483731 | 7.22E-14 | 1.94E-12 | Up |
| TRPC4 | 45.38175066 | 1.822577985 | 0.172704 | 10.55318 | 4.91E-26 | 5.42E-24 | Up |
| POSTN | 3935.793697 | 3.331946059 | 0.325 | 10.25214 | 1.16E-24 | 1.12E-22 | Up |
| KL | 252.1162977 | 1.536443961 | 0.161758 | 9.498382 | 2.13E-21 | 1.45E-19 | Up |
| STARD13 | 4360.031787 | -1.080104008 | 0.137604 | -7.84934 | 4.18E-15 | 1.34E-13 | Down |
| IRS4 | 139.1743461 | -2.428215393 | 0.331836 | -7.31751 | 2.53E-13 | 6.34E-12 | Down |
| BEX1 | 1424.743249 | -1.758114422 | 0.260171 | -6.75753 | 1.40E-11 | 2.70E-10 | Down |
| MYH11 | 5148.275384 | -1.154963586 | 0.250605 | -4.60871 | 4.05E-06 | 2.84E-05 | Down |
| C1QTNF6 | 576.2328991 | 1.088088622 | 0.169349 | 6.425138 | 1.32E-10 | 2.15E-09 | Up |
| GGT2 | 116.0060876 | 2.064410234 | 0.243825 | 8.466773 | 2.52E-17 | 1.05E-15 | Up |
| SEC14L4 | 10.66864085 | 1.550355269 | 0.346333 | 4.476485 | 7.59E-06 | 5.01E-05 | Up |
| NTS | 54.80451086 | -1.927679471 | 0.288473 | -6.68236 | 2.35E-11 | 4.36E-10 | Down |
| LRRIQ1 | 258.7863975 | -1.40926923 | 0.16495 | -8.5436 | 1.30E-17 | 5.61E-16 | Down |
| ARNTL | 2203.636934 | 1.350757798 | 0.215592 | 6.265336 | 3.72E-10 | 5.60E-09 | Up |
| LYVE1 | 1365.977123 | -2.372070701 | 0.240394 | -9.86744 | 5.76E-23 | 4.65E-21 | Down |
| MICAL2 | 2369.532148 | 1.101613424 | 0.158691 | 6.941861 | 3.87E-12 | 8.15E-11 | Up |
| VRTN | 7.447271907 | -2.125660745 | 0.720894 | -2.94865 | 0.003192 | 0.01059 | Down |
| LOXL2 | 694.331933 | 1.773258044 | 0.199101 | 8.906342 | 5.27E-19 | 2.75E-17 | Up |
| MRO | 1028.200603 | -1.509419702 | 0.298133 | -5.0629 | 4.13E-07 | 3.57E-06 | Down |
| IRAK2 | 604.6697166 | 1.057695817 | 0.160154 | 6.604262 | 4.00E-11 | 7.13E-10 | Up |
| BHLHE40 | 12579.67565 | 1.433137951 | 0.247867 | 5.781891 | 7.39E-09 | 8.81E-08 | Up |
| CHL1 | 191.8883573 | 1.015052824 | 0.352544 | 2.879219 | 0.003987 | 0.012848 | Up |
| GSTM1 | 772.5508364 | -1.292293517 | 0.518006 | -2.49475 | 0.012605 | 0.034121 | Down |
| REG4 | 3.446542148 | -1.38401301 | 0.593056 | -2.3337 | 0.019612 | 0.049298 | Down |
| TSPAN2 | 333.0052621 | -1.065603456 | 0.189997 | -5.60852 | 2.04E-08 | 2.25E-07 | Down |
| TSHB | 0.866779548 | -1.656224686 | 0.483651 | -3.42442 | 0.000616 | 0.002538 | Down |
| GSTM5 | 340.2730752 | -1.797763595 | 0.200334 | -8.97385 | 2.86E-19 | 1.54E-17 | Down |
| HMGCS2 | 71.78995967 | -2.154934847 | 0.531286 | -4.05607 | 4.99E-05 | 0.000273 | Down |
| VTCN1 | 79.37543204 | 4.667781814 | 0.488403 | 9.557227 | 1.21E-21 | 8.45E-20 | Up |
| PLEKHA8P1 | 112.5150258 | 1.205881087 | 0.139376 | 8.652021 | 5.06E-18 | 2.30E-16 | Up |
| MYCN | 216.7600159 | -1.20111444 | 0.174356 | -6.88884 | 5.62E-12 | 1.15E-10 | Down |
| SAA2 | 8.453984737 | 1.803117678 | 0.459062 | 3.927833 | 8.57E-05 | 0.00044 | Up |
| ERN2 | 3.509088627 | 1.850221595 | 0.493887 | 3.746245 | 0.00018 | 0.000852 | Up |
| HRH4 | 3.836674904 | 1.064406235 | 0.278371 | 3.823702 | 0.000131 | 0.000646 | Up |
| SOX5 | 269.4411859 | -1.354314783 | 0.165879 | -8.16446 | 3.23E-16 | 1.19E-14 | Down |
| SPX | 1912.534213 | -1.424226535 | 0.261813 | -5.43987 | 5.33E-08 | 5.49E-07 | Down |
| LRP4 | 5448.564593 | 4.430003219 | 0.251597 | 17.60754 | 2.16E-69 | 2.70E-65 | Up |
| DDB2 | 1141.846096 | 1.178947889 | 0.125238 | 9.413652 | 4.79E-21 | 3.18E-19 | Up |
| FOLH1B | 1.412354083 | 1.482105007 | 0.471469 | 3.143587 | 0.001669 | 0.006037 | Up |
| SPOCD1 | 55.59626017 | 2.775810969 | 0.251448 | 11.0393 | 2.47E-28 | 3.52E-26 | Up |
| DSC3 | 161.9474774 | 3.577521478 | 0.511588 | 6.99298 | 2.69E-12 | 5.80E-11 | Up |
| APLNR | 1912.180107 | 1.602351607 | 0.198581 | 8.069018 | 7.09E-16 | 2.48E-14 | Up |
| TCN1 | 8.44385792 | 2.547049152 | 0.531977 | 4.787891 | 1.69E-06 | 1.28E-05 | Up |
| C5AR2 | 106.5491241 | 1.847435181 | 0.22615 | 8.169066 | 3.11E-16 | 1.15E-14 | Up |
| CLDN10 | 348.7822375 | 6.020664505 | 0.46259 | 13.01512 | 1.00E-38 | 3.73E-36 | Up |
| ANXA1 | 42463.78192 | 1.089522021 | 0.251877 | 4.325617 | 1.52E-05 | 9.35E-05 | Up |
| MSI1 | 85.0842509 | 1.218626306 | 0.307597 | 3.961761 | 7.44E-05 | 0.000388 | Up |
| HNF1A | 1.195973928 | -1.415996836 | 0.56667 | -2.49881 | 0.012461 | 0.033794 | Down |
| HRK | 280.961871 | 1.857851805 | 0.256691 | 7.237703 | 4.56E-13 | 1.11E-11 | Up |
| CCDC146 | 1726.35212 | -1.287486003 | 0.152606 | -8.43667 | 3.26E-17 | 1.35E-15 | Down |
| FAM71F1 | 2.073044739 | -1.654365218 | 0.395303 | -4.18505 | 2.85E-05 | 0.000164 | Down |
| KCP | 294.6571866 | 2.895718704 | 0.231196 | 12.52497 | 5.45E-36 | 1.48E-33 | Up |
| ADGRB3 | 101.1695235 | 1.462054737 | 0.22679 | 6.446733 | 1.14E-10 | 1.89E-09 | Up |
| NT5E | 4041.10883 | 1.3479477 | 0.185839 | 7.253327 | 4.07E-13 | 9.94E-12 | Up |
| MRAP2 | 24.9159618 | -1.387008633 | 0.249885 | -5.55058 | 2.85E-08 | 3.07E-07 | Down |
| EPHA7 | 12.78425149 | -1.634886715 | 0.269222 | -6.07264 | 1.26E-09 | 1.72E-08 | Down |
| ELF5 | 6.453874327 | 1.727589331 | 0.345475 | 5.000623 | 5.71E-07 | 4.80E-06 | Up |
| PRPH | 26.69328641 | 1.145928229 | 0.205833 | 5.567262 | 2.59E-08 | 2.81E-07 | Up |
| GLS2 | 52.62373027 | 1.471827642 | 0.165263 | 8.905953 | 5.29E-19 | 2.76E-17 | Up |
| ITGA7 | 1385.286904 | 1.215692595 | 0.124543 | 9.761202 | 1.65E-22 | 1.27E-20 | Up |
| RDH5 | 150.2729048 | 2.720670257 | 0.218749 | 12.4374 | 1.64E-35 | 4.26E-33 | Up |
| KRT85 | 31.90108381 | -1.911619094 | 0.347739 | -5.49728 | 3.86E-08 | 4.06E-07 | Down |
| KRT87P | 233.1811944 | -1.68481368 | 0.249404 | -6.75536 | 1.42E-11 | 2.74E-10 | Down |
| HEY2 | 610.6802229 | 2.06847559 | 0.188169 | 10.99263 | 4.15E-28 | 5.74E-26 | Up |
| PKIB | 171.6477411 | 1.110949096 | 0.200226 | 5.548467 | 2.88E-08 | 3.10E-07 | Up |
| EGR4 | 11.00592825 | -1.181484336 | 0.391285 | -3.01949 | 0.002532 | 0.008668 | Down |
| DYSF | 2141.095253 | 1.38954166 | 0.143385 | 9.691006 | 3.29E-22 | 2.47E-20 | Up |
| BCO1 | 14.45540416 | -1.57862793 | 0.228718 | -6.90207 | 5.12E-12 | 1.06E-10 | Down |
| FHOD1 | 2352.968659 | 1.328877923 | 0.112456 | 11.81684 | 3.19E-32 | 6.55E-30 | Up |
| SLC9A5 | 113.5781579 | 1.70914899 | 0.168785 | 10.1262 | 4.23E-24 | 3.92E-22 | Up |
| AGT | 326.5563925 | 1.223084082 | 0.212369 | 5.759245 | 8.45E-09 | 1.00E-07 | Up |
| RGS8 | 1507.774252 | -1.396971723 | 0.40522 | -3.44744 | 0.000566 | 0.002354 | Down |
| WNT10A | 243.7268113 | 2.772288885 | 0.39871 | 6.953147 | 3.57E-12 | 7.57E-11 | Up |
| STAB2 | 168.0192215 | -2.056176615 | 0.252263 | -8.15092 | 3.61E-16 | 1.32E-14 | Down |
| PLXNC1 | 2489.079384 | 2.355858424 | 0.227067 | 10.37518 | 3.22E-25 | 3.32E-23 | Up |
| DRAM1 | 1158.769844 | 1.374288058 | 0.164002 | 8.379713 | 5.31E-17 | 2.14E-15 | Up |
| CNMD | 281.5132552 | -1.253927101 | 0.357272 | -3.50973 | 0.000449 | 0.001919 | Down |
| TBC1D4 | 6122.504322 | -1.39412553 | 0.131302 | -10.6177 | 2.46E-26 | 2.82E-24 | Down |
| SCEL | 1772.870202 | 4.140307088 | 0.299383 | 13.82944 | 1.69E-43 | 1.18E-40 | Up |
| RAPGEF5 | 1131.346241 | 1.270483216 | 0.131742 | 9.643709 | 5.23E-22 | 3.81E-20 | Up |
| MYO1G | 1269.027146 | 1.525973521 | 0.376974 | 4.047956 | 5.17E-05 | 0.000282 | Up |
| ADAMTS7 | 223.165711 | 1.333961181 | 0.160572 | 8.307568 | 9.77E-17 | 3.88E-15 | Up |
| ABHD17C | 1201.743505 | 1.110235494 | 0.138016 | 8.044248 | 8.68E-16 | 3.02E-14 | Up |
| TBR1 | 2.30624555 | -2.025231461 | 0.455593 | -4.44526 | 8.78E-06 | 5.73E-05 | Down |
| GALNT5 | 106.9048844 | 3.245907513 | 0.325356 | 9.976466 | 1.93E-23 | 1.67E-21 | Up |
| BLK | 331.43057 | -1.787356662 | 0.476134 | -3.75389 | 0.000174 | 0.000829 | Down |
| GATA4 | 8.387332399 | -2.828374917 | 0.486588 | -5.81267 | 6.15E-09 | 7.43E-08 | Down |
| IL36G | 0.582083356 | 1.621027619 | 0.625695 | 2.590764 | 0.009576 | 0.02713 | Up |
| IL1RN | 593.2437553 | 1.240893032 | 0.279873 | 4.433767 | 9.26E-06 | 6.01E-05 | Up |
| IL36A | 0.5664834 | 2.171455758 | 0.731691 | 2.967724 | 0.003 | 0.010039 | Up |
| IL36RN | 28.03177524 | 3.500647353 | 0.38008 | 9.210297 | 3.25E-20 | 1.96E-18 | Up |
| TMOD1 | 1557.818331 | -1.045211912 | 0.174328 | -5.99565 | 2.03E-09 | 2.69E-08 | Down |
| WDR38 | 21.86789413 | -1.423490836 | 0.217231 | -6.5529 | 5.64E-11 | 9.85E-10 | Down |
| GABBR2 | 2208.554265 | 2.874542104 | 0.236657 | 12.14643 | 5.99E-34 | 1.38E-31 | Up |
| HEMGN | 21.50961651 | -1.146840805 | 0.326781 | -3.50951 | 0.000449 | 0.00192 | Down |
| CCN3 | 1451.062349 | -1.01441924 | 0.141372 | -7.17555 | 7.20E-13 | 1.70E-11 | Down |
| CCL21 | 11943.86768 | -2.36334729 | 0.329632 | -7.16965 | 7.52E-13 | 1.77E-11 | Down |
| IGFBPL1 | 582.5596314 | -1.707851794 | 0.278726 | -6.12734 | 8.94E-10 | 1.25E-08 | Down |
| CAPN11 | 34.7407688 | 1.645943752 | 0.236323 | 6.964803 | 3.29E-12 | 7.02E-11 | Up |
| FHDC1 | 5095.709132 | -1.212215826 | 0.132865 | -9.12364 | 7.26E-20 | 4.24E-18 | Down |
| TLR2 | 700.3943279 | 1.035591098 | 0.181986 | 5.69051 | 1.27E-08 | 1.46E-07 | Up |
| TTC29 | 14.12772987 | -2.265716599 | 0.262527 | -8.63041 | 6.11E-18 | 2.75E-16 | Down |
| NXPE4 | 6.698835412 | 1.779136024 | 0.581647 | 3.058789 | 0.002222 | 0.007735 | Up |
| TMPRSS4 | 1356.593919 | 6.005963448 | 0.421088 | 14.26295 | 3.72E-46 | 3.18E-43 | Up |
| MMP7 | 373.7850782 | 3.308880975 | 0.50135 | 6.599946 | 4.11E-11 | 7.32E-10 | Up |
| MMP27 | 1.904779066 | -1.608953855 | 0.501766 | -3.20658 | 0.001343 | 0.005006 | Down |
| TRIM29 | 407.9233586 | 3.206588684 | 0.359922 | 8.909132 | 5.14E-19 | 2.69E-17 | Up |
| BTG4 | 1.509981602 | -1.863570321 | 0.435769 | -4.27651 | 1.90E-05 | 0.000114 | Down |
| POU2F3 | 381.5197004 | 1.696460482 | 0.170382 | 9.956793 | 2.36E-23 | 2.00E-21 | Up |
| MMP13 | 96.77873529 | 7.408296301 | 0.868207 | 8.532869 | 1.43E-17 | 6.12E-16 | Up |
| UNC13C | 181.2197177 | -1.873435484 | 0.276684 | -6.77102 | 1.28E-11 | 2.47E-10 | Down |
| ITGA11 | 400.8597835 | 1.331549959 | 0.231851 | 5.743127 | 9.29E-09 | 1.09E-07 | Up |
| ITPKA | 31.46821428 | 1.495405303 | 0.188866 | 7.917805 | 2.42E-15 | 7.98E-14 | Up |
| STRA6 | 646.5570583 | 5.254826141 | 0.348382 | 15.08354 | 2.08E-51 | 3.12E-48 | Up |
| GCOM1 | 10.14554951 | -1.161764438 | 0.218027 | -5.32854 | 9.90E-08 | 9.67E-07 | Down |
| CLCA2 | 13.59234932 | 2.097033946 | 0.463981 | 4.519657 | 6.19E-06 | 4.17E-05 | Up |
| LHCGR | 47.79027618 | -1.249875775 | 0.361424 | -3.4582 | 0.000544 | 0.002274 | Down |
| CYP1B1 | 2260.726153 | 2.69641132 | 0.316823 | 8.510789 | 1.73E-17 | 7.34E-16 | Up |
| ABCG5 | 9.287030315 | -1.082116713 | 0.220319 | -4.91159 | 9.03E-07 | 7.27E-06 | Down |
| TRIM54 | 107.0159709 | 2.51998915 | 0.282314 | 8.926196 | 4.41E-19 | 2.32E-17 | Up |
| DUSP5 | 5433.997907 | 2.12086385 | 0.302367 | 7.014215 | 2.31E-12 | 5.03E-11 | Up |
| ENTPD1 | 6964.518438 | 2.292999701 | 0.147724 | 15.52218 | 2.46E-54 | 4.85E-51 | Up |
| GPR87 | 8.410238024 | 2.948181928 | 0.481859 | 6.11835 | 9.45E-10 | 1.32E-08 | Up |
| PLA2G12B | 60.40150427 | 5.703056412 | 0.402915 | 14.15448 | 1.75E-45 | 1.40E-42 | Up |
| OIT3 | 78.41942778 | 3.571783199 | 0.365658 | 9.768089 | 1.54E-22 | 1.19E-20 | Up |
| ADAMTS14 | 128.1954267 | 3.644499459 | 0.393517 | 9.261354 | 2.02E-20 | 1.25E-18 | Up |
| AOX1 | 1535.692413 | -1.581847914 | 0.266337 | -5.93926 | 2.86E-09 | 3.69E-08 | Down |
| CCDC54 | 4.213788433 | -1.189485471 | 0.250059 | -4.75683 | 1.97E-06 | 1.47E-05 | Down |
| SHF | 826.5075336 | 1.008344905 | 0.117538 | 8.578904 | 9.58E-18 | 4.18E-16 | Up |
| CILP | 729.7154641 | 2.121502559 | 0.348265 | 6.091632 | 1.12E-09 | 1.54E-08 | Up |
| HCN4 | 102.0229419 | 4.309180521 | 0.34091 | 12.64023 | 1.27E-36 | 3.83E-34 | Up |
| ARHGAP24 | 3443.284238 | -1.153820054 | 0.181836 | -6.34538 | 2.22E-10 | 3.48E-09 | Down |
| PCDH10 | 75.01795126 | -1.352378563 | 0.237693 | -5.68959 | 1.27E-08 | 1.46E-07 | Down |
| PRKG2 | 80.4696851 | -1.198352551 | 0.382249 | -3.13501 | 0.001719 | 0.006193 | Down |
| BMPR1B | 46.50234512 | -1.301784609 | 0.268222 | -4.85339 | 1.21E-06 | 9.50E-06 | Down |
| MMRN1 | 2247.848383 | -2.377968746 | 0.247035 | -9.62604 | 6.21E-22 | 4.50E-20 | Down |
| PDE5A | 6856.783948 | 2.238509757 | 0.200609 | 11.15856 | 6.50E-29 | 9.84E-27 | Up |
| FRAS1 | 4853.193512 | -1.64949801 | 0.206566 | -7.98531 | 1.40E-15 | 4.75E-14 | Down |
| C4orf17 | 0.51340677 | 2.019147603 | 0.624139 | 3.235095 | 0.001216 | 0.004586 | Up |
| B4GALNT3 | 1099.705682 | 1.63399774 | 0.183305 | 8.914071 | 4.92E-19 | 2.58E-17 | Up |
| ERP27 | 747.2558841 | 1.658549688 | 0.267712 | 6.195266 | 5.82E-10 | 8.41E-09 | Up |
| PIK3C2G | 6.400685933 | -2.54445836 | 0.387706 | -6.56285 | 5.28E-11 | 9.26E-10 | Down |
| SLCO1C1 | 69.17517402 | 1.697802999 | 0.206491 | 8.222147 | 2.00E-16 | 7.58E-15 | Up |
| TMEM117 | 392.025043 | 1.002954686 | 0.097634 | 10.27257 | 9.37E-25 | 9.18E-23 | Up |
| PRICKLE1 | 1546.051743 | 1.150720502 | 0.180407 | 6.378474 | 1.79E-10 | 2.85E-09 | Up |
| PIANP | 101.4736116 | 2.132875355 | 0.244336 | 8.729275 | 2.56E-18 | 1.22E-16 | Up |
| AMIGO2 | 970.0884254 | 2.283436229 | 0.178315 | 12.80566 | 1.52E-37 | 5.02E-35 | Up |
| COL2A1 | 4.764454439 | 2.094068406 | 0.414657 | 5.050117 | 4.42E-07 | 3.79E-06 | Up |
| TPH2 | 2.473132115 | -1.493308113 | 0.395486 | -3.77588 | 0.000159 | 0.000768 | Down |
| LGR5 | 16.45097837 | 1.152153424 | 0.353208 | 3.261971 | 0.001106 | 0.004223 | Up |
| PTPRQ | 337.8698455 | -2.300401037 | 0.290862 | -7.90891 | 2.60E-15 | 8.52E-14 | Down |
| DUSP6 | 12183.0685 | 2.214605273 | 0.21872 | 10.12531 | 4.27E-24 | 3.94E-22 | Up |
| KERA | 7.687452439 | 2.143209978 | 0.742291 | 2.887292 | 0.003886 | 0.012562 | Up |
| TMEM132B | 708.5409912 | -1.213649417 | 0.159107 | -7.62789 | 2.39E-14 | 6.86E-13 | Down |
| FOXN4 | 1.623395413 | -1.553612107 | 0.436222 | -3.56152 | 0.000369 | 0.001612 | Down |
| GPR84 | 46.87684329 | 1.435208678 | 0.301943 | 4.753244 | 2.00E-06 | 1.50E-05 | Up |
| ITGB7 | 468.0135753 | 1.23103623 | 0.293711 | 4.191313 | 2.77E-05 | 0.00016 | Up |
| SRRM4 | 24.74559253 | -1.762064697 | 0.262665 | -6.70841 | 1.97E-11 | 3.68E-10 | Down |
| RNF113B | 0.889390383 | -1.822663962 | 0.555625 | -3.28039 | 0.001037 | 0.003994 | Down |
| CDH24 | 473.4740653 | 1.483321415 | 0.115335 | 12.86098 | 7.46E-38 | 2.55E-35 | Up |
| NOVA1 | 411.9922449 | 1.075769221 | 0.251413 | 4.278887 | 1.88E-05 | 0.000113 | Up |
| MDGA2 | 3.279326199 | -1.262212043 | 0.37757 | -3.34299 | 0.000829 | 0.003285 | Down |
| SYT16 | 34.02409225 | -1.711936787 | 0.197309 | -8.67644 | 4.08E-18 | 1.88E-16 | Down |
| RAB15 | 1237.586952 | 1.14653201 | 0.111125 | 10.31749 | 5.87E-25 | 5.86E-23 | Up |
| SLC27A2 | 566.3011543 | 1.006500893 | 0.16861 | 5.969393 | 2.38E-09 | 3.12E-08 | Up |
| HDC | 8.61988157 | 1.758028842 | 0.368164 | 4.775129 | 1.80E-06 | 1.35E-05 | Up |
| CYP1A1 | 8.66805237 | -1.317960141 | 0.407706 | -3.23263 | 0.001227 | 0.004619 | Down |
| PCSK6 | 137.2806536 | -1.103196338 | 0.150031 | -7.35312 | 1.94E-13 | 4.92E-12 | Down |
| RHCG | 2.157814771 | 1.08316132 | 0.430665 | 2.515092 | 0.0119 | 0.032526 | Up |
| NTRK3 | 356.9499122 | 1.593837866 | 0.22911 | 6.956652 | 3.48E-12 | 7.40E-11 | Up |
| MFGE8 | 7723.179851 | 2.071447929 | 0.157947 | 13.11482 | 2.71E-39 | 1.08E-36 | Up |
| ST8SIA2 | 38.64327999 | 1.219462842 | 0.285546 | 4.270635 | 1.95E-05 | 0.000117 | Up |
| MCTP2 | 2046.833829 | 1.165712007 | 0.096708 | 12.05391 | 1.85E-33 | 4.11E-31 | Up |
| SLC5A2 | 46.14026922 | 1.706840822 | 0.17737 | 9.623046 | 6.39E-22 | 4.62E-20 | Up |
| ABCC12 | 7.090346016 | 3.319331348 | 0.507798 | 6.536713 | 6.29E-11 | 1.09E-09 | Up |
| ADAMTS18 | 43.61072339 | 2.383020419 | 0.312269 | 7.631304 | 2.32E-14 | 6.70E-13 | Up |
| CMTM3 | 1679.874161 | 1.289510127 | 0.165065 | 7.812117 | 5.62E-15 | 1.77E-13 | Up |
| CDH11 | 1365.113328 | 1.878293453 | 0.24876 | 7.550615 | 4.33E-14 | 1.20E-12 | Up |
| CDH13 | 611.8979283 | 1.656329791 | 0.17502 | 9.463679 | 2.97E-21 | 2.00E-19 | Up |
| IRF8 | 1666.794977 | -1.179636326 | 0.398871 | -2.95744 | 0.003102 | 0.010334 | Down |
| PRELID3A | 174.6858605 | 1.849133945 | 0.154956 | 11.93329 | 7.94E-33 | 1.67E-30 | Up |
| ASXL3 | 388.0569474 | -1.656409901 | 0.270339 | -6.12715 | 8.95E-10 | 1.25E-08 | Down |
| TMC6 | 3028.820179 | 1.918384438 | 0.278647 | 6.884638 | 5.79E-12 | 1.18E-10 | Up |
| MAPK4 | 1581.844443 | -1.896628919 | 0.313788 | -6.0443 | 1.50E-09 | 2.03E-08 | Down |
| CBLN2 | 4.964105383 | -1.617811906 | 0.34252 | -4.72326 | 2.32E-06 | 1.71E-05 | Down |
| PMAIP1 | 260.0752916 | 1.182383811 | 0.329474 | 3.588703 | 0.000332 | 0.00147 | Up |
| GRB7 | 1121.286111 | 2.11401289 | 0.173863 | 12.15906 | 5.13E-34 | 1.20E-31 | Up |
| PNMT | 47.0749913 | 2.485992675 | 0.287326 | 8.652155 | 5.05E-18 | 2.30E-16 | Up |
| STAC2 | 8.031763618 | 1.198720324 | 0.26928 | 4.451578 | 8.52E-06 | 5.57E-05 | Up |
| SAMD1 | 1707.138676 | 1.04965714 | 0.116644 | 8.998806 | 2.28E-19 | 1.25E-17 | Up |
| PLPP2 | 900.1972595 | 1.583429284 | 0.254894 | 6.212111 | 5.23E-10 | 7.62E-09 | Up |
| SIK1 | 114.8226279 | 1.31689347 | 0.522364 | 2.521028 | 0.011701 | 0.032071 | Up |
| WTIP | 1279.199046 | 1.252150484 | 0.16406 | 7.632266 | 2.31E-14 | 6.66E-13 | Up |
| ADAMTS10 | 450.5713399 | 1.141292985 | 0.158855 | 7.184496 | 6.75E-13 | 1.60E-11 | Up |
| SLC6A3 | 105.648791 | -1.854977269 | 0.301684 | -6.14874 | 7.81E-10 | 1.10E-08 | Down |
| CACNG8 | 10.56504202 | 1.379024315 | 0.201219 | 6.853361 | 7.21E-12 | 1.45E-10 | Up |
| FBN3 | 938.0633538 | 1.227076904 | 0.276791 | 4.433224 | 9.28E-06 | 6.02E-05 | Up |
| SLC47A1 | 1380.009149 | 1.056821993 | 0.149249 | 7.080927 | 1.43E-12 | 3.23E-11 | Up |
| MMEL1 | 68.73430705 | -1.048494899 | 0.238168 | -4.40233 | 1.07E-05 | 6.83E-05 | Down |
| CFAP74 | 17.20111995 | 1.406488136 | 0.246304 | 5.710366 | 1.13E-08 | 1.30E-07 | Up |
| PRDM16 | 2962.961312 | -1.004896388 | 0.130816 | -7.68175 | 1.57E-14 | 4.65E-13 | Down |
| PADI1 | 3.140087847 | 2.545872511 | 0.418649 | 6.081161 | 1.19E-09 | 1.64E-08 | Up |
| MYOM3 | 70.14428781 | 1.979860615 | 0.249555 | 7.933566 | 2.13E-15 | 7.08E-14 | Up |
| DMRTA2 | 3.498151716 | -1.624574084 | 0.415862 | -3.90653 | 9.36E-05 | 0.000476 | Down |
| MAP3K6 | 1808.14275 | 1.297410093 | 0.1327 | 9.777015 | 1.41E-22 | 1.10E-20 | Up |
| FCN3 | 177.20775 | 1.688470497 | 0.218342 | 7.733163 | 1.05E-14 | 3.19E-13 | Up |
| SYTL1 | 2072.129063 | 1.633249297 | 0.203504 | 8.025622 | 1.01E-15 | 3.50E-14 | Up |
| CCN1 | 49297.11283 | -1.49029343 | 0.276809 | -5.38383 | 7.29E-08 | 7.30E-07 | Down |
| CYP4B1 | 1689.844179 | 1.647192922 | 0.248935 | 6.616973 | 3.67E-11 | 6.57E-10 | Up |
| IGSF3 | 2444.879065 | 1.067848849 | 0.181968 | 5.868336 | 4.40E-09 | 5.47E-08 | Up |
| GPR161 | 479.5267085 | 1.008568842 | 0.128623 | 7.841278 | 4.46E-15 | 1.42E-13 | Up |
| RXRG | 2065.077296 | 5.047796282 | 0.384561 | 13.12614 | 2.33E-39 | 9.52E-37 | Up |
| DPT | 4251.520192 | -2.200979564 | 0.252096 | -8.73071 | 2.53E-18 | 1.21E-16 | Down |
| NECTIN4 | 561.9724905 | 4.235105114 | 0.307168 | 13.7876 | 3.03E-43 | 1.83E-40 | Up |
| FCGR2A | 850.1796005 | 1.203380573 | 0.266865 | 4.50933 | 6.50E-06 | 4.36E-05 | Up |
| RGS5 | 19943.79627 | 1.161398339 | 0.140912 | 8.241991 | 1.69E-16 | 6.49E-15 | Up |
| CRABP2 | 2205.299 | 1.972991306 | 0.234536 | 8.412314 | 4.02E-17 | 1.65E-15 | Up |
| XPR1 | 4292.31903 | 1.360421075 | 0.12197 | 11.15376 | 6.86E-29 | 1.03E-26 | Up |
| FAM163A | 186.905849 | -1.659818557 | 0.384104 | -4.32128 | 1.55E-05 | 9.52E-05 | Down |
| TUFT1 | 1632.613645 | -1.011838584 | 0.255115 | -3.96621 | 7.30E-05 | 0.000382 | Down |
| ECM1 | 7950.123056 | 5.721504324 | 0.37621 | 15.20828 | 3.12E-52 | 5.08E-49 | Up |
| SUSD4 | 316.693687 | 1.508542819 | 0.196484 | 7.677701 | 1.62E-14 | 4.79E-13 | Up |
| EFNA3 | 110.1308102 | 1.008860987 | 0.145858 | 6.916754 | 4.62E-12 | 9.60E-11 | Up |
| LEFTY2 | 9.800174283 | -1.334646144 | 0.21365 | -6.24687 | 4.19E-10 | 6.23E-09 | Down |
| MBOAT2 | 2314.867874 | 1.016544245 | 0.153403 | 6.626635 | 3.43E-11 | 6.18E-10 | Up |
| REN | 34.23872929 | 4.695488458 | 0.31712 | 14.80666 | 1.33E-49 | 1.72E-46 | Up |
| ETNK2 | 1787.138161 | 1.35730509 | 0.140225 | 9.6795 | 3.69E-22 | 2.73E-20 | Up |
| OSR1 | 229.0341949 | -1.648218903 | 0.252613 | -6.52468 | 6.81E-11 | 1.17E-09 | Down |
| NAT8 | 6.853009379 | -1.835825176 | 0.374216 | -4.90579 | 9.31E-07 | 7.47E-06 | Down |
| C1QL2 | 44.55689366 | 1.964592235 | 0.488431 | 4.022252 | 5.76E-05 | 0.00031 | Up |
| CNGA3 | 16.04458324 | 2.217260404 | 0.288823 | 7.67689 | 1.63E-14 | 4.81E-13 | Up |
| SCN1A | 6.001475231 | -1.204798319 | 0.322039 | -3.74116 | 0.000183 | 0.000867 | Down |
| UNC80 | 137.6116079 | -1.702713701 | 0.241114 | -7.06187 | 1.64E-12 | 3.65E-11 | Down |
| NYAP2 | 3.940817895 | 2.083043798 | 0.42994 | 4.844969 | 1.27E-06 | 9.86E-06 | Up |
| TRPM8 | 4.536893186 | 2.413181231 | 0.482924 | 4.997023 | 5.82E-07 | 4.88E-06 | Up |
| HES6 | 441.3773708 | 1.610992345 | 0.300884 | 5.354206 | 8.59E-08 | 8.49E-07 | Up |
| MAR4 | 24.68519509 | 4.190945728 | 0.405558 | 10.33377 | 4.96E-25 | 4.97E-23 | Up |
| ACKR2 | 154.4684155 | -1.532100176 | 0.152645 | -10.037 | 1.05E-23 | 9.39E-22 | Down |
| STAC | 143.9843108 | 2.82500729 | 0.269572 | 10.47962 | 1.07E-25 | 1.15E-23 | Up |
| IL17RD | 416.1203059 | 2.031328882 | 0.196126 | 10.35725 | 3.88E-25 | 3.96E-23 | Up |
| LRIG1 | 5266.228263 | -1.059632938 | 0.115122 | -9.20446 | 3.43E-20 | 2.07E-18 | Down |
| LRTM1 | 1.3076246 | -1.966492224 | 0.553037 | -3.55581 | 0.000377 | 0.001644 | Down |
| COL8A1 | 11166.7628 | 1.523432109 | 0.148416 | 10.2646 | 1.02E-24 | 9.87E-23 | Up |
| MYH15 | 67.37566603 | -1.941838188 | 0.285072 | -6.81175 | 9.64E-12 | 1.90E-10 | Down |
| TAGLN3 | 35.89014044 | 1.934662697 | 0.320204 | 6.041964 | 1.52E-09 | 2.06E-08 | Up |
| TMEM108 | 731.1654152 | 1.634706891 | 0.192987 | 8.470549 | 2.44E-17 | 1.02E-15 | Up |
| TMEM44 | 652.1059115 | 1.00388046 | 0.078483 | 12.79106 | 1.84E-37 | 6.00E-35 | Up |
| UCN2 | 1.252760149 | 2.617649933 | 0.556918 | 4.700246 | 2.60E-06 | 1.90E-05 | Up |
| STXBP5L | 1439.116393 | -1.36815281 | 0.329529 | -4.15185 | 3.30E-05 | 0.000187 | Down |
| AHSG | 0.728901913 | 2.126809715 | 0.556523 | 3.821604 | 0.000133 | 0.000651 | Up |
| VWA5B2 | 72.4017772 | 1.045334497 | 0.195786 | 5.339165 | 9.34E-08 | 9.16E-07 | Up |
| EPHA5 | 297.8483735 | -2.480636948 | 0.315525 | -7.86194 | 3.78E-15 | 1.22E-13 | Down |
| CORIN | 57.35291558 | 1.160516431 | 0.193322 | 6.003009 | 1.94E-09 | 2.58E-08 | Up |
| SNCA | 454.4897267 | -1.281976867 | 0.157579 | -8.13546 | 4.10E-16 | 1.49E-14 | Down |
| ANK2 | 3126.907286 | -1.449071891 | 0.257731 | -5.62241 | 1.88E-08 | 2.09E-07 | Down |
| RNF175 | 46.42018206 | 1.217232519 | 0.236594 | 5.144812 | 2.68E-07 | 2.40E-06 | Up |
| NKD2 | 91.30854059 | 2.531992528 | 0.311691 | 8.123397 | 4.53E-16 | 1.63E-14 | Up |
| HAPLN1 | 100.0537654 | 6.083747666 | 0.485147 | 12.54001 | 4.51E-36 | 1.24E-33 | Up |
| SPATA9 | 36.24435329 | 1.571038498 | 0.146448 | 10.72762 | 7.55E-27 | 9.14E-25 | Up |
| CXCL14 | 9350.127757 | 3.959638117 | 0.414894 | 9.543739 | 1.38E-21 | 9.54E-20 | Up |
| SLC25A48 | 27.2028495 | 3.510054001 | 0.365251 | 9.609967 | 7.26E-22 | 5.21E-20 | Up |
| TIMD4 | 136.2121944 | -1.491447563 | 0.411451 | -3.62485 | 0.000289 | 0.001302 | Down |
| GABRA6 | 3.572724499 | 5.279707228 | 0.529725 | 9.966892 | 2.13E-23 | 1.83E-21 | Up |
| GABRB2 | 1826.670973 | 6.79248295 | 0.351221 | 19.33962 | 2.49E-83 | 9.36E-79 | Up |
| TENM2 | 155.4229525 | -1.368925647 | 0.247986 | -5.52018 | 3.39E-08 | 3.59E-07 | Down |
| KCNMB1 | 169.2374003 | 1.092075156 | 0.210337 | 5.192036 | 2.08E-07 | 1.91E-06 | Up |
| FAM217A | 3.579300126 | -1.133433257 | 0.311017 | -3.64428 | 0.000268 | 0.001217 | Down |
| PSD2 | 28.57332858 | 2.035667941 | 0.26608 | 7.650596 | 2.00E-14 | 5.82E-13 | Up |
| GFRA3 | 15.95021909 | -1.796474621 | 0.246194 | -7.29698 | 2.94E-13 | 7.31E-12 | Down |
| PLA2G7 | 1711.270969 | -1.019476116 | 0.169589 | -6.01146 | 1.84E-09 | 2.46E-08 | Down |
| TNFRSF21 | 1206.810748 | 1.342664917 | 0.219017 | 6.130419 | 8.76E-10 | 1.23E-08 | Up |
| LGSN | 2.245848399 | -2.458682769 | 0.535558 | -4.59088 | 4.41E-06 | 3.07E-05 | Down |
| SCUBE3 | 9029.441831 | -1.058289591 | 0.2126 | -4.97783 | 6.43E-07 | 5.34E-06 | Down |
| TCTE1 | 8.716427169 | -1.171744799 | 0.268218 | -4.36863 | 1.25E-05 | 7.86E-05 | Down |
| FAXC | 324.1142302 | 2.657687551 | 0.192331 | 13.8183 | 1.98E-43 | 1.30E-40 | Up |
| CLVS2 | 0.60844392 | 2.106420629 | 0.624394 | 3.373542 | 0.000742 | 0.002978 | Up |
| RSPO3 | 176.6255102 | -1.046585434 | 0.221383 | -4.72749 | 2.27E-06 | 1.68E-05 | Down |
| PNLDC1 | 9.117186894 | -1.598748929 | 0.346562 | -4.61317 | 3.97E-06 | 2.78E-05 | Down |
| SDK1 | 798.8904361 | 2.682260938 | 0.272126 | 9.856683 | 6.41E-23 | 5.14E-21 | Up |
| LINC00525 | 5.988689598 | 1.411503696 | 0.400592 | 3.523548 | 0.000426 | 0.001833 | Up |
| IGFBP3 | 3204.391686 | 1.042221229 | 0.202418 | 5.14885 | 2.62E-07 | 2.36E-06 | Up |
| IGFBP1 | 3.209405153 | 1.417498827 | 0.479153 | 2.958343 | 0.003093 | 0.010307 | Up |
| ASB15 | 2.281069592 | -2.539610802 | 0.57324 | -4.43027 | 9.41E-06 | 6.09E-05 | Down |
| SYTL5 | 449.033819 | 5.431090562 | 0.392917 | 13.8225 | 1.86E-43 | 1.26E-40 | Up |
| MSN | 38793.40711 | 1.050776514 | 0.094126 | 11.16356 | 6.15E-29 | 9.46E-27 | Up |
| CHST7 | 407.9574888 | 1.046093083 | 0.19288 | 5.423538 | 5.84E-08 | 5.98E-07 | Up |
| AWAT2 | 2.662366067 | 3.331585264 | 0.392512 | 8.487865 | 2.10E-17 | 8.87E-16 | Up |
| NXF3 | 5.657241999 | 1.919871967 | 0.335182 | 5.727852 | 1.02E-08 | 1.19E-07 | Up |
| RIPPLY1 | 6.457209645 | 2.129242877 | 0.270958 | 7.858202 | 3.90E-15 | 1.25E-13 | Up |
| RADX | 410.6283714 | -1.160460783 | 0.112569 | -10.3089 | 6.42E-25 | 6.36E-23 | Down |
| IGSF1 | 8364.776155 | 4.230345621 | 0.316614 | 13.3612 | 1.02E-40 | 4.78E-38 | Up |
| ARHGAP36 | 2935.288862 | 8.10553879 | 0.566691 | 14.30328 | 2.09E-46 | 1.82E-43 | Up |
| GPC3 | 862.3833185 | -2.513101408 | 0.233292 | -10.7724 | 4.65E-27 | 5.87E-25 | Down |
| CSGALNACT1 | 11415.73995 | -1.351933893 | 0.196853 | -6.86774 | 6.52E-12 | 1.32E-10 | Down |
| STAR | 14.54337157 | -1.17192932 | 0.370377 | -3.16415 | 0.001555 | 0.00569 | Down |
| CRH | 1.07635962 | 2.534740129 | 0.514358 | 4.927968 | 8.31E-07 | 6.74E-06 | Up |
| PMP2 | 11.73621703 | -3.402060416 | 0.353964 | -9.61132 | 7.16E-22 | 5.16E-20 | Down |
| SLC26A7 | 51024.19402 | -2.010616969 | 0.244738 | -8.21537 | 2.12E-16 | 7.98E-15 | Down |
| FAM135B | 8.932752496 | -1.252044647 | 0.223686 | -5.59733 | 2.18E-08 | 2.39E-07 | Down |
| CDKN2B | 285.767806 | 2.74806603 | 0.220324 | 12.47281 | 1.05E-35 | 2.80E-33 | Up |
| CDKN2A | 113.8067879 | 2.635660452 | 0.321104 | 8.208109 | 2.25E-16 | 8.45E-15 | Up |
| SHC3 | 351.2646102 | 1.526097269 | 0.220695 | 6.914965 | 4.68E-12 | 9.70E-11 | Up |
| PLPPR1 | 7.130456564 | 3.006518196 | 0.381601 | 7.878687 | 3.31E-15 | 1.07E-13 | Up |
| SLC25A25 | 3752.697786 | -1.165136154 | 0.2057 | -5.66424 | 1.48E-08 | 1.67E-07 | Down |
| LCN2 | 593.9585434 | 3.661226755 | 0.375452 | 9.751514 | 1.82E-22 | 1.39E-20 | Up |
| HMCN2 | 647.6864681 | -1.082297344 | 0.222113 | -4.87273 | 1.10E-06 | 8.71E-06 | Down |
| CACNA1B | 91.38088419 | -1.71873808 | 0.303986 | -5.65401 | 1.57E-08 | 1.76E-07 | Down |
| SLC39A12 | 2.71162544 | 2.295444126 | 0.431511 | 5.319549 | 1.04E-07 | 1.01E-06 | Up |
| TMEM236 | 20.43152513 | 1.397055779 | 0.244629 | 5.710915 | 1.12E-08 | 1.30E-07 | Up |
| ANKRD30A | 2.797302748 | -1.125958546 | 0.357749 | -3.14734 | 0.001648 | 0.00597 | Down |
| RGR | 1.062335659 | -1.708010174 | 0.515752 | -3.31169 | 0.000927 | 0.003626 | Down |
| ANKRD1 | 8.868487044 | -1.19605579 | 0.438497 | -2.72763 | 0.006379 | 0.019216 | Down |
| NPFFR1 | 78.35759459 | 1.335222436 | 0.344431 | 3.876599 | 0.000106 | 0.000533 | Up |
| PLEKHS1 | 9.924630835 | 1.449169705 | 0.440574 | 3.289274 | 0.001004 | 0.003888 | Up |
| CYP17A1 | 313.3916283 | -1.279640926 | 0.179641 | -7.12332 | 1.05E-12 | 2.44E-11 | Down |
| INA | 15.34265545 | 1.262787674 | 0.35971 | 3.51057 | 0.000447 | 0.001914 | Up |
| NKX6-2 | 1.696916558 | -1.371744368 | 0.467347 | -2.93517 | 0.003334 | 0.011006 | Down |
| ADAM12 | 379.2675243 | 2.999453161 | 0.360809 | 8.313137 | 9.32E-17 | 3.71E-15 | Up |
| ADM | 1278.359886 | 1.768880628 | 0.258017 | 6.855669 | 7.10E-12 | 1.43E-10 | Up |
| SLC5A12 | 7.615908468 | 1.597742695 | 0.36838 | 4.337212 | 1.44E-05 | 8.94E-05 | Up |
| LRRC4C | 402.5440803 | -1.510788131 | 0.199168 | -7.58551 | 3.31E-14 | 9.29E-13 | Down |
| SAA4 | 1.039307431 | 2.354008786 | 0.711689 | 3.307635 | 0.000941 | 0.00367 | Up |
| SYT8 | 11.94056626 | 2.836974537 | 0.541611 | 5.238029 | 1.62E-07 | 1.52E-06 | Up |
| GLYAT | 2.20329412 | -1.663970441 | 0.586632 | -2.83648 | 0.004561 | 0.014437 | Down |
| ENDOD1 | 4008.686052 | 1.26389614 | 0.117865 | 10.72328 | 7.91E-27 | 9.52E-25 | Up |
| NCAM1 | 7437.442308 | -1.330476216 | 0.240449 | -5.5333 | 3.14E-08 | 3.35E-07 | Down |
| DRD2 | 2.938130079 | 1.284430564 | 0.406931 | 3.156386 | 0.001597 | 0.005822 | Up |
| ADAM33 | 380.8568481 | -1.321844544 | 0.187755 | -7.04028 | 1.92E-12 | 4.22E-11 | Down |
| MS4A2 | 13.76595676 | 2.310121203 | 0.352278 | 6.55767 | 5.47E-11 | 9.56E-10 | Up |
| MPZL2 | 8339.378446 | 2.479630051 | 0.160304 | 15.46833 | 5.68E-54 | 1.07E-50 | Up |
| OCSTAMP | 1.92632899 | 1.676631228 | 0.568342 | 2.950042 | 0.003177 | 0.010551 | Up |
| CDH22 | 122.3236318 | 2.827798161 | 0.333946 | 8.467842 | 2.50E-17 | 1.05E-15 | Up |
| FAM57B | 5.246233067 | 1.021816763 | 0.306671 | 3.331968 | 0.000862 | 0.0034 | Up |
| HMGA2 | 909.1142655 | 5.187368336 | 0.398934 | 13.00307 | 1.18E-38 | 4.32E-36 | Up |
| MMP3 | 4.015600347 | 1.870125593 | 0.708058 | 2.641203 | 0.008261 | 0.023954 | Up |
| CNTN5 | 520.5998711 | -2.315333804 | 0.284732 | -8.13164 | 4.24E-16 | 1.53E-14 | Down |
| MKX | 124.6664394 | 1.919480444 | 0.256398 | 7.486339 | 7.08E-14 | 1.91E-12 | Up |
| FCGR1A | 141.3672556 | 1.064456903 | 0.389658 | 2.731775 | 0.006299 | 0.019005 | Up |
| CDH8 | 16.48863785 | 1.496864142 | 0.2262 | 6.617425 | 3.66E-11 | 6.56E-10 | Up |
| LYPD1 | 41.49728339 | 1.512037528 | 0.323769 | 4.670114 | 3.01E-06 | 2.17E-05 | Up |
| GPM6A | 1351.45185 | -2.061292306 | 0.245787 | -8.38651 | 5.01E-17 | 2.03E-15 | Down |
| DLG2 | 2669.837619 | -1.306746239 | 0.260856 | -5.00945 | 5.46E-07 | 4.60E-06 | Down |
| PRSS23 | 9784.930842 | 2.488974198 | 0.204562 | 12.16732 | 4.64E-34 | 1.10E-31 | Up |
| PPP1R1C | 86.82643303 | 1.841996011 | 0.315399 | 5.840209 | 5.21E-09 | 6.39E-08 | Up |
| C2orf50 | 19.47113706 | -1.142884721 | 0.171468 | -6.66531 | 2.64E-11 | 4.85E-10 | Down |
| ITPR1 | 11565.57696 | -1.343500406 | 0.202851 | -6.62309 | 3.52E-11 | 6.32E-10 | Down |
| ENKUR | 64.14068639 | -1.116518821 | 0.186449 | -5.98835 | 2.12E-09 | 2.80E-08 | Down |
| CACNA1C | 321.1661893 | 1.04984684 | 0.128776 | 8.15252 | 3.56E-16 | 1.30E-14 | Up |
| SLC2A13 | 444.5806478 | 1.194528053 | 0.139805 | 8.544218 | 1.29E-17 | 5.58E-16 | Up |
| THRSP | 492.3758401 | 3.13556413 | 0.302501 | 10.36547 | 3.56E-25 | 3.65E-23 | Up |
| ME3 | 804.3139098 | 1.282862583 | 0.137025 | 9.362253 | 7.81E-21 | 5.08E-19 | Up |
| ADAMTS12 | 193.2944977 | 1.149845613 | 0.192462 | 5.974413 | 2.31E-09 | 3.02E-08 | Up |
| PTPRO | 117.5266412 | 1.0646454 | 0.320821 | 3.3185 | 0.000905 | 0.003549 | Up |
| EPS8 | 4133.441132 | 1.872299945 | 0.117661 | 15.9126 | 5.18E-57 | 1.62E-53 | Up |
| ANO4 | 33.53162544 | -1.167610781 | 0.240899 | -4.84689 | 1.25E-06 | 9.77E-06 | Down |
| EDNRA | 728.6335458 | 1.098152392 | 0.156942 | 6.997182 | 2.61E-12 | 5.64E-11 | Up |
| AKR1C6P | 3.350636371 | -1.294082676 | 0.406286 | -3.18515 | 0.001447 | 0.005342 | Down |
| AKR1C2 | 310.8733198 | -2.041181355 | 0.217354 | -9.39103 | 5.94E-21 | 3.90E-19 | Down |
| DPYSL4 | 85.47733768 | 1.141889044 | 0.336594 | 3.392477 | 0.000693 | 0.002806 | Up |
| RNF144A | 1116.491502 | 1.018488427 | 0.162041 | 6.285367 | 3.27E-10 | 4.97E-09 | Up |
| KCNJ1 | 114.2772783 | -1.21825596 | 0.393646 | -3.0948 | 0.001969 | 0.006971 | Down |
| TDO2 | 66.20987518 | 1.692608262 | 0.497187 | 3.404368 | 0.000663 | 0.002703 | Up |
| SLC35F4 | 61.76328523 | -1.847254953 | 0.342961 | -5.3862 | 7.20E-08 | 7.22E-07 | Down |
| GABRA2 | 7.520218723 | -2.521550506 | 0.348505 | -7.23534 | 4.64E-13 | 1.13E-11 | Down |
| CCDC175 | 3.075630467 | -2.888441911 | 0.484391 | -5.96304 | 2.48E-09 | 3.23E-08 | Down |
| CENPJ | 1441.600301 | -1.516706927 | 0.167604 | -9.04932 | 1.44E-19 | 8.12E-18 | Down |
| GFRA1 | 649.0988186 | -1.605073187 | 0.224922 | -7.13612 | 9.60E-13 | 2.23E-11 | Down |
| GLT1D1 | 422.3076725 | 2.685819175 | 0.273107 | 9.834313 | 8.01E-23 | 6.36E-21 | Up |
| TMEM132D | 149.9602011 | 4.14987146 | 0.311107 | 13.33905 | 1.37E-40 | 6.36E-38 | Up |
| RBM46 | 22.86305675 | -1.040723525 | 0.400801 | -2.59661 | 0.009415 | 0.026744 | Down |
| ZNF37CP | 7.204556069 | -1.752774609 | 0.339492 | -5.16293 | 2.43E-07 | 2.20E-06 | Down |
| AP1S3 | 1387.05429 | -1.053093572 | 0.146219 | -7.20218 | 5.93E-13 | 1.42E-11 | Down |
| TMEM163 | 584.0671694 | 3.421131331 | 0.355904 | 9.612504 | 7.08E-22 | 5.11E-20 | Up |
| HSPB8 | 3951.972784 | -1.103064043 | 0.258443 | -4.26812 | 1.97E-05 | 0.000118 | Down |
| CYSLTR2 | 869.6656142 | 2.591650701 | 0.338655 | 7.652773 | 1.97E-14 | 5.74E-13 | Up |
| SH2D6 | 243.9286243 | -1.457911008 | 0.246674 | -5.91028 | 3.42E-09 | 4.33E-08 | Down |
| SPOCK1 | 1329.458134 | 2.416218584 | 0.230087 | 10.50134 | 8.52E-26 | 9.24E-24 | Up |
| OLAH | 34.31497205 | 2.776368411 | 0.321433 | 8.637472 | 5.75E-18 | 2.60E-16 | Up |
| TRIM36 | 147.7803936 | 2.465858342 | 0.316679 | 7.786625 | 6.88E-15 | 2.15E-13 | Up |
| GRIA4 | 11.03569487 | -1.225729146 | 0.226768 | -5.4052 | 6.47E-08 | 6.56E-07 | Down |
| IGSF10 | 378.9102629 | -2.336290041 | 0.278596 | -8.38594 | 5.03E-17 | 2.04E-15 | Down |
| DMP1 | 3.836981113 | 2.778935299 | 0.461435 | 6.022372 | 1.72E-09 | 2.31E-08 | Up |
| CAPSL | 202.2741802 | -1.061093456 | 0.307888 | -3.44636 | 0.000568 | 0.002362 | Down |
| CLEC4F | 29.80488711 | 1.045218882 | 0.377527 | 2.768595 | 0.00563 | 0.017262 | Up |
| ANKRD22 | 118.4252544 | 1.216104175 | 0.454739 | 2.67429 | 0.007489 | 0.02201 | Up |
| GRM1 | 26.22223335 | -1.047753672 | 0.182973 | -5.72627 | 1.03E-08 | 1.20E-07 | Down |
| PART1 | 32.24455473 | -1.174221025 | 0.447578 | -2.6235 | 0.008703 | 0.025033 | Down |
| NRSN1 | 25.79142248 | 1.03983291 | 0.367566 | 2.828966 | 0.00467 | 0.014718 | Up |
| ADGRA3 | 5103.536361 | -1.175029378 | 0.149571 | -7.85597 | 3.97E-15 | 1.27E-13 | Down |
| PTPRR | 85.08079856 | 1.191322828 | 0.194113 | 6.137277 | 8.39E-10 | 1.18E-08 | Up |
| PLA2R1 | 9672.513558 | -1.957164926 | 0.237517 | -8.24012 | 1.72E-16 | 6.57E-15 | Down |
| SCN3A | 176.9778726 | -1.106420307 | 0.230975 | -4.79022 | 1.67E-06 | 1.27E-05 | Down |
| ADGRF4 | 14.71949507 | 3.638266522 | 0.438024 | 8.306087 | 9.89E-17 | 3.92E-15 | Up |
| FRMD1 | 1.916882299 | -1.357648776 | 0.462699 | -2.9342 | 0.003344 | 0.011035 | Down |
| PLEKHG4B | 1370.568708 | -1.391429946 | 0.285608 | -4.87181 | 1.11E-06 | 8.75E-06 | Down |
| LURAP1L | 282.9163934 | 1.38457587 | 0.195547 | 7.080537 | 1.44E-12 | 3.24E-11 | Up |
| FAM92B | 12.57070126 | 1.594300264 | 0.630469 | 2.528753 | 0.011447 | 0.031461 | Up |
| TMPRSS11D | 3.424380245 | -1.07318125 | 0.265253 | -4.04588 | 5.21E-05 | 0.000284 | Down |
| SPHKAP | 37.33347069 | -1.575902016 | 0.517029 | -3.04799 | 0.002304 | 0.007977 | Down |
| PID1 | 464.6884684 | -2.021288161 | 0.198546 | -10.1805 | 2.42E-24 | 2.27E-22 | Down |
| ANKFN1 | 7.371016616 | -1.520464065 | 0.267322 | -5.68776 | 1.29E-08 | 1.48E-07 | Down |
| SEMA3D | 5282.892475 | -2.05038759 | 0.346204 | -5.92248 | 3.17E-09 | 4.06E-08 | Down |
| CHST9 | 3.711085656 | -1.604231237 | 0.343444 | -4.67101 | 3.00E-06 | 2.16E-05 | Down |
| DNAAF1 | 470.3817752 | -1.311775942 | 0.202197 | -6.48761 | 8.72E-11 | 1.47E-09 | Down |
| JPH3 | 30.88807086 | 1.536057153 | 0.2631 | 5.838303 | 5.27E-09 | 6.46E-08 | Up |
| NRGN | 585.9785333 | 1.009963235 | 0.14872 | 6.791017 | 1.11E-11 | 2.17E-10 | Up |
| ABI3BP | 5839.236311 | -1.568714808 | 0.208167 | -7.53586 | 4.85E-14 | 1.34E-12 | Down |
| ABCA9 | 488.4018852 | -1.990694725 | 0.232669 | -8.55591 | 1.17E-17 | 5.07E-16 | Down |
| ABCA10 | 198.2934516 | -1.316322252 | 0.194907 | -6.7536 | 1.44E-11 | 2.77E-10 | Down |
| ENPP3 | 115.7146155 | 1.408640642 | 0.319253 | 4.412296 | 1.02E-05 | 6.56E-05 | Up |
| TNIK | 1988.762074 | 1.149024891 | 0.135553 | 8.476589 | 2.32E-17 | 9.72E-16 | Up |
| CCDC173 | 172.5266457 | -1.626658598 | 0.218284 | -7.45203 | 9.19E-14 | 2.44E-12 | Down |
| PDLIM3 | 2972.442463 | -1.626382071 | 0.211787 | -7.67932 | 1.60E-14 | 4.73E-13 | Down |
| SORBS2 | 26054.43197 | -1.256258834 | 0.225267 | -5.57674 | 2.45E-08 | 2.67E-07 | Down |
| PSMA8 | 8.829693973 | -1.705034834 | 0.409029 | -4.16849 | 3.07E-05 | 0.000176 | Down |
| PDE1C | 292.0487261 | 1.665168872 | 0.201582 | 8.260505 | 1.45E-16 | 5.59E-15 | Up |
| WNT7A | 15.60704481 | 2.432681494 | 0.416921 | 5.83488 | 5.38E-09 | 6.59E-08 | Up |
| PIEZO2 | 416.8094881 | 1.270259936 | 0.161793 | 7.851145 | 4.12E-15 | 1.32E-13 | Up |
| EPHB1 | 1674.345983 | -1.567488701 | 0.213628 | -7.33747 | 2.18E-13 | 5.51E-12 | Down |
| SEP14 | 0.851117781 | -1.550862449 | 0.502783 | -3.08456 | 0.002039 | 0.007175 | Down |
| RSPH10B | 4.28251143 | -1.091415056 | 0.23414 | -4.66138 | 3.14E-06 | 2.26E-05 | Down |
| GOLGA7B | 341.75983 | 1.713715291 | 0.297866 | 5.753308 | 8.75E-09 | 1.03E-07 | Up |
| GRIA1 | 18.97751741 | -2.867766729 | 0.301799 | -9.50225 | 2.05E-21 | 1.40E-19 | Down |
| LRGUK | 52.69941449 | -1.257614365 | 0.181028 | -6.94708 | 3.73E-12 | 7.87E-11 | Down |
| OTOA | 12.14877812 | 1.027376542 | 0.340623 | 3.016166 | 0.00256 | 0.008745 | Up |
| DEPTOR | 8692.620906 | -1.379272153 | 0.192233 | -7.175 | 7.23E-13 | 1.70E-11 | Down |
| FMN2 | 104.6573645 | -2.256436291 | 0.363115 | -6.21411 | 5.16E-10 | 7.55E-09 | Down |
| ELMO1 | 8870.347829 | -1.049053294 | 0.207019 | -5.06742 | 4.03E-07 | 3.49E-06 | Down |
| SAXO1 | 8.455891587 | -1.017766696 | 0.184359 | -5.52056 | 3.38E-08 | 3.59E-07 | Down |
| ADCY8 | 123.6059731 | 5.437205297 | 0.60325 | 9.013184 | 2.00E-19 | 1.11E-17 | Up |
| AFF2 | 163.6792055 | 1.635886417 | 0.305288 | 5.358496 | 8.39E-08 | 8.31E-07 | Up |
| PSD3 | 3435.848216 | 1.758401524 | 0.182017 | 9.660652 | 4.43E-22 | 3.27E-20 | Up |
| WIF1 | 14.68415377 | 5.531694902 | 0.783235 | 7.062624 | 1.63E-12 | 3.64E-11 | Up |
| MMP16 | 405.646058 | 2.317135945 | 0.272146 | 8.514304 | 1.68E-17 | 7.13E-16 | Up |
| TIAM1 | 1033.161978 | 2.464399659 | 0.247995 | 9.937284 | 2.87E-23 | 2.41E-21 | Up |
| TDRD9 | 1448.108362 | -1.639086272 | 0.270033 | -6.06994 | 1.28E-09 | 1.75E-08 | Down |
| GDF6 | 43.20324763 | -1.143967329 | 0.226876 | -5.04227 | 4.60E-07 | 3.93E-06 | Down |
| CD109 | 755.479486 | 2.057640158 | 0.21455 | 9.590501 | 8.77E-22 | 6.22E-20 | Up |
| LRFN2 | 14.97430531 | 1.240340994 | 0.404878 | 3.063493 | 0.002188 | 0.007634 | Up |
| UNC5D | 113.7948773 | -1.123235145 | 0.346883 | -3.23808 | 0.001203 | 0.004544 | Down |
| GLYATL2 | 6.171477709 | -1.031471614 | 0.277687 | -3.71451 | 0.000204 | 0.000951 | Down |
| MAPK13 | 5562.386017 | 1.063480937 | 0.084567 | 12.57553 | 2.88E-36 | 8.06E-34 | Up |
| MS4A1 | 1934.776125 | -2.113725506 | 0.616728 | -3.42732 | 0.00061 | 0.002516 | Down |
| ADGRG4 | 1.605034584 | -1.602130425 | 0.500515 | -3.20096 | 0.00137 | 0.00509 | Down |
| LHFPL4 | 14.84238638 | 1.068955739 | 0.323545 | 3.303888 | 0.000954 | 0.003713 | Up |
| B3GNT7 | 535.076648 | 2.192191801 | 0.193936 | 11.3037 | 1.26E-29 | 2.05E-27 | Up |
| SLC6A1 | 76.1306822 | 3.289234835 | 0.27529 | 11.94827 | 6.63E-33 | 1.41E-30 | Up |
| TMEM171 | 1738.023439 | -1.840547697 | 0.199313 | -9.23447 | 2.60E-20 | 1.59E-18 | Down |
| C8A | 8.346252265 | 3.502055494 | 0.567067 | 6.175736 | 6.59E-10 | 9.41E-09 | Up |
| SYN2 | 44.10506316 | -1.859944028 | 0.278502 | -6.67838 | 2.42E-11 | 4.46E-10 | Down |
| MMP14 | 7498.391596 | 1.126372129 | 0.147802 | 7.620792 | 2.52E-14 | 7.23E-13 | Up |
| C1orf158 | 47.48851166 | -3.398831494 | 0.397698 | -8.54627 | 1.27E-17 | 5.49E-16 | Down |
| ARSE | 27.10079363 | 2.240008486 | 0.262887 | 8.520801 | 1.58E-17 | 6.76E-16 | Up |
| KIT | 6598.157284 | -1.110538482 | 0.296458 | -3.74602 | 0.00018 | 0.000852 | Down |
| PWWP3B | 1906.739568 | -1.919258372 | 0.318466 | -6.02658 | 1.67E-09 | 2.25E-08 | Down |
| KCNJ6 | 9.627513663 | 1.345237046 | 0.51724 | 2.600797 | 0.009301 | 0.02648 | Up |
| DGKI | 6253.394939 | -1.631601566 | 0.307114 | -5.31269 | 1.08E-07 | 1.04E-06 | Down |
| SVOPL | 104.3858449 | 1.247172932 | 0.252346 | 4.942313 | 7.72E-07 | 6.31E-06 | Up |
| SLC34A2 | 47813.09217 | 5.0541167 | 0.359972 | 14.04032 | 8.83E-45 | 6.76E-42 | Up |
| ACAN | 86.38449224 | 2.210702923 | 0.260388 | 8.490023 | 2.07E-17 | 8.73E-16 | Up |
| CABP1 | 36.5492515 | 1.188870065 | 0.192814 | 6.165892 | 7.01E-10 | 9.98E-09 | Up |
| DRC1 | 44.45005627 | -2.407969046 | 0.283944 | -8.48043 | 2.24E-17 | 9.43E-16 | Down |
| RADIL | 318.3261191 | -1.743322933 | 0.175247 | -9.94782 | 2.58E-23 | 2.17E-21 | Down |
| SSX8P | 3.276837369 | 1.245715389 | 0.332414 | 3.747484 | 0.000179 | 0.000848 | Up |
| EXTL1 | 52.67554578 | 1.162755499 | 0.181624 | 6.401978 | 1.53E-10 | 2.47E-09 | Up |
| TRIM63 | 53.93548183 | 2.35918985 | 0.458415 | 5.146402 | 2.66E-07 | 2.39E-06 | Up |
| GRHL3 | 106.6082737 | 4.565479388 | 0.29014 | 15.73545 | 8.64E-56 | 2.16E-52 | Up |
| NLRP14 | 18.68967582 | -1.373518558 | 0.23892 | -5.74885 | 8.98E-09 | 1.06E-07 | Down |
| TENT5B | 991.2432847 | -1.530763846 | 0.268642 | -5.69815 | 1.21E-08 | 1.40E-07 | Down |
| CLSTN2 | 399.7364867 | -1.21296399 | 0.235552 | -5.14945 | 2.61E-07 | 2.35E-06 | Down |
| RHBDL2 | 72.26964984 | 1.068936632 | 0.111907 | 9.552037 | 1.27E-21 | 8.87E-20 | Up |
| AUTS2 | 1370.910361 | 1.256965036 | 0.164783 | 7.628012 | 2.38E-14 | 6.86E-13 | Up |
| SHROOM4 | 1950.365773 | 2.678725336 | 0.180333 | 14.85432 | 6.52E-50 | 8.74E-47 | Up |
| CD1A | 223.7672707 | 4.594403093 | 0.508999 | 9.026348 | 1.77E-19 | 9.93E-18 | Up |
| SNX29P1 | 4.984573634 | -1.808614335 | 0.399222 | -4.53035 | 5.89E-06 | 3.98E-05 | Down |
| CD1B | 35.28144643 | 1.862995713 | 0.491998 | 3.786592 | 0.000153 | 0.00074 | Up |
| CD1E | 192.3087852 | 1.363291066 | 0.43665 | 3.122163 | 0.001795 | 0.006431 | Up |
| POM121L2 | 1.591579164 | -1.21210733 | 0.451251 | -2.68611 | 0.007229 | 0.02139 | Down |
| PFKFB1 | 41.73663613 | -1.020153479 | 0.196883 | -5.18151 | 2.20E-07 | 2.01E-06 | Down |
| ALAS2 | 16.41573488 | -1.161544168 | 0.378194 | -3.0713 | 0.002131 | 0.007461 | Down |
| PAGE5 | 1.14652401 | -1.727760295 | 0.634494 | -2.72305 | 0.006468 | 0.019434 | Down |
| HTR6 | 1.625637388 | -1.173495401 | 0.403438 | -2.90874 | 0.003629 | 0.01183 | Down |
| ITLN2 | 2.450147568 | -1.991371757 | 0.467709 | -4.25771 | 2.07E-05 | 0.000123 | Down |
| CDA | 156.0642003 | 1.681278706 | 0.210549 | 7.98523 | 1.40E-15 | 4.75E-14 | Up |
| FCER1G | 1114.683707 | 1.018489009 | 0.322619 | 3.156936 | 0.001594 | 0.005812 | Up |
| APOA2 | 3.661664326 | 4.296711805 | 0.63264 | 6.791713 | 1.11E-11 | 2.16E-10 | Up |
| WFDC8 | 7.606004541 | -2.040623426 | 0.352285 | -5.79253 | 6.93E-09 | 8.30E-08 | Down |
| WNT9B | 12.62449565 | 1.365719554 | 0.257727 | 5.299097 | 1.16E-07 | 1.12E-06 | Up |
| SV2A | 933.8541941 | 1.108581568 | 0.188019 | 5.896128 | 3.72E-09 | 4.69E-08 | Up |
| LAD1 | 1332.138194 | 2.361634573 | 0.189983 | 12.43075 | 1.78E-35 | 4.57E-33 | Up |
| TNNI1 | 152.7794077 | 1.531142801 | 0.166744 | 9.182614 | 4.21E-20 | 2.51E-18 | Up |
| C1QC | 3741.476425 | 1.099640154 | 0.354272 | 3.103942 | 0.00191 | 0.006786 | Up |
| RCAN1 | 7143.942557 | -1.312476625 | 0.28781 | -4.56022 | 5.11E-06 | 3.50E-05 | Down |
| RUNX1 | 1512.558584 | 2.220098529 | 0.27178 | 8.168727 | 3.12E-16 | 1.15E-14 | Up |
| AC005041.1 | 659.6482142 | 1.207238177 | 0.137199 | 8.799208 | 1.38E-18 | 6.75E-17 | Up |
| ACTC1 | 47.05466267 | -1.100770163 | 0.339149 | -3.24568 | 0.001172 | 0.004438 | Down |
| CLDN14 | 4.199347617 | 1.442347938 | 0.527764 | 2.732939 | 0.006277 | 0.018947 | Up |
| PADI4 | 9.378124422 | -1.01903351 | 0.418143 | -2.43705 | 0.014808 | 0.039038 | Down |
| CELF3 | 4.706861153 | -1.477095927 | 0.573465 | -2.57574 | 0.010003 | 0.028115 | Down |
| DRC7 | 15.81046033 | -1.794329405 | 0.217945 | -8.23293 | 1.83E-16 | 6.94E-15 | Down |
| TEPP | 438.1407876 | 1.244539744 | 0.258899 | 4.807051 | 1.53E-06 | 1.17E-05 | Up |
| ANKRD18CP | 17.35773999 | -1.239399475 | 0.231323 | -5.35788 | 8.42E-08 | 8.33E-07 | Down |
| AGRP | 3.478957351 | 1.158462216 | 0.272239 | 4.25532 | 2.09E-05 | 0.000124 | Up |
| ABR | 4224.431978 | 1.405396368 | 0.135362 | 10.38249 | 2.98E-25 | 3.09E-23 | Up |
| TNFRSF13C | 493.6415951 | -1.881949415 | 0.609024 | -3.09011 | 0.002001 | 0.007063 | Down |
| TFF3 | 41155.25623 | -2.174218618 | 0.360761 | -6.02676 | 1.67E-09 | 2.25E-08 | Down |
| TFF2 | 97.25587739 | -2.727890224 | 0.352429 | -7.74026 | 9.92E-15 | 3.03E-13 | Down |
| TFF1 | 66.25109163 | -2.463308023 | 0.379868 | -6.48464 | 8.89E-11 | 1.50E-09 | Down |
| TMPRSS3 | 618.8536845 | -1.233821636 | 0.195826 | -6.30059 | 2.97E-10 | 4.55E-09 | Down |
| PDE9A | 1148.169647 | 1.599847088 | 0.148895 | 10.74483 | 6.27E-27 | 7.76E-25 | Up |
| AIRE | 5.895347344 | -1.229738806 | 0.489669 | -2.51136 | 0.012027 | 0.032814 | Down |
| S100B | 331.0565799 | 2.588914438 | 0.387957 | 6.673198 | 2.50E-11 | 4.61E-10 | Up |
| FCN2 | 8.250275944 | -1.137166494 | 0.339812 | -3.34645 | 0.000819 | 0.00325 | Down |
| BRSK1 | 92.38949463 | 1.236159228 | 0.215986 | 5.723323 | 1.04E-08 | 1.22E-07 | Up |
| COX6B2 | 4.97117888 | 1.142858776 | 0.273214 | 4.183013 | 2.88E-05 | 0.000166 | Up |
| NLRP4 | 31.72924788 | -3.548695595 | 0.830841 | -4.27121 | 1.94E-05 | 0.000117 | Down |
| TLCD1 | 362.1284771 | 1.089385002 | 0.138318 | 7.875963 | 3.38E-15 | 1.10E-13 | Up |
| S100A1 | 11913.25404 | 1.592124568 | 0.210578 | 7.560733 | 4.01E-14 | 1.11E-12 | Up |
| CXCR5 | 28.16218445 | -1.896387225 | 0.649655 | -2.91907 | 0.003511 | 0.011505 | Down |
| PAQR6 | 153.5174047 | 1.333233588 | 0.17282 | 7.714579 | 1.21E-14 | 3.66E-13 | Up |
| FCRL3 | 333.0625997 | -1.535877483 | 0.544221 | -2.82216 | 0.00477 | 0.014976 | Down |
| HK3 | 238.4004066 | 1.265248824 | 0.362844 | 3.487033 | 0.000488 | 0.002067 | Up |
| LY6K | 28.22193645 | -1.346210678 | 0.329705 | -4.08307 | 4.44E-05 | 0.000246 | Down |
| IER2 | 25261.09391 | -1.415456447 | 0.205361 | -6.89253 | 5.48E-12 | 1.12E-10 | Down |
| LY6E | 6533.70176 | 1.646835035 | 0.216346 | 7.612027 | 2.70E-14 | 7.68E-13 | Up |
| PTGER1 | 10.45120176 | 2.849286601 | 0.326799 | 8.718787 | 2.81E-18 | 1.33E-16 | Up |
| COL26A1 | 37.39616503 | 1.308818714 | 0.305063 | 4.290326 | 1.78E-05 | 0.000108 | Up |
| FOXH1 | 3.441374194 | 1.087217365 | 0.26603 | 4.086825 | 4.37E-05 | 0.000242 | Up |
| MGAT4B | 6973.554171 | 1.057661737 | 0.088555 | 11.94349 | 7.02E-33 | 1.49E-30 | Up |
| SCGB3A1 | 92.26781606 | 1.211383596 | 0.33723 | 3.592164 | 0.000328 | 0.001454 | Up |
| AC008132.1 | 5.328558196 | 1.198079756 | 0.338855 | 3.535673 | 0.000407 | 0.001759 | Up |
| NPHS1 | 54.11216477 | -2.300024422 | 0.709048 | -3.24382 | 0.001179 | 0.004462 | Down |
| PLXDC1 | 538.1849766 | 1.494880969 | 0.211336 | 7.073493 | 1.51E-12 | 3.39E-11 | Up |
| GRIN2C | 3280.989693 | -1.22322516 | 0.268189 | -4.56106 | 5.09E-06 | 3.49E-05 | Down |
| KLHL10 | 28.03075158 | -1.159892897 | 0.183495 | -6.32113 | 2.60E-10 | 4.02E-09 | Down |
| CCDC155 | 3.266151769 | 1.048356135 | 0.385325 | 2.720703 | 0.006514 | 0.019552 | Up |
| FAM171A2 | 487.2789462 | 1.600422997 | 0.180727 | 8.855487 | 8.33E-19 | 4.21E-17 | Up |
| PLCD3 | 2789.468601 | 3.022283197 | 0.205758 | 14.68857 | 7.63E-49 | 9.55E-46 | Up |
| AQP5 | 735.6383949 | 2.575089111 | 0.306707 | 8.395923 | 4.62E-17 | 1.88E-15 | Up |
| IP6K3 | 1091.678775 | -1.039265987 | 0.25758 | -4.03474 | 5.47E-05 | 0.000296 | Down |
| CXCL16 | 2460.446958 | 1.037396661 | 0.156364 | 6.634481 | 3.26E-11 | 5.89E-10 | Up |
| FGF11 | 96.04652381 | -1.1376283 | 0.201079 | -5.65763 | 1.53E-08 | 1.73E-07 | Down |
| CCDC78 | 70.86755549 | 1.103528577 | 0.265337 | 4.158967 | 3.20E-05 | 0.000182 | Up |
| HS3ST6 | 8.168136449 | 2.563120228 | 0.413334 | 6.20109 | 5.61E-10 | 8.13E-09 | Up |
| BICDL2 | 771.7733629 | 1.132581714 | 0.152842 | 7.410124 | 1.26E-13 | 3.27E-12 | Up |
| PAQR4 | 280.6643905 | 1.369852119 | 0.174907 | 7.831896 | 4.81E-15 | 1.53E-13 | Up |
| SHANK2 | 2615.386553 | -1.462131107 | 0.175692 | -8.32211 | 8.64E-17 | 3.46E-15 | Down |
| FGF19 | 0.520360381 | 2.623194758 | 0.967163 | 2.712258 | 0.006683 | 0.019992 | Up |
| CYP4A22 | 1.089056323 | 1.227007703 | 0.414147 | 2.962737 | 0.003049 | 0.010183 | Up |
| PDZK1IP1 | 1181.115154 | 3.868913314 | 0.404553 | 9.563431 | 1.14E-21 | 7.99E-20 | Up |
| SLC1A7 | 73.78561901 | -1.538586019 | 0.279494 | -5.5049 | 3.69E-08 | 3.89E-07 | Down |
| FAM151A | 71.03358442 | -1.047360368 | 0.155067 | -6.75426 | 1.44E-11 | 2.76E-10 | Down |
| CTRC | 10.24098919 | 1.042478559 | 0.330687 | 3.152465 | 0.001619 | 0.005886 | Up |
| DRAXIN | 110.5548192 | 3.861783374 | 0.293454 | 13.15974 | 1.50E-39 | 6.31E-37 | Up |
| DHRS3 | 14954.24067 | 1.660060675 | 0.167591 | 9.905433 | 3.94E-23 | 3.25E-21 | Up |
| SDC3 | 4977.325559 | 1.291634527 | 0.152046 | 8.495035 | 1.98E-17 | 8.38E-16 | Up |
| CAMK2N1 | 3438.449588 | 3.138157265 | 0.241994 | 12.9679 | 1.86E-38 | 6.71E-36 | Up |
| ALPL | 715.0298918 | 1.571755507 | 0.170824 | 9.201046 | 3.54E-20 | 2.12E-18 | Up |
| TTLL10 | 15.43938738 | 1.076227591 | 0.226158 | 4.758732 | 1.95E-06 | 1.46E-05 | Up |
| MXRA8 | 7633.697807 | 2.171838493 | 0.174052 | 12.47812 | 9.83E-36 | 2.63E-33 | Up |
| DIRAS3 | 811.0494837 | 2.12223808 | 0.233643 | 9.083268 | 1.05E-19 | 6.03E-18 | Up |
| C1orf87 | 0.920299031 | -1.393576771 | 0.497513 | -2.80109 | 0.005093 | 0.015864 | Down |
| NEXN | 865.085494 | -1.005827391 | 0.169738 | -5.92578 | 3.11E-09 | 3.98E-08 | Down |
| DNAJB4 | 4473.632063 | -1.126628396 | 0.180555 | -6.23981 | 4.38E-10 | 6.51E-09 | Down |
| NTNG1 | 21.95527021 | -2.135692926 | 0.328367 | -6.50398 | 7.82E-11 | 1.33E-09 | Down |
| AKNAD1 | 6.902514855 | -1.556922669 | 0.262591 | -5.92908 | 3.05E-09 | 3.91E-08 | Down |
| HFM1 | 184.5983517 | -1.338993053 | 0.181634 | -7.37193 | 1.68E-13 | 4.30E-12 | Down |
| BRINP3 | 75.5781992 | -1.118267029 | 0.260819 | -4.28752 | 1.81E-05 | 0.000109 | Down |
| LMX1A | 9.64274517 | -1.672989431 | 0.303294 | -5.51606 | 3.47E-08 | 3.67E-07 | Down |
| LRRC52 | 10.80537201 | 2.366706511 | 0.351214 | 6.738654 | 1.60E-11 | 3.04E-10 | Up |
| FAM71A | 1.496785883 | -1.328598817 | 0.538441 | -2.46749 | 0.013606 | 0.036391 | Down |
| ATF3 | 17164.0888 | -1.10647301 | 0.369437 | -2.99503 | 0.002744 | 0.009291 | Down |
| TDRD5 | 93.09950743 | -1.077524255 | 0.164658 | -6.54401 | 5.99E-11 | 1.04E-09 | Down |
| C1orf115 | 6073.576466 | 1.508487224 | 0.166932 | 9.036553 | 1.62E-19 | 9.08E-18 | Up |
| KLHDC8A | 1010.98255 | 4.498210672 | 0.365809 | 12.2966 | 9.45E-35 | 2.27E-32 | Up |
| FCMR | 966.8522329 | -1.287320574 | 0.409737 | -3.14182 | 0.001679 | 0.006068 | Down |
| FCAMR | 221.0504608 | -2.203988733 | 0.869129 | -2.53586 | 0.011217 | 0.030911 | Down |
| LRRTM1 | 295.5716434 | 1.303254179 | 0.444278 | 2.93342 | 0.003353 | 0.011059 | Up |
| FAM84A | 777.1226496 | 2.511999354 | 0.17545 | 14.31748 | 1.70E-46 | 1.52E-43 | Up |
| ANKRD30BL | 6.359626635 | -1.683811029 | 0.319663 | -5.26746 | 1.38E-07 | 1.31E-06 | Down |
| EN1 | 7.801590628 | 1.725930632 | 0.376496 | 4.584197 | 4.56E-06 | 3.16E-05 | Up |
| SPATA18 | 335.0665841 | 1.58404925 | 0.243065 | 6.516985 | 7.17E-11 | 1.23E-09 | Up |
| CFAP221 | 353.6176438 | -1.033475873 | 0.151424 | -6.82503 | 8.79E-12 | 1.74E-10 | Down |
| INHBB | 4295.977157 | 1.547783488 | 0.194729 | 7.948415 | 1.89E-15 | 6.32E-14 | Up |
| HPGDS | 82.49005155 | 1.642978005 | 0.236281 | 6.953493 | 3.56E-12 | 7.56E-11 | Up |
| BNIPL | 689.5198783 | 1.7057762 | 0.208937 | 8.164054 | 3.24E-16 | 1.19E-14 | Up |
| C1QTNF7 | 373.2585161 | -1.34680043 | 0.226593 | -5.94369 | 2.79E-09 | 3.60E-08 | Down |
| CDC42EP3 | 2451.307512 | 1.559114822 | 0.177325 | 8.792418 | 1.46E-18 | 7.13E-17 | Up |
| S100A11 | 11165.81226 | 1.4557235 | 0.161736 | 9.000626 | 2.24E-19 | 1.23E-17 | Up |
| IVL | 96.80008291 | 5.023897301 | 0.735957 | 6.826345 | 8.71E-12 | 1.73E-10 | Up |
| SPRR2D | 0.718368491 | 2.562375187 | 0.826187 | 3.101446 | 0.001926 | 0.006837 | Up |
| TGFA | 2630.257274 | 2.504950919 | 0.157296 | 15.92506 | 4.25E-57 | 1.45E-53 | Up |
| ALPP | 0.981552812 | 1.224958935 | 0.51122 | 2.396147 | 0.016568 | 0.042842 | Up |
| GABRB1 | 5.305060733 | -1.67889428 | 0.30984 | -5.41859 | 6.01E-08 | 6.13E-07 | Down |
| DAPL1 | 2020.010034 | 1.339988532 | 0.26879 | 4.985261 | 6.19E-07 | 5.16E-06 | Up |
| CLDN1 | 10064.22893 | 2.893936527 | 0.331806 | 8.721776 | 2.74E-18 | 1.30E-16 | Up |
| DCST1 | 16.89561312 | 1.018113525 | 0.199182 | 5.111483 | 3.20E-07 | 2.83E-06 | Up |
| COL6A3 | 9450.328459 | 1.602292785 | 0.258632 | 6.195271 | 5.82E-10 | 8.41E-09 | Up |
| INAVA | 83.74689474 | 3.797091424 | 0.374863 | 10.12928 | 4.10E-24 | 3.81E-22 | Up |
| LMOD1 | 6695.14815 | -1.862081882 | 0.224099 | -8.30921 | 9.63E-17 | 3.83E-15 | Down |
| ELF3 | 1458.521192 | 1.725279638 | 0.254821 | 6.770559 | 1.28E-11 | 2.48E-10 | Up |
| PDCL2 | 2.163861965 | 3.012203314 | 0.51762 | 5.819335 | 5.91E-09 | 7.18E-08 | Up |
| TRIM46 | 47.34592287 | 1.614686073 | 0.283748 | 5.690567 | 1.27E-08 | 1.46E-07 | Up |
| TMEM79 | 556.5177528 | 1.837446584 | 0.143788 | 12.77882 | 2.15E-37 | 6.91E-35 | Up |
| ADORA1 | 1175.594931 | 2.662521284 | 0.182085 | 14.62241 | 2.02E-48 | 2.23E-45 | Up |
| NEK10 | 52.24094363 | -1.34308202 | 0.147706 | -9.09291 | 9.64E-20 | 5.55E-18 | Down |
| IHH | 23.25445153 | -3.573861053 | 0.440588 | -8.11158 | 5.00E-16 | 1.79E-14 | Down |
| FCRL1 | 177.6935795 | -2.067206419 | 0.675972 | -3.05812 | 0.002227 | 0.00775 | Down |
| NUAK2 | 3197.123397 | -1.062417088 | 0.16631 | -6.38818 | 1.68E-10 | 2.69E-09 | Down |
| EFHB | 125.9994318 | -1.003466278 | 0.194255 | -5.16572 | 2.40E-07 | 2.17E-06 | Down |
| ADAMTS9 | 2342.187362 | 2.789154459 | 0.202155 | 13.7971 | 2.65E-43 | 1.66E-40 | Up |
| PTX3 | 134.4042037 | -1.127301565 | 0.150988 | -7.46615 | 8.26E-14 | 2.21E-12 | Down |
| DCLK3 | 31.35245344 | 1.935382318 | 0.225292 | 8.590566 | 8.65E-18 | 3.81E-16 | Up |
| PCOLCE2 | 369.1812643 | -2.207536436 | 0.293947 | -7.50997 | 5.91E-14 | 1.61E-12 | Down |
| CXCL5 | 22.6765171 | 4.145848642 | 0.445496 | 9.306141 | 1.33E-20 | 8.39E-19 | Up |
| PPBP | 31.70753039 | 1.587207448 | 0.394275 | 4.02564 | 5.68E-05 | 0.000306 | Up |
| PF4 | 9.17421463 | -1.120649544 | 0.338806 | -3.30764 | 0.000941 | 0.00367 | Down |
| CXCL1 | 34.45637718 | 1.185221369 | 0.338803 | 3.498261 | 0.000468 | 0.001992 | Up |
| CPA3 | 67.87217633 | 2.38508887 | 0.358247 | 6.657659 | 2.78E-11 | 5.09E-10 | Up |
| UCN | 57.6869537 | 1.348053768 | 0.260192 | 5.180996 | 2.21E-07 | 2.02E-06 | Up |
| SLC6A20 | 228.2367982 | 5.641412334 | 0.445051 | 12.67589 | 8.04E-37 | 2.47E-34 | Up |
| RTP3 | 13.50100104 | -1.382097174 | 0.21885 | -6.31527 | 2.70E-10 | 4.17E-09 | Down |
| FBXO40 | 2.47888141 | -1.58347709 | 0.504375 | -3.13948 | 0.001692 | 0.006111 | Down |
| GRIK3 | 65.81858088 | 1.901430414 | 0.27687 | 6.867587 | 6.53E-12 | 1.32E-10 | Up |
| DNALI1 | 2896.785876 | -1.055272003 | 0.138794 | -7.60313 | 2.89E-14 | 8.18E-13 | Down |
| KLF15 | 719.401839 | -1.328137881 | 0.236121 | -5.62481 | 1.86E-08 | 2.06E-07 | Down |
| CAMK2N2 | 19.28798446 | 3.193410356 | 0.280681 | 11.37735 | 5.42E-30 | 9.18E-28 | Up |
| LIPH | 1387.061747 | 5.387478097 | 0.403039 | 13.36715 | 9.41E-41 | 4.47E-38 | Up |
| MELTF | 1161.494204 | 1.667256439 | 0.222554 | 7.491483 | 6.81E-14 | 1.84E-12 | Up |
| SPRY1 | 4897.148793 | 1.327217596 | 0.155587 | 8.530404 | 1.46E-17 | 6.23E-16 | Up |
| MST1R | 482.0785386 | 1.519299024 | 0.180996 | 8.394083 | 4.70E-17 | 1.91E-15 | Up |
| NPY5R | 28.50553516 | -1.447453121 | 0.263438 | -5.49447 | 3.92E-08 | 4.12E-07 | Down |
| HHIP | 69.71845883 | -1.826542463 | 0.306709 | -5.95529 | 2.60E-09 | 3.38E-08 | Down |
| ITGA2 | 1811.759858 | 2.586679105 | 0.222388 | 11.63137 | 2.85E-31 | 5.48E-29 | Up |
| SLC45A2 | 5.791192737 | 2.080760399 | 0.393469 | 5.28825 | 1.23E-07 | 1.18E-06 | Up |
| EDIL3 | 565.4810231 | 1.859480976 | 0.191238 | 9.723386 | 2.40E-22 | 1.82E-20 | Up |
| ZNF474 | 75.43561528 | -1.491151565 | 0.362804 | -4.11007 | 3.96E-05 | 0.000221 | Down |
| RANBP3L | 293.1752725 | -1.231826631 | 0.264956 | -4.64917 | 3.33E-06 | 2.38E-05 | Down |
| ADGRV1 | 1515.561981 | -1.819225277 | 0.242486 | -7.50239 | 6.27E-14 | 1.70E-12 | Down |
| F2RL2 | 73.45779013 | 3.342985344 | 0.344433 | 9.705769 | 2.85E-22 | 2.15E-20 | Up |
| ESM1 | 447.3586342 | 2.019424403 | 0.302595 | 6.673679 | 2.49E-11 | 4.60E-10 | Up |
| TMEM174 | 11.6124046 | -2.644311223 | 0.374861 | -7.0541 | 1.74E-12 | 3.85E-11 | Down |
| FOXQ1 | 1050.459805 | 1.819329789 | 0.256718 | 7.086874 | 1.37E-12 | 3.11E-11 | Up |
| ADGRF2 | 7.377948473 | 3.124138123 | 0.556754 | 5.611339 | 2.01E-08 | 2.22E-07 | Up |
| ACSL6 | 113.2738738 | -1.415542857 | 0.209077 | -6.77045 | 1.28E-11 | 2.48E-10 | Down |
| CSF2 | 29.55804409 | 5.588570988 | 0.453287 | 12.32899 | 6.32E-35 | 1.54E-32 | Up |
| FABP7 | 4.51322872 | -1.852958917 | 0.379549 | -4.882 | 1.05E-06 | 8.35E-06 | Down |
| CITED2 | 27361.23461 | -1.279179138 | 0.17948 | -7.12713 | 1.02E-12 | 2.37E-11 | Down |
| CALHM4 | 1.959249891 | 1.799953231 | 0.476862 | 3.774578 | 0.00016 | 0.000772 | Up |
| TMEM200A | 363.2387086 | 1.327791252 | 0.25341 | 5.239691 | 1.61E-07 | 1.51E-06 | Up |
| IL22RA2 | 14.74358623 | 2.454087326 | 0.698039 | 3.515687 | 0.000439 | 0.00188 | Up |
| IL31RA | 2.555786896 | 1.558691504 | 0.425496 | 3.663237 | 0.000249 | 0.001138 | Up |
| RAET1E | 33.14543601 | 2.208392372 | 0.20383 | 10.83447 | 2.36E-27 | 3.05E-25 | Up |
| PI16 | 1623.90721 | -1.913867844 | 0.266188 | -7.1899 | 6.48E-13 | 1.54E-11 | Down |
| MYOZ3 | 55.73986413 | -1.34650797 | 0.228333 | -5.89713 | 3.70E-09 | 4.67E-08 | Down |
| GPR85 | 37.95958823 | 1.040814699 | 0.193241 | 5.386084 | 7.20E-08 | 7.22E-07 | Up |
| FBXL21P | 5.374003055 | 1.325171072 | 0.441408 | 3.002144 | 0.002681 | 0.009107 | Up |
| KCNK5 | 2566.673943 | 1.231541028 | 0.133248 | 9.242485 | 2.41E-20 | 1.48E-18 | Up |
| KIF6 | 199.5789979 | -1.083232616 | 0.175503 | -6.17216 | 6.74E-10 | 9.61E-09 | Down |
| TEX47 | 4.659162053 | -2.279954053 | 0.361394 | -6.30878 | 2.81E-10 | 4.33E-09 | Down |
| USP49 | 976.543011 | -1.05430401 | 0.127927 | -8.24144 | 1.70E-16 | 6.52E-15 | Down |
| SHH | 4.030351012 | 1.24370983 | 0.383485 | 3.243179 | 0.001182 | 0.004472 | Up |
| COL1A2 | 31278.98097 | 1.8716552 | 0.264926 | 7.064835 | 1.61E-12 | 3.58E-11 | Up |
| SLC35G3 | 2.402716063 | -2.17608895 | 0.614171 | -3.54313 | 0.000395 | 0.001713 | Down |
| TNFRSF11B | 8531.482442 | -1.289435439 | 0.23488 | -5.48977 | 4.02E-08 | 4.22E-07 | Down |
| SBSPON | 345.7596107 | -1.736801642 | 0.162314 | -10.7002 | 1.02E-26 | 1.21E-24 | Down |
| EN2 | 4.931350852 | -2.035839123 | 0.316558 | -6.43117 | 1.27E-10 | 2.07E-09 | Down |
| TMEM184A | 262.855057 | 2.724609123 | 0.23425 | 11.63119 | 2.86E-31 | 5.48E-29 | Up |
| NOS3 | 705.3263805 | 1.117602897 | 0.158379 | 7.056518 | 1.71E-12 | 3.79E-11 | Up |
| CTHRC1 | 535.0715233 | 2.474909104 | 0.286014 | 8.653106 | 5.01E-18 | 2.29E-16 | Up |
| DCSTAMP | 1913.232983 | 5.186433838 | 0.582882 | 8.897911 | 5.69E-19 | 2.96E-17 | Up |
| C9orf24 | 41.59683854 | -1.218788776 | 0.229626 | -5.30771 | 1.11E-07 | 1.07E-06 | Down |
| DIRAS2 | 407.8587411 | -1.622802458 | 0.226005 | -7.1804 | 6.95E-13 | 1.65E-11 | Down |
| MAMDC2 | 862.8317342 | -1.649665547 | 0.230318 | -7.16256 | 7.92E-13 | 1.86E-11 | Down |
| ALDH1A1 | 20717.65822 | -1.082017945 | 0.164647 | -6.57174 | 4.97E-11 | 8.76E-10 | Down |
| SVEP1 | 1081.701875 | -1.261325749 | 0.21437 | -5.88389 | 4.01E-09 | 5.02E-08 | Down |
| LLCFC1 | 1.958798646 | -1.03737816 | 0.369913 | -2.80438 | 0.005041 | 0.015729 | Down |
| TMEM246 | 651.3567302 | -1.305688784 | 0.252938 | -5.16208 | 2.44E-07 | 2.21E-06 | Down |
| CFAP47 | 74.22488075 | -1.816038974 | 0.296845 | -6.11779 | 9.49E-10 | 1.32E-08 | Down |
| RNF183 | 48.80504679 | 3.365272623 | 0.303631 | 11.08343 | 1.51E-28 | 2.17E-26 | Up |
| VEGFD | 22.39342084 | -1.142385521 | 0.194763 | -5.8655 | 4.48E-09 | 5.56E-08 | Down |
| SLITRK5 | 752.9185361 | -2.182483714 | 0.360648 | -6.05156 | 1.43E-09 | 1.94E-08 | Down |
| ARMC3 | 30.21528754 | -3.148851003 | 0.36198 | -8.69896 | 3.35E-18 | 1.57E-16 | Down |
| DEUP1 | 55.61364756 | -1.170820319 | 0.194311 | -6.0255 | 1.69E-09 | 2.26E-08 | Down |
| CLDN2 | 417.3890557 | 3.920923033 | 0.331135 | 11.84085 | 2.40E-32 | 4.98E-30 | Up |
| LRFN5 | 65.34885864 | -1.965709212 | 0.226067 | -8.69526 | 3.46E-18 | 1.62E-16 | Down |
| HEPACAM | 3.169160625 | -1.54267416 | 0.347896 | -4.4343 | 9.24E-06 | 5.99E-05 | Down |
| PKNOX2 | 2038.565383 | -1.162082882 | 0.277167 | -4.19271 | 2.76E-05 | 0.000159 | Down |
| MAGEC3 | 7.743414014 | 1.963171372 | 0.282893 | 6.939637 | 3.93E-12 | 8.27E-11 | Up |
| AL121768.1 | 141.2510355 | -1.404602955 | 0.230107 | -6.10412 | 1.03E-09 | 1.43E-08 | Down |
| AMER2 | 6.354730069 | -1.390799241 | 0.369709 | -3.76187 | 0.000169 | 0.000807 | Down |
| AKR1E2 | 205.9341743 | -1.644451915 | 0.259663 | -6.33302 | 2.40E-10 | 3.75E-09 | Down |
| SSX5 | 14.24464222 | 1.427847978 | 0.30801 | 4.635713 | 3.56E-06 | 2.52E-05 | Up |
| DRGX | 1.304083784 | 1.680661314 | 0.576974 | 2.912889 | 0.003581 | 0.011696 | Up |
| DACT1 | 1087.854706 | -1.1500634 | 0.166311 | -6.91514 | 4.67E-12 | 9.69E-11 | Down |
| SLC18A2 | 18.81423488 | 2.413498918 | 0.31089 | 7.76319 | 8.28E-15 | 2.55E-13 | Up |
| FRMD7 | 1.154414487 | 1.873989521 | 0.588939 | 3.181978 | 0.001463 | 0.005394 | Up |
| RET | 186.8510522 | 1.486211189 | 0.321959 | 4.616152 | 3.91E-06 | 2.75E-05 | Up |
| C12orf50 | 1.756137369 | -1.947163515 | 0.418602 | -4.65158 | 3.29E-06 | 2.36E-05 | Down |
| C10orf82 | 167.9465876 | -1.025561076 | 0.215911 | -4.74991 | 2.04E-06 | 1.52E-05 | Down |
| UBTD1 | 1323.00826 | 1.110115036 | 0.128233 | 8.656994 | 4.84E-18 | 2.21E-16 | Up |
| ANKRD2 | 6.617047586 | 1.3955417 | 0.340651 | 4.096694 | 4.19E-05 | 0.000233 | Up |
| E2F7 | 28.36031495 | 1.235710031 | 0.326845 | 3.780719 | 0.000156 | 0.000755 | Up |
| SMCO2 | 4.013246154 | 1.628610727 | 0.486302 | 3.348969 | 0.000811 | 0.003225 | Up |
| CLMN | 1776.519259 | -1.290684764 | 0.140078 | -9.21402 | 3.14E-20 | 1.90E-18 | Down |
| PDZRN4 | 342.0587975 | 2.515039516 | 0.352105 | 7.142865 | 9.14E-13 | 2.13E-11 | Up |
| HACD1 | 342.4027573 | 1.011098667 | 0.171055 | 5.91095 | 3.40E-09 | 4.32E-08 | Up |
| ABTB2 | 702.0895368 | 2.230871768 | 0.17127 | 13.02544 | 8.77E-39 | 3.38E-36 | Up |
| HTRA1 | 5207.756461 | 1.012721131 | 0.119813 | 8.452487 | 2.85E-17 | 1.19E-15 | Up |
| IL25 | 1.341000689 | -1.322851824 | 0.397112 | -3.33118 | 0.000865 | 0.00341 | Down |
| SPATA19 | 4.001320083 | -1.80257477 | 0.44229 | -4.07555 | 4.59E-05 | 0.000253 | Down |
| AMN | 86.07221064 | -1.28304674 | 0.243235 | -5.27494 | 1.33E-07 | 1.26E-06 | Down |
| SPINT1 | 8703.86837 | 1.469318643 | 0.114161 | 12.87062 | 6.59E-38 | 2.27E-35 | Up |
| AVPR1A | 1549.313741 | -1.499675664 | 0.216205 | -6.93637 | 4.02E-12 | 8.45E-11 | Down |
| ASPG | 119.6045739 | -1.845500099 | 0.238477 | -7.7387 | 1.00E-14 | 3.06E-13 | Down |
| TMEM100 | 450.3187323 | 3.109867367 | 0.288961 | 10.76225 | 5.19E-27 | 6.47E-25 | Up |
| CCDC182 | 0.444272957 | 1.441759286 | 0.580981 | 2.481594 | 0.01308 | 0.035193 | Up |
| NETO1 | 45.87827249 | 1.972707633 | 0.37689 | 5.23417 | 1.66E-07 | 1.55E-06 | Up |
| USP54 | 11940.07563 | -1.052652301 | 0.136614 | -7.70529 | 1.31E-14 | 3.91E-13 | Down |
| C11orf74 | 2343.503661 | -1.357353976 | 0.174843 | -7.76329 | 8.28E-15 | 2.55E-13 | Down |
| SERPINB7 | 3.159395747 | 2.862546169 | 0.598513 | 4.782765 | 1.73E-06 | 1.31E-05 | Up |
| WDR72 | 8892.110301 | -1.38671622 | 0.20049 | -6.91664 | 4.62E-12 | 9.60E-11 | Down |
| CRABP1 | 6061.070364 | -1.046963123 | 0.330847 | -3.1645 | 0.001554 | 0.005685 | Down |
| TMEM130 | 183.6546147 | 3.400093242 | 0.297786 | 11.41789 | 3.40E-30 | 5.94E-28 | Up |
| PRTG | 2760.384751 | -1.30319182 | 0.182587 | -7.13737 | 9.51E-13 | 2.21E-11 | Down |
| MFAP4 | 3634.860701 | -1.51728014 | 0.246724 | -6.14972 | 7.76E-10 | 1.10E-08 | Down |
| BEAN1 | 241.3893344 | 1.921384031 | 0.206867 | 9.288006 | 1.57E-20 | 9.80E-19 | Up |
| CPLX4 | 0.758192783 | -1.327775874 | 0.496204 | -2.67587 | 0.007454 | 0.02193 | Down |
| CDH16 | 5988.491392 | -1.666812318 | 0.325972 | -5.11336 | 3.16E-07 | 2.80E-06 | Down |
| MC4R | 1.447560667 | 2.041937031 | 0.60115 | 3.396719 | 0.000682 | 0.00277 | Up |
| MMP10 | 4.587916473 | 2.470110426 | 0.570023 | 4.333356 | 1.47E-05 | 9.08E-05 | Up |
| HTR3A | 39.57613975 | -1.263438688 | 0.533033 | -2.37028 | 0.017774 | 0.045392 | Down |
| ACSM1 | 92.76820911 | 1.006490925 | 0.169122 | 5.951267 | 2.66E-09 | 3.45E-08 | Up |
| C16orf45 | 1335.343951 | 1.563897679 | 0.141049 | 11.08759 | 1.44E-28 | 2.08E-26 | Up |
| YPEL4 | 129.6379972 | 1.865149365 | 0.20416 | 9.135729 | 6.50E-20 | 3.82E-18 | Up |
| LDHAL6A | 19.31403976 | -1.178143931 | 0.217582 | -5.41471 | 6.14E-08 | 6.25E-07 | Down |
| PLIN1 | 119.0210891 | -1.040040413 | 0.253287 | -4.10617 | 4.02E-05 | 0.000224 | Down |
| ANPEP | 653.6249545 | 1.184839001 | 0.249823 | 4.742705 | 2.11E-06 | 1.57E-05 | Up |
| GPR182 | 46.80264099 | -1.782822243 | 0.247297 | -7.20923 | 5.63E-13 | 1.35E-11 | Down |
| MYO1A | 9.2651987 | 1.820538213 | 0.256014 | 7.111084 | 1.15E-12 | 2.64E-11 | Up |
| OR4D6 | 2.935279554 | 2.865026173 | 0.478215 | 5.991089 | 2.08E-09 | 2.76E-08 | Up |
| NAB2 | 5028.443363 | 1.351545097 | 0.249781 | 5.410918 | 6.27E-08 | 6.37E-07 | Up |
| ELFN2 | 38.61467989 | 3.883576416 | 0.394175 | 9.852411 | 6.69E-23 | 5.34E-21 | Up |
| C15orf48 | 461.1976592 | 1.227636606 | 0.298812 | 4.108386 | 3.98E-05 | 0.000222 | Up |
| SCG5 | 1362.414323 | 2.118581291 | 0.236014 | 8.97652 | 2.79E-19 | 1.51E-17 | Up |
| MS4A6E | 0.667429842 | 1.706482295 | 0.577444 | 2.955232 | 0.003124 | 0.010398 | Up |
| MS4A15 | 3.340850769 | 5.067229326 | 0.632757 | 8.008181 | 1.16E-15 | 4.00E-14 | Up |
| TERB2 | 2.870516238 | -1.543905913 | 0.33658 | -4.58704 | 4.50E-06 | 3.12E-05 | Down |
| B4GALNT2 | 861.90725 | -1.64906919 | 0.324814 | -5.07696 | 3.84E-07 | 3.34E-06 | Down |
| PBX3 | 7752.341126 | 1.332210153 | 0.195637 | 6.809604 | 9.79E-12 | 1.93E-10 | Up |
| PIP5KL1 | 171.8728424 | 1.678666289 | 0.203112 | 8.26472 | 1.40E-16 | 5.42E-15 | Up |
| TMEM92 | 129.5496741 | 6.211755801 | 0.484712 | 12.81536 | 1.35E-37 | 4.47E-35 | Up |
| UGT1A6 | 1.413181596 | -1.667648344 | 0.620334 | -2.68831 | 0.007182 | 0.021271 | Down |
| ISLR2 | 101.7317329 | 1.474377247 | 0.207479 | 7.106136 | 1.19E-12 | 2.73E-11 | Up |
| GPRC5B | 3870.670478 | 1.272787667 | 0.117641 | 10.81929 | 2.79E-27 | 3.58E-25 | Up |
| ART5 | 123.068624 | -1.438428501 | 0.214478 | -6.70664 | 1.99E-11 | 3.71E-10 | Down |
| OR51E2 | 9.929337808 | 1.258861467 | 0.327287 | 3.846356 | 0.00012 | 0.000596 | Up |
| JSRP1 | 159.4066957 | 1.463843727 | 0.450512 | 3.24929 | 0.001157 | 0.004388 | Up |
| FAM129C | 222.4042132 | -2.26477097 | 0.551132 | -4.10931 | 3.97E-05 | 0.000222 | Down |
| CDT1 | 350.1047961 | 1.385926368 | 0.267541 | 5.180233 | 2.22E-07 | 2.03E-06 | Up |
| CYP2S1 | 376.3988359 | 3.215244999 | 0.323 | 9.954323 | 2.41E-23 | 2.04E-21 | Up |
| ANKRD33 | 0.709119446 | 1.602386371 | 0.482744 | 3.319331 | 0.000902 | 0.003539 | Up |
| CDC42EP5 | 330.594802 | 1.53530091 | 0.198705 | 7.726534 | 1.11E-14 | 3.35E-13 | Up |
| C19orf33 | 787.314328 | 1.923813983 | 0.298575 | 6.443318 | 1.17E-10 | 1.93E-09 | Up |
| LY6D | 5.351720384 | 2.672780245 | 0.559689 | 4.775477 | 1.79E-06 | 1.35E-05 | Up |
| SEMA6B | 452.1149859 | 1.38643273 | 0.225658 | 6.143963 | 8.05E-10 | 1.13E-08 | Up |
| NXN | 2076.131219 | 1.061331192 | 0.157529 | 6.737381 | 1.61E-11 | 3.06E-10 | Up |
| GPT | 118.7900498 | 1.066080898 | 0.220054 | 4.844638 | 1.27E-06 | 9.87E-06 | Up |
| TRPV3 | 58.19923776 | 1.046875371 | 0.244681 | 4.278538 | 1.88E-05 | 0.000113 | Up |
| GGT6 | 190.8552059 | 3.019260899 | 0.34408 | 8.774886 | 1.71E-18 | 8.28E-17 | Up |
| KLK1 | 302.6936075 | -1.105620477 | 0.365122 | -3.02808 | 0.002461 | 0.008458 | Down |
| KLK4 | 254.7841631 | -1.810108087 | 0.280522 | -6.45265 | 1.10E-10 | 1.82E-09 | Down |
| KLK2 | 384.87792 | -1.70483983 | 0.326808 | -5.21663 | 1.82E-07 | 1.69E-06 | Down |
| KLK5 | 33.49550576 | 2.662638101 | 0.484702 | 5.493351 | 3.94E-08 | 4.14E-07 | Up |
| KLK6 | 79.84314584 | 6.253856094 | 0.678998 | 9.210413 | 3.25E-20 | 1.96E-18 | Up |
| KLK11 | 454.3817154 | 6.692430348 | 0.58164 | 11.50613 | 1.23E-30 | 2.22E-28 | Up |
| KLK13 | 37.37595165 | 2.6979326 | 0.261146 | 10.33115 | 5.09E-25 | 5.10E-23 | Up |
| KRT80 | 658.1149827 | 1.668618679 | 0.311763 | 5.352202 | 8.69E-08 | 8.57E-07 | Up |
| RCOR2 | 69.91286991 | 1.262952454 | 0.189638 | 6.659801 | 2.74E-11 | 5.02E-10 | Up |
| ANGPTL4 | 1765.225047 | 1.045878234 | 0.221078 | 4.730801 | 2.24E-06 | 1.66E-05 | Up |
| IGFBP6 | 4959.420085 | 2.471467872 | 0.254407 | 9.714604 | 2.61E-22 | 1.98E-20 | Up |
| TEKT1 | 33.02609914 | -2.997963175 | 0.40416 | -7.41775 | 1.19E-13 | 3.11E-12 | Down |
| EVPL | 4441.973388 | 1.184680844 | 0.119356 | 9.925596 | 3.22E-23 | 2.69E-21 | Up |
| GSDMA | 47.74942658 | 2.39664816 | 0.380212 | 6.303452 | 2.91E-10 | 4.47E-09 | Up |
| SOST | 4.363512751 | -2.854167036 | 0.627366 | -4.54944 | 5.38E-06 | 3.66E-05 | Down |
| DNASE1L2 | 212.3288396 | 1.941344875 | 0.243426 | 7.975081 | 1.52E-15 | 5.15E-14 | Up |
| SCARA5 | 1205.866309 | -2.245410626 | 0.323723 | -6.93622 | 4.03E-12 | 8.45E-11 | Down |
| VASN | 4328.965116 | 1.27950604 | 0.148545 | 8.613583 | 7.08E-18 | 3.15E-16 | Up |
| PTF1A | 0.756010219 | -1.555886138 | 0.646119 | -2.40805 | 0.016038 | 0.041683 | Down |
| INSM2 | 0.668681668 | 2.437823132 | 0.581048 | 4.19556 | 2.72E-05 | 0.000158 | Up |
| KCNG4 | 5.811826041 | -3.005653597 | 0.609044 | -4.93504 | 8.01E-07 | 6.53E-06 | Down |
| KLHL30 | 81.67810942 | 1.538977008 | 0.258161 | 5.961312 | 2.50E-09 | 3.26E-08 | Up |
| TNXB | 2379.580079 | -1.131768459 | 0.210683 | -5.3719 | 7.79E-08 | 7.75E-07 | Down |
| BMP1 | 3339.714154 | 1.507478633 | 0.094578 | 15.93902 | 3.40E-57 | 1.27E-53 | Up |
| PHYHIP | 739.2101835 | 2.137833 | 0.181288 | 11.79244 | 4.27E-32 | 8.71E-30 | Up |
| GBX2 | 3.293237272 | 3.68272515 | 0.586253 | 6.281799 | 3.35E-10 | 5.08E-09 | Up |
| SERINC2 | 5161.871206 | 1.585080923 | 0.163405 | 9.700311 | 3.01E-22 | 2.26E-20 | Up |
| COL3A1 | 29619.49058 | 2.264606821 | 0.323824 | 6.99332 | 2.68E-12 | 5.79E-11 | Up |
| DYNLRB2 | 111.3071817 | -1.228757543 | 0.143684 | -8.55182 | 1.21E-17 | 5.25E-16 | Down |
| ADAM29 | 1.361070065 | -1.863802483 | 0.640406 | -2.91035 | 0.00361 | 0.011779 | Down |
| GDNF | 5.348901621 | 2.804175849 | 0.411518 | 6.814221 | 9.48E-12 | 1.87E-10 | Up |
| UGT3A2 | 10.23855384 | 2.302873116 | 0.407285 | 5.654206 | 1.57E-08 | 1.76E-07 | Up |
| LRP1B | 2198.505165 | -1.760932897 | 0.3497 | -5.03555 | 4.76E-07 | 4.06E-06 | Down |
| CA7 | 26.44417351 | 2.510320118 | 0.258081 | 9.726858 | 2.32E-22 | 1.77E-20 | Up |
| FAM178B | 63.14051518 | 4.863165523 | 0.305801 | 15.90305 | 6.04E-57 | 1.74E-53 | Up |
| SEMA4C | 2074.196502 | 1.090048357 | 0.098476 | 11.06913 | 1.77E-28 | 2.54E-26 | Up |
| OR13J1 | 1.023537394 | 1.503074248 | 0.438463 | 3.428049 | 0.000608 | 0.002511 | Up |
| HTR1E | 46.14913382 | -2.253047947 | 0.3441 | -6.54765 | 5.84E-11 | 1.02E-09 | Down |
| SFTPB | 19151.60211 | 4.424023638 | 0.429225 | 10.307 | 6.55E-25 | 6.47E-23 | Up |
| GRM5 | 0.531899551 | 1.816875377 | 0.554847 | 3.274551 | 0.001058 | 0.004065 | Up |
| KLK7 | 686.6290259 | 5.616602075 | 0.486519 | 11.54445 | 7.87E-31 | 1.45E-28 | Up |
| IRS1 | 10007.54428 | -1.048121968 | 0.186115 | -5.63158 | 1.79E-08 | 1.99E-07 | Down |
| ZBBX | 4.508325861 | -1.62733904 | 0.412919 | -3.94106 | 8.11E-05 | 0.000419 | Down |
| ACTBL2 | 23.46612592 | 4.369610518 | 0.417011 | 10.47841 | 1.09E-25 | 1.16E-23 | Up |
| ROR2 | 1634.95479 | -1.003878159 | 0.208863 | -4.8064 | 1.54E-06 | 1.18E-05 | Down |
| AR | 1265.000975 | -1.078148204 | 0.21222 | -5.08033 | 3.77E-07 | 3.28E-06 | Down |
| PCSK9 | 5.114199421 | 2.472339235 | 0.410834 | 6.017861 | 1.77E-09 | 2.37E-08 | Up |
| RAB3B | 12.27062277 | 1.376935887 | 0.337727 | 4.077071 | 4.56E-05 | 0.000252 | Up |
| RSPO1 | 58.63134348 | 3.955581935 | 0.576459 | 6.861865 | 6.80E-12 | 1.37E-10 | Up |
| GPRIN1 | 70.29771557 | 1.856341628 | 0.269162 | 6.896746 | 5.32E-12 | 1.09E-10 | Up |
| STK32A | 1557.236596 | 2.49326208 | 0.180795 | 13.79058 | 2.90E-43 | 1.79E-40 | Up |
| IL1RAPL1 | 21.31748037 | -2.017284334 | 0.45383 | -4.44503 | 8.79E-06 | 5.73E-05 | Down |
| C22orf15 | 9.896645687 | -1.000380441 | 0.237458 | -4.21287 | 2.52E-05 | 0.000147 | Down |
| RNASE2 | 15.7965217 | 1.949926213 | 0.346597 | 5.625917 | 1.85E-08 | 2.05E-07 | Up |
| RNASE3 | 1.141342017 | 1.689831768 | 0.52816 | 3.19947 | 0.001377 | 0.005114 | Up |
| CXCL8 | 272.1373077 | 1.677480009 | 0.335927 | 4.993579 | 5.93E-07 | 4.96E-06 | Up |
| RASSF6 | 842.2414605 | -1.043908195 | 0.298668 | -3.49521 | 0.000474 | 0.002013 | Down |
| SPRR1B | 3.128286854 | 3.578591225 | 0.704528 | 5.07942 | 3.79E-07 | 3.30E-06 | Up |
| SLC38A11 | 95.48879577 | 1.607618304 | 0.257965 | 6.231919 | 4.61E-10 | 6.80E-09 | Up |
| GPR183 | 2219.303778 | -1.115149284 | 0.346041 | -3.2226 | 0.00127 | 0.004766 | Down |
| GJB1 | 21.45922993 | -1.00469907 | 0.380129 | -2.64305 | 0.008216 | 0.023845 | Down |
| BNC1 | 80.91616597 | 3.634383595 | 0.340547 | 10.67219 | 1.37E-26 | 1.61E-24 | Up |
| BCRP2 | 2.926437528 | 1.381075439 | 0.290867 | 4.748141 | 2.05E-06 | 1.53E-05 | Up |
| DRD5 | 0.654888573 | 2.47349711 | 0.741158 | 3.337343 | 0.000846 | 0.003344 | Up |
| NLGN1 | 85.3947559 | -2.365612595 | 0.252744 | -9.35972 | 7.99E-21 | 5.18E-19 | Down |
| LINGO1 | 202.3039639 | 1.379862961 | 0.159749 | 8.637707 | 5.74E-18 | 2.60E-16 | Up |
| TACR3 | 7.357474001 | -1.825540262 | 0.27781 | -6.57119 | 4.99E-11 | 8.79E-10 | Down |
| CTNND2 | 45.63774229 | -1.561219341 | 0.251615 | -6.20481 | 5.48E-10 | 7.95E-09 | Down |
| PYDC1 | 3.730504611 | 1.920787117 | 0.392131 | 4.898327 | 9.67E-07 | 7.74E-06 | Up |
| TM4SF4 | 86.83697436 | 2.857075862 | 0.396585 | 7.204189 | 5.84E-13 | 1.40E-11 | Up |
| TM4SF1 | 6062.057419 | 1.059969898 | 0.233752 | 4.534585 | 5.77E-06 | 3.91E-05 | Up |
| ZFPM2 | 1259.439663 | -1.276410693 | 0.215086 | -5.93443 | 2.95E-09 | 3.79E-08 | Down |
| TAS1R3 | 121.8604206 | 1.026866011 | 0.281372 | 3.649491 | 0.000263 | 0.001195 | Up |
| TMEM154 | 326.5781733 | 1.14205762 | 0.299977 | 3.807145 | 0.000141 | 0.000686 | Up |
| ZPLD1 | 6.771039115 | 1.635400613 | 0.274679 | 5.95387 | 2.62E-09 | 3.40E-08 | Up |
| SERPINA9 | 129.8982849 | -3.712587971 | 0.997015 | -3.7237 | 0.000196 | 0.000922 | Down |
| GPR37L1 | 68.17642646 | -1.141881109 | 0.263456 | -4.33423 | 1.46E-05 | 9.05E-05 | Down |
| NSG2 | 22.53317101 | -1.252208836 | 0.385847 | -3.24535 | 0.001173 | 0.004442 | Down |
| RNF150 | 2097.969939 | -1.792437848 | 0.202208 | -8.86435 | 7.70E-19 | 3.93E-17 | Down |
| ANKK1 | 68.48355158 | -1.014684344 | 0.22702 | -4.46958 | 7.84E-06 | 5.16E-05 | Down |
| ADRA1B | 120.9208799 | 2.022115024 | 0.221186 | 9.142137 | 6.12E-20 | 3.61E-18 | Up |
| FABP6 | 7.046154534 | 1.432833261 | 0.315349 | 4.54364 | 5.53E-06 | 3.76E-05 | Up |
| LGALS9B | 2.161757609 | -1.639999902 | 0.570845 | -2.87293 | 0.004067 | 0.013071 | Down |
| FRMPD2 | 72.53632914 | 1.148733847 | 0.225614 | 5.091586 | 3.55E-07 | 3.11E-06 | Up |
| FOS | 104557.3397 | -1.283021239 | 0.263075 | -4.87702 | 1.08E-06 | 8.54E-06 | Down |
| CST5 | 15.54304095 | 5.566112607 | 0.609492 | 9.132382 | 6.70E-20 | 3.93E-18 | Up |
| CST2 | 26.86976068 | 4.274907354 | 0.541908 | 7.888625 | 3.06E-15 | 9.94E-14 | Up |
| CST1 | 91.31868808 | 7.825615964 | 0.810963 | 9.649784 | 4.93E-22 | 3.61E-20 | Up |
| AC073611.1 | 19.16417385 | 1.121959032 | 0.334046 | 3.358701 | 0.000783 | 0.003128 | Up |
| SEMA3E | 276.3307955 | 1.938073641 | 0.331284 | 5.850183 | 4.91E-09 | 6.05E-08 | Up |
| VSTM2A | 9.988552989 | -3.572358937 | 0.435916 | -8.19507 | 2.50E-16 | 9.34E-15 | Down |
| SDR9C7 | 1.733759011 | 1.366142652 | 0.384908 | 3.549273 | 0.000386 | 0.00168 | Up |
| METTL7B | 1357.702532 | 4.063155964 | 0.230083 | 17.65952 | 8.60E-70 | 1.61E-65 | Up |
| KRT75 | 0.610786333 | 2.277311836 | 0.836491 | 2.722459 | 0.00648 | 0.01946 | Up |
| KISS1 | 4.034807827 | 3.335775261 | 0.389214 | 8.570549 | 1.03E-17 | 4.49E-16 | Up |
| LONRF2 | 6099.924534 | 1.409807427 | 0.245111 | 5.751699 | 8.84E-09 | 1.04E-07 | Up |
| KRT83 | 332.2933892 | -1.044673966 | 0.26181 | -3.9902 | 6.60E-05 | 0.000349 | Down |
| TMC7 | 96.49006348 | 1.218171331 | 0.118611 | 10.27027 | 9.59E-25 | 9.35E-23 | Up |
| CDH2 | 1690.404654 | 2.841787909 | 0.32089 | 8.855963 | 8.30E-19 | 4.21E-17 | Up |
| FOXA3 | 9.932646153 | 2.542728596 | 0.386194 | 6.584073 | 4.58E-11 | 8.10E-10 | Up |
| TTLL6 | 6.56387025 | -1.852764477 | 0.27846 | -6.65361 | 2.86E-11 | 5.23E-10 | Down |
| SYT9 | 173.5774928 | -2.343502579 | 0.205498 | -11.404 | 3.99E-30 | 6.84E-28 | Down |
| KCNS3 | 300.9291175 | 1.421229537 | 0.17329 | 8.201471 | 2.37E-16 | 8.88E-15 | Up |
| GPR37 | 25.75002137 | 2.874904548 | 0.355211 | 8.093506 | 5.80E-16 | 2.06E-14 | Up |
| SDR16C5 | 37.53132513 | 2.103870168 | 0.537277 | 3.915799 | 9.01E-05 | 0.00046 | Up |
| DYDC1 | 1.135301343 | -1.600385062 | 0.474553 | -3.37241 | 0.000745 | 0.002989 | Down |
| LMOD2 | 2.771920306 | -2.208545272 | 0.588071 | -3.75558 | 0.000173 | 0.000825 | Down |
| PSG6 | 0.813156132 | 2.267021257 | 0.652484 | 3.474445 | 0.000512 | 0.002154 | Up |
| MTSS1 | 1323.929394 | 1.051051498 | 0.158487 | 6.631783 | 3.32E-11 | 5.99E-10 | Up |
| OSCAR | 174.7630255 | 1.363445017 | 0.312713 | 4.360046 | 1.30E-05 | 8.15E-05 | Up |
| DCDC1 | 163.4349974 | -1.406036643 | 0.189738 | -7.41043 | 1.26E-13 | 3.27E-12 | Down |
| HAS2 | 288.2237662 | 1.215194156 | 0.413254 | 2.940548 | 0.003276 | 0.010844 | Up |
| PLAC1 | 1.422832619 | 1.011910632 | 0.356423 | 2.839075 | 0.004524 | 0.014332 | Up |
| HS6ST2 | 505.2717569 | 3.742291376 | 0.418832 | 8.935073 | 4.07E-19 | 2.16E-17 | Up |
| FPR2 | 18.56215669 | 1.190229117 | 0.369642 | 3.219947 | 0.001282 | 0.004804 | Up |
| ALK | 72.29793227 | 2.589791033 | 0.208065 | 12.44701 | 1.45E-35 | 3.81E-33 | Up |
| SIGLEC17P | 25.79011031 | 1.768241525 | 0.347304 | 5.091335 | 3.56E-07 | 3.12E-06 | Up |
| OBP2B | 1.622309501 | 2.810763086 | 0.540043 | 5.204698 | 1.94E-07 | 1.79E-06 | Up |
| FUT3 | 27.11853975 | 4.855719227 | 0.46321 | 10.48276 | 1.04E-25 | 1.11E-23 | Up |
| C9orf16 | 2294.96617 | 1.135284724 | 0.164079 | 6.919154 | 4.54E-12 | 9.46E-11 | Up |
| SMR3B | 24.18849945 | 1.832185931 | 0.612609 | 2.990792 | 0.002783 | 0.009403 | Up |
| CDC42BPG | 2387.97829 | 1.052484119 | 0.10543 | 9.982775 | 1.81E-23 | 1.57E-21 | Up |
| LRG1 | 267.1875143 | 2.974888395 | 0.288962 | 10.29509 | 7.41E-25 | 7.30E-23 | Up |
| CHST11 | 1243.192667 | 1.039701415 | 0.204104 | 5.09398 | 3.51E-07 | 3.08E-06 | Up |
| KRT19 | 7185.731001 | 2.26149176 | 0.26841 | 8.425522 | 3.59E-17 | 1.48E-15 | Up |
| KRT15 | 167.6447147 | 5.216352321 | 0.382293 | 13.6449 | 2.17E-42 | 1.21E-39 | Up |
| TPPP | 2622.323893 | -2.035951901 | 0.189731 | -10.7307 | 7.30E-27 | 8.93E-25 | Down |
| APLN | 139.7771672 | 2.418593059 | 0.282927 | 8.54848 | 1.25E-17 | 5.40E-16 | Up |
| KRT13 | 32.89808765 | 1.460046767 | 0.467463 | 3.12334 | 0.001788 | 0.006408 | Up |
| KRT27 | 72.45317103 | 2.262077278 | 0.292752 | 7.726934 | 1.10E-14 | 3.34E-13 | Up |
| SPACA5 | 1.089417571 | -1.1091472 | 0.472722 | -2.3463 | 0.018961 | 0.04788 | Down |
| OR1N2 | 0.811127557 | 2.50348114 | 0.961729 | 2.603105 | 0.009238 | 0.026328 | Up |
| LPAR3 | 25.70040274 | 1.308168346 | 0.305504 | 4.281994 | 1.85E-05 | 0.000112 | Up |
| ECEL1 | 124.0678606 | 1.215201805 | 0.286671 | 4.239017 | 2.25E-05 | 0.000132 | Up |
| SLC25A33 | 793.909838 | -1.303201553 | 0.144872 | -8.99551 | 2.35E-19 | 1.29E-17 | Down |
| ENC1 | 728.3607897 | 1.505259093 | 0.153308 | 9.818551 | 9.37E-23 | 7.39E-21 | Up |
| SPSB1 | 2189.548648 | -1.060864723 | 0.20085 | -5.28188 | 1.28E-07 | 1.22E-06 | Down |
| P2RY6 | 247.8231717 | 1.7078995 | 0.281629 | 6.064363 | 1.32E-09 | 1.81E-08 | Up |
| PLEKHG5 | 795.0954673 | 1.209123146 | 0.116769 | 10.35487 | 3.98E-25 | 4.04E-23 | Up |
| LKAAEAR1 | 5.281868871 | 1.108058254 | 0.338542 | 3.273027 | 0.001064 | 0.004084 | Up |
| ANO5 | 747.9310335 | -1.153649524 | 0.18585 | -6.20741 | 5.39E-10 | 7.84E-09 | Down |
| SPATA46 | 2.801881526 | 1.065555912 | 0.314252 | 3.390764 | 0.000697 | 0.00282 | Up |
| SYCE1 | 27.42366344 | -1.136984606 | 0.372511 | -3.05222 | 0.002272 | 0.007879 | Down |
| BCL2 | 9335.85048 | -1.262789374 | 0.15359 | -8.22182 | 2.00E-16 | 7.59E-15 | Down |
| COL8A2 | 3801.732525 | 1.685187287 | 0.21512 | 7.833707 | 4.74E-15 | 1.51E-13 | Up |
| ANGPTL7 | 8.452639098 | -1.826337409 | 0.32685 | -5.5877 | 2.30E-08 | 2.51E-07 | Down |
| PRND | 13.0029791 | 1.98179702 | 0.392997 | 5.042774 | 4.59E-07 | 3.92E-06 | Up |
| FRMD5 | 305.0575491 | 3.695253761 | 0.327812 | 11.27247 | 1.79E-29 | 2.87E-27 | Up |
| MIR31HG | 58.4288088 | 2.498417518 | 0.248414 | 10.05745 | 8.52E-24 | 7.66E-22 | Up |
| FBXW10 | 40.24238122 | -1.753121792 | 0.199796 | -8.77454 | 1.72E-18 | 8.30E-17 | Down |
| GAP43 | 155.2686983 | 5.315818754 | 0.411716 | 12.91137 | 3.88E-38 | 1.37E-35 | Up |
| EPHX4 | 203.0267321 | 1.044620994 | 0.203337 | 5.137387 | 2.79E-07 | 2.49E-06 | Up |
| CALB2 | 26.68608962 | 2.882210324 | 0.437259 | 6.591534 | 4.35E-11 | 7.71E-10 | Up |
| CCL11 | 1.538943364 | 2.291101512 | 0.56722 | 4.039176 | 5.36E-05 | 0.000291 | Up |
| FRMD3 | 4324.710534 | 2.332238106 | 0.181519 | 12.84843 | 8.78E-38 | 2.97E-35 | Up |
| ID4 | 33403.18385 | -1.034002058 | 0.158163 | -6.53758 | 6.25E-11 | 1.08E-09 | Down |
| AZU1 | 12.40993563 | 3.248586993 | 0.428251 | 7.585706 | 3.31E-14 | 9.28E-13 | Up |
| TPSAB1 | 61.39558791 | 2.148378761 | 0.342033 | 6.28121 | 3.36E-10 | 5.10E-09 | Up |
| C1QTNF4 | 35.72076609 | -1.360212927 | 0.250863 | -5.42213 | 5.89E-08 | 6.02E-07 | Down |
| NEGR1 | 507.2717451 | -1.583932685 | 0.179178 | -8.83999 | 9.57E-19 | 4.78E-17 | Down |
| MACROD2 | 770.4448757 | 2.068800808 | 0.2375 | 8.710727 | 3.02E-18 | 1.42E-16 | Up |
| OR5A1 | 1.135802573 | 3.615276066 | 0.66059 | 5.472796 | 4.43E-08 | 4.62E-07 | Up |
| CLEC12A | 78.83970555 | 1.065085403 | 0.345165 | 3.085732 | 0.002031 | 0.007151 | Up |
| ABCG4 | 83.94844817 | 1.383979991 | 0.18669 | 7.413263 | 1.23E-13 | 3.21E-12 | Up |
| FUT9 | 9.88281508 | 4.577620693 | 0.565956 | 8.088294 | 6.05E-16 | 2.14E-14 | Up |
| MAB21L4 | 17.26829647 | 1.397528413 | 0.481515 | 2.90236 | 0.003704 | 0.012048 | Up |
| AGXT | 0.898938847 | -1.17208599 | 0.501958 | -2.33503 | 0.019542 | 0.049142 | Down |
| ACOT12 | 8.149902675 | 5.062062207 | 0.54406 | 9.30423 | 1.35E-20 | 8.53E-19 | Up |
| CARNS1 | 1966.539444 | 1.39658629 | 0.146561 | 9.529063 | 1.59E-21 | 1.09E-19 | Up |
| FAM170B | 0.795598782 | -1.383737202 | 0.563413 | -2.45599 | 0.01405 | 0.037374 | Down |
| ZMAT3 | 2996.645131 | 1.566217382 | 0.11578 | 13.52752 | 1.08E-41 | 5.53E-39 | Up |
| COL6A5 | 5.750552325 | 2.053331297 | 0.486508 | 4.22055 | 2.44E-05 | 0.000143 | Up |
| CYP7B1 | 533.2650903 | -1.054625814 | 0.183301 | -5.75352 | 8.74E-09 | 1.03E-07 | Down |
| MYEOV | 28.66498707 | 1.751921467 | 0.305143 | 5.741314 | 9.39E-09 | 1.10E-07 | Up |
| ADH6 | 10.33433498 | -1.097138169 | 0.167189 | -6.56225 | 5.30E-11 | 9.29E-10 | Down |
| HPSE2 | 20.07322664 | 2.44107318 | 0.275645 | 8.855849 | 8.30E-19 | 4.21E-17 | Up |
| DEC1 | 1.252235854 | 1.402257015 | 0.403727 | 3.473277 | 0.000514 | 0.002163 | Up |
| RXFP4 | 4.427639114 | 1.295671042 | 0.287485 | 4.50692 | 6.58E-06 | 4.40E-05 | Up |
| RAPH1 | 763.5160551 | -1.033928805 | 0.209766 | -4.92896 | 8.27E-07 | 6.72E-06 | Down |
| MAB21L3 | 11.69072769 | -2.085811757 | 0.308996 | -6.75028 | 1.48E-11 | 2.82E-10 | Down |
| SYT12 | 1626.820327 | 6.282473991 | 0.467547 | 13.43708 | 3.67E-41 | 1.81E-38 | Up |
| C11orf86 | 1.906610952 | 4.701750257 | 0.776407 | 6.055781 | 1.40E-09 | 1.90E-08 | Up |
| LIPM | 8.960153076 | 1.44088935 | 0.466101 | 3.091367 | 0.001992 | 0.007039 | Up |
| SNCG | 739.1698232 | 1.083226805 | 0.230212 | 4.705348 | 2.53E-06 | 1.85E-05 | Up |
| NDNF | 95.87418689 | -1.047115258 | 0.193878 | -5.40089 | 6.63E-08 | 6.70E-07 | Down |
| OLR1 | 567.7351453 | 1.042680787 | 0.394371 | 2.643909 | 0.008195 | 0.023788 | Up |
| SAA1 | 41.63797983 | 1.845084137 | 0.511956 | 3.603987 | 0.000313 | 0.001397 | Up |
| RNASE11 | 0.569388039 | 3.187876839 | 0.91725 | 3.475473 | 0.00051 | 0.002147 | Up |
| AGR3 | 247.3573089 | -3.178066224 | 0.374161 | -8.49384 | 2.00E-17 | 8.46E-16 | Down |
| TNFRSF10C | 340.5064061 | 2.087546979 | 0.18204 | 11.46753 | 1.92E-30 | 3.40E-28 | Up |
| CSPG4 | 869.431848 | 1.536608738 | 0.165306 | 9.295514 | 1.46E-20 | 9.19E-19 | Up |
| C2orf70 | 126.406189 | -1.109185981 | 0.186661 | -5.94226 | 2.81E-09 | 3.63E-08 | Down |
| PC | 2350.963167 | 1.504800528 | 0.137147 | 10.97217 | 5.20E-28 | 7.12E-26 | Up |
| TRAPPC3L | 26.79960397 | 1.789143435 | 0.314576 | 5.68748 | 1.29E-08 | 1.48E-07 | Up |
| APOBEC4 | 0.688927413 | -1.365085313 | 0.578526 | -2.35959 | 0.018295 | 0.046474 | Down |
| C11orf80 | 565.5863809 | 1.387460168 | 0.145037 | 9.566248 | 1.11E-21 | 7.79E-20 | Up |
| HAP1 | 433.6144606 | -1.33002467 | 0.255583 | -5.20389 | 1.95E-07 | 1.80E-06 | Down |
| AC008736.1 | 73.78421293 | -2.645201262 | 0.381097 | -6.94102 | 3.89E-12 | 8.19E-11 | Down |
| MAR10 | 10.60409942 | 2.844795117 | 0.320354 | 8.880164 | 6.68E-19 | 3.44E-17 | Up |
| DPY19L1 | 1465.30098 | 1.001205176 | 0.110128 | 9.091326 | 9.78E-20 | 5.62E-18 | Up |
| CBX2 | 139.2382258 | 1.157672662 | 0.134294 | 8.620462 | 6.67E-18 | 2.97E-16 | Up |
| SPTBN2 | 402.0487201 | 3.14172473 | 0.240946 | 13.03913 | 7.33E-39 | 2.87E-36 | Up |
| SLCO4C1 | 201.8215713 | 2.1362536 | 0.333772 | 6.40033 | 1.55E-10 | 2.50E-09 | Up |
| SPERT | 1.105809293 | 2.727942275 | 0.773645 | 3.52609 | 0.000422 | 0.001819 | Up |
| TENT5D | 4.936318019 | -1.857571962 | 0.319765 | -5.80918 | 6.28E-09 | 7.58E-08 | Down |
| NWD2 | 70.63276249 | -3.641868374 | 0.396912 | -9.1755 | 4.49E-20 | 2.67E-18 | Down |
| AC020659.1 | 278.7160694 | 1.237257077 | 0.226392 | 5.465112 | 4.63E-08 | 4.82E-07 | Up |
| SELP | 694.7592525 | 1.162683909 | 0.255759 | 4.546015 | 5.47E-06 | 3.72E-05 | Up |
| PHLDA3 | 2352.971322 | 1.643222389 | 0.163016 | 10.08013 | 6.76E-24 | 6.15E-22 | Up |
| DIRC1 | 0.604197144 | 2.144313913 | 0.847889 | 2.529004 | 0.011439 | 0.031443 | Up |
| PODN | 1760.287489 | -1.277508624 | 0.242954 | -5.25824 | 1.45E-07 | 1.37E-06 | Down |
| MIR1-1HG-AS1 | 4.260012201 | -1.053685516 | 0.319951 | -3.29328 | 0.00099 | 0.003841 | Down |
| TRHR | 3.92317911 | 3.112908872 | 0.405837 | 7.670334 | 1.72E-14 | 5.05E-13 | Up |
| STARD6 | 2.274276591 | -1.314984945 | 0.333645 | -3.94127 | 8.11E-05 | 0.000419 | Down |
| ZCCHC12 | 9708.897443 | 6.382103025 | 0.436059 | 14.63586 | 1.66E-48 | 1.95E-45 | Up |
| GALNTL6 | 65.23587941 | 2.795884629 | 0.26447 | 10.57164 | 4.03E-26 | 4.50E-24 | Up |
| LINGO2 | 312.1859089 | -1.753910124 | 0.345039 | -5.08322 | 3.71E-07 | 3.24E-06 | Down |
| GCSAM | 455.1634559 | -1.124098741 | 0.26564 | -4.23166 | 2.32E-05 | 0.000136 | Down |
| MFSD4A | 265.9252759 | 1.339004649 | 0.176283 | 7.595752 | 3.06E-14 | 8.63E-13 | Up |
| KLK15 | 54.54283002 | -1.689560592 | 0.310502 | -5.44138 | 5.29E-08 | 5.44E-07 | Down |
| GOLT1A | 255.2612958 | 3.727773938 | 0.239789 | 15.54608 | 1.69E-54 | 3.53E-51 | Up |
| KY | 506.4394126 | -1.119006523 | 0.205117 | -5.45545 | 4.88E-08 | 5.06E-07 | Down |
| BRSK2 | 150.7779339 | -1.596138104 | 0.264225 | -6.04082 | 1.53E-09 | 2.07E-08 | Down |
| LEP | 3.738173263 | -1.306894229 | 0.418198 | -3.12506 | 0.001778 | 0.006377 | Down |
| WDR49 | 8.858428662 | -1.61644492 | 0.299445 | -5.39813 | 6.73E-08 | 6.79E-07 | Down |
| RIN1 | 240.6009519 | 2.093069341 | 0.18802 | 11.13215 | 8.75E-29 | 1.30E-26 | Up |
| NLRP6 | 26.85328113 | -1.03584464 | 0.420813 | -2.46153 | 0.013834 | 0.036893 | Down |
| CATSPERD | 1.715192851 | -1.139459301 | 0.467532 | -2.43718 | 0.014802 | 0.039031 | Down |
| SEZ6L2 | 768.2378617 | 3.585768909 | 0.267453 | 13.40709 | 5.50E-41 | 2.68E-38 | Up |
| ASPHD1 | 68.96230267 | 3.573893815 | 0.319552 | 11.18407 | 4.88E-29 | 7.54E-27 | Up |
| GPR149 | 0.557284065 | -1.560934321 | 0.559 | -2.79237 | 0.005232 | 0.016232 | Down |
| CD164L2 | 126.3151317 | 3.45610706 | 0.354024 | 9.762351 | 1.63E-22 | 1.26E-20 | Up |
| CHST2 | 3211.034546 | 1.928795111 | 0.175434 | 10.99441 | 4.07E-28 | 5.67E-26 | Up |
| DES | 1067.714525 | -2.095370334 | 0.334165 | -6.27046 | 3.60E-10 | 5.43E-09 | Down |
| RAG2 | 486.9190684 | -1.474418516 | 0.476614 | -3.09353 | 0.001978 | 0.006996 | Down |
| CADM2 | 65.3599456 | -1.610513654 | 0.326036 | -4.93969 | 7.82E-07 | 6.39E-06 | Down |
| HIGD2B | 4.591666664 | -1.150453892 | 0.282928 | -4.06624 | 4.78E-05 | 0.000262 | Down |
| GOLGA8A | 4558.252209 | 1.27075158 | 0.216856 | 5.859883 | 4.63E-09 | 5.73E-08 | Up |
| VWA3A | 25.69367517 | -1.49433956 | 0.245126 | -6.0962 | 1.09E-09 | 1.50E-08 | Down |
| CATSPER1 | 67.08465912 | 3.366176745 | 0.333619 | 10.08989 | 6.12E-24 | 5.62E-22 | Up |
| ANKRD30BP1 | 1.412995886 | 3.548737002 | 0.646588 | 5.488408 | 4.06E-08 | 4.25E-07 | Up |
| CST6 | 3267.608229 | 3.735618947 | 0.342285 | 10.91378 | 9.91E-28 | 1.33E-25 | Up |
| GRAMD2A | 868.5356885 | -1.606418436 | 0.139093 | -11.5492 | 7.45E-31 | 1.38E-28 | Down |
| PROP1 | 1.183684259 | -1.215045708 | 0.4506 | -2.69651 | 0.007007 | 0.02082 | Down |
| LPL | 1186.02092 | 2.623254409 | 0.240548 | 10.90534 | 1.09E-27 | 1.45E-25 | Up |
| DPP10 | 59.59161556 | 1.676066418 | 0.459099 | 3.650777 | 0.000261 | 0.001189 | Up |
| LONRF3 | 1318.237918 | -1.683215472 | 0.248103 | -6.78433 | 1.17E-11 | 2.27E-10 | Down |
| RMI2 | 392.9033273 | 1.157307605 | 0.264519 | 4.375148 | 1.21E-05 | 7.65E-05 | Up |
| TEX26 | 0.907628526 | -1.248119057 | 0.506018 | -2.46655 | 0.013642 | 0.036469 | Down |
| EIF4E1B | 0.64305437 | 1.663968756 | 0.522468 | 3.184822 | 0.001448 | 0.005347 | Up |
| C15orf53 | 1.526859095 | 1.698276133 | 0.424668 | 3.999064 | 6.36E-05 | 0.000338 | Up |
| PRIMA1 | 161.7583455 | -1.242280826 | 0.263589 | -4.71294 | 2.44E-06 | 1.79E-05 | Down |
| SFN | 522.6577161 | 3.240481291 | 0.370276 | 8.751526 | 2.10E-18 | 1.01E-16 | Up |
| ETV4 | 1183.378408 | 3.194719144 | 0.283494 | 11.26908 | 1.87E-29 | 2.97E-27 | Up |
| CREG2 | 5.215264627 | 2.023633441 | 0.288298 | 7.019236 | 2.23E-12 | 4.87E-11 | Up |
| DOK7 | 262.9431706 | 2.750001625 | 0.298401 | 9.215777 | 3.09E-20 | 1.88E-18 | Up |
| FO393415.1 | 2.493807285 | -1.048842885 | 0.434001 | -2.41668 | 0.015663 | 0.040863 | Down |
| KCNE5 | 8.697993097 | 1.20612185 | 0.299687 | 4.024607 | 5.71E-05 | 0.000307 | Up |
| CRYBG2 | 45.44985375 | 2.019673785 | 0.234206 | 8.623483 | 6.49E-18 | 2.90E-16 | Up |
| HSF5 | 10.20926917 | -1.069789158 | 0.402733 | -2.65632 | 0.0079 | 0.023058 | Down |
| CIDEA | 12.0671177 | -2.730113083 | 0.440324 | -6.20023 | 5.64E-10 | 8.17E-09 | Down |
| LRRTM4 | 1.997174107 | -1.555445156 | 0.534873 | -2.90807 | 0.003637 | 0.01185 | Down |
| ACBD7 | 85.95586395 | 1.64347027 | 0.227976 | 7.208958 | 5.64E-13 | 1.36E-11 | Up |
| PRR18 | 18.29766585 | -1.097182078 | 0.195652 | -5.60782 | 2.05E-08 | 2.26E-07 | Down |
| RIMS2 | 175.6985645 | 1.812182993 | 0.321093 | 5.643789 | 1.66E-08 | 1.86E-07 | Up |
| CLEC14A | 1783.215796 | 1.024368944 | 0.13794 | 7.426191 | 1.12E-13 | 2.93E-12 | Up |
| DIRAS1 | 99.08005706 | 1.490129728 | 0.300977 | 4.950983 | 7.38E-07 | 6.06E-06 | Up |
| PRR15 | 2567.032286 | 5.67192899 | 0.39394 | 14.39797 | 5.33E-47 | 5.13E-44 | Up |
| HORMAD2 | 2.747723124 | 2.455257659 | 0.321756 | 7.630815 | 2.33E-14 | 6.72E-13 | Up |
| OR10AB1P | 5.020518832 | -1.173507267 | 0.284008 | -4.13196 | 3.60E-05 | 0.000202 | Down |
| LINC00303 | 1.194535794 | 1.183650617 | 0.368918 | 3.20844 | 0.001335 | 0.004979 | Up |
| TCERG1L | 4563.899985 | 1.480957257 | 0.290286 | 5.101724 | 3.37E-07 | 2.97E-06 | Up |
| BASP1 | 3114.395254 | 1.168139484 | 0.217151 | 5.379385 | 7.47E-08 | 7.47E-07 | Up |
| IRX5 | 13.10944192 | 1.782427148 | 0.312053 | 5.711934 | 1.12E-08 | 1.29E-07 | Up |
| GRIN1 | 13.57429715 | 2.080736117 | 0.340542 | 6.110075 | 9.96E-10 | 1.38E-08 | Up |
| SOX11 | 23.98586456 | 2.904496834 | 0.441634 | 6.576698 | 4.81E-11 | 8.49E-10 | Up |
| TCIM | 4040.269273 | 2.877921449 | 0.270802 | 10.62742 | 2.22E-26 | 2.56E-24 | Up |
| TYMSOS | 16.52908948 | 1.636680109 | 0.29108 | 5.622789 | 1.88E-08 | 2.09E-07 | Up |
| FUT2 | 89.75180833 | 1.205439288 | 0.151692 | 7.946617 | 1.92E-15 | 6.41E-14 | Up |
| MUC20 | 516.0851452 | 1.059995137 | 0.198654 | 5.33588 | 9.51E-08 | 9.32E-07 | Up |
| AP000679.1 | 2.835968105 | 2.357432055 | 0.383842 | 6.141666 | 8.17E-10 | 1.15E-08 | Up |
| LINC00982 | 1138.328973 | -1.087910197 | 0.19051 | -5.71052 | 1.13E-08 | 1.30E-07 | Down |
| RIMKLA | 206.9429118 | -1.20346035 | 0.179408 | -6.70795 | 1.97E-11 | 3.69E-10 | Down |
| B3GNT8 | 195.594721 | 2.002579773 | 0.182053 | 10.99998 | 3.82E-28 | 5.35E-26 | Up |
| LINC01561 | 2.604263002 | 3.38791641 | 0.523459 | 6.472175 | 9.66E-11 | 1.61E-09 | Up |
| GJD4 | 4.655217527 | 1.623871992 | 0.379232 | 4.281997 | 1.85E-05 | 0.000112 | Up |
| KCNA2 | 86.37380914 | -1.007699822 | 0.297995 | -3.3816 | 0.000721 | 0.002902 | Down |
| AC024940.1 | 5.247805945 | -1.136900429 | 0.249894 | -4.54954 | 5.38E-06 | 3.66E-05 | Down |
| AC024940.2 | 25.47771958 | -1.029046882 | 0.325568 | -3.16078 | 0.001573 | 0.005743 | Down |
| LRRN4CL | 166.5910494 | -1.937759871 | 0.254751 | -7.6065 | 2.82E-14 | 7.99E-13 | Down |
| UMODL1 | 4.941410489 | -1.143044789 | 0.318186 | -3.59238 | 0.000328 | 0.001453 | Down |
| CD19 | 562.9867575 | -1.91069455 | 0.62286 | -3.06762 | 0.002158 | 0.007545 | Down |
| ERICH5 | 58.71324023 | 1.045039259 | 0.242293 | 4.313127 | 1.61E-05 | 9.83E-05 | Up |
| GPR4 | 605.2277832 | 1.395395505 | 0.172546 | 8.087101 | 6.11E-16 | 2.16E-14 | Up |
| NHLH2 | 1.382720184 | 2.324609736 | 0.539491 | 4.308898 | 1.64E-05 | 0.0001 | Up |
| JUN | 70228.29267 | -1.659506313 | 0.242899 | -6.83209 | 8.37E-12 | 1.67E-10 | Down |
| SRRM3 | 227.2979061 | 1.18153444 | 0.1496 | 7.897968 | 2.83E-15 | 9.27E-14 | Up |
| CRACR2B | 401.2431147 | 1.240892044 | 0.194332 | 6.385425 | 1.71E-10 | 2.73E-09 | Up |
| CD151 | 13296.03656 | 1.114012949 | 0.113589 | 9.807387 | 1.05E-22 | 8.22E-21 | Up |
| AC011944.1 | 18.52832404 | 2.670309933 | 0.470777 | 5.672132 | 1.41E-08 | 1.61E-07 | Up |
| FAM20C | 2676.560434 | 1.00958299 | 0.150087 | 6.726642 | 1.74E-11 | 3.27E-10 | Up |
| C2orf73 | 81.90716467 | -1.181135322 | 0.177562 | -6.65195 | 2.89E-11 | 5.27E-10 | Down |
| GPR150 | 11.41586005 | 1.304416017 | 0.303894 | 4.292342 | 1.77E-05 | 0.000107 | Up |
| TSPYL6 | 1.16403658 | -1.414186302 | 0.44495 | -3.17831 | 0.001481 | 0.005454 | Down |
| MLF1 | 1398.022742 | -1.409807407 | 0.17534 | -8.04044 | 8.95E-16 | 3.11E-14 | Down |
| ULK4P3 | 11.71318394 | -1.237263271 | 0.274128 | -4.51345 | 6.38E-06 | 4.28E-05 | Down |
| HTR3C | 2.946927984 | -1.8881162 | 0.374518 | -5.04145 | 4.62E-07 | 3.95E-06 | Down |
| GOLGA8Q | 75.48199727 | -1.038956576 | 0.346735 | -2.9964 | 0.002732 | 0.009253 | Down |
| AMER3 | 7.494205491 | -1.111658774 | 0.308185 | -3.60711 | 0.00031 | 0.001383 | Down |
| SPINK6 | 2.150139912 | 3.251421824 | 0.521863 | 6.230414 | 4.65E-10 | 6.87E-09 | Up |
| SH2D4B | 22.49326143 | -1.015903836 | 0.238448 | -4.26048 | 2.04E-05 | 0.000122 | Down |
| SLITRK1 | 1.417496188 | -1.708918652 | 0.463931 | -3.68357 | 0.00023 | 0.001059 | Down |
| FAM230I | 6.526732317 | -1.486233834 | 0.436249 | -3.40685 | 0.000657 | 0.002683 | Down |
| KCNG2 | 5.710937609 | -1.683008531 | 0.31479 | -5.34645 | 8.97E-08 | 8.83E-07 | Down |
| CALML3 | 4.12548588 | 1.529209465 | 0.53785 | 2.843189 | 0.004466 | 0.014171 | Up |
| CCDC185 | 1.411228426 | 2.156873354 | 0.453468 | 4.7564 | 1.97E-06 | 1.48E-05 | Up |
| AC068473.1 | 3.284155903 | -1.333073474 | 0.277961 | -4.79591 | 1.62E-06 | 1.23E-05 | Down |
| RPS3AP5 | 112.7361746 | -1.452852249 | 0.336003 | -4.32393 | 1.53E-05 | 9.42E-05 | Down |
| CTXN1 | 397.0544213 | 2.835363776 | 0.228384 | 12.41489 | 2.17E-35 | 5.46E-33 | Up |
| C5orf64 | 7.732616895 | -1.262472886 | 0.259525 | -4.86455 | 1.15E-06 | 9.04E-06 | Down |
| ERFE | 1376.776834 | 1.404238541 | 0.338401 | 4.149626 | 3.33E-05 | 0.000189 | Up |
| TMEM139 | 1136.91169 | -1.260554717 | 0.225418 | -5.59209 | 2.24E-08 | 2.46E-07 | Down |
| RFLNA | 38.55786286 | 1.655724221 | 0.220705 | 7.501969 | 6.29E-14 | 1.71E-12 | Up |
| SMIM10L2A | 2773.876975 | 1.993135285 | 0.199238 | 10.00381 | 1.47E-23 | 1.28E-21 | Up |
| Z73417.1 | 14.39354101 | 2.834895978 | 0.345605 | 8.202699 | 2.35E-16 | 8.79E-15 | Up |
| C9orf50 | 21.56503502 | 1.483715533 | 0.185615 | 7.993507 | 1.31E-15 | 4.47E-14 | Up |
| ZFP42 | 1.116549003 | 1.528019534 | 0.600096 | 2.546294 | 0.010887 | 0.030168 | Up |
| C12orf42 | 11.53532653 | -1.691456392 | 0.485928 | -3.48088 | 0.0005 | 0.002109 | Down |
| HTR1F | 17.89412846 | 1.512430373 | 0.211137 | 7.163261 | 7.88E-13 | 1.85E-11 | Up |
| C10orf67 | 42.31541464 | 1.588375182 | 0.213091 | 7.453972 | 9.06E-14 | 2.41E-12 | Up |
| LDLRAD3 | 350.5530351 | 1.100877434 | 0.123305 | 8.928083 | 4.33E-19 | 2.29E-17 | Up |
| CDH4 | 268.8297703 | 2.701689901 | 0.304902 | 8.860846 | 7.94E-19 | 4.05E-17 | Up |
| SMCO3 | 61.21806955 | -1.553623071 | 0.244178 | -6.36268 | 1.98E-10 | 3.13E-09 | Down |
| AC234031.1 | 0.800840909 | 1.002922134 | 0.427482 | 2.346113 | 0.01897 | 0.047895 | Up |
| EGR3 | 2524.564205 | -1.183065907 | 0.336129 | -3.51968 | 0.000432 | 0.001856 | Down |
| VWA1 | 4768.042537 | 1.518261649 | 0.169997 | 8.931132 | 4.22E-19 | 2.24E-17 | Up |
| FJX1 | 197.9436558 | 1.597681735 | 0.233123 | 6.853376 | 7.21E-12 | 1.45E-10 | Up |
| AL049647.1 | 1.128206738 | -1.879175091 | 0.538469 | -3.48985 | 0.000483 | 0.002049 | Down |
| MKRN3 | 10.97360814 | -1.376253099 | 0.429724 | -3.20264 | 0.001362 | 0.005067 | Down |
| ALOX12B | 7.321961513 | 1.453060663 | 0.234705 | 6.191 | 5.98E-10 | 8.63E-09 | Up |
| LBX2 | 27.38125534 | 1.538021641 | 0.173613 | 8.858926 | 8.08E-19 | 4.11E-17 | Up |
| SLITRK4 | 194.7608774 | 1.717855589 | 0.240874 | 7.13175 | 9.91E-13 | 2.30E-11 | Up |
| HTR1D | 9.678116023 | 1.519298868 | 0.362948 | 4.185998 | 2.84E-05 | 0.000164 | Up |
| LSMEM2 | 7.325743717 | -1.624010582 | 0.304112 | -5.34018 | 9.29E-08 | 9.11E-07 | Down |
| AP003471.1 | 1.350115726 | -2.903637555 | 0.884072 | -3.28439 | 0.001022 | 0.003945 | Down |
| ALOX15B | 1010.092781 | 2.520549519 | 0.331113 | 7.612363 | 2.69E-14 | 7.66E-13 | Up |
| FCER1A | 256.2250869 | 1.575650123 | 0.366676 | 4.297121 | 1.73E-05 | 0.000105 | Up |
| ARL14 | 175.3522312 | -2.76937618 | 0.582528 | -4.75407 | 1.99E-06 | 1.49E-05 | Down |
| APOBEC3B | 296.3985926 | -1.186902656 | 0.325662 | -3.64459 | 0.000268 | 0.001216 | Down |
| FAM216B | 7.606205047 | -1.640058715 | 0.594574 | -2.75838 | 0.005809 | 0.017736 | Down |
| MYADM | 17206.26522 | -1.126168885 | 0.254212 | -4.43004 | 9.42E-06 | 6.09E-05 | Down |
| GIPC3 | 325.6785017 | 1.045598661 | 0.147076 | 7.109245 | 1.17E-12 | 2.67E-11 | Up |
| C1orf194 | 20.15448191 | -1.922778222 | 0.203214 | -9.46185 | 3.03E-21 | 2.03E-19 | Down |
| B3GNT3 | 692.4563633 | 5.498152061 | 0.418936 | 13.1241 | 2.40E-39 | 9.67E-37 | Up |
| ITLN1 | 24.12775867 | -2.000131595 | 0.39799 | -5.02558 | 5.02E-07 | 4.26E-06 | Down |
| NRXN1 | 44.21788378 | -2.795253739 | 0.292791 | -9.54694 | 1.34E-21 | 9.27E-20 | Down |
| CCR8 | 13.69297457 | 1.889301163 | 0.414379 | 4.559353 | 5.13E-06 | 3.51E-05 | Up |
| LINC00652 | 37.10691943 | -1.005934649 | 0.16413 | -6.12889 | 8.85E-10 | 1.24E-08 | Down |
| SSC5D | 477.8292423 | 1.042546301 | 0.228323 | 4.566106 | 4.97E-06 | 3.41E-05 | Up |
| C3orf80 | 40.17244909 | 1.903554854 | 0.236513 | 8.048424 | 8.39E-16 | 2.92E-14 | Up |
| TMEM150B | 41.40241988 | 1.350091078 | 0.377108 | 3.580117 | 0.000343 | 0.001515 | Up |
| C10orf91 | 13.20749849 | 1.028076267 | 0.215043 | 4.780797 | 1.75E-06 | 1.32E-05 | Up |
| TDRD6 | 650.6675438 | -1.020818194 | 0.212871 | -4.79549 | 1.62E-06 | 1.24E-05 | Down |
| FAR2P1 | 16.40336852 | -1.562550292 | 0.337804 | -4.62561 | 3.74E-06 | 2.64E-05 | Down |
| FAM71C | 1.171271134 | -1.881318294 | 0.487181 | -3.86165 | 0.000113 | 0.000563 | Down |
| HERC2P3 | 137.1695439 | -1.156809636 | 0.303387 | -3.81298 | 0.000137 | 0.000672 | Down |
| LINC01869 | 10.6499243 | -1.026318692 | 0.243334 | -4.21773 | 2.47E-05 | 0.000144 | Down |
| PLD5 | 32.48817304 | -2.236698418 | 0.249631 | -8.96003 | 3.25E-19 | 1.74E-17 | Down |
| PNPLA1 | 8.467363727 | -1.251202418 | 0.260466 | -4.8037 | 1.56E-06 | 1.19E-05 | Down |
| ALX1 | 1.786335104 | -2.480719982 | 0.535794 | -4.62999 | 3.66E-06 | 2.59E-05 | Down |
| KCTD4 | 1.232093318 | -1.865855466 | 0.447036 | -4.17384 | 3.00E-05 | 0.000172 | Down |
| ITPRID1 | 0.851773481 | 1.244777025 | 0.488294 | 2.549236 | 0.010796 | 0.029961 | Up |
| CYP8B1 | 14.07276445 | -1.649842328 | 0.192379 | -8.57599 | 9.82E-18 | 4.29E-16 | Down |
| SERTM1 | 675.5979181 | -1.071431721 | 0.380071 | -2.81903 | 0.004817 | 0.015107 | Down |
| AC022148.1 | 29.45539232 | 2.754431354 | 0.278937 | 9.874729 | 5.36E-23 | 4.35E-21 | Up |
| ZSCAN4 | 12.47531542 | 1.821650567 | 0.270199 | 6.741883 | 1.56E-11 | 2.98E-10 | Up |
| CLRN3 | 4.910471359 | 3.1569352 | 0.393777 | 8.017067 | 1.08E-15 | 3.73E-14 | Up |
| CHST13 | 12.49583459 | 1.307179012 | 0.3189 | 4.099023 | 4.15E-05 | 0.000231 | Up |
| AGTR2 | 1.305894377 | -1.954588466 | 0.644759 | -3.0315 | 0.002433 | 0.008376 | Down |
| ANKRD30B | 15.3272588 | 2.95058946 | 0.344561 | 8.56334 | 1.10E-17 | 4.77E-16 | Up |
| OR51E1 | 39.29045913 | 1.053648672 | 0.212394 | 4.960824 | 7.02E-07 | 5.79E-06 | Up |
| ARSJ | 189.3423589 | 1.162482328 | 0.28362 | 4.098727 | 4.15E-05 | 0.000231 | Up |
| HOXC9 | 7.01375124 | 1.293289465 | 0.273507 | 4.728537 | 2.26E-06 | 1.68E-05 | Up |
| HOXC10 | 2.687076365 | 1.942543266 | 0.605896 | 3.206066 | 0.001346 | 0.005015 | Up |
| OXTR | 48.41702246 | 1.560318495 | 0.148632 | 10.49785 | 8.84E-26 | 9.56E-24 | Up |
| ADIPOQ | 62.34431627 | -2.439935172 | 0.54704 | -4.46025 | 8.19E-06 | 5.37E-05 | Down |
| F2R | 1793.205665 | 1.366608874 | 0.13786 | 9.913016 | 3.65E-23 | 3.03E-21 | Up |
| AC004832.1 | 3.479628293 | -2.48576777 | 0.48743 | -5.09974 | 3.40E-07 | 3.00E-06 | Down |
| HIST3H2A | 105.1913677 | 2.627358905 | 0.282757 | 9.29194 | 1.52E-20 | 9.48E-19 | Up |
| TMEM132C | 73.46854098 | -1.549007166 | 0.290559 | -5.33112 | 9.76E-08 | 9.54E-07 | Down |
| HEPHL1 | 68.66282265 | -1.078915747 | 0.206249 | -5.23112 | 1.68E-07 | 1.57E-06 | Down |
| LRRC75A | 640.668102 | 1.415124897 | 0.126175 | 11.21557 | 3.42E-29 | 5.39E-27 | Up |
| CCL13 | 98.23919241 | 2.532467941 | 0.339907 | 7.450482 | 9.30E-14 | 2.46E-12 | Up |
| UTS2R | 261.1981298 | -1.104793234 | 0.403689 | -2.73674 | 0.006205 | 0.018759 | Down |
| SAGE1 | 1.001221187 | 2.031104446 | 0.679824 | 2.987691 | 0.002811 | 0.009486 | Up |
| TNFSF15 | 429.7591782 | 1.542100787 | 0.192264 | 8.020729 | 1.05E-15 | 3.62E-14 | Up |
| PHLDA2 | 564.5656181 | 1.552035101 | 0.244114 | 6.357825 | 2.05E-10 | 3.22E-09 | Up |
| PLAG1 | 694.5197945 | 1.934973309 | 0.225094 | 8.596283 | 8.23E-18 | 3.64E-16 | Up |
| ADIG | 92.75633132 | -1.022476543 | 0.294818 | -3.46816 | 0.000524 | 0.0022 | Down |
| PTCHD3 | 15.2002739 | -1.332721996 | 0.428407 | -3.11088 | 0.001865 | 0.00665 | Down |
| FAM181B | 172.8942203 | -1.706872209 | 0.192773 | -8.85431 | 8.42E-19 | 4.25E-17 | Down |
| KCNIP1 | 33.89199448 | -2.017593235 | 0.227088 | -8.88462 | 6.41E-19 | 3.32E-17 | Down |
| ZAR1 | 0.955646174 | 1.907181673 | 0.534473 | 3.568341 | 0.000359 | 0.001576 | Up |
| KCNA4 | 1.749062755 | -1.953414319 | 0.481736 | -4.05495 | 5.01E-05 | 0.000274 | Down |
| GABRG3 | 102.6582856 | 2.217916366 | 0.33545 | 6.611766 | 3.80E-11 | 6.80E-10 | Up |
| LIPF | 6.694726318 | -1.634009064 | 0.391692 | -4.17167 | 3.02E-05 | 0.000173 | Down |
| ZNF804B | 745.7371427 | -2.288881377 | 0.376922 | -6.07256 | 1.26E-09 | 1.72E-08 | Down |
| AC138028.1 | 10.37110259 | -1.161654412 | 0.229174 | -5.06888 | 4.00E-07 | 3.46E-06 | Down |
| NXPH4 | 21.55986644 | 1.528856155 | 0.307704 | 4.968602 | 6.74E-07 | 5.58E-06 | Up |
| OR2B8P | 1.455460626 | -1.648816105 | 0.521176 | -3.16364 | 0.001558 | 0.005698 | Down |
| XKRX | 178.5336822 | 3.193702129 | 0.291525 | 10.95515 | 6.28E-28 | 8.54E-26 | Up |
| BGN | 14251.90295 | 1.088292903 | 0.178002 | 6.113943 | 9.72E-10 | 1.35E-08 | Up |
| CAV3 | 2.04928561 | -1.386015805 | 0.497897 | -2.78374 | 0.005374 | 0.016601 | Down |
| RNASE10 | 3.894813243 | 2.631035837 | 0.377505 | 6.969537 | 3.18E-12 | 6.80E-11 | Up |
| EPHB3 | 423.7159911 | 2.920611981 | 0.246401 | 11.85309 | 2.07E-32 | 4.32E-30 | Up |
| SNORC | 41.10297826 | 1.413557962 | 0.142945 | 9.888818 | 4.65E-23 | 3.81E-21 | Up |
| NTM | 329.2369041 | 3.010571949 | 0.276893 | 10.87271 | 1.56E-27 | 2.05E-25 | Up |
| PPP1R27 | 3.722831822 | -1.359009071 | 0.308692 | -4.40247 | 1.07E-05 | 6.83E-05 | Down |
| GALR2 | 7.353724952 | 1.60132716 | 0.390268 | 4.103146 | 4.08E-05 | 0.000227 | Up |
| SLC35D3 | 3.227878092 | 2.147593752 | 0.481988 | 4.455703 | 8.36E-06 | 5.48E-05 | Up |
| PAPPA | 239.5250853 | 1.182269823 | 0.240559 | 4.914687 | 8.89E-07 | 7.17E-06 | Up |
| HCAR2 | 29.54316478 | 1.433505034 | 0.324892 | 4.412244 | 1.02E-05 | 6.56E-05 | Up |
| C1orf116 | 2409.82006 | 1.459142348 | 0.234264 | 6.22863 | 4.71E-10 | 6.94E-09 | Up |
| ADGRG3 | 87.2798708 | -1.317892964 | 0.215763 | -6.10806 | 1.01E-09 | 1.40E-08 | Down |
| AC007685.1 | 4.883912631 | -1.356997697 | 0.312941 | -4.33628 | 1.45E-05 | 8.97E-05 | Down |
| TMEM95 | 1.820499938 | -1.969256466 | 0.602804 | -3.26683 | 0.001088 | 0.004161 | Down |
| TSPEAR-AS2 | 33.42015744 | -1.287071143 | 0.254076 | -5.06569 | 4.07E-07 | 3.52E-06 | Down |
| ODF3L1 | 166.8386473 | -1.720202519 | 0.363359 | -4.73416 | 2.20E-06 | 1.63E-05 | Down |
| GJC1 | 502.073484 | 1.511444142 | 0.137283 | 11.00969 | 3.43E-28 | 4.82E-26 | Up |
| MCEMP1 | 50.95217112 | 4.170655405 | 0.460777 | 9.051354 | 1.41E-19 | 7.98E-18 | Up |
| CAMK1D | 4586.737366 | -1.076528787 | 0.133232 | -8.08014 | 6.47E-16 | 2.28E-14 | Down |
| IGSF5 | 29.99165194 | -1.281047058 | 0.265417 | -4.82654 | 1.39E-06 | 1.07E-05 | Down |
| NKX2-5 | 1.473651104 | -2.001926895 | 0.51582 | -3.88106 | 0.000104 | 0.000524 | Down |
| FREM3 | 3.848217806 | 2.112165292 | 0.378757 | 5.576573 | 2.45E-08 | 2.67E-07 | Up |
| CSMD1 | 157.6277269 | 3.537577232 | 0.350285 | 10.09913 | 5.57E-24 | 5.14E-22 | Up |
| RIPPLY3 | 21.00773539 | 1.931679359 | 0.179737 | 10.74726 | 6.10E-27 | 7.59E-25 | Up |
| ANKRD20A2 | 1.106196641 | -1.535733942 | 0.512948 | -2.99393 | 0.002754 | 0.00932 | Down |
| BX322650.1 | 10.57832982 | 1.136065034 | 0.195381 | 5.814609 | 6.08E-09 | 7.35E-08 | Up |
| CHST6 | 50.05583288 | 2.600211974 | 0.252184 | 10.31078 | 6.30E-25 | 6.25E-23 | Up |
| WT1-AS | 3.12186785 | 1.713962398 | 0.488231 | 3.510557 | 0.000447 | 0.001914 | Up |
| RIMBP3C | 7.33125685 | 1.611802228 | 0.284731 | 5.660793 | 1.51E-08 | 1.70E-07 | Up |
| PRR36 | 468.6377056 | 2.826154809 | 0.193226 | 14.62617 | 1.91E-48 | 2.17E-45 | Up |
| CCBE1 | 48.2969207 | -1.887443432 | 0.243094 | -7.76426 | 8.21E-15 | 2.54E-13 | Down |
| TMEM121B | 34.62110531 | 1.20684613 | 0.242347 | 4.979826 | 6.36E-07 | 5.29E-06 | Up |
| EPHA10 | 206.8160661 | 3.818942094 | 0.297616 | 12.83179 | 1.09E-37 | 3.65E-35 | Up |
| CABCOCO1 | 92.3586657 | -1.013533278 | 0.327637 | -3.09346 | 0.001978 | 0.006997 | Down |
| SYNDIG1L | 67.27411552 | 2.891390886 | 0.37046 | 7.804873 | 5.96E-15 | 1.88E-13 | Up |
| IZUMO1R | 8.318163132 | -1.185313181 | 0.260544 | -4.54938 | 5.38E-06 | 3.66E-05 | Down |
| SERHL2 | 181.5620432 | -1.312338445 | 0.171182 | -7.66632 | 1.77E-14 | 5.21E-13 | Down |
| PGPEP1L | 5.771277013 | -1.948355552 | 0.296422 | -6.57291 | 4.93E-11 | 8.70E-10 | Down |
| TNFAIP8L3 | 230.2953513 | -1.083353233 | 0.17561 | -6.16908 | 6.87E-10 | 9.78E-09 | Down |
| CCR3 | 21.63361864 | 1.859536922 | 0.234138 | 7.942058 | 1.99E-15 | 6.64E-14 | Up |
| DGCR6 | 1143.151449 | 1.928913027 | 0.256197 | 7.529033 | 5.11E-14 | 1.40E-12 | Up |
| PRR32 | 4.092092017 | 5.721713085 | 0.948574 | 6.031913 | 1.62E-09 | 2.18E-08 | Up |
| C11orf88 | 18.93271957 | -1.775142737 | 0.239991 | -7.39672 | 1.40E-13 | 3.61E-12 | Down |
| MAR11 | 5.119406996 | 5.718819508 | 0.609091 | 9.389101 | 6.05E-21 | 3.96E-19 | Up |
| PSG9 | 0.488884905 | 2.276608822 | 0.947336 | 2.403169 | 0.016254 | 0.042153 | Up |
| BMP8A | 1736.099265 | -1.310836148 | 0.382721 | -3.42504 | 0.000615 | 0.002534 | Down |
| MRGPRX2 | 0.512477697 | 2.595717542 | 1.101587 | 2.356343 | 0.018456 | 0.046803 | Up |
| UPP1 | 1518.519917 | 1.627810939 | 0.201081 | 8.095307 | 5.71E-16 | 2.03E-14 | Up |
| OPCML | 187.1161523 | 4.889174031 | 0.422182 | 11.58074 | 5.16E-31 | 9.73E-29 | Up |
| MACC1 | 638.7392265 | 1.079617352 | 0.227207 | 4.751688 | 2.02E-06 | 1.51E-05 | Up |
| ACSM2A | 11.7759232 | -2.438971523 | 0.281368 | -8.66825 | 4.39E-18 | 2.02E-16 | Down |
| KCTD16 | 368.2741106 | -3.240363411 | 0.273762 | -11.8364 | 2.53E-32 | 5.22E-30 | Down |
| B3GALT5 | 456.3651641 | -1.550629893 | 0.298056 | -5.20247 | 1.97E-07 | 1.81E-06 | Down |
| SLC35F3 | 171.3628304 | 1.630121123 | 0.288711 | 5.646212 | 1.64E-08 | 1.84E-07 | Up |
| TUBA8 | 31.73246192 | -1.041453702 | 0.177759 | -5.8588 | 4.66E-09 | 5.76E-08 | Down |
| EMILIN3 | 45.39599188 | 1.718901874 | 0.302226 | 5.687465 | 1.29E-08 | 1.48E-07 | Up |
| ANKRD45 | 37.37310455 | -1.25385676 | 0.248575 | -5.04417 | 4.55E-07 | 3.90E-06 | Down |
| ZNF730 | 17.4673763 | 1.36430362 | 0.250298 | 5.450724 | 5.02E-08 | 5.18E-07 | Up |
| IQGAP3 | 101.5549205 | 1.85165597 | 0.298733 | 6.198367 | 5.71E-10 | 8.26E-09 | Up |
| ARSI | 36.07833607 | 2.221240285 | 0.371619 | 5.977204 | 2.27E-09 | 2.98E-08 | Up |
| SRARP | 85.91918069 | -2.012274305 | 0.314455 | -6.39925 | 1.56E-10 | 2.52E-09 | Down |
| DUSP5P1 | 1.696452048 | -1.254867851 | 0.422403 | -2.97078 | 0.00297 | 0.009952 | Down |
| PRKX | 8616.275187 | -1.41311841 | 0.169075 | -8.35791 | 6.38E-17 | 2.57E-15 | Down |
| KCNH8 | 46.35300172 | -1.341971538 | 0.195783 | -6.8544 | 7.16E-12 | 1.44E-10 | Down |
| NPW | 55.62350572 | 3.864829899 | 0.385801 | 10.01767 | 1.27E-23 | 1.13E-21 | Up |
| TBX1 | 197.7760825 | -1.388043766 | 0.208534 | -6.6562 | 2.81E-11 | 5.14E-10 | Down |
| AC091868.1 | 2.010212402 | -1.124955324 | 0.356324 | -3.15711 | 0.001593 | 0.00581 | Down |
| TREML3P | 1.00125277 | 1.335093526 | 0.448504 | 2.976772 | 0.002913 | 0.009786 | Up |
| RPL11P4 | 0.499841866 | 1.59362772 | 0.573383 | 2.779341 | 0.005447 | 0.016786 | Up |
| KCNQ3 | 544.8474218 | 3.865199869 | 0.246276 | 15.69459 | 1.65E-55 | 3.86E-52 | Up |
| ADRA2C | 116.3275912 | 1.596690616 | 0.274557 | 5.815526 | 6.04E-09 | 7.32E-08 | Up |
| C1QTNF12 | 41.77017681 | 1.881185437 | 0.213886 | 8.795259 | 1.43E-18 | 6.97E-17 | Up |
| DGAT2L6 | 2.623503027 | 2.203141841 | 0.362153 | 6.083455 | 1.18E-09 | 1.62E-08 | Up |
| OLIG1 | 3.523342687 | 1.340455606 | 0.482141 | 2.780217 | 0.005432 | 0.016751 | Up |
| PCDH9 | 489.5249146 | -1.291025586 | 0.257828 | -5.00731 | 5.52E-07 | 4.64E-06 | Down |
| ALDH1A3 | 494.7720274 | 2.337317038 | 0.286731 | 8.1516 | 3.59E-16 | 1.31E-14 | Up |
| HIST2H2AB | 3.898842855 | -1.876129493 | 0.420192 | -4.46494 | 8.01E-06 | 5.26E-05 | Down |
| TACSTD2 | 8167.63189 | 3.755766866 | 0.36707 | 10.23175 | 1.43E-24 | 1.37E-22 | Up |
| CCSER1 | 222.6772343 | -1.35644692 | 0.159783 | -8.4893 | 2.08E-17 | 8.78E-16 | Down |
| MAP7D2 | 23.59816285 | 1.435802379 | 0.226118 | 6.349796 | 2.16E-10 | 3.39E-09 | Up |
| COLEC10 | 92.09502381 | -1.096366558 | 0.242169 | -4.52727 | 5.98E-06 | 4.04E-05 | Down |
| A3GALT2 | 3.581610358 | -1.069364611 | 0.2949 | -3.6262 | 0.000288 | 0.001296 | Down |
| IRS3P | 13.25543532 | 1.694884413 | 0.279467 | 6.064707 | 1.32E-09 | 1.80E-08 | Up |
| CCR10 | 43.56725275 | 1.373555916 | 0.221077 | 6.213021 | 5.20E-10 | 7.59E-09 | Up |
| NCMAP | 914.7308821 | 1.045433581 | 0.168049 | 6.221006 | 4.94E-10 | 7.26E-09 | Up |
| BPIFC | 1.95848765 | -1.372332501 | 0.427113 | -3.21304 | 0.001313 | 0.004907 | Down |
| PTP4A3 | 1669.915199 | 1.768630867 | 0.200905 | 8.803315 | 1.33E-18 | 6.52E-17 | Up |
| PROS1 | 6010.800835 | 3.151580029 | 0.208291 | 15.13065 | 1.02E-51 | 1.59E-48 | Up |
| SLITRK6 | 53.8081384 | 1.614775395 | 0.434717 | 3.714541 | 0.000204 | 0.000951 | Up |
| LPAR5 | 1373.804397 | 3.156263428 | 0.228627 | 13.80528 | 2.37E-43 | 1.51E-40 | Up |
| TMEM173 | 3510.905827 | 1.730543988 | 0.181234 | 9.548683 | 1.31E-21 | 9.13E-20 | Up |
| C14orf180 | 16.21039069 | -2.664235472 | 0.295959 | -9.00204 | 2.22E-19 | 1.22E-17 | Down |
| FAM167A-AS1 | 53.1458994 | -1.671730147 | 0.308412 | -5.42044 | 5.95E-08 | 6.07E-07 | Down |
| NELL2 | 5626.293845 | 2.635547154 | 0.280362 | 9.400523 | 5.43E-21 | 3.57E-19 | Up |
| B3GALT5-AS1 | 110.8563176 | -1.397237349 | 0.30802 | -4.53619 | 5.73E-06 | 3.89E-05 | Down |
| TRARG1 | 17.03283677 | -2.14975739 | 0.373466 | -5.75623 | 8.60E-09 | 1.02E-07 | Down |
| TCEAL2 | 2518.35306 | -1.725014133 | 0.290794 | -5.93209 | 2.99E-09 | 3.84E-08 | Down |
| CLCNKB | 1562.50262 | -1.141347386 | 0.248788 | -4.58764 | 4.48E-06 | 3.11E-05 | Down |
| JAG2 | 1193.942456 | 1.405581862 | 0.142037 | 9.895875 | 4.34E-23 | 3.56E-21 | Up |
| LCN12 | 5237.160364 | 1.157208021 | 0.173849 | 6.656401 | 2.81E-11 | 5.13E-10 | Up |
| WT1 | 8.351253266 | 2.767835271 | 0.416333 | 6.648131 | 2.97E-11 | 5.41E-10 | Up |
| AQP12A | 0.495254354 | -1.592191486 | 0.663241 | -2.40062 | 0.016367 | 0.042398 | Down |
| IFNE | 23.76821598 | 2.867289 | 0.269622 | 10.63446 | 2.06E-26 | 2.39E-24 | Up |
| MROH2A | 355.914505 | -3.26368806 | 0.518413 | -6.29553 | 3.06E-10 | 4.68E-09 | Down |
| ANKS1B | 621.4226824 | -1.601455765 | 0.204558 | -7.82887 | 4.92E-15 | 1.56E-13 | Down |
| SLC24A3 | 573.1457372 | -1.143118432 | 0.179136 | -6.38129 | 1.76E-10 | 2.80E-09 | Down |
| FLRT2 | 1573.473146 | -1.020154743 | 0.178283 | -5.7221 | 1.05E-08 | 1.22E-07 | Down |
| AC093698.1 | 3.583251895 | -1.626119695 | 0.365555 | -4.44835 | 8.65E-06 | 5.65E-05 | Down |
| MANEAL | 526.6731388 | 1.65835322 | 0.165507 | 10.01985 | 1.25E-23 | 1.11E-21 | Up |
| FAM43A | 1245.61497 | 2.148143321 | 0.213047 | 10.08294 | 6.57E-24 | 6.00E-22 | Up |
| AQP12B | 1.615742553 | -2.952147493 | 0.685653 | -4.3056 | 1.67E-05 | 0.000101 | Down |
| LINC00313 | 1.2341988 | 2.451959492 | 0.527478 | 4.648461 | 3.34E-06 | 2.39E-05 | Up |
| PRSS57 | 5.705876862 | 2.277386263 | 0.325224 | 7.002517 | 2.51E-12 | 5.45E-11 | Up |
| NOTUM | 4.791802813 | 1.699985842 | 0.351206 | 4.840423 | 1.30E-06 | 1.01E-05 | Up |
| KLHL33 | 12.97599458 | -1.225070108 | 0.250984 | -4.88106 | 1.06E-06 | 8.38E-06 | Down |
| NUPR2 | 367.2205778 | -1.150928348 | 0.197195 | -5.83651 | 5.33E-09 | 6.53E-08 | Down |
| SFTPA2 | 61.25155901 | 4.44718511 | 0.461007 | 9.64667 | 5.08E-22 | 3.71E-20 | Up |
| C12orf56 | 22.02401244 | 2.64886358 | 0.306681 | 8.637206 | 5.76E-18 | 2.60E-16 | Up |
| TMEM105 | 62.3130508 | 1.420555922 | 0.293853 | 4.834241 | 1.34E-06 | 1.04E-05 | Up |
| HS6ST3 | 574.0995462 | -1.324953617 | 0.259353 | -5.10868 | 3.24E-07 | 2.87E-06 | Down |
| OR2V1 | 11.20493806 | -4.667130694 | 0.647679 | -7.20593 | 5.77E-13 | 1.38E-11 | Down |
| KRT6B | 1.663587473 | 2.33131665 | 0.825028 | 2.825742 | 0.004717 | 0.014846 | Up |
| ROR1 | 151.6459964 | 1.221942287 | 0.2375 | 5.145025 | 2.67E-07 | 2.40E-06 | Up |
| MUC1 | 3109.726629 | 1.938676483 | 0.286263 | 6.77235 | 1.27E-11 | 2.45E-10 | Up |
| SV2B | 83.43411212 | -1.165081201 | 0.236113 | -4.93443 | 8.04E-07 | 6.54E-06 | Down |
| AHNAK2 | 1748.05289 | 3.375029081 | 0.26728 | 12.6273 | 1.49E-36 | 4.48E-34 | Up |
| SPATA8 | 0.578693637 | 1.701243006 | 0.619283 | 2.747118 | 0.006012 | 0.018276 | Up |
| DBX2 | 10.4698277 | -1.943047538 | 0.302772 | -6.41752 | 1.39E-10 | 2.26E-09 | Down |
| PDIA2 | 4.272656463 | 1.023548774 | 0.332797 | 3.075593 | 0.002101 | 0.007365 | Up |
| NDUFA4L2 | 1313.643507 | 1.324348276 | 0.17298 | 7.656067 | 1.92E-14 | 5.61E-13 | Up |
| SHC4 | 64.05446026 | 1.146486244 | 0.196073 | 5.84724 | 5.00E-09 | 6.14E-08 | Up |
| KRT79 | 1.409302859 | 1.7492002 | 0.462274 | 3.783902 | 0.000154 | 0.000747 | Up |
| ADARB2 | 34.37811677 | 2.031208539 | 0.486294 | 4.176915 | 2.95E-05 | 0.00017 | Up |
| SRL | 1105.186288 | 1.324675641 | 0.216664 | 6.113969 | 9.72E-10 | 1.35E-08 | Up |
| C11orf87 | 1.632359478 | -2.416926471 | 0.468156 | -5.16265 | 2.43E-07 | 2.21E-06 | Down |
| KCNIP4 | 517.4559002 | -1.886124184 | 0.180354 | -10.4579 | 1.35E-25 | 1.43E-23 | Down |
| NLRP9 | 1.791668777 | -1.428782176 | 0.447352 | -3.19387 | 0.001404 | 0.005204 | Down |
| NAT8L | 1234.58009 | 3.064544671 | 0.295619 | 10.36652 | 3.52E-25 | 3.62E-23 | Up |
| LINC01405 | 1.291103681 | -1.880067422 | 0.634006 | -2.96538 | 0.003023 | 0.010107 | Down |
| KLHL34 | 7.056044684 | 1.129806575 | 0.438919 | 2.574069 | 0.010051 | 0.028224 | Up |
| CALHM1 | 3.502700125 | 1.717723961 | 0.424626 | 4.045263 | 5.23E-05 | 0.000284 | Up |
| C7orf61 | 18.72568491 | 1.421319485 | 0.232206 | 6.120929 | 9.30E-10 | 1.30E-08 | Up |
| SLITRK2 | 43.0617075 | -2.24821324 | 0.347651 | -6.46687 | 1.00E-10 | 1.67E-09 | Down |
| PLK5 | 2.198800548 | -1.638574834 | 0.386569 | -4.23877 | 2.25E-05 | 0.000133 | Down |
| LEMD1 | 167.177142 | 2.000288804 | 0.185758 | 10.76828 | 4.86E-27 | 6.10E-25 | Up |
| KRT73 | 1.310115078 | -2.439585007 | 0.771087 | -3.16383 | 0.001557 | 0.005695 | Down |
| AC013268.1 | 4.017474032 | 1.879670693 | 0.358463 | 5.243691 | 1.57E-07 | 1.48E-06 | Up |
| CYP4Z1 | 5.114061291 | 1.19468363 | 0.311972 | 3.829452 | 0.000128 | 0.000633 | Up |
| FFAR4 | 48.85347509 | 1.652342322 | 0.357607 | 4.620556 | 3.83E-06 | 2.70E-05 | Up |
| EDARADD | 444.2941302 | -1.242997075 | 0.192356 | -6.46197 | 1.03E-10 | 1.72E-09 | Down |
| GABRA5 | 15.82185856 | -1.130876255 | 0.354413 | -3.19085 | 0.001419 | 0.005248 | Down |
| RGS9BP | 14.98369532 | -1.053701058 | 0.158827 | -6.63427 | 3.26E-11 | 5.90E-10 | Down |
| TMEM212 | 0.694043946 | -1.95071812 | 0.543981 | -3.586 | 0.000336 | 0.001484 | Down |
| SLC36A2 | 1.505563526 | -1.193250899 | 0.451307 | -2.64399 | 0.008194 | 0.023784 | Down |
| THBS2 | 2461.291922 | 1.727444725 | 0.263245 | 6.562115 | 5.30E-11 | 9.30E-10 | Up |
| ANKRD37 | 637.9514609 | -1.24164852 | 0.186309 | -6.66446 | 2.66E-11 | 4.87E-10 | Down |
| LINC00643 | 5.196187684 | -1.6869012 | 0.563271 | -2.99483 | 0.002746 | 0.009295 | Down |
| GLDN | 295.2477142 | 3.932952855 | 0.317262 | 12.39653 | 2.73E-35 | 6.83E-33 | Up |
| TRDN | 4.729379035 | -1.987050437 | 0.438445 | -4.53204 | 5.84E-06 | 3.96E-05 | Down |
| KRT3 | 6.850085663 | -1.207359949 | 0.320048 | -3.77244 | 0.000162 | 0.000778 | Down |
| SPATA12 | 5.804255181 | 1.238512405 | 0.307891 | 4.022567 | 5.76E-05 | 0.000309 | Up |
| KLK12 | 28.72176141 | 4.000031798 | 0.631159 | 6.337595 | 2.33E-10 | 3.65E-09 | Up |
| MYT1L | 8.170011831 | -1.398264951 | 0.261158 | -5.35409 | 8.60E-08 | 8.49E-07 | Down |
| MIR22HG | 3375.72308 | -1.177911541 | 0.20208 | -5.82893 | 5.58E-09 | 6.81E-08 | Down |
| MPPED1 | 1.230665792 | -1.642623377 | 0.543838 | -3.02043 | 0.002524 | 0.008647 | Down |
| FOXI2 | 41.21806657 | 3.036540197 | 0.336602 | 9.021154 | 1.86E-19 | 1.04E-17 | Up |
| KRT16 | 9.59100836 | 2.214733747 | 0.526936 | 4.203044 | 2.63E-05 | 0.000153 | Up |
| KRT14 | 49.82193488 | 1.991022962 | 0.547294 | 3.637942 | 0.000275 | 0.001244 | Up |
| TRABD2A | 319.4730022 | 1.336508123 | 0.345527 | 3.868028 | 0.00011 | 0.00055 | Up |
| PDZD7 | 63.05682038 | 1.155440581 | 0.149631 | 7.721914 | 1.15E-14 | 3.47E-13 | Up |
| QRFPR | 31.20939692 | 1.787192407 | 0.283145 | 6.311932 | 2.76E-10 | 4.25E-09 | Up |
| TMEM232 | 317.5549252 | -1.069973806 | 0.129308 | -8.27462 | 1.29E-16 | 5.02E-15 | Down |
| LINC01551 | 1.761574903 | -1.618922948 | 0.591103 | -2.73882 | 0.006166 | 0.018664 | Down |
| FAM183A | 9.500901255 | 1.376604956 | 0.505486 | 2.723331 | 0.006463 | 0.019425 | Up |
| ESPN | 150.2630378 | 5.145198082 | 0.380496 | 13.52234 | 1.15E-41 | 5.78E-39 | Up |
| SAMD7 | 1.035590384 | -1.898825629 | 0.6556 | -2.89632 | 0.003776 | 0.012255 | Down |
| GPR141 | 38.60886374 | 1.886025163 | 0.31876 | 5.916748 | 3.28E-09 | 4.19E-08 | Up |
| TMPRSS6 | 902.8443426 | 6.515933867 | 0.427531 | 15.24084 | 1.89E-52 | 3.23E-49 | Up |
| CCK | 3.397761908 | -1.488989445 | 0.411057 | -3.62234 | 0.000292 | 0.001313 | Down |
| SLIT1 | 2421.406616 | 5.944221208 | 0.432093 | 13.7568 | 4.64E-43 | 2.72E-40 | Up |
| LYPD6 | 17.17567912 | -1.53318637 | 0.245777 | -6.23812 | 4.43E-10 | 6.56E-09 | Down |
| AKR1C1 | 615.1421178 | -1.948166472 | 0.209233 | -9.31097 | 1.27E-20 | 8.03E-19 | Down |
| FOXD3 | 3.555443007 | -3.100912663 | 0.42701 | -7.26192 | 3.82E-13 | 9.35E-12 | Down |
| ANGPTL5 | 38.97123031 | -1.054034862 | 0.266288 | -3.95824 | 7.55E-05 | 0.000394 | Down |
| AC092118.1 | 54.87033154 | 1.643742289 | 0.208604 | 7.879723 | 3.28E-15 | 1.07E-13 | Up |
| CIDEC | 20.86489949 | -2.030160461 | 0.439042 | -4.62406 | 3.76E-06 | 2.65E-05 | Down |
| DCC | 15.13898663 | 1.874735421 | 0.433785 | 4.321809 | 1.55E-05 | 9.50E-05 | Up |
| HIST1H1T | 1.291752624 | 1.161313973 | 0.420883 | 2.759231 | 0.005794 | 0.0177 | Up |
| CDHR4 | 2.429320037 | -1.07898771 | 0.382462 | -2.82116 | 0.004785 | 0.015019 | Down |
| COL4A1 | 13909.44403 | 1.313661894 | 0.155958 | 8.423173 | 3.66E-17 | 1.50E-15 | Up |
| C12orf74 | 8.465043048 | -1.63513323 | 0.6305 | -2.59339 | 0.009503 | 0.026961 | Down |
| GJA4 | 1894.298933 | 1.722495411 | 0.151399 | 11.37719 | 5.43E-30 | 9.18E-28 | Up |
| CYP26C1 | 9.933296324 | 1.25650274 | 0.273156 | 4.599939 | 4.23E-06 | 2.94E-05 | Up |
| NANOS3 | 41.29331185 | 1.089477595 | 0.206709 | 5.270591 | 1.36E-07 | 1.29E-06 | Up |
| PLEKHN1 | 197.8611146 | 3.472392796 | 0.264541 | 13.12609 | 2.33E-39 | 9.52E-37 | Up |
| TERF1P2 | 0.977843537 | 1.177442313 | 0.377381 | 3.120037 | 0.001808 | 0.006471 | Up |
| MYMK | 1.529092225 | 1.010367301 | 0.427946 | 2.360967 | 0.018227 | 0.046333 | Up |
| TCL6 | 38.70785634 | -2.231389506 | 0.611831 | -3.64707 | 0.000265 | 0.001205 | Down |
| SAMD11 | 425.514575 | 2.165685834 | 0.237844 | 9.10548 | 8.59E-20 | 4.98E-18 | Up |
| PERM1 | 85.08105989 | 2.87198015 | 0.303293 | 9.469333 | 2.82E-21 | 1.90E-19 | Up |
| SPRY4 | 2333.516819 | 1.358319586 | 0.148895 | 9.122682 | 7.33E-20 | 4.27E-18 | Up |
| AMTN | 2.757665534 | 1.991956064 | 0.574332 | 3.468303 | 0.000524 | 0.002199 | Up |
| SLC18A3 | 42.89268118 | 7.724981564 | 0.922004 | 8.378466 | 5.36E-17 | 2.16E-15 | Up |
| GABRD | 177.8062015 | 2.652263552 | 0.300158 | 8.836233 | 9.90E-19 | 4.93E-17 | Up |
| ADH1A | 3.643174924 | -1.126564987 | 0.339925 | -3.31416 | 0.000919 | 0.003598 | Down |
| HSPE1P11 | 1.891382763 | -1.625633355 | 0.42243 | -3.84829 | 0.000119 | 0.000592 | Down |
| DIPK1C | 13.33738633 | 1.826293151 | 0.320264 | 5.70247 | 1.18E-08 | 1.36E-07 | Up |
| FAM205C | 2.000890969 | -1.40919749 | 0.368119 | -3.82811 | 0.000129 | 0.000636 | Down |
| RTL4 | 252.2592891 | 5.674809196 | 0.429203 | 13.22175 | 6.57E-40 | 2.94E-37 | Up |
| TMEM220 | 1167.462322 | -1.011551612 | 0.115888 | -8.72872 | 2.58E-18 | 1.23E-16 | Down |
| P2RX2 | 7.103501821 | -1.66408602 | 0.626773 | -2.65501 | 0.007931 | 0.023139 | Down |
| PALM3 | 1490.690844 | 1.454812404 | 0.286512 | 5.077674 | 3.82E-07 | 3.32E-06 | Up |
| DMBT1 | 19.90205131 | 2.525397843 | 0.357425 | 7.065531 | 1.60E-12 | 3.57E-11 | Up |
| CLEC17A | 59.64205494 | -1.45004081 | 0.598264 | -2.42375 | 0.015361 | 0.04025 | Down |
| LCN10 | 144.3736987 | 1.491576992 | 0.273677 | 5.450127 | 5.03E-08 | 5.20E-07 | Up |
| PLA2G2C | 4.484038427 | 2.426853603 | 0.323589 | 7.49981 | 6.39E-14 | 1.73E-12 | Up |
| RINL | 1022.594235 | 1.035679382 | 0.111627 | 9.278046 | 1.73E-20 | 1.07E-18 | Up |
| C19orf67 | 7.228432024 | 1.073278182 | 0.246299 | 4.357629 | 1.31E-05 | 8.22E-05 | Up |
| NWD1 | 474.0598033 | -1.29007616 | 0.327757 | -3.93607 | 8.28E-05 | 0.000427 | Down |
| PLA2G4E | 4.264929814 | -1.539181425 | 0.360874 | -4.26515 | 2.00E-05 | 0.000119 | Down |
| TMEM215 | 349.7663938 | 5.49555878 | 0.481871 | 11.40463 | 3.96E-30 | 6.82E-28 | Up |
| OTOG | 2.434561522 | -1.740496319 | 0.513021 | -3.39264 | 0.000692 | 0.002804 | Down |
| PLA2G2A | 354.4359959 | 1.524834852 | 0.476665 | 3.198968 | 0.001379 | 0.005121 | Up |
| OR7A5 | 2.591370018 | 2.8610284 | 0.383845 | 7.453605 | 9.08E-14 | 2.41E-12 | Up |
| FAM230G | 1.23059353 | 3.436069337 | 0.614654 | 5.590251 | 2.27E-08 | 2.48E-07 | Up |
| HES4 | 233.5161289 | 1.193786137 | 0.280952 | 4.249079 | 2.15E-05 | 0.000127 | Up |
| IGFL1 | 3.205188104 | 5.413589734 | 0.800593 | 6.761974 | 1.36E-11 | 2.62E-10 | Up |
| ENO4 | 68.07782843 | -1.083946549 | 0.142362 | -7.61401 | 2.66E-14 | 7.58E-13 | Down |
| JAKMIP3 | 67.30351472 | -1.274589097 | 0.160833 | -7.92492 | 2.28E-15 | 7.57E-14 | Down |
| PPP3R2 | 0.645329162 | -1.330812627 | 0.56013 | -2.3759 | 0.017506 | 0.04481 | Down |
| GPR21 | 0.782536523 | 1.21452578 | 0.436922 | 2.779734 | 0.00544 | 0.016772 | Up |
| TCTEX1D4 | 43.928188 | -1.051584372 | 0.26754 | -3.93056 | 8.47E-05 | 0.000435 | Down |
| IER5L | 577.4213506 | 1.952992876 | 0.2329 | 8.385526 | 5.05E-17 | 2.04E-15 | Up |
| SERPINA5 | 169.5417111 | -2.281562434 | 0.368383 | -6.19345 | 5.89E-10 | 8.50E-09 | Down |
| CFAP77 | 16.39816626 | -1.57655792 | 0.265051 | -5.94813 | 2.71E-09 | 3.51E-08 | Down |
| HBA2 | 1959.628245 | -1.412327962 | 0.300621 | -4.69803 | 2.63E-06 | 1.92E-05 | Down |
| FBLL1 | 29.59552674 | 1.49867528 | 0.350527 | 4.275487 | 1.91E-05 | 0.000115 | Up |
| CFAP54 | 186.3417221 | -1.310786038 | 0.163971 | -7.99401 | 1.31E-15 | 4.45E-14 | Down |
| IGFL3 | 0.977560327 | 2.826049517 | 0.706406 | 4.000602 | 6.32E-05 | 0.000336 | Up |
| GOLGA8M | 222.3573072 | -1.03448655 | 0.254509 | -4.06463 | 4.81E-05 | 0.000264 | Down |
| CC2D2B | 23.41866241 | 2.298889744 | 0.197473 | 11.64155 | 2.53E-31 | 4.90E-29 | Up |
| IDO2 | 53.85508749 | -1.600143151 | 0.25723 | -6.22068 | 4.95E-10 | 7.27E-09 | Down |
| OSTN | 8.285098921 | -3.038584383 | 0.464733 | -6.53835 | 6.22E-11 | 1.08E-09 | Down |
| VWC2 | 15.69863789 | -2.369099066 | 0.366867 | -6.45766 | 1.06E-10 | 1.76E-09 | Down |
| FZD9 | 19.73960014 | -1.057288294 | 0.217301 | -4.86554 | 1.14E-06 | 9.00E-06 | Down |
| TMSB4XP2 | 4.198163646 | 1.360696348 | 0.377271 | 3.60668 | 0.00031 | 0.001385 | Up |
| SPRED3 | 128.138821 | 1.061836157 | 0.1237 | 8.583936 | 9.17E-18 | 4.01E-16 | Up |
| ADRB3 | 4.370161649 | 3.202220522 | 0.522026 | 6.13422 | 8.56E-10 | 1.20E-08 | Up |
| PLA2G2E | 6.871299068 | 5.829630025 | 0.618773 | 9.421269 | 4.46E-21 | 2.97E-19 | Up |
| SHISA6 | 216.4552624 | 3.222967124 | 0.2764 | 11.6605 | 2.03E-31 | 3.96E-29 | Up |
| CNR2 | 124.8886561 | -1.622506123 | 0.336753 | -4.81809 | 1.45E-06 | 1.12E-05 | Down |
| ENTPD8 | 85.22562811 | 2.13748567 | 0.247955 | 8.620443 | 6.67E-18 | 2.97E-16 | Up |
| BEND4 | 22.77253221 | -1.75973934 | 0.57361 | -3.06783 | 0.002156 | 0.00754 | Down |
| LRRK2 | 8268.328929 | 4.173064086 | 0.270471 | 15.42887 | 1.05E-53 | 1.87E-50 | Up |
| GJB3 | 322.0246195 | 4.329631954 | 0.349441 | 12.39018 | 2.95E-35 | 7.34E-33 | Up |
| UTS2B | 14.46114519 | -1.081123007 | 0.198526 | -5.44575 | 5.16E-08 | 5.32E-07 | Down |
| PROS2P | 1.176479812 | 2.770175625 | 0.39188 | 7.068946 | 1.56E-12 | 3.49E-11 | Up |
| ANKDD1B | 80.6695354 | 1.324410729 | 0.193518 | 6.843853 | 7.71E-12 | 1.54E-10 | Up |
| RELN | 708.2314995 | -2.401245401 | 0.252992 | -9.49141 | 2.28E-21 | 1.55E-19 | Down |
| FAM111B | 184.279476 | 1.925610489 | 0.360925 | 5.335208 | 9.54E-08 | 9.35E-07 | Up |
| APOD | 4237.001432 | -1.705777108 | 0.250659 | -6.80518 | 1.01E-11 | 1.98E-10 | Down |
| VSTM1 | 2.223942287 | 1.073433211 | 0.397704 | 2.699076 | 0.006953 | 0.020688 | Up |
| IL1RAPL2 | 5.035281574 | 1.094451098 | 0.421449 | 2.596876 | 0.009408 | 0.026729 | Up |
| PAX8-AS1 | 937.8499867 | -1.011558933 | 0.17428 | -5.80423 | 6.47E-09 | 7.79E-08 | Down |
| NUGGC | 532.5074264 | -1.399394378 | 0.280354 | -4.99153 | 5.99E-07 | 5.00E-06 | Down |
| DRICH1 | 27.67650088 | 1.248993296 | 0.209572 | 5.959741 | 2.53E-09 | 3.29E-08 | Up |
| FAM180A | 104.5306948 | -1.232410575 | 0.267543 | -4.60641 | 4.10E-06 | 2.86E-05 | Down |
| C6orf222 | 1.389023488 | 2.389002144 | 0.527587 | 4.528167 | 5.95E-06 | 4.02E-05 | Up |
| KIAA0408 | 9.28671501 | 2.639738683 | 0.3517 | 7.505661 | 6.11E-14 | 1.66E-12 | Up |
| CXCL17 | 323.8168662 | 3.401557564 | 0.41735 | 8.150369 | 3.63E-16 | 1.32E-14 | Up |
| GJB4 | 35.41913 | 3.619136438 | 0.390391 | 9.27054 | 1.85E-20 | 1.15E-18 | Up |
| IL1RAP | 891.8074704 | 2.252477306 | 0.182775 | 12.32379 | 6.74E-35 | 1.63E-32 | Up |
| PAX5 | 582.5467479 | -2.123206933 | 0.700867 | -3.0294 | 0.00245 | 0.008428 | Down |
| SPOCK3 | 17.70936407 | 2.687066528 | 0.505407 | 5.316636 | 1.06E-07 | 1.02E-06 | Up |
| VN1R2 | 1.250959568 | -1.538668252 | 0.444147 | -3.46433 | 0.000532 | 0.002229 | Down |
| SERPINA3 | 4.938186333 | -1.368094352 | 0.448948 | -3.04733 | 0.002309 | 0.00799 | Down |
| S100A4 | 5687.791792 | 1.386715585 | 0.246692 | 5.62125 | 1.90E-08 | 2.10E-07 | Up |
| PLEKHG4 | 754.0733607 | 1.140026644 | 0.169792 | 6.714243 | 1.89E-11 | 3.55E-10 | Up |
| KIF19 | 374.2141131 | -1.469719791 | 0.23758 | -6.18621 | 6.16E-10 | 8.86E-09 | Down |
| GREB1 | 123.4766659 | 1.54908326 | 0.268432 | 5.770853 | 7.89E-09 | 9.36E-08 | Up |
| RYR1 | 884.3573002 | 2.724937864 | 0.22805 | 11.94888 | 6.58E-33 | 1.41E-30 | Up |
| SFTA2 | 62.69584095 | 1.602608775 | 0.375034 | 4.273233 | 1.93E-05 | 0.000116 | Up |
| HLA-DRB9 | 11.29377798 | 1.112507667 | 0.340556 | 3.266739 | 0.001088 | 0.004162 | Up |
| CD55 | 9715.221969 | 2.395193787 | 0.263989 | 9.07308 | 1.16E-19 | 6.60E-18 | Up |
| CPNE4 | 196.7756528 | 4.123987972 | 0.425908 | 9.682821 | 3.57E-22 | 2.65E-20 | Up |
| NUDT11 | 156.7768896 | 1.975877878 | 0.258489 | 7.643939 | 2.11E-14 | 6.11E-13 | Up |
| SLC35F1 | 117.0801914 | 1.407595061 | 0.27193 | 5.176311 | 2.26E-07 | 2.07E-06 | Up |
| THEM5 | 95.9710687 | 1.47797314 | 0.246732 | 5.990196 | 2.10E-09 | 2.77E-08 | Up |
| PRTN3 | 10.47113298 | 2.210009455 | 0.299372 | 7.38214 | 1.56E-13 | 4.01E-12 | Up |
| S100A5 | 255.4586701 | 1.488696228 | 0.176364 | 8.441033 | 3.15E-17 | 1.30E-15 | Up |
| RFX8 | 30.7233123 | 1.798891789 | 0.194633 | 9.242464 | 2.41E-20 | 1.48E-18 | Up |
| ESRRG | 816.607643 | -1.10821437 | 0.209903 | -5.27965 | 1.29E-07 | 1.23E-06 | Down |
| AL138767.1 | 0.845770464 | -1.518617459 | 0.542549 | -2.79904 | 0.005125 | 0.015949 | Down |
| ANKRD20A19P | 10.78260645 | 1.738563494 | 0.405095 | 4.291739 | 1.77E-05 | 0.000107 | Up |
| MMP1 | 34.51048536 | 3.238164632 | 0.460159 | 7.037057 | 1.96E-12 | 4.31E-11 | Up |
| ADH1B | 1381.527108 | -2.175108981 | 0.308338 | -7.05431 | 1.73E-12 | 3.85E-11 | Down |
| HRH1 | 346.33383 | 2.342895028 | 0.218985 | 10.69887 | 1.03E-26 | 1.22E-24 | Up |
| FAM180B | 196.7783913 | -2.410910058 | 0.26423 | -9.12429 | 7.22E-20 | 4.22E-18 | Down |
| COL27A1 | 1806.780375 | -1.138283925 | 0.1896 | -6.00361 | 1.93E-09 | 2.57E-08 | Down |
| CLPSL2 | 5.664032348 | 2.049803774 | 0.348006 | 5.890142 | 3.86E-09 | 4.85E-08 | Up |
| POU3F4 | 2.609580747 | -1.503897243 | 0.560721 | -2.68208 | 0.007317 | 0.0216 | Down |
| OR14A16 | 1.198970628 | -1.392812165 | 0.468226 | -2.97466 | 0.002933 | 0.009846 | Down |
| TLE1 | 2307.77924 | -1.133950214 | 0.124674 | -9.09534 | 9.43E-20 | 5.45E-18 | Down |
| SPINK14 | 0.898933701 | 2.282060715 | 0.649369 | 3.514273 | 0.000441 | 0.001889 | Up |
| PATE2 | 0.937254853 | -1.941553615 | 0.483574 | -4.01501 | 5.94E-05 | 0.000318 | Down |
| HIST1H2AD | 9.281265278 | 1.139532394 | 0.2636 | 4.322955 | 1.54E-05 | 9.45E-05 | Up |
| LAMB3 | 4122.591722 | 4.359647064 | 0.321815 | 13.54705 | 8.25E-42 | 4.30E-39 | Up |
| HIST3H2BB | 10.69021126 | 2.468782826 | 0.329257 | 7.498046 | 6.48E-14 | 1.75E-12 | Up |
| PDLIM7 | 1859.763419 | 1.263596863 | 0.129146 | 9.784269 | 1.32E-22 | 1.03E-20 | Up |
| FAM163B | 4.872280472 | 1.346359988 | 0.559022 | 2.408421 | 0.016022 | 0.041649 | Up |
| SIGLEC15 | 170.1951555 | 4.152039633 | 0.353429 | 11.74787 | 7.24E-32 | 1.45E-29 | Up |
| OR2T32P | 5.014248481 | -1.050632217 | 0.280915 | -3.74004 | 0.000184 | 0.00087 | Down |
| NPSR1-AS1 | 1.442863551 | 3.629291627 | 0.673837 | 5.386004 | 7.20E-08 | 7.22E-07 | Up |
| GAL3ST4 | 292.8453301 | 1.184442481 | 0.290344 | 4.079444 | 4.51E-05 | 0.000249 | Up |
| ACSL5 | 1490.250114 | 1.168840962 | 0.208343 | 5.610186 | 2.02E-08 | 2.23E-07 | Up |
| ADGRA1 | 3.944865257 | -1.424123161 | 0.411365 | -3.46194 | 0.000536 | 0.002245 | Down |
| CYSRT1 | 144.7942006 | 1.134084112 | 0.173431 | 6.539126 | 6.19E-11 | 1.07E-09 | Up |
| SLC22A4 | 88.95883862 | 1.026039042 | 0.14101 | 7.276366 | 3.43E-13 | 8.45E-12 | Up |
| SERPINA1 | 37276.31431 | 4.382286975 | 0.301784 | 14.52126 | 8.89E-48 | 9.53E-45 | Up |
| TPSB2 | 65.06935287 | 2.318118457 | 0.380934 | 6.085351 | 1.16E-09 | 1.60E-08 | Up |
| C6orf141 | 53.30165242 | -1.31107977 | 0.260035 | -5.04193 | 4.61E-07 | 3.94E-06 | Down |
| HMGA2-AS1 | 265.4341107 | 2.931966028 | 0.278976 | 10.50974 | 7.79E-26 | 8.50E-24 | Up |
| PELI1 | 1351.4574 | 1.337304952 | 0.235503 | 5.678507 | 1.36E-08 | 1.55E-07 | Up |
| DIO3 | 46.26299066 | -1.336294159 | 0.281941 | -4.73962 | 2.14E-06 | 1.59E-05 | Down |
| DCHS2 | 75.3271735 | 2.638495775 | 0.27236 | 9.687548 | 3.41E-22 | 2.54E-20 | Up |
| GGT3P | 79.90591177 | 1.784076848 | 0.242456 | 7.35836 | 1.86E-13 | 4.74E-12 | Up |
| PDGFA | 562.4292971 | 1.254419997 | 0.142833 | 8.782452 | 1.60E-18 | 7.78E-17 | Up |
| COL13A1 | 477.6204451 | 3.496725336 | 0.212507 | 16.45465 | 7.77E-61 | 4.86E-57 | Up |
| SLC28A3 | 75.23399659 | 1.938016125 | 0.311962 | 6.212346 | 5.22E-10 | 7.61E-09 | Up |
| COL4A6 | 164.4457401 | -1.593206299 | 0.236658 | -6.7321 | 1.67E-11 | 3.16E-10 | Down |
| KCNMB2 | 19.21899013 | -1.852139776 | 0.289895 | -6.38901 | 1.67E-10 | 2.67E-09 | Down |
| DMBX1 | 22.01263846 | 5.490664293 | 0.563056 | 9.751538 | 1.82E-22 | 1.39E-20 | Up |
| KLKP1 | 7.26715672 | -1.47166904 | 0.352411 | -4.176 | 2.97E-05 | 0.00017 | Down |
| SERPINB2 | 10.1212802 | 1.909483065 | 0.439968 | 4.340051 | 1.42E-05 | 8.84E-05 | Up |
| DPP4 | 3239.699771 | 4.241686042 | 0.308153 | 13.76485 | 4.15E-43 | 2.47E-40 | Up |
| NMB | 2313.052037 | 1.22057011 | 0.247565 | 4.930299 | 8.21E-07 | 6.67E-06 | Up |
| CFAP43 | 503.8373892 | -1.061279493 | 0.153673 | -6.90611 | 4.98E-12 | 1.03E-10 | Down |
| CFD | 2565.10987 | -2.116676754 | 0.239105 | -8.85249 | 8.56E-19 | 4.32E-17 | Down |
| MAP1LC3C | 93.22133423 | -1.340066684 | 0.242523 | -5.52551 | 3.29E-08 | 3.49E-07 | Down |
| AC011450.1 | 25.33419429 | -1.246852975 | 0.159844 | -7.80046 | 6.17E-15 | 1.94E-13 | Down |
| AC122129.1 | 104.3631155 | -1.106282395 | 0.233941 | -4.7289 | 2.26E-06 | 1.67E-05 | Down |
| CFAP299 | 23.49548459 | -1.828642364 | 0.230984 | -7.91675 | 2.44E-15 | 8.04E-14 | Down |
| ADAMTSL2 | 1087.045075 | 1.003230152 | 0.130681 | 7.676916 | 1.63E-14 | 4.81E-13 | Up |
| PRB3 | 2.841317122 | 1.076079464 | 0.300819 | 3.577163 | 0.000347 | 0.001529 | Up |
| IFNA1 | 1.185358438 | 2.829619522 | 0.584026 | 4.845023 | 1.27E-06 | 9.86E-06 | Up |
| HES5 | 6.972640916 | 1.154730083 | 0.374713 | 3.08164 | 0.002059 | 0.007238 | Up |
| AADACL2 | 1.100305964 | -1.678966455 | 0.471943 | -3.55756 | 0.000374 | 0.001634 | Down |
| S100A6 | 58413.33632 | 1.459063778 | 0.18321 | 7.963884 | 1.67E-15 | 5.61E-14 | Up |
| KEL | 61.68093946 | -1.204330468 | 0.318851 | -3.7771 | 0.000159 | 0.000765 | Down |
| ZNF560 | 15.18309937 | 3.526524204 | 0.383063 | 9.206133 | 3.38E-20 | 2.04E-18 | Up |
| TMPRSS11F | 1.350719097 | 1.62224868 | 0.466902 | 3.474493 | 0.000512 | 0.002154 | Up |
| ADH4 | 2.575823887 | -1.538962386 | 0.417181 | -3.68895 | 0.000225 | 0.001039 | Down |
| AC025279.1 | 25.68897906 | -1.582949444 | 0.359257 | -4.40617 | 1.05E-05 | 6.73E-05 | Down |
| TOR4A | 1217.057272 | 1.005913593 | 0.117087 | 8.591147 | 8.61E-18 | 3.80E-16 | Up |
| LPAR1 | 1518.561621 | -1.094444261 | 0.144675 | -7.56486 | 3.88E-14 | 1.08E-12 | Down |
| OR2L3 | 1.327675538 | -1.490764319 | 0.528177 | -2.82247 | 0.004765 | 0.014964 | Down |
| FAM47C | 2.116793378 | -1.718121785 | 0.377632 | -4.54972 | 5.37E-06 | 3.66E-05 | Down |
| BPIFA1 | 0.928630836 | 1.759910636 | 0.477174 | 3.688194 | 0.000226 | 0.001042 | Up |
| AC131392.1 | 8.556501937 | -1.385102575 | 0.277205 | -4.99668 | 5.83E-07 | 4.88E-06 | Down |
| OR5BB1P | 0.935987424 | 3.925452111 | 0.744258 | 5.274315 | 1.33E-07 | 1.27E-06 | Up |
| AL354714.1 | 2.200811905 | -1.233643092 | 0.429647 | -2.87129 | 0.004088 | 0.01313 | Down |
| NTRK1 | 12.45041134 | 1.093381092 | 0.294096 | 3.717765 | 0.000201 | 0.000941 | Up |
| TATDN2P1 | 0.645334743 | -2.010317799 | 0.719454 | -2.79423 | 0.005202 | 0.016152 | Down |
| B3GNT6 | 1.602635088 | 1.445335452 | 0.479067 | 3.016979 | 0.002553 | 0.008727 | Up |
| C2CD4A | 153.1062957 | 4.272934485 | 0.384038 | 11.12633 | 9.34E-29 | 1.39E-26 | Up |
| C20orf203 | 30.17756169 | 1.181912616 | 0.226488 | 5.218426 | 1.80E-07 | 1.67E-06 | Up |
| ARC | 2366.20211 | -2.5218866 | 0.483065 | -5.22059 | 1.78E-07 | 1.66E-06 | Down |
| ZNF536 | 236.1966079 | -1.057882411 | 0.322074 | -3.2846 | 0.001021 | 0.003943 | Down |
| RYR2 | 2372.423897 | -2.206682715 | 0.254115 | -8.68379 | 3.83E-18 | 1.78E-16 | Down |
| LPA | 6.938226951 | -1.361874326 | 0.313118 | -4.3494 | 1.37E-05 | 8.50E-05 | Down |
| FAM19A2 | 292.9896152 | 2.525824424 | 0.264025 | 9.566616 | 1.10E-21 | 7.79E-20 | Up |
| PAPSS2 | 2897.098887 | -1.782911652 | 0.175591 | -10.1538 | 3.19E-24 | 2.98E-22 | Down |
| LRRTM3 | 0.839775685 | -1.812338279 | 0.512218 | -3.53822 | 0.000403 | 0.001743 | Down |
| EGFL6 | 37.2254687 | 2.37976235 | 0.378423 | 6.288622 | 3.20E-10 | 4.88E-09 | Up |
| APCDD1L | 13.82945538 | 2.713990398 | 0.413678 | 6.560633 | 5.36E-11 | 9.38E-10 | Up |
| RASSF9 | 680.6253402 | -1.858969069 | 0.18197 | -10.2158 | 1.68E-24 | 1.60E-22 | Down |
| MUC2 | 0.783211998 | 1.252545581 | 0.482349 | 2.596762 | 0.009411 | 0.026734 | Up |
| BRINP2 | 42.8335484 | 3.575610004 | 0.385579 | 9.273347 | 1.80E-20 | 1.12E-18 | Up |
| PNP | 4322.356931 | 2.717474306 | 0.290473 | 9.355351 | 8.33E-21 | 5.36E-19 | Up |
| PAX9 | 235.2497311 | 2.019357158 | 0.322899 | 6.253836 | 4.00E-10 | 5.99E-09 | Up |
| POU3F3 | 2.010324644 | -1.756865321 | 0.573553 | -3.06312 | 0.00219 | 0.007643 | Down |
| DMD | 2334.517825 | 1.69895871 | 0.200288 | 8.482583 | 2.20E-17 | 9.27E-16 | Up |
| MFAP3L | 370.0032767 | -1.10579917 | 0.214408 | -5.15746 | 2.50E-07 | 2.26E-06 | Down |
| TGM2 | 5272.411063 | 1.780052201 | 0.233273 | 7.630781 | 2.33E-14 | 6.72E-13 | Up |
| ARMCX6 | 1182.456435 | 1.10105536 | 0.100834 | 10.91947 | 9.30E-28 | 1.26E-25 | Up |
| RORB | 163.3866984 | -1.320858187 | 0.234934 | -5.62225 | 1.88E-08 | 2.09E-07 | Down |
| MIR219A1 | 5.394918449 | -1.416788758 | 0.261653 | -5.41477 | 6.14E-08 | 6.25E-07 | Down |
| MIR210 | 0.867426007 | -1.439793931 | 0.530906 | -2.71196 | 0.006689 | 0.020007 | Down |
| MIR342 | 1.89345563 | -1.957707717 | 0.59616 | -3.28386 | 0.001024 | 0.00395 | Down |
| RNU5E-1 | 12.91624269 | -1.76617435 | 0.349242 | -5.05716 | 4.26E-07 | 3.66E-06 | Down |
| RNU5F-1 | 22.54581004 | -3.734758648 | 0.400706 | -9.32044 | 1.16E-20 | 7.38E-19 | Down |
| RNA5SP301 | 0.708926317 | -1.701123376 | 0.500514 | -3.39875 | 0.000677 | 0.002751 | Down |
| RNU4-35P | 0.808680193 | -1.834088144 | 0.507492 | -3.61403 | 0.000301 | 0.001352 | Down |
| RF00019.36 | 9.656766156 | -1.031720257 | 0.19379 | -5.32392 | 1.02E-07 | 9.89E-07 | Down |
| RNA5SP477 | 1.368427748 | -1.11714861 | 0.423424 | -2.63837 | 0.008331 | 0.024113 | Down |
| RF00019.44 | 10.40086201 | -1.433705557 | 0.229249 | -6.25394 | 4.00E-10 | 5.99E-09 | Down |
| RNU5A-1 | 8.754239072 | -2.808110487 | 0.38439 | -7.30536 | 2.77E-13 | 6.90E-12 | Down |
| RNU6-1301P | 0.642291554 | -1.473463571 | 0.5737 | -2.56835 | 0.010218 | 0.028615 | Down |
| RNU6-1266P | 1.802443231 | -1.286741546 | 0.443006 | -2.90457 | 0.003678 | 0.011972 | Down |
| RN7SKP173 | 1.871008244 | -1.032530786 | 0.338929 | -3.04646 | 0.002316 | 0.00801 | Down |
| RNU1-120P | 2.166505293 | -2.384177144 | 0.503032 | -4.73961 | 2.14E-06 | 1.59E-05 | Down |
| RNY1P16 | 4.73200766 | 1.121826484 | 0.245915 | 4.561851 | 5.07E-06 | 3.47E-05 | Up |
| RF00410 | 3.860663842 | -1.055823176 | 0.294845 | -3.58095 | 0.000342 | 0.001511 | Down |
| RF00045 | 0.959348408 | -1.4731762 | 0.450833 | -3.26768 | 0.001084 | 0.00415 | Down |
| VTRNA1-1 | 1.657844378 | -1.869161766 | 0.533493 | -3.50363 | 0.000459 | 0.001958 | Down |
| RNU6-403P | 0.834528599 | 3.172545553 | 0.747338 | 4.24513 | 2.18E-05 | 0.000129 | Up |
| RNU6-181P | 2.870812488 | -1.280269069 | 0.284235 | -4.50426 | 6.66E-06 | 4.45E-05 | Down |
| RF00432 | 0.699680285 | -1.909984413 | 0.580555 | -3.28993 | 0.001002 | 0.00388 | Down |
| RNU5B-1 | 4.037726701 | -2.939841239 | 0.47229 | -6.22466 | 4.83E-10 | 7.11E-09 | Down |
| RNU5D-1 | 6.297550308 | -2.947948783 | 0.39608 | -7.4428 | 9.86E-14 | 2.60E-12 | Down |
| RF00019.121 | 1.420503814 | 1.248662536 | 0.333963 | 3.73892 | 0.000185 | 0.000873 | Up |
| RNA5SP507 | 0.932158068 | 1.243689768 | 0.452028 | 2.751355 | 0.005935 | 0.018063 | Up |
| RF00019.154 | 2.898411709 | -1.094824975 | 0.321174 | -3.40882 | 0.000652 | 0.002667 | Down |
| RF00019.155 | 3.73754037 | -3.773359608 | 0.862198 | -4.37644 | 1.21E-05 | 7.61E-05 | Down |
| RNU6-674P | 0.640740771 | -1.489835934 | 0.515297 | -2.89122 | 0.003837 | 0.012427 | Down |
| RN7SKP180 | 0.84852747 | -1.467339703 | 0.486684 | -3.01497 | 0.00257 | 0.008777 | Down |
| RF00019.186 | 1.661085115 | -1.000050282 | 0.351896 | -2.84189 | 0.004485 | 0.014224 | Down |
| RNU4-1 | 14.60931705 | -2.174115457 | 0.32515 | -6.68651 | 2.29E-11 | 4.24E-10 | Down |
| SNORA38 | 0.524321839 | -1.284709705 | 0.55028 | -2.33465 | 0.019562 | 0.049186 | Down |
| RF00019.191 | 1.692581961 | -1.335831371 | 0.37832 | -3.53096 | 0.000414 | 0.001788 | Down |
| RN7SKP245 | 1.017122252 | -1.564048202 | 0.502853 | -3.11035 | 0.001869 | 0.00666 | Down |
| RN7SKP87 | 0.66018135 | -1.521984944 | 0.564723 | -2.6951 | 0.007037 | 0.020895 | Down |
| RNY1 | 1.965481 | -1.657436254 | 0.495092 | -3.34774 | 0.000815 | 0.003237 | Down |
| RF00019.221 | 1.875476245 | 1.429806442 | 0.421432 | 3.392733 | 0.000692 | 0.002804 | Up |
| RNVU1-3 | 5.13959918 | -1.424835433 | 0.333067 | -4.27792 | 1.89E-05 | 0.000113 | Down |
| RF00019.251 | 1.055020633 | -2.377610153 | 0.941019 | -2.52663 | 0.011516 | 0.031612 | Down |
| RNA5SP515 | 11.8808131 | 2.254631888 | 0.281793 | 8.001034 | 1.23E-15 | 4.22E-14 | Up |
| RNA5SP78 | 1.302059193 | -1.705166281 | 0.427527 | -3.98844 | 6.65E-05 | 0.000352 | Down |
| RNU6-312P | 6.01773057 | 1.026295108 | 0.247098 | 4.153387 | 3.28E-05 | 0.000186 | Up |
| RNVU1-6 | 18.27636205 | -4.358168383 | 0.438255 | -9.94436 | 2.67E-23 | 2.25E-21 | Down |
| RF00019.271 | 1.288651783 | -2.759816069 | 0.479149 | -5.75982 | 8.42E-09 | 9.96E-08 | Down |
| RN7SKP124 | 0.789842384 | -1.289187442 | 0.477811 | -2.69811 | 0.006973 | 0.020735 | Down |
| RNU6-304P | 1.108664125 | -1.198368856 | 0.469895 | -2.55029 | 0.010763 | 0.029889 | Down |
| RNU5E-4P | 3.296069841 | -1.03427479 | 0.383034 | -2.70022 | 0.006929 | 0.020625 | Down |
| RF00019.308 | 0.888631848 | -1.34826427 | 0.494691 | -2.72547 | 0.006421 | 0.019319 | Down |
| SNORA23 | 1.467284109 | -1.316952377 | 0.461861 | -2.8514 | 0.004353 | 0.013863 | Down |
| RF00100 | 692.4186762 | -1.625098792 | 0.373609 | -4.34973 | 1.36E-05 | 8.49E-05 | Down |
| RN7SKP69 | 0.57962759 | -1.646878487 | 0.560877 | -2.93626 | 0.003322 | 0.010975 | Down |
| RNU6-117P | 1.543073975 | -2.66190578 | 0.565923 | -4.70365 | 2.56E-06 | 1.87E-05 | Down |
| RNA5SP277 | 0.940035151 | -2.127706107 | 0.538503 | -3.95115 | 7.78E-05 | 0.000404 | Down |
| RF00019.356 | 2.216455236 | 1.580180006 | 0.428323 | 3.689229 | 0.000225 | 0.001039 | Up |
| VTRNA1-3 | 5.554460957 | -2.784022505 | 0.489358 | -5.68914 | 1.28E-08 | 1.47E-07 | Down |
| RNU4-2 | 32.38127757 | -1.495939241 | 0.374904 | -3.99019 | 6.60E-05 | 0.000349 | Down |
| MIR421 | 3.745092709 | -1.055509056 | 0.303117 | -3.48218 | 0.000497 | 0.002101 | Down |
| MIR146B | 8.656856408 | 5.252601824 | 0.596463 | 8.806257 | 1.29E-18 | 6.38E-17 | Up |
| AL022323.1 | 104.9325527 | -1.079462361 | 0.187772 | -5.7488 | 8.99E-09 | 1.06E-07 | Down |
| TDRKH-AS1 | 46.2864866 | 1.430578327 | 0.128649 | 11.12 | 1.00E-28 | 1.48E-26 | Up |
| AC006019.1 | 0.843132012 | -1.994848361 | 0.497291 | -4.01143 | 6.04E-05 | 0.000322 | Down |
| E2F3P2 | 8.462675506 | -1.462826016 | 0.234916 | -6.22703 | 4.75E-10 | 7.00E-09 | Down |
| LINC00449 | 6.244711276 | -1.259526001 | 0.253019 | -4.978 | 6.42E-07 | 5.34E-06 | Down |
| INF2 | 4631.76715 | 1.489697368 | 0.147563 | 10.09532 | 5.79E-24 | 5.33E-22 | Up |
| LINC02408 | 28.29162835 | 4.074574814 | 0.32044 | 12.71557 | 4.85E-37 | 1.53E-34 | Up |
| AC005342.1 | 3.606528233 | 1.020777093 | 0.231162 | 4.41586 | 1.01E-05 | 6.46E-05 | Up |
| OR2L2 | 0.728104964 | -1.557882067 | 0.544872 | -2.85917 | 0.004248 | 0.013577 | Down |
| LINC02487 | 2.847378826 | 1.126385879 | 0.277346 | 4.061297 | 4.88E-05 | 0.000267 | Up |
| CAPN8 | 35.9780652 | 2.100496831 | 0.256469 | 8.190053 | 2.61E-16 | 9.72E-15 | Up |
| RAET1G | 57.21124991 | 1.509315021 | 0.183016 | 8.24689 | 1.63E-16 | 6.24E-15 | Up |
| SAMD5 | 520.6643559 | -1.314073815 | 0.255543 | -5.14227 | 2.71E-07 | 2.43E-06 | Down |
| LINC00272 | 0.906944689 | -1.483120808 | 0.476183 | -3.1146 | 0.001842 | 0.006574 | Down |
| TEDDM1 | 8.998801852 | -1.73083506 | 0.386547 | -4.47769 | 7.55E-06 | 4.99E-05 | Down |
| METTL11B | 1.393153093 | 3.135251173 | 0.774482 | 4.048192 | 5.16E-05 | 0.000281 | Up |
| FCGR3A | 1766.959895 | 1.354372497 | 0.358429 | 3.778631 | 0.000158 | 0.00076 | Up |
| ADAMTSL4-AS1 | 37.44368506 | -1.47493121 | 0.211929 | -6.95956 | 3.41E-12 | 7.27E-11 | Down |
| PLPP4 | 43.548862 | 2.988100898 | 0.32533 | 9.184836 | 4.12E-20 | 2.46E-18 | Up |
| BVES-AS1 | 8.217305954 | -1.20574078 | 0.23151 | -5.20817 | 1.91E-07 | 1.76E-06 | Down |
| PNLIPRP3 | 0.518760875 | -1.741295452 | 0.671986 | -2.59127 | 0.009562 | 0.027095 | Down |
| ADD3-AS1 | 60.11066703 | -1.149987956 | 0.142112 | -8.09212 | 5.86E-16 | 2.08E-14 | Down |
| CHIAP2 | 2.877269304 | 4.571050063 | 0.552473 | 8.273804 | 1.30E-16 | 5.05E-15 | Up |
| AL121827.1 | 8.044611342 | -2.271425781 | 0.364082 | -6.23878 | 4.41E-10 | 6.54E-09 | Down |
| DPPA5 | 1.612988095 | -1.665456159 | 0.500062 | -3.3305 | 0.000867 | 0.003415 | Down |
| CCDC160 | 17.28741778 | -1.415445156 | 0.185815 | -7.6175 | 2.59E-14 | 7.39E-13 | Down |
| LDLRAD1 | 4.40167593 | -2.114093145 | 0.574626 | -3.67908 | 0.000234 | 0.001076 | Down |
| AL355987.1 | 0.962571185 | 3.053275387 | 0.853537 | 3.577203 | 0.000347 | 0.001529 | Up |
| LIPJ | 14.81665225 | -1.130578627 | 0.191431 | -5.90595 | 3.51E-09 | 4.44E-08 | Down |
| TRPC5OS | 1.645159941 | 1.673488172 | 0.329746 | 5.075087 | 3.87E-07 | 3.36E-06 | Up |
| LRIT2 | 2.939737065 | -1.169306968 | 0.489273 | -2.38989 | 0.016854 | 0.04343 | Down |
| AL158151.1 | 19.47631681 | 1.363905204 | 0.192747 | 7.076153 | 1.48E-12 | 3.33E-11 | Up |
| TCEAL5 | 183.717525 | -2.560085499 | 0.259515 | -9.86487 | 5.91E-23 | 4.76E-21 | Down |
| TCEAL6 | 19.70651782 | -2.273307494 | 0.293962 | -7.73334 | 1.05E-14 | 3.19E-13 | Down |
| ARMCX7P | 9.946295682 | 2.920573482 | 0.554319 | 5.268764 | 1.37E-07 | 1.30E-06 | Up |
| MAFB | 8877.068661 | -1.247899594 | 0.174085 | -7.16835 | 7.59E-13 | 1.78E-11 | Down |
| AL162293.1 | 1.733099426 | -1.734305764 | 0.604151 | -2.87065 | 0.004096 | 0.013153 | Down |
| NHSL2 | 228.9403874 | 1.094242578 | 0.185869 | 5.887174 | 3.93E-09 | 4.93E-08 | Up |
| CLPSL1 | 0.729267514 | 1.383042421 | 0.572711 | 2.414905 | 0.015739 | 0.041034 | Up |
| PTPN20 | 90.21264501 | -1.461834357 | 0.337715 | -4.32861 | 1.50E-05 | 9.24E-05 | Down |
| COL11A2 | 551.5069366 | 1.090839552 | 0.298762 | 3.651199 | 0.000261 | 0.001187 | Up |
| COL5A2 | 2917.023204 | 1.795687275 | 0.226785 | 7.918018 | 2.41E-15 | 7.97E-14 | Up |
| TNRC6C-AS1 | 722.357879 | 2.516834142 | 0.309102 | 8.142406 | 3.87E-16 | 1.41E-14 | Up |
| COL15A1 | 4111.466254 | 1.306037309 | 0.172889 | 7.554184 | 4.21E-14 | 1.17E-12 | Up |
| TMEM225 | 0.862439028 | -1.849547092 | 0.68403 | -2.7039 | 0.006853 | 0.020429 | Down |
| BTBD17 | 29.92932479 | -1.429159178 | 0.310349 | -4.60501 | 4.12E-06 | 2.88E-05 | Down |
| AL590644.1 | 2.404804847 | 4.505884256 | 0.682173 | 6.605191 | 3.97E-11 | 7.09E-10 | Up |
| LAYN | 1632.651171 | -1.292251626 | 0.156138 | -8.27636 | 1.27E-16 | 4.96E-15 | Down |
| LY6G6C | 32.88136516 | 2.139289101 | 0.328983 | 6.502742 | 7.89E-11 | 1.34E-09 | Up |
| FAM155A | 128.123763 | 1.407074232 | 0.246794 | 5.701419 | 1.19E-08 | 1.37E-07 | Up |
| GUCY2EP | 1.3888559 | 1.24348225 | 0.443231 | 2.805494 | 0.005024 | 0.015682 | Up |
| PSORS1C2 | 3.364976838 | 1.029524115 | 0.414398 | 2.484387 | 0.012977 | 0.034958 | Up |
| CDSN | 14.92117133 | 3.826456844 | 0.317273 | 12.06044 | 1.71E-33 | 3.84E-31 | Up |
| C6orf15 | 1.939621219 | 3.719309713 | 0.505724 | 7.354426 | 1.92E-13 | 4.88E-12 | Up |
| MUC21 | 273.4916963 | 5.634745361 | 0.612311 | 9.202431 | 3.50E-20 | 2.10E-18 | Up |
| KRTAP5-10 | 9.760112148 | 1.341997569 | 0.230838 | 5.813579 | 6.12E-09 | 7.40E-08 | Up |
| LRCOL1 | 19.65720617 | 1.800282468 | 0.335573 | 5.364794 | 8.10E-08 | 8.05E-07 | Up |
| LINC01257 | 46.31579721 | -1.518181929 | 0.430182 | -3.52916 | 0.000417 | 0.001799 | Down |
| TRIM15 | 0.935827622 | 2.362696036 | 0.538523 | 4.387367 | 1.15E-05 | 7.28E-05 | Up |
| DISP3 | 7.615942879 | 1.18868733 | 0.211033 | 5.632707 | 1.77E-08 | 1.98E-07 | Up |
| HLA-G | 516.0939599 | 1.633941239 | 0.336753 | 4.852051 | 1.22E-06 | 9.56E-06 | Up |
| FAM153C | 18.21815683 | -1.300359695 | 0.279259 | -4.65647 | 3.22E-06 | 2.31E-05 | Down |
| C10orf113 | 1.416449384 | -1.391566543 | 0.435755 | -3.19346 | 0.001406 | 0.00521 | Down |
| INSYN2B | 30.36195038 | -1.10964549 | 0.444165 | -2.49827 | 0.01248 | 0.033835 | Down |
| IGKV1OR9-1 | 1.900376015 | 1.972728891 | 0.807373 | 2.443391 | 0.01455 | 0.038482 | Up |
| AL163540.1 | 8.22187808 | -2.126527417 | 0.397043 | -5.35592 | 8.51E-08 | 8.42E-07 | Down |
| MROH6 | 318.6377111 | 2.390319014 | 0.206475 | 11.57681 | 5.40E-31 | 1.01E-28 | Up |
| IGFL2 | 148.6087542 | 4.268267664 | 0.575898 | 7.411498 | 1.25E-13 | 3.25E-12 | Up |
| IGFL4 | 4.420547967 | 2.048977911 | 0.274922 | 7.452947 | 9.13E-14 | 2.42E-12 | Up |
| KRT25 | 0.77600811 | 2.166567732 | 0.861597 | 2.514594 | 0.011917 | 0.032567 | Up |
| LINC01545 | 13.61859194 | -1.016300441 | 0.159987 | -6.3524 | 2.12E-10 | 3.34E-09 | Down |
| CD177P1 | 0.485348707 | 2.78515121 | 1.128774 | 2.467413 | 0.013609 | 0.036397 | Up |
| ATP6V0E2-AS1 | 543.8553448 | -1.382252979 | 0.26822 | -5.15343 | 2.56E-07 | 2.31E-06 | Down |
| CD177 | 55.35665848 | 3.638940967 | 0.611541 | 5.95045 | 2.67E-09 | 3.46E-08 | Up |
| PSG5 | 1.951071576 | 2.677840545 | 0.56481 | 4.741136 | 2.13E-06 | 1.58E-05 | Up |
| PCDHA7 | 8.066848374 | 1.164188888 | 0.24116 | 4.827449 | 1.38E-06 | 1.07E-05 | Up |
| PCDHA1 | 7.233365357 | 1.186074936 | 0.240093 | 4.940073 | 7.81E-07 | 6.38E-06 | Up |
| PRSS1 | 77.71212608 | 6.160179078 | 0.544559 | 11.31223 | 1.14E-29 | 1.87E-27 | Up |
| OR4D8P | 0.376891375 | 2.677840831 | 0.986949 | 2.713252 | 0.006663 | 0.019937 | Up |
| SPIRE2 | 1434.262503 | -1.409023234 | 0.182248 | -7.73134 | 1.06E-14 | 3.23E-13 | Down |
| AC134312.1 | 5.983701885 | -1.114620204 | 0.320113 | -3.48196 | 0.000498 | 0.002102 | Down |
| PKHD1L1 | 13857.30693 | -2.351358309 | 0.346866 | -6.77887 | 1.21E-11 | 2.35E-10 | Down |
| LINC02397 | 37.77925169 | -1.864382234 | 0.596579 | -3.12512 | 0.001777 | 0.006377 | Down |
| TMEM88B | 2.142735279 | 4.064205191 | 0.570982 | 7.117918 | 1.10E-12 | 2.52E-11 | Up |
| C4orf47 | 252.9274775 | -1.150359391 | 0.138824 | -8.28648 | 1.17E-16 | 4.59E-15 | Down |
| C11orf91 | 3.358246981 | -1.082136939 | 0.376018 | -2.87789 | 0.004003 | 0.01289 | Down |
| VIT | 207.3380158 | -2.393093253 | 0.279985 | -8.54723 | 1.26E-17 | 5.45E-16 | Down |
| LINC01460 | 5.334000074 | 3.388700104 | 0.337673 | 10.03546 | 1.06E-23 | 9.49E-22 | Up |
| MT1A | 113.8372873 | -1.043714113 | 0.32037 | -3.25784 | 0.001123 | 0.004275 | Down |
| CFI | 3020.516692 | 2.078123679 | 0.183591 | 11.31934 | 1.05E-29 | 1.74E-27 | Up |
| AC007608.1 | 1.463536051 | -1.755356733 | 0.645719 | -2.71845 | 0.006559 | 0.019666 | Down |
| KRT6A | 16.20521668 | 1.987891127 | 0.546339 | 3.638567 | 0.000274 | 0.001241 | Up |
| AL592528.1 | 0.509185609 | 1.616822634 | 0.670202 | 2.412442 | 0.015846 | 0.041252 | Up |
| EXOC3L4 | 73.2845485 | -1.21140212 | 0.452655 | -2.67622 | 0.007446 | 0.021912 | Down |
| TP53TG3D | 4.918653833 | -1.131729189 | 0.350077 | -3.2328 | 0.001226 | 0.004618 | Down |
| TMSB4X | 48648.45939 | 1.000230874 | 0.261489 | 3.825139 | 0.000131 | 0.000643 | Up |
| C9orf92 | 16.74079886 | -2.021663196 | 0.232982 | -8.67733 | 4.05E-18 | 1.87E-16 | Down |
| LINC01597 | 8.898013008 | 1.170210277 | 0.372571 | 3.140908 | 0.001684 | 0.006084 | Up |
| LINC01446 | 1.82895446 | -2.052180311 | 0.694956 | -2.95297 | 0.003147 | 0.010463 | Down |
| ARSH | 3.284292502 | 1.508904602 | 0.322175 | 4.683498 | 2.82E-06 | 2.05E-05 | Up |
| ACOT6 | 3.652155034 | -1.173213078 | 0.257674 | -4.5531 | 5.29E-06 | 3.61E-05 | Down |
| TECRL | 15.59880612 | -1.808074575 | 0.514123 | -3.51681 | 0.000437 | 0.001873 | Down |
| LINC00634 | 3.115901038 | 1.525250846 | 0.350704 | 4.349112 | 1.37E-05 | 8.51E-05 | Up |
| C17orf107 | 313.5963328 | 1.232961366 | 0.207032 | 5.955425 | 2.59E-09 | 3.37E-08 | Up |
| CRLF2 | 28.52569312 | 3.500267053 | 0.434219 | 8.06107 | 7.56E-16 | 2.64E-14 | Up |
| LINC01531 | 2.827049255 | -1.897127958 | 0.381037 | -4.97885 | 6.40E-07 | 5.32E-06 | Down |
| CYS1 | 2211.831462 | 1.145359258 | 0.364105 | 3.145687 | 0.001657 | 0.006002 | Up |
| C16orf96 | 62.45882223 | -1.102044168 | 0.13492 | -8.16815 | 3.13E-16 | 1.15E-14 | Down |
| GMNC | 5.717904648 | 2.418151173 | 0.623616 | 3.877626 | 0.000105 | 0.000531 | Up |
| LINC00487 | 10.79549891 | -1.863600477 | 0.505989 | -3.68308 | 0.00023 | 0.001061 | Down |
| IFITM5 | 1.970021228 | -1.535729138 | 0.55864 | -2.74905 | 0.005977 | 0.01818 | Down |
| DOK6 | 306.8281016 | -1.015840371 | 0.190474 | -5.33323 | 9.65E-08 | 9.44E-07 | Down |
| SERPINB5 | 9.538148728 | 2.788869427 | 0.593742 | 4.697103 | 2.64E-06 | 1.92E-05 | Up |
| EGFEM1P | 35.15246782 | 3.588343465 | 0.343325 | 10.45174 | 1.44E-25 | 1.52E-23 | Up |
| GOLGA8O | 30.65128018 | -1.306431328 | 0.30069 | -4.34477 | 1.39E-05 | 8.67E-05 | Down |
| AC006305.1 | 152.0028985 | -1.863700231 | 0.258914 | -7.19815 | 6.10E-13 | 1.46E-11 | Down |
| FAM230H | 1.174478141 | 3.915580648 | 0.582904 | 6.717372 | 1.85E-11 | 3.48E-10 | Up |
| GYG2P1 | 2.199952551 | 3.016003784 | 1.102151 | 2.73647 | 0.00621 | 0.018773 | Up |
| HBA1 | 29.13172279 | -1.859254584 | 0.349682 | -5.31699 | 1.05E-07 | 1.02E-06 | Down |
| HBM | 2.157524371 | -1.900285425 | 0.559763 | -3.3948 | 0.000687 | 0.002787 | Down |
| ELOA2 | 2.658374985 | -1.501150265 | 0.323422 | -4.64146 | 3.46E-06 | 2.46E-05 | Down |
| ATP10A | 472.2394968 | 1.479029567 | 0.206136 | 7.175035 | 7.23E-13 | 1.70E-11 | Up |
| DUXAP8 | 24.03664668 | 2.279206905 | 0.292686 | 7.787202 | 6.85E-15 | 2.14E-13 | Up |
| FOXL2NB | 2.22834055 | -1.320951093 | 0.359462 | -3.6748 | 0.000238 | 0.001093 | Down |
| TMEM200C | 26.85673607 | 1.22900988 | 0.362362 | 3.391666 | 0.000695 | 0.002814 | Up |
| PRSS50 | 306.9145614 | 1.112193904 | 0.224668 | 4.950395 | 7.41E-07 | 6.08E-06 | Up |
| RF00019.382 | 1.349801708 | -1.127423807 | 0.404001 | -2.79065 | 0.00526 | 0.016305 | Down |
| RNVU1-7 | 4.002992636 | -1.890419099 | 0.366706 | -5.15514 | 2.53E-07 | 2.29E-06 | Down |
| RNU6-354P | 1.462930882 | -1.007206731 | 0.420297 | -2.39642 | 0.016556 | 0.042816 | Down |
| SNORD116-14 | 2.710584119 | -1.469965262 | 0.341875 | -4.29972 | 1.71E-05 | 0.000104 | Down |
| RNU6-1 | 0.726689136 | -1.53627105 | 0.529496 | -2.90139 | 0.003715 | 0.012081 | Down |
| SNORD60 | 3.943198709 | 1.083279524 | 0.312027 | 3.471745 | 0.000517 | 0.002173 | Up |
| RF00019.388 | 1.254001214 | -1.433607605 | 0.451333 | -3.17639 | 0.001491 | 0.005485 | Down |
| RNU1-1 | 2.087345716 | -3.76574364 | 0.57594 | -6.53843 | 6.22E-11 | 1.08E-09 | Down |
| RNU6-1189P | 9.766586487 | -1.192996707 | 0.415677 | -2.87001 | 0.004105 | 0.013174 | Down |
| SNORD116-18 | 0.789916449 | -1.400801619 | 0.508336 | -2.75566 | 0.005857 | 0.017856 | Down |
| RNVU1-18 | 1.425499662 | -2.515886512 | 0.58056 | -4.33355 | 1.47E-05 | 9.07E-05 | Down |
| RNU6-213P | 0.478891557 | -1.238574552 | 0.518389 | -2.38928 | 0.016882 | 0.043487 | Down |
| RNU1-129P | 0.425926676 | 1.780278908 | 0.688433 | 2.585987 | 0.00971 | 0.027445 | Up |
| RNU6-968P | 2.42014352 | -2.321293473 | 0.83593 | -2.7769 | 0.005488 | 0.016891 | Down |
| RNU1-138P | 2.40496413 | 3.154973865 | 0.460883 | 6.845503 | 7.62E-12 | 1.52E-10 | Up |
| RF00003 | 10.85982611 | -1.817980932 | 0.354664 | -5.12593 | 2.96E-07 | 2.64E-06 | Down |
| SNORD116-2 | 4.566051391 | -1.863417296 | 0.298091 | -6.25118 | 4.07E-10 | 6.08E-09 | Down |
| RNU1-2 | 2.469071703 | -2.614294791 | 0.494431 | -5.28748 | 1.24E-07 | 1.18E-06 | Down |
| SNORD116-3 | 0.644093372 | -1.268044256 | 0.495633 | -2.55843 | 0.010515 | 0.02931 | Down |
| RF00019.465 | 0.798316126 | -1.536943415 | 0.605047 | -2.54021 | 0.011079 | 0.030604 | Down |
| RF00019.468 | 1.536696999 | -1.558615321 | 0.455144 | -3.42444 | 0.000616 | 0.002538 | Down |
| SNORD116-1 | 5.241002317 | -1.18974542 | 0.292805 | -4.06326 | 4.84E-05 | 0.000265 | Down |
| RNU6-242P | 1.538438492 | -2.087160377 | 0.44457 | -4.69479 | 2.67E-06 | 1.94E-05 | Down |
| SNORD116-8 | 0.981931776 | -1.743891817 | 0.478486 | -3.6446 | 0.000268 | 0.001216 | Down |
| SNORD116-13 | 2.708572414 | -1.635162162 | 0.362159 | -4.51504 | 6.33E-06 | 4.25E-05 | Down |
| RF00019.491 | 2.080267215 | 1.865672746 | 0.418551 | 4.457451 | 8.29E-06 | 5.44E-05 | Up |
| RNU6-45P | 1.09928554 | -1.40441231 | 0.448021 | -3.1347 | 0.00172 | 0.006198 | Down |
| RNVU1-15 | 12.7964725 | -1.901437706 | 0.260083 | -7.31088 | 2.65E-13 | 6.63E-12 | Down |
| RF00019.501 | 2.944973648 | -1.924359935 | 0.428953 | -4.48618 | 7.25E-06 | 4.81E-05 | Down |
| SNORD116-29 | 0.569050099 | -1.453072335 | 0.498257 | -2.91631 | 0.003542 | 0.011586 | Down |
| SNORD116-16 | 1.2437155 | -1.518474769 | 0.459821 | -3.30231 | 0.000959 | 0.003731 | Down |
| RNU6-1160P | 2.237199075 | -1.837619308 | 0.39606 | -4.63975 | 3.49E-06 | 2.48E-05 | Down |
| SNORD116-24 | 18.79668844 | -1.480998309 | 0.237481 | -6.23628 | 4.48E-10 | 6.64E-09 | Down |
| RNU6-30P | 14.09498154 | 1.522671687 | 0.223803 | 6.80362 | 1.02E-11 | 2.00E-10 | Up |
| RNU6-851P | 0.923384228 | -1.069550804 | 0.447413 | -2.39052 | 0.016824 | 0.043372 | Down |
| RNU6-883P | 4.926743829 | -1.239078933 | 0.265166 | -4.67284 | 2.97E-06 | 2.15E-05 | Down |
| RNVU1-1 | 1.524054365 | 1.168190667 | 0.47482 | 2.460281 | 0.013883 | 0.037002 | Up |
| RNU6-2 | 7.023146588 | -3.17824181 | 0.420562 | -7.55712 | 4.12E-14 | 1.14E-12 | Down |
| RF00019.540 | 1.70772346 | -1.013459062 | 0.364716 | -2.77876 | 0.005457 | 0.016809 | Down |
| SNORA20 | 0.940276528 | -1.387063305 | 0.568007 | -2.44198 | 0.014607 | 0.038608 | Down |
| RNU6-768P | 1.754810007 | -1.919912546 | 0.44716 | -4.29357 | 1.76E-05 | 0.000107 | Down |
| SNORD116-6 | 2.019027411 | -1.904595888 | 0.383663 | -4.96424 | 6.90E-07 | 5.69E-06 | Down |
| SNORD15B | 2.931462621 | -1.010369051 | 0.366915 | -2.75369 | 0.005893 | 0.017947 | Down |
| RF00019.572 | 3.271377488 | -1.283239635 | 0.504372 | -2.54423 | 0.010952 | 0.03032 | Down |
| MIR578 | 0.692952128 | -1.553436624 | 0.596756 | -2.60314 | 0.009238 | 0.026328 | Down |
| MIR554 | 1.359381129 | -2.397415213 | 0.584506 | -4.10161 | 4.10E-05 | 0.000228 | Down |
| MIR193A | 1.559420731 | -1.343964509 | 0.45314 | -2.96589 | 0.003018 | 0.010092 | Down |
| MIR7-2 | 3.396094316 | -2.515678515 | 0.427176 | -5.88909 | 3.88E-09 | 4.88E-08 | Down |
| MIR222 | 2.894622511 | 1.964196514 | 0.531184 | 3.697769 | 0.000218 | 0.001007 | Up |
| MIR221 | 21.1784448 | 1.873338053 | 0.417698 | 4.484905 | 7.29E-06 | 4.84E-05 | Up |
| MIR138-1 | 9.539061726 | -2.345103382 | 0.346273 | -6.7724 | 1.27E-11 | 2.45E-10 | Down |
| MIR616 | 22.22285435 | -1.486956129 | 0.262193 | -5.67123 | 1.42E-08 | 1.61E-07 | Down |
| MIR3065 | 0.520538408 | 1.450618895 | 0.601191 | 2.412907 | 0.015826 | 0.041214 | Up |
| MIR760 | 1.439844497 | -1.377125462 | 0.471057 | -2.92348 | 0.003461 | 0.011372 | Down |
| AP000346.1 | 12.08763506 | 1.066992141 | 0.28273 | 3.773888 | 0.000161 | 0.000774 | Up |
| TRDV2 | 0.942111403 | 1.696581092 | 0.660418 | 2.568951 | 0.010201 | 0.028569 | Up |
| TRDJ2 | 1.099794887 | 3.201315535 | 0.845541 | 3.786117 | 0.000153 | 0.000741 | Up |
| TRDJ3 | 0.543454157 | 2.030555292 | 0.714824 | 2.840638 | 0.004502 | 0.014269 | Up |
| TRDC | 548.2489908 | 2.960655029 | 0.446467 | 6.631298 | 3.33E-11 | 6.00E-10 | Up |
| IGHE | 294.2089952 | -1.285326318 | 0.456452 | -2.81591 | 0.004864 | 0.01524 | Down |
| IGHG2 | 66315.20817 | 2.423567216 | 0.671386 | 3.609797 | 0.000306 | 0.00137 | Up |
| IGHD | 725.1293136 | -1.495733196 | 0.598623 | -2.49862 | 0.012468 | 0.033806 | Down |
| IGHV1-2 | 2707.809009 | 2.248191877 | 0.71196 | 3.157749 | 0.00159 | 0.005799 | Up |
| IGHV2-5 | 274.98564 | 1.724755328 | 0.729554 | 2.364122 | 0.018073 | 0.046022 | Up |
| IGHV4-34 | 2160.087285 | 1.751590544 | 0.710072 | 2.466778 | 0.013633 | 0.036448 | Up |
| IGHV7-81 | 38.74733723 | 3.054864051 | 0.786031 | 3.886442 | 0.000102 | 0.000514 | Up |
| MIR374B | 2.316177362 | -1.139881671 | 0.340441 | -3.34825 | 0.000813 | 0.003232 | Down |
| RF00568 | 0.832195458 | -2.158468103 | 0.648095 | -3.33048 | 0.000867 | 0.003415 | Down |
| RF00012.18 | 6.798937776 | -3.588451914 | 0.486772 | -7.37193 | 1.68E-13 | 4.30E-12 | Down |
| RF00019.576 | 1.20335659 | -1.11275762 | 0.425645 | -2.61428 | 0.008941 | 0.025618 | Down |
| RF00265 | 2.169108567 | 2.379168445 | 0.409241 | 5.813615 | 6.11E-09 | 7.40E-08 | Up |
| RNU6-724P | 0.517570808 | -1.216470472 | 0.50471 | -2.41024 | 0.015942 | 0.041467 | Down |
| RF00404 | 4.059942523 | -1.053980335 | 0.343754 | -3.06609 | 0.002169 | 0.007579 | Down |
| RNY4P36 | 1.953371337 | -1.828275069 | 0.461707 | -3.95982 | 7.50E-05 | 0.000391 | Down |
| RF00568.4 | 1.170400022 | -1.174819224 | 0.469956 | -2.49985 | 0.012425 | 0.033721 | Down |
| KRTAP2-3 | 11.59847972 | 3.917665049 | 0.645286 | 6.071203 | 1.27E-09 | 1.74E-08 | Up |
| LINC02656 | 10.73407362 | 1.014814582 | 0.238633 | 4.252621 | 2.11E-05 | 0.000126 | Up |
| EWSAT1 | 5.830379248 | 1.334419081 | 0.241927 | 5.515792 | 3.47E-08 | 3.68E-07 | Up |
| OR2A42 | 3.083878633 | -1.639745251 | 0.365768 | -4.48302 | 7.36E-06 | 4.88E-05 | Down |
| AC093281.1 | 0.446227454 | 1.420984519 | 0.591626 | 2.401828 | 0.016313 | 0.042278 | Up |
| HNRNPA1P40 | 36.41721308 | -1.48239168 | 0.217424 | -6.81796 | 9.23E-12 | 1.83E-10 | Down |
| AURKAP1 | 4.472064036 | -1.280484504 | 0.306867 | -4.17277 | 3.01E-05 | 0.000173 | Down |
| AL390955.1 | 2.322133694 | -1.120597596 | 0.336197 | -3.33316 | 0.000859 | 0.003387 | Down |
| CFAP45 | 93.62469757 | -1.201849295 | 0.217256 | -5.53196 | 3.17E-08 | 3.37E-07 | Down |
| CHCHD2P4 | 1.26876877 | 1.33926007 | 0.409181 | 3.273028 | 0.001064 | 0.004084 | Up |
| AL590867.1 | 11.3562497 | -2.588511737 | 0.344603 | -7.51157 | 5.84E-14 | 1.59E-12 | Down |
| EEF1DP5 | 0.941573265 | -1.675938439 | 0.589372 | -2.8436 | 0.004461 | 0.014157 | Down |
| AC084880.1 | 65.02657819 | -1.812943758 | 0.289155 | -6.2698 | 3.62E-10 | 5.44E-09 | Down |
| RF00017.6 | 0.970577544 | -1.365462807 | 0.533606 | -2.55894 | 0.010499 | 0.029273 | Down |
| YBX1P4 | 1.76965762 | -1.449464926 | 0.359353 | -4.03354 | 5.49E-05 | 0.000297 | Down |
| STRADBP1 | 0.932969974 | -1.558648073 | 0.573194 | -2.71923 | 0.006543 | 0.019624 | Down |
| NOC2LP1 | 205.0964412 | -1.556793947 | 0.283812 | -5.48529 | 4.13E-08 | 4.32E-07 | Down |
| PCNPP3 | 2.592205633 | 3.49413207 | 0.422785 | 8.264563 | 1.40E-16 | 5.42E-15 | Up |
| AP000593.1 | 1.714443301 | -1.666356909 | 0.38461 | -4.33258 | 1.47E-05 | 9.10E-05 | Down |
| AC022217.1 | 6.103783297 | -2.179233715 | 0.395177 | -5.51458 | 3.50E-08 | 3.70E-07 | Down |
| ARHGAP19 | 954.2987638 | 1.165528312 | 0.140832 | 8.276041 | 1.27E-16 | 4.97E-15 | Up |
| C1QBPP2 | 1.139994612 | 1.23635623 | 0.406857 | 3.038799 | 0.002375 | 0.008197 | Up |
| GPC2 | 17.88603658 | 1.007066459 | 0.287597 | 3.501655 | 0.000462 | 0.00197 | Up |
| KRT222 | 51.31000061 | 1.960429521 | 0.261137 | 7.50728 | 6.04E-14 | 1.64E-12 | Up |
| IMPDH1P5 | 35.48176599 | -1.34441587 | 0.260533 | -5.16026 | 2.47E-07 | 2.23E-06 | Down |
| AC016769.1 | 1.865169406 | 1.475118937 | 0.300543 | 4.908174 | 9.19E-07 | 7.39E-06 | Up |
| SLC25A1P3 | 0.730316199 | -1.389721135 | 0.481931 | -2.88365 | 0.003931 | 0.012684 | Down |
| AC098828.1 | 1.121309818 | 1.095920421 | 0.470206 | 2.330726 | 0.019768 | 0.049644 | Up |
| RPS10P14 | 1.428572718 | -1.071161499 | 0.414694 | -2.58301 | 0.009794 | 0.027641 | Down |
| AC093270.1 | 5.26480176 | -1.306527497 | 0.263106 | -4.96578 | 6.84E-07 | 5.66E-06 | Down |
| UGT2B11 | 59.80482913 | -2.958734274 | 0.272104 | -10.8735 | 1.54E-27 | 2.04E-25 | Down |
| AC107956.2 | 1.394885004 | -1.139432921 | 0.475799 | -2.39478 | 0.01663 | 0.04299 | Down |
| RPL7AP7 | 0.864364637 | 1.208079477 | 0.434047 | 2.783292 | 0.005381 | 0.016621 | Up |
| PPM1N | 44.35915054 | 1.28224197 | 0.259286 | 4.945272 | 7.60E-07 | 6.22E-06 | Up |
| HBE1 | 4.333340248 | 1.187735871 | 0.266885 | 4.450364 | 8.57E-06 | 5.60E-05 | Up |
| CLDN9 | 279.1164196 | 2.119367328 | 0.368555 | 5.750478 | 8.90E-09 | 1.05E-07 | Up |
| SEPHS1P6 | 38.19112141 | -1.650805304 | 0.235074 | -7.02248 | 2.18E-12 | 4.76E-11 | Down |
| API5P2 | 0.669144916 | -1.824249782 | 0.577222 | -3.1604 | 0.001576 | 0.005749 | Down |
| ZNF99 | 12.64957165 | 1.013725348 | 0.251541 | 4.030055 | 5.58E-05 | 0.000301 | Up |
| AC078899.1 | 251.7881213 | -1.022327245 | 0.182192 | -5.61127 | 2.01E-08 | 2.22E-07 | Down |
| PGAM1P4 | 8.647727111 | 1.865836948 | 0.291877 | 6.392548 | 1.63E-10 | 2.61E-09 | Up |
| LINC02347 | 2.600956195 | 2.751054358 | 0.531783 | 5.173261 | 2.30E-07 | 2.10E-06 | Up |
| UCA1 | 3.901485419 | 1.728166647 | 0.46104 | 3.748409 | 0.000178 | 0.000845 | Up |
| AL360182.1 | 1.029887389 | -1.520753789 | 0.456016 | -3.33487 | 0.000853 | 0.00337 | Down |
| PLAC9P1 | 7.491861344 | -1.780800719 | 0.284509 | -6.2592 | 3.87E-10 | 5.82E-09 | Down |
| LINC00887 | 61.33083641 | 2.230898273 | 0.279848 | 7.971834 | 1.56E-15 | 5.28E-14 | Up |
| KCTD9P4 | 9.707573929 | -1.105160811 | 0.283813 | -3.89398 | 9.86E-05 | 0.0005 | Down |
| AC046134.1 | 10.45135872 | -1.13009489 | 0.194176 | -5.81997 | 5.89E-09 | 7.16E-08 | Down |
| PDCL3P3 | 0.688729483 | 1.202191781 | 0.483852 | 2.484626 | 0.012969 | 0.034942 | Up |
| RPL39P5 | 8.104978809 | -1.838994229 | 0.318729 | -5.76978 | 7.94E-09 | 9.41E-08 | Down |
| FOXO1B | 2.550832794 | -3.334206074 | 0.578982 | -5.75874 | 8.47E-09 | 1.00E-07 | Down |
| VAC14-AS1 | 11.75520183 | 1.930003765 | 0.298962 | 6.455679 | 1.08E-10 | 1.78E-09 | Up |
| NEURL1B | 1087.388309 | 1.14992862 | 0.139375 | 8.250622 | 1.58E-16 | 6.06E-15 | Up |
| LINC00488 | 0.619402627 | -1.644097714 | 0.612841 | -2.68275 | 0.007302 | 0.021568 | Down |
| RPL7P29 | 1.779696172 | -2.476684908 | 0.46036 | -5.37988 | 7.45E-08 | 7.45E-07 | Down |
| SMARCE1P6 | 4.903599462 | -1.239290407 | 0.283783 | -4.36703 | 1.26E-05 | 7.91E-05 | Down |
| SEC14L6 | 33.60821575 | 2.268558012 | 0.226039 | 10.03615 | 1.06E-23 | 9.45E-22 | Up |
| KRT42P | 1.607282667 | 1.481708557 | 0.43781 | 3.384361 | 0.000713 | 0.002878 | Up |
| AC087491.1 | 1.723605729 | 2.062297461 | 0.377069 | 5.46929 | 4.52E-08 | 4.71E-07 | Up |
| LRRC37A11P | 12.30727332 | -1.118615661 | 0.221119 | -5.05888 | 4.22E-07 | 3.63E-06 | Down |
| EML6 | 902.1483575 | -1.082090525 | 0.161736 | -6.69048 | 2.22E-11 | 4.13E-10 | Down |
| AL513122.1 | 0.669591965 | -1.368749931 | 0.506787 | -2.70084 | 0.006917 | 0.020598 | Down |
| RPL6P12 | 1.089289216 | -1.89211019 | 0.491776 | -3.8475 | 0.000119 | 0.000593 | Down |
| AC004870.1 | 2.561930523 | -1.528972373 | 0.496968 | -3.0766 | 0.002094 | 0.007347 | Down |
| MS4A4E | 17.3433662 | 1.18882641 | 0.297136 | 4.000948 | 6.31E-05 | 0.000335 | Up |
| AP002358.1 | 109.9405648 | 5.929828891 | 0.50483 | 11.74619 | 7.39E-32 | 1.47E-29 | Up |
| FER1L6 | 250.9001466 | -2.175044882 | 0.407539 | -5.33702 | 9.45E-08 | 9.27E-07 | Down |
| RAD51AP2 | 5.539178553 | -1.308666092 | 0.272715 | -4.79867 | 1.60E-06 | 1.22E-05 | Down |
| LINC00612 | 25.68405863 | -1.551797214 | 0.190753 | -8.13512 | 4.12E-16 | 1.49E-14 | Down |
| APOC1P1 | 18.30274541 | 3.718798462 | 0.486357 | 7.646239 | 2.07E-14 | 6.02E-13 | Up |
| DCDC2C | 3.458721294 | -2.426799686 | 0.390708 | -6.21129 | 5.26E-10 | 7.66E-09 | Down |
| AC004540.1 | 26.79365505 | 1.82380592 | 0.216153 | 8.437569 | 3.24E-17 | 1.34E-15 | Up |
| ARHGEF28 | 2975.333852 | -1.068373997 | 0.155467 | -6.87202 | 6.33E-12 | 1.28E-10 | Down |
| HSPD1P7 | 0.957960943 | -1.558600887 | 0.60661 | -2.56936 | 0.010189 | 0.02854 | Down |
| AL590763.1 | 1.171512325 | -1.759617886 | 0.467481 | -3.76404 | 0.000167 | 0.000801 | Down |
| AC138409.1 | 5.103908761 | -1.357550692 | 0.334563 | -4.05768 | 4.96E-05 | 0.000271 | Down |
| TCEA1P3 | 3.526510558 | -2.081188972 | 0.360951 | -5.76586 | 8.12E-09 | 9.62E-08 | Down |
| C5orf49 | 122.7715551 | -1.840254895 | 0.218078 | -8.43852 | 3.21E-17 | 1.33E-15 | Down |
| UBE2QL1 | 628.4859197 | 2.336649765 | 0.202615 | 11.53245 | 9.05E-31 | 1.66E-28 | Up |
| CYP51A1P3 | 3.56974956 | -1.147287653 | 0.291186 | -3.94005 | 8.15E-05 | 0.000421 | Down |
| AC116351.1 | 27.58616836 | 1.006247638 | 0.287629 | 3.498417 | 0.000468 | 0.001991 | Up |
| GOLGA8B | 5232.909386 | 1.045253524 | 0.241283 | 4.332073 | 1.48E-05 | 9.12E-05 | Up |
| AP000523.1 | 2.209001753 | 1.466835573 | 0.324833 | 4.515659 | 6.31E-06 | 4.24E-05 | Up |
| TMCO5B | 9.338091027 | -1.643274166 | 0.409012 | -4.01767 | 5.88E-05 | 0.000315 | Down |
| AC135983.1 | 10.40566031 | -1.191084898 | 0.183767 | -6.4815 | 9.08E-11 | 1.52E-09 | Down |
| TMED11P | 1.71662434 | -1.040486236 | 0.382603 | -2.71949 | 0.006538 | 0.01961 | Down |
| AL022342.1 | 2.61414434 | -1.16800943 | 0.311743 | -3.74671 | 0.000179 | 0.000851 | Down |
| SKOR2 | 0.941889479 | 2.994220495 | 0.842111 | 3.555613 | 0.000377 | 0.001645 | Up |
| BCRP3 | 146.971873 | 1.4142594 | 0.193086 | 7.324505 | 2.40E-13 | 6.04E-12 | Up |
| FAM230B | 6.014421948 | 4.61207551 | 0.500683 | 9.211573 | 3.21E-20 | 1.95E-18 | Up |
| FRG1JP | 88.98379197 | 1.009020387 | 0.288631 | 3.49588 | 0.000473 | 0.002008 | Up |
| C20orf202 | 14.4984724 | 1.189745255 | 0.172262 | 6.906606 | 4.96E-12 | 1.02E-10 | Up |
| GUSBP9 | 18.39855564 | -1.318116309 | 0.270962 | -4.86458 | 1.15E-06 | 9.04E-06 | Down |
| LRRC37A14P | 1.377302247 | -1.443165082 | 0.509334 | -2.83343 | 0.004605 | 0.014551 | Down |
| KRT18P65 | 1.774466399 | 1.383157229 | 0.392922 | 3.520184 | 0.000431 | 0.001853 | Up |
| AL451074.1 | 3.703267404 | -1.118286896 | 0.252959 | -4.42082 | 9.83E-06 | 6.33E-05 | Down |
| ST13P20 | 3.550596724 | -2.120727745 | 0.372916 | -5.68688 | 1.29E-08 | 1.48E-07 | Down |
| TTC34 | 212.8464897 | -1.18027491 | 0.175385 | -6.72963 | 1.70E-11 | 3.21E-10 | Down |
| HIST1H1PS1 | 6.71221838 | 1.348211318 | 0.273664 | 4.926518 | 8.37E-07 | 6.79E-06 | Up |
| AL390237.1 | 0.601455169 | -1.535033493 | 0.622446 | -2.46613 | 0.013658 | 0.036505 | Down |
| AC093850.1 | 0.841641526 | 2.216340501 | 0.448826 | 4.938082 | 7.89E-07 | 6.44E-06 | Up |
| LY86-AS1 | 5.349464327 | -1.779799157 | 0.56424 | -3.15433 | 0.001609 | 0.005857 | Down |
| HSPD1P10 | 2.407259246 | -1.103240126 | 0.306907 | -3.5947 | 0.000325 | 0.001442 | Down |
| CYP2AC1P | 0.612252411 | -1.4856541 | 0.56098 | -2.64832 | 0.008089 | 0.02352 | Down |
| AL031777.1 | 8.007712514 | 1.444588084 | 0.276545 | 5.223702 | 1.75E-07 | 1.63E-06 | Up |
| UQCRFS1P3 | 2.704204499 | -1.223410923 | 0.316147 | -3.86976 | 0.000109 | 0.000547 | Down |
| DDX18P3 | 2.995374388 | -2.673222104 | 0.45352 | -5.89439 | 3.76E-09 | 4.73E-08 | Down |
| RPS16P5 | 2.317329526 | -1.98098162 | 0.452138 | -4.38136 | 1.18E-05 | 7.46E-05 | Down |
| CKLF | 446.4143156 | 1.167181252 | 0.155758 | 7.493573 | 6.70E-14 | 1.81E-12 | Up |
| AL353692.1 | 1.939397189 | -1.404905693 | 0.38369 | -3.66156 | 0.000251 | 0.001145 | Down |
| RPS3AP24 | 2.307291182 | -1.970008461 | 0.41796 | -4.71339 | 2.44E-06 | 1.79E-05 | Down |
| AL390719.1 | 128.9238768 | 1.209317406 | 0.165311 | 7.315422 | 2.57E-13 | 6.43E-12 | Up |
| KRT19P1 | 2.006978308 | 1.645012283 | 0.416145 | 3.952977 | 7.72E-05 | 0.000401 | Up |
| THAP12P5 | 2.183262032 | -2.469627846 | 0.477459 | -5.17245 | 2.31E-07 | 2.10E-06 | Down |
| AL021407.3 | 5.113172727 | -1.095821784 | 0.338973 | -3.23277 | 0.001226 | 0.004618 | Down |
| AL591034.1 | 6.278532625 | -3.518909383 | 0.472159 | -7.45281 | 9.14E-14 | 2.43E-12 | Down |
| HIST1H2APS3 | 1.520750725 | 1.979457871 | 0.49583 | 3.992212 | 6.55E-05 | 0.000347 | Up |
| CDC14C | 0.749146792 | -1.53624671 | 0.510531 | -3.00912 | 0.00262 | 0.008927 | Down |
| AC110619.1 | 27.37201129 | 1.695479612 | 0.200891 | 8.439783 | 3.18E-17 | 1.32E-15 | Up |
| AL353133.1 | 1.291790347 | -3.007226435 | 0.489344 | -6.14543 | 7.97E-10 | 1.13E-08 | Down |
| AL033519.1 | 1.822965701 | -2.369652321 | 0.484243 | -4.89352 | 9.90E-07 | 7.91E-06 | Down |
| AL391903.1 | 0.886888263 | 1.509702848 | 0.505559 | 2.986208 | 0.002825 | 0.009525 | Up |
| PAPOLB | 4.259456123 | -2.629620806 | 0.380512 | -6.91074 | 4.82E-12 | 9.97E-11 | Down |
| AL138725.1 | 7.738757789 | -2.179250866 | 0.308236 | -7.07006 | 1.55E-12 | 3.46E-11 | Down |
| RPS3AP2 | 2.697821465 | -1.776356997 | 0.364707 | -4.87064 | 1.11E-06 | 8.79E-06 | Down |
| AC011298.1 | 151.2488347 | -2.095169802 | 0.346055 | -6.05444 | 1.41E-09 | 1.91E-08 | Down |
| FTH1P19 | 1.015986753 | 1.71424146 | 0.489387 | 3.502832 | 0.00046 | 0.001963 | Up |
| AMZ2P2 | 1.043840892 | -1.322400642 | 0.53698 | -2.46266 | 0.013791 | 0.036788 | Down |
| CATSPERZ | 2.391838194 | -1.904732822 | 0.371213 | -5.13111 | 2.88E-07 | 2.57E-06 | Down |
| FAM19A5 | 89.3971842 | 2.103525065 | 0.258341 | 8.142422 | 3.87E-16 | 1.41E-14 | Up |
| SELENOKP2 | 1.252434073 | -1.124884603 | 0.447496 | -2.51373 | 0.011946 | 0.032628 | Down |
| GSTA8P | 0.460271701 | -1.610093868 | 0.569139 | -2.829 | 0.004669 | 0.014718 | Down |
| KRT8P42 | 8.172087983 | -1.041014734 | 0.385611 | -2.69965 | 0.006941 | 0.020657 | Down |
| VN1R12P | 0.874917737 | -1.140834315 | 0.483642 | -2.35884 | 0.018332 | 0.04654 | Down |
| SSR1P1 | 33.51353508 | -1.75516244 | 0.349977 | -5.01507 | 5.30E-07 | 4.48E-06 | Down |
| OR1F12 | 1.926328987 | -1.102286641 | 0.358033 | -3.07873 | 0.002079 | 0.0073 | Down |
| LINC02531 | 3.399511156 | -1.539255373 | 0.655115 | -2.34959 | 0.018794 | 0.047509 | Down |
| RNU6ATAC38P | 0.441967784 | -1.70267092 | 0.620155 | -2.74556 | 0.006041 | 0.018348 | Down |
| RF00599 | 1.638520869 | -1.453120879 | 0.525811 | -2.76358 | 0.005717 | 0.017501 | Down |
| SNORD88A | 0.823740218 | -1.169496414 | 0.493 | -2.3722 | 0.017682 | 0.045196 | Down |
| RNU6ATAC18P | 18.40627708 | 1.677947216 | 0.262368 | 6.395396 | 1.60E-10 | 2.57E-09 | Up |
| RNU6ATAC | 2.487347455 | -1.027056887 | 0.351373 | -2.92298 | 0.003467 | 0.011389 | Down |
| PSG3 | 0.778463353 | 2.400480728 | 0.722238 | 3.323669 | 0.000888 | 0.003491 | Up |
| OR2A5 | 0.959258213 | -1.246030935 | 0.501171 | -2.48624 | 0.01291 | 0.03483 | Down |
| PLXNA4 | 940.1193407 | 2.155824379 | 0.295676 | 7.291173 | 3.07E-13 | 7.61E-12 | Up |
| NPTXR | 1793.47201 | 1.510682864 | 0.130326 | 11.59159 | 4.55E-31 | 8.62E-29 | Up |
| OR2A1 | 6.594149222 | -1.589299393 | 0.265614 | -5.98349 | 2.18E-09 | 2.88E-08 | Down |
| MYBPHL | 17.20932488 | 1.976870675 | 0.253852 | 7.787478 | 6.84E-15 | 2.14E-13 | Up |
| OR2A2 | 0.859072956 | -1.540968712 | 0.57065 | -2.70038 | 0.006926 | 0.020622 | Down |
| AC112721.1 | 1.781668927 | 1.260393198 | 0.419963 | 3.001199 | 0.002689 | 0.009128 | Up |
| AC112721.2 | 2.298845667 | 1.398066489 | 0.499087 | 2.80125 | 0.005091 | 0.015858 | Up |
| AL031587.1 | 2.570938015 | -1.100025312 | 0.365297 | -3.01132 | 0.002601 | 0.008871 | Down |
| C10orf55 | 57.82414989 | 2.920026536 | 0.281184 | 10.38476 | 2.91E-25 | 3.02E-23 | Up |
| RN7SKP285 | 1.204391641 | -2.098319058 | 0.488766 | -4.2931 | 1.76E-05 | 0.000107 | Down |
| RNU2-28P | 0.876852836 | -2.191256106 | 0.512383 | -4.2766 | 1.90E-05 | 0.000114 | Down |
| RNU2-59P | 1.038211287 | -2.38020967 | 0.727709 | -3.27083 | 0.001072 | 0.004113 | Down |
| RNA5SP296 | 0.55293205 | 1.890661228 | 0.499601 | 3.784343 | 0.000154 | 0.000746 | Up |
| RN7SKP271 | 1.728207661 | -1.301756573 | 0.34703 | -3.75113 | 0.000176 | 0.000837 | Down |
| RNU2-70P | 0.753567471 | -2.1770451 | 0.512213 | -4.25028 | 2.14E-05 | 0.000127 | Down |
| RNU2-63P | 6.841229792 | -3.631508356 | 0.499733 | -7.2669 | 3.68E-13 | 9.02E-12 | Down |
| RN7SKP106 | 0.574360493 | 2.118187609 | 0.596858 | 3.548896 | 0.000387 | 0.001682 | Up |
| RNU4-22P | 2.341312711 | -1.168826276 | 0.365647 | -3.1966 | 0.001391 | 0.005159 | Down |
| RF00019.620 | 1.238762991 | 1.023203038 | 0.337837 | 3.02869 | 0.002456 | 0.008444 | Up |
| RNA5SP155 | 1.009286605 | -1.168102255 | 0.474029 | -2.4642 | 0.013732 | 0.036662 | Down |
| RN7SKP296 | 1.055048453 | 1.849140624 | 0.45287 | 4.083157 | 4.44E-05 | 0.000246 | Up |
| RF00019.623 | 1.247354532 | -1.057099398 | 0.391168 | -2.70242 | 0.006884 | 0.020512 | Down |
| RNA5SP111 | 3.390665344 | 1.715290778 | 0.313649 | 5.46883 | 4.53E-08 | 4.72E-07 | Up |
| RN7SKP273 | 0.483363759 | -1.393304449 | 0.584954 | -2.3819 | 0.017223 | 0.04421 | Down |
| RNU2-6P | 0.891983859 | -1.355851254 | 0.575907 | -2.35429 | 0.018558 | 0.047017 | Down |
| CLDN10-AS1 | 1.170126503 | 2.999705941 | 0.66749 | 4.49401 | 6.99E-06 | 4.65E-05 | Up |
| AL118511.1 | 34.86462282 | -1.167947139 | 0.145951 | -8.0023 | 1.22E-15 | 4.18E-14 | Down |
| MEG9 | 4.628540812 | 2.36081816 | 0.394198 | 5.988908 | 2.11E-09 | 2.79E-08 | Up |
| AC006022.1 | 2.108316385 | 4.368539201 | 0.832521 | 5.247364 | 1.54E-07 | 1.45E-06 | Up |
| TMSB4XP3 | 0.891584811 | 3.025785326 | 0.719511 | 4.205335 | 2.61E-05 | 0.000152 | Up |
| AL451065.1 | 0.660463885 | -1.976371131 | 0.486218 | -4.06479 | 4.81E-05 | 0.000264 | Down |
| LINC01615 | 10.52216174 | 2.009764569 | 0.295301 | 6.805806 | 1.00E-11 | 1.98E-10 | Up |
| KIF28P | 10.85637029 | -1.186259773 | 0.200537 | -5.91542 | 3.31E-09 | 4.21E-08 | Down |
| TMSB4XP4 | 32.88211574 | 1.165772337 | 0.272723 | 4.274561 | 1.92E-05 | 0.000115 | Up |
| SMPD4P1 | 1.69602486 | -1.437553762 | 0.435862 | -3.29819 | 0.000973 | 0.00378 | Down |
| AC005165.1 | 12.65963805 | -1.095106854 | 0.282668 | -3.87418 | 0.000107 | 0.000538 | Down |
| TINCR | 55.15461408 | 3.99231869 | 0.35388 | 11.28157 | 1.62E-29 | 2.61E-27 | Up |
| RFPL4A | 0.953662071 | -2.235318657 | 0.642852 | -3.47719 | 0.000507 | 0.002136 | Down |
| RPS24P14 | 1.580315911 | -1.523867067 | 0.41371 | -3.68342 | 0.00023 | 0.00106 | Down |
| DIP2A-IT1 | 1.658066239 | -1.380702294 | 0.395879 | -3.48769 | 0.000487 | 0.002063 | Down |
| AC027612.1 | 2.322484139 | 1.698867388 | 0.325122 | 5.225319 | 1.74E-07 | 1.62E-06 | Up |
| LINC01208 | 2.042324723 | 2.731635538 | 0.463598 | 5.892247 | 3.81E-09 | 4.79E-08 | Up |
| BX842568.2 | 21.46558913 | -1.421587049 | 0.185705 | -7.65509 | 1.93E-14 | 5.65E-13 | Down |
| RAD17P2 | 0.760452378 | -1.436639558 | 0.600218 | -2.39353 | 0.016687 | 0.043086 | Down |
| Z83838.1 | 1.714022358 | -1.123611277 | 0.367754 | -3.05534 | 0.002248 | 0.007814 | Down |
| LINC02593 | 222.9217242 | 2.190219376 | 0.223069 | 9.818559 | 9.37E-23 | 7.39E-21 | Up |
| LINP1 | 6.194153478 | 1.037589373 | 0.36126 | 2.872141 | 0.004077 | 0.0131 | Up |
| AL357507.1 | 1.423103282 | -2.164383069 | 0.508679 | -4.25491 | 2.09E-05 | 0.000124 | Down |
| CERS1 | 73.59547768 | 1.007546848 | 0.18645 | 5.403847 | 6.52E-08 | 6.60E-07 | Up |
| BOLA3P4 | 0.763943941 | -1.714353646 | 0.536068 | -3.19801 | 0.001384 | 0.005136 | Down |
| AC007255.1 | 125.4944134 | 4.813042452 | 0.36416 | 13.21683 | 7.02E-40 | 3.06E-37 | Up |
| AC007091.1 | 5.574856453 | -1.665280562 | 0.398575 | -4.17809 | 2.94E-05 | 0.000169 | Down |
| FAM95B1 | 4.967244288 | -1.381736668 | 0.285392 | -4.84155 | 1.29E-06 | 1.00E-05 | Down |
| GNL2P1 | 1.623094886 | -2.778388865 | 0.497331 | -5.5866 | 2.32E-08 | 2.53E-07 | Down |
| LINC02471 | 282.167209 | 5.385168152 | 0.52609 | 10.23622 | 1.36E-24 | 1.31E-22 | Up |
| LINC01014 | 8.38626437 | -1.093121267 | 0.322435 | -3.39021 | 0.000698 | 0.002824 | Down |
| AC104809.1 | 2.208164208 | -1.222507648 | 0.396229 | -3.08536 | 0.002033 | 0.007159 | Down |
| LINC01449 | 0.91731406 | 1.577641896 | 0.507702 | 3.107415 | 0.001887 | 0.006718 | Up |
| MIR181A2HG | 129.78549 | 1.766153559 | 0.202575 | 8.718514 | 2.82E-18 | 1.33E-16 | Up |
| GAPDHP55 | 1.097966563 | 1.553666472 | 0.393723 | 3.946092 | 7.94E-05 | 0.000411 | Up |
| LINC00691 | 0.864321959 | -1.714105011 | 0.628682 | -2.72651 | 0.006401 | 0.019267 | Down |
| AP000936.1 | 2.724384123 | -2.636050094 | 0.452473 | -5.82587 | 5.68E-09 | 6.92E-08 | Down |
| UBE2FP1 | 29.29294843 | -1.380287164 | 0.219481 | -6.28887 | 3.20E-10 | 4.87E-09 | Down |
| AC097468.1 | 1.743775606 | 3.473696116 | 0.493777 | 7.034952 | 1.99E-12 | 4.37E-11 | Up |
| ELMO1-AS1 | 0.839040631 | -1.241698311 | 0.50822 | -2.44323 | 0.014557 | 0.038494 | Down |
| AC112715.1 | 1.041841436 | 1.591556542 | 0.503259 | 3.162503 | 0.001564 | 0.005716 | Up |
| AMMECR1-IT1 | 0.688109337 | -1.515158733 | 0.508262 | -2.98106 | 0.002873 | 0.009669 | Down |
| AC099786.2 | 1.665245902 | -1.769138148 | 0.496222 | -3.56522 | 0.000364 | 0.001592 | Down |
| AL133264.2 | 1.466717674 | 1.127692478 | 0.419601 | 2.687537 | 0.007198 | 0.021308 | Up |
| LINC01357 | 4.455363297 | 1.15827278 | 0.416474 | 2.781144 | 0.005417 | 0.016718 | Up |
| AL137802.1 | 1.835401785 | 1.29559055 | 0.463025 | 2.798098 | 0.00514 | 0.015989 | Up |
| MIR3681HG | 6.237221637 | -1.450252408 | 0.613414 | -2.36423 | 0.018068 | 0.046011 | Down |
| SNX18P9 | 2.394165374 | -1.445637235 | 0.41225 | -3.5067 | 0.000454 | 0.001939 | Down |
| LINC01991 | 8.883427875 | -2.445558906 | 0.752638 | -3.24931 | 0.001157 | 0.004388 | Down |
| HAGLR | 126.5975632 | 2.602014414 | 0.261213 | 9.961256 | 2.25E-23 | 1.92E-21 | Up |
| PHEX-AS1 | 16.0184094 | -1.672605939 | 0.369032 | -4.53241 | 5.83E-06 | 3.95E-05 | Down |
| OR2L1P | 1.216959024 | -1.38587042 | 0.484761 | -2.85887 | 0.004252 | 0.013588 | Down |
| AC073508.1 | 1.055084491 | -1.654379123 | 0.535147 | -3.09145 | 0.001992 | 0.007039 | Down |
| AL391427.1 | 3.179700798 | -1.115689805 | 0.315439 | -3.53695 | 0.000405 | 0.001751 | Down |
| LINC01133 | 14.83261363 | -1.49027755 | 0.316134 | -4.71406 | 2.43E-06 | 1.78E-05 | Down |
| RPL12P14 | 5.39916617 | 1.03399478 | 0.191964 | 5.38639 | 7.19E-08 | 7.22E-07 | Up |
| AC005019.2 | 0.403018968 | 1.986429089 | 0.785313 | 2.529473 | 0.011423 | 0.031408 | Up |
| OACYLP | 6.215897113 | 1.646437196 | 0.252127 | 6.530197 | 6.57E-11 | 1.13E-09 | Up |
| PRR29 | 913.587201 | 1.044821277 | 0.146753 | 7.119589 | 1.08E-12 | 2.50E-11 | Up |
| SMIM25 | 120.1263855 | 1.112467473 | 0.264914 | 4.199354 | 2.68E-05 | 0.000155 | Up |
| SLC25A34-AS1 | 1.717681815 | 1.071474008 | 0.399249 | 2.683724 | 0.007281 | 0.021512 | Up |
| TTC21B-AS1 | 2.347304332 | -1.567819769 | 0.384648 | -4.07598 | 4.58E-05 | 0.000253 | Down |
| HNRNPA3P14 | 1.577845995 | -1.185558174 | 0.419949 | -2.8231 | 0.004756 | 0.014943 | Down |
| LINC02523 | 0.940715325 | 2.123516503 | 0.536932 | 3.954905 | 7.66E-05 | 0.000398 | Up |
| LINC00365 | 4.299635558 | 1.4946376 | 0.292057 | 5.117619 | 3.09E-07 | 2.75E-06 | Up |
| AC109309.1 | 48.72361846 | 1.298507444 | 0.275282 | 4.717001 | 2.39E-06 | 1.76E-05 | Up |
| AC092810.1 | 0.767617979 | -2.203749228 | 0.649269 | -3.3942 | 0.000688 | 0.002791 | Down |
| AP001439.1 | 2.426813843 | 1.112788402 | 0.295678 | 3.76352 | 0.000168 | 0.000803 | Up |
| HLA-DPB2 | 76.82317246 | 1.055260333 | 0.432716 | 2.438693 | 0.01474 | 0.038897 | Up |
| AL139158.2 | 3.672645588 | 2.494508765 | 0.376383 | 6.627578 | 3.41E-11 | 6.15E-10 | Up |
| RPL29P19 | 4.333944454 | 1.284575186 | 0.246125 | 5.219193 | 1.80E-07 | 1.67E-06 | Up |
| AC093425.1 | 5.770614061 | -1.209733128 | 0.330689 | -3.65822 | 0.000254 | 0.001159 | Down |
| AC095032.1 | 2.921425234 | -1.512335197 | 0.313415 | -4.82535 | 1.40E-06 | 1.08E-05 | Down |
| PDSS1P2 | 0.790789298 | -1.554556474 | 0.487422 | -3.18935 | 0.001426 | 0.005274 | Down |
| AC026954.1 | 2.860240561 | -1.7006131 | 0.406813 | -4.18033 | 2.91E-05 | 0.000167 | Down |
| LINC00885 | 6.117958828 | -1.096306923 | 0.286022 | -3.83294 | 0.000127 | 0.000625 | Down |
| AL513164.1 | 1.315985772 | -1.78804546 | 0.500225 | -3.57449 | 0.000351 | 0.001543 | Down |
| AC010907.1 | 0.443897317 | -1.582002511 | 0.60708 | -2.60592 | 0.009163 | 0.026148 | Down |
| GAPDHP46 | 0.629465391 | 1.418742056 | 0.446234 | 3.179367 | 0.001476 | 0.005437 | Up |
| LINC02671 | 1.648084889 | -2.286789324 | 0.524524 | -4.35974 | 1.30E-05 | 8.16E-05 | Down |
| Z82186.1 | 12.14642585 | 1.352145633 | 0.301177 | 4.489537 | 7.14E-06 | 4.75E-05 | Up |
| TEX26-AS1 | 12.96177467 | 1.720529502 | 0.36344 | 4.734015 | 2.20E-06 | 1.63E-05 | Up |
| MTCYBP21 | 1.347687298 | -1.150421051 | 0.429615 | -2.6778 | 0.007411 | 0.021827 | Down |
| LINC00853 | 65.72092382 | 1.086351698 | 0.161038 | 6.745937 | 1.52E-11 | 2.90E-10 | Up |
| AC093843.1 | 7.59556283 | -1.439519057 | 0.303215 | -4.74752 | 2.06E-06 | 1.54E-05 | Down |
| RORB-AS1 | 2.480074366 | -1.168292642 | 0.364046 | -3.20919 | 0.001331 | 0.004969 | Down |
| AL161908.1 | 9.896700055 | -2.102521281 | 0.403123 | -5.21559 | 1.83E-07 | 1.70E-06 | Down |
| AC068279.1 | 0.890582275 | -1.351848543 | 0.559962 | -2.41418 | 0.015771 | 0.041102 | Down |
| EIPR1-IT1 | 74.18710594 | -1.09105219 | 0.231856 | -4.70573 | 2.53E-06 | 1.85E-05 | Down |
| APOC4-APOC2 | 3.67999556 | 1.621046212 | 0.443666 | 3.653756 | 0.000258 | 0.001178 | Up |
| AL353150.1 | 43.494695 | 1.898122501 | 0.239412 | 7.928275 | 2.22E-15 | 7.37E-14 | Up |
| KCTD10P1 | 1.272030241 | -2.180481143 | 0.503745 | -4.32854 | 1.50E-05 | 9.24E-05 | Down |
| PGM5-AS1 | 13.79784589 | -1.404907458 | 0.223607 | -6.28293 | 3.32E-10 | 5.05E-09 | Down |
| AC017002.1 | 6.144867778 | -1.811801959 | 0.416342 | -4.35171 | 1.35E-05 | 8.42E-05 | Down |
| AC009303.1 | 1.711345637 | -1.089780859 | 0.353352 | -3.08412 | 0.002042 | 0.007184 | Down |
| LINC01645 | 1.251500688 | 1.669265512 | 0.509331 | 3.277369 | 0.001048 | 0.004032 | Up |
| AL121983.1 | 4.485206148 | -1.286846776 | 0.256479 | -5.01736 | 5.24E-07 | 4.43E-06 | Down |
| AC000067.1 | 2.297137363 | 1.958985386 | 0.342472 | 5.720132 | 1.06E-08 | 1.24E-07 | Up |
| EIF1AX-AS1 | 1.271500472 | -1.378910663 | 0.450065 | -3.06381 | 0.002185 | 0.007628 | Down |
| LINC01058 | 8.549842222 | -1.14581332 | 0.223745 | -5.12106 | 3.04E-07 | 2.70E-06 | Down |
| OR7E116P | 1.706243887 | -1.26782159 | 0.398041 | -3.18515 | 0.001447 | 0.005342 | Down |
| WWC3-AS1 | 1.08999468 | -1.46598763 | 0.558392 | -2.62537 | 0.008655 | 0.024913 | Down |
| DAP3P1 | 1.151401047 | -1.154840928 | 0.435111 | -2.65413 | 0.007951 | 0.02319 | Down |
| DPY19L2P5 | 0.909293161 | -1.263815722 | 0.483722 | -2.61269 | 0.008983 | 0.025714 | Down |
| LINC00237 | 2.529916033 | -1.913585643 | 0.423657 | -4.51683 | 6.28E-06 | 4.22E-05 | Down |
| AL358216.1 | 1.955560578 | -1.051685571 | 0.431714 | -2.43607 | 0.014848 | 0.039126 | Down |
| LINC00457 | 2.023267077 | 2.863659698 | 0.539024 | 5.312675 | 1.08E-07 | 1.04E-06 | Up |
| AL139230.1 | 0.606796086 | -1.658591331 | 0.619392 | -2.67777 | 0.007411 | 0.021827 | Down |
| DUXAP9 | 3.867377036 | 1.717056752 | 0.326448 | 5.25982 | 1.44E-07 | 1.36E-06 | Up |
| AC073367.1 | 4.097176974 | -1.034034193 | 0.312146 | -3.31266 | 0.000924 | 0.003615 | Down |
| AC007362.1 | 4.139395734 | 1.365111258 | 0.325241 | 4.197233 | 2.70E-05 | 0.000157 | Up |
| HSPA7 | 213.8288967 | 1.057115554 | 0.290441 | 3.639697 | 0.000273 | 0.001237 | Up |
| RPS2P1 | 1.200889879 | -1.059702568 | 0.402846 | -2.63054 | 0.008525 | 0.024594 | Down |
| ARMC8P1 | 3.99248787 | -2.258882789 | 0.394225 | -5.72994 | 1.00E-08 | 1.17E-07 | Down |
| AL121987.1 | 1.059248663 | -1.153359412 | 0.453171 | -2.54509 | 0.010925 | 0.030264 | Down |
| LINC01770 | 47.33946144 | 2.956137143 | 0.239702 | 12.33255 | 6.05E-35 | 1.48E-32 | Up |
| LINC00113 | 5.851625044 | 1.551581112 | 0.346994 | 4.471498 | 7.77E-06 | 5.12E-05 | Up |
| LINC01594 | 2.3243007 | -1.178619066 | 0.492378 | -2.39373 | 0.016678 | 0.043075 | Down |
| AF064860.1 | 3.51178253 | -1.778280258 | 0.347371 | -5.11925 | 3.07E-07 | 2.73E-06 | Down |
| AC079630.1 | 768.818142 | 4.106412318 | 0.289145 | 14.20191 | 8.92E-46 | 7.27E-43 | Up |
| SNX18P3 | 82.09952117 | -1.83859012 | 0.17442 | -10.5412 | 5.58E-26 | 6.14E-24 | Down |
| TMEM246-AS1 | 0.675795637 | -1.880512094 | 0.560863 | -3.35289 | 0.0008 | 0.003186 | Down |
| SFTA1P | 20.57456187 | 1.211037045 | 0.323799 | 3.740089 | 0.000184 | 0.00087 | Up |
| PGM5P4 | 8.775528353 | -1.195601185 | 0.203953 | -5.86213 | 4.57E-09 | 5.66E-08 | Down |
| TGIF2P1 | 1.158561234 | -1.177386477 | 0.434461 | -2.71 | 0.006728 | 0.020104 | Down |
| AL049648.1 | 1.237634101 | -2.11637324 | 0.491539 | -4.30561 | 1.67E-05 | 0.000101 | Down |
| LINC01671 | 2.555023357 | 1.334152461 | 0.534853 | 2.494429 | 0.012616 | 0.034144 | Up |
| RAB6C-AS1 | 13.89247224 | -1.858546732 | 0.26626 | -6.9802 | 2.95E-12 | 6.33E-11 | Down |
| BX119917.1 | 43.77391473 | 1.140543275 | 0.133116 | 8.568064 | 1.05E-17 | 4.58E-16 | Up |
| AC002064.1 | 4.662449605 | -1.686425844 | 0.296831 | -5.68144 | 1.34E-08 | 1.53E-07 | Down |
| CYP4A22-AS1 | 4.068280884 | 1.90416865 | 0.261595 | 7.279065 | 3.36E-13 | 8.29E-12 | Up |
| PCDH8P1 | 3.603498151 | -1.844872287 | 0.371116 | -4.97114 | 6.66E-07 | 5.51E-06 | Down |
| LINC00475 | 24.33949197 | 2.842888652 | 0.251804 | 11.29008 | 1.47E-29 | 2.38E-27 | Up |
| MKRN2OS | 67.4438732 | 1.78333119 | 0.217074 | 8.215322 | 2.12E-16 | 7.98E-15 | Up |
| SELENOKP3 | 1.732898796 | -1.415510211 | 0.408908 | -3.46168 | 0.000537 | 0.002247 | Down |
| CCT4P2 | 1.37922032 | -1.842068002 | 0.562508 | -3.27474 | 0.001058 | 0.004064 | Down |
| AC005281.1 | 1.446313225 | -1.570026046 | 0.544194 | -2.88505 | 0.003914 | 0.012634 | Down |
| LINC02158 | 21.52277794 | -1.241044356 | 0.202425 | -6.1309 | 8.74E-10 | 1.22E-08 | Down |
| ZNF469 | 142.309649 | 1.482356172 | 0.290829 | 5.096998 | 3.45E-07 | 3.04E-06 | Up |
| AC064875.1 | 6.384576605 | 1.419807949 | 0.275245 | 5.158351 | 2.49E-07 | 2.25E-06 | Up |
| FAM66E | 2.316039819 | -1.152013423 | 0.360815 | -3.1928 | 0.001409 | 0.005219 | Down |
| AL596247.1 | 9.839111456 | -2.134650058 | 0.405599 | -5.26295 | 1.42E-07 | 1.34E-06 | Down |
| AL049637.1 | 1.173156679 | -1.248153885 | 0.464685 | -2.68602 | 0.007231 | 0.021393 | Down |
| MIAT | 408.7894781 | 1.180978306 | 0.365109 | 3.234592 | 0.001218 | 0.004592 | Up |
| WARSP1 | 30.05406682 | 6.696716234 | 0.53328 | 12.55759 | 3.61E-36 | 1.00E-33 | Up |
| AC069281.1 | 2.568324204 | -1.111521376 | 0.345647 | -3.21577 | 0.001301 | 0.004867 | Down |
| LINC00626 | 5.044658674 | -1.139637544 | 0.246898 | -4.61582 | 3.92E-06 | 2.75E-05 | Down |
| AL157400.1 | 1.203844849 | -1.516300934 | 0.429128 | -3.53344 | 0.00041 | 0.001773 | Down |
| AL162431.1 | 2.206071999 | 1.277014462 | 0.341462 | 3.739845 | 0.000184 | 0.00087 | Up |
| AL022315.1 | 1.003918687 | 1.326164458 | 0.413529 | 3.206945 | 0.001342 | 0.005001 | Up |
| AC023590.1 | 20.48772745 | -2.26358519 | 0.451828 | -5.00984 | 5.45E-07 | 4.59E-06 | Down |
| AL391244.1 | 9.245852226 | 1.689022876 | 0.264191 | 6.393194 | 1.62E-10 | 2.61E-09 | Up |
| AL138767.3 | 12.92361814 | -1.964016305 | 0.217707 | -9.02139 | 1.86E-19 | 1.04E-17 | Down |
| AL592310.1 | 2.067236421 | -1.347613762 | 0.427766 | -3.15035 | 0.001631 | 0.005918 | Down |
| LINC01875 | 1.971524162 | -2.341946157 | 0.632068 | -3.70521 | 0.000211 | 0.000983 | Down |
| AL121574.1 | 20.84493454 | -1.134879615 | 0.170733 | -6.64712 | 2.99E-11 | 5.44E-10 | Down |
| LINC02648 | 5.035553358 | -1.549556636 | 0.341309 | -4.54004 | 5.62E-06 | 3.82E-05 | Down |
| ELFN1 | 96.67299849 | 1.910311354 | 0.210045 | 9.094783 | 9.48E-20 | 5.47E-18 | Up |
| ABHD11-AS1 | 114.642425 | 1.954542341 | 0.305256 | 6.402959 | 1.52E-10 | 2.46E-09 | Up |
| UBXN10-AS1 | 91.34934107 | 1.067362326 | 0.147927 | 7.215465 | 5.37E-13 | 1.30E-11 | Up |
| LINC02660 | 4.983600764 | -2.249883324 | 0.384143 | -5.85689 | 4.72E-09 | 5.82E-08 | Down |
| PRICKLE2-AS3 | 3.492990147 | -1.335492762 | 0.311869 | -4.28223 | 1.85E-05 | 0.000111 | Down |
| FGF13-AS1 | 31.72816122 | -1.8825929 | 0.240091 | -7.84118 | 4.46E-15 | 1.42E-13 | Down |
| AC016772.1 | 1.388386924 | -1.973304326 | 0.596643 | -3.30734 | 0.000942 | 0.003673 | Down |
| UQCRFS1P1 | 19.59968236 | -1.132173446 | 0.447751 | -2.52858 | 0.011452 | 0.031472 | Down |
| LINC02097 | 7.169177989 | -1.42167062 | 0.270127 | -5.26297 | 1.42E-07 | 1.34E-06 | Down |
| FTCDNL1 | 466.6797932 | -1.288213523 | 0.125931 | -10.2295 | 1.46E-24 | 1.40E-22 | Down |
| AL356124.1 | 3.318533158 | 1.171657904 | 0.252115 | 4.647314 | 3.36E-06 | 2.40E-05 | Up |
| AC124944.1 | 4.37125677 | 1.040838416 | 0.255715 | 4.070314 | 4.69E-05 | 0.000258 | Up |
| AL049548.1 | 0.598776372 | -1.850745832 | 0.575981 | -3.2132 | 0.001313 | 0.004905 | Down |
| LINC01733 | 0.697692347 | -1.606161256 | 0.644666 | -2.49146 | 0.012722 | 0.034378 | Down |
| LEMD1-AS1 | 12.04671079 | 1.521311472 | 0.20028 | 7.595932 | 3.06E-14 | 8.63E-13 | Up |
| ISM1-AS1 | 5.71745537 | -1.191985331 | 0.319539 | -3.73032 | 0.000191 | 0.0009 | Down |
| AL031123.1 | 7.427683669 | -1.167479819 | 0.227535 | -5.13098 | 2.88E-07 | 2.57E-06 | Down |
| AL008733.1 | 4.340438397 | -1.083787517 | 0.286474 | -3.7832 | 0.000155 | 0.000749 | Down |
| CFLAR-AS1 | 77.15759528 | -1.238739718 | 0.291784 | -4.2454 | 2.18E-05 | 0.000129 | Down |
| CROCC2 | 8.255838153 | -1.130754317 | 0.279127 | -4.05104 | 5.10E-05 | 0.000278 | Down |
| AC005682.1 | 32.83371084 | -1.28123431 | 0.227714 | -5.6265 | 1.84E-08 | 2.05E-07 | Down |
| NMD3P1 | 11.474383 | -1.456037914 | 0.19575 | -7.43825 | 1.02E-13 | 2.69E-12 | Down |
| RPS6P20 | 1.14648973 | -2.038224224 | 0.550704 | -3.70113 | 0.000215 | 0.000996 | Down |
| HAGLROS | 14.82018484 | 3.46798323 | 0.364048 | 9.526168 | 1.63E-21 | 1.12E-19 | Up |
| IFNA20P | 6.629723859 | -1.006257424 | 0.228974 | -4.39463 | 1.11E-05 | 7.05E-05 | Down |
| C12orf77 | 4.917174229 | -2.071154082 | 0.750809 | -2.75856 | 0.005806 | 0.01773 | Down |
| AL392089.1 | 2.583333391 | -2.392050426 | 0.411088 | -5.81883 | 5.93E-09 | 7.20E-08 | Down |
| AC104058.1 | 0.94052884 | -1.234168716 | 0.470568 | -2.62272 | 0.008723 | 0.025079 | Down |
| USP17L7 | 1.342534149 | -1.354550038 | 0.531326 | -2.54938 | 0.010792 | 0.029956 | Down |
| LINC02542 | 4.086511977 | 1.036361909 | 0.297997 | 3.47776 | 0.000506 | 0.002132 | Up |
| LINC02549 | 0.732219759 | 3.276153095 | 0.657866 | 4.979967 | 6.36E-07 | 5.29E-06 | Up |
| LINC01918 | 12.0449368 | 2.142601088 | 0.281582 | 7.609154 | 2.76E-14 | 7.83E-13 | Up |
| AL049569.1 | 0.907674235 | 1.592925268 | 0.44894 | 3.548193 | 0.000388 | 0.001686 | Up |
| AL445487.1 | 0.618969358 | 1.40270605 | 0.566664 | 2.475377 | 0.01331 | 0.035717 | Up |
| CYP4F26P | 2.357659977 | -2.776131535 | 0.492863 | -5.63267 | 1.77E-08 | 1.98E-07 | Down |
| AC092634.3 | 10.09598231 | -1.25115972 | 0.167927 | -7.4506 | 9.29E-14 | 2.46E-12 | Down |
| AC092155.1 | 1.958674871 | -1.84480974 | 0.432327 | -4.26716 | 1.98E-05 | 0.000119 | Down |
| SHANK2-AS1 | 1.584267823 | -2.279010831 | 0.461314 | -4.94026 | 7.80E-07 | 6.38E-06 | Down |
| AC006020.1 | 2.807999211 | -1.266508979 | 0.356376 | -3.55386 | 0.00038 | 0.001654 | Down |
| AL020998.1 | 0.673162933 | 1.99456748 | 0.834344 | 2.390583 | 0.016822 | 0.043368 | Up |
| AL360181.1 | 1.441763231 | -1.252379335 | 0.429136 | -2.91837 | 0.003519 | 0.011524 | Down |
| FGF12-AS3 | 0.993767836 | 1.036999214 | 0.415853 | 2.493669 | 0.012643 | 0.034195 | Up |
| AL512599.1 | 1.44675267 | 1.589211052 | 0.437394 | 3.63336 | 0.00028 | 0.001263 | Up |
| SMCR5 | 8.656636336 | -1.091849663 | 0.239561 | -4.55772 | 5.17E-06 | 3.54E-05 | Down |
| AC007365.1 | 29.23162783 | -1.319749822 | 0.185414 | -7.11784 | 1.10E-12 | 2.52E-11 | Down |
| TAS2R46 | 1.470354699 | -1.794515095 | 0.452109 | -3.96921 | 7.21E-05 | 0.000378 | Down |
| LINC02668 | 0.986567828 | -2.606416153 | 0.50438 | -5.16757 | 2.37E-07 | 2.15E-06 | Down |
| AC000124.1 | 2.28605954 | -1.229470391 | 0.371769 | -3.30708 | 0.000943 | 0.003675 | Down |
| AC110926.1 | 1.316697621 | -1.108107146 | 0.425966 | -2.6014 | 0.009284 | 0.026437 | Down |
| MROH5 | 1.128490186 | 1.670536372 | 0.43332 | 3.855199 | 0.000116 | 0.000577 | Up |
| AL117382.1 | 1.292774548 | 1.597091365 | 0.682944 | 2.33854 | 0.019359 | 0.048736 | Up |
| AC005082.1 | 241.9945491 | -1.100754711 | 0.23124 | -4.76023 | 1.93E-06 | 1.45E-05 | Down |
| AL390036.1 | 8.038983759 | 1.630562627 | 0.416321 | 3.916597 | 8.98E-05 | 0.000459 | Up |
| AC097059.1 | 9.117106113 | -2.256355348 | 0.532925 | -4.23391 | 2.30E-05 | 0.000135 | Down |
| LINC00348 | 1.385226302 | -1.666738075 | 0.499175 | -3.33898 | 0.000841 | 0.003326 | Down |
| AC004112.1 | 10.54473818 | -1.474636085 | 0.205154 | -7.18795 | 6.58E-13 | 1.56E-11 | Down |
| CEP164P1 | 7.217377333 | -1.026933228 | 0.253234 | -4.05527 | 5.01E-05 | 0.000274 | Down |
| LINC00423 | 1.433859239 | 3.007364066 | 0.441744 | 6.807941 | 9.90E-12 | 1.95E-10 | Up |
| AL512638.1 | 2.946128428 | 3.514056046 | 0.455803 | 7.709589 | 1.26E-14 | 3.79E-13 | Up |
| AC007557.1 | 1.062610455 | -1.667518991 | 0.494069 | -3.37508 | 0.000738 | 0.002962 | Down |
| SLC8A1-AS1 | 4.815156444 | -1.452794096 | 0.33143 | -4.38341 | 1.17E-05 | 7.40E-05 | Down |
| LINC00511 | 79.05132523 | 2.752912857 | 0.263318 | 10.45472 | 1.39E-25 | 1.48E-23 | Up |
| AC009961.2 | 3.024300123 | -1.360998097 | 0.394274 | -3.45191 | 0.000557 | 0.002321 | Down |
| Z98257.1 | 5.633230557 | 1.905616872 | 0.331788 | 5.743474 | 9.28E-09 | 1.09E-07 | Up |
| AL162584.1 | 1.326156219 | 1.151119651 | 0.469793 | 2.450271 | 0.014275 | 0.037866 | Up |
| AL158166.1 | 8.067808516 | 2.742940199 | 0.31253 | 8.776572 | 1.69E-18 | 8.17E-17 | Up |
| PIGQP1 | 3.102426332 | -1.091143295 | 0.314903 | -3.46501 | 0.00053 | 0.002224 | Down |
| AC104667.1 | 26.48479487 | -1.284703054 | 0.190768 | -6.73438 | 1.65E-11 | 3.12E-10 | Down |
| AC009238.1 | 4.999487266 | -2.070511721 | 0.420865 | -4.91966 | 8.67E-07 | 7.01E-06 | Down |
| AL160286.1 | 34.76227459 | -2.533557601 | 0.274867 | -9.21738 | 3.04E-20 | 1.85E-18 | Down |
| THEM7P | 2.578536906 | 1.382824118 | 0.33164 | 4.169656 | 3.05E-05 | 0.000175 | Up |
| WDR11-AS1 | 26.21293011 | -1.079276531 | 0.141228 | -7.64209 | 2.14E-14 | 6.20E-13 | Down |
| MGAT3-AS1 | 2.460468285 | 1.677609511 | 0.392743 | 4.271523 | 1.94E-05 | 0.000116 | Up |
| MIR663AHG | 1.896298876 | -2.063791525 | 0.506017 | -4.0785 | 4.53E-05 | 0.00025 | Down |
| ST7-AS1 | 368.7780804 | -1.261954443 | 0.143309 | -8.80584 | 1.30E-18 | 6.40E-17 | Down |
| AL157935.1 | 49.21513307 | -1.360578504 | 0.208601 | -6.5224 | 6.92E-11 | 1.19E-09 | Down |
| AL133346.1 | 30.22858809 | -2.275178075 | 0.443831 | -5.12622 | 2.96E-07 | 2.63E-06 | Down |
| AC017101.1 | 1.947495636 | -1.014786527 | 0.390108 | -2.6013 | 0.009287 | 0.026443 | Down |
| TTC39DP | 0.732027449 | -1.219753924 | 0.514765 | -2.36954 | 0.01781 | 0.045464 | Down |
| AC092042.1 | 2.217102894 | -1.552831549 | 0.384711 | -4.03636 | 5.43E-05 | 0.000294 | Down |
| LINC01985 | 11.6052167 | -1.026063721 | 0.211198 | -4.8583 | 1.18E-06 | 9.29E-06 | Down |
| AC110015.1 | 0.78985919 | 1.98224333 | 0.593225 | 3.341471 | 0.000833 | 0.0033 | Up |
| AC009229.1 | 1.387702038 | 1.951935361 | 0.38143 | 5.117413 | 3.10E-07 | 2.75E-06 | Up |
| AL139280.1 | 1.064163816 | -2.530686258 | 0.501729 | -5.04393 | 4.56E-07 | 3.90E-06 | Down |
| LINC00307 | 1.373850639 | -1.031339815 | 0.411622 | -2.50555 | 0.012226 | 0.033271 | Down |
| AL133410.1 | 50.90158171 | 1.121132978 | 0.162104 | 6.916149 | 4.64E-12 | 9.63E-11 | Up |
| HPN-AS1 | 9.563209588 | 1.305846289 | 0.248794 | 5.248711 | 1.53E-07 | 1.44E-06 | Up |
| AL022238.1 | 1.679245249 | -1.305828555 | 0.38489 | -3.39273 | 0.000692 | 0.002804 | Down |
| FCF1P1 | 1.404002499 | -2.036889706 | 0.459629 | -4.43159 | 9.35E-06 | 6.06E-05 | Down |
| AC073415.1 | 3.118789354 | -1.562479135 | 0.298173 | -5.24017 | 1.60E-07 | 1.50E-06 | Down |
| TSSK5P | 63.81888548 | -1.205392332 | 0.250534 | -4.81129 | 1.50E-06 | 1.15E-05 | Down |
| AC124861.1 | 4.70743111 | -1.453397625 | 0.300332 | -4.8393 | 1.30E-06 | 1.01E-05 | Down |
| CCDC148-AS1 | 1.491405137 | 1.067189489 | 0.313425 | 3.404928 | 0.000662 | 0.002699 | Up |
| AL157702.2 | 3.16252398 | 1.22029079 | 0.302984 | 4.02757 | 5.64E-05 | 0.000304 | Up |
| NCAM1-AS1 | 13.32943497 | -1.620752427 | 0.275854 | -5.87539 | 4.22E-09 | 5.26E-08 | Down |
| AC099066.2 | 2.116884217 | 2.559448477 | 0.587176 | 4.358915 | 1.31E-05 | 8.18E-05 | Up |
| LINC01268 | 17.86208717 | 1.253342087 | 0.235796 | 5.31537 | 1.06E-07 | 1.03E-06 | Up |
| AL160163.1 | 0.879983705 | -1.75000595 | 0.478401 | -3.65803 | 0.000254 | 0.00116 | Down |
| LINC01483 | 16.69413967 | 2.318419809 | 0.230223 | 10.07034 | 7.47E-24 | 6.74E-22 | Up |
| CNTN4-AS2 | 4.240968442 | -1.429925208 | 0.269512 | -5.3056 | 1.12E-07 | 1.08E-06 | Down |
| SOX21-AS1 | 5.576741816 | -1.467545025 | 0.266664 | -5.50335 | 3.73E-08 | 3.92E-07 | Down |
| AL391097.2 | 0.933416393 | -2.374357263 | 0.71956 | -3.29973 | 0.000968 | 0.003763 | Down |
| AL359915.1 | 8.738881647 | -1.751475444 | 0.253361 | -6.91295 | 4.75E-12 | 9.83E-11 | Down |
| AC092159.1 | 1.499379484 | -1.42560548 | 0.554451 | -2.5712 | 0.010135 | 0.028418 | Down |
| AP001271.1 | 6.946674512 | -2.182409337 | 0.302959 | -7.20364 | 5.86E-13 | 1.40E-11 | Down |
| AC098826.2 | 2.165863373 | -1.202185656 | 0.466006 | -2.57977 | 0.009887 | 0.027854 | Down |
| AP000344.1 | 11.34425915 | -1.474309221 | 0.314928 | -4.68141 | 2.85E-06 | 2.07E-05 | Down |
| AC002383.1 | 7.711895727 | -2.255303193 | 0.300707 | -7.5 | 6.38E-14 | 1.73E-12 | Down |
| MRLN | 2.58005596 | -1.141921673 | 0.385707 | -2.9606 | 0.00307 | 0.010239 | Down |
| SNAP25-AS1 | 8.417704085 | 1.530223247 | 0.277764 | 5.509069 | 3.61E-08 | 3.81E-07 | Up |
| LINC02344 | 2.154757878 | -1.740049271 | 0.355057 | -4.90076 | 9.55E-07 | 7.65E-06 | Down |
| LINC01738 | 1.058545789 | 1.770752873 | 0.608427 | 2.910381 | 0.00361 | 0.011779 | Up |
| AL390778.1 | 1.554950731 | 1.929221132 | 0.522979 | 3.688907 | 0.000225 | 0.001039 | Up |
| MTND5P1 | 1.751327774 | -2.004880775 | 0.468253 | -4.28161 | 1.86E-05 | 0.000112 | Down |
| AC020743.1 | 1.006607868 | -2.241865717 | 0.888441 | -2.52337 | 0.011624 | 0.031881 | Down |
| AC069257.1 | 1.110560329 | -1.076987789 | 0.430571 | -2.5013 | 0.012374 | 0.033602 | Down |
| AC078842.1 | 31.22120683 | -2.028149095 | 0.313898 | -6.46117 | 1.04E-10 | 1.73E-09 | Down |
| LINC01736 | 8.640526507 | 1.082062611 | 0.367113 | 2.947494 | 0.003204 | 0.010621 | Up |
| LYPLAL1-DT | 20.77729544 | -1.225144193 | 0.160779 | -7.62005 | 2.54E-14 | 7.26E-13 | Down |
| LINC01740 | 3.034695776 | -2.069578023 | 0.390437 | -5.30067 | 1.15E-07 | 1.11E-06 | Down |
| AC012368.2 | 2.269509599 | 1.069351094 | 0.39211 | 2.727172 | 0.006388 | 0.019236 | Up |
| AC092436.1 | 2.268384144 | 3.003436127 | 0.418616 | 7.174683 | 7.25E-13 | 1.71E-11 | Up |
| TLE1P1 | 25.74579611 | -1.187329264 | 0.18811 | -6.31189 | 2.76E-10 | 4.25E-09 | Down |
| HNRNPA1P21 | 26.93939222 | 1.795846928 | 0.357399 | 5.024765 | 5.04E-07 | 4.28E-06 | Up |
| RNF144A-AS1 | 8.868841929 | 1.763069808 | 0.339469 | 5.193608 | 2.06E-07 | 1.90E-06 | Up |
| AL355607.1 | 49.60274863 | -1.292643344 | 0.286849 | -4.50636 | 6.59E-06 | 4.41E-05 | Down |
| LINC01320 | 1.613084317 | -1.660699532 | 0.633035 | -2.62339 | 0.008706 | 0.025039 | Down |
| AL049712.1 | 6.960415312 | -1.084014414 | 0.240459 | -4.50811 | 6.54E-06 | 4.38E-05 | Down |
| GUSBP11 | 98.86844693 | 1.060070876 | 0.181765 | 5.832105 | 5.47E-09 | 6.69E-08 | Up |
| AC008440.1 | 31.56611607 | -2.258037686 | 0.285354 | -7.91312 | 2.51E-15 | 8.26E-14 | Down |
| MIR646HG | 59.00509843 | 1.259700195 | 0.258886 | 4.865849 | 1.14E-06 | 8.99E-06 | Up |
| RPS26P4 | 0.929001881 | -1.990585971 | 0.768569 | -2.58999 | 0.009598 | 0.027173 | Down |
| AC015971.1 | 19.15749195 | -1.145666537 | 0.225229 | -5.08666 | 3.64E-07 | 3.19E-06 | Down |
| GAS2L1P2 | 3.042718096 | -1.143175274 | 0.347638 | -3.28841 | 0.001008 | 0.003898 | Down |
| LINC01185 | 4.638115496 | -1.14821561 | 0.275554 | -4.16693 | 3.09E-05 | 0.000177 | Down |
| AL357552.2 | 1.247838139 | -1.176214188 | 0.456777 | -2.57503 | 0.010023 | 0.028159 | Down |
| LINC01546 | 2.767710959 | 1.461354517 | 0.30409 | 4.805672 | 1.54E-06 | 1.18E-05 | Up |
| CSTP1 | 1.354592902 | 3.488112571 | 0.545334 | 6.39629 | 1.59E-10 | 2.56E-09 | Up |
| AL137025.1 | 24.26917118 | -1.075926471 | 0.183686 | -5.85741 | 4.70E-09 | 5.81E-08 | Down |
| LINC01013 | 13.13743141 | -1.655036801 | 0.458352 | -3.61084 | 0.000305 | 0.001366 | Down |
| MIR34AHG | 5.034245178 | 1.458474261 | 0.198069 | 7.363465 | 1.79E-13 | 4.57E-12 | Up |
| AL157413.1 | 0.539845718 | -1.62339347 | 0.639341 | -2.53917 | 0.011112 | 0.030677 | Down |
| OR7E26P | 2.003929164 | 1.183243623 | 0.47957 | 2.467301 | 0.013614 | 0.0364 | Up |
| AL033519.3 | 285.9326435 | -1.028215777 | 0.158462 | -6.48873 | 8.66E-11 | 1.46E-09 | Down |
| AC007381.1 | 7.252374321 | -1.790842663 | 0.459644 | -3.89615 | 9.77E-05 | 0.000495 | Down |
| AL121759.1 | 3.317144211 | -1.908323103 | 0.35988 | -5.30266 | 1.14E-07 | 1.10E-06 | Down |
| AC141930.1 | 115.2655293 | -1.462518373 | 0.419629 | -3.48527 | 0.000492 | 0.002079 | Down |
| ZNF883 | 62.5561125 | 1.496318596 | 0.233435 | 6.410002 | 1.46E-10 | 2.36E-09 | Up |
| ROCR | 2.158437829 | -1.144938209 | 0.433712 | -2.63986 | 0.008294 | 0.02404 | Down |
| AC004692.2 | 1.045340179 | -1.743042531 | 0.619395 | -2.81411 | 0.004891 | 0.015314 | Down |
| LINC02521 | 0.80252746 | 2.608940533 | 0.523616 | 4.982546 | 6.28E-07 | 5.23E-06 | Up |
| LINC02577 | 1.345942418 | 1.472486773 | 0.577362 | 2.550372 | 0.010761 | 0.029884 | Up |
| LINC01672 | 24.43044923 | -1.726910236 | 0.287448 | -6.00773 | 1.88E-09 | 2.51E-08 | Down |
| EIF4BP2 | 3.531685728 | -1.20499944 | 0.319744 | -3.76864 | 0.000164 | 0.000789 | Down |
| HCG22 | 134.8004114 | 2.247006752 | 0.322151 | 6.975003 | 3.06E-12 | 6.56E-11 | Up |
| THRB-AS1 | 37.15028029 | -1.065226506 | 0.144163 | -7.38903 | 1.48E-13 | 3.82E-12 | Down |
| AL034428.1 | 1.745870035 | -1.080232278 | 0.375207 | -2.87903 | 0.003989 | 0.012854 | Down |
| MIR4500HG | 6.965198496 | -2.221845207 | 0.350404 | -6.34081 | 2.29E-10 | 3.58E-09 | Down |
| AL121985.1 | 13.75537137 | 1.326549141 | 0.42546 | 3.117915 | 0.001821 | 0.006511 | Up |
| AL591806.1 | 0.926491149 | 3.038932342 | 0.448783 | 6.771495 | 1.27E-11 | 2.47E-10 | Up |
| LINC01344 | 1.501820823 | -1.620830266 | 0.538328 | -3.01086 | 0.002605 | 0.008883 | Down |
| AC097381.1 | 3.433342995 | -1.132265584 | 0.282591 | -4.00673 | 6.16E-05 | 0.000328 | Down |
| MTCO1P3 | 0.726585174 | 1.499966095 | 0.508487 | 2.949859 | 0.003179 | 0.010555 | Up |
| AC107419.1 | 5.430859062 | -1.145756023 | 0.328143 | -3.49164 | 0.00048 | 0.002037 | Down |
| AC004485.1 | 0.614621995 | -1.785022996 | 0.535282 | -3.33474 | 0.000854 | 0.003371 | Down |
| AL591623.2 | 1.523335841 | -1.123328002 | 0.367721 | -3.05484 | 0.002252 | 0.007823 | Down |
| UBTFL6 | 8.568496181 | 1.337744233 | 0.237912 | 5.622863 | 1.88E-08 | 2.09E-07 | Up |
| AC006483.1 | 1.648312644 | 1.220791772 | 0.34696 | 3.518535 | 0.000434 | 0.001863 | Up |
| AL161457.1 | 1.971371015 | -1.290174753 | 0.392413 | -3.2878 | 0.00101 | 0.003905 | Down |
| MTCO1P25 | 0.874054767 | 1.962965802 | 0.613872 | 3.197677 | 0.001385 | 0.005142 | Up |
| LINC01788 | 1.198540043 | 3.150144917 | 0.452171 | 6.966715 | 3.24E-12 | 6.93E-11 | Up |
| RPS3AP54 | 2.159428161 | -1.360269035 | 0.421543 | -3.22688 | 0.001251 | 0.004703 | Down |
| LINC01165 | 2.608817988 | -1.024956943 | 0.383948 | -2.66952 | 0.007596 | 0.022273 | Down |
| PSMA6P2 | 0.838494504 | -1.477910472 | 0.565285 | -2.61445 | 0.008937 | 0.025609 | Down |
| ANKRD20A8P | 30.46311703 | -1.581045075 | 0.301448 | -5.24483 | 1.56E-07 | 1.47E-06 | Down |
| ELOCP20 | 3.890074365 | 4.695496501 | 0.456313 | 10.29009 | 7.81E-25 | 7.67E-23 | Up |
| BTBD6P1 | 11.45131425 | -1.820209235 | 0.296035 | -6.14864 | 7.82E-10 | 1.11E-08 | Down |
| AL137026.1 | 61.99570751 | 3.312938527 | 0.325818 | 10.16807 | 2.75E-24 | 2.58E-22 | Up |
| HCG4P8 | 1.124294511 | 1.430947139 | 0.548053 | 2.610966 | 0.009029 | 0.025825 | Up |
| AC009229.2 | 3.070010251 | 2.434520922 | 0.310703 | 7.835536 | 4.67E-15 | 1.49E-13 | Up |
| LINC00200 | 0.911076439 | -2.213658929 | 0.902486 | -2.45285 | 0.014173 | 0.037641 | Down |
| LINC00242 | 27.65069278 | 1.118499033 | 0.256263 | 4.364656 | 1.27E-05 | 7.99E-05 | Up |
| MT1P3 | 5.894769956 | -1.701968598 | 0.508498 | -3.34705 | 0.000817 | 0.003244 | Down |
| LPGAT1-AS1 | 1.019178419 | -1.820890935 | 0.489358 | -3.72098 | 0.000198 | 0.000931 | Down |
| AL160411.1 | 3.15313511 | -1.450839616 | 0.312722 | -4.63939 | 3.49E-06 | 2.48E-05 | Down |
| AL662860.1 | 0.828446903 | 2.018623262 | 0.530879 | 3.80242 | 0.000143 | 0.000698 | Up |
| AC010737.1 | 0.641157985 | 2.577077653 | 1.087887 | 2.368883 | 0.017842 | 0.045526 | Up |
| AC046143.1 | 33.64108941 | 1.693068777 | 0.146877 | 11.52714 | 9.63E-31 | 1.75E-28 | Up |
| DANT1 | 0.726854471 | -1.468144583 | 0.605792 | -2.42351 | 0.015371 | 0.040273 | Down |
| AP000568.1 | 1.41114366 | -1.553650589 | 0.651607 | -2.38434 | 0.01711 | 0.043988 | Down |
| AC007563.1 | 11.68271029 | -1.112771411 | 0.221625 | -5.02096 | 5.14E-07 | 4.36E-06 | Down |
| AL359853.1 | 21.87049183 | -1.515532253 | 0.364044 | -4.16305 | 3.14E-05 | 0.000179 | Down |
| KCNQ1-AS1 | 2.492257847 | -1.205204643 | 0.3323 | -3.62686 | 0.000287 | 0.001293 | Down |
| THEMIS3P | 0.830792856 | 1.932939864 | 0.712071 | 2.714531 | 0.006637 | 0.019868 | Up |
| RPS10P18 | 2.227279408 | -1.198424172 | 0.372147 | -3.2203 | 0.001281 | 0.004799 | Down |
| AC105053.1 | 47.34825247 | -2.004875042 | 0.314934 | -6.36601 | 1.94E-10 | 3.07E-09 | Down |
| GAS2L1P1 | 1.001945566 | -2.146877677 | 0.541896 | -3.96179 | 7.44E-05 | 0.000388 | Down |
| LINC01204 | 5.206256945 | 3.006713036 | 0.436222 | 6.892623 | 5.48E-12 | 1.12E-10 | Up |
| AL356753.1 | 2.101959941 | -2.103063533 | 0.464391 | -4.52865 | 5.94E-06 | 4.01E-05 | Down |
| GOLGA2P4 | 3.519916208 | -1.953759427 | 0.421545 | -4.63476 | 3.57E-06 | 2.53E-05 | Down |
| LINC01501 | 11.63059836 | -2.170854352 | 0.723144 | -3.00197 | 0.002682 | 0.009111 | Down |
| AC073115.1 | 1.702151392 | -1.398061139 | 0.480538 | -2.90937 | 0.003622 | 0.011812 | Down |
| AC087857.1 | 2.189283981 | -1.130236754 | 0.481606 | -2.34681 | 0.018935 | 0.047831 | Down |
| MYOSLID | 5.082819861 | 1.455806338 | 0.266027 | 5.472404 | 4.44E-08 | 4.63E-07 | Up |
| BUD31P2 | 1.171658997 | -1.330345741 | 0.445357 | -2.98715 | 0.002816 | 0.009501 | Down |
| LINC01150 | 7.478033753 | 1.088670515 | 0.346665 | 3.140414 | 0.001687 | 0.006093 | Up |
| AL450322.2 | 0.775887682 | -1.958141174 | 0.703674 | -2.78274 | 0.00539 | 0.016646 | Down |
| AC018644.1 | 3.255271081 | -1.014266695 | 0.307712 | -3.29616 | 0.00098 | 0.003806 | Down |
| LYPD9P | 1.32174702 | -2.409655284 | 0.958393 | -2.51427 | 0.011928 | 0.032585 | Down |
| EEF1DP3 | 18.90451957 | -1.362460542 | 0.225739 | -6.03555 | 1.58E-09 | 2.13E-08 | Down |
| FTLP16 | 3.963073131 | -1.355530256 | 0.369143 | -3.6721 | 0.000241 | 0.001103 | Down |
| AL021396.1 | 1.153957256 | -1.140639804 | 0.427204 | -2.67001 | 0.007585 | 0.022242 | Down |
| TRBV10-2 | 5.342962109 | -1.528818475 | 0.512545 | -2.9828 | 0.002856 | 0.009622 | Down |
| AC013444.1 | 5.871590212 | -2.413929753 | 0.673329 | -3.58507 | 0.000337 | 0.001489 | Down |
| AL671883.1 | 5.665420004 | 1.531034215 | 0.325221 | 4.707666 | 2.51E-06 | 1.83E-05 | Up |
| PGA3 | 2.406248501 | -2.212877653 | 0.496216 | -4.4595 | 8.22E-06 | 5.39E-05 | Down |
| CASC20 | 0.719262147 | -1.907516756 | 0.556143 | -3.4299 | 0.000604 | 0.002496 | Down |
| AC084290.1 | 0.454209122 | 1.558738035 | 0.587306 | 2.654049 | 0.007953 | 0.023192 | Up |
| HSPD1P21 | 1.899260768 | -2.778466352 | 0.551235 | -5.04044 | 4.64E-07 | 3.96E-06 | Down |
| AC128709.1 | 3.474520407 | -1.382929982 | 0.329894 | -4.19204 | 2.76E-05 | 0.00016 | Down |
| B3GALNT1P1 | 1.622241241 | -1.939576774 | 0.470775 | -4.11996 | 3.79E-05 | 0.000212 | Down |
| AL590666.2 | 4.533422985 | 1.422271076 | 0.392759 | 3.621232 | 0.000293 | 0.001317 | Up |
| Z98749.1 | 1.190968517 | -1.223900291 | 0.423035 | -2.89314 | 0.003814 | 0.012359 | Down |
| AC243428.1 | 1.201004522 | -1.76221974 | 0.498134 | -3.53764 | 0.000404 | 0.001746 | Down |
| AL022238.2 | 1.68257855 | -1.032694058 | 0.372488 | -2.77242 | 0.005564 | 0.017088 | Down |
| PPIAP39 | 112.1846584 | -1.340127047 | 0.271296 | -4.93973 | 7.82E-07 | 6.39E-06 | Down |
| TRPM2-AS | 8.211883809 | 1.130368391 | 0.246862 | 4.578943 | 4.67E-06 | 3.23E-05 | Up |
| RPL10P6 | 199.1290477 | -1.819658854 | 0.503364 | -3.61499 | 0.0003 | 0.001347 | Down |
| TCERG1L-AS1 | 14.34269119 | 2.205442542 | 0.338359 | 6.51806 | 7.12E-11 | 1.22E-09 | Up |
| TRBV5-4 | 37.13898284 | 1.103547909 | 0.383472 | 2.877778 | 0.004005 | 0.012894 | Up |
| LINC02028 | 155.3628671 | -1.069072122 | 0.204075 | -5.23862 | 1.62E-07 | 1.51E-06 | Down |
| AL022316.1 | 9.518387176 | 1.83162241 | 0.384863 | 4.759149 | 1.94E-06 | 1.46E-05 | Up |
| AC138207.1 | 1.238518607 | 1.091823482 | 0.406969 | 2.682815 | 0.007301 | 0.021565 | Up |
| SMYD3-IT1 | 0.79264879 | -1.25329059 | 0.514824 | -2.4344 | 0.014916 | 0.039268 | Down |
| LINC02603 | 10.32665799 | 1.115466621 | 0.200569 | 5.561509 | 2.67E-08 | 2.89E-07 | Up |
| LINC02370 | 3.743819201 | -1.653457823 | 0.582332 | -2.83938 | 0.00452 | 0.014321 | Down |
| MTCO2P27 | 0.703966952 | -1.174057214 | 0.486159 | -2.41496 | 0.015737 | 0.041031 | Down |
| KARSP2 | 2.727583682 | -1.371666682 | 0.322284 | -4.25609 | 2.08E-05 | 0.000124 | Down |
| LINC01747 | 9.756078734 | 3.222577438 | 0.359222 | 8.970999 | 2.94E-19 | 1.58E-17 | Up |
| LINC00856 | 4.242392296 | 1.756663822 | 0.399637 | 4.395652 | 1.10E-05 | 7.02E-05 | Up |
| ANKRD18B | 9.612840246 | -1.433807279 | 0.247129 | -5.80186 | 6.56E-09 | 7.89E-08 | Down |
| PROX1-AS1 | 137.2136839 | -1.110783323 | 0.239135 | -4.64501 | 3.40E-06 | 2.42E-05 | Down |
| AP000695.1 | 4.348056379 | 2.167086274 | 0.29576 | 7.327168 | 2.35E-13 | 5.93E-12 | Up |
| ATP5MC2P3 | 1.060518815 | -1.306296027 | 0.511537 | -2.55367 | 0.010659 | 0.02966 | Down |
| AL139383.1 | 8.703515958 | -1.06353868 | 0.264033 | -4.02805 | 5.62E-05 | 0.000303 | Down |
| AL132765.1 | 5.16816947 | -1.867470417 | 0.324342 | -5.75773 | 8.53E-09 | 1.01E-07 | Down |
| AL035409.1 | 2.949049988 | 3.995039039 | 0.521567 | 7.659687 | 1.86E-14 | 5.46E-13 | Up |
| MKX-AS1 | 1.494371978 | 2.723148969 | 0.616076 | 4.420152 | 9.86E-06 | 6.34E-05 | Up |
| AL354824.1 | 0.445153675 | 2.659834017 | 0.959827 | 2.771161 | 0.005586 | 0.017142 | Up |
| HMGB1P49 | 2.189693055 | -1.439236487 | 0.434487 | -3.3125 | 0.000925 | 0.003616 | Down |
| COL6A4P1 | 3.614238514 | -1.300257034 | 0.430407 | -3.021 | 0.002519 | 0.008631 | Down |
| AL392046.1 | 6.878613185 | -1.325860355 | 0.235986 | -5.61839 | 1.93E-08 | 2.14E-07 | Down |
| AL360268.1 | 2.32642554 | -2.509306417 | 0.453299 | -5.53565 | 3.10E-08 | 3.31E-07 | Down |
| LINC02580 | 34.22746832 | -1.83373713 | 0.202148 | -9.07124 | 1.18E-19 | 6.70E-18 | Down |
| GPAA1P2 | 3.545217165 | 2.005157293 | 0.373107 | 5.374219 | 7.69E-08 | 7.66E-07 | Up |
| AC104461.1 | 4.504388522 | -1.282264984 | 0.328392 | -3.90468 | 9.44E-05 | 0.00048 | Down |
| AL160408.3 | 2.28801335 | 1.265323081 | 0.439719 | 2.877573 | 0.004007 | 0.0129 | Up |
| KLF2P2 | 8.282347625 | -1.241887093 | 0.235202 | -5.28008 | 1.29E-07 | 1.23E-06 | Down |
| AC140479.2 | 11.6535872 | 1.177081428 | 0.255477 | 4.607389 | 4.08E-06 | 2.85E-05 | Up |
| AC012462.3 | 1.294631629 | 3.624644173 | 0.526291 | 6.887151 | 5.69E-12 | 1.16E-10 | Up |
| AL645608.2 | 4.977002454 | 1.669489225 | 0.286967 | 5.81771 | 5.97E-09 | 7.24E-08 | Up |
| AL589987.1 | 5.906830484 | -1.041630426 | 0.2337 | -4.45713 | 8.31E-06 | 5.44E-05 | Down |
| AC104024.1 | 6.124844345 | -1.510609861 | 0.610829 | -2.47305 | 0.013397 | 0.035917 | Down |
| AL590093.1 | 3.108082602 | -1.602064342 | 0.306144 | -5.23304 | 1.67E-07 | 1.56E-06 | Down |
| AL157714.1 | 2.94096186 | 4.950472422 | 0.72278 | 6.849215 | 7.43E-12 | 1.49E-10 | Up |
| MEIS1-AS2 | 1.292765514 | -1.687982259 | 0.470205 | -3.58988 | 0.000331 | 0.001464 | Down |
| SDAD1P2 | 1.337117239 | -1.270198601 | 0.445494 | -2.85122 | 0.004355 | 0.01387 | Down |
| AC007036.2 | 2.705588191 | -1.129958732 | 0.331146 | -3.41227 | 0.000644 | 0.002638 | Down |
| FOXD3-AS1 | 2.188437442 | -1.734045567 | 0.427695 | -4.0544 | 5.03E-05 | 0.000275 | Down |
| AL132709.1 | 2.213496766 | 4.343230077 | 0.622368 | 6.978562 | 2.98E-12 | 6.40E-11 | Up |
| LINC01362 | 2.49158835 | -1.389415443 | 0.340972 | -4.07486 | 4.60E-05 | 0.000254 | Down |
| LINC01293 | 4.326330092 | 1.825066345 | 0.525695 | 3.471718 | 0.000517 | 0.002173 | Up |
| LINC01614 | 12.2436544 | 4.037445394 | 0.480463 | 8.403233 | 4.34E-17 | 1.77E-15 | Up |
| AL445933.2 | 1.064404729 | -1.640327777 | 0.474632 | -3.456 | 0.000548 | 0.00229 | Down |
| MAGEA8-AS1 | 1.362908696 | -1.061575722 | 0.393317 | -2.69903 | 0.006954 | 0.020689 | Down |
| AL391807.1 | 49.17429715 | 3.120241687 | 0.280842 | 11.11031 | 1.12E-28 | 1.64E-26 | Up |
| AC008063.1 | 13.333272 | 4.629119237 | 0.409128 | 11.31459 | 1.11E-29 | 1.83E-27 | Up |
| AL079301.1 | 3.245217016 | -1.39203407 | 0.360926 | -3.85684 | 0.000115 | 0.000573 | Down |
| MIR205HG | 30.31105952 | 1.324403091 | 0.403816 | 3.27972 | 0.001039 | 0.004003 | Up |
| AP000356.1 | 2.38035412 | -1.059847454 | 0.335308 | -3.16081 | 0.001573 | 0.005743 | Down |
| SNX18P13 | 2.429641481 | -1.341271472 | 0.38067 | -3.52345 | 0.000426 | 0.001833 | Down |
| AC005703.1 | 1.738959557 | -1.891058388 | 0.437658 | -4.32086 | 1.55E-05 | 9.53E-05 | Down |
| AC013275.1 | 36.88896964 | 1.379252453 | 0.304734 | 4.526081 | 6.01E-06 | 4.06E-05 | Up |
| LINC01804 | 2.031138701 | -1.228620456 | 0.428277 | -2.86875 | 0.004121 | 0.013221 | Down |
| RPL7L1P9 | 0.724843777 | -1.399002288 | 0.504415 | -2.77351 | 0.005545 | 0.017046 | Down |
| AL035588.1 | 1.476754142 | -1.185985532 | 0.406054 | -2.92075 | 0.003492 | 0.011454 | Down |
| LINC01436 | 4.38542734 | 1.802665378 | 0.537939 | 3.351059 | 0.000805 | 0.003205 | Up |
| TCF7L1-IT1 | 1.739550486 | -1.390269408 | 0.387604 | -3.58683 | 0.000335 | 0.00148 | Down |
| AC002451.1 | 47.2132057 | -1.090534167 | 0.177822 | -6.13274 | 8.64E-10 | 1.21E-08 | Down |
| LINC01098 | 0.575110659 | 1.233879229 | 0.519214 | 2.376435 | 0.017481 | 0.044764 | Up |
| AC007099.1 | 0.765007746 | 2.26087299 | 0.514298 | 4.396039 | 1.10E-05 | 7.01E-05 | Up |
| AL954705.1 | 12.48047146 | -1.075652918 | 0.207843 | -5.17533 | 2.28E-07 | 2.08E-06 | Down |
| ZNF826P | 1166.714269 | -1.472303855 | 0.175461 | -8.39107 | 4.82E-17 | 1.96E-15 | Down |
| LINC01510 | 6.731442711 | 3.696795245 | 0.421548 | 8.769568 | 1.79E-18 | 8.66E-17 | Up |
| LINC01593 | 0.859676765 | -2.140222594 | 0.875934 | -2.44336 | 0.014551 | 0.038482 | Down |
| KLF2P1 | 4.007953126 | -1.641917292 | 0.411577 | -3.98933 | 6.63E-05 | 0.00035 | Down |
| C1DP1 | 28.45303131 | -1.573502313 | 0.571208 | -2.75469 | 0.005875 | 0.017902 | Down |
| AL445426.1 | 8.789301617 | -1.264400713 | 0.262717 | -4.81278 | 1.49E-06 | 1.14E-05 | Down |
| ITPR1-DT | 70.10879456 | -1.112815493 | 0.20408 | -5.45285 | 4.96E-08 | 5.13E-07 | Down |
| AC099792.1 | 5.966612469 | -1.581843124 | 0.29669 | -5.33164 | 9.73E-08 | 9.51E-07 | Down |
| PCED1CP | 1.059277157 | 1.654848674 | 0.467522 | 3.539618 | 0.000401 | 0.001734 | Up |
| MANCR | 1.006404789 | 2.067370961 | 0.504117 | 4.100973 | 4.11E-05 | 0.000229 | Up |
| AC112484.1 | 30.03841311 | -1.195025085 | 0.226227 | -5.28241 | 1.27E-07 | 1.22E-06 | Down |
| AL031772.1 | 6.274564548 | -1.000026133 | 0.277425 | -3.60467 | 0.000313 | 0.001394 | Down |
| GNA14-AS1 | 2.749344815 | -1.215370266 | 0.413234 | -2.94112 | 0.00327 | 0.010825 | Down |
| RNF2P1 | 6.723513128 | -1.044897281 | 0.342729 | -3.04876 | 0.002298 | 0.007958 | Down |
| AC005392.2 | 1.35275737 | 1.86444606 | 0.499452 | 3.732986 | 0.000189 | 0.000891 | Up |
| AC011840.1 | 0.866235156 | -2.131257105 | 0.915391 | -2.32825 | 0.019899 | 0.049926 | Down |
| FILNC1 | 7.214916481 | -1.696910683 | 0.387132 | -4.38329 | 1.17E-05 | 7.40E-05 | Down |
| LINC01750 | 6.015734809 | -1.194425417 | 0.25953 | -4.60226 | 4.18E-06 | 2.92E-05 | Down |
| AL512422.1 | 6.760051381 | 1.940099033 | 0.383572 | 5.057981 | 4.24E-07 | 3.65E-06 | Up |
| TIMM8AP1 | 35.41329321 | 4.75472612 | 0.361416 | 13.15583 | 1.58E-39 | 6.57E-37 | Up |
| AL022314.1 | 1.610642406 | 4.164906325 | 0.846069 | 4.922657 | 8.54E-07 | 6.91E-06 | Up |
| AL162253.1 | 1.165527069 | -1.180973819 | 0.45758 | -2.58091 | 0.009854 | 0.027777 | Down |
| AL162385.2 | 9.131349232 | -1.064476142 | 0.219 | -4.86063 | 1.17E-06 | 9.19E-06 | Down |
| PTCHD3P2 | 14.58436184 | 1.749678444 | 0.35349 | 4.949722 | 7.43E-07 | 6.10E-06 | Up |
| IGBP1P3 | 1.978220337 | 3.56404796 | 0.638373 | 5.583016 | 2.36E-08 | 2.58E-07 | Up |
| AC104462.1 | 2.051366524 | -1.356073684 | 0.329865 | -4.11099 | 3.94E-05 | 0.00022 | Down |
| AC013264.1 | 6.783777985 | -1.084532756 | 0.447831 | -2.42175 | 0.015446 | 0.040414 | Down |
| AL590428.1 | 7.387638406 | 1.289927262 | 0.257904 | 5.001575 | 5.69E-07 | 4.77E-06 | Up |
| RPS6KA2-AS1 | 0.631973123 | -1.712683241 | 0.600379 | -2.85267 | 0.004335 | 0.013817 | Down |
| AC011742.2 | 2.340157128 | -1.65661239 | 0.365372 | -4.53404 | 5.79E-06 | 3.92E-05 | Down |
| LINC01704 | 4.649840072 | 1.775526084 | 0.276837 | 6.413624 | 1.42E-10 | 2.31E-09 | Up |
| RPL21P43 | 0.610322593 | 2.013132228 | 0.591969 | 3.400737 | 0.000672 | 0.002734 | Up |
| LINC01090 | 0.962614533 | -1.684873871 | 0.492999 | -3.4176 | 0.000632 | 0.002594 | Down |
| LINC00574 | 9.889897016 | 1.138061695 | 0.292662 | 3.888655 | 0.000101 | 0.00051 | Up |
| AF064860.2 | 11.11902222 | -1.190464571 | 0.282123 | -4.21966 | 2.45E-05 | 0.000143 | Down |
| COX6CP2 | 0.633327407 | 1.528464768 | 0.489095 | 3.125089 | 0.001778 | 0.006377 | Up |
| AL353747.3 | 1.739739496 | -1.868613592 | 0.61275 | -3.04955 | 0.002292 | 0.00794 | Down |
| TSPAN19 | 35.16883283 | -2.321838784 | 0.281066 | -8.26082 | 1.45E-16 | 5.59E-15 | Down |
| EMBP1 | 100.450238 | 1.184096521 | 0.306028 | 3.869247 | 0.000109 | 0.000548 | Up |
| LINC01354 | 99.18462819 | -1.529502571 | 0.259924 | -5.88442 | 3.99E-09 | 5.00E-08 | Down |
| AC073342.1 | 4.033923001 | -1.744652082 | 0.295608 | -5.90191 | 3.59E-09 | 4.54E-08 | Down |
| AC099677.2 | 0.822702221 | -1.97490081 | 0.519645 | -3.80048 | 0.000144 | 0.000703 | Down |
| AL031848.1 | 0.559034243 | 1.632267985 | 0.559656 | 2.916554 | 0.003539 | 0.011579 | Up |
| AC099560.1 | 5.224722845 | 3.572843702 | 0.537705 | 6.644622 | 3.04E-11 | 5.52E-10 | Up |
| AL359551.1 | 2.983420721 | 1.511768304 | 0.471558 | 3.205901 | 0.001346 | 0.005017 | Up |
| F10-AS1 | 1.402684926 | -1.394302097 | 0.427497 | -3.26155 | 0.001108 | 0.004228 | Down |
| PSG1 | 29.82717281 | 5.068638749 | 0.554454 | 9.141673 | 6.15E-20 | 3.62E-18 | Up |
| AL731567.1 | 11.12304335 | 1.007251701 | 0.408138 | 2.467918 | 0.01359 | 0.036356 | Up |
| LINC01807 | 1.227402789 | -2.19234662 | 0.508939 | -4.30768 | 1.65E-05 | 0.000101 | Down |
| SILC1 | 2.432305147 | -2.060217899 | 0.464679 | -4.43364 | 9.27E-06 | 6.01E-05 | Down |
| TMEM253 | 59.82450179 | -1.624677991 | 0.242166 | -6.70895 | 1.96E-11 | 3.67E-10 | Down |
| LINC01031 | 1.091332681 | -1.300994421 | 0.450231 | -2.88962 | 0.003857 | 0.012482 | Down |
| LINC01697 | 7.95517342 | -2.701289031 | 0.336723 | -8.02228 | 1.04E-15 | 3.58E-14 | Down |
| DCST1-AS1 | 52.077206 | 1.180107575 | 0.117092 | 10.07843 | 6.88E-24 | 6.24E-22 | Up |
| AC073488.1 | 1.372618961 | -2.69283018 | 0.778936 | -3.45706 | 0.000546 | 0.002282 | Down |
| IMPDH1P10 | 3.915497307 | -1.037610055 | 0.395613 | -2.62279 | 0.008721 | 0.025075 | Down |
| CNOT7P2 | 0.828585523 | 2.890207181 | 0.50468 | 5.726811 | 1.02E-08 | 1.19E-07 | Up |
| Z99943.1 | 3.417474316 | 1.424421236 | 0.301989 | 4.716794 | 2.40E-06 | 1.76E-05 | Up |
| IGHV3-43 | 277.9025201 | 2.19141261 | 0.716069 | 3.060336 | 0.002211 | 0.007701 | Up |
| AC092431.2 | 2.408751579 | -1.18319387 | 0.373626 | -3.16679 | 0.001541 | 0.005646 | Down |
| DYNLT3P1 | 1.942532055 | 2.533034664 | 0.547543 | 4.626187 | 3.72E-06 | 2.63E-05 | Up |
| AL158166.2 | 3.718595462 | 2.583818994 | 0.335397 | 7.70377 | 1.32E-14 | 3.95E-13 | Up |
| AC239803.1 | 1.180204126 | 1.841308799 | 0.578958 | 3.180386 | 0.001471 | 0.00542 | Up |
| HSD17B3-AS1 | 5.710037744 | -1.65175929 | 0.289965 | -5.69641 | 1.22E-08 | 1.41E-07 | Down |
| SLC6A1-AS1 | 0.961389692 | 1.758445762 | 0.423404 | 4.15312 | 3.28E-05 | 0.000187 | Up |
| AL078590.3 | 10.42161316 | 1.769430583 | 0.221575 | 7.985708 | 1.40E-15 | 4.74E-14 | Up |
| AC084030.1 | 3.211983976 | -1.38451651 | 0.382241 | -3.6221 | 0.000292 | 0.001314 | Down |
| AF241726.1 | 7.635614467 | -1.609793539 | 0.36606 | -4.39762 | 1.09E-05 | 6.96E-05 | Down |
| GPR79 | 0.946827576 | -1.295754053 | 0.472992 | -2.73948 | 0.006154 | 0.018635 | Down |
| AC015712.1 | 17.72533214 | 1.574296698 | 0.216047 | 7.286841 | 3.17E-13 | 7.84E-12 | Up |
| RAD17P1 | 5.079955327 | -2.434748352 | 0.475581 | -5.11953 | 3.06E-07 | 2.72E-06 | Down |
| GXYLT1P3 | 0.720134328 | -1.776463765 | 0.499983 | -3.55305 | 0.000381 | 0.001659 | Down |
| AL354824.2 | 0.396564621 | 2.149417808 | 0.68901 | 3.119574 | 0.001811 | 0.006481 | Up |
| BMPR1AP2 | 2.326941654 | -1.000715285 | 0.320972 | -3.11776 | 0.001822 | 0.006512 | Down |
| TMA16P2 | 1.107660561 | -1.128611265 | 0.471458 | -2.39387 | 0.016671 | 0.04307 | Down |
| AL592437.2 | 1.473632791 | -1.190077638 | 0.399319 | -2.98027 | 0.00288 | 0.00969 | Down |
| AC073464.1 | 22.79839473 | -1.132164606 | 0.301896 | -3.75018 | 0.000177 | 0.00084 | Down |
| AC112198.2 | 11.81761795 | -1.683341006 | 0.207899 | -8.0969 | 5.64E-16 | 2.01E-14 | Down |
| AC012507.2 | 0.884334942 | 1.847511793 | 0.634624 | 2.911193 | 0.003601 | 0.01175 | Up |
| AL365436.2 | 5.065444226 | -1.119201707 | 0.297287 | -3.76471 | 0.000167 | 0.0008 | Down |
| AC253536.3 | 36.87915225 | -1.425400588 | 0.307733 | -4.63193 | 3.62E-06 | 2.57E-05 | Down |
| KANTR | 79.69592084 | -1.291165048 | 0.250441 | -5.15557 | 2.53E-07 | 2.28E-06 | Down |
| AL008707.1 | 13.96028869 | 3.63041286 | 0.326583 | 11.11635 | 1.04E-28 | 1.54E-26 | Up |
| HLA-DQB2 | 1825.835106 | 1.319704383 | 0.432431 | 3.051828 | 0.002275 | 0.007888 | Up |
| AC079465.1 | 3.650681037 | 1.437803042 | 0.316252 | 4.546388 | 5.46E-06 | 3.71E-05 | Up |
| LINC01780 | 5.159437089 | -1.771665117 | 0.293513 | -6.03608 | 1.58E-09 | 2.13E-08 | Down |
| LINC01656 | 0.669459156 | -1.419403959 | 0.581548 | -2.44074 | 0.014657 | 0.038725 | Down |
| AC004862.1 | 1.405359792 | -1.73941451 | 0.433777 | -4.00993 | 6.07E-05 | 0.000324 | Down |
| AL121830.1 | 0.388919553 | -1.605699692 | 0.64346 | -2.49541 | 0.012581 | 0.034067 | Down |
| LINC01705 | 3.953891976 | 3.885225799 | 0.6726 | 5.776429 | 7.63E-09 | 9.08E-08 | Up |
| AC002511.1 | 21.30528816 | -1.698118675 | 0.292569 | -5.80417 | 6.47E-09 | 7.79E-08 | Down |
| KIZ-AS1 | 13.63566303 | -1.05190741 | 0.214038 | -4.91457 | 8.90E-07 | 7.17E-06 | Down |
| AC007272.1 | 3.506928002 | -1.033714111 | 0.299919 | -3.44664 | 0.000568 | 0.00236 | Down |
| TRIM80P | 4.016545237 | -1.331087389 | 0.273222 | -4.87182 | 1.11E-06 | 8.75E-06 | Down |
| AC097717.1 | 4.377142634 | -1.180175585 | 0.320663 | -3.68043 | 0.000233 | 0.001071 | Down |
| AL139132.1 | 1.39762673 | -1.398766534 | 0.438993 | -3.18631 | 0.001441 | 0.005323 | Down |
| AL355483.3 | 5.376449637 | -2.242365461 | 0.417713 | -5.36819 | 7.95E-08 | 7.90E-07 | Down |
| AC016825.1 | 1.399721203 | -1.310392419 | 0.479997 | -2.73 | 0.006333 | 0.019098 | Down |
| GAPDHP74 | 0.542507951 | 1.614090941 | 0.520786 | 3.099335 | 0.00194 | 0.006878 | Up |
| AC003986.2 | 1.123916189 | 1.308227179 | 0.478021 | 2.736755 | 0.006205 | 0.018759 | Up |
| AC107057.1 | 9.960911807 | 2.229126873 | 0.324687 | 6.865462 | 6.63E-12 | 1.34E-10 | Up |
| SMG7-AS1 | 62.09598919 | -1.005036983 | 0.163173 | -6.15934 | 7.30E-10 | 1.04E-08 | Down |
| AL138787.1 | 21.18251908 | -1.389715884 | 0.241191 | -5.76188 | 8.32E-09 | 9.85E-08 | Down |
| CTAGE3P | 3.815207257 | -1.175114178 | 0.277282 | -4.23798 | 2.26E-05 | 0.000133 | Down |
| AF127936.2 | 6.632182344 | 1.44386487 | 0.542864 | 2.659716 | 0.007821 | 0.022862 | Up |
| AL499616.1 | 1.498143179 | 1.613107869 | 0.363464 | 4.438153 | 9.07E-06 | 5.90E-05 | Up |
| AL157823.2 | 7.038787294 | -1.531229986 | 0.319232 | -4.7966 | 1.61E-06 | 1.23E-05 | Down |
| AL158829.1 | 7.928961946 | -1.762249367 | 0.407518 | -4.32435 | 1.53E-05 | 9.40E-05 | Down |
| AP001062.2 | 3.036925631 | -1.008182589 | 0.3015 | -3.34388 | 0.000826 | 0.003275 | Down |
| CYP1B1-AS1 | 58.29332464 | 1.911189231 | 0.184868 | 10.33811 | 4.74E-25 | 4.77E-23 | Up |
| LINC00327 | 4.181764691 | 1.003128389 | 0.280364 | 3.577955 | 0.000346 | 0.001526 | Up |
| AC006970.2 | 25.76371943 | -1.79021564 | 0.302559 | -5.91691 | 3.28E-09 | 4.19E-08 | Down |
| AC243772.2 | 6.637928852 | 1.158609659 | 0.293279 | 3.950539 | 7.80E-05 | 0.000405 | Up |
| LINC01722 | 3.028709873 | -1.680476659 | 0.34884 | -4.81733 | 1.45E-06 | 1.12E-05 | Down |
| AL353621.1 | 0.888570117 | -1.559035496 | 0.469134 | -3.32322 | 0.00089 | 0.003495 | Down |
| TTLL7-IT1 | 1.109921317 | -1.616053628 | 0.510502 | -3.16562 | 0.001548 | 0.005668 | Down |
| PTCHD1-AS | 5.714775939 | -1.768567418 | 0.394934 | -4.47813 | 7.53E-06 | 4.98E-05 | Down |
| LINC01399 | 1.308396621 | -1.440223876 | 0.490113 | -2.93855 | 0.003297 | 0.010905 | Down |
| AC006042.2 | 14.866136 | -1.347280826 | 0.208184 | -6.4716 | 9.70E-11 | 1.62E-09 | Down |
| AC093797.1 | 152.3756982 | -1.599050778 | 0.25969 | -6.15753 | 7.39E-10 | 1.05E-08 | Down |
| ACTBP12 | 5.760223765 | -1.263028916 | 0.494919 | -2.55199 | 0.010711 | 0.029774 | Down |
| DIRC3-AS1 | 3.259530279 | -1.408046609 | 0.300241 | -4.68972 | 2.74E-06 | 1.99E-05 | Down |
| BPIFB5P | 3.965369228 | 3.998940572 | 0.520167 | 7.687801 | 1.50E-14 | 4.45E-13 | Up |
| RNF224 | 61.64417484 | 1.167244162 | 0.185009 | 6.309135 | 2.81E-10 | 4.32E-09 | Up |
| AC002511.2 | 15.57048958 | -1.143578742 | 0.310701 | -3.68064 | 0.000233 | 0.00107 | Down |
| LINC00167 | 10.84335974 | -1.012539265 | 0.191705 | -5.28177 | 1.28E-07 | 1.22E-06 | Down |
| AC133785.1 | 45.28447194 | -1.029062764 | 0.171667 | -5.99453 | 2.04E-09 | 2.71E-08 | Down |
| AC007743.1 | 342.1543297 | -1.504656874 | 0.173695 | -8.66266 | 4.61E-18 | 2.12E-16 | Down |
| AL355870.1 | 2.114482601 | -1.888000417 | 0.404474 | -4.66779 | 3.04E-06 | 2.20E-05 | Down |
| AC006042.3 | 42.87150475 | -1.470136217 | 0.260176 | -5.65055 | 1.60E-08 | 1.80E-07 | Down |
| AC009238.2 | 4.887552612 | -1.780269329 | 0.346024 | -5.14493 | 2.68E-07 | 2.40E-06 | Down |
| MTND3P10 | 0.59985789 | -1.338667588 | 0.567089 | -2.36059 | 0.018246 | 0.04637 | Down |
| AL513533.1 | 0.539318426 | 1.600016032 | 0.580354 | 2.756966 | 0.005834 | 0.017802 | Up |
| AL133343.2 | 1.648864459 | 3.713195738 | 0.594096 | 6.250161 | 4.10E-10 | 6.11E-09 | Up |
| AL122008.3 | 3.053477294 | 1.173079961 | 0.324607 | 3.613845 | 0.000302 | 0.001352 | Up |
| AL356124.2 | 2.071860002 | 1.495990612 | 0.308815 | 4.844294 | 1.27E-06 | 9.89E-06 | Up |
| CHRM3-AS2 | 50.92285455 | -1.62531398 | 0.495647 | -3.27917 | 0.001041 | 0.004009 | Down |
| AC006019.2 | 2.364627012 | -2.914554164 | 0.46459 | -6.2734 | 3.53E-10 | 5.34E-09 | Down |
| AC096537.1 | 2.005323144 | 1.477808048 | 0.409001 | 3.613216 | 0.000302 | 0.001355 | Up |
| AC239798.1 | 0.679332295 | 1.245287278 | 0.474998 | 2.621667 | 0.00875 | 0.025149 | Up |
| LINC02596 | 6.72760222 | -2.43994729 | 0.325529 | -7.49532 | 6.61E-14 | 1.79E-12 | Down |
| EEF1A1P6 | 1113.511789 | 1.061993214 | 0.169296 | 6.273 | 3.54E-10 | 5.35E-09 | Up |
| LINC00460 | 3.026687921 | 3.217306029 | 0.525876 | 6.11799 | 9.48E-10 | 1.32E-08 | Up |
| AC017104.3 | 1.043915251 | -1.236525632 | 0.455665 | -2.71367 | 0.006654 | 0.019915 | Down |
| AC011294.1 | 0.73369956 | 1.381681599 | 0.541137 | 2.553294 | 0.010671 | 0.029686 | Up |
| AL391845.2 | 14.01967979 | 3.475037187 | 0.305109 | 11.38949 | 4.72E-30 | 8.05E-28 | Up |
| LINC02609 | 182.4817389 | -1.010426162 | 0.15745 | -6.41742 | 1.39E-10 | 2.26E-09 | Down |
| LINC01137 | 282.5766724 | 1.002392842 | 0.141293 | 7.094441 | 1.30E-12 | 2.95E-11 | Up |
| LINC02625 | 3.335478958 | -1.704355258 | 0.316472 | -5.38549 | 7.22E-08 | 7.24E-07 | Down |
| AL445430.1 | 1.107906157 | -1.74003577 | 0.531372 | -3.27461 | 0.001058 | 0.004065 | Down |
| ANAPC1P1 | 4.678309921 | 1.152270227 | 0.391487 | 2.943318 | 0.003247 | 0.010755 | Up |
| HNRNPA1P27 | 49.7255563 | 3.693069675 | 0.283536 | 13.02503 | 8.82E-39 | 3.38E-36 | Up |
| AL357568.1 | 1.233652549 | -1.007231274 | 0.407753 | -2.4702 | 0.013504 | 0.036161 | Down |
| MYCNOS | 16.96701026 | -1.557780058 | 0.185303 | -8.40667 | 4.22E-17 | 1.72E-15 | Down |
| LINC01122 | 12.95571793 | 1.056818517 | 0.249507 | 4.235619 | 2.28E-05 | 0.000134 | Up |
| LINC00284 | 19.06640288 | 3.766882537 | 0.32767 | 11.49596 | 1.38E-30 | 2.47E-28 | Up |
| LINC01765 | 4.127321927 | -2.348958182 | 0.449487 | -5.22586 | 1.73E-07 | 1.61E-06 | Down |
| LINC00656 | 0.872981903 | 2.055148571 | 0.488791 | 4.204551 | 2.62E-05 | 0.000152 | Up |
| AP001628.2 | 1.207412444 | 1.236398326 | 0.43938 | 2.813959 | 0.004894 | 0.01532 | Up |
| LINC01136 | 12.05538908 | -1.577138894 | 0.325336 | -4.84773 | 1.25E-06 | 9.74E-06 | Down |
| AL162727.1 | 7.983329123 | -2.017747816 | 0.36275 | -5.56237 | 2.66E-08 | 2.88E-07 | Down |
| AP000695.2 | 3.422243648 | 1.818448731 | 0.346027 | 5.255223 | 1.48E-07 | 1.39E-06 | Up |
| AL391839.2 | 2.718221724 | -1.734003699 | 0.353897 | -4.89974 | 9.60E-07 | 7.69E-06 | Down |
| LINC01949 | 1.362806805 | 1.323536122 | 0.531689 | 2.489304 | 0.012799 | 0.034551 | Up |
| KCNQ5-IT1 | 4.135877003 | -2.001106851 | 0.381807 | -5.24115 | 1.60E-07 | 1.49E-06 | Down |
| AC093732.1 | 2.919369765 | -1.121090368 | 0.297816 | -3.76437 | 0.000167 | 0.000801 | Down |
| AC005304.1 | 0.935226079 | -1.439088279 | 0.54345 | -2.64806 | 0.008096 | 0.023536 | Down |
| AC105271.1 | 1.631884701 | -1.018563805 | 0.389994 | -2.61174 | 0.009008 | 0.025778 | Down |
| LINC01503 | 101.2171513 | 1.096162591 | 0.155704 | 7.040055 | 1.92E-12 | 4.22E-11 | Up |
| GTF2F2P2 | 1.370482649 | -2.252617543 | 0.497371 | -4.52905 | 5.92E-06 | 4.01E-05 | Down |
| RPL10P9 | 439.0248349 | -1.380831105 | 0.390131 | -3.5394 | 0.000401 | 0.001736 | Down |
| AL591501.1 | 6.980563469 | -1.699153188 | 0.308332 | -5.51079 | 3.57E-08 | 3.77E-07 | Down |
| AL390778.2 | 2.793404079 | 1.372710532 | 0.333803 | 4.112343 | 3.92E-05 | 0.000219 | Up |
| AC004012.1 | 4.34641295 | 1.091683413 | 0.326001 | 3.34871 | 0.000812 | 0.003227 | Up |
| FTLP15 | 1.39919999 | -1.658082745 | 0.62634 | -2.64726 | 0.008115 | 0.023588 | Down |
| AC009970.1 | 1.859569952 | -2.4989245 | 0.515539 | -4.84721 | 1.25E-06 | 9.76E-06 | Down |
| BTF3P6 | 0.633235471 | -1.270051567 | 0.51032 | -2.48873 | 0.01282 | 0.034601 | Down |
| AC138951.1 | 3.731185305 | -1.855551431 | 0.425476 | -4.36112 | 1.29E-05 | 8.11E-05 | Down |
| SPATA17-AS1 | 0.828410079 | -2.341392911 | 0.595284 | -3.93324 | 8.38E-05 | 0.000431 | Down |
| TPRG1-AS1 | 23.02224288 | -1.293947945 | 0.263604 | -4.90868 | 9.17E-07 | 7.37E-06 | Down |
| AL157392.2 | 1.005530446 | -1.306680422 | 0.469765 | -2.78156 | 0.00541 | 0.016702 | Down |
| AL445224.1 | 3.113849029 | 1.709929349 | 0.502509 | 3.402786 | 0.000667 | 0.002717 | Up |
| AC073529.1 | 23.87099013 | -1.306736322 | 0.154103 | -8.47963 | 2.26E-17 | 9.48E-16 | Down |
| AL035420.1 | 1.849613773 | -1.058991768 | 0.331135 | -3.19807 | 0.001384 | 0.005136 | Down |
| AL035446.1 | 7.553847879 | 1.292313812 | 0.280293 | 4.610588 | 4.02E-06 | 2.81E-05 | Up |
| LINC02535 | 5.727647213 | 1.806705358 | 0.354809 | 5.092044 | 3.54E-07 | 3.11E-06 | Up |
| AC016683.1 | 4.203940393 | -1.170580869 | 0.437797 | -2.6738 | 0.0075 | 0.022036 | Down |
| LINC01191 | 2.814234909 | -1.182265263 | 0.326456 | -3.62152 | 0.000293 | 0.001316 | Down |
| TMEM229A | 3.294089183 | -2.584928928 | 0.430013 | -6.01128 | 1.84E-09 | 2.46E-08 | Down |
| ZFX-AS1 | 3.825777738 | -1.176245448 | 0.307518 | -3.82497 | 0.000131 | 0.000643 | Down |
| AC093616.1 | 20.43102835 | 1.023397097 | 0.178804 | 5.723554 | 1.04E-08 | 1.21E-07 | Up |
| AC243772.3 | 1.065985518 | 1.657321661 | 0.522766 | 3.170295 | 0.001523 | 0.005588 | Up |
| TULP3P1 | 0.659817582 | 2.923630552 | 0.882665 | 3.312278 | 0.000925 | 0.003619 | Up |
| LANCL1-AS1 | 24.38314688 | -1.026332132 | 0.128984 | -7.95703 | 1.76E-15 | 5.91E-14 | Down |
| LINC01878 | 12.95312369 | 3.041048779 | 0.407358 | 7.465292 | 8.31E-14 | 2.22E-12 | Up |
| AC099794.1 | 1.319728712 | -1.24182862 | 0.400924 | -3.09742 | 0.001952 | 0.006918 | Down |
| SNX18P7 | 1.705586665 | -2.941454219 | 0.495126 | -5.94082 | 2.84E-09 | 3.66E-08 | Down |
| LINC01426 | 31.55816964 | 1.700544009 | 0.233841 | 7.27222 | 3.54E-13 | 8.69E-12 | Up |
| AL590822.1 | 2.191271862 | -1.631657109 | 0.373432 | -4.36936 | 1.25E-05 | 7.84E-05 | Down |
| AC015983.2 | 4.735627412 | -1.182471704 | 0.273636 | -4.32133 | 1.55E-05 | 9.51E-05 | Down |
| AC002064.2 | 16.48949596 | -1.082490956 | 0.279854 | -3.86805 | 0.00011 | 0.00055 | Down |
| TLR12P | 0.74664696 | 1.544619562 | 0.553607 | 2.790102 | 0.005269 | 0.016326 | Up |
| AC005041.2 | 0.562597889 | 1.206116147 | 0.485132 | 2.486159 | 0.012913 | 0.034834 | Up |
| AL354751.1 | 1.589034787 | 2.946968531 | 0.446161 | 6.605173 | 3.97E-11 | 7.09E-10 | Up |
| LNCTAM34A | 67.69776253 | 1.55769952 | 0.164531 | 9.467489 | 2.87E-21 | 1.93E-19 | Up |
| SYNE1-AS1 | 14.77545355 | -1.501049207 | 0.256748 | -5.8464 | 5.02E-09 | 6.17E-08 | Down |
| C2CD4D-AS1 | 64.43541019 | 1.09545004 | 0.220763 | 4.962115 | 6.97E-07 | 5.75E-06 | Up |
| SNRK-AS1 | 36.92143057 | -1.348520274 | 0.240617 | -5.60443 | 2.09E-08 | 2.30E-07 | Down |
| AL606970.3 | 3.428885949 | -1.555324994 | 0.441425 | -3.52342 | 0.000426 | 0.001833 | Down |
| PCCA-AS1 | 3.134757136 | -1.271415114 | 0.339673 | -3.74306 | 0.000182 | 0.000861 | Down |
| AL512625.3 | 103.8147527 | -1.362243666 | 0.438335 | -3.10777 | 0.001885 | 0.006712 | Down |
| FAM170B-AS1 | 5.14017166 | 4.366672249 | 0.454446 | 9.608783 | 7.34E-22 | 5.26E-20 | Up |
| AC012618.2 | 9.583863125 | -1.163193629 | 0.263252 | -4.41856 | 9.94E-06 | 6.38E-05 | Down |
| GULOP | 2.697988828 | -1.303229832 | 0.347036 | -3.75532 | 0.000173 | 0.000825 | Down |
| HSPA8P3 | 0.781041115 | -1.350458411 | 0.57405 | -2.35251 | 0.018647 | 0.047215 | Down |
| AC090505.1 | 15.07336573 | 3.327675006 | 0.348888 | 9.537957 | 1.46E-21 | 1.01E-19 | Up |
| LINC01135 | 159.5746425 | -1.083690858 | 0.163082 | -6.64508 | 3.03E-11 | 5.51E-10 | Down |
| Z82214.1 | 3.039537132 | 1.469867188 | 0.402905 | 3.648176 | 0.000264 | 0.0012 | Up |
| APOC2 | 4.75763669 | 1.286520285 | 0.469651 | 2.73931 | 0.006157 | 0.018642 | Up |
| LINC01659 | 13.29936618 | -1.513899015 | 0.324459 | -4.66591 | 3.07E-06 | 2.21E-05 | Down |
| LINC02623 | 0.875313635 | 1.542554765 | 0.621646 | 2.481404 | 0.013087 | 0.035209 | Up |
| AP001626.1 | 10.76155645 | 1.421602681 | 0.262474 | 5.416161 | 6.09E-08 | 6.21E-07 | Up |
| DPP10-AS1 | 8.654158771 | 1.885864965 | 0.495631 | 3.804978 | 0.000142 | 0.000691 | Up |
| LINC00940 | 29.53298675 | -1.429767469 | 0.366279 | -3.90349 | 9.48E-05 | 0.000482 | Down |
| MLIP-AS1 | 1.727177657 | -2.73332857 | 0.464587 | -5.88335 | 4.02E-09 | 5.03E-08 | Down |
| AC062015.1 | 665.900879 | -1.718318047 | 0.337138 | -5.09677 | 3.45E-07 | 3.04E-06 | Down |
| AC012074.1 | 54.75147218 | -1.058829048 | 0.148843 | -7.11374 | 1.13E-12 | 2.60E-11 | Down |
| AL031667.2 | 1.123993476 | -1.073355829 | 0.402788 | -2.66482 | 0.007703 | 0.022549 | Down |
| RPL7L1P3 | 4.749958917 | -1.182032622 | 0.258024 | -4.5811 | 4.63E-06 | 3.20E-05 | Down |
| AC118555.1 | 0.493805072 | -1.351153869 | 0.564906 | -2.39182 | 0.016765 | 0.043244 | Down |
| LINC00330 | 1.842372266 | -1.425323098 | 0.399637 | -3.56655 | 0.000362 | 0.001585 | Down |
| ANKRD65 | 405.2513016 | 1.867166513 | 0.256062 | 7.291845 | 3.06E-13 | 7.57E-12 | Up |
| LINC02532 | 46.30990267 | -1.319672069 | 0.169249 | -7.79723 | 6.33E-15 | 1.98E-13 | Down |
| SMIM1 | 435.4152262 | 1.000430174 | 0.140461 | 7.122495 | 1.06E-12 | 2.45E-11 | Up |
| LINC01366 | 9.327019076 | 1.271307543 | 0.254615 | 4.993054 | 5.94E-07 | 4.97E-06 | Up |
| AL031727.2 | 1.276473654 | -1.519525097 | 0.458245 | -3.31597 | 0.000913 | 0.003577 | Down |
| AL022318.1 | 6.831967922 | 1.355710675 | 0.25157 | 5.389004 | 7.08E-08 | 7.12E-07 | Up |
| KDM5C-IT1 | 1.050148757 | -1.396660322 | 0.460323 | -3.03409 | 0.002413 | 0.008316 | Down |
| LINC02331 | 3.540000353 | -1.35878079 | 0.367305 | -3.69932 | 0.000216 | 0.001002 | Down |
| Z75741.2 | 0.587039913 | -1.930409103 | 0.651711 | -2.96207 | 0.003056 | 0.010201 | Down |
| SMIM2-IT1 | 0.777845998 | 2.091266677 | 0.535942 | 3.902038 | 9.54E-05 | 0.000484 | Up |
| DUSP5P2 | 7.805021374 | -1.635820107 | 0.23606 | -6.92969 | 4.22E-12 | 8.82E-11 | Down |
| LINC02154 | 2.868021594 | 1.599983789 | 0.533552 | 2.998739 | 0.002711 | 0.009191 | Up |
| AL157896.1 | 1.144186388 | -1.962111643 | 0.475457 | -4.12679 | 3.68E-05 | 0.000207 | Down |
| AL133481.1 | 1.041711964 | -1.201833783 | 0.466208 | -2.57789 | 0.00994 | 0.027966 | Down |
| AC006159.1 | 10.61381032 | -1.943518627 | 0.447313 | -4.34487 | 1.39E-05 | 8.66E-05 | Down |
| DPY19L2P4 | 30.44769879 | -1.584503077 | 0.219914 | -7.2051 | 5.80E-13 | 1.39E-11 | Down |
| ESRRAP2 | 29.38024846 | -1.593899173 | 0.203209 | -7.84366 | 4.38E-15 | 1.40E-13 | Down |
| AC013270.1 | 6.139640765 | -1.020982573 | 0.224137 | -4.55518 | 5.23E-06 | 3.58E-05 | Down |
| BX842568.4 | 15.17271312 | -1.711955472 | 0.263829 | -6.48887 | 8.65E-11 | 1.46E-09 | Down |
| LINC00402 | 25.75487431 | -2.133372946 | 0.639521 | -3.33589 | 0.00085 | 0.003359 | Down |
| NFAM1 | 681.7349356 | 1.27169294 | 0.273253 | 4.653901 | 3.26E-06 | 2.33E-05 | Up |
| LINC00533 | 2.560780062 | -1.592629562 | 0.45811 | -3.47652 | 0.000508 | 0.00214 | Down |
| AC073150.1 | 1.124418688 | -1.205462239 | 0.450615 | -2.67515 | 0.00747 | 0.02197 | Down |
| AC008268.1 | 19.46200731 | -1.118055321 | 0.277152 | -4.03409 | 5.48E-05 | 0.000296 | Down |
| AC011247.1 | 11.20466266 | -1.379070968 | 0.256324 | -5.38019 | 7.44E-08 | 7.44E-07 | Down |
| RBMS3-AS1 | 1.54724881 | -2.33964997 | 0.45768 | -5.11197 | 3.19E-07 | 2.82E-06 | Down |
| AL353681.1 | 4.678869686 | 1.20274104 | 0.311183 | 3.865065 | 0.000111 | 0.000556 | Up |
| RPL36AP33 | 1.03143554 | -1.197841405 | 0.500809 | -2.39181 | 0.016765 | 0.043244 | Down |
| LINC00494 | 23.13759004 | -2.061973502 | 0.611388 | -3.37261 | 0.000745 | 0.002987 | Down |
| CYCSP52 | 1.67634708 | -1.288672497 | 0.392218 | -3.2856 | 0.001018 | 0.003932 | Down |
| AC013268.3 | 3.866957206 | 1.078338182 | 0.309434 | 3.484875 | 0.000492 | 0.002081 | Up |
| AC010148.1 | 8.723002565 | -1.248025119 | 0.26644 | -4.68407 | 2.81E-06 | 2.04E-05 | Down |
| LINC02250 | 1.311552472 | -1.699173401 | 0.501099 | -3.3909 | 0.000697 | 0.00282 | Down |
| AL031275.1 | 1.15389525 | 1.054923578 | 0.40697 | 2.592143 | 0.009538 | 0.02704 | Up |
| AL390860.1 | 4.904557517 | -1.233682722 | 0.367372 | -3.35813 | 0.000785 | 0.003133 | Down |
| AC138655.1 | 9.060234345 | -1.054710905 | 0.194541 | -5.42153 | 5.91E-08 | 6.04E-07 | Down |
| LINC00607 | 45.45077014 | 2.243148009 | 0.261344 | 8.583117 | 9.23E-18 | 4.04E-16 | Up |
| AL136099.1 | 0.810121427 | -1.825937032 | 0.620601 | -2.94221 | 0.003259 | 0.01079 | Down |
| Z93022.1 | 1.061824634 | -1.431337303 | 0.465326 | -3.07599 | 0.002098 | 0.007358 | Down |
| LINC00941 | 12.00586192 | 1.361720134 | 0.209623 | 6.496033 | 8.25E-11 | 1.40E-09 | Up |
| TSPEAR-AS1 | 30.09539811 | -1.065396579 | 0.238555 | -4.46603 | 7.97E-06 | 5.24E-05 | Down |
| LINC01564 | 5.453215585 | -2.337477683 | 0.367285 | -6.3642 | 1.96E-10 | 3.10E-09 | Down |
| AC108472.1 | 11.34576745 | -1.471706353 | 0.258875 | -5.685 | 1.31E-08 | 1.50E-07 | Down |
| LINC01779 | 1.849427712 | -1.155335589 | 0.441565 | -2.61646 | 0.008885 | 0.025477 | Down |
| AL138760.1 | 3.91081859 | -1.142548341 | 0.397108 | -2.87718 | 0.004013 | 0.012913 | Down |
| MTND1P21 | 0.414154557 | -1.39897285 | 0.587171 | -2.38257 | 0.017192 | 0.04416 | Down |
| EGOT | 26.43025609 | 1.548789288 | 0.264346 | 5.858956 | 4.66E-09 | 5.76E-08 | Up |
| VN2R19P | 10.22628864 | -1.185253501 | 0.266802 | -4.44244 | 8.89E-06 | 5.79E-05 | Down |
| AC018816.1 | 87.09227761 | 2.937142828 | 0.242539 | 12.11 | 9.35E-34 | 2.13E-31 | Up |
| AL357146.1 | 65.10047952 | -1.91365745 | 0.176231 | -10.8588 | 1.81E-27 | 2.37E-25 | Down |
| AC019117.1 | 4.333062015 | 1.606298733 | 0.37932 | 4.234679 | 2.29E-05 | 0.000135 | Up |
| Z82246.1 | 2.744257161 | -1.285473725 | 0.341623 | -3.76285 | 0.000168 | 0.000804 | Down |
| AL034399.2 | 3.245499393 | 1.172451764 | 0.299116 | 3.919717 | 8.87E-05 | 0.000454 | Up |
| SCN1A-AS1 | 8.431079157 | -1.715849347 | 0.385764 | -4.44793 | 8.67E-06 | 5.66E-05 | Down |
| AL021408.1 | 6.796093218 | -1.02206763 | 0.208589 | -4.89992 | 9.59E-07 | 7.68E-06 | Down |
| AL049612.1 | 5.142613388 | 1.238611491 | 0.323685 | 3.826593 | 0.00013 | 0.00064 | Up |
| PRKX-AS1 | 1.693122869 | -1.774157921 | 0.455672 | -3.8935 | 9.88E-05 | 0.0005 | Down |
| GRM7-AS1 | 0.586207471 | 1.608864881 | 0.682904 | 2.355918 | 0.018477 | 0.046844 | Up |
| AL356108.1 | 0.929922184 | -1.147747469 | 0.470402 | -2.43993 | 0.01469 | 0.038782 | Down |
| DIAPH2-AS1 | 2.972916777 | 1.218964532 | 0.335281 | 3.635647 | 0.000277 | 0.001253 | Up |
| PPIAP64 | 1.110636683 | -1.285639264 | 0.478924 | -2.68443 | 0.007265 | 0.02148 | Down |
| SHANK2-AS2 | 0.776669108 | -1.729679242 | 0.582688 | -2.96845 | 0.002993 | 0.01002 | Down |
| LINC01361 | 11.29734285 | -1.663930486 | 0.220753 | -7.53751 | 4.79E-14 | 1.32E-12 | Down |
| GACAT3 | 0.877584143 | -1.56069292 | 0.597685 | -2.61123 | 0.009022 | 0.025809 | Down |
| AC019117.2 | 7.706672934 | 2.071528889 | 0.439124 | 4.717413 | 2.39E-06 | 1.76E-05 | Up |
| TRHDE-AS1 | 8.712004697 | -2.039371788 | 0.321253 | -6.34818 | 2.18E-10 | 3.42E-09 | Down |
| AC026355.2 | 0.805383528 | 3.254103721 | 0.683757 | 4.759152 | 1.94E-06 | 1.46E-05 | Up |
| SLC35G4 | 0.66352068 | -1.367869067 | 0.565296 | -2.41974 | 0.015532 | 0.040591 | Down |
| CTSLP1 | 5.656743479 | -1.676859595 | 0.371279 | -4.51644 | 6.29E-06 | 4.23E-05 | Down |
| AC067956.1 | 18.02739732 | -1.431945039 | 0.304061 | -4.7094 | 2.48E-06 | 1.82E-05 | Down |
| AL158168.1 | 2.081403348 | -2.185351265 | 0.466901 | -4.68055 | 2.86E-06 | 2.08E-05 | Down |
| ATP13A5-AS1 | 1.48495327 | -1.220809856 | 0.412735 | -2.95786 | 0.003098 | 0.010322 | Down |
| AC011284.1 | 0.614085047 | 1.788467805 | 0.55966 | 3.195632 | 0.001395 | 0.005174 | Up |
| AL135791.1 | 36.07495863 | -1.414818856 | 0.181339 | -7.80206 | 6.09E-15 | 1.91E-13 | Down |
| KLF2P4 | 1.149732629 | -1.882964553 | 0.504484 | -3.73246 | 0.00019 | 0.000893 | Down |
| LINC01695 | 8.020110321 | -2.172893673 | 0.267678 | -8.11757 | 4.76E-16 | 1.71E-14 | Down |
| AL354761.1 | 0.698342519 | -1.943329503 | 0.56105 | -3.46374 | 0.000533 | 0.002233 | Down |
| STARD13-AS | 31.85713937 | -1.374511897 | 0.235149 | -5.84529 | 5.06E-09 | 6.21E-08 | Down |
| AL590302.2 | 3.594089128 | -1.300391385 | 0.519324 | -2.50401 | 0.01228 | 0.033395 | Down |
| AL161733.1 | 0.507278293 | 1.936803549 | 0.820572 | 2.360308 | 0.01826 | 0.046398 | Up |
| ARHGEF38 | 346.3604405 | 1.037775945 | 0.302846 | 3.426742 | 0.000611 | 0.002521 | Up |
| AC073316.3 | 3.8309599 | 2.117189706 | 0.437794 | 4.836042 | 1.32E-06 | 1.03E-05 | Up |
| SMAD9-IT1 | 29.49604443 | -1.811880777 | 0.226461 | -8.00086 | 1.24E-15 | 4.22E-14 | Down |
| OVAAL | 0.477416183 | 1.634843303 | 0.694441 | 2.354187 | 0.018563 | 0.047025 | Up |
| AL135929.2 | 1.952966859 | 3.531066242 | 0.631276 | 5.593536 | 2.22E-08 | 2.44E-07 | Up |
| AC094019.1 | 6.851712819 | -1.217465639 | 0.303076 | -4.01704 | 5.89E-05 | 0.000316 | Down |
| AC009237.9 | 16.49038623 | -1.693269371 | 0.313605 | -5.39937 | 6.69E-08 | 6.75E-07 | Down |
| SNX25P1 | 18.48368239 | 1.385969955 | 0.176337 | 7.859786 | 3.85E-15 | 1.24E-13 | Up |
| AC007750.1 | 4.442256943 | 1.67441896 | 0.360449 | 4.645365 | 3.39E-06 | 2.42E-05 | Up |
| AL157902.1 | 3.500582164 | -1.712429204 | 0.348435 | -4.91463 | 8.90E-07 | 7.17E-06 | Down |
| AC007563.2 | 7.497952521 | -1.412280598 | 0.250138 | -5.64601 | 1.64E-08 | 1.84E-07 | Down |
| LINC01141 | 1.520065894 | 2.308580296 | 0.39341 | 5.868125 | 4.41E-09 | 5.47E-08 | Up |
| GGT8P | 0.843320344 | 1.571728097 | 0.488717 | 3.216027 | 0.0013 | 0.004863 | Up |
| MLIP-IT1 | 10.78807899 | -1.107796757 | 0.273143 | -4.05575 | 5.00E-05 | 0.000273 | Down |
| WASF3-AS1 | 1.310851294 | -1.943611697 | 0.596507 | -3.25832 | 0.001121 | 0.004269 | Down |
| LINC01812 | 0.514173191 | 1.371021179 | 0.483562 | 2.835253 | 0.004579 | 0.014484 | Up |
| RPL21P33 | 1.26092879 | -1.501241179 | 0.516868 | -2.9045 | 0.003678 | 0.011973 | Down |
| AC100823.1 | 4.091876689 | -2.060779666 | 0.338297 | -6.09162 | 1.12E-09 | 1.54E-08 | Down |
| AL596218.1 | 1.118269082 | 1.773781172 | 0.477737 | 3.712885 | 0.000205 | 0.000957 | Up |
| AC092809.4 | 16.28002975 | 1.12258265 | 0.172661 | 6.501647 | 7.94E-11 | 1.35E-09 | Up |
| AL139805.1 | 61.23473523 | -1.239468782 | 0.198324 | -6.2497 | 4.11E-10 | 6.13E-09 | Down |
| LINC02652 | 2.067390693 | -1.882901747 | 0.399006 | -4.71898 | 2.37E-06 | 1.75E-05 | Down |
| C4orf51 | 4.387187984 | -1.8774479 | 0.320603 | -5.856 | 4.74E-09 | 5.85E-08 | Down |
| AL353718.1 | 0.733984377 | -1.648966598 | 0.673867 | -2.44702 | 0.014404 | 0.038166 | Down |
| AC068446.1 | 2.8231501 | -1.082066996 | 0.37301 | -2.9009 | 0.003721 | 0.012097 | Down |
| LINC01792 | 1.139342077 | -2.859553648 | 0.523603 | -5.4613 | 4.73E-08 | 4.91E-07 | Down |
| NR2F1-AS1 | 390.4943704 | 2.700200806 | 0.214248 | 12.60318 | 2.03E-36 | 5.90E-34 | Up |
| LINC01968 | 7.091668134 | 1.226763281 | 0.291333 | 4.210867 | 2.54E-05 | 0.000148 | Up |
| AC022173.1 | 1.015971589 | -1.145580259 | 0.454768 | -2.51904 | 0.011767 | 0.032231 | Down |
| AL049646.2 | 0.781589198 | 1.235264196 | 0.417823 | 2.956426 | 0.003112 | 0.010364 | Up |
| LINC01519 | 7.426004384 | -1.698411521 | 0.384624 | -4.41577 | 1.01E-05 | 6.46E-05 | Down |
| AL136982.3 | 0.95735942 | -1.276760812 | 0.501769 | -2.54452 | 0.010943 | 0.030297 | Down |
| CATIP-AS2 | 23.94552752 | -1.33807293 | 0.203921 | -6.56174 | 5.32E-11 | 9.32E-10 | Down |
| LINC01732 | 0.713261067 | 2.144927651 | 0.493859 | 4.343197 | 1.40E-05 | 8.72E-05 | Up |
| UPP2-IT1 | 1.238329195 | 1.362116206 | 0.377296 | 3.610202 | 0.000306 | 0.001369 | Up |
| RNF223 | 4.657668192 | 2.390716077 | 0.439172 | 5.443694 | 5.22E-08 | 5.38E-07 | Up |
| LINC01502 | 52.51730969 | -2.335275416 | 0.542468 | -4.30491 | 1.67E-05 | 0.000102 | Down |
| AL645634.2 | 1.239790768 | -1.158242622 | 0.488934 | -2.36891 | 0.01784 | 0.045525 | Down |
| LNCNEF | 86.71087114 | -2.98992917 | 0.361517 | -8.27052 | 1.33E-16 | 5.18E-15 | Down |
| FOXD2-AS1 | 49.45788561 | 1.262754773 | 0.189585 | 6.660614 | 2.73E-11 | 5.00E-10 | Up |
| BX293535.1 | 3.499892749 | 1.034025223 | 0.309422 | 3.341801 | 0.000832 | 0.003297 | Up |
| AL512635.1 | 0.803590552 | -1.794742182 | 0.468561 | -3.83032 | 0.000128 | 0.000631 | Down |
| HNRNPA1P57 | 1.792391116 | 1.056625672 | 0.377552 | 2.798622 | 0.005132 | 0.015967 | Up |
| LRRC52-AS1 | 308.4773137 | 7.015804021 | 0.429565 | 16.33235 | 5.81E-60 | 3.12E-56 | Up |
| AC007557.4 | 0.857182002 | -1.916740459 | 0.539595 | -3.55218 | 0.000382 | 0.001664 | Down |
| VN1R48P | 8.545135898 | -1.965483857 | 0.346413 | -5.67382 | 1.40E-08 | 1.59E-07 | Down |
| UNC5B-AS1 | 16.90797948 | 4.277537952 | 0.328495 | 13.02162 | 9.22E-39 | 3.49E-36 | Up |
| SHISA9 | 35.1778777 | 1.188939036 | 0.365879 | 3.24954 | 0.001156 | 0.004386 | Up |
| AC012668.3 | 5.879903622 | 2.984332349 | 0.325163 | 9.177953 | 4.39E-20 | 2.61E-18 | Up |
| AL672277.1 | 8.261868027 | -1.105101871 | 0.316435 | -3.49236 | 0.000479 | 0.002033 | Down |
| TTLL11-IT1 | 1.470081389 | 1.109430418 | 0.381855 | 2.905374 | 0.003668 | 0.011942 | Up |
| YWHABP1 | 1.663749578 | -1.541903562 | 0.48933 | -3.15105 | 0.001627 | 0.005909 | Down |
| LINC01497 | 11.87642584 | -1.378812482 | 0.268066 | -5.14356 | 2.70E-07 | 2.42E-06 | Down |
| Z97206.2 | 0.800364645 | 1.415080306 | 0.488759 | 2.89525 | 0.003789 | 0.012289 | Up |
| LINC01856 | 0.605437821 | -1.468648266 | 0.611156 | -2.40307 | 0.016258 | 0.042162 | Down |
| AL445220.1 | 14.56359296 | -2.877436227 | 0.368503 | -7.80845 | 5.79E-15 | 1.83E-13 | Down |
| AP000251.1 | 5.193104971 | 1.573744046 | 0.540409 | 2.912137 | 0.00359 | 0.01172 | Up |
| AF064858.2 | 1.918966507 | -1.076855363 | 0.41413 | -2.60028 | 0.009315 | 0.02651 | Down |
| AP002856.4 | 2.07469509 | -2.177859755 | 0.491961 | -4.4269 | 9.56E-06 | 6.17E-05 | Down |
| OXCT2P1 | 33.44156973 | -1.087460298 | 0.269482 | -4.03537 | 5.45E-05 | 0.000295 | Down |
| FRY-AS1 | 2.316672729 | -1.069089057 | 0.339585 | -3.14822 | 0.001643 | 0.005955 | Down |
| AC004522.2 | 1.486165578 | -1.044710316 | 0.38149 | -2.7385 | 0.006172 | 0.018675 | Down |
| HLCS-IT1 | 1.404959467 | -1.001352132 | 0.416336 | -2.40515 | 0.016166 | 0.04196 | Down |
| LINC01115 | 21.14288238 | -1.302388384 | 0.43706 | -2.97988 | 0.002884 | 0.0097 | Down |
| VDAC1P11 | 0.754114927 | -1.437174739 | 0.478922 | -3.00085 | 0.002692 | 0.009137 | Down |
| IFNWP2 | 2.924860169 | 2.845439807 | 0.405947 | 7.00939 | 2.39E-12 | 5.20E-11 | Up |
| AL031726.1 | 1.792366865 | -1.382549681 | 0.360619 | -3.83382 | 0.000126 | 0.000623 | Down |
| P4HA2-AS1 | 3.159630568 | 1.943030498 | 0.250338 | 7.761616 | 8.39E-15 | 2.58E-13 | Up |
| AC010980.1 | 130.4587219 | -1.608537744 | 0.257005 | -6.25877 | 3.88E-10 | 5.83E-09 | Down |
| DCTN1-AS1 | 7.304563492 | 2.19444829 | 0.28908 | 7.591151 | 3.17E-14 | 8.92E-13 | Up |
| NPAP1P3 | 1.153649861 | -1.255721266 | 0.467814 | -2.68423 | 0.00727 | 0.021489 | Down |
| AL365259.1 | 52.632294 | 1.7886962 | 0.30829 | 5.802002 | 6.55E-09 | 7.89E-08 | Up |
| ZNF101P1 | 1.070200889 | -2.134586674 | 0.614335 | -3.47463 | 0.000512 | 0.002153 | Down |
| GGTA2P | 8.437721761 | -1.005217365 | 0.218582 | -4.59882 | 4.25E-06 | 2.96E-05 | Down |
| AC022034.1 | 122.7231452 | -1.378112668 | 0.261948 | -5.26101 | 1.43E-07 | 1.35E-06 | Down |
| AC002066.1 | 75.33746603 | -1.223620093 | 0.232708 | -5.25819 | 1.45E-07 | 1.37E-06 | Down |
| AL139161.2 | 2.53355081 | -2.318267157 | 0.845829 | -2.74082 | 0.006129 | 0.018578 | Down |
| AL023584.2 | 1.50633232 | -1.242673481 | 0.531264 | -2.33909 | 0.019331 | 0.04868 | Down |
| NFIA-AS1 | 5.129529364 | -1.118518674 | 0.272174 | -4.10958 | 3.96E-05 | 0.000221 | Down |
| AL035425.1 | 143.4188853 | -1.705320691 | 0.264873 | -6.43825 | 1.21E-10 | 1.98E-09 | Down |
| AC073130.1 | 20.12930176 | -2.135950913 | 0.321332 | -6.64718 | 2.99E-11 | 5.44E-10 | Down |
| DGUOK-AS1 | 88.00221426 | 1.137416784 | 0.143629 | 7.91913 | 2.39E-15 | 7.91E-14 | Up |
| AL158840.1 | 1.288942687 | -1.829748949 | 0.483566 | -3.78387 | 0.000154 | 0.000747 | Down |
| KCNMB2-AS1 | 2.752040045 | 2.885071681 | 0.348498 | 8.278578 | 1.25E-16 | 4.87E-15 | Up |
| AL391832.2 | 10.75408004 | 1.21106349 | 0.253834 | 4.771083 | 1.83E-06 | 1.38E-05 | Up |
| ARMC4P1 | 25.98746467 | -1.096419404 | 0.256975 | -4.26664 | 1.98E-05 | 0.000119 | Down |
| LINC02257 | 1.912227456 | 3.237383874 | 0.550703 | 5.878636 | 4.14E-09 | 5.16E-08 | Up |
| ART2P | 13.29930658 | -1.089088895 | 0.378211 | -2.87958 | 0.003982 | 0.012836 | Down |
| SPATA3-AS1 | 2.410388786 | 1.612861404 | 0.581858 | 2.771918 | 0.005573 | 0.017111 | Up |
| AC010740.1 | 0.699927886 | -1.569689507 | 0.482727 | -3.25171 | 0.001147 | 0.004357 | Down |
| LINC01625 | 8.950877041 | -1.146649421 | 0.277415 | -4.13333 | 3.58E-05 | 0.000201 | Down |
| LINC01589 | 5.105350825 | -1.733644518 | 0.357388 | -4.85087 | 1.23E-06 | 9.61E-06 | Down |
| AL357055.3 | 52.53156245 | -1.004178048 | 0.139464 | -7.20028 | 6.01E-13 | 1.44E-11 | Down |
| AC009312.1 | 1.01318906 | -1.764218273 | 0.505868 | -3.48751 | 0.000488 | 0.002064 | Down |
| LINC01877 | 6.704818871 | -1.511539554 | 0.269448 | -5.60977 | 2.03E-08 | 2.23E-07 | Down |
| MKRN4P | 9.759153652 | 1.180163268 | 0.300931 | 3.921702 | 8.79E-05 | 0.00045 | Up |
| CCR12P | 2.157505461 | -3.038678352 | 0.906325 | -3.35275 | 0.0008 | 0.003188 | Down |
| LINC00707 | 2.237854633 | 1.541057148 | 0.414953 | 3.713814 | 0.000204 | 0.000953 | Up |
| IFNWP19 | 45.82332449 | 2.693039147 | 0.251989 | 10.68714 | 1.17E-26 | 1.38E-24 | Up |
| AC108058.1 | 28.26259616 | -1.878553296 | 0.323936 | -5.79915 | 6.67E-09 | 8.00E-08 | Down |
| AC004969.1 | 1.000158821 | -1.685517312 | 0.478213 | -3.52461 | 0.000424 | 0.001827 | Down |
| RNU1-13P | 1.975158365 | -2.985359445 | 0.645474 | -4.62507 | 3.74E-06 | 2.64E-05 | Down |
| RF00096.18 | 1.644777262 | -1.581192022 | 0.416428 | -3.79703 | 0.000146 | 0.000712 | Down |
| RF00096.19 | 1.247363167 | -1.075561294 | 0.415839 | -2.58648 | 0.009696 | 0.027412 | Down |
| SCARNA10 | 4.559308191 | -1.138427784 | 0.361624 | -3.1481 | 0.001643 | 0.005956 | Down |
| RBM43P1 | 3.993964185 | -1.337244954 | 0.429989 | -3.10995 | 0.001871 | 0.006668 | Down |
| AC092691.1 | 2.447350009 | -1.720437726 | 0.383412 | -4.48718 | 7.22E-06 | 4.79E-05 | Down |
| AC108693.1 | 1.135533631 | -1.473319126 | 0.45714 | -3.22291 | 0.001269 | 0.004762 | Down |
| RPS26P49 | 1.695002596 | 2.665682641 | 0.346682 | 7.689139 | 1.48E-14 | 4.41E-13 | Up |
| AC074043.1 | 1.076891239 | -1.469695889 | 0.468222 | -3.13888 | 0.001696 | 0.00612 | Down |
| AC019211.1 | 11.21028113 | -1.477960985 | 0.304835 | -4.8484 | 1.24E-06 | 9.72E-06 | Down |
| MARK2P8 | 1.545316113 | -1.291856698 | 0.42149 | -3.06497 | 0.002177 | 0.007602 | Down |
| PLCH1-AS1 | 2.068492179 | -1.933115847 | 0.453447 | -4.26316 | 2.02E-05 | 0.00012 | Down |
| RN7SL399P | 2.315069815 | -1.50610211 | 0.432359 | -3.48345 | 0.000495 | 0.002092 | Down |
| AC108749.1 | 0.559741581 | 1.192648691 | 0.474494 | 2.513518 | 0.011953 | 0.032643 | Up |
| AL513122.2 | 6.665703725 | -1.653584077 | 0.440666 | -3.75246 | 0.000175 | 0.000833 | Down |
| AL354710.2 | 3.855808501 | -1.546485237 | 0.342225 | -4.51892 | 6.22E-06 | 4.18E-05 | Down |
| AC093627.2 | 1.903812092 | 1.550319056 | 0.514656 | 3.012338 | 0.002592 | 0.008845 | Up |
| RN7SL688P | 1.343519634 | -2.385804421 | 0.463092 | -5.1519 | 2.58E-07 | 2.32E-06 | Down |
| RN7SL825P | 0.627642075 | 1.202495337 | 0.463574 | 2.593966 | 0.009488 | 0.026922 | Up |
| AC125618.1 | 6.737813685 | -1.296321081 | 0.232716 | -5.57041 | 2.54E-08 | 2.76E-07 | Down |
| AL121776.1 | 1.365540151 | -1.217969003 | 0.432981 | -2.81299 | 0.004908 | 0.015359 | Down |
| RPS27P25 | 1.493339942 | 1.315347955 | 0.518318 | 2.537722 | 0.011158 | 0.030779 | Up |
| C1orf226 | 253.5698276 | 1.394794086 | 0.172748 | 8.07414 | 6.80E-16 | 2.39E-14 | Up |
| AC092958.1 | 0.617962779 | -1.731534389 | 0.638295 | -2.71275 | 0.006673 | 0.019964 | Down |
| PRDX3P4 | 1.938795052 | -1.00710405 | 0.417305 | -2.41335 | 0.015806 | 0.041175 | Down |
| TRBV8-2 | 0.359119859 | 1.678670843 | 0.652294 | 2.573486 | 0.010068 | 0.028265 | Up |
| DWORF | 1.385775481 | -1.312579806 | 0.500151 | -2.62437 | 0.008681 | 0.024972 | Down |
| AC069431.1 | 1.943040427 | -1.305096308 | 0.384071 | -3.39806 | 0.000679 | 0.002757 | Down |
| RN7SL146P | 0.892570416 | -1.212988497 | 0.486901 | -2.49124 | 0.01273 | 0.034392 | Down |
| ETF1P2 | 3.507317296 | -1.027082472 | 0.29734 | -3.45423 | 0.000552 | 0.002304 | Down |
| AC087385.1 | 3.878319483 | -1.961442431 | 0.472021 | -4.15542 | 3.25E-05 | 0.000185 | Down |
| BX664727.3 | 18.36487526 | -1.051510919 | 0.177003 | -5.94063 | 2.84E-09 | 3.67E-08 | Down |
| B4GALT4-AS1 | 0.764876309 | -1.435853582 | 0.512533 | -2.80149 | 0.005087 | 0.015849 | Down |
| AC099328.1 | 3.935656649 | -1.550445296 | 0.330684 | -4.6886 | 2.75E-06 | 2.00E-05 | Down |
| AC091805.1 | 0.531387521 | -1.298033691 | 0.51781 | -2.50678 | 0.012184 | 0.033185 | Down |
| LINC00973 | 5.458388414 | 2.261371448 | 0.36518 | 6.19248 | 5.92E-10 | 8.55E-09 | Up |
| AC093010.1 | 6.543014443 | -2.609069517 | 0.645772 | -4.04024 | 5.34E-05 | 0.00029 | Down |
| CDKN2B-AS1 | 11.38747119 | 2.237850197 | 0.315239 | 7.098903 | 1.26E-12 | 2.87E-11 | Up |
| TNFRSF13B | 125.8989719 | -1.836338666 | 0.634212 | -2.89547 | 0.003786 | 0.012282 | Down |
| AC092979.1 | 0.774079599 | -1.814665537 | 0.683139 | -2.65636 | 0.007899 | 0.023057 | Down |
| TM4SF1-AS1 | 12.183758 | 1.932500883 | 0.319839 | 6.042098 | 1.52E-09 | 2.06E-08 | Up |
| THOC7-AS1 | 1.479548036 | -1.49862652 | 0.424744 | -3.5283 | 0.000418 | 0.001804 | Down |
| L1TD1 | 31.29764374 | -1.38126502 | 0.276571 | -4.99426 | 5.91E-07 | 4.94E-06 | Down |
| AQP1 | 7507.770876 | 1.152606576 | 0.167469 | 6.882508 | 5.88E-12 | 1.20E-10 | Up |
| RN7SL258P | 1.513984913 | -1.147991406 | 0.409982 | -2.8001 | 0.005109 | 0.015904 | Down |
| AADACP1 | 155.0642205 | -2.350678661 | 0.312309 | -7.52677 | 5.20E-14 | 1.43E-12 | Down |
| AC006539.1 | 5.510975368 | 1.195143652 | 0.271175 | 4.40728 | 1.05E-05 | 6.69E-05 | Up |
| AC146507.2 | 2.261296858 | -1.143811534 | 0.365473 | -3.12968 | 0.00175 | 0.006291 | Down |
| RN7SL559P | 1.913676435 | -1.888043258 | 0.414804 | -4.55165 | 5.32E-06 | 3.63E-05 | Down |
| PCDHGC5 | 25.70959947 | 2.205774553 | 0.320303 | 6.886532 | 5.72E-12 | 1.17E-10 | Up |
| AC092916.2 | 4.386339792 | -1.401158414 | 0.449645 | -3.11614 | 0.001832 | 0.006546 | Down |
| AC073934.1 | 2.393973782 | -1.593775574 | 0.343593 | -4.63855 | 3.51E-06 | 2.49E-05 | Down |
| AC126389.1 | 2.165491982 | -1.098787927 | 0.31388 | -3.50066 | 0.000464 | 0.001977 | Down |
| AC025271.1 | 2.420038696 | -1.16559614 | 0.347856 | -3.3508 | 0.000806 | 0.003206 | Down |
| RN7SL209P | 3.511976551 | -1.490582821 | 0.294301 | -5.06482 | 4.09E-07 | 3.53E-06 | Down |
| HOXA11-AS | 2.009417196 | 3.318382669 | 0.555247 | 5.976413 | 2.28E-09 | 2.99E-08 | Up |
| AC069454.1 | 1.095217834 | -1.348393666 | 0.447692 | -3.01188 | 0.002596 | 0.008858 | Down |
| AC105910.1 | 1.948154555 | -1.923916228 | 0.398673 | -4.8258 | 1.39E-06 | 1.08E-05 | Down |
| RPL22P2 | 8.270940226 | 1.609933941 | 0.246377 | 6.534434 | 6.39E-11 | 1.10E-09 | Up |
| PRICKLE2-AS2 | 1.802878483 | -2.172525051 | 0.468133 | -4.64083 | 3.47E-06 | 2.47E-05 | Down |
| AC092040.1 | 9.020753176 | -2.06251561 | 0.312755 | -6.59467 | 4.26E-11 | 7.56E-10 | Down |
| RPL7P41 | 3.967330582 | -4.085184343 | 0.654243 | -6.24414 | 4.26E-10 | 6.33E-09 | Down |
| LINC02066 | 1.552061273 | -1.838479053 | 0.474317 | -3.87606 | 0.000106 | 0.000534 | Down |
| TDGF1 | 78.4326724 | -1.112739578 | 0.204676 | -5.43659 | 5.43E-08 | 5.58E-07 | Down |
| IQCJ-SCHIP1-AS1 | 0.570401996 | -1.926097912 | 0.699148 | -2.75492 | 0.005871 | 0.017891 | Down |
| AC104472.2 | 4.224044943 | -1.032796316 | 0.259375 | -3.98186 | 6.84E-05 | 0.00036 | Down |
| CDRT1 | 14.38204714 | -1.239329776 | 0.205856 | -6.02038 | 1.74E-09 | 2.33E-08 | Down |
| AC092849.2 | 2.762568401 | -1.376475936 | 0.367637 | -3.74411 | 0.000181 | 0.000858 | Down |
| HNF1A-AS1 | 1.889465897 | -1.318400792 | 0.508 | -2.59528 | 0.009451 | 0.026827 | Down |
| AC092903.1 | 4.692122643 | -2.072748793 | 0.363778 | -5.69784 | 1.21E-08 | 1.40E-07 | Down |
| AC093010.2 | 21.57608734 | -2.849597639 | 0.480215 | -5.934 | 2.96E-09 | 3.80E-08 | Down |
| AC018638.3 | 1.383082375 | -1.265183511 | 0.408214 | -3.09932 | 0.00194 | 0.006878 | Down |
| AGGF1P3 | 3.131006739 | -1.035877819 | 0.434379 | -2.38473 | 0.017092 | 0.043943 | Down |
| AC134050.1 | 1.032414957 | -1.910717553 | 0.515398 | -3.70727 | 0.00021 | 0.000976 | Down |
| LINC02029 | 3.95641883 | -1.492184566 | 0.428142 | -3.48526 | 0.000492 | 0.002079 | Down |
| RN7SL338P | 1.234159674 | -1.132361556 | 0.428442 | -2.64298 | 0.008218 | 0.023846 | Down |
| AC099542.1 | 2.797887911 | -1.714685423 | 0.356992 | -4.80315 | 1.56E-06 | 1.19E-05 | Down |
| AC092757.1 | 0.74769599 | -1.376026975 | 0.539346 | -2.55129 | 0.010733 | 0.029821 | Down |
| INMT | 2404.520512 | 2.001602384 | 0.292614 | 6.840428 | 7.90E-12 | 1.58E-10 | Up |
| RN7SL253P | 0.70598797 | 1.92932283 | 0.426644 | 4.522092 | 6.12E-06 | 4.13E-05 | Up |
| AC018450.1 | 17.44301811 | 1.955975378 | 0.208165 | 9.396269 | 5.65E-21 | 3.72E-19 | Up |
| TMEFF1 | 23.60949014 | -1.273774062 | 0.250647 | -5.08194 | 3.74E-07 | 3.26E-06 | Down |
| RN7SL265P | 1.123187468 | -1.941531072 | 0.515793 | -3.76417 | 0.000167 | 0.000801 | Down |
| RPSAP52 | 27.6274838 | 3.94055052 | 0.380963 | 10.34365 | 4.47E-25 | 4.51E-23 | Up |
| LINC01324 | 2.659800135 | -1.035557554 | 0.323403 | -3.20206 | 0.001364 | 0.005074 | Down |
| RPL21P54 | 2.643539498 | -1.361377917 | 0.31295 | -4.35014 | 1.36E-05 | 8.47E-05 | Down |
| RN7SL363P | 2.036327066 | -1.251416395 | 0.389673 | -3.21145 | 0.001321 | 0.004932 | Down |
| DENND6A-DT | 4.208712979 | -1.525464473 | 0.281608 | -5.41698 | 6.06E-08 | 6.18E-07 | Down |
| RPL32P26 | 1.156683717 | -1.367828783 | 0.502939 | -2.71967 | 0.006535 | 0.019601 | Down |
| AKAP2 | 94.25297282 | -1.451205753 | 0.270113 | -5.37259 | 7.76E-08 | 7.73E-07 | Down |
| WWTR1-IT1 | 5.173126439 | -1.622134441 | 0.280932 | -5.77411 | 7.74E-09 | 9.19E-08 | Down |
| AC112493.1 | 3.305034557 | -1.366278802 | 0.333893 | -4.09196 | 4.28E-05 | 0.000237 | Down |
| AL160314.1 | 1.614822979 | -1.453313067 | 0.401422 | -3.62041 | 0.000294 | 0.001321 | Down |
| SLC5A4-AS1 | 2.72041984 | -1.199281165 | 0.434054 | -2.76298 | 0.005728 | 0.017529 | Down |
| AC068647.1 | 1.521208839 | -1.617941573 | 0.563037 | -2.8736 | 0.004058 | 0.013046 | Down |
| LINC02657 | 1.541650787 | 2.922418275 | 0.487997 | 5.988597 | 2.12E-09 | 2.79E-08 | Up |
| AC017015.2 | 4.186423031 | -1.442358232 | 0.312219 | -4.6197 | 3.84E-06 | 2.71E-05 | Down |
| LINC02082 | 13.02546296 | 4.625385813 | 0.505066 | 9.157987 | 5.29E-20 | 3.12E-18 | Up |
| AC074051.1 | 0.612924966 | -1.313391268 | 0.547968 | -2.39684 | 0.016537 | 0.042778 | Down |
| ZBTB20-AS5 | 2.50203017 | -1.452380231 | 0.428745 | -3.38752 | 0.000705 | 0.002848 | Down |
| AL049634.1 | 1.271341192 | 1.338120314 | 0.524545 | 2.551012 | 0.010741 | 0.029842 | Up |
| RPS12P31 | 1.676749028 | -1.825571382 | 0.449858 | -4.05811 | 4.95E-05 | 0.000271 | Down |
| RPL12P30 | 20.55414923 | -2.036572775 | 0.250648 | -8.12522 | 4.47E-16 | 1.61E-14 | Down |
| PCDHGC4 | 18.67670771 | 1.659056437 | 0.260428 | 6.370491 | 1.88E-10 | 2.98E-09 | Up |
| LINC01206 | 1.42700178 | -1.883946833 | 0.779089 | -2.41814 | 0.0156 | 0.040731 | Down |
| POU5F1P6 | 4.554080817 | -1.075584019 | 0.338682 | -3.17579 | 0.001494 | 0.005493 | Down |
| AC073288.2 | 3.090910111 | 1.173214337 | 0.279214 | 4.201843 | 2.65E-05 | 0.000154 | Up |
| RPS29P11 | 29.72092145 | 4.77354526 | 0.430373 | 11.09164 | 1.38E-28 | 2.00E-26 | Up |
| AC107464.1 | 6.892974473 | -1.163488222 | 0.32137 | -3.62041 | 0.000294 | 0.001321 | Down |
| RN7SL806P | 1.402207514 | -1.564367959 | 0.407854 | -3.83561 | 0.000125 | 0.000619 | Down |
| UQCRHP4 | 1.097533028 | -1.709166616 | 0.528087 | -3.23653 | 0.00121 | 0.004567 | Down |
| RPL23AP81 | 5.991932408 | -1.071788148 | 0.237329 | -4.51605 | 6.30E-06 | 4.23E-05 | Down |
| AL445430.2 | 3.892631443 | -1.958103598 | 0.353878 | -5.53328 | 3.14E-08 | 3.35E-07 | Down |
| RN7SL812P | 2.092557342 | -1.152642136 | 0.312151 | -3.69258 | 0.000222 | 0.001026 | Down |
| AADACL2-AS1 | 5.287013141 | -2.225206467 | 0.446281 | -4.98611 | 6.16E-07 | 5.14E-06 | Down |
| ERVW-1 | 16.74306849 | -1.27598009 | 0.287387 | -4.43994 | 9.00E-06 | 5.85E-05 | Down |
| AC096992.1 | 1.161849811 | -1.752455525 | 0.492018 | -3.56177 | 0.000368 | 0.001611 | Down |
| AL591242.1 | 2.630975529 | -1.317462629 | 0.313037 | -4.20865 | 2.57E-05 | 0.000149 | Down |
| MTAPP1 | 2.441829593 | -1.181188385 | 0.379994 | -3.10844 | 0.001881 | 0.006699 | Down |
| AC107029.1 | 2.895244945 | -1.289657787 | 0.348168 | -3.70413 | 0.000212 | 0.000986 | Down |
| PSG4 | 7.266726985 | 3.29500178 | 0.719099 | 4.582125 | 4.60E-06 | 3.19E-05 | Up |
| AC079760.2 | 9.6225884 | 1.465264585 | 0.259993 | 5.635778 | 1.74E-08 | 1.95E-07 | Up |
| AC106707.1 | 0.77537256 | -2.463086083 | 0.737162 | -3.34131 | 0.000834 | 0.003301 | Down |
| CCDC39-AS1 | 2.468482261 | -1.62791896 | 0.315174 | -5.16514 | 2.40E-07 | 2.18E-06 | Down |
| AC006387.1 | 1.391596112 | -1.338829424 | 0.463564 | -2.88812 | 0.003875 | 0.012533 | Down |
| AC006159.2 | 4.182337567 | 2.23192704 | 0.460223 | 4.849662 | 1.24E-06 | 9.66E-06 | Up |
| PCDHAC2 | 111.2229798 | 1.701819926 | 0.268872 | 6.329489 | 2.46E-10 | 3.83E-09 | Up |
| AC073130.2 | 36.77700823 | -1.365093406 | 0.21515 | -6.34485 | 2.23E-10 | 3.49E-09 | Down |
| RPS6P16 | 2.178163587 | -1.002145769 | 0.357652 | -2.80201 | 0.005079 | 0.01583 | Down |
| VSIG8 | 44.59543695 | -1.21546054 | 0.283768 | -4.28328 | 1.84E-05 | 0.000111 | Down |
| POM121L6P | 0.530254773 | -1.696495199 | 0.518907 | -3.26936 | 0.001078 | 0.004131 | Down |
| FGF14-IT1 | 3.212307523 | -1.054000065 | 0.316412 | -3.3311 | 0.000865 | 0.00341 | Down |
| AC083982.1 | 1.720112231 | -2.66154415 | 0.552777 | -4.81486 | 1.47E-06 | 1.13E-05 | Down |
| AC119424.1 | 11.85491297 | -1.231730123 | 0.305164 | -4.03629 | 5.43E-05 | 0.000294 | Down |
| OR7E29P | 4.376576862 | -1.032514229 | 0.277784 | -3.71696 | 0.000202 | 0.000944 | Down |
| PALM2 | 35.01868667 | -1.355413406 | 0.258966 | -5.23393 | 1.66E-07 | 1.55E-06 | Down |
| C4orf48 | 257.7517093 | 1.942851372 | 0.371639 | 5.227786 | 1.72E-07 | 1.60E-06 | Up |
| RPL7P51 | 1.233029645 | -1.995855806 | 0.789418 | -2.52826 | 0.011463 | 0.031495 | Down |
| RPS23P1 | 15.29422036 | -1.93691658 | 0.232218 | -8.34095 | 7.37E-17 | 2.96E-15 | Down |
| AC108729.2 | 1.963205428 | -1.175052777 | 0.367796 | -3.19485 | 0.001399 | 0.005187 | Down |
| WFDC6 | 3.915689706 | -3.166192537 | 0.508381 | -6.22799 | 4.72E-10 | 6.96E-09 | Down |
| AC020779.1 | 1.466247933 | -1.53966721 | 0.412826 | -3.72958 | 0.000192 | 0.000902 | Down |
| CFB | 558.977063 | 1.389533503 | 0.336081 | 4.134514 | 3.56E-05 | 0.0002 | Up |
| RPLP0P2 | 21.94876737 | 1.945714808 | 0.252306 | 7.711728 | 1.24E-14 | 3.74E-13 | Up |
| WDR86-AS1 | 777.7278932 | 1.015317274 | 0.158822 | 6.392796 | 1.63E-10 | 2.61E-09 | Up |
| GSTA1 | 4.621228792 | 1.207528146 | 0.497762 | 2.425917 | 0.01527 | 0.040055 | Up |
| PARAL1 | 5.967080818 | 4.367301136 | 0.590111 | 7.400815 | 1.35E-13 | 3.50E-12 | Up |
| RPL35P6 | 1.508241444 | 1.241018664 | 0.401544 | 3.090614 | 0.001997 | 0.007054 | Up |
| RPS2P24 | 0.810605105 | 1.212040325 | 0.471348 | 2.571432 | 0.010128 | 0.028404 | Up |
| IL12A-AS1 | 8.864919187 | 2.006826903 | 0.323725 | 6.199178 | 5.68E-10 | 8.22E-09 | Up |
| RN7SL417P | 2.073624446 | -1.102509133 | 0.350039 | -3.14967 | 0.001635 | 0.00593 | Down |
| AC093278.1 | 2.634279501 | -1.572027063 | 0.353419 | -4.44806 | 8.67E-06 | 5.66E-05 | Down |
| RN7SL659P | 0.552884066 | 2.10890903 | 0.601817 | 3.504235 | 0.000458 | 0.001954 | Up |
| ATP1B3-AS1 | 12.40038787 | -1.055449364 | 0.34848 | -3.02873 | 0.002456 | 0.008444 | Down |
| AC004889.1 | 143.5341192 | -1.321374857 | 0.197762 | -6.68163 | 2.36E-11 | 4.37E-10 | Down |
| FOXP1-AS1 | 3.750433115 | -1.491376778 | 0.262351 | -5.68466 | 1.31E-08 | 1.50E-07 | Down |
| IFITM10 | 134.7591658 | 1.242384392 | 0.265865 | 4.672996 | 2.97E-06 | 2.15E-05 | Up |
| AL589743.1 | 2.327992835 | 1.887594587 | 0.389387 | 4.847609 | 1.25E-06 | 9.75E-06 | Up |
| LY6G6D | 1.625984658 | -1.344011697 | 0.492167 | -2.7308 | 0.006318 | 0.019059 | Down |
| RN7SL251P | 3.212775987 | -1.58518103 | 0.418668 | -3.78625 | 0.000153 | 0.000741 | Down |
| ETV5 | 2930.426839 | 1.628960082 | 0.157181 | 10.36362 | 3.63E-25 | 3.71E-23 | Up |
| AC107029.2 | 5.639400023 | -1.668618958 | 0.329849 | -5.05873 | 4.22E-07 | 3.63E-06 | Down |
| OR2A1-AS1 | 644.8197746 | -1.025531085 | 0.159275 | -6.43875 | 1.20E-10 | 1.98E-09 | Down |
| SLC9A9-AS2 | 3.189788079 | -1.01787987 | 0.32587 | -3.12358 | 0.001787 | 0.006403 | Down |
| LINC01213 | 6.744747 | -1.234103473 | 0.262002 | -4.71027 | 2.47E-06 | 1.81E-05 | Down |
| PTCHD4 | 436.8974485 | 2.947553788 | 0.234344 | 12.57792 | 2.79E-36 | 7.88E-34 | Up |
| HBB | 7817.580491 | -1.571348373 | 0.297133 | -5.28837 | 1.23E-07 | 1.18E-06 | Down |
| LIFR-AS1 | 485.052315 | -1.157946991 | 0.170362 | -6.79698 | 1.07E-11 | 2.09E-10 | Down |
| AC092718.1 | 17.04520746 | -1.06938303 | 0.152378 | -7.01798 | 2.25E-12 | 4.91E-11 | Down |
| MIR3150BHG | 4.261329613 | -1.596591286 | 0.51688 | -3.0889 | 0.002009 | 0.007085 | Down |
| SMARCA5-AS1 | 1.918603976 | -1.260479752 | 0.346555 | -3.63717 | 0.000276 | 0.001247 | Down |
| AC105285.1 | 116.3504366 | 1.423159793 | 0.124402 | 11.44001 | 2.64E-30 | 4.63E-28 | Up |
| AC096564.1 | 9.319273591 | -1.451108324 | 0.415887 | -3.48919 | 0.000484 | 0.002053 | Down |
| AP005717.1 | 49.15836951 | -1.096124004 | 0.178602 | -6.13725 | 8.40E-10 | 1.18E-08 | Down |
| CXXC4-AS1 | 5.732777362 | -1.126917807 | 0.243054 | -4.63649 | 3.54E-06 | 2.51E-05 | Down |
| FAM111A-DT | 557.5059582 | 1.159615096 | 0.111649 | 10.38623 | 2.86E-25 | 2.99E-23 | Up |
| AC083805.1 | 9.700188802 | 1.020374746 | 0.233478 | 4.370324 | 1.24E-05 | 7.81E-05 | Up |
| LINC02211 | 2.955624309 | -2.204122159 | 0.582469 | -3.7841 | 0.000154 | 0.000746 | Down |
| AC008659.1 | 3.174577742 | -1.647850518 | 0.314968 | -5.2318 | 1.68E-07 | 1.56E-06 | Down |
| NADK2-AS1 | 18.45491441 | -1.132184928 | 0.263007 | -4.30476 | 1.67E-05 | 0.000102 | Down |
| LINC01096 | 2.382386976 | 1.708617648 | 0.466567 | 3.662102 | 0.00025 | 0.001143 | Up |
| LINC00900 | 211.6948946 | 1.250203333 | 0.143316 | 8.723375 | 2.70E-18 | 1.28E-16 | Up |
| LINC02458 | 3.969086867 | 1.828389744 | 0.342793 | 5.333804 | 9.62E-08 | 9.41E-07 | Up |
| AC073911.1 | 4.788101939 | -1.360096527 | 0.24235 | -5.61212 | 2.00E-08 | 2.21E-07 | Down |
| LINC002481 | 35.29117948 | 1.519018821 | 0.251469 | 6.040574 | 1.54E-09 | 2.07E-08 | Up |
| PLA2G4E-AS1 | 13.68764101 | -1.065734748 | 0.190968 | -5.5807 | 2.40E-08 | 2.61E-07 | Down |
| ZCCHC23 | 3.666812987 | -1.009946473 | 0.305369 | -3.3073 | 0.000942 | 0.003673 | Down |
| AC012377.1 | 3.801740579 | -1.145172178 | 0.230469 | -4.96889 | 6.73E-07 | 5.57E-06 | Down |
| MIR210HG | 88.55187074 | -1.157277441 | 0.166783 | -6.9388 | 3.95E-12 | 8.31E-11 | Down |
| LY6E-DT | 24.00603214 | 1.090360985 | 0.211242 | 5.161678 | 2.45E-07 | 2.22E-06 | Up |
| AP000802.1 | 46.67583392 | -1.860669027 | 0.256884 | -7.24323 | 4.38E-13 | 1.06E-11 | Down |
| LRP4-AS1 | 23.70848716 | 4.208600839 | 0.304489 | 13.82183 | 1.88E-43 | 1.26E-40 | Up |
| AC008609.1 | 0.384201478 | 1.974571161 | 0.773654 | 2.552267 | 0.010702 | 0.029757 | Up |
| AC068446.2 | 1.245226199 | -2.311697189 | 0.560367 | -4.12532 | 3.70E-05 | 0.000208 | Down |
| SNCA-AS1 | 1.599816057 | -2.174460842 | 0.451656 | -4.81441 | 1.48E-06 | 1.13E-05 | Down |
| AC021086.1 | 4.517023737 | -1.045054227 | 0.252132 | -4.14487 | 3.40E-05 | 0.000192 | Down |
| LINC00926 | 263.1274275 | -1.643627328 | 0.416809 | -3.94336 | 8.03E-05 | 0.000416 | Down |
| INSL3 | 15.12376415 | 1.244357118 | 0.468755 | 2.6546 | 0.00794 | 0.023163 | Up |
| AC008443.3 | 1.545882971 | -1.206854657 | 0.375952 | -3.21013 | 0.001327 | 0.004953 | Down |
| AC067942.2 | 2.067220549 | -1.049622288 | 0.331812 | -3.1633 | 0.00156 | 0.005703 | Down |
| ADH1C | 47.0051986 | -2.036603307 | 0.262055 | -7.77168 | 7.75E-15 | 2.41E-13 | Down |
| LINC02268 | 4.95834595 | -1.659797573 | 0.394954 | -4.20251 | 2.64E-05 | 0.000153 | Down |
| AC092658.1 | 7.639844093 | -1.363220177 | 0.281938 | -4.83518 | 1.33E-06 | 1.03E-05 | Down |
| AC074194.1 | 2.292436088 | -1.57124469 | 0.356016 | -4.41342 | 1.02E-05 | 6.53E-05 | Down |
| AC023794.1 | 4.572893824 | 1.383054524 | 0.267431 | 5.17164 | 2.32E-07 | 2.11E-06 | Up |
| AC106868.1 | 0.461014221 | 1.43993853 | 0.556925 | 2.585515 | 0.009723 | 0.027474 | Up |
| TNXA | 16.47482852 | 1.086125993 | 0.306712 | 3.541189 | 0.000398 | 0.001725 | Up |
| TUB-AS1 | 1.989063072 | -1.62338087 | 0.437445 | -3.71105 | 0.000206 | 0.000962 | Down |
| AC096577.1 | 1.769674502 | -1.89658718 | 0.758145 | -2.50162 | 0.012363 | 0.033587 | Down |
| AC073358.1 | 0.538618697 | 1.323337714 | 0.528339 | 2.504711 | 0.012255 | 0.033341 | Up |
| LINC02469 | 1.041880285 | 1.545216849 | 0.388259 | 3.979861 | 6.90E-05 | 0.000363 | Up |
| AC100850.1 | 0.503642133 | 2.678771309 | 0.815326 | 3.285523 | 0.001018 | 0.003933 | Up |
| FGF10-AS1 | 3.22887913 | -1.71121498 | 0.31083 | -5.5053 | 3.69E-08 | 3.89E-07 | Down |
| AC098827.1 | 2.878766273 | -1.256826911 | 0.303366 | -4.14294 | 3.43E-05 | 0.000194 | Down |
| AC107027.1 | 5.749853741 | -1.094153703 | 0.234908 | -4.65779 | 3.20E-06 | 2.30E-05 | Down |
| AC104137.1 | 17.02162226 | -2.6328075 | 0.347746 | -7.57107 | 3.70E-14 | 1.03E-12 | Down |
| AC005351.1 | 0.883038868 | -1.670130574 | 0.669939 | -2.49296 | 0.012668 | 0.034253 | Down |
| AC008443.4 | 18.21743237 | 1.230013625 | 0.282864 | 4.348433 | 1.37E-05 | 8.53E-05 | Up |
| LINC02058 | 0.818971887 | -1.440248478 | 0.582584 | -2.47217 | 0.01343 | 0.035988 | Down |
| AC022784.1 | 10.03421442 | 1.499341586 | 0.302364 | 4.958725 | 7.10E-07 | 5.84E-06 | Up |
| AC010931.2 | 13.62207341 | 1.223283741 | 0.241047 | 5.074867 | 3.88E-07 | 3.37E-06 | Up |
| AC092436.2 | 0.641707186 | 2.887837882 | 0.68713 | 4.202752 | 2.64E-05 | 0.000153 | Up |
| AC008620.2 | 4.058587645 | -3.91098587 | 0.656894 | -5.95375 | 2.62E-09 | 3.40E-08 | Down |
| AC079140.1 | 2.265570091 | -1.161937978 | 0.291036 | -3.99241 | 6.54E-05 | 0.000347 | Down |
| AC023794.2 | 2.105423271 | -1.149307843 | 0.323084 | -3.5573 | 0.000375 | 0.001635 | Down |
| GDNF-AS1 | 0.736617785 | 2.440176789 | 0.554736 | 4.39881 | 1.09E-05 | 6.93E-05 | Up |
| AC133963.1 | 1.411100135 | 2.198872093 | 0.378008 | 5.817004 | 5.99E-09 | 7.26E-08 | Up |
| HAS2-AS1 | 27.43032412 | 1.197182739 | 0.295194 | 4.055582 | 5.00E-05 | 0.000273 | Up |
| AC079594.2 | 0.872844711 | 1.197039774 | 0.418935 | 2.857341 | 0.004272 | 0.013646 | Up |
| ACTN3 | 8.931943179 | -1.239496724 | 0.322278 | -3.84604 | 0.00012 | 0.000596 | Down |
| LINC02115 | 1.311153605 | 1.687052403 | 0.422597 | 3.992104 | 6.55E-05 | 0.000347 | Up |
| AC008883.1 | 0.522338022 | -1.617913593 | 0.600476 | -2.69438 | 0.007052 | 0.020938 | Down |
| AC093297.1 | 1.024715179 | -1.954659725 | 0.491376 | -3.97793 | 6.95E-05 | 0.000366 | Down |
| C8orf34-AS1 | 9.296194262 | 1.6574434 | 0.303844 | 5.454919 | 4.90E-08 | 5.07E-07 | Up |
| AC078850.2 | 17.3588375 | -1.215994001 | 0.297211 | -4.09135 | 4.29E-05 | 0.000238 | Down |
| LINC02432 | 160.141891 | -1.585953145 | 0.215897 | -7.34589 | 2.04E-13 | 5.19E-12 | Down |
| AP003555.1 | 0.535024136 | -1.396706874 | 0.587509 | -2.37734 | 0.017438 | 0.044678 | Down |
| AC006427.1 | 0.54776719 | -1.346949536 | 0.560172 | -2.40453 | 0.016193 | 0.042017 | Down |
| AC097376.1 | 4.900739251 | -1.501822445 | 0.265304 | -5.66077 | 1.51E-08 | 1.70E-07 | Down |
| LINC02511 | 2.19152356 | -2.763453932 | 0.440425 | -6.27452 | 3.51E-10 | 5.31E-09 | Down |
| HHIP-AS1 | 32.62863047 | -1.028555522 | 0.214267 | -4.80035 | 1.58E-06 | 1.21E-05 | Down |
| AC131254.1 | 2.815508001 | 1.530523927 | 0.532947 | 2.871812 | 0.004081 | 0.013111 | Up |
| AL133372.2 | 0.755866786 | -1.730480224 | 0.531357 | -3.25672 | 0.001127 | 0.00429 | Down |
| AC126768.2 | 9.429044631 | -1.901441902 | 0.301305 | -6.31068 | 2.78E-10 | 4.28E-09 | Down |
| AC093895.1 | 2.094785698 | 3.396638976 | 0.495516 | 6.854758 | 7.14E-12 | 1.44E-10 | Up |
| AL691482.3 | 6.084206196 | 2.152266567 | 0.44016 | 4.88974 | 1.01E-06 | 8.05E-06 | Up |
| AC104819.1 | 1.409061266 | -1.219505021 | 0.462068 | -2.63923 | 0.008309 | 0.02407 | Down |
| AC093599.1 | 0.621219279 | -1.613114559 | 0.563316 | -2.8636 | 0.004189 | 0.013411 | Down |
| AC093903.1 | 4.79584851 | -1.212334335 | 0.430673 | -2.81498 | 0.004878 | 0.015282 | Down |
| AC097537.1 | 1.294377938 | -2.142582259 | 0.548711 | -3.90476 | 9.43E-05 | 0.000479 | Down |
| AC093821.1 | 0.397584111 | 1.657252874 | 0.653517 | 2.535899 | 0.011216 | 0.03091 | Up |
| SUDS3P1 | 39.97310857 | -1.023229123 | 0.185517 | -5.51556 | 3.48E-08 | 3.68E-07 | Down |
| PSD2-AS1 | 0.45167156 | 1.536140671 | 0.600485 | 2.558166 | 0.010523 | 0.029327 | Up |
| EPPIN-WFDC6 | 0.808869069 | -2.193599126 | 0.69551 | -3.15394 | 0.001611 | 0.005861 | Down |
| PRDX2P3 | 1.812952113 | -1.118463293 | 0.36069 | -3.1009 | 0.001929 | 0.006847 | Down |
| AC105389.2 | 7.611819123 | -1.032961859 | 0.240964 | -4.28678 | 1.81E-05 | 0.000109 | Down |
| BCL9P1 | 0.766351246 | -1.499335202 | 0.537404 | -2.78996 | 0.005271 | 0.016329 | Down |
| LINC02265 | 5.676081089 | -1.557622148 | 0.318653 | -4.88814 | 1.02E-06 | 8.11E-06 | Down |
| LINC02405 | 1.327665198 | -1.385276944 | 0.567577 | -2.44068 | 0.014659 | 0.038728 | Down |
| AC093274.1 | 1.689047564 | -1.07366247 | 0.377459 | -2.84445 | 0.004449 | 0.014126 | Down |
| CASC9 | 9.74551012 | -3.211630656 | 0.635957 | -5.05007 | 4.42E-07 | 3.79E-06 | Down |
| AC015909.1 | 1.364716754 | 3.259498217 | 0.479459 | 6.798279 | 1.06E-11 | 2.07E-10 | Up |
| NAIP | 73.47938229 | -1.125167638 | 0.184683 | -6.09243 | 1.11E-09 | 1.53E-08 | Down |
| AC092834.1 | 0.849533152 | -1.718657708 | 0.501329 | -3.4282 | 0.000608 | 0.00251 | Down |
| AC096711.1 | 0.854803143 | -2.230935342 | 0.786788 | -2.8355 | 0.004575 | 0.014474 | Down |
| LINC01470 | 0.755348081 | -1.651710511 | 0.509414 | -3.24237 | 0.001185 | 0.004483 | Down |
| ZEB2P1 | 2.006162585 | -1.86100722 | 0.456358 | -4.07795 | 4.54E-05 | 0.000251 | Down |
| TMEM251P1 | 0.925646157 | -2.120963317 | 0.887262 | -2.39046 | 0.016827 | 0.043374 | Down |
| LINC01258 | 1.675918671 | -1.827833121 | 0.455541 | -4.01245 | 6.01E-05 | 0.000321 | Down |
| BMPR1B-DT | 0.707820348 | -2.325665784 | 0.768211 | -3.02738 | 0.002467 | 0.008475 | Down |
| AC096564.2 | 1.962699049 | -1.160826148 | 0.348034 | -3.33538 | 0.000852 | 0.003365 | Down |
| AC080188.1 | 4.750919874 | -1.264081718 | 0.311977 | -4.05184 | 5.08E-05 | 0.000277 | Down |
| AC010255.2 | 8.37907912 | -2.093294716 | 0.399287 | -5.24258 | 1.58E-07 | 1.48E-06 | Down |
| AC079858.1 | 1.170450796 | -1.704622573 | 0.564826 | -3.01796 | 0.002545 | 0.008703 | Down |
| LINC00942 | 12.43721676 | 1.690588393 | 0.260788 | 6.482625 | 9.01E-11 | 1.51E-09 | Up |
| AC106772.1 | 0.985529622 | -1.285943258 | 0.48757 | -2.63746 | 0.008353 | 0.024165 | Down |
| AC106897.1 | 26.11747662 | -1.467102043 | 0.230682 | -6.35984 | 2.02E-10 | 3.19E-09 | Down |
| AC106795.2 | 244.1128122 | -1.105994144 | 0.234304 | -4.72033 | 2.35E-06 | 1.74E-05 | Down |
| AC026369.1 | 12.0592652 | 2.467546784 | 0.284682 | 8.66772 | 4.41E-18 | 2.03E-16 | Up |
| AC110760.2 | 11.75984909 | -1.517736928 | 0.260699 | -5.8218 | 5.82E-09 | 7.08E-08 | Down |
| LINC02242 | 3.229240214 | -1.60953768 | 0.487648 | -3.30062 | 0.000965 | 0.003752 | Down |
| AC116345.1 | 2.844891226 | 3.202278998 | 0.592469 | 5.404977 | 6.48E-08 | 6.56E-07 | Up |
| LINC02112 | 1.103238313 | -1.470925623 | 0.51028 | -2.88258 | 0.003944 | 0.012725 | Down |
| LINC00964 | 4.849468281 | 1.224276986 | 0.3323 | 3.684257 | 0.000229 | 0.001057 | Up |
| AC012636.1 | 19.32186148 | 1.36968328 | 0.254246 | 5.387242 | 7.15E-08 | 7.18E-07 | Up |
| AC116337.3 | 0.910819954 | 1.195097215 | 0.460906 | 2.592928 | 0.009516 | 0.026991 | Up |
| AC090833.1 | 8.616722252 | -1.443681028 | 0.248224 | -5.81603 | 6.03E-09 | 7.30E-08 | Down |
| AC005920.2 | 2.311566473 | -2.268578495 | 0.407065 | -5.57302 | 2.50E-08 | 2.72E-07 | Down |
| ALG1L11P | 1.62335853 | -1.651781133 | 0.4239 | -3.89663 | 9.75E-05 | 0.000495 | Down |
| AC006487.1 | 2.17344368 | 2.283093677 | 0.398307 | 5.731996 | 9.93E-09 | 1.16E-07 | Up |
| BRD9P2 | 3.953087595 | -1.476075502 | 0.327682 | -4.50459 | 6.65E-06 | 4.45E-05 | Down |
| AC117500.1 | 15.94041125 | -2.069937383 | 0.319623 | -6.47618 | 9.41E-11 | 1.57E-09 | Down |
| GBA3 | 41.963747 | -1.176552388 | 0.25484 | -4.61683 | 3.90E-06 | 2.74E-05 | Down |
| CCT7P2 | 0.591696454 | -1.582956079 | 0.535479 | -2.95615 | 0.003115 | 0.010372 | Down |
| TMEM158 | 43.41513484 | 1.066062381 | 0.300682 | 3.545485 | 0.000392 | 0.001701 | Up |
| BFSP2-AS1 | 4.132005161 | -1.39278034 | 0.451317 | -3.08603 | 0.002028 | 0.007146 | Down |
| AC122138.1 | 0.598934744 | -1.481483521 | 0.61098 | -2.42477 | 0.015318 | 0.040165 | Down |
| AC096719.1 | 4.206584636 | -1.609970502 | 0.2785 | -5.78086 | 7.43E-09 | 8.85E-08 | Down |
| AC114316.1 | 0.885163888 | -2.08783215 | 0.532628 | -3.91987 | 8.86E-05 | 0.000453 | Down |
| AC104827.1 | 1.877488947 | -1.151812008 | 0.394781 | -2.9176 | 0.003527 | 0.01155 | Down |
| AC093248.1 | 2.007243517 | 1.091810875 | 0.296897 | 3.677411 | 0.000236 | 0.001082 | Up |
| AC011379.1 | 4.548906324 | -1.118052086 | 0.29774 | -3.75513 | 0.000173 | 0.000826 | Down |
| AC025176.1 | 1.996315683 | 1.819729644 | 0.475864 | 3.824053 | 0.000131 | 0.000646 | Up |
| AC106795.3 | 6.854917101 | -1.864598819 | 0.286475 | -6.50877 | 7.58E-11 | 1.29E-09 | Down |
| AK3P2 | 4.735614324 | -1.979282074 | 0.302851 | -6.53549 | 6.34E-11 | 1.09E-09 | Down |
| LINC02232 | 64.71326806 | -1.339082533 | 0.430828 | -3.10816 | 0.001883 | 0.006704 | Down |
| AC116424.1 | 1.757146969 | -1.361373479 | 0.374838 | -3.63189 | 0.000281 | 0.00127 | Down |
| HOXC-AS2 | 1.007719742 | 1.407604352 | 0.496547 | 2.834785 | 0.004586 | 0.014498 | Up |
| AC093607.1 | 2.570486421 | -1.699483523 | 0.422039 | -4.02684 | 5.65E-05 | 0.000305 | Down |
| AC026801.1 | 0.739059402 | -2.181776981 | 0.599886 | -3.63698 | 0.000276 | 0.001247 | Down |
| LINC02276 | 10.90061388 | -1.541059742 | 0.223709 | -6.88869 | 5.63E-12 | 1.15E-10 | Down |
| AC099509.2 | 0.863141167 | -1.687704003 | 0.552398 | -3.05523 | 0.002249 | 0.007816 | Down |
| AC034206.1 | 3.370766345 | 3.056249529 | 0.534724 | 5.715563 | 1.09E-08 | 1.27E-07 | Up |
| AC097504.1 | 1.247273273 | -1.890847471 | 0.507271 | -3.72749 | 0.000193 | 0.000909 | Down |
| CIR1P2 | 1.696418535 | -1.831127424 | 0.451251 | -4.05789 | 4.95E-05 | 0.000271 | Down |
| ALDH1L1-AS1 | 0.718637635 | -1.57215108 | 0.543586 | -2.89218 | 0.003826 | 0.012395 | Down |
| AP002754.1 | 11.1194566 | 1.92557344 | 0.291371 | 6.608659 | 3.88E-11 | 6.94E-10 | Up |
| AC025754.1 | 0.592952606 | 1.416165689 | 0.467099 | 3.031833 | 0.002431 | 0.008368 | Up |
| AC008840.1 | 9.492006357 | 1.19964414 | 0.180894 | 6.631744 | 3.32E-11 | 5.99E-10 | Up |
| AC105383.1 | 2.088444195 | -1.399321908 | 0.423694 | -3.30267 | 0.000958 | 0.003727 | Down |
| AC110296.1 | 4.112819171 | -2.421622254 | 0.328454 | -7.37279 | 1.67E-13 | 4.28E-12 | Down |
| LINC02430 | 1.840905538 | -1.188871572 | 0.380063 | -3.12809 | 0.001759 | 0.006321 | Down |
| AC068647.2 | 5.464683938 | -1.951764443 | 0.463604 | -4.20998 | 2.55E-05 | 0.000149 | Down |
| AC034199.1 | 3.404347616 | 1.354954131 | 0.361809 | 3.744942 | 0.00018 | 0.000855 | Up |
| AC026124.1 | 1.273504544 | -1.397726339 | 0.504066 | -2.7729 | 0.005556 | 0.017067 | Down |
| TRMT9B | 6544.849981 | -1.239143078 | 0.156994 | -7.89293 | 2.95E-15 | 9.62E-14 | Down |
| AC113383.1 | 4.46610833 | 1.637361379 | 0.247868 | 6.605792 | 3.95E-11 | 7.07E-10 | Up |
| AC022101.1 | 1.118725731 | -1.436765161 | 0.502619 | -2.85855 | 0.004256 | 0.013599 | Down |
| PURPL | 4.955315731 | 2.795010304 | 0.488346 | 5.723426 | 1.04E-08 | 1.21E-07 | Up |
| STK32A-AS1 | 49.79939155 | 2.648666945 | 0.245681 | 10.78094 | 4.24E-27 | 5.39E-25 | Up |
| AC069272.1 | 1.005448847 | -1.133819542 | 0.471936 | -2.40248 | 0.016284 | 0.042223 | Down |
| AC108935.1 | 0.544556363 | -1.726641276 | 0.593438 | -2.90956 | 0.003619 | 0.011807 | Down |
| AC008808.2 | 1.242915369 | -1.992696011 | 0.524512 | -3.79914 | 0.000145 | 0.000707 | Down |
| AC008592.1 | 0.804964489 | 1.450638338 | 0.438859 | 3.305477 | 0.000948 | 0.003694 | Up |
| TUNAR | 16.00708465 | 3.172498376 | 0.648844 | 4.889462 | 1.01E-06 | 8.06E-06 | Up |
| AC114296.1 | 3.433355934 | 3.04980551 | 0.514238 | 5.930722 | 3.02E-09 | 3.87E-08 | Up |
| OR7E35P | 0.700567874 | 1.790004636 | 0.555476 | 3.222468 | 0.001271 | 0.004768 | Up |
| HOXC-AS1 | 1.216354674 | 1.358646598 | 0.413461 | 3.286036 | 0.001016 | 0.003927 | Up |
| AC122718.1 | 9.204979146 | -1.305117305 | 0.302062 | -4.3207 | 1.56E-05 | 9.53E-05 | Down |
| DUTP7 | 0.521784925 | -1.360228845 | 0.554022 | -2.45519 | 0.014081 | 0.037443 | Down |
| PPM1AP1 | 2.001190248 | 3.058988967 | 0.402884 | 7.592729 | 3.13E-14 | 8.82E-13 | Up |
| GPR162 | 124.0223919 | 1.536427409 | 0.153959 | 9.979441 | 1.88E-23 | 1.62E-21 | Up |
| LINC02125 | 0.704177274 | -1.703879327 | 0.566175 | -3.00946 | 0.002617 | 0.00892 | Down |
| AP002784.1 | 4.587411856 | -1.918372981 | 0.310026 | -6.18777 | 6.10E-10 | 8.78E-09 | Down |
| LINC01303 | 17.22501725 | -1.069906758 | 0.283105 | -3.77918 | 0.000157 | 0.000759 | Down |
| AC022424.1 | 1.222124462 | 1.022545678 | 0.433395 | 2.359382 | 0.018305 | 0.046491 | Up |
| LINC02492 | 1.213079324 | -2.503867053 | 0.58182 | -4.30351 | 1.68E-05 | 0.000102 | Down |
| AC055733.2 | 0.618751698 | 2.285267796 | 0.507571 | 4.502365 | 6.72E-06 | 4.49E-05 | Up |
| AC010210.1 | 178.7706893 | -1.071357638 | 0.177579 | -6.03313 | 1.61E-09 | 2.16E-08 | Down |
| AC093909.4 | 2.217098195 | -3.225403619 | 0.480326 | -6.71503 | 1.88E-11 | 3.53E-10 | Down |
| LY6G6F-LY6G6D | 3.587132903 | -1.515525792 | 0.367139 | -4.12793 | 3.66E-05 | 0.000206 | Down |
| AC068580.4 | 11.56160747 | 1.207519573 | 0.254876 | 4.737677 | 2.16E-06 | 1.61E-05 | Up |
| ST3GAL1P1 | 3.698817793 | -1.032624826 | 0.308155 | -3.351 | 0.000805 | 0.003205 | Down |
| AP001363.1 | 3.147003198 | -1.39368142 | 0.379283 | -3.67451 | 0.000238 | 0.001094 | Down |
| AC111000.4 | 2.398699749 | -2.69911983 | 0.524976 | -5.14141 | 2.73E-07 | 2.44E-06 | Down |
| CCDC169-SOHLH2 | 3.156386631 | -1.44453578 | 0.538424 | -2.6829 | 0.007299 | 0.021562 | Down |
| C8orf17 | 0.827938178 | -1.703794997 | 0.638802 | -2.66717 | 0.007649 | 0.022409 | Down |
| AC025419.1 | 38.62000563 | 4.862688005 | 0.338987 | 14.34476 | 1.15E-46 | 1.05E-43 | Up |
| AC106865.1 | 11.17079775 | 1.681689235 | 0.344515 | 4.881319 | 1.05E-06 | 8.37E-06 | Up |
| PRODH2 | 1.204862324 | -2.17448802 | 0.906374 | -2.39911 | 0.016435 | 0.042541 | Down |
| AC010255.3 | 3.376908761 | -1.956300157 | 0.558661 | -3.50176 | 0.000462 | 0.001969 | Down |
| AC074131.1 | 2.852717174 | -1.14231916 | 0.454901 | -2.51114 | 0.012034 | 0.032832 | Down |
| EPHA5-AS1 | 32.63203247 | -2.690121774 | 0.303992 | -8.84932 | 8.81E-19 | 4.43E-17 | Down |
| AC125807.2 | 385.680234 | -1.226269355 | 0.164367 | -7.46054 | 8.62E-14 | 2.29E-12 | Down |
| AC097467.3 | 4.787533139 | -1.083954983 | 0.269351 | -4.02432 | 5.71E-05 | 0.000307 | Down |
| AC008629.3 | 1.737040691 | -1.027599112 | 0.383437 | -2.67997 | 0.007363 | 0.021715 | Down |
| AC097658.2 | 1.152423935 | 1.471886708 | 0.39851 | 3.693479 | 0.000221 | 0.001023 | Up |
| LINC02600 | 37.12576026 | 1.981841706 | 0.345847 | 5.730402 | 1.00E-08 | 1.17E-07 | Up |
| AC244502.1 | 107.4792431 | 4.394067444 | 0.485741 | 9.046105 | 1.48E-19 | 8.35E-18 | Up |
| TMEM108-AS1 | 5.102640027 | 3.962277105 | 0.553912 | 7.153262 | 8.47E-13 | 1.98E-11 | Up |
| AC093281.2 | 1.711117356 | 2.315357426 | 0.42241 | 5.481299 | 4.22E-08 | 4.41E-07 | Up |
| AC097478.1 | 34.67058371 | 1.972725148 | 0.390977 | 5.045635 | 4.52E-07 | 3.87E-06 | Up |
| LINC01843 | 3.468493433 | 1.067411654 | 0.249917 | 4.271066 | 1.95E-05 | 0.000117 | Up |
| AC096741.1 | 1.873747094 | -1.785876602 | 0.398091 | -4.4861 | 7.25E-06 | 4.81E-05 | Down |
| TMEM92-AS1 | 3.074750743 | 3.364819152 | 0.453913 | 7.412919 | 1.24E-13 | 3.22E-12 | Up |
| AC010425.1 | 17.75721845 | -3.309626252 | 0.447069 | -7.40294 | 1.33E-13 | 3.45E-12 | Down |
| LINC00589 | 2.068371114 | 1.62227881 | 0.402608 | 4.029428 | 5.59E-05 | 0.000302 | Up |
| AL133330.1 | 11.66768591 | 1.086173488 | 0.270151 | 4.020615 | 5.80E-05 | 0.000312 | Up |
| AP001999.1 | 69.9487468 | -1.70481401 | 0.339085 | -5.02769 | 4.96E-07 | 4.22E-06 | Down |
| SLC25A15P3 | 6.778761566 | -2.462739324 | 0.401389 | -6.13554 | 8.49E-10 | 1.19E-08 | Down |
| AC021106.2 | 3.843416364 | -1.219096033 | 0.276588 | -4.40763 | 1.05E-05 | 6.69E-05 | Down |
| MTCYBP35 | 4.521189576 | 1.380837649 | 0.337326 | 4.093485 | 4.25E-05 | 0.000236 | Up |
| AC012625.1 | 1.361669229 | 3.031461076 | 0.516415 | 5.870206 | 4.35E-09 | 5.41E-08 | Up |
| LINC02315 | 13.28280384 | -3.185514746 | 0.41705 | -7.63821 | 2.20E-14 | 6.37E-13 | Down |
| AC099550.1 | 2.459925278 | -1.474764751 | 0.3315 | -4.44877 | 8.64E-06 | 5.64E-05 | Down |
| LINC00958 | 322.824434 | 1.127982117 | 0.240698 | 4.686296 | 2.78E-06 | 2.02E-05 | Up |
| AC008667.3 | 0.56259326 | 1.301775541 | 0.557335 | 2.335713 | 0.019506 | 0.049072 | Up |
| AC113378.1 | 2.582492963 | -1.136176381 | 0.364242 | -3.11929 | 0.001813 | 0.006486 | Down |
| AC016571.1 | 19.48680085 | -1.841262423 | 0.262334 | -7.01878 | 2.24E-12 | 4.88E-11 | Down |
| AC021151.1 | 0.59543662 | -1.574482696 | 0.585489 | -2.68917 | 0.007163 | 0.021224 | Down |
| AC135352.1 | 84.4207332 | -1.069516617 | 0.243714 | -4.38841 | 1.14E-05 | 7.24E-05 | Down |
| AL356488.1 | 4.3284179 | -1.130402944 | 0.256319 | -4.41015 | 1.03E-05 | 6.61E-05 | Down |
| OR7E28P | 13.57218415 | 1.664239575 | 0.357567 | 4.654347 | 3.25E-06 | 2.33E-05 | Up |
| IMPA1P1 | 0.894367031 | -1.693265776 | 0.496449 | -3.41075 | 0.000648 | 0.00265 | Down |
| AC108159.1 | 2.951558317 | -1.638527575 | 0.344681 | -4.75375 | 2.00E-06 | 1.49E-05 | Down |
| AC055717.1 | 0.800701693 | 2.453853735 | 0.989001 | 2.481144 | 0.013096 | 0.035233 | Up |
| AC005324.3 | 6.220461532 | -1.015664292 | 0.323446 | -3.14013 | 0.001689 | 0.006098 | Down |
| AC091887.1 | 2.620825372 | -1.213948239 | 0.299596 | -4.05195 | 5.08E-05 | 0.000277 | Down |
| HMGB1P35 | 0.832057268 | -1.498806776 | 0.523497 | -2.86307 | 0.004196 | 0.013427 | Down |
| LINC02106 | 0.739988409 | -1.925929792 | 0.656152 | -2.93519 | 0.003333 | 0.011006 | Down |
| LINC01267 | 3.474506868 | 2.205425038 | 0.354544 | 6.220462 | 4.96E-10 | 7.27E-09 | Up |
| HADHAP1 | 9.950902166 | -1.075017337 | 0.271296 | -3.96253 | 7.42E-05 | 0.000387 | Down |
| AC093916.1 | 2.771403876 | -2.355616092 | 0.524909 | -4.48767 | 7.20E-06 | 4.78E-05 | Down |
| AC116345.3 | 5.019171733 | -1.724445695 | 0.417555 | -4.12987 | 3.63E-05 | 0.000204 | Down |
| AL928654.1 | 176.0452985 | -1.064077337 | 0.129017 | -8.24756 | 1.62E-16 | 6.21E-15 | Down |
| AC136475.1 | 46.21965031 | -1.391261366 | 0.205534 | -6.76901 | 1.30E-11 | 2.50E-10 | Down |
| SNHG27 | 1.931601837 | -1.681166912 | 0.6172 | -2.72386 | 0.006452 | 0.019397 | Down |
| AC122718.2 | 3.316108454 | -1.555547953 | 0.418823 | -3.71409 | 0.000204 | 0.000953 | Down |
| RF00019.658 | 0.84071391 | -1.844251251 | 0.582173 | -3.16788 | 0.001536 | 0.005631 | Down |
| SNORD116-26 | 1.58532749 | -1.416471276 | 0.398687 | -3.55284 | 0.000381 | 0.00166 | Down |
| RNU7-71P | 1.131151337 | -1.05214613 | 0.404404 | -2.60172 | 0.009276 | 0.026421 | Down |
| RF00156.11 | 2.757965464 | -1.724918405 | 0.356924 | -4.83274 | 1.35E-06 | 1.04E-05 | Down |
| SNORD116-27 | 2.550038493 | -1.118098701 | 0.38501 | -2.90408 | 0.003683 | 0.011987 | Down |
| SCARNA5 | 5.018334276 | -1.033941272 | 0.407158 | -2.53941 | 0.011104 | 0.030657 | Down |
| RNU6-1262P | 3.056535472 | -1.660669737 | 0.349981 | -4.74503 | 2.08E-06 | 1.56E-05 | Down |
| RF00086 | 1.981697284 | -2.802326764 | 0.527165 | -5.31584 | 1.06E-07 | 1.03E-06 | Down |
| RNU6-720P | 1.304204773 | -1.769667421 | 0.459723 | -3.84942 | 0.000118 | 0.000589 | Down |
| RF00568.9 | 1.346965253 | -2.354959427 | 0.80439 | -2.92763 | 0.003416 | 0.011237 | Down |
| RNU7-3P | 2.279756457 | -1.096139457 | 0.334641 | -3.27557 | 0.001054 | 0.004054 | Down |
| RNA5SP470 | 2.152596052 | -2.018369514 | 0.376573 | -5.35984 | 8.33E-08 | 8.25E-07 | Down |
| RN7SKP21 | 0.559431736 | 1.234110819 | 0.52247 | 2.36207 | 0.018173 | 0.046233 | Up |
| RN7SKP83 | 0.738337016 | -1.430552536 | 0.521791 | -2.74162 | 0.006114 | 0.018543 | Down |
| RNU6-1061P | 1.566572145 | -1.272866415 | 0.390342 | -3.2609 | 0.001111 | 0.004236 | Down |
| RNA5SP434 | 0.632322418 | -1.267376033 | 0.532871 | -2.37839 | 0.017388 | 0.044575 | Down |
| RNA5SP466 | 1.036329124 | -2.071054453 | 0.678374 | -3.05297 | 0.002266 | 0.007861 | Down |
| RNU6ATAC36P | 1.024043383 | -1.779627466 | 0.656258 | -2.71178 | 0.006692 | 0.020014 | Down |
| RN7SKP272 | 1.484929028 | -1.344111209 | 0.408708 | -3.28868 | 0.001007 | 0.003895 | Down |
| RNA5SP290 | 0.801946236 | 1.900017611 | 0.579375 | 3.279424 | 0.00104 | 0.004006 | Up |
| SCARNA1 | 0.532158701 | -1.223082775 | 0.518142 | -2.36051 | 0.01825 | 0.046377 | Down |
| RNU4ATAC9P | 1.586719631 | -3.075234978 | 0.593988 | -5.17727 | 2.25E-07 | 2.06E-06 | Down |
| RNU6-844P | 1.804921612 | -1.377822933 | 0.453687 | -3.03695 | 0.00239 | 0.008246 | Down |
| RNU6-1231P | 0.91784391 | -1.989127189 | 0.535431 | -3.715 | 0.000203 | 0.00095 | Down |
| AP003548.1 | 11.6096937 | -1.584487334 | 0.334701 | -4.73404 | 2.20E-06 | 1.63E-05 | Down |
| AC114550.1 | 4.600447472 | -1.172602774 | 0.319892 | -3.66562 | 0.000247 | 0.001129 | Down |
| AC008632.1 | 7.850187612 | -2.400863745 | 0.407937 | -5.88538 | 3.97E-09 | 4.98E-08 | Down |
| AC037441.2 | 0.925980656 | -1.800791554 | 0.491838 | -3.66135 | 0.000251 | 0.001146 | Down |
| LINC01605 | 4.967473293 | 1.13364916 | 0.296885 | 3.818476 | 0.000134 | 0.000659 | Up |
| AC109479.1 | 1.318958556 | 1.135200921 | 0.409021 | 2.77541 | 0.005513 | 0.016954 | Up |
| TRBV6-7 | 1.162185062 | 1.434969888 | 0.486554 | 2.949252 | 0.003185 | 0.010575 | Up |
| KRT18P41 | 0.585249803 | 1.393994702 | 0.457475 | 3.047149 | 0.00231 | 0.007994 | Up |
| AC110998.1 | 15.02707011 | -1.022353165 | 0.227636 | -4.49118 | 7.08E-06 | 4.71E-05 | Down |
| C8orf88 | 203.2706177 | -1.74649251 | 0.191367 | -9.12642 | 7.08E-20 | 4.14E-18 | Down |
| AC022784.2 | 0.93167613 | 1.725684597 | 0.567041 | 3.043314 | 0.00234 | 0.008083 | Up |
| AC090541.1 | 6.346038552 | -1.550637252 | 0.368522 | -4.20773 | 2.58E-05 | 0.00015 | Down |
| AC046195.1 | 61.40874689 | -2.730071183 | 0.431463 | -6.32748 | 2.49E-10 | 3.87E-09 | Down |
| AC022217.2 | 7.538680821 | -1.519567677 | 0.353443 | -4.29933 | 1.71E-05 | 0.000104 | Down |
| AC018541.1 | 0.976812252 | 1.979809601 | 0.565219 | 3.502732 | 0.000461 | 0.001963 | Up |
| AC024451.2 | 13.05959022 | -1.073458088 | 0.24267 | -4.42352 | 9.71E-06 | 6.25E-05 | Down |
| AC009884.1 | 0.504180051 | 1.77240685 | 0.532076 | 3.331113 | 0.000865 | 0.00341 | Up |
| IGLV3-29 | 2.318320206 | 1.804274966 | 0.751414 | 2.401172 | 0.016343 | 0.042343 | Up |
| AC008514.1 | 3.331476814 | 1.59894013 | 0.285611 | 5.598319 | 2.16E-08 | 2.37E-07 | Up |
| AC084024.3 | 0.742597689 | -1.245770996 | 0.499338 | -2.49484 | 0.012601 | 0.034114 | Down |
| AC008708.1 | 5.84726646 | -1.166489174 | 0.358045 | -3.25794 | 0.001122 | 0.004274 | Down |
| AC069120.1 | 4.456686192 | 3.58569499 | 0.473605 | 7.57106 | 3.70E-14 | 1.03E-12 | Up |
| AC068672.2 | 3.907109847 | -3.358674384 | 0.488405 | -6.87682 | 6.12E-12 | 1.24E-10 | Down |
| AP003696.1 | 0.988925078 | -1.706426886 | 0.502657 | -3.39481 | 0.000687 | 0.002787 | Down |
| AC124067.2 | 4.712771605 | 1.082308898 | 0.254095 | 4.259462 | 2.05E-05 | 0.000122 | Up |
| LINC02159 | 13.28660947 | 2.066269191 | 0.327391 | 6.311328 | 2.77E-10 | 4.27E-09 | Up |
| AC103853.1 | 0.664695172 | -1.390759456 | 0.541697 | -2.56741 | 0.010246 | 0.028682 | Down |
| LINC01942 | 0.907387709 | -1.288863542 | 0.507707 | -2.5386 | 0.01113 | 0.030713 | Down |
| AC083843.1 | 2.540836731 | -1.405185768 | 0.3091 | -4.54606 | 5.47E-06 | 3.72E-05 | Down |
| AC103769.1 | 1.005384682 | -1.647093714 | 0.626202 | -2.63029 | 0.008531 | 0.024607 | Down |
| AC012213.1 | 13.14045079 | 1.303935663 | 0.367863 | 3.544626 | 0.000393 | 0.001705 | Up |
| AC104257.1 | 1.768819996 | 4.149658725 | 0.631913 | 6.566824 | 5.14E-11 | 9.04E-10 | Up |
| AC104966.1 | 1.828829548 | -1.074882317 | 0.398963 | -2.69419 | 0.007056 | 0.020947 | Down |
| AC090578.1 | 1.80734841 | 2.048823886 | 0.365068 | 5.612163 | 2.00E-08 | 2.21E-07 | Up |
| AC024568.1 | 2.754773814 | -1.257749907 | 0.335006 | -3.75441 | 0.000174 | 0.000828 | Down |
| AF181450.1 | 0.674300219 | -1.370814094 | 0.543643 | -2.52153 | 0.011684 | 0.032036 | Down |
| AC103952.1 | 1.952700933 | -1.050398649 | 0.343829 | -3.05501 | 0.002251 | 0.007819 | Down |
| AC107959.3 | 4.31113805 | 2.595161815 | 0.319011 | 8.135034 | 4.12E-16 | 1.49E-14 | Up |
| AC022893.1 | 43.78407421 | -1.044900169 | 0.118903 | -8.78785 | 1.52E-18 | 7.42E-17 | Down |
| GASAL1 | 57.95614757 | -1.473048766 | 0.186323 | -7.90589 | 2.66E-15 | 8.72E-14 | Down |
| IGKV2OR22-4 | 46.38018934 | 2.040078819 | 0.792228 | 2.575116 | 0.010021 | 0.028156 | Up |
| IGHV1-14 | 17.17024358 | 1.868490761 | 0.783515 | 2.384753 | 0.017091 | 0.043943 | Up |
| AC015909.2 | 2.115443713 | 1.581592225 | 0.271808 | 5.818791 | 5.93E-09 | 7.20E-08 | Up |
| AC091163.1 | 0.694154409 | -1.565985183 | 0.510846 | -3.06548 | 0.002173 | 0.007592 | Down |
| AC087620.1 | 1.111904717 | -1.218318962 | 0.465692 | -2.61615 | 0.008893 | 0.025496 | Down |
| IGHGP | 7069.667774 | 1.905695217 | 0.667476 | 2.855077 | 0.004303 | 0.013727 | Up |
| TPTE2P1 | 247.295918 | -1.662715237 | 0.279273 | -5.95374 | 2.62E-09 | 3.40E-08 | Down |
| LINC01170 | 8.722356977 | 3.6767904 | 0.367556 | 10.00335 | 1.47E-23 | 1.29E-21 | Up |
| AC090197.1 | 35.11657564 | 1.421774029 | 0.172574 | 8.238648 | 1.74E-16 | 6.64E-15 | Up |
| AC011383.1 | 2.872119485 | 2.515321075 | 0.332227 | 7.571083 | 3.70E-14 | 1.03E-12 | Up |
| AC245187.1 | 3.095297711 | -1.440214954 | 0.367609 | -3.91779 | 8.94E-05 | 0.000457 | Down |
| AC087752.3 | 41.3684796 | 1.23676201 | 0.131819 | 9.382302 | 6.45E-21 | 4.22E-19 | Up |
| ARF1P3 | 1.432957754 | -1.687074721 | 0.459954 | -3.66792 | 0.000245 | 0.00112 | Down |
| FAM85B | 10.29256197 | -1.128858824 | 0.346229 | -3.26044 | 0.001112 | 0.004242 | Down |
| AP002981.1 | 1.090129092 | -1.145316985 | 0.481918 | -2.37658 | 0.017474 | 0.044755 | Down |
| AC011369.1 | 0.769081152 | -1.684729371 | 0.550489 | -3.06042 | 0.00221 | 0.0077 | Down |
| AC007991.3 | 2.469411108 | -2.30036423 | 0.436131 | -5.27448 | 1.33E-07 | 1.27E-06 | Down |
| AC022730.4 | 5.441028666 | -1.375171375 | 0.564628 | -2.43553 | 0.01487 | 0.03917 | Down |
| AC016573.1 | 8.144701567 | -2.251026116 | 0.380332 | -5.91858 | 3.25E-09 | 4.15E-08 | Down |
| MAL2-AS1 | 18.54310575 | -1.32363301 | 0.307287 | -4.30748 | 1.65E-05 | 0.000101 | Down |
| AC087627.1 | 0.892677912 | 2.175031521 | 0.49298 | 4.412009 | 1.02E-05 | 6.56E-05 | Up |
| AC103726.2 | 0.684758287 | 3.315039724 | 0.985649 | 3.363308 | 0.00077 | 0.00308 | Up |
| AC104232.1 | 13.14680312 | -1.39831726 | 0.236434 | -5.9142 | 3.34E-09 | 4.24E-08 | Down |
| AC011676.2 | 0.728460656 | -1.581046321 | 0.506113 | -3.1239 | 0.001785 | 0.006399 | Down |
| AC083843.2 | 3.131481946 | -1.573804596 | 0.342043 | -4.60118 | 4.20E-06 | 2.93E-05 | Down |
| AC087273.2 | 1.5906977 | 2.254687158 | 0.476092 | 4.735819 | 2.18E-06 | 1.62E-05 | Up |
| AC084346.1 | 2.93665617 | -1.12223625 | 0.354781 | -3.16318 | 0.001561 | 0.005704 | Down |
| LINC02055 | 5.054385897 | -2.638340367 | 0.468511 | -5.63133 | 1.79E-08 | 1.99E-07 | Down |
| AC093367.1 | 5.427565957 | -1.158543184 | 0.267278 | -4.3346 | 1.46E-05 | 9.03E-05 | Down |
| AC113386.1 | 0.858942588 | 1.957229077 | 0.605986 | 3.229823 | 0.001239 | 0.004659 | Up |
| AC103853.2 | 2.077022538 | -1.292783649 | 0.374693 | -3.45024 | 0.00056 | 0.002333 | Down |
| AC022733.2 | 1.177513905 | 1.749294962 | 0.500081 | 3.498025 | 0.000469 | 0.001993 | Up |
| AC103957.2 | 63.64067948 | -1.65128045 | 0.287354 | -5.7465 | 9.11E-09 | 1.07E-07 | Down |
| AC110288.1 | 0.79893727 | -1.939274645 | 0.537485 | -3.60806 | 0.000308 | 0.001379 | Down |
| AC090739.1 | 51.67377879 | -1.601331625 | 0.219029 | -7.31106 | 2.65E-13 | 6.63E-12 | Down |
| IGHVIII-51-1 | 5.848268959 | 3.061763952 | 0.984332 | 3.110499 | 0.001868 | 0.006658 | Up |
| MAPK6P5 | 3.654237787 | -1.52994972 | 0.339496 | -4.50653 | 6.59E-06 | 4.41E-05 | Down |
| AC022034.3 | 4.289092277 | -1.707013318 | 0.321735 | -5.30565 | 1.12E-07 | 1.08E-06 | Down |
| LINC01933 | 14.20227948 | 1.737364589 | 0.326877 | 5.315035 | 1.07E-07 | 1.03E-06 | Up |
| AC022973.3 | 1.441602447 | -1.085842051 | 0.39958 | -2.71746 | 0.006579 | 0.019717 | Down |
| AC107953.2 | 3.74403032 | -1.477882127 | 0.312217 | -4.73352 | 2.21E-06 | 1.64E-05 | Down |
| AC124067.4 | 1.854308755 | -1.735692959 | 0.427073 | -4.06416 | 4.82E-05 | 0.000264 | Down |
| AC104232.2 | 0.658798981 | -1.739395757 | 0.727367 | -2.39136 | 0.016786 | 0.043292 | Down |
| AC091563.1 | 991.3803751 | -1.235391276 | 0.187381 | -6.59295 | 4.31E-11 | 7.64E-10 | Down |
| AC120036.3 | 10.31764809 | 1.671318605 | 0.19238 | 8.687584 | 3.70E-18 | 1.72E-16 | Up |
| MIR2052HG | 1.594693856 | -1.519358637 | 0.543264 | -2.79672 | 0.005162 | 0.016044 | Down |
| AC040914.1 | 1.493119457 | -1.491634601 | 0.403229 | -3.69923 | 0.000216 | 0.001002 | Down |
| AC048387.1 | 1.928359101 | -2.706155333 | 0.536871 | -5.0406 | 4.64E-07 | 3.96E-06 | Down |
| AL355432.1 | 4.05181228 | -1.622828004 | 0.370604 | -4.37887 | 1.19E-05 | 7.54E-05 | Down |
| LINC02584 | 1.734615335 | 1.148200811 | 0.425272 | 2.69992 | 0.006936 | 0.020642 | Up |
| AC084083.1 | 3.055740914 | 1.547926189 | 0.379145 | 4.082678 | 4.45E-05 | 0.000246 | Up |
| AP001646.1 | 1.216751785 | -1.236112509 | 0.459665 | -2.68916 | 0.007163 | 0.021224 | Down |
| OR7E11P | 0.55332352 | 2.042288435 | 0.70738 | 2.887115 | 0.003888 | 0.012566 | Up |
| AC091534.1 | 1.228964369 | -1.375896521 | 0.468304 | -2.93804 | 0.003303 | 0.010922 | Down |
| PPIAP41 | 4.510911013 | -1.307620091 | 0.280305 | -4.66499 | 3.09E-06 | 2.22E-05 | Down |
| OR4D10 | 11.86076546 | 6.706740411 | 0.64813 | 10.34784 | 4.28E-25 | 4.34E-23 | Up |
| AP001284.1 | 2.217418777 | 3.247748828 | 0.556182 | 5.839368 | 5.24E-09 | 6.42E-08 | Up |
| PTP4A2P2 | 16.48227182 | 1.618159973 | 0.220363 | 7.343167 | 2.09E-13 | 5.29E-12 | Up |
| MPPED2-AS1 | 95.67837857 | -2.330976999 | 0.27025 | -8.62526 | 6.39E-18 | 2.86E-16 | Down |
| AC104042.1 | 1.142612437 | -2.085701666 | 0.493514 | -4.22623 | 2.38E-05 | 0.000139 | Down |
| DEFB131E | 3.936321741 | 2.921553529 | 0.330047 | 8.851921 | 8.60E-19 | 4.33E-17 | Up |
| AP001107.5 | 18.03274767 | -1.383766886 | 0.370253 | -3.73735 | 0.000186 | 0.000878 | Down |
| AC090791.1 | 1.825661827 | -1.87314107 | 0.434058 | -4.31541 | 1.59E-05 | 9.74E-05 | Down |
| AP000757.1 | 67.36776137 | -1.239560663 | 0.256374 | -4.83496 | 1.33E-06 | 1.03E-05 | Down |
| AL354919.2 | 1.030251263 | 2.927245601 | 0.572371 | 5.114241 | 3.15E-07 | 2.79E-06 | Up |
| BBOX1-AS1 | 8.388568104 | 1.217096277 | 0.32781 | 3.712807 | 0.000205 | 0.000957 | Up |
| UBTFL9 | 0.88861433 | -1.896530959 | 0.787832 | -2.40728 | 0.016072 | 0.041759 | Down |
| MAGEL2 | 164.4878472 | -1.367898055 | 0.242395 | -5.64325 | 1.67E-08 | 1.87E-07 | Down |
| AP003396.2 | 0.725338267 | -1.837937922 | 0.577372 | -3.18328 | 0.001456 | 0.005372 | Down |
| AP003555.2 | 10.0634859 | 1.689118239 | 0.26853 | 6.290234 | 3.17E-10 | 4.83E-09 | Up |
| AC027031.2 | 37.859653 | 1.084712115 | 0.199703 | 5.431623 | 5.58E-08 | 5.73E-07 | Up |
| NAV2-AS4 | 13.0151485 | -1.361880559 | 0.30404 | -4.47928 | 7.49E-06 | 4.95E-05 | Down |
| AC116021.1 | 2.240699499 | 3.091222179 | 0.441581 | 7.000358 | 2.55E-12 | 5.52E-11 | Up |
| AC068858.1 | 4.593791888 | 2.552424245 | 0.373906 | 6.826377 | 8.71E-12 | 1.73E-10 | Up |
| AC103681.2 | 7.2730191 | -1.055221299 | 0.261313 | -4.03815 | 5.39E-05 | 0.000292 | Down |
| AP005018.1 | 0.931952003 | -1.843119678 | 0.478981 | -3.848 | 0.000119 | 0.000592 | Down |
| AP002358.2 | 9.336090984 | 4.938244381 | 0.55883 | 8.836758 | 9.85E-19 | 4.91E-17 | Up |
| AL512785.2 | 1.860105763 | 1.357680586 | 0.374109 | 3.629102 | 0.000284 | 0.001283 | Up |
| AC104237.1 | 1.244106868 | -1.524131502 | 0.469794 | -3.24425 | 0.001178 | 0.004457 | Down |
| MEX3A | 192.0816932 | 1.272860519 | 0.167087 | 7.617971 | 2.58E-14 | 7.37E-13 | Up |
| AP003396.3 | 0.684580999 | -1.647233901 | 0.543583 | -3.03032 | 0.002443 | 0.008404 | Down |
| AC084855.1 | 26.60014785 | 1.137314465 | 0.285992 | 3.976739 | 6.99E-05 | 0.000367 | Up |
| AC127526.1 | 3.498381483 | -1.121586785 | 0.247919 | -4.524 | 6.07E-06 | 4.09E-05 | Down |
| AP001893.2 | 0.858258111 | -1.175336357 | 0.502712 | -2.33799 | 0.019388 | 0.048794 | Down |
| AP001825.1 | 1.418009527 | -1.610559002 | 0.43412 | -3.70994 | 0.000207 | 0.000966 | Down |
| SLC25A47P1 | 47.06737023 | 7.075222315 | 0.549205 | 12.88265 | 5.64E-38 | 1.98E-35 | Up |
| AC022182.2 | 12.88495471 | -1.211025343 | 0.470182 | -2.57565 | 0.010005 | 0.028118 | Down |
| SLC22A18AS | 53.57727013 | 1.127517092 | 0.178163 | 6.328579 | 2.47E-10 | 3.85E-09 | Up |
| AP001893.3 | 3.127776193 | -1.260601263 | 0.350916 | -3.59232 | 0.000328 | 0.001453 | Down |
| AF131215.3 | 2.157690259 | 3.099410776 | 0.418237 | 7.410657 | 1.26E-13 | 3.27E-12 | Up |
| AC004923.2 | 0.719454465 | 2.18234433 | 0.571452 | 3.818946 | 0.000134 | 0.000658 | Up |
| AP001360.1 | 0.404108807 | 1.913813067 | 0.565531 | 3.384099 | 0.000714 | 0.00288 | Up |
| AC107884.2 | 6.520822033 | 1.037754993 | 0.195706 | 5.302612 | 1.14E-07 | 1.10E-06 | Up |
| AP006287.1 | 1.325209644 | -1.776087038 | 0.527512 | -3.36691 | 0.00076 | 0.003044 | Down |
| OPCML-IT1 | 0.79032275 | 3.521242276 | 1.030726 | 3.416273 | 0.000635 | 0.002605 | Up |
| AC091053.2 | 1.931192879 | -1.51316237 | 0.385947 | -3.92065 | 8.83E-05 | 0.000452 | Down |
| AC130366.1 | 0.493287191 | -1.674115279 | 0.605215 | -2.76615 | 0.005672 | 0.017374 | Down |
| AP000785.1 | 1.426886355 | -2.108026708 | 0.497248 | -4.23939 | 2.24E-05 | 0.000132 | Down |
| INMT-MINDY4 | 6.094940851 | 1.362802959 | 0.373224 | 3.651433 | 0.000261 | 0.001187 | Up |
| AP003498.1 | 1.109556094 | 1.430526444 | 0.38198 | 3.745033 | 0.00018 | 0.000855 | Up |
| AP001372.4 | 11.99744439 | -1.043541505 | 0.203354 | -5.13165 | 2.87E-07 | 2.57E-06 | Down |
| AP002008.1 | 0.566464084 | -1.264089136 | 0.493372 | -2.56214 | 0.010403 | 0.029049 | Down |
| AP001781.1 | 3.450775241 | -1.132856628 | 0.252615 | -4.48452 | 7.31E-06 | 4.85E-05 | Down |
| AP000755.1 | 0.805066976 | -1.601025703 | 0.515574 | -3.10533 | 0.001901 | 0.006758 | Down |
| AP000842.2 | 5.912885048 | -2.285314044 | 0.364589 | -6.2682 | 3.65E-10 | 5.50E-09 | Down |
| NAV2-AS5 | 9.63300522 | -1.348242799 | 0.292489 | -4.60955 | 4.04E-06 | 2.83E-05 | Down |
| AC104241.2 | 0.690135233 | 1.330620467 | 0.46315 | 2.872977 | 0.004066 | 0.01307 | Up |
| AF186192.2 | 253.3452811 | -1.10586738 | 0.123856 | -8.92864 | 4.31E-19 | 2.28E-17 | Down |
| AC010768.2 | 1.000750959 | -1.71289293 | 0.572398 | -2.99249 | 0.002767 | 0.009359 | Down |
| AC090458.1 | 0.865701772 | -2.280374815 | 0.609633 | -3.74057 | 0.000184 | 0.000868 | Down |
| AP000880.1 | 11.11477414 | -1.296670207 | 0.179914 | -7.20716 | 5.71E-13 | 1.37E-11 | Down |
| AC025857.1 | 1.149966791 | -1.432174528 | 0.490127 | -2.92205 | 0.003477 | 0.011415 | Down |
| AP003385.4 | 3.277465349 | 1.480859206 | 0.424773 | 3.486239 | 0.00049 | 0.002072 | Up |
| HTD2 | 5.835171474 | -1.029139812 | 0.227531 | -4.52307 | 6.09E-06 | 4.11E-05 | Down |
| AC022616.7 | 0.789476087 | 3.656907196 | 1.021206 | 3.580968 | 0.000342 | 0.001511 | Up |
| AP003555.3 | 1.799386 | 1.347871444 | 0.37378 | 3.606054 | 0.000311 | 0.001388 | Up |
| AP000755.2 | 0.688896894 | -1.303791039 | 0.519742 | -2.50853 | 0.012123 | 0.033039 | Down |
| MRPS36P4 | 0.607611998 | -1.487096892 | 0.587223 | -2.53242 | 0.011328 | 0.031175 | Down |
| AP002992.1 | 5.385166626 | -1.070977375 | 0.28014 | -3.82301 | 0.000132 | 0.000648 | Down |
| KC877392.1 | 2.273249943 | -1.46781399 | 0.432813 | -3.39134 | 0.000696 | 0.002816 | Down |
| ABCC6P2 | 43.89092933 | 2.093699359 | 0.217263 | 9.636709 | 5.60E-22 | 4.07E-20 | Up |
| WTAPP1 | 1.115772941 | 1.497731224 | 0.532258 | 2.813917 | 0.004894 | 0.015321 | Up |
| AP002370.2 | 10.49692944 | -1.259316735 | 0.30238 | -4.16469 | 3.12E-05 | 0.000178 | Down |
| ENPP7P8 | 5.121489507 | 1.155063156 | 0.27336 | 4.225426 | 2.38E-05 | 0.00014 | Up |
| AL096711.2 | 21.36095005 | 2.25880749 | 0.196241 | 11.51035 | 1.17E-30 | 2.12E-28 | Up |
| AP002957.1 | 3.355577946 | 2.512102796 | 0.347885 | 7.221067 | 5.16E-13 | 1.25E-11 | Up |
| NOX5 | 183.4558197 | 2.899556337 | 0.327949 | 8.841473 | 9.45E-19 | 4.73E-17 | Up |
| AC019227.1 | 1.298897867 | -1.695550243 | 0.472326 | -3.58979 | 0.000331 | 0.001464 | Down |
| SMILR | 0.686518995 | 1.885428455 | 0.718207 | 2.62519 | 0.00866 | 0.024924 | Up |
| AC120036.4 | 64.54551579 | 1.8156024 | 0.192076 | 9.452532 | 3.31E-21 | 2.21E-19 | Up |
| AP001258.1 | 1.027510163 | 1.304947461 | 0.401445 | 3.250626 | 0.001152 | 0.004372 | Up |
| AC124276.2 | 3.340716674 | -1.541382799 | 0.6342 | -2.43044 | 0.015081 | 0.039659 | Down |
| AC090092.1 | 12.28224289 | 1.821574622 | 0.221761 | 8.21413 | 2.14E-16 | 8.05E-15 | Up |
| AP002340.1 | 4.250703706 | -1.020683715 | 0.265596 | -3.843 | 0.000122 | 0.000603 | Down |
| AP002954.1 | 5.734653656 | 1.297014177 | 0.430204 | 3.014884 | 0.002571 | 0.008779 | Up |
| AP002008.3 | 2.448038531 | -2.265053553 | 0.458674 | -4.93826 | 7.88E-07 | 6.43E-06 | Down |
| AP001085.1 | 12.41938996 | -1.43085844 | 0.291128 | -4.91487 | 8.88E-07 | 7.16E-06 | Down |
| CD44-AS1 | 21.56149376 | 1.415723076 | 0.27111 | 5.221949 | 1.77E-07 | 1.64E-06 | Up |
| CYCSP27 | 0.536636753 | -1.567331532 | 0.580137 | -2.70166 | 0.0069 | 0.020551 | Down |
| AL353699.1 | 10.67910249 | -2.81068233 | 0.351192 | -8.00326 | 1.21E-15 | 4.15E-14 | Down |
| AL356215.1 | 37.65973903 | 1.187861183 | 0.234717 | 5.060831 | 4.17E-07 | 3.60E-06 | Up |
| SNRPCP5 | 1.1589227 | -1.04756135 | 0.430237 | -2.43484 | 0.014898 | 0.039234 | Down |
| NPIPB8 | 1.418775297 | -1.892589005 | 0.515755 | -3.66955 | 0.000243 | 0.001113 | Down |
| AP004608.1 | 55.07070202 | 1.040344533 | 0.177826 | 5.850336 | 4.91E-09 | 6.04E-08 | Up |
| RPA2P3 | 0.521045823 | -1.763333357 | 0.557839 | -3.16101 | 0.001572 | 0.005741 | Down |
| UNC93B6 | 2.374159859 | 1.115060088 | 0.339249 | 3.286849 | 0.001013 | 0.003917 | Up |
| AC018653.1 | 1.803282979 | -1.213718259 | 0.367151 | -3.30577 | 0.000947 | 0.003691 | Down |
| RAB44 | 3.796317421 | 2.02786927 | 0.275795 | 7.352819 | 1.94E-13 | 4.93E-12 | Up |
| AC007368.1 | 13.54929287 | 4.646635037 | 0.59556 | 7.802131 | 6.09E-15 | 1.91E-13 | Up |
| AP000997.1 | 2.081299162 | 2.774152307 | 0.395584 | 7.0128 | 2.34E-12 | 5.08E-11 | Up |
| AC093510.1 | 8.859449374 | -1.117111628 | 0.199037 | -5.6126 | 1.99E-08 | 2.20E-07 | Down |
| AP003174.1 | 3.5993567 | 2.466295809 | 0.314023 | 7.853882 | 4.03E-15 | 1.29E-13 | Up |
| LINC01489 | 1.382990404 | 1.152340814 | 0.374579 | 3.076365 | 0.002095 | 0.00735 | Up |
| AC011462.2 | 1.699500671 | 1.126666964 | 0.349779 | 3.221083 | 0.001277 | 0.004788 | Up |
| AC022509.1 | 2.756973713 | 1.583884706 | 0.392687 | 4.033454 | 5.50E-05 | 0.000297 | Up |
| LINC01479 | 1.214535921 | 1.144580612 | 0.434778 | 2.632563 | 0.008474 | 0.024459 | Up |
| AC090023.1 | 0.617850714 | 3.055864699 | 0.657499 | 4.647711 | 3.36E-06 | 2.40E-05 | Up |
| AC069234.1 | 5.980501452 | -2.069384546 | 0.355975 | -5.81328 | 6.13E-09 | 7.41E-08 | Down |
| AC007688.2 | 1.340562209 | -1.016534346 | 0.429185 | -2.36852 | 0.017859 | 0.045565 | Down |
| TAS2R63P | 2.153423878 | -2.120521321 | 0.403752 | -5.25204 | 1.50E-07 | 1.42E-06 | Down |
| ELOCP31 | 1.204487708 | -1.194265613 | 0.419214 | -2.84882 | 0.004388 | 0.013958 | Down |
| AL590560.1 | 3.453682806 | -1.970610802 | 0.40161 | -4.90678 | 9.26E-07 | 7.44E-06 | Down |
| MRPL40P1 | 3.276659663 | 2.151344705 | 0.386519 | 5.565941 | 2.61E-08 | 2.83E-07 | Up |
| MTRNR2L10 | 9.514166942 | 1.068831927 | 0.299689 | 3.566466 | 0.000362 | 0.001585 | Up |
| AC063926.1 | 4.283466785 | 2.691394927 | 0.419441 | 6.416628 | 1.39E-10 | 2.27E-09 | Up |
| EGLN3P1 | 1.385652388 | -1.830823932 | 0.47915 | -3.82099 | 0.000133 | 0.000653 | Down |
| AC007637.1 | 77.01147813 | -1.061678632 | 0.18387 | -5.77409 | 7.74E-09 | 9.19E-08 | Down |
| LINC02571 | 2.405970696 | 1.307397078 | 0.35888 | 3.642988 | 0.000269 | 0.001222 | Up |
| AC055720.2 | 30.72434579 | 1.243492825 | 0.132915 | 9.355563 | 8.32E-21 | 5.36E-19 | Up |
| TAS2R30 | 1.66778353 | -1.042575275 | 0.397933 | -2.61998 | 0.008794 | 0.025252 | Down |
| AC022509.2 | 33.38906958 | 1.522682645 | 0.16636 | 9.152933 | 5.54E-20 | 3.27E-18 | Up |
| LINC02454 | 42.89227532 | 4.429678749 | 0.305314 | 14.50859 | 1.07E-47 | 1.11E-44 | Up |
| TAS2R64P | 5.788434819 | -1.012241601 | 0.390935 | -2.58929 | 0.009618 | 0.027221 | Down |
| ABCC6P1 | 41.219897 | 2.646372551 | 0.225741 | 11.72307 | 9.71E-32 | 1.92E-29 | Up |
| AP006333.1 | 3.40053895 | -1.519490748 | 0.38642 | -3.93223 | 8.42E-05 | 0.000433 | Down |
| NTAN1P3 | 0.921252322 | 2.817240756 | 0.580517 | 4.852987 | 1.22E-06 | 9.52E-06 | Up |
| AC006206.2 | 3.139840046 | -2.938635786 | 0.933159 | -3.14913 | 0.001638 | 0.005939 | Down |
| SALL3 | 4.827510369 | -1.314152135 | 0.457919 | -2.86984 | 0.004107 | 0.01318 | Down |
| AP006333.2 | 19.12575515 | -1.01748016 | 0.273261 | -3.72348 | 0.000196 | 0.000923 | Down |
| AC023796.2 | 0.879760671 | 1.96591716 | 0.688496 | 2.855381 | 0.004299 | 0.013715 | Up |
| AC007368.2 | 0.495449393 | 2.494053444 | 0.949383 | 2.627025 | 0.008613 | 0.024809 | Up |
| AP003419.1 | 0.715858343 | 3.368056432 | 0.960612 | 3.506156 | 0.000455 | 0.001942 | Up |
| AC046130.2 | 3.053742585 | -1.030913012 | 0.278713 | -3.69883 | 0.000217 | 0.001004 | Down |
| AC007406.3 | 36.20815935 | 1.646470123 | 0.251211 | 6.554124 | 5.60E-11 | 9.78E-10 | Up |
| AC148477.2 | 102.6857319 | -1.53247913 | 0.246928 | -6.20617 | 5.43E-10 | 7.90E-09 | Down |
| AC092745.2 | 19.57746734 | -1.360556259 | 0.17146 | -7.93514 | 2.10E-15 | 7.01E-14 | Down |
| LINC01486 | 2.290982838 | 1.478158741 | 0.49589 | 2.980818 | 0.002875 | 0.009675 | Up |
| AC007552.2 | 23.99677663 | 1.040802963 | 0.262781 | 3.960728 | 7.47E-05 | 0.00039 | Up |
| AC084880.3 | 5.253592737 | -1.562944432 | 0.368897 | -4.23681 | 2.27E-05 | 0.000134 | Down |
| MTRNR2L1 | 214.4841272 | -4.624950955 | 0.602816 | -7.67224 | 1.69E-14 | 4.98E-13 | Down |
| AC005906.2 | 8.651843927 | -3.378641491 | 0.434423 | -7.7773 | 7.41E-15 | 2.31E-13 | Down |
| CLEC12B | 3.484847379 | 1.083617225 | 0.450036 | 2.407844 | 0.016047 | 0.041701 | Up |
| AC112777.1 | 4.383168906 | 1.015378075 | 0.429727 | 2.362843 | 0.018135 | 0.046168 | Up |
| AC026369.2 | 62.27746974 | 1.733217596 | 0.29071 | 5.962015 | 2.49E-09 | 3.25E-08 | Up |
| PGA5 | 43.27479158 | -1.547350625 | 0.258758 | -5.97992 | 2.23E-09 | 2.93E-08 | Down |
| AP002991.1 | 2.677611243 | 2.940115716 | 0.363791 | 8.081889 | 6.38E-16 | 2.25E-14 | Up |
| AP002840.1 | 0.626833765 | -1.797835608 | 0.587889 | -3.05812 | 0.002227 | 0.00775 | Down |
| AP000721.2 | 11.85821248 | -1.331372217 | 0.296726 | -4.48688 | 7.23E-06 | 4.80E-05 | Down |
| AC023157.2 | 0.827015844 | 1.426897572 | 0.594589 | 2.399804 | 0.016404 | 0.042472 | Up |
| SLC5A8 | 4686.461832 | -1.301907113 | 0.341465 | -3.81271 | 0.000137 | 0.000672 | Down |
| AC084880.4 | 2.624042894 | -1.87976591 | 0.466997 | -4.02522 | 5.69E-05 | 0.000306 | Down |
| IQSEC3P1 | 1.581263338 | -1.057369914 | 0.396539 | -2.66649 | 0.007665 | 0.022447 | Down |
| AC090023.2 | 3.109208493 | 3.60701534 | 0.538466 | 6.698682 | 2.10E-11 | 3.91E-10 | Up |
| AC148477.3 | 30.65074399 | -1.927946496 | 0.247095 | -7.80244 | 6.07E-15 | 1.91E-13 | Down |
| AP002518.2 | 3.087020223 | -2.305595464 | 0.484983 | -4.75397 | 1.99E-06 | 1.49E-05 | Down |
| AC007207.2 | 36.22136727 | 3.176100099 | 0.237191 | 13.39047 | 6.87E-41 | 3.31E-38 | Up |
| AP000438.1 | 9.472664347 | -1.022305749 | 0.210663 | -4.85281 | 1.22E-06 | 9.53E-06 | Down |
| GPR142 | 30.95392643 | -2.145526707 | 0.30978 | -6.92596 | 4.33E-12 | 9.04E-11 | Down |
| HP | 24.56141058 | 2.548843229 | 0.334694 | 7.615437 | 2.63E-14 | 7.50E-13 | Up |
| C11orf97 | 0.702630227 | -1.279488381 | 0.504812 | -2.53458 | 0.011258 | 0.031008 | Down |
| AC091078.1 | 2.737871114 | -2.645060688 | 0.741219 | -3.56853 | 0.000359 | 0.001575 | Down |
| AP000797.3 | 2.655707669 | 1.799736767 | 0.380413 | 4.731003 | 2.23E-06 | 1.66E-05 | Up |
| AP001453.3 | 7.630246294 | -1.560238765 | 0.282705 | -5.51897 | 3.41E-08 | 3.61E-07 | Down |
| RPEP6 | 0.84664144 | -2.000939487 | 0.570944 | -3.50462 | 0.000457 | 0.001951 | Down |
| CLIP1-AS1 | 9.944382043 | -1.566962471 | 0.235547 | -6.65245 | 2.88E-11 | 5.26E-10 | Down |
| NHLRC4 | 54.95688262 | 1.461411577 | 0.215965 | 6.766885 | 1.32E-11 | 2.54E-10 | Up |
| LINC02450 | 0.845468081 | 2.121804206 | 0.584353 | 3.631029 | 0.000282 | 0.001273 | Up |
| AC084398.2 | 0.952001351 | 1.617505099 | 0.506267 | 3.194964 | 0.001398 | 0.005186 | Up |
| LNCOG | 3.630369181 | 2.150009551 | 0.465482 | 4.618893 | 3.86E-06 | 2.72E-05 | Up |
| LINC02388 | 1.031765535 | -1.677103798 | 0.55759 | -3.00777 | 0.002632 | 0.008962 | Down |
| AL139020.2 | 21.76703311 | -2.663218235 | 0.919907 | -2.8951 | 0.00379 | 0.012293 | Down |
| AC127164.1 | 15.35188434 | 1.665492915 | 0.225477 | 7.386527 | 1.51E-13 | 3.89E-12 | Up |
| HNRNPA1P69 | 1.968063156 | -1.47559345 | 0.411534 | -3.58559 | 0.000336 | 0.001486 | Down |
| PRELID2P1 | 0.778508249 | 1.451886569 | 0.588861 | 2.465585 | 0.013679 | 0.036549 | Up |
| AC074031.1 | 1.034358114 | -1.978489204 | 0.515905 | -3.83499 | 0.000126 | 0.000621 | Down |
| PPFIA2-AS1 | 1.409942746 | -1.036017289 | 0.414621 | -2.49871 | 0.012465 | 0.0338 | Down |
| AC027288.1 | 2.238406094 | -1.823289158 | 0.406917 | -4.48073 | 7.44E-06 | 4.93E-05 | Down |
| AC007424.1 | 108.8414146 | 2.914216161 | 0.344973 | 8.447655 | 2.97E-17 | 1.24E-15 | Up |
| AL157935.2 | 31.61635 | 1.192133224 | 0.221174 | 5.390019 | 7.05E-08 | 7.08E-07 | Up |
| OR7E47P | 39.62664801 | 1.424804648 | 0.223601 | 6.372099 | 1.86E-10 | 2.96E-09 | Up |
| HLX-AS1 | 3.560674706 | -1.239360693 | 0.295661 | -4.19183 | 2.77E-05 | 0.00016 | Down |
| PPP1R12A-AS1 | 223.0368381 | -1.04724201 | 0.151595 | -6.90814 | 4.91E-12 | 1.01E-10 | Down |
| LINC00609 | 18.36793349 | -1.71615083 | 0.251993 | -6.81032 | 9.74E-12 | 1.92E-10 | Down |
| AC025154.2 | 51.34092722 | 1.549417558 | 0.276584 | 5.601982 | 2.12E-08 | 2.33E-07 | Up |
| AC027288.2 | 1.934218279 | -1.659799195 | 0.420289 | -3.94918 | 7.84E-05 | 0.000407 | Down |
| AC124312.2 | 109.9341972 | -1.295523936 | 0.244658 | -5.29524 | 1.19E-07 | 1.14E-06 | Down |
| LBX2-AS1 | 133.8628498 | 1.426668895 | 0.18387 | 7.759097 | 8.55E-15 | 2.63E-13 | Up |
| AC079385.2 | 9.657035924 | -1.066560892 | 0.29802 | -3.57882 | 0.000345 | 0.001522 | Down |
| AC089983.1 | 0.73108042 | -1.883323843 | 0.768167 | -2.45171 | 0.014218 | 0.037744 | Down |
| MGAM2 | 6.667389316 | -1.626876364 | 0.418408 | -3.88825 | 0.000101 | 0.00051 | Down |
| AL133304.2 | 86.32142712 | -1.57451562 | 0.405756 | -3.88045 | 0.000104 | 0.000526 | Down |
| AC021066.1 | 6.032425875 | -1.544619821 | 0.345898 | -4.46554 | 7.99E-06 | 5.25E-05 | Down |
| AC012038.2 | 9.959273443 | 4.941832122 | 0.399859 | 12.35893 | 4.36E-35 | 1.08E-32 | Up |
| AC089998.3 | 3.395539516 | -1.327962733 | 0.345534 | -3.84322 | 0.000121 | 0.000602 | Down |
| AC125603.2 | 1.219595036 | -1.387438134 | 0.425245 | -3.26268 | 0.001104 | 0.004214 | Down |
| AL133372.3 | 0.565920381 | -1.393167703 | 0.518175 | -2.6886 | 0.007175 | 0.021255 | Down |
| AC124784.1 | 1.86800255 | -1.737174973 | 0.402541 | -4.31552 | 1.59E-05 | 9.74E-05 | Down |
| LINC02409 | 5.488482028 | -1.425881195 | 0.282593 | -5.04571 | 4.52E-07 | 3.87E-06 | Down |
| AC008083.1 | 2.366302019 | -2.535046444 | 0.869533 | -2.91541 | 0.003552 | 0.011613 | Down |
| AC126177.5 | 4.881333401 | 1.832756718 | 0.360165 | 5.088662 | 3.61E-07 | 3.16E-06 | Up |
| AC011773.2 | 0.670517158 | -1.574595286 | 0.576354 | -2.73199 | 0.006295 | 0.018993 | Down |
| LINC02306 | 1.079540776 | 3.851483873 | 1.479442 | 2.603336 | 0.009232 | 0.026316 | Up |
| AC078864.1 | 39.26589874 | 1.759635459 | 0.244003 | 7.211531 | 5.53E-13 | 1.33E-11 | Up |
| AC126614.1 | 2.136179649 | -1.046209588 | 0.336265 | -3.11127 | 0.001863 | 0.006643 | Down |
| AC016705.1 | 1.462274489 | -1.166281094 | 0.440108 | -2.64999 | 0.008049 | 0.023422 | Down |
| AC073569.1 | 2.481591361 | -1.272114678 | 0.354686 | -3.58659 | 0.000335 | 0.001481 | Down |
| AC126177.6 | 3.597289946 | 1.824214566 | 0.319928 | 5.701951 | 1.18E-08 | 1.37E-07 | Up |
| AC078923.1 | 13.19963688 | -1.610268542 | 0.254311 | -6.33189 | 2.42E-10 | 3.78E-09 | Down |
| AL391832.3 | 4.526512531 | 1.703619907 | 0.291319 | 5.847952 | 4.98E-09 | 6.12E-08 | Up |
| OR9A4 | 0.491207341 | 2.102410937 | 0.616895 | 3.408051 | 0.000654 | 0.002673 | Up |
| AC078820.1 | 7.913757944 | -1.969553591 | 0.293607 | -6.70814 | 1.97E-11 | 3.68E-10 | Down |
| LINC02286 | 1.999668443 | -2.97366688 | 0.562274 | -5.28864 | 1.23E-07 | 1.18E-06 | Down |
| AC026116.1 | 81.48952845 | -1.495105991 | 0.396094 | -3.77463 | 0.00016 | 0.000772 | Down |
| MKRN9P | 23.51821354 | 2.668119982 | 0.348971 | 7.645684 | 2.08E-14 | 6.04E-13 | Up |
| AC007656.1 | 14.05435784 | -1.084799987 | 0.179585 | -6.04059 | 1.54E-09 | 2.07E-08 | Down |
| LINC02406 | 1.656445235 | -2.161104612 | 0.549443 | -3.93326 | 8.38E-05 | 0.000431 | Down |
| AC089998.4 | 1.927644473 | -1.441494365 | 0.613387 | -2.35006 | 0.018771 | 0.047463 | Down |
| CLEC5A | 204.325607 | 1.208520113 | 0.277571 | 4.353909 | 1.34E-05 | 8.35E-05 | Up |
| AC125603.3 | 7.518495971 | -1.125702453 | 0.265165 | -4.24528 | 2.18E-05 | 0.000129 | Down |
| AC012085.2 | 12.77989958 | -1.343728433 | 0.214714 | -6.25823 | 3.89E-10 | 5.85E-09 | Down |
| LINC02461 | 2.992330238 | 4.727565538 | 0.713275 | 6.627968 | 3.40E-11 | 6.13E-10 | Up |
| AC125611.4 | 3.704002308 | 1.067977841 | 0.288671 | 3.699632 | 0.000216 | 0.001001 | Up |
| AC078778.1 | 3.5198304 | -1.075585778 | 0.30589 | -3.51625 | 0.000438 | 0.001877 | Down |
| AC079174.1 | 1.181302848 | -1.120217938 | 0.430943 | -2.59946 | 0.009337 | 0.026563 | Down |
| AL355102.1 | 6.722790116 | -2.277458114 | 0.42589 | -5.34753 | 8.92E-08 | 8.78E-07 | Down |
| AL133153.1 | 0.647203174 | 1.894260289 | 0.493349 | 3.839598 | 0.000123 | 0.00061 | Up |
| AC023510.1 | 2.661728342 | -1.206883087 | 0.441498 | -2.73361 | 0.006264 | 0.01892 | Down |
| AC012651.1 | 140.7695313 | 1.810271218 | 0.247054 | 7.327443 | 2.35E-13 | 5.92E-12 | Up |
| AC005726.1 | 355.4320521 | -1.423908299 | 0.138708 | -10.2655 | 1.01E-24 | 9.80E-23 | Down |
| AC027013.1 | 1.735503052 | -1.03113589 | 0.413468 | -2.49387 | 0.012636 | 0.034188 | Down |
| DIO3OS | 69.12828959 | -1.368132179 | 0.27896 | -4.9044 | 9.37E-07 | 7.52E-06 | Down |
| AC007954.1 | 0.754814531 | -1.569590155 | 0.468213 | -3.3523 | 0.000801 | 0.003192 | Down |
| LINC02295 | 1.635372336 | -2.451818432 | 0.912195 | -2.68782 | 0.007192 | 0.021297 | Down |
| ASB9P1 | 5.426797394 | -1.187393687 | 0.249293 | -4.76304 | 1.91E-06 | 1.43E-05 | Down |
| AL079307.1 | 1.05676844 | 1.520476296 | 0.543433 | 2.797909 | 0.005143 | 0.015997 | Up |
| AC068896.1 | 84.87361168 | -1.012537116 | 0.155852 | -6.4968 | 8.20E-11 | 1.39E-09 | Down |
| AL157911.1 | 6.516148111 | -1.483128849 | 0.243632 | -6.08758 | 1.15E-09 | 1.58E-08 | Down |
| AC007376.2 | 2.923615685 | 1.03413738 | 0.251369 | 4.114015 | 3.89E-05 | 0.000217 | Up |
| AL163195.2 | 0.685384328 | 1.850430447 | 0.676782 | 2.734162 | 0.006254 | 0.018892 | Up |
| FAM181A-AS1 | 47.4341199 | -1.153465031 | 0.233005 | -4.95038 | 7.41E-07 | 6.08E-06 | Down |
| NBEAP1 | 17.43070251 | -1.294135131 | 0.214784 | -6.02529 | 1.69E-09 | 2.27E-08 | Down |
| AL161668.4 | 3.282517421 | -1.515553026 | 0.299128 | -5.06657 | 4.05E-07 | 3.50E-06 | Down |
| LINC02303 | 0.662840691 | 1.4845259 | 0.586104 | 2.532869 | 0.011313 | 0.031142 | Up |
| LINC02292 | 1.271131228 | -2.58887204 | 0.485532 | -5.33203 | 9.71E-08 | 9.50E-07 | Down |
| AL121821.1 | 11.17433761 | -2.013288906 | 0.30605 | -6.5783 | 4.76E-11 | 8.41E-10 | Down |
| LINC00930 | 29.11304008 | -1.36175192 | 0.239298 | -5.69062 | 1.27E-08 | 1.46E-07 | Down |
| AL079303.1 | 32.27818187 | 1.40148278 | 0.284964 | 4.918104 | 8.74E-07 | 7.06E-06 | Up |
| AL049874.3 | 3.626432732 | -1.44149952 | 0.337525 | -4.2708 | 1.95E-05 | 0.000117 | Down |
| LINC01397 | 1.058308619 | -1.50280515 | 0.570262 | -2.63529 | 0.008407 | 0.024301 | Down |
| LINC02308 | 17.02252684 | -2.135160204 | 0.223196 | -9.56631 | 1.11E-21 | 7.79E-20 | Down |
| AC091544.2 | 17.73537677 | -1.554793154 | 0.293205 | -5.30276 | 1.14E-07 | 1.10E-06 | Down |
| AL137786.1 | 3.216553807 | -1.282349796 | 0.470401 | -2.72608 | 0.006409 | 0.019289 | Down |
| AC025884.1 | 7.605767933 | -1.0650376 | 0.426551 | -2.49686 | 0.01253 | 0.033943 | Down |
| AL357093.2 | 0.883560475 | -1.755699753 | 0.503553 | -3.48662 | 0.000489 | 0.00207 | Down |
| RAP1AP | 12.65458755 | 2.390881983 | 0.257436 | 9.28727 | 1.58E-20 | 9.85E-19 | Up |
| NANOGP7 | 0.704277008 | -1.480946757 | 0.535694 | -2.76454 | 0.0057 | 0.017452 | Down |
| CKS1BP1 | 33.66732989 | -1.557501026 | 0.338568 | -4.60027 | 4.22E-06 | 2.94E-05 | Down |
| LINC00520 | 1.151171889 | 1.234111474 | 0.496237 | 2.486938 | 0.012885 | 0.034764 | Up |
| AL137230.1 | 1.289337039 | 1.617941062 | 0.572021 | 2.828464 | 0.004677 | 0.014737 | Up |
| AL133153.2 | 0.615518308 | 1.668647997 | 0.529618 | 3.15066 | 0.001629 | 0.005914 | Up |
| TRAV33 | 0.553212254 | 2.466776535 | 0.805434 | 3.062668 | 0.002194 | 0.007652 | Up |
| AC131532.1 | 1.552604947 | -2.913317413 | 0.878894 | -3.31475 | 0.000917 | 0.003591 | Down |
| AF111167.1 | 11.69835182 | -3.184024189 | 0.389319 | -8.17844 | 2.88E-16 | 1.06E-14 | Down |
| AL162511.1 | 102.3326019 | 1.89291792 | 0.173723 | 10.8962 | 1.20E-27 | 1.59E-25 | Up |
| AL139354.1 | 1.230042733 | -1.439136101 | 0.542815 | -2.65124 | 0.00802 | 0.023349 | Down |
| AC021979.2 | 2.551120932 | -2.716090036 | 0.473709 | -5.73367 | 9.83E-09 | 1.15E-07 | Down |
| AL133163.2 | 2.084987726 | -2.642715661 | 0.696894 | -3.79213 | 0.000149 | 0.000725 | Down |
| SETP1 | 0.411304315 | 2.129094181 | 0.628858 | 3.385651 | 0.00071 | 0.002865 | Up |
| BHLHB9P1 | 19.99143414 | -1.344158978 | 0.217534 | -6.17908 | 6.45E-10 | 9.24E-09 | Down |
| AL355916.2 | 66.19893505 | -1.409627447 | 0.246456 | -5.71958 | 1.07E-08 | 1.24E-07 | Down |
| TUBB3 | 48.23307888 | 2.768643168 | 0.370182 | 7.479143 | 7.48E-14 | 2.01E-12 | Up |
| HIF1AP1 | 0.498550907 | -1.471662438 | 0.593599 | -2.47922 | 0.013167 | 0.035393 | Down |
| TMEM179 | 7.948129752 | 1.617190822 | 0.354236 | 4.565297 | 4.99E-06 | 3.43E-05 | Up |
| AL355075.4 | 2.635715432 | -1.70724627 | 0.498478 | -3.42492 | 0.000615 | 0.002534 | Down |
| AC005479.1 | 32.74971948 | 1.828002228 | 0.246142 | 7.426615 | 1.11E-13 | 2.92E-12 | Up |
| AL355102.5 | 1.607843758 | -1.974362872 | 0.468302 | -4.216 | 2.49E-05 | 0.000145 | Down |
| AL161804.1 | 4.848713964 | -1.279911477 | 0.305912 | -4.18392 | 2.87E-05 | 0.000165 | Down |
| AC244502.3 | 16.24683605 | 5.21296553 | 0.57537 | 9.060203 | 1.30E-19 | 7.37E-18 | Up |
| MTCO1P2 | 16.75343463 | 1.070798254 | 0.28687 | 3.732695 | 0.000189 | 0.000892 | Up |
| LINC02332 | 5.488766634 | 3.105087465 | 0.503778 | 6.163601 | 7.11E-10 | 1.01E-08 | Up |
| AL163195.3 | 0.816575761 | 3.496301857 | 0.960215 | 3.641167 | 0.000271 | 0.00123 | Up |
| AC091544.3 | 2.182085103 | -1.76234328 | 0.36621 | -4.81239 | 1.49E-06 | 1.14E-05 | Down |
| AC013457.1 | 2.99739385 | 4.730692264 | 1.02175 | 4.629989 | 3.66E-06 | 2.59E-05 | Up |
| CAP2P1 | 1.557726418 | -1.314235607 | 0.460724 | -2.85255 | 0.004337 | 0.013819 | Down |
| AL591767.3 | 1.485768535 | 1.002097062 | 0.315356 | 3.177671 | 0.001485 | 0.005464 | Up |
| NMNAT1P1 | 14.66727418 | -1.495748937 | 0.30901 | -4.84046 | 1.30E-06 | 1.01E-05 | Down |
| AC012378.1 | 1.534354877 | -1.044628876 | 0.372359 | -2.80544 | 0.005025 | 0.015684 | Down |
| AC114546.1 | 1.323451048 | -1.316467115 | 0.501537 | -2.62486 | 0.008668 | 0.024944 | Down |
| AC109631.1 | 1.46088623 | -1.672628476 | 0.468457 | -3.5705 | 0.000356 | 0.001564 | Down |
| SLC35G6 | 4.55629283 | -1.391565666 | 0.29908 | -4.65282 | 3.27E-06 | 2.34E-05 | Down |
| LINC02345 | 15.38460997 | 2.536773064 | 0.29829 | 8.504377 | 1.83E-17 | 7.75E-16 | Up |
| GOLGA8VP | 60.88761376 | -1.058609804 | 0.183968 | -5.75432 | 8.70E-09 | 1.03E-07 | Down |
| AC002306.1 | 9.045432635 | -1.750350739 | 0.217525 | -8.04665 | 8.51E-16 | 2.96E-14 | Down |
| AC087286.1 | 3.975101043 | 2.092288057 | 0.457811 | 4.570205 | 4.87E-06 | 3.36E-05 | Up |
| LINC02349 | 0.738858152 | 3.335098203 | 0.851332 | 3.917506 | 8.95E-05 | 0.000457 | Up |
| LIPC-AS1 | 3.290042751 | -1.333450695 | 0.343604 | -3.88078 | 0.000104 | 0.000525 | Down |
| AC025580.1 | 42.88222966 | 2.533029714 | 0.283953 | 8.920608 | 4.64E-19 | 2.44E-17 | Up |
| AC025580.2 | 7.422253691 | 1.6975889 | 0.274463 | 6.185122 | 6.21E-10 | 8.91E-09 | Up |
| AC046168.1 | 5.504034485 | 1.107969972 | 0.215935 | 5.131034 | 2.88E-07 | 2.57E-06 | Up |
| AC013356.3 | 2.818463614 | -1.187198082 | 0.343302 | -3.45818 | 0.000544 | 0.002274 | Down |
| AC103740.1 | 20.30581767 | -1.125540541 | 0.258268 | -4.35803 | 1.31E-05 | 8.21E-05 | Down |
| NDUFB4P11 | 3.430397617 | -1.507570907 | 0.505098 | -2.98471 | 0.002838 | 0.009566 | Down |
| AC090970.1 | 2.378682948 | -1.683997498 | 0.496097 | -3.39449 | 0.000688 | 0.002789 | Down |
| CTXND1 | 910.5259151 | 3.647561898 | 0.349615 | 10.43309 | 1.75E-25 | 1.84E-23 | Up |
| AC091117.2 | 3.497884949 | -1.500855534 | 0.322335 | -4.65619 | 3.22E-06 | 2.31E-05 | Down |
| AC091100.1 | 1.42812499 | -1.07452968 | 0.433929 | -2.47628 | 0.013276 | 0.03565 | Down |
| AC011767.1 | 6.27088223 | -1.010216131 | 0.314649 | -3.21061 | 0.001325 | 0.004946 | Down |
| AC027237.3 | 65.46610748 | -1.147505392 | 0.235992 | -4.86248 | 1.16E-06 | 9.11E-06 | Down |
| CERS3-AS1 | 2.700362356 | 1.446372421 | 0.426201 | 3.393639 | 0.00069 | 0.002796 | Up |
| AC051619.3 | 1.744127328 | -1.548163792 | 0.446117 | -3.47031 | 0.00052 | 0.002184 | Down |
| LINC01833 | 0.938824913 | -1.771086542 | 0.72817 | -2.43224 | 0.015006 | 0.039478 | Down |
| AC025219.1 | 2.863498031 | -1.257111899 | 0.423457 | -2.96869 | 0.002991 | 0.010014 | Down |
| AC036108.1 | 9.25424265 | -1.606899395 | 0.26518 | -6.05967 | 1.36E-09 | 1.86E-08 | Down |
| AC024651.1 | 0.636810721 | -1.937408417 | 0.806453 | -2.40238 | 0.016289 | 0.042226 | Down |
| SORD2P | 962.3495751 | -1.517329459 | 0.215289 | -7.04788 | 1.82E-12 | 4.01E-11 | Down |
| AL355073.1 | 1.830595601 | -1.757952886 | 0.419671 | -4.18888 | 2.80E-05 | 0.000162 | Down |
| AC090519.2 | 1.349107169 | -1.181987169 | 0.4121 | -2.8682 | 0.004128 | 0.01324 | Down |
| UBE2Q2L | 1.217471375 | -1.548311568 | 0.454136 | -3.40936 | 0.000651 | 0.002662 | Down |
| HNRNPA1P5 | 1.326579247 | -1.134411455 | 0.439775 | -2.57953 | 0.009894 | 0.027867 | Down |
| AC079075.1 | 5.784213271 | -1.079931499 | 0.287537 | -3.7558 | 0.000173 | 0.000824 | Down |
| AC023034.1 | 2.456834242 | -1.172748586 | 0.346011 | -3.38934 | 0.000701 | 0.002832 | Down |
| AC023034.2 | 3.2226183 | -1.341156579 | 0.415291 | -3.22944 | 0.00124 | 0.004664 | Down |
| AC104574.2 | 15.4275558 | 3.882793283 | 0.722244 | 5.376013 | 7.62E-08 | 7.59E-07 | Up |
| AC104046.1 | 22.58801062 | -1.393074343 | 0.374538 | -3.71945 | 0.0002 | 0.000936 | Down |
| AC027237.4 | 2.536103249 | -2.017412594 | 0.532679 | -3.78729 | 0.000152 | 0.000738 | Down |
| DNM1P47 | 46.62977711 | -1.590342012 | 0.183229 | -8.67955 | 3.97E-18 | 1.84E-16 | Down |
| AC010478.1 | 16.59296835 | -2.226453517 | 0.416045 | -5.35147 | 8.72E-08 | 8.60E-07 | Down |
| AC084759.2 | 2.044444727 | -2.075889453 | 0.517697 | -4.00986 | 6.08E-05 | 0.000324 | Down |
| AC087612.1 | 1.080575057 | 1.77820617 | 0.437372 | 4.065659 | 4.79E-05 | 0.000263 | Up |
| AC018618.1 | 2.518152073 | 3.667343846 | 0.596498 | 6.148126 | 7.84E-10 | 1.11E-08 | Up |
| AC243562.2 | 17.15017317 | -1.374623178 | 0.215517 | -6.37827 | 1.79E-10 | 2.85E-09 | Down |
| LINC01220 | 47.20694504 | -1.502779337 | 0.204206 | -7.35914 | 1.85E-13 | 4.72E-12 | Down |
| AC107980.1 | 0.869952436 | -2.127966611 | 0.557266 | -3.81859 | 0.000134 | 0.000658 | Down |
| AC104041.1 | 3.640651994 | -1.520871166 | 0.456591 | -3.33092 | 0.000866 | 0.003412 | Down |
| LINC00677 | 1.798422672 | 1.253478242 | 0.382928 | 3.273406 | 0.001063 | 0.00408 | Up |
| AC090877.2 | 4.113723025 | -1.213969965 | 0.338146 | -3.59008 | 0.000331 | 0.001463 | Down |
| AC009269.4 | 10.91615669 | -1.074755597 | 0.226795 | -4.73889 | 2.15E-06 | 1.60E-05 | Down |
| AL596325.1 | 0.727571915 | -1.505403021 | 0.549543 | -2.73937 | 0.006156 | 0.01864 | Down |
| SLC22A31 | 1021.442245 | 6.860350646 | 0.423549 | 16.19731 | 5.27E-59 | 2.47E-55 | Up |
| AC009093.1 | 12.32195484 | 2.160126727 | 0.328409 | 6.577558 | 4.78E-11 | 8.45E-10 | Up |
| AL606760.3 | 5.266641177 | -1.432404349 | 0.267848 | -5.34782 | 8.90E-08 | 8.77E-07 | Down |
| AL355596.1 | 17.03323191 | -1.414539507 | 0.421381 | -3.35691 | 0.000788 | 0.003146 | Down |
| LINC02126 | 1.620359405 | 1.370815171 | 0.505438 | 2.712136 | 0.006685 | 0.019998 | Up |
| IGHV1OR16-2 | 0.644155883 | 1.992539616 | 0.786916 | 2.532088 | 0.011339 | 0.031202 | Up |
| AC105411.1 | 7.675933172 | 1.766472473 | 0.275039 | 6.422614 | 1.34E-10 | 2.18E-09 | Up |
| AC025259.3 | 243.7229621 | -1.594403469 | 0.281054 | -5.67295 | 1.40E-08 | 1.60E-07 | Down |
| AC068987.1 | 6.757814596 | 3.367482406 | 0.35041 | 9.610122 | 7.25E-22 | 5.21E-20 | Up |
| AC120498.1 | 21.53088996 | -1.518556248 | 0.297946 | -5.09676 | 3.46E-07 | 3.04E-06 | Down |
| AL138756.1 | 180.3766053 | 1.140639389 | 0.14691 | 7.764227 | 8.21E-15 | 2.54E-13 | Up |
| THSD4-AS1 | 3.587710383 | -1.692168092 | 0.415907 | -4.06862 | 4.73E-05 | 0.00026 | Down |
| LINC00261 | 25.77065166 | -2.67782831 | 0.40352 | -6.63617 | 3.22E-11 | 5.83E-10 | Down |
| AC017100.1 | 46.25549313 | 1.141807286 | 0.148968 | 7.664793 | 1.79E-14 | 5.26E-13 | Up |
| TGFBR3L | 18.28932122 | 3.259429831 | 0.370222 | 8.803981 | 1.32E-18 | 6.49E-17 | Up |
| AC132938.1 | 46.07711911 | -1.508469442 | 0.30338 | -4.97221 | 6.62E-07 | 5.48E-06 | Down |
| AC098934.4 | 1.047526965 | -1.463822596 | 0.554327 | -2.64072 | 0.008273 | 0.023985 | Down |
| AC076968.1 | 1.575332293 | -1.915709257 | 0.640942 | -2.98889 | 0.0028 | 0.009456 | Down |
| AC013355.1 | 2.220502515 | -1.525787621 | 0.362788 | -4.20573 | 2.60E-05 | 0.000151 | Down |
| AC112176.1 | 3.885830424 | 3.121518658 | 0.480249 | 6.499797 | 8.04E-11 | 1.37E-09 | Up |
| AC104066.2 | 1.159502581 | -1.67503 | 0.506146 | -3.30938 | 0.000935 | 0.003654 | Down |
| CSPG4P13 | 17.984824 | 1.033233936 | 0.291994 | 3.538548 | 0.000402 | 0.001741 | Up |
| AC020763.1 | 1.052261842 | 1.358952559 | 0.385549 | 3.524719 | 0.000424 | 0.001826 | Up |
| AC134312.3 | 0.524973095 | -1.292859159 | 0.529474 | -2.44178 | 0.014615 | 0.038626 | Down |
| AC093249.2 | 4.134921402 | 1.066623601 | 0.233358 | 4.57077 | 4.86E-06 | 3.35E-05 | Up |
| AC008269.2 | 1.26011559 | -1.008702165 | 0.399181 | -2.52693 | 0.011506 | 0.031592 | Down |
| AC108097.1 | 3.1143412 | -1.032967947 | 0.302688 | -3.41265 | 0.000643 | 0.002636 | Down |
| PPIAP48 | 1.646341668 | 2.007997927 | 0.777242 | 2.58349 | 0.009781 | 0.027607 | Up |
| AL138781.1 | 54.4066224 | 1.058042615 | 0.159536 | 6.632015 | 3.31E-11 | 5.98E-10 | Up |
| AC141273.1 | 0.847359872 | -2.367483902 | 0.551443 | -4.29325 | 1.76E-05 | 0.000107 | Down |
| AC084262.1 | 3.011169732 | 3.792411164 | 0.488056 | 7.770439 | 7.82E-15 | 2.43E-13 | Up |
| AP000997.2 | 2.221944272 | 3.03189941 | 0.42993 | 7.052084 | 1.76E-12 | 3.90E-11 | Up |
| LINC02562 | 22.71760438 | 1.524385726 | 0.58333 | 2.613249 | 0.008969 | 0.025682 | Up |
| AC098818.2 | 7.38375015 | -2.068657109 | 0.332723 | -6.21735 | 5.06E-10 | 7.40E-09 | Down |
| TPSP2 | 0.894439319 | 3.05583137 | 0.715959 | 4.268163 | 1.97E-05 | 0.000118 | Up |
| ARMH2 | 1.914195947 | -1.336748135 | 0.493318 | -2.70971 | 0.006734 | 0.020118 | Down |
| AC108206.1 | 19.52138255 | -3.410625204 | 0.898874 | -3.79433 | 0.000148 | 0.000719 | Down |
| AC104024.2 | 8.548084675 | 4.228877157 | 0.461654 | 9.160268 | 5.18E-20 | 3.06E-18 | Up |
| AC091544.4 | 80.00340247 | -1.157142898 | 0.207024 | -5.58943 | 2.28E-08 | 2.49E-07 | Down |
| AC068700.1 | 89.47333671 | -1.054117213 | 0.173971 | -6.05916 | 1.37E-09 | 1.86E-08 | Down |
| AC120498.3 | 26.50308268 | -1.68306387 | 0.296835 | -5.67004 | 1.43E-08 | 1.62E-07 | Down |
| AC012414.5 | 16.64418042 | -1.490586454 | 0.239687 | -6.21889 | 5.01E-10 | 7.34E-09 | Down |
| AL353746.1 | 120.9983644 | -1.997027031 | 0.370423 | -5.39121 | 7.00E-08 | 7.04E-07 | Down |
| AC127455.1 | 8.645202852 | -1.061568759 | 0.248329 | -4.27485 | 1.91E-05 | 0.000115 | Down |
| TPRKBP2 | 0.641880581 | -1.416019122 | 0.536164 | -2.64102 | 0.008266 | 0.023966 | Down |
| AL365181.1 | 2.953530937 | -1.104134977 | 0.300397 | -3.67559 | 0.000237 | 0.00109 | Down |
| AC018552.2 | 2.29229533 | -1.804907808 | 0.505448 | -3.57091 | 0.000356 | 0.001562 | Down |
| INSYN1-AS1 | 0.873541533 | -1.670967086 | 0.716217 | -2.33305 | 0.019646 | 0.04936 | Down |
| AL353719.1 | 20.46000175 | -1.155880194 | 0.178544 | -6.47393 | 9.55E-11 | 1.59E-09 | Down |
| AC116348.1 | 3.532505009 | 1.159511105 | 0.309945 | 3.741023 | 0.000183 | 0.000867 | Up |
| CISTR | 7.131347741 | -1.2179379 | 0.257336 | -4.73287 | 2.21E-06 | 1.64E-05 | Down |
| AC011773.4 | 13.07270306 | -1.145972729 | 0.225263 | -5.08725 | 3.63E-07 | 3.18E-06 | Down |
| AC009041.1 | 2.683841058 | -1.719342381 | 0.623948 | -2.75558 | 0.005859 | 0.017859 | Down |
| AC010336.2 | 2.923820962 | 2.161877454 | 0.321018 | 6.734442 | 1.65E-11 | 3.12E-10 | Up |
| BMS1P8 | 3.898465177 | -1.645117902 | 0.521615 | -3.15389 | 0.001611 | 0.005861 | Down |
| AC026771.1 | 4.12543107 | -1.22789908 | 0.315273 | -3.89471 | 9.83E-05 | 0.000498 | Down |
| AL035425.2 | 4.204039026 | -2.180920454 | 0.373898 | -5.83292 | 5.45E-09 | 6.66E-08 | Down |
| AC136618.1 | 0.471506365 | 2.362029678 | 0.772427 | 3.057932 | 0.002229 | 0.007753 | Up |
| PWRN2 | 0.650715924 | -1.943542481 | 0.559014 | -3.47673 | 0.000508 | 0.002139 | Down |
| AC126773.2 | 33.48043135 | 2.184041602 | 0.269618 | 8.100508 | 5.47E-16 | 1.95E-14 | Up |
| AC130456.3 | 3.356880837 | -1.467724241 | 0.366522 | -4.00447 | 6.22E-05 | 0.000331 | Down |
| AC011467.1 | 5.106426313 | -1.273456397 | 0.354741 | -3.58982 | 0.000331 | 0.001464 | Down |
| HMGN2P40 | 5.776342538 | -1.124314957 | 0.24127 | -4.65999 | 3.16E-06 | 2.27E-05 | Down |
| AC138028.5 | 0.986529985 | -1.573848037 | 0.456516 | -3.44752 | 0.000566 | 0.002354 | Down |
| AC142381.3 | 6.792905082 | 1.496243234 | 0.236321 | 6.331399 | 2.43E-10 | 3.79E-09 | Up |
| AC010207.1 | 65.17765477 | -1.905228238 | 0.294995 | -6.45851 | 1.06E-10 | 1.75E-09 | Down |
| AC092338.2 | 9.905699673 | -1.096509243 | 0.226529 | -4.84047 | 1.30E-06 | 1.01E-05 | Down |
| AL445531.1 | 8.429417513 | -1.525267297 | 0.2217 | -6.87988 | 5.99E-12 | 1.22E-10 | Down |
| AP007216.2 | 2.623958599 | 2.401990826 | 0.355632 | 6.754139 | 1.44E-11 | 2.76E-10 | Up |
| ANKRD20A1 | 3.673664609 | -1.325074645 | 0.288531 | -4.59249 | 4.38E-06 | 3.04E-05 | Down |
| AC026150.1 | 14.02811637 | -1.183157111 | 0.220788 | -5.35879 | 8.38E-08 | 8.30E-07 | Down |
| AC025284.1 | 1.158703638 | 2.746631639 | 0.620013 | 4.429956 | 9.43E-06 | 6.09E-05 | Up |
| LINC00543 | 5.439170209 | -1.567295464 | 0.277927 | -5.63923 | 1.71E-08 | 1.91E-07 | Down |
| AC120498.4 | 1.182960513 | 2.179116475 | 0.709244 | 3.072452 | 0.002123 | 0.007436 | Up |
| AL121839.2 | 195.295327 | -1.06187077 | 0.14415 | -7.36641 | 1.75E-13 | 4.48E-12 | Down |
| AC133552.1 | 5.464295653 | -1.586624439 | 0.253051 | -6.26999 | 3.61E-10 | 5.44E-09 | Down |
| AF067845.1 | 19.8393148 | -1.644042033 | 0.298085 | -5.51534 | 3.48E-08 | 3.68E-07 | Down |
| AC026471.3 | 26.09907367 | 1.412040483 | 0.213185 | 6.623544 | 3.51E-11 | 6.31E-10 | Up |
| AC099482.1 | 0.663432554 | -1.499152542 | 0.529106 | -2.83337 | 0.004606 | 0.014552 | Down |
| ACSM5P1 | 3.439236192 | -1.750848874 | 0.401443 | -4.36139 | 1.29E-05 | 8.11E-05 | Down |
| AC092338.3 | 8.046161598 | -1.447074918 | 0.219147 | -6.60322 | 4.02E-11 | 7.17E-10 | Down |
| Z84723.1 | 3.533903118 | -1.190723921 | 0.486427 | -2.4479 | 0.014369 | 0.038079 | Down |
| AC092803.2 | 338.3005541 | -1.279481784 | 0.175417 | -7.29396 | 3.01E-13 | 7.47E-12 | Down |
| AC009041.2 | 256.9927858 | 1.834691995 | 0.324025 | 5.662186 | 1.49E-08 | 1.69E-07 | Up |
| AC025280.1 | 2.989094966 | -1.200716144 | 0.351433 | -3.41663 | 0.000634 | 0.002603 | Down |
| AP005233.2 | 2.967332504 | 3.330252911 | 0.467767 | 7.119471 | 1.08E-12 | 2.50E-11 | Up |
| AC104072.1 | 0.583197792 | -1.865720298 | 0.603786 | -3.09004 | 0.002001 | 0.007063 | Down |
| XKR7 | 3.759003929 | 1.307217039 | 0.344205 | 3.797786 | 0.000146 | 0.00071 | Up |
| AC009093.3 | 3.589210278 | 1.031028377 | 0.42622 | 2.419006 | 0.015563 | 0.040659 | Up |
| AL158206.1 | 319.7454036 | 1.415172512 | 0.103402 | 13.68617 | 1.23E-42 | 6.98E-40 | Up |
| AC100835.1 | 2.190663679 | -1.33716962 | 0.342552 | -3.90355 | 9.48E-05 | 0.000481 | Down |
| AL031985.3 | 117.8532988 | 1.431073168 | 0.13354 | 10.71642 | 8.52E-27 | 1.02E-24 | Up |
| LINC02555 | 491.2190784 | 6.370207303 | 0.565055 | 11.27361 | 1.77E-29 | 2.84E-27 | Up |
| FOXC2-AS1 | 0.653223228 | 1.521770876 | 0.5503 | 2.765347 | 0.005686 | 0.017412 | Up |
| AC003985.2 | 1.421109206 | -1.632675688 | 0.502473 | -3.24928 | 0.001157 | 0.004388 | Down |
| AC022167.3 | 5.410163941 | -1.179719274 | 0.319531 | -3.69203 | 0.000222 | 0.001028 | Down |
| Z94057.1 | 1.91284575 | -2.234152146 | 0.816208 | -2.73723 | 0.006196 | 0.018739 | Down |
| DOCK9-DT | 349.9380705 | 1.741086406 | 0.152592 | 11.41008 | 3.72E-30 | 6.44E-28 | Up |
| BX255925.1 | 14.20633432 | 1.79629463 | 0.243354 | 7.381415 | 1.57E-13 | 4.03E-12 | Up |
| AC004847.1 | 140.5212451 | 2.236539489 | 0.403274 | 5.54596 | 2.92E-08 | 3.14E-07 | Up |
| AL008628.1 | 13.90441167 | -2.080206808 | 0.32037 | -6.49315 | 8.41E-11 | 1.42E-09 | Down |
| AC105046.1 | 30.45732185 | -1.348076166 | 0.432899 | -3.11407 | 0.001845 | 0.006583 | Down |
| LINC02544 | 19.5855718 | 1.438890398 | 0.324189 | 4.438428 | 9.06E-06 | 5.89E-05 | Up |
| AC007347.1 | 1.824110078 | -2.475982447 | 0.437464 | -5.65986 | 1.51E-08 | 1.71E-07 | Down |
| AL160286.2 | 7.112431839 | -2.012637222 | 0.315206 | -6.38515 | 1.71E-10 | 2.73E-09 | Down |
| AC124312.3 | 178.2291482 | -1.084370416 | 0.227286 | -4.77094 | 1.83E-06 | 1.38E-05 | Down |
| RUNX2-AS1 | 0.791243784 | -1.705202192 | 0.618565 | -2.75671 | 0.005839 | 0.017812 | Down |
| LINC02516 | 4.096254748 | 1.265003976 | 0.297271 | 4.255384 | 2.09E-05 | 0.000124 | Up |
| AC234775.3 | 41.09436965 | 1.219198905 | 0.217943 | 5.594115 | 2.22E-08 | 2.43E-07 | Up |
| AL133299.1 | 1.318796863 | -1.208735433 | 0.415027 | -2.91243 | 0.003586 | 0.01171 | Down |
| EPPK1 | 369.2472403 | 1.68026698 | 0.233358 | 7.200375 | 6.00E-13 | 1.44E-11 | Up |
| AC112484.3 | 23.74195566 | -1.50610546 | 0.251384 | -5.99126 | 2.08E-09 | 2.76E-08 | Down |
| LINC02188 | 3.436672569 | 1.292668987 | 0.306239 | 4.221108 | 2.43E-05 | 0.000142 | Up |
| AC134312.4 | 0.875272367 | -1.427933057 | 0.551688 | -2.5883 | 0.009645 | 0.027287 | Down |
| Z83847.1 | 11.23786932 | -1.289949483 | 0.197342 | -6.53663 | 6.29E-11 | 1.09E-09 | Down |
| AC140912.1 | 1.259528429 | -2.347508667 | 0.55908 | -4.19888 | 2.68E-05 | 0.000156 | Down |
| GOLGA8T | 11.06632924 | -1.209105712 | 0.263167 | -4.59445 | 4.34E-06 | 3.02E-05 | Down |
| AC137932.2 | 6.593026135 | 1.713159674 | 0.250053 | 6.851194 | 7.32E-12 | 1.47E-10 | Up |
| AP000821.1 | 1.212064731 | -2.191620665 | 0.539039 | -4.0658 | 4.79E-05 | 0.000263 | Down |
| ULK4P1 | 1.915854111 | -1.028824598 | 0.345938 | -2.97401 | 0.002939 | 0.009864 | Down |
| ATP2C2-AS1 | 30.29210622 | -1.294232865 | 0.187535 | -6.90127 | 5.15E-12 | 1.06E-10 | Down |
| AC110491.1 | 10.4450813 | -1.636606077 | 0.266467 | -6.14187 | 8.16E-10 | 1.15E-08 | Down |
| LINC01834 | 8.291426563 | -3.647615857 | 0.387625 | -9.41016 | 4.95E-21 | 3.28E-19 | Down |
| AC009061.1 | 2.201994316 | -2.657427696 | 0.553965 | -4.7971 | 1.61E-06 | 1.23E-05 | Down |
| AC134312.5 | 10.1062458 | 2.920630907 | 0.449381 | 6.499232 | 8.07E-11 | 1.37E-09 | Up |
| AL353803.4 | 4.642498306 | -3.035809927 | 0.407607 | -7.44789 | 9.48E-14 | 2.50E-12 | Down |
| AC010735.1 | 19.30656314 | -1.613313228 | 0.284889 | -5.66296 | 1.49E-08 | 1.68E-07 | Down |
| HSPE1P5 | 0.75719548 | 1.375852437 | 0.405767 | 3.390741 | 0.000697 | 0.00282 | Up |
| AL031710.1 | 239.4056112 | -1.325762012 | 0.264709 | -5.00838 | 5.49E-07 | 4.62E-06 | Down |
| AL035425.3 | 303.4778673 | -1.499300275 | 0.309243 | -4.8483 | 1.25E-06 | 9.72E-06 | Down |
| AL161938.1 | 2.146636186 | -2.141957388 | 0.443902 | -4.82529 | 1.40E-06 | 1.08E-05 | Down |
| AL022069.1 | 18.30994344 | -1.327640547 | 0.196968 | -6.7404 | 1.58E-11 | 3.01E-10 | Down |
| DPPA2P4 | 1.436092249 | -1.582781377 | 0.522676 | -3.02823 | 0.00246 | 0.008455 | Down |
| LINC01613 | 1.092557008 | -1.648194787 | 0.573415 | -2.87435 | 0.004049 | 0.013018 | Down |
| AL157394.1 | 41.97391238 | 1.204884411 | 0.179241 | 6.722162 | 1.79E-11 | 3.37E-10 | Up |
| AC009132.1 | 14.17305816 | -1.917520878 | 0.288203 | -6.65336 | 2.86E-11 | 5.23E-10 | Down |
| GOLGA8M.1 | 6.493443049 | -1.471616941 | 0.320873 | -4.5863 | 4.51E-06 | 3.13E-05 | Down |
| AC040174.1 | 9.334898169 | 1.075592482 | 0.350059 | 3.072605 | 0.002122 | 0.007433 | Up |
| AC005086.1 | 4.46422923 | -1.884651802 | 0.361876 | -5.208 | 1.91E-07 | 1.76E-06 | Down |
| AC027279.2 | 2.271942088 | -1.503701806 | 0.412188 | -3.6481 | 0.000264 | 0.0012 | Down |
| AC096996.2 | 2.470896902 | 4.96183516 | 0.758446 | 6.542104 | 6.07E-11 | 1.05E-09 | Up |
| AC010931.3 | 0.543699932 | 1.279445271 | 0.543036 | 2.356098 | 0.018468 | 0.046831 | Up |
| AC068987.4 | 58.29861082 | 1.146573796 | 0.182042 | 6.298415 | 3.01E-10 | 4.60E-09 | Up |
| LINC01858 | 0.863766291 | 2.398238464 | 0.452282 | 5.302534 | 1.14E-07 | 1.10E-06 | Up |
| LINC02177 | 3.127228958 | -1.381278641 | 0.3648 | -3.7864 | 0.000153 | 0.00074 | Down |
| AC026992.2 | 4.386252865 | -1.377676916 | 0.304218 | -4.52858 | 5.94E-06 | 4.01E-05 | Down |
| AC093849.1 | 2.771682106 | -1.44317747 | 0.330425 | -4.36763 | 1.26E-05 | 7.90E-05 | Down |
| AC134682.1 | 21.40833944 | -1.927201126 | 0.24828 | -7.7622 | 8.35E-15 | 2.57E-13 | Down |
| LINC00165 | 1.294113204 | 3.751169674 | 0.588099 | 6.378469 | 1.79E-10 | 2.85E-09 | Up |
| AL138690.1 | 2.123821805 | 2.232408763 | 0.394815 | 5.654321 | 1.56E-08 | 1.76E-07 | Up |
| AC002551.1 | 1.303186743 | -1.499373655 | 0.52318 | -2.86588 | 0.004158 | 0.013324 | Down |
| LINC00922 | 2.306034734 | 3.636068319 | 0.609222 | 5.96838 | 2.40E-09 | 3.13E-08 | Up |
| AC008555.1 | 57.16843205 | 1.107905934 | 0.152769 | 7.252163 | 4.10E-13 | 1.00E-11 | Up |
| AC006504.1 | 50.61133121 | -1.277254841 | 0.22553 | -5.66334 | 1.48E-08 | 1.68E-07 | Down |
| AC006058.1 | 2884.635089 | -1.453334874 | 0.213489 | -6.80756 | 9.93E-12 | 1.95E-10 | Down |
| AC093627.7 | 3.296378187 | 1.435996387 | 0.465673 | 3.083701 | 0.002044 | 0.007193 | Up |
| AC084782.2 | 3.802486972 | -1.246206098 | 0.364635 | -3.41768 | 0.000632 | 0.002594 | Down |
| AC046158.2 | 1.49043739 | -1.657092864 | 0.436067 | -3.80009 | 0.000145 | 0.000704 | Down |
| AC092718.6 | 7.191355033 | -1.301149554 | 0.235008 | -5.53661 | 3.08E-08 | 3.30E-07 | Down |
| MFSD1P1 | 1.408868992 | -1.041203575 | 0.420871 | -2.47392 | 0.013364 | 0.035855 | Down |
| AC116025.1 | 0.5077095 | -1.273316293 | 0.503062 | -2.53113 | 0.011369 | 0.031278 | Down |
| SNORD3B-2 | 18.54771348 | -2.8904792 | 0.403813 | -7.15796 | 8.19E-13 | 1.92E-11 | Down |
| AC139099.1 | 2.381752387 | 1.298233514 | 0.318643 | 4.074257 | 4.62E-05 | 0.000254 | Up |
| AC087392.2 | 2.024910132 | 1.005427745 | 0.420192 | 2.392779 | 0.016721 | 0.043157 | Up |
| LINC00514 | 14.80976992 | 2.142520692 | 0.314953 | 6.802673 | 1.03E-11 | 2.01E-10 | Up |
| LINC01978 | 2.659001095 | 1.490833297 | 0.297982 | 5.003102 | 5.64E-07 | 4.74E-06 | Up |
| AL157931.1 | 19.77202882 | 1.263246073 | 0.235363 | 5.367229 | 8.00E-08 | 7.94E-07 | Up |
| AC007952.4 | 14.42459746 | -2.859537044 | 0.390542 | -7.32196 | 2.44E-13 | 6.14E-12 | Down |
| AC129507.2 | 24.73838295 | 1.051127994 | 0.21739 | 4.835216 | 1.33E-06 | 1.03E-05 | Up |
| AC133065.2 | 12.54080688 | -1.787074723 | 0.568652 | -3.14265 | 0.001674 | 0.006054 | Down |
| AC104581.1 | 1.719110142 | -1.641975737 | 0.446202 | -3.67989 | 0.000233 | 0.001073 | Down |
| AC099489.2 | 2.584145436 | 1.478465043 | 0.318773 | 4.637983 | 3.52E-06 | 2.50E-05 | Up |
| AC040160.1 | 47.46848062 | 1.772173553 | 0.183474 | 9.659013 | 4.50E-22 | 3.32E-20 | Up |
| MRPS21P9 | 0.840014129 | -1.785688625 | 0.533519 | -3.347 | 0.000817 | 0.003244 | Down |
| LINC01977 | 32.76144211 | 3.628100095 | 0.279132 | 12.99777 | 1.26E-38 | 4.59E-36 | Up |
| U91319.1 | 1.413708247 | 1.178561902 | 0.497317 | 2.369842 | 0.017796 | 0.045433 | Up |
| AC012146.4 | 2.262417471 | 1.560936891 | 0.604549 | 2.581984 | 0.009823 | 0.027705 | Up |
| C19orf84 | 7.058590175 | 1.956780913 | 0.364861 | 5.363091 | 8.18E-08 | 8.12E-07 | Up |
| AC015921.1 | 13.66765985 | 2.099833715 | 0.196095 | 10.70822 | 9.31E-27 | 1.11E-24 | Up |
| AC027796.4 | 47.36219153 | 1.187008775 | 0.199816 | 5.940498 | 2.84E-09 | 3.67E-08 | Up |
| AC129507.4 | 5.293060614 | 1.69413346 | 0.346257 | 4.892702 | 9.95E-07 | 7.94E-06 | Up |
| AC137056.1 | 5.217785461 | -2.556682949 | 0.362123 | -7.06027 | 1.66E-12 | 3.69E-11 | Down |
| AF001548.1 | 1.935258241 | -1.160720492 | 0.40384 | -2.87421 | 0.00405 | 0.013023 | Down |
| AC009121.2 | 10.25659741 | 1.034562856 | 0.293655 | 3.52306 | 0.000427 | 0.001835 | Up |
| AC007114.2 | 27.89509744 | -1.133936021 | 0.183057 | -6.19443 | 5.85E-10 | 8.45E-09 | Down |
| AC007638.2 | 4.378142331 | -1.565132781 | 0.311804 | -5.0196 | 5.18E-07 | 4.39E-06 | Down |
| LINC01896 | 1.523326009 | -2.54198674 | 0.868137 | -2.9281 | 0.00341 | 0.011225 | Down |
| AC110285.3 | 8.87181447 | 2.391649995 | 0.31245 | 7.654516 | 1.94E-14 | 5.67E-13 | Up |
| MYZAP | 90.02676596 | -1.001790348 | 0.14723 | -6.80425 | 1.02E-11 | 2.00E-10 | Down |
| AC115099.1 | 3.054312159 | 1.130519666 | 0.329356 | 3.432512 | 0.000598 | 0.002474 | Up |
| MTND1P8 | 6.873139512 | -1.109638169 | 0.275024 | -4.0347 | 5.47E-05 | 0.000296 | Down |
| AC119396.1 | 46.64685312 | -1.266500052 | 0.361234 | -3.50604 | 0.000455 | 0.001943 | Down |
| LINC01975 | 32.52617145 | -1.988841381 | 0.328421 | -6.05576 | 1.40E-09 | 1.90E-08 | Down |
| TMEM238L | 3.424400901 | -1.777101556 | 0.38607 | -4.60306 | 4.16E-06 | 2.91E-05 | Down |
| AC100872.1 | 0.589441972 | 2.928104504 | 1.175386 | 2.491184 | 0.012732 | 0.034395 | Up |
| ANTXRLP1 | 8.750627616 | -1.234635993 | 0.501378 | -2.46249 | 0.013798 | 0.0368 | Down |
| AP005121.1 | 1.16365308 | 1.706403574 | 0.501898 | 3.3999 | 0.000674 | 0.002741 | Up |
| MSMB | 2.833152424 | 4.945474105 | 0.697918 | 7.086034 | 1.38E-12 | 3.12E-11 | Up |
| AL035696.3 | 3.874176739 | -1.67015857 | 0.382553 | -4.36583 | 1.27E-05 | 7.95E-05 | Down |
| MIR3197 | 1.092747999 | -1.953961102 | 0.52601 | -3.71468 | 0.000203 | 0.000951 | Down |
| LINC02210-CRHR1 | 0.446126659 | -1.411352557 | 0.560988 | -2.51583 | 0.011875 | 0.032469 | Down |
| AC068594.1 | 4.566727753 | 2.232071448 | 0.248184 | 8.993629 | 2.39E-19 | 1.30E-17 | Up |
| MIR5684 | 0.85031082 | -1.273293082 | 0.501882 | -2.53704 | 0.01118 | 0.030821 | Down |
| AC009831.1 | 47.93145836 | 1.044276237 | 0.128585 | 8.121288 | 4.61E-16 | 1.66E-14 | Up |
| MTND2P13 | 0.582610603 | 1.946411126 | 0.653735 | 2.977369 | 0.002907 | 0.009769 | Up |
| SNORD3A | 27.70091941 | -3.029606404 | 0.374723 | -8.08493 | 6.22E-16 | 2.20E-14 | Down |
| AC091138.1 | 2.499797604 | 3.535899611 | 0.653178 | 5.413379 | 6.18E-08 | 6.29E-07 | Up |
| RN7SL121P | 1.062939119 | -1.323496082 | 0.50564 | -2.61747 | 0.008859 | 0.025413 | Down |
| MIR5685 | 6.166862505 | -1.683343889 | 0.445807 | -3.77595 | 0.000159 | 0.000768 | Down |
| AC005291.1 | 1.286442572 | 1.372138246 | 0.54602 | 2.512981 | 0.011972 | 0.03268 | Up |
| MIR4795 | 0.800486426 | -1.51596632 | 0.505631 | -2.99817 | 0.002716 | 0.009203 | Down |
| AC005552.1 | 0.548042239 | -1.972914669 | 0.605028 | -3.26086 | 0.001111 | 0.004236 | Down |
| MIR3189 | 4.483143821 | 4.250393076 | 0.488225 | 8.705805 | 3.15E-18 | 1.48E-16 | Up |
| MAGOH2P | 43.29416914 | -1.045621858 | 0.171517 | -6.09632 | 1.09E-09 | 1.50E-08 | Down |
| AC011120.1 | 0.777837932 | -1.438662618 | 0.497066 | -2.89431 | 0.0038 | 0.012317 | Down |
| RNU4ATAC | 24.49827891 | -3.13233296 | 0.326969 | -9.57991 | 9.71E-22 | 6.88E-20 | Down |
| MIR4767 | 1.106433368 | -2.155189367 | 0.641187 | -3.36125 | 0.000776 | 0.003101 | Down |
| AC087749.1 | 1.473744017 | -1.465531479 | 0.415357 | -3.52837 | 0.000418 | 0.001804 | Down |
| AC107982.2 | 0.984666733 | -1.333143154 | 0.447547 | -2.97877 | 0.002894 | 0.009729 | Down |
| RN7SL431P | 2.720058261 | -1.055785354 | 0.36138 | -2.92154 | 0.003483 | 0.01143 | Down |
| MIR4653 | 2.704923249 | 1.044303729 | 0.279684 | 3.733872 | 0.000189 | 0.000889 | Up |
| MIR548AR | 1.306957337 | 1.201937847 | 0.382884 | 3.139168 | 0.001694 | 0.006115 | Up |
| MIR4257 | 1.037255924 | -1.304154009 | 0.531966 | -2.45157 | 0.014223 | 0.037756 | Down |
| DCXR-DT | 8.202165299 | -1.057456321 | 0.214555 | -4.92861 | 8.28E-07 | 6.72E-06 | Down |
| RN7SL15P | 5.502828997 | -1.423246448 | 0.321989 | -4.42018 | 9.86E-06 | 6.34E-05 | Down |
| AC005726.3 | 139.2933695 | -1.283295932 | 0.172894 | -7.42245 | 1.15E-13 | 3.01E-12 | Down |
| AC064805.2 | 3.047857625 | 2.601206336 | 0.452971 | 5.742541 | 9.33E-09 | 1.09E-07 | Up |
| KIAA0895LP1 | 3.164831289 | -1.195167328 | 0.346851 | -3.44577 | 0.000569 | 0.002366 | Down |
| MIR3117 | 0.779716482 | -1.244059732 | 0.483323 | -2.57397 | 0.010054 | 0.02823 | Down |
| AC005725.1 | 34.92142435 | 3.26360446 | 0.305927 | 10.66791 | 1.44E-26 | 1.68E-24 | Up |
| AC145207.8 | 10.29139843 | -1.223636573 | 0.25924 | -4.7201 | 2.36E-06 | 1.74E-05 | Down |
| MIR3616 | 1.146932919 | -1.324717233 | 0.440135 | -3.0098 | 0.002614 | 0.00891 | Down |
| AC015845.1 | 1.062531422 | -1.680179359 | 0.490627 | -3.42456 | 0.000616 | 0.002537 | Down |
| MIR3138 | 2.410429939 | -1.272975858 | 0.425495 | -2.99175 | 0.002774 | 0.009378 | Down |
| PRR29-AS1 | 29.42054709 | 1.651044354 | 0.230741 | 7.155395 | 8.34E-13 | 1.95E-11 | Up |
| GJA5 | 469.246669 | 1.044748772 | 0.15931 | 6.557965 | 5.45E-11 | 9.55E-10 | Up |
| AC068594.2 | 0.481230651 | 1.908885113 | 0.680025 | 2.80708 | 0.004999 | 0.015614 | Up |
| LRRC37A7P | 25.1026008 | 1.578603753 | 0.23751 | 6.646486 | 3.00E-11 | 5.46E-10 | Up |
| SNORD3B-1 | 29.34410432 | -4.042407649 | 0.488032 | -8.28308 | 1.20E-16 | 4.72E-15 | Down |
| ANXA8 | 6.750775258 | 2.036664544 | 0.52528 | 3.877294 | 0.000106 | 0.000532 | Up |
| AP005263.1 | 0.504772 | -1.271718766 | 0.517573 | -2.45708 | 0.014007 | 0.037295 | Down |
| FEM1AP2 | 9.756049785 | 1.348744585 | 0.262846 | 5.131319 | 2.88E-07 | 2.57E-06 | Up |
| MIR5188 | 1.470644118 | -2.229198691 | 0.53688 | -4.15214 | 3.29E-05 | 0.000187 | Down |
| LINC01899 | 1.480950211 | -2.611207659 | 0.619871 | -4.2125 | 2.53E-05 | 0.000147 | Down |
| AC104984.2 | 1.509096765 | -1.038019594 | 0.389426 | -2.66551 | 0.007687 | 0.022511 | Down |
| MIR3128 | 0.457142021 | -1.197345597 | 0.513578 | -2.33138 | 0.019733 | 0.049569 | Down |
| AP001021.1 | 3.457792985 | -1.285632393 | 0.303023 | -4.24268 | 2.21E-05 | 0.000131 | Down |
| FCGR1CP | 11.05561688 | 1.014305517 | 0.364584 | 2.782092 | 0.005401 | 0.016676 | Up |
| AC023301.1 | 21.27043837 | 2.127769111 | 0.415667 | 5.118928 | 3.07E-07 | 2.73E-06 | Up |
| AC106037.2 | 6.603695046 | -1.06984738 | 0.35078 | -3.04991 | 0.002289 | 0.007932 | Down |
| MIR4664 | 57.28425937 | -1.121397331 | 0.207368 | -5.40776 | 6.38E-08 | 6.47E-07 | Down |
| RARA-AS1 | 221.8477878 | 1.266455276 | 0.156874 | 8.073091 | 6.85E-16 | 2.41E-14 | Up |
| AC005821.1 | 2.022219034 | 1.301326749 | 0.49511 | 2.628357 | 0.00858 | 0.024729 | Up |
| AP001099.1 | 2.110627545 | -2.278203846 | 0.573099 | -3.97523 | 7.03E-05 | 0.000369 | Down |
| AP006565.1 | 4.621243162 | 4.22007474 | 0.552937 | 7.63211 | 2.31E-14 | 6.66E-13 | Up |
| LINC01900 | 0.982016775 | -2.040632701 | 0.811402 | -2.51495 | 0.011905 | 0.032537 | Down |
| LINC00668 | 3.629806132 | -3.502788102 | 0.763261 | -4.58924 | 4.45E-06 | 3.09E-05 | Down |
| AC005828.5 | 0.966308828 | 1.477252995 | 0.437742 | 3.374713 | 0.000739 | 0.002966 | Up |
| ESRG | 1.293369276 | -1.261279922 | 0.519663 | -2.42711 | 0.01522 | 0.039949 | Down |
| AC004585.1 | 125.6401301 | -1.429847359 | 0.472246 | -3.02776 | 0.002464 | 0.008465 | Down |
| AC103809.1 | 3.648301806 | 1.185493931 | 0.260597 | 4.549151 | 5.39E-06 | 3.67E-05 | Up |
| MIR3178 | 4.892936864 | -1.44935801 | 0.260857 | -5.55615 | 2.76E-08 | 2.98E-07 | Down |
| AC005324.5 | 2.451490417 | -1.011926827 | 0.31202 | -3.24315 | 0.001182 | 0.004472 | Down |
| AP005205.2 | 19.47992555 | -1.214771451 | 0.277214 | -4.38207 | 1.18E-05 | 7.44E-05 | Down |
| AL449423.1 | 0.378513531 | 2.160977558 | 0.73847 | 2.92629 | 0.00343 | 0.01128 | Up |
| AP001178.3 | 33.43079577 | -1.420770505 | 0.217329 | -6.53743 | 6.26E-11 | 1.08E-09 | Down |
| GDF10 | 590.8607558 | -1.342225919 | 0.302509 | -4.43698 | 9.12E-06 | 5.93E-05 | Down |
| MIR4530 | 1.354406986 | -1.170144986 | 0.471094 | -2.48389 | 0.012996 | 0.035 | Down |
| AC005304.3 | 1.119061536 | -1.815753556 | 0.472739 | -3.84092 | 0.000123 | 0.000607 | Down |
| GAPLINC | 9.571000493 | 1.352879278 | 0.218182 | 6.200681 | 5.62E-10 | 8.15E-09 | Up |
| ITM2BP1 | 2.195175245 | -1.292146158 | 0.407815 | -3.16846 | 0.001532 | 0.005621 | Down |
| AC007923.4 | 1.589713424 | -1.204179626 | 0.397359 | -3.03046 | 0.002442 | 0.008401 | Down |
| AP000902.1 | 7.530116144 | -1.343726126 | 0.426649 | -3.14949 | 0.001636 | 0.005933 | Down |
| AC005746.1 | 6.257751988 | -1.203113421 | 0.232571 | -5.17309 | 2.30E-07 | 2.10E-06 | Down |
| AP001010.1 | 3.313978877 | 1.178557373 | 0.284314 | 4.145273 | 3.39E-05 | 0.000192 | Up |
| AC012254.1 | 4.321170524 | -1.323795659 | 0.286592 | -4.61909 | 3.85E-06 | 2.71E-05 | Down |
| FXYD1 | 54.75405252 | -1.187624678 | 0.189977 | -6.25141 | 4.07E-10 | 6.07E-09 | Down |
| AC023421.1 | 155.9929312 | 1.303533417 | 0.406824 | 3.204168 | 0.001355 | 0.005044 | Up |
| FARSA-AS1 | 1.221212968 | -1.039578923 | 0.399866 | -2.59982 | 0.009327 | 0.026539 | Down |
| DHX40P1 | 4.72837512 | -1.458435997 | 0.513717 | -2.83899 | 0.004526 | 0.014335 | Down |
| AC020663.1 | 2.202516018 | -1.563386927 | 0.385573 | -4.05471 | 5.02E-05 | 0.000274 | Down |
| AP001542.1 | 2.61980777 | 1.147084409 | 0.438733 | 2.614537 | 0.008935 | 0.025605 | Up |
| LINC01929 | 9.168672561 | 1.610681369 | 0.286464 | 5.622625 | 1.88E-08 | 2.09E-07 | Up |
| LINC01532 | 10.73333293 | -2.076147976 | 0.296878 | -6.99327 | 2.69E-12 | 5.79E-11 | Down |
| AL136084.2 | 7.65215844 | 1.25426394 | 0.200112 | 6.267809 | 3.66E-10 | 5.51E-09 | Up |
| AC100793.2 | 1.283169738 | 1.300154612 | 0.393088 | 3.307543 | 0.000941 | 0.003671 | Up |
| AC005050.1 | 1.109848288 | -1.688693307 | 0.479814 | -3.51947 | 0.000432 | 0.001857 | Down |
| LINC01905 | 7.431778758 | -2.512610926 | 0.356259 | -7.05276 | 1.75E-12 | 3.88E-11 | Down |
| LINC02080 | 5.385651642 | 1.173460376 | 0.285273 | 4.113472 | 3.90E-05 | 0.000218 | Up |
| AP001269.2 | 5.134056675 | -1.073208865 | 0.325921 | -3.29285 | 0.000992 | 0.003846 | Down |
| AP001029.1 | 10.01221842 | 2.703501159 | 0.300992 | 8.981973 | 2.66E-19 | 1.44E-17 | Up |
| AC008105.3 | 221.2116667 | 1.246089835 | 0.378301 | 3.293907 | 0.000988 | 0.003833 | Up |
| AC008738.2 | 13.1484003 | -1.486346966 | 0.213644 | -6.9571 | 3.47E-12 | 7.39E-11 | Down |
| AC005746.2 | 8.43002752 | -1.003022504 | 0.405178 | -2.47551 | 0.013305 | 0.035709 | Down |
| LINC01842 | 1.209432788 | 1.386811614 | 0.421488 | 3.290274 | 0.001001 | 0.003876 | Up |
| AC007993.1 | 16.26488576 | 1.338472251 | 0.293331 | 4.563012 | 5.04E-06 | 3.46E-05 | Up |
| PHF5CP | 2.661879035 | -2.045857808 | 0.370699 | -5.51892 | 3.41E-08 | 3.61E-07 | Down |
| AP001029.2 | 36.26916982 | 2.270498375 | 0.226258 | 10.03501 | 1.07E-23 | 9.51E-22 | Up |
| LCN6 | 141.0718588 | 3.207129934 | 0.30662 | 10.45962 | 1.32E-25 | 1.41E-23 | Up |
| AC092068.1 | 8.766628957 | -1.587298638 | 0.348493 | -4.55475 | 5.24E-06 | 3.58E-05 | Down |
| AC005597.1 | 0.71648224 | -1.813251991 | 0.578403 | -3.13493 | 0.001719 | 0.006194 | Down |
| AC005381.1 | 3.62798096 | -1.454972288 | 0.346673 | -4.19695 | 2.71E-05 | 0.000157 | Down |
| AC008805.1 | 0.742476086 | -1.952685279 | 0.692034 | -2.82166 | 0.004778 | 0.014998 | Down |
| AC055813.1 | 4.54707482 | -1.094593049 | 0.264371 | -4.14037 | 3.47E-05 | 0.000196 | Down |
| AC011444.2 | 3.118945786 | -1.516436447 | 0.365518 | -4.14873 | 3.34E-05 | 0.00019 | Down |
| AC020911.2 | 1.895059117 | -1.997254824 | 0.472568 | -4.22639 | 2.37E-05 | 0.000139 | Down |
| AC090409.1 | 21.62239922 | 1.667998947 | 0.233367 | 7.147528 | 8.84E-13 | 2.06E-11 | Up |
| AC008649.1 | 2.973053456 | 1.948285524 | 0.494247 | 3.941924 | 8.08E-05 | 0.000418 | Up |
| AC011444.3 | 3.737902179 | -1.204518522 | 0.328345 | -3.66846 | 0.000244 | 0.001117 | Down |
| AC090409.2 | 0.650705081 | 1.302098933 | 0.492378 | 2.644513 | 0.008181 | 0.02375 | Up |
| AC009271.1 | 1.493632765 | -2.425105503 | 0.453457 | -5.34804 | 8.89E-08 | 8.76E-07 | Down |
| SEC24AP1 | 2.299404806 | -1.056739259 | 0.433425 | -2.43811 | 0.014764 | 0.038947 | Down |
| AC022706.1 | 46.28323408 | 1.195730745 | 0.257475 | 4.644065 | 3.42E-06 | 2.43E-05 | Up |
| KCNJ2-AS1 | 160.8933261 | 1.825499594 | 0.151863 | 12.02073 | 2.77E-33 | 6.07E-31 | Up |
| AC005330.1 | 4.70999757 | 1.399376448 | 0.307993 | 4.543538 | 5.53E-06 | 3.76E-05 | Up |
| AC016205.1 | 9.934960977 | 2.111161501 | 0.270568 | 7.802701 | 6.06E-15 | 1.91E-13 | Up |
| AC008649.2 | 2.667017931 | -3.282013413 | 0.496424 | -6.61131 | 3.81E-11 | 6.82E-10 | Down |
| AC011447.3 | 209.4115941 | -1.066199124 | 0.23531 | -4.53103 | 5.87E-06 | 3.97E-05 | Down |
| AC006305.2 | 10.97528962 | -1.892012751 | 0.26829 | -7.05213 | 1.76E-12 | 3.90E-11 | Down |
| AC068473.4 | 1.523953965 | 1.546471245 | 0.449401 | 3.441182 | 0.000579 | 0.002403 | Up |
| AC092068.2 | 2.87297651 | -1.006499693 | 0.309995 | -3.24683 | 0.001167 | 0.004423 | Down |
| AC010641.1 | 5.691638196 | -2.249734623 | 0.856408 | -2.62694 | 0.008616 | 0.024813 | Down |
| AC079210.1 | 4.404356723 | 1.751098001 | 0.364765 | 4.800619 | 1.58E-06 | 1.21E-05 | Up |
| AC021683.1 | 8.566153883 | 1.492574677 | 0.22939 | 6.506723 | 7.68E-11 | 1.31E-09 | Up |
| ZNF571-AS1 | 102.3460681 | 1.343639268 | 0.182354 | 7.368289 | 1.73E-13 | 4.42E-12 | Up |
| LINC01837 | 2.016608829 | -1.461829873 | 0.459548 | -3.18101 | 0.001468 | 0.00541 | Down |
| FAM215A | 10.90545861 | 1.022024533 | 0.292216 | 3.497492 | 0.00047 | 0.001996 | Up |
| NFE2L3P1 | 1.261568946 | 1.12709327 | 0.374593 | 3.008849 | 0.002622 | 0.008933 | Up |
| AC021683.2 | 8.133423116 | 1.181603668 | 0.206433 | 5.723908 | 1.04E-08 | 1.21E-07 | Up |
| AP001029.3 | 2.144582546 | 1.162782132 | 0.305467 | 3.806568 | 0.000141 | 0.000688 | Up |
| LINC01864 | 0.633556307 | 1.113894757 | 0.43438 | 2.564334 | 0.010337 | 0.028883 | Up |
| LINC01836 | 67.11326492 | -1.525164094 | 0.214955 | -7.09529 | 1.29E-12 | 2.94E-11 | Down |
| LINC00868 | 0.598037078 | 1.949148593 | 0.54874 | 3.552045 | 0.000382 | 0.001664 | Up |
| AC005264.1 | 4.013568738 | 1.832224647 | 0.250159 | 7.324249 | 2.40E-13 | 6.05E-12 | Up |
| AC007998.3 | 37.56145267 | -1.92190661 | 0.205306 | -9.36119 | 7.88E-21 | 5.12E-19 | Down |
| LINC01028 | 1.269281259 | -1.401618098 | 0.467558 | -2.99774 | 0.00272 | 0.009215 | Down |
| AC011511.5 | 25.25036442 | 1.08187299 | 0.335536 | 3.224317 | 0.001263 | 0.004742 | Up |
| AC040904.1 | 0.608895286 | 1.259440537 | 0.440332 | 2.860204 | 0.004234 | 0.013538 | Up |
| AC091198.1 | 0.789476811 | -1.61539206 | 0.478501 | -3.37594 | 0.000736 | 0.002954 | Down |
| AC008543.5 | 2.392638159 | -1.068729329 | 0.323048 | -3.30827 | 0.000939 | 0.003664 | Down |
| AC008742.1 | 0.99707499 | 1.059616602 | 0.446037 | 2.375625 | 0.017519 | 0.044834 | Up |
| AC002546.1 | 9.098690843 | -1.84194332 | 0.455424 | -4.04446 | 5.24E-05 | 0.000285 | Down |
| AC025809.1 | 12.75016101 | -1.163801328 | 0.206612 | -5.63279 | 1.77E-08 | 1.98E-07 | Down |
| LINC01539 | 108.38802 | -1.877703943 | 0.254595 | -7.37526 | 1.64E-13 | 4.21E-12 | Down |
| AC012254.3 | 28.4260983 | -1.266721818 | 0.212564 | -5.95925 | 2.53E-09 | 3.30E-08 | Down |
| AC006305.3 | 18.34423373 | -1.821746502 | 0.250204 | -7.28105 | 3.31E-13 | 8.18E-12 | Down |
| AC107896.1 | 0.688866188 | -1.790574135 | 0.499215 | -3.58678 | 0.000335 | 0.00148 | Down |
| AC104985.1 | 8.103944075 | -2.865600658 | 0.322874 | -8.87529 | 6.98E-19 | 3.58E-17 | Down |
| AC092068.3 | 4.823274153 | -1.407273299 | 0.334407 | -4.20827 | 2.57E-05 | 0.00015 | Down |
| RUNDC3A-AS1 | 222.7793401 | 1.693053019 | 0.142192 | 11.90682 | 1.09E-32 | 2.29E-30 | Up |
| MIR4527HG | 4.162434138 | 2.635965695 | 0.490592 | 5.373027 | 7.74E-08 | 7.71E-07 | Up |
| AC100793.3 | 2.617598778 | 1.832200068 | 0.441002 | 4.154634 | 3.26E-05 | 0.000185 | Up |
| AC022726.1 | 0.565948242 | -1.366030318 | 0.555249 | -2.46021 | 0.013885 | 0.037006 | Down |
| AC011447.4 | 1.772472076 | -1.386167784 | 0.409993 | -3.38096 | 0.000722 | 0.002909 | Down |
| LINC01791 | 0.64544243 | -1.419016169 | 0.549574 | -2.58203 | 0.009822 | 0.027705 | Down |
| SMIM22 | 45.76426304 | 2.24695433 | 0.375392 | 5.985628 | 2.16E-09 | 2.84E-08 | Up |
| MAN1A2P1 | 29.63953642 | -2.398347475 | 0.353882 | -6.77724 | 1.22E-11 | 2.38E-10 | Down |
| AL031666.2 | 13.29422274 | -1.696291442 | 0.264081 | -6.42337 | 1.33E-10 | 2.17E-09 | Down |
| AC007785.1 | 0.993459891 | 2.155849564 | 0.534251 | 4.035274 | 5.45E-05 | 0.000295 | Up |
| MIR3188 | 1.365300614 | -1.696815406 | 0.454251 | -3.73541 | 0.000187 | 0.000884 | Down |
| AC011523.1 | 3.52312643 | -1.799906062 | 0.332841 | -5.4077 | 6.38E-08 | 6.47E-07 | Down |
| LINC01785 | 11.25484661 | -2.677767359 | 0.437616 | -6.11898 | 9.42E-10 | 1.31E-08 | Down |
| BNIP3P40 | 2.051458838 | -1.273500716 | 0.339116 | -3.75535 | 0.000173 | 0.000825 | Down |
| AC016885.3 | 0.970813183 | -1.604394005 | 0.494813 | -3.24243 | 0.001185 | 0.004483 | Down |
| GABRQ | 24.02939486 | 1.476105159 | 0.341066 | 4.327911 | 1.51E-05 | 9.27E-05 | Up |
| SLC6A14 | 92.19983471 | 3.370971372 | 0.433168 | 7.782128 | 7.13E-15 | 2.22E-13 | Up |
| ARL14EPL | 3.599404007 | 2.355081638 | 0.478686 | 4.919885 | 8.66E-07 | 7.00E-06 | Up |
| AC011445.1 | 4.046679565 | -1.238238993 | 0.275147 | -4.50028 | 6.79E-06 | 4.53E-05 | Down |
| AC123912.3 | 4.908287395 | -1.115303166 | 0.30105 | -3.70472 | 0.000212 | 0.000984 | Down |
| LINC02560 | 45.40622881 | 3.238854544 | 0.300928 | 10.7629 | 5.15E-27 | 6.44E-25 | Up |
| AL132655.1 | 2.204997758 | -1.062451991 | 0.330621 | -3.2135 | 0.001311 | 0.004901 | Down |
| AC243960.3 | 23.20312383 | -1.462487278 | 0.292857 | -4.99386 | 5.92E-07 | 4.95E-06 | Down |
| AC010271.1 | 15.78223365 | -1.604650443 | 0.241757 | -6.63746 | 3.19E-11 | 5.79E-10 | Down |
| AC011473.1 | 0.91595677 | 3.456820894 | 0.817871 | 4.226608 | 2.37E-05 | 0.000139 | Up |
| MTDHP3 | 1.638768392 | -1.799431024 | 0.503857 | -3.57132 | 0.000355 | 0.00156 | Down |
| AC006262.1 | 17.28890911 | 3.326849027 | 0.386691 | 8.603375 | 7.74E-18 | 3.43E-16 | Up |
| MTDHP4 | 1.167444642 | 1.702104347 | 0.522169 | 3.259683 | 0.001115 | 0.004252 | Up |
| AC078899.3 | 100.5628587 | -1.345935191 | 0.212087 | -6.34614 | 2.21E-10 | 3.47E-09 | Down |
| IGFL2-AS1 | 2.470568939 | 3.208753183 | 0.486435 | 6.59647 | 4.21E-11 | 7.48E-10 | Up |
| LETM1P2 | 13.74233761 | -1.029641619 | 0.186607 | -5.51771 | 3.43E-08 | 3.64E-07 | Down |
| AC016586.1 | 18.32160293 | -1.128279752 | 0.208909 | -5.40081 | 6.63E-08 | 6.70E-07 | Down |
| ZNF723 | 0.62399325 | 1.379553344 | 0.532111 | 2.592602 | 0.009525 | 0.02701 | Up |
| AL158151.4 | 4.025813374 | 1.609559038 | 0.313195 | 5.139157 | 2.76E-07 | 2.47E-06 | Up |
| AC005261.4 | 1.464571001 | -1.059281561 | 0.44982 | -2.3549 | 0.018528 | 0.046954 | Down |
| LINC01081 | 1.001785648 | -1.148831613 | 0.459905 | -2.49798 | 0.01249 | 0.033858 | Down |
| ADGRE4P | 21.07895192 | 1.214035608 | 0.377431 | 3.216574 | 0.001297 | 0.004857 | Up |
| VN1R87P | 0.922195342 | 2.088322844 | 0.437772 | 4.770342 | 1.84E-06 | 1.39E-05 | Up |
| LINC02132 | 13.68585109 | -1.891409405 | 0.533993 | -3.54201 | 0.000397 | 0.00172 | Down |
| AL049747.1 | 1.378767299 | -1.490340101 | 0.442746 | -3.36613 | 0.000762 | 0.003052 | Down |
| AC011487.2 | 1.377242844 | -1.050576514 | 0.396035 | -2.65274 | 0.007984 | 0.023272 | Down |
| ESPNP | 8.904597831 | 1.376817254 | 0.335369 | 4.105375 | 4.04E-05 | 0.000225 | Up |
| IGFL1P1 | 1.427189496 | 3.63951301 | 0.560283 | 6.495848 | 8.26E-11 | 1.40E-09 | Up |
| AC011473.3 | 1.302534318 | 4.194971346 | 0.819601 | 5.11831 | 3.08E-07 | 2.74E-06 | Up |
| LINC01711 | 5.117972484 | 2.830313793 | 0.424123 | 6.673331 | 2.50E-11 | 4.61E-10 | Up |
| AC024563.1 | 1.167284049 | 1.313924399 | 0.378371 | 3.472585 | 0.000515 | 0.002168 | Up |
| BNIP3P18 | 31.3324133 | -1.339334612 | 0.235656 | -5.68344 | 1.32E-08 | 1.51E-07 | Down |
| TPRG1LP1 | 3.946864919 | 1.5396638 | 0.291097 | 5.289169 | 1.23E-07 | 1.18E-06 | Up |
| ZNF209P | 81.76433408 | -1.188617758 | 0.245004 | -4.85142 | 1.23E-06 | 9.59E-06 | Down |
| AC010336.5 | 1.607912521 | 2.205183664 | 0.435604 | 5.062355 | 4.14E-07 | 3.57E-06 | Up |
| AC078899.4 | 123.9212745 | -2.479480267 | 0.283558 | -8.74418 | 2.25E-18 | 1.07E-16 | Down |
| AC011503.1 | 1.855766965 | 1.507329726 | 0.325715 | 4.627753 | 3.70E-06 | 2.61E-05 | Up |
| AL356740.3 | 1.422466851 | 1.365081478 | 0.373237 | 3.657416 | 0.000255 | 0.001162 | Up |
| SPIB | 359.3294006 | -1.956894237 | 0.579752 | -3.3754 | 0.000737 | 0.002959 | Down |
| LINC01224 | 14.01425876 | 1.013098975 | 0.203321 | 4.982762 | 6.27E-07 | 5.22E-06 | Up |
| AL589765.6 | 8.504200466 | 1.373313071 | 0.229148 | 5.993131 | 2.06E-09 | 2.73E-08 | Up |
| AC110792.2 | 2.28193235 | -2.354245254 | 0.491141 | -4.79342 | 1.64E-06 | 1.25E-05 | Down |
| CYP4F23P | 0.663019877 | 1.468277903 | 0.530518 | 2.767629 | 0.005647 | 0.017309 | Up |
| OR7A8P | 0.455629869 | 2.106360824 | 0.780385 | 2.69913 | 0.006952 | 0.020686 | Up |
| MTDHP2 | 3.892639091 | 1.163879744 | 0.323226 | 3.600828 | 0.000317 | 0.001411 | Up |
| AC092723.1 | 0.728524969 | -1.880123521 | 0.65329 | -2.87793 | 0.004003 | 0.01289 | Down |
| AC006262.2 | 1.756359539 | 2.818821482 | 0.530754 | 5.310973 | 1.09E-07 | 1.05E-06 | Up |
| RNF225 | 7.915276094 | 2.286327281 | 0.312464 | 7.317101 | 2.53E-13 | 6.36E-12 | Up |
| ARHGAP19-SLIT1 | 4.675281156 | 3.661151616 | 0.433022 | 8.454883 | 2.79E-17 | 1.16E-15 | Up |
| AC018695.2 | 1.871104335 | 1.150475415 | 0.378096 | 3.042815 | 0.002344 | 0.008093 | Up |
| MIRLET7A1HG | 25.54510531 | 1.00652197 | 0.165499 | 6.081748 | 1.19E-09 | 1.63E-08 | Up |
| AC090181.2 | 29.4657776 | -1.516699089 | 0.272471 | -5.56647 | 2.60E-08 | 2.82E-07 | Down |
| AC022239.1 | 7.049868224 | -1.501629881 | 0.551832 | -2.72117 | 0.006505 | 0.019528 | Down |
| MEI4 | 317.6938414 | -1.116065338 | 0.231108 | -4.8292 | 1.37E-06 | 1.06E-05 | Down |
| AL020997.2 | 12.27814005 | -1.151897908 | 0.311498 | -3.69793 | 0.000217 | 0.001007 | Down |
| AL021328.1 | 2.199624291 | -1.323984363 | 0.341825 | -3.87328 | 0.000107 | 0.00054 | Down |
| AL513318.2 | 8.123044312 | -2.505132669 | 0.423406 | -5.91662 | 3.29E-09 | 4.19E-08 | Down |
| AC005479.2 | 38.45026162 | 1.778964365 | 0.242898 | 7.323911 | 2.41E-13 | 6.06E-12 | Up |
| AC009108.3 | 20.61115 | -1.02471933 | 0.216045 | -4.74308 | 2.10E-06 | 1.57E-05 | Down |
| Z93241.1 | 36.83514886 | -3.617261661 | 0.364267 | -9.93025 | 3.07E-23 | 2.58E-21 | Down |
| AL356488.2 | 1.34224286 | -2.323776196 | 0.589943 | -3.93899 | 8.18E-05 | 0.000423 | Down |
| MIR222HG | 677.8379244 | 1.157325763 | 0.321189 | 3.603253 | 0.000314 | 0.0014 | Up |
| AF131215.7 | 104.4691598 | 1.440886027 | 0.201407 | 7.154104 | 8.42E-13 | 1.97E-11 | Up |
| AP003117.1 | 12.71063107 | -1.106863863 | 0.175568 | -6.30449 | 2.89E-10 | 4.45E-09 | Down |
| LDHAL6EP | 1.072992982 | -1.897950559 | 0.497489 | -3.81506 | 0.000136 | 0.000667 | Down |
| AL365273.2 | 1.281044642 | 2.541484192 | 0.442167 | 5.747791 | 9.04E-09 | 1.06E-07 | Up |
| VTRNA2-1 | 2.989219595 | -2.909680811 | 0.589383 | -4.93682 | 7.94E-07 | 6.47E-06 | Down |
| AC092127.2 | 18.89473597 | -1.875117973 | 0.242995 | -7.71669 | 1.19E-14 | 3.61E-13 | Down |
| TERC | 3.394469117 | -2.578303515 | 0.456831 | -5.64389 | 1.66E-08 | 1.86E-07 | Down |
| AC018695.3 | 0.737070594 | -1.24624672 | 0.494704 | -2.51918 | 0.011763 | 0.032221 | Down |
| AC010530.1 | 4.632202117 | -1.105085548 | 0.267142 | -4.13669 | 3.52E-05 | 0.000199 | Down |
| AC004233.2 | 1.261911585 | 1.700464037 | 0.51422 | 3.306883 | 0.000943 | 0.003677 | Up |
| MTRNR2L11 | 0.684196784 | 1.213800996 | 0.493147 | 2.461336 | 0.013842 | 0.036903 | Up |
| MTND6P22 | 7.908482389 | 1.081977611 | 0.358105 | 3.021398 | 0.002516 | 0.008621 | Up |
| AC015849.1 | 5.504252384 | 1.661666791 | 0.325727 | 5.101408 | 3.37E-07 | 2.97E-06 | Up |
| BX255925.2 | 1.49582577 | 2.716737482 | 0.527507 | 5.150142 | 2.60E-07 | 2.34E-06 | Up |
| IGHV1OR15-4 | 1.099152521 | 2.81295187 | 1.053114 | 2.671081 | 0.007561 | 0.022186 | Up |
| AL451085.1 | 31.52596966 | -1.206711205 | 0.168903 | -7.14441 | 9.04E-13 | 2.11E-11 | Down |
| AC090950.1 | 6.278305204 | -1.063154694 | 0.210081 | -5.06069 | 4.18E-07 | 3.60E-06 | Down |
| AL136084.3 | 15.5122248 | 1.1387492 | 0.2357 | 4.831349 | 1.36E-06 | 1.05E-05 | Up |
| AC145285.5 | 2.933211681 | -1.386119567 | 0.31608 | -4.38534 | 1.16E-05 | 7.34E-05 | Down |
| OR7E108P | 2.027135539 | -1.714888891 | 0.434109 | -3.95037 | 7.80E-05 | 0.000405 | Down |
| AC093424.1 | 3.856150972 | -1.317643932 | 0.273563 | -4.81661 | 1.46E-06 | 1.12E-05 | Down |
| AC006557.5 | 2.745527971 | -1.139176368 | 0.305854 | -3.72458 | 0.000196 | 0.00092 | Down |
| AC113368.1 | 1.121891749 | -1.246776861 | 0.448291 | -2.78118 | 0.005416 | 0.016718 | Down |
| AC006023.1 | 0.732678832 | -1.400138925 | 0.498071 | -2.81113 | 0.004937 | 0.015439 | Down |
| AP000787.2 | 3.903739907 | 1.106668135 | 0.273811 | 4.041716 | 5.31E-05 | 0.000288 | Up |
| AL049840.6 | 7.420876501 | -1.788736419 | 0.266352 | -6.71568 | 1.87E-11 | 3.52E-10 | Down |
| PPIAP52 | 1.175213336 | 1.382199583 | 0.366842 | 3.767831 | 0.000165 | 0.000791 | Up |
| AC009549.1 | 76.09073846 | 1.826058885 | 0.270948 | 6.739507 | 1.59E-11 | 3.02E-10 | Up |
| AC008555.3 | 5.251051208 | 2.176746854 | 0.216438 | 10.05714 | 8.54E-24 | 7.67E-22 | Up |
| AL023806.1 | 54.33285986 | -1.202136111 | 0.162451 | -7.39998 | 1.36E-13 | 3.52E-12 | Down |
| AL031320.1 | 0.633460254 | -1.37740138 | 0.535889 | -2.57031 | 0.010161 | 0.028475 | Down |
| AC138473.1 | 1.964017452 | -1.313671748 | 0.341836 | -3.84299 | 0.000122 | 0.000603 | Down |
| AL162431.4 | 2.112110193 | -1.414155615 | 0.344651 | -4.10316 | 4.08E-05 | 0.000227 | Down |
| BNIP3P15 | 21.61966288 | 1.460766898 | 0.31411 | 4.650496 | 3.31E-06 | 2.37E-05 | Up |
| RF00003.2 | 3.055798159 | -2.14651 | 0.496736 | -4.32123 | 1.55E-05 | 9.52E-05 | Down |
| AC090953.1 | 1.487946044 | -1.683822909 | 0.494399 | -3.4058 | 0.00066 | 0.002692 | Down |
| GAS2L2 | 28.09999265 | 1.671303419 | 0.286064 | 5.842413 | 5.15E-09 | 6.31E-08 | Up |
| AC135983.5 | 2.597370121 | -1.355802189 | 0.552026 | -2.45605 | 0.014047 | 0.037374 | Down |
| RPS10-NUDT3 | 0.797836973 | 1.056310981 | 0.431082 | 2.450371 | 0.014271 | 0.037863 | Up |
| LINC00221 | 1.454651145 | -1.839146834 | 0.675669 | -2.72197 | 0.006489 | 0.019482 | Down |
| HNRNPDLP4 | 2.643206618 | -1.051475828 | 0.326417 | -3.22127 | 0.001276 | 0.004786 | Down |
| RPAP2P1 | 1.266979567 | -1.328031263 | 0.473828 | -2.80277 | 0.005067 | 0.015801 | Down |
| AC128687.1 | 2.771280651 | -1.540421899 | 0.35038 | -4.39644 | 1.10E-05 | 6.99E-05 | Down |
| HIST2H4A | 6.382762299 | 1.005355395 | 0.252024 | 3.989122 | 6.63E-05 | 0.000351 | Up |
| GRAMD4P8 | 1.259753393 | 1.106032431 | 0.380146 | 2.909497 | 0.00362 | 0.011808 | Up |
| AC060764.1 | 0.438613786 | 1.676822085 | 0.565339 | 2.966049 | 0.003017 | 0.010087 | Up |
| AC019257.2 | 0.805774288 | -1.280123503 | 0.495786 | -2.58201 | 0.009823 | 0.027705 | Down |
| AC139700.1 | 1.439709677 | -1.248734239 | 0.512467 | -2.43671 | 0.014821 | 0.03907 | Down |
| AL512631.1 | 8.271230255 | -2.857757033 | 0.675485 | -4.23067 | 2.33E-05 | 0.000137 | Down |
| SNRPGP17 | 0.628181206 | -1.224474846 | 0.522981 | -2.34134 | 0.019215 | 0.048424 | Down |
| NAMA | 15.38227724 | -1.440583234 | 0.211772 | -6.80251 | 1.03E-11 | 2.01E-10 | Down |
| AC083899.3 | 1.23356894 | -1.722444915 | 0.503255 | -3.42261 | 0.00062 | 0.002552 | Down |
| AC007029.1 | 1.042661456 | -1.086519372 | 0.455813 | -2.3837 | 0.01714 | 0.044049 | Down |
| AC010201.1 | 25.47362754 | 1.041857238 | 0.19251 | 5.41196 | 6.23E-08 | 6.34E-07 | Up |
| AC007326.1 | 1.050737397 | 1.753512121 | 0.510447 | 3.43525 | 0.000592 | 0.002452 | Up |
| DPRXP2 | 8.591616753 | 1.014156617 | 0.288294 | 3.517786 | 0.000435 | 0.001868 | Up |
| AL035417.2 | 1.116278866 | -1.439895424 | 0.531405 | -2.7096 | 0.006736 | 0.020122 | Down |
| AL034374.1 | 115.3661246 | -2.366819883 | 0.264169 | -8.9595 | 3.26E-19 | 1.74E-17 | Down |
| AP003973.2 | 9.220775451 | -1.701080472 | 0.254837 | -6.67517 | 2.47E-11 | 4.56E-10 | Down |
| RPL23AP90 | 1.045687945 | -1.239611766 | 0.447567 | -2.76967 | 0.005611 | 0.017213 | Down |
| AL031281.1 | 1.248629434 | -1.145238833 | 0.432628 | -2.64717 | 0.008117 | 0.023593 | Down |
| DPRXP7 | 0.654225022 | 3.089302798 | 0.650313 | 4.750484 | 2.03E-06 | 1.52E-05 | Up |
| AC005183.1 | 2.185575474 | -1.502827732 | 0.443129 | -3.3914 | 0.000695 | 0.002816 | Down |
| LINC02427 | 23.77433133 | -1.201032805 | 0.236588 | -5.07648 | 3.84E-07 | 3.34E-06 | Down |
| LINC02550 | 28.61812683 | -1.541700505 | 0.246701 | -6.24927 | 4.12E-10 | 6.14E-09 | Down |
| MILR1 | 201.6622507 | 1.182927468 | 0.293788 | 4.026471 | 5.66E-05 | 0.000305 | Up |
| ACTG1P22 | 2.015943341 | 1.89496545 | 0.608408 | 3.11463 | 0.001842 | 0.006574 | Up |
| IGHV3OR16-7 | 3.888753798 | 1.99302335 | 0.851255 | 2.341278 | 0.019218 | 0.048428 | Up |
| AC138894.3 | 1.42718413 | -1.080712866 | 0.421766 | -2.56235 | 0.010397 | 0.029034 | Down |
| AL139041.1 | 2.354901007 | 1.111875699 | 0.324512 | 3.426301 | 0.000612 | 0.002524 | Up |
| AC017002.4 | 0.759024116 | -1.552409971 | 0.585257 | -2.65253 | 0.007989 | 0.023281 | Down |
| RPL17P3 | 6.679882118 | -1.337697556 | 0.242353 | -5.51962 | 3.40E-08 | 3.60E-07 | Down |
| AC017033.1 | 0.975912146 | 1.84507856 | 0.498818 | 3.6989 | 0.000217 | 0.001003 | Up |
| AC010501.1 | 91.15884336 | -1.081793538 | 0.174001 | -6.21718 | 5.06E-10 | 7.41E-09 | Down |
| AC022001.2 | 1.536208956 | -1.262873752 | 0.402378 | -3.13852 | 0.001698 | 0.006126 | Down |
| AL138900.2 | 4.038125912 | 2.04138191 | 0.387625 | 5.266389 | 1.39E-07 | 1.32E-06 | Up |
| RN7SKP299 | 0.927306664 | -1.590465464 | 0.535531 | -2.96989 | 0.002979 | 0.009976 | Down |
| AC104794.4 | 19.2238234 | 1.496337982 | 0.389509 | 3.841602 | 0.000122 | 0.000606 | Up |
| AC012213.4 | 10.63871157 | 1.932834097 | 0.341215 | 5.66457 | 1.47E-08 | 1.67E-07 | Up |
| LINC02343 | 2.766035494 | 2.934064052 | 0.348645 | 8.415621 | 3.91E-17 | 1.60E-15 | Up |
| AL096865.1 | 36.32179139 | 3.374913084 | 0.325504 | 10.36827 | 3.46E-25 | 3.56E-23 | Up |
| AL589740.1 | 0.829564069 | -2.061507722 | 0.843952 | -2.44268 | 0.014579 | 0.038541 | Down |
| AL161716.1 | 0.802165542 | 1.98596091 | 0.517222 | 3.839668 | 0.000123 | 0.00061 | Up |
| AC012467.1 | 8.223919364 | -1.35998035 | 0.226847 | -5.99515 | 2.03E-09 | 2.70E-08 | Down |
| AC100803.3 | 14.69324276 | 2.079936857 | 0.256025 | 8.123972 | 4.51E-16 | 1.63E-14 | Up |
| AL354872.2 | 26.69753932 | -1.112345505 | 0.212262 | -5.24042 | 1.60E-07 | 1.50E-06 | Down |
| AC010904.2 | 2.456552873 | 1.397053119 | 0.27877 | 5.011497 | 5.40E-07 | 4.56E-06 | Up |
| AC008592.5 | 1.997757833 | 1.529202848 | 0.322932 | 4.735368 | 2.19E-06 | 1.62E-05 | Up |
| AC016405.2 | 10.77621413 | 3.479772721 | 0.320668 | 10.85164 | 1.96E-27 | 2.54E-25 | Up |
| AC016575.1 | 2.242725288 | 1.172252048 | 0.356005 | 3.292801 | 0.000992 | 0.003846 | Up |
| AC090186.1 | 7.263560568 | -1.107354919 | 0.217041 | -5.10206 | 3.36E-07 | 2.96E-06 | Down |
| AC004233.3 | 171.166784 | 1.433181424 | 0.232996 | 6.151095 | 7.69E-10 | 1.09E-08 | Up |
| AC244517.1 | 13.17830436 | 1.030657233 | 0.225134 | 4.577982 | 4.69E-06 | 3.24E-05 | Up |
| AL606537.1 | 2.219464671 | -1.452631571 | 0.425573 | -3.41335 | 0.000642 | 0.002631 | Down |
| CASC15 | 69.21672109 | 1.22564759 | 0.196975 | 6.222361 | 4.90E-10 | 7.20E-09 | Up |
| AC011306.1 | 15.6542681 | -1.433698988 | 0.241256 | -5.94264 | 2.80E-09 | 3.63E-08 | Down |
| AC005041.3 | 11.50832427 | 1.5733796 | 0.219016 | 7.183846 | 6.78E-13 | 1.61E-11 | Up |
| AL023583.1 | 33.81610602 | -1.271364441 | 0.190577 | -6.67114 | 2.54E-11 | 4.66E-10 | Down |
| RN7SL726P | 0.558751784 | -2.017996982 | 0.660669 | -3.05447 | 0.002255 | 0.00783 | Down |
| AC011373.1 | 1.305339272 | 2.34069071 | 0.438765 | 5.334727 | 9.57E-08 | 9.37E-07 | Up |
| AC022893.3 | 1.91778992 | -1.070333816 | 0.318848 | -3.35688 | 0.000788 | 0.003146 | Down |
| AC044849.1 | 43.14908975 | -1.463489681 | 0.289364 | -5.05761 | 4.25E-07 | 3.65E-06 | Down |
| AC021242.3 | 52.50628511 | 1.350549095 | 0.188202 | 7.176057 | 7.18E-13 | 1.70E-11 | Up |
| AL353581.1 | 0.627338368 | 2.136124548 | 0.787929 | 2.711064 | 0.006707 | 0.020051 | Up |
| AC116351.2 | 6.751645961 | 1.072515428 | 0.252381 | 4.249595 | 2.14E-05 | 0.000127 | Up |
| RNU4-89P | 1.418886902 | -1.374573184 | 0.44526 | -3.08712 | 0.002021 | 0.007124 | Down |
| AL591167.1 | 2.052069204 | -1.602778014 | 0.378936 | -4.22968 | 2.34E-05 | 0.000137 | Down |
| AC016405.3 | 53.63345078 | 3.791733071 | 0.28787 | 13.17167 | 1.28E-39 | 5.45E-37 | Up |
| AL365181.3 | 15.4529423 | 1.191772431 | 0.322665 | 3.693529 | 0.000221 | 0.001023 | Up |
| AL022394.1 | 0.985111488 | -1.292687579 | 0.520183 | -2.48506 | 0.012953 | 0.034917 | Down |
| BX284668.6 | 7.574003584 | -1.514636234 | 0.237865 | -6.36762 | 1.92E-10 | 3.04E-09 | Down |
| RNA5SP357 | 1.700466902 | -1.497383897 | 0.468335 | -3.19725 | 0.001387 | 0.005149 | Down |
| AL645608.6 | 2.469252523 | 1.973395492 | 0.348126 | 5.668622 | 1.44E-08 | 1.63E-07 | Up |
| AC113194.1 | 2.43136901 | -1.740007917 | 0.378388 | -4.59848 | 4.26E-06 | 2.96E-05 | Down |
| AC006273.1 | 11.27792645 | -1.372494066 | 0.216563 | -6.33762 | 2.33E-10 | 3.65E-09 | Down |
| AC254633.1 | 213.5059001 | 4.507709945 | 0.28602 | 15.76011 | 5.85E-56 | 1.57E-52 | Up |
| AC022001.3 | 2.008180926 | -1.353618327 | 0.338635 | -3.99728 | 6.41E-05 | 0.00034 | Down |
| AL645608.7 | 39.25463883 | 1.636621473 | 0.236076 | 6.932615 | 4.13E-12 | 8.65E-11 | Up |
| AL355578.1 | 1.604122735 | -1.974662016 | 0.53364 | -3.70036 | 0.000215 | 0.000999 | Down |
| SNORA28 | 2.580150934 | -1.269935744 | 0.326207 | -3.89304 | 9.90E-05 | 0.000501 | Down |
| LINC02538 | 7.155181513 | -1.18323676 | 0.247276 | -4.78508 | 1.71E-06 | 1.30E-05 | Down |
| AC006504.7 | 1.26592981 | 1.079888893 | 0.438207 | 2.464334 | 0.013727 | 0.036653 | Up |
| AC092807.2 | 21.21113512 | -1.260444336 | 0.235782 | -5.34579 | 9.00E-08 | 8.86E-07 | Down |
| AP005137.2 | 1.54485995 | -2.037530118 | 0.473988 | -4.2987 | 1.72E-05 | 0.000104 | Down |
| LINC02266 | 0.919866751 | 2.318005287 | 0.46276 | 5.009086 | 5.47E-07 | 4.61E-06 | Up |
| AC009336.1 | 0.999349878 | 1.572445428 | 0.452378 | 3.475952 | 0.000509 | 0.002144 | Up |
| AP000345.2 | 41.42817677 | 1.542159114 | 0.229652 | 6.715187 | 1.88E-11 | 3.53E-10 | Up |
| AC092954.1 | 7.628784756 | 4.158705193 | 0.538838 | 7.717909 | 1.18E-14 | 3.58E-13 | Up |
| AC073610.3 | 4.464147025 | 1.020862242 | 0.193643 | 5.271889 | 1.35E-07 | 1.28E-06 | Up |
| AC245100.7 | 6.046439694 | 1.360069526 | 0.393498 | 3.45636 | 0.000548 | 0.002287 | Up |
| AP000525.1 | 2.130813713 | 2.453599716 | 0.419375 | 5.850609 | 4.90E-09 | 6.04E-08 | Up |
| ATP6V1FNB | 105.0068066 | 1.842823806 | 0.186463 | 9.883067 | 4.93E-23 | 4.02E-21 | Up |
| AL390726.3 | 5.163084351 | -2.504216464 | 0.341614 | -7.33054 | 2.29E-13 | 5.80E-12 | Down |
| AL356608.1 | 3.661437776 | -1.122866402 | 0.295163 | -3.80423 | 0.000142 | 0.000693 | Down |
| AC107294.1 | 6.575212204 | 1.376023905 | 0.23552 | 5.842488 | 5.14E-09 | 6.31E-08 | Up |
| AL022324.3 | 1.705437746 | 1.065474235 | 0.365739 | 2.913207 | 0.003577 | 0.011685 | Up |
| AC107294.2 | 39.15785417 | 1.321463009 | 0.193069 | 6.844508 | 7.67E-12 | 1.53E-10 | Up |
| AC007349.4 | 3.326559903 | -1.048687162 | 0.288099 | -3.64003 | 0.000273 | 0.001235 | Down |
| AL355388.2 | 36.51019653 | 1.100109412 | 0.198268 | 5.548599 | 2.88E-08 | 3.10E-07 | Up |
| AC009229.3 | 1.402019888 | 1.904951143 | 0.512356 | 3.718025 | 0.000201 | 0.00094 | Up |
| DGCR9 | 45.59457761 | 1.00680262 | 0.255994 | 3.932909 | 8.39E-05 | 0.000432 | Up |
| AL390726.5 | 177.4050055 | -1.60118238 | 0.214011 | -7.48177 | 7.33E-14 | 1.97E-12 | Down |
| AL022334.2 | 0.72017344 | 2.170095071 | 0.54446 | 3.985776 | 6.73E-05 | 0.000355 | Up |
| AL596442.2 | 10.70415566 | 1.399232709 | 0.244224 | 5.729295 | 1.01E-08 | 1.18E-07 | Up |
| AC019129.2 | 1.829885482 | -1.275045023 | 0.40166 | -3.17444 | 0.001501 | 0.005516 | Down |
| AC093865.1 | 5.112068356 | 2.1871658 | 0.305235 | 7.165521 | 7.75E-13 | 1.82E-11 | Up |
| PACERR | 10.43827731 | 1.289466296 | 0.235523 | 5.474909 | 4.38E-08 | 4.57E-07 | Up |
| AL355312.4 | 19.09287838 | 4.236279089 | 0.303244 | 13.96987 | 2.38E-44 | 1.75E-41 | Up |
| AL355512.1 | 11.82537835 | 3.054512688 | 0.335651 | 9.100269 | 9.01E-20 | 5.22E-18 | Up |
| DGCR10 | 4.760988111 | 1.056996166 | 0.316186 | 3.342954 | 0.000829 | 0.003285 | Up |
| AC092954.2 | 2.13909974 | 4.051904973 | 0.641709 | 6.314238 | 2.71E-10 | 4.20E-09 | Up |
| AC019193.3 | 6.150225094 | -1.052452302 | 0.250374 | -4.20352 | 2.63E-05 | 0.000153 | Down |
| AC016737.2 | 7.273690436 | -1.463525854 | 0.273254 | -5.35592 | 8.51E-08 | 8.42E-07 | Down |
| AL049839.2 | 3.759221875 | -2.575257771 | 0.42917 | -6.00056 | 1.97E-09 | 2.62E-08 | Down |
| AC093458.1 | 2.399868295 | -1.109108822 | 0.3368 | -3.29308 | 0.000991 | 0.003843 | Down |
| AC016717.2 | 12.11940359 | 3.60406359 | 0.402053 | 8.964149 | 3.13E-19 | 1.68E-17 | Up |
| AL121749.1 | 1.957362683 | 1.779295159 | 0.385002 | 4.621526 | 3.81E-06 | 2.68E-05 | Up |
| RBAKDN | 1.073305054 | 1.105807596 | 0.399575 | 2.767457 | 0.00565 | 0.017315 | Up |
| AC099329.2 | 18.30652376 | -1.498923636 | 0.181202 | -8.27211 | 1.32E-16 | 5.12E-15 | Down |
| AC006566.1 | 14.53916288 | 1.440965089 | 0.169131 | 8.519799 | 1.60E-17 | 6.81E-16 | Up |
| AL137796.1 | 4.681034626 | -1.002672502 | 0.280528 | -3.57423 | 0.000351 | 0.001544 | Down |
| AC005291.2 | 1.09680127 | 1.392846866 | 0.523793 | 2.659156 | 0.007834 | 0.022895 | Up |
| AC107294.3 | 17.31138353 | 1.352083697 | 0.193246 | 6.996692 | 2.62E-12 | 5.66E-11 | Up |
| AL732292.2 | 5.266165904 | 1.330580505 | 0.220186 | 6.042992 | 1.51E-09 | 2.05E-08 | Up |
| AC004877.1 | 55.93060721 | -1.966435024 | 0.346067 | -5.68224 | 1.33E-08 | 1.52E-07 | Down |
| AL645608.8 | 4.235905057 | 2.553095203 | 0.413189 | 6.179001 | 6.45E-10 | 9.24E-09 | Up |
| AC112229.4 | 0.917603376 | 2.363015585 | 0.469578 | 5.032211 | 4.85E-07 | 4.13E-06 | Up |
| OR6R2P | 0.731600945 | -1.518823002 | 0.631528 | -2.405 | 0.016173 | 0.041975 | Down |
| AL354798.1 | 61.41498829 | -1.612088829 | 0.29531 | -5.45897 | 4.79E-08 | 4.97E-07 | Down |
| AC004223.4 | 16.03804932 | -1.126163503 | 0.21252 | -5.2991 | 1.16E-07 | 1.12E-06 | Down |
| RF00003.4 | 3.468375889 | -3.044401783 | 0.525203 | -5.79662 | 6.77E-09 | 8.12E-08 | Down |
| AC245041.1 | 0.770305639 | 1.918294586 | 0.675658 | 2.839152 | 0.004523 | 0.01433 | Up |
| RF00003.5 | 6.810594176 | -3.418272503 | 0.419217 | -8.15395 | 3.52E-16 | 1.29E-14 | Down |
| AC020658.4 | 0.854464658 | 1.890223402 | 0.429152 | 4.404551 | 1.06E-05 | 6.77E-05 | Up |
| AC022898.2 | 5.885775171 | -1.021680894 | 0.242924 | -4.20576 | 2.60E-05 | 0.000151 | Down |
| ENPP7P7 | 15.16095023 | 1.562473897 | 0.215122 | 7.263199 | 3.78E-13 | 9.27E-12 | Up |
| RF00017.23 | 1.077203003 | 1.129677094 | 0.462677 | 2.441612 | 0.014622 | 0.038639 | Up |
| AL138721.1 | 2.001829173 | -1.028180013 | 0.33171 | -3.09964 | 0.001938 | 0.006873 | Down |
| AL450423.1 | 2.391911885 | -3.013890887 | 0.468469 | -6.43349 | 1.25E-10 | 2.04E-09 | Down |
| RF00017.26 | 1.285789858 | -1.513289051 | 0.459128 | -3.29601 | 0.000981 | 0.003807 | Down |
| AL135999.3 | 6.92667272 | 1.610336339 | 0.395114 | 4.07562 | 4.59E-05 | 0.000253 | Up |
| AC024909.1 | 68.87414372 | 1.354303168 | 0.273753 | 4.947173 | 7.53E-07 | 6.17E-06 | Up |
| FAM230J | 2.288109746 | 4.985192216 | 0.933539 | 5.340098 | 9.29E-08 | 9.12E-07 | Up |
| AL449266.1 | 1.957743802 | -1.17209509 | 0.393995 | -2.9749 | 0.002931 | 0.009839 | Down |
| AC106739.1 | 13.45469827 | -1.072285992 | 0.332979 | -3.22028 | 0.001281 | 0.004799 | Down |
| AC026333.4 | 4.877955358 | 1.428197043 | 0.232753 | 6.136103 | 8.46E-10 | 1.19E-08 | Up |
| RF00003.6 | 17.45824034 | -3.131974651 | 0.364721 | -8.58733 | 8.90E-18 | 3.91E-16 | Down |
| AC243585.1 | 2.452131368 | 4.217654722 | 0.613046 | 6.879831 | 5.99E-12 | 1.22E-10 | Up |
| AJ011932.1 | 30.90526648 | -1.220081003 | 0.235228 | -5.18681 | 2.14E-07 | 1.96E-06 | Down |
| GGTLC3 | 1.769590119 | 1.357876419 | 0.408095 | 3.327357 | 0.000877 | 0.00345 | Up |
| AC138649.1 | 39.61397506 | -1.184330948 | 0.231474 | -5.11647 | 3.11E-07 | 2.76E-06 | Down |
| AL137060.1 | 6.574369749 | 1.016519237 | 0.271762 | 3.740481 | 0.000184 | 0.000869 | Up |
| ADRA2B | 85.8862349 | 1.077628139 | 0.260094 | 4.143219 | 3.42E-05 | 0.000194 | Up |
| AC091076.1 | 0.43089976 | 1.379813034 | 0.586291 | 2.353459 | 0.0186 | 0.047104 | Up |
| RF02271.5 | 0.516551715 | -1.345267468 | 0.518265 | -2.59571 | 0.00944 | 0.026805 | Down |
| AC004233.4 | 5.593158579 | 2.223873856 | 0.316261 | 7.031772 | 2.04E-12 | 4.47E-11 | Up |
| RF00003.7 | 1.04301605 | -1.8533402 | 0.676262 | -2.74056 | 0.006133 | 0.018587 | Down |
| AC008957.2 | 1.114489311 | -1.828671296 | 0.67176 | -2.72221 | 0.006485 | 0.019471 | Down |
| AC005790.1 | 7.384659344 | 1.017867892 | 0.265057 | 3.84018 | 0.000123 | 0.000609 | Up |
| RN7SL650P | 2.222203197 | -1.580946886 | 0.373898 | -4.22829 | 2.35E-05 | 0.000138 | Down |
| AC010205.1 | 54.06709443 | 1.551131677 | 0.250746 | 6.186064 | 6.17E-10 | 8.86E-09 | Up |
| AC080038.2 | 46.52928966 | 3.518465343 | 0.289269 | 12.1633 | 4.88E-34 | 1.15E-31 | Up |
| AC006249.1 | 2.661805337 | 3.017948417 | 0.484395 | 6.230346 | 4.65E-10 | 6.87E-09 | Up |
| RF00017.42 | 1.59891961 | -1.199577083 | 0.389646 | -3.07863 | 0.00208 | 0.007302 | Down |
| HIST1H4F | 0.745740962 | -1.622005096 | 0.546012 | -2.97064 | 0.002972 | 0.009955 | Down |
| AL133370.2 | 0.521736638 | 2.047810736 | 0.74569 | 2.746196 | 0.006029 | 0.018317 | Up |
| AL049757.1 | 10.43204233 | -1.823989584 | 0.311914 | -5.84772 | 4.98E-09 | 6.13E-08 | Down |
| AL136964.1 | 3.676864561 | 1.473260546 | 0.385047 | 3.826182 | 0.00013 | 0.000641 | Up |
| AC141273.2 | 1.210539961 | -1.628051781 | 0.464845 | -3.50235 | 0.000461 | 0.001966 | Down |
| AC243829.1 | 9.661626146 | -1.008165575 | 0.422594 | -2.38566 | 0.017049 | 0.043843 | Down |
| AC025166.1 | 0.91627686 | 1.525458037 | 0.457712 | 3.332787 | 0.00086 | 0.003391 | Up |
| AC132807.2 | 0.971188218 | -2.639338039 | 0.830654 | -3.17742 | 0.001486 | 0.005468 | Down |
| AC023043.4 | 6.316684245 | 1.524328366 | 0.197788 | 7.706884 | 1.29E-14 | 3.86E-13 | Up |
| AC011700.1 | 41.0266178 | -1.671886289 | 0.231763 | -7.21379 | 5.44E-13 | 1.31E-11 | Down |
| KRT89P | 46.61015105 | -1.737334051 | 0.26786 | -6.48597 | 8.82E-11 | 1.48E-09 | Down |
| AC079684.1 | 11.51079933 | -1.097717412 | 0.196977 | -5.57281 | 2.51E-08 | 2.72E-07 | Down |
| RN7SL600P | 4.71992314 | -2.058869535 | 0.316728 | -6.50043 | 8.01E-11 | 1.36E-09 | Down |
| RNU11 | 16.230497 | -3.504402342 | 0.446083 | -7.85595 | 3.97E-15 | 1.27E-13 | Down |
| AC020663.3 | 7.351974987 | -1.218823498 | 0.256493 | -4.75188 | 2.02E-06 | 1.51E-05 | Down |
| AC106028.5 | 2.58274362 | -2.624677983 | 0.44109 | -5.95044 | 2.67E-09 | 3.46E-08 | Down |
| AC012150.2 | 2.044383428 | 2.762610278 | 0.615944 | 4.485166 | 7.29E-06 | 4.83E-05 | Up |
| AC025253.1 | 1.193349687 | 3.004323418 | 0.495362 | 6.064902 | 1.32E-09 | 1.80E-08 | Up |
| SPDYE19P | 3.272603222 | -1.170623126 | 0.309923 | -3.77715 | 0.000159 | 0.000764 | Down |
| AL138781.2 | 10.76824178 | -1.075799602 | 0.205598 | -5.23254 | 1.67E-07 | 1.56E-06 | Down |
| CCL18 | 964.6206982 | 1.184726672 | 0.436192 | 2.716068 | 0.006606 | 0.019792 | Up |
| FCGBP | 64978.54935 | -1.243710874 | 0.191878 | -6.48177 | 9.07E-11 | 1.52E-09 | Down |
| HNF1B | 24.02898579 | 3.897227955 | 0.474765 | 8.208752 | 2.23E-16 | 8.41E-15 | Up |
| AL449403.2 | 0.642450068 | -1.484363911 | 0.526171 | -2.82107 | 0.004786 | 0.015022 | Down |
| AC007998.4 | 2.078625026 | -1.329797449 | 0.380912 | -3.49109 | 0.000481 | 0.002041 | Down |
| SNORD116-4 | 25.55429823 | -1.310715319 | 0.225628 | -5.8092 | 6.28E-09 | 7.58E-08 | Down |
| RNVU1-19 | 16.80495521 | -2.73977479 | 0.422739 | -6.481 | 9.11E-11 | 1.53E-09 | Down |
| AC242842.1 | 29.07138849 | 1.028187085 | 0.349403 | 2.9427 | 0.003254 | 0.010775 | Up |
| XKR5 | 7.079080487 | 2.830761633 | 0.304013 | 9.311325 | 1.26E-20 | 8.02E-19 | Up |
| AC243830.1 | 2.521275006 | 1.343936368 | 0.335291 | 4.008273 | 6.12E-05 | 0.000326 | Up |
| AL121827.2 | 47.68402363 | 3.5579631 | 0.360508 | 9.869305 | 5.66E-23 | 4.57E-21 | Up |
| AC025580.3 | 6.668682062 | -1.269921869 | 0.239323 | -5.30632 | 1.12E-07 | 1.08E-06 | Down |
| AL133320.1 | 1.155873544 | -1.623807642 | 0.514855 | -3.15391 | 0.001611 | 0.005861 | Down |
| AC008622.2 | 5.999854145 | 1.641024111 | 0.29216 | 5.61687 | 1.94E-08 | 2.15E-07 | Up |
| AL590227.1 | 0.458755422 | -1.344952274 | 0.565947 | -2.37646 | 0.017479 | 0.044764 | Down |
| ARHGAP23 | 1889.847121 | 1.67710297 | 0.152189 | 11.01986 | 3.07E-28 | 4.32E-26 | Up |
| PICSAR | 3.18008617 | 2.82544953 | 0.46246 | 6.109612 | 9.99E-10 | 1.38E-08 | Up |
| AL021578.1 | 51.60083024 | -1.502854011 | 0.316881 | -4.74264 | 2.11E-06 | 1.57E-05 | Down |
| PRSS2 | 228.9538593 | 6.124142327 | 0.491045 | 12.47164 | 1.07E-35 | 2.82E-33 | Up |
| AC021491.4 | 55.29268067 | -1.13075629 | 0.215706 | -5.24212 | 1.59E-07 | 1.49E-06 | Down |
| AC110285.6 | 7.986439396 | 1.129691567 | 0.362492 | 3.116463 | 0.00183 | 0.00654 | Up |
| CCL3L1 | 173.3732343 | -1.125423154 | 0.363657 | -3.09474 | 0.00197 | 0.006972 | Down |
| AC242988.1 | 0.89698506 | -1.205119546 | 0.465959 | -2.58632 | 0.009701 | 0.027421 | Down |
| AL591441.1 | 1.552700203 | -1.145371023 | 0.480597 | -2.38323 | 0.017162 | 0.044096 | Down |
| AC244153.1 | 16.03575982 | 1.091344085 | 0.188785 | 5.780895 | 7.43E-09 | 8.85E-08 | Up |
| RF00017.83 | 0.815150857 | -1.5622895 | 0.561353 | -2.78308 | 0.005385 | 0.016631 | Down |
| AC245014.3 | 33.29787751 | -1.787926401 | 0.245842 | -7.27266 | 3.52E-13 | 8.67E-12 | Down |
| PIK3R6 | 89.52274148 | 1.075932255 | 0.329123 | 3.26909 | 0.001079 | 0.004133 | Up |
| AC007786.2 | 1.940794316 | -1.37643511 | 0.370928 | -3.71079 | 0.000207 | 0.000963 | Down |
| AC022960.2 | 2.186646018 | -1.033192445 | 0.329615 | -3.13454 | 0.001721 | 0.006201 | Down |
| AC015726.2 | 3.077041354 | -1.052411841 | 0.325586 | -3.23237 | 0.001228 | 0.004622 | Down |
| AL136301.1 | 1.461144561 | -1.173074345 | 0.377631 | -3.1064 | 0.001894 | 0.006736 | Down |
| AL117372.1 | 1.316812791 | 2.195442638 | 0.644916 | 3.40423 | 0.000664 | 0.002704 | Up |
| AL713922.2 | 2.682603536 | -1.739802403 | 0.349389 | -4.97956 | 6.37E-07 | 5.30E-06 | Down |
| IGKV1OR1-1 | 4.490090333 | 1.821307606 | 0.727045 | 2.505082 | 0.012242 | 0.03331 | Up |
| YWHAEP7 | 30.41414548 | 2.542361328 | 0.236736 | 10.73922 | 6.66E-27 | 8.22E-25 | Up |
| AC137834.2 | 0.575236786 | 1.223737571 | 0.507204 | 2.41271 | 0.015834 | 0.041234 | Up |
| AC068234.3 | 32.58469632 | -1.420582239 | 0.210988 | -6.73301 | 1.66E-11 | 3.14E-10 | Down |
| MIR6730 | 4.058139699 | 1.822104825 | 0.324629 | 5.612883 | 1.99E-08 | 2.20E-07 | Up |
| AL354718.3 | 4.90063016 | -1.388352225 | 0.246931 | -5.62244 | 1.88E-08 | 2.09E-07 | Down |
| AC245041.2 | 3.168453041 | 1.717411505 | 0.363816 | 4.720554 | 2.35E-06 | 1.74E-05 | Up |
| AC002401.4 | 33.34675757 | 3.131584555 | 0.338687 | 9.246261 | 2.32E-20 | 1.43E-18 | Up |
| AC026124.2 | 25.34769223 | -1.321726295 | 0.238754 | -5.53594 | 3.10E-08 | 3.31E-07 | Down |
| AC099778.2 | 2.255463781 | -1.005279514 | 0.311988 | -3.22218 | 0.001272 | 0.004771 | Down |
| MIR7848 | 7.055501745 | -1.166053396 | 0.27132 | -4.2977 | 1.73E-05 | 0.000105 | Down |
| AC008760.2 | 24.56401434 | 1.091938215 | 0.352699 | 3.095948 | 0.001962 | 0.006946 | Up |
| AC148477.4 | 17.4403414 | -1.261514484 | 0.281375 | -4.4834 | 7.35E-06 | 4.87E-05 | Down |
| AL445670.1 | 2.435090026 | -1.353045731 | 0.342939 | -3.94544 | 7.97E-05 | 0.000412 | Down |
| AL589743.6 | 1.042341885 | 1.673360166 | 0.511813 | 3.269478 | 0.001077 | 0.00413 | Up |
| AC005696.4 | 12.84553963 | 1.102509531 | 0.232745 | 4.736987 | 2.17E-06 | 1.61E-05 | Up |
| SPANXA2-OT1 | 7.17355951 | 1.062032042 | 0.228987 | 4.637951 | 3.52E-06 | 2.50E-05 | Up |
| MIR6784 | 0.759097783 | 2.638765455 | 0.512252 | 5.1513 | 2.59E-07 | 2.33E-06 | Up |
| C10orf142 | 12.98523271 | 2.626933839 | 0.311112 | 8.443691 | 3.07E-17 | 1.27E-15 | Up |
| SPDYE20P | 1.009670521 | -2.01505577 | 0.475616 | -4.23673 | 2.27E-05 | 0.000134 | Down |
| AP001065.2 | 1.020049409 | -1.562988578 | 0.495277 | -3.15579 | 0.001601 | 0.005831 | Down |
| SRCIN1 | 539.0560841 | 3.097005452 | 0.217624 | 14.23101 | 5.88E-46 | 4.91E-43 | Up |
| AC005837.3 | 8.397678377 | -1.337908748 | 0.334479 | -3.99998 | 6.33E-05 | 0.000337 | Down |
| AL138999.1 | 23.24632684 | -1.096839309 | 0.186904 | -5.86845 | 4.40E-09 | 5.47E-08 | Down |
| RN7SL473P | 3.624852904 | -1.441239728 | 0.355136 | -4.05828 | 4.94E-05 | 0.000271 | Down |
| AL022345.3 | 1.758665001 | -1.424781751 | 0.412809 | -3.45143 | 0.000558 | 0.002324 | Down |
| AC107081.3 | 4.356296969 | -1.917535249 | 0.32963 | -5.81724 | 5.98E-09 | 7.26E-08 | Down |
| AC245123.1 | 1.976535681 | -1.305281277 | 0.468471 | -2.78626 | 0.005332 | 0.016488 | Down |
| GOLGA8IP | 18.46002785 | -1.217573255 | 0.309407 | -3.93518 | 8.31E-05 | 0.000428 | Down |
| AC063961.1 | 2.422585939 | -1.100994792 | 0.409588 | -2.68805 | 0.007187 | 0.021285 | Down |
| MIR7845 | 1.863971485 | -2.460186604 | 0.682983 | -3.60212 | 0.000316 | 0.001405 | Down |
| RNVU1-4 | 12.42185796 | -2.83541595 | 0.330214 | -8.5866 | 8.96E-18 | 3.93E-16 | Down |
| PGBD4P2 | 1.025956268 | -2.429284335 | 0.483208 | -5.02741 | 4.97E-07 | 4.22E-06 | Down |
| FP325317.1 | 1.074795737 | -2.213861779 | 0.522429 | -4.23763 | 2.26E-05 | 0.000133 | Down |
| AC126175.1 | 2.29970584 | -1.373387482 | 0.434142 | -3.16345 | 0.001559 | 0.005702 | Down |
| AC090340.1 | 0.755364799 | -1.431029578 | 0.50137 | -2.85424 | 0.004314 | 0.013756 | Down |
| AC026368.1 | 18.79006195 | 2.502583574 | 0.213269 | 11.73439 | 8.49E-32 | 1.69E-29 | Up |
| AL162574.1 | 2.078418921 | 1.219858533 | 0.344707 | 3.53883 | 0.000402 | 0.001739 | Up |
| NLRP3P1 | 5.909577748 | 1.937324773 | 0.299662 | 6.465024 | 1.01E-10 | 1.69E-09 | Up |
| RF00003.11 | 1.238232517 | -2.212586801 | 0.632291 | -3.49932 | 0.000466 | 0.001985 | Down |
| AC107308.1 | 4.196365616 | 3.842288238 | 0.435835 | 8.815928 | 1.19E-18 | 5.88E-17 | Up |
| RN7SL478P | 0.816876183 | -1.989843423 | 0.831237 | -2.39384 | 0.016673 | 0.043071 | Down |
| AC092745.3 | 0.835431703 | -1.452091365 | 0.54352 | -2.67164 | 0.007548 | 0.022157 | Down |
| AC139100.2 | 45.89517809 | -1.315437395 | 0.191146 | -6.88184 | 5.91E-12 | 1.20E-10 | Down |
| AL627171.1 | 18.95617312 | -1.132450512 | 0.19193 | -5.90034 | 3.63E-09 | 4.58E-08 | Down |
| AC118658.1 | 10.84216648 | -1.523804569 | 0.292271 | -5.21368 | 1.85E-07 | 1.71E-06 | Down |
| RDM1 | 12.5890492 | 1.397668597 | 0.316384 | 4.417632 | 9.98E-06 | 6.41E-05 | Up |
| AL133325.3 | 0.785623009 | -1.312445585 | 0.555803 | -2.36135 | 0.018209 | 0.046304 | Down |
| AC023490.3 | 10.82640033 | 3.745912597 | 0.360706 | 10.38496 | 2.90E-25 | 3.02E-23 | Up |
| RF00003.12 | 3.435860642 | -3.853261742 | 0.623729 | -6.17778 | 6.50E-10 | 9.30E-09 | Down |
| AL118505.1 | 27.73080959 | -1.537184948 | 0.247879 | -6.20134 | 5.60E-10 | 8.12E-09 | Down |
| SSTR3 | 68.67467029 | 2.656318078 | 0.535397 | 4.961395 | 7.00E-07 | 5.77E-06 | Up |
| RF02271.29 | 1.817859655 | -1.032169283 | 0.353967 | -2.916 | 0.003545 | 0.011595 | Down |
| VWA8-AS1 | 9.388276864 | -1.044793734 | 0.183614 | -5.69016 | 1.27E-08 | 1.46E-07 | Down |
| AC015712.7 | 1.677412344 | 1.55731018 | 0.363806 | 4.28061 | 1.86E-05 | 0.000112 | Up |
| AL161645.1 | 2.439274392 | 1.225014658 | 0.331837 | 3.691616 | 0.000223 | 0.00103 | Up |
| MIR6077 | 0.49546679 | -1.7640446 | 0.688644 | -2.56162 | 0.010419 | 0.029084 | Down |
| AC097374.2 | 15.99662879 | -1.48693741 | 0.322018 | -4.61757 | 3.88E-06 | 2.73E-05 | Down |
| HIST1H4A | 1.482583954 | -1.476294945 | 0.479292 | -3.08016 | 0.002069 | 0.00727 | Down |
| AP000346.4 | 1.873651369 | 1.033835981 | 0.390291 | 2.648883 | 0.008076 | 0.023488 | Up |
| AC006329.2 | 1.213565891 | -1.443925994 | 0.434117 | -3.32612 | 0.000881 | 0.003464 | Down |
| HIST1H4B | 5.093386105 | -1.159812012 | 0.359355 | -3.22749 | 0.001249 | 0.004694 | Down |
| AC013553.4 | 11.40719282 | -1.256084955 | 0.254393 | -4.93757 | 7.91E-07 | 6.45E-06 | Down |
| MIR6722 | 0.878441933 | 3.143566895 | 0.66274 | 4.74329 | 2.10E-06 | 1.57E-05 | Up |
| AC099811.5 | 2.195865716 | -1.171918617 | 0.358826 | -3.26598 | 0.001091 | 0.004171 | Down |
| BX539320.1 | 60.22798137 | -1.093122469 | 0.199471 | -5.4801 | 4.25E-08 | 4.44E-07 | Down |
| AC087821.1 | 0.467598245 | 1.313530892 | 0.557935 | 2.354273 | 0.018559 | 0.047017 | Up |
| AC010533.1 | 0.867323018 | 1.287755428 | 0.477955 | 2.694303 | 0.007054 | 0.020941 | Up |
| BANCR | 27.30707722 | -1.252884642 | 0.241173 | -5.19496 | 2.05E-07 | 1.88E-06 | Down |
| AC078883.3 | 1.12946688 | 1.094008905 | 0.3541 | 3.089549 | 0.002005 | 0.007072 | Up |
| CR381670.1 | 6.59700935 | -1.295073191 | 0.319009 | -4.05968 | 4.91E-05 | 0.000269 | Down |
| AC092645.1 | 477.4878675 | -1.346610166 | 0.263971 | -5.10135 | 3.37E-07 | 2.97E-06 | Down |
| AL138847.2 | 1.176999916 | -1.182872809 | 0.410559 | -2.88113 | 0.003963 | 0.012776 | Down |
| AC026310.3 | 1.304495717 | 1.632029573 | 0.479408 | 3.404263 | 0.000663 | 0.002704 | Up |
| AC007598.3 | 1.636356109 | -1.076012602 | 0.44444 | -2.42105 | 0.015476 | 0.040468 | Down |
| FP671120.2 | 14.65535293 | 1.157771316 | 0.271342 | 4.266832 | 1.98E-05 | 0.000119 | Up |
| AC110774.1 | 22.0058981 | -1.028132237 | 0.188375 | -5.45789 | 4.82E-08 | 5.00E-07 | Down |
| AC098476.1 | 12.66752508 | 5.910222159 | 0.700683 | 8.434944 | 3.31E-17 | 1.36E-15 | Up |
| AC015656.1 | 7.608618112 | 1.922531196 | 0.353008 | 5.446146 | 5.15E-08 | 5.31E-07 | Up |
| AC073592.3 | 1.5032575 | -1.038080213 | 0.427154 | -2.43022 | 0.01509 | 0.039679 | Down |
| AC138951.2 | 1.143392488 | -2.782554208 | 0.909237 | -3.06032 | 0.002211 | 0.007701 | Down |
| AL356585.2 | 1.132269225 | 1.877596938 | 0.413623 | 4.539391 | 5.64E-06 | 3.83E-05 | Up |
| AL121950.1 | 2.916274886 | -2.40824203 | 0.445802 | -5.40204 | 6.59E-08 | 6.66E-07 | Down |
| AC046158.3 | 3.529743022 | -1.9178291 | 0.398458 | -4.81313 | 1.49E-06 | 1.14E-05 | Down |
| AL590226.2 | 3.249776896 | 1.653332108 | 0.350206 | 4.721032 | 2.35E-06 | 1.73E-05 | Up |
| AC063926.2 | 0.846448272 | 1.448679816 | 0.586952 | 2.468139 | 0.013582 | 0.036337 | Up |
| AC027601.5 | 117.6398537 | -1.709479741 | 0.258366 | -6.6165 | 3.68E-11 | 6.59E-10 | Down |
| AC007656.2 | 1.140149338 | -1.315376253 | 0.46052 | -2.85628 | 0.004286 | 0.013683 | Down |
| PWAR5 | 508.2914278 | -1.507337101 | 0.27178 | -5.54616 | 2.92E-08 | 3.14E-07 | Down |
| CR381653.2 | 6.128121835 | -1.118818353 | 0.358475 | -3.12105 | 0.001802 | 0.006451 | Down |
| AL390961.3 | 2.948702915 | -1.236898225 | 0.315731 | -3.91757 | 8.94E-05 | 0.000457 | Down |
| AC068254.2 | 1.489227055 | -1.421141073 | 0.493791 | -2.87802 | 0.004002 | 0.012889 | Down |
| AC064801.2 | 3.168461441 | -1.023452738 | 0.310176 | -3.29958 | 0.000968 | 0.003764 | Down |
| AL161773.1 | 2.085858968 | 1.41742475 | 0.348755 | 4.064243 | 4.82E-05 | 0.000264 | Up |
| AP000769.3 | 3.753060092 | -1.441367729 | 0.340017 | -4.2391 | 2.24E-05 | 0.000132 | Down |
| AC007614.1 | 3.192918612 | 4.654991334 | 0.690087 | 6.745509 | 1.52E-11 | 2.91E-10 | Up |
| AL133373.2 | 2.347643516 | -1.493496873 | 0.380776 | -3.92224 | 8.77E-05 | 0.00045 | Down |
| AC073346.2 | 0.655863394 | 1.333923354 | 0.468115 | 2.849564 | 0.004378 | 0.01393 | Up |
| AL136164.3 | 28.54245054 | -1.326185285 | 0.17662 | -7.50869 | 5.97E-14 | 1.63E-12 | Down |
| AC134026.1 | 1.694698814 | -1.845103032 | 0.505873 | -3.64736 | 0.000265 | 0.001204 | Down |
| AC002525.1 | 18.84919105 | -1.364816904 | 0.234446 | -5.82147 | 5.83E-09 | 7.10E-08 | Down |
| AL158212.4 | 3.557195207 | 1.294141794 | 0.329524 | 3.927305 | 8.59E-05 | 0.000441 | Up |
| AL136359.1 | 1.926919293 | -1.259067409 | 0.35869 | -3.51018 | 0.000448 | 0.001916 | Down |
| AC127071.1 | 0.842894152 | 3.197417054 | 0.938932 | 3.405376 | 0.000661 | 0.002696 | Up |
| AGPAT4-IT1 | 12.49037615 | -1.649427584 | 0.233314 | -7.06955 | 1.55E-12 | 3.47E-11 | Down |
| AC007546.2 | 1.776940968 | 3.906520114 | 0.483651 | 8.077147 | 6.63E-16 | 2.33E-14 | Up |
| AC004777.1 | 3.110072419 | 1.385062682 | 0.317747 | 4.359017 | 1.31E-05 | 8.18E-05 | Up |
| CR392039.1 | 0.765547202 | -1.568693319 | 0.652188 | -2.40528 | 0.01616 | 0.041948 | Down |
| AC080188.2 | 64.82055635 | -1.906692699 | 0.374297 | -5.09406 | 3.50E-07 | 3.08E-06 | Down |
| AC021106.3 | 20.04666259 | -1.021652978 | 0.175314 | -5.82757 | 5.62E-09 | 6.86E-08 | Down |
| AC116456.2 | 0.673203415 | -2.273102398 | 0.566402 | -4.01323 | 5.99E-05 | 0.00032 | Down |
| AC008739.5 | 1.837465147 | -1.682520008 | 0.422931 | -3.97824 | 6.94E-05 | 0.000365 | Down |
| AC004925.1 | 2.090643504 | 2.343384035 | 0.529249 | 4.427751 | 9.52E-06 | 6.15E-05 | Up |
| AC135584.1 | 91.57494485 | -2.220008497 | 0.308088 | -7.20575 | 5.77E-13 | 1.39E-11 | Down |
| AL159972.1 | 1.046296549 | 1.257831683 | 0.486813 | 2.583807 | 0.009772 | 0.027586 | Up |
| AC073911.2 | 1.58889558 | -1.567097559 | 0.423106 | -3.70379 | 0.000212 | 0.000987 | Down |
| AC127071.2 | 1.095607208 | 3.679971981 | 0.801621 | 4.590664 | 4.42E-06 | 3.07E-05 | Up |
| AP005205.3 | 4.84592007 | -1.695292117 | 0.302686 | -5.60083 | 2.13E-08 | 2.34E-07 | Down |
| AC002094.5 | 3.569402684 | -1.000638262 | 0.304567 | -3.28545 | 0.001018 | 0.003933 | Down |
| AC005593.1 | 7.188294701 | -1.19123668 | 0.222222 | -5.36057 | 8.30E-08 | 8.22E-07 | Down |
| AC109326.1 | 178.9654483 | -2.281238565 | 0.267025 | -8.54317 | 1.31E-17 | 5.62E-16 | Down |
| AL358613.2 | 0.67665091 | 1.821744075 | 0.562116 | 3.240869 | 0.001192 | 0.004503 | Up |
| AL096817.1 | 0.917255369 | -1.13807633 | 0.458224 | -2.48367 | 0.013004 | 0.035017 | Down |
| AC005796.1 | 8.180084294 | -2.283496908 | 0.42038 | -5.43198 | 5.57E-08 | 5.72E-07 | Down |
| AC025280.2 | 5.198511334 | -2.078116882 | 0.4028 | -5.15918 | 2.48E-07 | 2.24E-06 | Down |
| AC131280.1 | 0.730570102 | -1.245141714 | 0.521771 | -2.38638 | 0.017015 | 0.043783 | Down |
| AP003108.4 | 2.211622543 | -1.06865395 | 0.299336 | -3.57008 | 0.000357 | 0.001566 | Down |
| AC018445.3 | 1.467830404 | -1.443766545 | 0.450555 | -3.20442 | 0.001353 | 0.00504 | Down |
| AC020978.9 | 96.77199489 | -1.21096051 | 0.26188 | -4.6241 | 3.76E-06 | 2.65E-05 | Down |
| AC110491.2 | 0.744431535 | -1.380990792 | 0.541118 | -2.55211 | 0.010707 | 0.029766 | Down |
| AL603839.4 | 4.69095468 | 1.436864834 | 0.24236 | 5.928631 | 3.05E-09 | 3.92E-08 | Up |
| AC080100.1 | 46.07452223 | -1.67261826 | 0.212136 | -7.88465 | 3.15E-15 | 1.03E-13 | Down |
| AC113410.3 | 4.583660623 | -1.026376652 | 0.352572 | -2.91111 | 0.003601 | 0.011752 | Down |
| AC011270.2 | 86.65912326 | -1.706099675 | 0.288749 | -5.90859 | 3.45E-09 | 4.38E-08 | Down |
| AC120349.3 | 1.445321485 | -1.034970106 | 0.402247 | -2.57297 | 0.010083 | 0.028296 | Down |
| AP004782.1 | 24.08436398 | -1.304894575 | 0.261437 | -4.99124 | 6.00E-07 | 5.01E-06 | Down |
| AC080038.4 | 11.9993275 | 2.826780982 | 0.278433 | 10.15245 | 3.23E-24 | 3.01E-22 | Up |
| Z95114.2 | 1.285961232 | -1.349813615 | 0.55772 | -2.42024 | 0.01551 | 0.040539 | Down |
| AL391005.1 | 5.024514809 | 2.147742649 | 0.452853 | 4.742695 | 2.11E-06 | 1.57E-05 | Up |
| AC112204.3 | 2.453266672 | -1.195423204 | 0.365317 | -3.27229 | 0.001067 | 0.004094 | Down |
| AC007342.8 | 2.320118968 | -1.315452905 | 0.449474 | -2.92665 | 0.003426 | 0.01127 | Down |
| AC092850.1 | 6.118880039 | -2.195505826 | 0.37186 | -5.90412 | 3.55E-09 | 4.49E-08 | Down |
| AP000997.3 | 8.215249717 | 3.794320206 | 0.263737 | 14.38675 | 6.27E-47 | 5.88E-44 | Up |
| AL133384.2 | 1.32296702 | -1.190063666 | 0.468742 | -2.53885 | 0.011122 | 0.030698 | Down |
| AC120057.4 | 5.142882395 | 1.559809732 | 0.316103 | 4.934492 | 8.04E-07 | 6.54E-06 | Up |
| AC073911.3 | 6.364786002 | -1.359326409 | 0.220308 | -6.17013 | 6.82E-10 | 9.72E-09 | Down |
| AC003957.1 | 3.602900729 | -1.094743631 | 0.42868 | -2.55375 | 0.010657 | 0.029657 | Down |
| AC136469.2 | 1.822298821 | -1.06011104 | 0.356481 | -2.97382 | 0.002941 | 0.009869 | Down |
| AL512383.1 | 2.831335532 | -1.351504807 | 0.359649 | -3.75785 | 0.000171 | 0.000819 | Down |
| AC068880.4 | 19.03530198 | -1.087333354 | 0.238351 | -4.5619 | 5.07E-06 | 3.47E-05 | Down |
| LINC01126 | 42.45448832 | -1.189016261 | 0.203236 | -5.85043 | 4.90E-09 | 6.04E-08 | Down |
| AP003108.5 | 11.06653937 | -1.575440529 | 0.357874 | -4.40222 | 1.07E-05 | 6.83E-05 | Down |
| AP005060.1 | 1.961911258 | -1.606725579 | 0.398194 | -4.03503 | 5.46E-05 | 0.000295 | Down |
| AC046158.4 | 1.185171387 | -1.156287515 | 0.484652 | -2.38581 | 0.017042 | 0.04383 | Down |
| AC092117.2 | 2.2814097 | -1.043350855 | 0.314835 | -3.31396 | 0.00092 | 0.003599 | Down |
| AC063926.3 | 183.1731598 | 3.796547628 | 0.456541 | 8.315894 | 9.11E-17 | 3.64E-15 | Up |
| AC026748.1 | 168.1572155 | -1.057589766 | 0.166409 | -6.35537 | 2.08E-10 | 3.27E-09 | Down |
| AC022784.6 | 17.44835902 | 1.432916594 | 0.224955 | 6.3698 | 1.89E-10 | 2.99E-09 | Up |
| AC018445.4 | 0.939454872 | -1.976613945 | 0.497506 | -3.97304 | 7.10E-05 | 0.000372 | Down |
| AC097637.2 | 10.1818129 | -1.010144356 | 0.343704 | -2.939 | 0.003293 | 0.010893 | Down |
| AC126544.1 | 37.31916583 | 1.220376075 | 0.171015 | 7.136068 | 9.60E-13 | 2.23E-11 | Up |
| AP002800.1 | 42.79590057 | 1.591998902 | 0.167848 | 9.484763 | 2.43E-21 | 1.65E-19 | Up |
| AC079597.1 | 0.451693061 | 2.179323608 | 0.838145 | 2.600175 | 0.009318 | 0.026516 | Up |
| AC011199.1 | 2.872714623 | -1.644106429 | 0.329338 | -4.99215 | 5.97E-07 | 4.99E-06 | Down |
| TMEM75 | 1.03138231 | -1.193435596 | 0.46093 | -2.58919 | 0.00962 | 0.027226 | Down |
| AL022069.2 | 17.72453115 | -1.123239231 | 0.190783 | -5.88753 | 3.92E-09 | 4.92E-08 | Down |
| AC011504.1 | 24.76687609 | -1.07739892 | 0.312055 | -3.45259 | 0.000555 | 0.002316 | Down |
| AL031719.1 | 5.614355531 | -1.080570338 | 0.220814 | -4.89357 | 9.90E-07 | 7.91E-06 | Down |
| AC011481.4 | 8.685038434 | 1.565450759 | 0.328504 | 4.765395 | 1.88E-06 | 1.42E-05 | Up |
| PLAC4 | 5.681737133 | -1.201543555 | 0.325978 | -3.68596 | 0.000228 | 0.00105 | Down |
| AL021920.2 | 2.404501334 | 2.376285688 | 0.447595 | 5.309004 | 1.10E-07 | 1.06E-06 | Up |
| AC231657.2 | 3.481491592 | -1.396655695 | 0.373766 | -3.73671 | 0.000186 | 0.00088 | Down |
| AC090983.1 | 8.475258623 | -1.41823875 | 0.31312 | -4.52938 | 5.92E-06 | 4.00E-05 | Down |
| AC016168.3 | 2.374186983 | -1.896684511 | 0.734629 | -2.58183 | 0.009828 | 0.027716 | Down |
| AC027290.2 | 445.2529789 | -1.134730148 | 0.203481 | -5.5766 | 2.45E-08 | 2.67E-07 | Down |
| AL021408.2 | 1.423431826 | -1.509313854 | 0.57452 | -2.62709 | 0.008612 | 0.024807 | Down |
| PCDH20 | 1.956560018 | 2.691234284 | 0.498254 | 5.401332 | 6.61E-08 | 6.68E-07 | Up |
| AC010329.5 | 6.672626834 | -1.208497182 | 0.326027 | -3.70674 | 0.00021 | 0.000977 | Down |
| AC004408.2 | 1.161301813 | -1.760668252 | 0.723127 | -2.4348 | 0.0149 | 0.039236 | Down |
| AC131888.1 | 3.277166773 | -1.44241371 | 0.327863 | -4.39944 | 1.09E-05 | 6.91E-05 | Down |
| AC067852.4 | 1.249819381 | -1.025957575 | 0.412157 | -2.48924 | 0.012802 | 0.034554 | Down |
| AC027279.4 | 0.713638169 | -1.294858356 | 0.476915 | -2.71507 | 0.006626 | 0.01984 | Down |
| AC106795.5 | 6.989450064 | -1.953075822 | 0.276958 | -7.05188 | 1.77E-12 | 3.90E-11 | Down |
| AC126564.1 | 8.809971166 | -1.761445974 | 0.323003 | -5.45334 | 4.94E-08 | 5.11E-07 | Down |
| AC093908.1 | 58.05051061 | 1.356169376 | 0.16885 | 8.031804 | 9.61E-16 | 3.33E-14 | Up |
| AL157414.3 | 12.76227775 | -1.544871528 | 0.347549 | -4.44505 | 8.79E-06 | 5.73E-05 | Down |
| MIR4697HG | 1084.061829 | -1.245775613 | 0.162783 | -7.65298 | 1.96E-14 | 5.73E-13 | Down |
| AC009145.4 | 3.02160677 | -1.528054501 | 0.316816 | -4.82316 | 1.41E-06 | 1.09E-05 | Down |
| AC009229.4 | 1.506802595 | 1.978382514 | 0.326599 | 6.057534 | 1.38E-09 | 1.88E-08 | Up |
| AP005264.7 | 10.04491873 | -1.040310459 | 0.283193 | -3.67351 | 0.000239 | 0.001098 | Down |
| AC023300.3 | 2.670574566 | 1.373254648 | 0.298861 | 4.594964 | 4.33E-06 | 3.01E-05 | Up |
| AC131212.4 | 1.155327296 | 1.104666668 | 0.41382 | 2.66944 | 0.007598 | 0.022277 | Up |
| AL035425.4 | 16.88764718 | -2.452330542 | 0.377868 | -6.48991 | 8.59E-11 | 1.45E-09 | Down |
| AC053503.6 | 1.318464331 | -1.971046509 | 0.652353 | -3.02144 | 0.002516 | 0.00862 | Down |
| AC091941.1 | 6.978801819 | -1.004804345 | 0.320186 | -3.13819 | 0.0017 | 0.006132 | Down |
| LINC01726 | 2.130672835 | -1.355785917 | 0.437118 | -3.10165 | 0.001924 | 0.006833 | Down |
| AC021766.1 | 0.7461619 | -1.793211196 | 0.588799 | -3.04554 | 0.002323 | 0.008032 | Down |
| AP003072.5 | 1.972417633 | -1.714660845 | 0.393936 | -4.35264 | 1.35E-05 | 8.39E-05 | Down |
| AC026362.2 | 3.605512512 | -1.413236262 | 0.348875 | -4.05084 | 5.10E-05 | 0.000278 | Down |
| AC096920.1 | 5.650652438 | -1.27540308 | 0.245355 | -5.1982 | 2.01E-07 | 1.85E-06 | Down |
| AC032044.2 | 2.673468252 | -1.267643458 | 0.329514 | -3.84701 | 0.00012 | 0.000594 | Down |
| AC005252.3 | 3.480675097 | -1.453536386 | 0.317802 | -4.57371 | 4.79E-06 | 3.30E-05 | Down |
| AC084876.2 | 17.61456888 | -1.060953946 | 0.205072 | -5.17357 | 2.30E-07 | 2.09E-06 | Down |
| AC006058.4 | 644.3929212 | -1.767018034 | 0.258599 | -6.83303 | 8.31E-12 | 1.66E-10 | Down |
| CR392039.5 | 0.499448566 | -1.613052532 | 0.609083 | -2.64833 | 0.008089 | 0.02352 | Down |
| AC004836.1 | 20.67532902 | -2.906428274 | 0.330417 | -8.79623 | 1.41E-18 | 6.92E-17 | Down |
| PCAT14 | 93.43594728 | -1.895785624 | 0.341338 | -5.55398 | 2.79E-08 | 3.02E-07 | Down |
| AC134981.1 | 1.864893951 | 2.455771793 | 0.963989 | 2.547509 | 0.010849 | 0.030088 | Up |
| SH3PXD2A-AS1 | 6.295769998 | 1.655208732 | 0.20981 | 7.889072 | 3.04E-15 | 9.92E-14 | Up |
| LINC00581 | 1.322856783 | 1.358591524 | 0.356345 | 3.812571 | 0.000138 | 0.000673 | Up |
| AC005154.5 | 2.921309068 | 1.552289515 | 0.383959 | 4.042847 | 5.28E-05 | 0.000287 | Up |
| TMEM272 | 6.906825177 | -1.502272888 | 0.436032 | -3.44533 | 0.00057 | 0.002369 | Down |
| AC007878.1 | 32.65606293 | -1.107284322 | 0.233477 | -4.74258 | 2.11E-06 | 1.57E-05 | Down |
| LINC01176 | 71.81859289 | 1.093280269 | 0.20685 | 5.285378 | 1.25E-07 | 1.20E-06 | Up |
| BLACAT1 | 335.8821974 | 1.278292733 | 0.14775 | 8.651717 | 5.07E-18 | 2.31E-16 | Up |
| DNAJB5-DT | 3.488585521 | -1.693412032 | 0.375806 | -4.50608 | 6.60E-06 | 4.42E-05 | Down |
| AP000851.2 | 9.125563262 | 2.8792091 | 0.743915 | 3.870347 | 0.000109 | 0.000545 | Up |
| LINC00538 | 1.755004705 | -2.210778753 | 0.429876 | -5.14282 | 2.71E-07 | 2.43E-06 | Down |
| PACRG-AS1 | 2.782406722 | -1.105042326 | 0.327443 | -3.37477 | 0.000739 | 0.002965 | Down |
| LINC01230 | 0.572409834 | 1.819066617 | 0.70126 | 2.593999 | 0.009487 | 0.026921 | Up |
| LINC00602 | 12.42761797 | -1.674808396 | 0.418267 | -4.00417 | 6.22E-05 | 0.000331 | Down |
| LINC00891 | 22.12328476 | 2.423953914 | 0.246172 | 9.846591 | 7.09E-23 | 5.64E-21 | Up |
| AC007389.5 | 8.043305743 | 1.319193202 | 0.319221 | 4.132533 | 3.59E-05 | 0.000202 | Up |

Table S7: Maker genes in the two PTMETA subgroups.

| feature_symbol | sc3_gene_filter | sc3_2_markers_clusts | sc3_2_markers_padj | sc3_2_markers_auroc |
| --- | --- | --- | --- | --- |
| ARHGEF12 | TRUE | C1-PTMC-proliferation | 1.90E-07 | 0.935223 |
| SRP68 | TRUE | C1-PTMC-proliferation | 4.54E-07 | 0.92915 |
| SPRED2 | TRUE | C1-PTMC-proliferation | 1.04E-06 | 0.923077 |
| KIAA1462 | TRUE | C1-PTMC-proliferation | 1.19E-06 | 0.922065 |
| CCND2 | TRUE | C1-PTMC-proliferation | 2.97E-06 | 0.91498 |
| BHLHE41 | TRUE | C1-PTMC-proliferation | 4.91E-06 | 0.910931 |
| FAM83H | TRUE | C1-PTMC-proliferation | 6.29E-06 | 0.908907 |
| PKP4 | TRUE | C1-PTMC-proliferation | 8.01E-06 | 0.906883 |
| RP11-255H23.2 | TRUE | C1-PTMC-proliferation | 8.01E-06 | 0.906883 |
| RBMS2 | TRUE | C1-PTMC-proliferation | 1.15E-05 | 0.903846 |
| SLC17A5 | TRUE | C1-PTMC-proliferation | 1.29E-05 | 0.902834 |
| SHE | TRUE | C1-PTMC-proliferation | 1.29E-05 | 0.902834 |
| GXYLT2 | TRUE | C1-PTMC-proliferation | 1.83E-05 | 0.899798 |
| CCNI | TRUE | C1-PTMC-proliferation | 2.06E-05 | 0.898785 |
| EPS8 | TRUE | C1-PTMC-proliferation | 2.30E-05 | 0.897773 |
| KIAA1522 | TRUE | C1-PTMC-proliferation | 2.89E-05 | 0.895749 |
| KANK2 | TRUE | C1-PTMC-proliferation | 3.23E-05 | 0.894737 |
| PRRG1 | TRUE | C1-PTMC-proliferation | 4.04E-05 | 0.892713 |
| NHSL2 | TRUE | C1-PTMC-proliferation | 4.04E-05 | 0.892713 |
| PARD6B | TRUE | C1-PTMC-proliferation | 4.51E-05 | 0.8917 |
| SAV1 | TRUE | C1-PTMC-proliferation | 4.51E-05 | 0.8917 |
| UACA | TRUE | C1-PTMC-proliferation | 4.51E-05 | 0.8917 |
| METTL7B | TRUE | C1-PTMC-proliferation | 4.51E-05 | 0.8917 |
| PGRMC2 | TRUE | C1-PTMC-proliferation | 4.51E-05 | 0.8917 |
| DDAH1 | TRUE | C1-PTMC-proliferation | 5.03E-05 | 0.890688 |
| INSR | TRUE | C1-PTMC-proliferation | 5.03E-05 | 0.890688 |
| MAP2 | TRUE | C1-PTMC-proliferation | 5.60E-05 | 0.889676 |
| SH3BGRL2 | TRUE | C1-PTMC-proliferation | 5.60E-05 | 0.889676 |
| YAP1 | TRUE | C1-PTMC-proliferation | 6.24E-05 | 0.888664 |
| SLC5A3 | TRUE | C1-PTMC-proliferation | 7.74E-05 | 0.88664 |
| SIAE | TRUE | C1-PTMC-proliferation | 7.74E-05 | 0.88664 |
| PSD3 | TRUE | C1-PTMC-proliferation | 7.74E-05 | 0.88664 |
| NUDT3 | TRUE | C1-PTMC-proliferation | 8.61E-05 | 0.885628 |
| LMO7 | TRUE | C1-PTMC-proliferation | 8.61E-05 | 0.885628 |
| SLC41A1 | TRUE | C1-PTMC-proliferation | 8.61E-05 | 0.885628 |
| LPAR5 | TRUE | C1-PTMC-proliferation | 8.61E-05 | 0.885628 |
| WASL | TRUE | C1-PTMC-proliferation | 8.61E-05 | 0.885628 |
| RXRG | TRUE | C1-PTMC-proliferation | 9.56E-05 | 0.884615 |
| SLC12A2 | TRUE | C1-PTMC-proliferation | 9.56E-05 | 0.884615 |
| MRPS6 | TRUE | C1-PTMC-proliferation | 9.56E-05 | 0.884615 |
| PC | TRUE | C1-PTMC-proliferation | 9.56E-05 | 0.884615 |
| PDE5A | TRUE | C1-PTMC-proliferation | 0.000106166 | 0.883603 |
| RPRD2 | TRUE | C1-PTMC-proliferation | 0.000145171 | 0.880567 |
| LTBP3 | TRUE | C1-PTMC-proliferation | 0.00016092 | 0.879555 |
| ERBB2 | TRUE | C1-PTMC-proliferation | 0.000178127 | 0.878543 |
| P4HTM | TRUE | C1-PTMC-proliferation | 0.000178127 | 0.878543 |
| ABI2 | TRUE | C1-PTMC-proliferation | 0.000178127 | 0.878543 |
| HEY2 | TRUE | C1-PTMC-proliferation | 0.000178127 | 0.878543 |
| ETNK2 | TRUE | C1-PTMC-proliferation | 0.000196937 | 0.87753 |
| DOCK9 | TRUE | C1-PTMC-proliferation | 0.000217928 | 0.876518 |
| XPO7 | TRUE | C1-PTMC-proliferation | 0.000217928 | 0.876518 |
| STXBP6 | TRUE | C1-PTMC-proliferation | 0.000240972 | 0.875506 |
| FOXP4 | TRUE | C1-PTMC-proliferation | 0.000266361 | 0.874494 |
| TJP1 | TRUE | C1-PTMC-proliferation | 0.000266361 | 0.874494 |
| TP53BP2 | TRUE | C1-PTMC-proliferation | 0.000266361 | 0.874494 |
| SLC39A10 | TRUE | C1-PTMC-proliferation | 0.00029414 | 0.873482 |
| CMYA5 | TRUE | C1-PTMC-proliferation | 0.00029414 | 0.873482 |
| PLAG1 | TRUE | C1-PTMC-proliferation | 0.00029414 | 0.873482 |
| APLP2 | TRUE | C1-PTMC-proliferation | 0.000324504 | 0.87247 |
| EMP2 | TRUE | C1-PTMC-proliferation | 0.000324504 | 0.87247 |
| PLOD2 | TRUE | C1-PTMC-proliferation | 0.000394817 | 0.870445 |
| CMTM4 | TRUE | C1-PTMC-proliferation | 0.000394817 | 0.870445 |
| STIM1 | TRUE | C1-PTMC-proliferation | 0.000394817 | 0.870445 |
| PREPL | TRUE | C1-PTMC-proliferation | 0.000394817 | 0.870445 |
| AEBP2 | TRUE | C1-PTMC-proliferation | 0.000434876 | 0.869433 |
| FRMD3 | TRUE | C1-PTMC-proliferation | 0.00047915 | 0.868421 |
| SEMA3F | TRUE | C1-PTMC-proliferation | 0.000527435 | 0.867409 |
| MLLT4 | TRUE | C1-PTMC-proliferation | 0.000527435 | 0.867409 |
| SIPA1L2 | TRUE | C1-PTMC-proliferation | 0.000527435 | 0.867409 |
| TMC4 | TRUE | C1-PTMC-proliferation | 0.000580288 | 0.866397 |
| QSER1 | TRUE | C1-PTMC-proliferation | 0.000580288 | 0.866397 |
| EPB41L1 | TRUE | C1-PTMC-proliferation | 0.000580288 | 0.866397 |
| KAT6B | TRUE | C1-PTMC-proliferation | 0.000638111 | 0.865385 |
| GNL1 | TRUE | C1-PTMC-proliferation | 0.000638111 | 0.865385 |
| RNF128 | TRUE | C1-PTMC-proliferation | 0.000770936 | 0.86336 |
| GID8 | TRUE | C1-PTMC-proliferation | 0.000770936 | 0.86336 |
| LINC00657 | TRUE | C1-PTMC-proliferation | 0.000846838 | 0.862348 |
| SYNPO | TRUE | C1-PTMC-proliferation | 0.000846838 | 0.862348 |
| C2orf40 | TRUE | C1-PTMC-proliferation | 0.000929362 | 0.861336 |
| PNMA1 | TRUE | C1-PTMC-proliferation | 0.000929362 | 0.861336 |
| NEDD4L | TRUE | C1-PTMC-proliferation | 0.000929362 | 0.861336 |
| MAML3 | TRUE | C1-PTMC-proliferation | 0.001019214 | 0.860324 |
| TRAK2 | TRUE | C1-PTMC-proliferation | 0.001019214 | 0.860324 |
| MEAF6 | TRUE | C1-PTMC-proliferation | 0.001019214 | 0.860324 |
| TTC3 | TRUE | C1-PTMC-proliferation | 0.001117683 | 0.859312 |
| SDC4 | TRUE | C1-PTMC-proliferation | 0.001225594 | 0.8583 |
| FARP1 | TRUE | C1-PTMC-proliferation | 0.001225594 | 0.8583 |
| PTPN14 | TRUE | C1-PTMC-proliferation | 0.001225594 | 0.8583 |
| PDK2 | TRUE | C1-PTMC-proliferation | 0.001225594 | 0.8583 |
| TESC | TRUE | C1-PTMC-proliferation | 0.001225594 | 0.8583 |
| TFE3 | TRUE | C1-PTMC-proliferation | 0.001225594 | 0.8583 |
| NAB1 | TRUE | C1-PTMC-proliferation | 0.001225594 | 0.8583 |
| LONRF2 | TRUE | C1-PTMC-proliferation | 0.001341296 | 0.857287 |
| MFSD6 | TRUE | C1-PTMC-proliferation | 0.001341296 | 0.857287 |
| DTX4 | TRUE | C1-PTMC-proliferation | 0.001341296 | 0.857287 |
| CBX5 | TRUE | C1-PTMC-proliferation | 0.001468778 | 0.856275 |
| FMR1 | TRUE | C1-PTMC-proliferation | 0.001468778 | 0.856275 |
| NIPAL3 | TRUE | C1-PTMC-proliferation | 0.001468778 | 0.856275 |
| NAT8L | TRUE | C1-PTMC-proliferation | 0.001468778 | 0.856275 |
| PPME1 | TRUE | C1-PTMC-proliferation | 0.001468778 | 0.856275 |
| SHROOM4 | TRUE | C1-PTMC-proliferation | 0.00175728 | 0.854251 |
| CST6 | TRUE | C1-PTMC-proliferation | 0.00175728 | 0.854251 |
| ATP13A4 | TRUE | C1-PTMC-proliferation | 0.001920795 | 0.853239 |
| SCD5 | TRUE | C1-PTMC-proliferation | 0.001920795 | 0.853239 |
| RPS6KA2 | TRUE | C1-PTMC-proliferation | 0.001920795 | 0.853239 |
| MIB1 | TRUE | C1-PTMC-proliferation | 0.001920795 | 0.853239 |
| PTPRF | TRUE | C1-PTMC-proliferation | 0.002098563 | 0.852227 |
| RAB27A | TRUE | C1-PTMC-proliferation | 0.002098563 | 0.852227 |
| AHCYL1 | TRUE | C1-PTMC-proliferation | 0.002098563 | 0.852227 |
| ARMCX3 | TRUE | C1-PTMC-proliferation | 0.002098563 | 0.852227 |
| ABCD3 | TRUE | C1-PTMC-proliferation | 0.002098563 | 0.852227 |
| ETV5 | TRUE | C1-PTMC-proliferation | 0.002291738 | 0.851215 |
| PPAP2B | TRUE | C1-PTMC-proliferation | 0.002291738 | 0.851215 |
| LONP2 | TRUE | C1-PTMC-proliferation | 0.002291738 | 0.851215 |
| TNFRSF12A | TRUE | C1-PTMC-proliferation | 0.002501564 | 0.850202 |
| CCDC148 | TRUE | C1-PTMC-proliferation | 0.002501564 | 0.850202 |
| ATP9A | TRUE | C1-PTMC-proliferation | 0.002501564 | 0.850202 |
| LCN12 | TRUE | C1-PTMC-proliferation | 0.002501564 | 0.850202 |
| C16orf45 | TRUE | C1-PTMC-proliferation | 0.002501564 | 0.850202 |
| NPC2 | TRUE | C1-PTMC-proliferation | 0.002501564 | 0.850202 |
| ZNF770 | TRUE | C1-PTMC-proliferation | 0.00272763 | 0.84919 |
| CELSR2 | TRUE | C1-PTMC-proliferation | 0.00272763 | 0.84919 |
| SGMS2 | TRUE | C1-PTMC-proliferation | 0.00272763 | 0.84919 |
| ST3GAL5 | TRUE | C1-PTMC-proliferation | 0.00272763 | 0.84919 |
| TOR1AIP2 | TRUE | C1-PTMC-proliferation | 0.00272763 | 0.84919 |
| HEG1 | TRUE | C1-PTMC-proliferation | 0.00272763 | 0.84919 |
| ZFHX3 | TRUE | C1-PTMC-proliferation | 0.00272763 | 0.84919 |
| SATB1 | TRUE | C1-PTMC-proliferation | 0.002972795 | 0.848178 |
| DDR1 | TRUE | C1-PTMC-proliferation | 0.002972795 | 0.848178 |
| RASA1 | TRUE | C1-PTMC-proliferation | 0.002972795 | 0.848178 |
| MPP5 | TRUE | C1-PTMC-proliferation | 0.002972795 | 0.848178 |
| SHROOM3 | TRUE | C1-PTMC-proliferation | 0.002972795 | 0.848178 |
| DAP | TRUE | C1-PTMC-proliferation | 0.002972795 | 0.848178 |
| PTPRM | TRUE | C1-PTMC-proliferation | 0.003237866 | 0.847166 |
| ZNF462 | TRUE | C1-PTMC-proliferation | 0.003237866 | 0.847166 |
| SLC2A10 | TRUE | C1-PTMC-proliferation | 0.003237866 | 0.847166 |
| TPD52L1 | TRUE | C1-PTMC-proliferation | 0.003527289 | 0.846154 |
| SNX1 | TRUE | C1-PTMC-proliferation | 0.003527289 | 0.846154 |
| TBX3 | TRUE | C1-PTMC-proliferation | 0.003527289 | 0.846154 |
| C11orf95 | TRUE | C1-PTMC-proliferation | 0.003527289 | 0.846154 |
| TENM1 | TRUE | C1-PTMC-proliferation | 0.003527289 | 0.846154 |
| LCA5 | TRUE | C1-PTMC-proliferation | 0.003527289 | 0.846154 |
| PURB | TRUE | C1-PTMC-proliferation | 0.003527289 | 0.846154 |
| KCNJ16 | TRUE | C1-PTMC-proliferation | 0.003837605 | 0.845142 |
| CSNK2A2 | TRUE | C1-PTMC-proliferation | 0.003837605 | 0.845142 |
| ECE1 | TRUE | C1-PTMC-proliferation | 0.004177902 | 0.84413 |
| RGAG4 | TRUE | C1-PTMC-proliferation | 0.004177902 | 0.84413 |
| PDCD4 | TRUE | C1-PTMC-proliferation | 0.004177902 | 0.84413 |
| FAM43A | TRUE | C1-PTMC-proliferation | 0.004177902 | 0.84413 |
| TMEM245 | TRUE | C1-PTMC-proliferation | 0.004545445 | 0.843117 |
| SNHG7 | TRUE | C1-PTMC-proliferation | 0.004545445 | 0.843117 |
| SLC7A8 | TRUE | C1-PTMC-proliferation | 0.004545445 | 0.843117 |
| TRPC5 | TRUE | C1-PTMC-proliferation | 0.004545445 | 0.843117 |
| TMEM98 | TRUE | C1-PTMC-proliferation | 0.004945347 | 0.842105 |
| TMCC3 | TRUE | C1-PTMC-proliferation | 0.005853985 | 0.840081 |
| PTK2 | TRUE | C1-PTMC-proliferation | 0.005853985 | 0.840081 |
| LRP4 | TRUE | C1-PTMC-proliferation | 0.006363759 | 0.839069 |
| EPHA2 | TRUE | C1-PTMC-proliferation | 0.006363759 | 0.839069 |
| C1orf116 | TRUE | C1-PTMC-proliferation | 0.006363759 | 0.839069 |
| KIAA1217 | TRUE | C1-PTMC-proliferation | 0.006363759 | 0.839069 |
| CHPT1 | TRUE | C1-PTMC-proliferation | 0.006363759 | 0.839069 |
| SH3D19 | TRUE | C1-PTMC-proliferation | 0.006363759 | 0.839069 |
| ACVR1 | TRUE | C1-PTMC-proliferation | 0.006909091 | 0.838057 |
| MPZL3 | TRUE | C1-PTMC-proliferation | 0.006909091 | 0.838057 |
| LAMC1 | TRUE | C1-PTMC-proliferation | 0.006909091 | 0.838057 |
| RARG | TRUE | C1-PTMC-proliferation | 0.006909091 | 0.838057 |
| MXRA8 | TRUE | C1-PTMC-proliferation | 0.007498067 | 0.837045 |
| RAB34 | TRUE | C1-PTMC-proliferation | 0.007498067 | 0.837045 |
| AKT3 | TRUE | C1-PTMC-proliferation | 0.007498067 | 0.837045 |
| FAM160A1 | TRUE | C1-PTMC-proliferation | 0.007498067 | 0.837045 |
| VAMP4 | TRUE | C1-PTMC-proliferation | 0.008137453 | 0.836032 |
| PGRMC1 | TRUE | C1-PTMC-proliferation | 0.008137453 | 0.836032 |
| UBQLN2 | TRUE | C1-PTMC-proliferation | 0.008137453 | 0.836032 |
| PPP1R9A | TRUE | C1-PTMC-proliferation | 0.008137453 | 0.836032 |
| FAM73A | TRUE | C1-PTMC-proliferation | 0.008137453 | 0.836032 |
| 44814 | TRUE | C1-PTMC-proliferation | 0.008137453 | 0.836032 |
| PHF10 | TRUE | C1-PTMC-proliferation | 0.008137453 | 0.836032 |
| NT5C2 | TRUE | C1-PTMC-proliferation | 0.008814377 | 0.83502 |
| GALNT7 | TRUE | C1-PTMC-proliferation | 0.009558276 | 0.834008 |
| GPR107 | TRUE | C1-PTMC-proliferation | 0.009558276 | 0.834008 |
| ZSWIM5 | TRUE | C1-PTMC-proliferation | 0.009558276 | 0.834008 |
| SUDS3 | TRUE | C1-PTMC-proliferation | 0.009558276 | 0.834008 |
| FXR1 | TRUE | C1-PTMC-proliferation | 0.009558276 | 0.834008 |
| ANKH | TRUE | C1-PTMC-proliferation | 0.009558276 | 0.834008 |
| ENTPD1 | TRUE | C1-PTMC-proliferation | 0.01034954 | 0.832996 |
| CHD4 | TRUE | C1-PTMC-proliferation | 0.01034954 | 0.832996 |
| PLEKHA4 | TRUE | C1-PTMC-proliferation | 0.01034954 | 0.832996 |
| SFTA3 | TRUE | C1-PTMC-proliferation | 0.01034954 | 0.832996 |
| GNA11 | TRUE | C1-PTMC-proliferation | 0.01034954 | 0.832996 |
| FAF1 | TRUE | C1-PTMC-proliferation | 0.011206716 | 0.831984 |
| DAPK2 | TRUE | C1-PTMC-proliferation | 0.011206716 | 0.831984 |
| PINK1 | TRUE | C1-PTMC-proliferation | 0.011206716 | 0.831984 |
| WDTC1 | TRUE | C1-PTMC-proliferation | 0.011206716 | 0.831984 |
| NKX2-1 | TRUE | C1-PTMC-proliferation | 0.011206716 | 0.831984 |
| CADM1 | TRUE | C1-PTMC-proliferation | 0.012127431 | 0.830972 |
| FGFRL1 | TRUE | C1-PTMC-proliferation | 0.012127431 | 0.830972 |
| ZBTB4 | TRUE | C1-PTMC-proliferation | 0.012127431 | 0.830972 |
| RNF146 | TRUE | C1-PTMC-proliferation | 0.013121492 | 0.82996 |
| ASAP2 | TRUE | C1-PTMC-proliferation | 0.013121492 | 0.82996 |
| ARMCX6 | TRUE | C1-PTMC-proliferation | 0.013121492 | 0.82996 |
| ZNF91 | TRUE | C1-PTMC-proliferation | 0.013121492 | 0.82996 |
| ZHX2 | TRUE | C1-PTMC-proliferation | 0.013121492 | 0.82996 |
| PHKA1 | TRUE | C1-PTMC-proliferation | 0.014185264 | 0.828947 |
| DCDC2 | TRUE | C1-PTMC-proliferation | 0.014185264 | 0.828947 |
| EFR3B | TRUE | C1-PTMC-proliferation | 0.014185264 | 0.828947 |
| ARMCX2 | TRUE | C1-PTMC-proliferation | 0.014185264 | 0.828947 |
| SPRYD3 | TRUE | C1-PTMC-proliferation | 0.014185264 | 0.828947 |
| UBXN10 | TRUE | C1-PTMC-proliferation | 0.0153293 | 0.827935 |
| KDM3B | TRUE | C1-PTMC-proliferation | 0.0153293 | 0.827935 |
| ZBTB33 | TRUE | C1-PTMC-proliferation | 0.0153293 | 0.827935 |
| SERGEF | TRUE | C1-PTMC-proliferation | 0.0153293 | 0.827935 |
| SLC48A1 | TRUE | C1-PTMC-proliferation | 0.0153293 | 0.827935 |
| GGCT | TRUE | C1-PTMC-proliferation | 0.016570129 | 0.826923 |
| INF2 | TRUE | C1-PTMC-proliferation | 0.016570129 | 0.826923 |
| ESRP1 | TRUE | C1-PTMC-proliferation | 0.016570129 | 0.826923 |
| ORMDL3 | TRUE | C1-PTMC-proliferation | 0.016570129 | 0.826923 |
| AP1M2 | TRUE | C1-PTMC-proliferation | 0.017904543 | 0.825911 |
| DSP | TRUE | C1-PTMC-proliferation | 0.017904543 | 0.825911 |
| PBX1 | TRUE | C1-PTMC-proliferation | 0.017904543 | 0.825911 |
| ALDH3A2 | TRUE | C1-PTMC-proliferation | 0.017904543 | 0.825911 |
| PALM | TRUE | C1-PTMC-proliferation | 0.017904543 | 0.825911 |
| NOL4L | TRUE | C1-PTMC-proliferation | 0.017904543 | 0.825911 |
| ZNF430 | TRUE | C1-PTMC-proliferation | 0.019334796 | 0.824899 |
| CLIP4 | TRUE | C1-PTMC-proliferation | 0.020889856 | 0.823887 |
| TMEM30B | TRUE | C1-PTMC-proliferation | 0.020889856 | 0.823887 |
| CYSLTR2 | TRUE | C1-PTMC-proliferation | 0.020889856 | 0.823887 |
| NAB2 | TRUE | C1-PTMC-proliferation | 0.020889856 | 0.823887 |
| PPFIBP1 | TRUE | C1-PTMC-proliferation | 0.020889856 | 0.823887 |
| OLFML2A | TRUE | C1-PTMC-proliferation | 0.020889856 | 0.823887 |
| CITED1 | TRUE | C1-PTMC-proliferation | 0.022541593 | 0.822874 |
| MTUS1 | TRUE | C1-PTMC-proliferation | 0.022541593 | 0.822874 |
| ERBB3 | TRUE | C1-PTMC-proliferation | 0.022541593 | 0.822874 |
| pk | TRUE | C1-PTMC-proliferation | 0.022541593 | 0.822874 |
| WWC3 | TRUE | C1-PTMC-proliferation | 0.022541593 | 0.822874 |
| FAM168A | TRUE | C1-PTMC-proliferation | 0.022541593 | 0.822874 |
| ARHGAP19 | TRUE | C1-PTMC-proliferation | 0.022541593 | 0.822874 |
| UNC119B | TRUE | C1-PTMC-proliferation | 0.022541593 | 0.822874 |
| LDB1 | TRUE | C1-PTMC-proliferation | 0.022541593 | 0.822874 |
| PTPRG | TRUE | C1-PTMC-proliferation | 0.022541593 | 0.822874 |
| IQCA1 | TRUE | C1-PTMC-proliferation | 0.024277125 | 0.821862 |
| ZNF714 | TRUE | C1-PTMC-proliferation | 0.024277125 | 0.821862 |
| CCND1 | TRUE | C1-PTMC-proliferation | 0.02619456 | 0.82085 |
| BAHD1 | TRUE | C1-PTMC-proliferation | 0.02619456 | 0.82085 |
| IFT122 | TRUE | C1-PTMC-proliferation | 0.02619456 | 0.82085 |
| HIPK2 | TRUE | C1-PTMC-proliferation | 0.028246766 | 0.819838 |
| GPRC5B | TRUE | C1-PTMC-proliferation | 0.028246766 | 0.819838 |
| PCDH1 | TRUE | C1-PTMC-proliferation | 0.028246766 | 0.819838 |
| WWC2 | TRUE | C1-PTMC-proliferation | 0.028246766 | 0.819838 |
| TGFA | TRUE | C1-PTMC-proliferation | 0.028246766 | 0.819838 |
| DAG1 | TRUE | C1-PTMC-proliferation | 0.028246766 | 0.819838 |
| ANKS6 | TRUE | C1-PTMC-proliferation | 0.028246766 | 0.819838 |
| KCNK5 | TRUE | C1-PTMC-proliferation | 0.030428271 | 0.818826 |
| ARHGAP12 | TRUE | C1-PTMC-proliferation | 0.032802833 | 0.817814 |
| DUOX2 | TRUE | C1-PTMC-proliferation | 0.032802833 | 0.817814 |
| TOMM20 | TRUE | C1-PTMC-proliferation | 0.032802833 | 0.817814 |
| DUOXA1 | TRUE | C1-PTMC-proliferation | 0.032802833 | 0.817814 |
| SSPN | TRUE | C1-PTMC-proliferation | 0.032802833 | 0.817814 |
| CAMSAP2 | TRUE | C1-PTMC-proliferation | 0.035302634 | 0.816802 |
| APH1B | TRUE | C1-PTMC-proliferation | 0.035302634 | 0.816802 |
| JMY | TRUE | C1-PTMC-proliferation | 0.035302634 | 0.816802 |
| MGAT4C | TRUE | C1-PTMC-proliferation | 0.038021681 | 0.815789 |
| EHD2 | TRUE | C1-PTMC-proliferation | 0.038021681 | 0.815789 |
| EIF4E3 | TRUE | C1-PTMC-proliferation | 0.038021681 | 0.815789 |
| PRNP | TRUE | C1-PTMC-proliferation | 0.038021681 | 0.815789 |
| SAMD4B | TRUE | C1-PTMC-proliferation | 0.038021681 | 0.815789 |
| BRAF | TRUE | C1-PTMC-proliferation | 0.038021681 | 0.815789 |
| USP22 | TRUE | C1-PTMC-proliferation | 0.040917221 | 0.814777 |
| C1orf115 | TRUE | C1-PTMC-proliferation | 0.040917221 | 0.814777 |
| NCKAP1 | TRUE | C1-PTMC-proliferation | 0.040917221 | 0.814777 |
| CA11 | TRUE | C1-PTMC-proliferation | 0.040917221 | 0.814777 |
| HIRA | TRUE | C1-PTMC-proliferation | 0.040917221 | 0.814777 |
| GNG12 | TRUE | C1-PTMC-proliferation | 0.040917221 | 0.814777 |
| RASEF | TRUE | C1-PTMC-proliferation | 0.040917221 | 0.814777 |
| GABRB2 | TRUE | C1-PTMC-proliferation | 0.043988007 | 0.813765 |
| CLEC16A | TRUE | C1-PTMC-proliferation | 0.043988007 | 0.813765 |
| DHRS3 | TRUE | C1-PTMC-proliferation | 0.043988007 | 0.813765 |
| MAPK14 | TRUE | C1-PTMC-proliferation | 0.043988007 | 0.813765 |
| RGS5 | TRUE | C1-PTMC-proliferation | 0.043988007 | 0.813765 |
| MYO1D | TRUE | C1-PTMC-proliferation | 0.047293674 | 0.812753 |
| GPR56 | TRUE | C1-PTMC-proliferation | 0.047293674 | 0.812753 |
| ACTN4 | TRUE | C1-PTMC-proliferation | 0.047293674 | 0.812753 |
| ARHGAP29 | TRUE | C1-PTMC-proliferation | 0.047293674 | 0.812753 |
| SYT12 | TRUE | C1-PTMC-proliferation | 0.047293674 | 0.812753 |
| PPP1R21 | TRUE | C1-PTMC-proliferation | 0.047293674 | 0.812753 |
| CAMK2N1 | TRUE | C1-PTMC-proliferation | 0.047293674 | 0.812753 |
| IGKV4-1 | TRUE | C2-PTMC-inflammatory | 1.66E-14 | 1 |
| IGHG1 | TRUE | C2-PTMC-inflammatory | 1.16E-13 | 0.996964 |
| IGKC | TRUE | C2-PTMC-inflammatory | 1.16E-13 | 0.996964 |
| IGKV1D-39 | TRUE | C2-PTMC-inflammatory | 1.99E-13 | 0.995951 |
| IGKV1-5 | TRUE | C2-PTMC-inflammatory | 1.99E-13 | 0.995951 |
| IGKV3-15 | TRUE | C2-PTMC-inflammatory | 1.99E-13 | 0.995951 |
| IGKV3-11 | TRUE | C2-PTMC-inflammatory | 3.15E-13 | 0.994939 |
| IGHV1-18 | TRUE | C2-PTMC-inflammatory | 4.97E-13 | 0.993927 |
| IGHV4-59 | TRUE | C2-PTMC-inflammatory | 4.97E-13 | 0.993927 |
| PIM2 | TRUE | C2-PTMC-inflammatory | 4.97E-13 | 0.993927 |
| IGLL5 | TRUE | C2-PTMC-inflammatory | 7.44E-13 | 0.992915 |
| IGHV3-21 | TRUE | C2-PTMC-inflammatory | 7.44E-13 | 0.992915 |
| IGHG3 | TRUE | C2-PTMC-inflammatory | 1.60E-12 | 0.990891 |
| IGLC1 | TRUE | C2-PTMC-inflammatory | 2.30E-12 | 0.989879 |
| IGKV2-28 | TRUE | C2-PTMC-inflammatory | 2.30E-12 | 0.989879 |
| IGHV4-39 | TRUE | C2-PTMC-inflammatory | 2.30E-12 | 0.989879 |
| IGKV3-20 | TRUE | C2-PTMC-inflammatory | 4.49E-12 | 0.987854 |
| IGHM | TRUE | C2-PTMC-inflammatory | 8.39E-12 | 0.98583 |
| IGHV3-23 | TRUE | C2-PTMC-inflammatory | 8.39E-12 | 0.98583 |
| IGLV2-14 | TRUE | C2-PTMC-inflammatory | 1.13E-11 | 0.984818 |
| IGLC3 | TRUE | C2-PTMC-inflammatory | 1.51E-11 | 0.983806 |
| GVINP1 | TRUE | C2-PTMC-inflammatory | 1.51E-11 | 0.983806 |
| TXNDC5 | TRUE | C2-PTMC-inflammatory | 2.00E-11 | 0.982794 |
| IGJ | TRUE | C2-PTMC-inflammatory | 2.00E-11 | 0.982794 |
| IGHG4 | TRUE | C2-PTMC-inflammatory | 2.63E-11 | 0.981781 |
| IGLC2 | TRUE | C2-PTMC-inflammatory | 2.63E-11 | 0.981781 |
| IGHV3-30 | TRUE | C2-PTMC-inflammatory | 3.44E-11 | 0.980769 |
| IGHA1 | TRUE | C2-PTMC-inflammatory | 9.50E-11 | 0.976721 |
| IGLV3-19 | TRUE | C2-PTMC-inflammatory | 2.43E-10 | 0.972672 |
| IGHA2 | TRUE | C2-PTMC-inflammatory | 5.84E-10 | 0.968623 |
| ST6GAL1 | TRUE | C2-PTMC-inflammatory | 8.88E-10 | 0.966599 |
| IL16 | TRUE | C2-PTMC-inflammatory | 1.63E-09 | 0.963563 |
| IGHG2 | TRUE | C2-PTMC-inflammatory | 2.41E-09 | 0.961538 |
| ITGA4 | TRUE | C2-PTMC-inflammatory | 2.41E-09 | 0.961538 |
| PIK3AP1 | TRUE | C2-PTMC-inflammatory | 3.52E-09 | 0.959514 |
| DOCK8 | TRUE | C2-PTMC-inflammatory | 3.52E-09 | 0.959514 |
| HERPUD1 | TRUE | C2-PTMC-inflammatory | 4.25E-09 | 0.958502 |
| BIRC3 | TRUE | C2-PTMC-inflammatory | 5.11E-09 | 0.95749 |
| NUP210 | TRUE | C2-PTMC-inflammatory | 5.11E-09 | 0.95749 |
| CCL5 | TRUE | C2-PTMC-inflammatory | 5.11E-09 | 0.95749 |
| TNFAIP3 | TRUE | C2-PTMC-inflammatory | 5.11E-09 | 0.95749 |
| IGLV2-23 | TRUE | C2-PTMC-inflammatory | 5.11E-09 | 0.95749 |
| HCLS1 | TRUE | C2-PTMC-inflammatory | 6.13E-09 | 0.956478 |
| CXCR4 | TRUE | C2-PTMC-inflammatory | 7.34E-09 | 0.955466 |
| IL10RA | TRUE | C2-PTMC-inflammatory | 8.78E-09 | 0.954453 |
| GBP5 | TRUE | C2-PTMC-inflammatory | 8.78E-09 | 0.954453 |
| IKZF1 | TRUE | C2-PTMC-inflammatory | 1.25E-08 | 0.952429 |
| GPRIN3 | TRUE | C2-PTMC-inflammatory | 1.48E-08 | 0.951417 |
| SEPT6 | TRUE | C2-PTMC-inflammatory | 1.48E-08 | 0.951417 |
| WDFY4 | TRUE | C2-PTMC-inflammatory | 1.76E-08 | 0.950405 |
| NCKAP1L | TRUE | C2-PTMC-inflammatory | 2.08E-08 | 0.949393 |
| CYLD | TRUE | C2-PTMC-inflammatory | 2.08E-08 | 0.949393 |
| PTPRC | TRUE | C2-PTMC-inflammatory | 2.46E-08 | 0.948381 |
| FAM46C | TRUE | C2-PTMC-inflammatory | 2.46E-08 | 0.948381 |
| CCL19 | TRUE | C2-PTMC-inflammatory | 2.91E-08 | 0.947368 |
| TYMP | TRUE | C2-PTMC-inflammatory | 3.43E-08 | 0.946356 |
| CD53 | TRUE | C2-PTMC-inflammatory | 3.43E-08 | 0.946356 |
| FYB | TRUE | C2-PTMC-inflammatory | 4.74E-08 | 0.944332 |
| IRAK1 | TRUE | C2-PTMC-inflammatory | 7.63E-08 | 0.941296 |
| ITGAL | TRUE | C2-PTMC-inflammatory | 7.63E-08 | 0.941296 |
| IL7R | TRUE | C2-PTMC-inflammatory | 8.92E-08 | 0.940283 |
| ARHGAP30 | TRUE | C2-PTMC-inflammatory | 8.92E-08 | 0.940283 |
| DOCK2 | TRUE | C2-PTMC-inflammatory | 1.64E-07 | 0.936235 |
| XBP1 | TRUE | C2-PTMC-inflammatory | 1.64E-07 | 0.936235 |
| PLEK | TRUE | C2-PTMC-inflammatory | 1.90E-07 | 0.935223 |
| CXCL9 | TRUE | C2-PTMC-inflammatory | 2.55E-07 | 0.933198 |
| APOL3 | TRUE | C2-PTMC-inflammatory | 2.95E-07 | 0.932186 |
| PPP1R16B | TRUE | C2-PTMC-inflammatory | 3.41E-07 | 0.931174 |
| SAMHD1 | TRUE | C2-PTMC-inflammatory | 3.41E-07 | 0.931174 |
| RGS1 | TRUE | C2-PTMC-inflammatory | 6.01E-07 | 0.927126 |
| CD84 | TRUE | C2-PTMC-inflammatory | 6.90E-07 | 0.926113 |
| LAP3 | TRUE | C2-PTMC-inflammatory | 9.08E-07 | 0.924089 |
| MPEG1 | TRUE | C2-PTMC-inflammatory | 1.04E-06 | 0.923077 |
| LAPTM5 | TRUE | C2-PTMC-inflammatory | 1.19E-06 | 0.922065 |
| SRGN | TRUE | C2-PTMC-inflammatory | 1.36E-06 | 0.921053 |
| SEMA4D | TRUE | C2-PTMC-inflammatory | 2.02E-06 | 0.918016 |
| HLA-DOA | TRUE | C2-PTMC-inflammatory | 2.02E-06 | 0.918016 |
| BTN3A1 | TRUE | C2-PTMC-inflammatory | 2.02E-06 | 0.918016 |
| CD4 | TRUE | C2-PTMC-inflammatory | 2.30E-06 | 0.917004 |
| HLA-F | TRUE | C2-PTMC-inflammatory | 2.61E-06 | 0.915992 |
| CORO1A | TRUE | C2-PTMC-inflammatory | 2.61E-06 | 0.915992 |
| HLA-DMA | TRUE | C2-PTMC-inflammatory | 2.97E-06 | 0.91498 |
| CD74 | TRUE | C2-PTMC-inflammatory | 3.37E-06 | 0.913968 |
| GBP4 | TRUE | C2-PTMC-inflammatory | 3.37E-06 | 0.913968 |
| CD163 | TRUE | C2-PTMC-inflammatory | 3.83E-06 | 0.912955 |
| HLA-DPB1 | TRUE | C2-PTMC-inflammatory | 4.91E-06 | 0.910931 |
| C1QA | TRUE | C2-PTMC-inflammatory | 5.56E-06 | 0.909919 |
| PLEKHO1 | TRUE | C2-PTMC-inflammatory | 5.56E-06 | 0.909919 |
| GBP1 | TRUE | C2-PTMC-inflammatory | 6.29E-06 | 0.908907 |
| HLA-E | TRUE | C2-PTMC-inflammatory | 7.10E-06 | 0.907895 |
| MAN2A1 | TRUE | C2-PTMC-inflammatory | 7.10E-06 | 0.907895 |
| C1QC | TRUE | C2-PTMC-inflammatory | 7.10E-06 | 0.907895 |
| HLA-DPA1 | TRUE | C2-PTMC-inflammatory | 7.10E-06 | 0.907895 |
| C1QB | TRUE | C2-PTMC-inflammatory | 8.01E-06 | 0.906883 |
| FCGR2B | TRUE | C2-PTMC-inflammatory | 8.01E-06 | 0.906883 |
| CIITA | TRUE | C2-PTMC-inflammatory | 9.03E-06 | 0.90587 |
| STK17B | TRUE | C2-PTMC-inflammatory | 9.03E-06 | 0.90587 |
| PSMB10 | TRUE | C2-PTMC-inflammatory | 1.02E-05 | 0.904858 |
| CD52 | TRUE | C2-PTMC-inflammatory | 1.15E-05 | 0.903846 |
| SEL1L | TRUE | C2-PTMC-inflammatory | 1.29E-05 | 0.902834 |
| HLA-DRA | TRUE | C2-PTMC-inflammatory | 1.45E-05 | 0.901822 |
| MS4A6A | TRUE | C2-PTMC-inflammatory | 1.45E-05 | 0.901822 |
| TRAF1 | TRUE | C2-PTMC-inflammatory | 1.63E-05 | 0.90081 |
| RASSF2 | TRUE | C2-PTMC-inflammatory | 2.06E-05 | 0.898785 |
| IRF1 | TRUE | C2-PTMC-inflammatory | 2.30E-05 | 0.897773 |
| EVL | TRUE | C2-PTMC-inflammatory | 2.30E-05 | 0.897773 |
| RNASET2 | TRUE | C2-PTMC-inflammatory | 2.58E-05 | 0.896761 |
| CSF2RB | TRUE | C2-PTMC-inflammatory | 2.89E-05 | 0.895749 |
| SLFN11 | TRUE | C2-PTMC-inflammatory | 2.89E-05 | 0.895749 |
| COTL1 | TRUE | C2-PTMC-inflammatory | 2.89E-05 | 0.895749 |
| PSMB9 | TRUE | C2-PTMC-inflammatory | 2.89E-05 | 0.895749 |
| EPSTI1 | TRUE | C2-PTMC-inflammatory | 2.89E-05 | 0.895749 |
| KIAA1551 | TRUE | C2-PTMC-inflammatory | 2.89E-05 | 0.895749 |
| PSME2 | TRUE | C2-PTMC-inflammatory | 3.23E-05 | 0.894737 |
| TNFAIP8 | TRUE | C2-PTMC-inflammatory | 3.23E-05 | 0.894737 |
| ITGB2 | TRUE | C2-PTMC-inflammatory | 3.61E-05 | 0.893725 |
| CECR1 | TRUE | C2-PTMC-inflammatory | 4.51E-05 | 0.8917 |
| STK4 | TRUE | C2-PTMC-inflammatory | 4.51E-05 | 0.8917 |
| FGL2 | TRUE | C2-PTMC-inflammatory | 4.51E-05 | 0.8917 |
| BTN3A3 | TRUE | C2-PTMC-inflammatory | 5.03E-05 | 0.890688 |
| IL32 | TRUE | C2-PTMC-inflammatory | 5.03E-05 | 0.890688 |
| PLSCR1 | TRUE | C2-PTMC-inflammatory | 5.03E-05 | 0.890688 |
| NLRC5 | TRUE | C2-PTMC-inflammatory | 5.03E-05 | 0.890688 |
| TAP1 | TRUE | C2-PTMC-inflammatory | 6.96E-05 | 0.887652 |
| PTAFR | TRUE | C2-PTMC-inflammatory | 7.74E-05 | 0.88664 |
| LCP1 | TRUE | C2-PTMC-inflammatory | 7.74E-05 | 0.88664 |
| TNFSF10 | TRUE | C2-PTMC-inflammatory | 8.61E-05 | 0.885628 |
| SMAP2 | TRUE | C2-PTMC-inflammatory | 8.61E-05 | 0.885628 |
| PAPSS2 | TRUE | C2-PTMC-inflammatory | 9.56E-05 | 0.884615 |
| LSP1 | TRUE | C2-PTMC-inflammatory | 9.56E-05 | 0.884615 |
| CYBB | TRUE | C2-PTMC-inflammatory | 0.000106166 | 0.883603 |
| CCL21 | TRUE | C2-PTMC-inflammatory | 0.000106166 | 0.883603 |
| NCOA3 | TRUE | C2-PTMC-inflammatory | 0.000106166 | 0.883603 |
| MAN2B1 | TRUE | C2-PTMC-inflammatory | 0.000145171 | 0.880567 |
| CCDC69 | TRUE | C2-PTMC-inflammatory | 0.00016092 | 0.879555 |
| CD14 | TRUE | C2-PTMC-inflammatory | 0.00016092 | 0.879555 |
| CELF2 | TRUE | C2-PTMC-inflammatory | 0.00016092 | 0.879555 |
| PLCG2 | TRUE | C2-PTMC-inflammatory | 0.00016092 | 0.879555 |
| MICAL1 | TRUE | C2-PTMC-inflammatory | 0.00016092 | 0.879555 |
| ARHGEF6 | TRUE | C2-PTMC-inflammatory | 0.000178127 | 0.878543 |
| SLCO2B1 | TRUE | C2-PTMC-inflammatory | 0.000178127 | 0.878543 |
| HIST2H4A | TRUE | C2-PTMC-inflammatory | 0.000178127 | 0.878543 |
| FPR3 | TRUE | C2-PTMC-inflammatory | 0.000178127 | 0.878543 |
| CP | TRUE | C2-PTMC-inflammatory | 0.000178127 | 0.878543 |
| VOPP1 | TRUE | C2-PTMC-inflammatory | 0.000196937 | 0.87753 |
| IFI16 | TRUE | C2-PTMC-inflammatory | 0.000217928 | 0.876518 |
| LRRK1 | TRUE | C2-PTMC-inflammatory | 0.000240972 | 0.875506 |
| AMICA1 | TRUE | C2-PTMC-inflammatory | 0.000266361 | 0.874494 |
| RAC2 | TRUE | C2-PTMC-inflammatory | 0.00029414 | 0.873482 |
| NABP1 | TRUE | C2-PTMC-inflammatory | 0.00029414 | 0.873482 |
| OAS2 | TRUE | C2-PTMC-inflammatory | 0.00029414 | 0.873482 |
| BTN3A2 | TRUE | C2-PTMC-inflammatory | 0.000324504 | 0.87247 |
| ITGAX | TRUE | C2-PTMC-inflammatory | 0.000324504 | 0.87247 |
| PPP1R18 | TRUE | C2-PTMC-inflammatory | 0.000324504 | 0.87247 |
| MAFB | TRUE | C2-PTMC-inflammatory | 0.000357883 | 0.871457 |
| FAM49B | TRUE | C2-PTMC-inflammatory | 0.000394817 | 0.870445 |
| MAN1A1 | TRUE | C2-PTMC-inflammatory | 0.000394817 | 0.870445 |
| BTG1 | TRUE | C2-PTMC-inflammatory | 0.000434876 | 0.869433 |
| HLA-DQA1 | TRUE | C2-PTMC-inflammatory | 0.00047915 | 0.868421 |
| BMP2K | TRUE | C2-PTMC-inflammatory | 0.00047915 | 0.868421 |
| PDE4B | TRUE | C2-PTMC-inflammatory | 0.00047915 | 0.868421 |
| GLIPR1 | TRUE | C2-PTMC-inflammatory | 0.000527435 | 0.867409 |
| C1R | TRUE | C2-PTMC-inflammatory | 0.000580288 | 0.866397 |
| PTPN6 | TRUE | C2-PTMC-inflammatory | 0.000638111 | 0.865385 |
| ANKRD44 | TRUE | C2-PTMC-inflammatory | 0.000701488 | 0.864372 |
| SLC8A1 | TRUE | C2-PTMC-inflammatory | 0.000701488 | 0.864372 |
| IFI30 | TRUE | C2-PTMC-inflammatory | 0.000846838 | 0.862348 |
| GNA13 | TRUE | C2-PTMC-inflammatory | 0.000846838 | 0.862348 |
| RARRES3 | TRUE | C2-PTMC-inflammatory | 0.000929362 | 0.861336 |
| PAG1 | TRUE | C2-PTMC-inflammatory | 0.000929362 | 0.861336 |
| TLR4 | TRUE | C2-PTMC-inflammatory | 0.001225594 | 0.8583 |
| WIPF1 | TRUE | C2-PTMC-inflammatory | 0.001468778 | 0.856275 |
| SPN | TRUE | C2-PTMC-inflammatory | 0.001468778 | 0.856275 |
| TBC1D4 | TRUE | C2-PTMC-inflammatory | 0.001468778 | 0.856275 |
| EPB41 | TRUE | C2-PTMC-inflammatory | 0.001468778 | 0.856275 |
| CD68 | TRUE | C2-PTMC-inflammatory | 0.001605577 | 0.855263 |
| VCAM1 | TRUE | C2-PTMC-inflammatory | 0.00175728 | 0.854251 |
| PLEKHA2 | TRUE | C2-PTMC-inflammatory | 0.00175728 | 0.854251 |
| FLI1 | TRUE | C2-PTMC-inflammatory | 0.00175728 | 0.854251 |
| TYROBP | TRUE | C2-PTMC-inflammatory | 0.001920795 | 0.853239 |
| IQGAP2 | TRUE | C2-PTMC-inflammatory | 0.002291738 | 0.851215 |
| RALGDS | TRUE | C2-PTMC-inflammatory | 0.002291738 | 0.851215 |
| SGPP2 | TRUE | C2-PTMC-inflammatory | 0.002501564 | 0.850202 |
| STAT1 | TRUE | C2-PTMC-inflammatory | 0.002501564 | 0.850202 |
| CYTH1 | TRUE | C2-PTMC-inflammatory | 0.00272763 | 0.84919 |
| TNFRSF14 | TRUE | C2-PTMC-inflammatory | 0.002972795 | 0.848178 |
| C1S | TRUE | C2-PTMC-inflammatory | 0.002972795 | 0.848178 |
| DOCK10 | TRUE | C2-PTMC-inflammatory | 0.002972795 | 0.848178 |
| FAM107B | TRUE | C2-PTMC-inflammatory | 0.003237866 | 0.847166 |
| SSR4 | TRUE | C2-PTMC-inflammatory | 0.003237866 | 0.847166 |
| MMP9 | TRUE | C2-PTMC-inflammatory | 0.003237866 | 0.847166 |
| TC2N | TRUE | C2-PTMC-inflammatory | 0.003527289 | 0.846154 |
| GIMAP4 | TRUE | C2-PTMC-inflammatory | 0.003527289 | 0.846154 |
| APOL2 | TRUE | C2-PTMC-inflammatory | 0.003527289 | 0.846154 |
| C3 | TRUE | C2-PTMC-inflammatory | 0.003837605 | 0.845142 |
| SAMD9L | TRUE | C2-PTMC-inflammatory | 0.003837605 | 0.845142 |
| ARHGEF1 | TRUE | C2-PTMC-inflammatory | 0.003837605 | 0.845142 |
| LYZ | TRUE | C2-PTMC-inflammatory | 0.004177902 | 0.84413 |
| IRF9 | TRUE | C2-PTMC-inflammatory | 0.004177902 | 0.84413 |
| CSF1R | TRUE | C2-PTMC-inflammatory | 0.004945347 | 0.842105 |
| NFKBIA | TRUE | C2-PTMC-inflammatory | 0.006909091 | 0.838057 |
| IFITM1 | TRUE | C2-PTMC-inflammatory | 0.006909091 | 0.838057 |
| FCGR2A | TRUE | C2-PTMC-inflammatory | 0.008137453 | 0.836032 |
| PARP1 | TRUE | C2-PTMC-inflammatory | 0.008137453 | 0.836032 |
| INPP5D | TRUE | C2-PTMC-inflammatory | 0.008137453 | 0.836032 |
| GSTK1 | TRUE | C2-PTMC-inflammatory | 0.008137453 | 0.836032 |
| ADCY7 | TRUE | C2-PTMC-inflammatory | 0.008814377 | 0.83502 |
| XAF1 | TRUE | C2-PTMC-inflammatory | 0.008814377 | 0.83502 |
| ATM | TRUE | C2-PTMC-inflammatory | 0.008814377 | 0.83502 |
| GM2A | TRUE | C2-PTMC-inflammatory | 0.009558276 | 0.834008 |
| IKZF3 | TRUE | C2-PTMC-inflammatory | 0.009558276 | 0.834008 |
| NIN | TRUE | C2-PTMC-inflammatory | 0.009558276 | 0.834008 |
| HLA-DRB1 | TRUE | C2-PTMC-inflammatory | 0.01034954 | 0.832996 |
| HMHA1 | TRUE | C2-PTMC-inflammatory | 0.01034954 | 0.832996 |
| CTSE | TRUE | C2-PTMC-inflammatory | 0.011206716 | 0.831984 |
| WARS | TRUE | C2-PTMC-inflammatory | 0.011206716 | 0.831984 |
| ELMO1 | TRUE | C2-PTMC-inflammatory | 0.011206716 | 0.831984 |
| AKNA | TRUE | C2-PTMC-inflammatory | 0.012127431 | 0.830972 |
| CMKLR1 | TRUE | C2-PTMC-inflammatory | 0.012127431 | 0.830972 |
| COL23A1 | TRUE | C2-PTMC-inflammatory | 0.012127431 | 0.830972 |
| TMEM176B | TRUE | C2-PTMC-inflammatory | 0.012127431 | 0.830972 |
| CTSZ | TRUE | C2-PTMC-inflammatory | 0.013121492 | 0.82996 |
| UBE2L6 | TRUE | C2-PTMC-inflammatory | 0.013121492 | 0.82996 |
| C17orf62 | TRUE | C2-PTMC-inflammatory | 0.013121492 | 0.82996 |
| DCP2 | TRUE | C2-PTMC-inflammatory | 0.013121492 | 0.82996 |
| AKR1B1 | TRUE | C2-PTMC-inflammatory | 0.014185264 | 0.828947 |
| CARD8 | TRUE | C2-PTMC-inflammatory | 0.014185264 | 0.828947 |
| TNFAIP2 | TRUE | C2-PTMC-inflammatory | 0.014185264 | 0.828947 |
| IGSF6 | TRUE | C2-PTMC-inflammatory | 0.014185264 | 0.828947 |
| NFKB2 | TRUE | C2-PTMC-inflammatory | 0.0153293 | 0.827935 |
| ANXA6 | TRUE | C2-PTMC-inflammatory | 0.016570129 | 0.826923 |
| ITGAM | TRUE | C2-PTMC-inflammatory | 0.016570129 | 0.826923 |
| RABGAP1L | TRUE | C2-PTMC-inflammatory | 0.017904543 | 0.825911 |
| SLFN5 | TRUE | C2-PTMC-inflammatory | 0.019334796 | 0.824899 |
| BCL2L11 | TRUE | C2-PTMC-inflammatory | 0.019334796 | 0.824899 |
| HIST1H1E | TRUE | C2-PTMC-inflammatory | 0.020889856 | 0.823887 |
| KIAA0930 | TRUE | C2-PTMC-inflammatory | 0.022541593 | 0.822874 |
| TAPBP | TRUE | C2-PTMC-inflammatory | 0.022541593 | 0.822874 |
| TNS3 | TRUE | C2-PTMC-inflammatory | 0.022541593 | 0.822874 |
| SERPING1 | TRUE | C2-PTMC-inflammatory | 0.022541593 | 0.822874 |
| MBNL1 | TRUE | C2-PTMC-inflammatory | 0.024277125 | 0.821862 |
| PARP10 | TRUE | C2-PTMC-inflammatory | 0.024277125 | 0.821862 |
| DPT | TRUE | C2-PTMC-inflammatory | 0.02619456 | 0.82085 |
| UGCG | TRUE | C2-PTMC-inflammatory | 0.02619456 | 0.82085 |
| PREX1 | TRUE | C2-PTMC-inflammatory | 0.028246766 | 0.819838 |
| SOAT1 | TRUE | C2-PTMC-inflammatory | 0.030428271 | 0.818826 |
| CTSS | TRUE | C2-PTMC-inflammatory | 0.030428271 | 0.818826 |
| HIST1H1D | TRUE | C2-PTMC-inflammatory | 0.032802833 | 0.817814 |
| SIRPA | TRUE | C2-PTMC-inflammatory | 0.032802833 | 0.817814 |
| EFEMP1 | TRUE | C2-PTMC-inflammatory | 0.032802833 | 0.817814 |
| IFI44L | TRUE | C2-PTMC-inflammatory | 0.032802833 | 0.817814 |
| HLA-B | TRUE | C2-PTMC-inflammatory | 0.035302634 | 0.816802 |
| LGMN | TRUE | C2-PTMC-inflammatory | 0.040917221 | 0.814777 |
| MGAT1 | TRUE | C2-PTMC-inflammatory | 0.040917221 | 0.814777 |
| NEK6 | TRUE | C2-PTMC-inflammatory | 0.043988007 | 0.813765 |
| CHST11 | TRUE | C2-PTMC-inflammatory | 0.043988007 | 0.813765 |
| PLEKHO2 | TRUE | C2-PTMC-inflammatory | 0.047293674 | 0.812753 |
